# Supplementary material for: Aedes aegypti Strain Subjected to Long-Term Exposure to Bacillus thuringiensis svar. israelensis Larvicides Displays an Altered Transcriptional Response to Zika Virus Infection
Source: Viruses. 2022 Dec 27;15(1):72. doi: 10.3390/v15010072 (PMC9866606; doi:10.3390/v15010072)
Supplement: Supplementary file 1 [file viruses-15-00072-s001.zip › viruses-2052671-supplementary.pdf]

Figure S1: Multidimensional scaling plot of the gene expression profile by RNA-seq comparing the *Aedes aegypti* females from a strain exposed to *Bacillus thuringiensis* svar. *israelensis* (RecBti F_{35}, black) and a reference strain (RecL, gray).

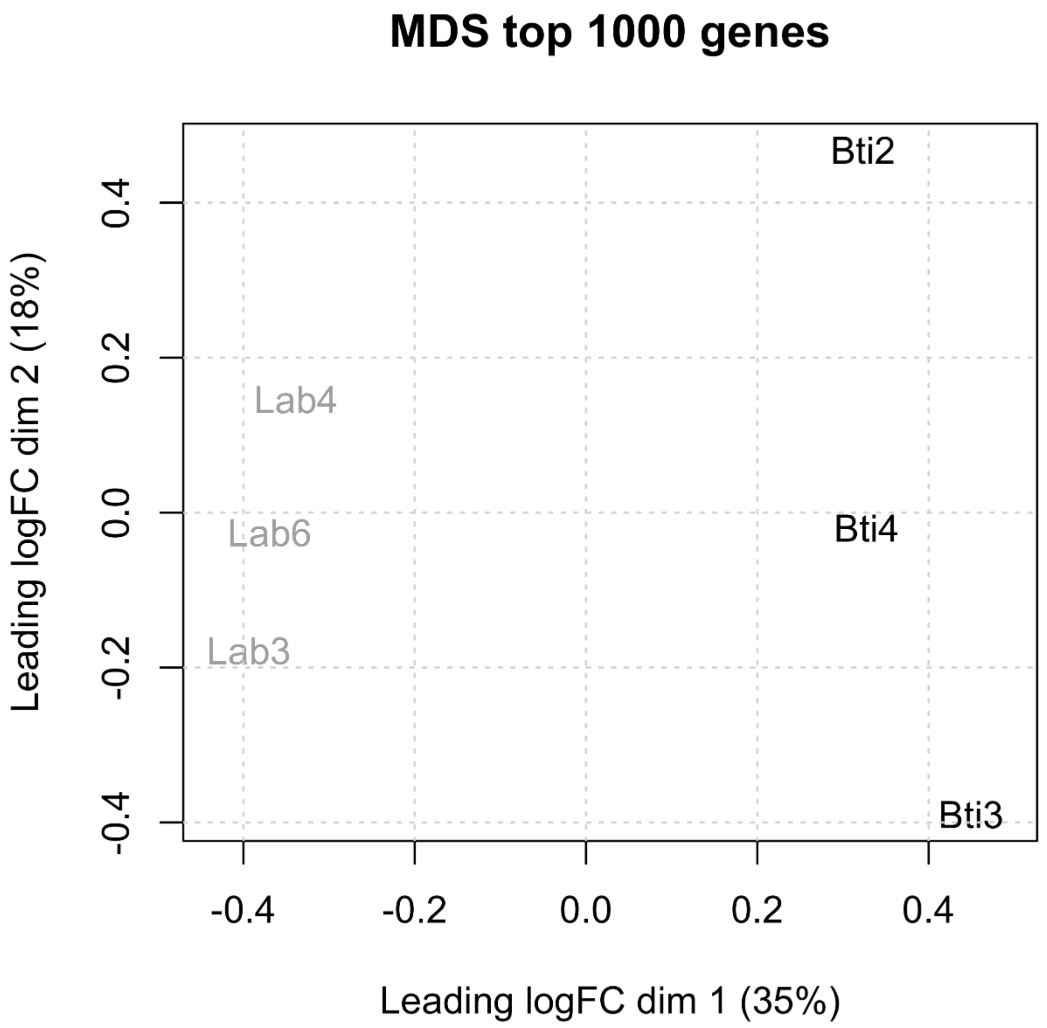

Figure S1: Multidimensional scaling plot of the gene expression profile by RNA-seq comparing the *Aedes aegypti* females from a strain exposed to *Bacillus thuringiensis* svar. *israelensis* (RecBti F_{35}, black) and a reference strain (RecL, gray).

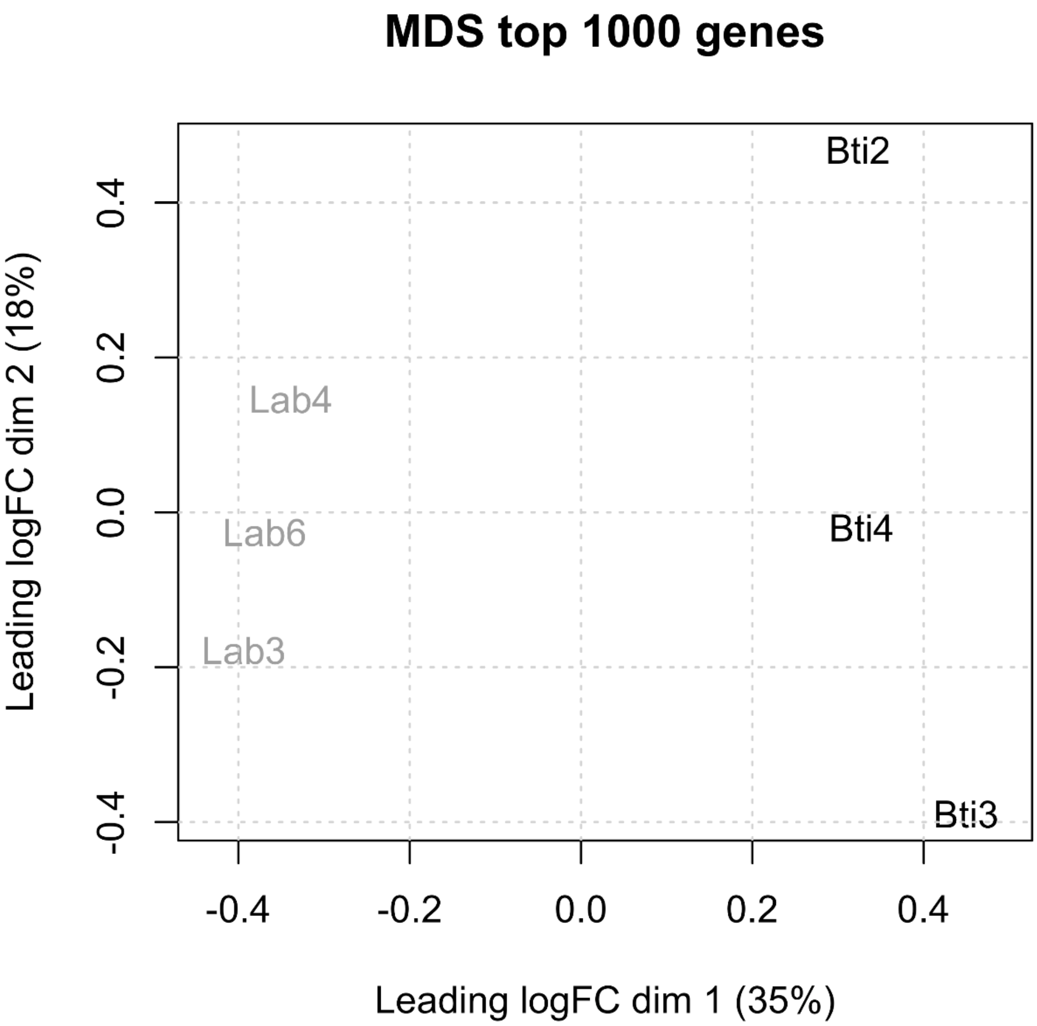

Table S2: Full dataset from RT-qPCR assays of genes from an *Aedes aegypti* strain continuously exposed to the larvicide *Bacillus thuringiensis* svar. *israelensis* (RecBti F_{30}) compared to a reference strains (RecL). RNA pools of five females (heads) orally fed blood with ZIKV-infected (INF) and noninfected (NINF) were analyzed per strain, and two independent assays were analysed. Statistical difference of *p < 0.05 or *p < 0.005 according to Student's t test.

**Comparison**	**Within strains**		**Between strains**	
	**RecL**	**RecBti**	**NINF**	**INF**
**Genes**	**NINF X INF**	**NINF X INF**	**RecL x RecBti**	**RecL x RecBti**
Hop	2.253	0.267	1.801	0.213
	1.249	0.253	1.448	0.293
Mean ± SD	1.751 ± 0.709	0.260 ± 0.009*	1.624 ± 0.249	0.253 ± 0.056*
Domeless	5.426	0.131	2.616	0.063
	2.324	0.135	2.720	0.135
Mean ± SD	3.875 ± 2.193	0.241 ± 0.003**	2.668 ± 0.073*	0.099 ± 0.050*
Relish 1	0.740	0.178	1.761	0.261
	0.517	0.283	1.246	0.601
Mean ± SD	0.628 ± 0.158	0.230 ± 0.074*	1.503 ± 0.363	0.431 ± 0.240
Cactus	0.681	0.423	1.402	1.595
	0.254	0.182	1.666	2.200
Mean ± SD	0.467 ± 0.302	0.302 ± 0.170	1.534 ± 0.187	1.897 ± 0.428
Defensin A	0.541	TND[a]	0.370	TND
	0.492	TND	0.488	TND
Mean ± SD	0.516 ± 0.035*	TND	0.429 ± 0.083*	TND
Cecropin G	2.068	TND	1.332	TND
	1.848	TND	0.506	TND
Mean ± SD	1.958 ± 1.556	TND	0.919 ± 0.292	TND
Gambicin	1.819	0.722	1.504	0.601
	0.540	0.134	1.252	0.281
Mean ± SD	1.179 ± 0.904	0.428 ± 0.416	1.378 ± 1.782	0.441 ± 0.226

[a] Transcripts not detected.

Table S3: Statistics for the RNA-seq libraries sequenced from *Aedes aegypti* females from a strain chronically exposed to *Bacillus thuringiensis* svar. *israelensis* (RecBti F_{35}) compared to a reference strain (RecL).

**Sample**	**RecBti**		**RecL**	
Total reads	10.103.560		13.058.581	
Total base pairs	1.515.534.000		1.958.787.150	
	N. of reads	%	N. of reads	%
Total mapped	9.777.029	96.77	12.668.760	97.01
Unique mapped	8.547.470	87.42	11.102.814	85.02
Multiple mapped	1.229.559	12.58	1.565.946	11.99
Unmapped	326.533	3.23	389.826	2.99
Total mapped in genes	8.144.728	95.29	10.589.297	95.37
Total mapped no gene	402.742	4.71	51.3517	4.63

Table S4. Differentially expressed genes from an Aedes aegypti strain continuously exposed to the larvicide Bacillus thuringiensis svar. israelensis (RecBti F35) for the previous 34 generations, compared to a reference strains (RecL), analysed by EdrgeR.

Gene_id	Description	logFC_edgeR	PValue_edgeR	FDR	KeggBiomart_id	Kegg_id	Blastpanotation	blast2go_nr_NCBIanotation
AAEL0279	NA	-492.106.991.474.932	0.0250701728348245	0.258627500477641	NA	NA	NA	NA
AAEL0388	ubiquitin	-284.752.394.744.115	0.139217487232566	0.549112437854296	NA	NA	ETN61806.1 ubiquiti	XP_019543921.1 ubiquitin-like
AAEL0005	uncharacterized LOC5564310	-267.057.005.032.688	2,49E+09	0.00411330740050911	NA	NA	KXJ79612.1 hypothe	XP_021707947.1uncharacterized protein LOC5564310
AAEL0256	NA	-257.366.446.988.672	0.162373410840023	0.580707394694867	NA	NA	NA	XP_019564322.1 adult cuticle protein 1
AAEL0082	uncharacterized LOC5570373	-235.411.250.638.448	0.000155732767543407	0.0150847676519414	NA	NA	KXJ71287.1 hypothe	XP_021708161.1uncharacterized protein LOC110678888
AAEL0197	NA	-186.072.594.570.043	0.000132954591027418	0.0135938682959367	00564 ; 04070 ; 00561	aag01100 ; aag	NA	XP_021697553.1diacylglycerol kinase 1 isoform X1
AAEL0215	NA	-179.721.748.099.443	5,99E+07	0.000344255096041256	NA	NA	NA	XP_021706092.1uncharacterized protein LOC110678029
AAEL0269	NA	-178.147.270.162.833	3,37E+09	0.00499914174438099	NA	NA	NA	NA
AAEL0149	adult-specific cuticular protein ACP-20	-175.583.985.466.865	0.00244062783353945	0.0760003211995279	NA	NA	NA	XP_001650153.1adult-specific cuticular protein ACP-20
AAEL0204	NA	-168.179.327.189.133	0.000356010976739148	0.0230705141405186	NA	NA	NA	XP_021701632.1uncharacterized protein LOC110676785
AAEL0057	NADP-dependent malic enzyme	-161.870.025.842.822	2,14E+04	9,86E+08	NA	aag01100 ; aag	XP_019528944.1 PRE	XP_001651437.2NADP-dependent malic enzyme isoform X1
AAEL0039	calpain-3	-159.722.842.009.397	0.00233224522939274	0.0747232890974067	NA	NA	KXJ72177.1 hypothe	XP_019552586.1 calpain-B-like
AAEL0063	17-beta-hydroxysteroid dehydrogenase 13	-154.886.613.880.226	0.000407690936821104	0.0251783355746793	NA	NA	KXJ71453.1 hypothe	XP_001651887.117-beta-hydroxysteroid dehydrogenase 13
AAEL0154	cuticle protein 19	-152.009.564.657.436	0.000162496677083983	0.0151039840659274	NA	NA	NA	XP_001647747.1cuticle protein 19
AAEL0019	acidic amino acid decarboxylase GADL1	-151.016.559.375.312	0.000665318678101746	0.0345890535361145	NA	NA	KXJ68824.1 hypothe	XP_001654195.2cysteine sulfinic acid decarboxylase isoform X1
AAEL0194	NA	-14.752.327.099.114	2,34E+07	0.000278745599142822	NA	aag04711	NA	XP_021703089.1protein timeless isoform X9
AAEL0134	alpha-amylase 2	-147.156.690.169.191	5,53E+09	0.00726721156045196	00500 ; 00500	NA	NA	XP_001663616.1alpha-amylase 2
AAEL0219	NA	-143.530.503.802.673	0.0324475508321646	0.28463170766102	NA	NA	NA	XP_021703473.1skin secretory protein xP2-like
AAEL0036	sodium-dependent nutrient amino acid transporter 1	-141.293.955.315.648	5,06E+07	0.000323434375000257	NA	NA	NA	XP_021707062.1sodium-dependent nutrient amino acid transporter 1
AAEL0098	metabotropic glutamate receptor 2	-141.148.738.588.305	0.00116610589115951	0.047691139601999	NA	NA	NA	XP_021710165.1metabotropic glutamate receptor 2 isoform X1
AAEL0126	uncharacterized LOC5576619	-13.939.397.027.426	0.000351678566715284	0.0229513912830783	NA	NA	KXJ80517.1 hypothe	XP_001662773.1uncharacterized protein LOC5576619
AAEL0042	glutathione S-transferase theta-1	-13.785.661.407.891	0.0033881846154945	0.0897536781871071	00980 ; 00982 ; 00983 ;	aag01100 ; aag	KXJ73112.1 hypothe	XP_001648735.1glutathione S-transferase theta-1
AAEL0056	BTB/POZ domain-containing protein 6	-136.096.742.103.233	0.00812635536649933	0.145627738554632	NA	NA	NA	XP_021695510.1BTB/POZ domain-containing protein 6 isoform X2
AAEL0061	NA	-134.633.804.334.755	0.0491925680724099	0.352961636078127	NA	NA	KXJ81607.1 hypothe	XP_019529726.1 glutactin-like
AAEL0012	transient receptor potential cation channel subfamily A member 1	-133.901.978.422.926	0.00906143803733075	0.155276262233738	NA	NA	NA	XP_021698453.1transient receptor potential cation channel subfamily A member 1 isoform X5
AAEL0290	NA	-133.192.201.749.977	0.0315010069405959	0.28423411888152	NA	NA	NA	AHH41648.1cecropin N
AAEL0079	clavesin-1	-133.180.840.819.059	0.00233016768993799	0.0747232890974067	NA	NA	KXJ72702.1 hypothe	XP_019527170.1 clavesin-1-like
AAEL0043	uncharacterized LOC5564692	-132.368.894.889.437	0.00796379433443271	0.144528409372715	NA	NA	KXJ70623.1 hypothe	XP_021695521.1uncharacterized protein LOC5564692
AAEL0101	uncharacterized LOC5572926	-131.058.405.987.192	1,86E+09	0.00335601152845418	NA	NA	NA	XP_001654261.2uncharacterized protein LOC5572926
AAEL0038	defensin-A-like	-128.158.308.891.312	0.0764715373433454	0.422703978006619	NA	NA	NA	AAD40113.1defensin isoform A2
AAEL0269	NA	-127.546.443.470.566	0.112734157301008	0.503856556775916	NA	NA	NA	XP_021702767.1zinc finger BED domain-containing protein 1-like
AAEL0197	NA	-127.212.405.015.412	0.0384075226422404	0.309719436954163	NA	NA	NA	XP_001661920.1venom allergen 5
AAEL0134	uncharacterized LOC5577949	-127.011.969.854.516	7,07E+06	0.000130083198070057	NA	NA	NA	XP_001663622.2uncharacterized protein LOC5577949 isoform X2
AAEL0203	NA	-126.578.736.473.677	1,94E+08	0.000656607599064131	00010 ; 00640 ; 00620 ;	aag01100 ; aag	NA	XP_001655519.1 acetyl-coenzyme A synthetase
AAEL0106	MAP kinase phosphatase with leucine-rich repeats protein 1	-126.361.979.141.786	3,70E+06	0.000113559469648025	NA	NA	KXJ71180.1 hypothe	XP_001660999.1MAP kinase phosphatase with leucine-rich repeats protein 1
AAEL0078	protein EFR3 homolog cmp44E-like	-12.635.207.646.004	0.000451121178145331	0.0273106386927193	NA	NA	NA	XP_021708831.1protein EFR3 homolog cmp44E-like isoform X2
AAEL0280	NA	-121.025.784.843.701	0.000141962644559238	0.0138972367578096	NA	aag01100 ; aag	NA	XP_001656046.1alanine--glyoxylate aminotransferase 2-like
AAEL0013	72 kDa inositol polyphosphate 5-phosphatase	-119.450.455.431.582	0.195463864000161	0.62802321107873	NA	aag01100 ; aag	XP_019554361.1 PRE	XP_001653179.272 kDa inositol polyphosphate 5-phosphatase
AAEL0063	hybrid signal transduction histidine kinase B	-119.153.988.433.768	0.000186310878867899	0.0161738934654944	NA	NA	ETN63671.1 hypothe	XP_001651938.1hybrid signal transduction histidine kinase B
AAEL0212	NA	-116.952.375.870.072	0.00114447394185131	0.0475020022701074	NA	NA	NA	XP_019543697.1 60S ribosomal protein L7-like
AAEL0200	NA	-116.673.305.390.982	0.0133400810119755	0.18599306889727	NA	NA	NA	XP_021705671.1CCR4-NOT transcription complex subunit 3-like
AAEL0221	NA	-115.479.623.908.059	0.00866385905814469	0.150424209534052	NA	NA	NA	XP_021700652.1thyrostimulin alpha-2 subunit
AAEL0197	NA	-115.290.257.318.542	0.0131632365425958	0.184874636813685	NA	NA	NA	XP_001651766.1cytochrome c oxidase subunit 7A1, mitochondrial-like
AAEL0246	NA	-115.060.779.717.934	0.0116944440826392	0.173848585538685	NA	NA	NA	NA
AAEL0135	GDNF-inducible zinc finger protein 1	-114.633.077.490.041	0.012869169344141	0.182489812221969	NA	NA	NA	XP_021697486.1GDNF-inducible zinc finger protein 1 isoform X2
AAEL0000	phosphoenolpyruvate carboxykinase [GTP]	-112.956.962.237.196	2,50E+09	0.00411330740050911	00010 ; 00020 ; 00620	aag01100 ; aag	KXJ70379.1 hypothe	XP_001647935.2phosphoenolpyruvate carboxykinase [GTP]
AAEL0036	serine protease easter	-111.749.694.678.049	0.00339429254608925	0.0897536781871071	NA	NA	XP_019541654.1 PRE	XP_001657086.2serine protease easter
AAEL0257	NA	-111.289.013.855.761	0.0145527133035641	0.193517439045371	NA	NA	NA	XP_021694025.1tRNA-specific adenosine deaminase 2 isoform X6
AAEL0103	GILT-like protein 3	-110.585.152.098.339	0.00989389966343727	0.161167614967993	NA	NA	NA	XP_001654520.1GILT-like protein 3
AAEL0260	NA	-109.921.060.347.697	5,27E+07	0.000323434375000257	NA	NA	NA	XP_021696765.1uncharacterized protein LOC110675610
AAEL0251	NA	-109.378.217.309.256	0.130446671430517	0.537318831917466	NA	NA	NA	---NA---
AAEL0048	probable chitinase 10	-10.632.965.017.141	0.0283545701452429	0.272927567444064	520	aag01100 ; aag	KFB36184.1 AGAP00	XP_021696877.1probable chitinase 10
AAEL0207	NA	-105.744.095.265.038	1,30E+08	0.000531236704279967	NA	NA	NA	XP_021697925.1uncharacterized protein LOC110676001
AAEL0219	NA	-104.655.096.431.623	0.00332640339630247	0.0895016492771208	NA	NA	NA	XP_021711894.1neurofilament heavy polypeptide-like
AAEL0006	peptide methionine sulfoxide reductase	-104.531.119.006.958	1,28E+09	0.00244895569246464	NA	NA	KFB50153.1 AGAP01	XP_001649797.1peptide methionine sulfoxide reductase
AAEL0145	pancreatic lipase-related protein 2	-104.294.245.600.171	7,74E+07	0.000399983581196831	NA	NA	NA	XP_021694874.1pancreatic lipase-related protein 2
AAEL0034	regulator of microtubule dynamics protein 1	-103.943.791.675.291	6,87E+08	0.00158071679752556	NA	NA	KXJ75853.1 hypothe	XP_001656801.1regulator of microtubule dynamics protein 1
AAEL0032	acyl-CoA Delta(11) desaturase	-102.200.182.926.213	8,69E+07	0.000399983581196831	NA	aag01100 ; aag	XP_001864523.1 acy	XP_021711176.1acyl-CoA Delta(11) desaturase isoform X1
AAEL0204	NA	-101.969.766.214.713	0.00050167770239264	0.0290898537620388	NA	NA	NA	XP_021694055.1venom protease-like
AAEL0024	tyrosine-protein kinase receptor torso	-101.051.775.669.785	0.162708509554998	0.581228146321853	NA	aag04013	XP_019539349.1 PRE	XP_021699633.1tyrosine-protein kinase receptor torso isoform X2
AAEL0020	uncharacterized LOC5573385	-100.824.748.710.463	0.0115308499458727	0.173372577261871	NA	NA	KXJ73009.1 hypothe	XP_019547421.1 probable serine/threonine-protein kinase tsuA
AAEL0263	NA	-100.816.572.642.697	0.290700804803279	0.722005075789413	NA	NA	NA	XP_021703346.1adult cuticle protein 1-like
AAEL0007	ficolin-3-like	-100.612.656.450.163	0.0322775071150141	0.284499636467777	NA	NA	AAV90674.1 angiopo	ABF18025.1angiopoietin-like protein variant
AAEL0268	NA	-100.488.402.411.059	9,64E+08	0.00206366028950833	NA	NA	NA	XP_021696766.1uncharacterized protein LOC110675611
AAEL0124	probable elastin-binding protein EbpS	-100.428.404.360.855	0.000272915387585317	0.0199314872742864	NA	NA	XP_019932228.1 PRE	ABF18171.1putative salivary mucin 6
AAEL0273	NA	-100.417.735.594.873	0.000563253010873661	0.0312232181087918	NA	NA	NA	NA
AAEL0205	NA	-0.995419882563193	0.000515990241661443	0.029253144144659	NA	NA	NA	XP_001661441.2pancreatic lipase-related protein 2
AAEL0089	uncharacterized LOC5571377	-0.99212235141645	0.0193557649318294	0.226893947646744	NA	NA	KXJ79893.1 hypothe	XP_001659659.2uncharacterized protein LOC5571377
AAEL0208	NA	-0.99005425626586	0.0212830368621418	0.2382560890577	NA	NA	NA	NA
AAEL0009	proprotein convertase subtilisin/kexin type 5	-0.986657318607085	0.000220969156846934	0.0181549837616561	NA	NA	KXJ74045.1 hypothe	XP_021707974.1proprotein convertase subtilisin/kexin type 5
AAEL0039	calpain-A	-0.981777718590641	0.000518176754572856	0.029253144144659	NA	NA	KXJ72175.1 hypothe	XP_021706015.1calpain-A isoform X1
AAEL0011	fatty acid synthase	-0.976857838284359	8,41E+07	0.000399983581196831	00780 ; 00061	aag01100 ; aag	KXJ76270.1 hypothe	XP_001658180.2fatty acid synthase
AAEL0120	uncharacterized LOC5575791	-0.972172868945048	0.0120007149103345	0.176972081097594	NA	NA	NA	XP_001662213.2uncharacterized protein LOC5575791
AAEL0095	glucose-6-phosphate 1-dehydrogenase	-0.97206531846485	1,35E+08	0.000531236704279967	00480 ; 00030	aag01100 ; aag	NA	XP_021704708.1glucose-6-phosphate 1-dehydrogenase isoform X1
AAEL0044	uncharacterized LOC5564886	-0.969777939029656	0.00554627084926411	0.119524085140347	NA	NA	KXJ71041.1 hypothe	XP_019562109.1 platelet glycoprotein Ib alpha chain-like
AAEL0123	cyclin-dependent kinase 2	-0.958234654051533	0.0252543393441847	0.259075173517489	NA	NA	KXJ69159.1 hypothe	XP_001662490.1cyclin-dependent kinase 2
AAEL0114	clavesin-2	-0.957066587292822	9,60E+09	0.0110128080760421	NA	NA	KXJ75161.1 hypothe	XP_019527135.1 clavesin-1-like
AAEL0078	cytochrome P450 4C1	-0.954802514596368	0.00483888538577811	0.110216394356263	NA	NA	KXJ78686.1 hypothe	XP_001652922.1cytochrome P450 4C1
AAEL0036	sodium-dependent nutrient amino acid transporter 1	-0.944915915371567	9,23E+09	0.0110128080760421	NA	NA	NA	XP_021705991.1sodium-dependent nutrient amino acid transporter 1
AAEL0106	uncharacterized LOC5573655	-0.94224127629007	0.000469583282083719	0.0280591257255479	NA	NA	KXJ75680.1 hypothe	XP_021709307.1uncharacterized protein LOC5573655

AAEL0005!	uncharacterized LOC5564316	-0.94046378092808	0.0280587601249555	0.271500221524543	NA	NA	XP_001848667.1 bea	XP_001848667.1beat protein
AAEL0279	NA	-0.93652860075636	0.0673166218042629	0.4007529665455	NA	NA	NA	XP_021698969.1paternally-expressed gene 3 protein
AAEL0114	probable sulfoacetate transporter SauU	-0.935842069450981	2,86E+08	0.000877435471211908	NA	NA	KFB40807.1 AGAP00	XP_001865723.1cis,cis-muconate transport protein MucK
AAEL0064!	NA	-0.929652541174147	6,72E+06	0.000130083198070057	NA	NA	KXJ72000.1 hypothe	XP_019530717.2 callisulfakinin
AAEL0115!	serine protease snake	-0.927006690042321	0.0465583388033014	0.344268052476481	NA	NA	XP_001844337.1 vita	XP_001661765.2serine protease snake
AAEL0088	selenide, water dikinase	-0.924235786917341	2,78E+07	0.000278745599142822	450	aag01100 ; aag	KFB49604.1 AGAP00	XP_001653469.1selenide, water dikinase
AAEL0069	chymotrypsin-1	-0.922237430579311	0.073739794128545	0.417913179290884	NA	NA	KXJ73565.1 hypothe	XP_019532832.1 chymotrypsin-1-like
AAEL0000	phosphoenolpyruvate carboxykinase [GTP]	-0.918776867917819	0.00474541894422196	0.108625236628683	00010 ; 00020 ; 00620	aag01100 ; aag	KXJ74853.1 hypothe	XP_001647937.2phosphoenolpyruvate carboxykinase [GTP]
AAEL0173	glycine-rich protein 23	-0.914783229284762	0.399163123414693	0.798648539435638	NA	NA	NA	XP_011493150.1glycine-rich protein 23
AAEL0076	plant intracellular Ras-group-related LRR protein 7	-0.911381543009482	0.0179420549824589	0.217240513090246	NA	NA	KXJ68680.1 hypothe	XP_021711673.1plant intracellular Ras-group-related LRR protein 7
AAEL0145	NA	-0.910841849476223	0.0108806983510681	0.168274304687137	NA	NA	NA	XP_001648916.2seminal metalloprotease 1
AAEL0172!	larval/pupal cuticle protein H1C	-0.910690258079843	6,00E+09	0.00756191347870889	NA	NA	NA	XP_011493509.1larval/pupal cuticle protein H1C
AAEL0103	UDP-glucuronosyltransferase 1-7	-0.910159584258336	1,17E+08	0.000511805769253077	NA	NA	NA	XP_001654498.2UDP-glucuronosyltransferase 1-7
AAEL0208!	NA	-0.909026511845381	0.0284012574323965	0.272967995267809	NA	NA	NA	XP_019540340.1 nephrin-like
AAEL0058	activator of 90 kDa heat shock protein ATPase homolog 1-like	-0.905929622209419	0.0114528988022704	0.172833431546296	NA	NA	KXJ68444.1 hypothe	XP_021712254.1activator of 90 kDa heat shock protein ATPase homolog 1-like
AAEL0142	UDP-glucuronosyltransferase 2B1	-0.904049464164703	3,65E+07	0.000305489628732982	NA	aag01100 ; aag	NA	XP_021696237.1UDP-glucuronosyltransferase isoform X1
AAEL0070!	pyridoxal phosphate phosphatase	-0.901276103475141	4,17E+08	0.00580768352948965	NA	aag00981	KXJ81040.1 hypothe	XP_001658123.1pyridoxal phosphate phosphatase isoform X1
AAEL0044	probable serine hydrolase	-0.901150585722499	0.00280894497003887	0.0820568622676118	NA	NA	KXJ72299.1 hypothe	XP_001649279.2probable serine hydrolase
AAEL0183	NA	-0.8987075692814	0.0325501037783737	0.28463170766102	NA	NA	NA	XP_021700991.1sodium/potassium/calcium exchanger Nckx30C isoform X2
AAEL0227	NA	-0.889446618711834	0.0497725936428855	0.355143569906322	NA	NA	NA	XP_021698482.1uncharacterized transmembrane protein DDB_G0289901-like isoform X1
AAEL0194	NA	-0.888570461462982	0.00223363611939506	0.074201875706402	NA	NA	NA	AAY21238.1lysozyme c-6
AAEL0034	maltase A3	-0.887408865968381	0.000104539801862617	0.0115900633342145	NA	NA	XP_001851486.1 alp	XP_001656785.2maltase A3
AAEL0050	multidrug resistance-associated protein 1	-0.885768927728429	0.000505732569361893	0.0290898537620388	NA	NA	KXJ83954.1 hypothe	XP_001650217.2multidrug resistance-associated protein 1 isoform X2
AAEL0171	uncharacterized LOC23687559	-0.88397374097929	0.00112569625686327	0.04722490950323	NA	NA	NA	KFB38856.1AGAP013348-like protein
AAEL0087!	F-box/SPRY domain-containing protein 1	-0.883705518412161	0.0514203578852335	0.359690131564899	NA	NA	KFB45932.1 AGAP01	XP_001659457.2F-box/SPRY domain-containing protein 1
AAEL0186!	NA	-0.883063744061782	0.502368871686883	0.858744381072989	190	aag01100 ; aag	NA	YP_009389260.1NADH dehydrogenase subunit 2 (mitochondrion)
AAEL0129!	protein D3	-0.872954948936346	3,31E+08	0.000951482456326449	NA	NA	NA	XP_001663135.1protein D3
AAEL0093	solute carrier family 46 member 3	-0.852813283364671	0.0225068362058328	0.243943353081359	NA	NA	NA	XP_001659917.1solute carrier family 46 member 3
AAEL0048	uncharacterized LOC5565490	-0.851043021790185	0.0230025482021539	0.246091471282416	NA	NA	KFB47345.1 AGAP01	KFB47345.1AGAP010540-like protein
AAEL0035(	transcription factor AP-1	-0.848406598380842	5,11E+07	0.000323434375000257	NA	aag04013 ; aag	XP_019536966.1 PRE	XP_001663792.2transcription factor AP-1 isoform X2
AAEL0044(	alpha-L-iduronidase	-0.848002257449442	0.0457378146054671	0.340242012934121	NA	aag01100 ; aag	KXJ74538.1 hypothe	XP_019548444.1 alpha-L-iduronidase-like
AAEL0042!	ATP-citrate synthase	-0.845891293775601	7,71E+08	0.00168993348751206	00720 ; 00640 ; 00020 ;	aag01100 ; aag	XP_001842482.1 ATF	XP_001648848.1ATP-citrate synthase isoform X2
AAEL0043	esterase B1	-0.844647117603614	0.000101729274497351	0.0114160095600564	NA	NA	KXJ68275.1 hypothe	XP_001648942.1esterase B1
AAEL0009	fatty acyl-CoA reductase wat	-0.843907970407149	0.0221404949187772	0.242070925206103	73	NA	KXJ72100.1 hypothe	XP_001651611.1fatty acyl-CoA reductase wat
AAEL0012	probable metabolite transport protein CsbC	-0.842914363147688	1,58E+09	0.00296561476447668	NA	NA	KXJ78750.1 hypothe	XP_019542173.1 facilitated trehalose transporter Tret1-like
AAEL0209	NA	-0.841008547426526	0.0103424490440876	0.163805879696547	NA	NA	NA	NA
AAEL0083	ATP-binding cassette sub-family A member 3	-0.836569036874643	0.0122710541138635	0.179285423221345	NA	NA	XP_001851801.1 ATF	XP_021709756.1ATP-binding cassette sub-family A member 3 isoform X1
AAEL0200	NA	-0.835894012259378	0.0945376396456515	0.465640264332134	NA	NA	NA	---NA---
AAEL0222!	NA	-0.835459562886397	0.0303120259457684	0.280995099606986	NA	aag04080	NA	XP_021704405.1histamine H2 receptor
AAEL0181	NA	-0.834945894463772	0.029684417842625	0.278209491466411	NA	NA	NA	XP_021706157.1voltage-dependent T-type calcium channel subunit alpha-1G isoform X3
AAEL0038	defensin-C	-0.828293247785244	0.094487631338425	0.465640264332134	NA	NA	KXJ76862.1 hypothe	AAD40116.2defensin isoform C1
AAEL0257	NA	-0.824395893910884	0.0319046734058026	0.284442054201394	NA	NA	NA	XP_021696581.1uncharacterized protein LOC110675564
AAEL0007	fibrinogen-like protein A	-0.823682720130574	0.000569459030366809	0.0313581563638191	NA	NA	KXJ79468.1 hypothe	XP_001650669.1fibrinogen-like protein A
AAEL0037	uncharacterized LOC5578921	-0.822537330041396	3,46E+08	0.000965310188524951	NA	NA	KXJ75767.1 hypothe	XP_019526410.1 neurofilament medium polypeptide-like
AAEL0103	gastrula zinc finger protein XlCGF48.2	-0.821624256221337	0.000952222703617745	0.0425356957217985	NA	NA	NA	XP_001654447.1gastrula zinc finger protein XlCGF48.2
AAEL0026!	72 kDa type IV collagenase	-0.821570765171952	0.00335006832654969	0.0896143277352042	NA	NA	KXJ68268.1 hypothe	XP_001662052.172 kDa type IV collagenase
AAEL0127!	mucin-17	-0.818066396475101	0.00695042414736882	0.137411930736898	NA	NA	XP_019553095.1 PRE	XP_021710732.1mucin-5AC isoform X2
AAEL0124	organic cation transporter protein	-0.815356603006453	0.00191429823636787	0.0684803907362971	NA	NA	KXJ76179.1 hypothe	XP_001662579.2organic cation transporter protein
AAEL0198	NA	-0.813733718600699	2,00E+07	0.000656607599064131	NA	NA	NA	XP_021695644.1uncharacterized protein LOC5578209
AAEL0124	probable 4-coumarate--CoA ligase 1	-0.813485515679697	0.0211099599616659	0.23689494093567	NA	NA	XP_019525713.1 PRE	XP_001662564.24-coumarate--CoA ligase-like 9
AAEL0274	NA	-0.813010302284996	0.0876326950502832	0.452523041443718	NA	NA	NA	XP_021703451.1netrin-B isoform X1
AAEL0236!	NA	-0.812473709331699	0.0628609790288642	0.391901577929274	NA	NA	NA	XP_019556495.1 mucin-5AC-like
AAEL0206	NA	-0.811108103652339	0.0196757467920266	0.227335564593981	NA	NA	NA	XP_021703888.1zinc finger protein ush
AAEL0212	NA	-0.806942766964415	0.000800874391460909	0.038584534817923	NA	NA	NA	ABF18036.1gSG8-like protein
AAEL0008	probable G-protein coupled receptor Mth-like 14	-0.806520708547848	0.000579905132266155	0.0315756628823264	NA	NA	KXJ81441.1 hypothe	XP_001651304.3probable G-protein coupled receptor Mth-like 14
AAEL0066	fibrinogen-like protein 1	-0.805961329835862	5,03E+08	0.00132200691337758	NA	NA	XP_019539661.1 PRE	XP_001657949.2fibrinogen-like protein 1
AAEL0270!	NA	-0.804303690108387	2,81E+08	0.00445101312117406	NA	NA	NA	---NA---
AAEL0279	NA	-0.803577529623324	0.00763184153390241	0.142450721693651	NA	NA	NA	NA
AAEL0248	NA	-0.79977447492361	0.08536692395981	0.443811544789927	NA	NA	NA	NA
AAEL0242!	NA	-0.799604329758633	0.00175976278441776	0.0640052851470837	NA	NA	NA	XP_001659917.1solute carrier family 46 member 3
AAEL0066	uncharacterized LOC5568174	-0.799503412346585	0.0037765020138555	0.0954708009107098	901	NA	KXJ68636.1 hypothe	XP_021703903.1uncharacterized protein LOC5568174 isoform X1
AAEL0183	NA	-0.798139963953808	0.000905027863715604	0.0413193578047436	NA	NA	NA	XP_021702546.1uncharacterized protein LOC5566796
AAEL0194	NA	-0.796713471816883	0.0136126395578813	0.187831676988403	NA	NA	NA	XP_021711444.1uncharacterized protein LOC5565987 isoform X7
AAEL0115	condensin-2 complex subunit D3	-0.795108254453843	0.0128378500426682	0.182489812221969	NA	NA	KXJ81720.1 hypothe	XP_021711057.1condensin-2 complex subunit D3
AAEL0061	melanization protease 1	-0.794893123720075	0.00205508437737934	0.0710935580475365	NA	NA	XP_019551990.1 PRE	XP_001657551.2melanization protease 1
AAEL0010	uncharacterized LOC5579916	-0.789388652981171	0.0635010118174791	0.393227665373111	NA	NA	XP_019528949.1 PRE	XP_019528950.1 constitutive coactivator of PPAR-gamma-like protein 1 isoform X2
AAEL0098	uncharacterized LOC5572540	-0.787917668527246	0.00233742284998388	0.0747232890974067	00564 ; 00591 ; 00565 ;	NA	NA	XP_001660476.2uncharacterized protein LOC5572540
AAEL0016	zinc finger protein ubi-d4	-0.787616501537257	0.0405339788074493	0.316225178415075	NA	NA	XP_019543335.1 PRE	XP_001659759.2zinc finger protein ubi-d4
AAEL0129	transferrin	-0.787004973215819	0.0173652489731798	0.214247748888425	NA	NA	NA	XP_019555266.1 transferrin-like
AAEL0112	protein D3	-0.7864336095592	0.00265238980759574	0.0792437883464128	NA	NA	XP_001850222.1 pho	XP_001661541.1protein D3
AAEL0077	potassium voltage-gated channel subfamily H member 8	-0.786367112506735	0.0053580002684943	0.117391234454011	NA	NA	NA	XP_021699749.1potassium voltage-gated channel subfamily H member 8 isoform X2
AAEL0059!	putative tricarboxylate transport protein, mitochondrial	-0.784536298719288	3,65E+08	0.000988185298028416	NA	NA	ETN58853.1 tricarbo	XP_001657348.1putative tricarboxylate transport protein, mitochondrial
AAEL0014	neurotrophin 1	-0.782979839597241	0.059535679978379	0.382389109224341	NA	NA	KXJ73752.1 hypothe	XP_001659184.2neurotrophin 1
AAEL0207	NA	-0.78265505962126	0.0284620008666793	0.273104621454831	NA	NA	NA	XP_021701990.1probable palmitoyltransferase ZDHHC24
AAEL0205	NA	-0.78226120177668	0.000166955541480016	0.0152111375514763	NA	NA	NA	XP_021697452.1integral membrane protein GPR155
AAEL0063	MAM and LDL-receptor class A domain-containing protein 1	-0.780657072637201	0.0176010910946993	0.214955509175633	NA	NA	KXJ82331.1 hypothe	XP_001651940.1MAM and LDL-receptor class A domain-containing protein 1
AAEL0257	NA	-0.780061969040769	0.000161206004390615	0.0151039840659274	NA	NA	NA	XP_021704240.1microfibril-associated glycoprotein 4-like
AAEL0170	RYamide receptor	-0.779448420102396	0.112960973879102	0.503928507434265	NA	NA	NA	XP_021696098.1RYamide receptor
AAEL0059!	phospholipase B1, membrane-associated	-0.778605880610186	0.0098822218698503	0.161167614967993	00564 ; 00591 ; 00565 ;	NA	KXJ79591.1 hypothe	XP_001651743.2phospholipase B1, membrane-associated
AAEL0183	NA	-0.77734505335678	0.00224730239405084	0.0743873260073952	NA	NA	NA	XP_021710995.1regulating synaptic membrane exocytosis protein 1 isoform X1
AAEL0205	NA	-0.774820981092964	0.000572502746046687	0.0313581563638191	NA	NA	NA	XP_021696179.1biofilm and cell wall regulator 1-like
AAEL0232!	NA	-0.774722589443676	0.0446838685216522	0.33483791379173	NA	NA	NA	XP_021703923.1zinc finger protein 135 isoform X2

AAEL00698	uncharacterized LOC5568619	-0.773338717935802	0.0267289258726645	0.266963842267995	NA	NA	KFB35774.1 hypothe	XP_001652453.1uncharacterized protein LOC5568619
AAEL00195	actin, indirect flight muscle	-0.773075036146474	0.0105022113881816	0.165036735008546	NA	aag04145 ; aag	KFB42600.1 AGAP00	XP_001654312.1actin, indirect flight muscle
AAEL00392	uncharacterized LOC5563775	-0.772866294936156	0.0228022581727474	0.245124275357034	NA	NA	KXJ74511.1 hypothet	XP_001648054.1uncharacterized protein LOC5563775
AAEL00106	myosin regulatory light chain, striated adductor muscle	-0.771496902300265	0.0543523398756312	0.367469712613638	NA	NA	ETN60400.1 myosin l	XP_001658012.1myosin regulatory light chain, striated adductor muscle
AAEL00504	uncharacterized LOC5565847	-0.770618623817553	0.0481697336101821	0.349572467413955	NA	NA	NA	XP_001650199.1uncharacterized protein LOC5565847
AAEL01995	NA	-0.767136850579432	0.0835323866158979	0.438667571938958	NA	NA	NA	XP_021706588.1 solute carrier family 26 member 10-like
AAEL02754	NA	-0.766964259552864	0.005007123133538	0.112922895714546	NA	NA	NA	XP_021697289.1nose resistant to fluoxetine protein 6
AAEL02660	NA	-0.766615607046891	0.00765105274652191	0.142520217355252	NA	NA	NA	XP_021695165.1transient receptor potential cation channel protein painless isoform X1
AAEL00260	SPRY domain-containing SOCS box protein 3	-0.766302829600296	0.0012456448223946	0.0494069985158408	NA	NA	KFB41674.1 hypothe	XP_001655691.1SPRY domain-containing SOCS box protein 3
AAEL01398	uncharacterized LOC5579043	-0.76257499995724	1,39E+08	0.000531236704279967	NA	NA	NA	XP_001657236.2uncharacterized protein LOC5579043
AAEL02235	NA	-0.762201166643912	0.0902513029466064	0.458582269306832	NA	NA	NA	XP_021703551.1tyrosine-protein phosphatase non-receptor type 23
AAEL02031	NA	-0.761802629409377	0.00351088588221991	0.0907504828319877	NA	NA	NA	XP_021712674.1protein NPC2 homolog
AAEL02019	NA	-0.760618670241926	0.0190360328816311	0.225171411092632	NA	NA	NA	XP_021711960.1SAM50-like protein CG7639
AAEL02278	NA	-0.759004886580877	0.0646316875141073	0.39649385900321	NA	NA	NA	XP_021700695.1ADAMTS-like protein 1 isoform X3
AAEL00129	clavesin-1	-0.755600612491062	0.00252307297717641	0.0776498914246733	NA	NA	NA	AAK73352.1CRALBP isoform 2
AAEL02126	NA	-0.750771777640689	0.0330015213175165	0.285413533048672	NA	NA	NA	NA
AAEL01017	leucokinins	-0.747890945500024	0.0808870826499014	0.433079891352508	NA	NA	NA	O02036.1RecName: Full=Leucokinins; Contains: RecName: Full=Leucokinin-1; Contains: RecName: Full=Leucokinin-3;
AAEL02655	NA	-0.74711943159431	0.125221782078145	0.530276501925028	NA	NA	NA	NA
AAEL01357	uncharacterized LOC5578233	-0.744891643169221	0.000108557253837273	0.0117522805860069	NA	NA	NA	XP_001663757.1uncharacterized protein LOC5578233
AAEL00183	sodium-coupled monocarboxylate transporter 1	-0.744829140047698	0.00179037135107663	0.0648621935929416	NA	NA	KXJ68697.1 hypothet	XP_001660396.2sodium-coupled monocarboxylate transporter 1
AAEL02725	NA	-0.744604713374996	0.022363557832905	0.242962761721832	NA	NA	NA	XP_021694815.1cartilage oligomeric matrix protein-like
AAEL02707	NA	-0.742895978237507	0.057110274207189	0.375057077651547	NA	NA	NA	XP_019530408.1 PHD finger protein rhinoceros-like isoform X2
AAEL02627	NA	-0.741953061268369	5,10E+09	0.00680489671107962	NA	NA	NA	XP_021706226.1protein takeout isoform X1
AAEL02169	NA	-0.739963083082439	0.042320847802709	0.323939778330243	NA	NA	NA	NA
AAEL00492	neuronal acetylcholine receptor subunit beta-3	-0.739235670107838	0.0277163506303136	0.271096286901832	NA	NA	KXJ76019.1 hypothet	XP_001650051.2neuronal acetylcholine receptor subunit beta-3
AAEL02643	NA	-0.738775699880214	0.0136900142449281	0.187831676988403	NA	NA	NA	XP_021700252.1uncharacterized protein LOC5575879 isoform X2
AAEL02466	NA	-0.737834237443612	0.0043645083921241	0.103594442242845	NA	NA	NA	XP_021702234.1pupal cuticle protein 20
AAEL00975	protein msta	-0.735599211006605	0.00528544281625891	0.116209032387834	NA	NA	NA	XP_001660316.1protein msta isoform X1
AAEL00917	beta-1,3-glucan-binding protein	-0.735559608886772	0.0079572933087275	0.144528409372715	NA	NA	KXJ79664.1 hypothet	XP_001659797.2beta-1,3-glucan-binding protein
AAEL02190	NA	-0.735257232549155	0.0724917876160855	0.413814782657084	NA	NA	NA	XP_021702720.1sushi, von Willebrand factor type A, EGF and pentraxin domain-containing protein 1
AAEL02061	NA	-0.735189792960008	0.020074073686658	0.229753266249536	NA	NA	NA	XP_019542173.1 facilitated trehalose transporter Tret1-like
AAEL00635	sulfotransferase family cytosolic 1B member 1	-0.734532783516921	0.0442937047923951	0.332998914623873	NA	NA	KXJ69441.1 hypothet	XP_021710674.1sulfotransferase family cytosolic 1B member 1
AAEL00323	larval cuticle protein LCP-30	-0.734178014556904	3,05E+09	0.00475732094034817	NA	NA	KXJ76682.1 hypothet	XP_011492990.2larval cuticle protein LCP-30
AAEL02263	NA	-0.732876755772639	5,10E+09	0.00680489671107962	NA	NA	NA	XP_021711922.1LOW QUALITY PROTEIN: uncharacterized protein LOC110674016
AAEL00671	BCL2/adenovirus E1B 19 kDa protein-interacting protein 3	-0.732778587659755	6,63E+08	0.00156538082998626	NA	aag04140 ; aag	XP_019525149.1 PRE	XP_001657980.1BCL2/adenovirus E1B 19 kDa protein-interacting protein 3 isoform X2
AAEL00745	cartilage oligomeric matrix protein	-0.729831161266828	0.00426335971916707	0.101899834118897	NA	NA	XP_001866775.1 thr	XP_021698876.1cartilage oligomeric matrix protein
AAEL01984	NA	-0.729117035997825	0.00943835660074098	0.158773875613038	NA	aag04320	NA	XP_021693473.1cytoplasmic polyadenylation element-binding protein 2
AAEL00776	39S ribosomal protein L40, mitochondrial-like	-0.727683076974983	0.00799609757036293	0.144528409372715	NA	NA	KXJ71920.1 hypothet	XP_001658631.239S ribosomal protein L40, mitochondrial
AAEL00868	uncharacterized LOC5570944	-0.727465379837883	0.0229188912984717	0.245803773576383	NA	NA	KXJ81659.1 hypothet	XP_021699203.1uncharacterized protein LOC5570944
AAEL00356	L-threonine ammonia-lyase	-0.727129458433198	0.0257817226459188	0.262052657185525	NA	NA	KXJ72210.1 hypothet	XP_001663948.2uncharacterized protein LOC5578556
AAEL00705	lipase member H	-0.726195841194431	4,08E+09	0.00577271140867643	NA	NA	XP_001845804.1 par	XP_001652526.1lipase member H
AAEL01953	NA	-0.722725244189785	0.00979471301773438	0.160948123552128	NA	NA	NA	XP_021698987.1guanine nucleotide-binding protein G(f) subunit alpha
AAEL01354	zinc finger protein 2 homolog	-0.722686731423774	0.067371125255702	0.4007529665455	NA	NA	NA	XP_021696078.1zinc finger protein 2 homolog
AAEL01461	cytochrome P450 9e2	-0.721923826879402	0.000263650028683666	0.0195653835802185	NA	NA	NA	XP_001649095.1cytochrome P450 9e2
AAEL00157	monocarboxylate transporter 12	-0.72130020468357	0.0631204631930171	0.39272109689124	NA	NA	XP_001842007.1 mo	XP_001653584.2monocarboxylate transporter 12
AAEL00362	sodium-dependent nutrient amino acid transporter 1	-0.719767251022711	0.0316496704018788	0.28423411888152	NA	NA	NA	XP_021710856.1sodium-dependent nutrient amino acid transporter 1
AAEL01038	esterase B1	-0.719331108834029	0.0250283006884006	0.258627500477641	NA	NA	NA	XP_001654509.1esterase B1
AAEL01135	nuclear factor of activated T-cells 5	-0.718961661683409	0.00954541918278973	0.158773875613038	NA	NA	NA	XP_021694711.1nuclear factor of activated T-cells 5 isoform X1
AAEL02573	NA	-0.718656872513166	0.0418759474664839	0.323199464702074	NA	NA	NA	NA
AAEL01746	angiotensin-converting enzyme	-0.717622247733494	0.00456414658383662	0.105525821267499	NA	NA	NA	XP_011493088.2angiotensin-converting enzyme
AAEL02669	NA	-0.717400457436334	0.20481060103304	0.637782699712521	NA	NA	NA	NA
AAEL00590	UDP-glucuronosyltransferase 2B15	-0.716874102712033	0.000111595644311885	0.0119407339413717	NA	NA	KXJ77283.1 hypothet	XP_001651580.2UDP-glucuronosyltransferase 2B15
AAEL00870	inositol oxygenase	-0.714965009914049	0.000318647246516123	0.0218820295704579	00053 ; 00562	aag01100 ; aag	KXJ77859.1 hypothet	XP_021706632.1inositol oxygenase
AAEL00901	probable cytochrome P450 6a14	-0.714494250336343	0.000244675076668022	0.0191849588638347	NA	NA	KXJ79383.1 hypothet	XP_001659661.2probable cytochrome P450 6a14
AAEL00842	cadherin-99C	-0.712760248966317	0.0741592401311024	0.418818002133309	NA	NA	KXJ68278.1 hypothet	XP_019525112.1 protocadherin-15-like
AAEL00055	perlucin	-0.712608399249929	0.000302978186070401	0.0211740264428904	NA	NA	AAV90641.1 putative	ABF18034.1putative salivary C-type lectin
AAEL00671	alpha-amylase I	-0.710628740606159	0.00678349822796267	0.13569945802981	00500 ; 00500	aag01100 ; aag	KXJ83067.1 hypothet	XP_001652172.1alpha-amylase I
AAEL02588	NA	-0.70984109547304	0.0336457382527593	0.288275682869545	NA	NA	NA	XP_021708085.1uncharacterized protein LOC110678861
AAEL00285	CCAAT/enhancer-binding protein	-0.70699455759465	0.00115115697742164	0.0475020022701074	NA	NA	KXJ80566.1 hypothet	XP_021705485.1CCAAT/enhancer-binding protein
AAEL00569	ejaculatory bulb-specific protein 3	-0.706559579789831	0.0220896562181706	0.242070925206103	NA	NA	KXJ81186.1 hypothet	XP_001651339.2ornithine decarboxylase isoform X1
AAEL00065	beta-1,3-glucan-binding protein 2	-0.706217745172556	0.0759618222116222	0.421847126126341	NA	aag04624	ETN63242.1 Gram ne	XP_021710530.1beta-1,3-glucan-binding protein 2
AAEL00840	26S proteasome non-ATPase regulatory subunit 10	-0.704591009081376	0.0501806767243743	0.356468207181388	NA	NA	KXJ72846.1 hypothet	XP_001653259.226S proteasome non-ATPase regulatory subunit 10
AAEL00853	acetylcholinesterase	-0.703006288739714	0.054824010670589	0.367972681393698	NA	NA	KXJ80906.1 hypothet	XP_019548135.1 juvenile hormone esterase-like
AAEL00313	uncharacterized LOC5577223	-0.70299366593377	0.104323668095631	0.487550225401725	NA	NA	NA	XP_021708607.1uncharacterized protein LOC5577223
AAEL02181	NA	-0.698546188710371	0.00806648047615918	0.144976080745345	NA	NA	NA	NA
AAEL02119	NA	-0.69648090492471	0.0123342178402451	0.179504888805347	NA	NA	NA	KFB48573.1AGAP005783-like protein
AAEL01441	cuticle protein CP14.6	-0.69372014745882	0.00416070364047616	0.100227211779219	NA	NA	NA	XP_021702230.1cuticle protein CP14.6
AAEL00404	uncharacterized LOC5564061	-0.691406271855806	0.00944811836715776	0.158773875613038	NA	NA	XP_001869600.1 con	XP_001648364.2uncharacterized protein LOC5564061
AAEL00711	protein pinocchio	-0.689916945110019	5,79E+08	0.00147879697348873	NA	NA	XP_019550483.1 PRE	XP_001652547.1protein pinocchio isoform X2
AAEL02213	NA	-0.688925136344593	0.0334644900063795	0.287794614054863	NA	NA	NA	XP_021708318.1pancreatic lipase-related protein 2
AAEL01330	integumentary mucin C.1	-0.688122366855238	0.0555479976468126	0.370725518234274	NA	NA	NA	XP_021698056.1integumentary mucin C.1
AAEL02078	NA	-0.688046363998973	0.000140646616130955	0.0138972367578096	NA	NA	NA	KFB38955.1AGAP000420-like protein
AAEL02629	protein toll	-0.686854881284731	0.00323643342334062	0.0889935739723594	NA	aag04624	NA	XP_021708718.1protein toll
AAEL01454	integrator complex subunit 14	-0.686486947877929	0.0170046791453244	0.21168364694759	NA	NA	NA	XP_001648973.2integrator complex subunit 14
AAEL00834	uncharacterized LOC5570448	-0.68633237955169	0.0015822021518439	0.0584715831376209	NA	NA	KFB42065.1 AGAP00	KFB42065.1AGAP000717-like protein
AAEL00064	maltase A3	-0.686251738534713	0.000504667874313437	0.0290898537620388	NA	aag01100 ; aag	ETN63249.1 alpha-ar	XP_001649787.1maltase A3
AAEL00438	uncharacterized LOC5564658	-0.686091896471889	0.000205798139100932	0.0173738942752915	NA	NA	NA	ABF18024.1putative 41 kDa salivary secreted protein
AAEL00564	perlucin	-0.684882350832246	0.000396136090244732	0.0249674267289864	NA	NA	XP_001863975.1 gala	XP_019553281.1 perlucin
AAEL02058	NA	-0.683925000085815	0.00870740902177239	0.150611988380356	NA	NA	NA	NA
AAEL02394	NA	-0.683739425868298	0.0550086425260589	0.368425454980292	NA	NA	NA	XP_021707134.1sulfotransferase 1C4-like
AAEL00585	proton-coupled amino acid transporter-like protein CG1139	-0.683512015405047	0.0651106407738045	0.39730198438886	NA	NA	XP_019529894.1 PRE	XP_021709821.1proton-coupled amino acid transporter-like protein CG1139 isoform X2
AAEL02734	NA	-0.681583281913631	0.0783939354899689	0.428723995967346	NA	NA	NA	XP_021703452.1zinc finger protein 808-like isoform X1

AAEL0066(	Krueppel-like factor 3	-0.680054531258485	6,63E+08	0.00156538082998626	NA	NA	KXJ68848.1 hypothe	XP_021703889.1Krueppel-like factor 3
AAEL0127:	serine protease persephone	-0.679126610127911	0.00857094976140011	0.149464458238032	NA	NA	XP_019538453.1 PRE	XP_021698329.1serine protease persephone
AAEL0226:	NA	-0.67863985379573	0.0126898400223591	0.182308324322341	NA	NA	NA	NA
AAEL0004:	larval cuticle protein A2B	-0.678293819985521	0.00343094747056033	0.0901744430841277	NA	NA	KXJ79051.1 hypothe	XP_001656455.2larval cuticle protein A2B isoform X2
AAEL0080:	myeloblastin	-0.67672707511917	0.00097772151057318	0.0432547756744923	NA	NA	XP_001865143.1 mye	XP_019530094.1 myeloblastin-like
AAEL0219:	NA	-0.675445747709974	0.116682774146035	0.514582987743323	NA	NA	NA	NA
AAEL0066(	uncharacterized LOC5568167	-0.673053822197912	0.000232329098978078	0.0188222659264569	NA	NA	ETN64215.1 hypothe	XP_001652104.1uncharacterized protein LOC5568167
AAEL0034:	uncharacterized LOC5578124	-0.672777862680933	0.00266569074543941	0.0792437883464128	NA	NA	KFB50017.1 AGAP01	KFB50017.1AGAP011936-like protein
AAEL0002:	NAD kinase	-0.67148739295159	2,15E+09	0.00373033264991284	760	aag01100 ; aag	KXJ75246.1 hypothe	XP_001654785.2NAD kinase isoform X3
AAEL0050:	serine protease easter	-0.669423059426774	0.000495182226830366	0.0290898537620388	NA	NA	XP_019530148.1 PRE	XP_001650347.2serine protease easter
AAEL0064:	trypsin-1	-0.667417276227679	0.000757317387239843	0.0376562079833735	NA	aag04080	KXJ78237.1 hypothe	XP_019535855.1 trypsin-1-like
AAEL0020:	uncharacterized LOC5573393	-0.667247453910016	0.00935778059915645	0.158773875613038	NA	NA	KXJ78793.1 hypothe	XP_021702056.1uncharacterized protein LOC5573393 isoform X2
AAEL0195:	NA	-0.663422713805573	0.00380611132492403	0.0958410502576181	NA	NA	NA	XP_021695722.1SPARC-related modular calcium-binding protein 2 isoform X2
AAEL0173:	probable cytochrome P450 9f2	-0.663304590006281	0.0321690229750427	0.284442054201394	NA	NA	NA	XP_021705963.1probable cytochrome P450 9f2
AAEL0226:	NA	-0.663216374178669	0.0711859668933623	0.409920692961652	NA	NA	NA	XP_021699104.1arrestin domain-containing protein 17
AAEL0199:	NA	-0.663110715007609	0.0795862644598359	0.431226849261068	NA	NA	NA	XP_021695329.1CD109 antigen
AAEL0238:	NA	-0.662249951096185	0.0538374057795699	0.366428852058877	NA	NA	NA	XP_021696418.1shematrin-like protein 1 isoform X2
AAEL0004:	trichohyalin	-0.660000729994166	0.0488409925715207	0.351987741182022	NA	NA	KXJ79049.1 hypothe	XP_019559367.1 zinc finger CCCH domain-containing protein 13-like
AAEL0201(	NA	-0.658382836879597	0.0388131977744093	0.31084338200184	NA	NA	NA	XP_021695706.1zinc finger protein 62
AAEL0081:	period circadian protein	-0.655699474389041	0.0557693251793926	0.371779217267728	NA	aag04711	XP_019527791.1 PRE	XP_021710527.1period circadian protein isoform X2
AAEL0182:	NA	-0.654990019856725	0.0400125924434144	0.31441514682591	NA	NA	NA	XP_001656120.2uncharacterized protein LOC5576417
AAEL0003:	probable cytochrome P450 313a2	-0.65474802249398	0.00198069827950508	0.0693618362591811	NA	NA	KXJ73598.1 hypothe	XP_001655570.2probable cytochrome P450 313a2
AAEL0072(	rhythmically expressed gene 5 protein	-0.652337051798238	0.000179528023242812	0.0158043227775695	NA	NA	KXJ78543.1 hypothe	XP_001658223.2rhythmically expressed gene 5 protein
AAEL0005:	neither inactivation nor afterpotential protein C	-0.651965317856812	2,75E+09	0.00444705716308738	NA	aag04745	KXJ77723.1 hypothe	XP_021703004.1neither inactivation nor afterpotential protein C
AAEL0084:	cystathionine beta-synthase	-0.651898111282021	0.00388943009608854	0.0964704467498835	00260 ; 00270	aag01100 ; aag	KFB40177.1 AGAP00	XP_001659242.1cystathionine beta-synthase
AAEL0249:	NA	-0.650473107397688	0.091352751429254	0.45924393656719	NA	NA	NA	XP_021693848.1uncharacterized protein LOC5577641
AAEL0232:	NA	-0.649060120934635	0.0218183564839261	0.241418388148241	NA	NA	NA	XP_021703906.1probable cationic amino acid transporter
AAEL0022:	uncharacterized LOC5574092	-0.647840610671833	0.0019064082414595	0.0684803907362971	NA	NA	XP_001688757.1 AG	XP_001661163.2uncharacterized protein LOC5574092
AAEL0143:	protein takeout	-0.6455821163666	0.000123884397856766	0.0131032670008961	NA	NA	NA	XP_001648617.2protein takeout
AAEL0218:	NA	-0.645138255240676	0.0807187865280397	0.433079891352508	NA	aag01100 ; aag	NA	XP_021707064.1beta-1,4-glucuronyltransferase 1
AAEL0038(	uncharacterized LOC5579001	-0.644915354406439	0.000506842260928841	0.0290898537620388	NA	NA	KXJ69749.1 hypothe	XP_001664180.2uncharacterized protein LOC5579001
AAEL0105:	maltase 1	-0.643942355701957	0.00998912821385285	0.161638111035661	NA	aag01100 ; aag	KXJ82055.1 hypothe	XP_001660908.1maltase 1
AAEL0013:	amphoterin-induced protein 2	-0.643668041401957	0.032671714554506	0.28463170766102	NA	aag04013	XP_001866205.1 kek	XP_001653183.2amphoterin-induced protein 2
AAEL0223(	NA	-0.642420930175411	0.0315991076202634	0.28423411888152	NA	aag04120	NA	XP_021701860.1ubiquitin-conjugating enzyme E2Q-like protein 1
AAEL0091:	probable cytochrome P450 6a13	-0.642314438504555	0.000360067997874568	0.0231702497653271	NA	NA	KXJ81549.1 hypothe	XP_001653674.1probable cytochrome P450 6a13
AAEL0022:	trypsin-3	-0.64170931827328	9,48E+09	0.0110128080760421	NA	NA	XP_019531494.1 PRE	XP_019931973.1 trypsin-3-like
AAEL0090:	uncharacterized LOC5571443	-0.641581502443799	0.000237543984467937	0.01884379090581	NA	NA	AAV90685.1 putative	AAL76016.1putative 56.5 kDa secreted protein
AAEL0007:	senecionine N-oxygenase	-0.63956851516616	0.0306875318355598	0.281224109837052	NA	NA	KXJ76794.1 hypothe	XP_001651282.2senecionine N-oxygenase
AAEL0135:	flexible cuticle protein 12	-0.639399604506245	0.0393612650014826	0.313353264954028	NA	NA	NA	XP_001663702.2flexible cuticle protein 12
AAEL0008:	uncharacterized LOC5567196	-0.637247261942861	0.000325259888841996	0.0221706777564744	NA	NA	AAV90646.1 putative	AAY41838.1putative secreted protein precursor
AAEL0221:	NA	-0.635805538033628	0.0631722862279908	0.392761482378267	NA	NA	NA	XP_021709877.1transmembrane protein 42
AAEL0006:	tenascin	-0.63572176182641	0.0116582506311351	0.173701685309403	NA	NA	XP_019557044.1 PRE	XP_021710163.1tenascin isoform X2
AAEL0019(	lactosylceramide 4-alpha-galactosyltransferase	-0.635628378245701	0.0573404529827352	0.375282253447461	NA	aag01100 ; aag	KXJ75779.1 hypothe	XP_001654206.1lactosylceramide 4-alpha-galactosyltransferase
AAEL0066:	uncharacterized LOC5568175	-0.634036878222911	0.000406051056701101	0.0251783355746793	901	NA	KXJ74283.1 hypothe	XP_001652111.2uncharacterized protein LOC5568175
AAEL0043:	uncharacterized LOC5564576	-0.632490586428262	0.0624270974543667	0.390519477073475	NA	NA	XP_001855861.1 con	XP_001648927.1uncharacterized protein LOC5564576
AAEL0136:	L-xylulose reductase	-0.631855359692175	0.00122091130821631	0.0490603749266657	NA	aag01100 ; aag	NA	XP_021701536.1L-xylulose reductase
AAEL0035:	putative sulfiredoxin	-0.631797789235921	0.0885871641466853	0.455153034325962	NA	NA	XP_019552325.1 PRE	XP_001657042.1putative sulfiredoxin isoform X1
AAEL0222:	NA	-0.631447346519558	0.0162371166197047	0.205804334896037	NA	NA	NA	XP_001843649.1Odorant-binding protein 56a
AAEL0034:	uncharacterized LOC5578211	-0.630425437351875	0.000125677378512457	0.0131418549667231	NA	NA	XP_001870800.1 con	XP_001656855.2uncharacterized protein LOC5578211
AAEL0073:	lipopolysaccharide-induced tumor necrosis factor-alpha factor homolog	-0.628127270196841	0.0173293756636615	0.214247748888425	NA	aag04142	ETN64689.1 hypothe	XP_001652693.2lipopolysaccharide-induced tumor necrosis factor-alpha factor homolog
AAEL0143:	uncharacterized LOC5564274	-0.623990184122571	0.00419797289492645	0.100860957125622	901	NA	NA	XP_001648624.2uncharacterized protein LOC5564274
AAEL0106:	uncharacterized LOC5573642	-0.623878912576277	0.0255695180991202	0.260925069284669	NA	NA	XP_001849593.1 con	EAT37351.1AAEL010633
AAEL0231:	NA	-0.622122316117168	0.00115012084194681	0.0475020022701074	NA	NA	NA	XP_021694340.1uncharacterized protein LOC5572392
AAEL0099:	uncharacterized LOC5572399	-0.62155346616597	0.0497071991466785	0.355128607568118	NA	NA	NA	XP_021706232.1uncharacterized protein LOC5572399
AAEL0143(	putative fatty acyl-CoA reductase CG5065	-0.620822647322205	0.0218429895900659	0.241418388148241	73	aag04146	NA	XP_021700067.1putative fatty acyl-CoA reductase CG5065 isoform X1
AAEL0043:	uncharacterized LOC5564644	-0.62069304169518	0.033110032594119	0.285726819198035	NA	NA	KXJ81936.1 hypothe	ABF18165.1putative salivary mucin
AAEL0102:	short-chain dehydrogenase/reductase family 16C member 6	-0.620649602183265	0.000464792422258529	0.02795437823283	NA	aag00830	NA	XP_021701800.1short-chain dehydrogenase/reductase family 16C member 6
AAEL0104:	facilitated trehalose transporter Tret1-2 homolog	-0.619548079453165	0.0978902279444988	0.473599304703091	NA	NA	NA	XP_021693624.1facilitated trehalose transporter Tret1-2 homolog
AAEL0088:	fatty acyl-CoA reductase 1	-0.618583936894871	0.0044500400250279	0.103991720320479	73	NA	XP_001849289.1 con	XP_021706040.1fatty acyl-CoA reductase 1
AAEL0123:	putative odorant-binding protein A10	-0.617728539475923	0.205989643463562	0.638221110825488	NA	NA	AGF68546.1 chemos	XP_001662524.1putative odorant-binding protein A10
AAEL0141:	NA	-0.616293077599241	0.229073532292203	0.662455890682856	NA	NA	NA	XP_001648188.2POU domain, class 6, transcription factor 2
AAEL0203:	NA	-0.614603385017013	0.112740779222083	0.503856556775916	NA	NA	NA	XP_021708171.1zinc finger CCHC domain-containing protein 10
AAEL0239:	NA	-0.613799938625665	0.00333870166991832	0.0895706494652722	NA	NA	NA	XP_021693720.1probable multidrug resistance-associated protein lethal(2)03659
AAEL0268:	NA	-0.609595260258865	0.00248653487236547	0.0770407201868926	NA	NA	NA	XP_021704848.1organic cation transporter protein
AAEL0027:	TBC1 domain family member 16	-0.608940358029286	0.0908992787826078	0.458582874647784	NA	NA	ETN62311.1 hypothe	XP_021705902.1TBC1 domain family member 16
AAEL0015:	uncharacterized LOC5571274	-0.608591691836358	0.00243105412081144	0.0760003211995279	NA	NA	KXJ81776.1 hypothe	XP_021695903.1uncharacterized protein LOC5571274
AAEL0031:	uncharacterized LOC5577135	-0.607860121490708	0.000233181733929155	0.0188222659264569	NA	NA	NA	XP_021708323.1uncharacterized protein LOC5577135 isoform X2
AAEL0208:	NA	-0.607584253144016	0.0538136418352012	0.366428852058877	NA	NA	NA	XP_021712429.1serine protease snake-like
AAEL0086(	zinc carboxypeptidase	-0.607547757964398	0.0147486135350923	0.195839454184587	NA	NA	XP_019549773.1 PRE	XP_001653328.2zinc carboxypeptidase
AAEL0255:	NA	-0.60740393061182	0.488597952765539	0.848846834445739	NA	NA	NA	---NA---
AAEL0105:	acetylcholine receptor subunit beta-like 1	-0.607033816823199	0.00879019139950364	0.151758613992932	NA	NA	ETN60728.1 acetylch	XP_001660921.1acetylcholine receptor subunit beta-like 1
AAEL0095:	histidine decarboxylase	-0.605111525826946	0.0904556345834436	0.458582874647784	NA	aag01100 ; aag	NA	XP_021702102.1histidine decarboxylase isoform X1
AAEL0204:	NA	-0.602824304716602	0.122393824049187	0.524553319917944	NA	NA	NA	XP_001861644.1predicted protein
AAEL0120(	NA	-0.602651521018143	0.0114531736004928	0.172833431546296	NA	NA	KXJ76930.1 hypothe	XP_001662137.2uncharacterized protein LOC5575688
AAEL0026:	4-coumarate--CoA ligase 1	-0.602382138439545	0.000911522455375239	0.0413193578047436	NA	NA	KXJ80796.1 hypothe	XP_001662047.14-coumarate--CoA ligase 1
AAEL0112:	protein D3	-0.601513758017883	0.000175832158361185	0.0157088108858216	NA	NA	KXJ70300.1 hypothe	XP_001661542.1protein D3
AAEL0124(	pantothenate kinase 3	-0.601099131346108	3,15E+09	0.00483391265405248	770	aag01100 ; aag	XP_019542772.1 PRE	XP_021705525.1pantothenate kinase 3 isoform X1
AAEL0195:	NA	-0.600119541575328	0.0202756932939642	0.231198178055834	NA	NA	NA	XP_021708780.1D-beta-hydroxybutyrate dehydrogenase, mitochondrial
AAEL0076:	allantoinase	-0.596272568401703	0.000250184206005234	0.0191849588638347	00230 ; 00240	aag01100 ; aag	KXJ74606.1 hypothe	XP_001658560.1uncharacterized protein LOC5569474
AAEL0032:	endocuticle structural glycoprotein ABD-4	-0.594690586608409	0.00127432851236213	0.0501126964562236	NA	NA	ETN59974.1 cuticula	XP_001656600.1endocuticle structural glycoprotein ABD-4
AAEL0171:	FK506-binding protein 5	-0.593936773739184	0.0147858570880174	0.196051090668496	NA	NA	NA	XP_021700449.1FK506-binding protein 5 isoform X2
AAEL0270:	NA	-0.593590594235676	0.0725384644648959	0.413824519532531	NA	NA	NA	XP_021703798.1MAM domain-containing glycosylphosphatidylinositol anchor protein 1 isoform X2

AAEL00800	clavesin-1	-0.593373305427196	9,69E+08	0.0110128080760421	NA	NA	KXJ75160.1 hypothet	ABF18203.1phosphatidylinositol transfer protein SEC14
AAEL00667	glycine N-acyltransferase-like protein 3	-0.591899263249496	0.0724535003364336	0.413814782657084	NA	NA	XP_019543714.1 PRE	XP_001657925.2glycine N-acyltransferase-like protein 3 isoform X1
AAEL01556	general odorant-binding protein 67	-0.589862919001306	0.00548371686097412	0.118732147187492	NA	NA	NA	XP_021695486.1general odorant-binding protein 67
AAEL00809	trypsin alpha-3	-0.58922872698167	0.00307929612154358	0.08718671664752	NA	NA	KXJ76281.1 hypothet	XP_001653091.2trypsin alpha-3
AAEL01813	NA	-0.588802514297672	0.0221499368641098	0.242070925206103	NA	NA	NA	XP_001652631.2uncharacterized protein LOC5568971
AAEL00452	serine protease persephone	-0.588391187989614	0.0149102834701481	0.197132799557907	NA	NA	KXJ77293.1 hypothet	XP_001649322.2serine protease persephone isoform X1
AAEL02798	NA	-0.587866412898341	0.0689635142409943	0.403354246998601	NA	NA	NA	XP_021711304.1uncharacterized protein LOC110679834
AAEL00952	NA	-0.587737960571419	0.0008638166644876	0.0409667993696665	NA	NA	NA	XP_001660189.2probable maltase
AAEL00405	diphosphomevalonate decarboxylase	-0.587279138176123	0.0374117433462492	0.305927960955648	900	aag01100 ; aag	XP_001844728.1 dip	XP_001648384.2diphosphomevalonate decarboxylase
AAEL01987	NA	-0.58517371370329	0.0102498565073917	0.163088391445208	NA	NA	NA	XP_021704753.1cysteine-rich secretory protein LCCL domain-containing 1
AAEL00565	myosin heavy chain, muscle	-0.58469436231731	0.000258651836166168	0.0195653835802185	NA	NA	XP_019530282.1 PRE	XP_021702575.1myosin heavy chain, muscle
AAEL01181	uncharacterized LOC5570124	-0.584448087309156	0.114303878420794	0.508373266905823	NA	NA	NA	XP_021693114.1uncharacterized protein LOC5570124
AAEL00577	alpha-tocopherol transfer protein-like	-0.584033372628412	0.0479053041426659	0.348612005978262	NA	NA	XP_019565353.1 PRE	XP_001651427.1alpha-tocopherol transfer protein-like isoform X1
AAEL00558	zinc finger protein 521	-0.582596430032	0.074546201809109	0.419169079991884	NA	NA	KXJ81460.1 hypothet	XP_021711345.1zinc finger protein 521
AAEL01279	pyrokinin-1 receptor	-0.582226138087494	0.105744988721551	0.490374022320757	NA	aag04080	ETN65601.1 g-protei	XP_021694461.1pyrokinin-1 receptor
AAEL01339	GPALPP motifs-containing protein 1	-0.581682970098349	0.148100898788358	0.562679383080301	NA	NA	NA	XP_021706430.1GPALPP motifs-containing protein 1
AAEL02079	NA	-0.57935479115833	0.112364074760824	0.503547757511443	NA	NA	NA	XP_021711339.1zinc finger protein Xfin-like isoform X1
AAEL01390	probable 3',5'-cyclic phosphodiesterase pde-5	-0.579345110079072	0.0404894924428262	0.316225178415075	NA	aag01100 ; aag	NA	XP_021700131.1probable 3',5'-cyclic phosphodiesterase pde-5
AAEL01985	NA	-0.579126210560111	0.0100385805811511	0.161638111035661	901	NA	NA	XP_021704849.1uncharacterized protein LOC110677773
AAEL00316	low molecular weight phosphotyrosine protein phosphatase 1	-0.57905586567409	0.0802052270120308	0.433079891352508	00740 ; 00730	NA	XP_019528151.1 PRE	XP_001656483.2low molecular weight phosphotyrosine protein phosphatase 1
AAEL00542	annexin B10	-0.578442629230903	0.000724269320916817	0.0369889266214859	NA	NA	KXJ70192.1 hypothet	XP_001650850.1annexin B10
AAEL01750	cuticle protein 12.5	-0.577828140206454	0.154980470282026	0.572513162398717	NA	NA	NA	XP_011492965.1cuticle protein 12.5
AAEL00511	serine-rich adhesin for platelets	-0.576249083977917	0.00563644048005895	0.120339965887477	NA	NA	KXJ81171.1 hypothet	XP_019525874.1 homeobox protein 2
AAEL00573	chaoptin	-0.575646548118439	0.0107812466350419	0.167582823539959	NA	NA	KXJ83773.1 hypothet	XP_019530720.1 toll-like receptor 7
AAEL01128	zinc finger Ran-binding domain-containing protein 2	-0.575345359377545	0.0211609209684721	0.237177581914593	NA	NA	XP_019544419.1 PRE	XP_001661556.2zinc finger Ran-binding domain-containing protein 2
AAEL02813	NA	-0.574269464069455	0.0542794772404054	0.367469712613638	NA	NA	NA	XP_021708721.1uncharacterized protein LOC110679080
AAEL02727	NA	-0.574043739943697	0.0448975147575279	0.335702315059737	NA	NA	NA	XP_001649035.2proteoglycan 4 isoform X3
AAEL02293	NA	-0.573138840325518	7,02E+09	0.00872653094146289	NA	NA	NA	XP_021694825.1transmembrane protein 41B isoform X1
AAEL01051	transcription factor cwo	-0.57273318182146	0.00112904586945344	0.04722490950323	NA	NA	NA	XP_021693046.1transcription factor cwo isoform X1
AAEL01236	uncharacterized LOC5576168	-0.572009591727173	0.0951000916263656	0.466773927728342	NA	NA	KXJ73506.1 hypothet	XP_021697659.1uncharacterized protein LOC5576168 isoform X2
AAEL02435	NA	-0.571909823442408	0.0290982796985367	0.275227088745645	NA	NA	NA	XP_021713007.1high-affinity choline transporter 1
AAEL00472	homocysteine S-methyltransferase	-0.570249272508118	0.0177801496177233	0.216133337889418	NA	aag01100 ; aag	KFB41458.1 AGAP00	XP_001850787.1homocysteine S-methyltransferase
AAEL02243	NA	-0.570057061185786	0.159987072729566	0.577560236664366	NA	NA	NA	XP_021696334.1kazrin isoform X1
AAEL01964	NA	-0.568369312095693	0.0206212513324651	0.233690584681458	NA	NA	NA	XP_021703462.1cell death abnormality protein 1 isoform X2
AAEL01224	glycine receptor subunit alpha-3	-0.568093254090669	0.0454072804193566	0.338330197910056	NA	NA	KXJ77459.1 hypothet	XP_021708175.1glycine receptor subunit alpha-3
AAEL00109	melanization protease 1	-0.567222487235838	0.00633840544660372	0.129902019865584	NA	NA	KXJ83231.1 hypothet	XP_001658014.1melanization protease 1
AAEL00805	uncharacterized LOC5569955	-0.567048400119824	0.0379091174605523	0.307682856236235	NA	NA	KXJ73764.1 hypothet	XP_001658853.2uncharacterized protein LOC5569955
AAEL02606	NA	-0.567022626715696	0.000194603783872979	0.0165809631407329	00061 ; 00254 ; 00620 ;	aag01100 ; aag	NA	XP_021698257.1acetyl-CoA carboxylase isoform X1
AAEL01345	uncharacterized LOC5577977	-0.566733032762027	0.0139373543549919	0.189161555714802	NA	NA	NA	XP_021698914.1uncharacterized protein LOC5577977
AAEL00389	NA	-0.566568782784668	0.00422765782566331	0.101309654457692	NA	NA	XP_019529367.1 PRE	XP_001664290.2probable cytochrome P450 28a5
AAEL02651	NA	-0.565279751496164	0.01029512717559	0.163337517706516	NA	NA	NA	NA
AAEL00798	uncharacterized LOC5569903	-0.565263989751631	0.00112223061071301	0.04722490950323	NA	NA	NA	ABF18039.1putative 18.6 kDa secreted protein variant 2
AAEL01322	tubulin alpha-1 chain	-0.563884889689015	0.000351468050676226	0.0229513912830783	NA	aag04145	NA	XP_001663407.1tubulin alpha-1 chain
AAEL00269	venom allergen 5.02	-0.563778767043028	0.000390367139960622	0.0247735063580527	NA	NA	AAV90675.1 salivary	XP_001662117.1venom allergen 5.02
AAEL01384	rabenosyn-5	-0.563562971329873	0.11015660823377	0.500079481483548	NA	aag04144	NA	XP_019555496.1 rabenosyn-5-like
AAEL00697	annulin	-0.561941410581441	0.00204746849977721	0.0710935580475365	NA	NA	XP_001845164.1 anr	XP_001652440.1annulin isoform X1
AAEL00722	nidogen-2	-0.561654067552516	0.00112462660124739	0.04722490950323	NA	NA	KXJ83140.1 hypothet	XP_019538107.1 nidogen-1-like
AAEL00536	AP-1 complex subunit gamma-1	-0.560900274889078	0.00616282757536091	0.128303935177536	NA	NA	XP_001843588.1 ada	XP_021709853.1AP-1 complex subunit gamma-1
AAEL00852	endothelin-converting enzyme 2	-0.560693124211282	0.146040160045596	0.560092931004593	NA	NA	XP_019548121.1 PRE	XP_021705832.1endothelin-converting enzyme 2
AAEL00151	WD repeat-containing protein 35	-0.559975909701585	0.150712141096052	0.566025396924239	NA	NA	ETN65290.1 wd-repe	XP_001659551.2WD repeat-containing protein 35
AAEL01502	myrosinase 1	-0.559252028015751	0.00321519295224638	0.088916783130334	NA	NA	NA	XP_021698828.1myrosinase 1
AAEL02118	NA	-0.558510363863075	0.0310306677783865	0.282437393567471	NA	NA	NA	XP_021695405.1microfibril-associated glycoprotein 4
AAEL00593	6-phosphogluconate dehydrogenase, decarboxylating	-0.558275180793702	0.000261720736711336	0.0195653835802185	00030 ; 00480	aag01100 ; aag	KXJ71124.1 hypothet	XP_021706629.16-phosphogluconate dehydrogenase, decarboxylating
AAEL00185	protein msta	-0.556522225333493	0.0282889464817268	0.272581031963194	NA	NA	KXJ71688.1 hypothet	XP_001654144.2protein msta
AAEL00875	uncharacterized LOC5571024	-0.555598922965132	0.0616619715517185	0.387312943494139	NA	NA	KXJ74337.1 hypothet	XP_001659458.2uncharacterized protein LOC5571024
AAEL01258	probable Rho GTPase-activating protein CG5521	-0.555502592185723	0.00933773084691493	0.158773875613038	NA	NA	XP_019554071.1 PRE	XP_021693736.1probable Rho GTPase-activating protein CG5521 isoform X1
AAEL00151	venom carboxylesterase-6	-0.55475852160895	0.0109924097895283	0.168586924805399	NA	NA	KXJ69371.1 hypothet	XP_021709982.1venom carboxylesterase-6 isoform X1
AAEL00690	collagenase	-0.553096388668421	0.0269780551262439	0.267809217385466	NA	NA	XP_001845462.1 seri	XP_019526462.1 collagenase
AAEL02502	NA	-0.552404369388453	0.177826574595555	0.602932991683234	NA	NA	NA	XP_021694328.1 melanization protease 1
AAEL01301	zinc finger protein 14	-0.551211375691152	0.07106960581633	0.409506895880945	NA	NA	NA	XP_001663215.2zinc finger protein 14
AAEL01982	NA	-0.546691132429534	0.00101953967552914	0.044681656837454	480	NA	NA	XP_021704584.1aminopeptidase N
AAEL00347	oocyte zinc finger protein XlCOF28	-0.54639856517318	0.146840417930413	0.561606619200193	NA	NA	KXJ76569.1 hypothet	XP_001656844.2oocyte zinc finger protein XlCOF28
AAEL00467	inactive rhomboid protein 1	-0.546306735925085	0.0397881432518662	0.313706406586116	NA	NA	XP_001868751.1 rho	XP_021706729.1inactive rhomboid protein 1 isoform X2
AAEL00397	zinc finger protein 329	-0.546256950431574	0.139301392843469	0.549207976411999	NA	NA	KXJ73603.1 hypothet	XP_001648105.2zinc finger protein 329
AAEL01038	xanthine dehydrogenase	-0.545263034761531	0.0139808860977388	0.189472921754628	NA	NA	NA	XP_001654510.2xanthine dehydrogenase
AAEL01803	NA	-0.545036800741503	0.0522937470989616	0.362627777546831	NA	NA	NA	XP_001658675.2transcription factor btd isoform X1
AAEL01040	protein dispatched	-0.544305422411542	0.0793574091131253	0.430824117202938	NA	NA	NA	XP_021693452.1protein dispatched
AAEL00990	stress response protein NST1	-0.543541078343184	0.0384351121724601	0.309719436954163	NA	NA	NA	XP_001654094.2stress response protein NST1
AAEL01248	NA	-0.542636484163703	0.00274777281034983	0.0807827648589109	NA	NA	NA	XP_021693525.1E3 ubiquitin-protein ligase RNF19B isoform X3
AAEL01428	SNAPIN protein homolog	-0.54093452441176	0.039637383221744	0.313353264954028	NA	NA	NA	XP_001648342.1SNAPIN protein homolog
AAEL01404	protein fem-1 homolog C	-0.540911279289199	0.102747940371196	0.485611991420516	NA	NA	NA	XP_001657331.2protein fem-1 homolog C
AAEL02048	NA	-0.540398010929151	0.0260099138007057	0.262907960607041	NA	NA	NA	XP_021699415.1CUGBP Elav-like family member 4 isoform X2
AAEL00669	ficolin-3	-0.539603696868823	0.000215256499325067	0.0180071846071751	NA	NA	KXJ78532.1 hypothet	XP_019551179.1 microfibril-associated glycoprotein 4-like
AAEL01727	alcohol dehydrogenase 1	-0.538530812959551	0.0738543277216948	0.417962806700514	NA	NA	NA	XP_011493079.2alcohol dehydrogenase 1
AAEL02822	NA	-0.537327998221624	0.0379255264143692	0.307682856236235	NA	NA	NA	XP_019561498.1 TPRXL
AAEL01969	NA	-0.53511157125878	0.00659455919633235	0.133369524669561	NA	NA	NA	XP_021693302.1myosin-I heavy chain isoform X1
AAEL00012	general odorant-binding protein 68	-0.534448413179887	0.0397587079805078	0.313706406586116	NA	NA	KFB50991.1 AGAP00	XP_001657658.1general odorant-binding protein 68
AAEL00654	uncharacterized LOC5568102	-0.534147580068744	0.181631408146284	0.607917241925556	NA	NA	KXJ74092.1 hypothet	XP_001652041.1uncharacterized protein LOC5568102
AAEL02068	NA	-0.533509566084115	0.179493687385052	0.604797111430702	NA	NA	NA	XP_021698281.1probable E3 ubiquitin protein ligase DRIPH
AAEL02477	NA	-0.533503615235721	0.180381971189195	0.605728027045765	NA	NA	NA	XP_021695048.1uncharacterized protein LOC110674848
AAEL00959	sterol O-acyltransferase 1	-0.532216741008963	0.00684307691843641	0.136594346645232	NA	aag00100	NA	XP_001660281.2sterol O-acyltransferase 1
AAEL02572	NA	-0.531989781045309	0.0326834009852068	0.28463170766102	NA	NA	NA	XP_021698112.1protein phosphatase Slingshot isoform X1

AAEL02464	NA	-0.530925105392652	0.0465783226834622	0.344268052476481	NA	NA	NA	NA
AAEL01013	uncharacterized LOC5572920	-0.53011619475774	0.242823879684659	0.677726824646112	NA	NA	NA	XP_001654253.2uncharacterized protein LOC5572920
AAEL00163	uncharacterized LOC5571683	-0.530055758320929	0.0968779342124512	0.470633908275356	NA	NA	ETN65836.1 hypothe	XP_021701183.1uncharacterized protein LOC5571683
AAEL01271	protein CutA homolog	-0.529862500134391	0.0939288118643083	0.464823706598816	NA	NA	KXJ69709.1 hypothet	XP_021694361.1protein CutA homolog
AAEL01185	uncharacterized LOC5575474	-0.528850035409968	0.0830581053245461	0.43799466200371	NA	NA	KXJ84239.1 hypothet	XP_019552850.1 cylicin-1-like
AAEL01038	xanthine dehydrogenase/oxidase	-0.528674783063842	0.000450485102623052	0.0273106386927193	NA	NA	NA	XP_001654511.2xanthine dehydrogenase/oxidase
AAEL01158	uncharacterized LOC5575041	-0.528356326044733	0.00316243516278544	0.0884520619086676	NA	NA	KFB36569.1 AGAP00	KFB36569.1AGAP007679-like protein
AAEL00144	T-complex protein 11-like protein 1	-0.527670791185995	0.00492953242387556	0.111727973804194	NA	NA	XP_019553406.1 PRE	XP_021700929.1T-complex protein 11-like protein 1 isoform X2
AAEL00313	retinol dehydrogenase 14	-0.527167219885022	0.00692211124848952	0.137411930736898	NA	NA	KFB37597.1 short-ch	XP_001656339.2retinol dehydrogenase 14 isoform X3
AAEL01097	protein FAM207A	-0.526576917418604	0.028868843079591	0.274601260220884	NA	NA	KXJ72764.1 hypothet	XP_001655063.1protein FAM207A isoform X1
AAEL01810	NA	-0.526013861129238	0.000180336219478896	0.0158043227775695	NA	NA	NA	XP_019530313.1 general odorant-binding protein 99b-like
AAEL02718	NA	-0.525950423852668	0.0926867941595237	0.462398967492132	NA	NA	NA	XP_021712484.1uncharacterized protein LOC110681024
AAEL01756	bifunctional endo-1,4-beta-xylanase XylA	-0.525844334396135	0.0193525529784722	0.226893947646744	NA	NA	NA	XP_011493432.1uncharacterized protein LOC23687983
AAEL01135	uncharacterized LOC5574703	-0.523548640877784	0.00220622932345701	0.0737239957572492	NA	NA	NA	XP_021697231.1uncharacterized protein LOC5574703 isoform X2
AAEL00668	uncharacterized LOC5564674	-0.52338371021361	0.00088808645420269	0.0413193578047436	NA	NA	KXJ78668.1 hypothet	XP_021701558.1uncharacterized protein LOC5564674 isoform X1
AAEL02166	NA	-0.521432305603408	0.261396626927076	0.694591903258144	NA	NA	NA	NA
AAEL02101	NA	-0.519778469257012	0.0176365365602698	0.214955509175633	NA	NA	NA	XP_021704067.1homeotic protein spalt-major isoform X2
AAEL02463	NA	-0.519720453294017	0.0409451998133462	0.318493430838894	NA	NA	NA	XP_021697799.1neuronal calcium sensor 2 isoform X3
AAEL01409	uncharacterized LOC5563737	-0.519324696409381	0.0528027017071256	0.362633125106993	NA	NA	NA	XP_021697438.1uncharacterized protein LOC5563737 isoform X1
AAEL00416	GDP-fucose protein O-fucosyltransferase 2	-0.518013811576399	0.0784250482345933	0.428723995967346	NA	aag00514	KXJ69771.1 hypothet	XP_001648601.2GDP-fucose protein O-fucosyltransferase 2
AAEL00520	capon-like protein	-0.517760284365954	0.0507127538457321	0.35814179653755	NA	NA	XP_019538678.1 PRE	XP_021695362.1capon-like protein isoform X3
AAEL00642	37 kDa salivary gland allergen Aed a 2	-0.516972654026158	0.00086812930635568	0.0409667993696665	NA	NA	3DXL_A Chain A, Crys	XP_001657779.137 kDa salivary gland allergen Aed a 2
AAEL00677	fatty acyl-CoA reductase wat	-0.516301582868378	0.0214295616326645	0.238631723223164	73	aag04146	KFB37832.1 AGAP01	XP_001658050.1fatty acyl-CoA reductase wat
AAEL01961	NA	-0.515964473490072	0.000781339133675292	0.0380417074501589	NA	NA	NA	XP_021707456.1GAS2-like protein pickled eggs
AAEL00504	TELO2-interacting protein 1 homolog	-0.515954085722406	0.159764449338551	0.577560236664366	NA	aag04150	KXJ77559.1 hypothet	XP_001650296.2TELO2-interacting protein 1 homolog
AAEL00484	F-BAR and double SH3 domains protein 2	-0.515653493046856	0.145567654582253	0.558950568057442	NA	NA	KXJ82642.1 hypothet	XP_021697738.1F-BAR and double SH3 domains protein 2 isoform X2
AAEL00240	GATA zinc finger domain-containing protein 14	-0.515149474279818	0.0317900165705785	0.284442054201394	NA	NA	KXJ80172.1 hypothet	XP_021701270.1GATA zinc finger domain-containing protein 14
AAEL02009	NA	-0.514362586092207	0.00327700397249374	0.0891942138367879	NA	NA	NA	NA
AAEL00142	venom acid phosphatase Acph-1	-0.513647744282421	0.00354960081170293	0.091238621981258	00740 ; 00730	NA	KFB51638.1 AGAP00	XP_001659203.1venom acid phosphatase Acph-1
AAEL01343	spaetzle 1C	-0.513092597823355	0.00710840921184135	0.137861850831157	NA	NA	NA	XP_021703650.1uncharacterized protein LOC5577955 isoform X1
AAEL02237	NA	-0.512603006592127	0.0678033283416905	0.40094231299198	NA	NA	NA	XP_021697296.1PR domain zinc finger protein 5
AAEL00003	general odorant-binding protein 19d	-0.512430322617427	0.0244138406933879	0.255291093250631	NA	NA	KXJ71014.1 hypothet	XP_021705225.1general odorant-binding protein 19d
AAEL00197	transcription initiation factor TFIID subunit 13	-0.511469912662328	0.0919059045942686	0.46005268034546	NA	aag03022	KXJ69137.1 hypothet	XP_001660799.1transcription initiation factor TFIID subunit 13
AAEL00124	uncharacterized protein C15orf41 homolog	-0.511113132796905	0.0910360962807765	0.459021456425044	NA	NA	KXJ83294.1 hypothet	XP_001652881.1uncharacterized protein C15orf41 homolog
AAEL01170	zinc finger protein Xfin	-0.51086293666332	0.168704312262462	0.58892909007556	NA	NA	XP_019532048.1 PRE	XP_001655647.2zinc finger protein Xfin isoform X2
AAEL00130	uncharacterized LOC5569810	-0.510418513305911	0.000798116578241877	0.038584534817923	NA	NA	NA	XP_001653027.2uncharacterized protein LOC5569810 isoform X2
AAEL02250	NA	-0.508493237042479	0.0875697074180118	0.452464151890787	NA	NA	NA	XP_021707406.1zinc finger protein 25-like
AAEL02903	NA	-0.507546378660314	0.0311460864178484	0.282649198438896	NA	NA	NA	EAT45022.1AAEL003655
AAEL00874	transmembrane protein 53-A	-0.507076717068367	0.00827175892005891	0.147227709056832	NA	NA	KXJ73539.1 hypothet	XP_001653445.1transmembrane protein 53-A
AAEL00750	alpha-tocopherol transfer protein-like	-0.506571164638113	0.0216777454349713	0.240625589255255	NA	NA	KXJ82198.1 hypothet	XP_001652786.2alpha-tocopherol transfer protein-like
AAEL00138	folliculin-interacting protein 2	-0.506321821482926	0.0467472208563327	0.345031868617454	NA	aag04150	KXJ75351.1 hypothet	XP_021699497.1folliculin-interacting protein 2
AAEL00679	uncharacterized LOC5579920	-0.506251444400016	0.0326962306516773	0.28463170766102	NA	NA	KXJ79849.1 hypothet	XP_001652209.2uncharacterized protein LOC5579920
AAEL02518	NA	-0.506098405461433	0.000303735219567651	0.0211740264428904	NA	NA	NA	XP_019549401.2 titin-like
AAEL01189	protein obstructor-E	-0.505939502680304	0.0920264539552414	0.460232298530506	NA	NA	KXJ84418.1 hypothet	XP_001662027.2protein obstructor-E
AAEL00132	protein takeout	-0.504311580355118	0.000508961796966774	0.0290898537620388	NA	NA	XP_019561180.1 PRE	XP_001653020.2protein takeout isoform X1
AAEL02402	NA	-0.503902151389409	0.237897948115689	0.672545904319684	NA	NA	NA	NA
AAEL00292	protein Spindly	-0.503428559704527	0.18985297637012	0.61841666851605	NA	NA	KXJ74844.1 hypothet	XP_021694237.1protein Spindly
AAEL00763	myosin light chain kinase 2, skeletal/cardiac muscle	-0.502925660435174	0.206266293786992	0.638433378885942	NA	NA	ETN65502.1 myosin l	XP_021697561.1myosin light chain kinase 2, skeletal/cardiac muscle
AAEL00083	senecionine N-oxygenase	-0.502513939225767	0.0013980158640913	0.0536975940053796	NA	NA	XP_019537918.1 PRE	XP_001651284.1senecionine N-oxygenase isoform X2
AAEL01380	protein singed	-0.502253567951031	0.00148742157647722	0.0560953006014075	NA	NA	NA	XP_001664000.1protein singed isoform X3
AAEL01435	serine protease easter	-0.502070693868437	0.00742600663810532	0.141410084049817	NA	NA	NA	XP_001648544.2serine protease easter
AAEL02818	NA	-0.501560048295533	0.00198241283809657	0.0693618362591811	NA	NA	NA	XP_021694158.1uncharacterized protein LOC23687573 isoform X2
AAEL01092	serum response factor homolog	-0.50110394062008	0.00768344204836219	0.142834411573796	NA	NA	XP_019564799.1 PRE	XP_021706604.1serum response factor homolog isoform X3
AAEL00865	uncharacterized LOC5570879	-0.500653148904676	0.0157810256406756	0.202259474938964	NA	NA	KXJ76287.1 hypothet	XP_001659394.1uncharacterized protein LOC5570879
AAEL01335	activating molecule in BECN1-regulated autophagy protein 1	-0.500149103395882	0.0501561736578128	0.356468207181388	NA	NA	NA	XP_001656679.2activating molecule in BECN1-regulated autophagy protein 1
AAEL00120	LIM domain transcription factor LMO4	-0.500104372273629	0.0845421372824725	0.441769873522608	NA	NA	CRL01584.1 CLUMA_	XP_021703037.1LIM domain transcription factor LMO4
AAEL02602	NA	-0.499931266164488	0.0985395410278591	0.475490748053676	NA	NA	NA	2NBM_AChain A, Solution Nmr Structure Of Ligand Free Sterol Carrier Protein 2 Like 2 From Aedes Aegypti
AAEL00031	uncharacterized oxidoreductase YjmC	-0.498242162875562	0.0194683331317406	0.22702918113002	NA	NA	KXJ81871.1 hypothet	XP_021697897.1uncharacterized protein LOC5575133
AAEL01319	uncharacterized LOC5577450	-0.49820436434288	0.34178226031111	0.762438298723025	NA	NA	NA	XP_001663376.2uncharacterized protein LOC5577450
AAEL02016	NA	-0.497880394334728	0.0472133081049421	0.346180765881814	NA	NA	NA	NA
AAEL00339	tankyrase	-0.497719293193444	0.0202077826402001	0.230709697090721	NA	NA	KXJ69712.1 hypothet	XP_019542977.1 tankyrase-like
AAEL00381	uncharacterized LOC5579004	-0.497224134655844	0.00501906806642571	0.112922895714546	NA	NA	NA	XP_021695774.1uncharacterized protein LOC5579004 isoform X2
AAEL00128	uncharacterized LOC5569807	-0.497057808154408	0.000422456923309702	0.0259163240553058	NA	NA	KXJ73628.1 hypothet	XP_019561183.1 circadian clock-controlled protein-like
AAEL00799	serine protease 7	-0.496614634300994	0.041901430274894	0.323199464702074	NA	NA	XP_019530696.1 PRE	XP_021693694.1serine protease 7 isoform X1
AAEL00821	solute carrier family 25 member 35	-0.496595590078234	0.123318484181231	0.526206332972576	NA	NA	KXJ80482.1 hypothet	XP_001659037.2solute carrier family 25 member 35
AAEL02513	NA	-0.496513628142396	0.0451476237252962	0.337214637597545	NA	NA	NA	XP_021712121.1 PAB-dependent poly(A)-specific ribonuclease subunit PAN3-like
AAEL02138	NA	-0.496361498465171	0.156114660511962	0.573938116672425	NA	NA	NA	XP_021693893.1polycomb protein Pcl
AAEL02432	NA	-0.496035157078503	0.161676793786002	0.578890994715483	NA	NA	NA	XP_021701683.1uncharacterized protein LOC5577127 isoform X1
AAEL02900	NA	-0.495587057577127	0.0117285077959219	0.174019880159419	NA	NA	NA	XP_001663167.2UDP-glucuronosyltransferase 1-8 isoform X1
AAEL02498	NA	-0.493373435566106	0.00140512302141053	0.0536975940053796	NA	NA	NA	XP_021693920.1uncharacterized protein LOC5568525 isoform X2
AAEL00988	bumetanide-sensitive sodium-(potassium)-chloride cotransporter	-0.493190857747971	0.0369568372633172	0.303911364161792	NA	NA	NA	XP_001654082.2bumetanide-sensitive sodium-(potassium)-chloride cotransporter isoform X1
AAEL00463	actin-87E	-0.492869345116124	0.000619429964575742	0.033333301368573	NA	aag04145 ; aag	AHW40461.1 actin, p	XP_019563901.1 actin-87E
AAEL00712	facilitated trehalose transporter Tret1-2 homolog	-0.4924854664703	0.000702021597472297	0.0360894007817881	NA	NA	XP_019533131.1 PRE	XP_021700714.1facilitated trehalose transporter Tret1-2 homolog isoform X2
AAEL00195	uncharacterized LOC5573185	-0.492481798004783	0.194259354235033	0.625563560641961	NA	NA	KXJ69134.1 hypothet	XP_001660802.1uncharacterized protein LOC5573185
AAEL01451	uncharacterized LOC5564571	-0.492433529931554	0.0735192652949337	0.417790090194633	NA	NA	NA	XP_021694963.1uncharacterized protein LOC5564571
AAEL00289	serine/threonine-protein kinase D1	-0.49216677949836	0.000300753006938093	0.0211740264428904	4070	NA	XP_019553380.1 PRE	XP_001662673.2serine/threonine-protein kinase D1 isoform X3
AAEL00609	gelsolin	-0.49162907140378	0.00105765010081423	0.0454789543350121	NA	NA	NA	XP_001657431.2gelsolin isoform X2
AAEL02198	NA	-0.491604343197631	0.206672878214781	0.639476740192473	NA	NA	NA	KFB46917.1AGAP004827-like protein
AAEL02550	NA	-0.488590402081815	0.0479616169928798	0.348612005978262	NA	NA	NA	XP_021705464.1uncharacterized protein LOC110677897
AAEL00064	maltase A1	-0.488582773890265	0.000662799513831307	0.0345890535361145	NA	aag01100 ; aag	KXJ74656.1 hypothet	XP_001649786.1maltase A1
AAEL01987	NA	-0.488085964532855	0.275091353358125	0.710865103510661	NA	NA	NA	XP_021710653.1activating signal cointegrator 1 complex subunit 2
AAEL00088	cytoplasmic dynein 2 heavy chain 1	-0.487393880686755	0.138006377461766	0.547441354014568	NA	aag04145	KXJ72098.1 hypothet	XP_021709178.1cytoplasmic dynein 2 heavy chain 1

AAEL01271	clotting factor B	-0.487309339949237	0.00214558451172441	0.0727761081152129	NA	NA	KXJ69333.1 hypothet	XP_001662820.2clotting factor B
AAEL00807	NA	-0.486980043600692	0.00301625175244014	0.0867360894561068	NA	NA	KXJ71600.1 hypothet	XP_001653094.2trypsin alpha-3
AAEL00997	uncharacterized LOC5572709	-0.486941016212166	0.155916646788414	0.573884755986765	NA	NA	NA	XP_001660522.2uncharacterized protein LOC5572709
AAEL00768	transcription factor MafB	-0.486438292675652	0.0845314971311897	0.441769873522608	NA	NA	KXJ82036.1 hypothet	XP_001658594.2transcription factor MafB
AAEL02167	NA	-0.485984161113833	0.0328046213172052	0.284781250340492	NA	NA	NA	XP_021703742.1protein sickie isoform X4
AAEL00682	lipid storage droplets surface-binding protein 2	-0.485600769215266	0.000402016380734339	0.0251656784729074	NA	NA	KFB40194.1 AGAP00	XP_001658058.1lipid storage droplets surface-binding protein 2
AAEL01282	leucine-rich melanocyte differentiation-associated protein	-0.485209358126672	0.030211425706389	0.280801691445466	NA	NA	KXJ71395.1 hypothet	XP_021693055.1leucine-rich melanocyte differentiation-associated protein
AAEL00994	uncharacterized LOC5572618	-0.485159225001586	0.074323472996649	0.418818002133309	NA	NA	NA	XP_001660484.1uncharacterized protein LOC5572618
AAEL01868	NA	-0.484495830870102	0.32169644985559	0.746595392577842	NA	aag01100 ; aag	NA	ABF18219.1NADH dehydrogenase subunit 4 (mitochondrion)
AAEL00806	muscle M-line assembly protein unc-89	-0.484432138509482	0.00232113228178168	0.0747232890974067	NA	NA	XP_019534696.1 PRE	XP_001658878.2muscle M-line assembly protein unc-89 isoform X1
AAEL00868	interferon-inducible double-stranded RNA-dependent protein kinase acti	-0.484379125170969	0.000902542676033498	0.0413193578047436	NA	NA	KXJ72239.1 hypothet	XP_021701497.1interferon-inducible double-stranded RNA-dependent protein kinase activator A homolog isoform X1
AAEL00201	putative odorant-binding protein A10	-0.484209262925222	0.0672557552391162	0.4007529665455	NA	NA	AEK32392.1 chemos	XP_021703244.1putative odorant-binding protein A10
AAEL00861	unconventional myosin-VIIa	-0.483999155064225	0.000745696098030595	0.0374568822266592	NA	NA	XP_019549776.1 PRE	XP_021702187.1unconventional myosin-VIIa isoform X2
AAEL00267	elongation of very long chain fatty acids protein AAEL008004	-0.483033122285012	0.0127986291987214	0.182311123663521	62	NA	ETN58473.1 Elongati	XP_021703498.1elongation of very long chain fatty acids protein AAEL008004
AAEL01441	probable cytochrome P450 304a1	-0.482196996829903	0.0321166511598773	0.284442054201394	NA	NA	NA	XP_001648727.1probable cytochrome P450 304a1
AAEL02410	NA	-0.482039865138218	0.264240808394367	0.698659663885272	NA	NA	NA	XP_001653501.2 FAD-linked sulfhydryl oxidase ALR
AAEL00674	uncharacterized transmembrane protein DDB_G0289901	-0.480951794450563	0.116961958669225	0.514582987743323	NA	NA	KXJ83066.1 hypothet	XP_021697099.1uncharacterized transmembrane protein DDB_G0289901
AAEL01094	protein tweety	-0.480633461617959	0.220483885560918	0.652412844728166	NA	NA	KFB48947.1 AGAP00	XP_001655040.1protein tweety isoform X1
AAEL00222	uncharacterized LOC5573957	-0.480426896357102	0.136375712310867	0.545589928345625	NA	NA	KXJ71507.1 hypothet	XP_001654995.2uncharacterized protein LOC5573957 isoform X2
AAEL00518	laminin subunit gamma-1	-0.48025586713526	0.000218433817815131	0.0181083602840976	NA	aag04512	XP_001843323.1 lam	XP_001650503.2laminin subunit gamma-1
AAEL01968	NA	-0.479748986545598	0.0175656318645254	0.214955509175633	NA	NA	NA	XP_021710343.1extracellular sulfatase SULF-1 homolog
AAEL02629	NA	-0.479567602234049	0.480011266299638	0.845454613425911	NA	NA	NA	NA
AAEL00108	synaptic vesicle glycoprotein 2B	-0.479404464442299	0.00441333879817194	0.103600876583618	NA	NA	KXJ75630.1 hypothet	XP_021693930.1synaptic vesicle glycoprotein 2B
AAEL00268	testisin	-0.479260204936594	0.000907205115426895	0.0413193578047436	NA	NA	KXJ68960.1 hypothet	XP_019561387.1 vitamin K-dependent protein C-like
AAEL01440	M-phase inducer phosphatase	-0.479057044067583	0.101675149048627	0.483848092317719	NA	NA	NA	XP_021713169.1M-phase inducer phosphatase
AAEL02621	NA	-0.47862550935655	0.00233865542926028	0.0747232890974067	NA	NA	NA	XP_021701099.1 myosin heavy chain, muscle
AAEL01324	uncharacterized LOC5577531	-0.477541215034693	0.0505560624542741	0.357859143618638	NA	NA	NA	XP_001663415.1uncharacterized protein LOC5577531
AAEL00331	extensin	-0.477467514564312	0.00265741029071378	0.0792437883464128	NA	NA	KXJ73647.1 hypothet	XP_019560238.1 extensin-like
AAEL00214	neprilysin-2	-0.476947315545239	0.00470643087871106	0.108001438767828	NA	NA	KXJ77254.1 hypothet	XP_001848831.1endothelin-converting enzyme 1
AAEL00641	suppressor of hairless protein	-0.476049722867921	0.0588301017907836	0.380164744858701	NA	aag04330	XP_018567908.1 sup	XP_001657802.1suppressor of hairless protein
AAEL01274	dihydroxyacetone phosphate acyltransferase	-0.475875395933221	0.0305371441237063	0.281224109837052	00564 ; 00561	aag04146 ; aag	KXJ67969.1 hypothet	XP_001656249.1dihydroxyacetone phosphate acyltransferase
AAEL01252	NA	-0.475785533041305	0.268147433204876	0.704184172798867	NA	NA	XP_019551082.1 PRE	XP_021701749.1histidine-rich glycoprotein isoform X2
AAEL00312	uncharacterized LOC5577231	-0.475613431992301	0.00801015961531024	0.144528409372715	NA	NA	KXJ75153.1 hypothet	XP_021706463.1amyloid protein-binding protein 2-like
AAEL01041	dual specificity protein phosphatase 10	-0.475415891699448	0.00227008642847295	0.0747074108366112	NA	aag04013 ; aag	NA	XP_001654548.2dual specificity protein phosphatase 7 isoform X1
AAEL01089	transmembrane protein 235	-0.474614375303882	0.0135621179945657	0.187825791015408	NA	NA	XP_312594.1 AGAP0	XP_001655028.1transmembrane protein 235
AAEL00134	zinc finger protein 628	-0.474549702018942	0.0523359349875898	0.362633125106993	NA	NA	KXJ71231.1 hypothet	XP_021708071.1zinc finger protein 628
AAEL02339	NA	-0.474372012779359	0.0026464022979809	0.0792437883464128	NA	NA	NA	XP_021706161.1uncharacterized protein LOC110678038 isoform X1
AAEL00806	sushi, von Willebrand factor type A, EGF and pentraxin domain-containin	-0.474167786802596	0.0196800535511039	0.227335564593981	NA	NA	XP_019532068.1 PRE	XP_021700031.1sushi, von Willebrand factor type A, EGF and pentraxin domain-containing protein 1 isoform X1
AAEL01946	NA	-0.474125475927814	0.0617665729315173	0.387441038933757	NA	aag04144	NA	XP_021695333.1neural Wiskott-Aldrich syndrome protein
AAEL00700	Kv channel-interacting protein 4	-0.474100202919648	0.166119407039011	0.584439853413553	NA	NA	ETN67180.1 calsenili	XP_001652499.1Kv channel-interacting protein 4 isoform X2
AAEL00482	uncharacterized LOC5565491	-0.473750335723385	0.00297476978191169	0.0858113841164621	NA	NA	KXJ69240.1 hypothet	XP_021694725.1uncharacterized protein LOC5565491
AAEL00764	uncharacterized LOC5569452	-0.473213670091276	0.0814865044122297	0.434941307193351	NA	NA	ETN65987.1 hypothe	XP_019561885.1 FERM domain-containing protein 8-like
AAEL00228	adult enhancer factor 1	-0.47315428506938	0.0780391440897283	0.42745012137719	NA	NA	XP_019531487.2 PRE	XP_001661223.1zinc finger protein 569 isoform X1
AAEL02261	NA	-0.472805906708231	0.114292438342943	0.508373266905823	790	aag00790	NA	XP_021694857.1molybdenum cofactor sulfurase 3 isoform X1
AAEL02644	NA	-0.472506971708472	0.0750984581923871	0.420011717015769	00966 ; 00770 ; 00290 ;	aag01100 ; aag	NA	XP_021693528.1branched-chain-amino-acid aminotransferase, cytosolic
AAEL00582	UPF0676 protein C1494.01	-0.471884653769879	0.00750802539324779	0.142155587215123	NA	NA	XP_001845189.1 con	XP_021712815.1UPF0676 protein C1494.01
AAEL01425	gastrula zinc finger protein XlCGF57.1	-0.471042547008791	0.270161780689312	0.706082729045778	NA	NA	NA	XP_021707646.1gastrula zinc finger protein XlCGF57.1
AAEL00907	NADH dehydrogenase [ubiquinone] 1 beta subcomplex subunit 5, mitoch	-0.470239364432169	0.00362131054673944	0.0923083092828153	NA	aag01100 ; aag	KXJ68957.1 hypothet	XP_001659703.1NADH dehydrogenase [ubiquinone] 1 beta subcomplex subunit 5, mitochondrial
AAEL02256	NA	-0.46966540114492	0.282795514639529	0.715753918648043	NA	NA	NA	XP_021708912.1serine protease snake
AAEL01181	uncharacterized LOC5575405	-0.469517045429589	0.000893432829755291	0.0413193578047436	NA	NA	KXJ78949.1 hypothet	XP_021704590.1uncharacterized protein LOC5575405
AAEL00336	uncharacterized protein C1orf131 homolog	-0.468736137791707	0.15968761005315	0.577560236664366	NA	NA	KXJ71152.1 hypothet	XP_021708057.1uncharacterized protein C1orf131 homolog
AAEL00054	C-type lectin 37Da	-0.467597594857064	0.00796773925607196	0.144528409372715	NA	NA	XP_019931003.1 PRE	XP_001647955.1C-type lectin 37Da
AAEL00600	major facilitator superfamily domain-containing protein 1	-0.466970968080261	0.00291116327912183	0.0845181302823045	NA	NA	XP_019540843.1 PRE	XP_001651759.1major facilitator superfamily domain-containing protein 1 isoform X1
AAEL02207	NA	-0.466924492893531	0.0524805966962206	0.362633125106993	NA	NA	NA	XP_021711503.1putative epidermal cell surface receptor isoform X2
AAEL01438	collectin-11	-0.466021780588725	0.0183030532505909	0.219588912662239	NA	NA	NA	XP_019557186.1 C-type lectin 37Db-like
AAEL00411	uncharacterized LOC5564131	-0.465621286618106	0.000330545367057473	0.0223223256699418	NA	NA	KXJ83382.1 hypothet	XP_021711054.1uncharacterized protein LOC5564131
AAEL01529	chymotrypsin-2	-0.465519302867079	0.0966011668483582	0.470579109231653	NA	NA	NA	ABF18054.1salivary chymotrypsin-like enzyme
AAEL00272	protein extra-macrochaetae	-0.464361735394031	0.00338089167381138	0.0897536781871071	NA	aag04350	XP_001848438.1 con	XP_001662181.2protein extra-macrochaetae
AAEL01016	uncharacterized LOC5572961	-0.463811018937511	0.00609812936781141	0.127824570484284	NA	NA	NA	XP_001654290.2uncharacterized protein LOC5572961
AAEL00712	facilitated trehalose transporter Tret1	-0.463651859071282	0.00327382851766391	0.0891942138367879	NA	NA	XP_001850643.1 sug	XP_001658147.1facilitated trehalose transporter Tret1
AAEL02017	NA	-0.463324200669549	0.187376547218755	0.614263978449227	NA	NA	NA	XP_021701842.1 coiled-coil domain-containing protein 93
AAEL02605	NA	-0.463194752463722	0.0109781115959027	0.168586924805399	NA	NA	NA	XP_021703692.1protein takeout
AAEL00402	uncharacterized LOC5563971	-0.462418498697795	0.00140633776953885	0.0536975940053796	901	NA	KXJ73581.1 hypothet	XP_001848858.1Juvenile hormone-inducible protein
AAEL00964	sprT-like domain-containing protein Spartan	-0.461916293666885	0.133242559250232	0.541323633651493	NA	NA	NA	XP_001653897.2sprT-like domain-containing protein Spartan
AAEL00951	glutamine--fructose-6-phosphate aminotransferase [isomerizing] 2	-0.461490774057315	0.00129131103247636	0.0505644430674361	00520 ; 00250	aag01100 ; aag	NA	XP_001660161.1glutamine--fructose-6-phosphate aminotransferase [isomerizing] 2
AAEL00577	general odorant-binding protein 99a	-0.461195452431003	0.0900819936169444	0.458542183179048	NA	NA	KXJ82322.1 hypothet	XP_001651444.2general odorant-binding protein 99a
AAEL01981	NA	-0.460499188257633	0.00235844997318377	0.0750950057205433	NA	NA	NA	XP_019930934.1 proline-rich receptor-like protein kinase PERK9 isoform X6
AAEL01489	NA	-0.460485343553046	0.0010196857135259	0.044681656837454	NA	NA	NA	XP_019536617.1 toll-like receptor 13
AAEL01429	xenotropic and polytropic retrovirus receptor 1	-0.460186167219256	0.194235760196613	0.625563560641961	NA	NA	NA	XP_001648404.2xenotropic and polytropic retrovirus receptor 1
AAEL01354	transcription factor grauzone	-0.46014386402992	0.0557951639911684	0.371779217267728	NA	NA	NA	XP_001663748.1transcription factor grauzone
AAEL01362	zinc finger protein 420	-0.459262625579691	0.170930060691575	0.592205729850856	NA	NA	NA	XP_021701686.1zinc finger protein 420
AAEL00635	sulfotransferase 1 family member D1	-0.458813275202471	0.0323570931290113	0.28463170766102	NA	NA	XP_019526494.1 PRE	XP_001651918.1sulfotransferase 1 family member D1 isoform X1
AAEL01986	NA	-0.458429730060137	0.072077925568324	0.412475790472461	NA	NA	NA	XP_021697247.1uncharacterized protein LOC5577573 isoform X1
AAEL00631	uncharacterized LOC5567864	-0.458092013680442	0.135042721111128	0.543359475148491	NA	NA	KFB44369.1 AGAP00	KFB44369.1AGAP005168-like protein
AAEL00214	lysM and putative peptidoglycan-binding domain-containing protein 2	-0.457774317040433	0.0273039744769728	0.268836665026084	NA	NA	KXJ72027.1 hypothet	XP_001654849.2lysM and putative peptidoglycan-binding domain-containing protein 2
AAEL00535	sugar transporter SWEET1	-0.457719024458678	0.0154963818492499	0.201125113930603	NA	NA	KFB53394.1 AGAP00	XP_001650785.1sugar transporter SWEET1
AAEL02129	NA	-0.45646364650163	0.236253043490965	0.670870063357945	NA	NA	NA	NA
AAEL00252	lopap	-0.455930527209404	0.030502299390774	0.281224109837052	906	NA	XP_019528445.1 PRE	XP_001655473.2lopap isoform X2
AAEL00495	pupal cuticle protein 36a	-0.455642099510849	0.0582333721576211	0.37790091015122	NA	NA	KXJ81116.1 hypothet	XP_001650103.1pupal cuticle protein 36a
AAEL00883	uncharacterized LOC5571138	-0.454682943312861	0.0658234623209983	0.39849177649857	NA	NA	KXJ72064.1 hypothet	XP_021700259.1uncharacterized protein LOC5571138
AAEL00411	UNC93-like protein	-0.454623962053026	0.0604048925414053	0.384941803020695	NA	NA	KXJ84175.1 hypothet	XP_001648444.1UNC93-like protein
AAEL00908	uncharacterized LOC5571446	-0.454562607974618	0.122123161333757	0.524358469989517	NA	NA	KFB48233.1 AGAP00	KFB48233.1AGAP005820-like protein

AAEL00507	endoplasmic reticulum aminopeptidase 2	-0.453884550978744	0.0423199349806262	0.323939778330243	NA	NA	XP_019932513.1 PRE	XP_021693909.1endoplasmic reticulum aminopeptidase 2 isoform X1
AAEL00004	dnaJ homolog subfamily C member 17	-0.453748448201485	0.0926215942667955	0.462398967492132	NA	NA	KFB36168.1 AGAP00	XP_001647945.1dnaJ homolog subfamily C member 17
AAEL02681	NA	-0.453645271258329	0.187722235141732	0.614740216289758	NA	NA	NA	XP_021694924.1uncharacterized protein LOC110674815
AAEL01376	protein obstructor-E	-0.453264163411616	0.182023804388103	0.608200090043328	NA	NA	NA	XP_001663971.1protein obstructor-E isoform X1
AAEL01078	calexcitin-1	-0.452623429450427	0.0337991537056322	0.289320755720212	NA	NA	XP_001847423.1 con	XP_019553998.1 calexcitin-1-like
AAEL02279	NA	-0.452330269464063	0.0304749095210355	0.281224109837052	NA	NA	NA	XP_021699787.1solute carrier family 2, facilitated glucose transporter member 3
AAEL00110	phosphoserine phosphatase	-0.452155811901577	0.00971455468406031	0.160203104305955	00680 ; 00260	aag01100 ; aag	XP_019536185.1 PRE	XP_001652419.1phosphoserine phosphatase isoform X1
AAEL00247	transcription factor Ken 2	-0.451702248481146	0.0752071170668593	0.420011717015769	NA	NA	KFB43460.1 AGAP00	XP_021709590.1transcription factor Ken 2
AAEL00847	uncharacterized LOC5579397	-0.451224837362415	0.0015027487715205	0.0562125780306163	NA	NA	KXJ69317.1 hypothe	XP_001659275.1uncharacterized protein LOC5579397 isoform X1
AAEL00497	galactosylgalactosylxylosylprotein 3-beta-glucuronosyltransferase S	-0.450025300847712	0.187994132740341	0.614974052426811	NA	NA	KXJ79350.1 hypothe	XP_001650141.1galactosylgalactosylxylosylprotein 3-beta-glucuronosyltransferase S isoform X1
AAEL00915	glutathione synthetase	-0.449847931789198	0.000861580652903326	0.0409667993696665	00270 ; 00480	aag01100 ; aag	KXJ68522.1 hypothe	XP_001653705.2glutathione synthetase isoform X1
AAEL00610	uncharacterized LOC5567431	-0.449486114612118	0.00279776259531615	0.0819904821722905	NA	NA	NA	XP_021707988.1uncharacterized protein LOC5567431 isoform X1
AAEL01820	NA	-0.448613738277132	0.00436811076275269	0.103594442242845	NA	NA	NA	XP_021695711.1carboxypeptidase D isoform X6
AAEL01348	retinol dehydrogenase 11	-0.447955675025309	0.0512224188046148	0.359690131564899	NA	NA	NA	XP_001663677.1retinol dehydrogenase 12 isoform X1
AAEL02212	NA	-0.447073904817838	0.0434292458837651	0.329189390957501	NA	NA	NA	XP_021708781.1dehydrogenase/reductase SDR family member on chromosome X
AAEL01157	serine/threonine-protein kinase OSR1	-0.446249109490225	0.215498080483087	0.64825542223124	04151 ; 05165 ; 04714 ;	NA	ETN62842.1 serine/tl	XP_001661758.1serine/threonine-protein kinase OSR1
AAEL02280	NA	-0.44565948047523	0.00515860964853556	0.114938319578267	NA	NA	NA	NA
AAEL02822	NA	-0.445279287666837	0.250224619986661	0.683335640523162	NA	NA	NA	NA
AAEL01802	NA	-0.44523705911239	0.240855475230562	0.675511149976115	NA	NA	NA	XP_021707737.1synaptotagmin-6 isoform X2
AAEL01460	probable cytochrome P450 9f2	-0.444291554481476	0.00103064761186967	0.0447359402095507	NA	NA	NA	XP_001649097.2probable cytochrome P450 9f2
AAEL02750	NA	-0.444256057252624	0.224866462517368	0.657521826528382	NA	NA	NA	XP_011493059.2mitochondrial inner membrane protein COX18
AAEL00300	WD repeat-containing protein 5	-0.444100475963465	0.0397945851885966	0.313706406586116	NA	NA	ETN59784.1 wd-repe	XP_001662893.1WD repeat-containing protein 5
AAEL00911	mpv17-like protein	-0.44364849413904	0.00304580841635924	0.0867725357502717	NA	aag04146	ETN60157.1 hypothe	XP_021692972.1mpv17-like protein
AAEL01730	mucin-5AC	-0.443148869012462	0.024781706123194	0.258221227488557	NA	NA	NA	XP_019547824.1 mucin-5AC-like
AAEL00822	NADPH-dependent 1-acyldihydroxyacetone phosphate reductase	-0.443082451060913	0.01146897378031	0.172833431546296	NA	NA	KXJ70948.1 hypothe	XP_001659060.1NADPH-dependent 1-acyldihydroxyacetone phosphate reductase
AAEL00496	PDZ domain-containing protein 2	-0.442637523908433	0.0214365977365421	0.238631723223164	NA	NA	NA	XP_021710873.1uncharacterized protein LOC5565736 isoform X1
AAEL02277	NA	-0.442461698229466	0.180591711485961	0.605944990070163	NA	NA	NA	XP_021698597.1serine protease 42
AAEL01314	ankyrin-3	-0.442437661124944	0.000645214279900686	0.0341621442326704	NA	NA	NA	XP_019541717.1 ankyrin-3-like
AAEL01095	uncharacterized LOC5574131	-0.442027895789552	0.216867852829857	0.649748124914163	NA	NA	NA	CAY77162.1adipokinetic hormone 2 preprohormone
AAEL00728	muscle M-line assembly protein unc-89	-0.441417396229223	0.00949133356121765	0.158773875613038	NA	NA	KFB35415.1 AGAP00	XP_021706828.1muscle M-line assembly protein unc-89
AAEL00904	peroxisomal N(1)-acetyl-spermine/spermidine oxidase	-0.441351917203625	0.178602552624952	0.604117469532757	NA	NA	KXJ78901.1 hypothe	XP_001653661.2peroxisomal N(1)-acetyl-spermine/spermidine oxidase
AAEL02143	NA	-0.440643342366087	0.21939917885578	0.652412844728166	NA	NA	NA	XP_021700199.1lachesin isoform X1
AAEL00710	transmembrane protease serine 9	-0.439773188245176	0.0259493304546546	0.262690581786284	NA	NA	KXJ72772.1 hypothe	XP_021698683.1transmembrane protease serine 9
AAEL00252	OTU domain-containing protein 6B	-0.439755810191847	0.0279680534019454	0.271500221524543	NA	NA	KXJ70527.1 hypothe	XP_001655459.2OTU domain-containing protein 6B
AAEL00828	alpha-tocopherol transfer protein	-0.439639816156722	0.317206417858159	0.745684464362801	NA	NA	KXJ68104.1 hypothe	XP_001659078.2alpha-tocopherol transfer protein
AAEL00129	protein takeout	-0.439294344279791	0.0029115678438916	0.0845181302823045	NA	NA	ETN63778.1 hypothe	XP_001653029.1protein takeout
AAEL00660	uncharacterized LOC5568164	-0.439251667548024	0.0755850813756019	0.420516274980827		901	NA	XP_001866179.1 Juv	XP_001866179.1Juvenile hormone-inducible protein
AAEL00615	nicalin-1	-0.439062200110079	0.238214348005963	0.67280858706937	NA	NA	KXJ80456.1 hypothe	XP_019542927.1 nicalin-1
AAEL01965	NA	-0.438966598491211	0.00315343379806122	0.0884520619086676	NA	NA	NA	XP_021700494.1LOW QUALITY PROTEIN: uncharacterized protein LOC5576948
AAEL00338	axin	-0.438947520460441	0.0199720720481942	0.228870494380427	NA	aag04310	XP_019530177.1 PRE	XP_021709960.1axin isoform X1
AAEL02627	NA	-0.438898307325176	0.0709370590055944	0.409506895880945	NA	NA	NA	XP_021699531.1corticotropin-releasing factor-binding protein
AAEL00811	ubiquitin-associated domain-containing protein 1	-0.438801723410879	0.214736464848899	0.648028428468258	NA	NA	KXJ83843.1 hypothe	XP_001653124.2ubiquitin-associated domain-containing protein 1 isoform X1
AAEL00596	condensin complex subunit 2	-0.438556695410624	0.0615201660175695	0.38668618011863	NA	NA	KXJ75812.1 hypothe	XP_001651734.2condensin complex subunit 2
AAEL00097	LIRP	-0.438218613908699	0.040164245294943	0.314545859748141	NA	aag04150 ; aag	KXJ69019.1 hypothe	ABI64118.2insulin-like peptide 2 precursor
AAEL00738	serine-rich adhesin for platelets	-0.438110299971544	0.29025948250814	0.721952579380462	NA	NA	NA	XP_019530114.1 uncharacterized serine-rich protein C215.13-like
AAEL00239	Krueppel homolog 1	-0.43781246476555	0.0075387879331645	0.142155587215123	NA	NA	KXJ84537.1 hypothe	XP_001655162.2Krueppel homolog 1 isoform X1
AAEL01269	uncharacterized LOC5576660	-0.437445296878387	0.0830272024662364	0.43799466200371		901	NA	NA	EAT35127.1AAEL012694
AAEL00576	uncharacterized LOC5567040	-0.436890920378507	0.00102505078942255	0.0447038737642953	NA	NA	KXJ80703.1 hypothe	XP_001651439.2uncharacterized protein LOC5567040
AAEL01177	protein abrupt	-0.436793840990488	0.0497864817625685	0.355143569906322	NA	NA	KXJ82558.1 hypothe	XP_021706049.1protein abrupt
AAEL01988	NA	-0.436252193197221	0.000901716621907379	0.0413193578047436	NA	NA	NA	XP_021696607.1double-stranded RNA-binding protein Staufen homolog 2
AAEL02674	NA	-0.436191345845644	0.0203018660957447	0.231210113630003	NA	NA	NA	XP_021696257.1retinaldehyde-binding protein 1 isoform X2
AAEL02557	NA	-0.436082770764445	0.0071106478771275	0.137861850831157	NA	NA	NA	XP_021707711.1uncharacterized protein C11D3.03c
AAEL01500	zinc finger protein 250	-0.435492339490205	0.18484955414201	0.612086485554846	NA	NA	NA	XP_021701753.1zinc finger protein 250
AAEL00167	actin, muscle	-0.435321586329105	0.00134849936841192	0.052138198269439	NA	aag04145 ; aag	ABW03225.1 beta ac	NP_001296055.1actin, muscle
AAEL00497	proclotting enzyme	-0.435056649249105	0.195287944497963	0.62789645886452	NA	NA	KXJ76539.1 hypothe	XP_001650120.1proclotting enzyme
AAEL00361	acyl-CoA Delta(11) desaturase	-0.434197888636562	0.00430789217776926	0.102697471035836	NA	aag01100 ; aag	ETN58289.1 stearoyl	XP_001657081.2acyl-CoA Delta(11) desaturase
AAEL00223	adult cuticle protein 1	-0.434066876794702	0.196058160595351	0.629273524171057	NA	NA	ETN66068.1 cuticle p	XP_001654960.1adult cuticle protein 1
AAEL02023	NA	-0.433520921514732	0.219615395344128	0.652412844728166	NA	NA	NA	XP_021698385.1protein N-terminal glutamine amidohydrolase
AAEL01330	uncharacterized LOC5577589	-0.432166507754735	0.357762057666812	0.773736338358957	NA	NA	NA	XP_001656582.2uncharacterized protein LOC5577589
AAEL02370	NA	-0.432125376057956	0.0155270905660979	0.201233331073453	NA	NA	NA	NA
AAEL02187	NA	-0.431214776923002	0.0157816021523773	0.202259474938964	NA	NA	NA	XP_019534145.1 phosphoinositide 3-kinase adapter protein 1-like isoform X2
AAEL01128	muscle calcium channel subunit alpha-1	-0.431074655394179	0.00146018511379511	0.0552947465726032	NA	NA	NA	XP_021699866.1muscle calcium channel subunit alpha-1 isoform X1
AAEL02791	NA	-0.430987100934284	0.113902733480587	0.507324759674907	NA	NA	NA	NA
AAEL00739	UDP-glucose 4-epimerase	-0.430738521885822	0.00107504651483613	0.0460119908349863	00052 ; 00520	aag01100 ; aag	KFB49841.1 AGAP01	XP_001652750.1UDP-glucose 4-epimerase
AAEL00480	NA	-0.43070457952787	0.00815021575262285	0.145627738554632	NA	NA	KXJ69661.1 hypothe	ABF18045.1putative secreted salivary protein
AAEL00540	WD repeat-containing protein 91	-0.430559000713416	0.187136069647593	0.614196367514984	NA	NA	XP_019528862.1 PRE	XP_001650856.2WD repeat-containing protein 91
AAEL00342	regulator of microtubule dynamics protein 1	-0.430404303358985	0.000748974823919288	0.0374568822266592	NA	NA	ETN67690.1 hypothe	XP_001656800.1regulator of microtubule dynamics protein 1
AAEL02797	NA	-0.43018108127071	0.245602539794739	0.679727114240672	NA	NA	NA	NA
AAEL01276	cytochrome P450 4C1	-0.430090463646246	0.184644732233015	0.612043911074917	NA	NA	XP_001867815.1 cyt	XP_021706946.1cytochrome P450 4C1
AAEL01737	probable G-protein coupled receptor Mth-like 1	-0.429863080211663	0.0483609985293778	0.35027523096122	NA	NA	NA	XP_011493653.2probable G-protein coupled receptor Mth-like 1 isoform X1
AAEL01799	NA	-0.429433789121649	0.136954332327179	0.545800678248029	NA	NA	NA	XP_021713378.1uncharacterized protein LOC110681571
AAEL00806	cartilage oligomeric matrix protein	-0.42903605821774	0.0198680404624362	0.228246826885565	NA	aag04145 ; aag	ETN67430.1 thromb	XP_021700042.1cartilage oligomeric matrix protein
AAEL02359	NA	-0.429035513786913	0.00126852346898576	0.0500985105648368	NA	NA	NA	XP_021710374.1myb-like protein V
AAEL00374	glucosylceramidase	-0.429033383449919	0.0319935858921165	0.284442054201394	NA	aag01100 ; aag	KXJ81602.1 hypothe	XP_019532306.1 glucosylceramidase-like
AAEL00234	chymotrypsin-1	-0.428626076755883	0.0570277509361346	0.375057077651547	NA	NA	KXJ76377.1 hypothe	XP_019526019.1 chymotrypsin-2-like
AAEL02300	NA	-0.428600942791563	0.274580073935672	0.710341816237294	NA	NA	NA	XP_021698234.1protein G12
AAEL00853	NA	-0.428215397146785	0.14842454305731	0.562679383080301	NA	NA	NA	XP_001659358.2transcription factor grauzone
AAEL00102	regucalcin	-0.427949014081902	0.00250373900779991	0.0773134441267611	NA	NA	KXJ72638.1 hypothe	XP_001657883.2regucalcin isoform X1
AAEL00218	adult cuticle protein 1	-0.427848439347406	0.00440180688078159	0.103594442242845	NA	NA	KXJ78190.1 hypothe	XP_001654939.2adult cuticle protein 1
AAEL01270	RNA-binding protein MEX3B	-0.427690795393943	0.245703786568234	0.679727114240672	NA	NA	KXJ83513.1 hypothe	XP_001662827.2RNA-binding protein MEX3B
AAEL02616	NA	-0.427539797130663	0.00981374832293448	0.160973461796155	NA	NA	NA	NA
AAEL02082	NA	-0.42648381424696	0.0344117613355087	0.293579823070091	NA	NA	NA	XP_021696705.1tyrosine-protein kinase PR2

AAEL02777	NA	-0.426292818370316	0.040908727737154	0.3184789447016	NA	NA	NA	NA
AAEL02793	NA	-0.42612659950808	0.00118129930480258	0.0480987442601474	NA	NA	NA	XP_021693362.1sarcalumenin isoform X1
AAEL00624	uncharacterized LOC5567679	-0.425622015249818	0.00411228041049078	0.0998448663254252	NA	NA	XP_019545771.1 PRE	XP_019545771.1 UPF0746 protein DDB_G0281095 isoform X1
AAEL00389	SNF-related serine/threonine-protein kinase	-0.424617095425659	0.00314371054208672	0.0884520619086676	NA	NA	XP_001845267.1 seri	XP_001664275.2SNF-related serine/threonine-protein kinase
AAEL00685	protein lin-37 homolog	-0.424056984347939	0.0994376609482691	0.478312852561358	NA	NA	KFB43323.1 AGAP00	XP_001652295.2protein lin-37 homolog
AAEL02102	NA	-0.423993199487748	0.346826379127269	0.765398366276275	NA	NA	NA	KFB52530.1AGAP009206-like protein
AAEL00313	Ig-like and fibronectin type-III domain-containing protein 1	-0.423842990915609	0.0020344951644834	0.0709144867559708	NA	NA	ETN61421.1 hypothe	XP_021707105.1Ig-like and fibronectin type-III domain-containing protein 1
AAEL00600	uncharacterized LOC5567333	-0.423700055549094	0.0158532389206684	0.202894999371337	NA	NA	KXJ77289.1 hypothet	XP_001651757.2uncharacterized protein LOC5567333
AAEL02284	NA	-0.423179302871217	0.0492888179048461	0.352961636078127	NA	NA	NA	XP_021711532.1putative polypeptide N-acetylgalactosaminyltransferase 9
AAEL01823	NA	-0.423049854741155	0.313734625459469	0.74451110779113	NA	NA	NA	XP_021698304.1potassium voltage-gated channel subfamily KQT member 1 isoform X3
AAEL00999	uncharacterized LOC5572722	-0.422718250233909	0.0554744852393016	0.37071620419176	NA	NA	NA	XP_001660538.2uncharacterized protein LOC5572722
AAEL01318	open rectifier potassium channel protein 1	-0.422484306297649	0.0127564802516259	0.182308324322341	NA	NA	NA	XP_021710157.1open rectifier potassium channel protein 1 isoform X1
AAEL00986	low-density lipoprotein receptor-related protein 2	-0.422297723535919	0.00414773061072013	0.100176947716133	NA	NA	NA	XP_001660469.2low-density lipoprotein receptor-related protein 2 isoform X3
AAEL00120	probable protein S-acyltransferase 23	-0.422230447684933	0.157240451748005	0.574474189691589	NA	NA	XP_019535906.1 PRE	XP_021695344.1probable protein S-acyltransferase 23
AAEL00888	probable cytochrome P450 6d5	-0.42151149196787	0.0667215744428961	0.399540230684495	NA	NA	KXJ83992.1 hypothet	XP_001653509.1probable cytochrome P450 6d5
AAEL01945	NA	-0.420686202353339	0.136796171586155	0.545589928345625	534	aag01100 ; aag	NA	XP_019560244.1 exostosin-1
AAEL00266	72 kDa type IV collagenase	-0.420661427238336	0.0135417792733056	0.187825791015408	NA	NA	KXJ73279.1 hypothet	XP_001662054.172 kDa type IV collagenase
AAEL00363	serine protease easter	-0.420492451109432	0.0227695034163136	0.245124275357034	NA	NA	KXJ76329.1 hypothet	XP_001657087.2serine protease easter
AAEL00602	lipase member H-A	-0.419202949215271	0.0187304670856932	0.223621149257916	NA	NA	KXJ75250.1 hypothet	XP_021706895.1phospholipase A1 4 isoform X2
AAEL02604	NA	-0.419175362027799	0.00529141323304741	0.116209032387834	NA	NA	NA	2KSH_AChain A, Solution Nmr Structure Of Apo Sterol Carrier Protein - 2 From Aedes Aegypti (Aescp-2)
AAEL00445	organic cation transporter protein	-0.418635227724813	0.0476756892296402	0.348460438674463	NA	NA	XP_019538575.1 PRE	XP_021705408.1organic cation transporter protein isoform X1
AAEL00513	protein kinase C-binding protein NELL2	-0.418409144834595	0.0421960133482198	0.323939778330243	NA	NA	XP_019527617.1 PRE	XP_021709168.1protein NEL isoform X2
AAEL00836	leucine-rich repeat-containing protein 23	-0.418264936012299	0.00269263462846975	0.0796708162417318	NA	NA	KXJ62405.1 hypothet	XP_001653208.1leucine-rich repeat-containing protein 23
AAEL00586	succinate dehydrogenase assembly factor 2, mitochondrial	-0.418134813093601	0.128774971713843	0.535335481333802	NA	NA	XP_019526916.1 PRE	XP_001651508.2succinate dehydrogenase assembly factor 2, mitochondrial-like
AAEL02478	NA	-0.417965538256085	0.0988659549992854	0.476815785064688	NA	NA	NA	XP_021694081.1uncharacterized protein LOC5571232 isoform X2
AAEL01834	NA	-0.417944769941358	0.0467757966408179	0.345031868617454	NA	NA	NA	KFB49544.1AGAP008403-like protein
AAEL00254	cystinosin homolog	-0.416331044048791	0.0617322964054292	0.387441038933757	NA	NA	XP_019544837.1 PRE	XP_021692930.1cystinosin homolog
AAEL00705	recQ-mediated genome instability protein 1	-0.415762458291465	0.155975415640393	0.573884755986765	NA	aag03460	KXJ73189.1 hypothet	XP_001652517.1recQ-mediated genome instability protein 1
AAEL02304	NA	-0.41569857892367	0.0652079333479371	0.39730198438886	NA	NA	NA	NA
AAEL01006	putative uncharacterized protein DDB_G0277255	-0.415655487254168	0.0290623317186461	0.275227088745645	NA	NA	NA	XP_019558193.1 probable serine/threonine-protein kinase DDB_G0272282
AAEL02626	NA	-0.415526330319885	0.241512467977007	0.67673499705372	NA	aag01100 ; aag	NA	XP_021702924.1adenylate cyclase type 3
AAEL02021	NA	-0.415406260669213	0.128346204458512	0.535049122410476	NA	NA	NA	XP_001660840.2dr1-associated corepressor homolog
AAEL02490	NA	-0.415383003321182	0.343258601324926	0.763685015811799	NA	NA	NA	XP_021706245.1F-box/LRR-repeat protein 2-like
AAEL01201	L-lactate dehydrogenase	-0.41516287244466	0.115912672347454	0.512764772622422	00640 ; 00270 ; 00620 ;	aag01100 ; aag	KXJ76135.1 hypothet	XP_001662150.1L-lactate dehydrogenase
AAEL00225	fringe glycosyltransferase	-0.415091190334067	0.167546880300487	0.587554508928878	NA	aag04330 ; aag	XP_017030345.1 PRE	XP_021696086.1fringe glycosyltransferase
AAEL01714	NA	-0.414840261490759	0.317712724127444	0.745684464362801	NA	NA	NA	XP_019564523.1 pro-resilin-like
AAEL00933	bone morphogenetic protein receptor type-1B	-0.414737385706092	0.0748023641147145	0.419458473238027	NA	aag04013 ; aag	NA	XP_001653798.2bone morphogenetic protein receptor type-1B
AAEL00258	protein obstructor-E	-0.414428061614902	0.176219370131381	0.601026925110811	NA	NA	CRL07705.1 CLUMA_	XP_001655685.1protein obstructor-E
AAEL01113	UPF0505 protein CG8202	-0.413880474462453	0.064410668412112	0.395929840165835	NA	NA	KFB43738.1 AGAP00	XP_001655113.1UPF0505 protein CG8202
AAEL01265	uncharacterized LOC5576615	-0.413120189749406	0.0421894939544052	0.323939778330243	NA	NA	NA	XP_001662769.2uncharacterized protein LOC5576615
AAEL00256	serine/threonine-protein kinase greatwall	-0.412215516254033	0.199467383824297	0.633375309392567	NA	NA	XP_019549636.1 PRE	XP_021713415.1serine/threonine-protein kinase greatwall isoform X1
AAEL00043	epoxide hydrolase 4	-0.411868217436816	0.00341248362711597	0.0899761442312928	NA	NA	KXJ70524.1 hypothet	XP_001656425.2epoxide hydrolase 4
AAEL02334	NA	-0.411787890943948	0.0682970778648811	0.401556773593974	NA	aag04214	NA	XP_021696579.1 ecdysone-induced protein 74EF
AAEL02756	NA	-0.411696041200981	0.0627706104790583	0.391603496697149	NA	NA	NA	XP_021712145.1uncharacterized protein LOC110680661
AAEL00405	zinc finger protein 2	-0.411493700008345	0.0803646498450366	0.433079891352508	NA	NA	KXJ77489.1 hypothet	XP_021703820.1zinc finger protein 2
AAEL01201	uncharacterized LOC5575710	-0.410807830796463	0.0157203967400217	0.202259474938964	NA	NA	KXJ72264.1 hypothet	XP_001662159.1uncharacterized protein LOC5575710 isoform X2
AAEL01984	NA	-0.410792070595144	0.00240713470735542	0.0760003211995279	NA	NA	NA	XP_021699760.1uncharacterized protein LOC5567708 isoform X1
AAEL01134	uncharacterized LOC5574705	-0.410490782317153	0.0435488828312713	0.329553305767565	NA	NA	KXJ75235.1 hypothet	XP_021697228.1uncharacterized protein LOC5574705
AAEL01277	cytochrome P450 4C1	-0.410107349798184	0.0250538370049304	0.258627500477641	NA	NA	XP_019543095.1 PRE	XP_001662864.3cytochrome P450 4C1
AAEL00531	tetratricopeptide repeat protein 7B	-0.409983152577008	0.064580856195341	0.396446323355255	NA	NA	KXJ75584.1 hypothet	XP_021705407.1tetratricopeptide repeat protein 7B
AAEL02126	NA	-0.409853197970641	0.308221845504235	0.741056393558619	NA	NA	NA	XP_021706587.1uncharacterized protein LOC110678272
AAEL00891	uncharacterized LOC5571217	-0.409329226827526	0.212518389669315	0.645843785766909	NA	NA	KXJ72631.1 hypothet	XP_001659600.2uncharacterized protein LOC5571217
AAEL02808	NA	-0.409303069706289	0.0550115817368965	0.368425454980292	NA	NA	NA	NA
AAEL01016	uncharacterized LOC5572963	-0.409253581580645	0.0210911745774407	0.23689494093567	NA	NA	NA	XP_001654292.2uncharacterized protein LOC5572963
AAEL00495	zinc finger and SCAN domain-containing protein 2	-0.409081858997516	0.333120150941039	0.755603950945478	NA	NA	KXJ80831.1 hypothet	XP_001650078.2zinc finger and SCAN domain-containing protein 2
AAEL00349	uncharacterized protein C2orf42 homolog	-0.409078449302612	0.176086668357242	0.600797004902981	NA	NA	KXJ71384.1 hypothet	XP_001663805.2uncharacterized protein C2orf42 homolog
AAEL02050	NA	-0.409002712936515	0.0142159892472986	0.19121517032758	62	NA	NA	XP_021693423.1elongation of very long chain fatty acids protein AAEL008004 isoform X1
AAEL02497	NA	-0.407841655118503	0.22407831560126	0.656259917301973	NA	NA	NA	XP_021697594.1DNA repair protein SWI5 homolog
AAEL01949	NA	-0.406982499807262	0.00141881366380464	0.0539500964228526	NA	NA	NA	XP_021709682.1uncharacterized protein LOC5575021 isoform X2
AAEL00070	very-long-chain 3-oxoacyl-CoA reductase	-0.40683947606616	0.035671366808702	0.299394338432267	NA	NA	XP_001846139.1 ster	XP_001650278.2very-long-chain 3-oxoacyl-CoA reductase
AAEL02503	NA	-0.40629571393879	0.0682996601182201	0.401556773593974	NA	NA	NA	XP_021704096.1uncharacterized protein LOC5564068
AAEL00275	tropomyosin-1	-0.406141802890388	0.00338553057713629	0.0897536781871071	NA	NA	KFB42387.1 AGAP00	XP_019532838.1 tropomyosin-1
AAEL00254	uncharacterized LOC5580171	-0.406134769202499	0.0145428533585988	0.193517439045371	NA	NA	KFB42333.1 AGAP00	KFB42333.1AGAP001624-like protein
AAEL01414	zinc finger protein 2	-0.405923506148824	0.10027289877373	0.480169431404357	NA	NA	NA	XP_001648100.2zinc finger protein 2
AAEL00203	uncharacterized LOC5573177	-0.40570998508266	0.0413075010392847	0.320441432491577	NA	NA	KXJ82229.1 hypothet	XP_001660794.2uncharacterized protein LOC5573177
AAEL02449	NA	-0.405500897308508	0.139417860364807	0.549341242757296	NA	NA	NA	XP_021709139.1uncharacterized protein LOC110679247
AAEL00409	protein SERAC1	-0.405344935867357	0.241870732416349	0.676742771771529	NA	NA	XP_019536325.1 PRE	XP_021711028.1protein SERAC1 isoform X1
AAEL01942	NA	-0.404510581966317	0.0771659445345133	0.424916680487529	NA	NA	NA	XP_021709778.1uncharacterized protein LOC5568949
AAEL00956	apolipoprotein D	-0.404234765978893	0.00626917844523429	0.129262654794788	NA	NA	NA	XP_001660225.2apolipoprotein D
AAEL00180	INO80 complex subunit C	-0.403690737835952	0.230505440701975	0.663469210303275	NA	NA	KFB49559.1 AGAP00	XP_001660390.1INO80 complex subunit C
AAEL00956	apolipoprotein D	-0.403408285456121	0.0155657436386601	0.201233331073453	NA	NA	NA	XP_001660233.2apolipoprotein D
AAEL00887	2-acylglycerol O-acyltransferase 2	-0.403161467058547	0.0321534710606386	0.284442054201394	NA	aag00561	XP_019530978.1 PRE	XP_001653515.12-acylglycerol O-acyltransferase 2 isoform X2
AAEL00777	uncharacterized LOC5569635	-0.402898632874637	0.00753419114018122	0.142155587215123	NA	NA	KXJ76606.1 hypothet	AAL76030.1putative 30.5 kDa secreted protein
AAEL00890	uncharacterized LOC5571213	-0.402524385694924	0.151190982627136	0.566025396924239	NA	NA	KXJ76214.1 hypothet	XP_021696002.1uncharacterized protein LOC5571213
AAEL00747	probable cytochrome P450 308a1	-0.402308025771257	0.00344940273479819	0.0901744430841277	NA	NA	KXJ69081.1 hypothet	XP_001658387.1probable cytochrome P450 308a1
AAEL00066	probable maltase	-0.401872917512108	0.00613054589357231	0.128094552708606	NA	NA	KXJ74658.1 hypothet	XP_001649784.1probable maltase
AAEL01288	venom carboxylesterase-6	-0.401623284118614	0.0314872452875687	0.28423411888152	NA	NA	NA	XP_001663023.1venom carboxylesterase-6
AAEL02155	NA	-0.40092580052948	0.00121286815367407	0.0489509331145123	NA	NA	NA	XP_021696081.1F-actin-methionine sulfoxide oxidase Mical isoform X3
AAEL00342	uncharacterized LOC5578122	-0.400696975410954	0.00384976327823331	0.0960041238111189	NA	NA	NA	XP_001656822.2uncharacterized protein LOC5578122 isoform X1
AAEL00241	NA	-0.400671035524792	0.00453386936654193	0.105355216946765	NA	NA	XP_019554020.1 PRE	XP_001655221.1troponin T, skeletal muscle isoform X3
AAEL01968	NA	-0.400162213331953	0.00752041547874608	0.142155587215123	NA	NA	NA	XP_021702036.1nucleoporin nup124 isoform X2
AAEL00878	NA	-0.400073405101116	0.0104871534917785	0.165036735008546	NA	NA	ETN63584.1 hypothe	XP_001659510.2uncharacterized protein LOC5571070

AAEL01355	zinc finger protein 37 homolog	-0.399660671978415	0.108794814487655	0.495854325366716	NA	NA	NA	XP_001663743.2zinc finger protein 37 homolog
AAEL00103	methyl-CpG-binding domain protein 3	-0.399514626079239	0.161142611596049	0.5783128882372	NA	NA	XP_001848947.1 me	XP_021710255.1methyl-CpG-binding domain protein 3 isoform X4
AAEL00599	disintegrin and metalloproteinase domain-containing protein 12	-0.399430750429526	0.170291564019305	0.590883473644663	NA	NA	KXJ75381.1 hypothet	XP_021693626.1disintegrin and metalloproteinase domain-containing protein 12
AAEL00915	NA	-0.399415659558132	0.00209377050495274	0.0718913290543847	NA	NA	XP_019553430.1 PRE	XP_021701247.1solute carrier family 41 member 1 isoform X2
AAEL01405	V-type proton ATPase 116 kDa subunit a	-0.399314980552928	0.0040436360127677	0.0989615388018307	NA	aag01100 ; aag	NA	XP_021706364.1V-type proton ATPase 116 kDa subunit a isoform X1
AAEL00377	autophagy-related protein 2 homolog A	-0.398871843879363	0.00192001095522328	0.0684803907362971	NA	aag04140 ; aag	ETN57873.1 autopha	XP_021712972.1autophagy-related protein 2 homolog A
AAEL02688	NA	-0.39869260105774	0.418764319696941	0.813348977896628	NA	NA	NA	XP_021705876.1polyadenylate-binding protein 1-B-like
AAEL02483	NA	-0.398388390803594	0.185970424984574	0.613809128661423	NA	NA	NA	XP_021712895.1uncharacterized protein LOC110681442
AAEL00760	general odorant-binding protein 56d	-0.398380793529794	0.00576436357043181	0.121659801777783	NA	NA	AGI04311.1 obp10 [A	XP_001658489.1general odorant-binding protein 56d
AAEL01442	gamma-1-syntrophin	-0.398341120365907	0.0607314428194728	0.384941803020695	NA	NA	NA	XP_019529557.1 gamma-1-syntrophin-like isoform X2
AAEL02064	NA	-0.397833753544142	0.00261514328607266	0.0792437883464128	NA	NA	NA	XP_021695737.1transmembrane protein 35A
AAEL00083	spermatogenesis-associated protein 5-like protein 1	-0.397061712442712	0.146559085141135	0.561212769576399	NA	NA	KXJ69868.1 hypothet	XP_001651297.1spermatogenesis-associated protein 5-like protein 1
AAEL01480	regulator of hypoxia-inducible factor 1	-0.39613863670439	0.0791201314965605	0.430297547299852	NA	NA	NA	XP_021697472.1regulator of hypoxia-inducible factor 1
AAEL00945	uncharacterized LOC5571975	-0.395633328178196	0.18665884387985	0.614196367514984	NA	NA	NA	XP_019547042.1 SET and MYND domain-containing protein 4
AAEL01044	protein lines	-0.395610690512843	0.231965953337857	0.666006459474246	NA	NA	NA	XP_021705674.1protein lines
AAEL00187	alpha-(1,3)-fucosyltransferase 10	-0.395588629380454	0.146132673111179	0.560092931004593	NA	NA	XP_001870924.1 fuc	XP_001654141.2alpha-(1,3)-fucosyltransferase 10 isoform X1
AAEL00596	actin-1-like	-0.395557811009659	0.00857609584326027	0.149464458238032	NA	aag04145 ; aag	CRK90035.1 CLUMA_	XP_019541792.1 actin-1-like
AAEL02597	NA	-0.395333658938533	0.0022112391685504	0.0737239957572492	NA	NA	NA	XP_021698741.1uncharacterized protein LOC5564765 isoform X3
AAEL01242	DE-cadherin	-0.394891203836921	0.0524386654372993	0.362633125106993	NA	NA	XP_001861552.1 DE-	XP_019528694.1 DE-cadherin-like
AAEL01485	neurogenic locus notch homolog protein 2	-0.394717269441358	0.0980662212383023	0.473943787004209	NA	NA	NA	XP_001649758.1neurogenic locus notch homolog protein 2
AAEL00310	uncharacterized LOC5577063	-0.393765367987196	0.091940543268344	0.46005268034546	NA	NA	XP_019933048.1 PRE	ABF18169.1putative salivary mucin 4
AAEL00042	glycerol-3-phosphate acyltransferase 3	-0.393731705156424	0.00607853441710595	0.127704734489061	NA	aag01100 ; aag	XP_019540734.1 PRE	XP_021708939.1glycerol-3-phosphate acyltransferase 3 isoform X2
AAEL01140	perlucin-like protein	-0.393562719751063	0.0159000673359643	0.203211693924366	NA	NA	KXJ74383.1 hypothet	XP_001661648.2perlucin-like protein
AAEL02758	NA	-0.393291407828142	0.02226356914032	0.242316778828459	NA	NA	NA	XP_021701404.1cGMP-dependent protein kinase 1
AAEL01002	NA	-0.392679192164227	0.11026820132295	0.50033924485887	NA	NA	NA	XP_001660568.1serine/threonine-protein kinase tricorner
AAEL01983	NA	-0.392285387093524	0.0998704447843921	0.47964918210124	NA	NA	NA	XP_021707813.1uncharacterized protein LOC5565745
AAEL00666	DNA-directed RNA polymerase II 16 kDa polypeptide	-0.392145111461316	0.0221321375087107	0.242070925206103	NA	aag03020	XP_001849109.1 DNA	XP_001652151.1DNA-directed RNA polymerase II 16 kDa polypeptide
AAEL01815	NA	-0.392017318834397	0.330224004208971	0.753838076589172	NA	NA	NA	XP_021700793.1homeobox protein aristaless
AAEL00758	uncharacterized LOC5579959	-0.391791626666545	0.0127341339260136	0.182308324322341	NA	NA	KXJ70544.1 hypothet	XP_021694056.1uncharacterized protein LOC5579959
AAEL02358	NA	-0.391726512956728	0.250898748731833	0.683858606596939	NA	NA	NA	XP_021697329.1transmembrane protease serine 11G-like
AAEL00043	glypican-4	-0.391683745114947	0.118462444952893	0.51638627117789	NA	aag04310	KXJ80119.1 hypothet	XP_019558269.1 glypican-6
AAEL00450	probable serine hydrolase	-0.39157864146562	0.0938274477570671	0.464823706598816	NA	NA	KXJ78144.1 hypothet	XP_001649281.2probable serine hydrolase
AAEL00877	laminin subunit alpha	-0.391424913184522	0.00381197830844254	0.0958410502576181	NA	aag04512	KXJ73623.1 hypothet	XP_001659481.2laminin subunit alpha
AAEL00367	histone-lysine N-methyltransferase SMYD3	-0.391422374053543	0.062186711916872	0.389280355822487	NA	aag01100 ; aag	XP_001867383.1 con	XP_001657162.2histone-lysine N-methyltransferase SMYD3
AAEL01304	nitrilase and fragile histidine triad fusion protein NitFhit	-0.390984927386297	0.153509313935797	0.568905641094322	230	NA	NA	XP_001663254.2nitrilase and fragile histidine triad fusion protein NitFhit
AAEL00439	follicle-stimulating hormone receptor	-0.389104965361112	0.285050592631657	0.717656786154995	NA	aag04080	XP_019527003.1 PRE	XP_021692996.1follicle-stimulating hormone receptor
AAEL00812	putative fatty acyl-CoA reductase CG5065	-0.388573697335492	0.128110952592163	0.53463808877691	73	aag04146	KFB46838.1 AGAP00	XP_001658954.1putative fatty acyl-CoA reductase CG5065
AAEL02584	NA	-0.388251785210972	0.151255862968562	0.566025396924239	NA	NA	NA	NA
AAEL01071	low-density lipoprotein receptor-related protein 4	-0.388048173668193	0.0136523753608399	0.187831676988403	NA	NA	XP_019559720.1 PRE	XP_001661035.2low-density lipoprotein receptor-related protein 4 isoform X1
AAEL01942	NA	-0.388013863646302	0.0319758409841047	0.284442054201394	NA	NA	NA	XP_021702832.1uncharacterized protein LOC5577896 isoform X2
AAEL02674	NA	-0.387535555159981	0.371026228777746	0.781817769662763	NA	NA	NA	XP_019559684.1 caspase-1-like
AAEL01223	NA	-0.386456520813527	0.00567887604879571	0.120826935771257	NA	aag04310 ; aag	KXJ80090.1 hypothet	XP_021694469.1myc protein
AAEL02161	NA	-0.386056398162517	0.0531735430806033	0.364607260378325	NA	NA	NA	XP_021703241.1fasciclin-1 isoform X4
AAEL00706	beta-1,3-glucan-binding protein	-0.38603418529712	0.00509723024288796	0.114123388552445	NA	NA	KXJ75885.1 hypothet	XP_001652521.1beta-1,3-glucan-binding protein
AAEL02273	NA	-0.385979812943866	0.271076620123789	0.706442100928661	NA	NA	NA	XP_021713239.1ATP-binding cassette sub-family G member 8 isoform X1
AAEL02188	NA	-0.385543457129691	0.0137003339243226	0.187831676988403	NA	NA	NA	XP_019932919.1 lateral signaling target protein 2 homolog
AAEL01156	ankyrin-3	-0.38431984439581	0.027722408821411	0.271096286901832	NA	aag04624	XP_019544057.1 PRE	XP_021709686.1ankyrin-3 isoform X2
AAEL00792	retinoid-inducible serine carboxypeptidase	-0.384242246650947	0.00713007213924243	0.137861850831157	NA	NA	KFB47556.1 AGAP01	XP_001658722.1retinoid-inducible serine carboxypeptidase
AAEL02591	NA	-0.384020391734045	0.00665048605618502	0.133911975249485	NA	NA	NA	XP_021696289.1aryl hydrocarbon receptor nuclear translocator-like protein 1 isoform X2
AAEL01163	WAP, Kazal, immunoglobulin, Kunitz and NTR domain-containing protein	-0.383803380561934	0.00519413504273014	0.115172122080007	NA	NA	KXJ70015.1 hypothet	XP_001661812.1WAP, Kazal, immunoglobulin, Kunitz and NTR domain-containing protein 2
AAEL00797	P protein	-0.383703396042731	0.0287751807099643	0.274601260220884	NA	NA	XP_019931581.1 PRE	XP_021703436.1P protein isoform X3
AAEL01179	uncharacterized LOC5576092	-0.383610125895216	0.196887574821074	0.630345746481079	NA	NA	KXJ75527.1 hypothet	XP_021711072.1uncharacterized protein LOC5576092
AAEL00486	major royal jelly protein 1	-0.383480753452953	0.0650689544955012	0.39730198438886	NA	NA	XP_001850268.1 yell	XP_021693794.1major royal jelly protein 1
AAEL00271	arrestin domain-containing protein 2	-0.382236438815454	0.238002529645662	0.672634913329047	NA	NA	KXJ80134.1 hypothet	XP_001662207.2arrestin domain-containing protein 2
AAEL00362	sodium-dependent nutrient amino acid transporter 1	-0.382041797974646	0.0145128640184697	0.193517439045371	NA	NA	XP_019558048.2 PRE	XP_001657071.2sodium-dependent nutrient amino acid transporter 1
AAEL02439	NA	-0.382009748581039	0.201903155953586	0.634701781910801	NA	NA	NA	XP_021709459.1ALK tyrosine kinase receptor
AAEL01351	pupal cuticle protein Edg-84A	-0.381996990718318	0.171064351690184	0.592447935360585	NA	NA	NA	XP_001663697.1pupal cuticle protein Edg-84A
AAEL00012	luciferin 4-monooxygenase	-0.38167935155299	0.162675709930717	0.581228146321853	NA	NA	KXJ68569.1 hypothet	XP_001657679.2luciferin 4-monooxygenase
AAEL00887	kynurenine 3-monooxygenase	-0.381397497729541	0.0232862674568667	0.247436758819963	380	aag01100 ; aag	XP_019531555.1 PRE	XP_001653516.2kynurenine 3-monooxygenase
AAEL01254	PI-PLC X domain-containing protein 3	-0.380754391357859	0.0833473575355471	0.438013925780757	NA	NA	ETN61918.1 glycosyl	XP_021700218.1PIC X domain-containing protein 3
AAEL01442	glycine dehydrogenase (decarboxylating), mitochondrial	-0.380618448382248	0.0280494119824631	0.271500221524543	260	aag01100 ; aag	NA	XP_001648761.2 glycine dehydrogenase (decarboxylating), mitochondrial
AAEL01037	multidrug resistance protein homolog 49	-0.380613638318054	0.0719807531074699	0.412176036151175	NA	aag02010	NA	XP_001654492.2multidrug resistance protein homolog 49
AAEL00054	uncharacterized LOC5563685	-0.380445881928163	0.0179964479089359	0.217327183278252	NA	NA	KXJ76095.1 hypothet	XP_019931012.1 serine/arginine-rich splicing factor 1-like
AAEL02019	NA	-0.380274529985495	0.199640054896764	0.633375309392567	NA	NA	NA	XP_021713228.1uncharacterized protein LOC5577057 isoform X1
AAEL00516	sodium-independent sulfate anion transporter	-0.379881938129418	0.0141409436186968	0.19051971182906	NA	NA	XP_019525168.1 PRE	XP_021708981.1sodium-independent sulfate anion transporter isoform X1
AAEL01264	uncharacterized LOC5565029	-0.379114998759069	0.234326837684798	0.668405319397245	NA	NA	NA	XP_021706847.1uncharacterized protein LOC5565029 isoform X1
AAEL00620	exonuclease 1	-0.378847862147031	0.318121146623412	0.745684464362801	NA	aag03430	KXJ82279.1 hypothet	XP_001657573.2exonuclease 1
AAEL02425	NA	-0.378684523396366	0.223020782608594	0.655580227356264	NA	NA	NA	ABI64123.1insulin-like peptide 7 precursor
AAEL02414	NA	-0.378375503658295	0.0699883987843589	0.407100660944166	NA	aag04391	NA	XP_019536511.1 hemicentin-2-like isoform X1
AAEL01105	DNA repair protein complementing XP-A cells homolog	-0.378357804092819	0.101436187240815	0.483635126937812	NA	aag03420	KXJ68622.1 hypothet	XP_001661332.1DNA repair protein complementing XP-A cells homolog
AAEL02814	NA	-0.377751679330187	0.032723258207556	0.28463170766102	NA	NA	NA	XP_019551178.1 ficolin-3-like
AAEL01179	heavy metal-associated isoprenylated plant protein 32	-0.377524315609296	0.318177403503916	0.745684464362801	NA	NA	KFB41784.1 AGAP00	XP_001661916.1heavy metal-associated isoprenylated plant protein 32
AAEL02732	NA	-0.377441535891677	0.0751783557261425	0.420011717015769	NA	NA	NA	XP_021710212.1EH domain-binding protein 1 isoform X4
AAEL00772	calumenin	-0.377388696172676	0.00539214883784895	0.117414467687443	NA	NA	XP_001843856.1 calu	XP_019565135.1 calumenin-like
AAEL02616	NA	-0.377355278306294	0.0197136209220689	0.227335564593981	NA	NA	NA	XP_021707411.1uncharacterized protein LOC5575323 isoform X2
AAEL00201	cell division cycle protein 123 homolog	-0.377257175158044	0.0819110795892354	0.435691187502974	NA	NA	KXJ70688.1 hypothet	XP_001660790.2cell division cycle protein 123 homolog
AAEL00601	mitoguardin	-0.377018067491616	0.0371960638788337	0.304789118266276	NA	NA	KXJ75252.1 hypothet	XP_019537482.1 mitoguardin-like
AAEL02048	NA	-0.376219351647128	0.01249243137405	0.180747411169824	NA	NA	NA	XP_021701446.1uncharacterized protein LOC5578768
AAEL00632	putative ferric-chelate reductase 1 homolog	-0.376130313271324	0.00320034853520158	0.088916783130334	NA	NA	XP_019562157.1 PRE	XP_021698252.1putative ferric-chelate reductase 1 homolog isoform X1
AAEL00310	uncharacterized LOC5577059	-0.375634824184569	0.0969681563821132	0.470633908275356	NA	NA	KXJ76610.1 hypothet	XP_001663159.2uncharacterized protein LOC5577059
AAEL00193	ATP-binding cassette sub-family A member 1	-0.375607277207726	0.153253931748726	0.568818435996349	NA	NA	KXJ73911.1 hypothet	XP_001654315.1ATP-binding cassette sub-family A member 1
AAEL00675	protein TSSC4	-0.374819010318747	0.225405972747002	0.658262697942846	NA	NA	KXJ79498.1 hypothet	XP_001652183.1protein TSSC4

AAEL0042(	DNA helicase MCM8	-0.374794993749012	0.146743529308952	0.561469420665687	NA	NA	KXJ83158.1 hypothet	XP_021705904.1DNA helicase MCM8
AAEL0092⁄	neurotactin	-0.374488999369319	0.094949023826281	0.466731259214443	NA	NA	NA	XP_019525918.1 neurotactin-like
AAEL0003:	cleavage and polyadenylation specificity factor subunit CG7185	-0.373899635136107	0.0769656381644058	0.42460299903409	NA	NA	KFB52034.1 AGAP00	XP_001655573.2cleavage and polyadenylation specificity factor subunit CG7185
AAEL0088!	CDK-activating kinase assembly factor MAT1	-0.373496468462324	0.140324620740792	0.550646976570049	NA	aag03420 ; aag	KFB49087.1 AGAP00	XP_001653470.1CDK-activating kinase assembly factor MAT1
AAEL0035⁄	gamma-tubulin complex component 4 homolog	-0.373113715005685	0.0681009635044172	0.401232883533236	NA	NA	KXJ80500.1 hypothet	XP_001656936.1gamma-tubulin complex component 4 homolog
AAEL0123⁄	NA	-0.372955425905473	0.126224345304338	0.530481545423179	NA	NA	XP_019534814.1 PRE	XP_001662494.2lipase 1
AAEL0140!	copper homeostasis protein cutC homolog	-0.371801784011365	0.223677204645051	0.655920215788323	NA	NA	NA	XP_001664302.1copper homeostasis protein cutC homolog
AAEL0195⁊	NA	-0.371150096884724	0.165465193830403	0.584439853413553	NA	NA	NA	XP_021711334.1zinc finger protein 91 isoform X3
AAEL0135⁊	protein king tubby 2	-0.370745300918413	0.310706567696142	0.742918550320373	NA	NA	NA	XP_021707309.1protein king tubby 2
AAEL01431	uncharacterized LOC5564099	-0.370691464741346	0.00399567663155817	0.098458066803174	NA	NA	NA	XP_001648425.2uncharacterized protein LOC5564099 isoform X1
AAEL0141ɛ	E3 ubiquitin-protein ligase TRIM37	-0.370278645154358	0.19014621295888	0.618521820583848	NA	NA	NA	XP_001648168.1E3 ubiquitin-protein ligase TRIM37
AAEL0198ɛ	NA	-0.369877210196621	0.0896660410660625	0.45743198048147	NA	NA	NA	XP_021709246.1calcineurin-binding protein cabin-1 isoform X5
AAEL0060ɛ	NADPH-dependent diflavin oxidoreductase 1-like	-0.369820100908301	0.32348003490918	0.74762391301976	NA	NA	XP_019544968.1 PRE	XP_021711981.1NADPH-dependent diflavin oxidoreductase 1-like
AAEL0151⁊	uncharacterized LOC5566324	-0.369377158722775	0.179432955593711	0.604797111430702	NA	NA	NA	XP_001650709.1uncharacterized protein LOC5566324
AAEL0033⁹	uncharacterized LOC5578020	-0.369173468676245	0.268417449303967	0.704184172798867	NA	NA	KXJ82984.1 hypothet	XP_001656760.1uncharacterized protein LOC5578020
AAEL0196:	NA	-0.369124306453401	0.0138340274138546	0.188314674944216	NA	NA	NA	XP_021698333.1protein furry isoform X4
AAEL0136ɛ	WD repeat-containing protein 89	-0.36873228810638	0.129399093293708	0.53564123098907	NA	NA	NA	XP_001660585.2WD repeat-containing protein 89
AAEL0172⁊	phosphatidylinositol 3,4,5-trisphosphate 3-phosphatase and dual-specifi	-0.367924564360109	0.0144654859840155	0.193195068250958	NA	NA	NA	XP_019542897.1 box A-binding factor-like isoform X1
AAEL0093ɛ	protein Skeletor, isoforms B/C	-0.367858895740592	0.031604325676385	0.28423411888152	NA	NA	NA	XP_021712422.1protein Skeletor, isoforms D/E isoform X2
AAEL0029⁹	nicotinamidase	-0.367508491549564	0.159680173330679	0.577560236664366	NA	NA	XP_321695.5 AGAP0	XP_021711481.1uncharacterized protein LOC5580264 isoform X2
AAEL0227(	NA	-0.367500373947426	0.0415246221659315	0.321207384249982	NA	NA	NA	---NA---
AAEL0229ɛ	NA	-0.367440716177318	0.0101630185954466	0.162643647157043	NA	NA	NA	XP_021697958.1basic proline-rich protein
AAEL0109⁄	alpha-tocopherol transfer protein-like	-0.366369761599935	0.0335759460262508	0.288214417288769	NA	NA	ETN60609.1 phospha	XP_001661206.1alpha-tocopherol transfer protein-like
AAEL0217!	NA	-0.366254781629416	0.176622906085959	0.601511466248332	NA	NA	NA	XP_021694165.1leucine-rich repeat protein soc-2 homolog
AAEL0270(	NA	-0.36624571516257	0.133992984344148	0.542662316284181	NA	NA	NA	XP_021706483.1phospholipase D1
AAEL0156:	NA	-0.366198244335948	0.1450565373039	0.558498015175938	250	aag01100 ; aag	NA	XP_001647586.2asparagine synthetase [glutamine-hydrolyzing] 1
AAEL0206:	NA	-0.366125581604949	0.343988553563223	0.764585185963472	NA	NA	NA	XP_021703444.1zinc finger protein 583-like
AAEL0036ɛ	NA	-0.366045295795247	0.322617950614777	0.746650319748046	NA	NA	XP_001861495.1 sele	XP_021701458.1selenoprotein H
AAEL0054ɛ	A disintegrin and metalloproteinase with thrombospondin motifs 12	-0.36603545909369	0.339197991517846	0.761115461750945	NA	NA	XP_019540412.1 PRE	XP_021704593.1A disintegrin and metalloproteinase with thrombospondin motifs 12
AAEL0027⁊	nuA4 complex subunit EAF3 homolog	-0.366028187397501	0.115365610534081	0.511858412793931	NA	NA	KXJ68922.1 hypothet	XP_001655962.2nuA4 complex subunit EAF3 homolog
AAEL0012⁄	PI-PLC X domain-containing protein 1	-0.365704777838755	0.202970652289673	0.635896319876174	NA	NA	KXJ79292.1 hypothet	XP_021696659.1PIC X domain-containing protein 1
AAEL0099⁄	retinal dehydrogenase 1	-0.36545331098855	0.0406623721548326	0.316829084308865	NA	NA	NA	XP_001660495.2retinal dehydrogenase 1
AAEL0223⁊	NA	-0.365093950051597	0.234952024478247	0.668737559309875	NA	NA	NA	XP_021696706.1tyrosine-protein kinase PR2
AAEL0234⁊	NA	-0.364933625582179	0.0168567471834701	0.210469182608266	NA	NA	NA	XP_021699291.1fibroin heavy chain
AAEL0100(	inverted formin-2	-0.364827885343962	0.0152606865829531	0.198907702459398	NA	NA	NA	XP_001660600.2serine/arginine repetitive matrix protein 2 isoform X1
AAEL0015⁹	uncharacterized LOC5571482	-0.364792140518247	0.437627522151838	0.820960220424486	NA	NA	XP_001844764.1 con	XP_001659740.2uncharacterized protein LOC5571482
AAEL0086⁊	microtubule-associated protein RP/EB family member 1	-0.364684931810446	0.287694409280092	0.719789675158259	NA	NA	KXJ81658.1 hypothet	XP_001653411.2microtubule-associated protein RP/EB family member 1
AAEL0226!	NA	-0.364668092661962	0.111551473030048	0.502879021478986	NA	NA	NA	XP_021700978.1O-acyltransferase like protein
AAEL0082⁊	uncharacterized LOC5570366	-0.364345066308921	0.00632709863103367	0.129902019865584	NA	NA	NA	XP_001659087.2uncharacterized protein LOC5570366
AAEL0245⁹	NA	-0.364319274114394	0.0148484446088179	0.196597679554449	NA	NA	NA	XP_019558764.1 RNA polymerase-associated protein LEO1-like
AAEL01061	serine palmitoyltransferase 1	-0.363864664705668	0.0357383641959821	0.299512228899296	NA	aag01100 ; aag	KXJ71519.1 hypothet	XP_001660943.1serine palmitoyltransferase 1
AAEL0199:	NA	-0.363848226397206	0.38623539635454	0.791224994795546	NA	NA	NA	XP_001649853.2zinc finger protein 883
AAEL0080!	potassium/sodium hyperpolarization-activated cyclic nucleotide-gated ch	-0.363521714950996	0.147377862595301	0.561952643558699	NA	NA	NA	XP_021700035.1uncharacterized protein LOC5569977 isoform X2
AAEL0268⁹	NA	-0.363393701407489	0.188293405024457	0.615250207631631	NA	NA	NA	XP_021712937.1dnaJ homolog subfamily B member 12-like
AAEL0144!	probable medium-chain specific acyl-CoA dehydrogenase, mitochondrial	-0.363367640246489	0.00743778316454158	0.141410084049817	NA	aag01100 ; aag	NA	XP_001648811.1probable medium-chain specific acyl-CoA dehydrogenase, mitochondrial
AAEL0120(	ecdysteroid-regulated 16 kDa protein	-0.362211130167729	0.00412483947830038	0.0998862444192635	NA	aag04142	ADC29876.1 MDL2 [/	XP_001662215.1ecdysteroid-regulated 16 kDa protein
AAEL0241⁊	NA	-0.361332022913848	0.228037817284672	0.66175420142127	NA	NA	NA	NA
AAEL0000:	serine protease easter	-0.361039903189178	0.00627911396362423	0.129262654794788	NA	NA	KXJ72916.1 hypothet	XP_001647870.2serine protease easter
AAEL0246:	NA	-0.360621969170772	0.277251610703411	0.71264506192536	NA	NA	NA	NA
AAEL0035(	POU domain protein CF1A	-0.360469653547459	0.260246305593069	0.693219418655219	NA	NA	XP_001843890.1 pou	XP_021699519.1POU domain protein CF1A
AAEL0271⁊	NA	-0.360367705145506	0.090750674634924	0.458582874647784	NA	NA	NA	XP_021706892.1glycoprotein endo-alpha-1,2-mannosidase
AAEL0047⁊	general odorant-binding protein 66	-0.359687656069674	0.0441403499653098	0.332214793948945	NA	NA	KXJ74685.1 hypothet	XP_001649647.2general odorant-binding protein 66
AAEL0146(	cytochrome P450 9e2	-0.359191645421287	0.440612588458362	0.822798752521693	NA	NA	NA	XP_001649093.1cytochrome P450 9e2
AAEL0072:	uncharacterized LOC5568940	-0.35882473781782	0.00408574005006671	0.099726737243273	NA	NA	NA	ABF18366.1farnesoic acid o-methyltransferase-like protein
AAEL0084⁊	uncharacterized LOC5570622	-0.358507850644826	0.0705365679762906	0.408510018438117	NA	NA	XP_001850204.1 con	XP_001659225.2uncharacterized protein LOC5570622
AAEL0077!	nucleoside diphosphate-linked moiety X motif 8	-0.35818688039427	0.211510702759546	0.643837739594224	NA	NA	KXJ72245.1 hypothet	XP_001658626.2nucleoside diphosphate-linked moiety X motif 8
AAEL0083⁊	neurofibromin	-0.35777701748686	0.0755839909548996	0.420516274980827	NA	NA	ETN62306.1 neurofik	XP_021706870.1neurofibromin isoform X2
AAEL0065!	nuclear envelope phosphatase-regulatory subunit 1 homolog	-0.35770797172021	0.0855762261820168	0.444397535737539	NA	NA	ETN62964.1 hypothe	XP_001652053.1nuclear envelope phosphatase-regulatory subunit 1 homolog
AAEL0046⁊	uncharacterized LOC5565214	-0.357596686206074	0.263013684112594	0.696876452981309	NA	NA	NA	XP_021706966.1uncharacterized protein LOC5565214
AAEL0017(	ATP-dependent RNA helicase dbp2	-0.35719843104466	0.00331070940367282	0.0893406097730125	NA	NA	KXJ71844.1 hypothet	XP_001653950.2ATP-dependent RNA helicase dbp2
AAEL0247⁹	NA	-0.357186381363232	0.220421883835273	0.652412844728166	NA	NA	NA	NA
AAEL0149(	NA	-0.357045609820053	0.14549273350393	0.558950568057442	NA	NA	NA	XP_001650112.2uncharacterized protein LOC5565753
AAEL0089(	FAST kinase domain-containing protein 3, mitochondrial	-0.357021370652526	0.0446760469577529	0.33483791379173	NA	NA	KXJ77085.1 hypothet	XP_001659585.2FAST kinase domain-containing protein 3, mitochondrial
AAEL0201⁊	NA	-0.356755787227753	0.0939024506225051	0.464823706598816	NA	NA	NA	XP_021710654.1glucose dehydrogenase [FAD, quinone]
AAEL0069:	suppressor of cytokine signaling 7	-0.356651593877409	0.122821714887116	0.525286835462997	NA	NA	NA	XP_021707736.1suppressor of cytokine signaling 7
AAEL0097(	USP6 N-terminal-like protein	-0.356526757449872	0.105968502383462	0.490750960710929	NA	NA	NA	XP_001654003.2USP6 N-terminal-like protein isoform X2
AAEL0008!	protein O-mannosyltransferase 1	-0.355890888314042	0.357312816508867	0.773736338358957	00515 ; 00514	aag01100 ; aag	XP_001847722.1 O-n	XP_001651617.2protein O-mannosyltransferase 1
AAEL0059!	phospholipase B1, membrane-associated	-0.355838462021487	0.33196625593234	0.754446403331537	00564 ; 00591 ; 00565 ;	NA	KXJ69680.1 hypothet	XP_021706845.1phospholipase B1, membrane-associated
AAEL0036⁹	zinc finger protein 2 homolog	-0.355831329336087	0.284447564000815	0.716726857594605	NA	NA	KXJ74998.1 hypothet	XP_001657146.2zinc finger protein 2 homolog
AAEL0104:	protein groucho	-0.35554271186138	0.0888414731344449	0.455343393357453	NA	aag04013 ; aag	NA	XP_021705186.1protein groucho isoform X9
AAEL0123⁊	probable chitinase 2	-0.355372967792105	0.223627640499322	0.655920215788323	520	NA	KXJ81211.1 hypothet	XP_001662520.2probable chitinase 2
AAEL0137ɛ	uncharacterized LOC5578595	-0.354322998484252	0.0360361754219406	0.300639062767631	NA	NA	NA	XP_001657018.1uncharacterized protein LOC5578595
AAEL0105:	maltase 2	-0.354261095719613	0.19232986374036	0.622956496352973	NA	NA	XP_019548060.1 PRE	XP_001660906.2maltase 2 isoform X1
AAEL0061⁊	G protein-coupled receptor kinase 2	-0.353911841399446	0.104624663875378	0.488256999039335	NA	aag04144 ; aag	ETN63677.1 G protei	XP_021693404.1G protein-coupled receptor kinase 2
AAEL0254⁄	NA	-0.353906452827676	0.0667381578845269	0.399540230684495	NA	NA	NA	NA
AAEL0097:	iodotyrosine deiodinase 1	-0.353824490134481	0.236661626514976	0.670905818604686	NA	NA	NA	XP_021696829.1iodotyrosine deiodinase 1
AAEL00281	DNA replication licensing factor Mcm5	-0.353322707805593	0.303910461532515	0.737301362252096	NA	aag03030	ETN67461.1 DNA rep	XP_001662466.1DNA replication licensing factor Mcm5
AAEL0275⁊	NA	-0.353275569034309	0.271560984916393	0.706703671719641	NA	NA	NA	XP_021697905.1double-stranded RNA-specific editase Adar isoform X1
AAEL0199:	NA	-0.353188219758674	0.114589680005949	0.509398181359779	NA	NA	NA	XP_021696600.1uncharacterized protein LOC5569218
AAEL0208(	NA	-0.353118526125558	0.374608786295959	0.782198815065306	NA	NA	NA	XP_021693028.1ovochymase-2 isoform X1
AAEL0272:	NA	-0.353101323141699	0.154235447479635	0.570677357341215	NA	NA	NA	XP_001647590.2uncharacterized protein LOC5579282
AAEL0074(	uncharacterized LOC5569145	-0.352583713167571	0.0127786208637155	0.182308324322341	NA	NA	NA	XP_021699736.1uncharacterized protein LOC5569145

AAEL00022	uncharacterized family 31 glucosidase KIAA1161	-0.352186297298841	0.151040782047226	0.560025396924239	NA	NA	KXJ75975.1 hypothet	XP_001660136.1uncharacterized family 31 glucosidase KIAA1161
AAEL00802	uncharacterized LOC5569924	-0.352092266087861	0.0243939313115352	0.255291093250631	NA	NA	KXJ76905.1 hypothet	XP_021695837.1uncharacterized protein LOC5569924
AAEL01076	inositol-trisphosphate 3-kinase A	-0.351969534494122	0.134507605497298	0.542662316284181	NA	aag01100 ; aag	XP_019549290.1 PRE	XP_021701282.1inositol-trisphosphate 3-kinase A isoform X3
AAEL00976	transmembrane and TPR repeat-containing protein CG4341	-0.351488767951343	0.262053123987204	0.695532981520119	NA	NA	NA	XP_021695335.1transmembrane and TPR repeat-containing protein CG4341
AAEL00013	uncharacterized LOC5567750	-0.351249351478843	0.197285022042757	0.630372589300064	NA	NA	AGI04310.1 obp5 [A.	XP_019536842.1 general odorant-binding protein 67-like
AAEL01067	monocyte to macrophage differentiation factor	-0.350904539617019	0.0325994010770855	0.28463170766102	NA	NA	KXJ77678.1 hypothet	XP_001660993.1monocyte to macrophage differentiation factor
AAEL01052	protein eiger	-0.350548112458725	0.0287911725380627	0.274601260220884	NA	aag04013 ; aag	NA	XP_001654630.1protein eiger
AAEL00892	G protein-activated inward rectifier potassium channel 3	-0.35031516414335	0.0137781332283386	0.187831676988403	NA	NA	XP_019529555.1 PRE	XP_001653531.1G protein-activated inward rectifier potassium channel 3 isoform X3
AAEL00486	adipocyte plasma membrane-associated protein	-0.35020309089411	0.125900275229073	0.530481545423179	NA	NA	KXJ80336.1 hypothet	XP_001649960.1adipocyte plasma membrane-associated protein
AAEL01276	uncharacterized LOC5576787	-0.350132107030724	0.0176145285996049	0.214955509175633	NA	NA	KXJ72565.1 hypothet	XP_001662874.2uncharacterized protein LOC5576787
AAEL00170	uncharacterized LOC5572075	-0.350047848347686	0.120347133633353	0.521361270092351	NA	NA	ETN58974.1 hypothe	XP_001653843.1uncharacterized protein LOC5572075
AAEL00647	uncharacterized LOC5568048	-0.349853602716581	0.180417549474594	0.605728027045765	NA	NA	KXJ80818.1 hypothet	XP_001651984.1uncharacterized protein LOC5568048
AAEL00042	uncharacterized LOC5577339	-0.349685474609494	0.222905148980636	0.655580227356264	NA	NA	NA	XP_021705227.1uncharacterized protein LOC5577339 isoform X2
AAEL01821	NA	-0.349454060309238	0.0123870467715057	0.179504888805347	NA	NA	NA	XP_021705130.1basement membrane-specific heparan sulfate proteoglycan core protein isoform X1
AAEL01432	out at first protein	-0.349241837184143	0.0174016626828274	0.214364257037989	NA	NA	NA	XP_021701513.1out at first protein
AAEL01268	neither inactivation nor afterpotential protein G	-0.348933692184466	0.0862353040389072	0.447315258041727	NA	NA	KXJ76593.1 hypothet	XP_021708468.1neither inactivation nor afterpotential protein G
AAEL01208	tyrosine-protein phosphatase 10D	-0.347777320196145	0.00439288294646415	0.103594442242845	NA	NA	XP_019529910.1 PRE	XP_021705335.1tyrosine-protein phosphatase 10D isoform X8
AAEL00579	tyrocidine synthase 3	-0.34766000227147	0.0127658772605593	0.182308324322341	NA	NA	KXJ71980.1 hypothet	XP_001651463.2uncharacterized protein LOC5567063
AAEL00770	lipoma-preferred partner homolog	-0.347142760467956	0.0329190904548147	0.285244952713941	NA	aag04391	KXJ76959.1 hypothet	XP_001652822.2lipoma-preferred partner homolog
AAEL01947	NA	-0.347113427528962	0.334675659327314	0.757087156181969	NA	NA	NA	XP_021700587.1protein disks lost
AAEL00093	alkaline phosphatase 4	-0.346865023919194	0.0167223592340141	0.209527649977971	00790 ; 00730	aag01100 ; aag	KFB49884.1 AGAP01	XP_001657478.1alkaline phosphatase 4
AAEL01813	NA	-0.346214592694832	0.0271558381650316	0.268836665026084	NA	aag04013 ; aag	NA	XP_021703015.1mitogen-activated protein kinase kinase kinase 7
AAEL02495	NA	-0.346135143460517	0.191732630710866	0.621678529880687	NA	NA	NA	XP_019528163.2 lachesin-like
AAEL01063	zinc finger protein 502	-0.346090433811849	0.0824641600804849	0.437257081788889	NA	NA	XP_019550877.1 PRE	XP_021700870.1zinc finger protein 502
AAEL00438	hexosaminidase D	-0.345907415049441	0.254962229752781	0.687016819380699	00513 ; 00603 ; 00604 ;	aag01100 ; aag	KXJ71711.1 hypothet	XP_001649003.1hexosaminidase D
AAEL00246	probable chitinase 10	-0.345670195708664	0.602634461357781	0.892698376274034	NA	NA	KXJ77819.1 hypothet	XP_001655365.1probable chitinase 10
AAEL02040	NA	-0.345622083877374	0.111593019111073	0.502879021478986	NA	NA	NA	XP_021708436.1uncharacterized protein LOC5579261 isoform X1
AAEL01560	solute carrier family 25 member 45	-0.345557830728385	0.106225429074199	0.490844601505817	NA	NA	NA	XP_001662917.2solute carrier family 25 member 45 isoform X1
AAEL02207	NA	-0.345175802586673	0.25580723668129	0.68833867617105	NA	NA	NA	XP_021712647.1uncharacterized protein LOC110681180 isoform X2
AAEL00458	uncharacterized LOC5565096	-0.344644973588979	0.0075793643174105	0.142337368262881	NA	NA	NA	AAY41833.1putative secreted protein precursor
AAEL02297	NA	-0.344536641409691	0.242541152803401	0.677312119697343	NA	NA	NA	NA
AAEL00811	uncharacterized LOC5570131	-0.344497162182138	0.218399214125806	0.651909019364208	NA	NA	KXJ71021.1 hypothet	XP_001653111.1uncharacterized protein LOC5570131
AAEL00241	hydroxysteroid dehydrogenase-like protein 2	-0.344012560828659	0.0167154742878907	0.209527649977971	NA	NA	XP_019554042.1 PRE	XP_001655211.2hydroxysteroid dehydrogenase-like protein 2 isoform X2
AAEL01366	regucalcin	-0.343656910391605	0.253372073894229	0.685145258966747	NA	NA	NA	XP_021707378.1regucalcin isoform X2
AAEL00396	calpain-B	-0.343478024424285	0.151060726865632	0.560025396924239	NA	NA	NA	XP_001648123.2calpain-B isoform X5
AAEL00517	cytochrome c oxidase subunit 4 isoform 1, mitochondrial	-0.343458899882846	0.0292008522461461	0.275879098941516	NA	aag01100 ; aag	XP_001846433.1 cyt	XP_021701509.1cytochrome c oxidase subunit 4 isoform 1, mitochondrial
AAEL02382	NA	-0.343112908275202	0.258592979471266	0.691547675112889	NA	NA	NA	XP_021695638.1zinc finger protein 883
AAEL00181	sodium-coupled monocarboxylate transporter 1	-0.342411781973627	0.15725869059113	0.574474189691589	NA	NA	XP_019536043.1 PRE	XP_021702456.1sodium-coupled monocarboxylate transporter 1 isoform X1
AAEL01177	agglutinin-like protein ARB_02240	-0.342344183422048	0.279561682175346	0.713638889794457	NA	NA	XP_001853598.1 con	XP_021698775.1agglutinin-like protein ARB_02240
AAEL01083	mitochondrial ubiquitin ligase activator of nfkb 1-A	-0.342206441379193	0.0939348941584184	0.464823706598816	NA	NA	XP_001850539.1 con	XP_001661088.1mitochondrial ubiquitin ligase activator of nfkb 1-A
AAEL00235	protein unc-13 homolog B	-0.342109179429955	0.10675882544742	0.492031204248678	NA	NA	NA	XP_001661348.1protein unc-13 homolog C isoform X4
AAEL01179	putative leucine-rich repeat-containing protein DDB_G0290503	-0.341945363415864	0.0954706544518139	0.467087334124813	NA	NA	KXJ75525.1 hypothet	XP_021707239.1putative leucine-rich repeat-containing protein DDB_G0290503
AAEL00534	protein ABHD18	-0.34161246348908	0.112431074096703	0.503547757511443	NA	NA	KXJ69464.1 hypothet	XP_021710584.1protein ABHD18
AAEL02304	NA	-0.341539094836699	0.259024346325782	0.691547675112889	NA	NA	NA	XP_021709173.1transient receptor potential channel pyrexia-like
AAEL01955	NA	-0.341511826183097	0.204625143423745	0.637782699712521	NA	NA	NA	XP_021701149.1YEATS domain-containing protein 2
AAEL00850	alanine--tRNA ligase, mitochondrial	-0.341011369404223	0.197254851068272	0.630372589300064	970	aag00970	KXJ76855.1 hypothet	XP_001659327.1alanine--tRNA ligase, mitochondrial
AAEL01972	NA	-0.340731230107488	0.0062678086530017	0.129262654794788	NA	NA	NA	XP_021699588.1trichohyalin isoform X6
AAEL00651	eukaryotic translation initiation factor 4E-binding protein Mextli	-0.340660335973117	0.00613885000483537	0.128094552708606	NA	NA	XP_019533068.1 PRE	XP_021703995.1eukaryotic translation initiation factor 4E-binding protein Mextli isoform X1
AAEL02220	NA	-0.34047951811709	0.226097816463403	0.660073638038144	NA	NA	NA	NA
AAEL01146	NA	-0.340461844481753	0.227070102914986	0.66123388829864	NA	NA	KXJ72608.1 hypothet	XP_019553299.1 biogenesis of lysosome-related organelles complex 1 subunit 6
AAEL02551	NA	-0.340238177405764	0.00673722504342888	0.135067417973056	NA	NA	NA	XP_021696069.1 obscurin
AAEL00256	twitchin	-0.34020532456494	0.0129545122987735	0.183396034112791	NA	NA	XP_019556125.1 PRE	XP_001655590.2twitchin isoform X3
AAEL02705	NA	-0.340043621187498	0.137721332520349	0.54743486041134	NA	NA	NA	XP_021699522.1protein downstream neighbor of son homolog
AAEL01482	N-acetyltransferase 6	-0.339632704280521	0.225156324860706	0.658047884618818	NA	NA	NA	XP_001649676.1N-acetyltransferase 6 isoform X2
AAEL02001	NA	-0.339533569737743	0.0783882649277973	0.428723995967346	NA	NA	NA	XP_021703666.1gastrula zinc finger protein XlCGF26.1 isoform X2
AAEL00942	peptidyl-prolyl cis-trans isomerase, rhodopsin-specific isozyme	-0.339481495086677	0.056247103079542	0.373169316898302	NA	NA	NA	XP_001660049.1peptidyl-prolyl cis-trans isomerase, rhodopsin-specific isozyme isoform X1
AAEL00718	ELKS/Rab6-interacting/CAST family member 1	-0.33896694729921	0.0749115496463199	0.419814908553859	NA	NA	NA	XP_001652593.3ELKS/Rab6-interacting/CAST family member 1 isoform X3
AAEL02125	NA	-0.338956299611704	0.23722534848975	0.671758630247645	NA	NA	NA	XP_021705378.1uncharacterized protein LOC5565035
AAEL00472	locomotion-related protein Hikaru genki	-0.338863466374156	0.0152176428986194	0.198907702459398	NA	NA	KXJ72377.1 hypothet	XP_021702536.1locomotion-related protein Hikaru genki isoform X1
AAEL01966	NA	-0.338603996842251	0.292035766996909	0.722978826937197	NA	NA	NA	XP_021711338.1zinc finger protein 184
AAEL00813	ATP-binding cassette sub-family G member 1	-0.338561875473127	0.069507809771724	0.404817003493294	NA	NA	XP_019527804.1 PRE	XP_001658974.2ATP-binding cassette sub-family G member 4 isoform X3
AAEL00313	copper transport protein ATOX1	-0.338223264346408	0.112975930323505	0.503928507434265	NA	NA	XP_001865898.1 ant	XP_001656348.2copper transport protein ATOX1
AAEL01476	nose resistant to fluoxetine protein 6	-0.337586071783242	0.103082666376396	0.485796149609891	NA	NA	NA	XP_021697467.1nose resistant to fluoxetine protein 6
AAEL00468	centrosomal protein of 83 kDa	-0.337517301583769	0.0371045023745656	0.304789118266276	NA	NA	XP_019550261.1 PRE	XP_021707435.1CAP-Gly domain-containing linker protein 1 isoform X3
AAEL01868	NA	-0.337454524243747	0.48853338355044	0.848846834445739	NA	NA	NA	NA
AAEL01816	NA	-0.337361607533379	0.369534188262845	0.781817769662763	NA	NA	NA	XP_001659579.2zinc finger protein 350
AAEL00863	ATP-binding cassette sub-family G member 4	-0.337272394873114	0.0221475320788849	0.242070925206103	NA	NA	KXJ78719.1 hypothet	XP_001653364.2ATP-binding cassette sub-family G member 4
AAEL01352	uncharacterized LOC5578143	-0.337089967109296	0.0410914807431541	0.319254212557132	NA	NA	NA	CRK88250.1CLUMA_CG002031, isoform A
AAEL00376	uncharacterized LOC5578922	-0.336961637621525	0.00868803486197016	0.150559880979001	NA	NA	KXJ75765.1 hypothet	XP_001657208.2uncharacterized protein LOC5578922
AAEL00287	heterogeneous nuclear ribonucleoprotein R	-0.336884464896778	0.0106708247271162	0.166147088221529	NA	NA	NA	XP_021693280.1heterogeneous nuclear ribonucleoprotein R isoform X10
AAEL01346	ankyrin-2	-0.336810064000902	0.00731676583443856	0.139976879851359	NA	aag04624	NA	XP_021701964.1ankyrin-3 isoform X2
AAEL02627	NA	-0.336771939268514	0.156785254813093	0.574474189691589	NA	NA	NA	XP_021705603.1synaptic vesicular amine transporter isoform X2
AAEL02364	NA	-0.336500761946922	0.242124883892891	0.676994125742042	NA	NA	NA	XP_021695592.1zinc finger protein 436 isoform X2
AAEL02031	NA	-0.336406645965571	0.0326355488707924	0.28463170766102	NA	NA	NA	XP_021704030.1uncharacterized protein LOC110677244
AAEL02626	NA	-0.336328369661654	0.108658700531031	0.495725018486141	NA	NA	NA	NA
AAEL01226	cytochrome P450 4C1	-0.336279377366545	0.0605302881858901	0.384941803020695	NA	NA	KFB49529.1 AGAP00	XP_001655966.1cytochrome P450 4C1
AAEL00118	uncharacterized LOC5568837	-0.336015255542751	0.035691761493175	0.299394338432267	NA	NA	NA	XP_001658203.1uncharacterized protein LOC5568837 isoform X2
AAEL00296	NA	-0.335636365161135	0.0316085330959961	0.28423411888152	NA	NA	KXJ77164.1 hypothet	XP_001656231.1 endochitinase
AAEL01087	protein lethal(2)denticleless	-0.335188322521944	0.278419211444558	0.713122992430066	NA	NA	KXJ82473.1 hypothet	XP_001661121.1protein lethal(2)denticleless
AAEL01387	uncharacterized LOC5578833	-0.334782484523333	0.0109622916035568	0.168586924805399	NA	aag01100 ; aag	NA	ABF18416.1putative mitochondrial NADH-ubiquinone oxidoreductase
AAEL00424	flightin	-0.334755542452734	0.0209544924622218	0.23627229311125	NA	NA	KFB40354.1 AGAP00	XP_001648786.1flightin isoform X1
AAEL00017	chorion transcription factor Cf2	-0.334739935406558	0.364252003845251	0.77844728212936	NA	NA	KXJ71309.1 hypothet	XP_001658901.1chorion transcription factor Cf2

AAEL0031!	alcohol dehydrogenase [NADP(+)]	-0.334228708573481	0.0197504607544883	0.227464004834545	NA	aag01100 ; aag	XP_001656491.2 alc	XP_001656491.2alcohol dehydrogenase [NADP(+)]
AAEL0141	zinc finger protein 652-B	-0.333793160678031	0.19447334836315	0.625933456326584	NA	NA	NA	XP_001648101.2zinc finger protein 652-B
AAEL0201	NA	-0.333634161313013	0.0375829669846728	0.306594381376736	NA	NA	NA	XP_021698692.1protein I'm not dead yet
AAEL0148	multiple inositol polyphosphate phosphatase 1	-0.333533479019826	0.0434699391455746	0.329226650220228	NA	aag01100 ; aag	NA	XP_021697516.1multiple inositol polyphosphate phosphatase 1
AAEL0209	NA	-0.333250674296764	0.0381050388259921	0.308392759258381	NA	NA	NA	NA
AAEL0098	uncharacterized LOC5572535	-0.333177087159965	0.0106329449198883	0.166147088221529	NA	NA	NA	XP_021698688.1uncharacterized protein LOC5572535
AAEL0216	NA	-0.33315044651284	0.336775484558032	0.758445425575872	NA	NA	NA	XP_021694867.1centrosomal protein of 162 kDa isoform X5
AAEL0025	serine proteinase stubble	-0.333024339186723	0.10520663866251	0.489439579864721	NA	NA	KXJ71089.1 hypothe	XP_001655709.1serine proteinase stubble
AAEL0144	uncharacterized LOC5564435	-0.332972203908006	0.458524946673686	0.833308308986236	NA	NA	NA	XP_001648775.2uncharacterized protein LOC5564435
AAEL0025	tryptase	-0.332818977019544	0.0100032526414244	0.161638111035661	NA	NA	XP_019531788.1 PRE	XP_019531788.1 tryptase-like
AAEL0105	aldose 1-epimerase	-0.33271169028306	0.0417970019127372	0.322935358187244	00010 ; 00052	aag01100 ; aag	KXJ77062.1 hypothe	XP_001654671.2aldose 1-epimerase
AAEL0044	probable ATP-dependent RNA helicase DDX28	-0.332710944258156	0.0641137454509227	0.395160539611112	NA	NA	KXJ70985.1 hypothe	XP_001649227.1probable ATP-dependent RNA helicase DDX28
AAEL0085	protein cueball	-0.332690531219496	0.00774810691687057	0.143746128727909	NA	NA	XP_019554788.1 PRE	XP_001653297.1protein cueball isoform X3
AAEL0197	NA	-0.331446432395582	0.0461001403595705	0.341281972315984	NA	NA	NA	XP_021711681.1uncharacterized protein LOC5569133 isoform X3
AAEL0134	TM2 domain-containing protein almondex	-0.330895995858741	0.213118027656914	0.646373717545434	NA	NA	NA	XP_001663631.1TM2 domain-containing protein almondex
AAEL0121	trifunctional nucleotide phosphoesterase protein YfkN	-0.330730619255572	0.0119706448500635	0.176811996645721	NA	aag01100 ; aag	NA	XP_021696043.1uncharacterized protein LOC5575910 isoform X1
AAEL0110	paxillin	-0.330497017100571	0.0479254353497696	0.348612005978262	NA	NA	KXJ70676.1 hypothe	XP_019552173.1 transforming growth factor beta-1-induced transcript 1 protein
AAEL0223	NA	-0.330390236370259	0.33404728636149	0.756373801451385	NA	NA	NA	NA
AAEL0290	NA	-0.330330535372739	0.765335801227895	0.939016005719879	NA	NA	NA	XP_001861740.1cecropin A
AAEL0222	NA	-0.330095924569412	0.148130125930283	0.562679383080301	NA	NA	NA	XP_021701851.1progestin and adipoQ receptor family member 3 isoform X1
AAEL0107	transmembrane protease serine 9	-0.329873968841323	0.0374347841812714	0.305927960955648	NA	NA	XP_019538268.1 PRE	XP_001654875.3transmembrane protease serine 9
AAEL0236	NA	-0.329869881488457	0.0525885799165915	0.362633125106993	NA	NA	NA	XP_021710043.1disco-interacting protein 2 isoform X8
AAEL0275	NA	-0.329716746523154	0.0420848913632536	0.323800309636003	NA	aag04320	NA	XP_021706628.1DNA-binding protein K10
AAEL0013	bifunctional endo-1,4-beta-xylanase XylA	-0.329683804333148	0.0174331324713232	0.214412616901924	NA	NA	NA	XP_001653158.1uncharacterized protein LOC5570331
AAEL0136	ribosome-releasing factor 2, mitochondrial	-0.329577201679655	0.0262191561788137	0.264259227992819	NA	NA	NA	XP_021701538.1ribosome-releasing factor 2, mitochondrial
AAEL0198	NA	-0.32935587223932	0.0114608310013493	0.172833431546296	NA	NA	NA	XP_021711062.1trichohyalin isoform X4
AAEL0003	uncharacterized LOC5575127	-0.329065002059775	0.262891764559686	0.696819172339274	NA	NA	KXJ75620.1 hypothe	XP_021697892.1uncharacterized protein LOC5575127 isoform X1
AAEL0097	uncharacterized LOC5572349	-0.328372407720253	0.0842993331296519	0.44100196899321	310	aag01100 ; aag	NA	ETN62222.1epsilon-trimethyllysine 2-oxoglutarate dioxygenase
AAEL0048	ankyrin repeat-containing protein kinase A	-0.32790420136667	0.0916835463333893	0.459954188094628	NA	NA	XP_001863747.1 con	XP_021697770.1ankyrin repeat-containing protein kinase A
AAEL0094	NA	-0.327588092442715	0.163745855296404	0.581771953836875	NA	NA	NA	XP_001660065.2proline-rich protein PRCC
AAEL0148	uncharacterized LOC5565331	-0.327489131124638	0.187921952264404	0.614956545070072	NA	NA	NA	KFB35878.1AGAP001594-like protein
AAEL0173	farnesol dehydrogenase-like	-0.327378331887296	0.0864125722317124	0.447730005448321	NA	aag00981 ; aag	NA	XP_011492953.2farnesol dehydrogenase-like
AAEL0134	transmembrane protein 39A-B	-0.327304345434609	0.10783275216906	0.493915871308953	NA	NA	NA	XP_001663666.2transmembrane protein 39A-B
AAEL0063	upstream stimulatory factor 1	-0.32691081933753	0.223267732330087	0.65560825511079	NA	NA	XP_019543518.1 PRE	XP_001651889.2upstream stimulatory factor 1 isoform X1
AAEL0155	rhythmically expressed gene 2 protein	-0.326793402447301	0.145546430271966	0.558950568057442	NA	NA	NA	XP_001647614.2rhythmically expressed gene 2 protein
AAEL0245	NA	-0.326277943326042	0.0190352065593318	0.225171411092632	NA	NA	NA	XP_021697964.1uncharacterized protein LOC5573987 isoform X5
AAEL0271	NA	-0.325500632766584	0.209073360422917	0.639832374753494	NA	NA	NA	NA
AAEL0103	nucleoporin GLE1	-0.325495381381623	0.204902419233046	0.637782699712521	NA	NA	NA	XP_001654472.1nucleoporin GLE1
AAEL0254	NA	-0.325086947489208	0.11101746522622	0.502395086491705	NA	NA	NA	XP_021706442.1uncharacterized protein LOC5565785
AAEL0100	ATP-binding cassette sub-family D member 2	-0.324999141133047	0.0116229211136465	0.173701685309403	NA	aag04146 ; aag	NA	XP_001654181.2ATP-binding cassette sub-family D member 2
AAEL0010	uncharacterized LOC5579889	-0.324164346073666	0.0457277563593015	0.340242012934121	NA	NA	KXJ73902.1 hypothe	XP_001657868.2uncharacterized protein LOC5579889
AAEL0022	gastrula zinc finger protein XlCGF7.1,myoneurin	-0.323896820193276	0.439390695897846	0.822472169172494	NA	NA	KXJ68065.1 hypothe	XP_019531435.1 zinc finger protein 84-like
AAEL0129	CCR4-NOT transcription complex subunit 6-like	-0.323846121727916	0.0254482468217181	0.260483612072803	NA	aag03018	NA	XP_021713020.1CCR4-NOT transcription complex subunit 6-like
AAEL0201	NA	-0.323647155065253	0.0777493044871216	0.427133790979399	NA	NA	NA	XP_021696498.1glycogen-binding subunit 76A isoform X4
AAEL0187	NA	-0.323596669111792	0.0276936581755348	0.271096286901832	NA	NA	NA	XP_021706681.1SLIT-ROBO Rho GTPase-activating protein 1 isoform X2
AAEL0008	protein germ cell-less	-0.323510920050126	0.0152503869376103	0.198907702459398	NA	NA	KXJ72310.1 hypothe	XP_001651601.1protein germ cell-less
AAEL0044	uncharacterized LOC5564929	-0.323445834058553	0.217053437946766	0.649748124914163	NA	NA	KFB39996.1 hypothe	XP_001649268.1uncharacterized protein LOC5564929
AAEL0235	NA	-0.323331985615206	0.00697805072925916	0.137411930736898	NA	NA	NA	XP_021698384.1uncharacterized protein LOC5568876
AAEL0121	troponin C	-0.323227286489537	0.00756109672796198	0.142284687302058	NA	NA	XP_001846408.1 tro	XP_001662251.1troponin C isoform X2
AAEL0202	NA	-0.323108221917375	0.0513998605087107	0.359690131564899	NA	NA	NA	XP_021694472.1uncharacterized protein LOC110674718 isoform X1
AAEL0142	uncharacterized LOC5563757	-0.32305713407452	0.0676285166504381	0.40094231299198	NA	NA	NA	XP_021709436.1uncharacterized protein LOC5563757
AAEL0057	serine protease snake	-0.323026705969466	0.00976443758101401	0.160737664795154	NA	NA	KXJ77889.1 hypothe	XP_001651417.1serine protease snake isoform X1
AAEL0083	uncharacterized LOC5570460	-0.322666899410071	0.107381737253576	0.492585616254939	NA	NA	KXJ81357.1 hypothe	XP_001653199.2uncharacterized protein LOC5570460
AAEL0131	Na(+)/H(+) exchange regulatory cofactor NHE-RF1	-0.322001176772909	0.00933888719363235	0.158773875613038	NA	NA	NA	XP_001663319.1Na(+)/H(+) exchange regulatory cofactor NHE-RF1
AAEL0054	macrophage erythroblast attacher	-0.321942418537685	0.0190332149471032	0.225171411092632	NA	NA	XP_001847899.1 con	XP_001650864.1macrophage erythroblast attacher
AAEL0203	NA	-0.321926597218539	0.00788860894736179	0.144528409372715	NA	NA	NA	XP_021693355.1probable basic-leucine zipper transcription factor L isoform X2
AAEL0195	NA	-0.321651231016434	0.0574533615814612	0.375471897505248	NA	NA	NA	XP_021708408.1A disintegrin and metalloproteinase with thrombospondin motifs 9 isoform X2
AAEL0238	NA	-0.32162055540427	0.117160858426088	0.514858748441671	4660	NA	NA	XP_021708390.1trithorax group protein osa
AAEL0241	NA	-0.321461460705746	0.0306335050731938	0.281224109837052	NA	NA	NA	XP_021708100.1huntingtin-interacting protein 1 isoform X3
AAEL0194	NA	-0.320335456629995	0.508062386772792	0.859730781244726	NA	NA	NA	XP_021704645.1LIM/homeobox protein Lhx5 isoform X2
AAEL0051	cytochrome b-c1 complex subunit 8	-0.320092277767584	0.0322200058833228	0.284442054201394	NA	aag01100 ; aag	ETN65382.1 low mol	XP_011493120.1cytochrome b-c1 complex subunit 8
AAEL0089	protein bowel	-0.320061022481789	0.207312704417348	0.639832374753494	NA	NA	KFB45138.1 hypothe	XP_021703124.1protein bowel
AAEL0047	uncharacterized LOC5565327	-0.319606312606934	0.111340333936277	0.502725099549375	NA	NA	KXJ83883.1 hypothe	XP_001649673.2uncharacterized protein LOC5565327 isoform X1
AAEL0195	NA	-0.319137770480797	0.104899500368041	0.489113833380617	NA	NA	NA	XP_021708880.1phospholipid-transporting ATPase ID isoform X8
AAEL0100	microfibrillar-associated protein 1	-0.318981385519892	0.0798450846164956	0.431435389689367	NA	NA	NA	XP_001660598.1microfibrillar-associated protein 1
AAEL0037	ER membrane protein complex subunit 1	-0.318976403386955	0.00782698377785128	0.144528409372715	NA	NA	XP_019561421.1 PRE	XP_001664165.2ER membrane protein complex subunit 1 isoform X2
AAEL0053	uncharacterized LOC5566351	-0.318746323578628	0.0534461088801305	0.365578024386907	NA	NA	KFB51772.1 AGAP00	KFB51772.1AGAP007547-like protein
AAEL0034	calpain-C	-0.318676766642166	0.230178709922484	0.663064401691905	NA	NA	ETN65261.1 calpain-	XP_019563070.1 calpain-C-like
AAEL0071	uncharacterized LOC5568756	-0.318631887403718	0.0574191845935181	0.375471897505248	NA	NA	KXJ81827.1 hypothe	XP_019562647.1 polyserase-2-like
AAEL0271	NA	-0.318611542134763	0.0353734081001862	0.297809790794065	NA	NA	NA	XP_001663301.2probable serine hydrolase isoform X1
AAEL0119	probable maleylacetoacetate isomerase 2	-0.318162071982323	0.0582214867525564	0.37790091015122	NA	aag01100 ; aag	XP_003436289.1 AG	XP_001662086.2probable maleylacetoacetate isomerase 2 isoform X1
AAEL0200	NA	-0.31772712023206	0.205640242226022	0.638023927878659	NA	NA	NA	XP_021703664.1zinc finger protein 689-like
AAEL0054	annexin B10	-0.317595727046255	0.0385071788852491	0.309740437152152	NA	NA	XP_019528870.1 PRE	XP_001650855.1annexin B10 isoform X2
AAEL0197	NA	-0.317531553719012	0.252969561695194	0.685139021532167	NA	NA	NA	XP_021711136.1POU domain, class 6, transcription factor 2 isoform X3
AAEL0004	SRR1-like protein	-0.317492380733176	0.0159449498164413	0.203220814696528	NA	NA	KXJ69268.1 hypothe	XP_001656422.2SRR1-like protein
AAEL0216	NA	-0.317490783201863	0.0571293658635268	0.375057077651547	NA	NA	NA	XP_021694176.1uncharacterized protein LOC5564210 isoform X2
AAEL0037	homeobox protein Nkx-2.1	-0.317375166482086	0.239748683764923	0.674660318489854	NA	NA	CRL03005.1 CLUMA_	XP_001664172.2homeobox protein Nkx-2.1
AAEL0098	WD repeat and FYVE domain-containing protein 3	-0.317222163842435	0.0327564636397544	0.28463170766102	NA	NA	NA	XP_021705139.1WD repeat and FYVE domain-containing protein 3 isoform X2
AAEL0225	NA	-0.317133049155956	0.0366464727768931	0.302711707803385	NA	NA	NA	XP_021694123.1thyroid receptor-interacting protein 11 isoform X1
AAEL0014	protein phosphatase 1 regulatory subunit pprA	-0.317113854350316	0.181451149193672	0.607780282383509	NA	NA	KXJ74863.1 hypothe	XP_001659165.1protein phosphatase 1 regulatory subunit pprA
AAEL0128	uncharacterized LOC5576908	-0.317072815143524	0.0258937273644172	0.262416386792254	NA	NA	NA	XP_019525032.1 serine/threonine-protein phosphatase 4 regulatory subunit 2-like
AAEL0021	neural/ectodermal development factor IMP-L2	-0.317027002169123	0.0411123931623779	0.319254212557132	NA	NA	KXJ81653.1 hypothe	XP_021704844.1neural/ectodermal development factor IMP-L2 isoform X1

AAEL01019	NA	-0.316658031525265	0.679671026012179	0.914629465876247	NA	NA	NA	XP_001660673.2trypsin 5G1-like
AAEL01975	NA	-0.316443528418512	0.0896155686483745	0.45743198048147	00564 ; 00591 ; 00565 ;	aag01100 ; aag	NA	XP_021708996.1uncharacterized protein LOC5571127 isoform X3
AAEL01145	perlucin	-0.316408735628499	0.0793022978811354	0.430779070308269	NA	NA	XP_019528442.1 PRE	ABF18198.1salivary C-type lectin
AAEL00374	4-hydroxybutyrate coenzyme A transferase	-0.316089810509753	0.00727053885496829	0.139382288632121	NA	NA	XP_001845554.1 4-H	XP_001845554.14-Hydroxybutyrate CoA-transferase
AAEL02363	NA	-0.315585897599216	0.489100321857538	0.849220154814801	NA	NA	NA	XP_021695107.1protein RCC2 homolog
AAEL02904	NA	-0.315506100301881	0.025100210290208	0.258647407716119	NA	NA	NA	ABF18188.1predicted salivary secreted peptide
AAEL00587	flap endonuclease 1	-0.315448652006732	0.0630476024842087	0.39272109689124	NA	aag03030 ; aag	ETN66057.1 flap end	XP_021711510.1flap endonuclease 1
AAEL00196	ejaculatory bulb-specific protein 3	-0.31527423903794	0.0155703251167462	0.201233331073453	NA	NA	KFB39459.1 AGAP00	XP_001660749.1ejaculatory bulb-specific protein 3
AAEL00413	TBC1 domain family member 10A	-0.315254187396229	0.0392737437415758	0.313353264954028	NA	NA	KXJ73657.1 hypothet	XP_001648503.2TBC1 domain family member 10A
AAEL01969	NA	-0.315210590615899	0.0502398130336336	0.356468207181388	NA	NA	NA	XP_021708108.1proline-rich extensin-like protein EPR1 isoform X2
AAEL00847	Golgi-associated plant pathogenesis-related protein 1	-0.315201835019279	0.0544439429359877	0.367469712613638	NA	NA	KXJ82507.1 hypothet	XP_001659272.1Golgi-associated plant pathogenesis-related protein 1
AAEL00313	uncharacterized LOC5577237	-0.314930011102623	0.0149371253020275	0.19720434294011	NA	NA	KXJ70659.1 hypothet	XP_001656343.1uncharacterized protein LOC5577237
AAEL00085	tyrosine kinase receptor Cad96Ca	-0.314923093791462	0.188923443484664	0.616603527036593	NA	NA	XP_019537938.1 PRE	XP_021705357.1tyrosine kinase receptor Cad96Ca
AAEL00495	oral cancer-overexpressed protein 1 homolog	-0.314825309887749	0.388498120795111	0.793025667159852	NA	NA	XP_001866634.1 con	XP_001650093.2oral cancer-overexpressed protein 1 homolog
AAEL01972	NA	-0.314502776865018	0.0206173378196056	0.233690584681458	NA	NA	NA	XP_021698753.1ion transport peptide-like isoform X1
AAEL00699	probable cytochrome P450 28a5	-0.314327556587563	0.153362247686967	0.568818435996349	NA	NA	KXJ73463.1 hypothet	XP_001652486.1probable cytochrome P450 28a5
AAEL01149	chitinase domain-containing protein 1	-0.314108215571795	0.150897109391931	0.566025396924239	NA	NA	KXJ80469.1 hypothet	XP_021701718.1chitinase domain-containing protein 1
AAEL00746	titin	-0.3140384603424	0.0106415546401586	0.166147088221529	NA	NA	XP_019525720.1 PRE	XP_021698681.1titin isoform X1
AAEL00413	short-chain specific acyl-CoA dehydrogenase, mitochondrial	-0.313855963117101	0.31904504900709	0.745684464362801	NA	NA	KXJ73655.1 hypothet	XP_001648505.2short-chain specific acyl-CoA dehydrogenase, mitochondrial
AAEL01951	NA	-0.313796311798489	0.24312919202023	0.677870931254677	NA	NA	NA	XP_019932032.1 titin-like
AAEL02004	NA	-0.313772921000184	0.525696775889557	0.865680248818465	NA	NA	NA	NA
AAEL01085	tyramine beta-hydroxylase	-0.313721884478215	0.407632655151319	0.803607536382376	NA	aag01100 ; aag	KFB41858.1 AGAP01	XP_021707312.1tyramine beta-hydroxylase
AAEL00786	striatin-interacting protein 1 homolog	-0.313620362642508	0.0306463224612641	0.281224109837052	NA	NA	XP_019544861.1 PRE	XP_021703868.1striatin-interacting protein 1 homolog isoform X3
AAEL00904	bicaudal D-related protein homolog	-0.313559956808166	0.0559457120965943	0.371974308318541	NA	NA	XP_019545187.1 PRE	XP_001653670.1bicaudal D-related protein homolog isoform X1
AAEL01168	CXXC-type zinc finger protein 1	-0.31347885397913	0.103609270022126	0.486291766544946	NA	NA	ETN60493.1 cpg bind	XP_021710746.1CXXC-type zinc finger protein 1
AAEL01493	sterile alpha and TIR motif-containing protein 1	-0.313454960337577	0.0130449428602998	0.183852669789472	NA	NA	NA	XP_021698316.1sterile alpha and TIR motif-containing protein 1 isoform X5
AAEL00469	protein unc-13 homolog 4B	-0.313279146247036	0.0394617601157755	0.313353264954028	NA	NA	XP_019541322.1 PRE	XP_021705668.1protein unc-13 homolog 4B isoform X1
AAEL00977	protein FAM32A	-0.313059197241078	0.12092970857094	0.522930064976407	NA	NA	NA	XP_001654043.1protein FAM32A
AAEL00381	mitochondrial GTPase 1-like	-0.312943294346121	0.380372117719126	0.786294135283846	NA	NA	XP_019525780.1 PRE	XP_021696520.1mitochondrial GTPase 1-like isoform X1
AAEL00802	cytoplasmic FMR1-interacting protein	-0.312758486683928	0.0935553483737437	0.464823706598816	NA	aag03013	ETN65677.1 specifica	XP_021695776.1cytoplasmic FMR1-interacting protein
AAEL02299	NA	-0.312586724529386	0.144913696326489	0.558181596314924	NA	NA	NA	XP_001660443.2exocyst complex component 7
AAEL00854	zinc finger protein 675	-0.312526844964239	0.371729229620509	0.781817769662763	NA	NA	XP_019533163.1 PRE	XP_021693851.1zinc finger protein 675
AAEL02538	NA	-0.312471837851196	0.0965490086467669	0.470574140660778	NA	NA	NA	XP_021703333.1uncharacterized protein LOC5573899
AAEL01134	NA	-0.312082343455095	0.153128491176915	0.568713993417967	NA	NA	KXJ74320.1 hypothet	XP_001661613.2dnaJ homolog subfamily C member 22
AAEL00955	serine protease 53	-0.312055310209182	0.384031763790244	0.790063144962496	NA	NA	NA	XP_021708575.1serine protease 53
AAEL00334	alpha-tocopherol transfer protein-like	-0.311539945366551	0.0195996671392731	0.227335564593981	NA	NA	KXJ72862.1 hypothet	XP_001656682.1retinaldehyde-binding protein 1 isoform X1
AAEL01267	uncharacterized LOC5576655	-0.311513015834762	0.365840872508423	0.779835687469172	901	NA	XP_001864962.1 Juv	XP_001864962.1Juvenile hormone-inducible protein
AAEL00073	probable peroxisomal acyl-coenzyme A oxidase 1	-0.311330933983549	0.201741227072612	0.634701781910801	00071 ; 00592	NA	KXJ82980.1 hypothet	XP_001650659.2probable peroxisomal acyl-coenzyme A oxidase 1
AAEL01733	mitogen-activated protein kinase kinase kinase kinase 3	-0.311185055894864	0.0533857982764843	0.365578024386907	04151 ; 05165 ; 04714 ;	NA	NA	XP_021710912.1mitogen-activated protein kinase kinase kinase kinase 3 isoform X3
AAEL01057	actin cytoskeleton-regulatory complex protein pan1	-0.311077657413666	0.476503443551498	0.848867278451347	NA	NA	KXJ77063.1 hypothet	XP_021693953.1actin cytoskeleton-regulatory complex protein pan1
AAEL02155	NA	-0.310794686082375	0.0298065604331207	0.278836039075975	NA	NA	NA	XP_021713275.1synaptic vesicle glycoprotein 2A
AAEL02715	NA	-0.310785438011971	0.012736194197323	0.182308324322341	901	NA	NA	XP_021705648.1uncharacterized protein LOC5572971
AAEL00736	succinate dehydrogenase assembly factor 3, mitochondrial	-0.310752464294211	0.168581401953646	0.58872336272389	NA	NA	KXJ79760.1 hypothet	XP_001652715.2succinate dehydrogenase assembly factor 3, mitochondrial
AAEL00810	glutamate--cysteine ligase	-0.310611433667302	0.0143580315487638	0.192318204238318	00270 ; 00480	aag01100 ; aag	KXJ83847.1 hypothet	XP_001653117.1glutamate--cysteine ligase
AAEL01282	endoplasmin	-0.310372681273979	0.00952165997684491	0.158773875613038	NA	aag04141	ETN62729.1 endopla	XP_019524856.1 endoplasmin-like
AAEL01957	NA	-0.309964348713799	0.0310633053466266	0.282455074900848	NA	NA	NA	XP_021698057.1filamin-B isoform X1
AAEL00882	uncharacterized LOC5571135	-0.309736688186749	0.0765295400303224	0.422703978006619	NA	aag04013	KXJ83088.1 hypothet	XP_001659577.1uncharacterized protein LOC5571135
AAEL01337	uncharacterized LOC5577784	-0.309186662102507	0.191659038196747	0.621678529880687	NA	NA	NA	XP_001663557.2uncharacterized protein LOC5577784
AAEL00046	alpha-ketoglutarate-dependent dioxygenase alkB homolog 6	-0.309124630064787	0.322464886157792	0.746650319748046	NA	NA	KXJ71577.1 hypothet	XP_001656428.1alpha-ketoglutarate-dependent dioxygenase alkB homolog 6
AAEL01203	transcriptional activator cubitus interruptus	-0.308722560178327	0.244916792120696	0.679727114240672	NA	aag04341	XP_019527861.1 PRE	XP_021713117.1transcriptional activator cubitus interruptus isoform X1
AAEL02561	NA	-0.308658195367288	0.0291019297271803	0.275227088745645	NA	NA	NA	XP_021692979.1centrosomal and chromosomal factor-like
AAEL02208	NA	-0.30863461361634	0.253227729979386	0.685145258966747	NA	NA	NA	NA
AAEL01489	probable cytochrome P450 6a14	-0.308551704146526	0.0239901600499227	0.252872225405943	NA	NA	NA	XP_001649932.1probable cytochrome P450 6a14
AAEL00670	angiopoietin-related protein 6	-0.308501136096498	0.0248062991849472	0.258221227488557	NA	NA	XP_019539651.1 PRE	XP_001657951.2angiopoietin-related protein 6 isoform X1
AAEL00872	HIG1 domain family member 1A, mitochondrial	-0.308043096282388	0.0197146034064331	0.227335564593981	NA	NA	XP_001867651.1 HIG	XP_001653423.1HIG1 domain family member 1A, mitochondrial isoform X1
AAEL02327	NA	-0.307997353587375	0.122950732612568	0.525360408607322	NA	NA	NA	NA
AAEL02739	NA	-0.307775504993616	0.0282871567641859	0.272581031963194	NA	NA	NA	XP_021709774.1kinesin-like protein unc-104 isoform X2
AAEL00435	sex-regulated protein janus-A	-0.307768068105163	0.0654764723227425	0.397837025777134	NA	NA	KXJ68510.1 hypothet	XP_021707753.1sex-regulated protein janus-A isoform X1
AAEL02193	NA	-0.307538368546187	0.0206738830877768	0.233998858762266	NA	NA	NA	XP_021697924.1uncharacterized protein LOC5578604 isoform X2
AAEL00634	uncharacterized LOC5567882	-0.307484772678585	0.283300081893591	0.715753918648043	NA	NA	NA	XP_021710671.1uncharacterized protein LOC5567882 isoform X1
AAEL00331	membrane-bound alkaline phosphatase	-0.30741791564507	0.208812341080613	0.639832374753494	00790 ; 00730	NA	KXJ70902.1 hypothet	XP_021704689.1membrane-bound alkaline phosphatase
AAEL01231	MICOS complex subunit MIC13 homolog QIL1	-0.30736510654711	0.0100357855105383	0.161638111035661	NA	NA	XP_001865753.1 con	XP_001662410.1MICOS complex subunit MIC13 homolog QIL1
AAEL00275	synaptotagmin-4	-0.307075206346121	0.080634177327235	0.433079891352508	NA	NA	XP_019532843.1 PRE	XP_021705688.1synaptotagmin-4 isoform X1
AAEL00026	zinc finger protein jing homolog	-0.307028387464433	0.345756486475774	0.76500389241406	NA	NA	XP_019552543.1 PRE	XP_001654760.2zinc finger protein jing homolog isoform X2
AAEL00197	uroporphyrinogen-III synthase	-0.306673149658173	0.223133804833712	0.655580227356264	860	aag01100 ; aag	KXJ77425.1 hypothet	XP_001660743.2uroporphyrinogen-III synthase
AAEL02398	NA	-0.306654871610153	0.152697573379089	0.568113296551786	62	NA	NA	XP_021708782.1elongation of very long chain fatty acids protein 7
AAEL01076	cytosolic 10-formyltetrahydrofolate dehydrogenase-like	-0.306594652746953	0.00969708637620787	0.160202134351642	00670 ; 00970 ; 00670	aag00670	KFB35479.1 AGAP00	XP_021701277.1cytosolic 10-formyltetrahydrofolate dehydrogenase-like
AAEL02465	NA	-0.306563810780231	0.456694246904535	0.832672966121563	NA	NA	NA	XP_021700464.1endoplasmic reticulum membrane-associated RNA degradation protein-like
AAEL01348	uncharacterized LOC5578050	-0.306461079223083	0.0371881286961749	0.304789118266276	NA	NA	NA	XP_001663672.1uncharacterized protein LOC5578050
AAEL00975	uncharacterized LOC5572368	-0.306414198178851	0.0907457222012133	0.458582874647784	NA	NA	NA	XP_001660327.1uncharacterized protein LOC5572368
AAEL01046	torso-like protein	-0.306375904109829	0.223057996464743	0.655580227356264	NA	aag04013	NA	XP_001660859.2torso-like protein
AAEL00092	polyribonucleotide nucleotidyltransferase 1, mitochondrial	-0.306202598474268	0.170220706418416	0.590883473644663	NA	aag03018	KFB35183.1 AGAP01	XP_001651608.1polyribonucleotide nucleotidyltransferase 1, mitochondrial
AAEL00910	autophagy-related protein 9A	-0.306077535581125	0.0088844359262371	0.152533826983712	NA	aag04140 ; aag	KXJ79488.1 hypothet	XP_021711494.1autophagy-related protein 9A isoform X1
AAEL01950	NA	-0.305786324975027	0.0681075596695191	0.401232883533236	NA	NA	NA	XP_021713154.1uncharacterized protein LOC5568719
AAEL00365	laminin subunit beta-1	-0.305784903113672	0.0131795002988238	0.184874636813685	NA	aag04512	KXJ70424.1 hypothet	XP_021708382.1laminin subunit beta-1
AAEL02599	NA	-0.305740145873268	0.0230418776765091	0.246091471282416	NA	aag03010	NA	XP_001648567.140S ribosomal protein S17
AAEL00942	nuclear pore complex protein Nup98-Nup96	-0.305719151803489	0.280787208866236	0.71494297066605	NA	aag03013	NA	XP_021700428.1nuclear pore complex protein Nup98-Nup96
AAEL00319	xaa-Pro aminopeptidase ApepP	-0.305691896010068	0.061489599729509	0.38668618011863	NA	NA	KXJ76909.1 hypothet	XP_001656516.2xaao aminopeptidase ApepP
AAEL00682	probable 4-coumarate--CoA ligase 3	-0.305603944317873	0.252310265538	0.684556086619159	NA	NA	KXJ68906.1 hypothet	XP_001658054.1probable 4-coumarate--CoA ligase 3
AAEL00473	trissin receptor	-0.305385894760471	0.356789615674833	0.773422389502901	NA	NA	KFB39152.1 hypothe	XP_021702489.1trissin receptor isoform X2
AAEL01815	NA	-0.305245533293485	0.037191624637666	0.304789118266276	NA	aag04745	NA	XP_021700001.1inactivation-no-after-potential D protein isoform X3
AAEL01434	GTP-binding protein RAD	-0.305191144800388	0.346938659889745	0.765398366276275	NA	NA	NA	XP_021699967.1GTP-binding protein RAD isoform X1

AAEL00503	uncharacterized LOC5565834	-0.305137423546405	0.0135691043851478	0.187825791015408	NA	NA	KXJ76335.1 hypothet	XP_021703643.1uncharacterized protein LOC5565834 isoform X1
AAEL00724	uncharacterized LOC5568938	-0.305084663723299	0.224493045807867	0.656638591075649	NA	NA	KXJ83143.1 hypothet	XP_001658260.2uncharacterized protein LOC5568938
AAEL00721	fatty acyl-CoA reductase wat	-0.305036766524329	0.0501556750876859	0.356468207181388	73	NA	XP_019535045.1 PRE	XP_001658219.2fatty acyl-CoA reductase wat isoform X2
AAEL00138	sphingomyelin synthase-related 1	-0.304805245106952	0.15236084158213	0.568113296551786	NA	NA	KXJ69673.1 hypothet	XP_021699512.1sphingomyelin synthase-related 1
AAEL00728	acyl-CoA synthetase short-chain family member 3, mitochondrial	-0.304770735361516	0.0379150235489433	0.307682856236235	00010 ; 00640 ; 00620 ;	aag01100 ; aag	KFB51925.1 hypothe	XP_021706231.1acyl-CoA synthetase short-chain family member 3, mitochondrial isoform X2
AAEL00664	uncharacterized LOC5568220	-0.304722770427085	0.356433292413355	0.772867683747772	NA	NA	KXJ70772.1 hypothet	XP_001652152.2uncharacterized protein LOC5568220
AAEL00579	protein abnormal spindle	-0.30449811235038	0.356416143697957	0.772867683747772	NA	NA	KXJ77883.1 hypothet	XP_021712945.1protein abnormal spindle
AAEL02702	NA	-0.304328329252637	0.246995228196647	0.680385311739523	NA	NA	NA	XP_021706017.1ribosome biogenesis protein NOP53-like
AAEL02287	NA	-0.304247000186438	0.325604884941203	0.748896673349414	NA	NA	NA	XP_021699078.1uncharacterized protein LOC5572637 isoform X1
AAEL02905	NA	-0.304243425064927	0.146615463818916	0.561212769576399	NA	NA	NA	EAT38081.1AAEL009992
AAEL00805	uncharacterized LOC5569954	-0.304191199367502	0.186981490095999	0.614196367514984	NA	NA	KXJ71449.1 hypothet	XP_001658855.2uncharacterized protein LOC5569954
AAEL02027	NA	-0.304167384579479	0.136534659141384	0.545589928345625	NA	NA	NA	XP_021705013.1uncharacterized protein LOC5572960
AAEL02179	NA	-0.304143590967103	0.010261701656898	0.163088391445208	NA	NA	NA	XP_021696764.1uncharacterized protein LOC110675609
AAEL01040	alkyldihydroxyacetonephosphate synthase	-0.304109799456759	0.0745685485706058	0.419169079991884	565	aag01100 ; aag	NA	XP_021693470.1alkyldihydroxyacetonephosphate synthase
AAEL01161	collectin-10	-0.30403682130647	0.151198140405056	0.566025396924239	NA	NA	XP_001688638.1 AG	XP_021698856.1collectin-10 isoform X2
AAEL02260	NA	-0.303785444891328	0.0482818592898024	0.350110062399339	NA	NA	NA	XP_001661186.2protein G12
AAEL00778	integrator complex subunit 12	-0.303603873447063	0.288812162352359	0.721217831038796	NA	NA	KXJ78610.1 hypothet	XP_021694321.1integrator complex subunit 12
AAEL00491	probable ATP-dependent RNA helicase DDX20	-0.303461285560947	0.0833401630293975	0.438013925780757	NA	aag03013	XP_019525224.1 PRE	XP_001650004.2probable ATP-dependent RNA helicase DDX20 isoform X1
AAEL00024	solute carrier family 25 member 35	-0.303327967552322	0.187476285215601	0.614323480626963	NA	NA	KXJ77550.1 hypothet	XP_001660132.1solute carrier family 25 member 35 isoform X1
AAEL01176	integumentary mucin C.1	-0.302812056075052	0.361546206820052	0.77623616312602	NA	NA	NA	XP_001661889.2integumentary mucin C.1 isoform X2
AAEL02290	NA	-0.302675220711457	0.0478316387796799	0.348612005978262	NA	NA	NA	XP_021693719.1huntingtin-interacting protein K
AAEL02318	NA	-0.302195025688652	0.0440543314958159	0.332013069962734	NA	aag04933 ; aag	NA	XP_021701992.1collagen alpha-1(IV) chain
AAEL00285	NA	-0.302183135764303	0.0177159851755932	0.21563822167435	NA	NA	NA	XP_021693313.1LOW QUALITY PROTEIN: uncharacterized protein LOC5576344
AAEL00504	RNA-binding protein squid	-0.301832586024831	0.270994251346944	0.706427507335574	NA	NA	KXJ78504.1 hypothet	XP_001650293.2RNA-binding protein squid
AAEL00580	aminopeptidase N	-0.301686998442735	0.103209244999711	0.485796149609891	480	NA	XP_019561879.1 PRE	XP_001651477.2aminopeptidase N
AAEL00336	dnaJ homolog subfamily B member 14	-0.301649969809639	0.0288865485648572	0.274601260220884	NA	aag04141	KXJ70557.1 hypothet	XP_001656767.1dnaJ homolog subfamily B member 14
AAEL01024	uncharacterized LOC5580039	-0.3013250139924	0.0404397581902632	0.316225178415075	NA	NA	NA	AAS22239.1putative 8.7 kDa secreted protein
AAEL02278	NA	-0.301264658131186	0.504685048934473	0.858744381072989	NA	NA	NA	XP_021694147.1histone deacetylase 11
AAEL00002	L-dopachrome tautomerase yellow-f	-0.301043472738815	0.0488468099858121	0.351987741182022	NA	aag01100 ; aag	KXJ72581.1 hypothet	XP_001647842.1L-dopachrome tautomerase yellow-f
AAEL01360	integrin alpha-PS2	-0.300972509941155	0.0190558352781888	0.225171411092632	NA	NA	NA	XP_021694910.1integrin alpha2 isoform X1
AAEL02678	NA	-0.300753992522169	0.03857381015906	0.310005415793598	NA	aag01100 ; aag	NA	XP_021699065.1xanthine dehydrogenase
AAEL02521	NA	-0.300674702391354	0.12752096874305	0.532418008834123	NA	NA	NA	XP_021703407.1signal-induced proliferation-associated 1-like protein 2 isoform X1
AAEL00462	DTW domain-containing protein 1	-0.300323074887819	0.297983947482858	0.7302583490254	NA	NA	KXJ82449.1 hypothet	XP_001649526.2DTW domain-containing protein 1
AAEL00037	integrin-linked protein kinase	-0.298995505783839	0.0140758266269445	0.190425211307095	NA	NA	ETN59472.1 integrin-	XP_001655514.1integrin-linked protein kinase
AAEL00545	WASH complex subunit 2	-0.298994704266451	0.305177744021646	0.738299809812121	NA	aag04144	KXJ82349.1 hypothet	XP_001650910.2WASH complex subunit 2
AAEL01039	uncharacterized LOC5573307	-0.298588208174833	0.405537037536213	0.801665267327225	NA	NA	NA	XP_021707322.1uncharacterized protein LOC5573307
AAEL01243	general transcriptional corepressor trfA	-0.298570141657768	0.283005907715897	0.715753918648043	NA	NA	NA	XP_001662585.2general transcriptional corepressor trfA isoform X2
AAEL00507	GTP-binding protein Di-Ras2	-0.298365359901176	0.281784324916438	0.715107379448722	NA	NA	KFB42719.1 AGAP00	XP_001650313.2GTP-binding protein Di-Ras2
AAEL01988	NA	-0.298239508783504	0.495813256131585	0.854555831227354	NA	NA	NA	XP_021703238.1zinc finger protein 2
AAEL01298	nuclear pore complex protein Nup85	-0.298152374808334	0.163211650373975	0.581366301644056	NA	aag03013	NA	XP_021706108.1nuclear pore complex protein Nup85
AAEL00257	myosin regulatory light chain 2	-0.297990745429324	0.0311201981274933	0.282649198438896	NA	NA	AAZ67334.1 putative	XP_001655578.1myosin regulatory light chain 2
AAEL01374	presenilins-associated rhomboid-like protein, mitochondrial	-0.297979188366126	0.183567218032764	0.611093779135432	NA	NA	NA	XP_001663953.1presenilins-associated rhomboid-like protein, mitochondrial
AAEL00681	microsomal glutathione S-transferase 1	-0.29792533013369	0.402353867874358	0.799341786858961	NA	NA	KFB40178.1 AGAP00	XP_021706344.1microsomal glutathione S-transferase 1
AAEL01241	slit homolog 1 protein	-0.297094007345405	0.263378893353569	0.697643228739073	NA	NA	KXJ73948.1 hypothet	XP_021698125.1slit homolog 1 protein
AAEL01976	NA	-0.296742499819672	0.176948759825586	0.601952860597058	NA	aag04146 ; aag	NA	XP_021695730.1superoxide dismutase [Cu-Zn]
AAEL00484	NA	-0.296711467038504	0.279577069953164	0.713638889794457	NA	NA	KXJ75168.1 hypothet	XP_001649918.2MIT domain-containing protein 1 isoform X1
AAEL01191	zinc finger protein 449	-0.296183519682018	0.382617590093608	0.788720220439378	NA	NA	KXJ68843.1 hypothet	XP_001662036.2zinc finger protein 449
AAEL00731	glucose-6-phosphate 1-epimerase	-0.295971236462465	0.106689806107174	0.492031204248678	10	aag01100 ; aag	CRL02671.1 CLUMA_	XP_019534297.1 glucose-6-phosphate 1-epimerase
AAEL01486	glycogenin-1	-0.295894734376805	0.0459274212954088	0.340551273779494	NA	NA	NA	XP_021695674.1anucleate primary sterigmata protein A isoform X1
AAEL02196	NA	-0.295720237194183	0.0764963004924787	0.422703978006619	310	aag01100 ; aag	NA	XP_021704601.1histone-lysine N-methyltransferase trithorax isoform X2
AAEL00016	NA	-0.295679016410773	0.183620332461364	0.611093779135432	NA	NA	KXJ72229.1 hypothet	XP_021707205.1D-amino-acid oxidase
AAEL01425	aquaporin-11	-0.295400260263414	0.043021185427351	0.327632237384908	NA	NA	NA	XP_019529216.1 aquaporin-12
AAEL01724	serpin B8	-0.295386522169002	0.440583895517347	0.822798752521693	NA	NA	NA	XP_011493153.2serpin B8
AAEL01024	anoctamin-10	-0.29502983249599	0.0298471526287585	0.278836039075975	NA	NA	NA	XP_019534229.1 anoctamin-10-like isoform X1
AAEL00155	uncharacterized LOC5571295	-0.294942341286673	0.541531339455154	0.869735874782513	NA	NA	XP_001841995.1 con	XP_001653598.1uncharacterized protein LOC5571295 isoform X2
AAEL01004	protein FAM50 homolog	-0.294919274965068	0.208282858331306	0.639832374753494	NA	NA	NA	XP_001654172.1protein FAM50 homolog
AAEL01014	uncharacterized LOC5572927	-0.294886116005657	0.013888098721107	0.188771468879804	NA	NA	NA	XP_021700684.1uncharacterized protein LOC5572927 isoform X4
AAEL01385	uncharacterized LOC5578811	-0.29477841562292	0.433203696670146	0.820258728065422	NA	NA	NA	XP_316466.3C-type lectin, galactose-binding (AGAP006430)
AAEL00125	facilitated trehalose transporter Tret1	-0.294774979740592	0.211253697803495	0.643406565246605	NA	NA	KXJ73638.1 hypothet	XP_001652873.1facilitated trehalose transporter Tret1
AAEL00594	28S ribosomal protein S23, mitochondrial	-0.294617901021361	0.0569983426340605	0.375057077651547	NA	NA	ETN67166.1 mitocho	XP_001651727.228S ribosomal protein S23, mitochondrial
AAEL02526	NA	-0.294072151235146	0.0710038034780006	0.409506895880945	NA	NA	NA	XP_021707401.1heterogeneous nuclear ribonucleoprotein L isoform X7
AAEL01271	protein CutA homolog	-0.294031261100536	0.134458681225784	0.542662316284181	NA	NA	NA	XP_001662826.2protein CutA homolog isoform X1
AAEL02537	NA	-0.293927776022967	0.376609401439556	0.783277809573714	NA	NA	NA	XP_021696720.1zinc finger protein 26-like
AAEL02016	NA	-0.293592116764391	0.205372681476481	0.638023927878659	NA	NA	NA	XP_021693083.1sodium-independent sulfate anion transporter isoform X1
AAEL01085	ubiquitin carboxyl-terminal hydrolase 2	-0.293287932776525	0.0151290822002565	0.198907702459398	NA	NA	XP_019558639.1 PRE	XP_021708368.1serine-rich adhesin for platelets isoform X1
AAEL00108	serine protease easter	-0.293070826292784	0.368985781044576	0.781817769662763	NA	NA	KXJ83229.1 hypothet	XP_001658011.2serine protease easter
AAEL00074	troponin C	-0.292466852813296	0.0279874992804784	0.271500221524543	NA	NA	ETN59588.1 troponir	XP_001650660.1troponin C
AAEL00062	GTP:AMP phosphotransferase AK3, mitochondrial	-0.292274398762565	0.143642249023098	0.555843555723525	00230 ; 00730 ; 00230	aag00230	KFB38356.1 hypothe	XP_001649167.1GTP:AMP phosphotransferase AK3, mitochondrial
AAEL00248	probable chitinase 10	-0.292041790230972	0.148328801266809	0.562679383080301	NA	NA	KXJ79418.1 hypothet	XP_001655366.2probable chitinase 10
AAEL00865	chaoptin	-0.291973477376323	0.0218963528444771	0.241595010641341	NA	NA	KXJ72231.1 hypothet	XP_019526840.1 leucine-rich repeats and immunoglobulin-like domains protein 1
AAEL00877	tyrosine-protein kinase Abl	-0.291721741866949	0.0669350694306005	0.399699227060601	4660	NA	NA	XP_001659488.2tyrosine-protein kinase Abl isoform X3
AAEL02708	NA	-0.291673560652055	0.127393724017002	0.532369231791303	NA	NA	NA	XP_021694991.1eukaryotic translation initiation factor 3 subunit A
AAEL01343	spaetzle 1A	-0.2914050585852	0.0302406516216536	0.280801691445466	NA	aag04624	NA	XP_021703617.1uncharacterized protein LOC5577953 isoform X4
AAEL00884	sarcoplasmic calcium-binding protein 1	-0.291321295482854	0.0398184180496179	0.313706406586116	NA	NA	KFB49609.1 AGAP00	XP_001653462.1sarcoplasmic calcium-binding protein 1
AAEL00397	elongation of very long chain fatty acids protein 4	-0.291080956796965	0.234687560624672	0.668737559309875	62	aag01100 ; aag	KXJ79972.1 hypothet	XP_001648136.1elongation of very long chain fatty acids protein 4
AAEL01824	NA	-0.290888014598021	0.0555564539931607	0.370725518234274	480	NA	NA	XP_021693815.1aminopeptidase N
AAEL02398	NA	-0.290792425549688	0.310385166997466	0.742918550320373	NA	NA	NA	NA
AAEL01979	NA	-0.290255804995789	0.0281332566121102	0.271910830247844	NA	NA	NA	XP_001661921.1venom allergen 5
AAEL00237	esterase B1	-0.290141853928178	0.0181068340611311	0.217871885086637	NA	NA	KXJ71545.1 hypothet	XP_021699612.1esterase B1
AAEL00689	ATP-dependent 6-phosphofructokinase	-0.290003023449404	0.0727155772044642	0.414305869631496	00010 ; 00052 ; 00051 ;	aag01100 ; aag	XP_019553806.1 PRE	XP_001652300.1ATP-dependent 6-phosphofructokinase isoform X2
AAEL01499	clavesin-2	-0.289913236984805	0.172776908285617	0.595911960286448	NA	NA	NA	XP_019557074.1 clavesin-2-like
AAEL00902	PDF receptor	-0.289801057321353	0.261832766532123	0.695344775901121	NA	NA	XP_019533514.1 PRE	XP_021711367.1PDF receptor isoform X1

AAEL01462	NA	-0.289773783761146	0.233646251627374	0.667084333687587	NA	NA	NA	XP_021698248.117-beta-hydroxysteroid dehydrogenase 13
AAEL01696	solute carrier family 23 member 2	-0.289563410256457	0.0394430836709053	0.313353264954028	NA	NA	NA	XP_021705930.1solute carrier family 23 member 2 isoform X1
AAEL00768	protein bunched, class 2/F/G isoform	-0.289250840816295	0.0181061129322648	0.217871885086637	NA	NA	KFB34953.1 AGAP01	XP_021700469.1protein bunched, class 2/F/G isoform
AAEL02609	NA	-0.289111405408488	0.276962886842374	0.712300862136256	NA	NA	NA	---NA---
AAEL01187	cytochrome c1, heme protein, mitochondrial	-0.289040438357249	0.0453838463911056	0.338330197910056	NA	aag01100 ; aag	ETN66619.1 cytochr	XP_001662013.1cytochrome c1, heme protein, mitochondrial
AAEL01985	NA	-0.288726622407551	0.116986264981421	0.514582987743323	901	NA	NA	XP_021704855.1uncharacterized protein LOC5564429
AAEL00828	pupal cuticle protein Edg-78E	-0.288691800610696	0.0205623624014343	0.233598591133332	NA	NA	ETN66852.1 cuticula	XP_019539066.1 pupal cuticle protein Edg-78E-like
AAEL01063	uncharacterized LOC5573649	-0.28848915289335	0.049606917912162	0.35468753584127	NA	NA	KXJ70273.1 hypothet	XP_001660975.1uncharacterized protein LOC5573649
AAEL00282	uncharacterized LOC5576223	-0.288422845248271	0.0471798967433995	0.346180765881814	NA	aag01100 ; aag	KXJ74193.1 hypothet	XP_001848343.1NADH:ubiquinone dehydrogenase
AAEL02777	NA	-0.288151435951591	0.0346153661009331	0.294935739685914	NA	aag01100 ; aag	NA	XP_021707104.1NADH-ubiquinone oxidoreductase subunit 8
AAEL00108	uncharacterized LOC5568364	-0.287813662613002	0.465805665345301	0.838483018891546	NA	NA	KFB53548.1 hypothe	XP_001658016.1uncharacterized protein LOC5568364
AAEL02533	NA	-0.287778500479556	0.152467929569436	0.568113296551786	NA	NA	NA	XP_021705052.1CD109 antigen-like
AAEL02019	NA	-0.287081992505128	0.0414451320922714	0.321025341340978	NA	NA	NA	XP_021703013.1microfibril-associated glycoprotein 4-like
AAEL01431	RNA polymerase II degradation factor 1	-0.287041929676701	0.0368375082875562	0.303201029751424	NA	NA	NA	XP_001648422.1RNA polymerase II degradation factor 1
AAEL01145	proline-rich extensin-like protein EPR1	-0.286719205495297	0.288763709512536	0.721217831038796	NA	NA	XP_019527181.1 PRE	XP_021696262.1proline-rich extensin-like protein EPR1 isoform X2
AAEL00715	probable ATP-dependent RNA helicase kurz	-0.286335982305835	0.0763761076460798	0.422703978006619	NA	NA	KXJ72274.1 hypothet	XP_021704688.1probable ATP-dependent RNA helicase kurz
AAEL00652	adapter molecule Crk	-0.28575588903145	0.505406422711254	0.858744381072989	NA	NA	ETN60685.1 crk [Ano	XP_001652002.1adapter molecule Crk
AAEL00138	probable small nuclear ribonucleoprotein Sm D1	-0.285682429266339	0.131516213583928	0.538981683630591	NA	aag03040	ETN68120.1 small nu	XP_001653165.1probable small nuclear ribonucleoprotein Sm D1
AAEL01959	NA	-0.285609714711332	0.0271896203770644	0.268836665026084	NA	NA	NA	XP_021695777.1facilitated trehalose transporter Tret1-2 homolog
AAEL00129	uncharacterized LOC5569806	-0.284838518723379	0.0808998708963497	0.433079891352508	NA	NA	ETN63779.1 hypothe	XP_011493405.1uncharacterized protein LOC5569806
AAEL00323	transmembrane protease serine 9	-0.284551022676877	0.22879661867396	0.662455890682856	NA	NA	XP_019531243.1 PRE	XP_001656659.2transmembrane protease serine 9
AAEL02045	NA	-0.28412225954091	0.0395397919765812	0.313353264954028	NA	NA	NA	XP_021696724.1 titin
AAEL00984	microtubule-associated protein futsch	-0.284119803843877	0.0289836338227999	0.275227088745645	NA	NA	NA	XP_021710788.1microtubule-associated protein futsch
AAEL01255	myosin heavy chain, striated muscle	-0.284086773070576	0.0512436511227153	0.359690131564899	NA	NA	KFB44848.1 Ofd1 pro	XP_001662695.1myosin heavy chain, striated muscle
AAEL00546	alpha-N-acetylgalactosaminidase	-0.284058125510295	0.0303225531308126	0.280995099606986	00561 ; 00052 ; 00603 ;	aag01100 ; aag	KFB39533.1 AGAP00	XP_019543023.1 alpha-N-acetylgalactosaminidase-like
AAEL01377	uncharacterized LOC5578593	-0.284051207251212	0.0365122746424301	0.301874170044602	NA	NA	NA	XP_021700849.1uncharacterized protein LOC5578593
AAEL00112	probable 4-coumarate--CoA ligase 1	-0.284024847636032	0.0163223150555526	0.20659964668665	NA	NA	OWR45044.1 putativ	XP_001652383.2probable 4-coumarate--CoA ligase 1
AAEL00606	probable methyltransferase-like protein 15 homolog	-0.283762468126221	0.0363770867414066	0.301569326301282	NA	NA	KXJ75553.1 hypothet	XP_001657398.1probable methyltransferase-like protein 15 homolog
AAEL00523	uncharacterized LOC5566175	-0.282920072663418	0.0914883395091747	0.459288434349932	NA	NA	ETN66491.1 hypothe	XP_001650523.1uncharacterized protein LOC5566175
AAEL00064	mpv17-like protein	-0.282906783905311	0.0969309976559201	0.470633908275356	NA	aag04146	ETN67758.1 hypothe	XP_001649804.1mpv17-like protein
AAEL00408	echinoderm microtubule-associated protein-like 2	-0.282538997264545	0.0356084843330783	0.299241345052956	NA	NA	XP_019556863.1 PRE	XP_001648438.2echinoderm microtubule-associated protein-like 2 isoform X2
AAEL00225	uncharacterized LOC5574102	-0.282038473770989	0.456033372570556	0.832617462606238	NA	NA	KXJ70501.1 hypothet	XP_001866315.1arylalkylamine N-acetyltransferase
AAEL00466	protein Fe65 homolog	-0.281682284294179	0.226532754184916	0.660446829146891	NA	NA	XP_019550238.1 PRE	XP_021706960.1protein Fe65 homolog isoform X5
AAEL00686	major facilitator superfamily domain-containing protein 12	-0.281404276831574	0.0401442449358897	0.314545859748141	NA	NA	KXJ75918.1 hypothet	XP_001652284.1major facilitator superfamily domain-containing protein 12
AAEL00272	leukocyte elastase inhibitor	-0.281266465746362	0.0179761751672368	0.217327183278252	NA	NA	XP_001848442.1 seri	XP_001662183.2leukocyte elastase inhibitor
AAEL01951	NA	-0.281026443576263	0.381371542193497	0.787361680182234	NA	NA	NA	XP_021710776.1reversion-inducing cysteine-rich protein with Kazal motifs isoform X1
AAEL01286	N-acylneuraminate cytidylyltransferase A	-0.280872093063836	0.489598885025022	0.849413450226291	540	aag01100 ; aag	NA	XP_001663017.1N-acylneuraminate cytidylyltransferase A
AAEL02226	NA	-0.280643522720377	0.306528597343146	0.738783696372872	NA	NA	NA	XP_021693403.1lysyl oxidase homolog 2B
AAEL01311	excitatory amino acid transporter 1	-0.280567566272075	0.0247053091013696	0.257753122846716	NA	NA	NA	XP_001656353.2excitatory amino acid transporter 1
AAEL00699	sodium-coupled monocarboxylate transporter 1	-0.280377008543324	0.0511538559573254	0.359690131564899	NA	NA	ETN61805.1 hypothe	XP_001652483.1sodium-coupled monocarboxylate transporter 1 isoform X1
AAEL01134	uncharacterized LOC5574706	-0.280375026270271	0.0941249350186221	0.464823706598816	NA	NA	KXJ75409.1 hypothet	XP_001661603.1uncharacterized protein LOC5574706
AAEL01067	uncharacterized LOC5573691	-0.280333764633524	0.152149400865319	0.568113296551786	NA	NA	XP_001843372.1 con	XP_021695485.1uncharacterized protein LOC5573691
AAEL01173	uncharacterized LOC5575275	-0.280083969560407	0.15839710729492	0.576186113377291	NA	NA	NA	XP_001661881.1uncharacterized protein LOC5575275 isoform X1
AAEL01504	mitochondrial carrier homolog 2	-0.279976784383855	0.125164604725619	0.530276501925028	NA	NA	NA	XP_001650362.2mitochondrial carrier homolog 2
AAEL00966	zinc finger CCHC domain-containing protein 24	-0.279961583704856	0.190909076938202	0.62010071513778	NA	NA	NA	XP_021706394.1zinc finger CCHC domain-containing protein 24 isoform X1
AAEL01141	switch-associated protein 70	-0.279091518586401	0.281419613244024	0.715107379448722	NA	NA	XP_019546529.1 PRE	XP_021708203.1switch-associated protein 70
AAEL02566	NA	-0.279036225742709	0.284812633722551	0.71725411692463	NA	NA	NA	XP_021708404.1uncharacterized protein LOC5575051
AAEL02747	NA	-0.278917811347444	0.0896769498792188	0.45743198048147	NA	NA	NA	XP_021698919.1probable ATP-dependent RNA helicase Dbp73D
AAEL00038	protein flightless-1 homolog	-0.27884532679572	0.477263719777132	0.845011493487133	NA	NA	XP_019556937.1 PRE	XP_021710795.1protein flightless-1 homolog
AAEL02767	NA	-0.278803519725901	0.0740534248539931	0.418712453878531	NA	NA	NA	XP_001662100.1protein hairy
AAEL01179	venom allergen 5	-0.278659753141922	0.0351470274338483	0.296718299491993	NA	NA	KXJ79581.1 hypothet	XP_001661917.1venom allergen 5
AAEL02492	NA	-0.278034946917797	0.033130897205423	0.285726819198035	NA	aag04130	NA	XP_021697366.1vesicle-associated membrane protein 2 isoform X1
AAEL01962	NA	-0.277873127247756	0.0329200325779402	0.285244952713941	NA	NA	NA	XP_021707940.1ras-associated and pleckstrin homology domains-containing protein 1 isoform X2
AAEL00372	uncharacterized LOC5578871	-0.277818434207515	0.383212935700221	0.788887121770344	NA	NA	KXJ73545.1 hypothet	XP_021699359.1uncharacterized protein LOC5578871
AAEL00984	uncharacterized LOC5572512	-0.277722280798818	0.0395974730534687	0.313353264954028	NA	NA	NA	XP_019559190.1 galectin-4-like
AAEL00682	uncharacterized LOC5568412	-0.277677881536935	0.0430295475957187	0.327632237384908	NA	NA	NA	XP_021697021.1uncharacterized protein LOC5568412 isoform X5
AAEL02713	NA	-0.277480478742963	0.177408940065167	0.602182613972581	NA	NA	NA	XP_021696054.1rap guanine nucleotide exchange factor 4 isoform X2
AAEL00726	protein kibra	-0.277450050045747	0.266288583957381	0.701714647644852	NA	aag04391 ; aag	XP_001869112.1 WV	XP_021692995.1protein kibra
AAEL01086	serine protease snake	-0.277407310007218	0.126566359527415	0.530481545423179	NA	NA	KXJ70392.1 hypothet	XP_001661116.2serine protease snake
AAEL02778	NA	-0.277400099257438	0.0164326662336393	0.206858269058754	NA	NA	NA	XP_021706770.1chromodomain-helicase-DNA-binding protein Mi-2 homolog
AAEL00369	glia-derived nexin	-0.277396971676869	0.0327422823392958	0.28463170766102	NA	NA	KXJ73148.1 hypothet	XP_001657103.3glia-derived nexin
AAEL00648	probable uridine nucleosidase 1	-0.276639156369081	0.136753913980563	0.545589928345625	NA	NA	KXJ68210.1 hypothet	XP_001651977.1probable uridine nucleosidase 1
AAEL00958	endonuclease G, mitochondrial	-0.276333418565712	0.282122876285748	0.715554087745416	NA	NA	NA	XP_001660269.1endonuclease G, mitochondrial
AAEL00177	homeotic protein distal-less	-0.2762954430288	0.37947596111661	0.785666748907449	NA	NA	XP_019562600.1 PRE	XP_021698711.1homeotic protein distal-less isoform X3
AAEL02245	NA	-0.276245350549099	0.515146042201521	0.861448202130317	NA	NA	NA	XP_021705663.1UPF0488 protein CG14286
AAEL01445	uncharacterized LOC5564470	-0.276204540942402	0.364049474907192	0.77844728212936	NA	NA	NA	XP_001648813.1uncharacterized protein LOC5564470
AAEL00975	uncharacterized LOC5572358	-0.276000300488007	0.238356443582498	0.67280858706937	NA	NA	NA	XP_021705669.1uncharacterized protein LOC5572358
AAEL00732	uncharacterized protein DDB_G0271670	-0.275736468098738	0.116940876796064	0.514582987743323	NA	NA	KXJ80100.1 hypothet	XP_019539302.1 phosphatidylinositol 3-kinase 2 isoform X1
AAEL01100	microfibril-associated glycoprotein 4	-0.275554598920087	0.0542994786939679	0.367469712613638	NA	NA	KXJ72641.1 hypothet	XP_001661294.1microfibril-associated glycoprotein 4
AAEL00160	uncharacterized LOC5571504	-0.275370654467485	0.543855627067604	0.870055542468027	NA	NA	KXJ83677.1 hypothet	XP_001659755.1uncharacterized protein LOC5571504 isoform X2
AAEL00992	exocyst complex component 2	-0.275207009339864	0.34030486508392	0.761379832492257	NA	NA	NA	XP_001654115.2exocyst complex component 2
AAEL01452	probable sodium/potassium/calcium exchanger CG1090	-0.275059808013294	0.0943747985274559	0.465640264332134	NA	NA	NA	XP_021713379.1probable sodium/potassium/calcium exchanger CG1090
AAEL00097	ESF1 homolog	-0.274926832468745	0.0484188321867716	0.35027523096122	NA	NA	KXJ69396.1 hypothet	XP_021711081.1ESF1 homolog
AAEL02492	NA	-0.274913399906828	0.298392762432836	0.730438157844279	NA	NA	NA	NA
AAEL00673	NADPH:adrenodoxin oxidoreductase, mitochondrial	-0.274771167228913	0.160308589918211	0.577674739374419	NA	NA	KXJ76370.1 hypothet	XP_001652198.1NADPH:adrenodoxin oxidoreductase, mitochondrial
AAEL00472	inositol-pentakisphosphate 2-kinase	-0.273972829107805	0.173628257526081	0.596612108198282	00562 ; 04070	aag01100 ; aag	KFB39145.1 AGAP00	XP_001649672.1inositol-pentakisphosphate 2-kinase
AAEL01987	NA	-0.273858297316041	0.282934544830089	0.715753918648043	NA	NA	NA	XP_021699378.1uncharacterized protein LOC5568462 isoform X3
AAEL00893	hornerin	-0.273619481514486	0.0957731594675779	0.468280878544448	NA	NA	KXJ71072.1 hypothet	XP_021708112.1hornerin
AAEL01407	serine protease inhibitor 27A	-0.273536888603619	0.0298240246757108	0.278836039075975	NA	NA	NA	XP_021698111.1serine protease inhibitor 27A
AAEL00260	proclotting enzyme	-0.273449375308962	0.0173689220463774	0.214247748888425	NA	NA	XP_019531740.1 PRE	XP_001655702.2suppressor of tumorigenicity 14 protein isoform X1
AAEL01261	general transcriptional corepressor trfA	-0.273444787123821	0.204748851453283	0.637782699712521	NA	NA	NA	XP_021705860.1general transcriptional corepressor trfA isoform X5
AAEL00242	BTB/POZ domain-containing protein 6-B	-0.273079892669084	0.0193164005510752	0.226893947646744	NA	NA	XP_019554046.1 PRE	XP_021709444.1BTB/POZ domain-containing protein 6-B isoform X1

AAEL02141	NA	-0.27249075416292	0.150951786440728	0.566025396924239	NA	NA	NA	XP_021698438.1cubilin homolog
AAEL02015	NA	-0.272178495806765	0.432385735298632	0.820005280371151	NA	NA	NA	XP_021704285.1high affinity cGMP-specific 3',5'-cyclic phosphodiesterase 9A-like
AAEL00698	putative phosphatidate phosphatase	-0.272123732387684	0.525895930099324	0.865680248818465	NA	NA	XP_019559027.1 PRE	XP_021700319.1putative phosphatidate phosphatase isoform X2
AAEL00989	endothelin-converting enzyme homolog	-0.271914811233208	0.0335407607278831	0.288181214022391	NA	NA	NA	XP_001654072.2endothelin-converting enzyme homolog isoform X2
AAEL01974	NA	-0.271852280343382	0.357775279256397	0.773736338358957	NA	NA	NA	XP_021708841.1RIMS-binding protein 2 isoform X7
AAEL01327	testicular acid phosphatase homolog	-0.271361217279016	0.350779894421512	0.768176246660341	NA	NA	NA	XP_001663437.2testicular acid phosphatase homolog
AAEL01531	cAMP-dependent protein kinase type II regulatory subunit	-0.271314250842954	0.0578123366571146	0.376859666960514	NA	NA	NA	XP_021697693.1cAMP-dependent protein kinase type II regulatory subunit isoform X7
AAEL01081	isocitrate dehydrogenase [NAD] subunit gamma, mitochondrial	-0.270808436099673	0.0662760799551305	0.399131209258581	NA	aag01100 ; aag	XP_001655018.1 isoc	XP_001655018.1isocitrate dehydrogenase [NAD] subunit gamma, mitochondrial isoform X1
AAEL00047	major facilitator superfamily domain-containing protein 6	-0.2706505777249	0.128778303416084	0.535335481333802	NA	NA	KFB53186.1 AGAP00	XP_021705543.1major facilitator superfamily domain-containing protein 6
AAEL00593	uncharacterized LOC5567268	-0.270468386174243	0.440129297512051	0.822798752521693	NA	NA	XP_019545874.1 PRE	XP_019545874.1 putative lysozyme-like protein isoform X1
AAEL00850	uncharacterized LOC5570687	-0.270416494744145	0.184140963063784	0.611957279573706	NA	NA	NA	XP_021702710.1uncharacterized protein LOC5570687
AAEL00547	uncharacterized LOC5566553	-0.270387527929938	0.0894529233175357	0.457261843652237	NA	NA	XP_311392.4 AGAP0	XP_001650930.2uncharacterized protein LOC5566553
AAEL00073	3 beta-hydroxysteroid dehydrogenase/Delta 5-->4-isomerase	-0.269777291983256	0.400774567098831	0.798648539435638	NA	aag01100	XP_019536475.1 PRE	XP_001650664.13 beta-hydroxysteroid dehydrogenase/Delta 5--
AAEL01310	rho guanine nucleotide exchange factor 11	-0.269371848314864	0.050171541202787	0.356468207181388	NA	NA	NA	XP_021709518.1rho guanine nucleotide exchange factor 11 isoform X5
AAEL01044	NA	-0.269294545522929	0.245920734300402	0.679727114240672	NA	NA	NA	XP_021709939.1membrane-associated guanylate kinase, WW and PDZ domain-containing protein 1 isoform X1
AAEL02792	NA	-0.268994931931338	0.35346860599527	0.770323402046695	NA	NA	NA	XP_021695163.1protein peste
AAEL00926	uncharacterized LOC5571728	-0.268945330600346	0.391938282947822	0.793285976044306	NA	NA	NA	XP_021700724.1uncharacterized protein LOC5571728
AAEL02245	NA	-0.268802925520933	0.11526013844798	0.511637141340239	NA	NA	NA	NA
AAEL01327	carcinine transporter	-0.268798888583373	0.167604297345919	0.587554508928878	NA	NA	NA	XP_021701957.1carcinine transporter
AAEL01047	nucleotide exchange factor SIL1	-0.268702161725089	0.188090263073248	0.615069865245212	NA	aag04141	NA	XP_021693611.1nucleotide exchange factor SIL1
AAEL02210	NA	-0.268538658090603	0.17331560227242	0.596392068481585	NA	NA	NA	NA
AAEL00637	uncharacterized LOC5567907	-0.268314517331423	0.0287711203541712	0.274601260220884	NA	NA	KXJ68524.1 hypothet	XP_019543339.1 asporin-like
AAEL02429	NA	-0.268209623343959	0.193393218381579	0.624628157151667	NA	NA	NA	XP_021698282.1uncharacterized protein LOC110676084
AAEL01488	zinc finger protein 26	-0.268157919960125	0.365675546716731	0.77982534898896	NA	NA	NA	XP_021697489.1zinc finger protein 26 isoform X1
AAEL00341	ATPase inhibitor mai-2, mitochondrial	-0.268093638059009	0.0601611024728507	0.38417936499318	NA	NA	KXJ77258.1 hypothet	XP_001656815.1ATPase inhibitor mai-2, mitochondrial
AAEL00753	uncharacterized LOC5569297	-0.267997288376558	0.205813376446706	0.638023927878659	NA	NA	KXJ76579.1 hypothet	XP_001658423.1uncharacterized protein LOC5569297
AAEL00207	myosin-2 heavy chain	-0.2677282945358	0.0388059589695581	0.31084338200184	NA	NA	XP_019549849.1 PRE	XP_021702057.1myosin-2 heavy chain isoform X1
AAEL02245	NA	-0.267530038595511	0.483414692555144	0.847379421050056	NA	NA	NA	XP_019535160.1 zinc finger protein 260-like
AAEL01713	cytochrome P450 4c21	-0.266804257416264	0.0751289213223871	0.420011717015769	NA	NA	NA	XP_021706633.1cytochrome P450 4c21
AAEL02337	NA	-0.266678342816786	0.311327768677966	0.743341496464619	230	aag01100 ; aag	NA	XP_021696188.1 guanylate cyclase soluble subunit beta-1
AAEL02259	NA	-0.266388211912588	0.363175814730088	0.778250660803901	NA	aag04711	NA	XP_021695531.1circadian locomoter output cycles protein kaput
AAEL01396	tumor necrosis factor alpha-induced protein 8-like protein	-0.266093414700911	0.163315788931741	0.581366301644056	NA	NA	NA	XP_021710539.1tumor necrosis factor alpha-induced protein 8-like protein isoform X1
AAEL02358	NA	-0.266089492325094	0.485882653679454	0.847790841886424	NA	NA	NA	XP_021705111.1uncharacterized protein LOC5563928 isoform X1
AAEL00914	beclin-1-like protein B	-0.265830973090022	0.399839736064184	0.798648539435638	NA	NA	XP_001689003.1 AG/	XP_001659776.1beclin-1-like protein B
AAEL01370	trypsin-1	-0.265804813157149	0.0430813961351596	0.327632237384908	NA	NA	NA	XP_019529704.1 trypsin-4-like
AAEL00054	juvenile hormone esterase	-0.265748518475442	0.134840182350858	0.543019412688227	NA	NA	KXJ69215.1 hypothet	XP_021709265.1juvenile hormone esterase
AAEL01317	deoxynucleotidyltransferase terminal-interacting protein 2	-0.265578657887627	0.0567507302033914	0.374465659921115	NA	NA	NA	XP_001663357.2deoxynucleotidyltransferase terminal-interacting protein 2
AAEL01101	septin-4	-0.265578201763811	0.131623132072049	0.538981683630591	NA	aag04215	KXJ71255.1 hypothet	XP_019552179.1 septin-4
AAEL00889	sulfotransferase 1 family member D1	-0.26510028490381	0.120316531809095	0.521361270092351	NA	NA	KXJ77087.1 hypothet	XP_001659587.1sulfotransferase 1 family member D1
AAEL00463	condensin complex subunit 1	-0.264745437957753	0.17236406313555	0.594934024371093	NA	NA	KXJ81410.1 hypothet	XP_001649509.2condensin complex subunit 1 isoform X1
AAEL01311	beta-lactamase-like protein 2 homolog	-0.264719111297163	0.138900161782754	0.549029662748535	NA	NA	NA	XP_001656361.1beta-lactamase-like protein 2 homolog
AAEL02158	NA	-0.26427692656617	0.198470519843231	0.631728026149226	NA	NA	NA	XP_021712414.1 protein borderless
AAEL01029	MAGUK p55 subfamily member 7	-0.263889918179848	0.081000053473289	0.433079891352508	230	NA	NA	XP_021701809.1MAGUK p55 subfamily member 7 isoform X1
AAEL01817	NA	-0.263811969450599	0.364995788118206	0.779097945317497	NA	NA	NA	XP_021706615.1zinc finger protein 135 isoform X3
AAEL00779	proclotting enzyme	-0.263733024470622	0.0438767730439264	0.331489380583096	NA	NA	KXJ71653.1 hypothet	XP_001652921.1proclotting enzyme isoform X3
AAEL01810	NA	-0.263708750422588	0.0614908077513875	0.38668618011863	NA	NA	NA	XP_021700900.1uncharacterized protein LOC5565763
AAEL01097	paramyosin, long form	-0.263705590727772	0.0321281268039762	0.284442054201394	NA	NA	KXJ80479.1 hypothet	XP_001655070.1paramyosin, long form isoform X1
AAEL02479	NA	-0.263641409978028	0.44124844416357	0.82360333814237	NA	NA	NA	NA
AAEL02271	NA	-0.263576306516597	0.366163250133111	0.78032288738418	NA	NA	NA	XP_021710962.1uncharacterized protein LOC5571100 isoform X1
AAEL01814	NA	-0.263179824252639	0.376750151975734	0.783277809573714	NA	NA	NA	XP_001658411.2delta(9)-fatty-acid desaturase fat-7
AAEL02037	NA	-0.262957926228694	0.308555409433529	0.74133861034134	NA	NA	NA	XP_021706974.1uncharacterized family 31 glucosidase KIAA1161-like
AAEL00373	exocyst complex component 6	-0.262885070321252	0.165690299271398	0.584439853413553	NA	NA	KXJ78922.1 hypothet	XP_001664059.2exocyst complex component 6
AAEL00099	DNA replication licensing factor Mcm7	-0.262625665984023	0.143056461143593	0.555375365647863	NA	aag03030	ETN58769.1 DNA rep	XP_001657714.1DNA replication licensing factor Mcm7
AAEL00241	monocarboxylate transporter 12	-0.262493857886977	0.105646589507151	0.490349840292762	NA	NA	KXJ81300.1 hypothet	XP_001655208.2monocarboxylate transporter 12
AAEL00670	uncharacterized LOC5568272	-0.26231533978985	0.180343525462823	0.605728027045765	NA	NA	XP_320618.4 AGAP0	XP_001657969.2uncharacterized protein LOC5568272
AAEL00069	inactive hydroxysteroid dehydrogenase-like protein 1	-0.262084441554024	0.382988339077251	0.788887121770344	NA	NA	KXJ73517.1 hypothet	XP_021703814.1inactive hydroxysteroid dehydrogenase-like protein 1
AAEL01730	elongation factor 1-alpha	-0.261901751991801	0.051143676719088	0.359690131564899	NA	aag03013	NA	XP_011493325.1elongation factor 1-alpha
AAEL00165	putative inorganic phosphate cotransporter	-0.261529301771206	0.341049000869694	0.762101239923001	NA	NA	XP_019536917.1 PRE	XP_001653764.1putative inorganic phosphate cotransporter isoform X2
AAEL00909	protein crumbs	-0.261223934832634	0.10309923016983	0.485796149609891	NA	aag04391	XP_019534325.1 PRE	XP_001659720.2protein crumbs isoform X3
AAEL02130	NA	-0.26120377115867	0.306795866374397	0.738847307609842	NA	NA	NA	XP_021708608.1putative helicase MOV-10
AAEL00187	protein decapentaplegic	-0.261139533812225	0.144843650702275	0.558145424523593	NA	aag04013 ; aag	KXJ77311.1 hypothet	XP_001654153.2protein decapentaplegic
AAEL00922	transformer-2 protein homolog alpha	-0.260948272288047	0.126086753661528	0.530481545423179	NA	aag03040	NA	XP_001659833.2transformer-2 protein homolog alpha isoform X1
AAEL01972	NA	-0.260727607328175	0.158416742756417	0.576186113377291	NA	NA	NA	XP_021696145.1collagen alpha-1(XVIII) chain isoform X10
AAEL02004	NA	-0.2607134474353	0.20192729361234	0.634701781910801	NA	NA	NA	XP_021694584.1uncharacterized protein LOC5574321 isoform X2
AAEL01424	solute carrier family 52, riboflavin transporter, member 3-A	-0.260584208361802	0.051298680836638	0.359690131564899	NA	NA	NA	XP_001648267.1solute carrier family 52, riboflavin transporter, member 3-A
AAEL01973	NA	-0.260167455620121	0.370495712357707	0.781817769662763	NA	NA	NA	XP_021694997.1uncharacterized protein LOC5573415 isoform X3
AAEL00576	protein artichoke	-0.260069498337452	0.123345692264926	0.526206332972576	NA	NA	XP_019530746.2 PRE	XP_001651407.1protein artichoke isoform X1
AAEL01958	NA	-0.259854246201085	0.50000412619116	0.858029916331016	NA	NA	NA	XP_001660660.2protein bicaudal C
AAEL00376	cytochrome P450 9b2	-0.259745195765985	0.320041015098301	0.746140719770603	NA	NA	KXJ75237.1 hypothet	XP_001657205.2cytochrome P450 9b2
AAEL00912	probable cytochrome P450 6a14	-0.259582090359316	0.137928728894164	0.547441354014568	NA	NA	KXJ73590.1 hypothet	XP_001653687.3probable cytochrome P450 6a14
AAEL00273	cytochrome c oxidase subunit 7C, mitochondrial	-0.259386526775097	0.059548314879209	0.382389109224341	NA	aag01100 ; aag	AAV90732.1 cytochr	XP_001655893.1cytochrome c oxidase subunit 7C, mitochondrial
AAEL01086	facilitated trehalose transporter Tret1	-0.259297128726945	0.449118515047843	0.829306073695304	NA	NA	KXJ72479.1 hypothet	XP_001661118.2facilitated trehalose transporter Tret1
AAEL02025	NA	-0.259092273051898	0.117337854115404	0.515089129249485	NA	NA	NA	XP_021708965.1protein DDI1 homolog 2 isoform X1
AAEL02169	NA	-0.258970660717076	0.5462560276008	0.870931414970375	NA	NA	NA	XP_021712866.1cytochrome b561 isoform X2
AAEL02155	NA	-0.25895857908379	0.345152663651625	0.764941296190615	NA	NA	NA	NA
AAEL02339	NA	-0.258712927225036	0.160336124645579	0.577674739374419	NA	NA	NA	XP_001661424.1cytoplasmic phosphatidylinositol transfer protein 1
AAEL00767	protein tiptop	-0.25866475766347	0.376504291387769	0.783277809573714	NA	NA	KFB38516.1 AGAP00	XP_021702620.1protein tiptop
AAEL00564	uncharacterized LOC5566815	-0.25861777753298	0.23360767773445	0.667084333687587	NA	NA	KXJ70019.1 hypothet	XP_019530286.1 transmembrane protease serine 9-like
AAEL01434	multiple epidermal growth factor-like domains protein 8	-0.258507685705228	0.29177157691376	0.722646576583698	NA	NA	NA	XP_021700230.1multiple epidermal growth factor-like domains protein 8
AAEL01333	uncharacterized LOC5577700	-0.258454975354863	0.341887757824547	0.762438298723025	563	aag01100 ; aag	NA	XP_001663482.1uncharacterized protein LOC5577700
AAEL01506	protein sarah	-0.25844890745044	0.125656332108984	0.530481545423179	NA	NA	NA	XP_021699212.1protein sarah isoform X1
AAEL02505	NA	-0.258413839915263	0.451932638150435	0.831404265545842	NA	NA	NA	XP_019561193.1 retinaldehyde-binding protein 1-like

AAEL01300	uncharacterized LOC5577104	-0.258294969378017	0.477859757254607	0.845011493487133	NA	NA	NA	XP_021701926.1uncharacterized protein LOC5577104 isoform X2
AAEL01988	NA	-0.258168053279102	0.22860485366436	0.662349453217709	NA	NA	NA	XP_021704263.1sex peptide receptor
AAEL01210	acetylcholine receptor subunit alpha-like	-0.25815415396657	0.435291827011189	0.820960220424486	NA	NA	NA	XP_001655864.2acetylcholine receptor subunit alpha-like isoform X3
AAEL01833	NA	-0.258134156788907	0.0460993725412965	0.341281972315984	NA	NA	NA	XP_021710304.1ABC transporter G family member 23 isoform X3
AAEL00682	putative epidermal cell surface receptor	-0.257949701147075	0.106284422001054	0.490844601505817	NA	NA	KXJ68906.1 hypothet	XP_021705509.1putative epidermal cell surface receptor
AAEL00796	NA	-0.257883562884375	0.0981159437342988	0.473943787004209	NA	aag04933 ; aag	ETN60200.1 collagen	XP_021701997.1collagen alpha-1(IV) chain
AAEL01015	cytochrome P450 4d2	-0.257715118591435	0.614560691519534	0.896794716676617	NA	NA	NA	XP_001660657.2cytochrome P450 4d2
AAEL01751	glycine-rich cell wall structural protein 1.0	-0.257641169879024	0.0518927624874947	0.361717594818022	NA	NA	NA	XP_011493477.2glycine-rich cell wall structural protein 1.0
AAEL01982	NA	-0.257381488268433	0.0458848892563798	0.340510283013877	04660 ; 04658	aag03015	NA	XP_021709141.1serine/threonine-protein phosphatase PP1-beta catalytic subunit isoform X1
AAEL00682	cytochrome P450 4g15	-0.257338294951123	0.108557982087548	0.495725018486141	NA	NA	KXJ83027.1 hypothet	XP_001658068.2cytochrome P450 4g15
AAEL01370	histone H4	-0.257303342104287	0.229005648697558	0.662455890682856	NA	NA	NA	XP_001865168.1histone 1
AAEL00255	sodium-coupled monocarboxylate transporter 1	-0.257215338706925	0.0662071030288235	0.399054441537196	NA	NA	XP_019556115.1 PRE	XP_021713420.1sodium-coupled monocarboxylate transporter 1 isoform X2
AAEL02206	NA	-0.256963147949726	0.31083334637852	0.742915855032037	NA	NA	NA	NA
AAEL00967	group 3 secretory phospholipase A2	-0.25641750441614	0.0997053839033349	0.479106497482239	00564 ; 00591 ; 00565 ;	aag01100 ; aag	NA	XP_001653934.2group 3 secretory phospholipase A2 isoform X2
AAEL00444	zinc finger protein 25	-0.256392651227173	0.0567253060695857	0.374465659921115	NA	NA	XP_019555254.1 PRE	XP_021710833.1zinc finger protein 12 isoform X2
AAEL00427	cytochrome b5-related protein	-0.256358066530238	0.0467941504058447	0.345031868617454	NA	NA	KFB49249.1 AGAP01	XP_001648852.1cytochrome b5-related protein
AAEL00310	atlastin	-0.256091695667971	0.0252521637652849	0.259075173517489	NA	NA	KXJ76608.1 hypothet	XP_019528497.1 atlastin
AAEL01534	uncharacterized LOC5570300	-0.255768567239846	0.401120341677579	0.798934968584928	NA	NA	NA	KFB49214.1AGAP009795-like protein
AAEL01005	abscission/NoCut checkpoint regulator	-0.25575028928289	0.315917373882168	0.745684464362801	NA	NA	NA	XP_001654174.2abscission/NoCut checkpoint regulator isoform X1
AAEL02157	NA	-0.25563393556957	0.239682112729913	0.674660318489854	NA	NA	NA	XP_021700143.1E3 ubiquitin-protein ligase TRIM37 isoform X4
AAEL00008	CD109 antigen	-0.255603449710381	0.0513376818883808	0.359690131564899	NA	NA	XP_019545531.1 PRE	XP_001647863.2CD109 antigen
AAEL01220	myosin light chain alkali	-0.255423959021579	0.0866360416063992	0.448635258785642	NA	NA	KXJ74141.1 hypothet	XP_001655930.1myosin light chain alkali isoform X2
AAEL00080	golgin subfamily A member 2	-0.25519206943056	0.118036755953526	0.515958562099766	NA	NA	KXJ76790.1 hypothet	XP_021704283.1golgin subfamily A member 2
AAEL01129	vitamin K epoxide reductase complex subunit 1	-0.255063938729868	0.245324608312464	0.679727114240672	NA	aag01100 ; aag	XP_019545284.1 PRE	XP_001661572.1vitamin K epoxide reductase complex subunit 1
AAEL01994	NA	-0.254980848921877	0.213460679989909	0.646565232806829	NA	NA	NA	XP_021706181.1squamous cell carcinoma antigen recognized by T-cells 3
AAEL00976	mite group 2 allergen Lep d 2	-0.254962560781308	0.285877280643372	0.717970179170389	NA	NA	NA	XP_001660331.1mite group 2 allergen Lep d 2
AAEL00558	zinc finger protein 62	-0.25487544402675	0.456044307350042	0.832617462606238	NA	NA	XP_019536253.1 PRE	XP_001651114.2zinc finger protein 62
AAEL00653	PXMP2/4 family protein 3	-0.254872428149133	0.0299079517855609	0.279120661593034	NA	aag04146	KFB44974.1 AGAP00	XP_001652038.1PXMP2/4 family protein 3
AAEL02901	NA	-0.254674771585511	0.193823838758656	0.624986606780741	NA	NA	NA	XP_001651700.1mediator of RNA polymerase II transcription subunit 22 isoform X2
AAEL01986	NA	-0.254503499723068	0.0273741207977961	0.269120362800555	NA	NA	NA	XP_019533023.1 splicing factor, proline- and glutamine-rich-like
AAEL00023	uncharacterized LOC5572032	-0.254457465425158	0.281307145592622	0.715107379448722	NA	NA	KXJ78228.1 hypothet	XP_001660139.1uncharacterized protein LOC5572032
AAEL00918	protein encore	-0.254394610504963	0.118852720438202	0.517352286410753	NA	aag04320	NA	XP_021697323.1protein encore isoform X3
AAEL01973	NA	-0.254392233724271	0.103858953711526	0.486414446815465	NA	aag03013	NA	XP_021701100.1translation initiation factor eIF-2B subunit epsilon
AAEL01078	zinc carboxypeptidase A 1	-0.254304794970347	0.0756441484530262	0.420590606685648	NA	NA	KXJ79113.1 hypothet	XP_001654889.1zinc carboxypeptidase A 1
AAEL02173	NA	-0.254202385209256	0.270365220603207	0.706188123755524	NA	NA	NA	XP_021707175.1protein charlatan isoform X1
AAEL00692	leucine-rich repeat-containing G-protein coupled receptor 6	-0.254189418161882	0.216054491266766	0.649293784956494	NA	NA	KXJ79334.1 hypothet	XP_001652334.2leucine-rich repeat-containing G-protein coupled receptor 6
AAEL00713	probable leucine--tRNA ligase, mitochondrial	-0.254102237889689	0.154018005709247	0.570102046877109	970	aag00970	KXJ76256.1 hypothet	XP_001658155.1probable leucine--tRNA ligase, mitochondrial isoform X2
AAEL00351	ribonuclease H2 subunit A	-0.254089114406687	0.367660065077262	0.781160914070875	NA	aag03030	KXJ80504.1 hypothet	XP_001656938.1ribonuclease H2 subunit A
AAEL00951	proton-coupled folate transporter	-0.253568705188401	0.0788136108461547	0.429137779293678	NA	NA	NA	XP_001660178.2proton-coupled folate transporter isoform X1
AAEL02575	NA	-0.253332147335044	0.272397948577441	0.707756103918734	00513 ; 00510	aag01100 ; aag	NA	XP_021693315.1alpha-1,6-mannosyl-glycoprotein 2-beta-N-acetylglucosaminyltransferase isoform X1
AAEL00807	serine/threonine-protein kinase Doa	-0.253211101047192	0.0657115509768498	0.398076163323879	NA	NA	NA	XP_021693360.1serine/threonine-protein kinase Doa isoform X10
AAEL00693	NA	-0.252670520432013	0.315190019102763	0.745618464895825	NA	NA	ETN62709.1 structur	XP_001652359.1structural maintenance of chromosomes protein 3
AAEL00482	superoxide dismutase [Mn], mitochondrial-like	-0.252556909702549	0.120890256481712	0.522930064976407	NA	NA	XP_001649850.1 sup	XP_001649850.1superoxide dismutase [Mn], mitochondrial
AAEL00105	SET and MYND domain-containing protein 4	-0.252546836219584	0.383152809482683	0.788887121770344	NA	NA	KXJ69386.1 hypothet	XP_001657892.1SET and MYND domain-containing protein 4
AAEL00243	trimeric intracellular cation channel type 1B.1	-0.252511191538835	0.0387836260882799	0.31084338200184	NA	NA	ETN64982.1 transme	XP_001655265.1trimeric intracellular cation channel type 1B.1
AAEL00830	mitogen-activated protein kinase kinase kinase 15	-0.252223322105287	0.134630895784509	0.542662316284181	NA	aag04141 ; aag	NA	XP_021698715.1mitogen-activated protein kinase kinase kinase 15 isoform X2
AAEL00890	probable cation-transporting ATPase 13A3	-0.252140574041852	0.0633922174521344	0.393083008756429	NA	NA	XP_001865950.1 cati	XP_001659588.2probable cation-transporting ATPase 13A3
AAEL01175	NA	-0.251979931884642	0.0368158341732733	0.303201029751424	NA	aag01100 ; aag	KXJ73427.1 hypothet	XP_021706303.1glutathione S-transferase 1
AAEL00296	endochitinase	-0.251942176466226	0.0362171176729886	0.300966157937731	NA	NA	KXJ80791.1 hypothet	XP_019932989.1 endochitinase-like
AAEL01404	allantoicase	-0.251930277961188	0.107364791765405	0.492585616254939	230	aag01100 ; aag	NA	XP_019528471.1 allantoicase-like
AAEL00180	cytochrome P450 9e2	-0.251600076951852	0.112508395267514	0.503547757511443	NA	NA	KXJ78124.1 hypothet	XP_021702449.1cytochrome P450 9e2
AAEL02770	NA	-0.251513789579216	0.145884855450712	0.559813361074833	NA	NA	NA	XP_021704545.1receptor-mediated endocytosis protein 6 homolog
AAEL00778	uncharacterized LOC5569630	-0.251321086973546	0.0590410778025901	0.381093648037575	NA	NA	KXJ76604.1 hypothet	ABF18065.1putative 30.5 kDa secreted protein second family member
AAEL00921	protein melted	-0.251060053091467	0.18721067593856	0.614196367514984	NA	NA	NA	XP_021696476.1protein melted
AAEL02625	NA	-0.251040875319059	0.566360854086983	0.880302387766415	NA	NA	NA	XP_021694833.1XK-related protein 6
AAEL01871	NA	-0.250954242509369	0.120152532482375	0.521284113108352	NA	NA	NA	XP_001660834.1ankyrin repeat domain-containing protein 13B isoform X2
AAEL00324	deoxyribose-phosphate aldolase	-0.25083470406006	0.173033819928985	0.596189604220472	30	aag01100 ; aag	KXJ79315.1 hypothet	XP_001656607.2deoxyribose-phosphate aldolase
AAEL02907	NA	-0.250573425947532	0.0541463567714237	0.367217832240442	NA	NA	NA	XP_021694531.1serine protease snake-like isoform X2
AAEL01992	NA	-0.250538372015626	0.526179017194125	0.865680248818465	230	NA	NA	XP_021704515.1adenylate cyclase type 6 isoform X1
AAEL01122	scavenger receptor class B member 1	-0.250243572624211	0.0627490223414624	0.391603496697149	NA	NA	ETN61638.1 scaveng	XP_001661497.1scavenger receptor class B member 1 isoform X1
AAEL00802	pituitary homeobox homolog Ptx1	-0.250133062899325	0.476510681577388	0.844867278451347	NA	NA	XP_001862650.1 hor	XP_001658818.2pituitary homeobox homolog Ptx1
AAEL00572	glycine--tRNA ligase	-0.250013447545202	0.0317464006830511	0.284442054201394	970	aag00970	KXJ77781.1 hypothet	XP_001651394.2glycine--tRNA ligase isoform X1
AAEL01252	general transcription factor IIH subunit 3	-0.249970675917889	0.208227298260693	0.639832374753494	NA	aag03420 ; aag	KXJ82797.1 hypothet	XP_001662634.2general transcription factor IIH subunit 3
AAEL01984	NA	-0.249909416040853	0.0913796617968404	0.45924393656719	4070	aag04144 ; aag	NA	XP_021707067.1atypical protein kinase C isoform X3
AAEL01355	cytochrome P450 4C1	-0.249800779983451	0.526147343866688	0.865680248818465	NA	NA	NA	XP_001663752.2cytochrome P450 4C1
AAEL01337	uncharacterized LOC5577782	-0.249386097378693	0.310989160599226	0.742918550320373	NA	NA	NA	XP_021700765.1uncharacterized protein LOC5577782
AAEL01977	NA	-0.249324882708067	0.0593933022365764	0.382167830159655	NA	NA	NA	XP_021700238.1protein outspread isoform X1
AAEL01237	protein tramtrack, beta isoform	-0.249306358941386	0.288995489349995	0.721217831038796	NA	NA	KXJ82539.1 hypothet	XP_021697973.1protein tramtrack, alpha isoform isoform X1
AAEL02444	NA	-0.249301178595978	0.291440415882809	0.722646576583698	NA	aag03022	NA	XP_021708632.1transcription initiation factor TFIID subunit 3
AAEL01439	NA	-0.249088586043988	0.0424439972056175	0.324139866024524	620	aag01100 ; aag	NA	XP_001648645.2lactoylglutathione lyase isoform X1
AAEL02307	NA	-0.249035698248136	0.115836237469423	0.512764772622442	NA	NA	NA	XP_001656346.1probable serine/threonine-protein kinase kinX
AAEL00819	protein atonal homolog 8	-0.248844342770934	0.161460195717978	0.578565701322753	NA	NA	KXJ83387.1 hypothet	XP_001659017.1protein atonal homolog 8
AAEL00822	N-alpha-acetyltransferase 38, NatC auxiliary subunit	-0.248834084270074	0.181897372856454	0.60799841083367	NA	NA	XP_001867901.1 LSN	XP_001659059.1N-alpha-acetyltransferase 38, NatC auxiliary subunit
AAEL01959	NA	-0.248527510472632	0.175367874558569	0.599233264644618	NA	NA	NA	XP_021693820.1transmembrane protease serine 9 isoform X2
AAEL01391	uncharacterized LOC5578912	-0.248472545732861	0.121692168164483	0.523911973275319	NA	NA	NA	XP_021711093.1uncharacterized protein LOC5578912
AAEL00411	serine/threonine-protein kinase PITSLRE	-0.248354358981418	0.193743384699598	0.624986606780741	NA	NA	KXJ83371.1 hypothet	XP_001648451.1serine/threonine-protein kinase PITSLRE
AAEL00970	tetratricopeptide repeat protein 1	-0.24833965697928	0.300476289802304	0.732445779804186	NA	NA	NA	XP_001654011.1tetratricopeptide repeat protein 1 isoform X2
AAEL02410	NA	-0.248325592073456	0.314608030654166	0.745179690625389	NA	NA	NA	XP_021700778.1zinc finger protein 572
AAEL00415	uncharacterized LOC5564261	-0.248236909152028	0.494909147043508	0.853797144937076	NA	NA	KXJ73355.1 hypothet	AAC38958.1ovary ecdysteroidogenic hormone I precursor
AAEL01458	reticulocalbin-2	-0.248180853765318	0.200060271305589	0.633719317230304	NA	NA	NA	XP_019931760.1 reticulocalbin-2-like
AAEL01186	putative cyclin-dependent serine/threonine-protein kinase DDB_G02727	-0.248018552879167	0.372306820509905	0.781817769662763	NA	NA	KXJ74869.1 hypothet	XP_021696065.1putative cyclin-dependent serine/threonine-protein kinase DDB_G0272797/DDB_G0274007
AAEL00674	uncharacterized LOC5568318	-0.247903597179212	0.226376842418084	0.660446829146891	NA	NA	KXJ76367.1 hypothet	XP_021697073.1uncharacterized protein LOC5568318 isoform X2

AAEL00895	sentrin-specific protease 1	-0.247847338318254	0.111135646262679	0.502540647129813	NA	NA	XP_001862733.1 sen	XP_001653624.2sentrin-specific protease 1	
AAEL01713	lysozyme c-1	-0.247523254231769	0.066778186784694	0.399540230684495	NA	NA	NA	XP_021696190.1lysozyme c-1 isoform X1	
AAEL00493	NA	-0.247518443406139	0.312548060121705	0.744018629406202	NA	NA	XP_019535832.1 PRE	XP_001650036.2lipase 1	
AAEL00976	BTB/POZ domain-containing protein 3	-0.247206077803404	0.321997826520718	0.746650319748046	NA	NA	NA	XP_001660335.1BTB/POZ domain-containing protein 3	
AAEL00993	uncharacterized LOC5572622	-0.246870570863277	0.207811432337909	0.639832374753494	NA	NA	NA	KFB36307.1AGAP008322-like protein	
AAEL00290	phosphatidylinositol 4,5-bisphosphate 3-kinase catalytic subunit beta iso	-0.246743272009987	0.216299580710086	0.649392737910022	05170 ; 05163 ; 00562 ;	aag01100 ; aag	XP_001847784.1 pho	XP_021700427.1phosphatidylinositol 4,5-bisphosphate 3-kinase catalytic subunit beta isoform	
AAEL00938	1-phosphatidylinositol 4,5-bisphosphate phosphodiesterase	-0.246694718231808	0.0818267576048699	0.435494403400817	00562 ; 04070	aag01100 ; aag	NA	XP_001653804.11-phosphatidylinositol 4,5-bisphosphate phosphodiesterase isoform X2	
AAEL02747	NA	-0.246604464300184	0.207799096032925	0.639832374753494	NA	NA	NA	XP_021709176.1transient receptor potential channel pyrexia-like	
AAEL01543	serine protease easter	-0.246406030852407	0.0652083186720943	0.39730198438886	NA	NA	NA	XP_001652078.2serine protease easter	
AAEL00171	THO complex subunit 2	-0.246285415835594	0.0978813946302756	0.473599304703091	NA	aag03013 ; aag	KXJ80145.1 hypothet	XP_001653823.1THO complex subunit 2	
AAEL00694	Golgi SNAP receptor complex member 1	-0.245833191612414	0.327771678978179	0.750660774006273	NA	aag04130	ETN60415.1 golgi fan	XP_001652356.1Golgi SNAP receptor complex member 1	
AAEL02729	NA	-0.245618232572843	0.419225625099294	0.813348977896628	NA	NA	NA	NA	
AAEL01825	NA	-0.245592866312508	0.479654820436238	0.845454613425911	NA	aag04013	NA	XP_021708908.1mitogen-activated protein kinase kinase kinase 11 isoform X1	
AAEL00078	ubiquitin carboxyl-terminal hydrolase calypso	-0.245347918165037	0.443457657002201	0.82545872799161	NA	NA	KXJ68377.1 hypothet	XP_001651006.2ubiquitin carboxyl-terminal hydrolase calypso	
AAEL00935	leucine-rich repeats and immunoglobulin-like domains protein sma-10	-0.245036616835023	0.164731664063034	0.583247700156998	NA	NA	NA	XP_001659998.1leucine-rich repeats and immunoglobulin-like domains protein sma-10	
AAEL00539	voltage-dependent calcium channel subunit alpha-2/delta-3	-0.24497068851894	0.0672938585058113	0.4007529665455	NA	NA	XP_019529000.1 PRE	XP_021700557.1voltage-dependent calcium channel subunit alpha-2/delta-3 isoform X1	
AAEL00244	uncharacterized LOC5580145	-0.24474445947042	0.131982382499409	0.538981683630591	NA	NA	KXJ73801.1 hypothet	XP_001655285.2uncharacterized protein LOC5580145	
AAEL00637	STAM-binding protein	-0.244709699078125	0.312036606673709	0.743490640759055	NA	aag04144	XP_001844659.1 am	XP_021699581.1STAM-binding protein	
AAEL01951	NA	-0.244677748090405	0.0818079002634391	0.435494403400817	NA	NA	NA	XP_021708402.1uncharacterized protein LOC5576866 isoform X3	
AAEL02692	NA	-0.24464125487525	0.251402887223283	0.684238204149262	480	aag01100 ; aag	NA	XP_021712943.1aminopeptidase N	
AAEL00868	disks large-associated protein 1	-0.24441634607488	0.0396201870123403	0.313353264954028	NA	NA	KXJ78102.1 hypothet	XP_021701494.1serine-rich adhesin for platelets isoform X1	
AAEL00075	zinc finger HIT domain-containing protein 3	-0.244352503070331	0.477171589310613	0.845011493487133	NA	NA	ETN65251.1 hypothe	XP_001650999.1zinc finger HIT domain-containing protein 3	
AAEL02740	NA	-0.244063911703957	0.536458487414508	0.868144476914536	NA	NA	NA	XP_001653279.1isochorismatase domain-containing protein 1	
AAEL01022	protein daughterless	-0.243623933678226	0.316961657035887	0.745684464362801	NA	NA	NA	XP_021702880.1protein daughterless isoform X3	
AAEL00285	protein patched	-0.24294944809735	0.21293494083731	0.646249117936982	NA	aag04341	KXJ74359.1 hypothet	XP_001656017.2protein patched	
AAEL00906	zinc finger protein swm	-0.242754849897714	0.197478622800282	0.630533756768978	NA	NA	KXJ81077.1 hypothet	XP_021700745.1zinc finger protein swm	
AAEL00644	alpha,alpha-trehalose-phosphate synthase [UDP-forming]	-0.242643397447602	0.118308224076643	0.516203071575757	500	aag01100 ; aag	KFB45146.1 AGAP00	XP_001657813.1alpha,alpha-trehalose-phosphate synthase [UDP-forming]	
AAEL00192	DNA N6-methyl adenine demethylase	-0.242510936747033	0.164602756075585	0.583247700156998	NA	NA	XP_019552818.1 PRE	XP_021699256.1DNA N6-methyl adenine demethylase isoform X1	
AAEL01080	rabankyrin-5	-0.242304417261665	0.141428409397364	0.552330595273689	NA	NA	XP_019531081.1 PRE	XP_019531082.1 rabankyrin-5-like isoform X2	
AAEL01725	protein argonaute-2	-0.242173993902004	0.0433432101242082	0.328808095270374	NA	NA	NA	XP_011493002.2protein argonaute-2	
AAEL00690	uncharacterized LOC5568491	-0.241901067368562	0.0572874224058685	0.375202036283845	NA	NA	KXJ77639.1 hypothet	XP_001658082.1uncharacterized protein LOC5568491 isoform X1	
AAEL02585	NA	-0.24177026774544	0.251114782839715	0.683858606596939	NA	NA	NA	XP_021711994.1uncharacterized protein LOC110680504	
AAEL00110	ATP-binding cassette sub-family F member 1	-0.241375933989915	0.0324449941515021	0.28463170766102	NA	NA	XP_001847128.1 ATP	XP_001652415.2ATP-binding cassette sub-family F member 1	
AAEL02459	NA	-0.241374007618053	0.0554639097521192	0.37071620419176	NA	NA	NA	XP_021699215.1bromodomain-containing protein 4 isoform X2	
AAEL00816	fatty acid synthase	-0.241303025101858	0.0807572135623234	0.433079891352508	00780 ; 00061	aag01100 ; aag	KXJ74732.1 hypothet	XP_001659008.2fatty acid synthase	
AAEL00937	N-alpha-acetyltransferase 20	-0.241180903708606	0.197549859531371	0.630542423658575	NA	NA	NA	XP_001653814.1N-alpha-acetyltransferase 20	
AAEL00688	proteoglycan 4	-0.240821980339583	0.5474094137433	0.87195108624993	NA	NA	KXJ75568.1 hypothet	XP_021695998.1proteoglycan 4 isoform X2	
AAEL00070	NF-kappa-B inhibitor cactus	-0.240748416593326	0.0483962092578902	0.35027523096122	NA	aag04624	KXJ73211.1 hypothet	XP_001650267.3NF-kappa-B inhibitor cactus isoform X1	
AAEL01420	protein CBFA2T2	-0.240740412378565	0.492189737074656	0.852141102645529	NA	NA	NA	XP_021710919.1protein CBFA2T2 isoform X1	
AAEL00306	chitinase-3-like protein 1	-0.24062093754724	0.122267797345058	0.524358469989517	520	NA	XP_019543589.1 PRE	XP_001663097.2chitinase-3-like protein 1	
AAEL01192	E3 ubiquitin-protein ligase mind-bomb	-0.240483845824086	0.0601049083255746	0.38408706000829	NA	NA	XP_019527699.1 PRE	XP_001662068.2E3 ubiquitin-protein ligase mind-bomb isoform X1	
AAEL00360	uncharacterized LOC5578631	-0.240341414118456	0.160959223073279	0.578121300046961	NA	NA	NA	AAL76018.1putative 34 kDa secreted protein	
AAEL01820	NA	-0.240340016829073	0.276612906135313	0.711897116008604	NA	NA	NA	XP_021706007.1uncharacterized protein LOC5573983	
AAEL00670	leishmanolysin-like peptidase	-0.240127725293321	0.260856785050358	0.694041487911236	NA	NA	XP_019525156.1 PRE	XP_001657977.2 leishmanolysin-like peptidase	
AAEL02743	NA	-0.23994650709831	0.576569029310067	0.881961491587822	NA	NA	NA	XP_021708274.1uncharacterized protein LOC110678927	
AAEL01354	elongation of very long chain fatty acids protein AAEL008004-like	-0.23983557723767	0.0990797913846106	0.477596773347924	62	NA	NA	XP_001663734.2elongation of very long chain fatty acids protein AAEL008004-like	
AAEL00186	NADH dehydrogenase [ubiquinone] 1 beta subcomplex subunit 10	-0.239823125742012	0.0702371814826732	0.407517366963152	NA	aag01100 ; aag	XP_001654150.1 NAI	XP_001654150.1NADH dehydrogenase [ubiquinone] 1 beta subcomplex subunit 10	
AAEL01277	serine protease Hayan	-0.239743019292824	0.330309359945554	0.753845915232883	NA	NA	XP_019531205.1 PRE	XP_001662880.2serine protease Hayan	
AAEL00217	interferon-related developmental regulator 2	-0.239638125811481	0.106192402171521	0.490844601505817	NA	NA	KXJ71629.1 hypothet	XP_001654817.2interferon-related developmental regulator 2	
AAEL01349	NA	-0.239542127248687	0.215630353217411	0.648302652610268	NA	aag03010	NA	XP_021699379.139S ribosomal protein L16, mitochondrial	
AAEL00677	transmembrane protein 14 homolog	-0.239375123701209	0.211299134989702	0.643406565246605	NA	NA	ETN61392.1 membra	XP_001658052.2transmembrane protein 14 homolog	
AAEL00950	zinc finger E-box-binding homeobox 1	-0.239367393055968	0.382342893666077	0.788330564085871	NA	NA	NA	XP_001660167.1zinc finger E-box-binding homeobox 1	
AAEL00888	cell growth-regulating nucleolar protein	-0.239362223639062	0.122285728819551	0.524358469989517	NA	NA	KXJ70997.1 hypothet	XP_021697945.1cell growth-regulating nucleolar protein	
AAEL01073	sorting nexin-21	-0.239343837156019	0.10647025661562	0.491343681733668	NA	NA	XP_019533291.1 PRE	XP_001661060.2sorting nexin-21 isoform X2	
AAEL00981	uncharacterized LOC5572481	-0.239044113737462	0.252393987147249	0.684556086619159	NA	NA	NA	XP_019541145.1 integral membrane protein DGCR2/IDD-like	
AAEL01022	diamine acetyltransferase 2	-0.238990432444611	0.498349874574826	0.856678287334277	330	NA	NA	XP_021708606.1diamine acetyltransferase 2	
AAEL01107	uncharacterized LOC5574304	-0.238725330609773	0.0375145193636062	0.306307548521654	NA	NA	ETN63566.1 C-type le	ABF18196.1c-type lectin	
AAEL01131	actin-like protein 6A	-0.238560310538788	0.256996604474878	0.689671261119226	NA	NA	KXJ79033.1 hypothet	XP_001661589.1actin-like protein 6A	
AAEL00695	contactin	-0.238549918727836	0.0962531338950809	0.469629553606858	NA	NA	XP_019534137.1 PRE	XP_001652371.2contactin isoform X1	
AAEL02349	NA	-0.238495853845432	0.374344027578434	0.782198815065306	NA	NA	NA	XP_021699175.1mucin-2-like isoform X2	
AAEL00470	NA	-0.23821414756748	0.17235608690018	0.594934024371093	00250 ; 00220	aag01100 ; aag	KXJ80285.1 hypothet	XP_001649600.2argininosuccinate synthase	
AAEL01159	ADP-ribosylation factor-like protein 13B	-0.238206854197998	0.368229554247724	0.781648986894477	NA	NA	KXJ84334.1 hypothet	XP_021708319.1ADP-ribosylation factor-like protein 13B	
AAEL02045	NA	-0.238193166000957	0.220824825186582	0.652797615862164	NA	NA	NA	XP_021707413.1cytochrome c oxidase assembly factor 6 homolog	
AAEL02208	NA	-0.238141995533831	0.0961583357787842	0.469415918215582	NA	NA	NA	XP_021697597.1protein wings apart-like	
AAEL00978	zinc finger protein 853	-0.238000836339614	0.322693797178654	0.746650319748046	NA	NA	NA	XP_001660344.2zinc finger protein 853	
AAEL00424	uncharacterized LOC5564404	-0.237750623033851	0.0534740296484218	0.365578024386907	901	NA	NA	XP_021700208.1uncharacterized protein LOC5564404 isoform X1	
AAEL01399	protein Asterix	-0.237694140785611	0.181807025622596	0.607917241925556	NA	NA	NA	XP_001657257.2protein Asterix	
AAEL01364	FMRFamide-related peptides	-0.237660632032517	0.331517124924063	0.754128446928769	NA	NA	NA	XP_021699688.1FMRFamide-related peptides isoform X1	
AAEL02608	NA	-0.237648051969529	0.139134926128067	0.549029662748535	NA	NA	NA	XP_021702239.1putative ATPase N2B	
AAEL00054	fasciclin-3	-0.237627335863467	0.0645430612909496	0.396446323355255	NA	NA	XP_019539757.1 PRE	XP_001647979.1fasciclin-3 isoform X1	
AAEL00307	negative elongation factor E	-0.237535671085826	0.263926487496438	0.698501621589975	NA	NA	KXJ81123.1 hypothet	XP_021699593.1negative elongation factor E isoform X2	
AAEL01328	single-strand selective monofunctional uracil DNA glycosylase,tRNA-speci	-0.237321655234868	0.50924780374541	0.860083070787677	NA	aag03410	NA	XP_021694151.1single-strand selective monofunctional uracil DNA glycosylase	
AAEL01030	zinc finger and BTB domain-containing protein 41	-0.237289044086462	0.223285836939493	0.65560825511079	NA	NA	NA	XP_001654446.2zinc finger and BTB domain-containing protein 41	
AAEL00023	protein stoned-B	-0.237252785691082	0.168285423630628	0.588255870124114	NA	NA	KXJ77547.1 hypothet	XP_021694526.1protein stoned-B	
AAEL01117	polycomb group protein Psc	-0.2371623654132	0.238723553046133	0.673462389161024	NA	NA	XP_001865431.1 pos	XP_001661478.2polycomb group protein Psc	
AAEL02782	NA	-0.236890119427458	0.358481704531666	0.773854174836904	561	NA	NA	XP_021694873.1pancreatic lipase-related protein 2	
AAEL01545	transferrin	-0.236850429709695	0.227234779535812	0.661294889718073	NA	NA	NA	AAL58075.1transferrin precursor	
AAEL00109	uridine diphosphate glucose pyrophosphatase	-0.236827062330 89	0.108121501738715	0.494284290519081	NA	NA	KXJ81678.1 hypothet	XP_001658021.2uridine diphosphate glucose pyrophosphatase	
AAEL00102	probable nucleoporin Nup58	-0.236712433423464	0.470939210117888	0.841469450155131	NA	aag03013	XP_001848945.1 con	XP_001657871.2probable nucleoporin Nup58	
AAEL00800	lambda-crystallin homolog	-0.236635986698303	0.110745995978741	0.501764970456116	00650 ; 00071 ; 00627 ;	aag01100 ; aag	XP_019527207.1 PRE	XP_001658792.2lambda-crystallin homolog	
AAEL00221	proton-coupled amino acid transporter-like protein CG1139	-0.236546558951812	0.123966616059648	0.527832049142223	NA	NA	XP_019551350.1 PRE	XP_001654984.1proton-coupled amino acid transporter-like protein CG1139 isoform X1	
AAEL00546	uncharacterized LOC5566549	-0.236520441806477	0.150467581251368	0.566025396924239	NA	NA	NA	XP_021708844.1uncharacterized protein LOC5566549 isoform X1	

AAEL0089!	transcription factor kayak	-0.236480565186423	0.065499140844638	0.397837025777134	NA	aag04013 ; aag	XP_019558499.1 PRE	XP_001653617.1transcription factor kayak isoform X3
AAEL0033{	60 kDa SS-A/Ro ribonucleoprotein homolog	-0.236343658284678	0.227216209394555	0.661294889718073	NA	NA	KXJ83881.1 hypothet	XP_001656757.260 kDa SS-A/Ro ribonucleoprotein homolog
AAEL0194!	NA	-0.23629558507191	0.173969671456373	0.597209919209252	NA	NA	NA	XP_021711392.1plasma membrane calcium-transporting ATPase 2 isoform X1
AAEL0044:	39S ribosomal protein L14, mitochondrial	-0.236200813689521	0.163267814952972	0.581366301644056	NA	aag03010	KXJ69640.1 hypothet	XP_001649135.139S ribosomal protein L14, mitochondrial
AAEL0086(	melanization protease 1	-0.236076962658831	0.391751757968321	0.793285976044306	NA	NA	KXJ77534.1 hypothet	XP_021700244.1melanization protease 1
AAEL0136:	probable 4-coumarate--CoA ligase 3	-0.236025325852935	0.596486117872669	0.889585230151692	NA	NA	NA	XP_001663820.1probable 4-coumarate--CoA ligase 3
AAEL0004:	4-coumarate--CoA ligase-like 7	-0.235849823412743	0.108951264188187	0.496321551019654	NA	NA	KXJ80822.1 hypothet	XP_001656463.14-coumarate--CoA ligase-like 7
AAEL0141!	probable cardiolipin synthase (CMP-forming)	-0.235711041131658	0.0413349250562379	0.320441432491577	NA	aag01100 ; aag	NA	XP_001648191.1probable cardiolipin synthase (CMP-forming)
AAEL0107:	putative pre-mRNA-splicing factor ATP-dependent RNA helicase DHX16	-0.235561226012856	0.259524767099687	0.692216494739513	NA	aag03040	XP_001845160.1 pre	XP_001661061.2putative pre-mRNA-splicing factor ATP-dependent RNA helicase DHX16
AAEL0111!	cartilage-associated protein	-0.235550478004256	0.282077714667415	0.715554087745416	NA	NA	KXJ79620.1 hypothet	XP_001661444.1cartilage-associated protein
AAEL0086!	zinc transporter 2	-0.235487279702435	0.268526653157056	0.704184172798867	NA	NA	ETN60102.1 cation e	XP_021701493.1zinc transporter 2
AAEL0210:	NA	-0.235323990738538	0.0445050830697015	0.334042230348608	NA	NA	NA	XP_021708803.1uncharacterized protein LOC5566878
AAEL0003:	tyrosine-protein kinase Dnt	-0.235240084016446	0.399764948178773	0.798648539435638	NA	aag04310	KFB48191.1 tyrosine-	XP_021702281.1tyrosine-protein kinase Dnt
AAEL0094:	eukaryotic translation initiation factor 2D	-0.235166996086073	0.0702097305426641	0.407517366963152	NA	NA	NA	XP_001660028.1eukaryotic translation initiation factor 2D
AAEL0061!	E3 ubiquitin-protein ligase TRIM37	-0.23507516252331	0.305567163244298	0.738400482188558	NA	NA	KXJ79967.1 hypothet	XP_001657507.1E3 ubiquitin-protein ligase TRIM37
AAEL0034:	NADH dehydrogenase [ubiquinone] 1 beta subcomplex subunit 1	-0.23493614947927	0.0571751962647625	0.375057077651547	NA	aag01100 ; aag	KFB48930.1 AGAP00	XP_001656776.1NADH dehydrogenase [ubiquinone] 1 beta subcomplex subunit 1
AAEL0170:	fibrous sheath CABYR-binding protein	-0.23491168746174	0.0817526435286841	0.435494403400817	NA	NA	NA	XP_021701309.1fibrous sheath CABYR-binding protein isoform X2
AAEL0030:	uncharacterized LOC5576890	-0.234880620123084	0.179177649375203	0.604695248815902	NA	NA	KXJ70065.1 hypothet	XP_001662990.2uncharacterized protein LOC5576890 isoform X1
AAEL0250!	NA	-0.234871375072717	0.0655695682150411	0.397878167603183	NA	NA	NA	XP_021707602.1uncharacterized protein LOC5565521 isoform X1
AAEL0137!	cytochrome P450 4d1	-0.234851825070468	0.4710290304009	0.841469450155131	NA	NA	NA	XP_001663987.1cytochrome P450 4d1
AAEL0196!	NA	-0.234813547121734	0.107050792011822	0.492398491132955	NA	aag04144	NA	XP_021710894.1IQ motif and SEC7 domain-containing protein 1 isoform X5
AAEL0003:	lamin Dm0	-0.23470661106172	0.207493802439214	0.639832374753494	NA	NA	KXJ83455.1 hypothet	XP_001655561.2lamin Dm0
AAEL0106:	regulator of G-protein signaling 7	-0.234704115697856	0.318033598852206	0.745684464362801	NA	NA	KXJ76557.1 hypothet	XP_021698214.1regulator of G-protein signaling 7
AAEL0002{	syntaxin-17	-0.234570459501137	0.320196522969445	0.746314185502744	NA	aag04140 ; aag	KFB45055.1 AGAP00	XP_019550153.1 syntaxin-17-like
AAEL0229:	NA	-0.234497898800426	0.313971595664701	0.74451110779113	230	aag01100 ; aag	NA	XP_021705604.1adenylate cyclase type 8
AAEL0036:	neuropeptide SIFamide receptor	-0.234472618252678	0.478455786318722	0.845416382162313	NA	NA	XP_019527807.1 PRE	XP_021707558.1neuropeptide SIFamide receptor isoform X1
AAEL0104!	FK506-binding protein 59	-0.234331994208392	0.142117348604767	0.553975467550517	NA	NA	NA	XP_001654614.1FK506-binding protein 59 isoform X2
AAEL0045:	NA	-0.234325332869353	0.338167957148063	0.760464697379393	NA	NA	XP_003436441.1 AG/	XP_019534534.1 synapsin-like
AAEL0026{	UDP-glucuronosyltransferase 2B37	-0.23401333853476	0.0650555988577845	0.39730198438886	NA	aag01100 ; aag	KXJ76125.1 hypothet	XP_001662128.2UDP-glucuronosyltransferase 2B37
AAEL0143!	uncharacterized LOC110674005	-0.234003407470922	0.404751459854152	0.800800458735306	NA	NA	NA	XP_021700220.1uncharacterized protein LOC110674005 isoform X1
AAEL0258:	NA	-0.23393481369227	0.236487846084502	0.670870063357945	NA	NA	NA	XP_021705901.1protein TAPT1 homolog
AAEL0128!	uncharacterized LOC5576921	-0.233823261919046	0.363668533085935	0.778250660803901	NA	NA	NA	XP_001663018.1uncharacterized protein LOC5576921
AAEL0264:	NA	-0.233804348405019	0.200379984445407	0.633770569380376	04151 ; 05165 ; 04714 ;	NA	NA	XP_021699127.1citron Rho-interacting kinase
AAEL0248:	NA	-0.233678833427406	0.202756055078189	0.635808115244934	NA	NA	NA	NA
AAEL0151:	RNA polymerase II subunit A C-terminal domain phosphatase	-0.233647112665856	0.246446101266056	0.679999107601274	04660 ; 04658	NA	NA	XP_021697650.1RNA polymerase II subunit A C-terminal domain phosphatase
AAEL0041:	1,5-anhydro-D-fructose reductase	-0.233515558988608	0.0570576646636465	0.375057077651547	NA	aag01100 ; aag	KXJ83374.1 hypothet	XP_001648457.11,5-anhydro-D-fructose reductase
AAEL0050:	GTP-binding protein 2	-0.23349738678711	0.0796077395309916	0.431226849261068	NA	NA	KXJ83938.1 hypothet	XP_021693896.1GTP-binding protein 2
AAEL0203!	NA	-0.233478669228912	0.320039421313374	0.746140719770603	NA	NA	NA	XP_021695080.1uncharacterized protein LOC5571723 isoform X1
AAEL0198!	NA	-0.233430918563156	0.263797335865336	0.698501621589975	NA	NA	NA	XP_021697814.1nuclear hormone receptor FTZ-F1 isoform X2
AAEL0058{	sortilin-related receptor	-0.233286205967985	0.0549055186789984	0.368251153705644	NA	NA	KXJ78372.1 hypothet	XP_021706160.1sortilin-related receptor
AAEL0203:	NA	-0.233255896663228	0.713980272634904	0.919530646436164	NA	NA	NA	XP_021698581.1uncharacterized protein LOC110676147
AAEL0005:	C-type lectin 37Db	-0.232959267742175	0.114658087258133	0.50945616559601	NA	NA	AAV90642.1 putative	XP_001647974.1C-type lectin 37Db
AAEL0140{	retinal dehydrogenase 2	-0.232949959349588	0.107712656100394	0.493785323831772	NA	aag01100 ; aag	NA	XP_001648014.1retinal dehydrogenase 2
AAEL0012:	myosin-VIIa	-0.232778789563767	0.150506302449951	0.566025396924239	NA	NA	KXJ70961.1 hypothet	XP_019535868.1 myosin-VIIa
AAEL0011(	probable NADH dehydrogenase [ubiquinone] 1 alpha subcomplex subuni	-0.232579077503787	0.0709140723031428	0.409506895880945	NA	aag01100 ; aag	ETN57796.1 NADH de	XP_001658194.1probable NADH dehydrogenase [ubiquinone] 1 alpha subcomplex subunit 12
AAEL0134!	phenoloxidase 2	-0.232416878094363	0.0806971980995103	0.433079891352508	NA	NA	NA	XP_001663686.2phenoloxidase 2
AAEL0234!	NA	-0.231895808298597	0.458320461053121	0.833283524364043	NA	NA	NA	XP_001656212.2zinc finger protein 2
AAEL0148{	4-aminobutyrate aminotransferase, mitochondrial	-0.231878133466502	0.0821769187321042	0.436407518662845	00410 ; 00640 ; 00250 ;	aag01100 ; aag	NA	XP_021697848.14-aminobutyrate aminotransferase, mitochondrial
AAEL0205:	NA	-0.231801969255315	0.237432703837625	0.671758630247645	NA	NA	NA	XP_001661190.2phenoloxidase 2
AAEL0025!	tryptase-2	-0.231464942046706	0.097730349585297	0.47357276297204	NA	NA	ART33387.1 CLIPA14	XP_001655706.1tryptase-2
AAEL0084:	zinc finger protein 62 homolog	-0.231463561392839	0.43804950221463	0.821130886001024	NA	NA	KXJ78788.1 hypothet	XP_001659209.1zinc finger protein 62 homolog
AAEL0047:	uncharacterized LOC5565311	-0.231383629716475	0.271441163730756	0.706703671719641	NA	NA	NA	XP_001649648.2uncharacterized protein LOC5565311
AAEL0204:	NA	-0.231181353329622	0.345253863651507	0.764941296190615	NA	NA	NA	XP_021699003.1probable deoxyhypusine synthase
AAEL0256(	NA	-0.231180085306407	0.196689251744595	0.630345746481079	NA	NA	NA	XP_019933257.1 calponin homology domain-containing protein DDB_G0272472-like
AAEL0199:	NA	-0.231148628678197	0.0642523789185086	0.395485211242887	04151 ; 05165 ; 04714 ;	NA	NA	XP_021699475.1triple functional domain protein isoform X2
AAEL0043(	uncharacterized LOC5564497	-0.231054371896231	0.255087179528466	0.687152290989738	NA	NA	KXJ82124.1 hypothet	AAO74647.1antennae-specific protein
AAEL0090:	nucleolysin TIA-1	-0.230994195396943	0.256072362461165	0.688406212440425	NA	NA	ETN62803.1 ribonucl	XP_001659686.2nucleolysin TIA-1 isoform X2
AAEL0002!	zinc finger protein 490	-0.230963141040116	0.513034130308781	0.861297436611866	NA	NA	KXJ74462.1 hypothet	XP_001654769.2zinc finger protein 490
AAEL0086(	CD109 antigen	-0.230827059914702	0.0595949204435115	0.382421518773496	NA	NA	NA	XP_021702195.1CD109 antigen isoform X4
AAEL0021{	methyltransferase-like protein 13	-0.230527923886178	0.163527323593603	0.581632574875608	NA	NA	KXJ77270.1 hypothet	XP_001654970.1methyltransferase-like protein 13
AAEL0047:	NA	-0.230506484606396	0.52499338104626	0.865680248818465	NA	NA	CRL04351.1 CLUMA_	XP_001649657.2cyclin-dependent kinase 5 activator 1
AAEL0008:	dipeptidase 1	-0.230489759456052	0.678801414359525	0.914481546552978	NA	NA	KFB37702.1 AGAP01	XP_001651296.1dipeptidase 1
AAEL0087(	uncharacterized LOC5571032	-0.230312281193925	0.487736749856945	0.848846834445739	NA	NA	KXJ68612.1 hypothet	XP_001659469.1uncharacterized protein LOC5571032
AAEL0074(	xylosyltransferase oxt	-0.230185226996596	0.329637516359644	0.753059688565404	00534 ; 00532	aag01100 ; aag	KFB48255.1 AGAP00	XP_021695603.1xylosyltransferase oxt
AAEL0103{	UDP-glucuronosyltransferase 2A3	-0.230170911631589	0.267011958991745	0.703016894604303	NA	NA	NA	XP_021699050.1UDP-glucuronosyltransferase 2A3
AAEL0073!	tRNA pseudouridine synthase A, mitochondrial	-0.230139533853522	0.0965383059482699	0.470574140660778	NA	NA	KFB40881.1 AGAP00	XP_001658327.1tRNA pseudouridine synthase A, mitochondrial
AAEL0232:	NA	-0.229528610046476	0.25375651552646	0.685574708125215	NA	NA	NA	XP_021698840.1transcriptional repressor CTCF
AAEL0009{	NADH dehydrogenase [ubiquinone] 1 beta subcomplex subunit 8, mitoch	-0.229465297940693	0.0734557660279312	0.417762644616207	NA	aag01100 ; aag	ETN59783.1 NADH-u	XP_001657727.1NADH dehydrogenase [ubiquinone] 1 beta subcomplex subunit 8, mitochondrial
AAEL0092(	sushi, von Willebrand factor type A, EGF and pentraxin domain-containin	-0.229218424299522	0.0847919258715028	0.441820669235316	NA	NA	NA	XP_021700723.1sushi, von Willebrand factor type A, EGF and pentraxin domain-containing protein 1
AAEL0007:	excitatory amino acid transporter	-0.229183661549941	0.0520705238936351	0.361717594818022	NA	NA	XP_001843794.1 sod	XP_021703849.1excitatory amino acid transporter isoform X1
AAEL0083!	gamma-aminobutyric acid receptor subunit beta	-0.228920759270835	0.377066451978435	0.783395849675881	NA	aag04080	NA	XP_001653205.2gamma-aminobutyric acid receptor subunit beta isoform X6
AAEL0096:	ubiquitin-like protein 3	-0.228890402476752	0.434318238460079	0.820960220424486	NA	NA	NA	XP_001653895.2ubiquitin-like protein 3
AAEL0194:	NA	-0.228853795105012	0.16770789130336	0.587554508928878	4150	aag04310 ; aag	NA	XP_021698320.1 serine/threonine-protein kinase NLK
AAEL0180:	NA	-0.228673895135451	0.3186919520822	0.745684464362801	NA	NA	NA	XP_021706018.1zinc finger protein 431-like
AAEL0027!	N-acylneuraminate-9-phosphatase	-0.228626465629452	0.160754516656858	0.578015100029063	NA	aag01100 ; aag	KXJ84308.1 hypothet	XP_019532406.1 N-acylneuraminate-9-phosphatase-like
AAEL0103:	uncharacterized LOC5573222	-0.228553129318586	0.256242537030323	0.688461255220739	NA	NA	NA	XP_001654438.2uncharacterized protein LOC5573222
AAEL0182(	NA	-0.228093154821289	0.183721296274616	0.611208737642449	NA	NA	NA	XP_021704993.1uncharacterized protein LOC5578644
AAEL0071:	potassium voltage-gated channel protein Shaw	-0.227891576254206	0.424519783212595	0.814623464645531	NA	NA	XP_019538113.1 PRE	XP_021703150.1potassium voltage-gated channel protein Shaw isoform X1
AAEL0015{	zinc finger protein 99	-0.227446663586787	0.328298714037067	0.751306830780675	NA	NA	KXJ74004.1 hypothet	XP_021706844.1zinc finger protein 99
AAEL0096(	rab3 GTPase-activating protein regulatory subunit	-0.22740859383733	0.140092536691266	0.550440445189165	NA	NA	NA	XP_021709653.1rab3 GTPase-activating protein regulatory subunit isoform X1
AAEL0213(	NA	-0.227329844087019	0.370027818058733	0.781817769662763	NA	NA	NA	XP_021693884.1transcription factor grauzone isoform X1
AAEL0104(	gamma-aminobutyric acid type B receptor subunit 2	-0.227155182090798	0.488626568361126	0.848846834445739	NA	aag04080	NA	XP_021693447.1gamma-aminobutyric acid type B receptor subunit 2

AAEL0074	brachyurin	-0.226947325253684	0.169530326012228	0.589443594700505	NA	NA	KXJ70461.1 hypothe	ACV07660.1serine protease I
AAEL0114	titin	-0.226738446643326	0.0966592360952819	0.470612852142214	NA	NA	NA	XP_021705084.1titin isoform X1
AAEL0027	tropomyosin-2	-0.226586468349305	0.0667638042757935	0.399540230684495	NA	NA	XP_003435942.1 AG	XP_001655958.1tropomyosin-1, isoforms 9A/A/B isoform X34
AAEL0103	alpha-tocopherol transfer protein	-0.226471850014965	0.251032949792995	0.683858606596939	NA	NA	NA	XP_001654466.1alpha-tocopherol transfer protein
AAEL0043	uncharacterized family 31 glucosidase KIAA1161	-0.226367528533153	0.252821543622026	0.685060024855679	NA	NA	KXJ75464.1 hypothe	XP_021708954.1uncharacterized family 31 glucosidase KIAA1161
AAEL0097	lipoamide acyltransferase component of branched-chain alpha-keto acid	-0.226349422101504	0.308891931481787	0.74192156324557	NA	aag01100 ; aag	NA	XP_001660333.1lipoamide acyltransferase comp. of branched-chain alpha-keto acid dehydrogenase complex, mitochondrial
AAEL0029	sesquipedalian-1	-0.226275893073966	0.161148320791351	0.5783128882372	NA	NA	KXJ70506.1 hypothe	XP_019533625.1 sesquipedalian-1-like
AAEL0002	potassium voltage-gated channel protein Shaker	-0.226241435821065	0.352111369902742	0.769212685559608	NA	NA	NA	XP_021694514.1potassium voltage-gated channel protein Shaker isoform X1
AAEL0088	protein Wnt-1	-0.226181972624571	0.479695201275567	0.845454613425911	NA	NA	XP_001843055.1 win	XP_021700288.1protein Wnt-1
AAEL0151	pre-mRNA-processing factor 19	-0.226131533043763	0.281703443965341	0.715107379448722	NA	aag03040 ; aag	NA	XP_001650919.1pre-mRNA-processing factor 19
AAEL0096	trehalase	-0.225911761895595	0.0492815680245164	0.352961636078127	500	aag01100 ; aag	NA	XP_021705793.1trehalase isoform X1
AAEL0138	DDB1- and CUL4-associated factor 10 homolog	-0.225810703919562	0.275657792838412	0.711001645592863	NA	NA	NA	XP_001664094.1DDB1- and CUL4-associated factor 10 homolog isoform X2
AAEL0090	alpha-tocopherol transfer protein	-0.225775744175683	0.233358888733958	0.666962220912214	NA	NA	KFB46100.1 AGAP00	XP_001653647.2alpha-tocopherol transfer protein
AAEL0085	probable peroxisomal acyl-coenzyme A oxidase 1	-0.225298241825026	0.0706486929376409	0.40887375623407	00071 ; 00592	aag01100 ; aag	KFB49756.1 AGAP01	XP_001653317.1probable peroxisomal acyl-coenzyme A oxidase 1
AAEL0275	NA	-0.225229962210367	0.232710664709072	0.666707644289445	NA	NA	NA	XP_021709391.1uncharacterized protein LOC5570605
AAEL0001	NADH dehydrogenase [ubiquinone] 1 alpha subcomplex subunit 6	-0.225207624804054	0.0810907034123419	0.433079891352508	NA	aag01100 ; aag	KXJ83442.1 hypothe	XP_019528146.1 NADH dehydrogenase [ubiquinone] 1 alpha subcomplex subunit 6
AAEL0182	NA	-0.225007319187222	0.13957398669491	0.549341242757296	NA	NA	NA	XP_021713362.1dedicator of cytokinesis protein 3 isoform X3
AAEL0108	zinc finger and SCAN domain-containing protein 21	-0.224839018719044	0.459031900578804	0.833631645771888	NA	NA	KXJ68772.1 hypothe	XP_001654912.1zinc finger and SCAN domain-containing protein 21
AAEL0211	NA	-0.224698044153375	0.0572094690523859	0.375057077651547	NA	NA	NA	XP_021703870.1maternal protein tudor
AAEL0087	prostaglandin E synthase 2	-0.224662101392913	0.143208867993248	0.555375365647863	590	aag01100 ; aag	KXJ73625.1 hypothe	XP_001659483.1prostaglandin E synthase 2
AAEL0030	uncharacterized LOC5580307	-0.224547564716752	0.0613254526745534	0.386518366788521	NA	NA	NA	XP_001662925.2uncharacterized protein LOC5580307 isoform X2
AAEL0025	MICOS complex subunit Mic10	-0.224544978036245	0.0770546603917386	0.424839415772785	NA	NA	ETN66410.1 hypothe	XP_001655445.1MICOS complex subunit Mic10
AAEL0126	G patch domain and KOW motifs-containing protein	-0.224471844396451	0.397157624630412	0.797539006884967	NA	NA	KXJ76596.1 hypothe	XP_001656191.2G patch domain and KOW motifs-containing protein
AAEL0026	probable 4-coumarate--CoA ligase 1	-0.224358851299705	0.313208108617943	0.744189298893767	NA	NA	KXJ68503.1 hypothe	XP_001662062.1probable 4-coumarate--CoA ligase 1
AAEL0103	succinate dehydrogenase [ubiquinone] iron-sulfur subunit, mitochondria	-0.224351050086909	0.101400011682674	0.483635126937812	00650 ; 00020 ; 00720 ;	aag01100 ; aag	NA	XP_001660815.1succinate dehydrogenase [ubiquinone] iron-sulfur subunit, mitochondrial
AAEL0183	NA	-0.224304766456984	0.0584983355576104	0.37907043866014	NA	NA	NA	XP_021700566.1uncharacterized protein LOC5566438 isoform X2
AAEL0032	melanization protease 1	-0.223785598871072	0.293027836635605	0.723631099647605	NA	NA	XP_001842729.1 coa	XP_001656657.2melanization protease 1 isoform X1
AAEL0136	putative ribosome-binding factor A, mitochondrial	-0.223716108546441	0.233542793708545	0.667084333687587	NA	NA	NA	XP_001656879.1putative ribosome-binding factor A, mitochondrial
AAEL0278	NA	-0.223446695118882	0.096811544967446	0.470633908275356	NA	NA	NA	XP_021712418.1ubiquitin carboxyl-terminal hydrolase MINDY-3 homolog
AAEL0180	NA	-0.223423936203225	0.168844009388791	0.589193240195545	NA	NA	NA	XP_021706144.1serine/threonine-protein kinase minibrain isoform X2
AAEL0049	neuronal acetylcholine receptor subunit alpha-5	-0.223360690727294	0.449191522785164	0.829306073695304	NA	NA	KXJ76020.1 hypothe	XP_001650053.1neuronal acetylcholine receptor subunit alpha-5
AAEL0259	NA	-0.223144631674818	0.168055711078851	0.588003290246231	NA	NA	NA	XP_001654684.1coiled-coil domain-containing protein 25
AAEL0113	uncharacterized LOC5574731	-0.222797354206355	0.110093208839717	0.500079481483548	NA	aag04711	NA	XP_001661622.1uncharacterized protein LOC5574731
AAEL0134	deformed epidermal autoregulatory factor 1	-0.222535147385623	0.376281815812707	0.78320408710892	NA	NA	NA	XP_001663598.1deformed epidermal autoregulatory factor 1
AAEL0050	uncharacterized LOC5565861	-0.222479509604168	0.372714676905186	0.781817769662763	NA	NA	KXJ83952.1 hypothe	XP_019561276.1 multidrug resistance-associated protein 1-like
AAEL0005	long-chain fatty acid transport protein 4	-0.222357320601136	0.105828684218953	0.490374022320757	NA	NA	KXJ84416.1 hypothe	XP_021710053.1long-chain fatty acid transport protein 4 isoform X1
AAEL0083	myophilin	-0.222306466787066	0.0458825966694826	0.340510283013877	NA	NA	XP_001850083.1 cal	XP_019564906.1 myophilin-like
AAEL0142	uncharacterized LOC5563994	-0.222266255614009	0.32247532584084	0.746650319748046	NA	NA	NA	XP_001648314.1uncharacterized protein LOC5563994
AAEL0076	histone acetyltransferase KAT8	-0.222232922551927	0.254782904663863	0.686935918170778	NA	NA	XP_019536161.1 PRE	XP_001658578.2histone acetyltransferase KAT8 isoform X2
AAEL0125	DNA replication licensing factor Mcm6	-0.222156102704563	0.24785948799944	0.68058844480365	NA	aag03030	KXJ80194.1 hypothe	XP_001662645.1DNA replication licensing factor Mcm6
AAEL0290	NA	-0.22215507103147	0.181501870896532	0.607780282383509	NA	NA	NA	KFB46585.1AGAP009916-like protein
AAEL0007	transcription factor grauzone	-0.222077931333599	0.555379534761903	0.876039097680603	NA	NA	KXJ68223.1 hypothe	XP_001650982.2transcription factor grauzone
AAEL0014	zinc finger protein SNAI2	-0.221950739558478	0.546671495056328	0.871077246321788	NA	NA	KXJ79521.1 hypothe	XP_001659302.2zinc finger protein SNAI2 isoform X1
AAEL0058	protein big brother	-0.221879562455988	0.3214124224147	0.746588710355934	NA	NA	ETN65178.1 Big brot	XP_001651492.1protein big brother
AAEL0046	nicastrin	-0.221876823880206	0.193273327092042	0.624628157151667	NA	aag04330	XP_019532430.1 PRE	XP_001649511.2nicastrin isoform X1
AAEL0124	prostaglandin reductase 1	-0.22185117437923	0.391112033188288	0.793285976044306	NA	NA	XP_019551937.1 PRE	XP_021702748.1prostaglandin reductase 1 isoform X1
AAEL0048	ADP-ribosylation factor-like protein 6-interacting protein 4	-0.221790286985344	0.558764757595924	0.877581080322399	NA	NA	KXJ70198.1 hypothe	XP_021693797.1ADP-ribosylation factor-like protein 6-interacting protein 4
AAEL0048	M-phase phosphoprotein 6	-0.221647210126028	0.358432467229005	0.773854174836904	NA	aag03018	ETN62430.1 M-phase	XP_001649842.1M-phase phosphoprotein 6
AAEL0062	organic cation transporter protein	-0.221434622736324	0.283943241359742	0.716319042203869	NA	NA	KXJ81860.1 hypothe	XP_021700355.1organic cation transporter protein
AAEL0131	excitatory amino acid transporter 3	-0.221179728542661	0.358859643211986	0.77388983997572	NA	NA	NA	XP_001656354.2excitatory amino acid transporter 3
AAEL0062	uncharacterized LOC5567711	-0.221020781145691	0.325279862232462	0.748808663732339	NA	NA	KXJ84015.1 hypothe	XP_001651877.2uncharacterized protein LOC5567711 isoform X1
AAEL0137	exocyst complex component 3	-0.220916146481394	0.318279916994263	0.745684464362801	NA	NA	NA	XP_001663973.1exocyst complex component 3
AAEL0171	U6 snRNA-associated Sm-like protein LSm6	-0.22073052215701	0.28050177759652	0.714609510945122	NA	aag03040 ; aag	NA	XP_013112899.1 U6 snRNA-associated Sm-like protein LSm6
AAEL0121	aminopeptidase N	-0.220575018542912	0.24970666906451	0.682244883827679	480	NA	KFB52120.1 AGAP00	XP_001655857.2aminopeptidase N isoform X1
AAEL0227	NA	-0.220565992959277	0.269123860901328	0.705147428250005	NA	NA	NA	XP_021704034.1apoptosis-resistant E3 ubiquitin protein ligase 1 isoform X4
AAEL0228	NA	-0.220553271699092	0.233300764351918	0.666962220912214	NA	NA	NA	XP_021697520.1trafficking protein particle complex subunit 8 isoform X1
AAEL0125	putative transporter SVOPL	-0.220546639165843	0.222260997845981	0.654269258534458	NA	NA	KXJ81417.1 hypothe	XP_001662720.1putative transporter SVOPL
AAEL0255	NA	-0.220442302120888	0.39103663222448	0.793285976044306	NA	NA	NA	XP_021699093.1uncharacterized protein LOC5564044
AAEL0090	G1/S-specific cyclin-E	-0.220280114143966	0.48533145711657	0.847655581875201	NA	aag04391	KXJ81080.1 hypothe	XP_001659684.1G1/S-specific cyclin-E
AAEL0195	NA	-0.220222591333878	0.136828233835043	0.545589928345625	NA	NA	NA	XP_019532315.1 trichohyalin-like
AAEL0153	probable G-protein coupled receptor 158	-0.220049399821245	0.548283400598645	0.872889939845802	NA	NA	NA	XP_021701827.1probable G-protein coupled receptor 158 isoform X5
AAEL0129	somatostatin receptor type 2	-0.219380367337155	0.467083215368026	0.839144816051655	NA	NA	NA	XP_021705393.1somatostatin receptor type 2
AAEL0003	A-agglutinin anchorage subunit	-0.219366754219041	0.0750020319358305	0.420011717015769	NA	NA	NA	XP_001655555.2A-agglutinin anchorage subunit isoform X3
AAEL0051	cyclin-dependent kinase 10	-0.219119144306518	0.299190329672004	0.730496185008314	NA	NA	KFB48544.1 AGAP00	XP_001650496.2cyclin-dependent kinase 10
AAEL0222	NA	-0.219101284255381	0.462495303069136	0.83694823576051	NA	NA	NA	NA
AAEL0040	cytochrome P450 4g15	-0.219060355057456	0.157233189990042	0.574474189691589	NA	NA	ETN64031.1 cytochr	XP_001648376.1cytochrome P450 4g15
AAEL0033	metaxin-2	-0.219052379156436	0.276651172228512	0.711897116008604	NA	NA	KXJ75625.1 hypothe	XP_019558703.1 metaxin-2-like
AAEL0019	nucleolin 1	-0.21904393490171	0.167844871920772	0.58771252336946	NA	NA	KXJ69743.1 hypothe	XP_001660735.2nucleolin 1
AAEL0059	40S ribosomal protein S3a	-0.219007675367955	0.13343479242376	0.541865383885013	NA	aag03010	B0WN96.2 RecName	XP_001663344.140S ribosomal protein S3a
AAEL0118	1-acyl-sn-glycerol-3-phosphate acyltransferase alpha	-0.219005233649043	0.133955924853414	0.542662316284181	00561 ; 00564	NA	KFB40007.1 AGAP00	XP_021703720.11-acyl-sn-glycerol-3-phosphate acyltransferase alpha
AAEL0254	NA	-0.218849206549643	0.106168785375149	0.490844601505817	NA	NA	NA	XP_021706785.1multidrug resistance-associated protein 4
AAEL0146	acyl-CoA synthetase family member 2, mitochondrial	-0.218791105053891	0.274992364916825	0.710808916282197	NA	NA	NA	XP_021701900.1acyl-CoA synthetase family member 2, mitochondrial
AAEL0237	NA	-0.218636226373049	0.551809447558094	0.874531710113044	NA	NA	NA	XP_021696465.1uncharacterized protein LOC5570496
AAEL0026	equilibrative nucleoside transporter 4	-0.218544765594582	0.2868729392451	0.718895294966742	NA	NA	KXJ73242.1 hypothe	XP_021704362.1equilibrative nucleoside transporter 4 isoform X2
AAEL0180	NA	-0.218448647392757	0.476825418778555	0.844867278451347	NA	NA	NA	XP_021704393.1somatomedin-B and thrombospondin type-1 domain-containing protein
AAEL0279	NA	-0.218433580200438	0.179837781585821	0.605070298410504	NA	NA	NA	NA
AAEL0035	solute carrier family 26 member 6	-0.218336274321514	0.0779503485598074	0.42745012137719	NA	NA	XP_001851314.1 sul	XP_021710741.1solute carrier family 26 member 6
AAEL0097	uncharacterized LOC5572408	-0.218084389811122	0.613026877147875	0.895501122588404	NA	NA	NA	XP_021693583.1uncharacterized protein LOC5572408
AAEL0215	NA	-0.217966655560673	0.0763649642742567	0.422703978006619	NA	NA	NA	XP_021711640.1proteoglycan Cow isoform X2
AAEL0253	NA	-0.217969016163934	0.125773611985195	0.530481545423179	NA	NA	NA	XP_021704075.1uncharacterized protein PFB0145c isoform X3
AAEL0197	NA	-0.217764261952787	0.129590418421951	0.536192010035429	NA	NA	NA	XP_021706440.1 dystrophin, isoforms A/C/F/G/H
AAEL0148	NA	-0.217718203656056	0.0906104479898707	0.458582874647784	NA	NA	NA	XP_001649682.2probable cytochrome P450 4ac1

AAEL00641	leucine--tRNA ligase, cytoplasmic	-0.217545748249906	0.0777161234650344	0.427133790979399	970	aag00970	KXJ78242.1 hypothet	XP_001657794.2leucine--tRNA ligase, cytoplasmic
AAEL00768	zinc finger CCCH domain-containing protein 11A	-0.217464030489641	0.0752506010218241	0.420011717015769	NA	NA	ETN67151.1 hypothe	XP_021700481.1zinc finger CCCH domain-containing protein 11A
AAEL00125	uncharacterized LOC5569603	-0.217374805803071	0.0835670782013685	0.438667571938958	NA	NA	NA	XP_001652883.2uncharacterized protein LOC5569603 isoform X1
AAEL00953	protein twisted gastrulation	-0.217290817244596	0.22870831460203	0.662440639272231	NA	NA	NA	XP_001660194.1protein twisted gastrulation
AAEL00134	uncharacterized LOC5570084	-0.217187043251225	0.326533617903948	0.749317294751154	NA	NA	XP_001844467.1 con	XP_001653071.1uncharacterized protein LOC5570084 isoform X3
AAEL00191	pheromone-regulated protein PRM7	-0.217151409869382	0.165861670580737	0.584439853413553	NA	NA	XP_019541797.1 PRE	XP_001654314.2pheromone-regulated protein PRM7 isoform X1
AAEL00750	charged multivesicular body protein 3	-0.216856794013876	0.235627457224234	0.669872619304915	NA	aag04144	ABI83750.1 vacuolar	XP_001658413.1charged multivesicular body protein 3
AAEL00928	peptidyl-prolyl cis-trans isomerase CWC27 homolog	-0.216757726825142	0.410827332278775	0.805855841282375	NA	NA	NA	XP_001659892.2peptidyl-prolyl cis-trans isomerase CWC27 homolog
AAEL00704	far upstream element-binding protein 1	-0.216703055012301	0.0544695379270813	0.367469712613638	NA	NA	KXJ77928.1 hypothet	XP_021709749.1far upstream element-binding protein 1
AAEL02036	NA	-0.21666529936862	0.269892782609607	0.705956050475727	NA	NA	NA	XP_021713120.1serine protease 7
AAEL00730	alpha-actinin, sarcomeric	-0.216642744708536	0.0810638242056379	0.433079891352508	NA	NA	XP_003435853.1 AGA	XP_021708469.1alpha-actinin, sarcomeric isoform X1
AAEL00211	ras-like protein 2	-0.216544798697381	0.079647583247392	0.431226849261068	NA	aag04140 ; aag	ETN61853.1 ras [Ano	XP_001654734.1ras-like protein 2
AAEL01365	SPARC	-0.216526417831761	0.112096570040152	0.503547757511443	NA	NA	NA	XP_019535253.1 SPARC-like
AAEL00372	probable hydroxyacid-oxoacid transhydrogenase, mitochondrial	-0.216447873155879	0.203368419492981	0.636096599651398	NA	NA	XP_019554905.1 PRE	XP_001664064.2probable hydroxyacid-oxoacid transhydrogenase, mitochondrial
AAEL01118	uncharacterized LOC5574458	-0.216359205837256	0.119152606212715	0.518222042865395	NA	NA	ETN61198.1 hypothe	XP_001661472.1uncharacterized protein LOC5574458
AAEL01109	FAD synthase	-0.216097121391504	0.15553560116838	0.573412901422848	NA	aag01100 ; aag	XP_019540616.1 PRE	XP_001661422.1FAD synthase isoform X2
AAEL01729	probable cytochrome P450 6a14	-0.21601475040174	0.257613624037364	0.690722776337945	NA	NA	NA	XP_011492959.1probable cytochrome P450 6a14
AAEL00830	uncharacterized LOC5570404	-0.215798153783219	0.201528335096144	0.634701781910801	NA	NA	AAV90679.1 putative	AAL76011.1putative 7.8 kDa secreted protein
AAEL00405	nucleoporin NUP53	-0.215732060471216	0.328063747320107	0.750955871353141	NA	aag03013	XP_001851090.1 nuc	XP_001648370.1nucleoporin NUP53
AAEL02106	NA	-0.215706334557757	0.375076722240187	0.782198815065306	NA	aag04140 ; aag	NA	XP_021695105.1ubiquitin-like-conjugating enzyme ATG10
AAEL00165	structural maintenance of chromosomes protein 4	-0.215671376927743	0.282455008047626	0.715753918648043	NA	NA	XP_001842620.1 stru	XP_001653748.2structural maintenance of chromosomes protein 4
AAEL02769	NA	-0.215666110278978	0.30975409436686	0.742889924206485	NA	NA	NA	XP_021693808.1transmembrane protein 132C isoform X3
AAEL00803	uncharacterized protein F58A4.6	-0.215658536938418	0.474510377079474	0.844332076309675	NA	NA	NA	XP_021708097.1uncharacterized protein F58A4.6
AAEL02250	NA	-0.215647748198522	0.125427815419503	0.530481545423179	00780 ; 00061	NA	NA	XP_021703368.1fatty acid synthase
AAEL00360	uncharacterized LOC5578630	-0.215538097827453	0.152617846013808	0.568113296551786	NA	NA	KXJ72659.1 hypothet	ABF18170.1putative 34 kDa family secreted salivary protein
AAEL00874	probable very-long-chain enoyl-CoA reductase art-1	-0.215491392302168	0.068286508704578	0.401556773593974	NA	aag01100 ; aag	XP_001847824.1 syn	XP_001653426.2probable very-long-chain enoyl-CoA reductase art-1
AAEL02529	NA	-0.215437941106133	0.420659270835458	0.813636239562413	NA	NA	NA	XP_021693867.1putative mediator of RNA polymerase II transcription subunit 26 isoform X6
AAEL00402	carboxylesterase 1F	-0.21516401612513	0.158587170807344	0.576519055919795	NA	NA	KXJ68641.1 hypothet	XP_001648280.1carboxylesterase 1F
AAEL00254	dipeptidase 2	-0.215029290967705	0.36472321839251	0.778761496713185	NA	NA	KXJ69809.1 hypothet	XP_001655617.2dipeptidase 2
AAEL00027	NA	-0.214854763584014	0.0887173617589288	0.455313532016544	NA	aag04070	NA	XP_021706099.1uncharacterized protein LOC5573750
AAEL00116	synaptotagmin-1	-0.214760623118775	0.430460521280301	0.819254956943399	NA	NA	KXJ69349.1 hypothet	XP_019932833.1 synaptotagmin-2-like
AAEL00790	uncharacterized LOC5569758	-0.214742459155062	0.379658705868204	0.785755940613825	NA	NA	NA	XP_021704072.1uncharacterized protein LOC5569758
AAEL00605	uncharacterized LOC5567389	-0.214577642710539	0.500160016431164	0.858029916331016	NA	NA	NA	XP_001659034.2myb-like protein AA isoform X1
AAEL01186	2-(3-amino-3-carboxypropyl)histidine synthase subunit 1	-0.214502860084418	0.0911503216433879	0.459220287049195	NA	NA	XP_001849514.1 con	XP_021704121.12-(3-amino-3-carboxypropyl)histidine synthase subunit 1
AAEL02136	NA	-0.214400719662517	0.453625914319408	0.832617462606238	NA	NA	NA	XP_021698687.1PAX-interacting protein 1
AAEL00810	kelch-like protein 5	-0.214358259822939	0.394626828373131	0.794957110501207	NA	NA	XP_001862213.1 acti	XP_001653119.2kelch-like protein 5
AAEL01204	sodium-independent sulfate anion transporter	-0.214283553128736	0.344731708380311	0.764706052029763	NA	NA	XP_019556156.1 PRE	XP_021713112.1sodium-independent sulfate anion transporter isoform X2
AAEL00403	uncharacterized LOC5563953	-0.214024934500666	0.528208309986656	0.865973876319045	NA	NA	NA	XP_001648304.2uncharacterized protein LOC5563953
AAEL00001	peptidyl-prolyl cis-trans isomerase D	-0.213967928177926	0.370249693481091	0.781817769662763	NA	NA	KXJ75457.1 hypothet	XP_021706246.1peptidyl-prolyl cis-trans isomerase D
AAEL02113	NA	-0.213880273377441	0.373093501314117	0.782051571547268	NA	NA	NA	XP_021712563.1protein hu-li tai shao-like isoform X2
AAEL00335	2-aminoethanethiol dioxygenase	-0.21368465945813	0.267931045041889	0.704026692311667	NA	aag01100 ; aag	KXJ76818.1 hypothet	XP_021697701.12-aminoethanethiol dioxygenase
AAEL00185	mitochondrial import inner membrane translocase subunit Tim23	-0.213566151410538	0.137945242744051	0.547441354014568	NA	NA	KFB48128.1 AGAP01(	XP_001654138.1mitochondrial import inner membrane translocase subunit Tim23
AAEL01277	cytochrome P450 4C1	-0.213553267560776	0.135008510078721	0.543359475148491	NA	NA	XP_019543098.1 PRE	XP_001662865.3cytochrome P450 4C1
AAEL02802	NA	-0.213198082551782	0.455438775229048	0.832617462606238	NA	aag03022	NA	ETN58564.1wd-repeat protein
AAEL02316	NA	-0.213121695393199	0.174040453169763	0.597209919209252	NA	NA	NA	XP_021703263.1jerky protein homolog-like
AAEL02456	NA	-0.212965697671196	0.174580878185713	0.598225197379102	NA	NA	NA	XP_021709988.1dendritic arbor reduction protein 1
AAEL00205	sodium/hydrogen exchanger 7	-0.212888447294009	0.190279682485814	0.618521820583848	NA	NA	NA	XP_021702079.1sodium/hydrogen exchanger 7 isoform X11
AAEL00401	eukaryotic translation initiation factor 4H	-0.21249241721956	0.0689038812173194	0.403354246998601	NA	NA	NA	XP_001648291.2eukaryotic translation initiation factor 4H isoform X3
AAEL01181	DNA replication licensing factor Mcm3	-0.212350345308143	0.285310698907378	0.717970179170389	NA	aag03030	KXJ80244.1 hypothet	XP_001661938.2DNA replication licensing factor Mcm3
AAEL01130	uridine 5'-monophosphate synthase	-0.212282652946257	0.076206816665878	0.422442847565909	00240 ; 00983 ; 00240	aag01100 ; aag	KXJ80255.1 hypothet	XP_001655239.2uridine 5'-monophosphate synthase
AAEL02745	NA	-0.211798998549432	0.319374665454541	0.745684464362801	00240 ; 00983 ; 00230	NA	NA	XP_021699336.1nucleoside diphosphate kinase-like
AAEL02328	NA	-0.211789917603778	0.407742185108597	0.803607536382376	NA	NA	NA	XP_021699857.1coiled-coil domain-containing protein AGAP005037 isoform X3
AAEL00560	trypsin	-0.211712262650507	0.381325699987469	0.787361680182234	NA	NA	XP_001847488.1 tryp	XP_001651142.1trypsin epsilon
AAEL02354	NA	-0.211699902862332	0.135254078988815	0.543887965700835	NA	NA	NA	XP_021694041.1dedicator of cytokinesis protein 1 isoform X1
AAEL01402	transcriptional coactivator yorkie	-0.211658511834093	0.12691733393	0.531343633677825	NA	aag04391 ; aag	NA	XP_001664241.2transcriptional coactivator yorkie isoform X1
AAEL01976	NA	-0.211422098925437	0.414234839915666	0.80872375702921	NA	NA	NA	XP_021703917.1zinc finger protein 709 isoform X1
AAEL00024	protocadherin-like wing polarity protein stan	-0.211275147736332	0.371837438205672	0.781817769662763	NA	NA	XP_019547035.1 PRE	XP_001660147.3protocadherin-like wing polarity protein stan isoform X1
AAEL01453	AN1-type zinc finger protein 2A	-0.211222734571948	0.358253033978439	0.773854174836904	NA	NA	NA	XP_001648956.2AN1-type zinc finger protein 2A
AAEL00458	uncharacterized LOC5565111	-0.211089491773849	0.279856554200188	0.713956199542592	NA	NA	XP_001849366.1 con	XP_001649458.1uncharacterized protein LOC5565111
AAEL01437	AP-2 complex subunit sigma	-0.211017051711787	0.112439926941372	0.503547757511443	NA	aag04144	NA	XP_001648627.1AP-2 complex subunit sigma
AAEL02328	NA	-0.210916117651913	0.513993557648699	0.861448202130317	NA	NA	NA	XP_021707621.1E3 ubiquitin-protein ligase TRIM37-like isoform X2
AAEL00185	protein sly1 homolog	-0.210875800059674	0.178862301249207	0.604350349136232	NA	NA	ETN67778.1 vesicle p	XP_001654152.1protein sly1 homolog
AAEL00589	heat shock protein 67B2	-0.210540839925565	0.169329589096637	0.589323327862049	NA	NA	KXJ75600.1 hypothet	XP_001651544.1heat shock protein 67B2
AAEL00595	lipid storage droplets surface-binding protein 1	-0.210539648971777	0.117694980601585	0.515237493575542	NA	NA	XP_019543181.1 PRE	XP_021693333.1lipid storage droplets surface-binding protein 1 isoform X1
AAEL00164	alkaline ceramidase	-0.210537351772988	0.349859874028367	0.767806000669934	NA	aag01100 ; aag	KXJ80726.1 hypothet	XP_021701211.1 alkaline ceramidase
AAEL00499	methyltransferase-like 26	-0.210535772827529	0.364267984878181	0.77844728212936	NA	NA	KXJ79348.1 hypothet	XP_001650144.1methyltransferase-like 26
AAEL01452	neuroendocrine convertase 2	-0.210258675368138	0.12704687011127	0.531526770119611	NA	NA	NA	XP_001648949.2neuroendocrine convertase 2
AAEL00121	WD repeat and FYVE domain-containing protein 2	-0.210247404709044	0.138602461923055	0.548805616375239	NA	NA	ETN67382.1 phospho	XP_001658314.1WD repeat and FYVE domain-containing protein 2
AAEL00657	troponin C	-0.210175798314431	0.107175370326675	0.492398491132955	NA	NA	XP_019553261.1 PRE	XP_001652071.1troponin C isoform X3
AAEL00487	F-box only protein 11	-0.21002787704907	0.157226634364528	0.574474189691589	NA	NA	KXJ80334.1 hypothet	XP_021693801.1F-box only protein 11
AAEL00207	UV excision repair protein RAD23 homolog B	-0.20944010386474	0.0648819540330198	0.39730198438886	NA	aag04141 ; aag	KFB36802.1 AGAP00	XP_001654581.2UV excision repair protein RAD23 homolog B
AAEL00961	uncharacterized LOC5572210	-0.209366411982286	0.528930994200581	0.865973876319045	NA	NA	NA	XP_001653878.2uncharacterized protein LOC5572210
AAEL00355	putative uncharacterized protein DDB_G0279653	-0.209115165753003	0.167957059891045	0.587881652764321	NA	NA	NA	XP_001663877.1probable serine/threonine-protein kinase tsuA isoform X3
AAEL01428	zinc finger protein 330 homolog	-0.209112282520583	0.208084853841851	0.639832374753494	NA	NA	NA	XP_001648347.1zinc finger protein 330 homolog
AAEL00101	vacuolar protein sorting-associated protein 45	-0.209090107020721	0.337916191847801	0.760148569536312	NA	aag04144	KXJ68093.1 hypothet	XP_001657872.2vacuolar protein sorting-associated protein 45
AAEL02063	NA	-0.209086701645277	0.117893201783777	0.515795660238079	NA	NA	NA	XP_021713077.1kinesin-like protein Klp98A isoform X1
AAEL00560	bifunctional 3'-phosphoadenosine 5'-phosphosulfate synthase	-0.208791251800759	0.0938415163010997	0.464823706598816	00920 ; 00230 ; 00920 ;	aag01100 ; aag	XP_019529902.1 PRE	XP_021705883.1bifunctional 3'-phosphoadenosine 5'-phosphosulfate synthase isoform X2
AAEL00754	H(+)/Cl(-) exchange transporter 7	-0.208608426219167	0.0798331637544626	0.431435389689367	NA	NA	XP_019538572.1 PRE	XP_021695732.1H(+)/Cl(-) exchange transporter 7 isoform X1
AAEL01118	phosphate carrier protein, mitochondrial	-0.208487308207761	0.184672239171682	0.612043911074917	NA	NA	ETN64196.1 mitocho	XP_021708166.1phosphate carrier protein, mitochondrial
AAEL01943	NA	-0.208457983101468	0.423941816106276	0.814428516035481	NA	NA	NA	XP_019527987.1 pinin-like isoform X6
AAEL01942	NA	-0.208436204899301	0.440638149579103	0.822798752521693	NA	NA	NA	XP_021697735.1basic proline-rich protein
AAEL00092	eIF-2-alpha kinase GCN2	-0.208291657698277	0.310834066222237	0.742918550320373	04151 ; 05165 ; 04714 ;	aag04141 ; aag	KFB49982.1 eukaryot	XP_001651607.1eIF-2-alpha kinase GCN2

AAEL00498	putative beta-carotene-binding protein	-0.208270456925935	0.0873490710238133	0.451819084632451	NA	NA	KXJ76538.1 hypothet	XP_001650121.1putative beta-carotene-binding protein isoform X1
AAEL00589	beta-amyloid-like protein	-0.20826447099886	0.100440311500046	0.480630133345515	NA	NA	NA	XP_021709738.1beta-amyloid-like protein isoform X1
AAEL00481	serine-rich adhesin for platelets	-0.208206231743357	0.500160016426595	0.858029916331016	NA	NA	XP_001846079.1 con	XP_001649858.2uncharacterized protein LOC5565485
AAEL00847	putative glycerol kinase 5	-0.207966957014321	0.458387437549531	0.833283524364043	561	NA	KXJ76915.1 hypothet	XP_001659270.2putative glycerol kinase 5
AAEL02601	NA	-0.207904500590025	0.0907234991091934	0.458582874647784	NA	NA	NA	XP_021708592.1UPF0692 protein CG33108 isoform X1
AAEL00874	tetratricopeptide repeat protein 4	-0.207894730735915	0.345465870556374	0.764941296190615	NA	NA	KXJ68246.1 hypothet	XP_001653434.1tetratricopeptide repeat protein 4
AAEL00194	four and a half LIM domains protein 2	-0.207824056121526	0.0713303320133147	0.410495131448731	NA	NA	KFB47014.1 AGAP00	XP_001654345.1four and a half LIM domains protein 2 isoform X2
AAEL01291	sodium/potassium-transporting ATPase subunit beta-1-interacting protei	-0.20765028690961	0.324087212015865	0.747806049390669	NA	NA	NA	XP_021698661.1sodium/potassium-transporting ATPase subunit beta-1-interacting protein isoform X1
AAEL00823	NA	-0.207611917101143	0.126479533430621	0.530481545423179	NA	NA	XP_019525751.1 PRE	XP_001659064.2facilitated trehalose transporter Tret1-2 homolog isoform X1
AAEL01749	carboxypeptidase D	-0.207611869611234	0.0640060453491364	0.395036706780325	NA	NA	NA	XP_011493731.1carboxypeptidase D isoform X1
AAEL02536	NA	-0.207496558879523	0.16247914018425	0.580859769998241	NA	NA	NA	NA
AAEL02602	NA	-0.207400370558412	0.305984137449265	0.738783696372872	NA	NA	NA	XP_021713221.1uncharacterized protein LOC5577062
AAEL00884	peroxisomal acyl-coenzyme A oxidase 3	-0.207282829194431	0.20917201969485	0.639832374753494	00071 ; 00592	aag01100 ; aag	XP_019559471.1 PRE	XP_001653457.2peroxisomal acyl-coenzyme A oxidase 3 isoform X1
AAEL00455	putative OPA3-like protein CG13603	-0.207171654119228	0.254476035350559	0.686571057876479	NA	NA	XP_019562546.1 PRE	XP_001649397.1putative OPA3-like protein CG13603 isoform X2
AAEL01955	NA	-0.207115642267492	0.391519975975405	0.793285976044306	NA	NA	NA	XP_019545581.1 zinc finger protein 384-like
AAEL02769	NA	-0.20704556422264	0.213327209322482	0.646373717545434	NA	NA	NA	XP_021708096.1uncharacterized protein LOC5564265 isoform X2
AAEL01089	uncharacterized LOC5574056	-0.206725564867226	0.291494997353402	0.722646576583698	NA	NA	KXJ71695.1 hypothet	XP_021713279.1uncharacterized protein LOC5574056
AAEL00037	peroxisomal acyl-coenzyme A oxidase 3	-0.206724494777705	0.170450710967157	0.591212756245676	00071 ; 00592	aag01100 ; aag	KFB47956.1 AGAP00	XP_001655562.1peroxisomal acyl-coenzyme A oxidase 3
AAEL01978	NA	-0.206717235946946	0.364126142089987	0.77844728212936	NA	NA	NA	XP_021712939.1uncharacterized protein LOC5567079 isoform X2
AAEL00259	tryptase	-0.206664114679217	0.0809107603547832	0.433079891352508	NA	NA	KFB49742.1 AGAP01	XP_019531773.1 tryptase-like isoform X1
AAEL01979	NA	-0.206396679317085	0.12916840726503	0.53537965541735	NA	NA	NA	XP_021706263.1muscle LIM protein 1 isoform X2
AAEL01186	uncharacterized LOC5575489	-0.206101038785714	0.425751593123715	0.814623464645531	NA	NA	KXJ76187.1 hypothet	XP_001662006.2uncharacterized protein LOC5575489
AAEL00005	general odorant-binding protein 72	-0.206060502318945	0.562226573439183	0.87919672735184	NA	NA	ACR43440.1 odorant	XP_001647922.2general odorant-binding protein 72
AAEL00920	solute carrier family 22 member 4	-0.206014113623539	0.366979348853818	0.780578423384551	NA	NA	NA	XP_001659801.1solute carrier family 22 member 4
AAEL00680	sodium leak channel non-selective protein	-0.205806797740643	0.150487654645277	0.566025396924239	NA	NA	KXJ71397.1 hypothet	XP_001652236.1sodium leak channel non-selective protein isoform X1
AAEL01948	NA	-0.205712599602714	0.506175779497924	0.858744381072989	NA	NA	NA	XP_021710994.1hybrid signal transduction histidine kinase A isoform X2
AAEL01364	fatty acyl-CoA reductase wat	-0.205603882261601	0.275990538812632	0.711135357225659	73	aag04146	NA	XP_001663834.1fatty acyl-CoA reductase wat
AAEL00120	putative inorganic phosphate cotransporter	-0.205542470411749	0.102488895654412	0.484636597025644	NA	NA	XP_019535875.1 PRE	XP_001658313.1putative inorganic phosphate cotransporter isoform X2
AAEL00146	lysoplasmalogenase-like protein TMEM86A	-0.205509541940637	0.421251021253471	0.81401761813827	NA	NA	XP_019531939.1 PRE	XP_021701068.1lysoplasmalogenase-like protein TMEM86A isoform X1
AAEL00701	sodium-coupled monocarboxylate transporter 1	-0.205462475766589	0.307729846721486	0.740321581576761	NA	NA	XP_001844203.1 sod	XP_001652484.1sodium-coupled monocarboxylate transporter 1
AAEL01389	26S proteasome non-ATPase regulatory subunit 9	-0.20542843022266	0.229828298664078	0.662910167685841	NA	NA	NA	XP_001664093.226S proteasome non-ATPase regulatory subunit 9
AAEL02471	NA	-0.205411344391906	0.241408336326783	0.676649257045098	330	NA	NA	XP_001660440.2pyrroline-5-carboxylate reductase 1, mitochondrial
AAEL01397	uncharacterized LOC5579040	-0.205136673109064	0.408122182627117	0.8040930806342	NA	NA	NA	XP_001664217.2uncharacterized protein LOC5579040
AAEL01372	protein aveugle	-0.205117905481487	0.484460667751073	0.847655581875201	NA	NA	NA	XP_001663916.1protein aveugle
AAEL01978	NA	-0.205032451777044	0.522318884730962	0.865018698932415	NA	NA	NA	XP_021694765.1protein ovo isoform X2
AAEL02350	NA	-0.204884039569582	0.557933650478347	0.877474867834857	NA	aag03022	NA	XP_001649759.1transcription initiation factor TFIID subunit 11
AAEL00653	acetylcholine receptor subunit alpha-like 1	-0.204811431912074	0.168295163860783	0.588255870124114	NA	NA	NA	XP_001652010.1acetylcholine receptor subunit alpha-like 1 isoform X2
AAEL00364	sodium-dependent nutrient amino acid transporter 1	-0.204764222038557	0.425864706540139	0.814623464645531	NA	NA	KXJ80826.1 hypothet	XP_021705242.1sodium-dependent nutrient amino acid transporter 1
AAEL00000	WD and tetratricopeptide repeats protein 1	-0.204671279326876	0.193907494279398	0.624986606780741	NA	NA	XP_019538005.1 PRE	XP_021704841.1WD and tetratricopeptide repeats protein 1
AAEL00944	activating transcription factor 3	-0.204628867573411	0.490209834945223	0.850081180289834	NA	NA	NA	XP_001660056.1activating transcription factor 3
AAEL01006	protein disulfide-isomerase A6 homolog	-0.204518194179963	0.082902118501321	0.437924968110882	NA	aag04141	NA	XP_001660597.1protein disulfide-isomerase A6 homolog
AAEL02182	NA	-0.204353354433795	0.177271299882283	0.602182613972581	NA	NA	NA	XP_021702448.1probable nuclear hormone receptor HR38 isoform X2
AAEL00651	sialin	-0.204106132163413	0.0764061252603873	0.422703978006619	NA	NA	XP_019533077.1 PRE	XP_021703998.1sialin isoform X1
AAEL00109	heat shock protein beta-1	-0.204050250276232	0.0790299074546	0.430061033942773	NA	NA	XP_019562135.1 PRE	XP_001657982.1heat shock protein beta-1 isoform X4
AAEL02045	NA	-0.203822296110873	0.562861519968435	0.879512940524628	NA	NA	NA	XP_021696886.1uncharacterized protein LOC110675641
AAEL01990	NA	-0.203796903473281	0.34771384250887	0.766202772693158	NA	NA	NA	XP_021705592.1small G protein signaling modulator 2 isoform X1
AAEL01951	NA	-0.203755412502633	0.111756477661852	0.503122851000177	230	aag04391	NA	XP_021704373.1MAGUK p55 subfamily member 5-A isoform X4
AAEL00600	EF-hand domain-containing protein D2 homolog	-0.203603534969722	0.220490778736545	0.652412844728166	NA	NA	KFB39821.1 AGAP00	XP_001651772.1EF-hand domain-containing protein D2 homolog
AAEL01997	NA	-0.203321070893801	0.297513136212126	0.729668411360336	NA	NA	NA	XP_021695396.1 DDB1- and CUL4-associated factor 5
AAEL00753	uncharacterized LOC5569294	-0.203227329671248	0.1241008593909	0.527832049142223	NA	NA	NA	XP_001658426.2uncharacterized protein LOC5569294 isoform X3
AAEL01088	protein Aatf	-0.203162218683856	0.266149551689082	0.701549176351456	NA	NA	KXJ71691.1 hypothet	XP_001655038.2protein Aatf
AAEL00267	ras suppressor protein 1	-0.203116914628806	0.189926701977699	0.618437902193483	NA	NA	ETN58470.1 ras supp	XP_001662051.2ras suppressor protein 1
AAEL00244	CLK4-associating serine/arginine rich protein	-0.202954591682756	0.258698592074707	0.691547675112889	NA	NA	KXJ75645.1 hypothet	XP_021704229.1CLK4-associating serine/arginine rich protein
AAEL00868	ubiquitin-related modifier 1 homolog	-0.202798376374745	0.0953413604486466	0.466913889754362	NA	aag04122	ETN67257.1 Ubiquiti	XP_001653419.1ubiquitin-related modifier 1 homolog isoform X1
AAEL00867	alpha-tubulin N-acetyltransferase	-0.202511481912125	0.108625748490122	0.495725018486141	NA	NA	ETN67261.1 hypothe	XP_001653412.2alpha-tubulin N-acetyltransferase
AAEL00098	NEDD4 family-interacting protein 1	-0.202327353240346	0.11355402920452	0.506016550479416	NA	NA	KXJ84284.1 hypothet	XP_001657712.2NEDD4 family-interacting protein 1
AAEL01168	luc7-like protein 3	-0.202291250368023	0.318744179910433	0.745684464362801	NA	NA	KXJ72207.1 hypothet	XP_001655621.1luc7-like protein 3
AAEL02226	NA	-0.202232180318248	0.498720450472693	0.856678287334277	NA	NA	NA	XP_019532768.1 cuticle protein 19-like
AAEL00932	putative carbonic anhydrase 3	-0.202176286346326	0.17733601481812	0.602182613972581	910	aag01100 ; aag	NA	XP_021709524.1putative carbonic anhydrase 3
AAEL01399	hexamerin-1.1	-0.202120446183195	0.195711690184884	0.628380660530809	NA	NA	NA	XP_019531188.1 hexamerin-1.1-like
AAEL00845	TATA element modulatory factor	-0.202119056926409	0.261199946345471	0.694470357200527	NA	NA	XP_001861895.1 con	XP_021706741.1TATA element modulatory factor
AAEL00491	brain tumor protein	-0.20208451171552	0.35241645376739	0.76935039265884	NA	NA	ETN63087.1 brat pro	XP_001650044.1brain tumor protein
AAEL02137	NA	-0.201982959196856	0.0904559735564008	0.458582874647784	NA	NA	NA	XP_021698831.1myrosinase 1-like
AAEL00359	kinesin-like protein KIN-7O	-0.20193451643237	0.187301764054545	0.614237645342095	NA	NA	XP_019528763.1 PRE	XP_021697940.1kinesin-like protein KIN-7O
AAEL02219	NA	-0.201917600258661	0.223499695842851	0.655920215788323	NA	NA	NA	XP_021697648.1glutamate receptor ionotropic, kainate 2 isoform X1
AAEL02101	NA	-0.201897822031117	0.212520645870702	0.645843785766909	NA	NA	NA	XP_021699077.1probable G-protein coupled receptor Mth-like 10 isoform X3
AAEL00328	protein-associating with the carboxyl-terminal domain of ezrin	-0.201772393796571	0.377494329102287	0.783777711281418	NA	NA	XP_019543635.1 PRE	XP_001656615.2protein-associating with the carboxyl-terminal domain of ezrin
AAEL02756	NA	-0.201765812693965	0.595423595535712	0.889050526332724	NA	aag04144 ; aag	NA	XP_021703577.1TGF-beta receptor type-1 isoform X1
AAEL02061	NA	-0.201751150250326	0.105833819640189	0.490374022320757	NA	NA	NA	XP_021694319.1protein gone early
AAEL02643	NA	-0.201682838639605	0.451309563076511	0.830666499866364	NA	NA	NA	XP_021702447.1fibrous sheath CABYR-binding protein
AAEL01349	retinol dehydrogenase 13	-0.201525456778257	0.102370420749741	0.484636597025644	NA	NA	NA	XP_001663676.1retinol dehydrogenase 13
AAEL01086	mediator of RNA polymerase II transcription subunit 8	-0.200750281915108	0.528537247055178	0.865973876319045	NA	NA	ETN64863.1 Mediatc	XP_001661113.1mediator of RNA polymerase II transcription subunit 8
AAEL00686	uncharacterized LOC5568450	-0.200739897743265	0.0993080963322529	0.478084745672	NA	NA	XP_001847369.1 con	XP_021706376.1uncharacterized protein LOC5568450
AAEL00430	uncharacterized LOC5564562	-0.200724882707411	0.336669474237571	0.758445425575872	NA	NA	KXJ81976.1 hypothet	XP_001648884.1uncharacterized protein LOC5564562
AAEL01170	NAD kinase 2, mitochondrial	-0.200638161529963	0.216181506572744	0.649374960388178	760	aag01100 ; aag	KXJ82262.1 hypothet	XP_021697545.1NAD kinase 2, mitochondrial
AAEL01433	uncharacterized LOC5564185	-0.200285904237873	0.213992141142333	0.647322709661982	NA	NA	NA	XP_001648523.2uncharacterized protein LOC5564185
AAEL01109	phosducin-like protein	-0.200263188932499	0.286844999174923	0.718895294966742	NA	NA	CRK92135.1 CLUMA_	XP_001661418.1phosducin-like protein
AAEL01050	ribulose-phosphate 3-epimerase	-0.199894027782014	0.184620808915549	0.612043911074917	00710 ; 00030 ; 00040	aag01100 ; aag	NA	XP_001660886.2ribulose-phosphate 3-epimerase
AAEL02634	NA	-0.199252833059439	0.424876492383809	0.814623464645531	NA	NA	NA	XP_019542546.1 trypsin-1
AAEL00833	uncharacterized LOC5570428	-0.199071009192705	0.200241257518152	0.633770569380376	NA	NA	KXJ74212.1 hypothet	XP_019540378.1 cGMP-dependent protein kinase, isozyme 2 forms cD4/T1/T3A/T3B-like isoform X2
AAEL01434	zinc metalloproteinase-disintegrin-like EoMP06	-0.199067163947129	0.585275818810534	0.885224865991869	NA	NA	NA	XP_001648534.2zinc metalloproteinase-disintegrin-like EoMP06
AAEL01756	patj homolog	-0.198864003810755	0.248006181685109	0.68058844480365	NA	aag04391	NA	XP_011493753.2patj homolog

AAEL01943	NA	-0.198408818765748	0.157492216185552	0.574839762234915	NA	NA	NA	XP_021713054.1PDZ and LIM domain protein Zasp isoform X1
AAEL00081	probable G-protein coupled receptor Mth-like 14	-0.198228602515726	0.319555705319235	0.745684464362801	NA	NA	ETN67413.1 hypothe	XP_021704360.1probable G-protein coupled receptor Mth-like 14
AAEL00985	uncharacterized LOC5572523	-0.198201144997867	0.103194768340784	0.485796149609891	NA	NA	NA	AAY41839.1putative secreted protein precursor
AAEL00816	protein elav	-0.198068061726438	0.137135123024115	0.546204444420855	NA	NA	KXJ84064.1 hypothet	XP_001658986.1protein elav
AAEL00220	uncharacterized LOC5573960	-0.197958171720811	0.590309835141051	0.887668474886052	NA	NA	KXJ80521.1 hypothet	XP_001654980.1uncharacterized protein LOC5573960
AAEL00487	GILT-like protein 1	-0.19782006013029	0.0842938295192018	0.44100196899321	NA	NA	KXJ73234.1 hypothet	XP_001649949.1GILT-like protein 1 isoform X1
AAEL01373	protein distal antenna	-0.197800603105212	0.51736185811063	0.861881144752444	NA	NA	NA	XP_021710803.1protein distal antenna isoform X2
AAEL00053	protein amalgam	-0.197707017196923	0.359662071900222	0.774255626424296	NA	NA	XP_019539760.1 PRE	XP_021709375.1protein amalgam isoform X2
AAEL00561	hemicentin-2	-0.197494436022479	0.672061980610938	0.912813925547137	NA	NA	KXJ70619.1 hypothet	XP_019559209.1 hemicentin-2
AAEL02433	NA	-0.197263191420218	0.175588508627861	0.599764460428203	NA	NA	NA	XP_021712948.1tolloid-like protein 1
AAEL01270	uncharacterized LOC5576671	-0.197245014461968	0.224020799431947	0.656259917301973	NA	NA	3BKR_A Chain A, Crys	ACA52025.1sterol carrier protein-2 like-3 variant 1
AAEL00458	beta-galactosidase	-0.197156338083304	0.456339926181738	0.832617462606238	00531 ; 00600 ; 00604 ;	aag01100 ; aag	KXJ73069.1 hypothet	XP_001649403.2beta-galactosidase isoform X1
AAEL01975	NA	-0.197066990797833	0.0877551077057414	0.452647141876812	NA	NA	NA	XP_021708853.1interferon regulatory factor 2-binding protein 1 isoform X4
AAEL00443	39S ribosomal protein L30, mitochondrial	-0.197047896927906	0.218876588813562	0.652412844728166	NA	aag03010	KXJ74889.1 hypothet	XP_001649114.139S ribosomal protein L30, mitochondrial
AAEL02338	NA	-0.196773966613654	0.590928714859443	0.888227055559718	NA	NA	NA	XP_021698044.1zinc finger protein 708
AAEL00323	cytochrome c oxidase subunit 6A2, mitochondrial	-0.196744408849858	0.178117997142256	0.603698640774602	NA	aag01100 ; aag	ETN66413.1 cytochro	XP_001656628.1cytochrome c oxidase subunit 6A2, mitochondrial
AAEL00283	inositol-3-phosphate synthase	-0.196686447850266	0.0901544689821978	0.458542183179048	00521 ; 00562	aag01100 ; aag	KXJ73050.1 hypothet	XP_001655998.2inositol-3-phosphate synthase
AAEL01174	glutathione S-transferase	-0.196621051160043	0.184484140619441	0.612043911074917	NA	aag01100 ; aag	XP_019529313.1 PRE	XP_021711349.1glutathione S-transferase isoform X2
AAEL00258	transmembrane protease serine 2	-0.196290214981702	0.0912009202987746	0.459220287049195	NA	NA	NA	XP_021706988.1transmembrane protease serine 2
AAEL01973	NA	-0.1962695971036	0.532029040215804	0.866700945213732	NA	NA	NA	XP_021712752.1BMP-2-inducible protein kinase-like
AAEL00654	uncharacterized LOC5568118	-0.196249925830089	0.237370922709737	0.671758630247645	NA	NA	ETN66115.1 hypothe	XP_021696651.1uncharacterized protein LOC5568118
AAEL01085	troponin I	-0.196204026086933	0.129277724033618	0.53537966541735	NA	NA	XP_019558632.1 PRE	XP_001661109.1troponin I isoform X2
AAEL01344	WD repeat-containing protein 47	-0.195947905497303	0.156469146474385	0.574474189691589	NA	NA	NA	XP_021698567.1WD repeat-containing protein 47 isoform X3
AAEL00128	NA	-0.195936794104515	0.099321309097949	0.478084745672	NA	NA	XP_019562398.1 PRE	XP_021698555.1anion exchange protein 3 isoform X2
AAEL00989	uncharacterized LOC5572580	-0.195931949946023	0.15577592457676	0.573702478134775	NA	NA	NA	XP_001654102.2uncharacterized protein LOC5572580
AAEL00921	acyl-CoA-binding protein homolog	-0.195899653683822	0.173369787349298	0.596392068481585	NA	NA	NA	XP_001659821.1acyl-CoA-binding protein homolog
AAEL00171	uncharacterized LOC5572072	-0.195808182264587	0.460586429963369	0.834457146359854	NA	NA	KXJ70004.1 hypothet	XP_022819414.1PHD finger protein 20 isoform X1
AAEL02726	NA	-0.19570266425778	0.569809253916119	0.880635379772131	NA	NA	NA	XP_021693090.1mucin-5AC isoform X5
AAEL02542	NA	-0.195693730683719	0.58150922320107	0.883594430630159	NA	NA	NA	XP_021701324.1uncharacterized protein LOC110676710
AAEL00902	aldehyde dehydrogenase, mitochondrial	-0.195542832470604	0.0821880275856021	0.436407518662845	NA	aag01100 ; aag	KFB41327.1 AGAP00	XP_001653648.2aldehyde dehydrogenase, mitochondrial
AAEL00050	uncharacterized LOC5578444	-0.195374073302225	0.449099402553672	0.829306073695304	NA	NA	KXJ81980.1 hypothet	XP_019526954.1 rho GTPase-activating protein gacR isoform X2
AAEL00645	asparagine synthetase domain-containing protein CG17486	-0.195241918947067	0.372928706255628	0.781903075566391	NA	NA	KXJ76164.1 hypothet	XP_021708297.1asparagine synthetase domain-containing protein CG17486
AAEL02697	NA	-0.195142757903813	0.497557079734529	0.856052403704179	NA	NA	NA	XP_021703803.1ATP-binding cassette sub-family G member 4
AAEL00894	protein misato	-0.195138887964585	0.336895031128637	0.758529013077004	NA	NA	XP_001862732.1 mis	XP_001653622.2protein misato
AAEL00070	serine palmitoyltransferase 2	-0.195119307883278	0.110696414579958	0.501764970456116	NA	aag01100 ; aag	KXJ79952.1 hypothet	XP_001650263.1serine palmitoyltransferase 2
AAEL00604	interleukin enhancer-binding factor 2 homolog	-0.195013329805966	0.222799555032968	0.655580227356264	NA	NA	KFB37419.1 AGAP00	XP_001651787.2interleukin enhancer-binding factor 2 homolog
AAEL01257	aspartate aminotransferase, cytoplasmic	-0.194921528391339	0.102996569093285	0.485796149609891	00960 ; 00401 ; 00360 ;	aag01100 ; aag	XP_001849180.1 con	XP_001662725.1aspartate aminotransferase, cytoplasmic
AAEL01724	aminopeptidase N	-0.194862157803501	0.129009777833811	0.535335481333802	480	NA	NA	XP_021693817.1aminopeptidase N
AAEL01106	protein gustavus	-0.19458518186352	0.340000723058971	0.761379832492257	NA	NA	KXJ83733.1 hypothet	XP_019550757.1 protein gustavus isoform X1
AAEL01971	NA	-0.19443641217316	0.279789255659172	0.713956199542592	NA	aag04144	NA	XP_021701971.1epidermal growth factor receptor substrate 15 isoform X2
AAEL00395	chromatin-remodeling complex ATPase chain Iswi	-0.194348861113856	0.526418472646926	0.865680248818465	NA	NA	KXJ69537.1 hypothet	XP_001648131.1chromatin-remodeling complex ATPase chain Iswi
AAEL01075	putative ATP synthase subunit f, mitochondrial	-0.194327706683446	0.166184688116254	0.584439853413553	NA	aag01100 ; aag	ETN65643.1 mitocho	XP_021707251.1putative ATP synthase subunit f, mitochondrial
AAEL00148	exportin-1	-0.194287382291692	0.131432024421527	0.538981683630591	NA	aag03013 ; aag	XP_001850861.1 chr	XP_019564754.1 exportin-1 isoform X1
AAEL00095	ubiquitin-like-conjugating enzyme ATG3	-0.19414938115678	0.18470397099055	0.612043911074917	NA	aag04140 ; aag	KXJ78032.1 hypothet	XP_001657463.1ubiquitin-like-conjugating enzyme ATG3
AAEL00765	activator of basal transcription 1	-0.194000616448794	0.419110115488822	0.813348977896628	NA	NA	KXJ62478.1 hypothet	XP_001658562.1activator of basal transcription 1
AAEL00483	uncharacterized LOC5565542	-0.193743094429276	0.152655181091604	0.568113296551786	NA	NA	NA	XP_001649897.2uncharacterized protein LOC5565542
AAEL02513	NA	-0.193702508156046	0.159906020706817	0.577560236664366	NA	NA	NA	XP_021694780.1rho guanine nucleotide exchange factor 10 isoform X2
AAEL00737	gamma-aminobutyric acid type B receptor subunit 1	-0.193591393073039	0.635184309113513	0.902538767814767	NA	aag04080	ETN62953.1 gaba-b r	XP_021700165.1 gamma-aminobutyric acid type B receptor subunit 1
AAEL01726	protein snwA	-0.193256877305495	0.100291854722797	0.480169431404357	NA	NA	NA	XP_011493255.1protein snwA
AAEL00765	partitioning defective 3 homolog	-0.19309949608462	0.169969602314564	0.59043423197381	NA	aag04144 ; aag	NA	XP_021694681.1partitioning defective 3 homolog isoform X4
AAEL01107	dedicator of cytokinesis protein 7	-0.19308735467812	0.316934814862967	0.745684464362801	NA	NA	XP_019550666.1 PRE	XP_001655092.2 dedicator of cytokinesis protein 7
AAEL01971	NA	-0.19303173315091	0.467279803401672	0.839170326093322	NA	aag04013 ; aag	NA	XP_021697922.1protein vein
AAEL01293	neuferricin homolog	-0.192999626699585	0.399419517958503	0.798648539435638	NA	NA	NA	XP_001656305.2neuferricin homolog
AAEL00929	lachesin	-0.192838478183306	0.174076824957691	0.597209919209252	NA	NA	NA	XP_019538560.1 lachesin
AAEL01132	gonadotropin-releasing hormone II receptor	-0.192820865467184	0.196836036314946	0.630345746481079	NA	aag04080	NA	XP_021700050.1gonadotropin-releasing hormone II receptor isoform X5
AAEL00852	protein sel-1 homolog 1	-0.19270476999557	0.117663254196359	0.515237493575542	NA	aag04141	KXJ67950.1 hypothet	XP_001659361.1protein sel-1 homolog 1
AAEL00258	toll-like receptor 7	-0.19255117333216	0.435591631233063	0.820960220424486	NA	NA	KFB49716.1 toll [Ano	XP_001655730.1toll-like receptor 7
AAEL02450	NA	-0.192519643149421	0.245605243848825	0.679727114240672	NA	NA	NA	XP_021694146.1uncharacterized protein LOC5565140 isoform X2
AAEL00334	RING finger and SPRY domain-containing protein 1	-0.19251297277372	0.279320498259239	0.713604060506006	NA	NA	XP_001846522.1 con	XP_021702827.1RING finger and SPRY domain-containing protein 1
AAEL01946	NA	-0.192479249211195	0.158229163593497	0.576186113377291	NA	NA	NA	XP_021693563.1papilin isoform X1
AAEL01821	NA	-0.192449311840552	0.12306759423067	0.525507193554816	NA	aag04013	NA	XP_001661653.2uncharacterized protein LOC5574833
AAEL00236	transmembrane protein 177	-0.192375921113854	0.357647006519909	0.773736338358957	NA	NA	KXJ76378.1 hypothet	XP_001661389.1transmembrane protein 177
AAEL02164	NA	-0.192343890627785	0.611352537221713	0.894646314231935	NA	NA	NA	XP_021705984.1WD repeat-containing and planar cell polarity effector protein fritz
AAEL02322	NA	-0.192298460105967	0.0975511162203219	0.473196387605561	NA	NA	NA	XP_021699182.1uncharacterized protein LOC5580119 isoform X1
AAEL01304	uncharacterized LOC5564174	-0.192288626349151	0.369567652070016	0.781817769662763	NA	NA	NA	XP_021700568.1uncharacterized protein LOC5564174 isoform X1
AAEL00018	protein GUCD1	-0.192250317808353	0.429966407371458	0.818483839601191	NA	NA	KXJ69358.1 hypothet	XP_001658936.1protein GUCD1
AAEL00021	turripeptide Pal9.2	-0.192232418729387	0.103422863460853	0.486208982530205	NA	NA	KXJ71313.1 hypothet	XP_001658905.1turripeptide Pal9.2
AAEL02200	NA	-0.192207929906011	0.159478282492559	0.577560236664366	NA	NA	NA	XP_019933416.1 nascent polypeptide-associated complex subunit alpha, muscle-specific form-like isoform X1
AAEL01379	splicing factor 3B subunit 4	-0.192093237548979	0.325939237562557	0.748896673349414	NA	aag03040	NA	XP_001663981.1splicing factor 3B subunit 4
AAEL01473	calcium-independent phospholipase A2-gamma	-0.192060971320269	0.492704680454004	0.852158101574964	NA	NA	NA	XP_021701745.1calcium-independent phospholipase A2-gamma isoform X1
AAEL00625	serine protease easter	-0.191962194465059	0.1015145413922	0.483758057944601	NA	NA	XP_019545784.1 PRE	XP_021706662.1transmembrane protease serine 9 isoform X1
AAEL00962	poly(U)-specific endoribonuclease homolog	-0.191909854091131	0.112938167892307	0.503928507434265	NA	NA	NA	XP_001660282.2poly(U)-specific endoribonuclease homolog
AAEL01487	bifunctional methylenetetrahydrofolate dehydrogenase/cyclohydrolase,	-0.191877288025697	0.404567541154953	0.800780923576657	00670 ; 00720	aag01100 ; aag	NA	XP_021706150.1bifunctional methylenetetrahydrofolate dehydrogenase/cyclohydrolase, mitochondrial isoform X1
AAEL00505	3'(2'),5'-bisphosphate nucleotidase 1	-0.191869324153649	0.235351131173721	0.669459384562775	NA	aag01100 ; aag	XP_001845434.1 pap	XP_001650308.23'(2'),5'-bisphosphate nucleotidase 1
AAEL00043	transcription factor grauzone	-0.191812411902796	0.558132975719629	0.877488406385106	NA	NA	KXJ80118.1 hypothet	XP_001656397.1transcription factor grauzone
AAEL00120	threonine--tRNA ligase, cytoplasmic	-0.191806771361372	0.109739638334467	0.499171602547586	970	aag00970	XP_019535879.1 PRE	XP_021703045.1threonine--tRNA ligase, cytoplasmic isoform X2
AAEL00022	prosialokinin	-0.191719191350912	0.167534055707325	0.587554508928878	NA	NA	NA	P42634.2RecName: Full=Prosialokinin; Contains: RecName: Full=Sialokinin; AltName: Full=Sialokinin I; Short=Sia I;
AAEL00253	plastin-1	-0.191710333761296	0.123752575483053	0.527255157278773	NA	NA	XP_019549657.1 PRE	XP_001655615.2plastin-1 isoform X2
AAEL01305	uncharacterized LOC5577171	-0.191704646574623	0.620300030786364	0.897385728224103	NA	NA	NA	XP_001663255.1uncharacterized protein LOC5577171
AAEL01982	NA	-0.191701179397329	0.242236301737327	0.676994125742042	NA	NA	NA	XP_021701207.1kinesin-like protein KIF21A isoform X4
AAEL00925	broad-complex core protein isoforms 1/2/3/4/5	-0.191510074319297	0.319004878503643	0.745684464362801	NA	NA	NA	XP_021695074.1broad-complex core protein isoforms 1/2/3/4/5 isoform X3
AAEL00981	ubiquitin-like modifier-activating enzyme ATG7	-0.191454373244836	0.358180793646352	0.773854174836904	NA	aag04140 ; aag	NA	XP_001660430.2ubiquitin-like modifier-activating enzyme ATG7

AAEL0061(	checkpoint protein HUS1	-0.191412104448348	0.665474482677075	0.911964500946328	NA	NA	KXJ77316.1 hypothe	XP_001657422.1checkpoint protein HUS1
AAEL0126	uncharacterized LOC5576617	-0.191359742920811	0.158633585045525	0.576519055919795	NA	NA	KXJ80514.1 hypothe	XP_001662771.2uncharacterized protein LOC5576617
AAEL0020	protein tipE	-0.191290912512987	0.30098950430793	0.733161661578971	NA	NA	KXJ78915.1 hypothe	XP_001654727.1protein tipE
AAEL0199	NA	-0.191102708787337	0.103966980914094	0.486414446815465	04151 ; 05165 ; 04714 ;	aag04150 ; aag	NA	XP_021706127.1serine/threonine-protein kinase ULK2 isoform X2
AAEL0018	uncharacterized LOC5572466	-0.191026919655136	0.290081109319371	0.721952579380462	NA	NA	KXJ73786.1 hypothe	XP_001660412.2uncharacterized protein LOC5572466
AAEL0060	vitellogenin-3	-0.190610083837129	0.175821535932071	0.600337578347651	NA	NA	XP_019548184.1 PRE	XP_019548184.1 vitellogenin-3-like
AAEL0027	neuropeptide F	-0.190439492988859	0.269080492566024	0.705147428250005	NA	NA	AFS50167.1 neurope	XP_021709892.1neuropeptide F isoform X4
AAEL0231	NA	-0.190391008164889	0.10812804573081	0.494284290519081	NA	NA	NA	XP_021713210.1filamin-A isoform X3
AAEL0023	probable ATP-dependent RNA helicase DDX17	-0.190213922911038	0.271452424503153	0.706703671719641	NA	NA	KXJ69994.1 hypothe	XP_001661370.1DEAD-box ATP-dependent RNA helicase 20 isoform X1
AAEL0106	farnesol dehydrogenase-like	-0.190187941420742	0.407564383886736	0.803607536382376	NA	NA	XP_001847204.1 oxi	XP_001661001.2farnesol dehydrogenase-like
AAEL0213	NA	-0.190161117174944	0.100086630044868	0.479706814084761	NA	aag04141 ; aag	NA	XP_021707648.1dual specificity mitogen-activated protein kinase kinase hemipterous isoform X2
AAEL0101	terminal uridylyltransferase Tailor	-0.190100194142175	0.427219854112894	0.815782298406567	NA	NA	NA	XP_001654283.2terminal uridylyltransferase Tailor
AAEL0207	NA	-0.190049932989609	0.333132496086264	0.755603950945478	NA	NA	NA	NA
AAEL0030	dual specificity mitogen-activated protein kinase kinase 4	-0.1899926779999	0.20479773838181	0.637782699712521	NA	aag04013	KFB53380.1 AGAP00	XP_001662902.1dual specificity mitogen-activated protein kinase kinase 4
AAEL0242	NA	-0.189874841516708	0.22357018337285	0.655920215788323	NA	aag04350	NA	XP_021699929.1dorsal-ventral patterning protein Sog
AAEL0182	NA	-0.189842748298323	0.520404634398235	0.864397733886744	NA	NA	NA	XP_021704777.1F-box/LRR-repeat protein 16
AAEL0046	peroxisomal (S)-2-hydroxy-acid oxidase GLO3	-0.189783950747237	0.10390737123618	0.486414446815465	NA	aag01100 ; aag	KXJ72899.1 hypothe	XP_001649576.2peroxisomal (S)-2-hydroxy-acid oxidase GLO3
AAEL0118	cAMP-dependent protein kinase catalytic subunit PRKX	-0.18976501478667	0.290164116376275	0.721952579380462	NA	NA	XP_019537732.1 PRE	XP_001661976.2cAMP-dependent protein kinase catalytic subunit PRKX
AAEL0124	uncharacterized oxidoreductase dhs-27	-0.189734080592153	0.647090012454237	0.906554905014882	901	NA	XP_313880.5 AGAP0	XP_001662618.1uncharacterized oxidoreductase dhs-27
AAEL0072	protein anachronism	-0.189662464445737	0.388836208413612	0.793285976044306	NA	NA	XP_019535043.1 PRE	XP_001658218.2protein anachronism
AAEL0026	60S ribosomal protein L36	-0.189645588676995	0.203865751858603	0.637218970313474	NA	aag03010	ETN64210.1 60S ribo	XP_001661966.160S ribosomal protein L36
AAEL0093	formin-like protein CG32138	-0.189633740135808	0.250721019053926	0.683858606596939	NA	NA	NA	XP_021709642.1formin-like protein CG32138
AAEL0015	peregrin	-0.189465290111576	0.464120447336041	0.837274753622609	NA	NA	KXJ79230.1 hypothe	XP_021695919.1peregrin isoform X2
AAEL0075	cysteine sulfinic acid decarboxylase	-0.189401120187144	0.147198308145425	0.561952643558699	NA	NA	XP_001851393.1 glu	XP_001658435.2cysteine sulfinic acid decarboxylase
AAEL0267	NA	-0.189339120009101	0.3904890726385	0.793285976044306	NA	NA	NA	XP_021702843.1DENN domain-containing protein 1B isoform X2
AAEL0035	uncharacterized LOC5578346	-0.189246278824703	0.541887219884041	0.869900911343623	NA	NA	KXJ68890.1 hypothe	XP_021704791.1uncharacterized protein LOC5578346
AAEL0215	NA	-0.18923587093111	0.116303638122812	0.514016368748824	NA	NA	NA	XP_021700085.1 junctophilin-3
AAEL0205	NA	-0.189216884582992	0.401203921955113	0.798934968584928	NA	NA	NA	XP_021703352.1uncharacterized protein LOC110677064
AAEL0096	pyruvate carboxylase, mitochondrial	-0.188988670467721	0.103950088630339	0.486414446815465	00020 ; 00720 ; 00620	aag01100 ; aag	NA	XP_001653941.1pyruvate carboxylase, mitochondrial isoform X3
AAEL0098	uncharacterized LOC5572521	-0.188970146743433	0.422705695513062	0.814192545906728	NA	NA	NA	XP_019559168.1 microtubule-associated protein futsch-like
AAEL0223	NA	-0.188955435256674	0.506842153445614	0.859256178927183	NA	NA	NA	XP_021707319.1nose resistant to fluoxetine protein 6 isoform X1
AAEL0075	leukocyte receptor cluster member 8 homolog	-0.18892618762526	0.0900968493375648	0.458542183179048	NA	NA	KXJ74975.1 hypothe	XP_001658437.2leukocyte receptor cluster member 8 homolog
AAEL0001	probable arginine--tRNA ligase, cytoplasmic	-0.188897853188898	0.182489405991949	0.60931332145788	970	aag00970	KXJ71316.1 hypothe	XP_001658908.2probable arginine--tRNA ligase, cytoplasmic
AAEL0127	ATP-binding cassette sub-family A member 3	-0.188727876770455	0.126449884159769	0.530481545423179	NA	NA	KXJ81821.1 hypothe	XP_001662813.2ATP-binding cassette sub-family A member 3
AAEL0083	acylphosphatase-2	-0.188633062081018	0.220331543994206	0.652412844728166	00620 ; 00627	aag01100 ; aag	KXJ75721.1 hypothe	XP_019558405.1 acylphosphatase-2 isoform X2
AAEL0214	NA	-0.188331656205976	0.543044707275924	0.869900911343623	NA	NA	NA	XP_021697557.1chromatin modification-related protein eaf-1
AAEL0010	serine protease easter	-0.188268262028974	0.279363776096671	0.713604060506006	NA	NA	KXJ83232.1 hypothe	XP_001658015.2serine protease easter
AAEL0103	polyadenylate-binding protein	-0.187940267667335	0.210705877197611	0.642876486065124	NA	aag03013 ; aag	NA	XP_001654420.1polyadenylate-binding protein
AAEL0147	NA	-0.187870793025136	0.403992135732774	0.800429773223221	NA	NA	NA	XP_001649539.2cytochrome b5
AAEL0037	regulator of nonsense transcripts 3B	-0.187818801554288	0.290688695576826	0.722005075789413	NA	aag03013 ; aag	XP_001847099.1 con	XP_021699343.1regulator of nonsense transcripts 3B
AAEL0111	protein app1	-0.18771940327517	0.138722219483678	0.548805616375239	NA	NA	XP_019545507.1 PRE	XP_001655114.1protein app1 isoform X1
AAEL0271	NA	-0.187646903165344	0.102956144630802	0.485796149609891	NA	NA	NA	XP_021695442.1aspartyl/asparaginyl beta-hydroxylase isoform X5
AAEL0024	NA	-0.187590805355837	0.164885165621808	0.583566651558413	NA	NA	NA	XP_021697003.1LOW QUALITY PROTEIN: uncharacterized protein LOC5574790
AAEL0092	histone H3-like centromeric protein A	-0.187444052971609	0.569809253840129	0.880635379772131	NA	NA	NA	XP_001659902.2histone H3-like centromeric protein A
AAEL0035	calcium-transporting ATPase type 2C member 1	-0.187427782007808	0.157997342678203	0.575615420985807	NA	NA	XP_019552700.1 PRE	XP_001656907.2calcium-transporting ATPase type 2C member 1 isoform X1
AAEL0211	NA	-0.187410104427353	0.507282854824636	0.859406428557307	NA	NA	NA	XP_021711315.1 sphingosine-1-phosphate lyase
AAEL0133	ADP-ribose pyrophosphatase, mitochondrial	-0.187326577431153	0.375611549399843	0.78228031361 52	NA	aag01100 ; aag	NA	XP_001663566.1ADP-ribose pyrophosphatase, mitochondrial
AAEL0194	NA	-0.187239317496921	0.217023648259383	0.649748124914163	NA	aag04320	NA	XP_021703807.1CUGBP Elav-like family member 2 isoform X1
AAEL0018	carboxypeptidase B	-0.186911089816725	0.21734856562374	0.650018359996468	NA	NA	NA	XP_001654128.2carboxypeptidase B
AAEL0065	calcium-transporting ATPase sarcoplasmic/endoplasmic reticulum type	-0.186699702327717	0.171988653382993	0.594083929590956	NA	NA	XP_019553378.1 PRE	XP_001652085.2calcium-transporting ATPase sarcoplasmic/endoplasmic reticulum type isoform X1
AAEL0019	protein bark beetle	-0.186689155479122	0.299248800541295	0.730496185008314	NA	NA	XP_019559241.1 PRE	XP_001654222.2protein bark beetle isoform X2
AAEL0006	protein pangolin, isoforms A/H/I/S	-0.186638417399298	0.495984762235545	0.854563339971561	NA	aag04310	KXJ84109.1 hypothe	XP_001649806.1protein pangolin, isoforms A/H/I/S
AAEL0187	NA	-0.186607531615669	0.565824829129489	0.880302387766415	NA	aag01100 ; aag	NA	XP_021700823.1glycerate kinase isoform X1
AAEL0064	adrenodoxin-like protein, mitochondrial	-0.186366629022253	0.319536810796353	0.745684464362801	NA	NA	XP_001846303.1 adr	XP_001657805.2adrenodoxin-like protein, mitochondrial
AAEL0098	nuclear cap-binding protein subunit 1	-0.186171533181356	0.267861605842884	0.704026692311667	NA	aag03013 ; aag	NA	XP_001660456.1nuclear cap-binding protein subunit 1
AAEL0230	NA	-0.186100548330793	0.103984834504265	0.486414446815465	NA	aag03040	NA	XP_021693796.1RNA-binding protein 25
AAEL0048	zinc finger protein 35	-0.186076894682779	0.67830604562719	0.914481546552978	NA	NA	KXJ80337.1 hypothe	XP_021693800.1zinc finger protein 35
AAEL0028	zinc finger and SCAN domain-containing protein 31	-0.186051937554941	0.409230416505132	0.805356078752463	NA	NA	KXJ77321.1 hypothe	XP_001662482.2zinc finger and SCAN domain-containing protein 31
AAEL0018	UDP-glucuronosyltransferase 2A3	-0.185999737633928	0.376683406652666	0.783277809573714	NA	aag01100 ; aag	KXJ80685.1 hypothe	XP_001660379.1UDP-glucuronosyltransferase 2A3
AAEL0016	ankyrin repeat domain-containing protein 49	-0.185958464644052	0.346984284341344	0.765398366276275	NA	NA	KXJ70873.1 hypothe	XP_001653736.1ankyrin repeat domain-containing protein 49
AAEL0232	NA	-0.185892404422683	0.404519908169346	0.800780923576657	NA	NA	NA	XP_021711980.1U3 small nucleolar ribonucleoprotein protein IMP4-like
AAEL0057	diphthine methyltransferase	-0.185680857679815	0.403349422235365	0.800263342692934	NA	NA	KXJ68556.1 hypothe	XP_001651369.1diphthine methyltransferase isoform X2
AAEL0243	NA	-0.185563251480872	0.572409169308672	0.880923047085115	NA	NA	NA	NA
AAEL0132	GA-binding protein subunit beta-2	-0.185484317380885	0.261881102905746	0.695344775901121	NA	NA	NA	XP_001663445.2GA-binding protein subunit beta-2
AAEL0051	uncharacterized serine-rich protein C215.13	-0.185200733912995	0.277577657195628	0.713084757541644	NA	NA	ETN59238.1 hypothe	XP_001650481.1uncharacterized serine-rich protein C215.13
AAEL0033	ATP synthase subunit beta, mitochondrial	-0.185142171095069	0.184661333497787	0.612043911074917	00195 ; 00190	aag01100 ; aag	XP_019542984.1 PRE	XP_001656737.2ATP synthase subunit beta, mitochondrial
AAEL0111	zinc finger CCCH domain-containing protein 14	-0.185033741842613	0.454856006251549	0.832617462606238	NA	NA	KXJ70795.1 hypothe	XP_021704944.1zinc finger CCCH domain-containing protein 14
AAEL0040	enhancer of split mbeta protein	-0.185007350160772	0.516242038152699	0.861448202130317	NA	NA	KFB37689.1 hypothe	XP_001648447.1enhancer of split mbeta protein
AAEL0123	NA	-0.184915736664282	0.187037144184319	0.614196367514984	NA	NA	NA	XP_001662501.2uncharacterized protein LOC5576154 isoform X1
AAEL0036	patched domain-containing protein 3	-0.184889026238393	0.166057535004344	0.584439853413553	NA	NA	KXJ79597.1 hypothe	XP_021710792.1patched domain-containing protein 3
AAEL0195	NA	-0.184846659013415	0.193369164296836	0.624628157151667	NA	aag01100 ; aag	NA	XP_021698189.1high affinity cAMP-specific and IBMX-insensitive 3',5'-cyclic phosphodiesterase 8 isoform X4
AAEL0128	RPII140-upstream gene protein	-0.184810476028102	0.285769857601336	0.719970179170389	NA	NA	KXJ75343.1 hypothe	XP_001662950.2RPII140-upstream gene protein
AAEL0122	peflin	-0.1846006470583	0.35910776099721	0.77388983997572	NA	NA	KFB36015.1 AGAP00	XP_019545356.1 peflin
AAEL0004	proteoglycan 4	-0.18457686160467	0.466610072301091	0.838900928502398	NA	NA	XP_001687841.1 AG	XP_001656062.1proteoglycan 4
AAEL0282	NA	-0.184556066145385	0.574951899347458	0.881197098233729	NA	aag01100 ; aag	NA	XP_021693731.1guanosine-3',5'-bis(diphosphate) 3'-pyrophosphohydrolase MESH1 isoform X1
AAEL0212	NA	-0.184503809573778	0.324811697081001	0.748164514778316	NA	NA	NA	XP_021705224.1 atypical kinase COQ8B, mitochondrial
AAEL0108	zinc finger protein 708	-0.184491367681561	0.589560864178617	0.887330564633895	NA	NA	KXJ72539.1 hypothe	XP_001661078.1zinc finger protein 708
AAEL0265	NA	-0.184329720831166	0.201760387721253	0.634701781910801	NA	NA	NA	NA
AAEL0281	NA	-0.184245685320372	0.456265823172464	0.832617462606238	NA	aag04310	NA	XP_021708227.1disheveled-associated activator of morphogenesis 1 isoform X3
AAEL0060	probable cytochrome P450 313a4	-0.184175552718183	0.132499432002214	0.539973327406722	NA	NA	KXJ83301.1 hypothe	XP_001651778.2probable cytochrome P450 313a4
AAEL0195	NA	-0.184100137991944	0.207829044855792	0.639832374753494	NA	NA	NA	XP_021706441.1protein unc-79 homolog
AAEL0199	NA	-0.184081452839563	0.334993162361124	0.757583455406012	NA	NA	NA	XP_021708913.1spastin isoform X1

AAEL0002(	uncharacterized LOC5570053	-0.184018725376228	0.226656279770255	0.660446829146891	NA	NA	KXJ78657.1 hypothe‹	XP_021707851.1uncharacterized protein LOC5570053
AAEL0130⁴	zinc finger and SCAN domain-containing protein 31	-0.183860862818372	0.585867695577436	0.885247050033426	NA	NA	NA	XP_001663265.1zinc finger and SCAN domain-containing protein 31
AAEL0117(	phenoloxidase 2	-0.183805796094395	0.251718056238783	0.684556086619159	NA	NA	XP_001870839.1 phe	XP_001661891.2phenoloxidase 2
AAEL01061	acyl carrier protein, mitochondrial	-0.183797520242056	0.162832852728586	0.581366301644056	NA	aag01100 ; aag	XP_019529314.1 PRE	XP_001660948.1acyl carrier protein, mitochondrial isoform X1
AAEL0194ż	NA	-0.183592852345504	0.159105602765329	0.577560236664366	NA	aag04391	NA	XP_021694599.1protein lap4 isoform X1
AAEL0052(	transmembrane protein 184B	-0.183499894298364	0.102290776166796	0.484636597025644	NA	NA	XP_019539032.1 PRE	XP_001650606.1transmembrane protein 184B isoform X2
AAEL0197ε	NA	-0.183496548692626	0.270946175653733	0.706427507335574	NA	NA	NA	XP_019545263.1 proteoglycan 4-like
AAEL0084‡	uncharacterized LOC5570645	-0.183465969658525	0.450052176962995	0.829603391909751	NA	NA	KFB42624.1 AGAP00	KFB42624.1AGAP002323-like protein
AAEL0047ꞇ	zinc finger protein 665	-0.183288389006113	0.352128502622581	0.769212685559608	NA	NA	KXJ81347.1 hypothe‹	XP_021700992.1zinc finger protein 665
AAEL0078ε	translation initiation factor IF-3	-0.183279480649501	0.214929663517184	0.648028428468258	NA	NA	KXJ69377.1 hypothe‹	XP_019565523.1 translation initiation factor IF-3, mitochondrial-like
AAEL0097‡	protein msta	-0.183129004066461	0.539990316642793	0.869709828277711	NA	NA	NA	XP_001660315.1protein msta
AAEL0028ꞇ	bone morphogenetic protein 1	-0.182971171240107	0.347344246037224	0.765755091527201	NA	NA	NA	XP_021693261.1cubilin isoform X3
AAEL0105⁴	NA	-0.182950337925124	0.342207718327917	0.762653287491765	230	aag01100 ; aag	XP_019526528.1 PRE	XP_001660904.2 calcium/calmodulin-dependent 3',5'-cyclic nucleotide phosphodiesterase 1
AAEL0124⁴	general transcription factor 3C polypeptide 1	-0.182883881577577	0.292951958268244	0.723631099647605	NA	NA	XP_019546939.1 PRE	XP_021713185.1general transcription factor 3C polypeptide 1
AAEL0026ꜫ	prestalk protein	-0.182816331651929	0.506049200173242	0.858744381072989	NA	NA	KXJ80183.1 hypothe‹	XP_001655707.2prestalk protein
AAEL0025ꜫ	glutamate receptor ionotropic, kainate 2	-0.182815856259189	0.443463020052134	0.82545872799161	NA	NA	KXJ71033.1 hypothe‹	XP_001655465.2glutamate receptor ionotropic, kainate 2
AAEL0004(	protein kish	-0.182723917307749	0.309756957701652	0.742889924206485	NA	NA	XP_001844384.1 trar	XP_001656444.1protein kish
AAEL0148(	protein diaphanous	-0.182488359689577	0.243987842377962	0.679121635076226	NA	NA	NA	XP_021701663.1protein diaphanous
AAEL02221	NA	-0.182458472075492	0.520152170578987	0.864134369681863	NA	NA	NA	XP_021707151.1GILT-like protein 1
AAEL0170ż	CD109 antigen	-0.182367740027732	0.136162019807504	0.545589928345625	NA	NA	NA	XP_021701341.1CD109 antigen
AAEL0211ꞇ	NA	-0.182345896968705	0.350997462717269	0.768287024720341	230	aag01100 ; aag	NA	XP_021694962.15-hydroxyisourate hydrolase
AAEL0021ꜫ	tubulin-folding cofactor B	-0.182278367903624	0.268481227551679	0.704184172798867	NA	NA	ETN63340.1 tubulin-:	XP_001654808.1tubulin-folding cofactor B
AAEL0050ż	zinc finger protein 266	-0.182194885443085	0.544174699021401	0.870383849238518	NA	NA	KXJ71836.1 hypothe‹	XP_021703603.1zinc finger protein 266 isoform X2
AAEL0103ꜫ	alpha-tocopherol transfer protein-like	-0.182162441778387	0.208870176910028	0.639832374753494	NA	NA	NA	XP_021702922.1alpha-tocopherol transfer protein isoform X1
AAEL0221ꜫ	NA	-0.182053087717853	0.1241474076824	0.527832049142223	NA	NA	NA	XP_021699228.1uncharacterized protein LOC5577147 isoform X1
AAEL0197(	NA	-0.18203628875995	0.55917112744372	0.877581080322399	NA	aag01100 ; aag	NA	XP_021696662.1chondroitin sulfate N-acetylgalactosaminyltransferase 1
AAEL0062ε	uncharacterized LOC5567718	-0.181818348187261	0.52204626365014	0.865018698932415	NA	NA	KXJ68207.1 hypothe‹	XP_001657635.1uncharacterized protein LOC5567718
AAEL0045(	kynurenine formamidase	-0.181634358296278	0.453644872486253	0.832617462606238	00380 ; 00630	aag01100 ; aag	KXJ72302.1 hypothe‹	XP_001649275.2kynurenine formamidase
AAEL0139ç	guanine nucleotide-binding protein subunit beta-1	-0.18160638341666	0.303887326814917	0.737301362252096	NA	NA	NA	CRK97668.1CLUMA_CG011050, isoform A
AAEL0040⁴	probable glucosamine 6-phosphate N-acetyltransferase	-0.181558490568774	0.120534282753751	0.521710475023524	NA	aag01100 ; aag	AAV84020.1 glucosaı	XP_001648374.1probable glucosamine 6-phosphate N-acetyltransferase
AAEL0065ε	NA	-0.181525695469401	0.14087193029724	0.552323605707371	NA	NA	KXJ84502.1 hypothe‹	XP_001652077.1serine protease easter
AAEL0085‡	glutaminase kidney isoform, mitochondrial	-0.181442670302562	0.410830155799738	0.805855841282375	00471 ; 00220 ; 00250	aag01100 ; aag	XP_019554783.1 PRE	XP_001653299.2glutaminase liver isoform, mitochondrial isoform X2
AAEL0196⁴	NA	-0.181418948427035	0.199207654326322	0.633198215927742	NA	NA	NA	XP_021700547.1uncharacterized protein LOC5566421 isoform X1
AAEL0208(	NA	-0.181274714311511	0.1196696014111	0.519433807634405	NA	NA	NA	XP_019559044.1 titin-like
AAEL0086ꞇ	ATP-binding cassette sub-family G member 1	-0.181254478545235	0.148698349463061	0.563197902632466	NA	NA	XP_019559121.1 PRE	XP_001659406.1ATP-binding cassette sub-family G member 1 isoform X2
AAEL0013ꜫ	integrator complex subunit 4	-0.181184106946334	0.390301953705117	0.793285976044306	NA	NA	KXJ76091.1 hypothe‹	XP_001653065.1integrator complex subunit 4
AAEL0199‡	NA	-0.181102910084965	0.342964953029312	0.76358426442382	4070	aag04150 ; aag	NA	XP_021711540.1protein kinase C, brain isozyme isoform X1
AAEL0067ꞇ	rab11 family-interacting protein 4B	-0.181079069415745	0.101135959681688	0.482703890555441	NA	aag04144	XP_019546738.1 PRE	XP_021704961.1rab11 family-interacting protein 4B isoform X1
AAEL0071ꜫ	facilitated trehalose transporter Tret1	-0.180960316925737	0.43758913634499	0.820960220424486	NA	NA	KXJ82001.1 hypothe‹	XP_021700712.1facilitated trehalose transporter Tret1
AAEL0111ż	G2/M phase-specific E3 ubiquitin-protein ligase	-0.18077052118527	0.528976218081868	0.865973876319045	NA	NA	NA	XP_001655100.1G2/M phase-specific E3 ubiquitin-protein ligase
AAEL0127(	ATP-binding cassette sub-family A member 1	-0.180749347398206	0.152062885320761	0.568113296551786	NA	NA	XP_019547189.1 PRE	XP_001662815.2ATP-binding cassette sub-family A member 1
AAEL0095ꜫ	F-box only protein 25	-0.180599495443326	0.498009986321392	0.856340365764943	NA	aag04068	NA	XP_001660205.1F-box only protein 25
AAEL00541	ER membrane protein complex subunit 6	-0.180524158806233	0.326177911521973	0.748896673349414	NA	NA	KFB52656.1 AGAP00	XP_001650857.1ER membrane protein complex subunit 6
AAEL00291	ATP-binding cassette sub-family D member 3	-0.180501360928177	0.103202666670031	0.485796149609891	NA	aag04146 ; aag	ETN60116.1 peroxisc	XP_001662679.2ATP-binding cassette sub-family D member 3
AAEL0194⁴	NA	-0.180475464397152	0.402155838885029	0.799283473604722	NA	NA	NA	XP_021695944.1potassium voltage-gated channel subfamily H member 7 isoform X8
AAEL0008⁴	UPF0587 protein GA18326	-0.180370499922127	0.22090186558698	0.652797615862164	NA	NA	KXJ69368.1 hypothe‹	XP_001651292.1UPF0587 protein GA18326
AAEL0019ꜫ	protein msta	-0.180342573895222	0.479534930312719	0.845454613425911	NA	NA	KXJ77028.1 hypothe‹	XP_001654323.2protein msta
AAEL0149ç	protein alan shepard	-0.180333063985488	0.166211594912331	0.584439853413553	NA	NA	NA	XP_021698039.1protein alan shepard isoform X13
AAEL0041ż	SH2B adapter protein 2	-0.180032836925587	0.310365231669584	0.742918550320373	NA	NA	XP_019531022.1 PRE	XP_021708000.1SH2B adapter protein 2 isoform X3
AAEL0093ꞇ	protein arginine methyltransferase NDUFAF7 homolog, mitochondrial	-0.179888323949147	0.587889263427033	0.886401261871374	NA	NA	NA	XP_001653802.1protein arginine methyltransferase NDUFAF7 homolog, mitochondrial
AAEL0254ꜫ	NA	-0.179736004298874	0.486701982767866	0.848547109782095	NA	NA	NA	NA
AAEL00671	splicing factor U2af 38 kDa subunit	-0.17968344857507	0.149909732544713	0.565429182817541	NA	aag03040	KFB36360.1 AGAP00	XP_001657976.1splicing factor U2af 38 kDa subunit
AAEL0015(	uncharacterized LOC5571113	-0.179553217417142	0.124345765912815	0.528268577068201	NA	NA	XP_019552753.1 PRE	XP_019552753.1 triple functional domain protein isoform X2
AAEL0020‡	neuroendocrine protein 7B2	-0.17928436790245	0.165370714152519	0.584439853413553	NA	NA	ETN59951.1 neuroen	XP_001654557.1neuroendocrine protein 7B2
AAEL0194(	NA	-0.179084790826475	0.171595365830778	0.593393670189711	480	aag04146	NA	XP_001648452.3peroxiredoxin 1
AAEL00081	protein kinase C	-0.178973106152173	0.351906429692006	0.769212685559608	4070	aag04933	XP_019931850.1 PRE	XP_021709054.1protein kinase C isoform X1
AAEL0102ꞇ	aminomethyltransferase, mitochondrial	-0.178767076144227	0.233246017551343	0.666962220912214	00670 ; 00260	aag01100 ; aag	NA	XP_001660716.2aminomethyltransferase, mitochondrial
AAEL0069ε	probable phospholipase A1 magnifin	-0.178687262510585	0.511633932912119	0.860862214418965	NA	NA	KXJ70519.1 hypothe‹	XP_001652443.1phospholipase A1 member A isoform X2
AAEL0132ç	uncharacterized LOC5577581	-0.178504703193645	0.285765716304752	0.717970179170389	NA	NA	NA	XP_001656572.2uncharacterized protein LOC5577581 isoform X2
AAEL0138⁴	uncharacterized LOC5578728	-0.178278094186004	0.230683278872607	0.66377346222193	NA	NA	NA	XP_021694739.1uncharacterized protein LOC5578728
AAEL02221	NA	-0.178244338761061	0.578881817625683	0.882283691803025	NA	NA	NA	XP_021703440.1zinc finger protein interacting with ribonucleoprotein K-like
AAEL0135⁴	transcription factor grauzone	-0.178158761123832	0.543782560356025	0.870055542468027	NA	NA	NA	XP_001663739.2transcription factor grauzone
AAEL0142ꞇ	clumping factor B	-0.178134732447081	0.184949461492976	0.612196023258404	NA	NA	NA	XP_019558447.1 clumping factor B-like isoform X4
AAEL0115(	choline-phosphate cytidylyltransferase B	-0.177963309844163	0.149840356084221	0.565429182817541	NA	aag01100 ; aag	NA	XP_011493173.2choline-phosphate cytidylyltransferase B isoform X2
AAEL0082⁴	poly(ADP-ribose) glycohydrolase ARH3	-0.177703512210765	0.314035895584846	0.74451110779113	NA	NA	KFB42807.1 AGAP00	XP_001653130.1poly(ADP-ribose) glycohydrolase ARH3
AAEL0125ꜫ	protein javelin	-0.177561213485617	0.218412900097789	0.651909019364208	NA	NA	KFB50655.1 AGAP00	XP_021697045.1protein javelin isoform X2
AAEL0261⁴	NA	-0.177458875981985	0.572642141173042	0.880923047085115	NA	NA	NA	XP_021694961.1prostaglandin reductase 1
AAEL0050‡	tubulin beta chain	-0.177397280047229	0.124553835601921	0.528664388933982	NA	aag04145	XP_001688163.1 AG.	XP_001650333.1tubulin beta chain isoform X2
AAEL0038ꞇ	glycerol-3-phosphate dehydrogenase, mitochondrial	-0.177217021814432	0.214563626335938	0.648028428468258	564	aag00564	XP_019533361.1 PRE	XP_001664283.1glycerol-3-phosphate dehydrogenase, mitochondrial isoform X2
AAEL0195(	NA	-0.176943161598009	0.164611671763684	0.583247700156998	NA	NA	NA	XP_021693903.1segmentation protein cap'n'collar isoform X1
AAEL01421	nicotinate phosphoribosyltransferase	-0.176844134688014	0.208951169393942	0.639832374753494	760	aag01100 ; aag	NA	XP_021701523.1nicotinate phosphoribosyltransferase isoform X2
AAEL0038ε	beta-1,3-glucan-binding protein	-0.176729241563559	0.263930682272152	0.698501621589975	NA	NA	KXJ68256.1 hypothe‹	XP_001664289.1beta-1,3-glucan-binding protein
AAEL0276ꜫ	NA	-0.176727243181697	0.666616989662451	0.912050766257029	NA	NA	NA	XP_001655572.2uncharacterized protein LOC5575189
AAEL0181⁴	NA	-0.176645342768177	0.455413996703338	0.832617462606238	NA	NA	NA	XP_021696307.1glutamate receptor 1 isoform X1
AAEL0144ε	uncharacterized LOC5564482	-0.176638051099016	0.265131537578252	0.699667453052788	NA	NA	NA	XP_021701938.1uncharacterized protein LOC5564482 isoform X3
AAEL0275ꞇ	NA	-0.176612732392118	0.566686058729539	0.880302387766415	NA	NA	NA	NA
AAEL0183(	NA	-0.176286298195908	0.640161822588868	0.904463241434479	NA	NA	NA	XP_021701444.1zinc finger protein 425
AAEL0034ç	uncharacterized LOC5578278	-0.176116345049912	0.420273921261477	0.813636239562413	NA	NA	KXJ71385.1 hypothe‹	XP_021692945.1uncharacterized protein LOC5578278
AAEL0054(	calbindin-32	-0.176015253743469	0.447241434493899	0.828502725039167	NA	NA	XP_019565498.1 PRE	XP_021708860.1calbindin-32 isoform X1
AAEL0034ꜫ	regulator of microtubule dynamics protein 1	-0.175892346380383	0.200420816876428	0.633770569380376	NA	NA	KXJ76233.1 hypothe‹	XP_001656799.2regulator of microtubule dynamics protein 1 isoform X2
AAEL0205ż	NA	-0.175855765532743	0.330711103057305	0.754128446928769	NA	NA	NA	XP_021699241.1uncharacterized protein LOC5572944 isoform X1
AAEL0032ꞇ	melanization protease 1	-0.175630611750028	0.143914175257286	0.556228061803319	NA	NA	KXJ71594.1 hypothe‹	XP_001656656.2melanization protease 1

AAEL0041(	protein deadpan	-0.175607242424977	0.540609414052419	0.869735874782513	NA	NA	KXJ71291.1 hypothe	XP_001648441.1protein deadpan
AAEL00222	fatty acid synthase	-0.175514156566786	0.209818028801821	0.640592402466607	00780 ; 00061	NA	KXJ74484.1 hypothe	XP_001654963.2fatty acid synthase
AAEL02465	NA	-0.175411076087182	0.647895146057866	0.906554905014882	NA	NA	NA	XP_021707946.1uncharacterized protein LOC5570570
AAEL01451	NA	-0.175122158824692	0.142916838727453	0.555375365647863	NA	NA	NA	XP_001654381.1AN1-type zinc finger protein 6
AAEL00255	WD repeat-containing protein 6	-0.175088878768591	0.556505197495408	0.876744014213565	NA	NA	XP_001850265.1 cor	XP_021713407.1WD repeat-containing protein 6
AAEL02414	NA	-0.175071108549962	0.141999906895877	0.553914007314906	NA	NA	NA	XP_021703940.1paired amphipathic helix protein Sin3a isoform X3
AAEL00065	maltase A3	-0.175008507795881	0.286948752272641	0.718895294966742	NA	NA	KXJ74659.1 hypothe	XP_001649788.1maltase A3
AAEL02433	NA	-0.174724036978696	0.598580221056049	0.890707502289419	NA	NA	NA	XP_021701517.1luciferin 4-monooxygenase
AAEL00912	cytochrome P450 6a8	-0.174236843593291	0.3746336106566	0.782198815065306	NA	NA	NA	XP_001653686.1cytochrome P450 6a8
AAEL01422	uncharacterized LOC5579527	-0.174223862547712	0.399536064832019	0.798648539435638	NA	NA	NA	XP_021710198.1uncharacterized protein LOC5579527 isoform X3
AAEL01491	NA	-0.174006421086633	0.312759995951725	0.744058294402217	NA	NA	NA	XP_001649987.2synaptic vesicle glycoprotein 2B
AAEL01947	NA	-0.173818756319317	0.455365908455306	0.832617462606238	NA	aag04330	NA	XP_021704863.1neurogenic protein mastermind isoform X2
AAEL01179	venom allergen 5	-0.173678930052216	0.143279843300155	0.555375365647863	NA	NA	KXJ79582.1 hypothe	XP_001661919.1venom allergen 5
AAEL00863	calsyntenin-1	-0.173579075123904	0.128814335989638	0.535335481333802	NA	NA	KXJ81032.1 hypothe	XP_019536954.1 calsyntenin-1-like
AAEL01353	cytoplasmic protein NCK1	-0.173492563057748	0.485795571415312	0.847790841886424	NA	NA	NA	XP_021700406.1cytoplasmic protein NCK1
AAEL01106	tubulin-specific chaperone E	-0.173464378315558	0.270171407190848	0.706082729045778	NA	NA	KXJ80200.1 hypothe	XP_021704242.1tubulin-specific chaperone E
AAEL01560	NA	-0.173382756397428	0.529607513259402	0.866236817812481	NA	NA	NA	XP_001647607.2probable chitinase 10
AAEL00300	uncharacterized LOC5580272	-0.173366367710942	0.270632231067089	0.706237745304114	NA	NA	KXJ69984.1 hypothe	XP_001662899.2uncharacterized protein LOC5580272
AAEL01019	FH1/FH2 domain-containing protein 1	-0.173222732663878	0.201440431816356	0.634701781910801	NA	NA	NA	XP_021698420.1serine-rich adhesin for platelets isoform X2
AAEL00403	acetylcholine receptor subunit alpha-like 2	-0.173097214026993	0.521368913314834	0.864991501344855	NA	NA	CRL05582.1 CLUMA_	XP_001648279.1acetylcholine receptor subunit alpha-like 2
AAEL00125	rap1 GTPase-GDP dissociation stimulator 1	-0.173093046104043	0.351894539746704	0.769212685559608	NA	NA	XP_019545833.1 PRE	XP_001652902.2rap1 GTPase-GDP dissociation stimulator 1 isoform X1
AAEL02634	NA	-0.172854435630374	0.623276592760799	0.898271200863489	NA	NA	NA	XP_021697809.1ADP,ATP carrier protein 1-like
AAEL00055	sn1-specific diacylglycerol lipase beta	-0.172853017809392	0.356022714056934	0.772867683747772	NA	NA	ETN64330.1 neural s	XP_021709577.1sn1-specific diacylglycerol lipase beta
AAEL00068	hydroxysteroid dehydrogenase-like protein 1	-0.172751805028455	0.570533033077844	0.880923047085115	NA	NA	KXJ73516.1 hypothe	XP_001650279.2hydroxysteroid dehydrogenase-like protein 1
AAEL00783	chitooligosaccharidolytic beta-N-acetylglucosaminidase	-0.172516687041191	0.47687900937582	0.844867278451347	00513 ; 00603 ; 00604 ;	aag01100 ; aag	KXJ76695.1 hypothe	XP_021693811.1chitooligosaccharidolytic beta-N-acetylglucosaminidase
AAEL01554	NA	-0.172479104356112	0.287696851253214	0.719789675158259	NA	NA	NA	XP_019529308.1 zinc finger protein 449-like
AAEL01305	uncharacterized LOC5577181	-0.172468439528085	0.609595712940068	0.893659351676678	NA	NA	NA	XP_001663269.2uncharacterized protein LOC5577181
AAEL02449	NA	-0.172399954201886	0.498613607441985	0.856678287334277	NA	NA	NA	XP_021698498.1gamma-aminobutyric acid receptor subunit alpha-4 isoform X4
AAEL02457	NA	-0.172301598204501	0.219376436219055	0.652412844728166	NA	NA	NA	XP_021697012.1uncharacterized protein LOC5574802
AAEL00499	probable ribosome production factor 1	-0.172008493180904	0.191484827261841	0.621532056600868	NA	NA	XP_001850750.1 ribo	XP_001650128.2probable ribosome production factor 1
AAEL00947	CAD protein	-0.171996530709976	0.14379208651788	0.556189483033851	00240 ; 00250 ; 00240 ;	aag01100 ; aag	NA	XP_021698953.1CAD protein
AAEL00372	lysozyme-like	-0.171982368781849	0.316051758677903	0.745684464362801	NA	NA	KXJ81084.1 hypothe	CAC19819.1putative lysozyme
AAEL02527	NA	-0.171883770855388	0.373700547152547	0.782198815065306	NA	NA	NA	NA
AAEL01347	LYR motif-containing protein 2	-0.17187244898479	0.407064712908927	0.803535299237735	NA	NA	NA	XP_001662421.2LYR motif-containing protein 2
AAEL00776	dipeptidyl peptidase 4	-0.171560636117009	0.129034350967004	0.535335481333802	NA	NA	XP_019537022.1 PRE	XP_021699731.1dipeptidyl peptidase 4 isoform X1
AAEL01821	NA	-0.17133915154509	0.138431371797293	0.548805616375239	04660 ; 04658	NA	NA	XP_021700082.1uncharacterized protein LOC5574376
AAEL00079	venom allergen 3	-0.171230718154026	0.27441288029079	0.710214476791569	NA	NA	KXJ75435.1 hypothe	XP_001651000.1venom allergen 3
AAEL00361	sodium-dependent nutrient amino acid transporter 1	-0.171103946618026	0.437869241669569	0.820960220424486	NA	NA	KXJ83117.1 hypothe	XP_021711080.1 sodium-dependent nutrient amino acid transporter 1
AAEL00633	apyrase	-0.17100634280246	0.275473566777824	0.711001645592863	NA	NA	KXJ70935.1 hypothe	XP_019556938.1 apyrase-like
AAEL00361	uncharacterized LOC5578695	-0.170946703123649	0.648820084354853	0.906554905014882	NA	NA	KXJ73774.1 hypothe	XP_001657094.2uncharacterized protein LOC5578695 isoform X1
AAEL00837	dipeptidyl peptidase 9	-0.170767166765958	0.180774277859528	0.606226277282573	NA	NA	XP_001862519.1 dip	XP_021710715.1dipeptidyl peptidase 9
AAEL01830	NA	-0.170710095099343	0.385419477653387	0.790599650772731	NA	NA	NA	XP_021693166.1calcium-activated potassium channel slowpoke isoform X19
AAEL00573	protein yellow	-0.170700819952761	0.318185256379732	0.745684464362801	NA	NA	ETN67900.1 yellow p	XP_001651385.1protein yellow
AAEL00093	alkylated DNA repair protein alkB homolog 8	-0.170637618384433	0.606342502190112	0.893547770777888	NA	NA	KFB50078.1 AGAP01	XP_001657457.1alkylated DNA repair protein alkB homolog 8
AAEL00947	peptidoglycan-recognition protein 2	-0.170536149321101	0.327747903539522	0.750660774006273	NA	NA	NA	XP_001660103.3peptidoglycan-recognition protein 2
AAEL01445	peptidyl-alpha-hydroxyglycine alpha-amidating lyase 2	-0.170441929139046	0.221599038386554	0.654067488761644	NA	NA	NA	XP_021697397.1peptidyl-alpha-hydroxyglycine alpha-amidating lyase 2
AAEL02473	NA	-0.170353224487312	0.553361676257398	0.874619399677186	NA	NA	NA	XP_021696208.1sodium-dependent dopamine transporter isoform X2
AAEL02310	NA	-0.170335604412968	0.264201766933932	0.698659663885272	NA	NA	NA	XP_021704761.1SH3 and multiple ankyrin repeat domains protein 1 isoform X4
AAEL02147	NA	-0.17024908910712	0.601073101677338	0.892108819618526	NA	NA	NA	XP_021711905.1zinc finger protein 2 homolog
AAEL00021	D-amino-acid oxidase	-0.169883573485565	0.298150469168488	0.7302583490254	NA	aag01100 ; aag	KXJ81152.1 hypothe	XP_001658885.2D-amino-acid oxidase
AAEL02022	NA	-0.169873284227876	0.201715959060714	0.634701781910801	NA	NA	NA	NA
AAEL02901	NA	-0.169812281187973	0.250314257223596	0.683335640523162	NA	NA	NA	XP_001660228.2apolipoprotein D isoform X2
AAEL01996	NA	-0.16953960408626	0.68332494401073	0.914629465876247	NA	NA	NA	XP_021699370.1uncharacterized protein LOC5571050 isoform X1
AAEL02677	NA	-0.169456539799648	0.265078776342764	0.699667453052788	NA	NA	NA	NA
AAEL00731	hillarin	-0.169272071876791	0.422428513060018	0.814192545906728	NA	NA	KXJ81329.1 hypothe	XP_019546113.1 hillarin
AAEL00343	NA	-0.169105746914681	0.431978112881509	0.820005280371151	NA	NA	KFB38240.1 Dwil\GK	KFB38240.1Dwil\GK12675-like protein
AAEL00212	venom serine protease Bi-VSP	-0.169099853626987	0.153749209920578	0.569537415169523	NA	NA	KXJ73780.1 hypothe	XP_001654732.1venom serine protease Bi-VSP
AAEL01163	inactive peptidyl-prolyl cis-trans isomerase shutdown	-0.169027259590805	0.252999608255412	0.685139021532167	NA	NA	KXJ68208.1 hypothe	XP_001661808.2inactive peptidyl-prolyl cis-trans isomerase shutdown
AAEL01276	leucine-rich repeats and immunoglobulin-like domains protein sma-10	-0.169007145612726	0.359362201419225	0.774255626424296	NA	NA	KXJ76759.1 hypothe	XP_001662875.2leucine-rich repeats and immunoglobulin-like domains protein sma-10 isoform X2
AAEL00442	ATP synthase subunit d, mitochondrial	-0.168899147813905	0.221226666256702	0.653524167863296	NA	aag01100 ; aag	ETN64697.1 ATP syn	XP_001649125.1ATP synthase subunit d, mitochondrial
AAEL02491	NA	-0.168857403747521	0.221694903004517	0.654067488761644	NA	NA	NA	EAT33931.1AAEL013800
AAEL00614	dynactin subunit 1	-0.168769325126296	0.151368710721985	0.566217429294191	NA	NA	NA	XP_001657515.1dynactin subunit 1 isoform X8
AAEL00452	transducin beta-like protein 3	-0.168764285017695	0.305365037616272	0.738299809812121	NA	aag03008	KXJ78992.1 hypothe	XP_021701383.1transducin beta-like protein 3
AAEL01812	NA	-0.168309266565311	0.290539293086791	0.722005075789413	04151 ; 05165 ; 04714 ;	aag04150 ; aag	NA	XP_001650653.2ribosomal protein S6 kinase beta-2 isoform X1
AAEL00922	kinesin-like protein KIF18A	-0.167829705804172	0.653483039478987	0.907272485895905	NA	NA	NA	XP_001659844.2kinesin-like protein KIF18A isoform X1
AAEL00526	cytochrome b-c1 complex subunit 2, mitochondrial	-0.167346295467736	0.240676054143658	0.675511149976115	NA	aag01100 ; aag	KXJ78639.1 hypothe	XP_001650609.2cytochrome b-c1 complex subunit 2, mitochondrial
AAEL00438	O-phosphoseryl-tRNA(Sec) selenium transferase	-0.167341065850592	0.569735803616288	0.880635379772131	00450 ; 00970	aag01100 ; aag	XP_019540680.1 PRE	XP_001649002.2O-phosphoseryl-tRNA(Sec) selenium transferase isoform X1
AAEL00040	translation machinery-associated protein 16 homolog	-0.167292331942678	0.413392104661244	0.808061732662297	NA	NA	XP_001848638.1 con	XP_001656418.2translation machinery-associated protein 16 homolog
AAEL02374	NA	-0.166914994781221	0.427779379192999	0.816177865920377	NA	aag04320 ; aag	NA	XP_021693722.1neurogenic locus Notch protein
AAEL00283	CDK5RAP1-like protein	-0.166444730548325	0.343837544265493	0.764585185963472	NA	NA	KXJ75133.1 hypothe	XP_021706267.1CDK5RAP1-like protein
AAEL00850	T-cell activation inhibitor, mitochondrial	-0.166298766905062	0.391899504220593	0.793285976044306	NA	NA	KXJ67856.1 hypothe	XP_001659285.2T-cell activation inhibitor, mitochondrial
AAEL02120	NA	-0.166248854075112	0.675795396558272	0.914438668430353	NA	NA	NA	XP_021694148.1sushi, von Willebrand factor type A, EGF and pentraxin domain-containing protein 1
AAEL02694	NA	-0.166169007107003	0.339912006955781	0.761379832492257	NA	NA	NA	XP_021707136.1protein tincar
AAEL01707	esterase B1	-0.166028584380073	0.330894880279517	0.754128446928769	NA	NA	NA	XP_011493543.2esterase B1
AAEL00664	tubulin alpha-1 chain	-0.166003076602538	0.165333573538181	0.584439853413553	NA	aag04145	ETN58805.1 tubulin a	XP_001652144.1tubulin alpha-1 chain
AAEL02552	NA	-0.165812414772345	0.648100343206705	0.906554905014882	NA	NA	NA	XP_001656074.1 TFIIH basal transcription factor complex helicase XPD subunit
AAEL00317	NA	-0.165806870305789	0.583590150916699	0.885175928718656	NA	NA	KNE88873.1 hypothe	XP_021700507.1uncharacterized protein LOC5577416
AAEL02068	NA	-0.165709088192027	0.153803572777251	0.569537415169523	NA	NA	NA	XP_021698161.1activating transcription factor 7-interacting protein 1
AAEL01320	post-GPI attachment to proteins factor 2	-0.165519499640614	0.60125473681452	0.892138904371716	NA	NA	NA	XP_001663392.2post-GPI attachment to proteins factor 2
AAEL00786	12 kDa FK506-binding protein	-0.165129177411245	0.1476887455028	0.562280445228287	NA	NA	ETN66228.1 fk506-bi	XP_001652969.112 kDa FK506-binding protein
AAEL00283	oxidative stress-induced growth inhibitor 2	-0.164994967372509	0.171217047106506	0.592753674745698	NA	NA	ETN65591.1 hypothe	XP_021708985.1oxidative stress-induced growth inhibitor 2 isoform X2
AAEL00852	tyrosine-protein phosphatase corkscrew	-0.164781059339386	0.156602416427684	0.574474189691589	NA	aag04013	XP_019548134.1 PRE	XP_001659363.2tyrosine-protein phosphatase corkscrew isoform X1

AAEL01027	polyserase-2	-0.164527951143445	0.671578125071129	0.912812708172959	NA	NA	NA	XP_019544424.1 polyserase-2-like
AAEL00224	signal transducing adapter molecule 2	-0.164470324011551	0.169187729232502	0.589285695468509	NA	aag04144	XP_019551341.1 PRE	XP_001654992.2signal transducing adapter molecule 2
AAEL01973	NA	-0.164444727173894	0.127521114048077	0.532418008834123	230	aag04391	NA	XP_021694498.1disks large 1 tumor suppressor protein isoform X1
AAEL00973	probable serine/threonine-protein kinase yakA	-0.164342697564151	0.480982780445977	0.846272188463456	NA	NA	NA	XP_021696820.1probable serine/threonine-protein kinase yakA isoform X1
AAEL02084	NA	-0.164290939027861	0.154844308392101	0.5722398898892	NA	NA	NA	XP_021698123.1uncharacterized protein LOC5568139
AAEL01811	NA	-0.164216214779214	0.145524296761149	0.558950568057442	NA	NA	NA	ABF18040.1putative salivary secreted mucin 3
AAEL02481	NA	-0.163897759991955	0.409065235360578	0.805352652072751	NA	NA	NA	XP_021703270.1integrator complex subunit 1
AAEL02764	NA	-0.163771692694438	0.286726180026861	0.718895294966742	NA	NA	NA	XP_021710599.1partner of xrn-2 protein 1-like
AAEL01393	mitogen-activated protein kinase ERK-A	-0.163752515298994	0.397914510812883	0.797539006884967	4150	aag04013 ; aag	NA	XP_021702009.1mitogen-activated protein kinase 1 isoform X1
AAEL00896	NA	-0.163614883603529	0.268100990819287	0.704184172798867	00960 ; 00400 ; 00350 ;	aag01100 ; aag	KXJ73193.1 hypothet	XP_001653614.2tyrosine aminotransferase
AAEL02604	NA	-0.163348688445652	0.281433381382082	0.715107379448722	NA	aag03040	NA	XP_021697878.1ATP-dependent RNA helicase DHX8
AAEL00467	uncharacterized LOC5565211	-0.163283147727473	0.658974906979324	0.909580313002131	NA	NA	XP_001868748.1 con	XP_021710883.1uncharacterized protein LOC5565211
AAEL01171	migration and invasion enhancer 1	-0.163239624135192	0.210845296434024	0.64308863698571	NA	NA	KXJ78467.1 hypothet	XP_001661860.2migration and invasion enhancer 1
AAEL01163	THO complex subunit 5 homolog B	-0.163227411954583	0.415424821130367	0.809573481450831	NA	aag03013	KXJ68389.1 hypothet	XP_001661802.1THO complex subunit 5 homolog B
AAEL00853	transcription factor grauzone	-0.163212332149843	0.535160935829184	0.866998403433126	NA	NA	KXJ68030.1 hypothet	XP_001659341.2transcription factor grauzone
AAEL00671	sugar transporter SWEET1	-0.163133387619817	0.191922180668848	0.622073936778705	NA	NA	KXJ81784.1 hypothet	XP_001652204.1sugar transporter SWEET1
AAEL01830	NA	-0.163070396443678	0.578514550109386	0.882283691803025	NA	NA	NA	XP_021712981.1transcription factor grauzone
AAEL01945	NA	-0.162914092669912	0.193879447841264	0.624986606780741	NA	NA	NA	XP_021696732.1titin homolog isoform X7
AAEL00289	uncharacterized LOC5576462	-0.162747623306927	0.598051278619367	0.890496418423206	NA	NA	NA	XP_021700312.1uncharacterized protein LOC5576462 isoform X3
AAEL01185	cytochrome P450 315a1, mitochondrial	-0.162617336940309	0.568559512686219	0.880635379772131	NA	aag00981	XP_019555634.1 PRE	XP_001661987.3cytochrome P450 315a1, mitochondrial
AAEL00769	regulatory-associated protein of mTOR	-0.162570692093858	0.302574331852952	0.735250294168217	NA	aag04150 ; aag	ETN67147.1 regulato	XP_001658591.1regulatory-associated protein of mTOR
AAEL02588	NA	-0.162545098874267	0.664057258846342	0.911592424421865	NA	NA	NA	XP_021711615.1uncharacterized protein LOC5566000
AAEL00689	dnaJ protein homolog 1	-0.162528668172723	0.205391980725622	0.638023927878659	NA	NA	KFB45481.1 AGAP01	XP_001658074.1dnaJ protein homolog 1
AAEL02059	NA	-0.162470797753748	0.185635128301395	0.612922300189966	NA	aag04146	NA	XP_021698930.1peroxisomal targeting signal 1 receptor
AAEL00547	calpain-7	-0.162422052208237	0.52098219352465	0.864960891721031	NA	NA	KXJ73724.1 hypothet	XP_019539753.1 calpain-7-like
AAEL00387	proteasome subunit alpha type-3	-0.162417923870463	0.205832537093089	0.638023927878659	NA	aag03050	KFB37456.1 AGAP00	XP_001664262.1proteasome subunit alpha type-3
AAEL00312	isovaleryl-CoA dehydrogenase, mitochondrial	-0.162298652943457	0.231085184695825	0.66425254967488	280	aag01100 ; aag	KXJ80523.1 hypothet	XP_001663227.2isovaleryl-CoA dehydrogenase, mitochondrial
AAEL01097	protein ELYS homolog	-0.161959619039815	0.38748773112504	0.791825725928371	NA	NA	KXJ72266.1 hypothet	XP_001661219.1protein ELYS homolog isoform X1
AAEL02564	NA	-0.161900474788199	0.222097394769332	0.654118491874251	NA	aag03040 ; aag	NA	XP_021709332.1heat shock 70 kDa protein cognate 2
AAEL01298	solute carrier family 22 member 8	-0.161810459879346	0.292717637246036	0.723512283028756	NA	NA	NA	XP_001663173.2solute carrier family 22 member 8
AAEL02737	NA	-0.161536521600856	0.670063924227121	0.912812708172959	NA	NA	NA	XP_021707798.1zinc finger protein 600
AAEL01962	NA	-0.161494886398923	0.371996975337569	0.781817769662763	NA	NA	NA	XP_001662365.2protein GPR107 isoform X2
AAEL00718	nocturnin	-0.161480072790687	0.149328800245322	0.564785704832493	NA	NA	NA	XP_021701475.1nocturnin isoform X3
AAEL00285	serine/threonine-protein phosphatase 2A regulatory subunit B'' subunit g	-0.161351573433806	0.659713986765872	0.909580313002131	NA	aag03015	KXJ74192.1 hypothet	XP_001656002.1serine/threonine-protein phosphatase 2A regulatory subunit B'' subunit gamma
AAEL00570	clavesin-2	-0.161294312261328	0.503911794493319	0.858744381072989	NA	NA	KFB46427.1 hypothe	XP_019560280.1 clavesin-2-like
AAEL00025	scavenger receptor class B member 1	-0.161218330364818	0.254868212188409	0.686964642225467	NA	NA	ACZ58351.1 epithelia	XP_001660151.1scavenger receptor class B member 1 isoform X1
AAEL02621	NA	-0.161161130396958	0.313571348277824	0.744448799497558	NA	NA	NA	XP_001846781.1ATP-binding cassette transporter
AAEL00948	uncharacterized LOC5572009	-0.161154098356504	0.514174561814765	0.861448202130317	NA	NA	NA	XP_001660115.2uncharacterized protein LOC5572009
AAEL01227	survival of motor neuron-related-splicing factor 30	-0.161041838089792	0.278909484444644	0.713552892930006	NA	aag03040	ETN67198.1 survival	XP_001662376.1survival of motor neuron-related-splicing factor 30
AAEL01344	toll-like receptor 13	-0.16102077107501	0.372698721614789	0.781817769662763	NA	NA	NA	XP_021698584.1toll-like receptor 13 isoform X1
AAEL02587	NA	-0.160903440490796	0.205634348420459	0.638023927878659	NA	NA	NA	NA
AAEL02374	NA	-0.160812478922372	0.505420142041592	0.858744381072989	NA	NA	NA	XP_021706128.1trophoblast glycoprotein
AAEL01345	glutamine synthetase 2 cytoplasmic	-0.160785370095319	0.157898586734307	0.575615420985807	00250 ; 00220 ; 00910 ;	aag01100 ; aag	NA	XP_001656718.1glutamine synthetase 2 cytoplasmic isoform X2
AAEL00304	netrin-B	-0.16075560576985	0.305295810047541	0.738299809812121	NA	NA	XP_019548443.1 PRE	XP_019548443.1 netrin-B
AAEL01413	serine protease inhibitor 28Dc	-0.160473404893194	0.488135461841676	0.848846834445739	NA	NA	NA	XP_001648088.2serine protease inhibitor 28Dc
AAEL01058	glutathione S-transferase 1-1	-0.160429089606356	0.444726436137861	0.826574967752089	NA	NA	KXJ77065.1 hypothet	XP_001654674.2glutathione S-transferase 1-1
AAEL00470	protein phosphatase 1L	-0.160423115060609	0.152729702220942	0.568113296551786	04660 ; 04658	NA	KXJ74686.1 hypothet	XP_001649651.1protein phosphatase 1L isoform X1
AAEL02422	NA	-0.160263223450047	0.220717340279361	0.652797615862164	00680 ; 00010	aag01100 ; aag	NA	XP_021701188.1 enolase
AAEL01217	ATP synthase subunit alpha, mitochondrial	-0.160242182099478	0.284234471045129	0.716726857594605	00195 ; 00190	aag01100 ; aag	KFB46888.1 AGAP00	XP_001655906.1ATP synthase subunit alpha, mitochondrial
AAEL00972	proclotting enzyme	-0.16019742465426	0.186341329615959	0.614196367514984	NA	NA	NA	XP_001654027.2proclotting enzyme
AAEL00750	homeobox protein araucan	-0.160133139662174	0.450368936352635	0.829854816242881	NA	NA	KFB48386.1 AGAP00	XP_021699828.1homeobox protein araucan
AAEL00071	rho guanine nucleotide exchange factor 7	-0.159976386365675	0.197794106742148	0.631103110347174	NA	NA	XP_019561040.1 PRE	XP_021703844.1rho guanine nucleotide exchange factor 7 isoform X4
AAEL00712	zwei Ig domain protein zig-8	-0.159929481160717	0.666643496396955	0.912050766257029	NA	NA	KXJ80015.1 hypothet	XP_021700717.1zwei Ig domain protein zig-8
AAEL01213	cationic amino acid transporter 3	-0.159740143837679	0.284347140978162	0.716726857594605	NA	NA	AKN80693.1 cationic	XP_001662274.1cationic amino acid transporter 3
AAEL01057	modifier of mdg4	-0.15961450570131	0.207960495236168	0.639832374753494	NA	NA	NA	XP_021693970.1modifier of mdg4 isoform X8
AAEL00028	ankyrin repeat, SAM and basic leucine zipper domain-containing protein :	-0.159591955911965	0.510270960727801	0.860452247709668	NA	NA	KXJ81835.1 hypothet	XP_001654775.2ankyrin repeat, SAM and basic leucine zipper domain-containing protein 1
AAEL01325	dendritic arbor reduction protein 1	-0.159477379125496	0.491420459908901	0.851610371390151	NA	NA	NA	XP_001656564.1homeotic protein female sterile isoform X1
AAEL01086	syndetin	-0.159463375911742	0.468612912119031	0.840088840311577	NA	NA	XP_001862996.1 con	XP_019558629.1 syndetin
AAEL01187	transmembrane protein 134	-0.159413531838551	0.671664295483493	0.912812708172959	NA	NA	XP_001849687.1 con	XP_001662016.1transmembrane protein 134 isoform X2
AAEL02517	NA	-0.159364351833491	0.189718220532152	0.618196552881326	00270 ; 00330	NA	NA	XP_021712696.1S-adenosylmethionine decarboxylase proenzyme-like
AAEL02907	NA	-0.159110977280706	0.325919769764295	0.748896673349414	NA	NA	NA	XP_021694530.1serine protease snake-like isoform X1
AAEL01220	E3 ubiquitin-protein ligase RING1	-0.159042270164993	0.642039703403727	0.905725793457166	NA	NA	XP_001851087.1 E3 (	XP_001655936.1E3 ubiquitin-protein ligase RING1
AAEL01263	uncharacterized LOC5576608	-0.159030727645971	0.289156543022338	0.721217831038796	NA	NA	KXJ82886.1 hypothet	XP_019548654.1 ATPase family AAA domain-containing protein 5
AAEL00014	single-stranded DNA-binding protein, mitochondrial	-0.158882456679829	0.482324475553198	0.846941547268585	NA	aag03030 ; aag	KXJ68309.1 hypothet	XP_001657692.1single-stranded DNA-binding protein, mitochondrial
AAEL02371	NA	-0.158753868925386	0.533421196416426	0.866700945213732	NA	NA	NA	XP_021706918.1synapse-associated protein of 47 kDa isoform X4
AAEL01471	adiponectin receptor protein	-0.15868954929419	0.155273339449572	0.573091811919643	NA	NA	NA	XP_001649426.1adiponectin receptor protein
AAEL01361	slit homolog 1 protein	-0.158626844300532	0.185529763193963	0.612794286041223	NA	NA	NA	XP_001656882.1neurogenic locus notch homolog protein 3 isoform X1
AAEL00396	chromatin-remodeling complex ATPase chain Iswi	-0.158598339853071	0.355382661034381	0.772799644918615	NA	NA	KFB51779.1 AGAP01	XP_001648130.1chromatin-remodeling complex ATPase chain Iswi
AAEL01303	serine protease snake	-0.158502135674515	0.181401179046607	0.607780282383509	NA	NA	NA	XP_019530559.1 polyserase-2-like
AAEL01527	NA	-0.158205682782861	0.507788826051061	0.859709016442863	NA	NA	NA	XP_001647790.2mitochondrial import inner membrane translocase subunit Tim21
AAEL00803	protein msta	-0.158140168539189	0.496270850881922	0.854864165072155	NA	NA	KXJ74253.1 hypothet	XP_001658830.2protein msta
AAEL00837	protein peste	-0.158032686183485	0.194290442981387	0.625563560641961	NA	NA	KXJ81808.1 hypothet	XP_021709728.1protein peste
AAEL00283	cathepsin L	-0.158021861203544	0.229951016654337	0.662910167685841	NA	aag04140 ; aag	XP_019552504.1 PRE	XP_001655999.2cathepsin L
AAEL01213	run domain Beclin-1-interacting and cysteine-rich domain-containing pro	-0.158004974947995	0.408163310111101	0.8040930806342	NA	aag04140	KXJ84056.1 hypothet	XP_001655872.2run domain Beclin-1-interacting and cysteine-rich domain-containing protein
AAEL01957	NA	-0.157941315599421	0.226600486272798	0.660446829146891	NA	NA	NA	XP_021704635.1neurobeachin-like protein 1 isoform X2
AAEL00347	methyltransferase-like protein 22	-0.157791222332367	0.552527950240118	0.874531710113044	NA	NA	KXJ70022.1 hypothet	XP_011493735.2methyltransferase-like protein 22
AAEL00991	putative peptidyl-prolyl cis-trans isomerase dodo	-0.15773242057804	0.339889120664474	0.761379832492257	NA	NA	NA	XP_001654110.2putative peptidyl-prolyl cis-trans isomerase dodo
AAEL01128	cytochrome b561	-0.157533119922577	0.175131304074022	0.598647199141587	NA	NA	XP_019535596.1 PRE	XP_001661561.2cytochrome b561 isoform X2
AAEL01022	30 kDa salivary gland allergen Aed a 3-like	-0.157338498791441	0.373359653861411	0.782192290591489	NA	NA	NA	XP_001660690.230 kDa salivary gland allergen Aed a 3-like
AAEL00816	malate dehydrogenase, mitochondrial	-0.157320962260265	0.223104442807949	0.655580227356264	00620 ; 00680 ; 00630 ;	aag01100 ; aag	KXJ74736.1 hypothet	XP_001659012.2malate dehydrogenase, mitochondrial
AAEL00139	mitochondrial enolase superfamily member 1	-0.157292887770416	0.534911762868657	0.866763821535675	51	aag01100 ; aag	XP_001864185.1 ma	XP_001659176.1mitochondrial enolase superfamily member 1
AAEL00443	kynurenine--oxoglutarate transaminase 3	-0.157144658754487	0.237830119846327	0.672545904319684	00450 ; 00380 ; 00270	aag01100 ; aag	1YIY_A Chain A, A. Ae	XP_001649143.1kynurenine--oxoglutarate transaminase 3

AAEL00018	cytochrome b-c1 complex subunit 9	-0.157087719716357	0.258958751509699	0.691547675112889	NA	aag01100 ; aag	ETN60487.1 ATP-dep	XP_001658910.1cytochrome b-c1 complex subunit 9
AAEL00702	tropomodulin	-0.156908311501133	0.226618163859356	0.660446829146891	NA	NA	XP_019552023.1 PRE	XP_001658087.2tropomodulin isoform X11
AAEL00482	NADH dehydrogenase [ubiquinone] 1 alpha subcomplex subunit 5	-0.156902850075348	0.230330476835467	0.663173043754683	NA	aag01100 ; aag	XP_001649896.2 NAI	XP_001649896.2NADH dehydrogenase [ubiquinone] 1 alpha subcomplex subunit 5
AAEL00227	COP9 signalosome complex subunit 3	-0.1568346568334	0.20228889787436	0.635311412368553	NA	NA	ETN65224.1 26S prot	XP_001661171.1COP9 signalosome complex subunit 3
AAEL02070	NA	-0.15682497351498	0.515049609611852	0.861448202130317	NA	NA	NA	XP_021694174.1uncharacterized protein LOC5579161
AAEL00252	perlucin-like protein	-0.156743939105138	0.437213457029789	0.820960220424486	NA	NA	XP_019526315.1 PRE	XP_001655474.2perlucin-like protein
AAEL00500	CBL-interacting protein kinase 18	-0.156677003075581	0.21549388547799	0.64825542223124	NA	NA	KXJ70580.1 hypothet	XP_021705217.1CBL-interacting protein kinase 18
AAEL02814	NA	-0.156552149770891	0.531100204022673	0.866691320214768	NA	NA	NA	XP_021695752.1HEAT repeat-containing protein 6 isoform X2
AAEL02666	NA	-0.15632363580052	0.432684606667892	0.820005280371151	NA	NA	NA	XP_021705244.1quinone oxidoreductase-like protein 2 homolog
AAEL02543	NA	-0.156161646322973	0.401424563687725	0.799028517208403	NA	aag04013 ; aag	NA	XP_021713061.1tumor necrosis factor receptor superfamily member wengen
AAEL00522	very-long-chain (3R)-3-hydroxyacyl-CoA dehydratase hpo-8	-0.156079822637148	0.516316726621545	0.861448202130317	62	aag01100 ; aag	KXJ76451.1 hypothet	XP_001650522.1very-long-chain (3R)-3-hydroxyacyl-CoA dehydratase hpo-8
AAEL01111	histone deacetylase 3	-0.156015527831054	0.398161457250581	0.797605077871316	NA	NA	ETN59692.1 histone	XP_001655108.1histone deacetylase 3
AAEL01335	uncharacterized LOC5577721	-0.155722268665314	0.169076045547713	0.589285695468509	NA	NA	NA	XP_011493717.1uncharacterized protein LOC5577721 isoform X1
AAEL01123	connectin	-0.155507255678985	0.531203982396359	0.866691320214768	NA	NA	KXJ79693.1 hypothet	XP_019545096.1 connectin-like
AAEL01203	sodium-independent sulfate anion transporter	-0.155446582568702	0.463570812001094	0.837274753622609	NA	NA	KXJ78344.1 hypothet	XP_001662165.1sodium-independent sulfate anion transporter
AAEL00683	transient receptor potential cation channel protein painless	-0.155429231512478	0.338698371600327	0.760778306749309	NA	NA	XP_001849122.1 trai	XP_001652261.2transient receptor potential cation channel protein painless
AAEL00765	putative vitellogenin receptor	-0.155321940901832	0.320538306336947	0.746543531995086	NA	NA	XP_019562857.1 PRE	XP_001658550.2putative vitellogenin receptor
AAEL00416	uncharacterized LOC5564218	-0.15525319420149	0.485931405391717	0.847790841886424	NA	NA	CRK95369.1 CLUMA_	CRK95369.1CLUMA_CG008673, isoform A
AAEL01196	cryptochrome-1	-0.155210070068535	0.239892258432819	0.674660318489854	NA	NA	KXJ75191.1 hypothet	XP_019560995.1 cryptochrome-1-like
AAEL01955	NA	-0.155200624536149	0.371370866704287	0.781817769662763	NA	NA	NA	XP_021701712.1cysteine-rich with EGF-like domain protein 2 isoform X2
AAEL00525	flocculation protein FLO11	-0.155161993265891	0.463912698392709	0.837274753622609	NA	NA	KXJ82226.1 hypothet	XP_021696854.1flocculation protein FLO11
AAEL00504	E3 ubiquitin-protein ligase siah-1	-0.155062380806691	0.332574859161152	0.755231182999982	NA	NA	KXJ71249.1 hypothet	XP_001650197.1E3 ubiquitin-protein ligase siah-1
AAEL01802	NA	-0.154971167472027	0.591231332188582	0.888297094263466	NA	NA	NA	XP_021706498.1uncharacterized protein LOC110678183
AAEL02775	NA	-0.154885192025187	0.295192830390862	0.72668925234262	NA	NA	NA	XP_021710899.1tubulin polyglutamylase TTLL5 isoform X2
AAEL01511	NA	-0.154841119742675	0.343334591438102	0.763685015811799	NA	NA	NA	XP_021697982.1la-related protein CG11505 isoform X2
AAEL01832	NA	-0.15479605489269	0.246265658863422	0.679908968755239	NA	NA	NA	XP_021702652.1uncharacterized protein LOC5564557 isoform X2
AAEL00517	phosphatidate phosphatase LPIN3	-0.154779427961275	0.169140021158704	0.589285695468509	00564 ; 00600 ; 00565 ;	aag01100 ; aag	KXJ83398.1 hypothet	XP_021695358.1phosphatidate phosphatase LPIN3
AAEL01423	aromatic-L-amino-acid decarboxylase	-0.154697870683083	0.280013865788742	0.714159532424614	NA	aag01100 ; aag	NA	XP_021702972.1aromatic-L-amino-acid decarboxylase isoform X1
AAEL01156	hemicentin-1	-0.154591533694421	0.201467138648356	0.634701781910801	NA	NA	KXJ69597.1 hypothet	XP_019544097.1 hemicentin-1-like
AAEL01524	membrane alanyl aminopeptidase	-0.154407711457099	0.252411610750756	0.684556086619159	NA	NA	NA	XP_021712940.1membrane alanyl aminopeptidase
AAEL01078	alpha-2-macroglobulin receptor-associated protein	-0.154270168862636	0.313321524787363	0.744189298893767	NA	NA	KXJ71857.1 hypothet	XP_001654906.1alpha-2-macroglobulin receptor-associated protein
AAEL02102	NA	-0.154160887364663	0.681332578815659	0.914629465876247	NA	NA	NA	XP_021703090.1beta-1,4-galactosyltransferase 2-like
AAEL00367	cytochrome b-c1 complex subunit Rieske, mitochondrial	-0.15410277795659	0.280578587594515	0.714609510945122	NA	aag01100 ; aag	XP_019526567.1 PRE	XP_001657170.1cytochrome b-c1 complex subunit Rieske, mitochondrial isoform X1
AAEL02364	NA	-0.154005308211234	0.341435850057132	0.762180211699347	NA	NA	NA	XP_021710051.1uncharacterized protein LOC5578084 isoform X1
AAEL02113	NA	-0.154001901095405	0.646919083370568	0.906554905014882	NA	NA	NA	XP_021712767.1uncharacterized protein LOC110681316
AAEL01414	CDP-diacylglycerol--inositol 3-phosphatidyltransferase	-0.153945250877784	0.209921185398179	0.640694775467343	00562 ; 00564 ; 04070	aag01100 ; aag	NA	XP_001648095.1CDP-diacylglycerol--inositol 3-phosphatidyltransferase
AAEL00818	arrestin domain-containing protein 2	-0.153787765998506	0.725414746534075	0.923145691827764	NA	NA	KXJ68733.1 hypothet	XP_001659001.1arrestin domain-containing protein 2
AAEL00306	semaphorin-1A	-0.153744441139774	0.379486644522223	0.785666748907449	NA	NA	XP_019539389.1 PRE	XP_021699594.1semaphorin-1A isoform X1
AAEL02347	NA	-0.153516134459118	0.370801267671817	0.781817769662763	NA	NA	NA	XP_021707466.1nucleolar protein 9
AAEL00113	serine/threonine-protein kinase BRSK2	-0.153495575904128	0.70305016456379	0.917134620685568	NA	NA	ETN57911.1 br serine	XP_001652422.2serine/threonine-protein kinase BRSK2
AAEL00882	interference hedgehog	-0.153392602378403	0.521559189639234	0.865018698932415	NA	aag04341	NA	XP_021707743.1interference hedgehog isoform X3
AAEL00695	transcription elongation factor SPT6	-0.153354499653773	0.181747830257279	0.607917241925556	NA	NA	XP_019534123.1 PRE	XP_001652370.2transcription elongation factor SPT6 isoform X2
AAEL00815	arrestin domain-containing protein 2	-0.153231759652436	0.497611856923892	0.856052403704179	NA	NA	XP_019534915.1 PRE	XP_001658997.1arrestin domain-containing protein 2
AAEL00284	tubulin beta-3 chain	-0.152976038245002	0.399831543772696	0.798648539435638	NA	aag04145	KFB47389.1 AGAP01	XP_019931448.1 tubulin beta-3 chain
AAEL00030	heat shock protein 75 kDa, mitochondrial	-0.152908337207683	0.16928190478161	0.589323327862049	NA	NA	KXJ79416.1 hypothet	XP_001654758.1heat shock protein 75 kDa, mitochondrial
AAEL00224	uncharacterized LOC5573898	-0.152811431328518	0.676480314068668	0.914438668430353	NA	NA	KXJ78322.1 hypothet	XP_019564388.1 glycine N-acyltransferase-like protein 3
AAEL00984	trypsin-1	-0.152719578193217	0.296627036689184	0.728660435561632	NA	NA	NA	XP_019559198.1 trypsin-1
AAEL00003	serine protease easter	-0.152683490512531	0.320872878430346	0.746566934846029	NA	NA	AAR88077.1 serine p	XP_001647873.3serine protease easter
AAEL00203	cytochrome P450 CYP12A2	-0.152656918280724	0.198673407547481	0.63193663887035	NA	NA	KXJ75006.1 hypothet	XP_021703205.1cytochrome P450 CYP12A2
AAEL01067	NADH dehydrogenase [ubiquinone] 1 alpha subcomplex subunit 1	-0.152460355166597	0.244661346827249	0.679727114240672	NA	aag01100 ; aag	ETN65742.1 6.3 kDa	XP_001660989.2 NADH dehydrogenase [ubiquinone] 1 alpha subcomplex subunit 1
AAEL00547	synaptogyrin	-0.152044539096478	0.67690918814204	0.914438668430353	NA	NA	KXJ83740.1 hypothet	XP_019539731.1 synaptogyrin-2-like
AAEL00255	large neutral amino acids transporter small subunit 1	-0.151964748162563	0.370774309319027	0.781817769662763	NA	NA	KXJ81475.1 hypothet	XP_021692931.1large neutral amino acids transporter small subunit 1
AAEL01805	NA	-0.151863087848122	0.298952186813132	0.73047212508084	NA	NA	NA	XP_021707598.1integrator complex subunit 7
AAEL01165	endoribonuclease rege-1	-0.151838071918208	0.266859772694658	0.702817294887306	NA	NA	XP_019547963.1 PRE	XP_021708829.1endoribonuclease rege-1 isoform X2
AAEL02282	NA	-0.151774121384556	0.635416760637421	0.902538767814767	04660 ; 04658	NA	NA	XP_001649578.2TGF-beta-activated kinase 1 and MAP3K7-binding protein 1
AAEL00254	triosephosphate isomerase	-0.151671988626674	0.309194751408546	0.742274780613523	00051 ; 00710 ; 00010 ;	aag01100 ; aag	KXJ81473.1 hypothet	XP_001655586.1triosephosphate isomerase
AAEL01975	NA	-0.151619873696902	0.16849326484927	0.588638960950259	NA	aag04310 ; aag	NA	XP_021703386.1casein kinase I
AAEL00782	mediator of RNA polymerase II transcription subunit 6	-0.151599279014602	0.62195972702587	0.898271200863489	NA	NA	XP_019533888.1 PRE	XP_021706516.1mediator of RNA polymerase II transcription subunit 6
AAEL01115	apoptosis-inducing factor 3	-0.151366785584551	0.375667974990824	0.7822803136152	NA	NA	XP_019545676.1 PRE	XP_001661452.2apoptosis-inducing factor 3 isoform X1
AAEL00187	uncharacterized LOC5572818	-0.151282816742998	0.34031245936108	0.761379832492257	NA	NA	KXJ73000.1 hypothet	XP_001654223.2uncharacterized protein LOC5572818
AAEL00559	uncharacterized LOC5566701	-0.151266822065716	0.208066934409657	0.639832374753494	NA	NA	NA	XP_001651109.1uncharacterized protein LOC5566701 isoform X3
AAEL00950	potential E3 ubiquitin-protein ligase ariadne-2	-0.151255869924392	0.30452302489378	0.737814869687351	NA	NA	NA	XP_021698148.1potential E3 ubiquitin-protein ligase ariadne-2
AAEL01026	uncharacterized LOC5573072	-0.151234939734701	0.195454992104472	0.62802321107873	NA	NA	NA	XP_021702139.1uncharacterized protein LOC5573072
AAEL00870	putative mediator of RNA polymerase II transcription subunit 26	-0.151216870541563	0.192701404946064	0.623720833033304	NA	NA	KXJ72695.1 hypothet	XP_021705367.1putative mediator of RNA polymerase II transcription subunit 26 isoform X2
AAEL00374	uncharacterized LOC5578869	-0.151177743220844	0.732347368735194	0.925440879854609	NA	NA	KXJ73546.1 hypothet	XP_001664080.2uncharacterized protein LOC5578869
AAEL00258	general odorant-binding protein 56d	-0.151143622054792	0.18000424575879	0.605409016620025	NA	NA	AGI04312.1 obp11 [A	XP_001655720.2general odorant-binding protein 56d
AAEL02102	NA	-0.151042128709433	0.494074025980796	0.853156161958207	NA	NA	NA	NA
AAEL01268	uncharacterized LOC5576654	-0.150740763957182	0.437812263007658	0.820960220424486	901	NA	XP_001869933.1 con	EAT35125.1AAEL012685
AAEL01512	uncharacterized LOC5566361	-0.150445328589565	0.702027004081199	0.916881840462067	NA	NA	NA	XP_021694897.1uncharacterized protein LOC5566361
AAEL00800	transcription factor hamlet	-0.150393475455065	0.430595306606302	0.819342020552356	NA	NA	XP_019560405.1 PRE	XP_021696243.1transcription factor hamlet isoform X2
AAEL00201	putative apoptosis-inducing factor 1, mitochondrial	-0.150262355583835	0.397226596185916	0.797539006884967	NA	aag04214	KXJ82231.1 hypothet	XP_021703282.1putative apoptosis-inducing factor 1, mitochondrial
AAEL01830	NA	-0.150253862017898	0.388866319303706	0.793285976044306	NA	aag04144	NA	XP_001656743.2protein spartin
AAEL01306	uncharacterized protein YJR142W	-0.150195610721117	0.419848967082053	0.813636239562413	NA	NA	NA	XP_001846962.1thiamin pyrophosphokinase
AAEL00722	E3 ubiquitin-protein ligase RNF123	-0.150093412002359	0.625724370017232	0.899577186361051	NA	NA	KXJ75047.1 hypothet	XP_021703194.1E3 ubiquitin-protein ligase RNF123
AAEL01006	protransforming growth factor alpha	-0.150040478298028	0.693511951367883	0.916122517126697	NA	aag04013 ; aag	NA	XP_001660618.2protransforming growth factor alpha
AAEL00535	poly(A) polymerase beta	-0.15000308147771	0.28105281193391	0.715107379448722	NA	aag03015	KXJ69424.1 hypothet	XP_001650777.2poly(A) polymerase beta
AAEL02400	NA	-0.149986018327918	0.507324588247998	0.859406428557307	NA	NA	NA	XP_021699398.1origin recognition complex subunit 1
AAEL02152	NA	-0.149974163806915	0.468182413392834	0.839644234660078	00564 ; 04070 ; 00561	aag01100 ; aag	NA	XP_021713144.1diacylglycerol kinase theta isoform X2
AAEL00451	uncharacterized LOC5564996	-0.149905406781361	0.619640986146282	0.897318336565571	NA	NA	KXJ78985.1 hypothet	XP_001649341.1uncharacterized protein LOC5564996
AAEL00359	leucine-rich repeat-containing protein 32	-0.149724488556979	0.371984455678973	0.781817769662763	NA	NA	XP_019564527.1 PRE	XP_001657047.2leucine-rich repeat-containing protein 32 isoform X2
AAEL02438	NA	-0.149699908218655	0.67079211965172	0.912812708172959	NA	NA	NA	XP_021698231.1uncharacterized protein LOC110676052
AAEL00799	protein starmaker	-0.149699532080613	0.229476955382769	0.662487607456997	NA	NA	XP_019560399.1 PRE	XP_021696252.1protein Ycf2 isoform X3

AAEL00017	protein unc-119 homolog	-0.149576692764003	0.732097253463332	0.925378973402415	NA	NA	KXJ84381.1 hypothet	XP_001658918.2protein unc-119 homolog
AAEL00798	uncharacterized LOC5569879	-0.149536375783711	0.69119917242657	0.916122517126697	NA	NA	KXJ77510.1 hypothet	XP_001658775.2uncharacterized protein LOC5569879
AAEL00376	protoporphyrinogen oxidase	-0.149531024629093	0.571233950957721	0.880923047085115	860	aag01100 ; aag	KXJ82424.1 hypothet	XP_021693141.1protoporphyrinogen oxidase
AAEL01250	venom carboxylesterase-6	-0.149499536111262	0.19455899053979	0.62599015068082	NA	NA	KXJ68547.1 hypothet	XP_001656119.2venom carboxylesterase-6
AAEL02676	NA	-0.149438662745909	0.207934351270012	0.639832374753494	NA	NA	NA	NA
AAEL00778	5-aminolevulinate synthase, nonspecific, mitochondrial	-0.149397465714594	0.214643275687833	0.648028428468258	00860 ; 00260	aag01100 ; aag	XP_019529079.1 PRE	XP_021710500.15-aminolevulinate synthase, erythroid-specific, mitochondrial isoform X1
AAEL01488	NA	-0.149353983310933	0.384154630246361	0.790063144962496	NA	aag01100 ; aag	NA	XP_021697512.1NADH dehydrogenase [ubiquinone] 1 alpha subcomplex subunit 8
AAEL00376	serine/arginine repetitive matrix protein 2	-0.149284566632252	0.282596549819032	0.715753918648043	NA	NA	XP_019526364.1 PRE	XP_021693203.1serine/arginine repetitive matrix protein 2 isoform X1
AAEL00463	WD repeat-containing protein 37	-0.14927353452653	0.751139551891409	0.932554085844433	NA	NA	KXJ81405.1 hypothet	XP_001649514.2WD repeat-containing protein 37
AAEL01965	NA	-0.149242935366748	0.375222651405241	0.782198815065306	NA	NA	NA	XP_019544559.1 plexin-B-like
AAEL01389	nose resistant to fluoxetine protein 6	-0.149213626033325	0.393997958792957	0.794384140405957	NA	NA	NA	XP_021694727.1nose resistant to fluoxetine protein 6
AAEL00260	general odorant-binding protein 56d	-0.149045927570592	0.203606988126197	0.636626403240661	NA	NA	AFS65329.1 odorant	XP_001655719.1general odorant-binding protein 56d
AAEL01322	protein aubergine	-0.148961860760016	0.18664856310974	0.614196367514984	NA	aag04320	NA	XP_001663409.1protein aubergine isoform X2
AAEL01718	protein brown	-0.148924724989171	0.472030477100395	0.842604161062625	NA	NA	NA	XP_021695975.1protein brown
AAEL02138	NA	-0.148867376974048	0.568929692782032	0.880635379772131	NA	NA	NA	XP_021699290.1zinc finger protein 91
AAEL02421	NA	-0.148607777810118	0.445955609139812	0.828189271329869	NA	NA	NA	XP_019540191.1 acetylcholinesterase-like
AAEL01295	NADH dehydrogenase [ubiquinone] 1 alpha subcomplex subunit 10, mito	-0.148546700410257	0.335757750956659	0.757849428425215	NA	aag01100 ; aag	NA	XP_001663120.1NADH dehydrogenase [ubiquinone] 1 alpha subcomplex subunit 10, mitochondrial
AAEL02206	NA	-0.148546354928508	0.355535006355524	0.772867683747772	NA	NA	NA	XP_001648946.1uncharacterized protein LOC5564600
AAEL00086	F-box only protein 42	-0.148514270695788	0.577294108311177	0.881961491587822	NA	NA	KXJ69261.1 hypothet	XP_021706240.1F-box only protein 42
AAEL01430	uncharacterized LOC5564085	-0.148451940607449	0.522318021130392	0.865018698932415	NA	NA	NA	XP_021700070.1uncharacterized protein LOC5564085 isoform X1
AAEL00547	cytochrome b5 reductase 4	-0.148373323172426	0.622330734444003	0.898271200863489	520	aag00520	KXJ83737.1 hypothet	XP_021708928.1cytochrome b5 reductase 4 isoform X2
AAEL00778	uncharacterized LOC5569634	-0.1482813490785	0.333234451802833	0.755632763673653	NA	NA	KXJ74384.1 hypothet	XP_019535656.1 toll-like receptor 4
AAEL00802	uncharacterized protein C6orf136 homolog	-0.148196609276317	0.185087732938812	0.612420502332469	NA	NA	KXJ74255.1 hypothet	XP_001658828.2uncharacterized protein C6orf136 homolog
AAEL01956	NA	-0.147968496545979	0.312728213112843	0.744058294402217	NA	NA	NA	XP_021706355.1uncharacterized protein LOC5567672
AAEL00391	ADAM 17-like protease	-0.147959457559602	0.582753229291793	0.884900200650674	NA	aag04330	XP_019546802.1 PRE	XP_021693218.1ADAM 17-like protease
AAEL00124	pleckstrin homology domain-containing family G member 3	-0.147864469947065	0.355063829993352	0.772770908922471	NA	NA	KXJ81481.1 hypothet	XP_021696661.1pleckstrin homology domain-containing family G member 3
AAEL00482	zinc finger protein 883	-0.147826895068075	0.539034655387356	0.869670565558805	NA	NA	NA	XP_001649853.2zinc finger protein 883
AAEL01160	DNA topoisomerase 3-beta	-0.147801545612137	0.402160669722871	0.799283473604722	NA	aag03460 ; aag	KXJ84335.1 hypothet	XP_001655482.1DNA topoisomerase 3-beta
AAEL00544	PRKCA-binding protein-like	-0.147698465981551	0.701096177107205	0.916795086221473	NA	NA	XP_019538627.1 PRE	XP_021701148.1PRKCA-binding protein-like isoform X3
AAEL01471	eukaryotic translation initiation factor 3 subunit L	-0.147582497638842	0.203028128650971	0.635896319876174	NA	NA	NA	XP_001653862.1eukaryotic translation initiation factor 3 subunit L
AAEL02680	NA	-0.147577697836882	0.2203644698831	0.652412844728166	NA	NA	NA	XP_021698409.1tripeptidyl-peptidase 2
AAEL00497	proline-rich extensin-like protein EPR1	-0.147317279986556	0.220005096256564	0.652412844728166	NA	NA	NA	XP_021700886.1protein piccolo isoform X2
AAEL00985	DNA polymerase zeta catalytic subunit	-0.147296139333824	0.408387986190266	0.804363495060537	NA	aag03460	NA	XP_021709936.1DNA polymerase zeta catalytic subunit
AAEL02264	NA	-0.147252715658444	0.248941045468518	0.681164287957569	NA	NA	NA	AAL93243.1larval chymotrypsin-like protein precursor
AAEL01169	glycerol kinase	-0.147061398884918	0.474875578897716	0.84437941358363	561	aag01100 ; aag	KXJ84323.1 hypothet	XP_001655635.2glycerol kinase
AAEL00127	uncharacterized LOC5569584	-0.146967069861076	0.384994458568184	0.790080064171371	NA	NA	KXJ75262.1 hypothet	XP_021698551.1uncharacterized protein LOC5569584
AAEL01815	NA	-0.146893006994581	0.271956888030176	0.707134016290952	NA	NA	NA	XP_021702173.1mucin-5AC isoform X2
AAEL01831	NA	-0.146502572050145	0.434568843388844	0.820960220424486	NA	NA	NA	XP_021706866.1tight junction protein ZO-1 isoform X12
AAEL02524	NA	-0.146386417363284	0.422488557831011	0.814192545906728	NA	NA	NA	XP_021703301.1probable serine/threonine-protein kinase DDB_G0267686 isoform X5
AAEL01951	NA	-0.146276984408329	0.213318417163605	0.646373717545434	NA	NA	NA	XP_021705402.1probable ubiquitin carboxyl-terminal hydrolase FAF
AAEL00196	ejaculatory bulb-specific protein 3	-0.14614501442283	0.234748181157025	0.668737559309875	NA	NA	ETN63879.1 sensory	XP_001660754.1ejaculatory bulb-specific protein 3
AAEL00863	ATP-binding cassette sub-family G member 1	-0.146102433507069	0.253378560840005	0.685145258966747	NA	NA	KXJ79717.1 hypothet	XP_001653357.2ATP-binding cassette sub-family G member 1
AAEL01421	transcriptional repressor CTCF	-0.146043356491841	0.672840335822468	0.912968243848131	NA	NA	NA	XP_021701525.1transcriptional repressor CTCF
AAEL00408	1,5-anhydro-D-fructose reductase	-0.146007681436976	0.336193941527309	0.758445425575872	NA	aag01100 ; aag	KXJ83373.1 hypothet	XP_001648454.11,5-anhydro-D-fructose reductase
AAEL01037	xanthine dehydrogenase/oxidase	-0.145975293461595	0.519776547515448	0.864080221844267	NA	NA	NA	XP_021699063.1xanthine dehydrogenase/oxidase isoform X1
AAEL00498	cullin-associated NEDD8-dissociated protein 1	-0.145905067049616	0.199585922841514	0.633375309392567	NA	NA	KXJ83218.1 hypothet	XP_001650136.1cullin-associated NEDD8-dissociated protein 1
AAEL01986	NA	-0.145804604599078	0.349225215379287	0.767244408961813	NA	NA	NA	XP_001659383.2angiopoietin-related protein 1
AAEL00938	protein crooked neck	-0.145789876583592	0.592147837591093	0.888529762455765	NA	aag03040	NA	XP_001653815.1protein crooked neck
AAEL02777	NA	-0.145677510801518	0.608922048700861	0.893547770777888	NA	NA	NA	XP_021697551.1uncharacterized protein LOC5575002
AAEL01511	phenoloxidase 2	-0.14566114339008	0.238734439626522	0.673462389161024	NA	NA	NA	XP_001650638.2phenoloxidase 2
AAEL00059	neural-cadherin	-0.145500399866143	0.39149130033712	0.793285976044306	NA	NA	KFB53759.1 AGAP00	XP_019549102.1 neural-cadherin
AAEL00923	long-chain-fatty-acid--CoA ligase 6	-0.14542909864956	0.207365840949744	0.639832374753494	NA	aag01100 ; aag	NA	XP_021709357.1long-chain-fatty-acid--CoA ligase 1 isoform X1
AAEL02299	NA	-0.14540368231767	0.72614319034307	0.923177868750456	NA	NA	NA	NA
AAEL00544	cardio acceleratory peptide 2b	-0.145366039822127	0.618984666870953	0.897318336565571	NA	NA	XP_019558479.1 PRE	XP_021696180.1cardio acceleratory peptide 2b isoform X1
AAEL01700	serine protease easter	-0.145350305820859	0.593293982909316	0.888529762455765	NA	NA	NA	XP_021693822.1serine protease easter isoform X1
AAEL00636	calcium-binding mitochondrial carrier protein Aralar1	-0.145171523442547	0.221956002107212	0.654118491874251	NA	NA	XP_019543357.1 PRE	XP_001651962.1calcium-binding mitochondrial carrier protein Aralar1 isoform X2
AAEL00123	uncharacterized peptidase C1-like protein F26E4.3	-0.145058950849796	0.358272811954733	0.773854174836904	NA	NA	KXJ79291.1 hypothet	XP_001658537.1uncharacterized peptidase C1-like protein F26E4.3
AAEL01958	NA	-0.145040812974317	0.204904628055212	0.637782699712521	NA	NA	NA	XP_021708688.1phosphorylase b kinase gamma catalytic chain, skeletal muscle/heart isoform isoform X1
AAEL01952	NA	-0.145021606765141	0.240189431231962	0.674980307372712	NA	aag01100 ; aag	NA	XP_021703518.1very long-chain-fatty-acid--CoA ligase bubblegum
AAEL00118	2-oxoglutarate and iron-dependent oxygenase domain-containing protei	-0.145007215182742	0.56321974467186	0.879621058497374	NA	NA	KXJ76528.1 hypothet	XP_001658179.22-oxoglutarate and iron-dependent oxygenase domain-containing protein 3
AAEL00809	nitric oxide-associated protein 1	-0.145002478266348	0.68532603425524	0.914629465876247	NA	NA	KXJ70709.1 hypothet	XP_021693122.1nitric oxide-associated protein 1
AAEL00643	serine protease 7	-0.144980001113221	0.209349940281417	0.639832374753494	NA	NA	KXJ84359.1 hypothet	XP_001657807.1polyserase-2 isoform X1
AAEL01569	C-factor	-0.144967733110226	0.37606500310078	0.782929900120673	NA	NA	NA	XP_021700506.1uncharacterized protein LOC5579259
AAEL02644	NA	-0.144929996728789	0.241882957656657	0.676742771771529	NA	NA	NA	XP_021698547.1heat shock 70 kDa protein cognate 4-like
AAEL01969	NA	-0.144781772974526	0.227435759685888	0.661670521855687	NA	NA	NA	XP_021697495.1PTB domain-containing adapter protein ced-6 isoform X1
AAEL01147	uncharacterized LOC5574865	-0.144740874884425	0.639758795657567	0.904449291387452	NA	NA	KXJ74261.1 hypothet	XP_001655388.1uncharacterized protein LOC5574865
AAEL00595	glucose-fructose oxidoreductase domain-containing protein 1	-0.144691676440365	0.494618153583192	0.853697860376435	NA	NA	ETN62097.1 oxidored	XP_001651748.1glucose-fructose oxidoreductase domain-containing protein 1
AAEL00085	zinc finger protein 236	-0.144323256028865	0.344790850888012	0.764706052029763	NA	NA	NA	XP_001651671.2zinc finger protein 236 isoform X1
AAEL02242	NA	-0.144231080249277	0.615180131934438	0.896845306410123	NA	NA	NA	XP_019539847.1 sialin-like
AAEL00506	serine protease easter	-0.144132397807634	0.234656247806762	0.668737559309875	NA	NA	KXJ78725.1 hypothet	XP_001650301.1serine protease easter
AAEL01491	pyruvate kinase	-0.143969978389923	0.247185607445331	0.680407406434919	00230 ; 00620 ; 00010	aag01100 ; aag	NA	XP_001649983.1pyruvate kinase isoform X1
AAEL00458	calcium-binding mitochondrial carrier protein SCaMC-2	-0.143799259800483	0.187069514912306	0.614196367514984	NA	NA	XP_019560455.1 PRE	XP_001649449.1calcium-binding mitochondrial carrier protein SCaMC-2 isoform X2
AAEL00522	leucine-rich repeat-containing protein 58	-0.143743327966186	0.273830167296068	0.709199324362066	NA	NA	KXJ83636.1 hypothet	XP_001650545.1leucine-rich repeat-containing protein 58
AAEL01384	eye-specific diacylglycerol kinase	-0.143728975474368	0.312031416132844	0.743490640759055	00564 ; 04070 ; 00561	aag01100 ; aag	NA	XP_021694724.1 eye-specific diacylglycerol kinase
AAEL01114	guanine nucleotide exchange factor DBS	-0.143697028446763	0.359866476224377	0.774255626424296	NA	NA	NA	XP_021695672.1uncharacterized protein LOC5576746 isoform X4
AAEL00569	NA	-0.143597802616183	0.250328672484632	0.683335640523162	NA	NA	XP_019551044.1 PRE	XP_021701720.1receptor-type tyrosine-protein phosphatase-like N isoform X1
AAEL00077	lissencephaly-1 homolog	-0.143552672966046	0.220909778852073	0.652797615862164	NA	aag01100 ; aag	Q17N69.2 RecName:	XP_021709871.1lissencephaly-1 homolog
AAEL02577	NA	-0.143543493276118	0.680229142598768	0.914629465876247	NA	NA	NA	XP_001657416.1THO complex subunit 3
AAEL01315	insulin-like growth factor-binding protein complex acid labile subunit	-0.143489966230482	0.700158747613849	0.916629842461068	NA	NA	NA	XP_021705874.1insulin-like growth factor-binding protein complex acid labile subunit
AAEL01711	H/ACA ribonucleoprotein complex subunit 1	-0.143336017178699	0.201956837365228	0.634701781910801	NA	aag03008	NA	XP_011493503.2H/ACA ribonucleoprotein complex subunit 1
AAEL00308	WD repeat-containing protein 92	-0.143220029989664	0.6140452994941	0.896326910841483	NA	NA	KXJ83059.1 hypothet	XP_001663056.2WD repeat-containing protein 92
AAEL00987	SAP domain-containing ribonucleoprotein	-0.143097922622955	0.400442153257753	0.798648539435638	NA	NA	NA	XP_001654078.2SAP domain-containing ribonucleoprotein

AAEL0077	uncharacterized LOC5569632	-0.143092061668059	0.216719508100931	0.649748124914163	NA	aag01100 ; aag	ETN58856.1 vacuola	ABF18127.1vacuolar H+-ATPase V0 sector accessory subunit S1
AAEL0038	vacuolar protein sorting-associated protein 11 homolog	-0.143072843625026	0.449359691807658	0.829306073695304	NA	NA	XP_001845273.1 vac	XP_001664280.1vacuolar protein sorting-associated protein 11 homolog
AAEL0060	tyrosine-protein phosphatase 99A	-0.143059910684529	0.301168341748371	0.733161661578971	NA	NA	XP_003436255.1 AG	XP_021710377.1tyrosine-protein phosphatase 99A isoform X1
AAEL0227	NA	-0.142929642044173	0.682720056989118	0.914629465876247	NA	NA	NA	XP_021694929.1G1/S-specific cyclin-D2
AAEL0084	protein expanded	-0.142662384294103	0.447884903936583	0.828929381742647	NA	aag04391 ; aag	KXJ70141.1 hypothe	XP_021702721.1protein expanded
AAEL0232	NA	-0.142659703506595	0.414955649936312	0.809508847956623	NA	aag04624	NA	XP_021707386.1modular serine protease
AAEL0169	glyceraldehyde-3-phosphate dehydrogenase 1	-0.142602946829809	0.347016081732734	0.765398366276275	00710 ; 00010	aag01100 ; aag	NA	XP_011493026.1glyceraldehyde-3-phosphate dehydrogenase 1
AAEL0242	NA	-0.142558745883466	0.51505080519674	0.861448202130317	NA	NA	NA	XP_021705581.1major facilitator superfamily domain-containing protein 10
AAEL0030	cell surface glycoprotein 1	-0.142431972765826	0.717662544715619	0.92129137591695	NA	NA	XP_019527887.1 PRE	XP_019556163.1 proteoglycan 4-like
AAEL0078	nucleolar transcription factor 1-B	-0.142284271366743	0.420415516655216	0.813636239562413	NA	NA	XP_019539703.1 PRE	XP_001652959.1nucleolar transcription factor 1-B isoform X1
AAEL0183	NA	-0.142235463312211	0.255487051961762	0.687826756042169	NA	aag04391 ; aag	NA	KFB49043.1AGAP000149-like protein
AAEL0266	NA	-0.142180043539196	0.20931011166721	0.639832374753494	NA	NA	NA	NA
AAEL0119	uncharacterized LOC5575534	-0.142067748214249	0.605186148070758	0.893547770777888	NA	NA	KXJ80958.1 hypothe	XP_001662031.1uncharacterized protein LOC5575534
AAEL0221	NA	-0.141962496339102	0.314220566664676	0.74451110779113	NA	NA	NA	XP_021713284.1uncharacterized protein LOC5574048
AAEL0005	golgin subfamily A member 1	-0.141943489415437	0.50057735829253	0.858585806338837	NA	NA	KXJ79608.1 hypothe	XP_021704706.1golgin subfamily A member 1
AAEL0137	uncharacterized LOC5578178	-0.141795008552855	0.291694340518987	0.722646576583698	NA	NA	NA	XP_001850009.1mitochondrial ribosomal protein L50
AAEL0140	leucine-rich repeat and calponin homology domain-containing protein 1	-0.14172713217141	0.524538160338125	0.865595409935377	NA	NA	NA	XP_021702017.1leucine-rich repeat and calponin homology domain-containing protein 1 isoform X2
AAEL0199	NA	-0.141538080014947	0.51610762717092	0.861448202130317	NA	NA	NA	XP_021693014.1probable G-protein coupled receptor Mth-like 5
AAEL0196	NA	-0.141389014941839	0.54317874240085	0.869900911343623	NA	NA	NA	XP_021694178.1uncharacterized protein LOC5564410
AAEL0022	trifunctional enzyme subunit beta, mitochondrial	-0.141387842008437	0.203171731260035	0.635913697637701	NA	aag01100 ; aag	ETN64676.1 trifuncti	XP_001661254.1trifunctional enzyme subunit beta, mitochondrial
AAEL0072	uncharacterized LOC5568996	-0.141354010053508	0.216695582531869	0.649748124914163	NA	NA	NA	XP_021709567.1uncharacterized protein LOC5568996
AAEL0025	ATP synthase subunit delta, mitochondrial	-0.141313539676887	0.343123652089221	0.763584968929871	NA	aag01100 ; aag	KXJ69947.1 hypothe	XP_011492970.2ATP synthase subunit delta, mitochondrial
AAEL0056	ER degradation-enhancing alpha-mannosidase-like protein 2	-0.140933675466316	0.310977568305328	0.742918550320373	NA	aag04141	KXJ72842.1 hypothe	XP_001651175.2ER degradation-enhancing alpha-mannosidase-like protein 2
AAEL0060	uncharacterized LOC5567390	-0.140913888128191	0.533843833135251	0.866700945213732	NA	NA	XP_001863002.1 con	XP_001651792.1uncharacterized protein LOC5567390
AAEL0254	NA	-0.140834715447608	0.511057367190991	0.860521480858463	NA	NA	NA	KFB46449.1AGAP008115-like protein
AAEL0196	NA	-0.140544093028071	0.28978832349761	0.721952579380462	NA	NA	NA	XP_021693829.1rho GTPase-activating protein 7
AAEL0001	uncharacterized LOC5570041	-0.14028820768569	0.233127063847256	0.666962220912214	NA	NA	KXJ74850.1 hypothe	XP_021710662.1uncharacterized protein LOC5570041 isoform X1
AAEL0002	ninjurin-2	-0.140216263691803	0.393123046092867	0.793953421864587	NA	NA	XP_001868860.1 con	XP_019550234.1 ninjurin-2
AAEL0242	NA	-0.14014769908918	0.707315130417819	0.91797784850989	NA	NA	NA	XP_021695702.1zinc finger protein 62 homolog isoform X1
AAEL0129	protein real-time	-0.140019068952489	0.314187737508096	0.74451110779113	NA	NA	NA	XP_021702626.1protein real-time isoform X2
AAEL0105	glutathione S-transferase D7	-0.139909504531553	0.700560944412229	0.916629842461068	NA	NA	NA	XP_001654620.1glutathione S-transferase D7 isoform X1
AAEL0014	uncharacterized LOC5570730	-0.139796635213195	0.56037915706116	0.87800595572912	NA	NA	NA	XP_021699542.1uncharacterized protein LOC5570730 isoform X5
AAEL0055	WD repeat-containing protein 20	-0.139746369581514	0.574472256553598	0.881197098233729	NA	NA	XP_001869611.1 wd	XP_021706625.1WD repeat-containing protein 20 isoform X1
AAEL0256	NA	-0.139422432207634	0.314158991049778	0.74451110779113	NA	aag03018	NA	XP_021709456.1heat shock 70 kDa protein cognate 5
AAEL0115	serine-rich adhesin for platelets	-0.139402727960829	0.529492010618073	0.866201863414668	NA	NA	XP_308189.4 AGAP	XP_021696040.1uncharacterized protein LOC5577804 isoform X3
AAEL0031	RING finger protein nhl-1	-0.13933711507023	0.200031195090802	0.633719317230304	NA	NA	KXJ81642.1 hypothe	XP_021696339.1RING finger protein nhl-1 isoform X1
AAEL0062	galactosylgalactosylxylosylprotein 3-beta-glucuronosyltransferase P	-0.139326426805017	0.327543510156785	0.750511797923987	NA	aag00514	XP_001849080.1 gal	XP_021704958.1galactosylgalactosylxylosylprotein 3-beta-glucuronosyltransferase P
AAEL0045	uncharacterized LOC5564989	-0.139301209357421	0.222046006956568	0.654118491874251	NA	NA	KXJ78987.1 hypothe	XP_001649339.1uncharacterized protein LOC5564989
AAEL0008	uncharacterized LOC5567237	-0.139267485370105	0.560561289927035	0.87800595572912	NA	aag04214 ; aag	KXJ69249.1 hypothe	XP_021710103.1uncharacterized protein LOC5567237 isoform X1
AAEL0063	apyrase	-0.139166177324355	0.403731893097978	0.800429773223221	NA	NA	AAV90659.1 salivary	P50635.2RecName: Full=Apyrase; AltName: Full=ATP-diphosphatase; Short=ADPase;
AAEL0043	general odorant-binding protein 99b	-0.139011441779353	0.323619522489051	0.74762391301976	NA	NA	KXJ80864.1 hypothe	XP_001648935.2general odorant-binding protein 99b
AAEL0150	NA	-0.138919022149419	0.372274258217531	0.781817769662763	NA	aag04120	NA	XP_021700150.1E3 SUMO-protein ligase PIAS2 isoform X1
AAEL0019	probable cytochrome P450 12a4, mitochondrial	-0.138872837278393	0.394172746958756	0.794388440103915	NA	NA	XP_019551560.1 PRE	XP_001660726.1probable cytochrome P450 12a4, mitochondrial isoform X1
AAEL0080	clavesin-1	-0.138652893402116	0.574768745439456	0.881197098233729	NA	NA	ETN63206.1 hypothe	XP_019527183.1 clavesin-1-like
AAEL0090	uncharacterized LOC5571429	-0.138593218429167	0.310852347922539	0.742918550320373	NA	aag00190	ETN66750.1 lethal 3	XP_001842154.1lethal 35Di
AAEL0079	MOB kinase activator-like 2	-0.138554068173957	0.209430027467673	0.639832374753494	NA	NA	NA	XP_001658720.2MOB kinase activator-like 2 isoform X3
AAEL0256	NA	-0.138460184608511	0.43540777143271	0.820960220424486	NA	NA	NA	XP_021713351.1phosphatidylglycerophosphatase and protein-tyrosine phosphatase 1 isoform X2
AAEL0006	leucine-rich repeat-containing protein 15	-0.138452321441552	0.248360790877055	0.68058844480365	NA	NA	KXJ71375.1 hypothe	XP_001649809.2leucine-rich repeat-containing protein 15
AAEL0029	histone-lysine N-methyltransferase setd3	-0.138077004024257	0.566051431039552	0.880302387766415	NA	NA	KXJ77697.1 hypothe	XP_021710443.1histone-lysine N-methyltransferase setd3
AAEL0215	NA	-0.137867666531989	0.349688882755697	0.767801448844281	NA	NA	NA	NA
AAEL0001	phosphatidylinositol 4-kinase alpha	-0.137832811833027	0.241960062759014	0.676752734804998	NA	aag01100 ; aag	NA	XP_001658919.2phosphatidylinositol 4-kinase alpha isoform X6
AAEL0032	proteasome assembly chaperone 4	-0.137742869037457	0.481472812428429	0.846648733033901	NA	NA	XP_019531585.1 PRE	XP_021702242.1proteasome assembly chaperone 4 isoform X2
AAEL0063	NA	-0.137731676888963	0.459900280251972	0.834169795839746	NA	NA	KXJ72006.1 hypothe	XP_001651958.2sphingomyelin phosphodiesterase
AAEL0195	NA	-0.137725223760032	0.496959289798126	0.855889834310753	NA	NA	NA	XP_021708811.1uncharacterized protein LOC5567165
AAEL0068	succinate--CoA ligase [ADP/GDP-forming] subunit alpha, mitochondrial	-0.137511452601781	0.292571773407598	0.723512283028756	00640 ; 00720 ; 00660 ;	aag01100 ; aag	ETN59000.1 Succinyl	XP_001652258.1succinate--CoA ligase [ADP/GDP-forming] subunit alpha, mitochondrial
AAEL0023	esterase B1	-0.137509284705335	0.556803439310022	0.876835490527944	NA	NA	XP_001841715.1 car	XP_001655125.2esterase B1
AAEL0076	uncharacterized LOC5569483	-0.137471056631973	0.633094483609183	0.902506916959539	NA	NA	KFB47851.1 AGAP00	KFB47851.1AGAP000961-like protein
AAEL0279	NA	-0.137429533106096	0.209409914266949	0.639832374753494	NA	NA	NA	XP_021695496.1protein draper isoform X1
AAEL0125	probable prefoldin subunit 2	-0.137257980491423	0.248599449388312	0.680886132809113	NA	NA	KXJ72673.1 hypothe	XP_001656123.2probable prefoldin subunit 2
AAEL0154	NA	-0.137199962769657	0.395815305590487	0.796651890210775	NA	NA	NA	XP_001658130.2WD repeat-containing protein 46
AAEL0115	uncharacterized LOC5575049	-0.137045527765197	0.242404849662697	0.677173474983649	NA	aag04137	XP_001851073.1 seri	XP_001851073.1serine/threonine protein kinase
AAEL0150	protein artichoke	-0.136988285639847	0.363801083243125	0.778353305743603	NA	NA	NA	XP_021698714.1protein artichoke
AAEL0217	NA	-0.136865947916461	0.390053703046026	0.793285976044306	NA	aag02010	NA	XP_021704756.1ATP-binding cassette sub-family A member 5
AAEL0009	mediator of RNA polymerase II transcription subunit 7	-0.136816766081794	0.565631167757153	0.880302387766415	NA	NA	KXJ71379.1 hypothe	XP_001657749.1mediator of RNA polymerase II transcription subunit 7
AAEL0207	NA	-0.13678466163375	0.769936961605325	0.941147080452193	NA	NA	NA	XP_021695225.1 ornithine decarboxylase antizyme-like
AAEL0115	60 kDa heat shock protein, mitochondrial	-0.136626109627644	0.220116110688512	0.652412844728166	NA	aag03018	ETN58825.1 60 kDa	XP_001661764.160 kDa heat shock protein, mitochondrial
AAEL0196	NA	-0.13643677807706	0.562674943906867	0.879512940524628	NA	NA	NA	XP_021712956.1probable serine/threonine-protein kinase cdc7
AAEL0033	FMRFamide receptor	-0.13635356807284	0.475831762207328	0.844841309241706	NA	NA	KXJ70560.1 hypothe	XP_021704574.1FMRFamide receptor
AAEL0023	protein lethal(2)k10201	-0.136351520048192	0.639953317423613	0.904463241434479	NA	NA	KXJ76560.1 hypothe	XP_001661244.1protein lethal(2)k10201
AAEL0023	40S ribosomal protein S11	-0.13628682270688	0.321438005517578	0.746588710355934	NA	aag03010	ABI83780.1 40S ribo	XP_001655146.140S ribosomal protein S11
AAEL0149	bromodomain-containing protein 4	-0.136245263248341	0.616623968751109	0.897164966965308	NA	NA	NA	XP_021703929.1bromodomain-containing protein 4
AAEL0274	NA	-0.136209195582829	0.321524077305898	0.746588710355934	NA	NA	NA	XP_021699713.1TRPL translocation defect protein 14 isoform X2
AAEL0062	coiled-coil domain-containing protein 97	-0.136201252492182	0.65994952720275	0.909580313002131	NA	NA	KFB49454.1 hypothe	XP_001657636.1coiled-coil domain-containing protein 97
AAEL0031	alpha-N-acetylglucosaminidase	-0.136114321702073	0.502947901989393	0.858744381072989	531	aag01100 ; aag	KXJ62517.1 hypothe	XP_019548476.1 alpha-N-acetylglucosaminidase-like
AAEL0116	AP-1 complex subunit beta-1	-0.136080522312468	0.258750849360062	0.691547675112889	NA	aag04142	XP_019547947.1 PRE	XP_001661831.1AP-1 complex subunit beta-1 isoform X4
AAEL0182	NA	-0.135963404761534	0.227060238292623	0.66123388829864	NA	aag00564	NA	XP_021700234.1glycerophosphocholine phosphodiesterase GPCPD1 isoform X2
AAEL0011	transcriptional regulator ATRX homolog	-0.135882526275035	0.254437941329694	0.686571057876479	NA	NA	KFB35025.1 transcrip	XP_021705422.1transcriptional regulator ATRX homolog
AAEL0248	NA	-0.135803594133726	0.269529152107542	0.705606616698038	NA	NA	NA	XP_001657781.2WD repeat-containing protein 55 homolog
AAEL0052	venom carboxylesterase-6	-0.13578203842929	0.374595632741063	0.782198815065306	NA	aag00981	KXJ81526.1 hypothe	XP_001650480.3venom carboxylesterase-6
AAEL0091	major facilitator superfamily domain-containing protein 8	-0.135704377184364	0.388924414221323	0.793285976044306	NA	aag04142	NA	XP_001659805.1major facilitator superfamily domain-containing protein 8
AAEL0050	exocyst complex component 5	-0.13568469584537	0.613109578307981	0.895501122588404	NA	NA	XP_001850932.1 exo	XP_001650302.2exocyst complex component 5
AAEL0096	very-long-chain 3-oxoacyl-CoA reductase	-0.135590248733999	0.331495820302659	0.754128446928769	NA	aag01100 ; aag	NA	XP_021713340.1very-long-chain 3-oxoacyl-CoA reductase isoform X1

AAEL01951	NA	-0.135522548694955	0.282518724751367	0.715753918648043	NA	NA	NA	XP_021693504.1sodium/calcium exchanger 1 isoform X1
AAEL00135	RNA-binding protein 1	-0.135447017373771	0.251682132649872	0.684556086619159	NA	NA	XP_001687957.1 AG/	XP_001653077.1RNA-binding protein 1 isoform X2
AAEL02684	NA	-0.135214368157219	0.384763051200656	0.790080064171371	NA	aag03022	NA	XP_021695542.1transcription initiation factor TFIID subunit 4 isoform X1
AAEL00679	TBC1 domain family member 4	-0.135190012819297	0.338619433502559	0.760778306749309	NA	NA	XP_019543414.1 PRE	XP_021710017.1TBC1 domain family member 4 isoform X3
AAEL00421	leucine-rich repeat-containing protein 20	-0.135151857067346	0.492945877124232	0.852167567405069	NA	NA	KFB52829.1 AGAP00	XP_001648693.1leucine-rich repeat-containing protein 20 isoform X1
AAEL00457	uncharacterized LOC5565067	-0.135007061277397	0.578108333823108	0.882214409260404	NA	NA	KFB52574.1 AGAP00	KFB52574.1AGAP001400-like protein
AAEL00217	methenyltetrahydrofolate synthase domain-containing protein	-0.134931687186804	0.245001923088518	0.679727114240672	NA	NA	XP_001846943.1 con	XP_021699647.1methenyltetrahydrofolate synthase domain-containing protein
AAEL01353	cyclin-K	-0.134913097703795	0.694081473880841	0.916122517126697	NA	NA	NA	XP_019555082.1 cyclin-K
AAEL01984	NA	-0.134774297485447	0.477417655324391	0.845011493487133	NA	aag03015	NA	XP_021708774.1RNA-binding protein Musashi homolog Rbp6 isoform X1
AAEL02400	NA	-0.134695931565794	0.593342923878781	0.888529762455765	NA	NA	NA	XP_021700516.1contactin-2 isoform X2
AAEL00121	probable NADH dehydrogenase [ubiquinone] iron-sulfur protein 6, mitoc	-0.134679228790546	0.324777809500152	0.748164514778316	NA	aag01100 ; aag	ETN66177.1 NADH de	XP_001658304.1probable NADH dehydrogenase [ubiquinone] iron-sulfur protein 6, mitochondrial
AAEL00215	zinc finger protein 224	-0.134666739340999	0.704875021521385	0.917749125552377	NA	NA	KXJ71625.1 hypothet	XP_001654819.1zinc finger protein 224
AAEL00209	golgin-84	-0.134663942826562	0.453986430205739	0.832617462606238	NA	NA	KFB40557.1 AGAP00	XP_019560514.1 golgin-84
AAEL00114	protein sprint	-0.134524358224611	0.348263488974192	0.766929259132545	NA	NA	KXJ80612.1 hypothet	XP_021710682.1protein sprint isoform X1
AAEL02588	NA	-0.134486770725707	0.672421275510828	0.912897695079764	NA	NA	NA	NA
AAEL01307	uncharacterized LOC5577194	-0.134258274761767	0.29568815544555	0.72751936000266	NA	NA	NA	XP_021695013.1uncharacterized protein LOC5577194
AAEL00064	zinc finger protein 436	-0.134177683841082	0.485013849686577	0.847655581875201	NA	NA	ETN63243.1 zinc fing	XP_021710531.1zinc finger protein 436
AAEL01441	eukaryotic initiation factor 4A-III	-0.134100930374358	0.346402272351493	0.765398366276275	NA	aag03013 ; aag	NA	XP_001648723.1eukaryotic initiation factor 4A-III
AAEL01374	NADH dehydrogenase [ubiquinone] iron-sulfur protein 4, mitochondrial	-0.134082166404264	0.253523104410104	0.685145258966747	NA	aag01100 ; aag	NA	XP_001663929.2NADH dehydrogenase [ubiquinone] iron-sulfur protein 4, mitochondrial
AAEL01342	uncharacterized LOC5577938	-0.133995022038324	0.613187630235768	0.895501122588404	NA	NA	NA	KFB36464.1AGAP013121-like protein
AAEL00737	protein yellow	-0.133948154246633	0.24379190750687	0.678781583321699	NA	NA	KFB45517.1 AGAP01	XP_001652709.2protein yellow
AAEL00699	synapse-associated protein of 47 kDa	-0.133937910821672	0.465579308136568	0.838483018891546	NA	NA	NA	XP_021706918.1synapse-associated protein of 47 kDa isoform X4
AAEL00744	protein Gawky	-0.133921319514867	0.420270708794289	0.813636239562413	NA	NA	KXJ72981.1 hypothet	XP_001652769.2protein Gawky
AAEL00782	cGMP-dependent protein kinase, isozyme 2 forms cD4/T1/T3A/T3B	-0.133853697111141	0.239505642361578	0.67449511186165	NA	NA	KFB43052.1 cgmp-de	XP_021700790.1cGMP-dependent protein kinase, isozyme 2 forms cD4/T1/T3A/T3B isoform X2
AAEL00122	engulfment and cell motility protein 1	-0.133818991001352	0.616429425499794	0.897164966965308	NA	NA	KFB42915.1 AGAP00	XP_001658311.1engulfment and cell motility protein 1
AAEL00264	DNA repair and recombination protein RAD54-like	-0.133749185166765	0.667762839976437	0.912418872165226	NA	aag03440	XP_001848380.1 DN/	XP_001661961.1DNA repair and recombination protein RAD54-like
AAEL01116	NA	-0.133628603775067	0.329116401585282	0.752367596512046	NA	NA	KXJ69682.1 hypothet	XP_019547683.1 G protein alpha i subunit
AAEL00617	another transcription unit protein	-0.133603154218726	0.36978966307645	0.781817769662763	NA	NA	KXJ80250.1 hypothet	XP_021704529.1another transcription unit protein
AAEL01409	NA	-0.133490011411039	0.532811296612869	0.866700945213732	970	aag00970	NA	XP_001648027.2valine--tRNA ligase
AAEL00769	embryonic polarity protein dorsal	-0.133485198583032	0.232487829399338	0.666707644289445	NA	aag04624	XP_019540833.1 PRE	XP_001652840.2embryonic polarity protein dorsal isoform X4
AAEL00157	neurogenic locus notch homolog protein 1	-0.133390530463797	0.254421518037821	0.686571057876479	NA	NA	KXJ81775.1 hypothet	XP_021695905.1neurogenic locus notch homolog protein 1
AAEL00748	protein transport protein Sec23A	-0.133354695441521	0.260027877903354	0.693156585303205	NA	aag04141	XP_001846775.1 trar	XP_001652784.1protein transport protein Sec23A isoform X1
AAEL00944	general odorant-binding protein 83a	-0.133319936991738	0.59707846841729	0.889911899315825	NA	NA	NA	XP_001660077.1general odorant-binding protein 83a
AAEL00543	transient receptor potential protein	-0.133287506585707	0.38537559098226	0.790599650772731	NA	aag04745	KXJ76346.1 hypothet	XP_001650890.2transient receptor potential protein isoform X1
AAEL01962	NA	-0.133185174431726	0.282853820237193	0.715753918648043	NA	NA	NA	XP_021709718.1sodium-dependent neutral amino acid transporter B(0)AT3 isoform X2
AAEL00648	sodium-coupled monocarboxylate transporter 1	-0.133134925778251	0.429343178299026	0.817466568737354	NA	NA	KXJ77184.1 hypothet	XP_001651974.1sodium-coupled monocarboxylate transporter 1
AAEL01387	glucosamine-6-phosphate isomerase	-0.133098647226007	0.425375223196494	0.814623464645531	520	aag01100 ; aag	NA	XP_021703325.1glucosamine-6-phosphate isomerase
AAEL00912	probable cytochrome P450 6a14	-0.133071793937488	0.449197248910547	0.829306073695304	NA	NA	AAF97937.1 cytochrc	XP_001653673.1probable cytochrome P450 6a14
AAEL02047	NA	-0.133007728762506	0.296936484801301	0.729225922909414	NA	NA	NA	XP_021700063.1pheromone-processing carboxypeptidase KEX1 isoform X2
AAEL01169	exosome complex exonuclease RRP44	-0.132878309962669	0.521244997880488	0.864991501344855	NA	aag03018	XP_001845598.1 mit	XP_001655640.2exosome complex exonuclease RRP44
AAEL01135	prostaglandin reductase 1	-0.132876504208304	0.610290628605353	0.894250694972366	NA	NA	KXJ82523.1 hypothet	XP_021694721.1prostaglandin reductase 1
AAEL02354	NA	-0.132818168832812	0.538783520092206	0.869670565558805	NA	NA	NA	XP_001651551.2uncharacterized protein LOC5567124
AAEL00833	NA	-0.132807249146503	0.325773190121792	0.748896673349414	NA	NA	NA	XP_021692980.1afadin isoform X1
AAEL02605	NA	-0.132622458844559	0.700021071505439	0.916629842461068	NA	NA	NA	XP_021699815.1uncharacterized protein LOC110676395 isoform X1
AAEL00889	bolA-like protein 3	-0.13261809522924	0.45135106491326	0.830666499866364	NA	NA	KXJ75042.1 hypothet	XP_001659581.1bolA-like protein 3
AAEL00098	protein ECT2	-0.132391145551047	0.463096939557262	0.837264939413983	NA	NA	XP_019931396.1 PRE	XP_021695571.1protein ECT2 isoform X1
AAEL02802	NA	-0.13237766212504	0.656925683916257	0.909239282344833	NA	NA	NA	XP_021703456.1zinc finger protein 184-like
AAEL00769	solute carrier organic anion transporter family member 1C1	-0.132372694325427	0.568038682957996	0.880422457819946	NA	NA	KXJ84224.1 hypothet	XP_021700472.1solute carrier organic anion transporter family member 1C1
AAEL01251	low density lipoprotein receptor adapter protein 1-A	-0.132311073547309	0.334588214614369	0.757087156181969	NA	aag04144	KXJ78582.1 hypothet	XP_001662631.1low density lipoprotein receptor adapter protein 1-A
AAEL00340	39S ribosomal protein L15, mitochondrial	-0.132111566662775	0.396181092373109	0.796693358396638	NA	aag03010	KXJ69300.1 hypothet	XP_001656814.239S ribosomal protein L15, mitochondrial
AAEL00462	band 4.1-like protein 5	-0.132100212220411	0.540832444030384	0.869735874782513	NA	NA	XP_001847268.1 bar	XP_021694131.1band 4.1-like protein 5
AAEL02507	NA	-0.132041746842458	0.378258950079104	0.78478947482763	NA	aag03013 ; aag	NA	XP_021705972.1protein SEC13 homolog
AAEL00676	BLOC-1-related complex subunit 7	-0.13198183228315	0.605158916869986	0.893547770777888	NA	NA	KFB37818.1 AGAP01	XP_001658037.1BLOC-1-related complex subunit 7 isoform X1
AAEL00786	uncharacterized LOC5569727	-0.131895554172196	0.586002547254919	0.885305440788009	NA	NA	KXJ70891.1 hypothet	XP_001652948.1uncharacterized protein LOC5569727
AAEL01032	phosphotriesterase-related protein	-0.131799355685598	0.383072200608845	0.788887121770344	NA	NA	NA	XP_001654444.1phosphotriesterase-related protein
AAEL00961	opsin, ultraviolet-sensitive	-0.131636495463067	0.245861476466043	0.679727114240672	NA	NA	NA	XP_021698798.1opsin, ultraviolet-sensitive
AAEL00147	ethanolaminephosphotransferase 1	-0.131625899540798	0.504897346249341	0.858744381072989	NA	aag01100 ; aag	KXJ80976.1 hypothet	XP_001653396.1ethanolaminephosphotransferase 1
AAEL01422	innexin shaking-B	-0.131606213764814	0.570792602049368	0.880923047085115	NA	NA	NA	XP_001647835.1innexin shaking-B isoform X5
AAEL02780	NA	-0.131562464490588	0.602014017575069	0.892353896540881	NA	NA	NA	XP_021701400.1RIP-like protein
AAEL01268	40S ribosomal protein S23	-0.131540424546765	0.36293554419219	0.778129747823051	NA	aag03010	ETN58508.1 hypothe	XP_001656190.140S ribosomal protein S23
AAEL00811	protein mab-21	-0.131539219633245	0.645141505712383	0.906554905014882	NA	NA	KFB48871.1 AGAP00	XP_001653106.2protein mab-21
AAEL01324	protein FAM173B	-0.13137047125919	0.505143810852916	0.858744381072989	NA	NA	NA	XP_001663417.2protein FAM173B
AAEL02001	NA	-0.131202731237542	0.422962123556825	0.814192545906728	NA	NA	NA	XP_021699308.1glucose transporter type 1 isoform X1
AAEL01413	serine protease 7	-0.130865539361442	0.519804616364966	0.864080221844267	NA	NA	NA	XP_021701295.1serine protease 7 isoform X1
AAEL00925	cytochrome c oxidase subunit 6C-2	-0.130765011490701	0.324754658709826	0.748164514778316	NA	aag01100 ; aag	NA	XP_001659877.1cytochrome c oxidase subunit 6C-2
AAEL00709	putative phospholipase B-like lamina ancestor	-0.130635655094743	0.250846038172098	0.683858606596939	NA	NA	KXJ81041.1 hypothet	XP_001658122.1putative phospholipase B-like lamina ancestor
AAEL00426	neurocalcin homolog	-0.130602399923541	0.562834312739258	0.879512940524628	NA	NA	XP_004933143.1 neu	XP_001648788.1neurocalcin homolog
AAEL00872	zinc finger protein 436	-0.130542699480422	0.658883242861587	0.909580313002131	NA	NA	KXJ72318.1 hypothet	XP_001659449.2zinc finger protein 436
AAEL01445	uncharacterized LOC5564471	-0.130422171470016	0.65413563826259	0.907635089858856	NA	NA	NA	XP_001648814.1uncharacterized protein LOC5564471
AAEL00850	alpha-amylase 3	-0.130372714456153	0.306790896276899	0.738847307609842	NA	NA	XP_001842566.1 con	XP_001659278.2uncharacterized protein LOC5570689
AAEL01974	NA	-0.130358672233908	0.257452082867155	0.690490838398007	NA	NA	NA	XP_021697224.1protein GDAP2 homolog
AAEL00884	ATP synthase subunit gamma, mitochondrial	-0.130324054058857	0.374830255170948	0.782198815065306	NA	aag01100 ; aag	ETN65841.1 ATP synt	XP_001653466.1ATP synthase subunit gamma, mitochondrial
AAEL00253	60S ribosomal protein L10	-0.130224737300269	0.354144009062969	0.771321460685784	NA	NA	ETN59955.1 60S ribo	XP_001655466.160S ribosomal protein L10
AAEL00595	chloride channel protein 2	-0.130157220371912	0.257336196925436	0.690381249011039	NA	NA	XP_019543168.1 PRE	XP_021693331.1chloride channel protein 2
AAEL00635	MAM and LDL-receptor class A domain-containing protein 2	-0.130089864836986	0.622583074156947	0.898271200863489	NA	NA	XP_001847563.1 SR-	XP_021710624.1MAM and LDL-receptor class A domain-containing protein 2
AAEL00751	complexin	-0.130020143735892	0.310972403164786	0.742918550320373	NA	NA	NA	XP_021712455.1complexin isoform X1
AAEL02525	NA	-0.129930471422721	0.595676682470753	0.889262951345858	NA	NA	NA	XP_021699225.1HIV Tat-specific factor 1
AAEL01698	cleavage and polyadenylation specificity factor subunit 4	-0.129871088936194	0.57602942980287	0.881764069566298	NA	aag03015	NA	XP_021700101.1cleavage and polyadenylation specificity factor subunit 4
AAEL01402	F-box/LRR-repeat protein 13	-0.129849297856506	0.624164654894068	0.898836174387357	NA	NA	NA	XP_001664247.2F-box/LRR-repeat protein 13
AAEL01960	NA	-0.129747996510223	0.437803104102213	0.820960220424486	NA	aag01100 ; aag	NA	XP_021701939.1polypeptide N-acetylgalactosaminyltransferase 5 isoform X1
AAEL01332	myoneurin	-0.129738655924009	0.566793943520989	0.880302387766415	NA	NA	NA	XP_019557164.1 myoneurin-like
AAEL01023	30 kDa salivary gland allergen Aed a 3	-0.129633991095787	0.419167718060012	0.813348977896628	NA	NA	NA	XP_001660691.230 kDa salivary gland allergen Aed a 3

AAEL0231€	NA	-0.129614482895219	0.307857538608292	0.740435198712364	NA	aag03015	NA	XP_021713081.1serine/threonine-protein phosphatase 2A 56 kDa regulatory subunit epsilon isoform
AAEL0196Z	NA	-0.12960841798538	0.539039788737845	0.869670565558805	NA	NA	NA	XP_021706738.1regulating synaptic membrane exocytosis protein 1 isoform X1
AAEL0085Z	oxysterol-binding protein-related protein 11	-0.129595947740427	0.630114241296305	0.902180060433888	NA	NA	KXJ68940.1 hypothet	XP_001653312.1oxysterol-binding protein-related protein 11
AAEL0004Z	uncharacterized LOC5577360	-0.129357431529779	0.318945515876538	0.745684464362801	NA	NA	XP_001844380.1 con	XP_001656438.1uncharacterized protein LOC5577360
AAEL0102€	NA	-0.129249167816762	0.302745421061006	0.735250294168217	NA	NA	NA	XP_001660703.2venom allergen 5 isoform X1
AAEL0218Z	NA	-0.129145996374242	0.269691924258198	0.705750472775923	NA	aag04142	NA	XP_021702253.1CD63 antigen
AAEL0016Z	uncharacterized LOC5571644	-0.128991057988167	0.612062954961535	0.895007422182805	NA	NA	KXJ80722.1 hypothet	XP_001653735.2uncharacterized protein LOC5571644
AAEL0196Z	NA	-0.1288947174528	0.42860656641872	0.816740033999805	NA	NA	NA	XP_021699703.1probable serine/threonine-protein kinase DDB_G0282963 isoform X4
AAEL0195Z	NA	-0.128846015810372	0.326306462536706	0.748982805752749	NA	NA	NA	XP_001657110.2titin homolog isoform X2
AAEL0244Z	NA	-0.128727407318055	0.484613278605595	0.847655581875201	NA	aag01100 ; aag	NA	XP_021695522.1chondroitin sulfate synthase 1 isoform X1
AAEL0104€	ets DNA-binding protein pokkuri	-0.128715038059661	0.540139653348312	0.869709828277711	NA	aag04013 ; aag	NA	XP_021703673.1ets DNA-binding protein pokkuri
AAEL0135Z	phosphatidylinositol 3-kinase regulatory subunit alpha	-0.128680538288385	0.344776703645665	0.764706052029763	NA	NA	NA	XP_021697483.1phosphatidylinositol 3-kinase regulatory subunit alpha isoform X2
AAEL00741	protein phosphatase inhibitor 2	-0.128675176940041	0.258058265942146	0.691310673420561	NA	NA	KXJ72634.1 hypothet	XP_001658336.1protein phosphatase inhibitor 2 isoform X2
AAEL0260Z	NA	-0.128663312608252	0.503909804061966	0.858744381072989	NA	aag04080	NA	XP_021700696.15-hydroxytryptamine receptor 2B
AAEL0089Z	forkhead box protein K1	-0.128607288786518	0.274991399792968	0.710808916282197	NA	NA	XP_001845339.1 con	XP_021696531.1forkhead box protein K1
AAEL0129Z	CCR4-NOT transcription complex subunit 3	-0.128518389198826	0.328522978650094	0.751633130168613	NA	aag03018	NA	XP_021702628.1CCR4-NOT transcription complex subunit 3 isoform X2
AAEL0019Z	actin-1	-0.128428462338002	0.285461425021792	0.717970179170389	NA	aag04145 ; aag	ETN60496.1 actin [Ar	P49128.2RecName: Full=Actin-1; Flags: Precursor
AAEL0195€	NA	-0.12839269053808	0.288957462895369	0.721217831038796	NA	NA	NA	XP_021704918.1protein split ends isoform X3
AAEL0011Z	magnesium transporter NIPA2	-0.128325653569561	0.397791487697349	0.797539006884967	NA	NA	KXJ72370.1 hypothet	XP_001658166.2magnesium transporter NIPA2
AAEL0148Z	17-beta-hydroxysteroid dehydrogenase 13	-0.128273513558049	0.66986429811898	0.912812708172959	NA	NA	NA	XP_001649748.117-beta-hydroxysteroid dehydrogenase 13
AAEL0052Z	TOM1-like protein 2	-0.128212741583013	0.3864803267262	0.791367050591446	NA	NA	XP_019539041.1 PRE	XP_001650612.2TOM1-like protein 2 isoform X2
AAEL0028€	polyserase-2	-0.128149672754049	0.465388410966771	0.838483018891546	NA	NA	KXJ71145.1 hypothet	XP_019537145.1 serine protease 53-like
AAEL02771	NA	-0.127913834881385	0.719293350369802	0.921583569204186	NA	NA	NA	NA
AAEL0212Z	NA	-0.12785444633858	0.294174780458448	0.724764746928686	NA	NA	NA	XP_021699961.1sex determination protein fruitless isoform X1
AAEL0276€	NA	-0.127486890622857	0.357159400448256	0.773736338358957	NA	NA	NA	XP_021709351.1ATP-binding cassette sub-family G member 4
AAEL0134€	beta-parvin	-0.127345921147931	0.376898916704913	0.783277809573714	NA	NA	NA	XP_019527949.1 beta-parvin
AAEL0234€	NA	-0.127313537800681	0.585219270669067	0.885224865991869	NA	NA	NA	XP_021696580.1uncharacterized protein LOC110675563
AAEL0008Z	tetratricopeptide repeat protein 36 homolog	-0.127259737449239	0.549383263589112	0.873281187000692	NA	NA	KXJ82047.1 hypothet	XP_001651638.2tetratricopeptide repeat protein 36 homolog
AAEL0086Z	probable cytochrome P450 49a1	-0.127248767389355	0.422394728599018	0.814192545906728	NA	NA	XP_001841736.1 Cyp	XP_001659378.1probable cytochrome P450 49a1
AAEL0078Z	xaa-Pro aminopeptidase ApepP	-0.127221326316444	0.360688392440662	0.77528862253545	NA	NA	XP_001866008.1 xaa	XP_001652979.2xaao aminopeptidase ApepP
AAEL0045Z	28S ribosomal protein S7, mitochondrial	-0.127137532064099	0.521420110623768	0.864991501344855	NA	aag03010	ETN61601.1 mitocho	XP_001649336.128S ribosomal protein S7, mitochondrial
AAEL0107€	sodium/potassium-transporting ATPase subunit beta-2	-0.126980747109338	0.278560789877264	0.713122992430066	NA	NA	KXJ72578.1 hypothet	XP_001654893.1sodium/potassium-transporting ATPase subunit beta-2
AAEL0093€	neuronal membrane glycoprotein M6-a	-0.126954214874692	0.351633910063592	0.769131267032369	NA	NA	NA	XP_021710762.1neuronal membrane glycoprotein M6-a isoform X1
AAEL0146Z	protein C12orf4 homolog	-0.126746677984116	0.700579903970951	0.916629842461068	NA	NA	NA	XP_001649155.2protein C12orf4 homolog
AAEL0107€	ATP-dependent RNA helicase dbp2	-0.126384232744404	0.412279434784221	0.806884477085645	NA	aag03040	KXJ84012.1 hypothet	XP_001654897.1ATP-dependent RNA helicase dbp2
AAEL02061	NA	-0.12635308420111	0.485773404131203	0.847790841886424	04151 ; 05165 ; 04714 ;	aag03015	NA	XP_021704389.1serine/threonine-protein kinase Smg1
AAEL0097Z	CCAAT/enhancer-binding protein gamma	-0.126336151558512	0.510919523381822	0.860452247709668	NA	NA	NA	XP_001660303.2CCAAT/enhancer-binding protein gamma
AAEL0002Z	uncharacterized LOC5573751	-0.126322037093937	0.37343457531575	0.782192290591489	NA	NA	KXJ81838.1 hypothet	XP_001654779.2uncharacterized protein LOC5573751
AAEL0099€	uncharacterized LOC5572710	-0.126226169487516	0.770809624181329	0.941147080452193	NA	NA	NA	XP_001660530.2uncharacterized protein LOC5572710
AAEL0122Z	protein obstructor-E	-0.126171785613354	0.649751682646954	0.90663577761678	NA	NA	KXJ77304.1 hypothet	XP_001662353.1protein obstructor-E
AAEL0003Z	zinc-type alcohol dehydrogenase-like protein C1773.06c	-0.125721563908084	0.418568954906096	0.813275237129623	NA	NA	XP_019543236.1 PRE	XP_021702288.1zinc-type alcohol dehydrogenase-like protein C1773.06c isoform X1
AAEL0081Z	3-oxoacyl-[acyl-carrier-protein] reductase FabG	-0.125678793738738	0.414484297996937	0.808925664934849	NA	NA	KXJ74733.1 hypothet	XP_001659009.1uncharacterized protein LOC5570230
AAEL0074€	transcription factor Ouib	-0.125669261565446	0.731778477938425	0.925260275963946	NA	NA	KXJ69978.1 hypothet	XP_021698961.1transcription factor Ouib
AAEL0032Z	GATA zinc finger domain-containing protein 8	-0.125577666411458	0.582985508446259	0.884917975521096	NA	NA	KXJ73643.1 hypothet	XP_001663551.2GATA zinc finger domain-containing protein 8
AAEL0021Z	uricase	-0.125535504785512	0.278071560498596	0.713122992430066	00232 ; 00230	aag01100 ; aag	KXJ80084.1 hypothet	ABV44610.1urate oxidase
AAEL0252Z	NA	-0.125398601842645	0.316565820962354	0.745684464362801	NA	NA	NA	XP_019531735.1 protein chiffon
AAEL0014Z	transcription elongation factor S-II	-0.125364517098414	0.326187553443214	0.748896673349414	NA	NA	KXJ79286.1 hypothet	XP_021700677.1transcription elongation factor S-II isoform X1
AAEL01401	ketimine reductase mu-crystallin	-0.125315356196284	0.642331639024998	0.905763856274616	NA	NA	NA	XP_001664232.2ketimine reductase mu-crystallin
AAEL0000Z	glycosylated lysosomal membrane protein	-0.1252818972122	0.411719628166398	0.806720047134699	NA	NA	XP_001867233.1 con	XP_021706130.1glycosylated lysosomal membrane protein
AAEL0006Z	transmembrane protein 64	-0.125205578301278	0.432904330802331	0.820005280371151	NA	NA	ETN63239.1 hypothe	XP_001649773.1transmembrane protein 64
AAEL0113€	NADH-ubiquinone oxidoreductase 49 kDa subunit	-0.12520341013534	0.359744598610101	0.774255626424296	NA	aag01100 ; aag	ETN63841.1 NADH-u	XP_001655316.1NADH-ubiquinone oxidoreductase 49 kDa subunit
AAEL0082€	uncharacterized LOC5570369	-0.125036115629914	0.585145354316502	0.885224865991869	NA	NA	XP_019530512.1 PRE	XP_019530512.1 protein split ends isoform X1
AAEL0051Z	39S ribosomal protein L42, mitochondrial	-0.125015085695283	0.321515385170871	0.746588710355934	NA	NA	KFB48545.1 AGAP00	XP_001650495.139S ribosomal protein L42, mitochondrial
AAEL0025Z	anosmin-1	-0.124939028978495	0.406688254025972	0.803250764873792	NA	NA	NA	XP_021713410.1anosmin-1 isoform X3
AAEL0064Z	serine/threonine-protein kinase 40	-0.124879094141981	0.398756367479848	0.798554100881298	04151 ; 05165 ; 04714 ;	NA	ETN58900.1 hypothe	XP_001657826.1serine/threonine-protein kinase 40
AAEL0091Z	cytochrome P450 6d3	-0.124816210563705	0.30645709786205	0.738783696372872	NA	NA	XP_019538136.1 PRE	XP_001653690.1cytochrome P450 6d3
AAEL0278€	NA	-0.124771515933623	0.311588795926284	0.743434862302544	NA	NA	NA	XP_021704550.1potassium channel subfamily K member 18 isoform X2
AAEL0153Z	lipase 1	-0.124724717991171	0.487171188505674	0.848846834445739	NA	NA	NA	XP_001651730.2lipase 1
AAEL00901	glutathione S-transferase theta-1	-0.124715523157961	0.49292737965036	0.852167567405069	00980 ; 00982 ; 00983 ;	aag01100 ; aag	KXJ75704.1 hypothet	XP_001659667.2glutathione S-transferase theta-1
AAEL0242C	NA	-0.124679084506438	0.393314613731944	0.793953421864587	NA	NA	NA	XP_021697380.1F-box/LRR-repeat protein 6
AAEL0080Z	uncharacterized LOC5569942	-0.124646466634863	0.61114862311623	0.894646314231935	NA	NA	XP_001688356.2 AG	XP_020803929.1integral membrane protein DGCR2/IDD
AAEL0085€	innexin inx7	-0.124553238538828	0.349021787833576	0.767244408961813	NA	NA	ETN60917.1 innexin		XP_001653301.1innexin inx7
AAEL0174€	hippocampus abundant transcript 1 protein	-0.12440051505585	0.278370799452051	0.713122992430066	NA	NA	NA	XP_011493428.2hippocampus abundant transcript 1 protein isoform X2
AAEL0129Z	glucose-6-phosphate isomerase	-0.124358709419665	0.404129181307877	0.800429773223221	00010 ; 00030 ; 00500 ;	aag01100 ; aag	NA	XP_001663180.1glucose-6-phosphate isomerase
AAEL0059Z	heterogeneous nuclear ribonucleoprotein H	-0.124332058214386	0.260982896235631	0.694093818254416	NA	NA	XP_019543165.1 PRE	XP_001651726.1heterogeneous nuclear ribonucleoprotein H isoform X2
AAEL0076€	28S ribosomal protein S29, mitochondrial	-0.124233694388153	0.368814325704949	0.781817769662763	NA	NA	KXJ84204.1 hypothet	XP_001658571.228S ribosomal protein S29, mitochondrial
AAEL0197Z	NA	-0.124188752368662	0.714890934014743	0.920089841424905	NA	aag04080	NA	XP_021696796.1diuretic hormone receptor isoform X1
AAEL0077Z	uncharacterized LOC5569644	-0.124135381922541	0.489273591726589	0.849220154814801	NA	NA	KXJ70066.1 hypothet	XP_021693472.1uncharacterized protein LOC5569644
AAEL0056€	matrix metalloproteinase-14	-0.12404518330265	0.43384537932175	0.820605381401592	NA	NA	XP_019553504.1 PRE	XP_001651241.1matrix metalloproteinase-14 isoform X1
AAEL0148Z	uncharacterized LOC5565339	-0.123964140429271	0.706114302207219	0.917749125552377	NA	NA	NA	XP_021705340.1uncharacterized protein LOC5565339
AAEL0109Z	cysteine sulfinic acid decarboxylase	-0.123752693247466	0.510922743043428	0.860452247709668	NA	NA	KXJ74848.1 hypothet	XP_021706761.1cysteine sulfinic acid decarboxylase
AAEL0219€	NA	-0.123545410992035	0.442311032615511	0.824760338905837	NA	aag03040	NA	XP_021695709.1U2 snRNP-associated SURP motif-containing protein
AAEL0007€	vesicular inhibitory amino acid transporter	-0.123543608749284	0.56480865571426	0.880302387766415	NA	NA	KFB51336.1 hypothe	XP_021707046.1vesicular inhibitory amino acid transporter
AAEL0146C	4-hydroxyphenylpyruvate dioxygenase	-0.123538429124457	0.424142057817833	0.814488795690304	NA	aag01100 ; aag	NA	XP_001649088.24-hydroxyphenylpyruvate dioxygenase
AAEL0043Z	phosphatidylinositol 4-kinase type 2-alpha	-0.12352749017189	0.322640657116024	0.746650319748046	04070 ; 00562	aag01100 ; aag	KFB45281.1 AGAP00	XP_001649004.2phosphatidylinositol 4-kinase type 2-alpha isoform X1
AAEL0194Z	NA	-0.123512850769241	0.271534917006151	0.706703671719641	NA	NA	NA	XP_021705916.1serine/threonine-protein kinase MARK2 isoform X7
AAEL0111Z	secreted 45 kDa protein	-0.123472785416207	0.401291857018305	0.798937184829606	NA	NA	XP_019538051.1 PRE	XP_019546662.1 golgin subfamily A member 4 isoform X3
AAEL0095Z	mitochondrial import receptor subunit TOM7 homolog	-0.123421999442387	0.270767959729723	0.706237745304114	NA	aag04137	NA	XP_001660268.1mitochondrial import receptor subunit TOM7 homolog
AAEL0100Z	hsp90 co-chaperone Cdc37	-0.123409867923998	0.304809615112322	0.737926355765217	NA	NA	NA	XP_001660603.1hsp90 co-chaperone Cdc37
AAEL0139€	NADH dehydrogenase [ubiquinone] 1 beta subcomplex subunit 2, mitoch	-0.123360788557785	0.30110010758081	0.733161661578971	NA	aag01100 ; aag	NA	XP_001664201.1NADH dehydrogenase [ubiquinone] 1 beta subcomplex subunit 2, mitochondrial
AAEL0106Z	3-ketoacyl-CoA thiolase, mitochondrial	-0.123146322671637	0.280501141843693	0.714609510945122	NA	aag01100 ; aag	KXJ78588.1 hypothet	XP_011493552.23-ketoacyl-CoA thiolase, mitochondrial
AAEL0005€	peroxisome assembly factor 2	-0.123141593169965	0.532829721333733	0.866700945213732	NA	aag04146	KXJ79611.1 hypothet	XP_001648668.1peroxisome assembly factor 2

AAEL0044	RAB6A-GEF complex partner protein 2	-0.12307757959914	0.621389966741176	0.898072950204539	NA	NA	KXJ74676.1 hypothet	XP_001649210.1RAB6A-GEF complex partner protein 2
AAEL0110	ficolin-1	-0.122929631372695	0.3092677145047	0.742274780613523	NA	NA	AAR88166.1 aslectin	XP_019546605.1 ficolin-1-like
AAEL0245	NA	-0.122891494724678	0.379846024761082	0.785823543132077	NA	NA	NA	XP_021696415.1unconventional myosin-Vb isoform X2
AAEL0274	NA	-0.122725711934298	0.292854312566892	0.723631099647605	NA	aag04140 ; aag	NA	XP_021697929.1inositol 1,4,5-trisphosphate receptor isoform X4
AAEL0254	NA	-0.122724545460244	0.387391786330021	0.791825725928371	NA	NA	NA	XP_019540899.1 nesprin-1
AAEL0067	ATP-binding cassette sub-family B member 7, mitochondrial	-0.12260386692607	0.361069975976174	0.77557561132884	NA	aag02010	KXJ83060.1 hypothet	XP_001652166.1ATP-binding cassette sub-family B member 7, mitochondrial isoform X2
AAEL0065	muscle segmentation homeobox	-0.122489591918225	0.702832206832626	0.917106722424789	NA	NA	XP_019556240.1 PRE	XP_021710128.1muscle segmentation homeobox isoform X1
AAEL0141	probable ATP-dependent RNA helicase DHX35	-0.122483686346361	0.308959294757338	0.74192156324557	NA	NA	NA	XP_001648145.2ceramide phosphoethanolamine synthase
AAEL0032	cell division cycle protein 23 homolog	-0.122317116360646	0.596125707000096	0.889500365788046	NA	aag04120	ETN60860.1 anaphas	XP_001656619.1cell division cycle protein 23 homolog
AAEL0034	RNA-binding protein 24-A	-0.122050029519526	0.287158547829276	0.718902142868179	NA	NA	CRK95562.1 CLUMA_	XP_001656804.2RNA-binding protein 24-A
AAEL0182	NA	-0.121857881189543	0.339753943541048	0.761379832492257	NA	NA	NA	XP_021707407.1zinc finger protein hangover
AAEL0197	NA	-0.121831785958709	0.344378342368225	0.764706052029763	NA	NA	NA	XP_021711018.1mucin-5AC isoform X1
AAEL0234	NA	-0.121694016149368	0.534291597223866	0.866700945213732	NA	NA	NA	XP_021704811.1conserved oligomeric Golgi complex subunit 1
AAEL0078	uncharacterized LOC5569697	-0.121693721702659	0.69634362823346	0.916122517126697	NA	NA	KXJ74652.1 hypothet	XP_001658685.2uncharacterized protein LOC5569697
AAEL0186	NA	-0.121669177954613	0.29229904520596	0.723436206020777	NA	NA	NA	XP_021696694.1uncharacterized protein LOC5576943 isoform X12
AAEL0122	protein unc-80 homolog	-0.12158434472453	0.574743801459932	0.881197098233729	NA	NA	XP_019549143.1 PRE	XP_021708233.1protein unc-80 homolog isoform X1
AAEL0014	intersectin-1	-0.121581262091401	0.383129762814924	0.788887121770344	NA	NA	XP_019529461.1 PRE	XP_021700599.1intersectin-1 isoform X1
AAEL0173	glutamic acid-rich protein	-0.12148411646682	0.670156360732783	0.912812708172959	NA	NA	NA	XP_021706305.1glutamic acid-rich protein
AAEL0197	NA	-0.121442281667369	0.338720791431419	0.760778306749309	NA	NA	NA	XP_001651766.1cytochrome c oxidase subunit 7A1, mitochondrial-like
AAEL0022	bromodomain-containing protein 7	-0.121426011066591	0.579357084205205	0.882283691803025	NA	NA	XP_019551397.1 PRE	XP_001654928.2bromodomain-containing protein 7 isoform X1
AAEL0237	NA	-0.12137260388745	0.744768376903135	0.931365937538534	NA	NA	NA	XP_021693684.1uncharacterized protein LOC110674159
AAEL0233	NA	-0.12129975096749	0.421020551861247	0.813840460658889	NA	NA	NA	XP_021697637.1mitochondrial thiamine pyrophosphate carrier
AAEL0124	nuclear pore complex protein Nup133	-0.12128532784245	0.609173341628895	0.893547770777888	NA	aag03013	XP_001847396.1 Nup	XP_001662611.1nuclear pore complex protein Nup133
AAEL0117	nascent polypeptide-associated complex subunit alpha	-0.121177467259896	0.325623669489819	0.748896673349414	NA	NA	XP_001850614.1 nas	XP_001655652.1nascent polypeptide-associated complex subunit alpha
AAEL0008	GPI mannosyltransferase 4	-0.12116484449488	0.651888557230041	0.907241152999219	NA	aag00563	KXJ82747.1 hypothet	XP_001651650.2GPI mannosyltransferase 4
AAEL0124	NA	-0.121066076454171	0.44621294122902	0.828189271329869	NA	NA	KXJ84388.1 hypothet	XP_021702660.1zinc finger SWIM domain-containing protein 8
AAEL0080	protein aubergine	-0.120958464491997	0.502127333663655	0.858744381072989	NA	aag04320	KXJ68003.1 hypothet	XP_001653082.1protein aubergine
AAEL0023	aspartate aminotransferase, mitochondrial	-0.120938563133721	0.319290402044425	0.745684464362801	00960 ; 00401 ; 00360 ;	aag01100 ; aag	XP_001848088.1 asp	XP_001655159.1aspartate aminotransferase, mitochondrial
AAEL0027	serine protease inhibitor 42Dd	-0.120876555644201	0.552663520378066	0.874531710113044	NA	NA	KXJ73358.1 hypothet	XP_001662188.1serine protease inhibitor 42Dd
AAEL0037	uncharacterized LOC5578862	-0.120798948823792	0.655045328702741	0.908200559699054	NA	NA	KXJ77949.1 hypothet	XP_001664071.2uncharacterized protein LOC5578862
AAEL0035	uncharacterized LOC5578489	-0.120612327976678	0.377863217816542	0.784189745229548	NA	NA	KXJ77664.1 hypothet	XP_001663876.2uncharacterized protein LOC5578489
AAEL0044	cyclin-dependent kinases regulatory subunit	-0.120565919872493	0.607597055334588	0.893547770777888	NA	NA	ETN59287.1 cyclin-de	XP_001649286.1cyclin-dependent kinases regulatory subunit
AAEL0196	NA	-0.120449653399194	0.370267653108754	0.781817769662763	NA	NA	NA	EAT47226.1AAEL001659
AAEL0138	cleavage stimulation factor subunit 2 tau variant	-0.120419603140003	0.514794978118261	0.861448202130317	NA	aag03015	NA	XP_001664044.1cleavage stimulation factor subunit 2 tau variant
AAEL0085	glutamate [NMDA] receptor subunit 1	-0.120385797317869	0.47529977918252	0.84437941358363	NA	aag04080	ETN60916.1 hypothe	XP_021705513.1glutamate [NMDA] receptor subunit 1
AAEL0132	ubiquitin carboxyl-terminal hydrolase CYLD	-0.120332770580277	0.319884789087122	0.746140719770603	NA	aag04013	NA	XP_021702790.1ubiquitin carboxyl-terminal hydrolase CYLD
AAEL0061	exocyst complex component 4	-0.120286536088489	0.400349701763476	0.798648539435638	NA	NA	XP_019554641.1 PRE	XP_021704211.1exocyst complex component 4 isoform X2
AAEL0109	serine-rich adhesin for platelets	-0.120217928642664	0.478225413407888	0.845416382162313	NA	NA	KXJ72764.1 hypothet	XP_021698430.1uncharacterized protein LOC5574157
AAEL0145	protein Wnt-5	-0.119991668274032	0.704368235489598	0.917749125552377	NA	aag04150 ; aag	NA	XP_021700182.1protein Wnt-5
AAEL0030	merozoite surface antigen 2, allelic form 2	-0.11997537807909	0.317595785455858	0.745684464362801	NA	NA	XP_019543580.1 PRE	XP_001663089.2merozoite surface antigen 2, allelic form 2 isoform X2
AAEL0250	NA	-0.119969971011961	0.355145422064022	0.772770908922471	NA	NA	NA	XP_021696636.1serine/arginine repetitive matrix protein 5 isoform X6
AAEL0048	NA	-0.119789599847579	0.685315266793522	0.914629465876247	NA	NA	XP_001868508.1 con	XP_001649892.2uncharacterized protein LOC5565533
AAEL0008	cleavage and polyadenylation specificity factor subunit 1	-0.119709429862026	0.434548310946915	0.820960220424486	NA	aag03015	KXJ75685.1 hypothet	XP_021710072.1cleavage and polyadenylation specificity factor subunit 1
AAEL0025	thioredoxin domain-containing protein 5 homolog	-0.119652458838022	0.332526928640817	0.755231182999982	NA	aag04141	XP_019526297.1 PRE	XP_001655446.2thioredoxin domain-containing protein 5 homolog
AAEL0039	guanine nucleotide-binding protein G(s) subunit alpha	-0.119611817647414	0.370664249304029	0.781817769662763	NA	NA	CRK92729.1 CLUMA_	XP_019565130.1 guanine nucleotide-binding protein G(s) subunit alpha
AAEL0008	phosphatidylinositol 4,5-bisphosphate 5-phosphatase A	-0.119519348150252	0.37691307767794	0.783277809573714	NA	aag01100 ; aag	XP_019537941.1 PRE	XP_021710311.1phosphatidylinositol 4,5-bisphosphate 5-phosphatase A isoform X1
AAEL0043	chorion peroxidase-like	-0.119507740363181	0.531922326068519	0.866700945213732	940	NA	KXJ75129.1 hypothet	XP_001649031.3chorion peroxidase-like
AAEL0076	protein KRTCAP2 homolog	-0.11946731230905	0.482375641081793	0.846941547268585	NA	NA	KXJ83500.1 hypothet	XP_001658521.1protein KRTCAP2 homolog
AAEL0039	PHD and RING finger domain-containing protein 1	-0.119451247402962	0.325335474636154	0.748808663732339	NA	NA	KXJ74304.1 hypothet	XP_021706346.1PHD and RING finger domain-containing protein 1
AAEL0099	ras-related protein Rab-8A	-0.119416646707184	0.540046194754718	0.869709828277711	NA	NA	NA	XP_001660517.1ras-related protein Rab-8A
AAEL0005	fibulin-1	-0.119380569874371	0.335572208917708	0.757775574591595	NA	NA	XP_019931033.1 PRE	XP_021709534.1fibulin-2 isoform X2
AAEL0256	NA	-0.11926179300182	0.349094157806261	0.767244408961813	NA	NA	NA	XP_021704114.1integrin alpha1 isoform X2
AAEL0203	NA	-0.118914985460339	0.692374516596763	0.916122517126697	NA	NA	NA	XP_019525569.1 cadherin-86C-like
AAEL0196	NA	-0.118893837029352	0.552934979799277	0.874531710113044	NA	NA	NA	XP_021705229.1protein winged eye
AAEL0104	protein NRDE2 homolog	-0.118867700674368	0.6954501645183	0.916122517126697	NA	NA	NA	XP_001660842.2protein NRDE2 homolog
AAEL0116	helicase POLQ-like	-0.118759181939796	0.667042544462636	0.912189849033315	NA	NA	XP_019532125.1 PRE	XP_001661818.2helicase POLQ-like isoform X1
AAEL0100	phosphoglucomutase	-0.118634751787169	0.358629414505721	0.77388983997572	520	aag01100 ; aag	NA	AAX47078.1phosphoglucomutase 1
AAEL0050	evolutionarily conserved signaling intermediate in Toll pathway, mitochon	-0.118619526268729	0.489307075283262	0.849220154814801	NA	NA	KXJ83937.1 hypothet	XP_001650325.1evolutionarily conserved signaling intermediate in Toll pathway, mitochondrial
AAEL0197	NA	-0.118590603315425	0.331194894029656	0.754128446928769	NA	aag04013	NA	XP_021697063.1mitogen-activated protein kinase kinase kinase kinase 4 isoform X8
AAEL0196	NA	-0.118588690680413	0.513109383558601	0.861297436611866	NA	NA	NA	XP_021697786.1protein still life, isoform SIF type 1 isoform X2
AAEL0018	zinc finger protein 853	-0.118397867440708	0.738228047789185	0.928033958695205	NA	NA	KXJ73002.1 hypothet	XP_021702705.1zinc finger protein 853
AAEL0011	NA	-0.11832092144577	0.594774742764598	0.888948786307326	NA	NA	ETN62134.1 lachesin	XP_021701340.1protein amalgam
AAEL0175	cyclic AMP response element-binding protein B	-0.118147445958652	0.31630135184138	0.745684464362801	NA	NA	NA	XP_021694662.1cyclic AMP response element-binding protein B isoform X1
AAEL0194	NA	-0.118145135676347	0.364572924430697	0.778761496713185	NA	NA	NA	XP_021703324.1protein slowmo
AAEL0020	ATP-dependent RNA helicase dbp2	-0.117856806006823	0.373486105880827	0.782192290591489	NA	aag03040	KXJ81257.1 hypothet	XP_001654592.2ATP-dependent RNA helicase dbp2
AAEL0145	succinate dehydrogenase [ubiquinone] cytochrome b small subunit, mito	-0.117852557407298	0.373500643866442	0.782192290591489	NA	aag01100 ; aag	NA	XP_021713360.1succinate dehydrogenase [ubiquinone] cytochrome b small subunit, mitochondrial
AAEL0111	uncharacterized LOC5574440	-0.117825925837389	0.68286589929779	0.914629465876247	NA	NA	NA	XP_001661455.2uncharacterized protein LOC5574440 isoform X1
AAEL0048	sodium/potassium/calcium exchanger 4	-0.117431200283085	0.322440557627974	0.746650319748046	NA	NA	KXJ75462.1 hypothet	XP_001649855.2sodium/potassium/calcium exchanger 4
AAEL0211	NA	-0.117368706117314	0.57916475581009	0.882283691803025	NA	NA	NA	XP_019526392.1 helicostatins
AAEL0078	MPN domain-containing protein CG4751	-0.11732822049225	0.53329233144473	0.866700945213732	NA	NA	KFB43041.1 hypothe	XP_021700780.1MPN domain-containing protein CG4751
AAEL0044	torsin-like protein	-0.117286943472997	0.504329782296083	0.858744381072989	NA	NA	KXJ74886.1 hypothet	XP_001649118.1torsin-like protein isoform X3
AAEL0029	actin-related protein 10	-0.117279295900444	0.576950727414946	0.881961491587822	NA	NA	KXJ69655.1 hypothet	XP_001656213.1actin-related protein 10
AAEL0197	NA	-0.117263375830723	0.346908926683476	0.765398366276275	NA	NA	NA	XP_021711014.1serine protease 7-like isoform X1
AAEL0152	signal recognition particle 9 kDa protein	-0.117096294335071	0.529304276302912	0.866048710977845	NA	aag03060	NA	XP_001651131.1signal recognition particle 9 kDa protein
AAEL0024	uncharacterized LOC5574804	-0.116921722090718	0.760315733453701	0.935871383184374	NA	NA	KXJ77825.1 hypothet	XP_001655362.1uncharacterized protein LOC5574804
AAEL0018	uncharacterized LOC5572784	-0.116818320361401	0.644125662963039	0.906332602788493	901	NA	KXJ71196.1 hypothet	XP_001654187.2uncharacterized protein LOC5572784
AAEL0006	DNA-directed RNA polymerases I, II, and III subunit RPABC3	-0.116656268013229	0.574170520306522	0.881197098233729	NA	aag03020	ETN63247.1 DNA-dir	XP_001649793.1DNA-directed RNA polymerases I, II, and III subunit RPABC3
AAEL0115	uncharacterized LOC5574901	-0.116601374860515	0.616764353512556	0.897164966965308	NA	NA	NA	XP_021697035.1uncharacterized protein LOC5574901 isoform X2
AAEL0099	coiled-coil domain-containing protein 12	-0.116599533774654	0.664719212877694	0.911808086169482	NA	aag03040	NA	XP_001654122.2coiled-coil domain-containing protein 12
AAEL0032	uncharacterized LOC5577775	-0.116560677810045	0.331210293007907	0.754128446928769	NA	NA	AAV90654.1 Gly-rich	AAV90654.1Gly-rich secreted peptide
AAEL0052	WD repeat-containing protein 61	-0.116516546360625	0.438709507220045	0.821865815439505	NA	aag03018	KXJ83403.1 hypothet	XP_001650506.1WD repeat-containing protein 61
AAEL0278	NA	-0.116482896779279	0.511401780146377	0.8607863875813	230	aag01100 ; aag	NA	XP_021709920.1adenylate cyclase type 2 isoform X1

AAEL01968	NA	-0.116282005750746	0.352172684662316	0.769212685559608	NA	NA	NA	XP_021707629.1muscleblind-like protein 2a isoform X2
AAEL00003	40S ribosomal protein S6	-0.116249268247078	0.414016188693584	0.808526521298463	NA	aag03010 ; aag	XP_019544926.1 PRE	XP_001647931.140S ribosomal protein S6
AAEL00588	uncharacterized LOC5567125	-0.116184237093471	0.519871323555235	0.864080221844267	NA	NA	KXJ70029.1 hypothet	XP_001651549.2uncharacterized protein LOC5567125
AAEL01995	NA	-0.116177797353221	0.320620374039301	0.746545719106691	00480 ; 00460 ; 00430	aag01100 ; aag	NA	XP_021704350.1gamma-glutamyltranspeptidase 1 isoform X3
AAEL00038	isoleucine--tRNA ligase, cytoplasmic	-0.116051643774687	0.343040355262777	0.76358426442382	970	aag00970	XP_019539964.1 PRE	XP_001656071.2isoleucine--tRNA ligase, cytoplasmic
AAEL01046	vesicular acetylcholine transporter	-0.116048709215606	0.688173398511076	0.916010156153545	NA	NA	NA	XP_021713001.1vesicular acetylcholine transporter
AAEL02748	NA	-0.115975385692688	0.477642545188299	0.845011493487133	NA	NA	NA	XP_021702884.1tyrosine-protein phosphatase non-receptor type 21 isoform X1
AAEL00057	apyrase	-0.115866658693695	0.340300742219548	0.761379832492257	NA	NA	KXJ77401.1 hypothet	XP_019551817.1 apyrase-like
AAEL01456	pleckstrin homology domain-containing family F member 1 homolog	-0.115754581676851	0.344209760553542	0.764706052029763	NA	NA	NA	XP_021700187.1pleckstrin homology domain-containing family F member 1 homolog
AAEL02399	NA	-0.115739993717488	0.36684062394536	0.780578423384551	NA	NA	NA	XP_021694223.1uncharacterized protein LOC110674263
AAEL01372	serine/threonine-protein kinase Genghis Khan	-0.115724043381432	0.370670908618922	0.781817769662763	04151 ; 05165 ; 04714 ;	NA	NA	XP_021711533.1serine/threonine-protein kinase Genghis Khan isoform X1
AAEL02020	NA	-0.115712257567241	0.384680771466174	0.790080064171371	NA	aag04310	NA	XP_021695067.1adenomatous polyposis coli protein-like
AAEL00124	RNA pseudouridylate synthase domain-containing protein 2	-0.115631323881122	0.521830173620559	0.865018698932415	NA	NA	XP_019550549.1 PRE	XP_021702395.1RNA pseudouridylate synthase domain-containing protein 2 isoform X1
AAEL02309	NA	-0.115601517655331	0.279395760792449	0.713604060506006	NA	aag03013	NA	XP_021705605.1eukaryotic translation initiation factor 4 gamma 2
AAEL00992	uncharacterized LOC5572603	-0.115599860701165	0.353602048494375	0.770323402046695	NA	NA	NA	XP_021694172.1uncharacterized protein LOC5572603
AAEL00946	titin	-0.115286339410256	0.345588598006913	0.764941296190615	NA	NA	NA	XP_001660084.2titin isoform X2
AAEL00020	uncharacterized LOC5570040	-0.11511900240449	0.345161097671957	0.764941296190615	NA	NA	AAV90667.1 putative	AHV85170.1Vago protein
AAEL01997	NA	-0.115035388429464	0.505169366686402	0.858744381072989	NA	NA	NA	XP_021707873.1homeobox protein cut isoform X3
AAEL00813	ATP-binding cassette sub-family B member 6, mitochondrial	-0.11497946836303	0.317118188672842	0.745684464362801	NA	aag02010	KXJ75945.1 hypothet	XP_001658950.2ATP-binding cassette sub-family B member 6, mitochondrial
AAEL00348	arf-GAP with SH3 domain, ANK repeat and PH domain-containing protein	-0.114931565559986	0.530588546414023	0.866691320214768	NA	aag04144	KXJ78574.1 hypothet	XP_021695658.1arf-GAP with SH3 domain, ANK repeat and PH domain-containing protein 2 isoform X1
AAEL00869	cytochrome c oxidase subunit 5B, mitochondrial	-0.114763443702656	0.432354027583575	0.820005280371151	NA	aag01100 ; aag	ETN61530.1 cytochro	XP_001663201.1cytochrome c oxidase subunit 5B, mitochondrial
AAEL00011	4-coumarate--CoA ligase 1	-0.114741766018256	0.520886800281533	0.864960891721031	NA	NA	ETN61533.1 AMP de	XP_001657682.14-coumarate--CoA ligase 1
AAEL02364	NA	-0.11462873373416	0.567103912422593	0.880302387766415	NA	aag03022	NA	XP_021712143.1 transcription initiation factor TFIID subunit 1-like
AAEL00574	uncharacterized LOC5566967	-0.114457705414865	0.584619754077046	0.885175928718656	NA	NA	AHG94988.1 allatost	XP_001651381.1uncharacterized protein LOC5566967
AAEL00396	calpain-B	-0.114331318828615	0.506584051725349	0.859256178927183	NA	NA	KXJ74454.1 hypothet	XP_019552580.1 calpain-B-like
AAEL00667	melanization protease 1	-0.114294640528703	0.37683861608288	0.783277809573714	NA	NA	KXJ78669.1 hypothet	XP_001657945.2melanization protease 1
AAEL00687	muscle-specific protein 20	-0.114111676602264	0.313771146496235	0.74451110779113	NA	NA	KFB49782.1 AGAP01	XP_001652273.1muscle-specific protein 20
AAEL00596	actin, indirect flight muscle	-0.114066147876172	0.454144499999148	0.832617462606238	NA	NA	XP_001862810.1 acti	XP_001651744.2actin, indirect flight muscle
AAEL00676	tryptophan--tRNA ligase, mitochondrial	-0.113892319905518	0.573925174179419	0.881197098233729	970	aag00970	KXJ72729.1 hypothet	XP_001658036.2tryptophan--tRNA ligase, mitochondrial
AAEL00887	succinate dehydrogenase cytochrome b560 subunit, mitochondrial	-0.113844738629339	0.414973482468237	0.809508847956623	NA	aag01100 ; aag	KFB47587.1 AGAP01	XP_001653486.1succinate dehydrogenase cytochrome b560 subunit, mitochondrial
AAEL01974	NA	-0.113825677728127	0.396268800410934	0.796693358396638	NA	NA	NA	XP_001651542.2uncharacterized protein LOC5567141
AAEL01393	serine protease inhibitor 28Dc	-0.113787356019651	0.316399412616544	0.745684464362801	NA	NA	NA	XP_011493764.2serine protease inhibitor 28Dc isoform X6
AAEL00618	fatty-acid amide hydrolase 2-A	-0.113783275451315	0.409519673336588	0.805356078752463	970	NA	KXJ70534.1 hypothet	XP_001651825.1fatty-acid amide hydrolase 2-B isoform X1
AAEL02135	NA	-0.113712436493285	0.609325392483291	0.893547770777888	NA	NA	NA	XP_021703738.1protein sickie-like
AAEL00254	hamartin	-0.113668907414068	0.382080124295899	0.788141964530569	NA	aag04150 ; aag	KXJ70671.1 hypothet	XP_019556098.1 hamartin
AAEL00337	transcription factor mef2A	-0.113529160165625	0.464071218566868	0.837274753622609	NA	NA	XP_019527763.1 PRE	XP_021704564.1transcription factor mef2A isoform X1
AAEL00649	very-long-chain 3-oxoacyl-CoA reductase	-0.113366632168118	0.763086366802861	0.937631292204557	NA	NA	KXJ75332.1 hypothet	XP_001651985.1very-long-chain 3-oxoacyl-CoA reductase
AAEL01284	tubulin gamma-1 chain	-0.113005604667406	0.592582293027884	0.888529762455765	NA	NA	NA	XP_001662960.1tubulin gamma-1 chain
AAEL00848	60S ribosomal protein L18	-0.112949680870249	0.422598526610995	0.814192549506728	NA	aag03010	KFB37602.1 60S ribo	XP_001659268.160S ribosomal protein L18 isoform X2
AAEL00262	tryptase	-0.112924134793345	0.358502537201665	0.773854174836904	NA	NA	XP_019531785.1 PRE	XP_019546316.1 serine protease 44-like
AAEL02319	NA	-0.1128556850674	0.417981755064577	0.812788254564712	NA	NA	NA	XP_021705794.1serine/arginine repetitive matrix protein 2-like isoform X1
AAEL02714	NA	-0.112827288435402	0.736433658134139	0.927293722242795	NA	NA	NA	XP_001659919.1protein Peter pan
AAEL00651	ubiquitin-60S ribosomal protein L40	-0.112800658822919	0.469102962344547	0.840476140867313	NA	aag03010	KRT80223.1 ribosom	NP_476776.1ribosomal protein L40, isoform A
AAEL00127	protein transport protein Sec24A	-0.112536039779058	0.345388260842573	0.764941296190615	NA	aag04141	ETN65321.1 transpo	XP_001652901.1protein transport protein Sec24A
AAEL00601	probable histone-lysine N-methyltransferase CG1716	-0.112496671564555	0.527798563429545	0.865973876319045	310	aag01100 ; aag	KXJ75251.1 hypothet	XP_021706896.1probable histone-lysine N-methyltransferase CG1716
AAEL00832	chromosome transmission fidelity protein 18 homolog	-0.112352653601598	0.648834014825909	0.906554905014882	NA	NA	XP_019549816.1 PRE	XP_001659131.2chromosome transmission fidelity protein 18 homolog
AAEL00109	integrator complex subunit 6	-0.112336946197094	0.509258857986517	0.860083070787677	NA	NA	KXJ75550.1 hypothet	XP_001657984.1integrator complex subunit 6
AAEL00329	membrane-bound alkaline phosphatase	-0.112265565393219	0.345949541172886	0.765136884941381	00790 ; 00730	aag01100 ; aag	KXJ70901.1 hypothet	XP_001663535.2membrane-bound alkaline phosphatase
AAEL02210	NA	-0.112234182836387	0.477591066721581	0.845011493487133	NA	aag03010	NA	XP_021708362.160S ribosomal protein L3
AAEL01292	28S ribosomal protein S24, mitochondrial	-0.11219617461938	0.403711544647608	0.800429773223221	NA	NA	NA	XP_001663109.228S ribosomal protein S24, mitochondrial
AAEL01117	uncharacterized LOC5574456	-0.112088172143349	0.506158078909465	0.858744381072989	NA	NA	KXJ68452.1 hypothet	XP_001661471.2uncharacterized protein LOC5574456
AAEL02648	NA	-0.11208549184382	0.543157597503521	0.869900911343623	230	aag01100 ; aag	NA	XP_021713067.1Ca(2+)/calmodulin-responsive adenylate cyclase isoform X1
AAEL02440	NA	-0.111858702751377	0.324563751291726	0.748164514778316	NA	NA	NA	XP_019531346.1 interaptin-like isoform X5
AAEL02298	NA	-0.111830567881094	0.553179528720435	0.874531710113044	NA	NA	NA	NA
AAEL00093	calcium channel flower	-0.11182816560383	0.319131413914853	0.745684464362801	NA	NA	KXJ78031.1 hypothet	XP_001657464.1calcium channel flower isoform X1
AAEL02801	NA	-0.111813905802835	0.344219258247735	0.764706052029763	480	aag01100 ; aag	NA	XP_021694071.1glutathione-specific gamma-glutamylcyclotransferase 1
AAEL02107	NA	-0.111742987817756	0.492138776606122	0.852141102645529	NA	NA	NA	XP_021711354.1arginine/serine-rich coiled-coil protein 2 isoform X1
AAEL00692	dihydrolipoyl dehydrogenase, mitochondrial	-0.111726017799324	0.393043211116825	0.793953421864587	00640 ; 00260 ; 00620 ;	aag01100 ; aag	KXJ78449.1 hypothet	XP_001652343.2dihydrolipoyl dehydrogenase, mitochondrial
AAEL01985	NA	-0.111644447827914	0.307284170722762	0.73963613366227	04151 ; 05165 ; 04714 ;	NA	NA	XP_021707236.1serine/threonine-protein kinase 10 isoform X2
AAEL02700	NA	-0.111625726188781	0.649923793559702	0.906554905014882	NA	NA	NA	XP_021712307.1diphthine--ammonia ligase-like
AAEL01396	syndecan	-0.111453523637481	0.399432301143987	0.798648539435638	NA	NA	NA	XP_021695972.1syndecan isoform X3
AAEL02062	NA	-0.111368770937622	0.369681172209664	0.781817769662763	NA	NA	NA	XP_021709603.1sarcolemmal membrane-associated protein
AAEL00905	actin-related protein 2/3 complex subunit 5	-0.111332424847883	0.387985070934877	0.79232992071521	NA	aag04144	ABI83786.1 Arp2/3 c	XP_001659673.1actin-related protein 2/3 complex subunit 5
AAEL01940	NA	-0.111154362569189	0.458334095608755	0.833283524364043	NA	NA	NA	XP_021709284.1fasciclin-3 isoform X3
AAEL00875	venom carboxylesterase-6	-0.111104431734633	0.661141292996779	0.909914495882133	NA	NA	KXJ73122.1 hypothet	XP_001659467.2venom carboxylesterase-6
AAEL02069	NA	-0.110973444286213	0.400673471160093	0.798648539435638	NA	NA	NA	XP_021708724.1zinc finger CCCH domain-containing protein 18
AAEL01813	NA	-0.110906338615915	0.461384224785619	0.835430467626382	NA	NA	NA	XP_021704479.1regulator of G-protein signaling loco isoform X1
AAEL00856	AFG3-like protein 2	-0.110895490643223	0.307417635951154	0.739763882328065	NA	NA	KXJ81315.1 hypothet	XP_021698601.1AFG3-like protein 2
AAEL01060	succinate dehydrogenase [ubiquinone] flavoprotein subunit, mitochondr	-0.110521248835607	0.39748388247785	0.797539006884967	00650 ; 00020 ; 00720 ;	aag01100 ; aag	KXJ72205.1 hypothet	XP_021710179.1succinate dehydrogenase [ubiquinone] flavoprotein subunit, mitochondrial
AAEL02263	NA	-0.11051617208545	0.433425230313864	0.820316530100406	NA	NA	NA	XP_021694698.1uncharacterized protein LOC5575968
AAEL01227	eukaryotic translation initiation factor 3 subunit J	-0.110427657690367	0.346702187187827	0.765398366276275	NA	aag03013	KFB49341.1 AGAP00	XP_001662374.1eukaryotic translation initiation factor 3 subunit J
AAEL00703	J domain-containing protein	-0.110214252609462	0.339284462519276	0.761115461750945	NA	NA	XP_013100872.1 PRE	XP_001658089.1J domain-containing protein
AAEL00156	tafazzin homolog	-0.109974416317666	0.528820123935703	0.865973876319045	NA	aag00564	XP_019537436.1 PRE	XP_001653587.2tafazzin homolog isoform X2
AAEL00620	uncharacterized LOC5567598	-0.109949556988292	0.530107904839695	0.866593167584806	NA	NA	KXJ73890.1 hypothet	XP_019553096.1 mucin-5AC-like
AAEL01136	facilitated trehalose transporter Tret1	-0.109824843347367	0.656628496281904	0.909162717843227	NA	NA	ETN64119.1 sugar tra	XP_021700710.1facilitated trehalose transporter Tret1
AAEL01835	NA	-0.109684230653739	0.435546657887505	0.820960220424486	NA	NA	NA	XP_019549103.1 rho GTPase-activating protein 92B-like
AAEL00723	mitochondrial uncoupling protein 4	-0.109492093458913	0.409348490598877	0.805356078752463	NA	NA	XP_019538105.1 PRE	XP_021703173.1mitochondrial uncoupling protein 4 isoform X2
AAEL00888	cytochrome c oxidase subunit 6B1	-0.109456478186619	0.411419333187949	0.806364367198191	NA	aag01100 ; aag	KFB34820.1 AGAP00	XP_021697944.1cytochrome c oxidase subunit 6B1
AAEL00483	uncharacterized protein C45G9.7	-0.109451497370572	0.625851739163531	0.899577186361051	NA	NA	ETN61655.1 hypothe	KFB47042.1AGAP005623-like protein
AAEL00282	ATP synthase subunit beta, mitochondrial	-0.109386645076904	0.473060212222726	0.843297185756203	00195 ; 00190	aag01100 ; aag	ETN62038.1 ATP syn	XP_001655991.1ATP synthase subunit beta, mitochondrial
AAEL00974	40S ribosomal protein S18	-0.109346757279806	0.455185604383431	0.832617462606238	NA	aag03010	NA	XP_001660320.140S ribosomal protein S18
AAEL00225	ovochymase-2	-0.109294151907106	0.419176964883485	0.813348977896628	NA	NA	KXJ73500.1 hypothet	XP_019933168.1 transmembrane protease serine 9-like

AAEL01998	NA	-0.109292908761712	0.44722810886679	0.828502725039167	NA	NA	NA	XP_021709814.1probable Ras GTPase-activating protein isoform X4
AAEL00788	spondin-1	-0.109279615315974	0.366691837425656	0.780578423384551	NA	NA	KXJ72335.1 hypothet	XP_019565470.1 spondin-1-like
AAEL02608	NA	-0.109258092440891	0.566772546543485	0.880302387766415	NA	NA	NA	XP_021695025.1protein AF-9
AAEL02307	NA	-0.109091609580841	0.74641022368516	0.931365937538534	NA	NA	NA	KFB50624.1AGAP007129-like protein
AAEL00103	S-formylglutathione hydrolase	-0.109066786774068	0.554327014884368	0.875297464116348	680	aag01100 ; aag	KXJ73792.1 hypothet	XP_001657864.1S-formylglutathione hydrolase
AAEL01507	DDB1- and CUL4-associated factor 6	-0.108952901439915	0.362776402827706	0.777969810957947	NA	NA	NA	XP_021694829.1DDB1- and CUL4-associated factor 6
AAEL00475	enoyl-CoA delta isomerase 1, mitochondrial	-0.108887623854915	0.32608532998155	0.748896673349414	NA	aag00071	KXJ75516.1 hypothet	XP_001649724.2enoyl-CoA delta isomerase 1, mitochondrial
AAEL01286	uncharacterized LOC5576918	-0.108881620709996	0.635193775501688	0.902538767814767	NA	NA	NA	ABF18231.1predicted acetyltransferase
AAEL00184	sepiapterin reductase	-0.108612510076006	0.562276976794782	0.87919672735184	790	aag01100 ; aag	KXJ77214.1 hypothet	XP_021700839.1sepiapterin reductase
AAEL00807	NADH-quinone oxidoreductase subunit B 2-like	-0.108575314593022	0.374070766725287	0.782198815065306	NA	aag01100 ; aag	XP_001869339.1 NAI	XP_001869339.1NADH dehydrogenase iron-sulfur protein 7, mitochondrial
AAEL02436	NA	-0.108488260338525	0.654187292334256	0.907635089858856	NA	NA	NA	XP_021703445.1zinc finger protein 37 homolog isoform X1
AAEL01378	protein farnesyltransferase/geranylgeranyltransferase type-1 subunit alp	-0.108416151570203	0.423407181896141	0.814192545906728	NA	aag00900	NA	XP_021706173.1protein farnesyltransferase/geranylgeranyltransferase type-1 subunit alpha
AAEL00954	uncharacterized LOC5572135	-0.108349707904814	0.772723148457322	0.941230906670349	NA	NA	NA	XP_001660211.2uncharacterized protein LOC5572135 isoform X4
AAEL01355	transcription factor grauzone	-0.10830961956289	0.756822506041889	0.934188766995886	NA	NA	NA	XP_001663741.1transcription factor grauzone
AAEL01233	TATA-box-binding protein	-0.108251459869635	0.644749816357133	0.906554905014882	NA	aag03022	ETN67643.1 tata-box	XP_001662432.1TATA-box-binding protein
AAEL01557	mitochondrial import inner membrane translocase subunit Tim17-A	-0.108192682538926	0.330471135686635	0.754028115692639	NA	NA	NA	XP_021711938.1mitochondrial import inner membrane translocase subunit Tim17-A isoform X1
AAEL02495	NA	-0.108165938882743	0.457867411761884	0.833283524364043	NA	NA	NA	XP_021713171.1organic cation transporter protein
AAEL00027	26S proteasome non-ATPase regulatory subunit 7	-0.108097542812635	0.456573322789728	0.832617462606238	NA	aag03050	KFB51318.1 AGAP01	XP_001654781.126S proteasome non-ATPase regulatory subunit 7
AAEL01832	NA	-0.107889923763546	0.677200687783985	0.914481546552978	NA	NA	NA	XP_021696985.1solute carrier family 28 member 3 isoform X1
AAEL00320	glutaredoxin 3	-0.107808210394771	0.387322985566204	0.791825725928371	NA	NA	KXJ76623.1 hypothet	XP_001656530.1glutaredoxin 3
AAEL02500	NA	-0.107562397078296	0.569585034626026	0.880635379772131	531	aag01100 ; aag	NA	XP_001653975.2iduronate 2-sulfatase
AAEL00672	probable aminoacyl tRNA synthase complex-interacting multifunctional p	-0.107535015468	0.378322767912993	0.78478947482763	NA	NA	KXJ76368.1 hypothet	XP_001652196.1probable aminoacyl tRNA synthase complex-interacting multifunctional protein 2 isoform X2
AAEL02237	NA	-0.107428745894463	0.527696149680074	0.865973876319045	NA	aag04391 ; aag	NA	XP_001657364.2moesin/ezrin/radixin homolog 2 isoform X1
AAEL00940	N(4)-(Beta-N-acetylglucosaminyl)-L-asparaginase	-0.107310090920061	0.428793826374457	0.816927699854607	NA	aag04142 ; aag	NA	XP_019539674.1 N(4)-(Beta-N-acetylglucosaminyl)-L-asparaginase-like
AAEL01132	zinc finger protein Xfin	-0.107298665538394	0.460492045116088	0.834457146359854	NA	NA	KXJ69791.1 hypothet	XP_021710987.1zinc finger protein Xfin
AAEL00168	nuclear migration protein nudC	-0.107293184035126	0.727126940792162	0.923566968708516	NA	NA	KXJ83055.1 hypothet	XP_001659940.1nuclear migration protein nudC
AAEL00730	uncharacterized LOC5569017	-0.107274712375931	0.725556052394127	0.923177868750456	NA	aag01100 ; aag	KXJ69335.1 hypothet	XP_021704299.1uncharacterized protein LOC5569017 isoform X1
AAEL01082	ATP synthase subunit O, mitochondrial	-0.107236480156553	0.437393372558386	0.820960220424486	NA	aag01100 ; aag	KXJ69740.1 hypothet	XP_001655005.1ATP synthase subunit O, mitochondrial
AAEL00373	uncharacterized LOC5578870	-0.107110538950746	0.764587796141494	0.938598839393547	NA	NA	KXJ73547.1 hypothet	XP_001664081.2uncharacterized protein LOC5578870
AAEL01178	probable citrate synthase 2, mitochondrial	-0.106920369111583	0.433938758388588	0.820613328132303	NA	aag01100 ; aag	KFB50224.1 hypothe	XP_001655738.1probable citrate synthase 2, mitochondrial
AAEL00204	probable cytochrome P450 305a1	-0.106773387517834	0.52530637759497	0.865680248818465	NA	NA	XP_019528612.1 PRE	XP_001654598.1probable cytochrome P450 305a1
AAEL01289	probable aconitate hydratase, mitochondrial	-0.106703992686948	0.44173781517131	0.824183166100242	00290 ; 00630 ; 00720 ;	aag01100 ; aag	NA	XP_001663037.1probable aconitate hydratase, mitochondrial
AAEL02228	NA	-0.106641786022917	0.436173147308382	0.820960220424486	NA	NA	NA	NA
AAEL00632	tyrosine-protein phosphatase 69D	-0.106430771533687	0.407113997978226	0.803535299237735	NA	NA	KXJ82559.1 hypothet	XP_021708822.1tyrosine-protein phosphatase 69D
AAEL00296	protein ABHD17B	-0.106415371838547	0.792336817269693	0.945793949302124	NA	NA	KXJ80792.1 hypothet	XP_001656235.1protein ABHD17B
AAEL01093	UPF0454 protein C12orf49 homolog	-0.106391992553818	0.466384509661017	0.838900928502398	NA	NA	KXJ84575.1 hypothet	XP_001655054.1UPF0454 protein C12orf49 homolog
AAEL00393	neurofilament heavy polypeptide	-0.106107383846159	0.557605762323231	0.877259057086403	NA	aag03420 ; aag	KXJ70360.1 hypothet	XP_001648058.1neurofilament heavy polypeptide
AAEL00586	uncharacterized LOC5579836	-0.106009494717421	0.371033480127171	0.781817769662763	NA	NA	KXJ82306.1 hypothet	XP_001651511.1uncharacterized protein LOC5579836
AAEL01289	meiotic nuclear division protein 1 homolog	-0.106001837265212	0.709363014772019	0.918410624324032	NA	NA	NA	XP_001663031.1meiotic nuclear division protein 1 homolog
AAEL02624	NA	-0.105970846595739	0.411834953914163	0.806720047134699	NA	aag03013 ; aag	NA	XP_001651200.1THO complex subunit 4-A
AAEL00763	katanin p60 ATPase-containing subunit A-like 1	-0.105963767901047	0.549123179730684	0.873169431463929	NA	NA	XP_001854132.1 kat	XP_021707528.1katanin p60 ATPase-containing subunit A-like 1
AAEL02622	NA	-0.105901229888697	0.579832208695363	0.882503470793042	NA	NA	NA	XP_019543109.1 neogenin-like
AAEL01960	NA	-0.105795110964874	0.54626349261726	0.870931414970375	NA	NA	NA	XP_019545728.1 major facilitator superfamily domain-containing protein 6-like
AAEL00449	pre-rRNA-processing protein TSR1 homolog	-0.105783966429989	0.358149885497852	0.773854174836904	NA	NA	KXJ78145.1 hypothet	XP_001649282.2pre-rRNA-processing protein TSR1 homolog isoform X2
AAEL01233	protein goliath	-0.105746171844689	0.45968641812657	0.834049651682047	NA	NA	XP_019931118.1 PRE	XP_021710000.1protein goliath isoform X1
AAEL02013	NA	-0.105663474875486	0.629818891328874	0.902037889184171	NA	NA	NA	XP_021707780.1cholinephosphotransferase 1 isoform X1
AAEL00034	peroxidasin	-0.105585282770504	0.536635055777079	0.868163815622483	940	NA	ETN68124.1 peroxida	XP_021697884.1peroxidasin isoform X1
AAEL01826	NA	-0.10543861483581	0.758186998195067	0.935439571457873	NA	NA	NA	XP_021707552.1uncharacterized protein LOC5578567
AAEL02813	NA	-0.105305561802063	0.615873410119241	0.897164966965308	NA	NA	NA	NA
AAEL01530	glucoside xylosyltransferase 1	-0.105233988212232	0.650563966761019	0.906932039402093	NA	aag00514	NA	XP_021700854.1glucoside xylosyltransferase 1
AAEL01014	trifunctional enzyme subunit alpha, mitochondrial	-0.105126540506475	0.392359909354445	0.793341218606811	00062 ; 00362 ; 00930 ;	aag01100 ; aag	NA	XP_001654258.2trifunctional enzyme subunit alpha, mitochondrial
AAEL00623	uncharacterized LOC5567651	-0.105001132596491	0.538250369756522	0.869670565558805	NA	NA	ETN64136.1 hypothe	XP_001651846.1uncharacterized protein LOC5567651
AAEL00651	V-type proton ATPase subunit H	-0.104966725042377	0.348794763118597	0.767244408961813	NA	aag01100 ; aag	KFB39891.1 vacuolar	XP_001652018.1V-type proton ATPase subunit H isoform X2
AAEL00768	NADH dehydrogenase [ubiquinone] flavoprotein 1, mitochondrial	-0.104958855336754	0.418871385951616	0.813348977896628	NA	aag01100 ; aag	ETN67150.1 NADH-u	XP_001658587.2NADH dehydrogenase [ubiquinone] flavoprotein 1, mitochondrial
AAEL00771	60S ribosomal protein L21	-0.104904816181765	0.476773019749926	0.844867278451347	NA	aag03010	KXJ71908.1 hypothet	XP_001652845.160S ribosomal protein L21
AAEL02746	NA	-0.104849753577499	0.426384619443257	0.814868383824891	NA	NA	NA	XP_001662009.2FACT complex subunit spt16 isoform X2
AAEL02581	NA	-0.104691449924717	0.482797270828981	0.847196889048109	NA	aag04013	NA	XP_021709402.1GTPase-activating protein isoform X1
AAEL00640	uncharacterized LOC110677017	-0.104687056730897	0.545084550416949	0.870466764449305	NA	NA	NA	AAL76035.1putative 16.9 kDa secreted protein
AAEL02428	NA	-0.104480958621638	0.374318783926284	0.782198815065306	NA	NA	NA	XP_021702202.1protein bric-a-brac 2 isoform X13
AAEL00030	RNA-binding protein FUS	-0.104464071718416	0.412304847316402	0.806884477085645	NA	NA	KXJ83457.1 hypothet	XP_021702276.1RNA-binding protein FUS
AAEL00400	toll-like receptor Tollo	-0.104407192485231	0.684045863249109	0.914629465876247	NA	NA	ETN65330.1 toll [Anc	XP_001648238.1toll-like receptor Tollo
AAEL01221	vacuolar protein sorting-associated protein 72 homolog	-0.104349201785533	0.608381493460051	0.893547770777888	NA	NA	KXJ74139.1 hypothet	XP_001655934.2vacuolar protein sorting-associated protein 72 homolog
AAEL00542	BTB/POZ domain-containing protein KCTD3	-0.104243437585382	0.476877585581024	0.844867278451347	NA	NA	XP_019526084.1 PRE	XP_001650869.2BTB/POZ domain-containing protein KCTD3 isoform X2
AAEL00036	BAG family molecular chaperone regulator 3	-0.104118537464597	0.723099340388446	0.921934742632737	NA	NA	NA	XP_001655513.1uncharacterized protein LOC5575136 isoform X1
AAEL02378	NA	-0.104093628504244	0.543194831496458	0.869900911343623	NA	NA	NA	NA
AAEL00520	feline leukemia virus subgroup C receptor-related protein 2	-0.104087797633129	0.533619332272674	0.866700945213732	NA	NA	KFB48526.1 AGAP00	XP_001650482.1feline leukemia virus subgroup C receptor-related protein 2
AAEL00534	protein hook	-0.104052045157849	0.572397094113176	0.880923047085115	NA	NA	KXJ80579.1 hypothet	XP_001650717.1protein hook isoform X1
AAEL01262	elongator complex protein 6	-0.10405073030336	0.695990221372746	0.916122517126697	NA	NA	KXJ81575.1 hypothet	XP_021693635.1elongator complex protein 6 isoform X1
AAEL01324	serine protease 7	-0.104020106688241	0.588918259652057	0.88684610505111	NA	NA	NA	XP_021712954.1coagulation factor IX isoform X1
AAEL01944	NA	-0.103895040439624	0.545407822225991	0.870571167410854	NA	NA	NA	XP_021705682.1maternal protein pumilio isoform X1
AAEL01272	uncharacterized LOC5576701	-0.103871076840659	0.791498564164882	0.945793949302124	NA	NA	KXJ69337.1 hypothet	XP_001662839.1uncharacterized protein LOC5576701
AAEL01700	ubiquitin carboxyl-terminal hydrolase 46	-0.103847586053606	0.537050338910932	0.868682935253717	NA	NA	NA	XP_021706106.1ubiquitin carboxyl-terminal hydrolase 46
AAEL01063	zinc finger protein 1 homolog	-0.103789146740258	0.774141074805071	0.941230906670349	NA	NA	XP_001867320.1 zinc	XP_021700866.1zinc finger protein 1 homolog isoform X2
AAEL00826	YY1-associated factor 2	-0.103763518592185	0.600965898979156	0.892093596129407	NA	NA	XP_001849445.1 con	XP_021700178.1YY1-associated factor 2
AAEL01093	pyridoxal-dependent decarboxylase domain-containing protein 1	-0.103623074658766	0.606387522102688	0.893547770777888	NA	NA	KXJ73812.1 hypothet	XP_021704459.1pyridoxal-dependent decarboxylase domain-containing protein 1
AAEL01017	WD repeat-containing protein 87	-0.103336239803511	0.545106455386701	0.870466764449305	NA	NA	NA	XP_021709439.1WD repeat-containing protein 87 isoform X1
AAEL00666	uncharacterized LOC5568231	-0.103260056603513	0.538345402242068	0.869670565558805	NA	NA	KXJ72875.1 hypothet	XP_001657924.1uncharacterized protein LOC5568231
AAEL00184	DNA-directed RNA polymerase, mitochondrial	-0.103226733459188	0.369806063649667	0.781817769662763	NA	NA	KXJ71689.1 hypothet	XP_001654145.2DNA-directed RNA polymerase, mitochondrial isoform X2
AAEL00931	3-hydroxy-3-methylglutaryl-coenzyme A reductase	-0.103145364018954	0.492755190010909	0.852158101574964	900	aag01100 ; aag	NA	XP_001659923.13-hydroxy-3-methylglutaryl-coenzyme A reductase
AAEL00700	DNA replication licensing factor Mcm2	-0.103089000090807	0.599748686332789	0.891725224048203	NA	aag03030	KXJ80931.1 hypothet	XP_001652504.2DNA replication licensing factor Mcm2
AAEL00550	NADH dehydrogenase [ubiquinone] flavoprotein 2, mitochondrial	-0.103075717460134	0.432466912078294	0.820005280371151	NA	aag01100 ; aag	XP_001651019.1 NAI	XP_001651019.1NADH dehydrogenase [ubiquinone] flavoprotein 2, mitochondrial
AAEL00576	fructose-bisphosphate aldolase	-0.102993408868899	0.474289071278094	0.844332076309675	00680 ; 00710 ; 00010 ;	aag01100 ; aag	XP_003436155.1 AG	XP_001651424.1fructose-bisphosphate aldolase isoform X2

AAEL00151	uncharacterized LOC5571097	-0.102931139899828	0.350724563210124	0.768176246660341	NA	NA	KXJ71240.1 hypothet	XP_001659542.1uncharacterized protein LOC5571097
AAEL00406	MICOS complex subunit Mic19	-0.102825592788983	0.432819295482315	0.820005280371151	NA	NA	XP_001851093.1 con	XP_001648362.2MICOS complex subunit Mic19
AAEL02367	NA	-0.102791719767075	0.500139248740926	0.858029916331016	NA	NA	NA	XP_021712995.1probable GPI-anchored adhesin-like protein PGA55 isoform X2
AAEL00386	DNA repair protein complementing XP-C cells homolog	-0.102583950098726	0.723256854162003	0.921934742632737	NA	aag03420	KFB35511.1 AGAP00	XP_001664274.1DNA repair protein complementing XP-C cells homolog
AAEL01075	40S ribosomal protein S19a	-0.102567011796068	0.475854434449007	0.844841309241706	NA	aag03010	ACL37989.1 40S ribo	XP_001654856.140S ribosomal protein S19a
AAEL01054	protein vestigial	-0.102468917462909	0.577577000884867	0.881961491587822	NA	NA	XP_017839737.1 PRE	XP_001654640.2protein vestigial isoform X2
AAEL00481	sodium/potassium/calcium exchanger 4	-0.102421244274509	0.679351863968285	0.914481546552978	NA	NA	XP_001846078.1 sod	XP_021707036.1sodium/potassium/calcium exchanger 4
AAEL00623	uncharacterized LOC5567650	-0.102406866266871	0.566936408945303	0.880302387766415	NA	NA	XP_019559001.1 PRE	XP_019559001.1 SWI/SNF chromatin-remodeling complex subunit SNF5-like isoform X1
AAEL01953	NA	-0.102396664248199	0.630617693037648	0.90233929580663	NA	NA	NA	XP_021694862.1ataxin-2 homolog
AAEL02109	NA	-0.102329813419372	0.436579889384229	0.820960220424486	NA	NA	NA	XP_021698235.1solute carrier organic anion transporter family member 2A1
AAEL02765	NA	-0.102304037415137	0.446380137618682	0.828189271329869	NA	NA	NA	XP_021695873.1short-chain dehydrogenase/reductase family 16C member 6 isoform X1
AAEL02453	NA	-0.102228906711204	0.419594771207491	0.813550586736479	NA	NA	NA	XP_019547618.1 protocadherin Fat 4-like
AAEL00839	receptor-type guanylate cyclase Gyc76C	-0.10221830374309	0.54222383712343	0.869900911343623	NA	aag01100 ; aag	KXJ79765.1 hypothet	XP_021708902.1receptor-type guanylate cyclase Gyc76C
AAEL00557	transient-receptor-potential-like protein	-0.102188130412754	0.372390154288654	0.781817769662763	NA	aag04745	KXJ68981.1 hypothet	XP_001651106.2transient-receptor-potential-like protein
AAEL00222	calcium and integrin-binding protein 1	-0.102166254190522	0.518753706257327	0.863455012004955	NA	NA	NP_001299270.1 calc	XP_001654973.1calcium and integrin-binding protein 1
AAEL02043	NA	-0.102115556457225	0.527026201425714	0.865973876319045	NA	NA	NA	XP_021697371.1cohesin subunit SA-1
AAEL01275	protein phosphatase 1 regulatory subunit 12A	-0.102027424831602	0.410103910122367	0.805356078752463	NA	NA	NA	XP_021696939.1protein phosphatase 1 regulatory subunit 12A isoform X5
AAEL00883	uncharacterized LOC5571132	-0.101995062671185	0.42423873046551	0.814488795690304	NA	NA	XP_019557147.1 PRE	XP_019557147.1 LIM domain-containing protein A
AAEL00745	protein odd-skipped	-0.101980203764927	0.783121758052385	0.94397254618785	NA	NA	KXJ80160.1 hypothet	XP_021701066.1protein odd-skipped
AAEL01495	RNA polymerase II-associated protein 3	-0.10197591739087	0.493704762885992	0.852730548401194	NA	NA	NA	XP_021699825.1RNA polymerase II-associated protein 3 isoform X1
AAEL00233	zinc finger protein 2	-0.101927655245378	0.749643837613844	0.932554085844433	NA	NA	XP_019544793.1 PRE	XP_021694587.1zinc finger protein 2
AAEL00993	protein lethal(2) giant larvae	-0.101893834895518	0.404713129310552	0.800800458735306	NA	aag04391	NA	XP_021694167.1protein lethal(2) giant larvae
AAEL02805	NA	-0.101726265816248	0.655000194538289	0.908200559699054	NA	aag01100 ; aag	NA	XP_021713271.1adenylate cyclase type 9 isoform X2
AAEL02907	NA	-0.101533233713081	0.712300562825484	0.919252089784076	00790 ; 00730	NA	NA	XP_001663539.2uncharacterized protein LOC5577765
AAEL02740	NA	-0.101381706962151	0.372357471326197	0.781817769662763	NA	NA	NA	XP_019554904.1 protein artichoke-like isoform X5
AAEL01267	activating transcription factor of chaperone	-0.101360380813383	0.395637781007355	0.796468794756001	NA	aag04141 ; aag	XP_019543695.1 PRE	XP_021701619.1activating transcription factor of chaperone isoform X1
AAEL02735	NA	-0.101353955862971	0.767666624337018	0.939827742988808	NA	NA	NA	XP_021703227.1oocyte zinc finger protein XlCOF6 isoform X1
AAEL00987	protein xmas-2	-0.101223213341475	0.658555157595558	0.909580313002131	NA	NA	NA	XP_001660477.2protein xmas-2
AAEL01543	serine protease easter	-0.101147753828433	0.515767301120272	0.861448202130317	NA	NA	NA	XP_021704443.1serine protease easter
AAEL00661	protein phosphatase 1 regulatory subunit 14B	-0.101137865809801	0.61143563032	0.894646314231935	NA	NA	CRL02858.1 CLUMA_	XP_019561337.1 protein phosphatase 1 regulatory subunit 14B-like
AAEL00510	regulation of nuclear pre-mRNA domain-containing protein 1B	-0.101129099646515	0.602514080437776	0.892663752727165	NA	NA	ETN64253.1 hypothe	XP_001650398.1regulation of nuclear pre-mRNA domain-containing protein 1B
AAEL00736	phospholipase A2 inhibitor	-0.100943406226978	0.631800521574138	0.902506916959539	NA	NA	KXJ71826.1 hypothet	XP_021705057.1phospholipase A2 inhibitor
AAEL00452	dual 3',5'-cyclic-AMP and -GMP phosphodiesterase 11	-0.100834311661257	0.358945514129188	0.77388983997572	NA	aag01100 ; aag	XP_019525355.1 PRE	XP_021701355.1dual 3',5'-cyclic-AMP and -GMP phosphodiesterase 11 isoform X1
AAEL00873	RNA polymerase II transcriptional coactivator	-0.100832281701151	0.513536894141192	0.861309357197051	NA	NA	ETN61999.1 p31A [A	XP_001653441.1RNA polymerase II transcriptional coactivator
AAEL00431	uncharacterized LOC5564560	-0.100806451725471	0.69659142320373	0.916122517126697	NA	NA	NA	XP_001648906.1uncharacterized protein LOC5564560 isoform X1
AAEL02586	NA	-0.100796179423714	0.644058669184742	0.906332602788493	NA	NA	NA	XP_019561494.1 spatacsin
AAEL02111	NA	-0.100492417278089	0.410984727574243	0.805855841282375	NA	NA	NA	XP_021706028.1uncharacterized protein LOC5576007 isoform X1
AAEL02522	NA	-0.100378754159211	0.649784306144867	0.90663577761678	NA	NA	NA	XP_021696874.1LON peptidase N-terminal domain and RING finger protein 2
AAEL00423	kinesin-like protein Klp10A	-0.1002914499557	0.419554408942704	0.813550586736479	NA	NA	XP_019530397.1 PRE	XP_021700191.1kinesin-like protein Klp10A isoform X5
AAEL02351	NA	-0.100278225899455	0.58461457273247	0.885175928718656	NA	NA	NA	XP_021699154.1phosphatase and actin regulator 4 isoform X7
AAEL01804	NA	-0.100119595799855	0.451823410223884	0.831369530269982	NA	NA	NA	XP_021709353.1ATP-binding cassette sub-family A member 13
AAEL01326	NA	-0.100093076534325	0.7924665147952	0.945793949302124	NA	NA	NA	XP_001663426.1high affinity copper uptake protein 1
AAEL01084	uncharacterized LOC5573992	-0.100073274409922	0.739740960579758	0.928416888458789	NA	NA	KXJ72540.1 hypothet	XP_001661077.1uncharacterized protein LOC5573992
AAEL00621	glycogen-binding subunit 76A	-0.100030834507956	0.34890667725927	0.767244408961813	NA	NA	NA	XP_021696499.1glycogen-binding subunit 76A isoform X5
AAEL00975	endophilin-B1	-0.099964326058342	0.484552844916311	0.847655581875201	NA	aag04144 ; aag	NA	XP_021705286.1endophilin-B1 isoform X2
AAEL02052	NA	-0.0998991476712027	0.363558259205117	0.778250660803901	NA	aag03013	NA	XP_021709475.1eukaryotic translation initiation factor 4 gamma 3-like isoform X4
AAEL00876	pro-resilin	-0.0998719334744311	0.684848278580578	0.914629465876247	NA	NA	KFB45925.1 cuticle p	XP_019544304.1 pro-resilin
AAEL00338	NA	-0.0998376916485469	0.439630342560704	0.822753388701159	NA	NA	KXJ70241.1 hypothet	XP_001656735.1zinc finger protein 622
AAEL00136	coronin-6	-0.0997369774534289	0.37933169461743	0.785666748907449	NA	NA	KXJ83210.1 hypothet	XP_001653160.1coronin-6 isoform X1
AAEL00837	enhancer of mRNA-decapping protein 3	-0.0996412913881448	0.603690761650487	0.893543893953319	NA	aag03018	KXJ75718.1 hypothet	XP_021705253.1enhancer of mRNA-decapping protein 3
AAEL02573	NA	-0.099586061558691	0.750508317089457	0.932554085844433	NA	NA	NA	XP_021702845.1toll-like receptor Tollo
AAEL00960	pyridoxal kinase	-0.0995520425424218	0.402917137918497	0.799923085895579	750	aag01100 ; aag	NA	XP_001653877.1pyridoxal kinase
AAEL02044	NA	-0.0995043217480593	0.514928089299737	0.861448202130317	NA	NA	NA	XP_021701841.1VWFA and cache domain-containing protein CG16868
AAEL02638	NA	-0.0994054458429719	0.569510669396627	0.880635379772131	NA	aag03010	NA	XP_021708202.128S ribosomal protein S10, mitochondrial
AAEL00067	toll-like receptor 6	-0.0992444699178052	0.725251907246845	0.923066120399097	NA	NA	KXJ82551.1 hypothet	XP_021710321.1toll-like receptor 6
AAEL00677	cytochrome c oxidase assembly protein COX11, mitochondrial	-0.099235124355224	0.574550611507298	0.881197098233729	NA	aag01100 ; aag	ETN61161.1 cytochro	XP_001658032.1cytochrome c oxidase assembly protein COX11, mitochondrial
AAEL01983	NA	-0.099150937442978	0.547160865817002	0.871706074662059	4660	NA	NA	XP_021693531.1tyrosine-protein kinase Fer isoform X1
AAEL00281	uncharacterized LOC5576124	-0.0990465539678851	0.393499802436165	0.793953421864587	NA	NA	NA	XP_001662470.1uncharacterized protein LOC5576124
AAEL00722	decorin	-0.099034911316652	0.618641499357673	0.897318336565571	NA	NA	KXJ79722.1 hypothet	XP_019538091.1 decorin-like
AAEL00665	anoctamin-1	-0.0989607170524163	0.573235362251178	0.881060932593176	NA	NA	XP_019534842.1 PRE	XP_001652136.1anoctamin-1 isoform X2
AAEL00643	probable phosphorylase b kinase regulatory subunit beta	-0.0988975597065841	0.390539299123593	0.793285976044306	NA	NA	XP_019530278.1 PRE	XP_001657816.1probable phosphorylase b kinase regulatory subunit beta isoform X6
AAEL00756	glycoprotein-N-acetylgalactosamine 3-beta-galactosyltransferase 1	-0.0988891355638764	0.474032663073527	0.844049645046942	512	aag01100 ; aag	XP_019932914.1 PRE	XP_021700453.1glycoprotein-N-acetylgalactosamine 3-beta-galactosyltransferase 1 isoform X2
AAEL01176	RAB6-interacting golgin	-0.0988818194057085	0.748148541389955	0.932554085844433	NA	NA	KXJ78200.1 hypothet	XP_001661892.2RAB6-interacting golgin
AAEL02389	NA	-0.0986714430772092	0.428983938119691	0.816951613943997	NA	NA	NA	XP_021694136.1ankyrin repeat and KH domain-containing protein mask isoform X4
AAEL00460	uncharacterized LOC5565167	-0.0984795932848529	0.675066510225176	0.914330589798656	NA	NA	KXJ67765.1 hypothet	XP_001649504.1uncharacterized protein LOC5565167
AAEL01001	zinc finger and BTB domain-containing protein 41	-0.0984457838103929	0.696538597486475	0.916122517126697	NA	NA	NA	XP_021712036.1zinc finger and BTB domain-containing protein 41
AAEL01139	28S ribosomal protein S9, mitochondrial	-0.0983915265870731	0.389416791389459	0.793285976044306	NA	aag03010	XP_001870809.1 28S	XP_001661636.128S ribosomal protein S9, mitochondrial
AAEL00388	uncharacterized LOC5579160	-0.0983122689858906	0.460053435545176	0.834169795839746	NA	NA	ETN64663.1 hypothe	KFB49350.1AGAP000126-like protein
AAEL00429	dihydrolipoyllysine-residue acetyltransferase component of pyruvate deh	-0.0982995764995844	0.478326342845323	0.845416382162313	00620 ; 00020 ; 00010	aag01100 ; aag	KXJ82125.1 hypothet	XP_001648832.1dihydrolipoyllysine-residue acetyltransferase component of pyruvate dehydrogenase complex, mitochondrial isoform X1
AAEL01201	uncharacterized LOC5575696	-0.0982316152476771	0.45971526117489	0.834049651682047	NA	NA	ETN61967.1 hypothe	ABF18463.1conserved insect protein
AAEL02297	NA	-0.0982054793785507	0.494666049937747	0.853697860376435	NA	NA	NA	XP_021701832.1putative RNA-binding protein Luc7-like 2 isoform X3
AAEL01247	NA	-0.0981434440662876	0.622482258332547	0.898271200863489	NA	NA	KXJ84026.1 hypothet	XP_021699726.1kinesin light chain
AAEL01834	NA	-0.0980557698872096	0.431705988439023	0.820005280371151	NA	aag04140	NA	XP_001649174.2RB1-inducible coiled-coil protein 1 isoform X1
AAEL02269	NA	-0.0980355173845505	0.56650850698556	0.880302387766415	NA	NA	NA	XP_021699543.1BTB/POZ domain-containing adapter for CUL3-mediated RhoA degradation protein 3
AAEL01805	NA	-0.0980135691884553	0.577613373124716	0.881961491587822	NA	NA	NA	XP_021705353.1YLP motif-containing protein 1 isoform X2
AAEL02074	NA	-0.0980119201218658	0.525247192040786	0.865680248818465	NA	aag03010	NA	XP_019555418.1 60S ribosomal protein L27a
AAEL00769	protein aubergine	-0.097952798010065	0.409702815447266	0.805356078752463	NA	aag04320	XP_001844068.1 piw	XP_001652831.1protein aubergine
AAEL00230	probable E3 ubiquitin-protein ligase HERC2	-0.0979502639649879	0.47302435859997	0.843297185756203	NA	aag04120	KXJ81509.1 hypothet	XP_021707347.1probable E3 ubiquitin-protein ligase HERC2
AAEL02761	NA	-0.0979013532956561	0.367052226993945	0.780578423384551	NA	NA	NA	XP_021706606.1 spectrin beta chain, non-erythrocytic 5
AAEL00172	spermatogenesis-associated protein 20	-0.097894170432616	0.456981880855443	0.832702429234017	NA	NA	KXJ79783.1 hypothet	XP_001653842.3spermatogenesis-associated protein 20 isoform X2
AAEL01069	importin-4	-0.0978331381630878	0.392073964252282	0.793285976044306	NA	NA	KXJ68300.1 hypothet	XP_019527462.1 importin-4-like
AAEL01002	SEC14-like protein 2	-0.0978188474386555	0.567149619582254	0.880302387766415	NA	NA	NA	XP_021697622.1SEC14-like protein 2

AAEL00881	NADH dehydrogenase [ubiquinone] 1 alpha subcomplex assembly factor	-0.0978060894976197	0.594954505791948	0.888948786307326	NA	NA	KXJ75028.1 hypothet	XP_001659497.1NADH dehydrogenase [ubiquinone] 1 alpha subcomplex assembly factor 3
AAEL01399	zinc finger protein 624	-0.0977954203853354	0.766702455316493	0.939190095024277	NA	NA	NA	XP_001657251.2zinc finger protein 624
AAEL01340	tetraspanin-2	-0.0977504792468353	0.645427622733441	0.906554905014882	NA	NA	NA	XP_001663594.1tetraspanin-2 isoform X2
AAEL01967	NA	-0.0977289391296479	0.59292736564101	0.888529762455765	NA	aag03015	NA	XP_021706199.1mRNA-capping enzyme
AAEL00174	protein bric-a-brac 2	-0.0977005910046566	0.710788951449078	0.918935795219545	NA	NA	KXJ81889.1 hypothet	XP_001660260.2protein bric-a-brac 2
AAEL01970	NA	-0.0976720010528652	0.659715452002438	0.909580313002131	NA	aag04150 ; aag	NA	XP_019548561.1 frizzled-2-like
AAEL00188	glutamine synthetase 1, mitochondrial	-0.0975673885474836	0.435494517209625	0.820960220424486	00250 ; 00220 ; 00910 ;	aag01100 ; aag	KXJ69239.1 hypothet	XP_001654185.1glutamine synthetase 1, mitochondrial
AAEL01291	Golgi pH regulator	-0.0975625137444916	0.618961360876717	0.897318336565571	NA	NA	NA	XP_001663047.1Golgi pH regulator
AAEL00991	rootletin	-0.0973538608091319	0.750774172077419	0.932554085448433	NA	NA	NA	XP_021713034.1rootletin isoform X1
AAEL00188	eukaryotic translation initiation factor 4E-binding protein 1	-0.0973239600273049	0.391134905997479	0.793285976044306	NA	aag03013 ; aag	XP_001846418.1 Euk	XP_001654151.1eukaryotic translation initiation factor 4E-binding protein 1
AAEL00391	peripheral plasma membrane protein CASK	-0.0972987588867502	0.590050803652905	0.887668474886052	230	NA	XP_019933087.1 PRE	XP_021693237.1peripheral plasma membrane protein CASK isoform X7
AAEL00236	chymotrypsin-2	-0.0972936714331453	0.721621418865942	0.921632240999916	NA	NA	XP_019525841.1 PRE	XP_019525841.1 chymotrypsin-1-like
AAEL01009	small integral membrane protein 14	-0.0972762601573925	0.359849682660653	0.774255626424296	NA	NA	NA	XP_001654241.1small integral membrane protein 14
AAEL01050	LDLR chaperone boca	-0.0970895689741039	0.59262092859707	0.888529762455765	NA	NA	NA	XP_001660887.1LDLR chaperone boca
AAEL00079	polyubiquitin-C	-0.0969742943942686	0.436362122928503	0.820960220424486	NA	NA	XP_019543209.1 PRE	XP_019895692.1 polyubiquitin isoform X1
AAEL00590	protein TANC2	-0.0969606703413674	0.556516168987332	0.876744014213565	NA	NA	XP_019931121.1 PRE	XP_021708756.1protein TANC2 isoform X1
AAEL00763	dihydropyrimidinase	-0.0969541604066386	0.423109646794038	0.814192545906728	NA	aag01100 ; aag	XP_019537242.1 PRE	XP_001658524.2dihydropyrimidinase isoform X2
AAEL01212	probable 26S proteasome non-ATPase regulatory subunit 3	-0.0969326466259763	0.399908640686987	0.798648539435638	NA	aag03050	KXJ80696.1 hypothet	XP_001662261.1probable 26S proteasome non-ATPase regulatory subunit 3
AAEL00606	proteasome subunit alpha type-2	-0.0967760234311244	0.43587310422919	0.820960220424486	NA	aag03050	ETN63429.1 proteaso	XP_001651784.1proteasome subunit alpha type-2
AAEL01499	rab5 GDP/GTP exchange factor	-0.0967628826176754	0.566752778542149	0.880302387766415	NA	NA	NA	XP_021698848.1rab5 GDP/GTP exchange factor
AAEL00665	voltage-gated potassium channel subunit beta-1	-0.0966912808723193	0.556398293519523	0.876744014213565	NA	NA	XP_019534862.1 PRE	XP_019541076.1 voltage-gated potassium channel subunit beta-2-like
AAEL01408	general odorant-binding protein lush	-0.0966057995724942	0.742277521291679	0.929911685600432	NA	NA	NA	XP_001648015.1general odorant-binding protein lush
AAEL02539	NA	-0.0965932048420333	0.812509207733396	0.950268140513817	NA	NA	NA	NA
AAEL02266	NA	-0.0964973853137592	0.77160769621677	0.941147080452193	NA	NA	NA	XP_021701685.1zinc finger protein 2
AAEL02690	NA	-0.0964967137467786	0.598175752493415	0.890537659673905	NA	NA	NA	XP_021700119.1uncharacterized protein LOC5575905
AAEL02555	NA	-0.0964626425891588	0.508899547984098	0.860083070787677	NA	aag03010	NA	XP_001656254.260S ribosomal protein L15
AAEL00706	peptidyl-prolyl cis-trans isomerase-like 1	-0.0964046929058494	0.641610651231606	0.905347513233564	NA	aag03040	ETN59794.1 cyclophi	XP_001652536.1peptidyl-prolyl cis-trans isomerase-like 1
AAEL00606	UPF0547 protein C16orf87 homolog	-0.0963756901972634	0.738603521470729	0.928033958695205	NA	NA	KFB42182.1 AGAP00	XP_001657402.1UPF0547 protein C16orf87 homolog
AAEL01294	E3 ubiquitin-protein ligase MARCH5	-0.0960894267315171	0.573733822850026	0.881197098233729	NA	NA	NA	XP_001656302.2E3 ubiquitin-protein ligase MARCH5
AAEL02655	NA	-0.0960754333469597	0.64591951486357	0.906554905014882	NA	NA	NA	XP_019933026.1 ankyrin-2-like
AAEL02326	NA	-0.0959994052084938	0.713454810230996	0.919252089784076	NA	NA	NA	XP_021708035.1sodium channel protein para isoform X4
AAEL02131	NA	-0.0959334322210128	0.538787639065273	0.869670565558805	NA	NA	NA	XP_021694753.1uncharacterized protein LOC110674772 isoform X2
AAEL02471	NA	-0.0958602311179408	0.499369005551808	0.857312236770101	NA	NA	NA	XP_021697831.1ankyrin repeat domain-containing protein 11
AAEL01007	GPN-loop GTPase 1	-0.0958379017825142	0.6048480926444	0.893547770777888	NA	NA	NA	XP_001660613.1GPN-loop GTPase 1
AAEL00981	myotubularin-related protein 10-B	-0.0958114494024455	0.559346783233889	0.877581080322399	NA	NA	NA	XP_001660425.1myotubularin-related protein 10-B
AAEL01697	ABC transporter F family member 4	-0.095807110357015	0.43611421453493	0.820960220424486	NA	NA	NA	XP_011493091.2ABC transporter F family member 4
AAEL01322	60S ribosomal protein L10a	-0.0957790247773255	0.529135181206563	0.866002245975191	NA	aag03010	NA	XP_001656504.160S ribosomal protein L10a isoform X1
AAEL00916	extended synaptotagmin-2	-0.0957555104409123	0.458324182406697	0.833283524364043	NA	NA	NA	XP_001659792.2extended synaptotagmin-2 isoform X3
AAEL01047	calcineurin subunit B type 2	-0.0956830371796423	0.402363742309357	0.799341786858961	NA	aag04310	NA	XP_001660865.1calcineurin subunit B type 2
AAEL00958	protein obstructor-E	-0.0956308903164055	0.61836889804328	0.897318336565571	NA	NA	NA	XP_001660247.2protein obstructor-E isoform X2
AAEL00840	b(0,+)-type amino acid transporter 1	-0.0955466604903046	0.554582464417802	0.875346112791185	NA	NA	XP_001865021.1 cati	XP_001654593.1b(0,+)-type amino acid transporter 1
AAEL01275	NA	-0.0955208859897017	0.432562860420105	0.820005280371151	NA	aag04391	XP_019530798.1 PRE	XP_001662857.2arginine-glutamic acid dipeptide repeats protein
AAEL01043	exocyst complex component 8	-0.0955143269693683	0.576951978250936	0.881961491587822	NA	NA	NA	XP_001660822.1exocyst complex component 8
AAEL01175	putative aldehyde dehydrogenase family 7 member A1 homolog	-0.0955002498158075	0.522715210260357	0.865112475686296	NA	aag01100 ; aag	KXJ73424.1 hypothet	XP_001655664.1putative aldehyde dehydrogenase family 7 member A1 homolog
AAEL00503	uncharacterized LOC5565844	-0.0953135803645797	0.776837270701404	0.942198044680943	NA	NA	XP_019530854.1 PRE	XP_019530854.1 gastrula zinc finger protein xFG20-1 isoform X1
AAEL02074	NA	-0.0953098523267536	0.746136105235671	0.931365937538534	NA	NA	NA	NA
AAEL01984	NA	-0.0952391495437648	0.694622846300493	0.916122517126697	NA	NA	NA	XP_021693726.1uncharacterized protein LOC5575947 isoform X1
AAEL00606	shootin-1	-0.0951660353292979	0.558515261828668	0.877581080322399	NA	NA	XP_001845443.1 con	XP_019561859.1 shootin-1-like
AAEL00968	chymotrypsin-2	-0.0951307830678982	0.554456522314083	0.875297464116348	NA	NA	NA	XP_019525646.1 chymotrypsin-2-like
AAEL00856	TPPP family protein CG45057	-0.0950720505972027	0.505410760848386	0.858744381072989	NA	NA	XP_019554789.1 PRE	XP_001653296.1TPPP family protein CG45057 isoform X1
AAEL02218	NA	-0.0949620863868424	0.464992840992972	0.838171228759516	NA	NA	NA	XP_021698137.1formin-binding protein 1-like isoform X1
AAEL01710	ribonuclease H1	-0.0949171930748721	0.682471529313667	0.914629465876247	NA	aag03030	NA	XP_011493451.1ribonuclease H1
AAEL02224	NA	-0.0948051000741168	0.446455517160591	0.828189271329869	NA	NA	NA	XP_001650683.2UPF0518 protein AAEL005291 isoform X2
AAEL01364	transcription factor A, mitochondrial	-0.0947972767405003	0.534921945501098	0.866763821535675	NA	NA	NA	XP_021701535.1transcription factor A, mitochondrial
AAEL01125	pre-mRNA-splicing factor RBM22	-0.0947476370460034	0.705117654065456	0.917749125552377	NA	aag03040	ETN65205.1 RNA bin	XP_001661533.1pre-mRNA-splicing factor RBM22
AAEL01293	methyltransferase-like protein 6	-0.0947439685062294	0.469976925384227	0.840895910438588	NA	NA	NA	XP_001656312.1methyltransferase-like protein 6
AAEL00845	alpha-amylase A	-0.0945545914307008	0.574828388399656	0.881197098233729	00500 ; 00500	aag01100 ; aag	XP_001846545.1 alp	XP_001653283.2alpha-amylase A
AAEL00409	hemicentin-1	-0.0944798086900841	0.37932106447942	0.785666748907449	NA	NA	XP_019536295.1 PRE	XP_019536295.1 hemicentin-1-like isoform X1
AAEL00041	monocarboxylate transporter 14	-0.0944578463517988	0.422336371075006	0.814192545906728	NA	NA	XP_019540725.1 PRE	XP_021705996.1monocarboxylate transporter 14 isoform X2
AAEL00009	SUMO-activating enzyme subunit 1	-0.0943829066522787	0.579496047952346	0.882283691803025	NA	aag04120	ETN60043.1 sumo-1-	XP_001647884.1SUMO-activating enzyme subunit 1
AAEL00781	trypsin 3A1	-0.094349602658447	0.555402314265229	0.876039097680603	NA	NA	XP_019565084.1 PRE	XP_001652944.1trypsin 3A1 isoform X2
AAEL00542	cyclin-G-associated kinase	-0.0942953082967556	0.562687565145058	0.879512940524628	NA	NA	XP_001850042.1 cyc	XP_021693131.1cyclin-G-associated kinase
AAEL01471	uncharacterized LOC5565079	-0.0942904140319955	0.606704095387204	0.893547770777888	NA	NA	NA	4FD4_AChain A, Crystal Structure Of Mosquito Arylalkylamine N-Acetyltransferase Like 5b
AAEL00972	zinc finger protein Xfin-like	-0.0942001973658528	0.766182667561782	0.939178856256477	NA	NA	NA	XP_021713124.1zinc finger protein Xfin-like isoform X2
AAEL00546	6-phosphofructo-2-kinase/fructose-2,6-bisphosphatase 1	-0.0941400858808586	0.437317674700382	0.820960220424486	51	NA	XP_019538618.1 PRE	XP_021701154.16-phosphofructo-2-kinase/fructose-2,6-bisphosphatase 1 isoform X1
AAEL00908	protein KRI1 homolog	-0.0939051200036487	0.634341744866311	0.902506916959539	NA	NA	KXJ79330.1 hypothet	XP_001659705.2protein KRI1 homolog
AAEL02751	NA	-0.0938265236114901	0.668003253833637	0.912418872165226	NA	NA	NA	XP_021709310.1uncharacterized protein LOC110679302 isoform X1
AAEL00780	semaphorin-5A	-0.0937848094776355	0.588005212591401	0.886401261871374	NA	NA	XP_001862914.1 sen	XP_019529147.1 semaphorin-5A-like
AAEL02797	NA	-0.093772207424203	0.42858107258063	0.816740033999805	NA	NA	NA	XP_021710585.1cytochrome c oxidase assembly protein COX16 homolog, mitochondrial
AAEL01944	NA	-0.0936284759420487	0.506760407634654	0.859256178927183	NA	NA	NA	XP_021705125.1actin-binding Rho-activating protein isoform X3
AAEL01396	tRNA (cytosine(34)-C(5))-methyltransferase	-0.093628413763015	0.40155306712963	0.799111445442658	NA	NA	NA	XP_001664204.1tRNA (cytosine(34)-C(5))-methyltransferase
AAEL01380	BLOC-1-related complex subunit 5	-0.0934616882819324	0.824415143468064	0.954996283354524	NA	NA	NA	XP_021693098.1BLOC-1-related complex subunit 5
AAEL01026	prohormone-3	-0.0933933079997809	0.498646197185925	0.856678287334277	NA	NA	NA	XP_001660713.1prohormone-3 isoform X3
AAEL01208	uncharacterized LOC5575814	-0.0933409683961572	0.425923438520341	0.814623464645531	NA	NA	KXJ73110.1 hypothet	XP_019932596.1 protein artichoke-like
AAEL00455	synaptosomal-associated protein 25	-0.0933132465821294	0.417324564113612	0.811885970184662	NA	NA	XP_019541304.1 PRE	XP_021705500.1synaptosomal-associated protein 25 isoform X3
AAEL00916	protein 60A	-0.0929682949417031	0.605845279541283	0.893547770777888	NA	aag04350	ETN63639.1 bone mo	XP_001659788.1protein 60A
AAEL02402	NA	-0.0929116873810179	0.605721334515779	0.893547770777888	NA	NA	NA	XP_021703869.1A-kinase anchor protein 10, mitochondrial
AAEL00613	nuclear cap-binding protein subunit 2	-0.0928731107341514	0.727991017305022	0.923566968708516	NA	aag03013 ; aag	ETN63045.1 nuclear	XP_001657521.1nuclear cap-binding protein subunit 2
AAEL00159	zinc finger protein 271	-0.0928388447543083	0.785554647224688	0.944372632355572	NA	NA	KXJ69279.1 hypothet	XP_021707419.1zinc finger protein 271
AAEL02696	NA	-0.0928347613481712	0.412386187321646	0.806884477085645	NA	NA	NA	XP_021712075.1 troponin T, skeletal muscle-like
AAEL00800	NADH dehydrogenase [ubiquinone] 1 beta subcomplex subunit 11, mitoc	-0.0928093124392739	0.437005425569627	0.820960220424486	NA	aag01100 ; aag	ETN63210.1 mitocho	XP_001658796.1NADH dehydrogenase [ubiquinone] 1 beta subcomplex subunit 11, mitochondrial
AAEL00914	prolyl endopeptidase	-0.0927816179972097	0.440229703957389	0.822798752521693	NA	NA	KXJ72049.1 hypothet	XP_001659779.1prolyl endopeptidase

AAEL02536	NA	-0.0927573190075644	0.469640420533733	0.84065046403825	NA	aag03010	NA	XP_001655466.160S ribosomal protein L10
AAEL00047	beta-catenin-like protein 1	-0.0927524336779924	0.604006946148401	0.893547770777888	NA	aag03040	KXJ76861.1 hypothet	XP_001656979.1beta-catenin-like protein 1
AAEL00719	NA	-0.0927054190200699	0.440008779091389	0.822798752521693	NA	NA	XP_019526624.1 PRE	XP_001658216.2proton-coupled amino acid transporter-like protein pathetic isoform X2
AAEL00336	nucleolar complex protein 4 homolog A	-0.092621025414176	0.538697559004905	0.869670565558805	NA	NA	KXJ68214.1 hypothet	XP_001656754.1nucleolar complex protein 4 homolog A
AAEL00786	cytochrome b-c1 complex subunit 7	-0.0924518067808905	0.512318922049142	0.861177338330732	NA	aag01100 ; aag	ETN66225.1 ubiquin	XP_001652964.1cytochrome b-c1 complex subunit 7
AAEL01833	NA	-0.0923376355028786	0.702592599535929	0.917106722424789	NA	NA	NA	XP_001650493.2uncharacterized protein LOC5566132
AAEL00450	40S ribosomal protein S21	-0.0923171224689081	0.536160648840115	0.867854052880692	NA	NA	KXJ68578.1 hypothet	XP_001647949.140S ribosomal protein S21
AAEL01221	ubiquitin carboxyl-terminal hydrolase 15	-0.0922862823608325	0.547550628246868	0.872025074615382	NA	aag04144 ; aag	KXJ73186.1 hypothet	XP_021711514.1ubiquitin carboxyl-terminal hydrolase 15 isoform X2
AAEL02348	NA	-0.0922128327783405	0.45248017497156	0.832078850936909	NA	NA	NA	XP_021706136.1uncharacterized protein LOC5572713 isoform X1
AAEL00407	ubiquitin-like modifier-activating enzyme 5	-0.0921886619824214	0.530268787742339	0.866606412563369	NA	NA	KXJ71409.1 hypothet	XP_021703960.1ubiquitin-like modifier-activating enzyme 5
AAEL00839	leucine-rich repeat serine/threonine-protein kinase 1	-0.0921668663100831	0.573340207846356	0.881074915264223	04151 ; 05165 ; 04714 ;	NA	XP_019538485.1 PRE	XP_021698550.1leucine-rich repeat serine/threonine-protein kinase 1 isoform X1
AAEL00639	V-type proton ATPase 116 kDa subunit a	-0.0921166970840922	0.402704412955237	0.799845889923179	NA	aag01100 ; aag	KXJ74452.1 hypothet	XP_001657753.1V-type proton ATPase 116 kDa subunit a
AAEL00374	V-type proton ATPase 116 kDa subunit a	-0.0920810872504523	0.398195797407175	0.797605077871316	NA	aag01100 ; aag	XP_019526360.1 PRE	XP_001657232.1V-type proton ATPase 116 kDa subunit a isoform X3
AAEL02122	NA	-0.0920376528970773	0.647870926045885	0.906554905014882	NA	NA	NA	XP_021704142.1protein enabled isoform X6
AAEL00989	uncharacterized LOC5572554	-0.0920009191431578	0.573722383558815	0.881197098233729	NA	NA	NA	XP_001654068.2uncharacterized protein LOC5572554 isoform X2
AAEL00967	membrane-associated protein Hem	-0.091901385385853	0.569452032023452	0.880635379772131	NA	NA	NA	XP_021699423.1membrane-associated protein Hem
AAEL01180	zinc finger protein 569	-0.0918524078757356	0.801440093349172	0.946215055875588	NA	NA	KXJ72289.1 hypothet	XP_021706088.1zinc finger protein 569
AAEL00281	nischarin	-0.0918481454957819	0.644109250804831	0.906332602788493	NA	NA	KXJ74360.1 hypothet	XP_001656019.1nischarin isoform X2
AAEL02443	NA	-0.0918246814053979	0.54117336716696	0.869735874782513	NA	NA	NA	XP_021704810.160S ribosomal protein L13a-like
AAEL02638	NA	-0.0918004693634867	0.688779203825647	0.916010156153545	NA	NA	NA	XP_021709497.1AT-rich interactive domain-containing protein 5B
AAEL01017	peptidoglycan-recognition protein LB	-0.0917624329335141	0.462973749225413	0.837264939413983	NA	NA	NA	XP_001654275.2peptidoglycan-recognition protein LB isoform X2
AAEL00294	late secretory pathway protein AVL9 homolog	-0.0917445634875754	0.652121514164826	0.907272485895905	NA	NA	KXJ70507.1 hypothet	XP_021697338.1late secretory pathway protein AVL9 homolog
AAEL00395	calpain-A	-0.091714248272602	0.450718924059937	0.830259097859495	NA	NA	NA	XP_021708585.1calpain-A isoform X3
AAEL00082	kinesin-like protein Klp68D	-0.0916251206498684	0.607228941211247	0.893547770777888	NA	NA	XP_019537920.1 PRE	XP_001651285.1kinesin-like protein Klp68D
AAEL01316	cactin	-0.0916104405397443	0.668003253829937	0.912418872165226	NA	NA	NA	XP_019531299.1 cactin-like
AAEL00753	NA	-0.0916005440059666	0.579027304868263	0.882283691803025	NA	NA	NA	XP_021707895.1transcription intermediary factor 1-alpha isoform X4
AAEL00445	cytochrome c-2	-0.0915155004426436	0.527223966264189	0.865973876319045	NA	aag04214 ; aag	ETN58438.1 Cytochr	XP_001649236.1cytochrome c-2
AAEL01081	zinc finger protein 420	-0.0914345625910548	0.677694107429349	0.914481546552978	NA	NA	XP_019529612.1 PRE	XP_021704560.1zinc finger protein 26 isoform X1
AAEL01174	uncharacterized LOC5575270	-0.091432506025598	0.482174346847938	0.846941547268585	NA	NA	KXJ82316.1 hypothet	XP_001661875.1uncharacterized protein LOC5575270
AAEL00395	serine/threonine-protein kinase MARK2	-0.0913669739106147	0.420986049524092	0.813840460658889	04151 ; 05165 ; 04714 ;	NA	NA	XP_021704652.1MAP/microtubule affinity-regulating kinase 3 isoform X4
AAEL00301	NADH dehydrogenase [ubiquinone] 1 alpha subcomplex subunit 2	-0.0910914194738998	0.498058426165396	0.856340365764943	NA	aag01100 ; aag	ETN58785.1 NADH-u	XP_001662904.1NADH dehydrogenase [ubiquinone] 1 alpha subcomplex subunit 2
AAEL00703	prolyl 4-hydroxylase subunit alpha-1	-0.091031291645098	0.491008152622523	0.851056134946781	330	aag01100 ; aag	XP_019552003.1 PRE	XP_001658091.2prolyl 4-hydroxylase subunit alpha-1
AAEL00214	oocyte zinc finger protein XlCOF6	-0.0909819549483697	0.59516291865151	0.889050526332724	NA	NA	KXJ72030.1 hypothet	XP_021693080.1oocyte zinc finger protein XlCOF6
AAEL00416	CDGSH iron-sulfur domain-containing protein 3, mitochondrial	-0.0909315542070602	0.553811355133471	0.874920236325033	NA	NA	KXJ67776.1 hypothet	XP_001648564.1CDGSH iron-sulfur domain-containing protein 3, mitochondrial
AAEL02332	NA	-0.0909181556669369	0.62224933163385	0.898271200863489	NA	aag04146	NA	XP_021712993.1peroxisomal membrane protein PEX16
AAEL02063	NA	-0.0908957299369856	0.542944076890355	0.869900911343623	NA	NA	NA	XP_019554124.1 ankyrin repeat domain-containing protein 50-like
AAEL01188	hsc70-interacting protein 1	-0.0908479325373347	0.446231255693131	0.828189271329869	NA	NA	KXJ73379.1 hypothet	XP_001655769.1hsc70-interacting protein 1
AAEL00202	ejaculatory bulb-specific protein 3	-0.0908008971360595	0.770130431795041	0.941147080452193	NA	NA	KXJ74363.1 hypothet	XP_001660785.1ejaculatory bulb-specific protein 3
AAEL00406	translocator protein	-0.0907580557877059	0.513968502614931	0.861448202130317	NA	aag04214 ; aag	KFB36810.1 AGAP00	XP_001648359.1translocator protein
AAEL00752	peroxisomal N(1)-acetyl-spermine/spermidine oxidase	-0.0906493650197049	0.650640712931095	0.906932039402093	NA	NA	KXJ69112.1 hypothet	XP_001652819.2peroxisomal N(1)-acetyl-spermine/spermidine oxidase
AAEL01997	NA	-0.0906052981816728	0.758466842872848	0.935439571457873	NA	NA	NA	XP_021704383.1protein tweety-2 isoform X2
AAEL02344	NA	-0.0905407091592298	0.767733660513678	0.939827742988808	NA	NA	NA	NA
AAEL01143	threonylcarbamoyladenosine tRNA methylthiotransferase	-0.0905373094388895	0.574821267366804	0.881197098233729	NA	NA	XP_001842139.1 aq_	XP_021699838.1threonylcarbamoyladenosine tRNA methylthiotransferase
AAEL00972	zinc finger protein 366	-0.0904715416399457	0.772283477990022	0.941147080452193	NA	NA	NA	XP_021713125.1zinc finger protein 366
AAEL00259	WW domain-binding protein 2	-0.0903871685126066	0.510506378316949	0.860452247709668	NA	NA	ETN61711.1 WW dor	XP_001655696.1WW domain-binding protein 2
AAEL00919	NA	-0.0903747944940315	0.422363161127655	0.814192545906728	NA	NA	NA	XP_019557912.1 neurotrypsin-like isoform X1
AAEL02143	NA	-0.0903303160666717	0.729373224661306	0.923905484505959	564	aag00564	NA	AAB35001.1acetylcholinesterase, Ace
AAEL01283	cytochrome b561	-0.0902018677548318	0.684084560209508	0.914629465876247	NA	NA	NA	XP_021712866.1cytochrome b561 isoform X2
AAEL01080	cytochrome b-c1 complex subunit 6, mitochondrial	-0.090067018166923	0.509508153792997	0.860083070787677	NA	aag01100 ; aag	KFB35239.1 hypothe	XP_001654917.1cytochrome b-c1 complex subunit 6, mitochondrial
AAEL00636	N-acetyl-D-glucosamine kinase	-0.0900553221450629	0.534500891880192	0.866700945213732	520	aag01100 ; aag	KXJ71477.1 hypothet	XP_001651965.2N-acetyl-D-glucosamine kinase
AAEL02761	NA	-0.0900441509045463	0.571912529960953	0.880923047085115	NA	NA	NA	XP_021696083.1mediator of RNA polymerase II transcription subunit 13 isoform X1
AAEL00086	protein unzipped	-0.0898442594868046	0.440888450340252	0.823099111387908	NA	NA	KXJ72309.1 hypothet	XP_001651602.1protein unzipped
AAEL01241	N-alpha-acetyltransferase 30	-0.0898061938749364	0.588370802920868	0.886554466755825	NA	NA	KXJ77568.1 hypothet	XP_001656034.2N-alpha-acetyltransferase 30
AAEL00139	peptidylglycine alpha-hydroxylating monooxygenase	-0.089783345499364	0.423586650379731	0.814192545906728	NA	NA	KXJ72445.1 hypothet	XP_001659177.2peptidylglycine alpha-hydroxylating monooxygenase
AAEL00183	carboxypeptidase B	-0.0896795642165583	0.7820961258645	0.943962424451135	NA	NA	XP_019537524.1 PRE	XP_001654131.2carboxypeptidase B
AAEL02301	NA	-0.0896462754462141	0.476141763932089	0.844867278451347	NA	NA	NA	XP_021709303.1potential E3 ubiquitin-protein ligase ariadne-1
AAEL01131	zinc finger protein 708	-0.0892526071450706	0.677028762797883	0.914438668430353	NA	NA	XP_001847687.1 zinc	XP_021709503.1zinc finger protein 708
AAEL00987	alanine aminotransferase 1	-0.089244902781316	0.476557024002511	0.844867278451347	NA	aag01100 ; aag	NA	XP_001660470.2alanine aminotransferase 1
AAEL02245	NA	-0.0892056918493937	0.720307765609754	0.921583569204186	NA	NA	NA	XP_021705310.1uncharacterized protein LOC110677878
AAEL02134	NA	-0.0890963345725738	0.420698782209626	0.813636239562413	NA	aag01100 ; aag	NA	XP_001661299.1V-type proton ATPase subunit d 1
AAEL01464	uncharacterized LOC5564898	-0.0890536364735782	0.619396663577183	0.897318336565571	NA	NA	NA	XP_001649248.2uncharacterized protein LOC5564898
AAEL01012	equilibrative nucleoside transporter 1	-0.0890281663622747	0.539675230353932	0.869709828277711	NA	NA	NA	XP_001660636.1equilibrative nucleoside transporter 1
AAEL01164	transferrin	-0.0890251299525058	0.534599446656398	0.866700945213732	NA	NA	KFB47544.1 AGAP01	XP_019542280.1 transferrin-like
AAEL01433	uncharacterized protein F13E9.13, mitochondrial	-0.0888963161513032	0.646453817263145	0.906554905014882	NA	NA	NA	XP_001648526.1uncharacterized protein F13E9.13, mitochondrial isoform X1
AAEL01201	DEAD-box helicase Dbp80	-0.0888587743289861	0.735091896134878	0.926618579210021	NA	NA	KXJ76898.1 hypothet	XP_001662155.1DEAD-box helicase Dbp80
AAEL00589	MICOS complex subunit MIC27	-0.0887042818784273	0.472754881818033	0.843242958240148	NA	NA	KXJ80674.1 hypothet	XP_001651576.2MICOS complex subunit MIC27
AAEL01112	O-glucosyltransferase rumi homolog	-0.0886370723699201	0.775934778201776	0.941848282418248	NA	aag00514	KXJ70493.1 hypothet	XP_001655120.1O-glucosyltransferase rumi homolog
AAEL02433	NA	-0.088589187578475	0.588864950381633	0.88684610505111	NA	NA	NA	XP_019528526.1 sialin-like
AAEL01997	NA	-0.0884155294330103	0.755957482431076	0.934127170333935	NA	NA	NA	XP_001848250.1wd-repeat protein
AAEL01135	DNA polymerase subunit gamma-2, mitochondrial	-0.0883874894698425	0.670839628488309	0.912812708172959	970	NA	XP_001869239.1 mit	XP_001655300.2DNA polymerase subunit gamma-2, mitochondrial
AAEL01325	ras-related protein Rab-14	-0.0883845067227212	0.465937838263263	0.838483018891546	NA	NA	NA	XP_001656550.2ras-related protein Rab-14 isoform X1
AAEL01975	NA	-0.0881696494304085	0.715721117518009	0.920421919040713	NA	NA	NA	XP_021713398.1 neurexin-1
AAEL01999	NA	-0.0881612122229436	0.586397838081789	0.885466509029968	NA	NA	NA	XP_021704754.1protein unc-45 homolog B
AAEL01050	vacuolar protein sorting-associated protein 13C	-0.0881540901233328	0.414292957034959	0.80872375702921	NA	NA	NA	XP_021697205.1vacuolar protein sorting-associated protein 13C isoform X2
AAEL00072	RNA-binding protein Musashi homolog Rbp6	-0.088127950229939	0.580647799408776	0.883199181536083	NA	aag03015	XP_019536480.1 PRE	XP_019536480.1 RNA-binding protein Musashi homolog Rbp6 isoform X1
AAEL00559	zinc finger protein 316	-0.0880880778416152	0.848047257687002	0.963435772275933	NA	NA	XP_019536254.1 PRE	XP_001651115.1zinc finger protein 316
AAEL00394	60S ribosomal protein L44	-0.0879920806595888	0.591503772571646	0.888365874849728	NA	aag03010	ETN66160.1 60S ribo	XP_001648064.160S ribosomal protein L44
AAEL00281	ATP synthase-coupling factor 6, mitochondrial	-0.0879726545660728	0.530304358742049	0.866606412563369	NA	aag01100 ; aag	ACL37991.1 mitocho	XP_001662452.2ATP synthase-coupling factor 6, mitochondrial
AAEL00738	serine-threonine kinase receptor-associated protein	-0.0878396430597553	0.417025839192713	0.811697838844202	NA	aag03013	ETN67896.1 serine-th	XP_001652722.1serine-threonine kinase receptor-associated protein
AAEL01276	cytochrome P450 4C1	-0.0877758052247872	0.80981260912287	0.949044272688315	NA	NA	XP_019543097.1 PRE	XP_001662866.2cytochrome P450 4C1
AAEL02641	NA	-0.0875611460288867	0.529765407006967	0.866341083219852	NA	aag01100 ; aag	NA	XP_021711516.1putative polypeptide N-acetylgalactosaminyltransferase 9 isoform X1
AAEL01134	protocadherin Fat 3	-0.087490861958962	0.563823604738438	0.879838896781538	NA	NA	KFB42209.1 AGAP00	XP_021697227.1protocadherin Fat 3

AAEL0100[illegible]	protein PTCD3 homolog, mitochondrial	-0.0874137694674428	0.544562272915775	0.870429396451444	NA	NA	NA	XP_001660592.2protein PTCD3 homolog, mitochondrial
AAEL0112[illegible]	PQ-loop repeat-containing protein 3	-0.0873891761801796	0.530752746349184	0.866691320214768	NA	NA	XP_001862340.1 con	XP_001655189.2PQ-loop repeat-containing protein 3
AAEL0104[illegible]	CSC1-like protein 1	-0.0873835211335797	0.516541210222206	0.861530579862055	NA	NA	NA	XP_001654529.1CSC1-like protein 1
AAEL0016[illegible]	zinc transporter ZIP1	-0.0872707384650283	0.617255969846792	0.897318336565571	NA	NA	KXJ74939.1 hypothet	XP_001659748.2zinc transporter ZIP1
AAEL0197[illegible]	NA	-0.0872612884294496	0.445588748103927	0.82793174864877	NA	aag01100 ; aag	NA	XP_021696839.1succinyl-CoA:3-ketoacid coenzyme A transferase 1, mitochondrial isoform X2
AAEL0121[illegible]	32 kDa beta-galactoside-binding lectin lec-3	-0.0872333819665162	0.616685492340708	0.897164966965308	NA	NA	KXJ68925.1 hypothet	XP_001655870.132 kDa beta-galactoside-binding lectin lec-3
AAEL0216[illegible]	NA	-0.087145111563537	0.513024828225165	0.861297436611866	NA	NA	NA	XP_021697226.1uncharacterized protein LOC5572744
AAEL0062[illegible]	superoxide dismutase [Cu-Zn], chloroplastic	-0.0870181876807264	0.438421385437861	0.821493298472653	NA	aag04146 ; aag	KXJ75688.1 hypothet	XP_021699793.1superoxide dismutase [Cu-Zn], chloroplastic
AAEL0210[illegible]	NA	-0.087009429647396	0.615165513628951	0.896845306410123	230	aag01100 ; aag	NA	XP_021704103.1cAMP-specific 3',5'-cyclic phosphodiesterase isoform X4
AAEL0017[illegible]	DNA polymerase epsilon subunit 3	-0.0869314252001451	0.689704991295105	0.916122517126697	NA	aag03420 ; aag	XP_001867416.1 DN	XP_001653952.2DNA polymerase epsilon subunit 3
AAEL0006[illegible]	protein flightless-1 homolog	-0.0869195665082499	0.651712522002361	0.907172139858737	NA	NA	XP_019556937.1 PRE	XP_001649779.2protein flightless-1 homolog
AAEL0092[illegible]	putative mediator of RNA polymerase II transcription subunit 12	-0.0869192857941176	0.64236197848817	0.905763856274616	NA	NA	NA	XP_001659839.2putative mediator of RNA polymerase II transcription subunit 12 isoform X1
AAEL0274[illegible]	NA	-0.0868844044421322	0.619161437408984	0.897318336565571	NA	NA	NA	XP_021699004.1SURF1-like protein
AAEL0106[illegible]	phospholipid scramblase 1	-0.086832851409386	0.485196300422117	0.847655581875201	NA	NA	NA	XP_001661000.2phospholipid scramblase 2 isoform X2
AAEL0274[illegible]	NA	-0.0867081648800748	0.802755003497583	0.94649029363412	NA	NA	NA	XP_021713246.1ATP-binding cassette sub-family G member 5
AAEL0062[illegible]	uncharacterized LOC5567696	-0.0866427799336881	0.70672396710185	0.91797784850989	NA	aag01100 ; aag	NA	XP_001651860.2uncharacterized protein LOC5567696
AAEL0260[illegible]	NA	-0.0866312453132728	0.440579399909387	0.822798752521693	NA	NA	NA	XP_021698471.1 protein FAM13A
AAEL0092[illegible]	phosphatidylinositol 4-kinase beta	-0.0866176598089211	0.478619314727004	0.845416382162313	NA	aag01100 ; aag	NA	XP_021695754.1phosphatidylinositol 4-kinase beta isoform X2
AAEL0195[illegible]	NA	-0.0865918385067747	0.469657034951167	0.84065046403825	NA	NA	NA	XP_021711641.1uncharacterized protein LOC5568599 isoform X1
AAEL0212[illegible]	NA	-0.0865280173862251	0.460018902218294	0.834169795839746	NA	NA	NA	XP_021709445.1transmembrane protein 53-A isoform X1
AAEL0132[illegible]	ubiquitin carboxyl-terminal hydrolase 64E	-0.0865097206580565	0.424890984607904	0.814623464645531	NA	NA	NA	XP_001663387.2ubiquitin carboxyl-terminal hydrolase 64E isoform X1
AAEL0049[illegible]	neuronal acetylcholine receptor subunit alpha-4	-0.0864068368696979	0.524030871363137	0.865215478807089	NA	NA	XP_019529517.1 PRE	XP_001650052.2neuronal acetylcholine receptor subunit alpha-4 isoform X1
AAEL0010[illegible]	cyclin-dependent kinase 7	-0.0863951415340284	0.716675353929068	0.920939339038582	NA	aag03420 ; aag	KXJ74709.1 hypothet	XP_001657865.1cyclin-dependent kinase 7
AAEL0061[illegible]	uncharacterized LOC5567579	-0.0863720530696558	0.661740349810832	0.909942423634082	NA	NA	XP_001843133.1 con	XP_021707137.1uncharacterized protein LOC5567579
AAEL0145[illegible]	homeobox protein PKNOX1	-0.0863687516351433	0.876008717050619	0.969652664055335	NA	NA	NA	XP_021696774.1homeobox protein PKNOX1
AAEL0239[illegible]	NA	-0.0862997608375003	0.572004461778785	0.880923047085115	NA	NA	NA	XP_021712810.1uncharacterized protein LOC110681377
AAEL0066[illegible]	ELAV-like protein 4	-0.0862506209648755	0.531197850112012	0.866691320214768	NA	NA	ETN58341.1 RNA-bin	XP_021701569.1ELAV-like protein 3 isoform X1
AAEL0142[illegible]	complex I intermediate-associated protein 30, mitochondrial	-0.0862463082562853	0.584090433679801	0.885175928718656	NA	NA	NA	XP_001648190.1complex I intermediate-associated protein 30, mitochondrial
AAEL0114[illegible]	perlucin-like protein	-0.0862050299723759	0.641600942791941	0.905347513233564	NA	NA	XP_019541732.1 PRE	XP_001661683.2perlucin-like protein
AAEL0027[illegible]	uncharacterized LOC5576103	-0.0861661097033587	0.560054741978814	0.877810208770064	NA	NA	KXJ74749.1 hypothet	ABF18360.1mitochondrial NADH-ubiquinone oxidoreductase 9 kDa subunit-like protein
AAEL0006[illegible]	peptide methionine sulfoxide reductase	-0.0860645889832874	0.780531608241072	0.943817589886248	NA	NA	KXJ75616.1 hypothet	XP_001649798.1peptide methionine sulfoxide reductase
AAEL0072[illegible]	protein ROP	-0.0859951121080756	0.476701697998446	0.844867278451347	NA	NA	KXJ69336.1 hypothet	XP_001652658.1protein ROP isoform X1
AAEL0107[illegible]	GRIP and coiled-coil domain-containing protein 1	-0.0859546127888061	0.713320675640109	0.919252089784076	NA	NA	KXJ68920.1 hypothet	XP_001654860.1GRIP and coiled-coil domain-containing protein 1
AAEL0252[illegible]	NA	-0.0858806819682744	0.818311993170559	0.952816267386497	NA	NA	NA	XP_021695630.1uncharacterized protein LOC5575770 isoform X3
AAEL0098[illegible]	E3 ubiquitin-protein ligase znrf2	-0.0857953486764537	0.453471497526569	0.832617462606238	NA	NA	NA	XP_021698233.1E3 ubiquitin-protein ligase znrf2 isoform X1
AAEL0241[illegible]	NA	-0.0857873183912624	0.505738543069133	0.858744381072989	62	NA	NA	XP_021693689.1elongation of very long chain fatty acids protein AAEL008004 isoform X1
AAEL0040[illegible]	flotillin-2	-0.0857458985172367	0.542912772072461	0.869900911343623	NA	NA	XP_019531613.1 PRE	XP_021710660.1flotillin-2 isoform X2
AAEL0242[illegible]	NA	-0.0857077093956631	0.759034178422674	0.935439571457873	NA	NA	NA	XP_021699408.1zinc finger protein Xfin-like
AAEL0053[illegible]	syntenin-1	-0.0856562650652933	0.478934875645384	0.845454613425911	NA	NA	XP_019528994.1 PRE	ABF18402.1syndecan binding protein
AAEL0063[illegible]	microtubule-associated serine/threonine-protein kinase 3	-0.0854683382151119	0.508208578472856	0.859730781244726	04151 ; 05165 ; 04714 ;	NA	KXJ78329.1 hypothet	XP_001651908.2microtubule-associated serine/threonine-protein kinase 3 isoform X1
AAEL0017[illegible]	uncharacterized LOC5572286	-0.0854674130255281	0.696274413374934	0.916122517126697	NA	NA	XP_001846575.1 con	XP_019562499.1 pigment-dispersing hormone peptides-like
AAEL0013[illegible]	serine/arginine repetitive matrix protein 2	-0.085465342992774	0.691424158050578	0.916122517126697	NA	NA	KXJ68339.1 hypothet	XP_001653059.1serine/arginine repetitive matrix protein 2
AAEL0141[illegible]	Y+L amino acid transporter 2	-0.0852607493248512	0.553475296171197	0.874648750707084	NA	NA	NA	XP_021704627.1Y+L amino acid transporter 2
AAEL0104[illegible]	glutamate dehydrogenase, mitochondrial	-0.0851522547943358	0.44335296009352	0.82545872799161	NA	aag01100 ; aag	NA	XP_001660861.1glutamate dehydrogenase, mitochondrial isoform X2
AAEL0028[illegible]	Down syndrome critical region protein 3 homolog	-0.0850528810392037	0.818559200121903	0.952837221640997	NA	NA	KXJ74314.1 hypothet	XP_001662458.2Down syndrome critical region protein 3 homolog isoform X1
AAEL0145[illegible]	protein-serine O-palmitoleoyltransferase porcupine	-0.0849351514115688	0.817553221960679	0.952514760254484	NA	aag04310	NA	XP_021699864.1protein-serine O-palmitoleoyltransferase porcupine isoform X1
AAEL0196[illegible]	NA	-0.0848885858897568	0.434449642828839	0.820960220424486	NA	NA	NA	XP_021698211.1synaptic vesicle membrane protein VAT-1 homolog-like
AAEL0100[illegible]	DNA replication licensing factor MCM4	-0.0848728949177591	0.672241650276048	0.912897695079764	NA	aag03030	NA	XP_021699399.1DNA replication licensing factor MCM4
AAEL0151[illegible]	glycosylphosphatidylinositol anchor biosynthesis protein 11	-0.0847409069295279	0.803710997249384	0.94649029363412	NA	aag01100 ; aag	NA	XP_021697526.1glycosylphosphatidylinositol anchor biosynthesis protein 11
AAEL0090[illegible]	autophagy protein 12-like	-0.084714994586113	0.743999853495629	0.930834351035592	NA	aag04140 ; aag	ETN65795.1 autopha	XP_001659704.1autophagy protein 12-like
AAEL0095[illegible]	DNA-binding protein Ets97D	-0.0846898918576254	0.672051101134399	0.912813925547137	NA	NA	NA	XP_021713207.1DNA-binding protein Ets97D
AAEL0050[illegible]	multidrug resistance-associated protein 1	-0.0846544736438254	0.833444843319269	0.959902797120062	NA	NA	XP_019535568.1 PRE	XP_021703626.1multidrug resistance-associated protein 1
AAEL0044[illegible]	grpE protein homolog, mitochondrial	-0.0846288225115646	0.48809126632234	0.848846834445739	NA	NA	KXJ69638.1 hypothet	XP_001649137.1grpE protein homolog, mitochondrial
AAEL0122[illegible]	fatty acid hydroxylase domain-containing protein 2	-0.0846197857615048	0.653189294827662	0.907272485895905	NA	NA	KFB52171.1 AGAP00	XP_001662401.2fatty acid hydroxylase domain-containing protein 2
AAEL0252[illegible]	NA	-0.0845752674783247	0.648104703309208	0.906554905014882	NA	NA	NA	XP_021699422.1uncharacterized protein LOC5577131
AAEL0097[illegible]	ubiquitin carboxyl-terminal hydrolase 20	-0.0843730938155999	0.572374085033992	0.880923047085115	NA	NA	NA	XP_001653999.2ubiquitin carboxyl-terminal hydrolase 20 isoform X2
AAEL0268[illegible]	NA	-0.08428731656876	0.742352884418689	0.929911685600432	NA	NA	NA	XP_021703420.1glycine receptor subunit alpha-2
AAEL0148[illegible]	innexin inx1	-0.0842553630647001	0.48146994529052	0.846648733033901	NA	NA	NA	XP_001649754.1innexin inx1
AAEL0241[illegible]	NA	-0.0842475176824704	0.572766427899043	0.880923047085115	NA	NA	NA	XP_021705667.1uncharacterized protein LOC5578183
AAEL0255[illegible]	NA	-0.0841916085093184	0.790258068302423	0.945793949302124	NA	NA	NA	---NA---
AAEL0088[illegible]	uncharacterized LOC5571179	-0.0841797262924406	0.567380452595632	0.880302387766415	NA	NA	KXJ72955.1 hypothet	XP_021706034.1uncharacterized protein LOC5571179
AAEL0241[illegible]	NA	-0.084108613010691	0.809374010532633	0.948933370455872	NA	NA	NA	XP_021705126.1uncharacterized protein LOC5567511
AAEL0031[illegible]	uncharacterized LOC5577236	-0.0840570137801139	0.782911760061045	0.943966721184714	NA	NA	KXJ71895.1 hypothet	XP_001656342.2uncharacterized protein LOC5577236
AAEL0119[illegible]	28S ribosomal protein S31, mitochondrial	-0.0840056090221381	0.592164960358438	0.888529762455765	NA	NA	KXJ71360.1 hypothet	XP_001662069.228S ribosomal protein S31, mitochondrial
AAEL0056[illegible]	UTP--glucose-1-phosphate uridylyltransferase	-0.0839954887905685	0.529181767998098	0.866002245975191	00040 ; 00520 ; 00500 ;	aag01100 ; aag	XP_019529925.1 PRE	XP_021709649.1UTP--glucose-1-phosphate uridylyltransferase isoform X1
AAEL0076[illegible]	T-complex protein 1 subunit beta	-0.0839750739703422	0.442398328914186	0.824760338905837	NA	NA	ETN62822.1 t-compl	XP_021694661.1T-complex protein 1 subunit beta
AAEL0000[illegible]	GPI mannosyltransferase 3	-0.0839296963861876	0.612999936875027	0.895501122588404	NA	aag01100 ; aag	KXJ77539.1 hypothet	XP_001647883.2GPI mannosyltransferase 3
AAEL0202[illegible]	NA	-0.0838930072620909	0.576527477731483	0.881961491587822	NA	aag03010	NA	XP_019539684.1 60S ribosomal protein L39
AAEL0034[illegible]	sphingomyelin phosphodiesterase	-0.0838190253380164	0.514585139569643	0.861448202130317	NA	aag01100 ; aag	XP_019526047.1 PRE	XP_021710800.1sphingomyelin phosphodiesterase isoform X1
AAEL0145[illegible]	ran-binding protein 3	-0.0838040843115969	0.477856652245079	0.845011493487133	NA	NA	NA	XP_001648952.2ran-binding protein 3
AAEL0218[illegible]	NA	-0.0837236665920483	0.573182812802875	0.881060932593176	NA	NA	NA	XP_021709421.1protein pellino
AAEL0055[illegible]	IWS1-like protein	-0.0837089045795446	0.587644605504496	0.886401261871374	NA	NA	KXJ80554.1 hypothet	XP_021694540.1IWS1-like protein
AAEL0195[illegible]	NA	-0.0836257848072535	0.544979889391486	0.870466764449305	NA	NA	NA	XP_021709071.1probable phospholipid-transporting ATPase IA isoform X5
AAEL0121[illegible]	NA	-0.0835958231665799	0.717368356252004	0.921283483156443	NA	NA	KXJ71521.1 hypothet	XP_001662288.2probable N6-adenosine-methyltransferase MT-A70-like protein
AAEL0282[illegible]	NA	-0.0835897183914149	0.549634263008532	0.873333609382774	NA	aag04120	NA	XP_021693093.1kelch-like ECH-associated protein 1
AAEL0015[illegible]	tolloid-like protein 1	-0.0835476864522825	0.572100758069959	0.880923047085115	NA	NA	KXJ77965.1 hypothet	XP_001653551.1tolloid-like protein 1
AAEL0073[illegible]	soma ferritin	-0.0835417624277837	0.48874676707723	0.848895385172644	NA	NA	XP_001845699.1 ferr	XP_001652728.1soma ferritin
AAEL0141[illegible]	cytosolic Fe-S cluster assembly factor NUBP2 homolog	-0.0835245510322174	0.604942360042685	0.893547770777888	NA	NA	NA	XP_001658970.1cytosolic Fe-S cluster assembly factor NUBP2 homolog
AAEL0031[illegible]	uncharacterized LOC5577238	-0.0834808019224937	0.517628915828812	0.86196548741526	NA	NA	NA	XP_021711624.1uncharacterized protein LOC5577238 isoform X1
AAEL0008[illegible]	pre-mRNA-splicing factor cwc25	-0.0834438223088677	0.745839788706723	0.931365937538534	NA	aag03040	KXJ68904.1 hypothet	XP_001651670.2pre-mRNA-splicing factor cwc25
AAEL0052[illegible]	hepatic leukemia factor	-0.08335448923426	0.458086654149673	0.833283524364043	NA	aag04711	XP_019550111.1 PRE	XP_001650592.2hepatic leukemia factor isoform X4
AAEL0144[illegible]	ABC transporter G family member 20	-0.0833272991031916	0.649365488390275	0.906609198022654	NA	NA	NA	XP_021708064.1ABC transporter G family member 20

AAEL0024	39S ribosomal protein L27, mitochondrial	-0.0833247542164681	0.663511793461328	0.911560991852962	NA	aag03010	XP_001864516.1 mit	XP_001655229.239S ribosomal protein L27, mitochondrial
AAEL0074	NA	-0.0831317890485956	0.782805614104174	0.943962424451135	NA	aag04150	XP_001851546.1 TBC	XP_021713262.1TBC1 domain family member 7-like
AAEL0219	NA	-0.0829747332382238	0.703678113981527	0.917303584765266	NA	NA	NA	NA
AAEL0066	substance-K receptor	-0.0829696856499368	0.792870123302267	0.945793949302124	NA	NA	XP_019557995.1 PRE	XP_021707016.1substance-K receptor
AAEL0066	uncharacterized LOC5568215	-0.0828630056916935	0.698379418728473	0.916606967023218	NA	NA	XP_001849101.1 chr	XP_019553168.1 titin-like
AAEL0089	uncharacterized protein CG5902	-0.0827345712637808	0.685070322426116	0.914629465876247	NA	NA	KXJ73192.1 hypothe	XP_001845343.1AMME syndrome candidate gene 1 protein
AAEL0075	uncharacterized LOC5569299	-0.0826233140344708	0.834159605452574	0.959972072207927	NA	NA	KXJ75908.1 hypothe	XP_001658422.1uncharacterized protein LOC5569299
AAEL0135	transcription factor grauzone	-0.0826144260446621	0.665728025737685	0.911964500946328	NA	NA	NA	XP_001663746.1transcription factor grauzone
AAEL0078	serine/arginine repetitive matrix protein 1	-0.082573437094314	0.714211077386002	0.919699179136019	NA	NA	KXJ82080.1 hypothe	XP_001658699.2serine/arginine repetitive matrix protein 1
AAEL0046	uncharacterized LOC5565231	-0.0824814500842545	0.664129060348205	0.911592424421865	NA	NA	NA	XP_001649562.2uncharacterized protein LOC5565231
AAEL0114	FACT complex subunit Ssrp1	-0.0824665808858099	0.515097500432867	0.861448202130317	NA	NA	ETN63178.1 structur	XP_001655339.1FACT complex subunit Ssrp1
AAEL0013	uncharacterized LOC5569814	-0.0824003364738131	0.662147787185371	0.91036664241443	NA	NA	KXJ78770.1 hypothe	ABF18016.1secreted protein
AAEL0212	NA	-0.082347749268484	0.880847687542884	0.969743049934758	NA	NA	NA	XP_021693031.1limulus clotting factor C isoform X2
AAEL0202	NA	-0.0821677476184575	0.607882795627672	0.893547770777888	NA	NA	NA	XP_021694801.1E3 ubiquitin-protein ligase UBR3
AAEL0200	NA	-0.0819727171426211	0.505347987413228	0.858744381072989	NA	aag04341	NA	XP_019528061.1 protein roadkill isoform X3
AAEL0075	U3 small nucleolar RNA-associated protein 18 homolog	-0.0819440237132317	0.579433940804903	0.882283691803025	NA	aag03008	KXJ79024.1 hypothe	XP_001658467.1U3 small nucleolar RNA-associated protein 18 homolog
AAEL0088	uncharacterized LOC5571093	-0.0818243823078653	0.45399645689093	0.832617462606238	NA	NA	KXJ80598.1 hypothe	XP_021706131.1uncharacterized protein LOC5571093
AAEL0128	uncharacterized LOC5576917	-0.0818103402962905	0.504268002039788	0.858744381072989	NA	NA	NA	XP_001663014.2uncharacterized protein LOC5576917
AAEL0093	NA	-0.0817296832418796	0.502255113713549	0.858744381072989	NA	NA	NA	XP_021711085.1uncharacterized protein LOC5571896
AAEL0181	NA	-0.0816573221905228	0.586306554957655	0.885466509029968	NA	NA	NA	XP_021704185.1protein maelstrom homolog
AAEL0044	protein yellow	-0.0815591310072767	0.469567403692746	0.84065046403825	NA	NA	ETN62427.1 hypothe	XP_021704367.1protein yellow
AAEL0182	NA	-0.081548331442698	0.796759349030756	0.945890571556554	NA	NA	NA	XP_021699545.1BMP-binding endothelial regulator protein
AAEL0113	TBC1 domain family member 22B	-0.0815006672900108	0.661695012129701	0.909942423634082	NA	NA	KXJ70100.1 hypothe	XP_021713381.1TBC1 domain family member 22B
AAEL0096	UPF0235 protein C15orf40 homolog	-0.0813812625629999	0.8290276307908	0.957779316828242	NA	NA	NA	XP_001653923.1UPF0235 protein C15orf40 homolog
AAEL0096	estradiol 17-beta-dehydrogenase 11	-0.0813764801360353	0.480011915297835	0.845454613425911	NA	NA	NA	XP_001660285.1estradiol 17-beta-dehydrogenase 11
AAEL0229	NA	-0.0813587048839827	0.519161537279399	0.863892308507238	NA	aag02010	NA	XP_021697020.1 ATP-binding cassette sub-family B member 8, mitochondrial
AAEL0266	NA	-0.0812795767612067	0.706388190000259	0.917845823832587	NA	NA	NA	XP_021696849.1NFX1-type zinc finger-containing protein 1
AAEL0101	sodium/potassium-transporting ATPase subunit beta-2	-0.0812280248388617	0.50958192805199	0.860083070787677	NA	NA	NA	XP_011493072.1sodium/potassium-transporting ATPase subunit beta-2 isoform X1
AAEL0043	60S ribosomal protein L5	-0.0811671183224203	0.572625958507131	0.880923047085115	NA	aag03010	ETN65819.1 60S ribo	XP_001648896.160S ribosomal protein L5
AAEL0290	NA	-0.08115463745022	0.568174959351304	0.880422457819946	00790 ; 00730	NA	NA	XP_001663539.2uncharacterized protein LOC5577765
AAEL0195	NA	-0.0811071690538087	0.4702322611536	0.841189204342035	4070	aag04070	NA	XP_021696387.1inositol hexakisphosphate and diphosphoinositol-pentakisphosphate kinase isoform X8
AAEL0007	39S ribosomal protein L4, mitochondrial	-0.0810231001570431	0.646904227539961	0.906554905014882	NA	aag03010	KXJ73214.1 hypothe	XP_001650271.239S ribosomal protein L4, mitochondrial
AAEL0071	trafficking protein particle complex subunit 4	-0.0809369669004278	0.651060068154997	0.906932039402093	NA	NA	KFB49458.1 AGAP00	XP_021702147.1trafficking protein particle complex subunit 4
AAEL0002	probable cytosolic oligopeptidase A	-0.08091772815238	0.472286568438628	0.84283732647081	NA	NA	KXJ77549.1 hypothe	XP_001660130.1probable cytosolic oligopeptidase A
AAEL0173	vesicle transport through interaction with t-SNAREs homolog 1A	-0.0808982544902908	0.76826039904083	0.940222395527825	NA	aag04130	NA	XP_011493488.1vesicle transport through interaction with t-SNAREs homolog 1A
AAEL0175	putative SERF-like protein	-0.0807006669243263	0.486509575666207	0.848528684036191	NA	NA	NA	XP_011493374.1putative SERF-like protein
AAEL0062	uncharacterized LOC5567606	-0.0805894498513489	0.72598562483029	0.923177868750456	901	NA	KXJ69094.1 hypothe	XP_001657583.2uncharacterized protein LOC5567606
AAEL0133	protein lethal(2)essential for life	-0.0803680303142567	0.631620708062269	0.902506916959539	NA	aag04141 ; aag	NA	XP_001663495.1protein lethal(2)essential for life
AAEL0002	peptide deformylase, mitochondrial	-0.0802716538347654	0.701453711630327	0.916878235043446	NA	NA	KXJ78959.1 hypothe	XP_001654759.2peptide deformylase, mitochondrial
AAEL0137	transcription factor grauzone	-0.0802622766750741	0.785287936450868	0.944372632355572	NA	NA	NA	XP_001663979.1transcription factor grauzone
AAEL0238	NA	-0.0801069484883485	0.516185484371285	0.861448202130317	00970 ; 00860 ; 00970	aag01100 ; aag	NA	XP_021712876.1bifunctional glutamate/proline--tRNA ligase
AAEL0238	NA	-0.0801017304684329	0.671270469482028	0.912812708172959	NA	NA	NA	XP_021698611.1uncharacterized protein LOC5570598
AAEL0120	uncharacterized LOC5575821	-0.0800306537213064	0.784484129689086	0.944372632355572	NA	NA	KXJ76206.1 hypothe	XP_019562173.1 dual specificity protein kinase splA
AAEL0198	NA	-0.0798838863039261	0.756040885670738	0.934127170333935	NA	NA	NA	XP_021699829.1ankyrin repeat domain-containing protein SOWAHA isoform X1
AAEL0061	coiled-coil domain-containing protein 134	-0.079657952524032	0.750397477628484	0.932554085844433	NA	NA	KXJ80453.1 hypothe	XP_001657522.1coiled-coil domain-containing protein 134
AAEL0104	probable ATP-dependent RNA helicase DDX43	-0.079653791692842	0.488617617338798	0.848846834445739	NA	NA	NA	XP_021693457.1probable ATP-dependent RNA helicase DDX43
AAEL0034	uncharacterized LOC5578210	-0.0796449444666459	0.603096458997897	0.892951506950708	NA	NA	NA	ABF18223.1hemolymph protein-like protein
AAEL0273	NA	-0.079620106628299	0.688835294341112	0.916010156153545	NA	aag04140	NA	XP_021710559.1AF4/FMR2 family member 4 isoform X2
AAEL0276	NA	-0.079580006341484	0.844606582248501	0.963198678766584	NA	NA	NA	XP_021699374.1proteoglycan 4-like
AAEL0290	NA	-0.0795516720038969	0.470775305639822	0.841342855408359	00650 ; 00071 ; 00627 ;	aag01100 ; aag	NA	XP_001655993.1hydroxyacyl-coenzyme A dehydrogenase, mitochondrial isoform X1
AAEL0066	putative oxidoreductase GLYR1 homolog	-0.0794228548838367	0.588949853071868	0.88684610505111	NA	NA	KXJ80367.1 hypothe	XP_021701587.1putative oxidoreductase GLYR1 homolog isoform X1
AAEL0136	thymosin beta	-0.0793885772398206	0.479208535237115	0.845454613425911	NA	NA	NA	XP_001663844.1thymosin beta
AAEL0212	NA	-0.0793245465272039	0.538816231516414	0.869670565558805	NA	NA	NA	XP_021698892.1CKLF-like MARVEL transmembrane domain-containing protein 4
AAEL0011	fatty-acid amide hydrolase 2	-0.0793006139274288	0.608699347229479	0.893547770777888	970	NA	KFB39538.1 AGAP00	XP_001658184.1fatty-acid amide hydrolase 2
AAEL0010	transcription factor grauzone	-0.0792767765009914	0.789629846447018	0.945746953924959	NA	NA	KXJ71627.1 hypothe	XP_001657707.1transcription factor grauzone
AAEL0196	NA	-0.079255197553139	0.768565546138766	0.940470765368209	NA	aag04144 ; aag	NA	XP_021706708.1G protein-coupled receptor kinase 1
AAEL0196	NA	-0.079217464820008	0.843856217003856	0.963198678766584	NA	NA	NA	XP_021699564.1uncharacterized protein LOC23687899 isoform X1
AAEL0121	delta(24)-sterol reductase	-0.0791758867755789	0.74562372966136	0.931365937538534	100	aag01100 ; aag	XP_001842235.1 24-	XP_001655874.2delta(24)-sterol reductase
AAEL0016	probable ATP-dependent RNA helicase DDX23	-0.0791460629681583	0.619736331131139	0.897318336565571	NA	aag03040	KXJ78316.1 hypothe	XP_021701189.1probable ATP-dependent RNA helicase DDX23
AAEL0022	zinc finger protein 665	-0.0791386565186645	0.688102913126933	0.916010156153545	NA	NA	KXJ70419.1 hypothe	XP_021708028.1zinc finger protein 665
AAEL0100	MICOS complex subunit Mic60	-0.0790681800433659	0.510643780854206	0.860452247709668	NA	NA	NA	XP_001660551.2MICOS complex subunit Mic60 isoform X2
AAEL0008	probable serine/threonine-protein kinase kinX	-0.0790344537971171	0.532020673238575	0.866700945213732	NA	NA	NA	XP_021708669.1probable serine/threonine-protein kinase kinX isoform X3
AAEL0057	hormone-sensitive lipase	-0.0789429305757589	0.577977707294054	0.882161363828145	NA	NA	KXJ82042.1 hypothe	XP_021710981.1hormone-sensitive lipase
AAEL0014	transmembrane protein 47	-0.0789320957942904	0.47970462754399	0.845454613425911	NA	NA	XP_019553392.1 PRE	XP_001659307.2transmembrane protein 47 isoform X2
AAEL0122	cytoplasmic dynein 1 intermediate chain	-0.0788683115239988	0.512337641390096	0.861177338330732	NA	aag04145	XP_019563275.1 PRE	XP_019563319.1 cytoplasmic dynein 1 intermediate chain-like isoform X10
AAEL0098	39S ribosomal protein L46, mitochondrial	-0.0788100843590861	0.660304856133373	0.909580313002131	NA	NA	NA	XP_001660444.239S ribosomal protein L46, mitochondrial
AAEL0238	NA	-0.0787426745789837	0.487104073667883	0.848846834445739	04151 ; 05165 ; 04714 ;	aag04141 ; aag	NA	XP_021710632.1serine/threonine-protein kinase/endoribonuclease IRE1
AAEL0119	glutamate decarboxylase	-0.0785892552859088	0.533887871983773	0.866700945213732	NA	aag01100 ; aag	KFB52927.1 AGAP00	XP_001655801.1glutamate decarboxylase
AAEL0104	oxysterol-binding protein-related protein 8	-0.0785238621713662	0.485361580189137	0.847655581875201	NA	NA	NA	XP_021693597.1oxysterol-binding protein-related protein 8 isoform X3
AAEL0150	uncharacterized LOC5566075	-0.0784531471745554	0.746641502482336	0.931365937538534	NA	NA	NA	XP_001650439.2uncharacterized protein LOC5566075
AAEL0149	uncharacterized LOC5565630	-0.0783264657049487	0.551238309886166	0.874531710113044	NA	NA	NA	XP_001649986.2uncharacterized protein LOC5565630
AAEL0094	CCR4-NOT transcription complex subunit 7	-0.0783124658601513	0.506944337686989	0.859256178927183	NA	aag03018	NA	XP_001660002.1CCR4-NOT transcription complex subunit 7 isoform X1
AAEL0110	L-selectin	-0.078297716700743	0.682756314657947	0.914629465876247	NA	NA	KFB45679.1 AGAP01	XP_019550727.1 L-selectin
AAEL0022	palmitoyltransferase ZDHHC3	-0.0781994721400798	0.741758828544864	0.929800400527154	NA	NA	KFB38703.1 AGAP00	XP_001654991.1palmitoyltransferase ZDHHC3
AAEL0007	regucalcin	-0.0781621207142807	0.554085348293753	0.875161924991267	NA	NA	KXJ77200.1 hypothe	XP_021700697.1regucalcin isoform X1
AAEL0074	zinc finger protein 250	-0.0781406072057148	0.839427868342686	0.962903919781775	NA	NA	XP_019525770.1 PRE	XP_021699364.1zinc finger protein 250
AAEL0049	ATP-dependent helicase brm	-0.0781183984689862	0.558961173862729	0.877581080322399	NA	NA	XP_019537633.1 PRE	XP_001650089.2ATP-dependent helicase brm isoform X1
AAEL0282	NA	-0.0781085714283006	0.522492242004078	0.865018698932415	4660	NA	NA	XP_021704997.1focal adhesion kinase 1 isoform X1
AAEL0101	importin-5	-0.0780930773323468	0.50474239279697	0.858744381072989	NA	NA	NA	XP_019553123.1 importin-5-like
AAEL0130	zinc finger protein 271	-0.0780027520326615	0.70209750986966	0.916881840462067	NA	NA	NA	XP_001663296.2zinc finger protein 271 isoform X1
AAEL0095	Niemann-Pick type protein homolog 1B	-0.0779012025749252	0.796842394121539	0.945890571556554	NA	aag04142	NA	XP_001660190.2Niemannck type protein homolog 1B
AAEL0071	uncharacterized LOC5568792	-0.0778857297476206	0.650548868495649	0.906932039402093	NA	NA	XP_001870469.1 con	XP_001658157.2uncharacterized protein LOC5568792 isoform X1

AAEL01996	NA	-0.0777637063563855	0.791749597136825	0.945793949302124	NA	NA	NA	XP_021699296.1zinc finger protein 431
AAEL01961	NA	-0.0777216178861191	0.52112109169831	0.864963255015846	NA	NA	NA	XP_019549401.2 titin-like
AAEL01274	ATP synthase lipid-binding protein, mitochondrial	-0.0776772236088231	0.617610047461961	0.897318336565571	NA	aag01100 ; aag	ETN66401.1 mitocho	XP_021695120.1ATP synthase lipid-binding protein, mitochondrial
AAEL00498	RNA-binding protein 5-A	-0.077526045118396	0.573713122350263	0.881197098233729	NA	NA	KXJ83217.1 hypothet	XP_021700881.1RNA-binding protein 5-A
AAEL01194	melanotransferrin	-0.0774891852840751	0.538110760711348	0.869670565558805	NA	NA	NA	XP_021699710.1melanotransferrin isoform X2
AAEL00027	probable RNA-binding protein 18	-0.0774850566752272	0.774050723235535	0.941230906670349	NA	NA	XP_019552667.1 PRE	XP_001654782.2probable RNA-binding protein 18 isoform X1
AAEL00657	troponin C	-0.0774567953443336	0.585766765819794	0.885239904594145	NA	NA	XP_019553231.1 PRE	XP_001652068.1troponin C isoform X3
AAEL01018	uncharacterized LOC5572959	-0.0773170527756788	0.567599531858109	0.880302387766415	NA	NA	NA	XP_001654288.2uncharacterized protein LOC5572959 isoform X2
AAEL00104	mucin-6	-0.077149178496764	0.620086800928974	0.897385728224103	NA	NA	NA	XP_001657867.2mucin-6 isoform X2
AAEL00769	60S ribosomal protein L9	-0.0770728329208878	0.606341168403405	0.893547770777888	NA	aag03010	AAV84245.1 ribosom	XP_001652827.160S ribosomal protein L9
AAEL00736	8-oxo-dGDP phosphatase NUDT18	-0.0770295488276247	0.692847454459106	0.916122517126697	NA	NA	ETN59927.1 Nudt18	XP_001658319.18-oxo-dGDP phosphatase NUDT18
AAEL00860	uncharacterized LOC5570833	-0.0769160127409731	0.677317319309352	0.914481546552978	NA	NA	ETN64902.1 hypothe	XP_001653341.1uncharacterized protein LOC5570833
AAEL02419	NA	-0.076734655219075	0.480143147042576	0.845454613425911	NA	NA	NA	XP_021697761.1MOG interacting and ectopic P-granules protein 1 isoform X1
AAEL02802	NA	-0.0767340298866929	0.52693282577448	0.865973876319045	NA	NA	NA	XP_021694803.1uncharacterized protein LOC5564091 isoform X1
AAEL01970	NA	-0.0767302175678739	0.50252516208677	0.858744381072989	NA	aag03015 ; aag	NA	XP_021713312.1protein phosphatase PP2A 55 kDa regulatory subunit isoform X1
AAEL01048	protein THEM6	-0.0764763324197749	0.614805432895916	0.896845306410123	NA	NA	NA	XP_021693612.1protein THEM6
AAEL00199	NA	-0.0764619019722756	0.498965645355349	0.856939505143695	NA	NA	NA	XP_021703213.1uncharacterized protein LOC5573115 isoform X3
AAEL00060	putative uncharacterized protein DDB_G0282129	-0.076415601207411	0.608951596550333	0.893547770777888	NA	NA	NA	XP_021700939.1putative uncharacterized protein DDB_G0282129
AAEL02217	NA	-0.0763103844463729	0.728639015562182	0.923566968708516	NA	NA	NA	XP_021694198.1homeotic protein antennapedia isoform X2
AAEL01171	ARL14 effector protein	-0.0762481441493362	0.853559885797843	0.964653958545747	NA	NA	ETN65519.1 hypothe	XP_001655655.1ARL14 effector protein
AAEL00892	myosin regulatory light chain sqh	-0.0761984439486752	0.575701247230054	0.88173468538301	NA	NA	XP_003437136.1 AG	XP_001653523.2myosin regulatory light chain sqh isoform X1
AAEL02180	NA	-0.076168977663898	0.835452352754482	0.961219373599242	NA	NA	NA	XP_021697346.1uncharacterized protein LOC5576646
AAEL00759	serine protease snake	-0.0760585068381865	0.479384376471686	0.845454613425911	NA	NA	XP_019542007.1 PRE	XP_001658478.3serine protease snake isoform X1
AAEL01471	pyrimidine-specific ribonucleoside hydrolase RihA	-0.0760238092621155	0.692827333560931	0.916122517126697	NA	NA	NA	AAR18422.1salivary purine nucleosidase
AAEL02024	NA	-0.075889932866305	0.617370389890651	0.897318336565571	NA	NA	NA	XP_001658237.2protein phosphatase 1 regulatory subunit 21
AAEL00241	LIM and senescent cell antigen-like-containing domain protein 1	-0.0758884399727057	0.700081000579772	0.916629842461068	NA	NA	XP_019554031.1 PRE	XP_021709512.1LIM and senescent cell antigen-like-containing domain protein 1 isoform X1
AAEL00169	serine protease SP24D	-0.0758353772874012	0.861585257612142	0.96610329632302	NA	NA	XP_019539612.1 PRE	XP_001659970.2serine protease SP24D
AAEL01177	actin-interacting protein 1	-0.0757833016866653	0.572512153852159	0.880923047085115	NA	NA	ETN60327.1 wd-repe	XP_001661907.1actin-interacting protein 1
AAEL00597	uncharacterized LOC5579875	-0.0756187049203488	0.628720933056726	0.901045385683245	NA	NA	NA	XP_021695533.1uncharacterized protein LOC5579875 isoform X2
AAEL00312	neuroligin-4, X-linked	-0.0755225841498933	0.637970016416759	0.903725383477066	NA	NA	KXJ81478.1 hypothet	XP_001656324.2neuroligin-4, X-linked isoform X1
AAEL00149	laminin subunit alpha-1	-0.0754682407030433	0.761227007029051	0.936472047952049	NA	aag04512	KFB44910.1 laminin	XP_021700675.1laminin subunit alpha-1 isoform X3
AAEL00316	adenylosuccinate synthetase	-0.0752986976283539	0.509421881867972	0.860083070787677	00230 ; 00250	aag01100 ; aag	XP_001845298.1 ade	XP_001656488.1adenylosuccinate synthetase
AAEL02550	NA	-0.075269314017239	0.722275587833684	0.921934742632737	NA	aag04120	NA	XP_021694075.1cell division cycle protein 16 homolog
AAEL00210	sulfide:quinone oxidoreductase, mitochondrial	-0.0752552334969974	0.564516759061862	0.880156424413293	NA	aag01100 ; aag	KXJ80400.1 hypothet	XP_001654715.2sulfide:quinone oxidoreductase, mitochondrial
AAEL02334	NA	-0.074975325432019	0.522476454250826	0.865018698932415	NA	NA	NA	XP_021705248.1putative leucine-rich repeat-containing protein DDB_G0290503
AAEL01261	uncharacterized LOC5576551	-0.0748080949543049	0.728239335532027	0.923566968708516	NA	NA	NA	XP_021697853.1uncharacterized protein LOC5576551
AAEL00960	protein lingerer	-0.0747527700047775	0.541480449585277	0.869735874782513	NA	NA	NA	XP_021698801.1protein lingerer isoform X1
AAEL01231	vitellogenin-1	-0.0747220492646217	0.519496572032829	0.864080221844267	NA	NA	KXJ68117.1 hypothet	XP_019534645.1 vitellogenin-1-like
AAEL01212	bone morphogenetic protein receptor type-1B	-0.0746680005028414	0.644582969605598	0.906554905014882	NA	NA	XP_019533279.1 PRE	XP_021702939.1bone morphogenetic protein receptor type-1B isoform X1
AAEL01377	F-actin-capping protein subunit alpha	-0.0746142337836621	0.524093792531049	0.865215478807089	NA	aag04144	NA	XP_001657019.1F-actin-capping protein subunit alpha
AAEL00889	uncharacterized LOC5571211	-0.074603487842216	0.730522556075797	0.924658674141607	NA	NA	KXJ75484.1 hypothet	XP_021708731.1uncharacterized protein LOC5571211
AAEL01956	NA	-0.0745928634720641	0.512987651617879	0.861297436611866	00030 ; 00680 ; 00710 ;	NA	NA	XP_021695290.1vacuolar protein sorting-associated protein 41 homolog
AAEL01719	probable 60S ribosomal protein L37-A	-0.0745278888355783	0.611233471778091	0.894646314231935	NA	aag03010	NA	XP_011493218.1probable 60S ribosomal protein L37-A
AAEL00739	uncharacterized LOC5569132	-0.0744118177236992	0.649050130842301	0.906581557985861	NA	NA	KXJ81144.1 hypothet	XP_019556612.1 orcokinin peptides type B-like
AAEL00009	serine protease easter	-0.0743922326566256	0.534280106692341	0.866700945213732	NA	NA	KXJ68341.1 hypothet	XP_001647864.2serine protease easter
AAEL00753	carnitine O-palmitoyltransferase 2, mitochondrial	-0.0743045471083538	0.642922018608235	0.905972350539329	NA	aag01212 ; aag	KXJ74976.1 hypothet	XP_021701616.1carnitine O-palmitoyltransferase 2, mitochondrial
AAEL00908	rho GTPase-activating protein 68F	-0.0742618093206388	0.502293281337425	0.858744381072989	NA	NA	KFB40302.1 rho/rac/	XP_021695479.1rho GTPase-activating protein 68F
AAEL01403	uncharacterized LOC5579199	-0.0742302924371519	0.594327274855939	0.888690215018581	NA	NA	NA	KFB40169.1AGAP000669-like protein
AAEL01326	uncharacterized LOC5577555	-0.0742120600409056	0.6942876239256	0.916122517126697	NA	NA	NA	XP_019549496.1 beta-1,4-galactosyltransferase galt-1-like
AAEL01948	NA	-0.074131724942119	0.522014476410209	0.865018698932415	NA	NA	NA	XP_011493090.2protein tamozhennic
AAEL02686	NA	-0.0741116470777641	0.665264291796133	0.911964500946328	NA	NA	NA	XP_021713135.1nucleolin-like isoform X1
AAEL00990	PDZ and LIM domain protein 7	-0.0738831743953677	0.548740794331961	0.873169431463929	NA	NA	NA	XP_021713064.1PDZ and LIM domain protein 7 isoform X2
AAEL01994	NA	-0.0738404611508048	0.73014993334658	0.924483711082029	NA	NA	NA	XP_001655775.1squamous cell carcinoma antigen recognized by T-cells 3
AAEL00287	NAD(P)H-hydrate epimerase	-0.0736396862931603	0.726214499052982	0.923177868750456	NA	NA	Q17GW6.2 RecName	XP_021693309.1NAD(P)H-hydrate epimerase isoform X2
AAEL00540	C-terminal-binding protein	-0.0735275606660314	0.495762160197129	0.854555831227354	NA	aag04310 ; aag	NA	XP_001650859.1C-terminal-binding protein isoform X7
AAEL00187	COP9 signalosome complex subunit 4	-0.0735168099111192	0.698984610959775	0.916629842461068	NA	NA	KFB46632.1 AGAP00	XP_001654142.1COP9 signalosome complex subunit 4
AAEL00391	glycine-rich RNA-binding protein GRP2A	-0.0734821226947142	0.61938895115055	0.897318336565571	NA	NA	KXJ73364.1 hypothet	XP_001648003.1glycine-rich RNA-binding protein GRP2A
AAEL02526	NA	-0.0733622521927622	0.735047363234733	0.926618579210021	NA	NA	NA	XP_021697629.1uncharacterized protein LOC110675914
AAEL00642	uncharacterized LOC5567953	-0.0733122134465922	0.703490820580201	0.917303584765266	NA	NA	AAV90663.1 short D7	AAL16050.1short form D7Cclu23 salivary protein
AAEL00486	1,2-dihydroxy-3-keto-5-methylthiopentene dioxygenase	-0.0732709105771968	0.594261052752686	0.888690215018581	270	aag01100 ; aag	KXJ70011.1 hypothet	XP_001649899.11,2-dihydroxy-3-keto-5-methylthiopentene dioxygenase
AAEL01425	protein-glutamate O-methyltransferase	-0.0732060853810481	0.624756676479618	0.898996686480879	NA	NA	NA	XP_021704243.1protein-glutamate O-methyltransferase
AAEL02122	NA	-0.0731542040646776	0.786988052772402	0.944549897171207	NA	NA	NA	NA
AAEL01011	MIP18 family protein CG30152	-0.0731098738636268	0.670442452094526	0.912812708172959	NA	NA	NA	XP_001654239.2MIP18 family protein CG30152
AAEL01024	uncharacterized LOC5580037	-0.0730913312388407	0.796439977201964	0.945890571556554	NA	NA	NA	XP_001660685.1uncharacterized protein LOC5580037
AAEL01000	uncharacterized LOC5572728	-0.073031250802343	0.676492102950535	0.914438668430353	NA	NA	NA	XP_019931226.1 golgin-45-like
AAEL00346	H/ACA ribonucleoprotein complex subunit 2-like protein	-0.072971901410387	0.534153455386823	0.866700945213732	NA	aag03008	ETN63893.1 nucleola	XP_001656828.1H/ACA ribonucleoprotein complex subunit 2-like protein
AAEL00664	pantothenate kinase 4	-0.0729698545772629	0.660226222635645	0.909580313002131	770	aag01100 ; aag	ETN63140.1 pantoth	XP_021705142.1pantothenate kinase 4 isoform X1
AAEL00044	OTU domain-containing protein 5-B	-0.0729638081892844	0.57569060052397	0.88173468538301	NA	NA	KXJ68886.1 hypothet	XP_001656442.1OTU domain-containing protein 5-B isoform X2
AAEL01060	1,4-alpha-glucan-branching enzyme	-0.0729177455895804	0.527702567709602	0.865973876319045	500	aag01100 ; aag	KFB41349.1 AGAP01	XP_001660945.11,4-alpha-glucan-branching enzyme
AAEL00594	NADH dehydrogenase [ubiquinone] 1 subunit C2	-0.0729063212467894	0.574087260931858	0.881197098233729	NA	aag01100 ; aag	KFB41137.1 AGAP00	XP_001651717.1NADH dehydrogenase [ubiquinone] 1 subunit C2
AAEL00582	katanin p80 WD40 repeat-containing subunit B1	-0.0728370355051347	0.747251359354288	0.931735367043111	NA	NA	KXJ74081.1 hypothet	XP_021701568.1katanin p80 WD40 repeat-containing subunit B1
AAEL00985	steroidogenic acute regulatory protein-like	-0.0728080739461356	0.584954881823315	0.885175928718656	NA	NA	NA	XP_021698705.1steroidogenic acute regulatory protein-like isoform X1
AAEL02108	NA	-0.0725547236798736	0.622705317716104	0.898271200863489	NA	aag03010	NA	XP_021705595.140S ribosomal protein S25
AAEL01199	uncharacterized LOC5575681	-0.0724025824985455	0.568192594046574	0.880422457819946	NA	aag01100 ; aag	XP_001842502.1 mit	ABF18142.1mitochondrial NADH dehydrogenase (ubiquinone) Fe-S protein
AAEL00283	40S ribosomal protein S26	-0.0723007858116816	0.625261554693987	0.8994304871493	NA	aag03010	ETN62301.1 40S ribo	XP_001655992.140S ribosomal protein S26
AAEL01332	protein takeout	-0.0722926860535566	0.846595974512068	0.963198678766584	NA	NA	NA	XP_001663470.2protein takeout
AAEL00701	sodium-dependent phosphate transporter 2	-0.0722689849030597	0.521025670811745	0.864960891721031	NA	NA	ETN58046.1 phospha	XP_001652493.1sodium-dependent phosphate transporter 2
AAEL01942	NA	-0.0722448737113255	0.648464889314495	0.906554905014882	NA	NA	NA	XP_021697861.1histone deacetylase 4 isoform X3
AAEL00943	39S ribosomal protein L10, mitochondrial	-0.072195545098052	0.591926978313599	0.888529762455765	NA	aag03010	NA	XP_001660051.239S ribosomal protein L10, mitochondrial
AAEL00610	NA	-0.0721743234916996	0.549603863418912	0.873333609382774	NA	NA	XP_019525244.1 PRE	XP_019529723.1 gelsolin
AAEL02074	NA	-0.0721358512397989	0.708349565566998	0.918037119649945	NA	NA	NA	XP_021702023.1uncharacterized protein LOC5563795
AAEL00198	zinc finger protein 391	-0.0720586992764335	0.808091778719026	0.948477110685265	NA	NA	KXJ73936.1 hypothet	XP_001660736.2zinc finger protein 391

AAEL01004	calcitonin gene-related peptide type 1 receptor	-0.0720449903305556	0.72469425847737	0.92261158916834	NA	aag04080	NA	XP_021703099.1calcitonin gene-related peptide type 1 receptor isoform X1
AAEL00315	protein SMG8	-0.071842633083325	0.719997430356615	0.921583569204186	NA	NA	KXJ68880.1 hypothet	XP_001656499.1protein SMG8
AAEL00099	uncharacterized LOC5567847	-0.0716900879780318	0.526444976351677	0.865680248818465	NA	NA	NA	XP_001657708.2uncharacterized protein LOC5567847 isoform X7
AAEL00184	ski oncogene	-0.0714637179103123	0.760091260917131	0.935871383184374	NA	NA	NA	XP_021700818.1ski oncogene isoform X2
AAEL01427	mitochondrial amidoxime-reducing component 1	-0.0714463554643103	0.634346070422862	0.902506916959539	790	NA	NA	XP_001648335.2mitochondrial amidoxime-reducing component 1 isoform X2
AAEL02445	NA	-0.0714392445897776	0.868918211607971	0.968652831735134	NA	NA	NA	CRL06716.1CLUMA_CG019625, isoform A
AAEL00466	chitooligosaccharidolytic beta-N-acetylglucosaminidase	-0.0713328890974892	0.546568691190384	0.871064270234484	00513 ; 00603 ; 00604 ;	aag01100 ; aag	XP_001864406.1 chit	XP_021696980.1chitooligosaccharidolytic beta-N-acetylglucosaminidase isoform X2
AAEL00124	poly(rC)-binding protein 3	-0.0711001083408126	0.74039633124493	0.928618984286445	NA	NA	NA	XP_021698537.1poly(rC)-binding protein 3 isoform X7
AAEL02135	NA	-0.0709875332689242	0.693406149597391	0.916122517126697	04660 ; 04151 ; 05165 ;	NA	NA	XP_019537705.1 activated CDC42 kinase 1
AAEL00784	protein wntless	-0.070894173395801	0.705811210291156	0.917749125552377	NA	NA	KXJ75542.1 hypothet	XP_001658688.2protein wntless
AAEL00838	kinase D-interacting substrate of 220 kDa	-0.0708408137223245	0.526918692991325	0.865973876319045	NA	NA	NA	XP_021704252.1kinase D-interacting substrate of 220 kDa isoform X6
AAEL00995	ARF GTPase-activating protein GIT2	-0.0707279597415295	0.668324802627089	0.912587154440492	NA	aag04144	NA	XP_001660508.1ARF GTPase-activating protein GIT2
AAEL01425	putative ATP-dependent RNA helicase DHX33	-0.0706899462162743	0.680895039591484	0.914629465876247	NA	NA	NA	XP_001648316.1putative ATP-dependent RNA helicase DHX33
AAEL00423	uncharacterized LOC5564403	-0.0706409215654883	0.66895925169191	0.912812708172959	NA	aag04144	NA	XP_001648746.2uncharacterized protein LOC5564403 isoform X5
AAEL00216	putative hydroxypyruvate isomerase	-0.07061963231566	0.755247488624216	0.934127170333935	NA	aag01100 ; aag	KXJ71701.1 hypothet	XP_001654840.2putative hydroxypyruvate isomerase
AAEL01994	NA	-0.07046543720687	0.664979794205977	0.911808086169482	NA	NA	NA	XP_001658934.2uncharacterized protein LOC5570044 isoform X1
AAEL00091	glutactin	-0.0704444022090362	0.736390489213234	0.927293722242795	NA	NA	XP_019524869.1 PRE	XP_001651646.3glutactin isoform X1
AAEL00308	focadhesin	-0.070430061467061	0.721316606570684	0.921583569204186	NA	NA	KXJ83169.1 hypothet	XP_019554266.1 focadhesin-like isoform X1
AAEL00242	cytoplasmic polyadenylation element-binding protein 1	-0.070198388038132	0.595437773721862	0.889050526332724	NA	aag04320	XP_001851543.1 cyto	XP_021709672.1cytoplasmic polyadenylation element-binding protein 1
AAEL00832	60S ribosomal protein L24	-0.0699117925908975	0.625852330698049	0.899577186361051	NA	aag03010	ACJ74462.1 ribosoma	XP_001659139.160S ribosomal protein L24
AAEL00687	insulin-like growth factor 2 mRNA-binding protein 1	-0.0697856801515173	0.765794242086548	0.939178856256477	NA	NA	NA	XP_001652287.1insulin-like growth factor 2 mRNA-binding protein 1 isoform X5
AAEL02259	NA	-0.0696761223490499	0.818640629677649	0.952837221640997	NA	aag03420 ; aag	NA	XP_021693415.1DNA excision repair protein ERCC-1
AAEL01220	microtubule-associated protein tau	-0.0695735055722206	0.60444122016478	0.893547770777888	NA	NA	NA	XP_021708281.1microtubule-associated protein tau isoform X6
AAEL00980	low-density lipoprotein receptor-related protein 6	-0.0694143759644101	0.720908954266889	0.921583569204186	NA	aag04150 ; aag	NA	XP_021709286.1low-density lipoprotein receptor-related protein 6
AAEL00339	60S ribosomal protein L32	-0.0693800659801504	0.619187092066092	0.897318336565571	NA	aag03010	ETN60962.1 60S ribo	XP_001656734.160S ribosomal protein L32
AAEL00292	protein Mo25	-0.0692808946719131	0.537588693713007	0.869400906774532	NA	aag04150	ETN61034.1 calcium	XP_019546170.1 protein Mo25
AAEL01463	cytochrome c-type heme lyase	-0.0692069157201878	0.533003428788782	0.866700945213732	860	aag00860	NA	XP_001649157.2cytochrome c-type heme lyase
AAEL01071	gamma-aminobutyric acid receptor subunit beta-like	-0.0690384335734276	0.879745328075011	0.969652664055335	NA	aag04080	XP_019559721.1 PRE	XP_021694936.1gamma-aminobutyric acid receptor subunit beta-like
AAEL01120	aminoacylase-1B	-0.0689730616014154	0.533850437832442	0.866700945213732	220	aag01100 ; aag	KXJ75120.1 hypothet	XP_001655181.1aminoacylase-1B isoform X1
AAEL00075	ubiquitin-like modifier-activating enzyme 1	-0.068949470317875	0.539023068184697	0.869670565558805	NA	aag04120	KXJ68405.1 hypothet	XP_001650993.1 ubiquitin-like modifier-activating enzyme 1
AAEL00440	venom allergen 5	-0.0689340150837965	0.63247796915734	0.902506916959539	NA	NA	KXJ74537.1 hypothet	XP_001649072.2venom allergen 5
AAEL02509	NA	-0.0689260518686356	0.800695414838381	0.946215055875588	NA	NA	NA	XP_021697716.1uncharacterized protein LOC110675945
AAEL02803	NA	-0.0688475172058958	0.528020626115576	0.865973876319045	NA	NA	NA	XP_021702518.1trafficking kinesin-binding protein milt isoform X5
AAEL00484	actin-related protein 6	-0.0688153965844082	0.859935857337694	0.965722450478577	NA	NA	KFB48552.1 AGAP00	XP_001649905.1actin-related protein 6
AAEL00917	protein CREBRF homolog	-0.0687768849408778	0.542738984539655	0.869900911343623	NA	NA	XP_001843294.1 con	XP_001659791.1protein CREBRF homolog isoform X2
AAEL01300	uncharacterized LOC5577109	-0.0687586706506633	0.843149056713431	0.963198678766584	NA	NA	NA	XP_001663195.2uncharacterized protein LOC5577109
AAEL01942	NA	-0.0687458402871582	0.676069835545077	0.914438668430353	NA	NA	NA	XP_021696570.1rab GTPase-activating protein 1-like isoform X1
AAEL00397	proton-coupled amino acid transporter-like protein pathetic	-0.0686798382745086	0.62328424445918	0.898271200863489	NA	NA	XP_001861307.1 ami	XP_001648128.2proton-coupled amino acid transporter-like protein pathetic
AAEL00797	P protein	-0.0684997656538032	0.565541479109744	0.880302387766415	NA	NA	XP_019551327.1 PRE	XP_021703426.1P protein
AAEL00863	SAP30-binding protein	-0.0684680745984836	0.709517618813252	0.918410624324032	NA	NA	KXJ82294.1 hypothet	XP_001659382.1SAP30-binding protein
AAEL00024	ubiquinone biosynthesis protein COQ9, mitochondrial	-0.0683941029674694	0.579302436430474	0.882283691803025	NA	NA	XP_001688565.1 AGA	XP_021699957.1ubiquinone biosynthesis protein COQ9, mitochondrial isoform X3
AAEL01053	mesoderm induction early response protein 1	-0.0683866969726675	0.711521141158363	0.918935795219545	NA	NA	NA	XP_021701422.1mesoderm induction early response protein 1 isoform X1
AAEL00703	neutral ceramidase	-0.0683800914673916	0.656528935719693	0.909161665386398	600	aag01100 ; aag	KXJ78038.1 hypothet	XP_001658093.2neutral ceramidase
AAEL00941	PHD finger protein 20-like protein 1	-0.0683800425750408	0.686933168393892	0.916010156153545	NA	NA	NA	XP_021708662.1PHD finger protein 20-like protein 1
AAEL01031	caprin homolog	-0.0683562062841616	0.540555687696807	0.869735874782513	NA	NA	NA	XP_001654445.1caprin homolog
AAEL00098	protein pygopus	-0.0683374696560428	0.716475840781077	0.920811548445177	NA	NA	KXJ69392.1 hypothet	XP_001657729.1protein pygopus isoform X1
AAEL01828	NA	-0.0683243629960818	0.577550571798152	0.881961491587822	NA	NA	NA	XP_021709211.1microtubule-actin cross-linking factor 1, isoforms 1/2/3/5 isoform X20
AAEL00555	succinate--CoA ligase [GDP-forming] subunit beta, mitochondrial	-0.0683218564243305	0.557809885455011	0.877430182214874	00640 ; 00720 ; 00660 ;	aag01100 ; aag	XP_001847263.1 suc	XP_011493288.2succinate--CoA ligase [GDP-forming] subunit beta, mitochondrial
AAEL01225	uncharacterized LOC5576017	-0.0683145621400096	0.684552589219581	0.914629465876247	NA	NA	XP_001847210.1 leu	XP_001653208.1leucine-rich repeat-containing protein 23
AAEL00437	SWI/SNF-related matrix-associated actin-dependent regulator of chromat	-0.0682482692450419	0.760922461571362	0.936472047952049	NA	NA	AGL76706.1 SWI/SNF	XP_021707148.1SWI/SNF-related matrix-associated actin-dependent regulator of chromatin subfamily B member 1
AAEL00652	protein turtle	-0.068207569592328	0.676885707500142	0.914438668430353	NA	NA	XP_019532630.1 PRE	XP_021703968.1protein turtle isoform X1
AAEL00607	(E3-independent) E2 ubiquitin-conjugating enzyme UBE2O	-0.0680760094162042	0.762170734978676	0.937006693824152	NA	aag04120	KXJ69233.1 hypothet	XP_001657404.2(E3-independent) E2 ubiquitin-conjugating enzyme UBE2O
AAEL00406	protein stunted	-0.0677199491723621	0.600804785669161	0.892093596129407	NA	aag01100 ; aag	KXJ77977.1 hypothet	XP_001648383.1protein stunted
AAEL00358	40S ribosomal protein S13	-0.0675865541279183	0.653332062434207	0.907272485895905	NA	aag03010	ETN60832.1 ribosom	XP_001657049.140S ribosomal protein S13
AAEL02051	NA	-0.0675390842899113	0.70165795818039	0.916878235043446	NA	NA	NA	NA
AAEL01005	ATP-binding cassette sub-family E member 1	-0.0674646888613084	0.590259139372695	0.887668474886052	NA	NA	NA	XP_001660605.1ATP-binding cassette sub-family E member 1
AAEL02147	NA	-0.0672420446688125	0.534619977000859	0.866700945213732	NA	NA	NA	XP_021696773.1dnaJ homolog subfamily C member 2
AAEL00216	obg-like ATPase 1	-0.0672326677135434	0.591264910059088	0.888297094263466	970	NA	KFB40079.1 AGAP00	XP_001654823.1obg-like ATPase 1
AAEL01113	ATPase ASNA1 homolog	-0.067216459691535	0.595312035351937	0.889050526332724	NA	NA	KXJ74379.1 hypothet	XP_001661438.1ATPase ASNA1 homolog
AAEL00996	ATP synthase subunit e, mitochondrial	-0.0670927393816684	0.614460214868028	0.896790308836731	NA	aag01100 ; aag	NA	XP_001660515.1ATP synthase subunit e, mitochondrial
AAEL02601	NA	-0.067049040554188	0.77377561545288	0.941230906670349	NA	aag04150	NA	XP_021694382.1rapamycin-insensitive companion of mTOR
AAEL00359	uncharacterized LOC5578619	-0.0669663005826956	0.632168914144793	0.902506916959539	NA	NA	NA	XP_001657034.2uncharacterized protein LOC5578619
AAEL00428	FAS-associated factor 2	-0.0669396313223826	0.590364167170467	0.887668474886052	NA	NA	KFB45624.1 hypothe	XP_001648835.1FAS-associated factor 2
AAEL00095	uncharacterized LOC5567466	-0.0668568950137547	0.552705278749527	0.874531710113044	NA	NA	NA	XP_019539818.1PREDICTED: uncharacterized protein LOC109410752
AAEL01834	NA	-0.0665015730558539	0.594983218307631	0.888948786307326	NA	NA	NA	XP_021711424.1calcium/calmodulin-dependent protein kinase type IV isoform X3
AAEL00558	uncharacterized LOC5566694	-0.0664672840148945	0.63255980862351	0.902506916959539	NA	NA	KXJ80444.1 hypothet	XP_001651102.1uncharacterized protein LOC5566694
AAEL02281	NA	-0.0664185677691593	0.762542461322572	0.937135944078655	NA	NA	NA	XP_021709520.1protein FAM192A
AAEL02576	NA	-0.0663625365785783	0.654630000345403	0.907898306432314	NA	NA	NA	XP_021706837.1A-kinase anchor protein 9 isoform X3
AAEL00624	acid phosphatase type 7	-0.0663041100786062	0.632538605151338	0.902506916959539	NA	NA	KXJ70224.1 hypothet	XP_001651840.1acid phosphatase type 7
AAEL00485	dosage compensation regulator	-0.0662969843207517	0.567196576726365	0.880302387766415	NA	NA	KXJ82286.1 hypothet	XP_021697769.1dosage compensation regulator
AAEL00110	protein Vhl	-0.0662496225803832	0.728857131793224	0.923566968708516	NA	aag04120	KXJ73202.1 hypothet	XP_001652392.1protein Vhl
AAEL00834	adenosylhomocysteinase	-0.0662297881548823	0.584791526985113	0.885175928718656	270	aag01100 ; aag	AAC29475.1 S-adeno	ABF18417.1S-adenosyl-L-homocysteine hydrolase
AAEL01110	sodium/hydrogen exchanger 9B2	-0.0662234555321401	0.570918990474722	0.880923047085115	NA	NA	XP_019540631.1 PRE	XP_021700079.1sodium/hydrogen exchanger 9B2 isoform X1
AAEL00728	surfeit locus protein 6 homolog	-0.0660239133113672	0.674736296556573	0.914017871472631	NA	NA	XP_001237398.2 AGA	XP_001652642.1surfeit locus protein 6 homolog
AAEL00548	uncharacterized family 31 glucosidase KIAA1161	-0.0659852899958967	0.534665690422854	0.866700945213732	NA	NA	KXJ73691.1 hypothet	XP_021709164.1uncharacterized family 31 glucosidase KIAA1161 isoform X2
AAEL02306	NA	-0.0659787622876121	0.611357261441037	0.894646314231935	NA	NA	NA	XP_021695611.1ubiquitin carboxyl-terminal hydrolase 36 isoform X2
AAEL00520	golgin subfamily A member 6-like protein 22	-0.0657928980269618	0.798634602664099	0.946215055875588	NA	NA	KXJ83397.1 hypothet	XP_001650497.2golgin subfamily A member 6-like protein 22
AAEL00377	uncharacterized LOC5578977	-0.0657680931707688	0.871807229112855	0.969029518523332	NA	NA	KXJ78148.1 hypothet	XP_001664158.2uncharacterized protein LOC5578977
AAEL02297	NA	-0.065764728618024	0.543796036422409	0.870055542468027	NA	NA	NA	XP_021708815.1protein 4.1 homolog isoform X2
AAEL00646	peroxisome assembly protein 12	-0.0657308218372441	0.741952289625034	0.929911685600432	NA	aag04146	KXJ75922.1 hypothet	XP_001657838.1peroxisome assembly protein 12
AAEL00526	40S ribosomal protein S14	-0.0656739886639853	0.642714502535688	0.905972350539329	NA	aag03010	CRK97780.1 CLUMA_	XP_001650616.140S ribosomal protein S14
AAEL01992	NA	-0.0655795134502877	0.643627843981856	0.906297934312942	NA	aag01100 ; aag	NA	XP_001652228.1atrial natriuretic peptide receptor 1

AAEL00568	probable glutamate--tRNA ligase, mitochondrial	-0.0655374204893683	0.746325371654886	0.931365937538534	00970 ; 00860	aag01100 ; aag	KXJ69212.1 hypothet	XP_001651352.1probable glutamate--tRNA ligase, mitochondrial
AAEL00846	AP-3 complex subunit delta	-0.0654327602271749	0.671001230962183	0.912812708172959	NA	aag04142	KXJ73568.1 hypothet	XP_001659245.1AP-3 complex subunit delta
AAEL01870	NA	-0.0652776012525925	0.561349720147885	0.878642647525231	NA	NA	NA	XP_019549463.1 serine/arginine repetitive matrix protein 1-like
AAEL01371	trypsin 5G1-like	-0.0652165822887397	0.593079212223371	0.888529762455765	NA	aag04144	NA	XP_021707950.1beta-arrestin-1 isoform X1
AAEL01995	NA	-0.0652076194714393	0.657361584605218	0.909356780146906	NA	NA	NA	XP_021708003.1 solute carrier family 26 member 10
AAEL00692	vacuolar protein sorting-associated protein 37A	-0.065160983081886	0.807486577527882	0.948190794639012	NA	aag04144	KXJ69188.1 hypothet	XP_001652330.2vacuolar protein sorting-associated protein 37A
AAEL02351	NA	-0.06513011609611	0.786529032657094	0.944549897171207	NA	NA	NA	XP_001651557.2activator of 90 kDa heat shock protein ATPase homolog 1
AAEL00149	acylphosphatase-2	-0.0650527994373573	0.678526902337049	0.914481546552978	00620 ; 00627	aag01100 ; aag	CRK94385.1 CLUMA_	XP_019564776.1 acylphosphatase-2
AAEL00056	ragulator complex protein LAMTOR1	-0.065024797800721	0.684443879278359	0.914629465876247	NA	aag04150	ETN67883.1 hypothe	XP_001647964.1ragulator complex protein LAMTOR1
AAEL00942	serine/threonine-protein kinase RIO3	-0.064973391165727	0.640512677078859	0.904464102888003	04151 ; 05165 ; 04714 ;	NA	NA	XP_001660046.2serine/threonine-protein kinase RIO3
AAEL00045	uncharacterized LOC5577355	-0.0649187316986894	0.574630755855309	0.881197098233729	NA	NA	ETN58673.1 hypothe	XP_001656432.1uncharacterized protein LOC5577355
AAEL02711	NA	-0.0648304736450219	0.738251697304595	0.928033958695205	NA	aag03020	NA	XP_021710745.1DNA-directed RNA polymerase III subunit RPC1
AAEL00841	mitochondrial import inner membrane translocase subunit TIM44	-0.0647141968647798	0.68204731784092	0.914629465876247	NA	NA	XP_001868783.1 mit	XP_001653263.1mitochondrial import inner membrane translocase subunit TIM44
AAEL00237	coiled-coil domain-containing protein 43	-0.0646305491570885	0.631891377908832	0.902506916959539	NA	NA	ETN60628.1 NBP2b p	XP_001655165.1coiled-coil domain-containing protein 43
AAEL01119	39S ribosomal protein L38, mitochondrial	-0.0645216390167414	0.66573251843915	0.911964500946328	NA	NA	KXJ80040.1 hypothet	XP_001655173.139S ribosomal protein L38, mitochondrial
AAEL02025	NA	-0.0643980645682299	0.725696383662601	0.923177868750456	NA	NA	NA	CRK89809.1CLUMA_CG003479, isoform A
AAEL01148	RNA polymerase-associated protein CTR9 homolog	-0.0643886066781339	0.714780227958224	0.920089841424905	NA	NA	KFB35409.1 AGAP00	XP_001655398.1RNA polymerase-associated protein CTR9 homolog
AAEL00485	BTB/POZ domain-containing protein 10	-0.0641601830351666	0.705315033835084	0.917749125552377	NA	NA	KXJ70013.1 hypothet	XP_021697747.1BTB/POZ domain-containing protein 10 isoform X1
AAEL02132	NA	-0.0641000190259125	0.564924866160423	0.880302387766415	NA	NA	NA	XP_021697849.1nucleosome-remodeling factor subunit NURF301
AAEL00076	oligodendrocyte-myelin glycoprotein	-0.0640886949182112	0.844602859570324	0.963198678766584	NA	NA	KXJ77298.1 hypothet	XP_021705011.1oligodendrocyte-myelin glycoprotein
AAEL00769	F-box/WD repeat-containing protein 5	-0.0640660900955552	0.553211158940234	0.874531710113044	NA	NA	NA	XP_021700478.1F-box/WD repeat-containing protein 5 isoform X3
AAEL01363	zinc finger with UFM1-specific peptidase domain protein	-0.0640356595954765	0.734893580417159	0.926618579210021	NA	NA	NA	XP_021701539.1zinc finger with UFM1-specific peptidase domain protein
AAEL02621	NA	-0.0640329258131352	0.851634188980164	0.964536838405106	NA	NA	NA	XP_021710661.1neuralized-like protein 4
AAEL02678	NA	-0.063850663856523	0.829649486244412	0.958094785081062	NA	NA	NA	XP_021711287.1uncharacterized protein LOC110679832
AAEL00557	GTPase Era, mitochondrial	-0.0638432252184974	0.71775025798181	0.92129137591695	NA	NA	KXJ83719.1 hypothet	XP_001651093.2GTPase Era, mitochondrial
AAEL00441	serine-rich adhesin for platelets	-0.0638223957342178	0.752972449192313	0.932927491243795	NA	NA	XP_001849805.1 pho	XP_019565607.1 probable tyrosine-protein phosphatase cdc-14 isoform X2
AAEL00171	T-complex protein 1 subunit epsilon	-0.0638085194695576	0.572360119602948	0.880923047085115	NA	NA	KXJ80468.1 hypothet	XP_021703581.1T-complex protein 1 subunit epsilon
AAEL00729	putative fatty acyl-CoA reductase CG8306	-0.0637878752174086	0.720722470185439	0.921583569204186	73	aag04146	CRK97583.1 CLUMA_	XP_001652661.1putative fatty acyl-CoA reductase CG8306
AAEL00976	ethanolamine kinase	-0.0637681349233346	0.677838510619544	0.914481546552978	NA	aag01100 ; aag	NA	XP_001660334.1ethanolamine kinase
AAEL00206	mitochondrial-processing peptidase subunit alpha	-0.0637470971005655	0.575050398699506	0.88120129372737	NA	NA	ETN59954.1 mitocho	XP_001654562.1mitochondrial-processing peptidase subunit alpha
AAEL01056	tyrosine--tRNA ligase, mitochondrial	-0.0637162575991254	0.82527504291673	0.955377826472606	970	aag00970	KXJ71001.1 hypothet	XP_021703231.1tyrosine--tRNA ligase, mitochondrial
AAEL00106	uncharacterized LOC5568338	-0.0636988799565626	0.859808622609573	0.965722450478577	NA	NA	XP_001865314.1 con	XP_021710740.1uncharacterized protein LOC5568338
AAEL02434	NA	-0.063615131188028	0.717863590311378	0.921308334455412	NA	NA	NA	XP_021699952.1F-actin-uncapping protein LRRC16A isoform X7
AAEL01221	aminopeptidase N	-0.063613728393554	0.682716886436415	0.914629465876247	NA	aag01100 ; aag	KFB46929.1 AGAP00	XP_001662326.1aminopeptidase N
AAEL00794	eukaryotic translation initiation factor 3 subunit H	-0.063541426729236	0.628540807805168	0.901045385683245	NA	aag03013	XP_001846678.1 euk	XP_001658759.1eukaryotic translation initiation factor 3 subunit H
AAEL01802	NA	-0.0634854799984184	0.707148901343997	0.91797784850989	NA	NA	NA	XP_021700941.1uncharacterized protein LOC5564837 isoform X1
AAEL00966	histone-lysine N-methyltransferase ash1	-0.0633161695469002	0.670636235767656	0.912812708172959	310	aag01100 ; aag	NA	XP_021707226.1histone-lysine N-methyltransferase ash1 isoform X1
AAEL00138	palmitoyltransferase app	-0.0632766622295307	0.593544061053639	0.888529762455765	NA	aag04391	XP_003435719.1 AG/	XP_021699465.1palmitoyltransferase app isoform X1
AAEL01255	NADH-ubiquinone oxidoreductase 75 kDa subunit, mitochondrial	-0.0630336555696185	0.633165080219456	0.902506916959539	NA	aag01100 ; aag	KXJ75707.1 hypothet	XP_001662693.2NADH-ubiquinone oxidoreductase 75 kDa subunit, mitochondrial
AAEL02114	NA	-0.0629461722800915	0.588172799933342	0.886401261871374	NA	NA	NA	ABF18368.1reverse transcriptase-like protein
AAEL01224	uncharacterized LOC5576016	-0.0629430673083846	0.71927333843484	0.921583569204186	NA	NA	KXJ77458.1 hypothet	XP_021705482.1uncharacterized protein LOC5576016
AAEL00254	zinc finger C2HC domain-containing protein 1C	-0.0629259099491353	0.710354598756664	0.918935795219545	NA	NA	NA	XP_021713403.1zinc finger C2HC domain-containing protein 1C isoform X3
AAEL01158	60S ribosomal protein L27	-0.0628157895460428	0.63640833756796	0.902624772241117	NA	aag03010	ACF72873.1 60S ribo	XP_001661768.160S ribosomal protein L27
AAEL00214	NFU1 iron-sulfur cluster scaffold homolog, mitochondrial	-0.0627954606416731	0.639753123333449	0.904449291387452	NA	NA	KFB41827.1 AGAP00	XP_001654807.1NFU1 iron-sulfur cluster scaffold homolog, mitochondrial
AAEL00732	protein held out wings	-0.0627729379981419	0.593277515988017	0.888529762455765	NA	NA	XP_019539292.1 PRE	XP_021701852.1protein held out wings
AAEL01173	general odorant-binding protein 99a	-0.0626730560497315	0.737612978523075	0.927599209713406	NA	NA	KXJ77350.1 hypothet	XP_001661864.2general odorant-binding protein 99a
AAEL00124	zinc finger protein 706	-0.0626719515075324	0.594292004769208	0.888690215018581	NA	NA	XP_001843968.1 con	XP_001652874.1zinc finger protein 706
AAEL00453	uncharacterized LOC5564978	-0.0626615487399033	0.579467167291664	0.882283691803025	NA	NA	ETN62478.1 hypothe	KFB39321.1AGAP009181-like protein
AAEL00143	post-GPI attachment to proteins factor 2-like	-0.0626381709546678	0.734504657203778	0.926618579210021	NA	NA	KXJ72134.1 hypothet	XP_021694950.1post-GPI attachment to proteins factor 2-like isoform X1
AAEL00597	transmembrane protein 131 homolog	-0.0625184629425453	0.623084959600169	0.898271200863489	NA	NA	KXJ70860.1 hypothet	XP_001651750.1transmembrane protein 131 homolog
AAEL00513	tetraspanin-11	-0.0624107803456447	0.749189432005519	0.932554085844433	NA	NA	KXJ72547.1 hypothet	XP_019540209.1 tetraspanin-11-like
AAEL01232	protein preli-like	-0.0624087352668246	0.688415540807741	0.916010156153545	NA	NA	KXJ80311.1 hypothet	XP_001662433.1protein preli-like
AAEL00626	ras-related protein Rab-3	-0.0623736420129734	0.664930109302135	0.911808086169482	NA	NA	XP_001853751.1 GTF	XP_001651875.1ras-related protein Rab-3 isoform X2
AAEL00978	NA	-0.062340983776666	0.784190205279345	0.944372632355572	NA	NA	NA	XP_001660343.2inner centromere protein
AAEL00599	probable fatty acid-binding protein	-0.0623208215225503	0.5856871264466	0.885239904594145	NA	NA	KFB48504.1 AGAP01	XP_001657349.1probable fatty acid-binding protein
AAEL01110	U4/U6.U5 small nuclear ribonucleoprotein 27 kDa protein	-0.0623162489804844	0.656528666546455	0.909161665386398	NA	aag03040	ETN63138.1 U4/U6.U	XP_001661409.1U4/U6.U5 small nuclear ribonucleoprotein 27 kDa protein
AAEL01232	calmodulin	-0.0622774567012364	0.588112495298394	0.886401261871374	NA	aag04070 ; aag	ACT88125.1 AT1514:	APA33948.1seminal fluid protein
AAEL01127	uridine-cytidine kinase-like 1	-0.0622600145081022	0.587016981968079	0.885965272768618	00983 ; 00240	aag01100 ; aag	KXJ75061.1 hypothet	XP_001661551.2uridine-cytidine kinase-like 1
AAEL00762	nuclear factor NF-kappa-B p110 subunit	-0.0622047096135906	0.607911160697098	0.893547770777888	NA	aag04624 ; aag	XP_019536711.1 PRE	XP_001658517.2nuclear factor NF-kappa-B p110 subunit isoform X2
AAEL00397	desumoylating isopeptidase 2	-0.0621967809130669	0.620711829001915	0.897385728224103	NA	NA	KFB50205.1 AGAP01	XP_001648110.2desumoylating isopeptidase 2 isoform X2
AAEL00762	gram-negative bacteria-binding protein 1	-0.0621740297899302	0.792231925254102	0.945793949302124	NA	NA	KXJ76737.1 hypothet	XP_001658512.3gram-negative bacteria-binding protein 1
AAEL01449	xanthine dehydrogenase	-0.0621109874715786	0.779552347250482	0.943566949556407	230	NA	NA	XP_001648863.2xanthine dehydrogenase
AAEL01458	60S acidic ribosomal protein P2	-0.0621090386194954	0.687309782779918	0.916010156153545	NA	aag03010	NA	XP_001649058.160S acidic ribosomal protein P2
AAEL01958	NA	-0.0620319669042601	0.59424929894061	0.888690215018581	NA	NA	NA	XP_021695026.1neurobeachin isoform X1
AAEL01031	uncharacterized LOC5573228	-0.0618370700331819	0.723301298915951	0.921934742632737	NA	NA	NA	XP_001654433.1uncharacterized protein LOC5573228
AAEL01967	NA	-0.0617565262141061	0.564868778581295	0.880302387766415	NA	aag04068 ; aag	NA	XP_021708375.1forkhead box protein O isoform X2
AAEL00819	40S ribosomal protein S3	-0.061636975763681	0.667776037997837	0.912418872165226	NA	aag03010	XP_001864493.1 40S	XP_001659022.140S ribosomal protein S3
AAEL00816	40S ribosomal protein S12, mitochondrial	-0.0615498170900126	0.827252623484824	0.956432333168643	NA	aag03010	ETN58401.1 30S ribo	XP_001658980.240S ribosomal protein S12, mitochondrial
AAEL01120	protein ABHD16A	-0.0615241135259657	0.621745717756298	0.89816390813084	NA	NA	KXJ84009.1 hypothet	XP_001655194.1protein ABHD16A isoform X2
AAEL00103	putative elongator complex protein 1	-0.061509032847867	0.695441137727767	0.916122517126697	NA	NA	KXJ68558.1 hypothet	XP_001657869.2putative elongator complex protein 1 isoform X1
AAEL00591	uncharacterized LOC5567273	-0.0614361899530357	0.705241577538772	0.917749125552377	NA	NA	XP_001851400.1 mo	XP_001851400.1monocarboxylate transporter
AAEL02723	NA	-0.0613619194432872	0.793001520800263	0.945793949302124	NA	NA	NA	XP_021693814.1transcription factor grauzone
AAEL00267	matrix metalloproteinase-19	-0.0613523255392948	0.733250532684084	0.925884845335185	NA	NA	KXJ73280.1 hypothet	XP_021703488.1matrix metalloproteinase-19
AAEL00102	regucalcin	-0.0612445131483835	0.60148208235687	0.892140251748536	NA	NA	NA	XP_021711598.1regucalcin isoform X3
AAEL00756	dual oxidase	-0.0612201695290473	0.749735988683067	0.932554085844433	NA	aag04013 ; aag	XP_001844503.1 dua	XP_021700455.1dual oxidase isoform X1
AAEL00916	S-phase kinase-associated protein 1	-0.0611265020521785	0.572413281202269	0.880923047085115	NA	aag04141 ; aag	ETN58923.1 skp1 [Ar	XP_001653700.1S-phase kinase-associated protein 1
AAEL00505	protein cereblon homolog	-0.0610324480882499	0.740647598616837	0.92869177533756	NA	NA	ETN62811.1 hypothe	XP_001650321.1protein cereblon homolog
AAEL00954	pyruvate dehydrogenase phosphatase regulatory subunit, mitochondrial	-0.0609522826506602	0.627950528235927	0.901045385683245	00670 ; 00260	NA	NA	XP_021704323.1pyruvate dehydrogenase phosphatase regulatory subunit, mitochondrial isoform X1
AAEL01721	DNA-directed RNA polymerase II subunit RPB2	-0.0607990926513507	0.583982936327262	0.885175928718656	NA	aag03020	NA	XP_011493096.1DNA-directed RNA polymerase II subunit RPB2
AAEL00854	kinesin heavy chain	-0.0607809414990068	0.587393498768685	0.886388156062551	NA	aag04144	XP_019539434.1 PRE	XP_021693683.1kinesin heavy chain isoform X2
AAEL00707	eukaryotic translation initiation factor 3 subunit A	-0.0607270301471762	0.676671804193285	0.914438668430353	NA	aag03013	KXJ70460.1 hypothet	XP_001658105.1eukaryotic translation initiation factor 3 subunit A
AAEL00375	nucleoplasmin-like protein	-0.0607026944971528	0.645738999214738	0.906554905014882	NA	NA	ETN60353.1 Nucleop	XP_001657213.1nucleoplasmin-like protein

AAEL00356	protein eva-1	-0.0606234002968327	0.861029491047879	0.96610329632302	NA	NA	KXJ72111.1 hypothet	XP_021704510.1protein eva-1
AAEL01940	NA	-0.0603263061948967	0.705583477684822	0.917749125552377	NA	aag03040 ; aag	NA	XP_021707697.1heat shock 70 kDa protein cognate 4
AAEL00910	mitochondrial 2-oxodicarboxylate carrier	-0.0602460252611716	0.801025300047723	0.946215055875588	NA	NA	ETN63783.1 mitocho	XP_001659718.2mitochondrial 2-oxodicarboxylate carrier
AAEL02098	NA	-0.0601769415791572	0.845819491207711	0.963198678766584	NA	NA	NA	NA
AAEL00684	DCN1-like protein 4	-0.0601498584062276	0.571748738857644	0.880923047085115	NA	NA	ETN60321.1 hypothe	XP_001652247.1DCN1-like protein 4 isoform X1
AAEL01024	rRNA 2'-O-methyltransferase fibrillarin	-0.0601073648107099	0.636005371147356	0.902538767814767	NA	aag03008	NA	XP_001654398.2rRNA 2'-O-methyltransferase fibrillarin
AAEL00479	sorting nexin-13	-0.0600600403157563	0.749438991167275	0.932554085844433	NA	NA	XP_019532839.1 PRE	XP_001649816.2sorting nexin-13 isoform X1
AAEL00393	uncharacterized LOC5563773	-0.0600225337512995	0.626062290232175	0.899738434283378	NA	NA	KXJ74511.1 hypothet	ABF18510.1putative 16.8 kDa salivary secreted protein
AAEL00080	dipeptidase 1	-0.0600014466952197	0.660381599840346	0.909580313002131	NA	NA	KFB37703.1 AGAP01	XP_001651295.1dipeptidase 1
AAEL00485	ADP,ATP carrier protein 1	-0.0598826333248457	0.670630991186661	0.912812708172959	NA	NA	Q27238.2 RecName:	XP_001649911.1ADP,ATP carrier protein 1
AAEL01119	protein phosphatase 1 regulatory subunit 3A	-0.0597752331380931	0.600695482823026	0.892093596129407	NA	NA	KXJ69696.1 hypothet	XP_001661490.1protein phosphatase 1 regulatory subunit 3A
AAEL00672	2-oxoglutarate dehydrogenase, mitochondrial	-0.0597260206224329	0.665522523442588	0.911964500946328	00020 ; 00310 ; 00380	aag01100 ; aag	XP_019557672.1 PRE	XP_001652168.12-oxoglutarate dehydrogenase, mitochondrial isoform X6
AAEL01417	uncharacterized LOC5563841	-0.059702360900813	0.716226953251301	0.920748871726526	NA	NA	NA	XP_001648157.1uncharacterized protein LOC5563841
AAEL01413	abl interactor 2	-0.0596231310710729	0.707227701162684	0.91797784850989	NA	NA	NA	XP_001648084.1abl interactor 2
AAEL01500	60S ribosomal protein L23	-0.059586986982586	0.734722782610679	0.926618579210021	NA	aag03010	NA	XP_001663765.160S ribosomal protein L23
AAEL00314	NA	-0.0594893797459643	0.845028682020944	0.963198678766584	NA	NA	KXJ80175.1 hypothet	XP_001656347.1bestrophin-4 isoform X2
AAEL01469	uncharacterized LOC5565020	-0.0594673991656438	0.702158914337773	0.916881840462067	NA	NA	NA	XP_001649360.1uncharacterized protein LOC5565020
AAEL00771	ubiquitin thioesterase otubain-like	-0.0594401183811341	0.677963459084569	0.914481546552978	NA	NA	KFB39861.1 AGAP00	XP_021704011.1ubiquitin thioesterase otubain-like
AAEL01214	small subunit processome component 20 homolog	-0.0594196461005803	0.680764456540641	0.914629465876247	NA	NA	XP_001850659.1 con	XP_021700127.1small subunit processome component 20 homolog
AAEL01205	oxysterol-binding protein-related protein 2	-0.0593462205830021	0.694340894088375	0.916122517126697	NA	NA	XP_019536421.1 PRE	XP_001655845.2oxysterol-binding protein-related protein 2 isoform X2
AAEL01291	leucine-rich repeat-containing protein let-4	-0.0592949040302501	0.659160882179252	0.909580313002131	NA	NA	NA	XP_001663048.2leucine-rich repeat-containing protein let-4 isoform X1
AAEL00541	equilibrative nucleoside transporter 1	-0.0592051165780444	0.636311485767259	0.902624772241117	NA	NA	XP_001847898.1 equ	XP_021693095.1equilibrative nucleoside transporter 1
AAEL00719	m-AAA protease-interacting protein 1, mitochondrial	-0.0590581956463461	0.671385978416488	0.912812708172959	NA	NA	KXJ73528.1 hypothet	XP_001658212.1m-AAA protease-interacting protein 1, mitochondrial
AAEL01221	mitochondrial import inner membrane translocase subunit Tim16	-0.0590065176266536	0.695762401772563	0.916122517126697	NA	NA	KXJ80926.1 hypothet	XP_001662322.1mitochondrial import inner membrane translocase subunit Tim16
AAEL01473	alpha-aminoadipic semialdehyde synthase, mitochondrial	-0.0589923856889205	0.634420827235799	0.902506916959539	NA	aag01100 ; aag	NA	XP_001649464.1alpha-aminoadipic semialdehyde synthase, mitochondrial
AAEL00886	probable oligoribonuclease	-0.0589256600548397	0.80376587140849	0.94649029363412	NA	aag03008	XP_019535121.1 PRE	XP_001653489.2oligoribonuclease isoform X1
AAEL00037	inositol monophosphatase 1	-0.0588734383478275	0.650860177538788	0.906932039402093	04070 ; 00562 ; 00521	aag01100 ; aag	KXJ70076.1 hypothet	XP_021697915.1inositol monophosphatase 1
AAEL00785	la-related protein 7	-0.0588212657930306	0.855011909012973	0.964980728557884	NA	NA	KXJ68225.1 hypothet	XP_001658707.2la-related protein 7
AAEL01834	NA	-0.058741800942244	0.732703378433106	0.925763626025187	NA	NA	NA	XP_021701119.1tyrosine-protein kinase receptor torso isoform X1
AAEL01207	putative glutathione-specific gamma-glutamylcyclotransferase 2	-0.0584768969885842	0.609141995047286	0.893547770777888	480	aag01100 ; aag	ETN67555.1 hypothe	XP_001662226.1putative glutathione-specific gamma-glutamylcyclotransferase 2
AAEL01010	DNA methyltransferase 1-associated protein 1	-0.0584754510755564	0.806188602445615	0.947693857908092	NA	NA	NA	XP_001654230.1DNA methyltransferase 1-associated protein 1
AAEL00341	spondin-1	-0.0584739393810546	0.596500197501677	0.889585230151692	NA	NA	KXJ81428.1 hypothet	XP_019527642.1 spondin-1-like
AAEL02192	NA	-0.0583225597661303	0.889866022831807	0.972006599130629	NA	NA	NA	---NA---
AAEL00045	probable isocitrate dehydrogenase [NAD] subunit alpha, mitochondrial	-0.0583121408326042	0.688797164836813	0.916010156153545	NA	aag01100 ; aag	XP_001656451.2 pro	XP_001656451.2probable isocitrate dehydrogenase [NAD] subunit alpha, mitochondrial isoform X1
AAEL01224	SCY1-like protein 2	-0.0582894575739221	0.799019847558596	0.946215055875588	NA	NA	KXJ70968.1 hypothet	XP_001662347.2SCY1-like protein 2
AAEL00416	bone morphogenetic protein receptor type-2	-0.0582025633105839	0.837316805994915	0.962281659643463	NA	aag04350	KXJ73300.1 hypothet	XP_021698350.1bone morphogenetic protein receptor type-2
AAEL00187	protein-lysine N-methyltransferase CG9154	-0.0581316031831025	0.781318184646491	0.943962424451135	NA	NA	KXJ70368.1 hypothet	XP_001654135.1protein-lysine N-methyltransferase CG9154
AAEL00582	membrane alanyl aminopeptidase	-0.0581025488624667	0.670127563414384	0.912812708172959	NA	NA	XP_019561868.1 PRE	XP_021712899.1membrane alanyl aminopeptidase
AAEL01376	homeobox protein vnd	-0.0580075235576397	0.758690170025369	0.935439571457873	NA	NA	NA	XP_001663964.2homeobox protein vnd isoform X1
AAEL00407	uncharacterized LOC5564033	-0.0579726197140483	0.652989004571224	0.907272485895905	NA	NA	KXJ77145.1 hypothet	XP_001648388.2uncharacterized protein LOC5564033
AAEL00702	peroxisomal multifunctional enzyme type 2	-0.0579665696343053	0.614863460768835	0.896845306410123	NA	aag01100 ; aag	KXJ80570.1 hypothet	XP_001652466.1peroxisomal multifunctional enzyme type 2 isoform X1
AAEL00250	protein yippee	-0.0576816991125682	0.698658239549934	0.916606967023218	NA	NA	ETN59009.1 yippee p	XP_001655467.1protein yippee
AAEL00629	methyltransferase-like protein 14 homolog	-0.0575866573144565	0.800138682425156	0.946215055875588	NA	NA	KFB49453.1 AGAP00	XP_021702143.1methyltransferase-like protein 14 homolog
AAEL01261	NAD(P) transhydrogenase, mitochondrial	-0.0574369625269683	0.61662977293764	0.897164966965308	00250 ; 00430	aag01100 ; aag	KXJ72786.1 hypothet	XP_001662741.2NAD(P) transhydrogenase, mitochondrial
AAEL00353	arf-GAP domain and FG repeat-containing protein 1	-0.0574081683873804	0.591700706978348	0.888387896109256	NA	NA	XP_019536557.1 PRE	XP_021700519.1arf-GAP domain and FG repeat-containing protein 1 isoform X4
AAEL00430	protein lunapark-B	-0.05740701478238	0.675867107250782	0.914438668430353	NA	NA	KXJ62518.1 hypothet	XP_001648847.1protein lunapark-B
AAEL00434	eukaryotic translation initiation factor 3 subunit M	-0.0573661471793347	0.61114974993317	0.894646314231935	NA	NA	KFB49817.1 AGAP01	XP_001648944.1eukaryotic translation initiation factor 3 subunit M
AAEL01991	NA	-0.0572640539902484	0.804453900427897	0.946758462778864	NA	NA	NA	XP_021698790.1potassium voltage-gated channel protein Shab isoform X3
AAEL00991	probable small nuclear ribonucleoprotein Sm D2	-0.0571947192105536	0.7259338817208	0.923177868750456	NA	aag03040	NA	XP_001654090.1probable small nuclear ribonucleoprotein Sm D2
AAEL01261	RNA-binding protein 34	-0.0571570053025814	0.671577727829479	0.912812708172959	NA	NA	KXJ72783.1 hypothet	XP_001662743.2RNA-binding protein 34
AAEL02366	NA	-0.0571285110559272	0.87589020619925	0.969652664055335	NA	NA	NA	NA
AAEL00695	peritrophin-1-like	-0.0570352622598138	0.700744791408314	0.916629842461068	NA	NA	KXJ79641.1 hypothet	XP_019533670.1 probable chitinase 3
AAEL01119	actin-5C	-0.0570016261337523	0.664910758724079	0.911808086169482	NA	aag04145 ; aag	AGR44824.1 actin-4	NP_511052.1actin 5C, isoform B
AAEL00154	2-hydroxyacylsphingosine 1-beta-galactosyltransferase	-0.0568286977460168	0.698235017971113	0.916570418740397	NA	aag01100 ; aag	ETN67850.1 glucosyl	XP_001653602.12-hydroxyacylsphingosine 1-beta-galactosyltransferase
AAEL00082	senecionine N-oxygenase	-0.0567752816591899	0.697730989461027	0.916454522859806	NA	NA	KXJ76797.1 hypothet	XP_001651279.2senecionine N-oxygenase
AAEL00345	uncharacterized LOC5578203	-0.056712793498132	0.868509013682368	0.96863134437578	NA	NA	KXJ70024.1 hypothet	XP_001656845.2uncharacterized protein LOC5578203
AAEL01086	serine protease snake	-0.0566785279754948	0.658097373313783	0.909516556517693	NA	NA	KXJ70394.1 hypothet	XP_001661117.2serine protease snake
AAEL02260	NA	-0.0566204593566911	0.684360068556401	0.914629465876247	NA	NA	NA	XP_021698076.1PDZ and LIM domain protein Zasp isoform X6
AAEL01215	ras-specific guanine nucleotide-releasing factor RalGPS2	-0.0566171679232633	0.71572832155112	0.920421919040713	NA	NA	XP_019543882.1 PRE	XP_021700117.1ras-specific guanine nucleotide-releasing factor RalGPS2 isoform X4
AAEL02513	NA	-0.0565946515133542	0.602775811680435	0.892764046206882	NA	NA	NA	XP_019552218.1 chloride intracellular channel exc-4 isoform X1
AAEL02257	NA	-0.0565192782875518	0.705525636729773	0.917749125552377	NA	NA	NA	XP_021699984.1longitudinals lacking protein, isoforms F/I/K/T-like
AAEL00573	short-chain specific acyl-CoA dehydrogenase, mitochondrial	-0.0564846784922563	0.77787699064529	0.942713560900561	NA	aag01100 ; aag	KXJ77782.1 hypothet	XP_001651395.1short-chain specific acyl-CoA dehydrogenase, mitochondrial
AAEL01992	NA	-0.0562985241662694	0.804912805047959	0.94691992227708	NA	NA	NA	XP_001652227.2atrial natriuretic peptide receptor 3
AAEL00858	uncharacterized LOC5570812	-0.0562592107455943	0.686819982833009	0.916010156153545	NA	NA	XP_551859.3 AGAP0	XP_001653319.2uncharacterized protein LOC5570812
AAEL01234	lipase 3	-0.0562089821206185	0.701912175611827	0.916881840462067	NA	aag04142 ; aag	KFB37176.1 AGAP00	XP_021694068.1lipase 3 isoform X1
AAEL00839	reactive oxygen species modulator 1	-0.0562047635272177	0.632105092157089	0.902506916959539	NA	NA	XP_001843850.1 con	XP_001653245.1reactive oxygen species modulator 1
AAEL01014	isocitrate dehydrogenase [NAD] subunit beta, mitochondrial	-0.0561170021981888	0.678782580372912	0.914481546552978	NA	aag01100 ; aag	NA	XP_001654260.2isocitrate dehydrogenase [NAD] subunit beta, mitochondrial
AAEL02192	NA	-0.0561081861018381	0.650933850723512	0.906932039402093	NA	NA	NA	XP_021710148.1organic solute transporter alpha-like protein
AAEL00588	arginyl-tRNA--protein transferase 1	-0.0559157932707175	0.815658680491854	0.952015623780574	NA	NA	XP_019536510.1 PRE	XP_001651565.1arginyl-tRNA--protein transferase 1 isoform X1
AAEL00408	aldose reductase	-0.05590291272977	0.607674153662586	0.893547770777888	NA	aag01100 ; aag	XP_019536318.1 PRE	XP_001648463.1aldose reductase isoform X2
AAEL00920	exocyst complex component 1	-0.0558938187874402	0.728779897675805	0.923566968708516	NA	NA	NA	XP_001659809.1exocyst complex component 1
AAEL02249	NA	-0.0558669700736706	0.633661787978961	0.902506916959539	NA	aag01100 ; aag	NA	XP_021695402.1V-type proton ATPase subunit C isoform X1
AAEL00008	elongin-B	-0.0558524403916526	0.617295853719112	0.897318336565571	NA	aag04120	KXJ78178.1 hypothet	XP_019565637.1 transcription elongation factor B polypeptide 2-like isoform X1
AAEL00704	proteasome subunit beta type-4	-0.0558018020074897	0.645959840343733	0.906554905014882	NA	aag03050	KXJ77927.1 hypothet	XP_001652543.1proteasome subunit beta type-4
AAEL02011	NA	-0.0557525641390062	0.688029160087612	0.916010156153545	NA	NA	NA	XP_021697753.1mediator of RNA polymerase II transcription subunit 12 isoform X2
AAEL01210	gametogenetin-binding protein 2-like	-0.0557006462008691	0.749084933351307	0.932554085844433	NA	NA	CRK96469.1 CLUMA_	XP_001655865.1gametogenetin-binding protein 2-like
AAEL02145	NA	-0.0556282943624849	0.687765425314958	0.916010156153545	NA	NA	NA	XP_021710569.1N-acetylgalactosaminyltransferase 6
AAEL00688	40S ribosomal protein S28	-0.0555828125782853	0.724299127972546	0.922491429149255	NA	aag03010	XP_001865978.1 ribo	XP_001652294.140S ribosomal protein S28
AAEL00017	eukaryotic translation initiation factor 3 subunit C	-0.0555820844729988	0.625608381771585	0.899577186361051	NA	aag03013	XP_001842307.1 euk	XP_001658896.1eukaryotic translation initiation factor 3 subunit C
AAEL01501	transmembrane protein 135	-0.0555742038106246	0.671211104024703	0.912812708172959	NA	NA	NA	XP_021697267.1transmembrane protein 135
AAEL00289	putative pterin-4-alpha-carbinolamine dehydratase	-0.0554935387566094	0.687245964084191	0.916010156153545	790	aag01100 ; aag	KXJ78872.1 hypothet	XP_001662676.2putative pterin-4-alpha-carbinolamine dehydratase
AAEL00048	interleukin-1 receptor accessory protein-like 1	-0.0554614693510178	0.685754798880098	0.914803661828742	NA	NA	KXJ82406.1 hypothet	XP_021707032.1interleukin-1 receptor accessory protein-like 1

AAEL0120(	NA	-0.0554145681452255	0.855946254689735	0.964980728557884	NA	aag04120	XP_001853971.1 pep	XP_001662138.2peptidyl-prolyl cis-trans isomerase-like 2
AAEL0047	phosphatidylcholine:ceramide cholinephosphotransferase 1	-0.0553869072688205	0.779661148599603	0.943566949556407	NA	aag01100 ; aag	NA	XP_001649662.2phosphatidylcholine:ceramide cholinephosphotransferase 1 isoform X3
AAEL0051(	39S ribosomal protein L11, mitochondrial	-0.0549939688383782	0.717564149697987	0.92129137591695	NA	aag03010	ETN67957.1 50S ribo	XP_001650452.139S ribosomal protein L11, mitochondrial
AAEL0135	uncharacterized LOC5578154	-0.0549686484472183	0.720998464582707	0.921583569204186	NA	NA	NA	ABF18056.1salivary secreted protein
AAEL0133(	high affinity copper uptake protein 1	-0.0548187241493238	0.636004119554842	0.902538767814767	NA	NA	NA	XP_001663456.1high affinity copper uptake protein 1 isoform X1
AAEL0108	sodium- and chloride-dependent neutral and basic amino acid transporte	-0.0547619640150286	0.68367604518527	0.914629465876247	NA	NA	KXJ78248.1 hypothe	XP_021698770.1sodium- and chloride-dependent neutral and basic amino acid transporter B(0+) isoform X2
AAEL0245	NA	-0.0547301174973079	0.697135604809676	0.916312126946326	NA	aag04013	NA	XP_021695167.1proto-oncogene tyrosine-protein kinase ROS isoform X1
AAEL0052	stAR-related lipid transfer protein 7, mitochondrial	-0.0545411061103977	0.638592321294916	0.90377215326912	NA	NA	KXJ74781.1 hypothe	XP_021710916.1stAR-related lipid transfer protein 7, mitochondrial isoform X1
AAEL0118	MAU2 chromatid cohesion factor homolog	-0.0545165740488414	0.806951818900947	0.948040966581563	NA	NA	ETN66681.1 MAU2 c	XP_001661928.2MAU2 chromatid cohesion factor homolog
AAEL0240	NA	-0.0544364242750002	0.765157682847439	0.938922656029088	NA	NA	NA	XP_001659709.2protein IWS1 homolog
AAEL0062	solute carrier family 2, facilitated glucose transporter member 1	-0.0543989150923445	0.816461291555276	0.952319314767154	NA	NA	XP_019537838.1 PRE	XP_021699789.1solute carrier family 2, facilitated glucose transporter member 1 isoform X2
AAEL0224	NA	-0.0540809974263878	0.817846024860973	0.952514760254484	NA	NA	NA	XP_021695039.1serine/threonine-protein kinase ULK3
AAEL0196	NA	-0.0540735483240083	0.846631770214271	0.963198678766584	NA	NA	NA	XP_021698959.1uncharacterized protein LOC5571331
AAEL0067	nuclear transcription factor Y subunit alpha	-0.0540649618016921	0.661115045334745	0.909914495882133	NA	NA	XP_001855154.1 con	XP_021705817.1nuclear transcription factor Y subunit alpha
AAEL0276	NA	-0.0540128418946229	0.789097365205873	0.945355286372144	NA	aag03430	NA	XP_021700166.1DNA mismatch repair protein spellchecker 1
AAEL0030(	uncharacterized LOC5580285	-0.0539605993095604	0.833682151117533	0.959902797120062	NA	NA	NA	XP_021713093.1uncharacterized protein LOC5580285
AAEL0044	ATP-dependent RNA helicase bel	-0.0538676048654449	0.629765511232615	0.902037889184171	NA	NA	KXJ74677.1 hypothe	XP_001649212.2ATP-dependent RNA helicase bel
AAEL0095	allatotropin	-0.0536182700125988	0.790679026874233	0.945793949302124	NA	NA	NA	XP_001660202.1 allatotropin
AAEL0134	proline dehydrogenase 1, mitochondrial	-0.0536133137841188	0.695874549469398	0.916122517126697	330	aag01100 ; aag	NA	XP_021704425.1proline dehydrogenase 1, mitochondrial isoform X4
AAEL0171	kelch-like protein 18	-0.0535935146720589	0.751790177079002	0.932629041660887	NA	NA	NA	XP_021704779.1kelch-like protein 18
AAEL0047	ornithine decarboxylase antizyme-like	-0.0535330997521841	0.665989751420779	0.911964500946328	NA	NA	XP_019528340.1 PRE	XP_001649725.2 ornithine decarboxylase antizyme-like
AAEL0097	geminin	-0.0534851613115406	0.777343797037576	0.942439739175201	NA	NA	NA	XP_001654036.2geminin isoform X1
AAEL0212	NA	-0.0534368999706054	0.649795545571641	0.90663577761678	NA	NA	NA	XP_021698756.1protein takeout isoform X1
AAEL0017	CD109 antigen	-0.0534217414118855	0.627105957872214	0.900535116157945	NA	NA	XP_019550461.1 PRE	XP_021702423.1CD109 antigen
AAEL0139	LIM domain transcription factor LMO4	-0.0533944682718766	0.786714439289667	0.944549897171207	NA	NA	NA	AAK53386.1reverse transcriptase-like polymerase
AAEL0066	uroporphyrinogen decarboxylase	-0.0533936752733507	0.62770135951715	0.900968321677868	860	aag01100 ; aag	XP_019539632.1 PRE	XP_001657956.2uroporphyrinogen decarboxylase isoform X2
AAEL0090	probable uridine-cytidine kinase	-0.0533048076056206	0.626272806587091	0.899759932273913	00983 ; 00240	aag01100 ; aag	XP_019534333.1 PRE	XP_001659722.1probable uridine-cytidine kinase isoform X5
AAEL0045	bombyxin B-1 homolog	-0.053290546637571	0.679193067059501	0.914481546552978	NA	NA	NA	XP_001649391.2bombyxin B-1 homolog isoform X2
AAEL0275	NA	-0.0531426685759741	0.760020889214456	0.935871383184374	NA	NA	NA	XP_019560412.1 hyccin
AAEL0227	NA	-0.0529325718262282	0.688009510605633	0.916010156153545	NA	NA	NA	XP_021698994.1LIM and SH3 domain protein Lasp isoform X1
AAEL0060	uncharacterized LOC5567396	-0.0529250661931144	0.669967935146684	0.912812708172959	NA	NA	KXJ79708.1 hypothe	XP_013794661.1normal mucosa of esophagus-specific gene 1 protein-like
AAEL0180	NA	-0.0528539027538398	0.738725187332334	0.928033958695205	NA	NA	NA	XP_021694590.1tau-tubulin kinase homolog Asator
AAEL0004	E3 ubiquitin-protein ligase RFWD2	-0.052852878828393	0.645361594779468	0.906554905014882	480	aag01100 ; aag	KXJ71581.1 hypothe	XP_001656430.1cytosol aminopeptidase isoform X2
AAEL0064	polypeptide N-acetylgalactosaminyltransferase 3	-0.0527312822087	0.86247816675088	0.966468488016169	NA	aag01100 ; aag	KXJ71146.1 hypothe	XP_001657811.1polypeptide N-acetylgalactosaminyltransferase 3
AAEL0278	NA	-0.0526396069947514	0.862587432331572	0.966468488016169	NA	NA	NA	XP_021702410.1nucleolin 2-like
AAEL0101	putative defense protein 1	-0.0524736302630902	0.699422839918134	0.916629842461068	NA	NA	NA	XP_001654252.1putative defense protein 1
AAEL0014	lysozyme 2	-0.0524612989296479	0.740672564573174	0.92869177533756	NA	NA	KXJ80191.1 hypothe	XP_001653399.1lysozyme 2
AAEL0031(	AP-2 complex subunit mu	-0.0524331773445618	0.63401885802489	0.902506916959539	NA	aag04144	KXJ78618.1 hypothe	XP_001845524.1clathrin coat assembly protein AP50
AAEL0172	lysosomal alpha-glucosidase	-0.0523997981803309	0.64360963870936	0.906297934312942	NA	NA	NA	XP_011493504.1lysosomal alpha-glucosidase
AAEL0025	uncharacterized LOC5580180	-0.0523990814041685	0.721612608348503	0.921632240999916	NA	NA	KFB45469.1 AGAP00	KFB45469.1AGAP001637-like protein
AAEL0035	acyl-coenzyme A thioesterase 9, mitochondrial	-0.0523769374896106	0.713179047448014	0.919252089784076	NA	NA	KXJ70538.1 hypothe	XP_021706026.1acyl-coenzyme A thioesterase 9, mitochondrial isoform X2
AAEL0139	group XIIB secretory phospholipase A2-like protein	-0.0523630913869793	0.667767797503545	0.912418872165226	00564 ; 00591 ; 00565 ;	aag01100 ; aag	NA	XP_001657241.1group XIIB secretory phospholipase A2-like protein
AAEL0128	85/88 kDa calcium-independent phospholipase A2	-0.0523298818111634	0.650632027065012	0.906932039402093	NA	aag01100 ; aag	XP_019536660.1 PRE	XP_021697530.185/88 kDa calcium-independent phospholipase A2 isoform X4
AAEL0086	37 kDa salivary gland allergen Aed a 2-like	-0.0523177117047648	0.676650972999204	0.914438668430353	NA	NA	5V13_B Chain B, Mo	XP_001659374.137 kDa salivary gland allergen Aed a 2-like
AAEL0022	transcription factor grauzone	-0.052295398902366	0.793842837200201	0.945793949302124	NA	NA	KXJ67820.1 hypothe	XP_001661156.1transcription factor grauzone
AAEL0014	non-lysosomal glucosylceramidase	-0.0520956256771697	0.781724994114119	0.943962424451135	00600 ; 00511	aag01100 ; aag	KXJ79285.1 hypothe	XP_021700676.1non-lysosomal glucosylceramidase
AAEL0118(	glucose dehydrogenase [FAD, quinone]	-0.0519880805163279	0.866929578081687	0.96822706250455	NA	NA	XP_001661924.2 glu	XP_021707233.1glucose dehydrogenase [FAD, quinone] isoform X1
AAEL0042	titin homolog	-0.0519776559246511	0.865928893351377	0.967927335900485	310	NA	KXJ82122.1 hypothe	XP_021695283.1histone-lysine N-methyltransferase eggless
AAEL0053	protein PAT1 homolog 1	-0.0517582800958789	0.892085878231446	0.97252322736992	NA	aag03018	KXJ80006.1 hypothe	XP_001650812.2protein PAT1 homolog 1
AAEL0194	NA	-0.051757875853164	0.718472600025477	0.921583569204186	NA	NA	NA	XP_021703791.1ATP-binding cassette sub-family G member 4
AAEL0115	eukaryotic translation initiation factor 2 subunit 2	-0.0517346230498467	0.640772108114493	0.904492244035828	NA	aag03013	KXJ82012.1 hypothe	XP_001661734.1eukaryotic translation initiation factor 2 subunit 2 isoform X2
AAEL0135	protein KIAA0100	-0.0516979094892899	0.764249570943479	0.93832620035364	NA	NA	NA	XP_021696070.1protein KIAA0100 isoform X1
AAEL0017	ras-related and estrogen-regulated growth inhibitor	-0.0516794828619204	0.869747652206542	0.968652831735134	NA	NA	KXJ82333.1 hypothe	XP_001660263.1ras-related and estrogen-regulated growth inhibitor isoform X1
AAEL0104	nucleolin	-0.0515323731050337	0.792745527611364	0.945793949302124	NA	NA	NA	XP_019542956.1 triadin-like
AAEL0110	cytoplasmic dynein 1 light intermediate chain 1	-0.0514642095737925	0.651075098053708	0.906932039402093	NA	aag04145	KFB42059.1 AGAP00	XP_001661298.1cytoplasmic dynein 1 light intermediate chain 1
AAEL0093	uncharacterized LOC5571898	-0.0514472462500691	0.67882960192939	0.914481546552978	NA	NA	NA	XP_021705906.1uncharacterized protein LOC5571898
AAEL0058	vinculin	-0.0512267815026906	0.708085934440687	0.91797784850989	NA	NA	KFB51753.1 AGAP00	XP_019539080.1 vinculin-like
AAEL0014	MAP kinase-activating death domain protein	-0.0511296773585005	0.774668068299913	0.94130404918735	NA	NA	XP_019553410.1 PRE	XP_021700967.1MAP kinase-activating death domain protein isoform X4
AAEL0102	protein FAM98A	-0.0511156277986713	0.796152401575233	0.945890571556554	NA	NA	NA	XP_021705805.1protein FAM98A isoform X2
AAEL0109	NA	-0.0510637906934101	0.745832463414118	0.931365937538534	NA	aag03008	XP_019558804.1 PRE	XP_019558804.1 RNA exonuclease 1 homolog isoform X1
AAEL0069	uncharacterized LOC5568606	-0.0510632830806118	0.681854548507709	0.914629465876247	NA	NA	KFB39117.1 AGAP00	XP_001846492.1ubiquitin conjugating enzyme 7 interacting protein
AAEL0102	NADH dehydrogenase [ubiquinone] 1 beta subcomplex subunit 9	-0.0510487948690513	0.705813510751578	0.917749125552377	NA	aag01100 ; aag	NA	XP_001660686.1NADH dehydrogenase [ubiquinone] 1 beta subcomplex subunit 9
AAEL0072	putative fatty acyl-CoA reductase CG5065	-0.0510353175938146	0.666882717651006	0.912189849033315	73	aag04146	KXJ73387.1 hypothe	XP_001652667.1putative fatty acyl-CoA reductase CG5065
AAEL0126	uncharacterized LOC5576552	-0.0509460867709612	0.693596231881923	0.916122517126697	NA	NA	KXJ75128.1 hypothe	ABF18457.1hypothetical conserved protein
AAEL0000	60S ribosomal protein L36	-0.0509460786364324	0.723839797164435	0.922102780092934	NA	aag03010	ETN64210.1 60S ribo	XP_019534790.1 60S ribosomal protein L36-like
AAEL0093	lipoyl synthase, mitochondrial	-0.0509400124050988	0.64685215879293	0.906554905014882	785	aag01100 ; aag	NA	XP_001659989.1lipoyl synthase, mitochondrial
AAEL0029	frataxin homolog, mitochondrial	-0.0508774811607402	0.794732148328726	0.945793949302124	860	aag00860	KXJ67782.1 hypothe	XP_001656178.2frataxin homolog, mitochondrial
AAEL0069	myophilin	-0.050724383192968	0.647423567322055	0.906554905014882	NA	NA	KXJ68498.1 hypothe	XP_019563763.1 myophilin
AAEL0091	40S ribosomal protein S15Aa	-0.0506425591888956	0.737422928531358	0.927599209713406	NA	aag03010	KFB42518.1 30S ribo	XP_001653696.140S ribosomal protein S15Aa
AAEL0055	YTH domain-containing family protein 3	-0.0506210725005223	0.664509429149251	0.911808086169482	NA	NA	XP_019544774.1 PRE	XP_021707190.1YTH domain-containing family protein 3 isoform X1
AAEL0136	PHD finger protein 12	-0.0506008469025761	0.772430285175547	0.941194846952513	NA	NA	NA	XP_021705145.1 PHD finger protein 12
AAEL0217	NA	-0.0505937622508342	0.841375118736814	0.963198678766584	NA	NA	NA	XP_021710452.1E3 ubiquitin-protein ligase SH3RF1
AAEL0045(	elongation factor 2	-0.0505930051060174	0.733403025173676	0.925884845335185	NA	NA	KXJ72294.1 hypothe	XP_001649284.1elongation factor 2 isoform X1
AAEL0085	ring canal kelch homolog	-0.0505667902058259	0.712025559593005	0.919162227294442	NA	NA	Q70JS2.2 RecName:	XP_021702205.1ring canal kelch homolog isoform X1
AAEL0213	NA	-0.0505514425584625	0.794240536686883	0.945793949302124	NA	aag01100 ; aag	NA	XP_021705141.1 phosphatidylinositide phosphatase SAC2
AAEL0123	2-amino-3-ketobutyrate coenzyme A ligase, mitochondrial	-0.0504970756369518	0.795616689805784	0.945890571556554	260	aag01100 ; aag	KXJ72463.1 hypothe	XP_001662406.22-amino-3-ketobutyrate coenzyme A ligase, mitochondrial
AAEL0170	zinc finger CCCH domain-containing protein 13	-0.0504710173012488	0.732896627152868	0.925880664890264	NA	NA	NA	XP_021709004.1zinc finger CCCH domain-containing protein 13 isoform X2
AAEL0046	transmembrane protein 267	-0.0504529751580363	0.797237289332519	0.945982283238732	NA	NA	XP_019535058.1 PRE	XP_021696981.1transmembrane protein 267
AAEL0045	mpv17-like protein 2	-0.0503428229466691	0.816396532130208	0.952319314767154	NA	aag04146	ETN64189.1 sym-1 [A	XP_001649412.1mpv17-like protein 2
AAEL0082	thioredoxin-like protein 1	-0.0502672224011077	0.675224969848009	0.914410621418893	NA	NA	ETN66855.1 Thiored	XP_001659108.1thioredoxin-like protein 1
AAEL0274	NA	-0.0500834361371578	0.682438050499243	0.914629465876247	NA	NA	NA	XP_001660583.2glutamate-rich WD repeat-containing protein 1
AAEL0031(	tyrosine-protein phosphatase non-receptor type 4	-0.0500581011958375	0.708337990369052	0.918037119649945	NA	NA	KXJ78617.1 hypothe	XP_001663169.2tyrosine-protein phosphatase non-receptor type 4

AAEL0138	oxidoreductase-like domain-containing protein 1	-0.0500408961091006	0.755588737744886	0.934127170333935	NA	NA	NA	XP_001657184.1oxidoreductase-like domain-containing protein 1
AAEL0097	NA	-0.0499433366593772	0.695349602871681	0.916122517126697	NA	aag04013 ; aag	NA	XP_001660306.2ras-related protein Rac1
AAEL01401	probable cytochrome P450 4ac1	-0.0499319685491121	0.779958457972283	0.943566949556407	NA	NA	NA	XP_001664239.2probable cytochrome P450 4ac1 isoform X2
AAEL00851	sphingosine kinase 2	-0.0498678996898953	0.794148399419278	0.945793949302124	NA	aag01100 ; aag	ETN59614.1 hect E3	XP_021697372.1sphingosine kinase 2
AAEL0064	lon protease homolog, mitochondrial	-0.0498673137873035	0.708068840059767	0.91797784850989	NA	NA	XP_019527474.1 PRE	XP_001657828.1lon protease homolog, mitochondrial isoform X2
AAEL0001	phytanoyl-CoA dioxygenase domain-containing protein 1	-0.0498537030341156	0.66987427847813	0.912812708172959	NA	NA	KXJ69214.1 hypothet	XP_001657669.2 phytanoyl-CoA dioxygenase domain-containing protein 1
AAEL0054	carnitine O-palmitoyltransferase 1, liver isoform	-0.0497735193499521	0.665385507441679	0.911964500946328	71	aag01212 ; aag	XP_019538632.1 PRE	XP_001650914.1carnitine O-palmitoyltransferase 1, liver isoform isoform X1
AAEL0016	proton-coupled folate transporter	-0.0497612122013419	0.80382760755821	0.94649029363412	NA	NA	KXJ83554.1 hypothet	XP_001659953.1proton-coupled folate transporter
AAEL0062	uncharacterized LOC5567603	-0.0497395098608706	0.709187918010034	0.918371407476546	NA	NA	NA	XP_001657581.2uncharacterized protein LOC5567603
AAEL0080	probable ATP-dependent RNA helicase DHX34	-0.049738622383451	0.812306107309991	0.950268140513817	NA	NA	KXJ80602.1 hypothet	XP_021710191.1GPI mannosyltransferase 2 isoform X3
AAEL0114	dynein light chain 1, cytoplasmic	-0.0496674633176123	0.639741106934966	0.904449291387452	NA	NA	XP_015836539.1 PRE	XP_021709064.1dynein light chain 1, cytoplasmic isoform X1
AAEL0200	NA	-0.0496052960155485	0.771753302976672	0.941147080452193	NA	NA	NA	XP_019541668.1 bestrophin-2
AAEL0137	basic-leucine zipper transcription factor A	-0.0494445037403733	0.885536051042854	0.970789723835142	NA	NA	NA	XP_021697271.1basic-leucine zipper transcription factor A isoform X1
AAEL0115	proton-associated sugar transporter A	-0.0493988711266732	0.707859759815701	0.91797784850989	NA	NA	KXJ71193.1 hypothet	XP_001661720.2proton-associated sugar transporter A isoform X2
AAEL0061	putative sodium-dependent multivitamin transporter	-0.0493809362218584	0.67046064139454	0.912812708172959	NA	NA	KXJ70994.1 hypothet	XP_021710706.1putative sodium-dependent multivitamin transporter
AAEL0098	proteasome subunit alpha type-6	-0.0493378471420465	0.719920378132278	0.921583569204186	NA	aag03050	NA	XP_001654091.1proteasome subunit alpha type-6
AAEL0120	DET1- and DDB1-associated protein 1	-0.049282450739212	0.680886859514328	0.914629465876247	NA	NA	ETN61349.1 DET1- ar	XP_001662161.1DET1- and DDB1-associated protein 1
AAEL0035	FGFR1 oncogene partner 2 homolog	-0.0492776969867177	0.794965553211062	0.945793949302124	NA	NA	KXJ82565.1 hypothet	XP_001656904.2FGFR1 oncogene partner 2 homolog
AAEL0214	NA	-0.0490990883690177	0.793113352100786	0.945793949302124	04070 ; 00562	aag01100 ; aag	NA	XP_021694312.1type I inositol 3,4-bisphosphate 4-phosphatase isoform X2
AAEL0128	periodic tryptophan protein 1 homolog	-0.0490666471946888	0.670890512925	0.912812708172959	NA	NA	NA	XP_001663005.2periodic tryptophan protein 1 homolog
AAEL0032	inhibitor of nuclear factor kappa-B kinase subunit beta	-0.0490588707708123	0.785337178335698	0.944372632355572	NA	aag04150 ; aag	KXJ72408.1 hypothet	XP_001656614.2inhibitor of nuclear factor kappa-B kinase subunit beta
AAEL01251	translation initiation factor eIF-2B subunit delta	-0.0490538612543394	0.734903455385989	0.926618579210021	270	aag03013	KXJ74494.1 hypothet	XP_001656114.1translation initiation factor eIF-2B subunit delta isoform X1
AAEL00461	actin-87E	-0.0489434898905438	0.727362024286553	0.923566968708516	NA	aag04145 ; aag	ABD48797.2 beta-act	XP_019563902.1 actin-87E-like
AAEL00741	protein FAM136A	-0.0489294774046204	0.851510497549979	0.964536838405106	NA	NA	XP_001861591.1 con	XP_001658351.2protein FAM136A
AAEL0046	sorting nexin-12	-0.0487622871063343	0.657177838173819	0.909239282344833	NA	aag04144	XP_001843163.1 sor	XP_021694009.1sorting nexin-12
AAEL0054	uncharacterized LOC5566480	-0.048642319616401	0.701600954192158	0.916878235043446	NA	NA	KXJ68082.1 hypothet	XP_019560733.1 transmembrane and ubiquitin-like domain-containing protein 1
AAEL0139	RNA-binding protein lark	-0.0484565370884256	0.716837758471195	0.921019415449866	NA	NA	NA	XP_001657237.1RNA-binding protein lark
AAEL0198	NA	-0.0484416386966991	0.836447128219431	0.9615223577608	NA	aag03013 ; aag	NA	XP_021695645.1ribonuclease P protein subunit p25-like protein
AAEL0150	protein SGT1 homolog	-0.0482973155541783	0.727184399710458	0.923566968708516	NA	NA	NA	XP_001650442.1protein SGT1 homolog
AAEL0036	la protein homolog	-0.0482316092672892	0.696471347492411	0.916122517126697	NA	NA	KXJ84170.1 hypothet	XP_001657165.1la protein homolog
AAEL0125	sialin	-0.0481894197837797	0.841719160849444	0.963198678766584	NA	NA	KXJ77387.1 hypothet	XP_019535572.1 sialin-like
AAEL0045	protein rhomboid	-0.0480450938100424	0.844339465833454	0.963198678766584	NA	NA	KXJ83153.1 hypothet	XP_021703763.1protein rhomboid
AAEL00171	ATP-dependent RNA helicase DHX36	-0.0479458810635587	0.821494175093107	0.954280102887015	NA	aag03018	KXJ77478.1 hypothet	XP_001653827.1ATP-dependent RNA helicase DHX36
AAEL0138	CD63 antigen	-0.047902168869284	0.790961964476892	0.945793949302124	NA	NA	NA	XP_021697876.1CD63 antigen isoform X1
AAEL0070	NADH dehydrogenase [ubiquinone] 1 beta subcomplex subunit 3	-0.0478909382916112	0.723679067518651	0.922102780092934	NA	aag01100 ; aag	KFB49975.1 hypothe	XP_001652532.1NADH dehydrogenase [ubiquinone] 1 beta subcomplex subunit 3
AAEL0100	NA	-0.0478597440201218	0.875232602881778	0.969652664055335	NA	NA	NA	XP_001660617.1guanine nucleotide-binding protein subunit beta-5
AAEL0115	probable protein phosphatase 2C T23F11.1	-0.0478315974673322	0.697194362386309	0.916312126946326	04660 ; 04658	NA	KFB46822.1 hypothe	XP_001661751.1probable protein phosphatase 2C T23F11.1
AAEL00571	protein mini spindles	-0.0478277449421815	0.672615957593929	0.912968243848131	NA	NA	XP_019541161.1 PRE	XP_021706616.1protein mini spindles isoform X1
AAEL0103	ATP-binding cassette sub-family F member 3	-0.0476071121900058	0.693245786425914	0.916122517126697	NA	NA	NA	XP_001654470.1ATP-binding cassette sub-family F member 3
AAEL0135	zinc finger Y-chromosomal protein 1	-0.0475951356300775	0.884273388067889	0.970521528629931	NA	NA	NA	XP_001663744.2zinc finger Y-chromosomal protein 1
AAEL0237	NA	-0.0474167860712057	0.798345566224348	0.946215055875588	NA	NA	NA	XP_021695288.1A disintegrin and metalloproteinase with thrombospondin motifs 7
AAEL0087	protein DGCR14 homolog	-0.0473549875042369	0.887698728353736	0.970996238637524	NA	NA	KXJ74176.1 hypothet	XP_001659529.1protein DGCR14 homolog
AAEL0011	sodium-coupled monocarboxylate transporter 1	-0.0472156234519093	0.724463929869066	0.92257363446653	NA	NA	KXJ73721.1 hypothet	XP_001658302.1sodium-coupled monocarboxylate transporter 1
AAEL0233	NA	-0.0472063775149775	0.771127801530219	0.941147080452193	NA	NA	NA	XP_021694111.1neurochondrin homolog
AAEL0194	NA	-0.0470979225005565	0.695232147563543	0.916122517126697	04660 ; 04658	aag04310	NA	XP_021707007.1serine/threonine-protein phosphatase 2B catalytic subunit 3
AAEL0232	NA	-0.0470378155375961	0.860099318236069	0.965788154534265	NA	NA	NA	XP_021695341.1 neural-cadherin-like
AAEL0120	uncharacterized LOC5575748	-0.0468364067408134	0.782683731860425	0.943962424451135	NA	NA	NA	XP_021703199.1uncharacterized protein LOC5575748 isoform X1
AAEL01421	sister chromatid cohesion protein PDS5 homolog B-B	-0.046782609182752	0.712890970495835	0.919252089784076	NA	NA	NA	XP_001648205.1sister chromatid cohesion protein PDS5 homolog B-B
AAEL0195	NA	-0.0467573861354844	0.82220490194773	0.954452060294489	NA	NA	NA	XP_021703304.1voltage-dependent L-type calcium channel subunit beta-1 isoform X2
AAEL0143	26S proteasome non-ATPase regulatory subunit 12	-0.0467442058861535	0.706077612057807	0.917749125552377	NA	aag03050	NA	XP_001648476.126S proteasome non-ATPase regulatory subunit 12
AAEL00911	zinc finger FYVE domain-containing protein 9	-0.0465957374396919	0.723893771287911	0.922102780092934	NA	aag04144 ; aag	KXJ76108.1 hypothet	XP_021692970.1zinc finger FYVE domain-containing protein 9
AAEL0200	NA	-0.046554507345094	0.878768096615666	0.969652664055335	NA	NA	NA	XP_021703321.1ras-interacting protein RIP3 isoform X2
AAEL0150	spectrin alpha chain	-0.0464824177183324	0.683705612261746	0.914629465876247	NA	NA	NA	XP_001650465.1spectrin alpha chain isoform X4
AAEL0140	metal transporter CNNM4	-0.0464593848661307	0.750941918180588	0.932554085844433	NA	NA	NA	XP_001664301.1metal transporter CNNM4
AAEL0103	serine/arginine-rich splicing factor 2	-0.0464449102907829	0.671906505842178	0.912813925547137	NA	aag03040	NA	XP_001654458.1serine/arginine-rich splicing factor 2
AAEL0219	NA	-0.046356204806773	0.731692202711891	0.925260275963946	NA	NA	NA	NA
AAEL0071	BTB/POZ domain-containing protein kctd15	-0.046225271250022	0.894998779215653	0.972921295492314	NA	NA	KFB50521.1 AGAP00	XP_001658134.1BTB/POZ domain-containing protein kctd15
AAEL0095	NA	-0.0461987616206239	0.752691862184458	0.93283104590187	NA	NA	NA	XP_021702083.1uncharacterized protein LOC5572175 isoform X1
AAEL0023	alpha-1,3-mannosyl-glycoprotein 2-beta-N-acetylglucosaminyltransfera	-0.0461924728198387	0.791199191552038	0.945793949302124	NA	aag01100 ; aag	KXJ72813.1 hypothet	XP_001661375.1alpha-1,3-mannosyl-glycoprotein 2-beta-N-acetylglucosaminyltransferase
AAEL0195	NA	-0.0461063168970564	0.712769998084278	0.919252089784076	NA	NA	NA	XP_021704200.1furin-like protease 2
AAEL0099	60S ribosomal protein L4	-0.0459643052283779	0.749311894844513	0.932554085844433	NA	aag03010	NA	XP_001660544.160S ribosomal protein L4
AAEL0064	uncharacterized LOC5568062	-0.0459099859038458	0.881742162077364	0.97020104931674	NA	NA	NA	XP_021706333.1uncharacterized protein LOC5568062 isoform X1
AAEL0030	60S ribosomal export protein NMD3	-0.0458801472225174	0.72868201717687	0.923566968708516	NA	aag03013 ; aag	KFB48750.1 AGAP00	XP_001662996.160S ribosomal export protein NMD3
AAEL0070	protein DEK	-0.0457826546024049	0.667031239309645	0.912189849033315	NA	NA	XP_019933434.1 PRE	XP_021708350.1protein DEK isoform X2
AAEL0081	zinc finger RNA-binding protein	-0.0457223971812339	0.68756900701298	0.916010156153545	NA	NA	XP_019551786.1 PRE	XP_001659016.2zinc finger RNA-binding protein isoform X1
AAEL0120	HEAT repeat-containing protein 5B	-0.0457087602073825	0.774220627449473	0.941230906670349	NA	NA	NA	XP_021705383.1HEAT repeat-containing protein 5B isoform X1
AAEL0066	phospholipase A-2-activating protein	-0.0456992176560827	0.788990240049051	0.945335024600439	NA	aag04141	KXJ80368.1 hypothet	XP_001657931.2phospholipase A-2-activating protein
AAEL0081	40S ribosomal protein S8	-0.0455530042814083	0.751696052141683	0.932629041660887	NA	aag03010	AAV90709.1 ribosom	XP_001653121.140S ribosomal protein S8
AAEL0038	galectin-9	-0.0455382801615396	0.733349026473344	0.925884845335185	NA	NA	XP_019527089.1 PRE	XP_001657305.2galectin-9 isoform X2
AAEL0252	NA	-0.0454873838953901	0.738590222735191	0.928033958695205	NA	NA	NA	XP_021707129.1ras-related protein Rab-18A-like
AAEL0147	transmembrane protein 183	-0.0454232332328129	0.866465061665302	0.968074250389273	NA	NA	NA	XP_001649363.2transmembrane protein 183
AAEL0029	uncharacterized LOC5580309	-0.0453899341544034	0.874960625264828	0.969652664055335	NA	NA	XP_001689119.2 AG	XP_019527857.1 rho-related GTP-binding protein RhoU-like
AAEL0018	mediator of RNA polymerase II transcription subunit 17	-0.0450992019556209	0.843587209165093	0.963198678766584	NA	NA	KXJ73001.1 hypothet	XP_001654224.2mediator of RNA polymerase II transcription subunit 17
AAEL0022	mediator of RNA polymerase II transcription subunit 18	-0.0450928846839803	0.870526589339132	0.968899314929262	NA	NA	KFB40929.1 AGAP01	XP_001661240.1mediator of RNA polymerase II transcription subunit 18
AAEL0001	protein TIC 214	-0.0450919640655631	0.851510739465563	0.964536838405106	NA	NA	KXJ71830.1 hypothet	XP_019538533.1 cell wall protein DAN4-like isoform X2
AAEL0045	probable tRNA N6-adenosine threonylcarbamoyltransferase, mitochondr	-0.0450862947683718	0.838390275509575	0.962348963212321	NA	NA	XP_001862089.1 O-s	XP_001649414.2probable tRNA N6-adenosine threonylcarbamoyltransferase, mitochondrial
AAEL0055	uncharacterized LOC5566687	-0.0450585639903199	0.711322939725255	0.918935795219545	NA	aag04141	KXJ83718.1 hypothet	XP_019536217.1 X-box-binding protein 1-like
AAEL01811	NA	-0.0449905120865167	0.82387311839737	0.954821213538111	NA	NA	NA	XP_001652059.2protein FAM49B
AAEL0247	NA	-0.0449384924644519	0.81556030621713	0.952015623780574	NA	NA	NA	XP_021711785.1eukaryotic translation initiation factor 3 subunit D-like
AAEL0064	serine/arginine-rich splicing factor 1A	-0.0448789340447547	0.695120174950896	0.916122517126697	NA	aag03040	ETN66014.1 arginine	XP_001657837.1serine/arginine-rich splicing factor 1A
AAEL0096	NA	-0.0446541379682595	0.700770587015172	0.916629842461068	NA	NA	NA	XP_021708958.1uncharacterized protein LOC110679171 isoform X2
AAEL0196	NA	-0.0444511887358426	0.731906718606054	0.92526523212157	NA	NA	NA	XP_021700262.1probable cytochrome P450 9f2 isoform X1

AAEL01978	NA	-0.0444444972042446	0.838630334275114	0.962348963212321	NA	NA	NA	XP_021700441.1uncharacterized protein LOC5571949 isoform X1
AAEL00551	uncharacterized LOC5579827	-0.0444390049005268	0.8463013483636	0.963198678766584	NA	NA	KXJ72329.1 hypothet	XP_001651058.2uncharacterized protein LOC5579827
AAEL00848	calcyphosin-like protein	-0.0444360026172501	0.763681775445787	0.937987145975992	NA	NA	XP_019538032.1 PRE	XP_001659255.1calcyphosin-like protein isoform X2
AAEL00007	serine protease easter	-0.0444069391881042	0.716332467241982	0.920755882603816	NA	NA	KXJ72917.1 hypothet	XP_001647872.2serine protease easter
AAEL00523	cyclin-dependent kinases regulatory subunit	-0.0443727433757411	0.891017547146519	0.972336404299425	NA	NA	KXJ83642.1 hypothet	XP_001650539.1cyclin-dependent kinases regulatory subunit
AAEL01323	proteasome subunit beta type-5	-0.0441079431417478	0.739747079567468	0.928416888458789	NA	aag03050	NA	XP_001663403.2proteasome subunit beta type-5
AAEL00207	RING finger protein 141	-0.0440513720232783	0.801077973275278	0.946215055875588	NA	NA	KFB36805.1 AGAP00	XP_001654584.1RING finger protein 141
AAEL00095	sorting nexin-8	-0.0440104871960238	0.785679767842087	0.944372632355572	NA	NA	XP_019539822.1 PRE	XP_001657470.1sorting nexin-8 isoform X3
AAEL02448	NA	-0.0439489032142082	0.770308115770694	0.941147080452193	NA	NA	NA	XP_021697042.1uncharacterized protein LOC110675699
AAEL01197	fumarylacetoacetase	-0.0439430721317695	0.739741930438733	0.928416888458789	00643 ; 00350	aag01100 ; aag	KXJ77662.1 hypothet	XP_019543619.1 fumarylacetoacetase-like
AAEL00738	ferritin subunit	-0.0438758185345174	0.720484673822222	0.921583569204186	NA	NA	P41822.2 RecName:	XP_001652731.2ferritin subunit isoform X2
AAEL00163	protein shuttle craft	-0.0438346435220246	0.687661672131148	0.916010156153545	NA	NA	KXJ80724.1 hypothet	XP_001653734.1protein shuttle craft
AAEL01835	NA	-0.0437857199763688	0.701510279596753	0.916878235043446	NA	NA	NA	XP_021708223.1oxysterol-binding protein 1 isoform X4
AAEL02304	NA	-0.0436302640314151	0.875967216166725	0.969652664055335	NA	NA	NA	KFB40758.1AGAP001925-like protein
AAEL02214	NA	-0.0435608584900855	0.78302251428024	0.94397254618785	NA	NA	NA	NA
AAEL00176	E3 ubiquitin-protein ligase LRSAM1	-0.0434497088267543	0.768814541199179	0.940525313495725	NA	NA	KXJ71849.1 hypothet	XP_001653956.2E3 ubiquitin-protein ligase LRSAM1
AAEL02644	NA	-0.0434393030104141	0.755231958660409	0.934127170333935	NA	aag04013	NA	XP_021700055.1uncharacterized protein LOC5564083 isoform X1
AAEL00396	dehydrogenase/reductase SDR family member 7	-0.0433253166289238	0.873598442436272	0.969470919838227	NA	NA	KXJ79971.1 hypothet	XP_001648135.2dehydrogenase/reductase SDR family member 7
AAEL00747	uncharacterized aarF domain-containing protein kinase 5	-0.0431446759586249	0.847866873203491	0.963435772275933	NA	NA	KXJ79918.1 hypothet	XP_001658384.1uncharacterized aarF domain-containing protein kinase 5
AAEL01119	inactive hydroxysteroid dehydrogenase-like protein 1	-0.0430632149527347	0.782405929655225	0.943962424451135	NA	NA	XP_001850197.1 ster	XP_021701876.1inactive hydroxysteroid dehydrogenase-like protein 1
AAEL01097	ATP-binding cassette sub-family F member 2	-0.0429956872539838	0.746074745160607	0.931365937538534	NA	NA	ETN65975.1 hypothe	XP_001661220.1ATP-binding cassette sub-family F member 2
AAEL01298	pescadillo homolog	-0.0428793747657423	0.700574622147021	0.916629842461068	NA	NA	NA	XP_001656318.2pescadillo homolog
AAEL00070	glycogen phosphorylase	-0.0427634904877454	0.79237317083991	0.945793949302124	500	aag01100 ; aag	KFB39704.1 hypothe	XP_001650265.1glycogen phosphorylase
AAEL00849	uncharacterized protein C18orf8	-0.0426412130065618	0.802498114108213	0.94649029363412	NA	NA	KXJ71556.1 hypothet	XP_001659265.1uncharacterized protein C18orf8
AAEL00314	uncharacterized LOC5577225	-0.042578883245711	0.834004774617535	0.959935536312959	NA	NA	KXJ80112.1 hypothet	XP_019558256.1 zinc finger protein 84-like
AAEL02398	NA	-0.042492868424762	0.775631443896273	0.941847042163971	NA	aag03010	NA	XP_019531418.1 40S ribosomal protein S4
AAEL00120	diacylglycerol O-acyltransferase 1	-0.0424084562511738	0.699299947048794	0.916629842461068	00561 ; 00073	aag01100 ; aag	KXJ76146.1 hypothet	XP_021703032.1diacylglycerol O-acyltransferase 1
AAEL00514	liprin-beta-2	-0.0423065747840309	0.74416428352764	0.930913504217149	NA	NA	NA	XP_021711268.1liprin-beta-2 isoform X8
AAEL00694	syntaxin-binding protein 5	-0.0421664098237585	0.738937710862831	0.928033958695205	NA	NA	XP_019534132.1 PRE	XP_021707650.1syntaxin-binding protein 5 isoform X4
AAEL01698	transforming acidic coiled-coil-containing protein 1	-0.0420968062868617	0.70946216913357	0.918410624324032	NA	NA	NA	XP_021713132.1transforming acidic coiled-coil-containing protein 2 isoform X3
AAEL01047	ataxin-10	-0.042072709043245	0.76798447586771	0.940009729573645	NA	NA	NA	XP_019562355.1 ataxin-10
AAEL00314	zinc finger protein 93	-0.0418742452777239	0.839867111137136	0.963047620770582	NA	NA	KFB36177.1 AGAP00	XP_001656328.1zinc finger protein 93
AAEL01166	corepressor interacting with RBPJ 1	-0.0417725072414629	0.838041502521917	0.962348963212321	NA	aag04330	KFB48459.1 AGAP00	XP_001661842.1corepressor interacting with RBPJ 1
AAEL00871	myosin-10	-0.0416040595241032	0.766453392441091	0.939178856256477	NA	NA	KXJ72317.1 hypothet	XP_019532934.1 transcriptional regulator ATRX homolog isoform X3
AAEL01025	E3 ubiquitin-protein ligase SMURF2	-0.041562579752337	0.707640588258238	0.91797784850989	NA	aag04144 ; aag	NA	XP_021709006.1E3 ubiquitin-protein ligase SMURF2
AAEL01352	tissue inhibitor of metalloproteases	-0.0415396844391112	0.752353953876227	0.932695238282356	NA	NA	NA	XP_001663710.1tissue inhibitor of metalloproteases
AAEL00484	protein lin-7 homolog C	-0.0414346474639352	0.795013714176475	0.945793949302124	NA	NA	KFB48553.1 AGAP00	XP_001649906.1protein lin-7 homolog C
AAEL01831	NA	-0.0412743135257317	0.897166275915993	0.973750254467651	NA	NA	NA	XP_021710370.1voltage-dependent calcium channel type A subunit alpha-1 isoform X14
AAEL01080	protein SET	-0.0412478593110863	0.742734173483891	0.930112144847571	NA	NA	KFB37348.1 SET, put	XP_001655006.1protein SET
AAEL00871	uncharacterized protein CG7065	-0.0412394855485314	0.790410307937579	0.945793949302124	NA	NA	NA	XP_021710338.1uncharacterized protein CG7065 isoform X2
AAEL00064	gonadal protein gdl	-0.0411127169692319	0.871757497410498	0.969029518523332	NA	NA	XP_001847521.1 gor	XP_021710495.1gonadal protein gdl
AAEL00242	transcription factor IIIB 90 kDa subunit	-0.0410502117659373	0.88081765126647	0.969743049934758	NA	NA	XP_019554049.1 PRE	XP_001655206.2transcription factor IIIB 90 kDa subunit
AAEL02269	NA	-0.0409807332298642	0.860626498626758	0.966023193660905	NA	NA	NA	XP_021713286.1uncharacterized protein LOC5574046
AAEL02129	NA	-0.0408734274367108	0.792573675210499	0.945793949302124	NA	NA	NA	XP_021710307.1zinc finger protein Xfin
AAEL01012	carbohydrate sulfotransferase 11	-0.0408257765352357	0.880654880866273	0.969743049934758	NA	NA	NA	XP_021702733.1carbohydrate sulfotransferase 11
AAEL02801	NA	-0.0407869963668235	0.910495850892956	0.97691422813284	NA	NA	NA	NA
AAEL01341	NA	-0.0406610233881958	0.808386586546417	0.948581148865102	NA	NA	NA	XP_001663604.2uncharacterized protein LOC5577934
AAEL00056	C-type lectin 37Da	-0.0405924685531664	0.877511252601258	0.969652664055335	NA	NA	KXJ70118.1 hypothet	XP_001647954.2C-type lectin 37Da
AAEL00392	tRNA (guanine(10)-N2)-methyltransferase homolog	-0.0405639109052482	0.84714212333874	0.963198678766584	NA	NA	XP_001847378.1 con	XP_001647995.2tRNA (guanine(10)-N2)-methyltransferase homolog
AAEL00705	facilitated trehalose transporter Tret1	-0.0405358882866155	0.843036619691284	0.963198678766584	NA	NA	ETN66996.1 sugar tra	XP_001652519.1facilitated trehalose transporter Tret1
AAEL00870	protein suppressor of white apricot	-0.040530684880993	0.837652312380809	0.962348963212321	NA	NA	KXJ77860.1 hypothet	XP_001659442.2protein suppressor of white apricot
AAEL00885	RING finger protein 10	-0.0403904135227329	0.722654680291116	0.921934742632737	NA	NA	KXJ72061.1 hypothet	XP_001653450.2RING finger protein 10
AAEL00850	SRSF protein kinase 3	-0.0403523515280611	0.745202600890243	0.931365937538534	NA	NA	CRL00473.1 CLUMA_	XP_021697362.1SRSF protein kinase 3
AAEL00970	uncharacterized LOC5572322	-0.0403521323339281	0.912159402599613	0.977296280428831	NA	NA	NA	XP_001653998.1uncharacterized protein LOC5572322
AAEL00605	uncharacterized LOC5567384	-0.0403029780214765	0.867216632162845	0.96822706250455	NA	NA	XP_001848991.1 bet	XP_001651785.2uncharacterized protein LOC5567384
AAEL00501	uncharacterized LOC5565853	-0.0402609600937805	0.863526975446148	0.967038506393142	NA	NA	KXJ71835.1 hypothet	XP_001650200.1uncharacterized protein LOC5565853
AAEL00856	titin homolog	-0.0402076450176851	0.752379564634854	0.932695238282356	NA	NA	KXJ75296.1 hypothet	XP_021698592.1titin homolog isoform X1
AAEL00759	serine protease snake	-0.040128407245113	0.778289186656284	0.942840586573345	NA	NA	KXJ82933.1 hypothet	XP_001658476.2serine protease snake
AAEL00173	protein bric-a-brac 1	-0.0400946243263873	0.898284504855188	0.973750254467651	NA	NA	XP_019543238.1 PRE	XP_001660258.2protein bric-a-brac 1 isoform X4
AAEL00284	PEST proteolytic signal-containing nuclear protein	-0.0399503223646484	0.872873228480402	0.969029518523332	NA	NA	KXJ75132.1 hypothet	XP_021707215.1PEST proteolytic signal-containing nuclear protein
AAEL00934	protein l(2)37Cc	-0.0398878307215018	0.71085004907481	0.918935795219545	NA	NA	NA	XP_001653792.1protein l(2)37Cc
AAEL01223	thioredoxin domain-containing protein 17	-0.0398760751124349	0.761139867640105	0.936472047952049	NA	NA	ETN66960.1 glutared	XP_001662342.1thioredoxin domain-containing protein 17
AAEL01138	protein halfway	-0.0396308310799643	0.787688280232107	0.944774185961398	NA	NA	KXJ82525.1 hypothet	XP_021694834.1protein halfway
AAEL01235	nucleoside diphosphate kinase	-0.0395966189758428	0.790410128570887	0.945793949302124	00240 ; 00983 ; 00230	aag01100 ; aag	XP_011210175.2 PRE	XP_001662512.1nucleoside diphosphate kinase
AAEL01523	UPF0729 protein AAEL015238	-0.0395835578593142	0.73877884638915	0.928033958695205	NA	NA	NA	XP_001651159.1UPF0729 protein AAEL015238
AAEL00175	40S ribosomal protein S9	-0.0395775091678106	0.803157408994061	0.94649029363412	NA	aag03010	AAW31599.1 ribosor	XP_001653969.140S ribosomal protein S9
AAEL00834	uncharacterized LOC5570450	-0.0395370180981742	0.885547592031317	0.970789723835142	NA	NA	KXJ70132.1 hypothet	XP_001659154.1uncharacterized protein LOC5570450
AAEL01029	40S ribosomal protein S12	-0.0395293095787644	0.784604996133347	0.944372632355572	NA	aag03010	NA	XP_019556645.1 40S ribosomal protein S12
AAEL02038	NA	-0.0395215529747849	0.820500030839763	0.953915512796905	970	aag00970	NA	XP_021713032.1isoleucine--tRNA ligase, mitochondrial
AAEL00300	probable pseudouridine-5'-phosphatase	-0.0394893868619526	0.728511785812811	0.923566968708516	NA	NA	KXJ69498.1 hypothet	XP_021709148.1probable pseudouridine-5'-phosphatase isoform X2
AAEL00045	MORN repeat-containing protein 4	-0.039485048939136	0.780291063014032	0.94365072438627	NA	NA	ETN67872.1 retinoph	XP_001656412.1MORN repeat-containing protein 4
AAEL00713	dynein heavy chain, cytoplasmic	-0.0394490110897098	0.746853863627129	0.931365937538534	NA	aag04145	NA	XP_021709253.1dynein heavy chain, cytoplasmic isoform X5
AAEL02790	NA	-0.0393066136855454	0.720983071259558	0.921583569204186	NA	aag03013	NA	XP_021709839.1polyadenylate-binding protein-interacting protein 1 isoform X2
AAEL01429	40S ribosomal protein S24	-0.0392757014143717	0.777634808153584	0.94254419186371	NA	aag03010	NA	XP_001649979.140S ribosomal protein S24
AAEL00627	uncharacterized LOC5567703	-0.0391695492871301	0.868175312044693	0.96863134437578	NA	NA	KXJ80548.1 hypothet	XP_001651869.2uncharacterized protein LOC5567703
AAEL01382	calcium/calmodulin-dependent protein kinase type II alpha chain	-0.039142203448578	0.90732732441171	0.976056966800435	NA	aag04310 ; aag	NA	XP_019559689.1 calcium/calmodulin-dependent protein kinase type II alpha chain
AAEL02375	NA	-0.0391114194883824	0.84620846954705	0.963198678766584	NA	NA	NA	XP_021695130.1uncharacterized protein LOC110674873 isoform X2
AAEL01146	histidine triad nucleotide-binding protein 1	-0.0391027708580869	0.74984268871567	0.932554085844433	NA	NA	KXJ76262.1 hypothet	XP_001661688.1histidine triad nucleotide-binding protein 1
AAEL02346	NA	-0.0390678426235292	0.922968517172476	0.981343310857761	NA	NA	NA	XP_019531106.1 probable serine/threonine-protein kinase kinX
AAEL02194	NA	-0.0390571935019409	0.789520403672568	0.945738968314888	NA	NA	NA	NA
AAEL01176	phenoloxidase 2	-0.038958477040801	0.761759381297077	0.93687648044583	NA	NA	KXJ82706.1 hypothet	XP_001661890.1phenoloxidase 2
AAEL00097	transcription factor GAGA	-0.0389425002971909	0.819168273868726	0.952969210637171	NA	NA	XP_019531707.1 PRE	XP_021708534.1transcription factor GAGA isoform X1

AAEL00497	protein transport protein Sec24C	-0.0389332191787352	0.72279400569162	0.921934742632737	NA	aag04141	KXJ83215.1 hypothet	XP_001650130.2protein transport protein Sec24C
AAEL00306	NA	-0.0388629884156604	0.816219331096653	0.952319314767154	NA	NA	NA	AAK72504.1putative chymotrypsin-like protein
AAEL01716	cleavage and polyadenylation specificity factor subunit CG7185	-0.0387224634361009	0.808352351020908	0.948581148865102	NA	aag03015	NA	XP_011493066.2cleavage and polyadenylation specificity factor subunit CG7185 isoform X2
AAEL02155	NA	-0.0387223722982453	0.736026047708733	0.927161080221186	NA	NA	NA	XP_021697717.1uncharacterized protein LOC5574982 isoform X1
AAEL00605	nuclear protein localization protein 4 homolog	-0.038665387581787	0.79652825752884	0.945890571556554	NA	aag04141	XP_019528200.1 PRE	XP_021704785.1nuclear protein localization protein 4 homolog isoform X1
AAEL00490	geranylgeranyl pyrophosphate synthase	-0.0385841147506974	0.84667095425196	0.963198678766584	NA	aag01100 ; aag	ETN62676.1 quemao	XP_001650014.1geranylgeranyl pyrophosphate synthase
AAEL00063	COP9 signalosome complex subunit 7b	-0.0385614932708643	0.743341791973276	0.930390529072101	NA	NA	XP_019557026.1 PRE	XP_001649790.1COP9 signalosome complex subunit 7b isoform X2
AAEL01007	spermidine synthase-like	-0.0385518313182305	0.805874785783985	0.947456393630119	00410 ; 00270 ; 00330 ;	aag01100 ; aag	NA	XP_001660614.1spermidine synthase isoform X1
AAEL00549	ABC transporter G family member 20	-0.0382622394032914	0.830756933999155	0.958693918567873	NA	NA	XP_019540395.1 PRE	XP_001650952.2ABC transporter G family member 20 isoform X1
AAEL01203	uncharacterized LOC5575726	-0.0382301980682756	0.918990808221795	0.979901902347272	NA	NA	KXJ78342.1 hypothet	XP_001662163.1uncharacterized protein LOC5575726 isoform X1
AAEL02077	NA	-0.0381876164459087	0.795467545623083	0.945890571556554	NA	NA	NA	NA
AAEL00687	sorting nexin-17	-0.0381709308266778	0.841021339833924	0.963198678766584	NA	NA	KXJ84570.1 hypothet	XP_001652281.1sorting nexin-17
AAEL00623	uncharacterized LOC5567640	-0.0381608240918616	0.924287572879198	0.981795480276391	NA	NA	KXJ77715.1 hypothet	CAD27928.1putative G-protein coupled receptor
AAEL00882	RAC serine/threonine-protein kinase	-0.038121850796382	0.789887628037133	0.945793949302124	04151 ; 05165 ; 04714 ;	aag04150 ; aag	XP_001849257.1 rac	XP_001659505.1RAC serine/threonine-protein kinase
AAEL00152	zinc finger protein 665	-0.0381162440763719	0.786345984560329	0.944549897171207	NA	NA	NA	XP_021711477.1zinc finger protein 665 isoform X6
AAEL02789	NA	-0.0379852537779448	0.895834914147141	0.97325535773105	NA	NA	NA	XP_021693924.1transcription factor grauzone
AAEL00631	26S proteasome non-ATPase regulatory subunit 2	-0.0379410355043675	0.752004184937614	0.932629041660887	NA	aag03050	ETN59915.1 26S prot	XP_001651894.126S proteasome non-ATPase regulatory subunit 2
AAEL00201	phosphorylated adapter RNA export protein	-0.0379232799087193	0.804308859570721	0.946758462778864	NA	aag03013	KXJ74362.1 hypothet	XP_021703269.1phosphorylated adapter RNA export protein isoform X2
AAEL00638	39S ribosomal protein L39, mitochondrial	-0.0379049399651304	0.779385480525962	0.943566949556407	NA	NA	KXJ69955.1 hypothet	XP_001657756.239S ribosomal protein L39, mitochondrial
AAEL00430	39S ribosomal protein L19, mitochondrial	-0.0378512073171623	0.800540304981258	0.946215055875588	NA	aag03010	KXJ69143.1 hypothet	XP_001648902.139S ribosomal protein L19, mitochondrial
AAEL00155	mucolipin-3	-0.0378099611502919	0.782095459033584	0.943962424451135	NA	NA	KXJ77966.1 hypothet	XP_021695946.1mucolipin-3 isoform X1
AAEL00383	ryanodine receptor	-0.037734602002014	0.744509016690338	0.931218155713536	NA	NA	NA	XP_021707515.1ryanodine receptor isoform X3
AAEL00237	UPF0428 protein CG16865	-0.0377188105215872	0.857754559053501	0.964980728557884	NA	NA	XP_001841725.1 con	XP_001655133.2UPF0428 protein CG16865
AAEL00498	peroxisomal biogenesis factor 19	-0.0376848278381508	0.729628203239782	0.924035057282201	NA	aag04146	XP_001850749.1 per	XP_001650131.1peroxisomal biogenesis factor 19
AAEL01197	alpha-mannosidase 2	-0.0376617634476052	0.794848225506626	0.945793949302124	00513 ; 00510	aag01100 ; aag	KXJ72595.1 hypothet	XP_021693765.1alpha-mannosidase 2 isoform X2
AAEL01001	cytochrome b5	-0.0375163043991512	0.729425488473788	0.923905484505959	NA	NA	NA	XP_001660580.2cytochrome b5
AAEL00641	37 kDa salivary gland allergen Aed a 2-like	-0.037464346518231	0.833620174666218	0.959902797120062	NA	NA	AAV90665.1 long for	XP_001657778.137 kDa salivary gland allergen Aed a 2-like
AAEL00713	peroxiredoxin-5, mitochondrial	-0.0373155028079964	0.743804483865428	0.930716461861527	480	aag04146	KXJ73433.1 hypothet	XP_001658149.1peroxiredoxin-5, mitochondrial
AAEL00098	60S ribosomal protein L8	-0.0372688310740903	0.809086334738348	0.948933370455872	NA	aag03010	ETN58771.1 60S ribo	XP_001657711.160S ribosomal protein L8
AAEL02434	NA	-0.0372634782729965	0.850034426422298	0.96443951617769	NA	NA	NA	NA
AAEL00581	negative elongation factor A	-0.0372019314916118	0.82439754150494	0.954996283354524	NA	NA	KXJ71978.1 hypothet	XP_021712857.1negative elongation factor A
AAEL01174	eukaryotic peptide chain release factor subunit 1	-0.0371675364368491	0.751610224809225	0.932629041660887	NA	aag03015	ETN68111.1 eukaryo	XP_001661879.1eukaryotic peptide chain release factor subunit 1
AAEL00679	endoribonuclease Dicer	-0.0369989995098619	0.766986907790825	0.939413486888563	NA	NA	AEX31247.1 Dicer 2 i	XP_001652212.1endoribonuclease Dicer
AAEL00300	glutamate-gated chloride channel	-0.0369807119661119	0.824543085334894	0.954996283354524	NA	NA	XP_019548992.1 PRE	XP_001662897.2glutamate-gated chloride channel isoform X9
AAEL01202	regulator of gene activity	-0.0368997121444117	0.766364632972825	0.939178856256477	NA	aag03018	XP_019563593.1 PRE	XP_001655826.2regulator of gene activity isoform X1
AAEL00989	protein kinase C-binding protein 1	-0.0367056055977927	0.783464763226663	0.94413865259452	NA	NA	NA	XP_021708614.1protein kinase C-binding protein 1 isoform X1
AAEL01238	NA	-0.0367035749205401	0.898567209120305	0.973780616635202	NA	NA	KFB42793.1 AGAP00	XP_021706790.1 lachesin
AAEL00305	chitinase-3-like protein 1	-0.0365113748414785	0.785711461998941	0.944372632355572	520	NA	XP_001841679.1 chit	XP_001663099.2chitinase-3-like protein 1
AAEL02674	NA	-0.036488354740352	0.759076821653001	0.935439571457873	NA	NA	NA	NA
AAEL02755	NA	-0.0363913579597187	0.871959590284984	0.969029518523332	NA	NA	NA	XP_021703450.1zinc finger protein 585A
AAEL02309	NA	-0.0362420329392137	0.918571529215868	0.979722358282524	NA	NA	NA	XP_021693789.1transcription factor grauzone
AAEL00745	uncharacterized LOC5569178	-0.0362410225086187	0.74970601467263	0.932554085844433	NA	NA	NA	XP_001652757.1uncharacterized protein LOC5569178 isoform X7
AAEL00770	E3 ubiquitin-protein ligase HECTD1	-0.0361790884328287	0.739698108664234	0.928416888458789	NA	NA	KXJ76966.1 hypothet	XP_021704014.1E3 ubiquitin-protein ligase HECTD1 isoform X1
AAEL00469	RING-box protein 1A	-0.0361685459742146	0.781514128821393	0.943962424451135	NA	aag04141 ; aag	ETN65005.1 hypothe	XP_001649597.1RING-box protein 1A isoform X2
AAEL00618	solute carrier family 12 member 1	-0.0360211461882089	0.823457878102849	0.954580422562662	NA	NA	XP_019546027.1 PRE	XP_021709422.1solute carrier family 12 member 1 isoform X1
AAEL00696	endothelial lipase	-0.0359720779181121	0.788412163412932	0.945232821987723	NA	NA	XP_019561736.1 PRE	XP_011493040.1endothelial lipase isoform X2
AAEL01052	probable lysine-specific demethylase 4A	-0.0358204509326608	0.854449064888805	0.964859528176068	NA	NA	NA	XP_021696026.1probable lysine-specific demethylase 4A
AAEL00849	NADH dehydrogenase [ubiquinone] 1 beta subcomplex subunit 7	-0.0357567158630409	0.796773542411645	0.945890571556554	NA	aag01100 ; aag	KFB36547.1 AGAP00	XP_021695784.1NADH dehydrogenase [ubiquinone] 1 beta subcomplex subunit 7 isoform X2
AAEL02357	NA	-0.0356988366083087	0.879795922556768	0.969652664055335	NA	NA	NA	XP_021698055.1uncharacterized protein LOC110676020 isoform X2
AAEL00490	uncharacterized LOC5565651	-0.0356466687539867	0.772908911234024	0.941230906670349	NA	NA	KXJ70232.1 hypothet	XP_019525207.1 monocarboxylate transporter 9-like
AAEL02064	NA	-0.0356016163985536	0.907630406766221	0.976056966800435	NA	NA	NA	XP_021694824.1uncharacterized protein LOC5564702
AAEL00354	probable RNA methyltransferase bin3	-0.0353984511290626	0.833064946275111	0.959902797120062	NA	NA	XP_001842602.1 bic	XP_001656911.2probable RNA methyltransferase bin3
AAEL00920	DNA-directed RNA polymerase III subunit RPC7	-0.0353941926884983	0.876593125460597	0.969652664055335	NA	aag03020	NA	XP_001659808.1DNA-directed RNA polymerase III subunit RPC7
AAEL00643	piezo-type mechanosensitive ion channel component	-0.0353544332207768	0.770172240988009	0.941147080452193	NA	NA	NA	XP_021703544.1piezo-type mechanosensitive ion channel component isoform X4
AAEL01237	dyslexia-associated protein KIAA0319-like protein	-0.0352609873530582	0.846243035777386	0.963198678766584	NA	NA	KXJ75399.1 hypothet	XP_021701542.1dyslexia-associated protein KIAA0319-like protein
AAEL00066	MAPK regulated corepressor interacting protein 2	-0.0352122685444383	0.804825482059948	0.94691992227708	NA	NA	ETN67757.1 hypothe	XP_001649800.1MAPK regulated corepressor interacting protein 2
AAEL02497	NA	-0.0351732462286271	0.892042630245596	0.97252322736992	NA	NA	NA	XP_021700880.1poly(ADP-ribose) glycohydrolase
AAEL02493	NA	-0.0351681923389126	0.793167892736099	0.945793949302124	NA	aag04144	NA	XP_021698992.1protein disabled isoform X2
AAEL00755	SWI/SNF-related matrix-associated actin-dependent regulator of chromat	-0.0351477547300002	0.785414606431708	0.944372632355572	NA	NA	KXJ70348.1 hypothet	XP_021700450.1SWI/SNF-related matrix-associated actin-dependent reg. chromatin subfamily A containing DEAD/H box 1 homolog
AAEL01225	developmentally-regulated GTP-binding protein 2	-0.0350912198933548	0.804049652903505	0.946630617453691	970	NA	ETN57996.1 develop	XP_001662357.1developmentally-regulated GTP-binding protein 2
AAEL01993	NA	-0.034995472817511	0.751766021384274	0.932629041660887	NA	NA	NA	XP_021696385.1serine-rich adhesin for platelets isoform X6
AAEL01950	NA	-0.0349643480181684	0.827342031299978	0.956432333168643	NA	NA	NA	XP_021705259.1solute carrier family 12 member 4 isoform X5
AAEL00364	probable basic-leucine zipper transcription factor J	-0.0348554553154813	0.784875623307888	0.944372632355572	NA	NA	XP_019543026.1 PRE	XP_021709689.1probable basic-leucine zipper transcription factor J isoform X2
AAEL00019	protein FAM57A	-0.0348259804273	0.773878895393292	0.941230906670349	NA	NA	ETN59669.1 hypothe	XP_001658939.1protein FAM57A
AAEL02616	NA	-0.0346923356323372	0.864409395999478	0.967038506393142	NA	NA	NA	XP_021704044.1 E3 ubiquitin-protein ligase highwire
AAEL01977	NA	-0.034657389191925	0.821606637887323	0.954280102887015	NA	NA	NA	XP_021710379.1sterol regulatory element-binding protein cleavage-activating protein
AAEL00650	ATP synthase subunit g, mitochondrial	-0.0346226570177252	0.781610411581231	0.943962424451135	NA	aag01100 ; aag	ASC55683.1 mitocho	XP_001652015.1ATP synthase subunit g, mitochondrial
AAEL00739	mitochondrial import receptor subunit TOM20 homolog	-0.0345036643650152	0.760234473761201	0.935871383184374	NA	NA	ETN66935.1 mitocho	XP_021704184.1mitochondrial import receptor subunit TOM20 homolog
AAEL02368	NA	-0.0344673638398628	0.803315405425288	0.94649029363412	NA	NA	NA	NA
AAEL00417	microtubule-associated protein RP/EB family member 1	-0.034318717816344	0.75926952393163	0.935439571457873	NA	NA	XP_019534575.1 PRE	XP_001648566.1microtubule-associated protein RP/EB family member 1 isoform X3
AAEL00055	pacifastin-like protease inhibitor cvp4	-0.0340896179676294	0.770485726013738	0.941147080452193	NA	NA	KFB40411.1 pacifasti	XP_001647959.1pacifastin-like protease inhibitor cvp4
AAEL00111	uncharacterized serine-rich protein C215.13	-0.0340837714485703	0.882316254383971	0.970357617045106	NA	NA	KXJ80955.1 hypothet	XP_001652426.2uncharacterized serine-rich protein C215.13
AAEL02084	NA	-0.0340424675263726	0.857717013646924	0.964980728557884	NA	aag04140 ; aag	NA	XP_021702308.1serine/threonine-protein kinase TBK1
AAEL00591	programmed cell death protein 6	-0.0340421267024714	0.821007049698695	0.954106679666959	NA	NA	XP_001850177.1 sor	XP_001651590.1programmed cell death protein 6
AAEL01178	glycoprotein 3-alpha-L-fucosyltransferase A	-0.0339920163320286	0.918335992705801	0.979657756188126	NA	aag01100 ; aag	KFB35003.1 hypothe	XP_001661902.2glycoprotein 3-alpha-L-fucosyltransferase A
AAEL00982	vacuolar fusion protein CCZ1 homolog	-0.0338421216272522	0.853237662505588	0.964653958545747	NA	NA	NA	XP_001660442.2vacuolar fusion protein CCZ1 homolog
AAEL01340	NA	-0.0338360759611569	0.83202530479508	0.959075141516263	NA	NA	NA	XP_001663590.1E3 ubiquitin-protein ligase Mdm2 isoform X3
AAEL00115	uncharacterized LOC5568811	-0.0337859705800765	0.894420148198792	0.972739913152176	NA	aag01100 ; aag	KXJ76526.1 hypothet	CAJ84706.1beta-1,3-galactosyltransferase 6
AAEL00867	phospholipid phosphatase 6	-0.0337844024412151	0.811870451839456	0.950261122927395	NA	NA	ETN67258.1 presqua	XP_001653418.1phospholipid phosphatase 6
AAEL00549	septin-1	-0.0337413429156959	0.785478678297928	0.944372632355572	NA	NA	KFB46050.1 septin [A	XP_021704187.1septin-1 isoform X1
AAEL01082	60S acidic ribosomal protein P0	-0.033481059444899	0.820747737899125	0.954082956562373	NA	aag03010	AAM97779.1 ribosor	XP_001655016.160S acidic ribosomal protein P0
AAEL01426	proline-, glutamic acid- and leucine-rich protein 1	-0.0334745396207364	0.845949888872587	0.963198678766584	NA	NA	NA	XP_021693759.1proline-, glutamic acid- and leucine-rich protein 1

AAEL00045	uncharacterized LOC5577375	-0.0334474214763943	0.885937973500121	0.9709344693273	NA	NA	KXJ81286.1 hypothet	XP_001656458.2uncharacterized protein LOC5577375
AAEL00591	mitochondrial import inner membrane translocase subunit TIM14	-0.033445262873331	0.830690664069123	0.958693918567873	NA	NA	CRL05696.1 CLUMA_	XP_001651592.1mitochondrial import inner membrane translocase subunit TIM14
AAEL01515	lysosomal acid phosphatase	-0.033273817523406	0.78600945159391	0.944484065495843	NA	aag01100 ; aag	NA	XP_021713013.1lysosomal acid phosphatase
AAEL01064	SUMO-activating enzyme subunit 2	-0.0331432599988711	0.819823355614266	0.953369710395865	NA	aag04120	KXJ77119.1 hypothet	XP_021700868.1SUMO-activating enzyme subunit 2
AAEL00842	uncharacterized LOC5570603	-0.0331303005114276	0.914126767425525	0.977828474226146	NA	NA	XP_001846553.1 con	XP_019564121.1 zinc finger protein 26-like isoform X1
AAEL0044(	PRADC1-like protein	-0.032999887027825	0.846801862459674	0.963198678766584	NA	NA	XP_001864704.1 PA	XP_001649070.1PRADC1-like protein
AAEL0060:	dihydroorotate dehydrogenase (quinone), mitochondrial	-0.0329456632331167	0.857702295178606	0.964980728557884	240	aag01100 ; aag	XP_001843565.1 dih	XP_001657386.1dihydroorotate dehydrogenase (quinone), mitochondrial
AAEL01187	protein arginine N-methyltransferase 7	-0.0329001456055627	0.882699047129847	0.970357617045106	NA	NA	XP_019541694.1 PRE	XP_001662010.2protein arginine N-methyltransferase 7 isoform X1
AAEL00141	yrdC domain-containing protein, mitochondrial	-0.0327974698515935	0.930892710102811	0.98347583693566	NA	NA	ETN61266.1 ischemia	XP_021696425.1yrdC domain-containing protein, mitochondrial
AAEL00645	alcohol dehydrogenase class-3	-0.0327391042994144	0.800431439169778	0.946215055875588	00626 ; 00260 ; 00592 ;	aag01100 ; aag	KXJ76169.1 hypothet	XP_021705167.1alcohol dehydrogenase class-3
AAEL00584	mediator of RNA polymerase II transcription subunit 12	-0.0327378816226698	0.758232325971356	0.935439571457873	NA	NA	KXJ75245.1 hypothet	XP_001651515.2mediator of RNA polymerase II transcription subunit 12 isoform X1
AAEL01052	uncharacterized LOC5573481	-0.032572722430716	0.771730304827363	0.941147080452193	00564 ; 00591 ; 00565 ;	NA	NA	XP_021696025.1uncharacterized protein LOC5573481
AAEL02012	NA	-0.0324840520903655	0.845044693918794	0.963198678766584	NA	NA	NA	XP_019524901.1 FK506-binding protein 5-like
AAEL01197	ER membrane protein complex subunit 7 homolog	-0.0323994727297957	0.803692743984493	0.94649029363412	NA	NA	KXJ68818.1 hypothet	XP_001655787.1ER membrane protein complex subunit 7 homolog
AAEL02567	NA	-0.0322251534683268	0.914283437539857	0.977828474226146	NA	NA	NA	XP_019562153.1 dentin sialophosphoprotein-like
AAEL01978	NA	-0.0320613219883906	0.786833751271699	0.944549897171207	NA	aag03013	NA	XP_021710520.1nucleoprotein TPR isoform X2
AAEL0205(	NA	-0.0320477254970625	0.781125831030499	0.943915941844077	NA	NA	NA	XP_021694927.1protein lava lamp isoform X1
AAEL01739	CREB-binding protein	-0.0320364425869381	0.814830005726223	0.952015623780574	NA	aag04310 ; aag	NA	XP_011493407.2CREB-binding protein
AAEL00627	uncharacterized LOC5567702	-0.0320078560028501	0.875029568514496	0.969652664055335	NA	NA	KXJ80547.1 hypothet	XP_001651868.2uncharacterized protein LOC5567702
AAEL01247	cytokine receptor	-0.0318752966968871	0.81127218999283	0.95010454605765	NA	NA	KXJ75591.1 hypothet	XP_001662596.2cytokine receptor
AAEL0113(	calcium uptake protein 3, mitochondrial	-0.0318727135679989	0.853352151805455	0.964653958545747	NA	NA	ETN62282.1 hypothe	XP_021711097.1calcium uptake protein 3, mitochondrial
AAEL0009(	organic cation transporter protein	-0.0318238590405631	0.796796533328226	0.945890571556554	NA	NA	KFB47260.1 AGAP01	XP_001651636.1organic cation transporter protein
AAEL00647	dmX-like protein 2	-0.031682893410782	0.785580277124042	0.944372632355572	NA	NA	XP_019527390.1 PRE	XP_021711001.1dmX-like protein 2 isoform X2
AAEL01957	NA	-0.0316502362252036	0.816570268736952	0.952319314767154	NA	NA	NA	XP_021693883.1growth/differentiation factor 8
AAEL01437	uncharacterized LOC5564282	-0.0315592407294313	0.772176684358961	0.941147080452193	NA	NA	NA	XP_019560966.1 intracellular protein transport protein USO1-like
AAEL01132	guanine nucleotide-binding protein subunit gamma-1	-0.0315452397457926	0.775917695505476	0.941848282418248	NA	NA	CRK89209.1 CLUMA_	XP_019537662.1 guanine nucleotide-binding protein subunit gamma-1
AAEL0135(	leukocyte receptor cluster member 1 homolog	-0.0314670420981979	0.929080637031292	0.983153242837943	NA	NA	NA	XP_001663683.2leukocyte receptor cluster member 1 homolog
AAEL0139(	trafficking protein particle complex subunit 12	-0.0312488093944548	0.892096127171212	0.97252322736992	NA	NA	NA	XP_001664113.2trafficking protein particle complex subunit 12
AAEL00177	WASH complex subunit 3	-0.0312155000523693	0.886586181910204	0.9709344693273	NA	aag04144	XP_001845661.1 coil	XP_001653954.1WASH complex subunit 3
AAEL00775	transmembrane protein 205	-0.0312047635155531	0.846229196453807	0.963198678766584	NA	NA	KXJ73238.1 hypothet	XP_001658614.1transmembrane protein 205
AAEL01354	steroid hormone receptor ERR2	-0.0311901278983784	0.878836315026149	0.969652664055335	NA	NA	NA	XP_021712961.1steroid hormone receptor ERR2 isoform X1
AAEL0235(	NA	-0.031132784087368	0.886630877513905	0.9709344693273	NA	aag01100 ; aag	NA	XP_021693399.1cGMP-specific 3',5'-cyclic phosphodiesterase isoform X4
AAEL02149	NA	-0.0309650401166997	0.788144533726793	0.945198227466955	NA	NA	NA	XP_021694948.1histone deacetylase 6 isoform X1
AAEL00581	60S ribosomal protein L26	-0.0309556794372603	0.840860900708859	0.963198678766584	NA	aag03010	XP_001845703.1 60S	XP_001651456.160S ribosomal protein L26
AAEL00857	probable helicase with zinc finger domain	-0.0309500678914637	0.879948559928169	0.969652664055335	NA	NA	KXJ79514.1 hypothet	XP_021709050.1probable helicase with zinc finger domain
AAEL00668	probable splicing factor 3B subunit 5	-0.030891317425279	0.874018860681975	0.969652664055335	NA	aag03040	ETN67735.1 splicing	XP_001657963.1probable splicing factor 3B subunit 5
AAEL01131	juvenile hormone epoxide hydrolase 1	-0.030836339926712	0.813784169946755	0.951275651911844	NA	aag00981	XP_001842662.1 juv	XP_001661591.1juvenile hormone epoxide hydrolase 1
AAEL00932	3-hydroxyacyl-CoA dehydrogenase type-2	-0.0308168474932354	0.879066646152863	0.969652664055335	NA	aag01100 ; aag	NA	XP_001659937.23-hydroxyacyl-CoA dehydrogenase type-2 isoform X2
AAEL0258(	NA	-0.0307950780270068	0.943509500269492	0.987236796220786	NA	NA	NA	NA
AAEL00439	transient receptor potential cation channel protein painless	-0.0307486211837403	0.830129814570993	0.958428949419039	NA	NA	KXJ73830.1 hypothet	XP_001649039.2transient receptor potential cation channel protein painless
AAEL01365	uncharacterized LOC5578378	-0.0307341989640779	0.790314246439659	0.945793949302124	NA	NA	NA	XP_021695131.1uncharacterized protein LOC5578378
AAEL00724	GTPase-activating protein CdGAPr	-0.0306857073632136	0.838478352286908	0.962348963212321	NA	NA	XP_019538075.1 PRE	XP_021703195.1GTPase-activating protein CdGAPr isoform X1
AAEL01199	venom protease	-0.0305446269053832	0.888351414848536	0.970996238637524	NA	NA	XP_019538334.1 PRE	XP_001655815.2venom protease isoform X2
AAEL02418	NA	-0.0304818018640446	0.809700624009425	0.949044272688315	NA	NA	NA	XP_001660916.2sterol regulatory element-binding protein 1
AAEL0099(	battenin	-0.0304171159787538	0.846715891275152	0.963198678766584	NA	aag04142	NA	XP_019527975.1 battenin-like
AAEL01998	NA	-0.0303074169376809	0.86422200300845	0.967038506393142	NA	aag03040	NA	XP_021704111.1pre-mRNA-splicing factor ATP-dependent RNA helicase PRP16 isoform X2
AAEL01165	probable RNA-binding protein CG14230	-0.0302856376825087	0.871281118545332	0.969029518523332	NA	NA	KXJ75936.1 hypothet	XP_021709386.1probable RNA-binding protein CG14230
AAEL0140:	NIF3-like protein 1	-0.0302612716266911	0.878252063483352	0.969652664055335	NA	NA	NA	XP_001657324.1NIF3-like protein 1
AAEL00859	protein maelstrom homolog	-0.0302513978499297	0.879441925950088	0.969652664055335	NA	NA	KXJ71798.1 hypothet	XP_001653339.1protein maelstrom homolog
AAEL0024(	cytochrome b561 domain-containing protein 2	-0.0302339666964875	0.869105570321999	0.968652831735134	NA	NA	KXJ71716.1 hypothet	XP_001655155.1cytochrome b561 domain-containing protein 2
AAEL00319	inorganic pyrophosphatase	-0.0301223545793344	0.799293543644722	0.946215055875588	190	aag00190	XP_019931214.1 PRE	XP_001656514.2inorganic pyrophosphatase isoform X2
AAEL01941	NA	-0.0300994755804231	0.800059521812095	0.946215055875588	NA	NA	NA	XP_021705248.1putative leucine-rich repeat-containing protein DDB_G0290503
AAEL01977	NA	-0.0300876173002131	0.809375514978023	0.948933370455872	NA	NA	NA	XP_021700107.1heterogeneous nuclear ribonucleoprotein U
AAEL0198(	NA	-0.0300181029635397	0.879940284598033	0.969652664055335	NA	NA	NA	XP_021694481.1protein spitz
AAEL02108	NA	-0.0299092398478202	0.910102265918713	0.97691422813284	NA	NA	NA	XP_019553323.1 uncharacterized threonine-rich GPI-anchored glycoprotein PJ4664.02-like
AAEL01387	23 kDa integral membrane protein	-0.0297939710591727	0.797415750572099	0.945982283238732	NA	aag04142	NA	XP_001657181.223 kDa integral membrane protein isoform X1
AAEL01207	ubiquitin-associated protein 1	-0.0297303088091713	0.872990079173921	0.969029518523332	NA	NA	KXJ81899.1 hypothet	XP_001662233.1ubiquitin-associated protein 1
AAEL02559	NA	-0.0295940850886643	0.812392873344362	0.950268140513817	NA	NA	NA	XP_021696036.1protein DENND6A
AAEL02545	NA	-0.029588150277789	0.906733509115125	0.975993693196454	NA	NA	NA	XP_021705325.1inactive rhomboid protein 1
AAEL00251	uncharacterized LOC5574964	-0.0295575380903198	0.906730301794949	0.975993693196454	NA	NA	KXJ75374.1 hypothet	XP_001655438.1uncharacterized protein LOC5574964
AAEL0034(	aromatic-L-amino-acid decarboxylase	-0.029447870368479	0.931293870792072	0.98347583693566	NA	NA	KXJ75182.1 hypothet	XP_001656851.2aromatic-L-amino-acid decarboxylase
AAEL0040:	probable 28S ribosomal protein S6, mitochondrial	-0.0294227150829155	0.836298463486749	0.961471571839713	NA	aag03010	KXJ72125.1 hypothet	XP_001648287.1probable 28S ribosomal protein S6, mitochondrial
AAEL01135	carnitine O-acetyltransferase	-0.0293762583444574	0.916554237048755	0.978778239447911	NA	aag04146	KXJ74900.1 hypothet	XP_021694717.1carnitine O-acetyltransferase
AAEL00479	zinc finger protein rotund	-0.0293730792761604	0.897504509422351	0.973750254467651	NA	NA	KXJ77376.1 hypothet	XP_001649817.2zinc finger protein rotund isoform X2
AAEL0114:	myosin-VIIa	-0.0292584422945977	0.886732389408019	0.9709344693273	NA	NA	XP_014089710.1 PRE	XP_019544369.1 unconventional myosin-XV-like
AAEL00861	kallikrein 1-related peptidase b3	-0.0291813958017102	0.864263520508037	0.967038506393142	NA	NA	AAV90658.1 trypsin	XP_001659373.1kallikrein 1-related peptidase b3
AAEL00135	SAFB-like transcription modulator	-0.0291681765313462	0.801745026017892	0.946215055875588	NA	NA	KXJ74496.1 hypothet	XP_001653056.1SAFB-like transcription modulator
AAEL00292	uncharacterized LOC5576600	-0.0290946721163793	0.883721899027989	0.970521528629931	NA	NA	NA	XP_001656184.2uncharacterized protein LOC5576600 isoform X1
AAEL0103:	uncharacterized LOC5573256	-0.0290591082858789	0.860718019743578	0.966023193660905	NA	NA	NA	XP_001654467.1uncharacterized protein LOC5573256
AAEL00081	proteasome activator complex subunit 4B	-0.028983170039451	0.818515705460343	0.952837221640997	NA	aag03050	XP_019543208.1 PRE	XP_001651267.2proteasome activator complex subunit 4B isoform X2
AAEL00165	uncharacterized LOC5571655	-0.028948082164117	0.941226912782563	0.986042559017697	NA	NA	NA	XP_021701194.1uncharacterized protein LOC5571655 isoform X1
AAEL00028	protein FAM133A	-0.028570070758919	0.794046500966918	0.945793949302124	NA	NA	NA	XP_021696927.1protein FAM133A isoform X2
AAEL0136(	UDP-N-acetylglucosamine transporter	-0.0285336811386784	0.84145371492505	0.963198678766584	NA	NA	NA	XP_001663817.1UDP-N-acetylglucosamine transporter
AAEL01335	eukaryotic initiation factor 4A	-0.0284584378686495	0.836069134073145	0.961379090928137	NA	aag03013	NA	XP_001656676.1eukaryotic initiation factor 4A
AAEL01251	calcium-binding protein E63-1	-0.0282702245317177	0.803217840280197	0.94649029363412	NA	aag04070 ; aag	XP_019530827.1 PRE	XP_021696961.1calcium-binding protein E63-1 isoform X5
AAEL0019(	ejaculatory bulb-specific protein 3	-0.0282035643199965	0.825057947688735	0.955377826472606	NA	NA	XP_001844697.1 sen	XP_021703267.1ejaculatory bulb-specific protein 3 isoform X1
AAEL01247	protein vav	-0.0280390344026741	0.888016085136494	0.970996238637524	NA	NA	XP_019537008.1 PRE	XP_001662594.1protein vav isoform X2
AAEL01744	casein kinase II subunit beta	-0.0279883844796998	0.799608350253401	0.946215055875588	NA	aag03008 ; aag	NA	XP_021711618.1casein kinase II subunit beta isoform X1
AAEL0226(	NA	-0.0279684917734097	0.797853363275858	0.946188620016073	NA	NA	NA	XP_021696323.1actin-binding LIM protein 2 isoform X1
AAEL00865	secernin-1	-0.027892735185098	0.860850547026101	0.96604228460173	NA	NA	KXJ81935.1 hypothet	XP_019526861.1 secernin-1-like
AAEL0004(	teneurin-m	-0.0278925961644168	0.823031594521165	0.954452060294489	NA	NA	NA	XP_021710864.1teneurin-m isoform X3
AAEL0195:	NA	-0.0278749147026852	0.85687527260909	0.964980728557884	NA	NA	NA	XP_021713040.1DNA polymerase alpha subunit B

AAEL0093	carbonic anhydrase	-0.02785545025996	0.868724740550854	0.968652831735134	910	aag01100 ; aag	NA	XP_001659934.2carbonic anhydrase
AAEL0181	NA	-0.0278446694130408	0.897654461034872	0.973750254467651	NA	NA	NA	XP_021699795.1uncharacterized protein LOC5567692 isoform X2
AAEL0010	proton-coupled folate transporter	-0.0278275756271398	0.842218457524109	0.963198678766584	NA	NA	KXJ69387.1 hypothe	XP_001657891.1proton-coupled folate transporter
AAEL0078	ran GTPase-activating protein	-0.0278071644673011	0.864107543417326	0.967038506393142	NA	aag03013	KXJ75540.1 hypothe	XP_001658696.2ran GTPase-activating protein
AAEL0078	ras-related protein Rab-5B	-0.027782854471419	0.798426943347301	0.946215055875588	NA	aag04144 ; aag	ETN59321.1 rab5 [Ar	XP_001658691.1ras-related protein Rab-5B
AAEL0195	NA	-0.0277419840303866	0.840426753560133	0.963198678766584	NA	NA	NA	XP_021712987.1uncharacterized protein LOC5578982
AAEL0107	probable cysteine desulfurase, mitochondrial	-0.0276804146744707	0.793426018892387	0.945793949302124	730	aag01100 ; aag	KXJ76410.1 hypothe	XP_021702976.1probable cysteine desulfurase, mitochondrial
AAEL0078	cytochrome P450 4d2	-0.0276709600950058	0.829714732690423	0.958094785081062	NA	NA	XP_019529141.1 PRE	XP_001652925.2cytochrome P450 4d2 isoform X1
AAEL0104	probable histone-lysine N-methyltransferase Mes-4	-0.0275574121537643	0.890512277011682	0.972299099904304	310	aag01100 ; aag	NA	XP_001654536.2probable histone-lysine N-methyltransferase Mes-4 isoform X2
AAEL0066	uncharacterized LOC5568173	-0.0273837077918068	0.847896509689378	0.963435772275933	901	NA	KXJ69933.1 hypothe	XP_001652109.1uncharacterized protein LOC5568173
AAEL0232	NA	-0.0272897756864684	0.874400497624693	0.969652664055335	NA	NA	NA	XP_021698769.1muscle-specific protein 20
AAEL0054	annexin B10	-0.0272809073702063	0.874583876793728	0.969652664055335	NA	NA	KXJ70191.1 hypothe	XP_001650849.1annexin B10 isoform X1
AAEL0017	transmembrane 7 superfamily member 3	-0.0272646660853444	0.86441789129878	0.967038506393142	NA	NA	KXJ82648.1 hypothe	XP_001653967.3transmembrane 7 superfamily member 3
AAEL0130	adenylyl cyclase-associated protein 1	-0.0272102122782627	0.810335109148404	0.949535677369618	NA	NA	NA	XP_021700501.1microtubule-associated protein futsch isoform X1
AAEL0069	NA	-0.0271339935850066	0.909622728717732	0.97691422813284	970	aag00970	KXJ78887.1 hypothe	XP_001652351.2serine--tRNA synthetase-like protein Slimp
AAEL0032	zinc finger FYVE domain-containing protein 26 homolog	-0.0271095820611756	0.821966430040594	0.954414522300763	NA	NA	XP_001843658.1 con	XP_021693882.1zinc finger FYVE domain-containing protein 26 homolog
AAEL0052	tyrosine-protein phosphatase Lar	-0.0270293046614257	0.861420106505889	0.96610329632302	NA	NA	NA	XP_021700966.1tyrosine-protein phosphatase Lar isoform X2
AAEL0225	NA	-0.026725700821632	0.822641530229041	0.954452060294489	NA	NA	NA	XP_021711033.1serine protease easter
AAEL0018	NA	-0.0266067359650152	0.857859342193171	0.964980728557884	NA	NA	KXJ78456.1 hypothe	XP_019546847.1 protein CNPPD1-like
AAEL0089	cysteine-rich hydrophobic domain-containing protein 2	-0.0265532377498877	0.809750157245585	0.949044272688315	NA	NA	KXJ80109.1 hypothe	XP_001653537.1cysteine-rich hydrophobic domain-containing protein 2
AAEL0080	uncharacterized LOC5569932	-0.0265292430935472	0.811218737535502	0.95010454605765	NA	NA	KXJ74252.1 hypothe	XP_001868638.1monocarboxylate transporter
AAEL0084	structure-specific endonuclease subunit SLX4	-0.0264353222354034	0.888307941435225	0.970996238637524	NA	NA	KXJ82508.1 hypothe	XP_001659271.2structure-specific endonuclease subunit SLX4
AAEL0267	NA	-0.0263918160742069	0.806835517967965	0.948040966581563	NA	NA	NA	XP_021700598.1protein ref(2)P
AAEL0066	tachykinins	-0.0263607516619039	0.900379980056249	0.974065297962641	NA	NA	XP_019534849.1 PRE	XP_001652135.2tachykinins isoform X1
AAEL0231	NA	-0.0263269089028789	0.948354836076227	0.987881609350457	NA	NA	NA	XP_021701669.1protein turtle homolog A
AAEL0183	NA	-0.0262561145153799	0.85380696007737	0.964653958545747	NA	NA	NA	XP_019534720.1 ATP-dependent DNA helicase Q5
AAEL0194	NA	-0.0261853484725853	0.831316864540533	0.958742264774772	NA	NA	NA	XP_019538909.1 ras-related protein Rap-2a
AAEL0021	transcription initiation factor IIA subunit 2	-0.0261565794485015	0.853847695322225	0.964653958545747	NA	aag03022	XP_001848846.1 tra	XP_001654851.1transcription initiation factor IIA subunit 2
AAEL0109	ecdysone 20-monooxygenase	-0.0261461913547622	0.947026699656139	0.987707093985696	NA	aag00981	NA	XP_021712285.1ecdysone 20-monooxygenase
AAEL0210	NA	-0.0260444609000764	0.869434505100424	0.968652831735134	NA	NA	NA	NA
AAEL0103	uncharacterized LOC5573210	-0.025967171351557	0.822838010390093	0.954452060294489	NA	NA	NA	XP_001654430.2uncharacterized protein LOC5573210 isoform X2
AAEL0028	thioredoxin reductase 1, mitochondrial	-0.0259326967409105	0.812225020241409	0.950268140513817	450	aag00450	XP_019531873.1 PRE	XP_001662666.1thioredoxin reductase 1, mitochondrial isoform X2
AAEL0119	oxysterol-binding protein-related protein 9	-0.0258905353470486	0.842871848122647	0.963198678766584	NA	NA	KFB43298.1 hypothe	XP_021699693.1oxysterol-binding protein-related protein 9 isoform X1
AAEL0023	NA	-0.0258685985576786	0.866001494943349	0.967927335900485	NA	NA	XP_019551005.1 PRE	XP_001655138.2oocyte zinc finger protein XlCOF6 isoform X2
AAEL0073	C2 domain-containing protein 5	-0.0258395197730564	0.898797997107023	0.973780616635202	NA	NA	XP_019546213.1 PRE	XP_021704770.1C2 domain-containing protein 5
AAEL0094	uncharacterized LOC5572004	-0.0257637328765551	0.912955591735483	0.977561298149576	NA	NA	NA	XP_001660110.2uncharacterized protein LOC5572004 isoform X1
AAEL0054	melanization protease 1	-0.0257221897087965	0.876394536372094	0.969652664055335	NA	NA	KXJ72527.1 hypothe	XP_001650899.2melanization protease 1
AAEL0051	esterase B1	-0.0256768494191357	0.91352565706533	0.977731438769662	NA	NA	NA	XP_021698893.1esterase B1 isoform X4
AAEL0042	glycogen [starch] synthase	-0.0255828324528868	0.872506532684875	0.969029518523332	500	aag01100 ; aag	ETN67493.1 glycoger	XP_001648704.1glycogen [starch] synthase
AAEL0043	rab proteins geranylgeranyltransferase component A	-0.025532472253510 1	0.867186974588511	0.96822706250455	NA	NA	XP_001855828.1 rab	XP_021704602.1rab proteins geranylgeranyltransferase component A
AAEL0144	cytochrome c oxidase protein 20 homolog	-0.0255166376283254	0.865043278703439	0.967527624717057	NA	NA	NA	XP_001648865.1cytochrome c oxidase protein 20 homolog
AAEL0199	NA	-0.0254930231072573	0.827756456876655	0.956671052019465	NA	aag04512	NA	XP_021699177.1uncharacterized protein LOC5577287 isoform X1
AAEL0058	structural maintenance of chromosomes protein 1A	-0.0254752915652597	0.857070906960091	0.964980728557884	NA	NA	CAD59403.1 SMC1 p	XP_001651465.2structural maintenance of chromosomes protein 1A
AAEL0000	uncharacterized LOC5563641	-0.025472603165731	0.909906436710582	0.97691422813284	NA	NA	KXJ82836.1 hypothe	XP_001647895.2uncharacterized protein LOC5563641
AAEL0142	probable sulfite oxidase, mitochondrial	-0.0254097174764965	0.893132349380332	0.972739913152176	NA	aag01100 ; aag	NA	XP_021706109.1probable sulfite oxidase, mitochondrial
AAEL0244	NA	-0.025333845937668 5	0.933223373981601	0.98347583693566	NA	NA	NA	XP_021711737.1uncharacterized protein LOC110680229
AAEL0121	caspase	-0.0251406075724245	0.912565562068164	0.977354318220583	NA	aag04214 ; aag	KXJ84059.1 hypothe	XP_019563286.1 caspase-like
AAEL0016	uncharacterized LOC5571835	-0.0249912671323413	0.89487682468168	0.972903655567205	NA	NA	NA	XP_001659966.1uncharacterized protein LOC5571835 isoform X3
AAEL0009	probable elongation factor 1-beta	-0.0249653130044203	0.869684677168178	0.968652831735134	NA	NA	KXJ68189.1 hypothe	XP_001657499.1probable elongation factor 1-beta
AAEL0096	glyoxylate reductase/hydroxypyruvate reductase	-0.0249481431271207	0.846408493004475	0.963198678766584	NA	aag01100 ; aag	NA	XP_001653924.1glyoxylate reductase/hydroxypyruvate reductase
AAEL0136	N-chimaerin	-0.0248739055127542	0.94416509368866	0.987296271832165	NA	NA	NA	XP_019545658.1 beta-chimaerin-like
AAEL0181	NA	-0.0248330364570063	0.881034075522842	0.969743049934758	NA	NA	NA	XP_019562558.1 mucin-5AC isoform X4
AAEL0003	RNA-binding protein 45	-0.0247998231081152	0.880641142108553	0.969743049934758	NA	NA	KXJ76011.1 hypothe	XP_001656064.1RNA-binding protein 45
AAEL0282	NA	-0.0247830244469234	0.854148207065892	0.964756573145985	NA	NA	NA	XP_021704122.1mediator of RNA polymerase II transcription subunit 1
AAEL0199	NA	-0.0247143021525096	0.918323949098633	0.979657756188126	NA	NA	NA	XP_021702049.1receptor-type tyrosine-protein phosphatase kappa isoform X2
AAEL0259	NA	-0.0247034706378647	0.89331088778957	0.972739913152176	00980 ; 00982 ; 00983 ;	aag01100 ; aag	NA	XP_021694972.1glutathione S-transferase theta-1
AAEL0100	protein tramtrack, beta isoform	-0.0245413340003782	0.859883407466176	0.965724450478577	NA	NA	NA	XP_021711703.1sex determination protein fruitless isoform X1
AAEL0084	pyrroline-5-carboxylate reductase	-0.0244846775502534	0.865547022236653	0.9678896219008	330	aag01100 ; aag	XP_019525120.1 PRE	XP_001653270.2pyrroline-5-carboxylate reductase
AAEL0085	uncharacterized LOC5570785	-0.024448913289747	0.917277105878546	0.979322882967209	NA	NA	NA	XP_001653294.1uncharacterized protein LOC5570785 isoform X1
AAEL0011	putative sodium-coupled neutral amino acid transporter 10	-0.024425292343838	0.872419696442924	0.969029518523332	NA	NA	KXJ80608.1 hypothe	XP_001652402.1putative sodium-coupled neutral amino acid transporter 10
AAEL0226	NA	-0.0243236683436202	0.891102619489752	0.972336404299425	NA	NA	NA	XP_021710334.1 SWI/SNF complex subunit SMARCC2
AAEL0011	UBX domain-containing protein 1	-0.0240977327303154	0.894153459352977	0.972739913152176	NA	NA	KXJ72369.1 hypothe	XP_001658165.2UBX domain-containing protein 1
AAEL0105	polypeptide N-acetylgalactosaminyltransferase 16	-0.0239375208643886	0.89425281187588	0.972739913152176	NA	NA	KXJ76480.1 hypothe	XP_001654672.2polypeptide N-acetylgalactosaminyltransferase 16
AAEL0196	NA	-0.0238550619103726	0.856681742813456	0.964980728557884	NA	NA	NA	XP_021701904.1uncharacterized protein LOC5575208 isoform X1
AAEL0016	vesicle-fusing ATPase 1	-0.0236083385237898	0.839647973556806	0.962916332585959	NA	NA	KXJ76058.1 hypothe	XP_021704098.1vesicle-fusing ATPase 1
AAEL0065	pre-miRNA 5'-monophosphate methyltransferase	-0.0235555112126848	0.93232374000991	0.98347583693566	NA	NA	XP_019532629.1 PRE	XP_001652030.2pre-miRNA 5'-monophosphate methyltransferase
AAEL0041	tectonin beta-propeller repeat-containing protein	-0.0235063522168673	0.869874151321082	0.968652831735134	NA	NA	KXJ78881.1 hypothe	XP_001648588.2tectonin beta-propeller repeat-containing protein
AAEL0099	zinc finger protein 678	-0.0233616418762157	0.903230516176238	0.975437735306161	NA	NA	NA	XP_001654124.2zinc finger protein 678
AAEL0198	NA	-0.0232758936212109	0.915706988134367	0.978554837395476	NA	NA	NA	XP_021709754.1uncharacterized protein LOC5568958
AAEL0044	NA	-0.0231636963752371	0.843030156388127	0.963198678766584	04151 ; 05165 ; 04714 ;	aag04150	XP_019538573.1 PRE	XP_001649206.2 ribosomal protein S6 kinase 2 beta
AAEL0105	uncharacterized LOC5573484	-0.0231486821929251	0.918864243249032	0.979880492105411	NA	NA	NA	XP_001654632.1uncharacterized protein LOC5573484
AAEL0117	trimethylguanosine synthase	-0.023065519156232	0.898067638843434	0.973750254467651	NA	aag03013	KXJ82315.1 hypothe	XP_021708990.1trimethylguanosine synthase
AAEL0223	NA	-0.0230452951531694	0.912298962060819	0.977296280428831	NA	NA	NA	XP_021702264.1UPF0565 protein C2orf69 homolog
AAEL0131	serine protease 53	-0.0230207101741261	0.856367941070111	0.964980728557884	NA	NA	NA	XP_001656391.2serine protease 53
AAEL0028	phosphoinositide 3-kinase regulatory subunit 4	-0.0230040357948808	0.903477645055304	0.975494447471861	NA	aag04140 ; aag	KXJ84353.1 hypothe	XP_001662654.2phosphoinositide 3-kinase regulatory subunit 4
AAEL0170	zinc finger protein 37 homolog	-0.022933041681266	0.925876216122163	0.982258702730868	NA	NA	NA	XP_021706082.1zinc finger protein 37 homolog
AAEL0097	probable nuclear transport factor 2	-0.0229076567568153	0.836016763903128	0.961379090928137	NA	NA	NA	XP_001654040.1probable nuclear transport factor 2 isoform X2
AAEL0021	snRNA-activating protein complex subunit 3	-0.0228144162655698	0.948374933384258	0.987881609350457	NA	NA	KXJ72618.1 hypothe	XP_001654697.2snRNA-activating protein complex subunit 3
AAEL0110	dopamine N-acetyltransferase	-0.0227804296592199	0.831528147703484	0.958742264774772	NA	NA	XP_019533201.1 PRE	XP_001661400.1dopamine N-acetyltransferase isoform X1
AAEL0123	protein 5NUC	-0.0227649723395775	0.851851171719551	0.964536838405106	NA	aag01100 ; aag	KXJ80594.1 hypothe	XP_021704325.1protein 5NUC
AAEL0136	pyruvate dehydrogenase E1 component subunit alpha, mitochondrial	-0.0225951644511465	0.86719589600609	0.96822706250455	00010 ; 00620 ; 00020	aag01100 ; aag	NA	XP_021705977.1pyruvate dehydrogenase E1 component subunit alpha, mitochondrial
AAEL0083	probable glutathione peroxidase 2	-0.0225631629874928	0.843152630564967	0.963198678766584	NA	NA	KXJ83512.1 hypothe	XP_001653252.2probable glutathione peroxidase 2

AAEL00697	repressor of RNA polymerase III transcription MAF1 homolog	-0.0225590469729649	0.872725470279068	0.969029518523332	NA	NA	ETN60775.1 represso	XP_021702789.1repressor of RNA polymerase III transcription MAF1 homolog
AAEL00652	acetylcholine receptor subunit alpha-like 1	-0.0225036621015253	0.867982230571587	0.96863134437578	NA	NA	XP_019533063.1 PRE	XP_001652011.2acetylcholine receptor subunit alpha-like 1
AAEL00318	uncharacterized LOC5577495	-0.0224946497100414	0.913729832937815	0.977804619455027	NA	NA	KXJ77113.1 hypothet	AAL76022.1putative serpin
AAEL01018	uncharacterized LOC5572962	-0.0224370359335184	0.892231585107831	0.972555679479065	NA	NA	NA	KFB37108.1AGAP003775-like protein
AAEL01831	NA	-0.022358159942567	0.886856082856659	0.9709344693273	NA	aag03013	NA	XP_021701093.1protein rigor mortis
AAEL00234	plexin-A2	-0.0223388812270581	0.84443378999578	0.963198678766584	NA	NA	XP_001848140.1 ple:	XP_019562811.1 plexin-A2-like
AAEL00818	arrestin domain-containing protein 17	-0.022292160143316	0.852960253171148	0.964653958545747	NA	NA	KXJ74731.1 hypothet	XP_001659007.1arrestin domain-containing protein 17
AAEL02423	NA	-0.0222846430029996	0.847204602235309	0.963198678766584	NA	NA	NA	XP_021696487.1talin-2 isoform X5
AAEL00458	contactin-associated protein-like 4	-0.0222531770442237	0.842181757224151	0.963198678766584	NA	NA	KXJ82550.1 hypothet	XP_021696168.1contactin-associated protein-like 4 isoform X3
AAEL00813	putative GTP-binding protein 6	-0.0222035550298983	0.875965270423627	0.969652664055335	NA	NA	KXJ76584.1 hypothet	XP_021709430.1putative GTP-binding protein 6
AAEL01296	probable ATP-dependent RNA helicase DDX52	-0.0221238414096231	0.918301719380351	0.979657756188126	NA	NA	NA	XP_001663141.2probable ATP-dependent RNA helicase DDX52
AAEL01965	NA	-0.0220706386721853	0.89448781846013	0.972739913152176	NA	NA	NA	XP_001658864.2myosin light chain kinase, smooth muscle isoform X1
AAEL00608	N-alpha-acetyltransferase 15, NatA auxiliary subunit	-0.0220338860419623	0.846269538934842	0.963198678766584	NA	NA	ETN66349.1 NMDA r	XP_021710553.1N-alpha-acetyltransferase 15, NatA auxiliary subunit
AAEL00501	malectin-A	-0.0219525809975875	0.901799343326177	0.974443113819573	NA	NA	KXJ71843.1 hypothet	XP_019546378.1 malectin-A-like
AAEL01353	ribosome biogenesis protein NSA2 homolog	-0.0217524676165453	0.884720398966213	0.970521528629931	NA	NA	NA	XP_019555035.1 ribosome biogenesis protein NSA2 homolog
AAEL01005	protein TBRG4	-0.0217315934486484	0.846854643306274	0.963198678766584	NA	NA	NA	XP_001654178.2protein TBRG4 isoform X1
AAEL02791	NA	-0.0216968715265128	0.927801676422398	0.982649508778163	NA	NA	NA	XP_021713418.1sodium-coupled monocarboxylate transporter 1 isoform X2
AAEL01332	uncharacterized LOC5577691	-0.0216939569439124	0.844972615486994	0.963198678766584	NA	NA	NA	XP_021705925.1uncharacterized protein LOC5577691
AAEL00285	tubulin beta-1 chain	-0.0214711281094789	0.852680857662961	0.964653958545747	NA	aag04145	NP_523795.2 beta-Tu	XP_001656025.1tubulin beta-1 chain
AAEL01465	protein C19orf12 homolog	-0.0214073454137266	0.884881072071845	0.970521528629931	NA	NA	NA	XP_001649261.1protein C19orf12 homolog
AAEL00488	gastrula zinc finger protein 5-1	-0.0213786859489691	0.906927651596007	0.975993693196454	NA	NA	KXJ81212.1 hypothet	XP_001649967.2gastrula zinc finger protein 5-1
AAEL01044	NA	-0.0212292557658406	0.954855325266958	0.989303184592491	NA	NA	NA	XP_001660841.2muscle M-line assembly protein unc-89
AAEL01961	NA	-0.0211991490419345	0.9111426024852	0.976991789015945	NA	NA	NA	XP_021703567.1dual specificity tyrosine-phosphorylation-regulated kinase 2 isoform X1
AAEL01127	mitochondrial glutamate carrier 1	-0.0211785462381137	0.846353566650888	0.963198678766584	NA	NA	ETN66666.1 mitocho	XP_001661549.2mitochondrial glutamate carrier 1
AAEL01010	protein fem-1 homolog B	-0.0211509274999473	0.862462863084574	0.966468488016169	NA	NA	NA	XP_001654238.1protein fem-1 homolog B isoform X1
AAEL01016	40S ribosomal protein S2	-0.0210865109380269	0.892615731273477	0.972739913152176	NA	aag03010	NA	XP_001654299.140S ribosomal protein S2
AAEL00783	probable cytochrome P450 4d14	-0.0210236737912112	0.940163374371404	0.985798796572901	NA	NA	KXJ70416.1 hypothet	XP_001658673.2probable cytochrome P450 4d14 isoform X2
AAEL02058	NA	-0.0210105198346004	0.910281470161792	0.97691422813284	NA	NA	NA	XP_021707848.1transmembrane protein 222
AAEL00367	heterogeneous nuclear ribonucleoprotein M	-0.0209321017277165	0.842118244620127	0.963198678766584	NA	NA	CAH05070.1 Hrp59 p	XP_001657115.1heterogeneous nuclear ribonucleoprotein M isoform X2
AAEL00078	PRKR-interacting protein 1 homolog	-0.0208150561632676	0.937539702417099	0.984619988774498	NA	NA	KXJ77956.1 hypothet	XP_001651008.2PRKR-interacting protein 1 homolog
AAEL01232	membrane-bound transcription factor site-2 protease	-0.0206641942775317	0.925599857261577	0.982258702730868	NA	aag04141	XP_019552037.1 PRE	XP_001662422.2membrane-bound transcription factor site-2 protease
AAEL01072	sphingosine-1-phosphate phosphatase 1	-0.0205199135087553	0.891962924035653	0.97252322736992	NA	aag01100 ; aag	KXJ84052.1 hypothet	XP_001661054.1sphingosine-1-phosphate phosphatase 1
AAEL00338	b(0,+)-type amino acid transporter 1	-0.0204555982775265	0.871146624293415	0.969029518523332	NA	NA	XP_019527793.1 PRE	XP_001656755.2b(0,+)-type amino acid transporter 1 isoform X1
AAEL00162	UDP-N-acetylhexosamine pyrophosphorylase	-0.0203687775463382	0.89755891719702	0.973750254467651	NA	aag01100 ; aag	KXJ77610.1 hypothet	XP_001659746.2UDP-N-acetylhexosamine pyrophosphorylase
AAEL00941	uncharacterized LOC5571928	-0.0203660380666106	0.868467353592553	0.96863134437578	NA	NA	NA	XP_019539683.1 28S ribosomal protein S27, mitochondrial-like
AAEL00432	zinc finger MYM-type protein 4	-0.0203230810615031	0.861639834049448	0.96610329632302	NA	NA	KXJ69144.1 hypothet	XP_001648900.1zinc finger MYM-type protein 4
AAEL00396	neurotrimin	-0.0201577654214821	0.91048915197927	0.97691422813284	NA	NA	KXJ77644.1 hypothet	XP_019552617.1 neurotrimin-like
AAEL01957	NA	-0.0201271560167808	0.896224044013877	0.973419069671699	NA	NA	NA	XP_021703832.1uncharacterized protein LOC5565895 isoform X4
AAEL01377	PR domain zinc finger protein 1	-0.0198794000194111	0.939751758434553	0.985798796572901	NA	NA	NA	XP_021697776.1PR domain zinc finger protein 1 isoform X1
AAEL00387	translationally-controlled tumor protein homolog	-0.0198647558699906	0.890963529869734	0.972336404299425	NA	NA	ETN66077.1 translati	XP_001664282.1translationally-controlled tumor protein homolog
AAEL01119	tRNA:m(4)X modification enzyme TRM13 homolog	-0.0198267648730309	0.91994029456636	0.980340877547081	NA	NA	KXJ75118.1 hypothet	XP_001655179.1tRNA:m(4)X modification enzyme TRM13 homolog
AAEL00576	lysosomal alpha-mannosidase	-0.0197842152932425	0.86259570659387	0.966468488016169	511	aag04142 ; aag	XP_019932137.1 PRE	XP_021703511.1lysosomal alpha-mannosidase isoform X2
AAEL00080	dnaJ homolog subfamily C member 3	-0.0197710076751246	0.857548714560535	0.964980728557884	NA	aag04141	KXJ83782.1 hypothet	XP_001651257.2dnaJ homolog subfamily C member 3
AAEL00102	solute carrier family 25 member 51	-0.0196607675959504	0.900767783240199	0.974252903247646	NA	NA	KFB46839.1 AGAP00	XP_001657866.1solute carrier family 25 member 51
AAEL02178	NA	-0.01962976658608	0.888268869778689	0.970996238637524	NA	NA	NA	XP_021700536.1 rho GTPase-activating protein conundrum
AAEL00414	phagocyte signaling-impaired protein	-0.0196010821162619	0.904119026785015	0.9756893731061	NA	NA	Q17DK2.2 RecName:	XP_021709566.1phagocyte signaling-impaired protein
AAEL00622	dehydrogenase/reductase SDR family member 4	-0.0195895727036815	0.886643804444578	0.9709344693273	NA	aag01100 ; aag	KXJ81809.1 hypothet	XP_001657588.2dehydrogenase/reductase SDR family member 4
AAEL01073	zinc finger protein 664	-0.0195654054035618	0.932100531828731	0.98347583693566	NA	NA	KXJ76411.1 hypothet	XP_021702977.1zinc finger protein 664
AAEL00365	inhibitor of growth protein 1	-0.0194966510832606	0.922777293629489	0.981343310857761	NA	NA	KXJ73146.1 hypothet	XP_021705140.1inhibitor of growth protein 1
AAEL00960	probable 28S ribosomal protein S16, mitochondrial	-0.0194806931951547	0.899575129665013	0.97393667311859	NA	aag03010	NA	XP_001653853.2probable 28S ribosomal protein S16, mitochondrial isoform X1
AAEL00418	ubiquinone biosynthesis monooxygenase COQ6, mitochondrial	-0.0194204220176657	0.903823274714912	0.975599035064706	NA	aag01100 ; aag	XP_019541459.1 PRE	XP_021694709.1ubiquinone biosynthesis monooxygenase COQ6, mitochondrial
AAEL00094	ER membrane protein complex subunit 4	-0.0194094703955414	0.899637222506848	0.97393667311859	NA	NA	XP_001847576.1 tra	XP_001657468.1ER membrane protein complex subunit 4 isoform X2
AAEL00275	anaphase-promoting complex subunit 5	-0.0192573193582784	0.949874082860946	0.98806088919239	NA	aag04120	KXJ70237.1 hypothet	XP_021697300.1anaphase-promoting complex subunit 5
AAEL01237	uncharacterized LOC5576178	-0.0192393270901434	0.961093014260681	0.989923653148286	NA	NA	KXJ71885.1 hypothet	XP_001655981.1uncharacterized protein LOC5576178
AAEL00872	conserved oligomeric Golgi complex subunit 8	-0.0192171516149048	0.905333623314283	0.975993693196454	NA	NA	KXJ73537.1 hypothet	XP_001653443.2conserved oligomeric Golgi complex subunit 8
AAEL00706	mRNA export factor	-0.0190146775298015	0.915363808853822	0.978301715339473	NA	aag03013	KFB49976.1 hypothe	XP_001652531.1mRNA export factor
AAEL00517	adenylate cyclase type 2	-0.0190118921311308	0.920467852858811	0.980340877547081	230	aag01100 ; aag	XP_019529866.1 PRE	XP_021695355.1adenylate cyclase type 2 isoform X2
AAEL00211	ubiquitin-conjugating enzyme E2-17 kDa	-0.0189621887535958	0.870019088707985	0.968652831735134	NA	aag04120	NP_001298509.1 ubi	NP_001298509.1ubiquitin-conjugating enzyme E2-17 kDa
AAEL02512	NA	-0.0189454760225287	0.927152187493988	0.982472473084084	NA	NA	NA	XP_021697245.1golgin subfamily A member 4-like
AAEL00076	serine protease 7	-0.0189010130816449	0.922920055170364	0.981343310857761	NA	NA	XP_019550188.1 PRE	XP_001650979.2serine protease 7 isoform X1
AAEL00118	ras-related GTP-binding protein A	-0.0187431087431017	0.887106670030811	0.970995072870646	NA	aag04150 ; aag	XP_001849340.1 GTI	XP_001658202.2ras-related GTP-binding protein A
AAEL01306	guanine nucleotide-binding protein subunit beta-like protein	-0.0187239054583563	0.906479998718263	0.975993693196454	NA	NA	NA	XP_001663282.1guanine nucleotide-binding protein subunit beta-like protein
AAEL01010	tetraspanin-9	-0.0186442023898708	0.861222698674432	0.96610329632302	NA	NA	NA	XP_019560416.1 tetraspanin-9-like
AAEL02532	NA	-0.0185850907969904	0.878807994055907	0.969652664055335	NA	NA	NA	XP_021693915.1electron transfer flavoprotein subunit beta
AAEL00691	acetyl-CoA acetyltransferase	-0.0184434085782872	0.901950731022943	0.974492265688989	NA	aag01100 ; aag	KXJ70120.1 hypothet	XP_001865693.1acetyl-coA acetyl transferase II
AAEL00770	malate dehydrogenase, cytoplasmic	-0.0183286944802513	0.876467071661762	0.969652664055335	00620 ; 00680 ; 00630 ;	aag01100 ; aag	KXJ76965.1 hypothet	XP_001652832.2malate dehydrogenase, cytoplasmic isoform X1
AAEL01708	cytochrome c oxidase copper chaperone	-0.0183187821119262	0.866334894787955	0.968074250389273	NA	aag01100 ; aag	NA	XP_011493011.1cytochrome c oxidase copper chaperone
AAEL01445	protein borderless	-0.0183163285643975	0.876171142840438	0.969652664055335	NA	NA	NA	XP_021701512.1protein borderless isoform X2
AAEL00141	inner nuclear membrane protein Man1	-0.0182559343703103	0.901366541824065	0.974380285920978	NA	NA	KXJ79713.1 hypothet	XP_001659175.1inner nuclear membrane protein Man1
AAEL00817	GTP-binding protein Rheb homolog	-0.0181378069405622	0.879334353979631	0.969652664055335	NA	aag04150 ; aag	ABV60338.1 putative	XP_001659013.1GTP-binding protein Rheb homolog
AAEL00723	nipped-B protein	-0.0181234082357699	0.893739094495841	0.972739913152176	NA	NA	KXJ77108.1 hypothet	XP_021703191.1nipped-B protein
AAEL00590	protein NASP homolog	-0.0180724266062793	0.88387567411018	0.970521528629931	NA	NA	KXJ83868.1 hypothet	XP_021710813.1protein NASP homolog
AAEL00908	epsin-1	-0.0180600372347931	0.872864019781666	0.969029518523332	NA	aag04144	KXJ70363.1 hypothet	XP_021695461.1epsin-1 isoform X1
AAEL01804	NA	-0.0180134089646446	0.921312283300832	0.980672716128891	NA	aag01100 ; aag	NA	XP_021702424.1UDP-glucuronosyltransferase 2C1 isoform X1
AAEL00560	glycerophosphodiester phosphodiesterase 1	-0.0179828890395158	0.927974813223455	0.982649508778163	NA	NA	XP_001847486.1 con	XP_001651141.1glycerophosphodiester phosphodiesterase 1
AAEL01040	uncharacterized LOC5573314	-0.0179514219311403	0.961093014250065	0.989923653148286	NA	NA	NA	XP_001654533.1uncharacterized protein LOC5573314
AAEL00248	sestrin homolog	-0.0178567197668287	0.879023309354075	0.969652664055335	NA	NA	XP_001688028.1 AG	XP_021697010.1sestrin homolog
AAEL01489	charged multivesicular body protein 7	-0.0177963960519969	0.932343815228383	0.98347583693566	NA	aag04144	NA	XP_001649939.1charged multivesicular body protein 7
AAEL00948	uncharacterized LOC5572006	-0.0177931603572293	0.933199422960252	0.98347583693566	NA	NA	NA	XP_001660112.2uncharacterized protein LOC5572006
AAEL01805	NA	-0.0177508436650485	0.936292886428856	0.98424319806035	04151 ; 05165 ; 04714 ;	aag04350	NA	XP_001651046.2 rho-associated protein kinase 1
AAEL02614	NA	-0.0176154543722213	0.909666883026544	0.97691422813284	NA	NA	NA	XP_021709128.1 protein slit
AAEL01717	farnesol dehydrogenase-like	-0.0176148803704538	0.918148002953915	0.979657756188126	NA	aag00981 ; aag	NA	XP_011493633.2farnesol dehydrogenase-like

AAEL0130	uncharacterized LOC5577164	-0.0175733341409574	0.954799069754688	0.989303184592491	NA	NA	NA	XP_001663251.1uncharacterized protein LOC5577164
AAEL0081	protein purity of essence	-0.0175702296928853	0.879335963172982	0.969652664055335	NA	NA	KXJ72043.1 hypothe	XP_021706331.1protein purity of essence
AAEL0069	NA	-0.0175065071515051	0.902201533420929	0.974554188590838	NA	NA	KXJ73726.1 hypothe	XP_001658076.1uncharacterized protein LOC5568497
AAEL0063	serine protease HTRA2, mitochondrial	-0.0174998461541586	0.935349669610493	0.984004534098063	NA	aag04214 ; aag	KXJ71478.1 hypothe	XP_001651967.2serine protease HTRA2, mitochondrial
AAEL0019	sorting nexin-16	-0.0174396167180114	0.91246412832214	0.977354318220583	NA	NA	KXJ71947.1 hypothe	XP_001654339.2sorting nexin-16
AAEL0015	kinesin-like protein KIF13A	-0.0173675925554616	0.919954785048762	0.980340877547081	NA	NA	XP_019537630.1 PRE	XP_021695842.1kinesin-like protein KIF13A isoform X2
AAEL0130	mediator of RNA polymerase II transcription subunit 16	-0.0173626401374676	0.951504863957341	0.988479552477666	NA	NA	NA	XP_001663311.2mediator of RNA polymerase II transcription subunit 16
AAEL0057	probable N-acetyltransferase san	-0.0173130704994013	0.874293558562772	0.969652664055335	NA	NA	KXJ69210.1 hypothe	XP_001651349.2 probable N-acetyltransferase san
AAEL0109	queuine tRNA-ribosyltransferase accessory subunit 2	-0.0172766365427025	0.93931912290652	0.985701285093602	NA	NA	KXJ82951.1 hypothe	XP_001655069.1queuine tRNA-ribosyltransferase accessory subunit 2
AAEL0132	cysteine proteinase inhibitor 1	-0.0171029722812072	0.926088565671563	0.982258702730868	NA	NA	NA	XP_021694027.1cysteine proteinase inhibitor 1
AAEL0043	short coiled-coil protein homolog	-0.0170319116242591	0.905198195283907	0.975993693196454	NA	NA	ETN59713.1 short co	XP_001648899.1short coiled-coil protein homolog
AAEL0220	NA	-0.0169858622142178	0.916002547767643	0.978630706287391	NA	NA	NA	NA
AAEL0156	mono [ADP-ribose] polymerase PARP16	-0.0169366705047833	0.950926386182512	0.988386462829441	NA	NA	NA	XP_001647568.1mono [ADP-ribose] polymerase PARP16
AAEL0149	cytochrome c oxidase subunit 5A, mitochondrial	-0.0168081226498501	0.89802577754301	0.973750254467651	NA	aag01100 ; aag	NA	XP_001650058.1cytochrome c oxidase subunit 5A, mitochondrial
AAEL0057	NA	-0.01680234894292	0.91421698963581	0.977828474226146	NA	NA	KXJ80705.1 hypothe	XP_001651441.2uncharacterized protein LOC5567042
AAEL0198	NA	-0.0167894384158937	0.94007003221799	0.985798796572901	NA	NA	NA	XP_019548739.1 double-stranded RNA-binding protein Staufen homolog 2
AAEL0054	uncharacterized LOC5566544	-0.0167202318324686	0.879982022393852	0.969652664055335	NA	NA	KFB40414.1 AGAP01	KFB40414.1AGAP011317-like protein
AAEL0229	NA	-0.0166717886750706	0.958940152115388	0.989441920233345	NA	NA	NA	NA
AAEL0018	uncharacterized LOC5572454	-0.0166382205177557	0.900391156756164	0.974065297962641	NA	NA	KXJ72738.1 hypothe	XP_019550432.1 tyrosine-protein phosphatase non-receptor type 13-like isoform X2
AAEL0114	general transcription factor IIE subunit 2	-0.0166075134640474	0.942563346885587	0.986630408149377	NA	aag03022	KXJ75165.1 hypothe	XP_001661672.2general transcription factor IIE subunit 2
AAEL0228	NA	-0.0164095397969155	0.879587866240916	0.969652664055335	00561 ; 00564	aag01100 ; aag	NA	XP_021698645.11-acyl-sn-glycerol-3-phosphate acyltransferase delta
AAEL0062	UDP-glucuronic acid decarboxylase 1	-0.0163489750290265	0.927160074938275	0.982472473084084	NA	aag01100 ; aag	KFB51950.1 AGAP00	XP_021705060.1UDP-glucuronic acid decarboxylase 1
AAEL0135	cullin-1	-0.016182789552666	0.884746813818067	0.970521528629931	NA	aag04141 ; aag	NA	XP_019565215.1 cullin-1-like
AAEL0124	RING finger protein unkempt	-0.0161184745055556	0.909343985382735	0.97691422813284	NA	NA	XP_019551932.1 PRE	XP_021702704.1RING finger protein unkempt isoform X4
AAEL0123	lipase 3	-0.0161064001595674	0.9962434231062	0.999035469616496	NA	NA	KXJ71264.1 hypothe	XP_001662497.2lipase 3
AAEL0205	NA	-0.0160729476444296	0.957215732087386	0.989441920233345	NA	NA	NA	XP_021699670.1origin recognition complex subunit 2
AAEL0046	succinate-semialdehyde dehydrogenase [NADP(+)] GabD	-0.0160321054750326	0.891198295739694	0.972336404299425	NA	aag01100 ; aag	KXJ81994.1 hypothe	XP_001649617.2uncharacterized protein LOC5565255
AAEL0044	tubulin-specific chaperone cofactor E-like protein	-0.0159709520470192	0.888121943110281	0.970996238637524	NA	NA	KXJ82969.1 hypothe	XP_021707615.1tubulin-specific chaperone cofactor E-like protein isoform X1
AAEL0105	ELAV-like protein 2	-0.0159546613110367	0.9147768943537	0.977901601050505	NA	NA	ETN63955.1 ELAV pr	XP_019526635.1 ELAV-like protein 2
AAEL0194	NA	-0.0158649188122563	0.909550306598076	0.97691422813284	NA	aag04140 ; aag	NA	XP_021710693.1protein similar isoform X2
AAEL0250	NA	-0.0158139896819184	0.884134332663659	0.970521528629931	NA	aag04130	NA	XP_021695759.1syntaxin-1A isoform X1
AAEL0013	transmembrane protein 256 homolog	-0.015778762064894	0.90354516537468	0.975494447471861	NA	NA	XP_001238550.1 AG	XP_001653054.1transmembrane protein 256 homolog
AAEL0054	polycomb protein Sfmbt	-0.0157144430658931	0.918609487857163	0.979722358282524	NA	NA	KFB48142.1 AGAP01	XP_021701152.1polycomb protein Sfmbt
AAEL0079	zinc finger protein 791	-0.0156121449542309	0.943306134537332	0.987183333334758	NA	NA	KXJ71222.1 hypothe	XP_021703442.1zinc finger protein 791
AAEL0044	1-phosphatidylinositol 4,5-bisphosphate phosphodiesterase gamma-1	-0.0156028281445501	0.91305518355785	0.977561298149576	00562 ; 04070	aag01100 ; aag	XP_001849443.1 1-p	XP_021710614.11-phosphatidylinositol 4,5-bisphosphate phosphodiesterase gamma-1
AAEL0068	ras-related protein Rab-32	-0.0155713393016639	0.905480454486208	0.975993693196454	NA	NA	ETN63559.1 hypothe	XP_011493357.2serine-rich adhesin for platelets isoform X1
AAEL0136	zinc finger protein 436	-0.0155498300233663	0.952982810027412	0.989291842952044	NA	NA	NA	XP_001663856.2zinc finger protein 436
AAEL0088	xaa-Pro dipeptidase	-0.0155365005172302	0.900197398104356	0.974065297962641	NA	NA	XP_001865452.1 xaa	XP_021708173.1xaao dipeptidase
AAEL0136	eukaryotic translation initiation factor 2 subunit 1	-0.0154770987830304	0.88837397664522	0.970996238637524	NA	aag03013 ; aag	NA	XP_001663853.2eukaryotic translation initiation factor 2 subunit 1
AAEL0122	uncharacterized LOC5580229	-0.0154113786177522	0.904404946227267	0.975769060286471	NA	NA	XP_001863665.1 con	XP_021708204.1uncharacterized protein LOC5580229
AAEL0046	actin	-0.0153707016803383	0.971968563099895	0.992802528468446	NA	NA	KXJ82456.1 hypothe	XP_019532457.1 actin-like
AAEL0069	uncharacterized protein T28D9.3	-0.0153488143077841	0.917737472840331	0.979587080973985	NA	NA	XP_019559027.1 PRE	XP_021700321.1putative phosphatidate phosphatase isoform X1
AAEL0197	NA	-0.015335794533804	0.958101416005025	0.989441920233345	NA	aag04150 ; aag	NA	XP_019558143.1 frizzled-like
AAEL0022	leucine-rich repeat neuronal protein 3	-0.0153195575229205	0.896913247715384	0.973750254467651	NA	NA	KXJ81202.1 hypothe	XP_001661261.1leucine-rich repeat neuronal protein 3
AAEL0004	iron-sulfur cluster assembly 1 homolog, mitochondrial	-0.0152921314689132	0.905594891289655	0.975993693196454	NA	NA	ETN63311.1 hypothe	XP_001656446.1iron-sulfur cluster assembly 1 homolog, mitochondrial
AAEL0084	isochorismatase domain-containing protein 1	-0.0152765594519533	0.924102704446585	0.981740109661555	NA	NA	KFB41223.1 AGAP00	XP_001653279.1isochorismatase domain-containing protein 1
AAEL0003	mitochondrial inner membrane protease ATP23 homolog	-0.0152749005326764	0.943968198806655	0.987296271832165	NA	NA	KXJ72748.1 hypothe	XP_001655524.2mitochondrial inner membrane protease ATP23 homolog
AAEL0020	ubiquitin thioesterase trabid	-0.0152461017321289	0.906329455468783	0.975993693196454	NA	NA	XP_019560493.1 PRE	XP_021711487.1ubiquitin thioesterase trabid isoform X1
AAEL0033	chaoptin	-0.015111507016817	0.964480004400084	0.991092399652587	NA	NA	KXJ76711.1 hypothe	XP_019558700.1 insulin-like growth factor-binding protein complex acid labile subunit
AAEL0105	amphiphysin	-0.0150711161949635	0.911894813128468	0.977278855888727	NA	aag04144	NA	XP_021697186.1amphiphysin isoform X3
AAEL0037	NA	-0.0150526721829571	0.933923429157899	0.98347583693566	NA	NA	KXJ68853.1 hypothe	XP_001664181.2uncharacterized protein LOC5579002
AAEL0027	28S ribosomal protein S22, mitochondrial	-0.0150306907816009	0.907052604239956	0.975993693196454	NA	NA	KXJ68747.1 hypothe	XP_001662176.128S ribosomal protein S22, mitochondrial
AAEL0045	beta-galactosidase	-0.014921568129489	0.93420749700255	0.98347583693566	00531 ; 00600 ; 00604 ;	aag01100 ; aag	XP_019541280.1 PRE	XP_001649402.2beta-galactosidase isoform X1
AAEL0186	NA	-0.0149084592301457	0.925528547564494	0.982258702730868	NA	NA	NA	XP_021693037.1metastasis-associated protein MTA3
AAEL0015	protein transport protein Sec31A	-0.0148528101175257	0.90686504343156	0.975993693196454	NA	aag04141	NA	XP_021709267.1protein transport protein Sec31A isoform X1
AAEL0138	serine/threonine-protein kinase Warts	-0.014770776789955	0.90032351706995	0.974065297962641	04151 ; 05165 ; 04714 ;	aag04391 ; aag	NA	XP_021707878.1serine/threonine-protein kinase Warts isoform X1
AAEL0108	eukaryotic translation initiation factor 2 subunit 3	-0.0147397145310337	0.897879713567738	0.973750254467651	310	aag01100 ; aag	KXJ69742.1 hypothe	XP_021709631.1histone-lysine N-methyltransferase Su(var)3-9 isoform X1
AAEL0073	protein cornichon	-0.0147086469146224	0.920429376124514	0.980340877547081	NA	NA	KXJ80103.1 hypothe	XP_001652686.1protein cornichon
AAEL0046	uncharacterized LOC5565266	-0.0146707284368268	0.90126838899866	0.974380285920978	NA	NA	KXJ69968.1 hypothe	XP_021707995.1uncharacterized protein LOC5565266
AAEL0105	NA	-0.0146511176030754	0.91407081164556	0.977828474226146	NA	NA	NA	XP_001660893.2aminoacyl tRNA synthase complex-interacting multifunctional protein 1
AAEL0215	NA	-0.0146230775420263	0.949790604600123	0.98806088919239	NA	NA	NA	XP_001658335.2elongator complex protein 5
AAEL0000	serine-rich adhesin for platelets	-0.0144847904330152	0.949798010978121	0.98806088919239	NA	NA	NA	XP_021693060.1uncharacterized protein LOC5563615 isoform X2
AAEL0273	NA	-0.0143613000756077	0.956492407531872	0.989441920233345	NA	NA	NA	XP_021711528.1serine-enriched protein isoform X1
AAEL0277	NA	-0.0143344662474845	0.94663866855038	0.987646532539731	NA	NA	NA	NA
AAEL0130	uncharacterized LOC5577106	-0.0143094579688342	0.921145287073961	0.980608390982715	NA	NA	NA	XP_021701928.1uncharacterized protein LOC5577106
AAEL0206	NA	-0.0142509559178419	0.932464328579003	0.98347583693566	NA	NA	NA	XP_021696681.1uncharacterized protein LOC5577556 isoform X2
AAEL0048	LIM domain-containing protein jub	-0.0142469861824984	0.945987796518964	0.987518968073456	NA	aag04391 ; aag	XP_019534015.1 PRE	XP_001649952.2LIM domain-containing protein jub isoform X2
AAEL0071	uncharacterized LOC5568863	-0.014121235201043	0.956124546160096	0.989441920233345	NA	NA	KFB38425.1 AGAP00	KFB38425.1AGAP003660-like protein
AAEL0043	titin homolog	-0.0140354020111746	0.946637401904235	0.987646532539731	NA	NA	KXJ73232.1 hypothe	XP_001648926.2titin homolog
AAEL0267	NA	-0.0139058121964977	0.929091567020078	0.983153242837943	NA	NA	NA	NA
AAEL0047	zinc finger CCCH domain-containing protein 13	-0.0138530104612347	0.923100523193504	0.981343310857761	NA	NA	XP_019553518.1 PRE	XP_001649596.2serine/arginine repetitive matrix protein 2 isoform X2
AAEL0147	NA	-0.0138326470690252	0.903249830748076	0.975437735306161	NA	aag04137	NA	XP_001649625.2FUN14 domain-containing protein 2 isoform X2
AAEL0032	melanization protease 1	-0.0137753680673539	0.911742256396872	0.977278855888727	NA	aag04624	KXJ71591.1 hypothe	XP_001656658.2melanization protease 1
AAEL0079	zinc finger protein 62 homolog	-0.0137614286760018	0.956579212329063	0.989441920233345	NA	NA	NA	XP_021703455.1zinc finger protein 62 homolog
AAEL0269	NA	-0.0137283449924333	0.916097033683937	0.978630706287391	NA	NA	NA	XP_021710418.1patronin isoform X23
AAEL0011	putative polypeptide N-acetylgalactosaminyltransferase 9	-0.0137239432874115	0.933157390148985	0.98347583693566	NA	NA	KXJ84274.1 hypothe	XP_001652408.2putative polypeptide N-acetylgalactosaminyltransferase 9
AAEL0223	NA	-0.0136971326972952	0.930557108510609	0.98347583693566	NA	NA	NA	XP_001653709.2breast carcinoma-amplified sequence 3 homolog isoform X1
AAEL0172	hypoxia up-regulated protein 1	-0.0136503206180859	0.902219857922772	0.974554188590838	NA	aag04141	NA	XP_011493168.2hypoxia up-regulated protein 1
AAEL0044	RRP15-like protein	-0.0136486562026012	0.947705749818908	0.987743607411212	NA	NA	KXJ74675.1 hypothe	XP_001649209.1RRP15-like protein isoform X2
AAEL0196	NA	-0.0134831232236104	0.949968449919511	0.98806088919239	NA	NA	NA	XP_021695117.1death-associated protein kinase related
AAEL0133	thymidylate synthase	-0.0134341770808943	0.934933569557885	0.983679248464631	00240 ; 00670	aag01100 ; aag	NA	XP_001663452.1thymidylate synthase
AAEL0092	feline leukemia virus subgroup C receptor-related protein 2	-0.0134041205520701	0.923394560093002	0.981343310857761	NA	NA	NA	XP_001659806.1feline leukemia virus subgroup C receptor-related protein 2

AAEL00942	protein peste	-0.013330886929196	0.904848970717734	0.975993693196454	NA	NA	NA	XP_001660042.3protein peste
AAEL02023	NA	-0.0132497867322856	0.930907488475868	0.98347583693566	NA	aag03010	NA	XP_001653087.240S ribosomal protein SA
AAEL02161	NA	-0.0132176708386301	0.925729310015305	0.982258702730868	NA	NA	NA	XP_021693310.1saccharopine dehydrogenase-like oxidoreductase
AAEL01816	NA	-0.0131878575630892	0.904367535223224	0.975769060286471	NA	NA	NA	AAX20152.1AMPK-gamma subunit
AAEL02635	NA	-0.0130406774367836	0.930718790076459	0.98347583693566	NA	NA	NA	XP_021706289.1host cell factor isoform X2
AAEL00689	RNA polymerase II degradation factor 1	-0.0130130099812722	0.921115459079536	0.980608390982715	NA	NA	KXJ73954.1 hypothet	XP_001652307.1RNA polymerase II degradation factor 1 isoform X2
AAEL00034	O-acetyl-ADP-ribose deacetylase 1	-0.0129936802091378	0.95467230814264	0.989303184592491	NA	NA	XP_019528773.1 PRE	XP_021697898.1O-acetyl-ADP-ribose deacetylase 1 isoform X1
AAEL01011	calcium uptake protein 1 homolog, mitochondrial	-0.0128116681015231	0.92017426566712	0.980340877547081	NA	NA	NA	XP_001660642.1calcium uptake protein 1 homolog, mitochondrial isoform X2
AAEL02792	NA	-0.0128006097184295	0.973463653411974	0.993357763885677	NA	NA	NA	XP_021703439.1zinc finger protein 432-like
AAEL00048	mitochondrial proton/calcium exchanger protein	-0.0126400857011062	0.910668360022115	0.97691422813284	NA	NA	XP_019532118.1 PRE	XP_001656963.1mitochondrial proton/calcium exchanger protein isoform X1
AAEL00444	importin-9	-0.0126121047345961	0.923151770554363	0.981343310857761	NA	NA	XP_019541175.1 PRE	XP_001649140.1importin-9 isoform X2
AAEL02787	NA	-0.012497830241662	0.931536272236162	0.98347583693566	NA	NA	NA	XP_019529228.1 serine-rich adhesin for platelets-like isoform X3
AAEL00025	protein stoned-A	-0.0124454958973816	0.956870653823086	0.989441920233345	NA	NA	KXJ77547.1 hypothet	XP_001660127.1protein stoned-A
AAEL02640	NA	-0.0123441411019024	0.924729729690665	0.982151774309037	NA	NA	NA	NA
AAEL02053	NA	-0.0122757703985189	0.948506989334032	0.987907336259396	NA	NA	NA	NA
AAEL00497	ATP-dependent RNA helicase vasa	-0.0122224455998216	0.915832620895114	0.978575450241157	NA	NA	KXJ83213.1 hypothet	XP_001650127.1ATP-dependent RNA helicase vasa
AAEL01090	SWI/SNF-related matrix-associated actin-dependent regulator of chromat	-0.0121501298415425	0.947587380415425	0.987743607411212	NA	NA	KXJ75138.1 hypothet	XP_001661139.1SWI/SNF-related matrix-associated actin-dependent regulator of chromatin subfamily E member 1
AAEL00860	39S ribosomal protein L28, mitochondrial	-0.0121002227529296	0.936238278607133	0.98424319806035	NA	aag03010	KXJ68532.1 hypothet	XP_001653335.139S ribosomal protein L28, mitochondrial
AAEL00657	NA	-0.0120115897826252	0.930929663572128	0.98347583693566	NA	NA	XP_019553295.1 PRE	XP_001652075.2venom protease
AAEL00780	putative Dol-P-Glc:Glc(2)Man(9)GlcNAc(2)-PP-Dol alpha-1,2-glucosyltrans	-0.0115720482833292	0.951956726152693	0.988479552477666	510	aag01100 ; aag	KXJ78683.1 hypothet	XP_001652924.1putative DolGlc:Glc(2)Man(9)GlcNAc(2)-Dol alpha-1,2-glucosyltransferase
AAEL00723	splicing factor 45	-0.0115718285714302	0.950753997884593	0.988345943124042	NA	aag03040	KFB45880.1 AGAP00	XP_001658243.1splicing factor 45
AAEL00159	glycerol-3-phosphate dehydrogenase [NAD(+)], cytoplasmic	-0.0114964101953994	0.944999919797062	0.987398325447749	00564 ; 00564	aag00564	XP_001653595.1 glyc	XP_001653595.1glycerol-3-phosphate dehydrogenase [NAD(+)], cytoplasmic isoform X3
AAEL01163	uncharacterized LOC5575079	-0.0113756337260398	0.959326395126588	0.989441920233345	NA	NA	KXJ68119.1 hypothet	XP_001661807.2uncharacterized protein LOC5575079
AAEL01368	calcium and integrin-binding family member 2	-0.0113555357743427	0.930040448623584	0.98347583693566	NA	NA	NA	XP_021697643.1calcium and integrin-binding family member 2
AAEL02472	NA	-0.0113431661939817	0.931271172923036	0.98347583693566	NA	aag03008 ; aag	NA	XP_021712980.15'-3' exoribonuclease 2 homolog
AAEL02365	NA	-0.0112939298756286	0.931961001545188	0.98347583693566	00670 ; 00720	aag01100 ; aag	NA	XP_019525707.1 methylenetetrahydrofolate reductase-like
AAEL01696	uncharacterized LOC23687380	-0.0112251555035099	0.954005084470974	0.989303184592491	NA	NA	NA	XP_001844120.1elongation factor 1-alpha
AAEL00229	sugar transporter ERD6-like 1	-0.0111310119372817	0.9593469666487	0.989441920233345	NA	NA	KXJ70121.1 hypothet	XP_001661245.1sugar transporter ERD6-like 1
AAEL01223	protein max	-0.0110274488839907	0.919564863891459	0.980340877547081	NA	NA	ETN66963.1 bhlhzip	XP_001662341.2protein max
AAEL00468	cell growth regulator with RING finger domain protein 1	-0.0109781858752441	0.944157301893298	0.987296271832165	NA	NA	XP_019549511.1 PRE	XP_021707898.1cell growth regulator with RING finger domain protein 1 isoform X1
AAEL00713	facilitated trehalose transporter Tret1	-0.0109587869728339	0.929266580831965	0.983225373900855	NA	NA	XP_019533127.1 PRE	XP_001658148.2facilitated trehalose transporter Tret1
AAEL00996	apyrase-like	-0.0108466671913612	0.959450796200167	0.989441920233345	NA	NA	NA	XP_019547523.1 apyrase
AAEL01166	39S ribosomal protein L41, mitochondrial	-0.0105820675193601	0.957137681048369	0.989441920233345	NA	NA	ETN59694.1 39S ribo	XP_001661823.139S ribosomal protein L41, mitochondrial
AAEL01484	putative tyrosine-protein kinase Wsck	-0.0105430717388735	0.983530076917832	0.995648379295697	NA	NA	NA	XP_001649753.1putative tyrosine-protein kinase Wsck
AAEL00392	translation factor GUF1 homolog, mitochondrial	-0.0105340181975618	0.963539869983882	0.990998530286399	NA	NA	KXJ75822.1 hypothet	XP_001648056.1translation factor GUF1 homolog, mitochondrial
AAEL01170	diacylglycerol kinase epsilon	-0.0105317507884976	0.962102155511147	0.990224178616807	00564 ; 04070 ; 00561	aag01100 ; aag	KXJ82264.1 hypothet	XP_021697543.1diacylglycerol kinase epsilon
AAEL00504	RNA-binding protein 39	-0.0104630752232042	0.936319085039199	0.98424319806035	NA	NA	XP_001869868.1 spli	XP_021703642.1RNA-binding protein 39
AAEL00479	dipeptidyl aminopeptidase-like protein 6	-0.0104244597616566	0.957977475938185	0.989441920233345	NA	NA	NA	XP_021712707.1dipeptidyl aminopeptidase-like protein 6 isoform X4
AAEL01201	muskelin	-0.0104092999274963	0.955420318568369	0.989441920233345	NA	NA	XP_019541671.1 PRE	XP_001662154.1muskelin isoform X3
AAEL01100	uncharacterized LOC5574236	-0.010357079462614	0.964362487752957	0.991092399652587	NA	NA	XP_001849205.1 con	EAT36959.1AAEL011004
AAEL00960	serine-aspartate repeat-containing protein C	-0.0103129817623917	0.928642759340345	0.98291452348119	NA	NA	NA	XP_019525870.1 protein starmaker isoform X1
AAEL01165	40S ribosomal protein S15	-0.0103070787587085	0.943075074703145	0.987053780416098	NA	aag03010	KXJ82298.1 hypothet	XP_001661827.140S ribosomal protein S15
AAEL00443	transketolase-like protein 2	-0.0102750184785527	0.93356116368116	0.98347583693566	00900 ; 00730	aag01100 ; aag	ETN59360.1 transket	XP_001649141.1transketolase-like protein 2
AAEL02247	NA	-0.0102624188940011	0.950053769965574	0.98806088919239	NA	NA	NA	XP_021703658.1transcription factor YY2-like
AAEL00314	39S ribosomal protein L21, mitochondrial	-0.0101957266793751	0.945306778466983	0.987398325447749	NA	aag03010	KXJ68599.1 hypothet	XP_001656350.239S ribosomal protein L21, mitochondrial
AAEL00201	tetratricopeptide repeat protein 19 homolog, mitochondrial	-0.0101128374672279	0.95630864563358	0.989441920233345	NA	NA	KXJ82916.1 hypothet	XP_001660810.2tetratricopeptide repeat protein 19 homolog, mitochondrial
AAEL02415	NA	-0.00997451623820332	0.951898538244393	0.988479552477666	NA	NA	NA	XP_021697221.1protein FAM46C isoform X3
AAEL02454	NA	-0.00997337044954303	0.930233813142392	0.98347583693566	NA	NA	NA	---NA---
AAEL00509	60S ribosomal protein L13	-0.00996524925447106	0.940839304982462	0.986042559017697	NA	aag03010	CRL03320.1 CLUMA_	XP_021705544.160S ribosomal protein L13
AAEL00428	protein drumstick	-0.00994804826321629	0.971939231015396	0.992802528468446	NA	NA	KXJ80325.1 hypothet	XP_019555922.1 protein drumstick-like
AAEL01128	elongation factor 1-gamma	-0.00994246825039335	0.941204647962574	0.986042559017697	NA	NA	KXJ69323.1 hypothet	XP_001661570.1elongation factor 1-gamma
AAEL00290	26S proteasome non-ATPase regulatory subunit 1	-0.00989931697309596	0.936345531384613	0.98424319806035	NA	aag03050	KXJ67908.1 hypothet	XP_001662672.226S proteasome non-ATPase regulatory subunit 1
AAEL00235	UPF0389 protein CG9231	-0.00986462408517295	0.933809575340806	0.98347583693566	NA	NA	XP_001845741.1 gro	XP_021698908.1UPF0389 protein CG9231
AAEL01105	lysophospholipid acyltransferase 1	-0.00980276109261756	0.927940477306833	0.982649508778163	NA	NA	XP_019549480.1 PRE	XP_021696709.1lysophospholipid acyltransferase 1 isoform X1
AAEL00688	14-3-3 protein zeta	-0.00968923877967733	0.93732598220982	0.984619988774498	NA	aag04013 ; aag	AHJ25930.1 14-3-3 z	XP_001652301.114-3-3 protein zeta isoform X1
AAEL00441	poly(U)-binding-splicing factor half pint	-0.00957197767642286	0.950106412952697	0.98806088919239	NA	aag03040	XP_019542113.1 PRE	XP_001649078.2poly(U)-binding-splicing factor half pint isoform X1
AAEL00023	scavenger receptor class B member 1	-0.00954753972507401	0.934674343499097	0.983518960420662	NA	NA	NA	XP_021699922.1platelet glycoprotein 4 isoform X1
AAEL01961	NA	-0.00954649082151377	0.95724380232696	0.989441920233345	NA	NA	NA	XP_021708482.1mucin-3A isoform X1
AAEL00700	protein bride of sevenless	-0.00947201340527922	0.959813176293076	0.989602335938251	NA	aag04013	KXJ78554.1 hypothet	XP_021710276.1protein bride of sevenless
AAEL01120	xenotropic and polytropic retrovirus receptor 1	-0.0094266276250631	0.948276983516067	0.987881609350457	NA	NA	KFB49009.1 AGAP00	XP_001655172.1xenotropic and polytropic retrovirus receptor 1
AAEL00573	COP9 signalosome complex subunit 2	-0.00941955778846395	0.941813245680421	0.986181780467824	NA	NA	CRL00639.1 CLUMA_	XP_001651398.1COP9 signalosome complex subunit 2
AAEL01464	ubiquitin-conjugating enzyme E2 N	-0.00937056053825837	0.930391878456163	0.98347583693566	NA	aag04120 ; aag	NA	XP_019562464.1 ubiquitin-conjugating enzyme E2 N
AAEL01728	trithorax group protein osa	-0.00930898685820876	0.936079408841004	0.98424319806035	NA	NA	NA	XP_021707721.1trithorax group protein osa isoform X6
AAEL01980	NA	-0.00908446015481912	0.934526485347251	0.98347583693566	NA	NA	NA	XP_001654478.1protein RER1
AAEL01173	transcription factor MafK	-0.00892353171454328	0.960303317795005	0.989916707542076	NA	NA	ETN57761.1 transcrip	XP_019542801.1 transcription factor MafK isoform X1
AAEL00389	facilitated trehalose transporter Tret1	-0.00889928054216755	0.947657105316755	0.987743607411212	NA	NA	KXJ74030.1 hypothet	XP_001664287.1facilitated trehalose transporter Tret1
AAEL00516	dnaJ homolog subfamily A member 1	-0.00885335127099906	0.948645086497224	0.987939348794415	NA	aag04141	XP_019563210.1 PRE	XP_021704640.1dnaJ homolog subfamily A member 1 isoform X3
AAEL00865	ELMO domain-containing protein 2	-0.00885093853997791	0.951219011125994	0.988479552477666	NA	NA	KXJ76286.1 hypothet	XP_001659393.1ELMO domain-containing protein 2
AAEL02339	NA	-0.00882404626759848	0.976653089648986	0.994190331004269	NA	NA	NA	XP_021713128.1uncharacterized protein LOC110681513
AAEL01130	annexin B9	-0.008814391666972	0.935697736463188	0.98424319806035	NA	NA	XP_019554675.1 PRE	XP_001655244.1annexin B9 isoform X9
AAEL01389	protein unc-50 homolog	-0.00876144428002212	0.959025422216214	0.989441920233345	NA	NA	NA	XP_001664104.1protein unc-50 homolog
AAEL00180	chondroitin sulfate proteoglycan 4	-0.00870898903370747	0.957905153415862	0.989441920233345	NA	NA	KXJ80689.1 hypothet	XP_021702429.1chondroitin sulfate proteoglycan 4
AAEL00941	NADH dehydrogenase [ubiquinone] 1 alpha subcomplex subunit 9, mitoc	-0.00844296729323001	0.95070243904552	0.988345943124042	NA	aag01100 ; aag	NA	XP_001660001.1NADH dehydrogenase [ubiquinone] 1 alpha subcomplex subunit 9, mitochondrial
AAEL01251	tumor susceptibility gene 101 protein	-0.00833416408338518	0.954814872352316	0.989303184592491	NA	aag04144	KXJ74490.1 hypothet	XP_001656115.2tumor susceptibility gene 101 protein isoform X1
AAEL00243	NA	-0.00821489629621211	0.973838602430929	0.993377986871678	NA	aag04146	KXJ82848.1 hypothet	XP_001655224.2peroxisomal membrane protein 11C
AAEL00051	acetylcholinesterase	-0.00804011067830602	0.966654347348817	0.991210985301503	NA	NA	BAE71346.1 acetylch	XP_021707023.1acetylcholinesterase isoform X1
AAEL00893	G protein-activated inward rectifier potassium channel 3	-0.00797605939430172	0.963046176499289	0.990715585930292	NA	NA	KXJ73971.1 hypothet	XP_001653528.1G protein-activated inward rectifier potassium channel 3
AAEL02732	NA	-0.00790767436286538	0.96543831942611	0.991092399652587	NA	aag04142	NA	XP_021711460.1ADP-ribosylation factor-binding protein GGA2 isoform X1
AAEL01294	60S ribosomal protein L11	-0.00782157268782874	0.958341294220221	0.989441920233345	NA	aag03010	NA	XP_001663129.160S ribosomal protein L11
AAEL00141	myosin heavy chain, non-muscle	-0.00771607229311045	0.943666281993395	0.987236796220786	NA	NA	XP_019535077.1 PRE	XP_001659192.2myosin heavy chain, non-muscle
AAEL00085	fumarylacetoacetate hydrolase domain-containing protein 2A	-0.00770043791488532	0.952680053555276	0.989119017580464	NA	NA	XP_001869488.1 fun	XP_021706801.1fumarylacetoacetate hydrolase domain-containing protein 2 isoform X1
AAEL00683	delta-1-pyrroline-5-carboxylate synthase	-0.00765258013327041	0.950029814010647	0.98806088919239	00330 ; 00332 ; 00330 ;	aag01100 ; aag	KXJ81895.1 hypothet	XP_001652254.1delta-1-pyrroline-5-carboxylate synthase

AAEL00806	integrator complex subunit 3 homolog	-0.00764135113780706	0.954181879179771	0.989303184592491	NA	NA	XP_015031969.2 inte	XP_021700011.1integrator complex subunit 3 homolog
AAEL00013	RUN domain-containing protein 1	-0.00761941629440588	0.985682889851758	0.995754644700487	NA	NA	KXJ83438.1 hypothet	XP_001657703.1RUN domain-containing protein 1
AAEL00397	RNA polymerase-associated protein Rtf1	-0.00749423708662974	0.945428640888416	0.987398325447749	NA	NA	KXJ77757.1 hypothet	XP_001648109.1RNA polymerase-associated protein Rtf1
AAEL00618	acyl-CoA-binding domain-containing protein 5	-0.00733846849837678	0.954389387781241	0.989303184592491	NA	NA	KXJ82281.1 hypothet	XP_001657570.1acyl-CoA-binding domain-containing protein 5
AAEL02536	NA	-0.00731968768720162	0.953061528773079	0.989291842952044	NA	NA	NA	XP_021694729.1NADPH--cytochrome P450 reductase isoform X1
AAEL01992	NA	-0.0072682047465434	0.966629477982344	0.991210985301503	NA	NA	NA	XP_019530132.1 uncharacterized threonine-rich GPI-anchored glycoprotein PJ4664.02-like
AAEL00584	synaptic vesicle glycoprotein 2C	-0.00726613512280156	0.959454583044755	0.989441920233345	NA	aag04512	ETN67247.1 synaptic	XP_001651533.1synaptic vesicle glycoprotein 2C
AAEL00192	uncharacterized LOC5573003	-0.00724506580107927	0.993196385731787	0.999035469616496	NA	NA	XP_001862863.1 con	XP_001654330.2uncharacterized protein LOC5573003
AAEL00025	serine protease snake	-0.0071867897099004	0.97580391691297	0.994190331004269	NA	NA	NA	XP_001660122.1serine protease snake
AAEL01496	GDP-mannose 4,6 dehydratase	-0.00717513956797725	0.977796896330195	0.994190331004269	00051 ; 00520	aag01100 ; aag	NA	XP_001650108.2GDP-mannose 4,6 dehydratase
AAEL00569	serine incorporator 1	-0.00717059374789836	0.945538260476332	0.987398325447749	NA	NA	ETN58148.1 membra	XP_001651350.1serine incorporator 1 isoform X2
AAEL00564	RING finger and CHY zinc finger domain-containing protein 1	-0.00714884254560107	0.966966454322624	0.991210985301503	NA	aag04120	ETN61932.1 vitelloge	XP_001651215.1RING finger and CHY zinc finger domain-containing protein 1
AAEL01099	long-chain-fatty-acid--CoA ligase 4	-0.00713370840973309	0.950610637921414	0.988345943124042	NA	aag01100 ; aag	XP_019539970.1 PRE	XP_001661275.2long-chain-fatty-acid--CoA ligase 4 isoform X2
AAEL00492	HEAT repeat-containing protein 3	-0.00711576424663382	0.946608438967348	0.987646532539731	NA	NA	KXJ81823.1 hypothet	XP_021700574.1HEAT repeat-containing protein 3
AAEL00438	chorion peroxidase	-0.00706817021672985	0.960705027908544	0.989923653148286	940	NA	KXJ75130.1 hypothet	XP_001649030.2chorion peroxidase
AAEL01972	NA	-0.00703110775980618	0.949908774857945	0.98806088919239	NA	NA	NA	XP_021710944.1single-stranded DNA-binding protein 3 isoform X3
AAEL00906	superkiller viralicidic activity 2-like 2	-0.00693667459211292	0.963644300043762	0.990998530286399	NA	aag03018	KXJ75949.1 hypothet	XP_021700747.1superkiller viralicidic activity 2-like 2
AAEL02540	NA	-0.0067698065467393	0.953663237721408	0.989303184592491	NA	aag00564	NA	XP_021704521.1neuropathy target esterase sws isoform X2
AAEL00387	transcription factor Sp4	-0.00669881623717042	0.964787092706755	0.991092399652587	NA	NA	XP_019533368.1 PRE	XP_021710246.1transcription factor Sp4 isoform X2
AAEL00706	mucin-5B	-0.0066032587912646	0.969031247541212	0.991531930743849	NA	NA	NA	XP_021704230.1mucin-5B
AAEL01201	NA	-0.00659906011194417	0.975298103325095	0.994190331004269	NA	aag04120	ETN61969.1 nedd8-c	XP_001662144.1NEDD8-conjugating enzyme UBE2F
AAEL00600	histidine protein methyltransferase 1 homolog	-0.00659604668832377	0.975759822672811	0.994190331004269	NA	NA	KXJ79129.1 hypothet	XP_001651773.1histidine protein methyltransferase 1 homolog
AAEL00912	probable cytochrome P450 6a14	-0.00656104914187262	0.971609761589053	0.992802528468446	NA	NA	AEB77680.1 cytochro	XP_001653682.1probable cytochrome P450 6a14
AAEL02434	NA	-0.0065096877985447	0.982082456243901	0.995058661347322	NA	NA	NA	XP_021697531.1transmembrane protein 209
AAEL01955	NA	-0.00648463691446795	0.951660775190973	0.988479552477666	NA	NA	NA	XP_021703279.1protein kinase C and casein kinase II substrate protein 3 isoform X2
AAEL00907	NA	-0.00641689006453421	0.954639866852316	0.989303184592491	NA	NA	KXJ84045.1 hypothet	XP_021693885.1la-related protein 1 isoform X1
AAEL00940	protein RMD5 homolog A	-0.00639993977840806	0.959550064786173	0.989441920233345	NA	NA	NA	XP_001660018.1protein RMD5 homolog A isoform X2
AAEL00301	epimerase family protein SDR39U1	-0.00634627437225356	0.95749521672967	0.989441920233345	NA	NA	KXJ82099.1 hypothet	XP_021703495.1epimerase family protein SDR39U1 isoform X1
AAEL01296	uncharacterized LOC5577042	-0.00626153669638279	0.954163736376586	0.989303184592491	NA	NA	NA	XP_019533647.1 double-stranded RNA-specific editase B2-like isoform X4
AAEL02204	NA	-0.00622777991196906	0.962066304067506	0.990224178616807	NA	NA	NA	XP_019556378.1 unconventional myosin-IXb-like isoform X5
AAEL00591	multidrug resistance-associated protein 4	-0.00613804364018076	0.965762418405741	0.991210985301503	NA	NA	XP_001861842.1 mu	XP_001651693.2multidrug resistance-associated protein 4
AAEL01373	electron transfer flavoprotein subunit alpha, mitochondrial	-0.00613049662250065	0.954900117968975	0.989303184592491	NA	NA	NA	XP_001663924.1electron transfer flavoprotein subunit alpha, mitochondrial
AAEL01036	polycomb protein Asx	-0.0060268907396167	0.972322264313956	0.992802528468446	NA	NA	NA	XP_021706586.1polycomb protein Asx
AAEL01069	5-formyltetrahydrofolate cyclo-ligase	-0.00595770124865292	0.968188161846535	0.991220636062002	NA	aag01100 ; aag	KXJ75227.1 hypothet	XP_001654754.15-formyltetrahydrofolate cyclo-ligase
AAEL00617	proteasome subunit beta type-3	-0.00595289115352187	0.960551800171856	0.989920222329647	NA	aag03050	KFB35883.1 hypothe	XP_001651824.1proteasome subunit beta type-3
AAEL00137	cleavage and polyadenylation specificity factor subunit 5	-0.00581116110729195	0.975596785764348	0.994190331004269	NA	aag03015	XP_019533445.1 PRE	XP_001653167.1cleavage and polyadenylation specificity factor subunit 5
AAEL01110	NA	-0.00575710229593473	0.960841068972243	0.989923653148286	NA	NA	XP_019535613.1 PRE	XP_001661417.2protein hu-li tai shao isoform X2
AAEL00769	solute carrier organic anion transporter family member 3A1	-0.00569561239946795	0.967923073294095	0.991220636062002	NA	NA	KXJ84223.1 hypothet	XP_021700473.1solute carrier organic anion transporter family member 3A1
AAEL02818	NA	-0.00555466668349942	0.977776187671245	0.994190331004269	NA	NA	NA	XP_021697568.1serine/arginine repetitive matrix protein 1-like
AAEL01237	general odorant-binding protein 72	-0.00539952348199195	0.972411344173669	0.992802528468446	NA	NA	AGI04325.1 obp55 [A	XP_001655977.1general odorant-binding protein 72
AAEL00737	uncharacterized LOC5569107	-0.00539778158182452	0.965458842695695	0.991092399652587	NA	NA	NA	XP_021700167.1uncharacterized protein LOC5569107
AAEL00618	eIF-2-alpha kinase activator GCN1	-0.00532462489895584	0.9666836871679	0.991210985301503	NA	NA	KXJ70533.1 hypothet	XP_001651826.2eIF-2-alpha kinase activator GCN1
AAEL01187	ubiquitin-conjugating enzyme E2 variant 1	-0.00513404113535078	0.961372476941386	0.989989876098325	NA	aag04624	CRK92682.1 CLUMA_	XP_021699132.1ubiquitin-conjugating enzyme E2 variant 1
AAEL00924	coronin-7	-0.00513345228202903	0.979624166290473	0.994485178621519	NA	NA	NA	XP_021695075.1coronin-7 isoform X1
AAEL01946	NA	-0.00500856049963806	0.976494830851177	0.994190331004269	NA	aag04391 ; aag	NA	XP_021701909.1protein mothers against dpp isoform X1
AAEL01105	protein tumorous imaginal discs, mitochondrial	-0.00498096449112565	0.962963199090121	0.990715585930292	NA	NA	XP_019549477.1 PRE	XP_001661333.1protein tumorous imaginal discs, mitochondrial isoform X2
AAEL01290	rab GDP dissociation inhibitor alpha	-0.00497011495037185	0.965231456117519	0.991092399652587	NA	NA	NA	XP_001663045.1rab GDP dissociation inhibitor alpha
AAEL01284	mitochondrial import inner membrane translocase subunit TIM44	-0.00492102582817754	0.972214141346211	0.992802528468446	NA	NA	NA	XP_001662961.1mitochondrial import inner membrane translocase subunit TIM44
AAEL01826	NA	-0.00491497400895602	0.965144415248579	0.991092399652587	NA	aag02010	NA	XP_021697387.1probable multidrug resistance-associated protein lethal(2)03659
AAEL01182	UPF0668 protein C10orf76 homolog	-0.00490041079017108	0.973999390305766	0.993431876479014	NA	NA	NP_649841.1 CG837	XP_001661944.2UPF0668 protein C10orf76 homolog
AAEL00394	WD repeat-containing protein 43	-0.00479293169631576	0.972329376175354	0.992802528468446	NA	aag03008	KXJ69536.1 hypothet	XP_001648132.1WD repeat-containing protein 43
AAEL02699	NA	-0.00469306750063125	0.976128032236521	0.994190331004269	NA	aag03018	NA	XP_021696919.1PAB-dependent poly(A)-specific ribonuclease subunit PAN3
AAEL00406	anaphase-promoting complex subunit 1	-0.00461738622722161	0.98253899306167	0.995302049114211	NA	aag04120	KXJ77493.1 hypothet	XP_021703880.1anaphase-promoting complex subunit 1
AAEL00263	probable cytochrome P450 9f2	-0.0045970601102084	0.96636733331388	0.991210985301503	NA	NA	KXJ83348.1 hypothet	XP_001661969.1probable cytochrome P450 9f2
AAEL00702	probable cytochrome P450 28a5	-0.00453866672198026	0.978294464380392	0.994190331004269	NA	NA	NA	XP_001652488.1probable cytochrome P450 28a5
AAEL01812	NA	-0.00452412284051796	0.980244929290238	0.99449893454789	NA	NA	NA	XP_021707578.1calpain-D isoform X1
AAEL00508	uncharacterized protein C14orf119	-0.00436500486217327	0.983675178099718	0.995685731918778	NA	NA	KXJ78895.1 hypothet	XP_001650299.2uncharacterized protein C14orf119
AAEL00233	mannose-6-phosphate isomerase	-0.0043334703912227	0.976987100569761	0.994190331004269	00051 ; 00520	aag01100 ; aag	KXJ77907.1 hypothet	XP_001661364.1mannose-6-phosphate isomerase
AAEL02674	NA	-0.00430923509737599	0.987706269219401	0.996578609724835	73	NA	NA	XP_021708477.1fatty acyl-CoA reductase wat
AAEL00350	ras-related protein rab7	-0.00406923551435572	0.970449128432514	0.992120084416842	NA	aag04144 ; aag	ABL74414.1 Rab7 [A.	XP_001663804.1ras-related protein rab7
AAEL00306	glucose-6-phosphate exchanger SLC37A2	-0.00406061754852731	0.97460208859099	0.993683129891922	NA	NA	KXJ82682.1 hypothet	XP_021699574.1glucose-6-phosphate exchanger SLC37A2 isoform X2
AAEL00067	G2/mitotic-specific cyclin-A	-0.00389051412381594	0.979256294285287	0.994485178621519	NA	NA	ETN63235.1 cyclin a	XP_001649769.1G2/mitotic-specific cyclin-A
AAEL00046	E3 ubiquitin-protein ligase KCMF1	-0.00382776875827816	0.9735627412226	0.993357763885677	NA	NA	KXJ74124.1 hypothet	XP_001656420.2E3 ubiquitin-protein ligase KCMF1
AAEL01149	protein THEM6-like	-0.00382613339559145	0.983863317370933	0.995692124835154	NA	NA	KXJ81304.1 hypothet	XP_021712349.1protein THEM6
AAEL01352	U1 small nuclear ribonucleoprotein C	-0.00378439138468602	0.980342000493523	0.99449893454789	NA	aag03040	NA	XP_001663717.1U1 small nuclear ribonucleoprotein C
AAEL01207	pyruvate dehydrogenase phosphatase regulatory subunit, mitochondrial	-0.00369151178286476	0.978186620954075	0.994190331004269	00670 ; 00260	NA	NA	XP_001662223.2pyruvate dehydrogenase phosphatase regulatory subunit, mitochondrial isoform X2
AAEL00230	protein TIS11	-0.00366262309694432	0.976169929426195	0.994190331004269	NA	NA	XP_019563336.1 PRE	XP_021707483.1protein TIS11
AAEL00250	26S proteasome regulatory subunit 6A-B	-0.0034505987973505	0.976861497043688	0.994190331004269	NA	aag03050	CRK91871.1 CLUMA_	XP_001655453.126S proteasome regulatory subunit 6A-B
AAEL00827	zinc finger protein 714	-0.0034167467834316	0.989328905853641	0.997058916995799	NA	NA	XP_019530555.1 PRE	XP_021710435.1zinc finger protein 93 isoform X2
AAEL02810	NA	-0.00340341690115683	0.981895720397075	0.994979013224742	NA	aag04144	NA	XP_021696032.1vacuolar protein sorting-associated protein 35 isoform X1
AAEL01473	homocysteine-responsive endoplasmic reticulum-resident ubiquitin-like c	-0.00337054335293617	0.985721077484863	0.995754644700487	NA	NA	NA	XP_001649465.2homocysteine-responsive endoplasmic reticulum-resident ubiquitin-like domain member 2 protein isoform X1
AAEL00160	ras-GEF domain-containing family member 1B	-0.00330817062413102	0.985113659484269	0.995717914606134	NA	NA	XP_019558810.1 PRE	XP_021704282.1ras-GEF domain-containing family member 1B isoform X2
AAEL00880	uncharacterized protein C1orf43 homolog	-0.00325477373767899	0.982359113270984	0.995229391205504	NA	NA	NA	XP_001659508.2uncharacterized protein C1orf43 homolog isoform X2
AAEL02005	NA	-0.00325240554152854	0.978921017002475	0.99426392918949	NA	NA	NA	XP_019537160.2 protein scylla isoform X1
AAEL00552	putative adenosylhomocysteinase 3	-0.00315559678515799	0.977547368052263	0.994190331004269	270	aag01100 ; aag	ETN63843.1 adenosy	XP_001651051.1putative adenosylhomocysteinase 3 isoform X1
AAEL01731	heat shock 70 kDa protein 4	-0.00313610693751544	0.978731304423629	0.99426392918949	NA	aag04141	NA	XP_021708353.1heat shock 70 kDa protein 4 isoform X2
AAEL01943	NA	-0.00299620359705698	0.987952692032642	0.996578609724835	NA	NA	NA	XP_021695540.1sensory neuron membrane protein 2 isoform X1
AAEL02610	NA	-0.00291725237763082	0.989710801579652	0.997058916995799	NA	NA	NA	XP_021708548.1THAP domain-containing protein 4-like isoform X4
AAEL01372	polypyrimidine tract-binding protein 2	-0.00284089620234798	0.988278289665298	0.996726964215264	NA	NA	NA	XP_021713303.1polypyrimidine tract-binding protein 2 isoform X3
AAEL01114	integrator complex subunit 2	-0.00281179198028464	0.988022892471166	0.996578609724835	NA	NA	KXJ79094.1 hypothet	XP_021711347.1integrator complex subunit 2
AAEL01729	translation machinery-associated protein 7 homolog	-0.0027638264765918	0.980785203248715	0.994557508631324	NA	NA	NA	XP_011493391.1translation machinery-associated protein 7 homolog
AAEL00777	60S ribosomal protein L22	-0.00270186747828845	0.985669927490747	0.995754644700487	NA	aag03010	ACJ74463.1 cytosolic	XP_011492944.160S ribosomal protein L22

AAEL0128	transmembrane protein 94	-0.0026554208197207	0.985907473143462	0.995754644700487	NA	NA	NA	XP_021695162.1transmembrane protein 94 isoform X3
AAEL0035	F-box-like/WD repeat-containing protein TBL1XR1	-0.00259954462998335	0.988845521099541	0.997058916995799	NA	aag04013 ; aag	ETN64525.1 wd-repe	XP_001656940.1F-box-like/WD repeat-containing protein TBL1XR1
AAEL0057	lysosomal alpha-mannosidase	-0.00250542672948277	0.984900748720582	0.995717914606134	511	aag04142 ; aag	KXJ74904.1 hypothet	XP_001651410.1lysosomal alpha-mannosidase
AAEL0008	60S ribosomal protein L35a	-0.00247663275977734	0.986147829773397	0.995888095870807	NA	aag03010	ETN62742.1 60S ribo	XP_001651256.160S ribosomal protein L35a
AAEL0021	transcriptional adapter 2B	-0.00241009538576599	0.986829040417092	0.996195840265819	NA	NA	KXJ72032.1 hypothet	XP_021694314.1transcriptional adapter 2B
AAEL0026	non-specific lipid-transfer protein	-0.00237935875602781	0.984407156189283	0.995692124835154	NA	aag01100 ; aag	XP_001846129.1 nor	XP_001662127.2non-specific lipid-transfer protein
AAEL0004	SAM50-like protein CG7639	-0.00237646795516606	0.98887428427759	0.997058916995799	NA	NA	KXJ68887.1 hypothet	XP_021706269.1SAM50-like protein CG7639
AAEL0104	ras-related protein Rab-4B	-0.00230433827226745	0.985669027848074	0.995754644700487	NA	NA	NA	XP_001660824.1ras-related protein Rab-4B
AAEL0134	cingulin	-0.00223348116893258	0.995892429980456	0.999035469616496	NA	NA	NA	XP_019541225.1 cingulin-like
AAEL0090	putative serine protease F56F10.1	-0.00220426936252968	0.986470423438427	0.996104557937057	NA	NA	KXJ70163.1 hypothet	XP_021697410.1putative serine protease F56F10.1
AAEL0108	NA	-0.00218441785240433	0.984018921342045	0.995692124835154	NA	NA	NA	XP_021707119.1probable WRKY transcription factor protein 1 isoform X2
AAEL0022	5'-AMP-activated protein kinase subunit beta-1	-0.00215071415692497	0.984905489734892	0.995717914606134	NA	aag04068 ; aag	KXJ74955.1 hypothet	XP_001654986.25'-AMP-activated protein kinase subunit beta-1
AAEL0067	protein TSSC1	-0.00193830105650049	0.993773310032545	0.999035469616496	NA	NA	ETN64279.1 TSSC1 [A	XP_001652176.1protein TSSC1
AAEL0085	G patch domain-containing protein 1 homolog	-0.00174537813329475	0.99360281378227	0.999035469616496	NA	NA	KXJ76573.1 hypothet	XP_021709306.1G patch domain-containing protein 1 homolog
AAEL0035	E3 UFM1-protein ligase 1 homolog	-0.00168931197189074	0.992013317766098	0.99841480368409	NA	NA	KXJ80505.1 hypothet	XP_001656941.2E3 UFM1-protein ligase 1 homolog
AAEL0001	probable cleavage and polyadenylation specificity factor subunit 2	-0.00148448634368302	0.993024357052312	0.999035469616496	NA	aag03015	ETN63991.1 cleavage	XP_021712246.1probable cleavage and polyadenylation specificity factor subunit 2
AAEL0078	NA	-0.0013897607502762	0.989742109502109	0.997058916995799	NA	NA	XP_019563402.1 PRE	XP_001653981.2upstream-binding protein 1 isoform X2
AAEL0199	NA	-0.00133831813669957	0.994344400905005	0.999035469616496	NA	NA	NA	XP_019549514.1 sex determination protein fox-1-like isoform X3
AAEL0205	NA	-0.0011734199343098	0.99521643249109	0.999035469616496	NA	NA	NA	XP_021709692.1furin-like protease 1
AAEL0194	NA	-0.00102214862329923	0.996928741539372	0.999035469616496	NA	NA	NA	XP_021701621.1uncharacterized protein LOC5564190
AAEL0011	adenosine kinase	-0.00101380315814318	0.994300417659903	0.999035469616496	230	aag01100 ; aag	KXJ73934.1 hypothet	XP_001652432.2adenosine kinase
AAEL0114	60S ribosomal protein L14	-0.000982954150194059	0.994860502101803	0.999035469616496	NA	aag03010	KXJ71484.1 hypothet	XP_021696259.160S ribosomal protein L14
AAEL0007	synaptotagmin 1	-0.000858787050888284	0.995086705554019	0.999035469616496	NA	NA	XP_019561055.1 PRE	XP_021703825.1synaptotagmin 1 isoform X3
AAEL0132	protein aubergine	-0.000767280942188293	0.994570094371416	0.999035469616496	NA	NA	NA	XP_021707306.1protein aubergine
AAEL0034	proteasome subunit beta type-7	-0.000692307348234844	0.995601024686739	0.999035469616496	NA	aag03050	KXJ82808.1 hypothet	XP_001656819.1proteasome subunit beta type-7
AAEL0236	NA	-0.000664882190236232	0.998048946567567	0.999569700295467	NA	NA	NA	XP_021697301.1WD repeat, SAM and U-box domain-containing protein 1-like isoform X1
AAEL0019	28 kDa heat- and acid-stable phosphoprotein	-0.000630849341971169	0.995396568101201	0.999035469616496	NA	NA	KXJ78704.1 hypothet	XP_001654319.128 kDa heat- and acid-stable phosphoprotein
AAEL0281	NA	-0.000604097804172751	0.996372697249881	0.999035469616496	NA	NA	NA	XP_021696446.1uncharacterized protein LOC5570533
AAEL0180	NA	-0.000581786589832851	0.995852303192755	0.999035469616496	NA	NA	NA	XP_021700278.1uncharacterized protein KIAA1109 isoform X5
AAEL0024	V-type proton ATPase subunit F	-0.000579367928374839	0.996423517544035	0.999035469616496	NA	aag01100 ; aag	ETN63992.1 vacuolar	XP_001655376.1V-type proton ATPase subunit F
AAEL0027	homeotic protein ocelliless	-0.000576316429726534	0.998994218389153	0.999716356318826	NA	NA	XP_019542132.1 PRE	XP_021703670.1homeotic protein ocelliless isoform X2
AAEL0002	sodium- and chloride-dependent glycine transporter 2	-0.000536824338819758	0.999035044578142	0.999716356318826	NA	NA	KXJ81836.1 hypothet	XP_001654776.2sodium- and chloride-dependent glycine transporter 2
AAEL0030	myotubularin-related protein 8	-0.000471579003782502	0.996923312823311	0.999035469616496	04070 ; 00562	aag01100 ; aag	XP_019543592.1 PRE	XP_001663101.2myotubularin-related protein 8 isoform X2
AAEL0035	serine--pyruvate aminotransferase	-0.00044694098694434	0.997267942850478	0.999124090292425	440	NA	KXJ69417.1 hypothet	XP_001656924.2serine--pyruvate aminotransferase
AAEL0135	NA	-0.000375661609598647	0.998604886028378	0.999716356318826	NA	NA	NA	XP_021697046.1calcium uniporter protein, mitochondrial
AAEL0119	zinc finger protein 620	-0.000318606584781329	0.999103231954955	0.999716356318826	NA	NA	KXJ71669.1 hypothet	XP_021705370.1zinc finger protein 620
AAEL0000	histone H4 transcription factor	-0.000310018561613669	0.999173150300396	0.999716356318826	NA	NA	XP_001867230.1 con	XP_021705403.1histone H4 transcription factor
AAEL0234	NA	-0.000288576940037485	0.998458810466999	0.999716356318826	NA	NA	NA	XP_021698152.1zinc finger protein 2 isoform X4
AAEL0098	nucleolar GTP-binding protein 1	-0.000246307853433423	0.998614126798524	0.999716356318826	NA	aag03008	NA	XP_001654061.2nucleolar GTP-binding protein 1
AAEL0044	NA	-0.000175677893161601	0.998738255770886	0.999716356318826	NA	aag04214 ; aag	KXJ70328.1 hypothet	XP_001649226.2transmembrane GTPase Marf isoform X2
AAEL0145	protein-glucosylgalactosylhydroxylysine glucosidase	-0.0001118333595596	0.999991536502291	0.999991536502291	NA	NA	NA	XP_021701920.1protein-glucosylgalactosylhydroxylysine glucosidase isoform X1
AAEL0249	NA	-6,04E+09	0.999586378764374	0.999864905417492	NA	aag04144 ; aag	NA	XP_021697527.1E3 ubiquitin-protein ligase Nedd-4 isoform X1
AAEL0126	probable ribosome biogenesis protein RLP24	-5,92E+09	0.999647590723856	0.999864905417492	NA	aag03010	ETN58510.1 ribosom	XP_001656187.1probable ribosome biogenesis protein RLP24
AAEL0147	lysine--tRNA ligase	-5,04E+08	0.999640867540021	0.999864905417492	970	aag00970	NA	XP_021697661.1lysine--tRNA ligase isoform X2
AAEL0117	actin-related protein 2	-1,99E+09	0.999916291332243	0.999991536502291	NA	NA	KFB38107.1 AGAP00	XP_001655658.1actin-related protein 2 isoform X2
AAEL0028	uncharacterized LOC5576238	0.000218969639829601	0.999103297244426	0.999716356318826	NA	NA	KXJ78077.1 hypothet	XP_001656016.2uncharacterized protein LOC5576238
AAEL0182	NA	0.000432807238803698	0.997278283996514	0.999124090292425	NA	NA	NA	XP_021702294.1cysteine/serine-rich nuclear protein 1
AAEL0004	T-complex protein 1 subunit eta	0.000488321734141861	0.99668359656437	0.999035469616496	NA	NA	KFB42366.1 AGAP00	XP_001656981.1T-complex protein 1 subunit eta
AAEL0139	39S ribosomal protein L20, mitochondrial	0.000528986663016912	0.996972692619896	0.999035469616496	NA	aag03010	NA	XP_001664205.139S ribosomal protein L20, mitochondrial
AAEL0014	E3 ubiquitin-protein ligase SHPRH	0.000566250683759593	0.997865370501861	0.999494627120728	NA	NA	KXJ78401.1 hypothet	XP_001659181.2E3 ubiquitin-protein ligase SHPRH
AAEL0196	NA	0.000586849235467373	0.99687523705175	0.999035469616496	NA	NA	NA	XP_021702392.1leucine-rich repeats and immunoglobulin-like domains protein 3 isoform X2
AAEL0217	NA	0.000597869732993917	0.996052735676297	0.999035469616496	NA	NA	NA	XP_021699402.1COP9 signalosome complex subunit 1b
AAEL0091	mitochondrial inner membrane protein COX18	0.000599394120606812	0.996489653774687	0.999035469616496	NA	NA	KXJ84165.1 hypothet	XP_021697327.1mitochondrial inner membrane protein COX18
AAEL0004	GRAM domain-containing protein 2B	0.000638811538350162	0.995892224388066	0.999035469616496	NA	NA	NA	XP_021707269.1GRAM domain-containing protein 2B isoform X2
AAEL0182	NA	0.000652051457217218	0.99779896513338	0.999494627120728	NA	NA	NA	XP_001661437.2ubiquitin carboxyl-terminal hydrolase 45
AAEL0073	methyltransferase-like protein 9	0.000936782936848571	0.996179870121142	0.999035469616496	NA	NA	XP_011179493.1 PRE	XP_021704304.1methyltransferase-like protein 9 isoform X2
AAEL0214	NA	0.000974544698338818	0.996087323500232	0.999035469616496	NA	aag04320	NA	XP_021702165.1protein spire isoform X4
AAEL0258	NA	0.00104186250624161	0.995195589612063	0.999035469616496	NA	NA	NA	XP_021701706.1uncharacterized protein LOC5577187
AAEL0101	sodium/potassium-transporting ATPase subunit beta-2	0.00104708884499844	0.992696925859977	0.998884320586497	NA	NA	NA	XP_001654264.1sodium/potassium-transporting ATPase subunit beta-2
AAEL0111	serine/threonine-protein kinase rio2	0.00112887406600117	0.994879866181454	0.999035469616496	04151 ; 05165 ; 04714 ;	aag03008	KXJ69732.1 hypothet	XP_001655107.1serine/threonine-protein kinase rio2
AAEL0059	single-pass membrane and coiled-coil domain-containing protein 4 homo	0.00113013101808182	0.994919301513595	0.999035469616496	NA	NA	KFB34831.1 AGAP00	XP_001651591.1single-pass membrane and coiled-coil domain-containing protein 4 homolog
AAEL0047	methionine aminopeptidase 2	0.00120612300280942	0.990879292868898	0.997774694479122	NA	NA	KFB45569.1 hypothe	XP_001649726.1methionine aminopeptidase 2
AAEL0123	IDLSRF-like peptide	0.00125769487199304	0.994382185537601	0.999035469616496	NA	NA	ETN67141.1 Prohorn	XP_001662506.1IDLSRF-like peptide
AAEL0134	nucleolar protein 10	0.00134416936182029	0.994309402891074	0.999035469616496	NA	NA	NA	XP_001656717.2nucleolar protein 10
AAEL0117	GPN-loop GTPase 2	0.00142716473678266	0.996224509970815	0.999035469616496	NA	NA	KFB35001.1 AGAP00	XP_001661906.1GPN-loop GTPase 2
AAEL0125	60S ribosomal protein L7	0.00153579822636968	0.991028769248146	0.997774694479122	NA	aag03010	ETN65433.1 60S ribo	XP_001656140.160S ribosomal protein L7
AAEL0119	myosin-IB	0.00154117440296022	0.989674750291224	0.997058916995799	NA	NA	XP_019538866.1 PRE	XP_021708644.1myosin-IB isoform X2
AAEL0079	CWF19-like protein 2	0.00159669380183972	0.99475205723503	0.999035469616496	NA	NA	KXJ74146.1 hypothet	XP_001658727.1CWF19-like protein 2
AAEL0041	60S ribosomal protein L29	0.00168176184494209	0.991160452318371	0.997774694479122	NA	aag03010	XP_001846341.1 60S	XP_001648594.160S ribosomal protein L29
AAEL0074	F-box/LRR-repeat protein 20	0.0016957193953415	0.99113294051929	0.997774694479122	NA	NA	KXJ70748.1 hypothet	XP_021701053.1F-box/LRR-repeat protein 20
AAEL0269	NA	0.00180620854124927	0.987280046493696	0.996375409940227	NA	NA	NA	XP_021707478.1C-myc promoter-binding protein isoform X2
AAEL0135	WD repeat-containing protein 7	0.00183738022629587	0.987053055435807	0.996255590229275	NA	NA	NA	XP_021700396.1WD repeat-containing protein 7 isoform X3
AAEL0097	homeobox protein abdominal-A homolog	0.00189965260272232	0.991351423274513	0.99785777696041	NA	NA	NA	NP_001345961.1homeobox protein abdominal-A homolog
AAEL0014	hydroxysteroid dehydrogenase-like protein 2	0.00196676249876799	0.994728499042757	0.999035469616496	NA	NA	KXJ71511.1 hypothet	XP_001659208.2hydroxysteroid dehydrogenase-like protein 2
AAEL0143	adrenodoxin	0.00201366193617407	0.985368376958256	0.995754644700487	NA	NA	NA	XP_019560972.1 adrenodoxin-like
AAEL0105	valacyclovir hydrolase	0.00202910311717163	0.993096116633404	0.999035469616496	NA	NA	KXJ76479.1 hypothet	XP_021693961.1valacyclovir hydrolase
AAEL0102	BUD13 homolog	0.00211490691498165	0.995238177132998	0.999035469616496	NA	NA	NA	XP_001654397.1BUD13 homolog
AAEL0005	myotubularin-related protein 3	0.00213985275767056	0.984438921076965	0.995692124835154	NA	aag01100 ; aag	NA	XP_001648676.2myotubularin-related protein 3 isoform X4
AAEL0217	NA	0.00232412353914873	0.987446715907952	0.99643433268834	NA	NA	NA	XP_021700330.1peptidylprolyl isomerase domain and WD repeat-containing protein 1 isoform X1
AAEL0245	NA	0.00249949387049112	0.988956621596363	0.997058916995799	NA	NA	NA	XP_021699069.1G-protein coupled receptor Mth2
AAEL0120	TM2 domain-containing protein CG10795	0.00254940163848975	0.992551748441878	0.998847461631908	NA	NA	XP_001865009.1 TM	XP_001655841.2 TM2 domain-containing protein CG10795
AAEL0066	ankyrin repeat domain-containing protein 13C	0.00262406325555394	0.985099792534568	0.995717914606134	NA	NA	XP_019535751.1 PRE	XP_021701564.1ankyrin repeat domain-containing protein 13C isoform X1

AAEL0120(	sodium/potassium-transporting ATPase subunit alpha	0.00265977855009475	0.983346155207112	0.995614477295785	NA	NA	NA	XP_021693479.1sodium/potassium-transporting ATPase subunit alpha isoform X1
AAEL0209!	NA	0.00266115808231567	0.980355557750158	0.99449893454789	NA	NA	NA	XP_019553800.1 transcription factor HIVEP3 isoform X2
AAEL0108(	ADP,ATP carrier protein 2	0.00271466250149621	0.986842187578808	0.996195840265819	NA	NA	KFB38207.1 hypothe	XP_021713281.1ADP,ATP carrier protein 2
AAEL0015:	protein yippee-like CG15309	0.0027472000685636	0.981775742582571	0.994967002560002	NA	NA	ETN65289.1 fad nad	XP_001659550.1protein yippee-like CG15309 isoform X1
AAEL0269:	NA	0.00276197346871288	0.980249993501983	0.99449893454789	NA	aag04142 ; aag	NA	XP_019553531.1 beta-mannosidase-like
AAEL0029:	probable cytosolic iron-sulfur protein assembly protein Ciao1	0.00288866660064997	0.985879643487411	0.995754644700487	NA	NA	KXJ78870.1 hypothe	XP_001662680.2probable cytosolic iron-sulfur protein assembly protein Ciao1
AAEL0022(	decaprenyl-diphosphate synthase subunit 1	0.00293831988651996	0.980831274813004	0.994557508631324	NA	aag00900	KFB39644.1 AGAP00	XP_021694884.1decaprenyl-diphosphate synthase subunit 1
AAEL0042(	igLON family member 5	0.00298624784579021	0.980449286483205	0.99449893454789	NA	NA	XP_019542861.1 PRE	XP_021708440.1protein amalgam isoform X1
AAEL0004:	ATP-binding cassette sub-family B member 10, mitochondrial	0.00298953254098459	0.984086658781631	0.995692124835154	NA	aag02010	KXJ80823.1 hypothe	XP_001656464.2ATP-binding cassette sub-family B member 10, mitochondrial
AAEL0246(	NA	0.00301518806688066	0.989799305233278	0.997058916995799	NA	NA	NA	XP_021713205.1uncharacterized protein LOC23687519
AAEL0119:	lipase maturation factor 2	0.00311059310175944	0.983243210965065	0.995614477295785	NA	NA	KXJ79463.1 hypothe	XP_021705363.1lipase maturation factor 2
AAEL0237:	NA	0.00317903789064673	0.9911053783849	0.997774694479122	NA	NA	NA	NA
AAEL0194(	NA	0.00320917948190008	0.977660817133377	0.994190331004269	NA	NA	NA	XP_021702806.1growth hormone-inducible transmembrane protein
AAEL0049(	phosphoglycerate kinase	0.00324689641651191	0.984223936520111	0.995692124835154	00010 ; 00710	aag01100 ; aag	XP_019544436.1 PRE	XP_001650133.1phosphoglycerate kinase
AAEL0228:	NA	0.00328158482680195	0.976935568821109	0.994190331004269	NA	aag04144 ; aag	NA	XP_021710115.1clathrin heavy chain isoform X1
AAEL0260:	NA	0.00345170202520938	0.989621613492473	0.997058916995799	NA	NA	NA	NA
AAEL0125:	wiskott-Aldrich syndrome protein family member 2	0.00357845669428536	0.984565355619014	0.995710561864619	NA	NA	KXJ78584.1 hypothe	XP_001662630.2wiskott-Aldrich syndrome protein family member 2
AAEL0049:	uncharacterized LOC5565742	0.00364905425095885	0.989780997662054	0.997058916995799	NA	NA	KXJ67934.1 hypothe	XP_001650082.2uncharacterized protein LOC5565742
AAEL0195:	NA	0.00368045344914498	0.983388392104041	0.995614477295785	NA	NA	NA	XP_021701531.1histone demethylase UTY isoform X1
AAEL0109(	importin subunit alpha-6	0.00376362426906002	0.973602876818617	0.993357763885677	NA	NA	KFB47017.1 AGAP00	XP_021697593.1importin subunit alpha-6
AAEL0021(	tRNA-dihydrouridine(16/17) synthase [NAD(P)(+)]-like	0.00379902168948272	0.977215941283596	0.994190331004269	NA	NA	KXJ69942.1 hypothe	XP_021703402.1tRNA-dihydrouridine(16/17) synthase [NAD(P)(+)]-like
AAEL0280:	NA	0.00385115047009707	0.978283733311589	0.994190331004269	NA	aag03010	NA	XP_021696321.160S ribosomal protein L28
AAEL0010(	nuclear RNA export factor 1	0.00389651276038365	0.974353777740678	0.993573078764375	NA	aag03013 ; aag	KXJ71205.1 hypothe	XP_001657723.1nuclear RNA export factor 1
AAEL0217:	NA	0.00390964828717691	0.974157197974745	0.993482714813655	NA	NA	NA	XP_021699637.1 dnaJ homolog subfamily C member 13
AAEL0195:	NA	0.00397037179659423	0.979787288565821	0.994485178621519	4660	aag04013	NA	XP_021710428.1tyrosine-protein kinase shark isoform X2
AAEL0120:	V-type proton ATPase subunit E	0.00399206033141462	0.972850155140492	0.993140351409231	NA	aag01100 ; aag	ETN59925.1 vacuolar	XP_001655825.1V-type proton ATPase subunit E
AAEL0047:	very long-chain specific acyl-CoA dehydrogenase, mitochondrial	0.00410282644719244	0.970040076221191	0.992008473067494	NA	aag01100 ; aag	KXJ72282.1 hypothe	XP_021701024.1very long-chain specific acyl-CoA dehydrogenase, mitochondrial
AAEL0217:	NA	0.00416444206193503	0.975679771024678	0.994190331004269	NA	aag04141 ; aag	NA	XP_001648070.2eukaryotic translation initiation factor 2-alpha kinase
AAEL0198:	NA	0.00419265890362725	0.970124347873764	0.992008473067494	NA	NA	NA	XP_021702265.1leupaxin isoform X3
AAEL0154:	zinc finger imprinted 3	0.00421035668534849	0.986885598768007	0.996195840265819	NA	NA	NA	XP_001656484.2zinc finger imprinted 3
AAEL0043:	alpha-mannosidase 2	0.00425403286787102	0.977945637222698	0.994190331004269	NA	aag00511	XP_001845213.1 ma	XP_021693010.1alpha-mannosidase 2
AAEL0091:	fasciclin-2	0.00439391440845858	0.978566656808592	0.994233231307571	NA	NA	NA	XP_001659793.2fasciclin-2 isoform X6
AAEL0076(	casein kinase I	0.0044209656083957	0.967120869889706	0.991210985301503	NA	aag04310 ; aag	ETN65174.1 casein k	XP_001658552.1casein kinase I
AAEL0196:	NA	0.00444618481628745	0.968288709338803	0.991220636062002	NA	NA	NA	XP_021703463.1von Willebrand factor D and EGF domain-containing protein-like
AAEL0149(	F-box only protein 9	0.00446541675305607	0.967352886071841	0.991210985301503	NA	NA	NA	XP_021697723.1F-box only protein 9
AAEL0209:	NA	0.00447388746355199	0.969335062918025	0.991531930743849	NA	aag01100 ; aag	NA	XP_021696749.1carbonic anhydrase 1
AAEL0009:	mitochondrial import inner membrane translocase subunit Tim8	0.00453358260877523	0.966857754162266	0.991210985301503	NA	NA	KXJ69528.1 hypothe	XP_001657489.1mitochondrial import inner membrane translocase subunit Tim8
AAEL0124(	integrin beta-PS	0.00455570001560663	0.968839890116019	0.991466266553337	NA	aag04145 ; aag	NA	XP_021695415.1integrin beta isoform X1
AAEL0140:	NA	0.00461102032725663	0.984673789875844	0.995710792795332	NA	NA	NA	NP_001345960.1homeotic protein ultrabithorax
AAEL0145:	sideroflexin-1	0.00461317853877669	0.966183839282131	0.991210985301503	NA	NA	NA	XP_019541044.1 sideroflexin-1-like
AAEL0115(	angiogenic factor with G patch and FHA domains 1	0.00463939124728251	0.980372658582947	0.99449893454789	NA	NA	XP_019544102.1 PRE	XP_021697033.1angiogenic factor with G patch and FHA domains 1 isoform X2
AAEL0043:	eukaryotic translation initiation factor 1A, X-chromosomal	0.0047159935252017	0.967880518257725	0.991220636062002	NA	aag03013	ETN59410.1 eukaryo	XP_001649007.1eukaryotic translation initiation factor 1A, X-chromosomal
AAEL0068:	protein FRG1 homolog	0.00472586231899729	0.981775411629963	0.994967002560002	NA	NA	KXJ73901.1 hypothe	XP_001652245.1protein FRG1 homolog
AAEL0018:	uncharacterized LOC5572647	0.00475770212457071	0.967282662447493	0.991210985301503	NA	NA	KXJ79697.1 hypothe	XP_001654146.2uncharacterized protein LOC5572647
AAEL0086:	G protein alpha o subunit	0.00477381091213734	0.973310485164315	0.993357763885677	NA	NA	CRL06717.1 CLUMA_	KFB50440.1GTP-binding protein (o) alpha subunit, gnao
AAEL0131(	uncharacterized LOC5577253	0.00483229068698094	0.979716592336403	0.994485178621519	NA	NA	NA	XP_021704983.1uncharacterized protein LOC5577253 isoform X3
AAEL0197(	NA	0.00489020131366591	0.989656969077885	0.997058916995799	NA	NA	NA	XP_021707464.1UPF0769 protein CG18675
AAEL0132:	60S ribosomal protein L37a	0.00494703833389647	0.973240555303271	0.993357763885677	NA	aag03010	NA	XP_001663436.160S ribosomal protein L37a
AAEL0009:	clathrin light chain	0.00495433437075437	0.967462393222662	0.991210985301503	NA	NA	NA	XP_001657495.2clathrin light chain isoform X2
AAEL0224(	NA	0.00501196091261296	0.96531913666126	0.991092399652587	NA	NA	NA	XP_021708342.1transcription factor E2f1
AAEL0227:	NA	0.00501715108532141	0.971682259146447	0.992802528468446	NA	aag04120	NA	XP_001648569.1ubiquitin-conjugating enzyme E2-22 kDa
AAEL0001:	histone deacetylase complex subunit SAP30 homolog	0.00507963428429211	0.981558561978577	0.994966059410318	NA	NA	ETN65024.1 sap30 [A	XP_001657686.1histone deacetylase complex subunit SAP30 homolog
AAEL0121:	tRNA-dihydrouridine(20) synthase [NAD(P)+]-like	0.00510128659943747	0.981458836880502	0.994966059410318	NA	NA	KXJ68617.1 hypothe	XP_001655871.1tRNA-dihydrouridine(20) synthase [NAD(P)+]-like
AAEL0116(	exopolyphosphatase PRUNE1	0.00510180080489774	0.98328935961507	0.995614477295785	190	aag01100 ; aag	KXJ69594.1 hypothe	XP_001655485.2exopolyphosphatase PRUNE1
AAEL0001:	omega-amidase NIT2	0.00539572592231726	0.962138054880773	0.990224178616807	NA	aag01100 ; aag	XP_001864905.1 hyp	XP_001657671.1omega-amidase NIT2
AAEL0139(	ATP-dependent Clp protease ATP-binding subunit clpX-like, mitochondria	0.00540543800458054	0.96387278297536	0.99101283120624	NA	NA	NA	XP_001664208.1ATP-dependent Clp protease ATP-binding subunit clpX-like, mitochondrial
AAEL0277:	NA	0.00540591141535507	0.981250413338893	0.994872884921165	NA	NA	NA	KFB48800.1Dyak\GE21606-like protein
AAEL0205:	NA	0.00544166803086616	0.987934756445178	0.996578609724835	NA	aag04146	NA	XP_021694157.1peroxisome biogenesis factor 10
AAEL0254:	NA	0.00547286471635015	0.966529358751863	0.991210985301503	NA	aag01100 ; aag	NA	XP_021699388.1L-2-hydroxyglutarate dehydrogenase, mitochondrial
AAEL0015(	2-hydroxyacylsphingosine 1-beta-galactosyltransferase	0.00557494299136931	0.971617056412983	0.992802528468446	NA	NA	XP_001687884.1 AG	XP_001653600.12-hydroxyacylsphingosine 1-beta-galactosyltransferase
AAEL0115:	vacuolar protein-sorting-associated protein 36	0.00558896986119791	0.976857518709603	0.994190331004269	NA	aag04144	XP_001844544.1 con	XP_001655431.2vacuolar protein-sorting-associated protein 36
AAEL0129:	nuclear speckle splicing regulatory protein 1	0.00565768489536077	0.977728276613139	0.994190331004269	NA	NA	NA	XP_001663121.1nuclear speckle splicing regulatory protein 1
AAEL0067(	transcription initiation factor TFIID subunit 9	0.0056777639909334	0.978844449699041	0.99426392918949	NA	aag03022	KFB45294.1 hypothe	XP_001652244.1transcription initiation factor TFIID subunit 9
AAEL0213:	NA	0.00580534074369771	0.984420387364042	0.995692124835154	NA	NA	NA	NA
AAEL0019:	phosphatidate cytidylyltransferase, photoreceptor-specific	0.00581515347171207	0.968108831550078	0.991220636062002	00564 ; 04070	aag01100 ; aag	XP_001851458.1 con	XP_021699250.1phosphatidate cytidylyltransferase, photoreceptor-specific
AAEL0127:	uncharacterized LOC5576697	0.00586732966176242	0.963873596967599	0.99101283120624	NA	NA	KXJ73701.1 hypothe	XP_019931000.1 leucine-rich repeat-containing G-protein coupled receptor 4-like
AAEL0096:	uncharacterized LOC5580019	0.00588709452748106	0.958943492724758	0.989441920233345	NA	NA	NA	XP_001653901.2uncharacterized protein LOC5580019
AAEL0060:	PH-interacting protein	0.00593918514238235	0.968824814195029	0.991466266553337	NA	NA	KXJ76938.1 hypothe	XP_021706973.1PH-interacting protein
AAEL0049:	ADP-ribosylation factor 6	0.00597964072478032	0.958625677380691	0.989441920233345	NA	aag04144	KFB45724.1 hypothe	XP_001650010.1ADP-ribosylation factor 6
AAEL0075(	prolyl 3-hydroxylase sudestada1	0.00610186717243134	0.967435118398573	0.991210985301503	NA	NA	KXJ73459.1 hypothe	XP_001652781.2prolyl 3-hydroxylase sudestada1 isoform X2
AAEL0133:	mitotic spindle assembly checkpoint protein MAD2B	0.00618065493222782	0.97972311524582	0.994485178621519	NA	NA	NA	XP_001663462.2mitotic spindle assembly checkpoint protein MAD2B
AAEL0039(	prostatic acid phosphatase	0.00620444941804511	0.966148093466357	0.991210985301503	00740 ; 00730	aag01100 ; aag	KXJ73369.1 hypothe	XP_001647999.2prostatic acid phosphatase
AAEL0141:	WD repeat-containing protein CG11141	0.00621979984674633	0.970992401189071	0.992565216145504	NA	NA	NA	XP_001648099.1WD repeat-containing protein CG11141
AAEL0199:	NA	0.00627795395662324	0.978091722454249	0.994190331004269	NA	NA	NA	XP_019527789.1 mediator of RNA polymerase II transcription subunit 13
AAEL0058:	arginine-hydroxylase NDUFAF5, mitochondrial	0.00633435037522884	0.964167956172655	0.991092399652587	NA	NA	KXJ82301.1 hypothe	XP_001651507.1arginine-hydroxylase NDUFAF5, mitochondrial
AAEL0195:	NA	0.00636022470412984	0.956143332589379	0.989441920233345	NA	NA	NA	XP_021702030.1vascular endothelial growth factor receptor 1 isoform X4
AAEL0032:	leucine-rich repeat-containing protein 40	0.00637321530125007	0.969245330133231	0.991531930743849	NA	NA	XP_019545175.1 PRE	XP_001656638.2leucine-rich repeat-containing protein 40 isoform X1
AAEL0273:	NA	0.00638388023088705	0.966107921718867	0.991210985301503	NA	NA	NA	XP_021710717.1 receptor expression-enhancing protein 2
AAEL0019:	zinc finger protein 836	0.00640226697375405	0.968384429275961	0.991220636062002	NA	NA	KXJ70277.1 hypothe	XP_001654191.2zinc finger protein 836
AAEL0108:	NADH dehydrogenase [ubiquinone] iron-sulfur protein 3, mitochondrial	0.00641436169869616	0.962451921966164	0.99043643322888	NA	aag01100 ; aag	KXJ80847.1 hypothe	XP_021697979.1NADH dehydrogenase [ubiquinone] iron-sulfur protein 3, mitochondrial isoform X1
AAEL0194:	NA	0.00647681962326987	0.954043511923531	0.989303184592491	NA	NA	NA	XP_021705976.1nudix hydrolase 2
AAEL0070(	NA	0.00650599907114153	0.97841639182511	0.994190331004269	561	NA	KXJ77941.1 hypothe	XP_001658110.2FGGY carbohydrate kinase domain-containing protein

AAEL00959	mucin-5AC	0.00652817877659705	0.976628562155591	0.994190331004269	NA	NA	NA	XP_019544286.1 mucin-3A-like
AAEL02019	NA	0.00665797580567334	0.964784118798122	0.991092399652587	NA	aag04068	NA	XP_021701521.1G2/mitotic-specific cyclin-B3 isoform X1
AAEL01456	60S ribosomal protein L12	0.00666562902118053	0.961962846326498	0.990224178616807	NA	aag03010	NA	XP_001647834.160S ribosomal protein L12
AAEL00191	eukaryotic translation initiation factor 4E	0.00674437788737496	0.950157662297702	0.98806088919239	NA	aag03013 ; aag	KXJ84244.1 hypothet	XP_001654344.1eukaryotic translation initiation factor 4E isoform X2
AAEL01034	ruvB-like helicase 2	0.00706525490212344	0.960906816854121	0.989923653148286	NA	NA	NA	XP_001654452.1ruvB-like helicase 2
AAEL00456	unc-112-related protein	0.0071230070507094	0.946821512381953	0.987707093985696	NA	NA	KFB36913.1 AGAP00	XP_001649404.1unc-112-related protein
AAEL00942	zinc finger protein on ecdysone puffs	0.00714884470104581	0.949166209872839	0.98806088919239	NA	NA	NA	XP_021698458.1zinc finger protein on ecdysone puffs isoform X1
AAEL02365	NA	0.00715442511890487	0.983200781561203	0.995614477295785	NA	NA	NA	NA
AAEL00051	uncharacterized LOC5578440	0.00730637816584804	0.955044267102976	0.989341139916873	NA	NA	XP_312940.5 AGAP0	XP_001656957.2uncharacterized protein LOC5578440
AAEL00378	exportin-6-A	0.00731194737873369	0.955554706782479	0.989441920233345	NA	NA	XP_019525741.1 PRE	XP_021696427.1exportin-6-A isoform X2
AAEL00771	tuberin	0.00737600677595891	0.960333237147154	0.989916707542076	NA	aag04150 ; aag	XP_019565128.1 PRE	XP_001652850.2tuberin isoform X1
AAEL00173	fatty acyl-CoA reductase wat	0.00739258274327161	0.967582897440637	0.991210985301503	73	NA	KXJ76239.1 hypothet	XP_001660265.2fatty acyl-CoA reductase wat
AAEL00914	vacuolar fusion protein MON1 homolog A	0.00747161820283038	0.970293057332315	0.99207074595244	NA	NA	KXJ72052.1 hypothet	XP_001659778.1vacuolar fusion protein MON1 homolog A
AAEL01479	NA	0.00747509611421148	0.953672420470724	0.989303184592491	NA	aag01100 ; aag	NA	XP_001649622.2glutaryl-CoA dehydrogenase, mitochondrial
AAEL00527	uncharacterized LOC5566220	0.00748313322331382	0.958750999741161	0.989441920233345	NA	NA	KXJ79965.1 hypothet	ABF18352.1farnesoic acid O-methyl transferase-like protein
AAEL00867	acylglycerol kinase, mitochondrial	0.00750073876781471	0.964425914127556	0.991092399652587	NA	aag01100 ; aag	KXJ72072.1 hypothet	XP_001653417.2acylglycerol kinase, mitochondrial
AAEL00731	uncharacterized LOC5569024	0.00750531530242893	0.993337360407771	0.999035469616496	NA	NA	KXJ79670.1 hypothet	XP_001658277.1uncharacterized protein LOC5569024
AAEL00836	2-oxoisovalerate dehydrogenase subunit alpha, mitochondrial	0.00770418819341739	0.969804979956678	0.991902348067284	00640 ; 00280	aag01100 ; aag	KXJ75720.1 hypothet	XP_001653215.12-oxoisovalerate dehydrogenase subunit alpha, mitochondrial
AAEL00230	leucine-rich repeat neuronal protein 3	0.00770586985881564	0.980631884829606	0.994557508631324	NA	NA	XP_019563352.1 PRE	XP_001661262.1leucine-rich repeat neuronal protein 3 isoform X2
AAEL02900	NA	0.00772567101980761	0.945458920383637	0.987398325447749	NA	NA	NA	XP_001655529.2elongation factor Ts, mitochondrial isoform X1
AAEL02172	NA	0.00781791255463211	0.961792683272085	0.990224178616807	NA	NA	NA	XP_021694959.1probable alpha-aspartyl dipeptidase
AAEL02663	NA	0.0078298088242964	0.945283850970751	0.987398325447749	NA	aag03018	NA	XP_021701553.1mediator of RNA polymerase II transcription subunit 13 isoform X4
AAEL00341	carboxy-terminal domain RNA polymerase II polypeptide A small phospha	0.00794000839867426	0.956406493961579	0.989441920233345	04660 ; 04658	NA	XP_001849639.1 nuc	XP_021711079.1 carboxy-terminal domain RNA polymerase II polypeptide A small phosphatase 1
AAEL01083	mitogen-activated protein kinase kinase kinase 11	0.00796260929362697	0.963609953592665	0.990998530286399	NA	NA	XP_019531551.1 PRE	XP_021698005.1mitogen-activated protein kinase kinase kinase 11 isoform X1
AAEL00255	polyadenylate-binding protein-interacting protein 2	0.00797205014714224	0.947488311817901	0.987743607411212	NA	NA	ETN58296.1 hypothe	XP_001655603.1polyadenylate-binding protein-interacting protein 2
AAEL02700	NA	0.00815319362298021	0.967620982499827	0.991210985301503	NA	NA	NA	XP_021703913.1guanine nucleotide exchange factor MSS4 homolog
AAEL00696	uncharacterized LOC5568624	0.00823196379844079	0.940376204772541	0.985798796572901	NA	NA	KXJ77168.1 hypothet	XP_001652458.1uncharacterized protein LOC5568624
AAEL00015	protein NipSnap	0.00833562149816465	0.94542844819487	0.987398325447749	NA	NA	XP_001864008.1 con	XP_001658894.2protein NipSnap
AAEL01099	queuine tRNA-ribosyltransferase catalytic subunit	0.00842243071069315	0.964922859317248	0.991092399652587	NA	NA	KXJ84342.1 hypothet	XP_001661277.2queuine tRNA-ribosyltransferase catalytic subunit
AAEL00026	uncharacterized protein C12orf31 homolog	0.00849529164065419	0.946646643881811	0.987646532539731	NA	NA	ETN59585.1 hypothe	XP_019525229.1 protein LLP homolog
AAEL01222	uncharacterized LOC5575970	0.00868841914185726	0.969317088953114	0.991531930743849	NA	NA	KXJ73188.1 hypothet	XP_001662323.1uncharacterized protein LOC5575970
AAEL00547	uncharacterized protein CG10915	0.00885248253176655	0.979639259849307	0.994485178621519	NA	NA	NA	XP_001650934.2uncharacterized protein CG10915 isoform X4
AAEL01340	catalase	0.00889769210723746	0.939765114301796	0.985798796572901	00630 ; 00380	aag01100 ; aag	NA	XP_001663600.1catalase isoform X1
AAEL01476	uncharacterized protein C6orf106 homolog	0.00889990136205933	0.947320371811578	0.987743607411212	NA	NA	NA	XP_021706928.1uncharacterized protein C6orf106 homolog isoform X1
AAEL00405	aladin	0.00890889278558169	0.968236059198298	0.991220636062002	NA	aag03013	KXJ74668.1 hypothet	XP_019558903.1 aladin-like
AAEL01746	chorion peroxidase	0.00898060416836106	0.964623239766836	0.991092399652587	NA	NA	NA	XP_021708476.1chorion peroxidase
AAEL01323	coatomer subunit delta	0.00901657973777128	0.937263671619404	0.984619988774498	NA	NA	NA	XP_001663404.1coatomer subunit delta
AAEL02158	NA	0.00915804669416559	0.949472132755931	0.98806088919239	NA	NA	NA	NA
AAEL00198	ejaculatory bulb-specific protein 3	0.00929439168823261	0.976859308125911	0.994190331004269	NA	NA	KXJ81669.1 hypothet	XP_001660753.1ejaculatory bulb-specific protein 3
AAEL02350	NA	0.00931716433692701	0.95839314643167	0.989441920233345	NA	NA	NA	XP_001653285.1solute carrier family 35 member F5
AAEL01011	kelch domain-containing protein 4	0.0093208334119749	0.956578676021135	0.989441920233345	NA	NA	NA	XP_001660646.2kelch domain-containing protein 4
AAEL01225	platelet-activating factor acetylhydrolase IB subunit beta homolog	0.00932867685802534	0.965436934829985	0.991092399652587	NA	aag01100 ; aag	XP_001861941.1 plat	XP_001662362.1platelet-activating factor acetylhydrolase IB subunit beta homolog
AAEL00151	bcl-2-related ovarian killer protein	0.00934579042947632	0.945129947076321	0.987398325447749	NA	aag04214 ; aag	BAV03492.1 proapop	XP_001659566.1bcl-2-related ovarian killer protein
AAEL01375	uncharacterized LOC5578565	0.0095899099997449	0.977060520366544	0.994190331004269	NA	NA	NA	XP_001663955.2uncharacterized protein LOC5578565 isoform X1
AAEL02704	NA	0.00963221280976158	0.961234059136193	0.989958121115976	NA	NA	NA	NA
AAEL01407	histone acetyltransferase Tip60	0.00965303541347569	0.973767255454139	0.993377986871678	NA	NA	NA	XP_021706851.1histone acetyltransferase Tip60
AAEL02751	NA	0.00966648561278523	0.977676838934034	0.994190331004269	NA	aag04150 ; aag	NA	XP_021700021.1protein LST8 homolog
AAEL00824	mediator of RNA polymerase II transcription subunit 12	0.00971181678887956	0.940133148039672	0.985798796572901	NA	NA	KFB42818.1 AGAP00	XP_021708637.1mediator of RNA polymerase II transcription subunit 12
AAEL00618	CDC42 small effector protein homolog	0.010011388705434	0.958612873977682	0.989441920233345	NA	NA	ETN63571.1 CDC42 s	XP_021705766.1CDC42 small effector protein homolog
AAEL00486	cyclin G	0.0100185940522381	0.931844278985248	0.98347583693566	NA	aag04068	KXJ81212.1 hypothet	XP_001649965.1cyclin G
AAEL00066	mpv17-like protein 2	0.0102143008291994	0.95754431201323	0.989441920233345	NA	aag04146	NA	XP_001649766.2mpv17-like protein 2 isoform X1
AAEL02195	NA	0.0102445555633395	0.947640797879044	0.987743607411212	NA	NA	NA	XP_021713230.1uncharacterized protein LOC5574629
AAEL01034	facilitated trehalose transporter Tret1-2 homolog	0.010343244632884	0.933834213922123	0.98347583693566	NA	NA	NA	XP_021702905.1facilitated trehalose transporter Tret1-2 homolog isoform X1
AAEL02622	NA	0.0104064464268637	0.934024021141886	0.98347583693566	NA	aag04120	NA	XP_021699270.1F-box/WD repeat-containing protein 7 isoform X1
AAEL00318	solute carrier family 22 member 21	0.0104767754069342	0.936259575289469	0.98424319806035	NA	NA	KXJ76913.1 hypothet	XP_021711550.1solute carrier family 22 member 21 isoform X1
AAEL01977	NA	0.0105345145417025	0.942027434009224	0.986293827255989	NA	NA	NA	XP_021696112.1uncharacterized protein LOC5574095 isoform X1
AAEL00964	brahma-associated protein of 60 kDa	0.0105450417736427	0.95424768333406	0.989303184592491	NA	NA	NA	XP_001653905.2brahma-associated protein of 60 kDa
AAEL01217	putative RNA-binding protein 15	0.0105512424472447	0.929877447948672	0.98347583693566	NA	NA	XP_019554385.1 PRE	XP_001655916.2putative RNA-binding protein 15 isoform X2
AAEL02080	NA	0.0105756634032858	0.938566143970945	0.985210738070975	NA	NA	NA	XP_021697807.1uncharacterized protein LOC5564472
AAEL00298	chromatin target of PRMT1 protein	0.010889816082949	0.941362165757694	0.986042559017697	NA	NA	ETN60698.1 hypothe	XP_001662894.2chromatin target of PRMT1 protein
AAEL00743	myosin-2 essential light chain	0.0110682292525351	0.918224398378874	0.979657756188126	NA	NA	ETN67344.1 myosin l	XP_001652766.1myosin-2 essential light chain isoform X1
AAEL00584	spectrin beta chain	0.0111632505784796	0.927545615656194	0.982649508778163	NA	NA	XP_019527004.1 PRE	XP_021708504.1 spectrin beta chain
AAEL00154	uncharacterized LOC5571300	0.0112839555655538	0.971199905482802	0.992667058786265	NA	NA	NA	XP_021695818.1uncharacterized protein LOC5571300 isoform X1
AAEL01823	NA	0.0113210307880757	0.926062614466105	0.982258702730868	04660 ; 04658	NA	NA	XP_001659319.2probable protein phosphatase CG10417
AAEL00702	glutathione S-transferase C-terminal domain-containing protein homolog	0.0113488860907093	0.947883827358024	0.987817324954535	NA	NA	ETN65614.1 jumping	XP_001652465.1protein JTB isoform X2
AAEL01990	NA	0.0114076484356071	0.951007796336869	0.988386462829441	NA	NA	NA	XP_021704447.1polyhomeotic-proximal chromatin protein
AAEL01124	UDP-glucose 6-dehydrogenase	0.0116050327686008	0.944893789565529	0.987398325447749	00053 ; 00040 ; 00520	aag01100 ; aag	XP_001844884.1 UDI	XP_001661518.1UDP-glucose 6-dehydrogenase
AAEL00239	uncharacterized LOC5574472	0.0116439884911461	0.914722422037634	0.977901601050505	NA	NA	KXJ80310.1 hypothet	XP_001655131.2uncharacterized protein LOC5574472
AAEL00345	MOB kinase activator-like 4	0.0116852468461164	0.931047864580419	0.98347583693566	NA	NA	ETN65806.1 preimpl	XP_001656861.1MOB kinase activator-like 4
AAEL00813	UMP-CMP kinase	0.0117343204346008	0.920222662404121	0.980340877547081	00983 ; 00240 ; 00230 ;	aag01100 ; aag	ETN62945.1 cytidylat	XP_001658961.1UMP-CMP kinase
AAEL02473	NA	0.0117806305766994	0.949136527270355	0.98806088919239	04151 ; 05165 ; 04714 ;	aag03450	NA	XP_021704766.1DNA-dependent protein kinase catalytic subunit
AAEL00207	TBP-related factor	0.0117861024681066	0.972116515450679	0.992802528468446	NA	aag03022	ETN59952.1 TATA-bo	XP_001654561.1TBP-related factor
AAEL01136	endothelin-converting enzyme homolog	0.0118364353797865	0.91134007911933	0.977065292794603	NA	NA	XP_019529531.1 PRE	XP_021700699.1endothelin-converting enzyme homolog isoform X1
AAEL02638	NA	0.0118729079917562	0.938220657468066	0.984997888194084	NA	NA	NA	XP_021712412.1 2-hydroxyacyl-CoA lyase 1
AAEL00607	phosphoglycerate mutase 2	0.0119695995698234	0.941442685700423	0.986042559017697	00680 ; 00010 ; 00260	aag01100 ; aag	ETN62733.1 phospho	XP_001657403.1phosphoglycerate mutase 2
AAEL01127	NA	0.0120393118851984	0.928652343745432	0.98291452348119	NA	aag04144	KXJ69388.1 hypothet	XP_001661554.1programmed cell death 6-interacting protein
AAEL01985	NA	0.0121177038393391	0.934012776914509	0.98347583693566	NA	NA	NA	XP_021708633.1unconventional myosin-XVIIIa isoform X2
AAEL02099	NA	0.012242171992821	0.906973378823877	0.975993693196454	NA	NA	NA	XP_019565602.1 tetraspanin-13 isoform X1
AAEL00568	ejaculatory bulb-specific protein 3	0.0123168255372248	0.958706768049723	0.989441920233345	NA	NA	KXJ71463.1 hypothet	XP_001651345.1ejaculatory bulb-specific protein 3
AAEL00179	uncharacterized LOC5572452	0.0123318724067652	0.943679076239734	0.987236796220786	NA	NA	XP_001843637.1 con	XP_021702454.1uncharacterized protein LOC5572452 isoform X2
AAEL01743	uncharacterized LOC23687855	0.0123511929051862	0.943888600904268	0.987296271832165	NA	NA	NA	CRL06976.1CLUMA_CG020014, isoform A
AAEL00587	E3 ubiquitin-protein ligase AMFR	0.0123799311076325	0.909549583980472	0.97691422813284	NA	aag04141	XP_019549431.1 PRE	XP_001651539.2E3 ubiquitin-protein ligase AMFR isoform X1

AAEL02205	NA	0.0124557291266357	0.917228504157907	0.979322882967209	NA	NA	NA	NA
AAEL00197	WW domain-binding protein 11	0.0126533798713978	0.962614429061954	0.990492896816292	NA	aag03040	KXJ81252.1 hypothet	XP_001660809.2WW domain-binding protein 11
AAEL01730	farnesol dehydrogenase	0.0127275586403084	0.932109334524716	0.98347583693566	NA	aag00981 ; aag	NA	XP_011492954.2farnesol dehydrogenase
AAEL00966	uncharacterized LOC5572227	0.0128857821008863	0.944352806596136	0.987380357493199	NA	NA	NA	XP_001660297.2uncharacterized protein LOC5572227
AAEL01111	polymerase delta-interacting protein 2	0.0130350896335426	0.903953369363168	0.975625018165596	NA	NA	KXJ70796.1 hypothet	XP_001655117.1polymerase delta-interacting protein 2
AAEL01827	NA	0.0130628491363971	0.938188672584238	0.984997888194084	NA	NA	NA	XP_001648755.2mitotic spindle assembly checkpoint protein MAD1
AAEL00194	NA	0.0131667509153091	0.933514927396306	0.98347583693566	NA	NA	NA	XP_001654332.2uncharacterized protein LOC5573004
AAEL02037	NA	0.0133083795375091	0.9328462003068	0.98347583693566	NA	NA	NA	XP_021702619.1BTB/POZ domain-containing protein KCTD5
AAEL01299	rho GDP-dissociation inhibitor 1	0.0135168268624173	0.905586617411861	0.975993693196454	NA	NA	NA	XP_001663181.1rho GDP-dissociation inhibitor 1 isoform X2
AAEL00522	60S ribosomal protein L30	0.0135989025889113	0.927167024153682	0.982472473084084	NA	aag03010	CRK98941.1 CLUMA_	XP_001650291.160S ribosomal protein L30
AAEL00675	probable serine/threonine-protein kinase DDB_G0282963	0.0136045737336417	0.897919338186192	0.973750254467651	NA	NA	XP_001862122.1 con	XP_001652180.1probable serine/threonine-protein kinase DDB_G0282963
AAEL01133	MOXD1 homolog 2	0.0136779640348068	0.948885497328868	0.98806088919239	NA	NA	XP_001846760.1 dop	XP_021710974.1MOXD1 homolog 2
AAEL01075	WW domain-containing adapter protein with coiled-coil homolog	0.013725421848199	0.913077522016475	0.977561298149576	NA	NA	XP_019529255.1 PRE	XP_021707250.1WW domain-containing adapter protein with coiled-coil homolog isoform X2
AAEL00821	myb-like protein AA	0.0138108136584467	0.914415226068703	0.977855771096363	NA	NA	KXJ79411.1 hypothet	XP_001659034.2myb-like protein AA isoform X1
AAEL02083	NA	0.0138237368045926	0.90992642031271	0.97691422813284	NA	NA	NA	XP_021705657.1MAP kinase-interacting serine/threonine-protein kinase 1
AAEL00555	putative uncharacterized zinc finger protein 814	0.0138524897178987	0.941646696812507	0.986119597595162	NA	NA	NA	XP_001651125.2putative uncharacterized zinc finger protein 814
AAEL00210	ero1-like protein	0.0139421411594986	0.925509837606988	0.982258702730868	NA	aag04141	KXJ78916.1 hypothet	XP_021704945.1ero1-like protein
AAEL00801	midnolin homolog	0.0139788196998355	0.916286658783347	0.978701629893685	NA	NA	XP_001862643.1 con	XP_001658824.2midnolin homolog
AAEL02523	NA	0.014024267013818	0.957957784537643	0.989441920233345	NA	NA	NA	XP_021704562.1uncharacterized protein LOC110677715 isoform X1
AAEL02382	NA	0.0142236979052433	0.917700888935081	0.979587080973985	NA	NA	NA	XP_021694921.1protein retinal degeneration B isoform X4
AAEL00125	proline-rich protein 2	0.0143578826572022	0.940301494929176	0.985798796572901	NA	NA	XP_019535984.1 PRE	XP_021698467.1proline-rich protein 2 isoform X2
AAEL00414	phosphatidylinositol transfer protein alpha isoform	0.0144471393836978	0.894620504782315	0.972739913152176	NA	NA	XP_001851402.1 pho	XP_001648499.1phosphatidylinositol transfer protein alpha isoform isoform X1
AAEL02246	NA	0.0145587230381399	0.945445981927191	0.987398325447749	NA	NA	NA	XP_019525045.1 proteoglycan 4
AAEL00341	NA	0.0146429166589248	0.90971904962584	0.97691422813284	NA	aag04214	XP_001851487.1 lam	XP_021710074.1 lamin Dm0
AAEL01389	alpha-1,3-mannosyl-glycoprotein 4-beta-N-acetylglucosaminyltransferase	0.0146641933025702	0.930127142517109	0.98347583693566	NA	aag01100 ; aag	NA	XP_001664101.2alpha-1,3-mannosyl-glycoprotein 4-beta-N-acetylglucosaminyltransferase B
AAEL02809	NA	0.0146806604934022	0.911964087754456	0.977278855888727	NA	NA	NA	XP_021693078.1fibroblast growth factor receptor homolog 1 isoform X2
AAEL01985	NA	0.0147232172807157	0.9746776712024	0.993683129891922	230	NA	NA	XP_021701678.1MAGUK p55 subfamily member 6 isoform X3
AAEL01365	acyl-CoA dehydrogenase family member 9, mitochondrial	0.0147317770879413	0.931087543594413	0.98347583693566	NA	NA	NA	XP_001663840.2acyl-CoA dehydrogenase family member 9, mitochondrial
AAEL01476	NA	0.0147327708581352	0.956883769244559	0.989441920233345	NA	NA	NA	XP_001649580.2uncharacterized protein LOC5565239
AAEL00026	protein crossbronx homolog	0.0147958521295842	0.936432210282369	0.98424319806035	NA	NA	KXJ81839.1 hypothet	XP_001654780.2protein crossbronx homolog
AAEL02216	NA	0.0148670446588493	0.932001558080935	0.98347583693566	NA	aag03010	NA	XP_021705245.139S ribosomal protein L35, mitochondrial
AAEL00488	uncharacterized LOC5565610	0.0148785934221415	0.966527709013635	0.991210985301503	NA	NA	XP_001867639.1 ada	XP_021693782.1uncharacterized protein LOC5565610
AAEL01981	NA	0.0149025302137077	0.920410704261639	0.980340877547081	NA	NA	NA	XP_021700487.1probable phospholipid-transporting ATPase VD isoform X1
AAEL01166	uncharacterized LOC5575101	0.0150483236195519	0.896299806949421	0.973419069671699	NA	aag03015	KXJ76008.1 hypothet	XP_021705928.1uncharacterized protein LOC5575101 isoform X2
AAEL01953	NA	0.0152132385103638	0.900285661898286	0.974065297962641	NA	NA	NA	XP_021693430.1glutamyl aminopeptidase isoform X2
AAEL01306	serine/threonine-protein phosphatase PP2A	0.0152318462847442	0.889932144933226	0.972006599130629	04660 ; 04658	aag04013 ; aag	NA	XP_001663281.1serine/threonine-protein phosphatase PP2A
AAEL00417	tubulin alpha-8 chain	0.0153102465637831	0.953552200834388	0.989303184592491	NA	NA	KXJ77464.1 hypothet	XP_021698338.1tubulin alpha-8 chain
AAEL01709	tyrosine 3-monooxygenase	0.0153413986867828	0.887960963865238	0.970996238637524	00350 ; 00950 ; 00790	aag01100 ; aag	NA	XP_011493493.2tyrosine 3-monooxygenase isoform X1
AAEL02416	NA	0.0154069958753802	0.941465977345086	0.986042559017697	NA	NA	NA	XP_021702255.1thiamine transporter 1-like
AAEL02278	NA	0.0154280405222678	0.951832963529483	0.988479552477666	NA	NA	NA	XP_001649156.2uncharacterized protein LOC5564808
AAEL00946	coactosin-like protein	0.0154996203180068	0.925687821613243	0.982258702730868	NA	NA	NA	XP_001660081.1coactosin-like protein
AAEL00818	ribosomal protein S6 kinase delta-1	0.015528527146072	0.953628758033952	0.989303184592491	NA	NA	KXJ77460.1 hypothet	XP_021710073.1ribosomal protein S6 kinase delta-1
AAEL00441	putative pre-mRNA-splicing factor ATP-dependent RNA helicase PRP1	0.0156982192371071	0.884860610943521	0.970521528629931	NA	aag03040	ETN60552.1 ATP-dep	XP_001649136.1putative pre-mRNA-splicing factor ATP-dependent RNA helicase PRP1
AAEL00143	39S ribosomal protein L22, mitochondrial	0.0158344736093546	0.922567582233514	0.981343310857761	NA	aag03010	KXJ69404.1 hypothet	XP_001659205.139S ribosomal protein L22, mitochondrial
AAEL01170	RYamide neuropeptides	0.0158649672562709	0.95615460986336	0.989441920233345	NA	NA	XP_001850615.1 con	XP_001655654.1RYamide neuropeptides
AAEL00403	importin-11	0.0158765795017456	0.923215224070738	0.981343310857761	NA	NA	KXJ72128.1 hypothet	XP_019531657.1 importin-11-like
AAEL00048	SPRY domain-containing protein 7	0.0160006118969413	0.940198811393057	0.985798796572901	NA	NA	KXJ82402.1 hypothet	XP_001656971.1SPRY domain-containing protein 7
AAEL00863	stress-activated protein kinase JNK	0.0160191044724861	0.882589319352336	0.970357617045106	4150	aag04141 ; aag	KXJ81744.1 hypothet	XP_021703776.1stress-activated protein kinase JNK isoform X1
AAEL00212	bromodomain-containing protein 3	0.0160269986588698	0.887695555572744	0.970996238637524	NA	NA	NA	XP_001654740.2homeotic protein female sterile isoform X1
AAEL00921	28S ribosomal protein S35, mitochondrial	0.016046696578123	0.912246472870579	0.977296280428831	NA	NA	NA	XP_001659845.228S ribosomal protein S35, mitochondrial
AAEL01172	NA	0.0161131335891216	0.911942027741369	0.977278855888727	NA	aag01100 ; aag	XP_001688291.2 AG/	XP_021694851.1mannosyl-oligosaccharide alpha-1,2-mannosidase IA isoform X1
AAEL00041	mucin-17	0.0161422628175239	0.94805850547657	0.987881609350457	NA	NA	KXJ84209.1 hypothet	XP_019558266.1 mucin-17-like isoform X1
AAEL00876	transmembrane protein 164	0.0162038006022266	0.92681013867498	0.982472473084084	NA	NA	ETN66894.1 transme	XP_001659460.1transmembrane protein 164
AAEL00908	importin-7	0.0162623397916212	0.892928722761663	0.972739913152176	NA	aag04013	KFB40301.1 hypothe	XP_019538752.1 importin-7-like
AAEL01322	NADH-cytochrome b5 reductase 3	0.016307825956604	0.893661153612574	0.972739913152176	NA	aag00520	NA	XP_001656510.2NADH-cytochrome b5 reductase 3 isoform X2
AAEL00159	protein YIPF1	0.0163878466838298	0.904845517928104	0.975993693196454	NA	NA	ETN66095.1 hypothe	XP_001653569.1protein YIPF1
AAEL01138	non-structural maintenance of chromosomes element 3 homolog	0.0164067713246328	0.960482814427654	0.989920222329647	NA	NA	KXJ79676.1 hypothet	XP_001655313.1non-structural maintenance of chromosomes element 3 homolog
AAEL00542	delta-1-pyrroline-5-carboxylate dehydrogenase, mitochondrial	0.0164249386414515	0.89389115314798	0.972739913152176	00250 ; 00330	aag01100 ; aag	KXJ81633.1 hypothet	XP_001650875.1delta-1-pyrroline-5-carboxylate dehydrogenase, mitochondrial
AAEL01180	erythrocyte band 7 integral membrane protein	0.0164365107360985	0.90670542410638	0.975993693196454	NA	NA	XP_019548944.1 PRE	XP_001661914.1band 7 protein AGAP004871 isoform X3
AAEL00469	E3 ubiquitin-protein ligase HRD1	0.0165019686103783	0.893675440902581	0.972739913152176	NA	aag04141 ; aag	KXJ69970.1 hypothet	XP_021707970.1E3 ubiquitin-protein ligase HRD1
AAEL01307	probable Dol-P-Man:Man(7)GlcNAc(2)-PP-Dol alpha-1,6-mannosyltransfe	0.0165234951639657	0.906385829832292	0.975993693196454	00513 ; 00510	aag01100 ; aag	NA	XP_021695017.1probable DolMan:Man(7)GlcNAc(2)-Dol alpha-1,6-mannosyltransferase
AAEL00637	sodium-independent sulfate anion transporter	0.0166429361431882	0.899490812165112	0.97393667311859	NA	NA	XP_019543349.1 PRE	XP_001651949.2sodium-independent sulfate anion transporter isoform X3
AAEL02029	NA	0.0166614955546289	0.990292769298563	0.997446810758031	NA	NA	NA	XP_021701765.1roundabout homolog 1
AAEL00656	transmembrane emp24 domain-containing protein 5	0.0166641526477315	0.908140930243735	0.976356507260987	NA	NA	KFB44977.1 AGAP00	XP_001652035.1transmembrane emp24 domain-containing protein 5
AAEL00014	zinc finger protein 41	0.0166944505918385	0.959010996885564	0.989441920233345	NA	NA	XP_001849423.1 con	XP_001657705.1GDNF-inducible zinc finger protein 1 isoform X1
AAEL00782	40S ribosomal protein S29	0.0167507547238781	0.903620372772239	0.975494447471861	NA	aag03010	CAD27766.1 putative	XP_001652933.140S ribosomal protein S29
AAEL00819	uncharacterized protein CG1161	0.0167864356669643	0.886489550379965	0.9709344693273	NA	NA	KXJ79409.1 hypothet	XP_001659030.1uncharacterized protein CG1161
AAEL00678	ras-like GTP-binding protein RhoL	0.016788407686638	0.879723000940129	0.969652664055335	NA	aag04013 ; aag	XP_001238816.1 AG/	XP_001652240.1ras-like GTP-binding protein RhoL
AAEL01993	NA	0.0168129926194443	0.878075726224982	0.969652664055335	NA	NA	NA	XP_021709761.1transforming growth factor-beta-induced protein ig-h3 isoform X1
AAEL00070	pre-mRNA-splicing factor CWC22 homolog	0.0168138380705606	0.933114300340967	0.98347583693566	NA	NA	KXJ80283.1 hypothet	XP_001650254.2pre-mRNA-splicing factor CWC22 homolog
AAEL00321	carotenoid isomerooxygenase	0.0168503221703458	0.907487331664935	0.976056966800435	NA	NA	KXJ70865.1 hypothet	XP_001656637.1carotenoid isomerooxygenase isoform X1
AAEL00130	alpha-tocopherol transfer protein-like	0.0169353189693875	0.900812503497144	0.974252903247646	NA	NA	ETN63770.1 CRAL/TF	XP_021703684.1alpha-tocopherol transfer protein-like
AAEL00149	vacuolar protein sorting-associated protein 54	0.0170473210731849	0.92059785999141	0.980365872889823	NA	NA	KXJ72804.1 hypothet	XP_021700653.1vacuolar protein sorting-associated protein 54
AAEL01969	NA	0.0171113956260977	0.91637614031341	0.978701629893685	NA	NA	NA	XP_021700214.1short neuropeptide F
AAEL01991	NA	0.0171292359043435	0.897135748595355	0.973750254467651	901	NA	NA	XP_021703899.1uncharacterized protein LOC5568178 isoform X4
AAEL00328	uncharacterized LOC5577633	0.0172155069201562	0.876697662877173	0.969652664055335	NA	NA	NA	XP_021693877.1uncharacterized protein LOC5577633 isoform X3
AAEL00065	N-acetylgalactosaminyltransferase 6	0.0172342285807183	0.901677863840259	0.974426271645104	NA	aag01100 ; aag	KXJ69756.1 hypothet	XP_021710563.1N-acetylgalactosaminyltransferase 6
AAEL00579	V-type proton ATPase subunit B	0.0172575728453322	0.894002564035978	0.972739913152176	00195 ; 00190	aag01100 ; aag	ETN67054.1 ATP synt	XP_001651458.1V-type proton ATPase subunit B
AAEL01444	uncharacterized LOC5564426	0.017328793267857	0.945863226608369	0.987518968073456	901	NA	NA	XP_001648771.2uncharacterized protein LOC5564426
AAEL01405	CUE domain-containing protein 2	0.017332890948237	0.954090089366357	0.989303184592491	NA	NA	NA	XP_001657343.1CUE domain-containing protein 2
AAEL00363	small glutamine-rich tetratricopeptide repeat-containing protein beta	0.0173645164674934	0.876920591190806	0.969652664055335	NA	NA	KFB40825.1 AGAP00	XP_001657084.1small glutamine-rich tetratricopeptide repeat-containing protein beta
AAEL00965	uncharacterized LOC5580030	0.0174765399740701	0.89455750552923	0.972739913152176	NA	aag03010	NA	CRL04840.1CLUMA_CG017893, isoform A

AAEL02719	NA	0.0175316376726673	0.87965270880171	0.969652664055335	NA	NA	NA	XP_021694695.1solute carrier family 35 member E1 homolog
AAEL01321	histone-lysine N-methyltransferase E(z)	0.017638431340194	0.933758350875177	0.98347583693566	310	aag01100 ; aag	NA	XP_001663394.2histone-lysine N-methyltransferase E(z) isoform X2
AAEL00353	bromodomain-containing protein DDB_G0270170	0.0176845668096143	0.910812061379495	0.976946997335368	NA	NA	KXJ68806.1 hypothe	XP_021711621.1bromodomain-containing protein DDB_G0270170
AAEL01269	ATP-binding cassette sub-family A member 3	0.0177312932102936	0.898858134937992	0.973780616635202	NA	NA	KXJ71644.1 hypothe	XP_021697250.1ATP-binding cassette sub-family A member 3
AAEL00700	mitochondrial import receptor subunit TOM40 homolog 1	0.0177487556851487	0.886704491616068	0.9709344693273	NA	NA	ETN59300.1 mitocho	XP_021705463.1mitochondrial import receptor subunit TOM40 homolog 1
AAEL00230	protein FRA10AC1 homolog	0.0177654777351553	0.959010996897101	0.989441920233345	NA	NA	KXJ68064.1 hypothe	XP_021708031.1protein FRA10AC1 homolog
AAEL00781	mitosis initiation protein fs(1)Ya	0.0177818023255514	0.910363054425336	0.97691422813284	NA	NA	KXJ69055.1 hypothe	XP_001652912.1mitosis initiation protein fs(1)Ya
AAEL00777	uncharacterized LOC5569577	0.0178347740907666	0.890622594337468	0.972299099904304	NA	NA	KFB40964.1 hypothe	XP_001658640.1uncharacterized protein LOC5569577
AAEL01987	NA	0.0178852354102987	0.883606875745376	0.970521528629931	NA	aag03018	NA	XP_021708017.1non-canonical poly(A) RNA polymerase protein Trf4-1 isoform X1
AAEL01394	chromatin assembly factor 1 subunit B	0.0179442486754582	0.959470521107241	0.989441920233345	NA	NA	NA	XP_001664137.1chromatin assembly factor 1 subunit B
AAEL00392	ubiquitin-like protein 4A-B	0.0179564834389075	0.905257227400665	0.975993693196454	NA	NA	KXJ73366.1 hypothe	XP_001648001.1ubiquitin-like protein 4A-B
AAEL02419	NA	0.0179623380921456	0.923348717477082	0.981343310857761	NA	NA	NA	XP_021697895.1acyl-coenzyme A thioesterase 13-like
AAEL01985	NA	0.0179883483623354	0.898725690774843	0.973780616635202	NA	aag04150	NA	XP_021702015.1 restin homolog
AAEL01971	NA	0.0180178123086702	0.891290759646343	0.972336404299425	NA	NA	NA	XP_021708169.1phosphate carrier protein, mitochondrial
AAEL00960	receptor expression-enhancing protein 5	0.018195922786386	0.869344162917209	0.968652831735134	NA	NA	NA	XP_011493497.1receptor expression-enhancing protein 5 isoform X2
AAEL00313	B-cell receptor-associated protein 31	0.0182630315825236	0.865118158679846	0.967527624717057	NA	aag04141	NA	XP_001656332.2B-cell receptor-associated protein 31 isoform X3
AAEL01474	ras-related protein Ral-a	0.0182836126678072	0.894192448664316	0.972739913152176	NA	NA	NA	XP_019559857.1 ras-related protein Ral-a-like isoform X2
AAEL01952	NA	0.0184312126875651	0.926111335024235	0.982258702730868	NA	NA	NA	XP_021704743.1protein timeless homolog
AAEL00544	putative uncharacterized protein DDB_G0285119	0.0184599052661654	0.978310322878755	0.994190331004269	NA	NA	NA	XP_021696155.1putative uncharacterized protein DDB_G0293878 isoform X4
AAEL01384	acyl-CoA-binding protein	0.0184773340950835	0.874348280318859	0.969652664055335	NA	NA	NA	XP_021694733.1acyl-CoA-binding protein
AAEL01138	116 kDa U5 small nuclear ribonucleoprotein component	0.0184945282480106	0.922876769929856	0.981343310857761	NA	aag03040	KFB37716.1 hypothe	XP_001655314.1116 kDa U5 small nuclear ribonucleoprotein component
AAEL01007	uncharacterized LOC5572850	0.0188070759235547	0.934357292856797	0.98347583693566	NA	NA	NA	XP_021705523.1uncharacterized protein LOC5572850
AAEL01174	succinate--CoA ligase [ADP-forming] subunit beta, mitochondrial	0.0188426162422308	0.883451249040914	0.970521528629931	00640 ; 00720 ; 00660 ;	aag01100 ; aag	XP_001661866.1 suc	XP_001661866.1succinate--CoA ligase [ADP-forming] subunit beta, mitochondrial isoform X2
AAEL00281	uncharacterized protein KIAA1683 homolog	0.0188505861148897	0.882067201946736	0.970357617045106	NA	NA	ETN67463.1 hypothe	EAT45974.1AAEL002817
AAEL01317	group XV phospholipase A2	0.0188662781920644	0.890409754629324	0.972299099904304	NA	aag04142 ; aag	NA	XP_001663352.2group XV phospholipase A2
AAEL00240	endoplasmic reticulum metallopeptidase 1	0.0189300529631585	0.927651024663702	0.982649508778163	NA	NA	KXJ82850.1 hypothe	XP_001655226.2endoplasmic reticulum metallopeptidase 1
AAEL00859	protein spaetzle 3	0.0189372266645705	0.889694148215971	0.971977389514825	NA	NA	KXJ75686.1 hypothe	XP_021702207.1protein spaetzle 3
AAEL00018	eukaryotic translation initiation factor 5	0.0189540383853492	0.86831295811267	0.96863134437578	NA	aag03013 ; aag	KFB44785.1 AGAP01	XP_001658942.1eukaryotic translation initiation factor 5
AAEL00752	electron transfer flavoprotein-ubiquinone oxidoreductase, mitochondrial	0.0190302911612374	0.857332607894857	0.964980728557884	NA	NA	KXJ77724.1 hypothe	XP_021699682.1electron transfer flavoprotein-ubiquinone oxidoreductase, mitochondrial
AAEL00836	peptidyl-prolyl cis-trans isomerase FKBP2	0.0191806365305047	0.880190035085222	0.969652664055335	NA	NA	ETN61115.1 FK506-b	XP_001653210.1peptidyl-prolyl cis-trans isomerase FKBP2
AAEL01413	melanization protease 1	0.0191885945634133	0.911165348112893	0.976991789015945	NA	NA	NA	XP_001648090.1melanization protease 1
AAEL00932	synaptic functional regulator FMR1	0.0193474960412249	0.866558560797657	0.968074250389273	NA	aag03013	NA	XP_021709946.1synaptic functional regulator FMR1 isoform X5
AAEL02588	NA	0.0193566468936258	0.85747712167818	0.964980728557884	NA	NA	NA	XP_019552691.1 lysozyme-like
AAEL01177	alpha-soluble NSF attachment protein	0.0194774998492652	0.855901855083855	0.964980728557884	NA	NA	KFB35002.1 AGAP00	XP_001661903.1alpha-soluble NSF attachment protein
AAEL00215	ribonuclease P protein subunit p40	0.0196663617290289	0.878326875654576	0.969652664055335	NA	aag03013 ; aag	KXJ69691.1 hypothe	XP_001654809.1ribonuclease P protein subunit p40 isoform X2
AAEL02223	NA	0.0197558518093921	0.951587105641683	0.988479552477666	NA	aag04142	NA	XP_021713253.1sulfatase-modifying factor 1 isoform X1
AAEL00346	cullin-4A	0.0198533399434377	0.854841178385688	0.964980728557884	NA	aag04120 ; aag	ETN61630.1 cullin [A	XP_019539059.1 cullin-4A
AAEL00494	probable cytochrome P450 6g2	0.0200194394548203	0.857801413493762	0.964980728557884	NA	NA	KXJ70812.1 hypothe	XP_001650095.3probable cytochrome P450 6g2 isoform X2
AAEL01971	NA	0.0200976316330512	0.926933007931972	0.982472473084084	00562 ; 04070	aag01100 ; aag	NA	XP_021702740.11-phosphatidylinositol 4,5-bisphosphate phosphodiesterase classes I and II isoform X1
AAEL00406	28S ribosomal protein S14, mitochondrial	0.0201154601000072	0.869266148694336	0.968652831735134	NA	aag03010	KFB35490.1 AGAP00	XP_001648358.128S ribosomal protein S14, mitochondrial
AAEL01068	dymeclin	0.0201459450353179	0.872549339053397	0.969029518523332	NA	NA	KXJ73999.1 hypothe	XP_001661020.1dymeclin isoform X2
AAEL02531	NA	0.020190183097368	0.907641758847134	0.976056966800435	NA	NA	NA	NA
AAEL00981	PP2C-like domain-containing protein CG9801	0.0202223823280896	0.907250184737345	0.976056966800435	04660 ; 04658	NA	NA	XP_001660435.1PP2C-like domain-containing protein CG9801
AAEL01165	probable asparagine--tRNA ligase, mitochondrial	0.0202704798080724	0.941122980948316	0.986042559017697	970	aag00970	KXJ71003.1 hypothe	XP_001661824.1probable asparagine--tRNA ligase, mitochondrial
AAEL01370	DNA-directed RNA polymerase II subunit RPB11	0.0202831390006236	0.910911240723479	0.976946997335368	NA	aag03020	NA	XP_019547096.1 DNA-directed RNA polymerase II subunit RPB11
AAEL00158	protein FAM91A1	0.020319239272703	0.887951926091645	0.970996238637524	NA	NA	KXJ74219.1 hypothe	XP_001653556.1protein FAM91A1
AAEL00765	histidine-rich glycoprotein	0.0204593166388603	0.850778532777888	0.964536838405106	NA	aag04350	KFB38866.1 AGAP00	XP_001658559.1histidine-rich glycoprotein
AAEL00823	segment polarity protein dishevelled	0.0205798045874295	0.906721200620858	0.975993693196454	NA	aag04150 ; aag	XP_019545266.1 PRE	XP_021709135.1segment polarity protein dishevelled isoform X4
AAEL02082	NA	0.0205805626283664	0.849586865556956	0.96410141039032	NA	NA	NA	XP_021707527.1E3 ubiquitin-protein ligase UBR1
AAEL00444	probable ATP-dependent RNA helicase DDX56	0.0206469695053776	0.848111900208511	0.963435772275933	NA	NA	CRK92134.1 CLUMA_	XP_021704882.1probable ATP-dependent RNA helicase DDX56
AAEL00597	adenine phosphoribosyltransferase	0.0206684699068881	0.864033042679083	0.967038506393142	230	aag01100 ; aag	KXJ83185.1 hypothe	XP_001664324.1adenine phosphoribosyltransferase
AAEL00162	dual specificity mitogen-activated protein kinase kinase 3	0.0207729111215911	0.869478528922037	0.968652831735134	NA	aag04013 ; aag	XP_019563137.1 PRE	XP_001659756.2dual specificity mitogen-activated protein kinase kinase 3 isoform X1
AAEL00261	general odorant-binding protein 56d	0.0207938225165663	0.942502068052677	0.986630408149377	NA	NA	XP_001863133.1 odc	XP_001655721.2general odorant-binding protein 56d
AAEL00367	gastrula zinc finger protein XlCGF26.1	0.0210241037567567	0.959010996937038	0.989441920233345	NA	NA	KXJ74993.1 hypothe	XP_001657160.2gastrula zinc finger protein XlCGF26.1
AAEL00613	tubulin-specific chaperone A	0.0210576469886789	0.886945788868212	0.9709344693273	NA	NA	KXJ80450.1 hypothe	XP_001657525.1tubulin-specific chaperone A isoform X2
AAEL01294	uncharacterized LOC5577032	0.0210908154479672	0.870556350340702	0.968899314929262	NA	NA	NA	XP_001663126.1uncharacterized protein LOC5577032 isoform X1
AAEL02349	NA	0.0211047266661622	0.878350094854592	0.969652664055335	NA	aag03010	NA	XP_021697651.128S ribosomal protein S17, mitochondrial
AAEL00093	F-box/LRR-repeat protein 20	0.0211230182645533	0.945550319913667	0.987398325447749	NA	NA	KFB49850.1 AGAP01	XP_001657490.1F-box/LRR-repeat protein 20
AAEL00882	IQ domain-containing protein G	0.0211251600374339	0.934325098190231	0.98347583693566	NA	NA	KXJ75030.1 hypothe	XP_001659500.2IQ domain-containing protein G
AAEL01006	T-complex protein 1 subunit gamma	0.0211865217338868	0.843830115743681	0.963198678766584	NA	NA	NA	XP_001660595.1T-complex protein 1 subunit gamma
AAEL01084	ubiquitin-conjugating enzyme E2 J2	0.0212051303208228	0.870949032500264	0.969029518523332	NA	aag04141 ; aag	KFB47151.1 AGAP00	XP_001661087.1ubiquitin-conjugating enzyme E2 J2
AAEL00650	pre-rRNA-processing protein TSR2 homolog	0.0212424069045611	0.891535269603473	0.972487855724415	NA	NA	KXJ76510.1 hypothe	XP_001651995.1pre-rRNA-processing protein TSR2 homolog
AAEL02734	NA	0.0212553299845037	0.878194576455963	0.969652664055335	NA	NA	NA	XP_021707489.1ubiquitin carboxyl-terminal hydrolase 32 isoform X1
AAEL00518	uncharacterized LOC5566124	0.0212591990417774	0.959271599691719	0.989441920233345	NA	NA	XP_315867.3 AGAP0	XP_001650486.2uncharacterized protein LOC5566124
AAEL00314	inositol polyphosphate 5-phosphatase OCRL-1	0.0214654970598533	0.86282052341532	0.966528259617591	04070 ; 00562	aag01100 ; aag	KXJ72221.1 hypothe	XP_021709736.1inositol polyphosphate 5-phosphatase OCRL-1
AAEL00968	farnesol dehydrogenase-like	0.0214944665531991	0.851891832431807	0.964536838405106	NA	NA	NA	XP_001653943.1farnesol dehydrogenase-like
AAEL00562	exostosin-2	0.0216210858906444	0.898301011755693	0.973750254467651	NA	aag01100 ; aag	KXJ72841.1 hypothe	XP_019545638.1 exostosin-2-like
AAEL00722	cysteine protease ATG4B	0.0216839637322631	0.925412752215834	0.982258702730868	NA	aag04140 ; aag	KXJ83145.1 hypothe	XP_001658259.2cysteine protease ATG4B
AAEL00365	uncharacterized LOC5578774	0.021753050323714	0.844034097178004	0.963198678766584	NA	NA	KXJ75002.1 hypothe	XP_001657142.2uncharacterized protein LOC5578774
AAEL00283	protein angel homolog 2	0.0218016783184677	0.895778364572854	0.97325535773105	NA	NA	XP_019552500.1 PRE	XP_001656004.2protein angel homolog 2 isoform X1
AAEL00023	uncharacterized LOC5572041	0.0218280028738257	0.861421128434046	0.96610329632302	NA	NA	NA	XP_001660149.2uncharacterized protein LOC5572041
AAEL01049	oocyte zinc finger protein XlCOF6	0.0220331370207776	0.945987642483299	0.987518968073456	NA	NA	NA	XP_021703661.1oocyte zinc finger protein XlCOF6 isoform X1
AAEL00563	uncharacterized LOC5566765	0.0220667457732765	0.938637528870706	0.985210738070975	NA	NA	KXJ80629.1 hypothe	XP_021701470.1uncharacterized protein LOC5566765
AAEL01226	uncharacterized LOC5576029	0.0221633724047224	0.841060568137285	0.963198678766584	NA	NA	NA	XP_019563615.1 embigin isoform X1
AAEL00688	multiple C2 and transmembrane domain-containing protein 1	0.0222677606197923	0.937496808996192	0.984619988774498	NA	NA	XP_019553820.1 PRE	XP_001652305.2multiple C2 and transmembrane domain-containing protein 1 isoform X1
AAEL00033	putative zinc finger protein 727	0.0224655248805609	0.939026383961161	0.985506476415443	NA	NA	XP_001845496.1 con	XP_001655543.1putative zinc finger protein 727
AAEL00521	proton-coupled amino acid transporter-like protein pathetic	0.022499020998057	0.853844013578032	0.964653958545747	NA	NA	XP_001843326.1 con	XP_021695372.1proton-coupled amino acid transporter-like protein pathetic
AAEL00611	uncharacterized LOC5567509	0.0225091223528363	0.845415121343313	0.963198678766584	NA	NA	KXJ79723.1 hypothe	XP_021693418.1uncharacterized protein LOC5567509
AAEL00252	neutral and basic amino acid transport protein rBAT	0.0226472523507095	0.854431233077494	0.964859528176068	NA	aag04150	XP_019538419.1 PRE	XP_001655437.2neutral and basic amino acid transport protein rBAT isoform X2
AAEL01181	regulator of nonsense transcripts 1 homolog	0.0226506897927781	0.838770240058819	0.962389494890431	NA	aag03013 ; aag	KXJ68805.1 hypothe	XP_001661931.2regulator of nonsense transcripts 1 homolog
AAEL02757	NA	0.0227628041473414	0.831491338172876	0.958742264774772	NA	NA	NA	NA
AAEL00276	nuclear receptor subfamily 2 group F member 1-B	0.0228811664974737	0.892653667097498	0.972739913152176	NA	NA	XP_019532830.1 PRE	XP_021704716.1nuclear receptor subfamily 2 group F member 1-B

AAEL00276	dihydrolipoyllysine-residue succinyltransferase component of 2-oxogluta	0.0229002468224779	0.854123158933053	0.964756573145985	00310 ; 00020 ; 00380	aag01100 ; aag	KXJ77740.1 hypothet	XP_011493660.2dihydrolipoyllysine-residue succinyltransferase component of 2-oxoglutarate dehydrogenase complex mitochondrial
AAEL00506	S-phase kinase-associated protein 2	0.0229309082342036	0.89542042181945	0.973074772918953	NA	aag04120 ; aag	KXJ83935.1 hypothet	XP_001650323.1S-phase kinase-associated protein 2
AAEL00775	cytochrome c oxidase subunit 7A, mitochondrial	0.0229343033909732	0.848687427051424	0.963483240428003	NA	aag01100 ; aag	KFB49000.1 AGAP00	XP_001658629.1cytochrome c oxidase subunit 7A, mitochondrial
AAEL00250	glutamate receptor ionotropic, kainate 2	0.0229854441195125	0.847099719115806	0.963198678766584	NA	NA	XP_019526260.1 PRE	XP_001655464.2glutamate receptor ionotropic, kainate 2
AAEL00725	zinc finger protein 2 homolog	0.0230321723474647	0.925592863233139	0.982258702730868	NA	NA	KXJ76944.1 hypothet	XP_001652629.1zinc finger protein 2 homolog
AAEL00942	protein peste	0.0230755921813219	0.900988068532653	0.974252903247646	NA	NA	NA	XP_001660043.1protein peste
AAEL01999	NA	0.0231591950456089	0.899475087571332	0.97393667311859	NA	NA	NA	XP_019564505.1 homeotic protein female sterile
AAEL01362	ras-related protein Rab-35	0.0232361687868635	0.8760776070845	0.969652664055335	NA	NA	NA	XP_001656883.2ras-related protein Rab-35 isoform X1
AAEL00669	60S ribosomal protein L31	0.023237244933903	0.872930974091448	0.969029518523332	NA	aag03010	AAV90720.1 60S ribo	XP_001657955.160S ribosomal protein L31
AAEL01211	aminopeptidase N	0.02325874482541	0.841527382450045	0.963198678766584	NA	NA	KXJ78002.1 hypothet	XP_001655856.2aminopeptidase N isoform X1
AAEL00442	zinc finger CCHC domain-containing protein 4	0.0232694580141983	0.93666081050784	0.984371034524114	NA	NA	XP_005186567.1 PRE	XP_001649112.1zinc finger CCHC domain-containing protein 4
AAEL00756	dual oxidase maturation factor 1	0.0233322339612165	0.908933766425247	0.976875556954581	NA	NA	ETN58938.1 moladie	XP_001658450.1dual oxidase maturation factor 1
AAEL02728	NA	0.0234277600768474	0.934522334555357	0.98347583693566	NA	NA	NA	XP_021694845.1alpha-ketoglutarate-dependent dioxygenase alkB homolog 7, mitochondrial
AAEL00311	leucine-rich repeat neuronal protein 2	0.023437515303133	0.946930456176613	0.987707093985696	NA	NA	KXJ69248.1 hypothet	XP_021711247.1leucine-rich repeat neuronal protein 2
AAEL00289	B-cell CLL/lymphoma 7 protein family member B-B	0.0234956240318785	0.857519791349169	0.964980728557884	NA	NA	KFB36773.1 AGAP00	XP_021700479.1B-cell CLL/lymphoma 7 protein family member B-B
AAEL01139	39S ribosomal protein L9, mitochondrial	0.0235742032827274	0.871326496954978	0.969029518523332	NA	aag03010	KXJ71322.1 hypothet	XP_001655318.139S ribosomal protein L9, mitochondrial
AAEL02759	NA	0.0240417333879983	0.83349000747389	0.959902797120062	62	aag01100 ; aag	NA	XP_021706249.1very-long-chain (3R)-3-hydroxyacyl-CoA dehydratase 2 isoform X2
AAEL00158	cytosolic non-specific dipeptidase	0.0240421716349907	0.846975720651098	0.963198678766584	NA	aag01100 ; aag	ETN61410.1 glutama	XP_001653563.1cytosolic non-specific dipeptidase
AAEL01218	glutaminyl-peptide cyclotransferase	0.0240544230511349	0.886685784286906	0.9709344693273	NA	NA	KXJ76435.1 hypothet	XP_001662292.2glutaminyl-peptide cyclotransferase
AAEL00253	zinc finger protein 62	0.0241564861370458	0.921824526485273	0.981104475215994	NA	NA	KXJ70567.1 hypothet	XP_021697749.1zinc finger protein 62
AAEL01705	NA	0.0241806216568195	0.913919271038789	0.977828474226146	NA	NA	NA	XP_021701735.1zinc finger protein Xfin isoform X1
AAEL01173	NF-kappa-B-repressing factor	0.0243419359245824	0.884723529728128	0.970521528629931	NA	NA	KXJ72931.1 hypothet	XP_001661884.1NF-kappa-B-repressing factor
AAEL00943	protein split ends	0.0244544020407161	0.859648363373082	0.965722450478577	NA	NA	NA	XP_021700435.1protein split ends isoform X6
AAEL01947	NA	0.024471929721428	0.94613268933297	0.987558190476633	NA	NA	NA	XP_021697076.1breast cancer anti-estrogen resistance protein 1 isoform X2
AAEL00242	endoplasmic reticulum metallopeptidase 1	0.0245066174896911	0.838569649077433	0.962348963212321	NA	NA	XP_001864515.1 con	XP_001655228.2endoplasmic reticulum metallopeptidase 1
AAEL00817	biogenesis of lysosome-related organelles complex 1 subunit 1	0.0245092662264071	0.926824961771956	0.982472473084084	NA	NA	ETN58392.1 RT14 pr	XP_001658991.1biogenesis of lysosome-related organelles complex 1 subunit 1
AAEL00838	tudor domain-containing protein 3	0.0245514785461037	0.877423468011749	0.969652664055335	NA	NA	KXJ75960.1 hypothet	XP_021704717.1tudor domain-containing protein 3
AAEL00532	NA	0.0246019128170274	0.817818054304281	0.952514760254484	NA	NA	ETN58760.1 hypothe	XP_021696745.1uncharacterized protein LOC5566341
AAEL02379	NA	0.0246106580932731	0.932937063590419	0.98347583693566	NA	NA	NA	XP_021703189.1leucine-rich repeat-containing protein 40-like
AAEL00836	serine protease inhibitor 88Ea	0.0247210876980765	0.830319015949639	0.958428949419039	NA	NA	KXJ75722.1 hypothet	XP_021704939.1serine protease inhibitor 88Ea isoform X1
AAEL00137	coiled-coil domain-containing protein 6	0.0247243550439563	0.840547120713899	0.963198678766584	NA	NA	XP_001843899.1 con	XP_001653153.1coiled-coil domain-containing protein 6
AAEL01069	ribonucleoside-diphosphate reductase subunit M2	0.0248023442725966	0.859920988811503	0.965722450478577	00983 ; 00230 ; 00480 ;	aag01100 ; aag	AAD30422.1 ribonuc	XP_001654749.2ribonucleoside-diphosphate reductase subunit M2 isoform X2
AAEL01966	NA	0.024866972268029	0.951333299063734	0.988479552477666	NA	NA	NA	XP_021693914.1zinc finger protein 260
AAEL00260	serine protease 42	0.0248674141335275	0.831103951664739	0.958742264774772	NA	NA	XP_001868413.1 seri	XP_021704385.1serine protease 42 isoform X1
AAEL00583	uridine phosphorylase 1	0.0248993155750847	0.843558724246905	0.963198678766584	00983 ; 00240	aag01100 ; aag	XP_019537879.1 PRE	XP_001651486.2uridine phosphorylase 1 isoform X1
AAEL00739	uncharacterized LOC5569140	0.0250528686913454	0.853156647911957	0.964653958545747	NA	NA	NA	ABF18061.1short salivary D7 protein
AAEL00727	NA	0.0250654073057902	0.86147973658938	0.96610329632302	NA	NA	KXJ69282.1 hypothet	XP_001658269.2 NADPH-dependent aldo-keto reductase, chloroplastic
AAEL01165	zinc finger HIT domain-containing protein 2	0.0250809690699043	0.920311202645168	0.980340877547081	NA	NA	KXJ69577.1 hypothet	XP_001661820.1zinc finger HIT domain-containing protein 2
AAEL00029	V-type proton ATPase 16 kDa proteolipid subunit	0.0250855338822596	0.839407067786155	0.962903919781775	NA	aag01100 ; aag	ABV60304.1 putative	XP_001654757.1V-type proton ATPase 16 kDa proteolipid subunit
AAEL01453	inhibitor of Bruton tyrosine kinase	0.0251008868329557	0.896972590930438	0.973750254467651	NA	NA	NA	XP_021701962.1inhibitor of Bruton tyrosine kinase
AAEL00374	cytochrome P450 9e2	0.0251377901200812	0.906559123965764	0.975993693196454	NA	NA	KXJ75238.1 hypothet	XP_001657206.1cytochrome P450 9e2
AAEL00414	tetraspanin-33	0.025245051363951	0.893291491046704	0.972739913152176	NA	NA	KFB42358.1 AGAP00	XP_019553001.1 tetraspanin-33-like
AAEL00787	anaphase-promoting complex subunit 10-like	0.0253392607770671	0.933055805189223	0.98347583693566	NA	aag04120	XP_001855904.1 ana	XP_021708664.1anaphase-promoting complex subunit 10-like
AAEL00688	growth arrest and DNA damage-inducible protein GADD45 alpha	0.0255988035279773	0.875238953091934	0.969652664055335	NA	aag04068	XP_001862887.1 con	XP_001652310.1growth arrest and DNA damage-inducible protein GADD45 alpha
AAEL02375	NA	0.0256565254354072	0.89813623713486	0.973750254467651	NA	NA	NA	XP_021699868.1proteoglycan 4
AAEL00937	uncharacterized protein CG43867	0.0256758007967082	0.895321600960447	0.973074772918953	NA	NA	NA	XP_021704744.1uncharacterized protein CG43867 isoform X7
AAEL00266	4-coumarate--CoA ligase 1	0.0256992882604529	0.899474924282211	0.97393667311859	NA	NA	KXJ80799.1 hypothet	XP_001662046.14-coumarate--CoA ligase 1
AAEL00975	TP53-regulated inhibitor of apoptosis 1	0.0257155564126706	0.905981059985848	0.975993693196454	NA	NA	NA	XP_001660329.1TP53-regulated inhibitor of apoptosis 1
AAEL00678	palmitoyltransferase Hip14	0.0257157702328302	0.886238962544138	0.9709344693273	NA	NA	ETN61393.1 Hunting	XP_021711084.1palmitoyltransferase Hip14 isoform X2
AAEL00383	dihydroceramide fatty acyl 2-hydroxylase FAH2	0.0257742909142053	0.897332497234504	0.973750254467651	NA	NA	KXJ80223.1 hypothet	XP_001657313.2dihydroceramide fatty acyl 2-hydroxylase FAH2
AAEL01274	uncharacterized LOC5576751	0.0257785213634747	0.833479979155574	0.959902797120062	NA	NA	NA	XP_021708356.1uncharacterized protein LOC5576751 isoform X2
AAEL01990	NA	0.0257976833688585	0.851600207731576	0.964536838405106	NA	NA	NA	XP_001654214.1uncharacterized protein LOC5572813
AAEL00611	cystinosin homolog	0.025825772943126	0.853274204310171	0.964653958545747	NA	aag04142	XP_019531955.1 PRE	XP_001651807.1cystinosin homolog isoform X3
AAEL00169	charged multivesicular body protein 4b	0.0258644998306084	0.811883606656725	0.950261122927395	NA	aag04144	ETN65923.1 charged	XP_001659957.1charged multivesicular body protein 4b
AAEL00817	telomerase Cajal body protein 1	0.0259552398105884	0.933457781035043	0.98347583693566	NA	NA	KXJ84061.1 hypothet	XP_001658989.1telomerase Cajal body protein 1
AAEL00059	E3 ubiquitin-protein ligase MYLIP	0.0260216393204824	0.835721494822363	0.961379090928137	NA	NA	KXJ79613.1 hypothet	XP_001648670.1E3 ubiquitin-protein ligase MYLIP
AAEL00295	uncharacterized LOC5576729	0.026079195271122	0.829717163911214	0.958094785081062	NA	NA	KXJ80794.1 hypothet	XP_001656237.1uncharacterized protein LOC5576729
AAEL01311	GPI inositol-deacylase	0.0261630337656956	0.872799775502179	0.969029518523332	NA	aag01100 ; aag	NA	XP_021705012.1GPI inositol-deacylase
AAEL00395	cofilin/actin-depolymerizing factor homolog	0.0262728435963131	0.81728811906492	0.952514760254484	NA	NA	CRL08088.1 CLUMA_	XP_001648107.1cofilin/actin-depolymerizing factor homolog
AAEL00144	lysophosphatidylcholine acyltransferase	0.0263983399263708	0.826678531455324	0.956332647735617	NA	aag01100 ; aag	KXJ72411.1 hypothet	XP_021700994.1lysophosphatidylcholine acyltransferase isoform X1
AAEL01057	uncharacterized LOC5573521	0.0265488796488619	0.928279008524765	0.982756983779826	NA	NA	NA	XP_021703454.1uncharacterized protein LOC110677080
AAEL01244	baculoviral IAP repeat-containing protein 6	0.0266424677569001	0.812750154292893	0.950429142178574	NA	aag04120 ; aag	XP_019540457.1 PRE	XP_021713190.1baculoviral IAP repeat-containing protein 6 isoform X2
AAEL00154	ubiquitin carboxyl-terminal hydrolase 31	0.0268411530358275	0.847161717471438	0.963198678766584	NA	NA	XP_019537407.1 PRE	XP_001653586.2ubiquitin carboxyl-terminal hydrolase 31
AAEL01834	NA	0.0268437070211253	0.868307117903255	0.96863134437578	NA	aag04310	NA	XP_021708940.1catenin delta-2
AAEL01147	60S ribosomal protein L17	0.0269498616137202	0.85125863062888	0.964536838405106	NA	aag03010	ETN59901.1 60S ribo	XP_001655390.160S ribosomal protein L17
AAEL00328	serrate RNA effector molecule homolog	0.0271557367406323	0.815103673676194	0.952015623780574	NA	NA	NA	XP_001663544.1serrate RNA effector molecule homolog isoform X4
AAEL01983	NA	0.0271827325391634	0.913555195668502	0.977731438769662	NA	NA	NA	XP_021697130.1protein boule isoform X1
AAEL00769	DNA polymerase alpha subunit B	0.0271981522557657	0.886736129069902	0.9709344693273	NA	aag03030	KXJ71173.1 hypothet	XP_021695253.1DNA polymerase alpha subunit B
AAEL00920	uncharacterized LOC5571609	0.0272090084572466	0.835897822043233	0.961379090928137	NA	NA	NA	ABF18418.1hypothetical conserved protein
AAEL01110	centromere/kinetochore protein zw10	0.0274045322485775	0.877266109896078	0.969652664055335	NA	NA	XP_001868647.1 cen	XP_021700099.1centromere/kinetochore protein zw10
AAEL01285	uncharacterized LOC5576907	0.0274161837627948	0.856945876041642	0.964980728557884	NA	NA	NA	XP_001663006.2uncharacterized protein LOC5576907
AAEL01339	serine/threonine-protein kinase polo	0.0275190064497164	0.846251509109541	0.963198678766584	NA	aag04068	NA	XP_001663586.1serine/threonine-protein kinase polo isoform X1
AAEL00778	protein artichoke	0.027595277300212	0.910607669200579	0.97691422813284	NA	NA	XP_001849247.1 leu	XP_021708237.1protein artichoke
AAEL02057	NA	0.0276087707677779	0.923898964174408	0.981722663779781	NA	NA	NA	XP_021709985.1uncharacterized protein LOC110679499
AAEL01096	uncharacterized protein C9orf78	0.027610603618685	0.888853474651135	0.971195739725201	NA	NA	ETN59055.1 hypothe	XP_001655062.1uncharacterized protein C9orf78
AAEL02119	NA	0.0276131548671419	0.930832237576834	0.98347583693566	NA	NA	NA	XP_021708917.1succinate dehydrogenase assembly factor 2, mitochondrial
AAEL00278	galactokinase	0.0277077638407986	0.80699901013186	0.948040966581563	00052 ; 00520	aag01100 ; aag	XP_019537340.1 PRE	XP_021705151.1galactokinase isoform X1
AAEL00949	40S ribosomal protein S7	0.0277178876503086	0.85576960583801	0.964980728557884	NA	aag03010	NA	XP_001660169.140S ribosomal protein S7
AAEL00808	bifunctional arginine demethylase and lysyl-hydroxylase PSR	0.0278413763987913	0.838315588767637	0.962348963212321	NA	NA	KXJ71599.1 hypothet	XP_021693147.1bifunctional arginine demethylase and lysyl-hydroxylase PSR
AAEL01993	NA	0.0279265696078693	0.817750667747505	0.952514760254484	NA	aag04146 ; aag	NA	XP_001654772.1superoxide dismutase [Cu-Zn]
AAEL01818	NA	0.0280417461895074	0.842449336438113	0.963198678766584	NA	NA	NA	XP_021709044.1uncharacterized protein LOC5572229
AAEL00612	deoxyhypusine hydroxylase	0.0281302296154491	0.862859123316508	0.966528259617591	NA	NA	XP_001845226.1 dec	XP_001657523.1deoxyhypusine hydroxylase

AAEL02141	NA	0.0281322541094555	0.956662532278838	0.989441920233345	NA	NA	NA	XP_021692952.1tail-anchored protein insertion receptor WRB
AAEL00712	protein PET117 homolog, mitochondrial	0.0281758504237563	0.885495098178884	0.970789723835142	NA	NA	XP_001849747.1 PET	XP_001658150.2protein PET117 homolog, mitochondrial
AAEL01409	probable tRNA (uracil-O(2)-)-methyltransferase	0.0282141999766504	0.940689675340187	0.986015080587812	NA	NA	NA	XP_021697833.1probable tRNA (uracil-O(2)-)-methyltransferase
AAEL00255	uncharacterized LOC5580174	0.0282219357202599	0.870217775791867	0.968756831942506	NA	NA	KXJ72380.1 hypothet	XP_001655579.2uncharacterized protein LOC5580174
AAEL00816	fumarate hydratase, mitochondrial	0.0282552011256005	0.825130415423104	0.955377826472606	00020 ; 00720 ; 00620	aag01100 ; aag	KXJ84062.1 hypothet	XP_001658987.1fumarate hydratase, mitochondrial isoform X1
AAEL02222	NA	0.0282859957635009	0.939945322283211	0.985798796572901	NA	NA	NA	XP_021694015.1uncharacterized protein LOC5565165
AAEL01138	mitochondrial cardiolipin hydrolase	0.0283375004162446	0.929829965206945	0.98347583693566	NA	NA	KXJ79673.1 hypothet	XP_021708813.1mitochondrial cardiolipin hydrolase
AAEL00882	carbonic anhydrase 2	0.0284279703985021	0.870015940029942	0.968652831735134	NA	aag01100 ; aag	KFB39570.1 AGAP00	XP_001659576.1carbonic anhydrase 2
AAEL02766	NA	0.0285007890324877	0.877289573894606	0.969652664055335	NA	NA	NA	XP_001659733.2zinc finger CCHC domain-containing protein 8 homolog
AAEL00390	ubiquitin conjugation factor E4 A	0.0286874428085578	0.822790530683396	0.954452060294489	NA	aag04120	KXJ80153.1 hypothet	XP_021693240.1ubiquitin conjugation factor E4 A
AAEL01476	zinc transporter ZIP13 homolog	0.0287584351256577	0.813222273792987	0.950739596422699	NA	NA	NA	XP_021713167.1zinc transporter ZIP13 homolog
AAEL00728	dynamin	0.0287863920216287	0.81293553649456	0.950525134284999	NA	aag04144	XP_019546242.1 PRE	XP_001652669.1dynamin isoform X2
AAEL00590	splicing factor 3B subunit 6-like protein	0.0288195255457596	0.890564893987172	0.972299099904304	NA	aag03040	ETN67867.1 pre-mRN	XP_001651583.1splicing factor 3B subunit 6-like protein
AAEL00304	uncharacterized LOC5576894	0.0288901998194057	0.811329290544954	0.95010454605765	NA	aag04142	XP_001864689.1 sap	XP_021703285.1uncharacterized protein LOC5576894
AAEL00202	chitinase-like protein Idgf4	0.0288915045140711	0.800575891728655	0.946215055875588	NA	NA	XP_001844673.1 ima	XP_001660748.2chitinase-like protein Idgf4
AAEL00921	longitudinals lacking protein, isoforms A/B/D/L	0.0290440576877935	0.803255270277697	0.94649029363412	NA	NA	NA	XP_001659825.1longitudinals lacking protein, isoforms H/M/V isoform X12
AAEL00147	uncharacterized protein PB18E9.04c	0.0290497402981128	0.937083811118422	0.984590686219652	NA	NA	KXJ70366.1 hypothet	XP_001653389.1uncharacterized protein PB18E9.04c
AAEL02525	NA	0.0290991704676896	0.919994505566599	0.980340877547081	NA	NA	NA	XP_021693241.1leucine-rich repeat-containing protein 24
AAEL01064	trifunctional purine biosynthetic protein adenosine-3	0.0291167770096504	0.850557470381841	0.964536838405106	00230 ; 00970 ; 00670 ;	aag01100 ; aag	KXJ68564.1 hypothet	XP_001660978.1trifunctional purine biosynthetic protein adenosine-3
AAEL02666	NA	0.0291288315547699	0.853447680850618	0.964653958545747	NA	NA	NA	XP_021705129.1bromodomain adjacent to zinc finger domain protein 1A
AAEL01109	uncharacterized LOC5574380	0.0291973377565185	0.879872427445176	0.969652664055335	NA	NA	KFB37508.1 AGAP00	KFB37508.1AGAP004466-like protein
AAEL00337	small G protein signaling modulator 3 homolog	0.0292181946136445	0.905213163795421	0.975993693196454	NA	NA	KXJ68704.1 hypothet	XP_001656725.1small G protein signaling modulator 3 homolog
AAEL00565	ethanolamine-phosphate cytidylyltransferase	0.0292413527058645	0.799400331730097	0.946215055875588	NA	aag01100 ; aag	XP_019530276.1 PRE	XP_001651208.1ethanolamine-phosphate cytidylyltransferase isoform X3
AAEL00963	exportin-7	0.0294098416643921	0.80274365822174	0.94649029363412	NA	NA	NA	XP_021713348.1exportin-7 isoform X1
AAEL01709	elongation factor 1-alpha	0.0294360636064276	0.848453090138382	0.963483240428003	NA	aag03013	NA	XP_011493435.1elongation factor 1-alpha
AAEL01978	NA	0.0295857467192005	0.785977533141262	0.944484065495843	NA	aag03013	NA	XP_021698824.1eukaryotic translation initiation factor 5B isoform X2
AAEL00390	uncharacterized LOC5579183	0.0297346252103871	0.803487645789533	0.94649029363412	NA	NA	KXJ82112.1 hypothet	XP_021709131.1uncharacterized protein LOC5579183
AAEL00494	zinc finger protein 729	0.0297477253815464	0.896302953415378	0.973419069671699	NA	NA	KXJ67823.1 hypothet	XP_021705323.1zinc finger protein 729
AAEL01021	mediator of RNA polymerase II transcription subunit 29	0.0297919812510753	0.883640029712406	0.970521528629931	NA	NA	NA	XP_001654364.1mediator of RNA polymerase II transcription subunit 29 isoform X1
AAEL00548	sorting nexin-27	0.0297999769786216	0.879629135822296	0.969652664055335	NA	NA	ETN58601.1 sorting r	XP_001650959.1sorting nexin-27 isoform X1
AAEL01238	uncharacterized LOC5576199	0.0298548420375052	0.823099344777823	0.954452060294489	NA	NA	KXJ72121.1 hypothet	XP_001662538.1uncharacterized protein LOC5576199
AAEL00278	39S ribosomal protein L37, mitochondrial	0.0298868706598587	0.882379549190285	0.970357617045106	NA	NA	KXJ77324.1 hypothet	XP_001662478.239S ribosomal protein L37, mitochondrial
AAEL02417	NA	0.0299179417949598	0.931731081081132	0.98347583693566	NA	NA	NA	XP_021709834.1WSCD family member AAEL009094
AAEL01082	sodium-independent sulfate anion transporter	0.0299367228391765	0.873105421813118	0.969040657523135	NA	NA	KXJ80875.1 hypothet	XP_001654997.1sodium-independent sulfate anion transporter
AAEL01490	serine/threonine-protein kinase ATM	0.0299933674051451	0.883588880696762	0.970521528629931	04151 ; 05165 ; 04714 ;	aag04068 ; aag	NA	XP_021700538.1serine/threonine-protein kinase ATM isoform X1
AAEL01206	probable phospholipid hydroperoxide glutathione peroxidase	0.0300693493045122	0.784178325386442	0.944372632355572	NA	aag01100 ; aag	KXJ76355.1 hypothet	XP_021705458.1probable phospholipid hydroperoxide glutathione peroxidase isoform X2
AAEL01094	probable 18S rRNA (guanine-N(7))-methyltransferase	0.0301295276668879	0.881488514980702	0.970037947243772	NA	NA	ETN60614.1 williams	XP_001661209.1probable 18S rRNA (guanine-N(7))-methyltransferase
AAEL00734	lipopolysaccharide-induced tumor necrosis factor-alpha factor homolog	0.0301374191970826	0.805145817546505	0.947029572155807	NA	NA	KFB42948.1 AGAP00	XP_001652694.2lipopolysaccharide-induced tumor necrosis factor-alpha factor homolog
AAEL02055	NA	0.0301470191705995	0.794034117038549	0.945793949302124	NA	aag04933	NA	XP_021695034.1signal transducer and transcription activator isoform X5
AAEL00804	ammonium transporter Rh type A	0.030238791667 7674	0.87574393920107	0.969652664055335	NA	NA	KXJ71166.1 hypothet	XP_001658851.2ammonium transporter Rh type A isoform X3
AAEL01442	serine-rich adhesin for platelets	0.0302681269230722	0.906164228350722	0.975993693196454	NA	NA	NA	XP_021708027.1uncharacterized protein LOC5564417 isoform X1
AAEL00672	ubiquitin-conjugating enzyme E2 C	0.0302775978051461	0.792205428616044	0.945793949302124	NA	aag04120	ETN64278.1 ubiquitin	XP_001652173.1ubiquitin-conjugating enzyme E2 C
AAEL00179	protein tyrosine phosphatase type IVA 1	0.0303308255056834	0.788671899617905	0.945232821987723	NA	NA	KXJ74397.1 hypothet	XP_001660410.1protein tyrosine phosphatase type IVA 1 isoform X1
AAEL00068	transport and Golgi organization protein 6	0.0303833856784483	0.908014490461068	0.976343693493138	NA	NA	KXJ82444.1 hypothet	XP_001650247.2transport and Golgi organization protein 6
AAEL00546	zinc finger CCCH domain-containing protein 18	0.0303867203027504	0.858365967348463	0.965255240320244	NA	NA	XP_019539749.1 PRE	XP_001650929.2zinc finger CCCH domain-containing protein 18
AAEL00112	REST corepressor	0.0304561486166048	0.801067537853186	0.946215055875588	NA	NA	KXJ73079.1 hypothet	XP_001652381.1REST corepressor isoform X2
AAEL00783	protein FAM107B	0.0305063740451145	0.858848801802732	0.965328928656042	NA	NA	XP_001869806.1 con	XP_021699826.1protein FAM107B isoform X2
AAEL00349	pyridoxal phosphate homeostasis protein	0.0305730761486259	0.790766943291305	0.945793949302124	473	NA	KXJ72990.1 hypothet	XP_021692940.1pyridoxal phosphate homeostasis protein
AAEL00964	dehydrodolichyl diphosphate synthase complex subunit nus1	0.030653720821109	0.788824853645374	0.945274945076797	900	aag00900	NA	XP_001653915.2dehydrodolichyl diphosphate synthase complex subunit nus1
AAEL02442	NA	0.0307225794834901	0.927842831508772	0.982649508778163	NA	aag01100 ; aag	NA	XP_021712271.1GPI transamidase component PIG-T-like
AAEL00995	uncharacterized LOC5572684	0.0307426308210594	0.923435310662612	0.981343310857761	NA	NA	NA	XP_021705774.1uncharacterized protein LOC5572684 isoform X1
AAEL00480	ubiquinone biosynthesis O-methyltransferase, mitochondrial	0.0307998780599907	0.881115153282385	0.969743049934758	130	aag01100 ; aag	KXJ81190.1 hypothet	XP_001649843.1ubiquinone biosynthesis O-methyltransferase, mitochondrial
AAEL00527	rab11 family-interacting protein 5	0.0308315415654196	0.837808200607872	0.962348963212321	NA	aag04144	KXJ83422.1 hypothet	XP_021694475.1rab11 family-interacting protein 5 isoform X1
AAEL01001	cold shock domain-containing protein E1	0.030880809680528	0.77661400520719	0.942167671783845	NA	NA	NA	XP_001660573.2cold shock domain-containing protein E1 isoform X1
AAEL00428	serine/threonine-protein phosphatase 6 catalytic subunit	0.0309308569132283	0.796021740023097	0.945890571556554	04660 ; 04658	NA	KFB40517.1 AGAP00	XP_001648846.1serine/threonine-protein phosphatase 6 catalytic subunit
AAEL01049	ubiquitin carboxyl-terminal hydrolase 14	0.0309566568867125	0.808721001547966	0.948852563591022	NA	NA	NA	XP_021703657.1ubiquitin carboxyl-terminal hydrolase 14
AAEL00286	zinc finger protein 2	0.0310340826737013	0.908238611405569	0.976356507260987	NA	NA	XP_019537154.1 PRE	XP_001656106.2zinc finger protein 2
AAEL01539	NA	0.0311692783278304	0.901423535540675	0.974380285920978	NA	aag04150	NA	XP_001657767.2GATOR complex protein NPRL2
AAEL00773	nedd8-activating enzyme E1 regulatory subunit	0.0312198958651571	0.909974985376621	0.97691422813284	NA	NA	KXJ62460.1 hypothet	XP_001658604.1nedd8-activating enzyme E1 regulatory subunit
AAEL02483	NA	0.0312654063279428	0.85961011843663	0.965722450478577	NA	NA	NA	XP_021693916.1luciferin 4-monooxygenase isoform X1
AAEL02467	NA	0.031431141645354	0.774418018845486	0.941230906670349	NA	NA	NA	XP_021700530.1aquaporin AQPAe.a isoform X1
AAEL01265	PDZ domain-containing protein GIPC3	0.0314446758736442	0.791925678687941	0.945793949302124	NA	NA	KFB36615.1 AGAP00	XP_021695763.1PDZ domain-containing protein GIPC3
AAEL01211	V-type proton ATPase 21 kDa proteolipid subunit	0.0314683775736044	0.773906001632626	0.941230906670349	NA	aag01100 ; aag	KFB39033.1 hypothe	XP_001662256.1V-type proton ATPase 21 kDa proteolipid subunit
AAEL00500	aquaporin AQPAn.G	0.0315608507198785	0.790540467625235	0.945793949302124	NA	NA	XP_019560305.1 PRE	XP_001650168.2aquaporin AQPAn.G isoform X1
AAEL00553	CD63 antigen	0.0316257833410232	0.795992256024678	0.945890571556554	NA	NA	KXJ71418.1 hypothet	XP_001656132.1CD63 antigen
AAEL01322	uncharacterized LOC5577481	0.0316542835857117	0.891858208518727	0.97252322736992	NA	NA	NA	XP_001656511.2uncharacterized protein LOC5577481
AAEL01037	Usher syndrome type-1G protein homolog	0.0316665860225402	0.851962119382385	0.964536838405106	NA	NA	NA	XP_021699042.1Usher syndrome type-1G protein homolog
AAEL01500	proteasome subunit alpha type-1	0.0317291582835923	0.847667840110253	0.963435772275933	NA	aag03050	NA	XP_001650228.1proteasome subunit alpha type-1
AAEL02352	NA	0.0318066924285943	0.828336094808635	0.95710054550842	NA	NA	NA	XP_021709548.1methionine-R-sulfoxide reductase B1 isoform X1
AAEL01469	ATP-binding cassette sub-family A member 1	0.0318378366215476	0.816187090463898	0.952319314767154	NA	NA	NA	XP_021708709.1ATP-binding cassette sub-family A member 1 isoform X1
AAEL00067	NEDD8	0.0318783735356104	0.799702683002864	0.946215055875588	NA	NA	XP_002015294.1 GL1	XP_019544852.1 NEDD8 isoform X1
AAEL00972	uncharacterized LOC5572339	0.0320282090622486	0.925494370953453	0.982258702730868	NA	NA	NA	XP_021713126.1uncharacterized protein LOC5572339
AAEL01505	myrosinase 1	0.0320800011946717	0.855024689358854	0.964980728557884	NA	NA	NA	XP_001650404.2myrosinase 1
AAEL01740	pre-mRNA-splicing factor 38B	0.0321172806625317	0.856470617931207	0.964980728557884	NA	aag03040	NA	XP_021705127.1pre-mRNA-splicing factor 38B isoform X1
AAEL00694	T-complex protein 1 subunit zeta	0.0321280925783826	0.773948362874208	0.941230906670349	NA	NA	KFB41422.1 hypothe	XP_001652354.1T-complex protein 1 subunit zeta
AAEL01977	NA	0.0321726343438246	0.852660871293203	0.964653958545747	NA	NA	NA	XP_021700108.1proteoglycan 4-like
AAEL00926	UV radiation resistance-associated gene protein	0.0321767194118455	0.872476734124469	0.969029518523332	NA	aag04140	NA	XP_001659882.2UV radiation resistance-associated gene protein
AAEL00435	pre-mRNA-splicing factor 38	0.0322836045058719	0.893114548530359	0.972739913152176	NA	aag03040	KXJ75471.1 hypothet	XP_001648995.1pre-mRNA-splicing factor 38
AAEL00093	probable insulin-like peptide 2	0.0323422936834858	0.860499682150054	0.966023193660905	NA	aag04150 ; aag	KXJ69526.1 hypothet	XP_001657487.1probable insulin-like peptide 2 isoform X1
AAEL02441	NA	0.0324669395075099	0.801651732473773	0.946215055875588	NA	NA	NA	XP_021698170.1protein-tyrosine sulfotransferase
AAEL00366	eukaryotic translation initiation factor 6	0.0326819520501235	0.797916071650155	0.946188620016073	NA	aag03008	ETN64241.1 translati	XP_001657106.1eukaryotic translation initiation factor 6
AAEL01946	NA	0.0327646073912931	0.774138137438988	0.941230906670349	NA	NA	NA	XP_021698921.1probable RNA helicase armi

AAEL00796	serine protease snake	0.0327903208598067	0.882814759881772	0.970357617045106	NA	NA	KXJ71168.1 hypothet	XP_001658781.1serine protease snake
AAEL01051	cysteine protease ATG4D	0.0328762614499509	0.896637474610465	0.973667458268291	NA	aag04140 ; aag	NA	XP_001660889.2cysteine protease ATG4D isoform X1
AAEL02565	NA	0.032992601126843	0.882454157638126	0.970357617045106	511	aag00511	NA	XP_021713331.1cytosolic endo-beta-N-acetylglucosaminidase isoform X2
AAEL00071	reticulon-1-A	0.03299927885324	0.776072925540573	0.941891725247211	NA	NA	XP_019561060.1 PRE	XP_001650264.1reticulon-1 isoform X1
AAEL00384	DPH3 homolog	0.033034815887853	0.819059421034442	0.952969210637171	NA	NA	KXJ76070.1 hypothet	XP_021705727.1DPH3 homolog
AAEL00254	probable transcriptional regulatory protein TTE1135	0.0333453180676676	0.831527623232935	0.958742264774772	NA	NA	KXJ81472.1 hypothet	XP_001655587.1uncharacterized protein LOC5580162
AAEL00793	gustatory receptor 20	0.0334400612934335	0.937696624274759	0.984672410883981	NA	NA	XP_019558163.1 PRE	NP_001345205.1gustatory receptor 20 isoform h
AAEL01227	armadillo repeat-containing protein 6 homolog	0.0334638962249475	0.878885358832083	0.969652664055335	NA	NA	KXJ84516.1 hypothet	XP_021698308.1armadillo repeat-containing protein 6 homolog
AAEL01488	dynactin subunit 6	0.0334687475300104	0.915299740189951	0.978301715339473	NA	NA	NA	XP_001663785.1dynactin subunit 6
AAEL02096	NA	0.0334805715514178	0.770924575303173	0.941147080452193	NA	NA	NA	XP_021694639.1zinc carboxypeptidase
AAEL00423	CDGSH iron-sulfur domain-containing protein 2 homolog	0.0335366513880369	0.748927981409121	0.932554085844433	NA	NA	ETN58699.1 CDGSH i	XP_001648745.1CDGSH iron-sulfur domain-containing protein 2 homolog
AAEL00353	sprouty-related, EVH1 domain-containing protein 1	0.0335707606525779	0.850811422733331	0.964536838405106	NA	NA	XP_019552712.1 PRE	XP_021710765.1sprouty-related, EVH1 domain-containing protein 1 isoform X2
AAEL01967	NA	0.0335744747093924	0.848608992473057	0.963483240428003	NA	NA	NA	XP_021710908.1graves disease carrier protein homolog isoform X2
AAEL00791	disks large homolog 5	0.0336046092672845	0.823139703379381	0.954452060294489	NA	NA	KXJ82599.1 hypothet	XP_021704088.1disks large homolog 5 isoform X1
AAEL01202	zinc finger protein 510	0.0336559592647498	0.900944116367557	0.974252903247646	NA	NA	KXJ72265.1 hypothet	XP_001662156.2zinc finger protein 510
AAEL00093	DPH4 homolog	0.0336745431882181	0.900184393558197	0.974065297962641	NA	NA	KXJ70074.1 hypothet	XP_001657497.1DPH4 homolog
AAEL00147	protoheme IX farnesyltransferase, mitochondrial	0.0337184689984813	0.756344909118705	0.934127170333935	860	aag01100 ; aag	KXJ80973.1 hypothet	XP_001653393.1protoheme IX farnesyltransferase, mitochondrial
AAEL00164	WD repeat-containing protein 81	0.0337388306010313	0.816819081227326	0.952403596737691	NA	NA	KXJ81394.1 hypothet	XP_021701168.1WD repeat-containing protein 81
AAEL00858	28S ribosomal protein S2, mitochondrial	0.0337392230366747	0.797433663451733	0.945982283238732	NA	aag03010	KXJ68945.1 hypothet	XP_001653316.228S ribosomal protein S2, mitochondrial
AAEL02074	NA	0.0337518710298418	0.846668859270518	0.963198678766584	NA	NA	NA	XP_021704551.1ribosome biogenesis ATPase RIX7-like
AAEL02779	NA	0.0337797444823058	0.866414029220594	0.968074250389273	NA	NA	NA	XP_021694545.1microtubule-associated protein futsch isoform X1
AAEL02749	NA	0.0337944659423361	0.766106515851462	0.939178856256477	NA	NA	NA	XP_021699558.1helicase domino isoform X3
AAEL00233	eukaryotic translation initiation factor 3 subunit E	0.0338251696227568	0.778665015797494	0.943047575068247	NA	aag03013	ETN66627.1 eukaryo	XP_001661371.1eukaryotic translation initiation factor 3 subunit E
AAEL02306	NA	0.0338543059459856	0.760005807735981	0.935871383184374	NA	aag03050	NA	XP_001649429.126S proteasome regulatory subunit 8
AAEL02393	NA	0.0338602534718245	0.922842020036399	0.981343310857761	NA	NA	NA	XP_021708214.1zinc finger protein OZF
AAEL02173	NA	0.0338845530096029	0.846503149352225	0.963198678766584	NA	NA	NA	XP_019559588.1 enhancer of rudimentary homolog
AAEL02189	NA	0.0338868503239067	0.771478161194443	0.941147080452193	NA	NA	NA	NA
AAEL01988	NA	0.0340447658184724	0.779989122666957	0.943566949556407	NA	aag04120	NA	XP_021697450.1probable E3 ubiquitin-protein ligase HERC4 isoform X2
AAEL00009	secretory carrier-associated membrane protein 2	0.034076957581215	0.756377042616621	0.934127170333935	NA	NA	XP_019544927.1 PRE	XP_001647933.1secretory carrier-associated membrane protein 2 isoform X3
AAEL01956	NA	0.0340937815806263	0.822251721079721	0.954452060294489	270	aag01100 ; aag	NA	XP_021703760.1S-adenosylmethionine synthase isoform X2
AAEL00446	N-acetylglucosamine-6-sulfatase	0.0340977438181322	0.765530083852604	0.939129160326845	531	aag01100 ; aag	KXJ76976.1 hypothet	XP_001649229.1N-acetylglucosamine-6-sulfatase isoform X1
AAEL00754	actin-related protein 2/3 complex subunit 1A	0.0342571890266096	0.77670984113804	0.942167671783845	NA	aag04144	KXJ74977.1 hypothet	XP_001658440.2actin-related protein 2/3 complex subunit 1A
AAEL02901	NA	0.0342856063291452	0.778599044907601	0.943047575068247	NA	NA	NA	XP_001653504.1cytochrome c oxidase subunit 6B1 isoform X2
AAEL01956	NA	0.034440908145233	0.848728009879308	0.963483240428003	04151 ; 05165 ; 04714 ;	aag04392	NA	XP_021708527.1serine/threonine-protein kinase PAK 1 isoform X4
AAEL00927	protein KTI12 homolog	0.0344878267834317	0.863129143542312	0.966713045140744	NA	NA	NA	XP_001653777.1protein KTI12 homolog
AAEL01091	kelch-like protein diablo	0.0345184478193415	0.751847385388689	0.932629041660887	NA	NA	B0WWP2.2 RecName	XP_001661146.1kelch-like protein diablo
AAEL00150	sodium/hydrogen exchanger 3	0.0345217136385835	0.769585020058815	0.941147080452193	NA	NA	NA	XP_021700615.1sodium/hydrogen exchanger 3 isoform X7
AAEL00642	programmed cell death protein 10	0.0345609573447432	0.748761243306271	0.932554085844433	NA	NA	ETN58348.1 program	XP_001657782.1programmed cell death protein 10
AAEL01020	xylulose kinase	0.0347464382487707	0.783320489510597	0.944088427567323	00561 ; 00040	aag01100 ; aag	NA	XP_001654349.2xylulose kinase
AAEL01376	NA	0.0348467216538325	0.936803397071194	0.984408457216984	NA	NA	NA	XP_001663970.2intraflagellar transport protein 122 homolog
AAEL00925	TLD domain-containing protein 1	0.0348683001358169	0.805522588803501	0.947273975996143	NA	NA	NA	XP_001659873.1TLD domain-containing protein 1
AAEL00565	lysophospholipid acyltransferase 7	0.0349190972293534	0.751150932871	0.932554085844433	NA	aag00564	XP_001847441.1 leu	XP_021702566.1lysophospholipid acyltransferase 7
AAEL00926	uncharacterized LOC5571740	0.0349244071786273	0.876762522072127	0.969652664055335	NA	NA	NA	XP_021700739.1uncharacterized protein LOC5571740 isoform X2
AAEL00968	protein Red	0.0350282407741131	0.878548303135666	0.969652664055335	NA	NA	NA	XP_001653938.2protein Red
AAEL01982	NA	0.0350370593043375	0.926522145320467	0.98246793976139	NA	NA	NA	XP_011296389.1 beta-1-syntrophin isoform X2
AAEL01944	NA	0.0351472147804924	0.771411474485125	0.941147080452193	NA	NA	NA	XP_019548390.1 homeobox protein extradenticle isoform X2
AAEL00692	calmodulin	0.0352044161065695	0.868972334176875	0.968652831735134	NA	NA	KXJ70754.1 hypothet	XP_001652329.1calmodulin isoform X2
AAEL00948	uncharacterized LOC5571997	0.035232094298698	0.792003030951616	0.945793949302124	NA	NA	NA	XP_001660102.2uncharacterized protein LOC5571997
AAEL01972	NA	0.0352560466890895	0.801076891046757	0.946215055875588	NA	aag03015	NA	XP_021705442.1uncharacterized protein LOC5577684 isoform X4
AAEL01034	aryl hydrocarbon receptor nuclear translocator homolog	0.0352736607078478	0.847151601752625	0.963198678766584	NA	NA	NA	XP_021702898.1aryl hydrocarbon receptor nuclear translocator homolog isoform X1
AAEL01397	DCN1-like protein 3	0.0353670971674692	0.846559464835539	0.963198678766584	NA	NA	NA	XP_001664211.1DCN1-like protein 3
AAEL00747	probable E3 ubiquitin-protein ligase makorin-1	0.0353888914781796	0.774114419723159	0.941230906670349	NA	NA	XP_001870762.1 ma	XP_021699577.1probable E3 ubiquitin-protein ligase makorin-1
AAEL00515	probable 39S ribosomal protein L23, mitochondrial	0.0353955968181553	0.7822381251076	0.943962424451135	NA	aag03010	KXJ78356.1 hypothet	XP_001650427.2probable 39S ribosomal protein L23, mitochondrial
AAEL01319	uncharacterized LOC5577452	0.0354020134638481	0.92828990469618	0.982756983779826	NA	NA	NA	XP_001663378.1uncharacterized protein LOC5577452
AAEL00265	uncharacterized LOC5575557	0.0354095618058206	0.801675516762397	0.946215055875588	NA	NA	AAV90700.1 putative	ABF18042.1putative 23 kDa secreted salivary protein
AAEL00046	mediator of RNA polymerase II transcription subunit 28	0.03547246160895	0.751371231810459	0.932629041660887	NA	NA	ETN58670.1 mediato	XP_021704715.1mediator of RNA polymerase II transcription subunit 28
AAEL02137	NA	0.0355326083935412	0.792752089657273	0.945793949302124	NA	NA	NA	XP_019533670.1 probable chitinase 3
AAEL01042	ADP-ribosylation factor-related protein 1	0.0355649873371534	0.877632900637349	0.969652664055335	NA	NA	NA	XP_001654530.1ADP-ribosylation factor-related protein 1
AAEL02543	NA	0.0355919807458695	0.806697362879172	0.948040966581563	NA	NA	NA	XP_021699911.1josephin-like protein
AAEL00344	L-threonine 3-dehydrogenase, mitochondrial	0.0356710569548662	0.825828025869172	0.955602219852662	NA	aag01100 ; aag	XP_001849632.1 thr	XP_001656812.2L-threonine 3-dehydrogenase, mitochondrial
AAEL01974	NA	0.0356911909168623	0.802516530031616	0.94649029363412	NA	NA	NA	XP_021702630.1arginine/serine-rich protein PNISR
AAEL01226	CD109 antigen	0.0357488520958238	0.796062829050073	0.945890571556554	NA	NA	KFB49540.1 AGAP00	XP_001655971.2CD109 antigen
AAEL02690	NA	0.0358056153803545	0.795637807481796	0.945890571556554	NA	NA	NA	NA
AAEL01736	eukaryotic peptide chain release factor GTP-binding subunit ERF3A	0.0358725341341413	0.764263733063522	0.93832620035364	NA	aag03015	NA	XP_011493540.1eukaryotic peptide chain release factor GTP-binding subunit ERF3A isoform X1
AAEL01428	COP9 signalosome complex subunit 6	0.0358863611490225	0.844943373540477	0.963198678766584	NA	NA	NA	XP_001648346.1COP9 signalosome complex subunit 6
AAEL00622	pentatricopeptide repeat-containing protein 1, mitochondrial	0.0360015248839132	0.884119586680797	0.970521528629931	NA	NA	KXJ81811.1 hypothet	XP_001657591.2pentatricopeptide repeat-containing protein 1, mitochondrial
AAEL00572	60S ribosomal protein L7a	0.0360935149770068	0.802321501762205	0.94649029363412	NA	aag03010	KXJ79056.1 hypothet	XP_021702823.160S ribosomal protein L7a
AAEL01981	NA	0.0361277510209343	0.804466900724618	0.946758462778864	NA	NA	NA	XP_021706309.1centrosomin isoform X2
AAEL01966	NA	0.036143917671571	0.84390257640835	0.963198678766584	NA	NA	NA	XP_021696832.1ribosomal protein S6 kinase alpha-5
AAEL01198	scaffold protein salvador	0.0362389926147167	0.855531931412452	0.964980728557884	NA	aag04391 ; aag	XP_001847886.1 sca	XP_021693741.1scaffold protein salvador
AAEL01490	probable ATP-dependent RNA helicase Dbp45A	0.0362957452085687	0.801236879723869	0.946215055875588	NA	NA	NA	XP_001649978.2probable ATP-dependent RNA helicase Dbp45A
AAEL00349	collagen type IV alpha-3-binding protein	0.0363315249636211	0.78858541441051	0.945232821987723	NA	NA	XP_001841758.1 goo	XP_021699680.1 collagen type IV alpha-3-binding protein
AAEL01125	guanine nucleotide-binding protein-like 3 homolog	0.0363889590627769	0.782258502934169	0.943962424451135	NA	aag03008	KXJ69866.1 hypothet	XP_001661529.1guanine nucleotide-binding protein-like 3 homolog
AAEL00265	protein cup	0.0364166685523222	0.774505588492489	0.941230906670349	NA	NA	KXJ70699.1 hypothet	XP_001661956.1protein cup isoform X2
AAEL00704	zinc finger and SCAN domain-containing protein 2	0.0364696602353799	0.878236391884227	0.969652664055335	NA	NA	XP_019558021.1 PRE	XP_021705673.1zinc finger and SCAN domain-containing protein 2
AAEL00218	F-actin-capping protein subunit beta	0.0364751962264185	0.799094056312574	0.946215055875588	NA	aag04144	KXJ80083.1 hypothet	XP_001654929.2F-actin-capping protein subunit beta
AAEL00643	mevalonate kinase	0.0365816317511286	0.866658951826433	0.968074250389273	900	aag01100 ; aag	KFB52451.1 hypothe	XP_001657825.1mevalonate kinase
AAEL02128	NA	0.0367150885897517	0.848618610097496	0.963483240428003	NA	NA	NA	XP_021710957.1probable basic-leucine zipper transcription factor P
AAEL01210	uncharacterized LOC5575827	0.0367608873345726	0.872213514308496	0.969029518523332	NA	NA	KXJ78004.1 hypothet	AAS93542.1NOS protein
AAEL00715	cytospin-A	0.0368041678197997	0.782012581424251	0.943962424451135	NA	NA	XP_019532898.1 PRE	XP_001652575.2cytospin-A isoform X8
AAEL02356	NA	0.0368279887317048	0.882833511182529	0.970357617045106	NA	NA	NA	XP_021709187.1mucin-5AC isoform X4
AAEL00133	RNA polymerase II-associated factor 1 homolog	0.0368668541990769	0.756832999125661	0.934188766995886	NA	NA	KXJ69833.1 hypothet	XP_021705981.1RNA polymerase II-associated factor 1 homolog

AAEL01501	protein artichoke	0.03687587692 7609	0.852146577680438	0.964626990751063	NA	NA	NA	XP_021698717.1protein artichoke
AAEL01362	uncharacterized LOC5578310	0.0368811416048337	0.827228171106333	0.956432333168643	NA	NA	NA	XP_001656894.2uncharacterized protein LOC5578310
AAEL01493	transcription factor BTF3 homolog 4	0.0369191140832602	0.764773292698699	0.938701325785438	NA	aag04214	NA	XP_001662369.1transcription factor BTF3 homolog 4
AAEL01944	NA	0.0369388653595439	0.807744798867117	0.948190794639012	NA	aag03040	NA	XP_021693013.1intron-binding protein aquarius
AAEL00826	F-box/LRR-repeat protein 14	0.0369850821015885	0.839613306543875	0.962916332585959	NA	NA	KFB45523.1 Dsec\GN	XP_021700181.1F-box/LRR-repeat protein 14
AAEL00617	general odorant-binding protein 83a	0.0369905435498304	0.833376000638219	0.959902797120062	NA	NA	KXJ68133.1 hypothet	XP_001651827.2general odorant-binding protein 83a
AAEL01267	D-aspartate oxidase	0.0370843799409442	0.87922549883216	0.969652664055335	NA	aag01100 ; aag	XP_001851437.1 d-a	XP_001662804.1D-aspartate oxidase
AAEL00255	DNA topoisomerase I, mitochondrial	0.0371649451234842	0.753250844594744	0.933081420650645	NA	NA	KXJ80888.1 hypothet	XP_021713406.1DNA topoisomerase I, mitochondrial
AAEL01163	UPF0598 protein CG30010	0.0372959176503799	0.871656200727105	0.969029518523332	NA	NA	XP_001845840.1 con	XP_001661806.2UPF0598 protein CG30010
AAEL01947	NA	0.037486509413638	0.817220955133013	0.952514760254484	NA	aag00534	NA	XP_001656973.2heparan-sulfate 6-O-sulfotransferase 2
AAEL00847	27 kDa hemolymph protein	0.0375289117351519	0.774257326051801	0.941230906670349	NA	NA	KXJ83464.1 hypothet	XP_001659256.227 kDa hemolymph protein
AAEL01252	YEATS domain-containing protein 4	0.0375719201062449	0.926522145321598	0.98246793976139	NA	NA	KXJ78581.1 hypothet	XP_001662633.1YEATS domain-containing protein 4
AAEL00690	PITH domain-containing protein GA19395	0.0375849436385132	0.826736167434996	0.956332647735617	NA	NA	XP_001845469.1 thic	XP_001658071.1PITH domain-containing protein GA19395
AAEL01406	thioredoxin domain-containing protein 17	0.0376489701310563	0.742816143499761	0.930112144847571	NA	NA	NA	XP_001664307.1thioredoxin domain-containing protein 17
AAEL01181	ER membrane protein complex subunit 2	0.0376933613587735	0.771887318828948	0.941147080452193	NA	NA	KXJ80243.1 hypothet	XP_001661937.2ER membrane protein complex subunit 2
AAEL00978	chitinase-like protein 3	0.0376937551658602	0.801052252200503	0.946215055875588	520	aag01100 ; aag	NA	XP_001654045.2chitinase-like protein 3
AAEL00755	protein valois	0.0377037234959295	0.853509193200095	0.964653958545747	NA	NA	KXJ78546.1 hypothet	XP_001658432.1protein valois
AAEL01985	NA	0.0377142528967253	0.737321379465262	0.927599209713406	NA	NA	NA	XP_019550247.1 putative mediator of RNA polymerase II transcription subunit 24 isoform X3
AAEL01079	WD repeat domain phosphoinositide-interacting protein 3	0.0377292737308959	0.770364541762654	0.941147080452193	NA	NA	ETN67399.1 Autopha	XP_001654902.2WD repeat domain phosphoinositide-interacting protein 3
AAEL02216	NA	0.0377976629379731	0.721248916697581	0.921583569204186	NA	NA	NA	XP_021701614.1 vacuolar protein sorting-associated protein 13D
AAEL00585	exosome complex exonuclease RRP42	0.0378049162812706	0.865845830266082	0.967927335900485	NA	aag03018	ETN62123.1 exosome	XP_001651510.2exosome complex exonuclease RRP42
AAEL01162	serine protease snake	0.0378214572306879	0.805211842267663	0.947029572155807	NA	NA	XP_019551449.1 PRE	XP_001661794.2serine protease snake isoform X2
AAEL00239	uncharacterized LOC5574475	0.0378592299356494	0.791685738232871	0.945793949302124	NA	NA	KXJ81911.1 hypothet	XP_021699621.1uncharacterized protein LOC5574475
AAEL01441	probable cytochrome P450 304a1	0.0379070681820865	0.745463461142271	0.931365937538534	NA	NA	NA	XP_001648725.1probable cytochrome P450 304a1
AAEL01198	tRNA selenocysteine 1-associated protein 1	0.0380061824630712	0.815142820490977	0.952015623780574	NA	NA	KXJ75050.1 hypothet	XP_001655804.1tRNA selenocysteine 1-associated protein 1
AAEL00476	metallo-beta-lactamase domain-containing protein 1	0.0380783485746467	0.791919338276131	0.945793949302124	NA	NA	XP_011554239.1 PRE	XP_021701015.1metallo-beta-lactamase domain-containing protein 1
AAEL01336	trafficking protein particle complex subunit 1	0.0381009525694963	0.852811417604873	0.964653958545747	NA	NA	NA	XP_001663510.1trafficking protein particle complex subunit 1
AAEL01294	uncharacterized LOC5577021	0.0381483388826369	0.857677982882026	0.964980728557884	NA	NA	NA	XP_001656307.2uncharacterized protein LOC5577021 isoform X1
AAEL01943	NA	0.0381721635704131	0.811467119872938	0.950136205251403	NA	aag04214	NA	XP_021710216.1ecdysone receptor isoform X1
AAEL00427	17-beta-hydroxysteroid dehydrogenase 13	0.0382144345134118	0.870722500002709	0.968967038943636	NA	NA	XP_001866722.1 sho	XP_001648844.117-beta-hydroxysteroid dehydrogenase 13
AAEL00862	ATP-binding cassette sub-family G member 4	0.0382425660168291	0.822821397077704	0.954452060294489	NA	NA	XP_001863367.1 ATF	XP_001653359.2ATP-binding cassette sub-family G member 4
AAEL01959	NA	0.0382769661493009	0.855276001795953	0.964980728557884	NA	NA	NA	XP_021700787.1insulin receptor substrate 1 isoform X6
AAEL02109	NA	0.0382869481224716	0.923398564247881	0.981343310857761	NA	NA	NA	XP_021712617.1uncharacterized protein LOC110681157
AAEL00686	CCR4-NOT transcription complex subunit 9	0.0382893757139115	0.832689526449852	0.959600378508646	NA	aag03018	KFB50224.1 hypothe	XP_001652289.1CCR4-NOT transcription complex subunit 9
AAEL00449	stomatin-like protein 2, mitochondrial	0.0383721708416888	0.79439537719505	0.945793949302124	NA	NA	KXJ72295.1 hypothet	XP_001649283.2stomatin-like protein 2, mitochondrial
AAEL01975	NA	0.0383973583712265	0.720757491585192	0.921583569204186	NA	NA	NA	XP_021703399.1uncharacterized protein LOC5573942 isoform X5
AAEL02657	NA	0.0384109362168287	0.899548546458598	0.97393667311859	NA	NA	NA	XP_021693908.1transcription factor grauzone
AAEL00466	septin-2	0.0384647302313442	0.73300928732768	0.925884845335185	NA	NA	KXJ77224.1 hypothet	XP_019550231.1 septin-2-like
AAEL00065	exosome complex component RRP43	0.0385997505135511	0.860728555186455	0.966023193660905	NA	aag03018	KXJ76762.1 hypothet	XP_001649768.1exosome complex component RRP43
AAEL02480	NA	0.038627537736552	0.776516811612675	0.942167671783845	NA	NA	NA	XP_021697398.1vesicle-associated membrane protein/synaptobrevin-binding protein-like
AAEL00890	protein ecdysoneless	0.0386954519429018	0.872574640856518	0.969029518523332	NA	NA	KXJ72630.1 hypothet	XP_001659598.2protein ecdysoneless
AAEL00501	basic salivary proline-rich protein 4	0.0387033740622703	0.745447312428552	0.931365937538534	NA	NA	KXJ69375.1 hypothet	XP_001650215.1basic salivary proline-rich protein 4
AAEL01007	nardilysin	0.0387109727556365	0.73408083874445	0.926486336322374	NA	NA	NA	XP_001660612.2nardilysin isoform X1
AAEL00394	28S ribosomal protein S33, mitochondrial	0.0387484897327619	0.807724719547618	0.948190794639012	NA	NA	KFB44642.1 AGAP00	XP_001648069.128S ribosomal protein S33, mitochondrial
AAEL00430	proteasome subunit alpha type-5	0.0388219618861152	0.738499386437912	0.928033958695205	NA	aag03050	ETN61753.1 proteaso	XP_001648894.1proteasome subunit alpha type-5
AAEL00094	macoilin-2	0.038834328101451	0.842767350146038	0.963198678766584	NA	NA	ETN62240.1 hypothe	XP_019531448.1 macoilin-like
AAEL01716	probable G-protein coupled receptor Mth-like 1	0.038870301223305	0.785305230106842	0.944372632355572	NA	NA	NA	XP_021693015.1probable G-protein coupled receptor Mth-like 1
AAEL00765	probable phosphorylase b kinase regulatory subunit alpha	0.0388763268421727	0.742177187353116	0.929911685600432	NA	NA	XP_019545093.1 PRE	XP_021694658.1probable phosphorylase b kinase regulatory subunit alpha isoform X3
AAEL00234	E3 ubiquitin-protein ligase listerin	0.0391086748904839	0.761166731217533	0.936472047952049	NA	NA	KXJ76375.1 hypothet	XP_021698906.1E3 ubiquitin-protein ligase listerin
AAEL00976	xaa-Pro aminopeptidase ApepP	0.0392625246370067	0.814662865242801	0.952015623780574	NA	NA	NA	XP_001660338.2xaao aminopeptidase ApepP isoform X1
AAEL00498	probable phospholipid-transporting ATPase IIA	0.0393680651394333	0.761524019728656	0.936712208199851	NA	NA	XP_019537956.1 PRE	XP_021700874.1probable phospholipid-transporting ATPase IIA isoform X2
AAEL00870	U3 small nucleolar RNA-associated protein 14 homolog A	0.0394142773570045	0.772089912278502	0.941147080452193	NA	aag03008	KXJ77858.1 hypothet	XP_001659444.2U3 small nucleolar RNA-associated protein 14 homolog A
AAEL00797	zinc finger protein 34	0.0394689333584601	0.894425984109632	0.972739913152176	NA	NA	KXJ78291.1 hypothet	XP_001658769.2zinc finger protein 34
AAEL00351	cyclic AMP-dependent transcription factor ATF-6 alpha	0.0395456253319036	0.780203853072432	0.94365072438627	NA	aag04141	KXJ84412.1 hypothet	XP_001656926.2cyclic AMP-dependent transcription factor ATF-6 alpha
AAEL01399	ubiquitin thioesterase OTU1	0.0395468832200409	0.888873127577227	0.971195739725201	NA	aag04141	NA	XP_001657253.1ubiquitin thioesterase OTU1
AAEL00377	transmembrane protein 129	0.0397110978543175	0.869876036078686	0.968652831735134	NA	NA	KXJ75741.1 hypothet	XP_001664176.2transmembrane protein 129
AAEL01076	alpha-ketoglutarate-dependent dioxygenase alkB homolog 4	0.0397507463345124	0.83402353975463	0.959935536312959	NA	NA	XP_019538277.1 PRE	XP_001654880.1alpha-ketoglutarate-dependent dioxygenase alkB homolog 4
AAEL01464	ubiquinone biosynthesis protein COQ4 homolog, mitochondrial	0.0397656433905228	0.884559744646938	0.970521528629931	NA	NA	NA	XP_001649247.2ubiquinone biosynthesis protein COQ4 homolog, mitochondrial
AAEL01300	protein seele	0.0398185684402334	0.75876811534914	0.935439571457873	NA	NA	NA	XP_001663197.2protein seele
AAEL02370	NA	0.0398329856678117	0.861849736878793	0.966220916028101	NA	NA	NA	XP_021710087.1ral guanine nucleotide dissociation stimulator-like 1 isoform X3
AAEL00388	polyubiquitin-C	0.040038879242501	0.746128604577477	0.931365937538534	NA	NA	KXJ73533.1 hypothet	XP_001664265.1polyubiquitin isoform X2
AAEL01830	NA	0.0401390069076284	0.893661195722341	0.972739913152176	NA	NA	NA	XP_021710860.1sodium-dependent nutrient amino acid transporter 1 isoform X2
AAEL00957	elongation of very long chain fatty acids protein AAEL008004	0.0403231837909404	0.754215708117368	0.933839201573738	62	NA	NA	XP_001660239.2elongation of very long chain fatty acids protein AAEL008004 isoform X2
AAEL00656	mucin-5AC	0.0403549885514609	0.715577386351336	0.920421919040713	NA	NA	NA	XP_021704320.1rho GTPase-activating protein gacF isoform X4
AAEL00373	homeodomain-interacting protein kinase 2	0.0403560081942239	0.755424633190721	0.934127170333935	NA	NA	XP_019560057.1 PRE	XP_021699297.1homeodomain-interacting protein kinase 2 isoform X1
AAEL00433	pyruvate dehydrogenase E1 component subunit beta, mitochondrial	0.040383242150376	0.737461256128127	0.927599209713406	00010 ; 00620 ; 00020	aag01100 ; aag	KFB41658.1 AGAP01	XP_001648922.2pyruvate dehydrogenase E1 component subunit beta, mitochondrial
AAEL02551	NA	0.0404196589232722	0.727202230736267	0.923566968708516	NA	NA	NA	XP_021708243.1putative mediator of RNA polymerase II transcription subunit 26
AAEL01041	translin-associated protein X	0.0404588879836631	0.901633457585932	0.974426271645104	NA	NA	NA	XP_001654528.1translin-associated protein X
AAEL01081	F-BAR domain only protein 2	0.0405591414804203	0.776637243647311	0.942167671783845	NA	NA	ETN64419.1 hypothe	XP_021707819.1F-BAR domain only protein 2 isoform X1
AAEL00815	WAS/WASL-interacting protein family member 2	0.0406190210717762	0.76497734873888	0.9388265621626	NA	aag04144	XP_001849878.1 con	XP_021708077.1WAS/WASL-interacting protein family member 1 isoform X2
AAEL01833	NA	0.0406311643303494	0.821643257462923	0.954280102887015	NA	NA	NA	XP_001650319.2protein cereblon homolog
AAEL01216	aldehyde dehydrogenase, dimeric NADP-preferring	0.0407868098705081	0.739178100262412	0.928209181033667	NA	NA	NA	XP_021699429.1aldehyde dehydrogenase, dimeric NADP-preferring isoform X5
AAEL00280	DNA polymerase epsilon catalytic subunit A	0.0407911084745114	0.855957739983354	0.964980728557884	NA	aag03420 ; aag	KXJ76714.1 hypothet	XP_001662453.2DNA polymerase epsilon catalytic subunit A
AAEL00095	division abnormally delayed protein	0.0408152079346224	0.711892217222786	0.919157034219739	NA	aag04310 ; aag	KXJ83819.1 hypothet	XP_021695577.1division abnormally delayed protein
AAEL00545	60S ribosomal protein L38	0.0409815180820983	0.781011796687311	0.943902095234652	NA	aag03010	ACF72870.1 ribosom	XP_001650915.160S ribosomal protein L38
AAEL00776	protein kintoun	0.0409815871738484	0.712383575933322	0.919252089784076	NA	NA	KXJ84018.1 hypothet	XP_001658637.2protein kintoun
AAEL00288	cytochrome c oxidase subunit NDUFA4	0.0410540468369727	0.774256490993036	0.941230906670349	NA	aag01100 ; aag	ETN67053.1 NADH:u	XP_001656091.1cytochrome c oxidase subunit NDUFA4
AAEL00278	26S proteasome non-ATPase regulatory subunit 8	0.0410841193694187	0.726001096492649	0.923177868750456	NA	aag03050	KFB53622.1 AGAP00	XP_001662445.126S proteasome non-ATPase regulatory subunit 8
AAEL00100	calreticulin	0.0411314164190772	0.735460269015857	0.926702094411052	NA	aag04141 ; aag	XP_019545053.1 PRE	AAL76026.1putative calreticulin
AAEL01116	uncharacterized LOC5574435	0.0411395407399439	0.708814709559014	0.918089462824826	NA	NA	NA	XP_001661442.2uncharacterized protein LOC5574435
AAEL02362	NA	0.04115848239016	0.762069929075214	0.937006693824152	NA	aag04144	NA	XP_021694798.1rab GTPase-binding effector protein 1 isoform X2
AAEL02461	NA	0.0412047476105524	0.821083337308711	0.954106679666959	NA	NA	NA	XP_021694742.1serine/arginine repetitive matrix protein 2

AAEL02357	NA	0.0412208949790295	0.880129115221946	0.969652664055335	NA		NA	NA	XP_001655696.1WW domain-binding protein 2
AAEL01095	uncharacterized LOC5574129	0.0412839102245292	0.749918929772507	0.932554085844433	NA		NA	NA	XP_021709848.1uncharacterized protein LOC5574129 isoform X1
AAEL00178	deoxyribodipyrimidine photo-lyase	0.0412884374320697	0.858492941766247	0.96528006477676	NA		NA	KXJ71848.1 hypothet	XP_021698749.1deoxyribodipyrimidine photo-lyase isoform X1
AAEL00138	IST1 homolog	0.0413013688741036	0.756760263384909	0.934188766995886	NA		aag04144	XP_019554320.1 PRE	XP_001653151.2IST1 homolog isoform X1
AAEL01814	NA	0.0413341678435953	0.782527528239909	0.943962424451135	NA		NA	NA	XP_001658589.2tribbles homolog 2
AAEL00382	dynamin-like 120 kDa protein, mitochondrial	0.041364694858973	0.713464293114309	0.919252089784076	NA		NA	XP_019527070.1 PRE	XP_001657306.2dynamin-like 120 kDa protein, mitochondrial isoform X1
AAEL00432	valine--tRNA ligase	0.0414539674455871	0.695715356311611	0.916122517126697		970	aag00970	KXJ73231.1 hypothet	XP_001648925.2valine--tRNA ligase
AAEL01477	TBC1 domain family member 23	0.0414881145022166	0.756245712226207	0.934127170333935	NA		NA	NA	XP_001649589.1TBC1 domain family member 23
AAEL00073	uncharacterized LOC5566275	0.0415222740384577	0.914774471460968	0.977901601050505	NA		NA	AAV90682.1 putative	AAL76021.1putative secreted protein
AAEL01239	glucosidase 2 subunit beta	0.0416706572988558	0.720638376306236	0.921583569204186	NA		aag04141	KFB46281.1 AGAP00	XP_001662536.1glucosidase 2 subunit beta
AAEL00353	60S acidic ribosomal protein P1	0.0416900619280867	0.801372273783211	0.946215055875588	NA		aag03010	XP_001857537.1 acic	XP_001656939.160S acidic ribosomal protein P1
AAEL01320	uncharacterized LOC5577458	0.0417345578568123	0.796083561317594	0.945890571556554	NA		NA	NA	XP_001663389.1uncharacterized protein LOC5577458
AAEL01062	uncharacterized protein C6orf203	0.0417868912958608	0.774494071030662	0.941230906670349	NA		NA	NA	XP_001660939.2uncharacterized protein C6orf203
AAEL01318	UPF0568 protein C14orf166	0.0417971711274206	0.827481768818389	0.956473713938803	NA		NA	NA	XP_001663377.1UPF0568 protein C14orf166
AAEL00563	protein lin-9 homolog	0.0419853793285652	0.924128757866593	0.981740109661555	NA		NA	KXJ72837.1 hypothet	XP_021701011.1protein lin-9 homolog
AAEL01407	vacuolar protein sorting-associated protein 52 homolog	0.0420007463237174	0.816643307197089	0.952319314767154	NA		NA	NA	XP_001662260.1vacuolar protein sorting-associated protein 52 homolog
AAEL00854	cdc42 homolog	0.0422152429072971	0.692778193771714	0.916122517126697	NA		aag04144 ; aag	ACL83849.1 Cdc42, p	XP_001659371.1cdc42 homolog
AAEL01950	NA	0.0424862686990198	0.736661676492476	0.927453926266762	NA		NA	NA	XP_019563348.1 protein phosphatase 1 regulatory subunit 16A isoform X1
AAEL01530	NA	0.0425225568492703	0.690976354542838	0.916122517126697	NA		NA	NA	XP_001651535.2ester hydrolase C11orf54 homolog isoform X1
AAEL02699	NA	0.0427027515166762	0.762581389834924	0.937135944078655	NA		aag04146	NA	XP_021704572.1peroxisome biogenesis factor 1
AAEL00133	mannose-1-phosphate guanyltransferase beta	0.042792296188347	0.705702294945187	0.917749125552377	NA		aag01100 ; aag	KFB42785.1 hypothe	XP_001653051.1mannose-1-phosphate guanyltransferase beta
AAEL00405	cystathionine gamma-lyase	0.0427954156799873	0.784850613919018	0.944372632355572	NA		aag01100 ; aag	XP_019558869.1 PRE	XP_001648394.2cystathionine gamma-lyase
AAEL00263	protein ABHD13	0.042867943438605	0.815317213620343	0.952015623780574	NA		NA	KFB39225.1 AGAP00	XP_001661960.1protein ABHD13
AAEL01110	histone acetyltransferase KAT6B	0.0428719684244014	0.703611899306341	0.917303584765266	NA		NA	NA	XP_021700092.1histone acetyltransferase KAT6A isoform X2
AAEL01079	uncharacterized LOC5573869	0.0429010506248808	0.884526833929981	0.970521528629931	NA		NA	KXJ84014.1 hypothet	XP_001654903.2uncharacterized protein LOC5573869
AAEL00404	UNC93-like protein	0.0430538112375167	0.720162761103204	0.921583569204186	NA		NA	KFB40120.1 AGAP00	XP_001648381.1UNC93-like protein
AAEL01716	uncharacterized LOC23687587	0.0430833075084751	0.871328005886167	0.969029518523332	NA		NA	NA	XP_021694447.1uncharacterized protein LOC23687587
AAEL01086	tetratricopeptide repeat protein 17	0.0430880124502649	0.798465537857234	0.946215055875588	NA		NA	ETN64622.1 TRP-pho	XP_001661100.1tetratricopeptide repeat protein 17
AAEL02466	NA	0.0431084993533819	0.730417659929873	0.924653089376075	NA		NA	NA	XP_021711560.1serine protease easter isoform X2
AAEL00925	uncharacterized LOC5571739	0.0432391478176572	0.887662398732812	0.970996238637524	NA		NA	NA	XP_001659883.2uncharacterized protein LOC5571739 isoform X2
AAEL00490	ras-related protein Rab-2A	0.0432617279681629	0.698661298272207	0.916606967023218	NA		NA	XP_004526582.1 ras-	XP_001650005.1ras-related protein Rab-2A
AAEL01127	putative 1-phosphatidylinositol 3-phosphate 5-kinase	0.0433760014037331	0.695645308275618	0.916122517126697	NA		aag01100 ; aag	ETN66830.1 1-phosp	XP_021699897.1putative 1-phosphatidylinositol 3-phosphate 5-kinase
AAEL02375	NA	0.0433946868984659	0.827042344699693	0.956432333168643	NA		NA	NA	XP_021701656.1facilitated trehalose transporter Tret1-2 homolog
AAEL00571	protein suppressor of forked	0.0434009693939569	0.81914045969776	0.952969210637171	NA		aag03015	XP_001845372.1 sup	XP_001651356.2protein suppressor of forked
AAEL00031	uncharacterized LOC5575171	0.0434842789126959	0.736844429396413	0.92755710524019	NA		NA	XP_317453.2 AGAP0	XP_021702279.1uncharacterized protein LOC5575171
AAEL00994	NA	0.0436702308689817	0.700950839362711	0.916735307534916	NA		aag04141	NA	XP_001660492.2dnaJ homolog shv
AAEL00974	uncharacterized LOC5572359	0.0436739033882948	0.898554287495346	0.973780616635202	NA		NA	NA	XP_001660317.1uncharacterized protein LOC5572359
AAEL00730	MKL/myocardin-like protein 2	0.0436756137443829	0.825897028307783	0.955602219852662	NA		NA	XP_019546219.1 PRE	XP_021710983.1MKL/myocardin-like protein 2 isoform X2
AAEL02369	NA	0.0437018475016843	0.808912557222522	0.948933370455872	NA		NA	NA	XP_019537085.1 zinc finger protein 423
AAEL02641	NA	0.0438880467547285	0.844549318908041	0.963198678766584	NA		NA	NA	XP_021696024.1phospholipid scramblase 3
AAEL02109	NA	0.0439890089602745	0.862434581800746	0.966468488016169	NA		NA	NA	XP_021695576.1transmembrane protein 43 homolog
AAEL00407	uncharacterized LOC5564052	0.0440166781335234	0.888627233477013	0.971157696253619	NA		NA	XP_001844731.1 con	XP_001648386.2uncharacterized protein LOC5564052
AAEL01293	coiled-coil-helix-coiled-coil-helix domain-containing protein 10, mitochon	0.044230359528511	0.698047136570787	0.916454522859806	NA		NA	NA	XP_001656310.2coiled-coil-helix-coiled-coil-helix domain-containing protein 10, mitochondrial
AAEL00034	uncharacterized LOC5575134	0.0442833143472694	0.729420892283504	0.923905484505959	NA		NA	NA	XP_021697902.1uncharacterized protein LOC5575134 isoform X1
AAEL00718	V-type proton ATPase subunit G	0.0443467749984178	0.696443686164625	0.916122517126697	NA		aag01100 ; aag	ETN65749.1 vacuolar	XP_001652605.1V-type proton ATPase subunit G
AAEL00016	uncharacterized LOC5570039	0.0444735604054756	0.709655144650191	0.918459443188615	NA		NA	XP_019528532.1 PRE	XP_019533825.1 toxin-like protein 14 isoform X4
AAEL00846	surfeit locus protein 4 homolog	0.0444834751147829	0.711629462526063	0.918946718238118	NA		NA	ETN62512.1 surfeit lo	XP_001659248.1surfeit locus protein 4 homolog
AAEL00029	probable 28S ribosomal protein S25, mitochondrial	0.044491329368569	0.752642158529141	0.93283104590187	NA		NA	ETN65708.1 mitocho	XP_001654783.1probable 28S ribosomal protein S25, mitochondrial
AAEL00584	integrator complex subunit 8	0.0445125195466408	0.867824258129659	0.96863134437578	NA		NA	ETN66056.1 hypothe	XP_001651505.2integrator complex subunit 8
AAEL00347	uncharacterized LOC5578189	0.0445709040604781	0.895457202464431	0.973074772918953	NA		NA	KFB49660.1 hypothe	XP_001656831.2uncharacterized protein LOC5578189
AAEL01231	proliferation-associated protein 2G4	0.044611559846556	0.677697528155428	0.914481546552978	NA		NA	KXJ74376.1 hypothet	XP_001662411.1proliferation-associated protein 2G4
AAEL01482	uncharacterized LOC5565340	0.0446570342090366	0.705401915825714	0.917749125552377	NA		NA	NA	XP_021704816.1uncharacterized protein LOC5565340
AAEL01962	NA	0.0446825203575774	0.815521183461719	0.952015623780574	NA		NA	NA	XP_021705232.1flocculation protein FLO11-like isoform X2
AAEL00409	1,5-anhydro-D-fructose reductase	0.0446915090565214	0.762451245354944	0.937135944078655	NA		NA	KXJ83377.1 hypothet	XP_001648460.21,5-anhydro-D-fructose reductase
AAEL00474	multidrug resistance-associated protein 1	0.0447314269488524	0.720031583670174	0.921583569204186	NA		aag02010	NA	XP_021701003.1multidrug resistance-associated protein 1 isoform X8
AAEL01546	serine protease easter	0.0447785790266475	0.718564296728377	0.921583569204186	NA		NA	NA	XP_021711070.1serine protease easter
AAEL01368	uncharacterized LOC5578392	0.0449137114164347	0.679259576081238	0.914481546552978	NA		NA	NA	XP_021694039.1uncharacterized protein LOC5578392 isoform X3
AAEL01828	NA	0.0450489405299769	0.738925337794825	0.928033958695205	NA		NA	NA	XP_001660803.2RING finger protein 121
AAEL00342	40S ribosomal protein S16	0.0450568538407387	0.722634711121303	0.921934742632737	NA		aag03010	AAV90714.1 ribosom	XP_001656820.140S ribosomal protein S16
AAEL02358	NA	0.0450851110938908	0.794812701900482	0.945793949302124	NA		aag04391 ; aag	NA	XP_021694036.1cellular tumor antigen p53 isoform X3
AAEL01822	NA	0.0453007104808343	0.713413502432855	0.919252089784076	NA		NA	NA	XP_021708008.1protein strawberry notch isoform X1
AAEL00643	dnaJ homolog subfamily C member 21	0.0453563481474141	0.780841213181441	0.94381989277494	NA		NA	KXJ69168.1 hypothet	XP_001657822.1dnaJ homolog subfamily C member 21
AAEL00571	uncharacterized LOC5566952	0.0453925441395842	0.857766319015271	0.964980728557884	NA		NA	KFB46098.1 hypothe	XP_019532884.1 cyclin-dependent kinase 2-associated protein 2 isoform X1
AAEL00461	phenylalanine--tRNA ligase beta subunit	0.0454956624902582	0.719817737536513	0.921583569204186		970	aag00970	KXJ82448.1 hypothet	XP_001649522.2phenylalanine--tRNA ligase beta subunit
AAEL02253	NA	0.0455434602717985	0.767149929734254	0.939488109317888	NA		aag01100 ; aag	NA	XP_021694453.1inositol polyphosphate 1-phosphatase
AAEL01266	eukaryotic translation initiation factor 3 subunit G	0.0456989474576463	0.697765653502894	0.916454522859806	NA		aag03013	KFB36620.1 AGAP00	XP_001652269.1eukaryotic translation initiation factor 3 subunit G
AAEL02299	NA	0.0457083758460268	0.694450329022053	0.916122517126697	NA		NA	NA	NA
AAEL00804	39S ribosomal protein L34, mitochondrial	0.0457485299985764	0.805883633225705	0.947456393630119	NA		aag03010	XP_019537333.1 PRE	XP_001658838.139S ribosomal protein L34, mitochondrial
AAEL00383	eukaryotic translation initiation factor 5A	0.0457980675681049	0.703407816421669	0.917303584765266	NA		NA	ETN67073.1 initiation	XP_001657309.1eukaryotic translation initiation factor 5A
AAEL00525	syntaxin-8	0.0458179577351528	0.847221267761001	0.963198678766584	NA		aag04130	KFB36076.1 AGAP00	XP_019539036.1 syntaxin-8
AAEL00191	uncharacterized LOC5572798	0.0458251460404609	0.684298133139799	0.914629465876247	NA		NA	NA	XP_001654198.2uncharacterized protein LOC5572798
AAEL00910	eukaryotic translation initiation factor 3 subunit F	0.0459107531051294	0.711045252406187	0.918935795219545	NA		aag03013	KFB41773.1 AGAP00	XP_001659715.1eukaryotic translation initiation factor 3 subunit F
AAEL00241	renin receptor	0.0459591878189948	0.688086100911651	0.916010156153545	NA		NA	KXJ82843.1 hypothet	XP_001655215.2renin receptor
AAEL02186	NA	0.0459727532190954	0.913311900981831	0.97769847752848	NA		NA	NA	XP_021693140.1zinc finger protein 710-like
AAEL00648	nuclear pore complex protein Nup88	0.0459835434653703	0.752199004882683	0.932695238282356	NA		aag03013	XP_001689384.2 AGA	XP_021710032.1nuclear pore complex protein Nup88
AAEL01484	innexin inx2	0.0461667323866952	0.674403530282132	0.913701602717341	NA		NA	NA	XP_001649755.1innexin inx2
AAEL00942	ralBP1-associated Eps domain-containing protein 1	0.0462366281097686	0.793435912147009	0.945793949302124	NA		NA	NA	XP_021700411.1ralBP1-associated Eps domain-containing protein 1
AAEL00446	AP-2 complex subunit alpha	0.0462466692529228	0.685319698779293	0.914629465876247	NA		aag04144	ETN58439.1 adaptin,	XP_001649235.1AP-2 complex subunit alpha
AAEL01130	uncharacterized LOC5574662	0.0462909437072511	0.822612150214547	0.954452060294489	NA		NA	KXJ80254.1 hypothet	XP_001655241.2uncharacterized protein LOC5574662
AAEL00893	G protein-activated inward rectifier potassium channel 3	0.0463944273715812	0.766591218141969	0.939178856256477	NA		NA	XP_019529551.1 PRE	XP_021707003.1G protein-activated inward rectifier potassium channel 3 isoform X2
AAEL00859	mannose-P-dolichol utilization defect 1 protein homolog	0.0464049582532621	0.698490190060307	0.916606967023218	NA		NA	KXJ71768.1 hypothet	XP_001653333.1mannosedolichol utilization defect 1 protein homolog
AAEL01948	NA	0.0464187921426479	0.736140866840057	0.927178792316206	NA		aag04013	NA	XP_021702037.1tyrosine-protein kinase Btk29A isoform X1

AAEL0153	uncharacterized LOC5567331	0.0464302494555607	0.777306948895838	0.942439739175201	NA	NA	NA	XP_001657357.1uncharacterized protein LOC5567331
AAEL0076	transmembrane 9 superfamily member 4	0.0465524310911934	0.793141521792526	0.945793949302124	NA	NA	KXJ73252.1 hypothet	XP_001658596.1transmembrane 9 superfamily member 4
AAEL0050	translation initiation factor IF-2	0.0465994499214303	0.65784132286151	0.909516556517693	NA	NA	KFB39069.1 AGAP00	XP_019535576.1 spidroin-2-like
AAEL0052	glucose-induced degradation protein 4 homolog	0.0466070158753779	0.676695557617541	0.914438668430353	NA	NA	XP_001870747.1 con	XP_021694479.1glucose-induced degradation protein 4 homolog
AAEL0030	zinc finger protein 704	0.0466397807128753	0.71281362159875	0.919252089784076	NA	NA	XP_001867495.1 con	XP_021709150.1zinc finger protein 704 isoform X2
AAEL0042	uncharacterized protein YdcI	0.0466444096037823	0.85813708611485	0.965115798879107	NA	NA	KXJ74278.1 hypothet	XP_001846863.1S1 RNA binding domain protein
AAEL0086	ATP-binding cassette sub-family G member 1	0.0466573289754384	0.6836916431525	0.914629465876247	NA	NA	KXJ79717.1 hypothet	XP_001653355.2ATP-binding cassette sub-family G member 1
AAEL0050	serine--tRNA ligase, cytoplasmic	0.0466627619054987	0.697700987563797	0.916454522859806	970	aag00970	KFB45238.1 AGAP00	XP_001650201.1serine--tRNA ligase, cytoplasmic
AAEL0021	uncharacterized LOC5580078	0.0466762317448444	0.701382801362505	0.916878235043446	NA	NA	XP_001844140.1 con	XP_021693067.1uncharacterized protein LOC5580078
AAEL0203	NA	0.0466776153809904	0.793959772227918	0.945793949302124	NA	aag02010	NA	XP_021703912.1multidrug resistance-associated protein 7
AAEL0041	MD-2-related lipid-recognition protein	0.0466895067973916	0.672991681754112	0.912968243848131	NA	aag04142	KXJ79265.1 hypothet	XP_001648436.1MD-2-related lipid-recognition protein
AAEL0020	40S ribosomal protein S10b	0.0467956548130661	0.723133759155411	0.921934742632737	NA	aag03010	ACF72877.1 ribosom	XP_001654587.140S ribosomal protein S10b
AAEL0111	vacuolar protein sorting-associated protein 29	0.0468706675954996	0.799278047951453	0.946215055875588	NA	aag04144	ETN61873.1 vacuolar	XP_001661488.1vacuolar protein sorting-associated protein 29
AAEL0033	suppressor of fused homolog	0.046879309697035	0.852668690055801	0.964653958545747	NA	aag04341	KXJ76391.1 hypothet	XP_001656738.1suppressor of fused homolog
AAEL0244	NA	0.0470386263908092	0.828071409173776	0.95691486967438	NA	NA	NA	XP_021696761.1uncharacterized protein LOC110675608
AAEL0021	uncharacterized LOC5573955	0.0471488837497555	0.801732172445753	0.946215055875588	NA	NA	KXJ74950.1 hypothet	XP_001654993.2uncharacterized protein LOC5573955
AAEL0113	U1 small nuclear ribonucleoprotein 70 kDa	0.047262431836553	0.740412680690029	0.928618984286445	NA	aag03040	KXJ72650.1 hypothet	XP_021697229.1U1 small nuclear ribonucleoprotein 70 kDa
AAEL0098	integumentary mucin C.1	0.0472982519358971	0.780016712157747	0.943566949556407	NA	NA	NA	XP_001660461.1integumentary mucin C.1
AAEL0078	zinc finger protein 879	0.0473302359825409	0.851585398363921	0.964536838405106	NA	NA	KXJ68226.1 hypothet	XP_001658708.2zinc finger protein 879
AAEL0064	uncharacterized LOC5567978	0.047365266424732	0.811181456908216	0.95010454605765	NA	NA	KFB44191.1 ubiquitin	XP_001845910.1ubiquitin-conjugating enzyme morgue
AAEL0023	peroxiredoxin-6	0.0476295797765633	0.675716161824238	0.914438668430353	480	aag01100	KFB50140.1 AGAP01	XP_019563346.1 peroxiredoxin-6-like
AAEL0125	E3 ubiquitin-protein ligase CHIP	0.0476720612243392	0.672349949386639	0.912897695079764	NA	aag04141 ; aag	KXJ75494.1 hypothet	XP_001656145.1E3 ubiquitin-protein ligase CHIP
AAEL0196	NA	0.047729890132839	0.715772576902341	0.920421919040713	NA	NA	NA	XP_021708868.1uncharacterized protein LOC5564326 isoform X1
AAEL0112	uncharacterized LOC5574653	0.0477920869892212	0.85504394800894	0.964980728557884	NA	aag03460	KXJ75861.1 hypothet	XP_001661569.2uncharacterized protein LOC5574653
AAEL0098	SIFamide-related peptide	0.0479008135204986	0.807205419466229	0.948098103944877	NA	NA	NA	XP_001654051.1SIFamide-related peptide
AAEL0145	E3 ubiquitin-protein ligase CBL-B-B	0.0479553963841751	0.795835694119755	0.945890571556554	NA	aag04144 ; aag	NA	XP_021696813.1E3 ubiquitin-protein ligase CBL-B-B isoform X1
AAEL0051	growth arrest and DNA damage-inducible proteins-interacting protein 1-l	0.0479784827863278	0.876610927594458	0.969652664055335	NA	NA	KXJ72551.1 hypothet	XP_001650410.2growth arrest and DNA damage-inducible proteins-interacting protein 1
AAEL0105	guanine nucleotide-binding protein G(q) subunit alpha	0.048038385379939	0.661378762716045	0.909942423634082	NA	aag04745	NA	XP_001660884.1guanine nucleotide-binding protein G(q) subunit alpha isoform X1
AAEL0233	NA	0.048187047221605	0.658484858738669	0.909580313002131	NA	aag03013	NA	XP_021713031.1eukaryotic translation initiation factor 3 subunit D
AAEL0195	NA	0.0482653494625025	0.706076508761649	0.917749125552377	NA	aag04013	NA	XP_019540884.1 mucin-19-like
AAEL0139	phosphorylated CTD-interacting factor 1	0.0483872454913762	0.794223988382726	0.945793949302124	NA	NA	NA	XP_001664114.2phosphorylated CTD-interacting factor 1
AAEL0237	NA	0.0484046939357923	0.921037238083929	0.980608390982715	NA	NA	NA	XP_021707969.1trypsin 5G1-like
AAEL0229	NA	0.0484756322015178	0.658546886591783	0.909580313002131	NA	aag01100 ; aag	NA	XP_021703756.1neutral alpha-glucosidase AB
AAEL0041	membrane-associated progesterone receptor component 1	0.0485593375747327	0.655251572383904	0.908212828600194	NA	NA	ETN58007.1 membra	XP_001648600.1membrane-associated progesterone receptor component 1
AAEL0114	possible lysine-specific histone demethylase 1	0.048560883301205	0.798220810381656	0.946215055875588	NA	NA	KXJ77405.1 hypothet	XP_001655324.1possible lysine-specific histone demethylase 1
AAEL0119	uncharacterized LOC5575641	0.0485655444342257	0.801845795013892	0.946215055875588	NA	NA	KXJ68820.1 hypothet	XP_001655789.2uncharacterized protein LOC5575641
AAEL0037	JNK1/MAPK8-associated membrane protein	0.0486170922712806	0.851540370584166	0.964536838405106	NA	NA	KXJ79598.1 hypothet	XP_001657116.1JNK1/MAPK8-associated membrane protein
AAEL0088	uncharacterized LOC5571180	0.0487734716592535	0.849542752077411	0.96410141039032	NA	NA	KXJ72956.1 hypothet	XP_001653478.2uncharacterized protein LOC5571180
AAEL0174	CCHC-type zinc finger protein CG3800	0.0488324232158037	0.689520102687881	0.916122517126697	NA	NA	NA	XP_011493210.1CCHC-type zinc finger protein CG3800
AAEL0025	uncharacterized LOC5575331	0.0488327472384735	0.884182882676501	0.970521528629931	NA	NA	KXJ71090.1 hypothet	XP_001655708.1uncharacterized protein LOC5575331
AAEL0070	proclotting enzyme	0.0490002266573367	0.799248895235296	0.946215055875588	NA	NA	KXJ68366.1 hypothet	XP_001652481.1proclotting enzyme
AAEL0246	NA	0.0490160576028684	0.670262207825532	0.912812708172959	NA	NA	NA	XP_021703695.1uncharacterized protein LOC5569804 isoform X1
AAEL0001	protein polybromo-1	0.0490211325154429	0.724584061321867	0.922598939018102	NA	NA	NA	XP_021709317.1protein polybromo-1 isoform X3
AAEL0075	ras-related protein Rab-43	0.0490366778121995	0.760331934436686	0.935871383184374	NA	NA	ETN61514.1 rab 19, 4	XP_001652810.1ras-related protein Rab-43
AAEL0129	26S proteasome regulatory subunit 7	0.0490711820784977	0.682130749349647	0.914629465876247	NA	aag03050	NA	XP_001663131.126S proteasome regulatory subunit 7
AAEL0265	NA	0.0492111349616923	0.851659582930811	0.964536838405106	NA	NA	NA	XP_021703464.1laminin subunit gamma-1
AAEL0087	V-type proton ATPase catalytic subunit A	0.0492199619980033	0.661735485400476	0.909942423634082	00195 ; 00190	aag01100 ; aag	ETN60811.1 ATP synt	XP_019537311.1 V-type proton ATPase catalytic subunit A-like
AAEL0124	RILP-like protein homolog	0.0492727344834965	0.825723115545078	0.955602219852662	NA	NA	KXJ75594.1 hypothet	XP_021699715.1RILP-like protein homolog
AAEL0222	NA	0.0493271111907602	0.758256588468381	0.935439571457873	NA	NA	NA	XP_001649277.2probable serine hydrolase
AAEL0113	probable glutamine--tRNA ligase	0.0493435242832403	0.648528195467898	0.906554905014882	970	aag01100 ; aag	CRK94415.1 CLUMA_	XP_001661633.1probable glutamine--tRNA ligase
AAEL0216	NA	0.0496058554311951	0.646193052431925	0.906554905014882	NA	NA	NA	XP_021702286.1cytokine receptor isoform X1
AAEL0195	NA	0.0496524651092968	0.715880815481143	0.920432480656348	NA	NA	NA	XP_021698228.1semaphorin-2A isoform X2
AAEL0081	alpha-(1,6)-fucosyltransferase	0.0496618172120269	0.721383009017361	0.921583569204186	00513 ; 00510 ; 00533	aag01100 ; aag	KFB40711.1 AGAP00	XP_019557337.1 alpha-(1,6)-fucosyltransferase
AAEL0271	NA	0.0497337364970736	0.882491389387833	0.970357617045106	NA	NA	NA	XP_021707174.1zinc finger and BTB domain-containing protein 17
AAEL0121	zinc finger protein-like 1 homolog	0.0498362072019572	0.731802248257509	0.925260275963946	NA	NA	ETN62540.1 zinc-fing	XP_001655852.1zinc finger protein-like 1 homolog
AAEL0258	NA	0.0499918220779615	0.890911871490042	0.972336404299425	NA	NA	NA	XP_021697326.1F-box only protein 33
AAEL0032	uncharacterized LOC5577649	0.0500095411734273	0.637842020706341	0.903683183146997	NA	NA	NA	XP_021693828.1uncharacterized protein LOC5577649 isoform X2
AAEL0057	active breakpoint cluster region-related protein	0.0500145615655177	0.708686705665129	0.918089462824826	NA	NA	XP_019551943.1 PRE	XP_021702784.1active breakpoint cluster region-related protein isoform X3
AAEL0070	store-operated calcium entry regulator STIMATE	0.0500337067869019	0.683022260500734	0.914629465876247	NA	NA	XP_019559009.1 PRE	XP_001652472.1store-operated calcium entry regulator STIMATE
AAEL0062	chaoptin	0.0502322453882821	0.683681408769703	0.914629465876247	NA	NA	KXJ73706.1 hypothet	XP_019558156.1 chaoptin-like
AAEL0129	uracil phosphoribosyltransferase homolog	0.050283444316651	0.819338189875552	0.953046394038027	NA	aag01100 ; aag	NA	XP_001656317.1uracil phosphoribosyltransferase homolog
AAEL0122	protein CDV3 homolog	0.0502975123845716	0.669296225706102	0.912812708172959	NA	NA	KXJ78364.1 hypothet	XP_021701696.1protein CDV3 homolog
AAEL0073	39S ribosomal protein S18a, mitochondrial	0.0503134494114772	0.688348835028102	0.916010156153545	NA	aag03010	KXJ71819.1 hypothet	XP_001658323.139S ribosomal protein S18a, mitochondrial
AAEL0044	chromobox protein homolog 1	0.0504521983002849	0.661691752859806	0.909942423634082	NA	NA	KXJ76976.1 hypothet	XP_021706118.1chromobox protein homolog 1
AAEL0265	NA	0.0504593674860085	0.694089543122567	0.916122517126697	NA	aag03008	NA	XP_021707584.1RNA-binding protein 28
AAEL0057	forkhead box protein E3	0.0504949549417615	0.661225628555078	0.909914495882133	NA	NA	XP_019530764.1 PRE	XP_001651392.2forkhead box protein E3 isoform X3
AAEL0049	triokinase/FMN cyclase	0.0507148628897805	0.777530910563513	0.942542410618554	NA	NA	KXJ76542.1 hypothet	XP_001650117.2triokinase/FMN cyclase
AAEL0270	NA	0.0507767255930665	0.880944123328489	0.969743049934758	NA	NA	NA	XP_021706593.1rRNA-processing protein FYV7-like
AAEL0196	NA	0.0508023280708962	0.658078008022198	0.909516556517693	NA	aag04013	NA	XP_021695876.1myocyte-specific enhancer factor 2 isoform X1
AAEL0043	WD repeat-containing protein 3	0.050922377492268	0.730634846563179	0.9246736154689	NA	aag03008	KXJ75472.1 hypothet	XP_001648996.2WD repeat-containing protein 3
AAEL0086	ATP-binding cassette sub-family G member 4	0.0510210711254062	0.864470631774612	0.967038506393142	NA	NA	KXJ81701.1 hypothet	XP_021703795.1ATP-binding cassette sub-family G member 4
AAEL0123	charged multivesicular body protein 5	0.0510749276865501	0.786243606293373	0.944549897171207	NA	aag04144	XP_001865745.1 cha	XP_001651451.1charged multivesicular body protein 5
AAEL0059	gamma-secretase subunit pen-2	0.0510802956730964	0.779499667277839	0.943566949556407	NA	aag04330	ETN62102.1 gamma-	XP_001651746.1gamma-secretase subunit pen-2
AAEL0007	snurportin-1	0.0511232299014366	0.799129048237265	0.946215055875588	NA	aag03013	KXJ81446.1 hypothet	XP_019536485.1 snurportin-1-like
AAEL0025	transmembrane protein 203	0.0511379359896377	0.693138626140695	0.916122517126697	NA	NA	KFB38198.1 AGAP01	XP_001655606.2transmembrane protein 203 isoform X2
AAEL0055	ras GTPase-activating protein-binding protein 1	0.0511722599311857	0.670284428688017	0.912812708172959	NA	NA	AKM16732.1 rasputi	XP_001651045.1ras GTPase-activating protein-binding protein 1 isoform X1
AAEL0050	ribosome biogenesis protein WDR12 homolog	0.0514588492244713	0.684665063434935	0.914629465876247	NA	NA	B0W517.2 RecName:	XP_001650205.1ribosome biogenesis protein WDR12 homolog
AAEL0069	cullin-2	0.0514705824940411	0.660389705625651	0.909580313002131	NA	aag04120	KXJ69821.1 hypothet	XP_019546249.1 cullin-2-like
AAEL0210	NA	0.0515117394915921	0.633273337021	0.902506916959539	NA	NA	NA	XP_019551899.1 junctophilin-1-like isoform X1
AAEL0199	NA	0.0515896876780321	0.692238497477039	0.916122517126697	NA	NA	NA	XP_001662267.1ORM1-like protein
AAEL0273	NA	0.0516029413219723	0.814098952784402	0.951522744001279	NA	NA	NA	XP_021697759.1uncharacterized protein LOC5565532
AAEL0034	coenzyme Q-binding protein COQ10, mitochondrial,coenzyme Q-binding	0.0516095041204012	0.687054695391015	0.916010156153545	NA	NA	XP_001870796.1 con	XP_001656848.2coenzyme Q-binding protein COQ10, mitochondrial

AAEL0037ɛ	zinc carboxypeptidase	0.0516228014199536	0.847513749372953	0.963412170689304	NA	NA	XP_019530911.1 PRE	XP_001664196.2zinc carboxypeptidase isoform X1
AAEL0252ɜ	NA	0.0516867408104869	0.757544951717006	0.934942146687217	NA	aag03018	NA	XP_021709561.1exosome complex component rrp45
AAEL0084ɜ	ER lumen protein-retaining receptor	0.0517654772171321	0.662265876629993	0.910392978301344	NA	NA	ETN62448.1 er lumer	XP_019547453.1 ER lumen protein-retaining receptor
AAEL0035ɛ	transcription termination factor 5, mitochondrial	0.0518127765891441	0.855637813952997	0.964980728557884	NA	NA	KXJ70443.1 hypothet	XP_001663942.2transcription termination factor 5, mitochondrial
AAEL0033ɛ	coiled-coil domain-containing protein 115	0.0518337718266632	0.858868519646006	0.965328928656042	NA	NA	KXJ68333.1 hypothet	XP_001656747.2coiled-coil domain-containing protein 115
AAEL0043ɛ	cysteine--tRNA ligase, cytoplasmic	0.0518369849254879	0.678605045738602	0.914481546552978	970	aag00970	XP_001855148.1 cys	XP_001648945.2cysteine--tRNA ligase, cytoplasmic
AAEL0227ɜ	NA	0.0518770548166316	0.742575151747306	0.930063501616811	NA	NA	NA	XP_021702046.1uncharacterized protein LOC110676873
AAEL0181ɛ	NA	0.0518774717746382	0.777013317400601	0.94228736778075	NA	aag01100 ; aag	NA	XP_011493159.2phosphatidylinositol 3-kinase catalytic subunit type 3
AAEL0278ɛ	NA	0.0519550053088415	0.634352766958856	0.902506916959539	NA	aag04624	NA	XP_019530987.1 FAS-associated factor 1 isoform X1
AAEL0280(	NA	0.0519946983572202	0.770884172527492	0.941147080452193	NA	NA	NA	XP_021704204.1uncharacterized protein LOC5571068
AAEL0182ɜ	NA	0.0520343236094105	0.728546818261693	0.923566968708516	NA	NA	NA	XP_021697834.1TBC1 domain family member 9
AAEL0147ɛ	putative glutamate synthase [NADPH]	0.052049651791614	0.698005475829729	0.916454522859806	00910 ; 00250	aag01100 ; aag	NA	XP_021697111.1putative glutamate synthase [NADPH] isoform X1
AAEL0015ɛ	endoribonuclease rege-1	0.0520529607374466	0.772150204990858	0.941147080452193	NA	NA	KXJ83000.1 hypothet	XP_001653593.1endoribonuclease rege-1
AAEL0216ɜ	NA	0.052203130748364	0.750520245535191	0.932554085844433	NA	NA	NA	XP_021705146.1protein SON
AAEL0245ɜ	NA	0.0522318214347515	0.863904918859177	0.967038506393142	NA	NA	NA	XP_021699894.1Down syndrome cell adhesion molecule-like protein Dscam2
AAEL0239ɜ	NA	0.0523237742328848	0.883881848264828	0.970521528629931	NA	NA	NA	XP_021697694.1putative leucine-rich repeat-containing protein DDB_G0290503
AAEL0181ɛ	NA	0.0523903470927597	0.785217186702811	0.944372632355572	NA	NA	NA	XP_001659377.1cyclin-dependent kinase 5 homolog
AAEL0148ɜ	endochitinase A	0.0524051268460239	0.726253578784674	0.923177868750456	NA	NA	NA	XP_021693605.1endochitinase A
AAEL0001ɜ	molybdenum cofactor biosynthesis protein 1	0.0524214509043488	0.682574033656422	0.914629465876247	00790 ; 00790	aag01100 ; aag	ETN65533.1 molybdc	XP_001657654.1molybdenum cofactor biosynthesis protein 1
AAEL0020ɜ	histidine--tRNA ligase, cytoplasmic	0.0524905186439192	0.700743002362076	0.916629842461068	970	aag00970	KXJ83570.1 hypothet	XP_001654585.1histidine--tRNA ligase, cytoplasmic isoform X2
AAEL0031ɜ	uncharacterized LOC5577150	0.0525525594500972	0.887603514707391	0.970996238637524	NA	NA	KFB51592.1 AGAP00	KFB51592.1AGAP006779-like protein
AAEL0019ɜ	PRA1 family protein 3	0.0525581032130458	0.635255207360869	0.902538767814767	NA	NA	KXJ79167.1 hypothet	XP_001654308.1PRA1 family protein 3
AAEL0268ɜ	NA	0.0525687502901996	0.660169945888561	0.909580313002131	NA	NA	NA	XP_021709495.1uncharacterized protein LOC110679374
AAEL0001ɜ	sodium/hydrogen exchanger 9B2	0.0527055387055463	0.815656789295201	0.952015623780574	NA	NA	XP_019525131.1 PRE	XP_021709102.1sodium/hydrogen exchanger 9B2 isoform X3
AAEL0112ɜ	type 1 phosphatidylinositol 4,5-bisphosphate 4-phosphatase	0.0527215955775117	0.621131773800405	0.89784080780888	4070	aag04070	KFB36012.1 AGAP00	XP_021699261.1type 1 phosphatidylinositol 4,5-bisphosphate 4-phosphatase
AAEL0061ɜ	uncharacterized protein ZK1073.1	0.0527784637139731	0.652685984094886	0.907272485895905	NA	NA	NA	XP_021704227.1uncharacterized protein ZK1073.1 isoform X8
AAEL0281ɜ	NA	0.0528265109070638	0.723718075806969	0.922102780092934	NA	NA	NA	XP_021696770.1neurexin-4 isoform X4
AAEL0102ɜ	methylosome subunit pICln	0.0529101334201132	0.806707003689378	0.948040966581563	NA	aag03013	NA	XP_001654387.1methylosome subunit pICln isoform X2
AAEL0012(	uncharacterized LOC5569073	0.052928939676911	0.717443755737782	0.921283483156443	NA	NA	XP_019535891.1 PRE	XP_019535890.1 uncharacterized membrane protein DDB_G0293934-like isoform X1
AAEL0049ɜ	uncharacterized LOC5565721	0.0530004249109953	0.616629348349308	0.897164966965308	NA	NA	KXJ80329.1 hypothet	XP_021705160.1uncharacterized protein LOC5565721
AAEL0233ɜ	NA	0.0530006094985631	0.631585872331743	0.902506916959539	NA	NA	NA	XP_021709018.1venom dipeptidyl peptidase 4 isoform X1
AAEL0098(	V-type proton ATPase subunit D 1	0.053010017011315	0.617550495841403	0.897318336565571	NA	aag01100 ; aag	NA	XP_001660426.1V-type proton ATPase subunit D 1
AAEL0062ɜ	uncharacterized LOC5567705	0.0531049256547295	0.807253710542068	0.948098103944877	NA	NA	NA	XP_021699784.1uncharacterized protein LOC5567705 isoform X2
AAEL0140ɛ	activating signal cointegrator 1 complex subunit 3	0.0531322334354138	0.730183396233883	0.924483711082029	NA	NA	NA	XP_021709809.1activating signal cointegrator 1 complex subunit 3
AAEL0047ɜ	chromodomain-helicase-DNA-binding protein 1	0.0532079517524735	0.646298168546861	0.906554905014882	NA	NA	XP_019549572.1 PRE	XP_001649665.2chromodomain-helicase-DNA-binding protein 1 isoform X1
AAEL0093ɜ	T-complex protein 1 subunit alpha	0.0533189264962024	0.621686601541541	0.89816390813084	NA	NA	NA	XP_001659922.2T-complex protein 1 subunit alpha
AAEL0133ɜ	protein lethal(2)essential for life	0.0534895736095763	0.681434399784859	0.914629465876247	NA	NA	NA	XP_001663499.2protein lethal(2)essential for life
AAEL0061ɜ	isopentenyl-diphosphate Delta-isomerase 1	0.0536027618197245	0.723331307083086	0.921934742632737	900	aag01100 ; aag	ETN62790.1 isopente	XP_001657533.1isopentenyl-diphosphate Delta-isomerase 1
AAEL0071ɛ	ubiquilin-1	0.0536687726431706	0.608888900884536	0.893547770777888	NA	aag04141	CRK86955.1 CLUMA_	XP_019532912.1 ubiquilin-1
AAEL0082ɜ	alpha-tocopherol transfer protein	0.0536824643214675	0.878360276396671	0.969652664055335	NA	NA	NA	XP_021707295.1alpha-tocopherol transfer protein isoform X1
AAEL0135ɜ	syntaxin-5	0.053980388310781	0.724827889058383	0.922654064893518	NA	aag04130	NA	XP_019554933.1 syntaxin-5
AAEL0028ɜ	trafficking protein particle complex subunit 5	0.0539930227432749	0.797698861517631	0.946174906378608	NA	NA	ETN62705.1 hypothe	XP_001656000.1trafficking protein particle complex subunit 5
AAEL0105ɛ	vesicular integral-membrane protein VIP36	0.054075365498448	0.64934451148715	0.906609198022654	NA	aag04141	KXJ72087.1 hypothet	XP_021693955.1vesicular integral-membrane protein VIP36
AAEL0061ɜ	golgin subfamily A member 4	0.0540908018071466	0.850294856978103	0.964536838405106	NA	NA	KXJ74442.1 hypothet	XP_001657531.1golgin subfamily A member 4
AAEL0082ɜ	eukaryotic translation initiation factor 2A	0.0541035178530543	0.673249719283021	0.912968243848131	NA	NA	KXJ75846.1 hypothet	XP_001653135.2eukaryotic translation initiation factor 2A
AAEL0129ɜ	cyclin-dependent kinase 14	0.0541385337271734	0.735581321297519	0.926727727078281	NA	NA	NA	XP_021698650.1cyclin-dependent kinase 14 isoform X2
AAEL0025ɜ	uncharacterized LOC5575316	0.0541489570004531	0.851223351332081	0.964536838405106	NA	NA	KXJ75009.1 hypothet	XP_021706347.1uncharacterized protein LOC5575316
AAEL0059ɜ	uncharacterized LOC5579864	0.0541880457963851	0.878360276441191	0.969652664055335	NA	NA	KXJ83019.1 hypothet	XP_001664326.2uncharacterized protein LOC5579864
AAEL0123ɜ	putative serine protease F56F10.1	0.0542099287960622	0.797217870904383	0.945982283238732	NA	NA	XP_001868268.1 thy	XP_001662504.2putative serine protease F56F10.1 isoform X1
AAEL0032ɜ	NA	0.0542502403191377	0.838497224009435	0.962348963212321	NA	NA	KXJ69534.1 hypothet	XP_021705900.1 angiopoietin-related protein 6
AAEL0198(	NA	0.0543218293867847	0.908563664520476	0.976591851526389	NA	aag04080	NA	XP_019546047.2 alpha-2B adrenergic receptor
AAEL0062ɛ	sex peptide receptor	0.0543340940452611	0.807696409567132	0.948190794639012	NA	NA	XP_019546126.1 PRE	XP_001651852.3sex peptide receptor isoform X1
AAEL0068ɛ	uncharacterized LOC5568483	0.0544562987421894	0.877891506565676	0.969652664055335	NA	NA	KFB36637.1 AGAP00	KFB36637.1AGAP007656-like protein
AAEL0061ɛ	lysosomal aspartic protease	0.0545156878511395	0.643395948289208	0.906249734602371	NA	aag04140 ; aag	XP_019543076.1 PRE	XP_001657556.2lysosomal aspartic protease
AAEL0088(	probable 28S ribosomal protein S26, mitochondrial	0.0545843565221208	0.756112276120583	0.934127170333935	NA	NA	KXJ83360.1 hypothet	XP_001659519.2probable 28S ribosomal protein S26, mitochondrial
AAEL0005ɛ	serine/threonine-protein kinase 26	0.0546629168932732	0.631073309285938	0.902506916959539	NA	NA	XP_019532606.1 PRE	XP_001648658.2germinal center kinase 1 isoform X2
AAEL0132ɜ	succinate dehydrogenase assembly factor 4, mitochondrial	0.0547819614502879	0.722777397977098	0.921934742632737	NA	NA	NA	XP_001656560.1succinate dehydrogenase assembly factor 4, mitochondrial
AAEL0034(	uncharacterized LOC5578108	0.0548231304990603	0.717001120978255	0.921100700159417	NA	NA	NA	XP_001656806.1uncharacterized protein LOC5578108 isoform X2
AAEL0023ɜ	myotubularin-related protein 14	0.0549187923010206	0.702726470485024	0.917106722424789	NA	aag01100 ; aag	XP_019538312.1 PRE	XP_021701245.1myotubularin-related protein 14
AAEL0001ɜ	zinc finger protein 70	0.055151954612003	0.801098541881502	0.946215055875588	NA	NA	KXJ83435.1 hypothet	XP_001657706.1zinc finger protein 70 isoform X1
AAEL0035ɜ	uncharacterized LOC5578337	0.0552570402521071	0.885131214847563	0.970680185797554	NA	NA	KXJ82567.1 hypothet	XP_001656906.2uncharacterized protein LOC5578337
AAEL0175ɜ	transcriptional regulatory protein AlgP	0.0552965696730516	0.694634352467696	0.916122517126697	NA	aag03010	NA	AAY41435.1ribosomal protein L23a
AAEL0136ɜ	homogentisate 1,2-dioxygenase	0.0553987758432812	0.707080693394105	0.91797784850989	00350 ; 00643	aag01100 ; aag	NA	XP_001663831.2homogentisate 1,2-dioxygenase
AAEL0086(	inosine triphosphate pyrophosphatase	0.0554122828354909	0.774180303082857	0.941230906670349	00230 ; 00740 ; 00770 ;	aag01100 ; aag	KXJ68531.1 hypothet	XP_001653334.1inosine triphosphate pyrophosphatase
AAEL0255ɜ	NA	0.0555253376315129	0.639589048264411	0.904449291387452	NA	NA	NA	XP_019555856.1 myosin-IA-like
AAEL0037ɜ	bifunctional coenzyme A synthase	0.0555374061893066	0.748901130644503	0.932554085844433	770	aag01100 ; aag	KXJ82428.1 hypothet	XP_001657223.1bifunctional coenzyme A synthase
AAEL0194ɜ	NA	0.0555646035720844	0.880784093479091	0.969743049934758	NA	NA	NA	XP_021707135.1dual specificity protein phosphatase MPK-4
AAEL0121ɜ	rab-like protein 3	0.0555881543020828	0.695078867530504	0.916122517126697	NA	NA	XP_019553292.1 PRE	XP_001662311.1rab-like protein 3 isoform X1
AAEL0001ɜ	uncharacterized LOC5567796	0.0556254030743538	0.797129106562626	0.945982283238732	NA	NA	KXJ83441.1 hypothet	XP_001657700.2uncharacterized protein LOC5567796
AAEL0120ɜ	protein windpipe	0.0556702097679971	0.655218118345542	0.908212828600194	NA	NA	KXJ82829.1 hypothet	XP_001662247.2protein windpipe
AAEL0265ɜ	NA	0.0557011926543809	0.698031678281559	0.916454522859806	NA	NA	NA	NA
AAEL0290ɜ	NA	0.0557312236621135	0.673065129924405	0.912968243848131	NA	NA	NA	XP_001650589.1uncharacterized protein LOC5566223 isoform X2
AAEL0035ɜ	mini-chromosome maintenance complex-binding protein	0.0557437133268893	0.844373881513649	0.963198678766584	NA	NA	KXJ73685.1 hypothet	XP_001656900.2mini-chromosome maintenance complex-binding protein
AAEL0134ɜ	vacuolar protein sorting-associated protein 53 homolog	0.0557466410310296	0.790464859416478	0.945793949302124	NA	NA	NA	XP_001663661.1vacuolar protein sorting-associated protein 53 homolog
AAEL0243ɜ	NA	0.0557549671358009	0.849244906633263	0.963951107788243	NA	NA	NA	XP_021711937.1 SWI/SNF complex subunit SMARCC2-like
AAEL0046ɛ	ceramide-1-phosphate transfer protein	0.0557557778175999	0.746564931584441	0.931365937538534	NA	NA	KXJ70344.1 hypothet	XP_001649551.1ceramide-1-phosphate transfer protein
AAEL0103ɜ	porphobilinogen deaminase	0.0558243522160293	0.727606906135039	0.923566968708516	860	aag01100 ; aag	NA	ABF18151.1hydroxymethylbilane synthase-like protein
AAEL0041ɜ	cryptochrome-1	0.055910070032975	0.643725635586762	0.906297934312942	NA	NA	KXJ69012.1 hypothet	Q17DK5.1RecName: Full=Cryptochrome-1
AAEL0250ɜ	NA	0.055935332048601	0.763642776850818	0.937987145975992	NA	NA	NA	XP_001658569.2uncharacterized protein LOC5569484
AAEL0090ɜ	NEDD8-conjugating enzyme Ubc12	0.0559598397722629	0.635822342788365	0.902538767814767	NA	aag04120	ETN63803.1 ubiquitir	XP_001653639.1NEDD8-conjugating enzyme Ubc12
AAEL0035ɜ	monocarboxylate transporter 10	0.05596259397193	0.684806692374111	0.914629465876247	NA	NA	KFB49640.1 AGAP00	XP_021700520.1monocarboxylate transporter 10
AAEL0128ɛ	upstream activation factor subunit spp27	0.0560488959262692	0.610001623888231	0.894093217379261	NA	NA	NA	XP_001663011.1upstream activation factor subunit spp27

AAEL01262	serine-arginine protein 55	0.0560735991859778	0.605505873863612	0.893547770777888	NA	aag03040	XP_019528261.1 PRE	XP_021693638.1serine-arginine protein 55 isoform X1
AAEL00697	serine/threonine-protein phosphatase PP2A 65 kDa regulatory subunit	0.0560868696229486	0.602441356152466	0.892663752727165	NA	aag03015 ; aag	ETN60749.1 serine/t	XP_001652452.1serine/threonine-protein phosphatase PP2A 65 kDa regulatory subunit
AAEL00327	titin	0.0562819571237081	0.648649950281224	0.906554905014882	NA	NA	KXJ68128.1 hypothet	XP_019545582.1 titin
AAEL00911	NA	0.0563206395477433	0.676968375856688	0.914438668430353	NA	NA	NA	NP_001345310.1protein doublesex isoform 1
AAEL00635	uncharacterized LOC5567871	0.0564389671842288	0.815013776609528	0.952015623780574	NA	NA	NA	ABF18030.1putative salivary mucin 2
AAEL02814	NA	0.0564476878598836	0.619892704362892	0.897318336565571	NA	NA	NA	NA
AAEL00251	serine hydroxymethyltransferase, cytosolic	0.0566388723180587	0.749139621745335	0.932554085844433	00630 ; 00670 ; 00260 ;	aag01100 ; aag	KXJ69944.1 hypothet	XP_021697775.1serine hydroxymethyltransferase, cytosolic isoform X1
AAEL00412	fatty acid hydroxylase domain-containing protein 2	0.0568549349118998	0.700010716277363	0.916629842461068	NA	NA	KXJ83247.1 hypothet	XP_001648508.2fatty acid hydroxylase domain-containing protein 2 isoform X1
AAEL01706	protein jagunal	0.0569208788962594	0.611052212691254	0.894646314231935	NA	NA	NA	XP_011493321.1protein jagunal isoform X1
AAEL02501	NA	0.0569321607585994	0.691508376275965	0.916122517126697	NA	NA	NA	XP_019538602.1 flotillin-1 isoform X1
AAEL00626	solute carrier family 25 member 35	0.0569503217255707	0.718173455427281	0.921577483871404	NA	NA	XP_019537841.1 PRE	XP_021699791.1solute carrier family 25 member 35
AAEL00019	alkaline phosphatase, tissue-nonspecific isozyme	0.0570998364414533	0.794074311124311	0.945793949302124	00790 ; 00730	NA	KXJ78655.1 hypothet	XP_001658940.1alkaline phosphatase, tissue-nonspecific isozyme
AAEL00248	ATP-dependent RNA helicase Ddx1	0.0571668335779547	0.759849430638158	0.935871383184374	NA	NA	KXJ68738.1 hypothet	XP_001655375.2ATP-dependent RNA helicase Ddx1
AAEL00737	dihydrofolate reductase	0.0574059060696373	0.753763261264276	0.933480114379509	00790 ; 00670	aag01100 ; aag	KFB45515.1 AGAP01	XP_001652716.2dihydrofolate reductase
AAEL00288	armadillo segment polarity protein	0.0574838379680963	0.608857293413035	0.893547770777888	NA	aag04310	KXJ76424.1 hypothet	XP_001662670.1armadillo segment polarity protein isoform X1
AAEL00674	cap-specific mRNA (nucleoside-2'-O-)-methyltransferase 2	0.0575527435719491	0.811622872822975	0.950197668666287	NA	NA	ETN64292.1 hypothe	XP_001652191.1cap-specific mRNA (nucleoside-2'-O-)-methyltransferase 2
AAEL01163	microfibril-associated glycoprotein 4	0.0575791615868038	0.86577713780836	0.967927335900485	NA	NA	XP_019548314.1 PRE	XP_001661796.2microfibril-associated glycoprotein 4
AAEL00942	uncharacterized protein CG5098	0.0575877884221617	0.705365668181828	0.917749125552377	NA	NA	NA	XP_021698462.1uncharacterized protein CG5098
AAEL01217	gastrula zinc finger protein XlCGF53.1	0.0575933807207839	0.816260484653153	0.952319314767154	NA	NA	XP_019554390.1 PRE	XP_021699446.1gastrula zinc finger protein XlCGF53.1 isoform X1
AAEL02735	NA	0.0577007125154454	0.709863737374598	0.918600212532843	NA	NA	NA	NA
AAEL01251	NF-kappa-B essential modulator	0.0577875992014369	0.750950913241883	0.932554085844433	NA	aag04624	KXJ74495.1 hypothet	XP_001656112.2NF-kappa-B essential modulator
AAEL00723	uncharacterized LOC5568939	0.0578792834672899	0.856511565898674	0.964980728557884	NA	NA	KXJ83144.1 hypothet	XP_001658261.2uncharacterized protein LOC5568939
AAEL00706	ADP-ribosylation factor 1	0.0579753206008833	0.620719426227604	0.897385728224103	NA	aag04144	NP_476955.1 ADP rib	NP_476955.1ADP ribosylation factor at 79F, isoform B
AAEL02660	NA	0.0579936271488086	0.809408609509687	0.948933370455872	NA	NA	NA	XP_021704232.1L-ascorbate oxidase
AAEL02743	NA	0.0580080622461251	0.700069840163256	0.916629842461068	NA	aag03013 ; aag	NA	XP_021700543.1RNA-binding protein with serine-rich domain 1-A-like
AAEL00243	modifier of mdg4	0.0580587911617349	0.601747884380623	0.892353896540881	NA	NA	XP_019542242.1 PRE	XP_001655268.1modifier of mdg4 isoform X5
AAEL01560	NA	0.0581318548131872	0.787141274177743	0.944610590112622	NA	aag03018	NA	XP_001647606.2m7GpppN-mRNA hydrolase
AAEL01714	glycine-rich protein 5	0.0581424155248502	0.675814530847448	0.914438668430353	NA	NA	NA	XP_011493147.1glycine-rich protein 5
AAEL01089	WD40 repeat-containing protein SMU1	0.0581494701117758	0.765958936660328	0.939178856256477	NA	NA	XP_001842407.1 WD	XP_001655027.1WD40 repeat-containing protein SMU1
AAEL02451	NA	0.0582102472889508	0.846618055109604	0.963198678766584	04151 ; 05165 ; 04714 ;	NA	NA	XP_001661758.1serine/threonine-protein kinase OSR1
AAEL01995	NA	0.0582729145826075	0.727823116172509	0.923566968708516	NA	NA	NA	XP_021696099.1cAMP-dependent protein kinase type I regulatory subunit isoform X1
AAEL00137	spermine synthase	0.0582736499718209	0.685440008127825	0.914648920358504	00410 ; 00330 ; 00270 ;	aag01100 ; aag	KXJ83054.1 hypothet	XP_001653177.1spermine synthase isoform X2
AAEL01007	CTD small phosphatase-like protein 2	0.058346468914422	0.6384430446493	0.90377215326912	NA	NA	NA	XP_021699391.1CTD small phosphatase-like protein 2
AAEL01031	tudor domain-containing protein 7A	0.0583871607598243	0.592502571926538	0.888529762455765	NA	NA	NA	XP_001654423.1tudor domain-containing protein 7A isoform X1
AAEL01964	NA	0.0583990956916941	0.722793277373348	0.921934742632737	NA	NA	NA	XP_021697016.1zinc finger protein 26 isoform X1
AAEL00315	protein RTF2 homolog	0.0584952454437234	0.745651281624544	0.931365937538534	NA	NA	KXJ68881.1 hypothet	XP_001656500.1protein RTF2 homolog
AAEL01274	T-complex protein 1 subunit theta	0.0585222953129623	0.590209429090183	0.887668474886052	NA	NA	XP_001847689.1 T-c	XP_001662847.2T-complex protein 1 subunit theta
AAEL00174	fatty acyl-CoA reductase wat	0.0585420370093965	0.755440126979792	0.934127170333935	73	NA	KXJ76240.1 hypothet	XP_021696300.1fatty acyl-CoA reductase wat
AAEL00223	SUN domain-containing ossification factor	0.0585516894387148	0.616567428211831	0.897164966965308	NA	NA	NA	XP_001654972.2SUN domain-containing protein 2 isoform X1
AAEL00504	serine palmitoyltransferase small subunit A	0.0585517236839874	0.667852866875041	0.912418872165226	NA	NA	XP_019544008.1 PRE	XP_021693928.1serine palmitoyltransferase small subunit A isoform X1
AAEL00348	glycosyltransferase 25 family member	0.0585538417043063	0.645386962210204	0.906554905014882	NA	aag01100 ; aag	ETN63890.1 glycosyl	XP_001656834.1glycosyltransferase 25 family member
AAEL01490	large subunit GTPase 1 homolog	0.0586269161777866	0.663751023364761	0.911573284643631	NA	aag03008	NA	XP_021710265.1large subunit GTPase 1 homolog
AAEL02597	NA	0.0586717759008998	0.786958749771382	0.944549897171207	NA	NA	NA	XP_021694031.1uncharacterized protein LOC5577579 isoform X1
AAEL00878	CAAX prenyl protease 1 homolog	0.0587176295629019	0.639526110203495	0.904449291387452	900	aag00900	KXJ78516.1 hypothet	XP_001659506.1CAAX prenyl protease 1 homolog
AAEL02692	NA	0.0587503195804497	0.787605181834883	0.944774185961398	NA	aag03040	NA	XP_021699260.1zinc finger matrin-type protein 2
AAEL00533	D-3-phosphoglycerate dehydrogenase	0.0587752008551841	0.670021876931511	0.912812708172959	NA	aag01100 ; aag	XP_001850727.1 d-3	XP_001650713.1D-3-phosphoglycerate dehydrogenase
AAEL01750	presenilin homolog	0.0588922210863999	0.69150942706204	0.916122517126697	NA	aag04310 ; aag	NA	XP_011493212.2presenilin homolog isoform X2
AAEL01020	uncharacterized LOC5573031	0.0589013506882119	0.679161462446806	0.914481546552978	NA	NA	NA	XP_001654360.1uncharacterized protein LOC5573031 isoform X1
AAEL00269	uncharacterized LOC5575664	0.0589257878331673	0.820003282446991	0.953458454015316	NA	NA	XP_001866240.1 bea	XP_001866240.1beat protein
AAEL00399	SET and MYND domain-containing protein 4	0.0590171240236941	0.731574656846713	0.925260275963946	NA	NA	XP_019530462.1 PRE	XP_021709764.1SET and MYND domain-containing protein 4
AAEL01816	NA	0.0590360338769371	0.768755515149167	0.940525313495725	NA	NA	NA	XP_021708517.1USP6 N-terminal-like protein isoform X3
AAEL01390	gamma-tubulin complex component 2 homolog	0.0590843463725752	0.736950086088656	0.92756321873722	NA	NA	NA	XP_001664107.1gamma-tubulin complex component 2 homolog isoform X2
AAEL01109	polypeptide N-acetylgalactosaminyltransferase 2	0.0591042229675546	0.750978054268843	0.932554085844433	NA	aag01100 ; aag	KXJ71333.1 hypothet	XP_021700118.1polypeptide N-acetylgalactosaminyltransferase 2
AAEL00970	thioredoxin, mitochondrial	0.0591480791181912	0.608445079330015	0.893547770777888	NA	NA	NA	XP_001654006.1thioredoxin, mitochondrial
AAEL00843	A-kinase anchor protein 1, mitochondrial	0.0591742450238208	0.618252945306288	0.897318336565571	NA	NA	XP_019533877.1 PRE	XP_001659213.2A-kinase anchor protein 1, mitochondrial isoform X1
AAEL00024	GTP-binding protein 1	0.0591933648742183	0.651657442271246	0.907172139858737	NA	NA	ETN66820.1 GTP bin	XP_001660145.2GTP-binding protein 1
AAEL00031	protein pigeon	0.0592155266983983	0.728814690581361	0.923566968708516	NA	NA	XP_019543275.1 PRE	XP_021702284.1protein pigeon isoform X2
AAEL01362	40S ribosomal protein S5	0.0592467221030319	0.697401012876149	0.91639070690937	NA	aag03010	NA	XP_001656889.140S ribosomal protein S5
AAEL01494	uncharacterized protein CG16817	0.0595168241471484	0.607986785627241	0.893547770777888	NA	aag01100 ; aag	NA	XP_001650059.1uncharacterized protein CG16817
AAEL00645	zinc finger protein 37 homolog	0.0595960633367959	0.840882496776016	0.963198678766584	NA	NA	KXJ76170.1 hypothet	XP_001657841.2zinc finger protein 37 homolog
AAEL01301	uncharacterized LOC5577112	0.0596153837279457	0.711473821373547	0.918935795219545	NA	NA	NA	XP_021701504.1uncharacterized protein LOC5577112
AAEL01374	golgin subfamily A member 7	0.0596469068992962	0.695644184304528	0.916122517126697	NA	NA	NA	XP_001663931.1golgin subfamily A member 7
AAEL00928	GTP-binding nuclear protein Ran	0.0596682033136134	0.577655065181461	0.881961491587822	NA	aag03013 ; aag	NA	XP_001659895.1GTP-binding nuclear protein Ran
AAEL00340	NA	0.0597206795204167	0.775121003831558	0.941605739572012	NA	NA	XP_019527231.1 PRE	XP_021706798.1zinc finger protein 227-like
AAEL01247	JNK-interacting protein 3	0.0597527280487933	0.622824244640868	0.898271200863489	NA	NA	XP_019537018.1 PRE	XP_021699729.1JNK-interacting protein 3 isoform X2
AAEL01037	protein SHQ1 homolog	0.0601083169756564	0.851395477613045	0.964536838405106	NA	NA	NA	XP_021699051.1protein SHQ1 homolog
AAEL00210	histone H1, gonadal	0.0601204385318864	0.711137861543181	0.918935795219545	NA	NA	KXJ72616.1 hypothet	XP_001654695.2histone H1, gonadal
AAEL00643	dual specificity protein phosphatase 3	0.0602364866202817	0.592763371208855	0.888529762455765	04660 ; 04658	NA	KXJ74690.1 hypothet	XP_001657812.1dual specificity protein phosphatase 3 isoform X4
AAEL02368	NA	0.0602694967279423	0.830261760922255	0.958428949419039	NA	NA	NA	XP_021693124.1protein glass isoform X2
AAEL01990	NA	0.0602813008513147	0.842521781959716	0.963198678766584	NA	NA	NA	XP_021704450.1polyhomeotic-proximal chromatin protein isoform X2
AAEL00696	uncharacterized LOC5568620	0.0603064512311047	0.687294457458404	0.916010156153545	NA	NA	KXJ70487.1 hypothet	XP_001652454.1uncharacterized protein LOC5568620
AAEL02539	NA	0.0603831488363745	0.756173418371711	0.934127170333935	NA	NA	NA	XP_021693609.1vacuolar protein sorting-associated protein 13B
AAEL00825	threonine aspartase 1	0.0603843365232087	0.812497819014044	0.950268140513817	NA	NA	KXJ75395.1 hypothet	XP_001653128.1threonine aspartase 1
AAEL01497	facilitated trehalose transporter Tret1	0.0604319713585594	0.632107952009633	0.902506916959539	NA	NA	NA	XP_019534717.1 facilitated trehalose transporter Tret1 isoform X3
AAEL00170	gamma-butyrobetaine dioxygenase	0.0604341974006111	0.841587930699979	0.963198678766584	NA	aag01100 ; aag	KXJ82107.1 hypothet	XP_001653834.1gamma-butyrobetaine dioxygenase
AAEL01018	39S ribosomal protein L51, mitochondrial	0.06053999526303	0.688576385560888	0.916010156153545	NA	NA	NA	XP_001654295.139S ribosomal protein L51, mitochondrial
AAEL00123	lysosomal thioesterase PPT2 homolog	0.0606241942064977	0.733593527280999	0.925998304257854	NA	aag01100 ; aag	KXJ75544.1 hypothet	XP_001658534.2lysosomal thioesterase PPT2 homolog
AAEL01441	uncharacterized LOC5564378	0.0606783786601301	0.821182884477539	0.954106679666959	NA	NA	NA	XP_021693779.1uncharacterized protein LOC5564378
AAEL01988	NA	0.0608910834152777	0.597560911665148	0.890198398760352	NA	aag04142	NA	XP_021702837.1Niemannck C1 protein isoform X1
AAEL00782	protein argonaute-3	0.0609128283456016	0.582351583763558	0.884436255783506	NA	aag04320	KXJ80216.1 hypothet	XP_001652945.1protein argonaute-3
AAEL02030	NA	0.0609505867629862	0.8242801762076	0.954996283354524	04660 ; 04658	NA	NA	XP_019556712.1 tyrosine-protein phosphatase vhp-1-like
AAEL00025	nuclear factor 1	0.0609933998337535	0.858956016934978	0.965328928656042	NA	NA	XP_019555649.1 PRE	XP_021694494.1nuclear factor 1 isoform X1

AAEL00243	symplekin	0.0610213739629957	0.72175035888731	0.921668998401475	NA	aag03015	KXJ75644.1 hypothet	XP_019932162.1 symplekin-like
AAEL00208	sin3 histone deacetylase corepressor complex component SDS3	0.0610941209427167	0.775377115588648	0.941792531368366	NA	NA	KXJ79250.1 hypothet	XP_021712942.1sin3 histone deacetylase corepressor complex component SDS3
AAEL00407	iron-sulfur cluster assembly scaffold protein IscU	0.0611343574370317	0.60740616680144	0.893547770777888	NA	NA	ETN66548.1 nitrogen	KFB48253.1AGAP005813-like protein
AAEL01989	NA	0.0611498066208076	0.832243885402582	0.95920694307046	NA	NA	NA	XP_021697345.1protein dead ringer isoform X5
AAEL02804	NA	0.061174639047014	0.655563335520918	0.908328180248866	NA	NA	NA	NA
AAEL00611	39S ribosomal protein L55, mitochondrial	0.0612825278790215	0.743683677698271	0.930691853961579	NA	NA	KXJ62435.1 hypothet	XP_001651805.139S ribosomal protein L55, mitochondrial
AAEL00566	serine protease inhibitor 27A	0.0612926025914304	0.654494395880863	0.9078470652541	NA	NA	KXJ74881.1 hypothet	XP_001651232.2serine protease inhibitor 27A
AAEL00454	coatomer subunit beta	0.0613024355949972	0.582901717836115	0.884917975521096	NA	NA	KFB52575.1 AGAP00	XP_001649392.1coatomer subunit beta
AAEL02722	NA	0.0613091737240268	0.60835511027528	0.893547770777888	NA	NA	NA	NA
AAEL01555	NA	0.061551758276291	0.76765337593585	0.939827742988808	NA	NA	NA	XP_001647648.2succinate--hydroxymethylglutarate CoA-transferase
AAEL01136	uncharacterized LOC5574726	0.0615747025820528	0.755712388422231	0.934127170333935	NA	NA	KXJ82632.1 hypothet	XP_021698639.1uncharacterized protein LOC5574726
AAEL00215	soma ferritin	0.0615970110654767	0.584776026931307	0.885175928718656	NA	NA	KXJ69687.1 hypothet	XP_001654814.1soma ferritin
AAEL00748	mediator of RNA polymerase II transcription subunit 27	0.0616090856749583	0.817519433826638	0.952514760254484	NA	NA	KFB46004.1 AGAP00	XP_001652785.1mediator of RNA polymerase II transcription subunit 27
AAEL02384	NA	0.0616958897607948	0.77116917885042	0.941147080452193	NA	NA	NA	XP_019528973.1 cadherin-87A-like
AAEL00605	uncharacterized LOC5567400	0.0617786175681182	0.775502769073264	0.941820836876359	NA	NA	NA	XP_021704781.1uncharacterized protein LOC5567400 isoform X1
AAEL01093	ataxin-1	0.0618066412397684	0.755566330818675	0.934127170333935	NA	aag04330	KXJ71232.1 hypothet	XP_019558787.1 ataxin-1
AAEL00764	protein Tube	0.0619342424431263	0.784219155049108	0.944372632355572	NA	aag04624	KXJ82023.1 hypothet	XP_001658540.2protein Tube
AAEL01017	solute carrier family 35 member G1	0.0619543143129194	0.8783602765069	0.969652664055335	NA	NA	NA	XP_001654273.2solute carrier family 35 member G1
AAEL02568	NA	0.0620018960195337	0.640534751224342	0.904464102888003	NA	NA	NA	NA
AAEL01040	EH domain-containing protein 3	0.0621656048042293	0.605865005969274	0.893547770777888	NA	aag04144	NA	XP_001654538.1EH domain-containing protein 3 isoform X2
AAEL00493	uncharacterized LOC5565682	0.0622199249967144	0.560349942844097	0.87800595572912	NA	NA	KXJ81824.1 hypothet	XP_019541538.1 histone-lysine N-methyltransferase SETD1B-like
AAEL01402	transcription initiation factor TFIID subunit 2	0.0622282032823853	0.678476184116391	0.914481546552978	NA	aag03022	NA	XP_001664244.2transcription initiation factor TFIID subunit 2
AAEL01056	chromosome-associated kinesin KIF4A	0.0622891977166659	0.83178166624726	0.958914419043759	NA	NA	KXJ77915.1 hypothet	XP_021703730.1chromosome-associated kinesin KIF4A
AAEL02065	NA	0.0623139554359217	0.578831890371337	0.882283691803025	NA	aag03018	NA	XP_021701849.1CCR4-NOT transcription complex subunit 1
AAEL00790	cleavage and polyadenylation specificity factor 73	0.0623324336697773	0.756570680098122	0.934188766995886	NA	aag03015	XP_001866010.1 clea	XP_001652976.1cleavage and polyadenylation specificity factor 73
AAEL00179	maf-like protein CPR_2112	0.0623791858714071	0.587984383494887	0.886401261871374	NA	NA	KXJ74398.1 hypothet	XP_019536345.1 N-acetylserotonin O-methyltransferase-like protein
AAEL01976	NA	0.0623877013581455	0.720727661726446	0.921583569204186	NA	NA	NA	XP_021703168.1AF4/FMR2 family member 4 isoform X1
AAEL01115	ribosomal protein 63, mitochondrial	0.0624643993983228	0.600309501820982	0.892093596129407	NA	NA	ETN64402.1 hypothe	XP_001661454.1ribosomal protein 63, mitochondrial
AAEL00557	GTP-binding protein 128up	0.0625051313743456	0.623593046772072	0.898575511493361	970	NA	ETN58057.1 develop	XP_001651096.1GTP-binding protein 128up
AAEL00782	protein LSM12 homolog A	0.0625051997945999	0.605759058823632	0.893547770777888	NA	aag04711	XP_001868919.1 con	XP_001652936.2protein LSM12 homolog A
AAEL00216	cell cycle control protein 50A	0.0625098777983868	0.5725301868338	0.880923047085115	NA	NA	KXJ78912.1 hypothet	XP_001654806.1cell cycle control protein 50A
AAEL01451	protein sprouty	0.0625210530595265	0.737596926377805	0.927599209713406	NA	aag04013	NA	XP_021698821.1protein sprouty
AAEL02230	NA	0.0626414498418693	0.607339231306834	0.893547770777888	NA	NA	NA	XP_019550035.1 voltage-dependent anion-selective channel-like
AAEL02498	NA	0.0627030572895131	0.831495787834748	0.958742264774772	NA	NA	NA	XP_021693809.1uncharacterized protein LOC110674174
AAEL00986	uncharacterized LOC5572538	0.0628159807041728	0.750999844533076	0.932554085844433	NA	NA	NA	XP_001654057.2uncharacterized protein LOC5572538
AAEL00863	guanine nucleotide-binding protein subunit alpha homolog	0.0629121074671321	0.840838727224741	0.963198678766584	NA	NA	ETN59294.1 GTP-bin	XP_001653369.1guanine nucleotide-binding protein subunit alpha homolog
AAEL00149	uncharacterized LOC5570921	0.0629615891410927	0.584899913549737	0.885175928718656	NA	NA	KXJ80192.1 hypothet	XP_001653400.2uncharacterized protein LOC5570921
AAEL02505	NA	0.062975564136347	0.559126687222246	0.877581080322399	NA	NA	NA	XP_021700832.1nuclear receptor-binding protein isoform X1
AAEL01037	charged multivesicular body protein 6	0.0629791624081116	0.679254092268581	0.914481546552978	NA	aag04144	NA	XP_001654507.1charged multivesicular body protein 6
AAEL01978	NA	0.0630342575324456	0.65816164548555	0.909516556517693	NA	aag04144	NA	XP_021703476.1centaurin-gamma-1A isoform X1
AAEL01157	cysteine and histidine-rich protein 1 homolog	0.0630735303446001	0.600535790486423	0.892093596129407	NA	NA	XP_019544130.1 PRE	XP_001661753.2cysteine and histidine-rich protein 1 homolog
AAEL00751	ATP-dependent RNA helicase SUV3 homolog, mitochondrial	0.0631908756157589	0.745924014602235	0.931365937538534	NA	NA	XP_001845729.1 ATF	XP_001658414.2ATP-dependent RNA helicase SUV3 homolog, mitochondrial
AAEL01533	FIT family protein CG10671	0.0632676186376516	0.759064272636391	0.935439571457873	NA	NA	NA	XP_001657345.1FIT family protein CG10671
AAEL02772	NA	0.063281522930879	0.794344624972245	0.945793949302124	NA	aag03420 ; aag	NA	XP_021705412.1DNA polymerase delta catalytic subunit
AAEL00497	uncharacterized LOC5565767	0.0634399958359078	0.823651473052225	0.954684576776241	NA	NA	KXJ76543.1 hypothet	XP_001650116.1uncharacterized protein LOC5565767
AAEL01702	protein henna	0.0634600664807721	0.577570717138617	0.881961491587822	00360 ; 00400 ; 00790	aag01100 ; aag	NA	XP_011493674.1protein henna isoform X2
AAEL00601	fibronectin type-III domain-containing protein 3a	0.0634665400572636	0.720425578846296	0.921583569204186	NA	NA	XP_019540856.1 PRE	XP_021704046.1fibronectin type-III domain-containing protein 3a isoform X2
AAEL01952	NA	0.063532717597176	0.58123147755401	0.883594430630159	NA	aag03040	NA	XP_021695618.1splicing factor 3B subunit 3 isoform X1
AAEL00189	tubulin polyglutamylase TTLL4	0.063676974541844	0.593882808067263	0.888690215018581	NA	NA	XP_019559089.1 PRE	XP_021702687.1tubulin polyglutamylase TTLL4 isoform X1
AAEL02750	NA	0.0637003537537831	0.5761842371552	0.881764069566298	NA	NA	NA	XP_021707726.1clustered mitochondria protein homolog
AAEL00894	probable rRNA-processing protein EBP2 homolog	0.0638307230453457	0.63769820842854	0.903618557739363	NA	NA	ETN63463.1 rRNA pr	XP_021696269.1probable rRNA-processing protein EBP2 homolog
AAEL00068	uncharacterized LOC5565916	0.0639677666242514	0.608077374032131	0.893547770777888	NA	NA	KXJ73216.1 hypothet	XP_001650272.2uncharacterized protein LOC5565916
AAEL00966	uncharacterized LOC5572233	0.0640110543530794	0.66381792875429	0.911573284643631	NA	NA	NA	XP_001653919.1uncharacterized protein LOC5572233
AAEL01386	WD repeat-containing protein 44	0.0640231495500912	0.622969303120137	0.898271200863489	NA	NA	NA	XP_001664046.2WD repeat-containing protein 44 isoform X2
AAEL00278	nucleolar and coiled-body phosphoprotein 1	0.0640479615247874	0.710850113169228	0.918935795219545	NA	NA	KXJ74750.1 hypothet	XP_021709845.1nucleolar and coiled-body phosphoprotein 1
AAEL01995	NA	0.0640610977383338	0.6384455622955	0.90377215326912	NA	NA	NA	XP_021703482.1disintegrin and metalloproteinase domain-containing protein 10 isoform X1
AAEL00977	uncharacterized LOC5572384	0.0640757511926178	0.790378902323796	0.945793949302124	NA	NA	NA	XP_001654041.2uncharacterized protein LOC5572384
AAEL00240	proteasome subunit beta type-2	0.0640962431940854	0.576112200365939	0.881764069566298	NA	aag03050	KXJ82419.1 hypothet	XP_001655145.1proteasome subunit beta type-2
AAEL00930	protein O-GlcNAcase	0.0641071753882891	0.696599570999293	0.916122517126697	NA	NA	NA	XP_021712744.1protein O-GlcNAcase
AAEL01209	casein kinase II subunit alpha	0.0642270128746716	0.632997056746313	0.902506916959539	NA	aag03008 ; aag	ETN66867.1 casein ki	XP_001655863.1casein kinase II subunit alpha
AAEL01042	beclin-1-like protein	0.0642396132532604	0.794784309184707	0.945793949302124	NA	aag04140 ; aag	NA	XP_001654535.1beclin-1-like protein
AAEL00286	uncharacterized LOC5576364	0.064244975311579	0.68493543626164	0.914629465876247	NA	NA	KXJ72213.1 hypothet	XP_001656102.1uncharacterized protein LOC5576364
AAEL01829	NA	0.0643245237649648	0.643189217476575	0.906097241154232	NA	NA	NA	XP_021693874.1probable E3 ubiquitin-protein ligase MGRN1
AAEL01065	NA	0.0643709196798728	0.569078307801542	0.880635379772131	NA	NA	XP_019556605.1 PRE	XP_021698195.1protein lethal(2)essential for life isoform X2
AAEL01325	protein quiver	0.0644026026046692	0.864310574172553	0.967038506393142	NA	NA	NA	XP_001656556.3protein quiver isoform X1
AAEL00422	dolichyl-diphosphooligosaccharide--protein glycosyltransferase subunit S	0.064407064071473	0.56317597631862	0.879621058497374	NA	aag01100 ; aag	KXJ74280.1 hypothet	XP_001648748.1dolichyl-diphosphooligosaccharide--protein glycosyltransferase subunit STT3A
AAEL00583	proteasome subunit alpha type-7-1	0.064460111223447	0.644515090755977	0.906554905014882	NA	aag03050	ETN62449.1 proteas	XP_001651498.1proteasome subunit alpha type-7-1
AAEL00640	probable elongator complex protein 3	0.0645502286272961	0.593947291408971	0.888690215018581	NA	NA	ETN59877.1 Elongat	XP_001657798.1probable elongator complex protein 3
AAEL01966	NA	0.0646094716185093	0.8225028847553	0.954452060294489	NA	NA	NA	XP_021701314.1histone acetyltransferase KAT7 isoform X1
AAEL01226	probable cytosolic Fe-S cluster assembly factor AAEL012261	0.0646275641656005	0.655787627350799	0.908408512250798	NA	NA	B0WU52.2 RecName	XP_001655974.1probable cytosolic Fe-S cluster assembly factor AAEL012261
AAEL00453	glyoxylate reductase/hydroxypyruvate reductase	0.0646984959958241	0.623922035786718	0.898781922601764	NA	NA	KXJ78988.1 hypothet	XP_021701381.1glyoxylate reductase/hydroxypyruvate reductase isoform X1
AAEL01108	DNA-directed RNA polymerases I, II, and III subunit RPABC1	0.0647347360296474	0.652221013884771	0.907272485895905	NA	aag03020	ETN62245.1 DNA-dir	XP_001661404.1DNA-directed RNA polymerases I, II, and III subunit RPABC1
AAEL01947	NA	0.0647732484250247	0.66075863681699	0.909914495882133	NA	NA	NA	XP_021704060.1rap1 GTPase-activating protein 1 isoform X8
AAEL00291	uncharacterized LOC5576475	0.0648339505436128	0.772547755360923	0.941213351626005	901	NA	KXJ76422.1 hypothet	XP_001662668.2uncharacterized protein LOC5576475
AAEL02092	NA	0.0648460295092546	0.83120566749515	0.958742264774772	NA	aag03420 ; aag	NA	XP_021696504.1replication factor C subunit 2
AAEL01745	major latex allergen Hev b 5	0.064847578304138	0.620443099912677	0.897385728224103	NA	NA	NA	XP_021703520.1major latex allergen Hev b 5
AAEL00735	cullin-5	0.0648559314286919	0.733285198600043	0.925884845335185	NA	aag04120	KXJ71822.1 hypothet	XP_019564291.1 cullin-5
AAEL01448	leptin receptor gene-related protein	0.0648848640215371	0.571391852525322	0.880923047085115	NA	NA	NA	XP_021701636.1leptin receptor gene-related protein
AAEL01106	plexin domain-containing protein 2	0.0649232809315759	0.543144834436713	0.869900911343623	NA	NA	NA	XP_001661338.2plexin domain-containing protein 2 isoform X4
AAEL00618	uncharacterized LOC5567587	0.065051116457374	0.628120826781238	0.901045385683245	NA	NA	KXJ80251.1 hypothet	XP_001651813.2uncharacterized protein LOC5567587
AAEL01009	phosphoglycolate phosphatase 1B, chloroplastic	0.0650713450399424	0.778071984701484	0.942719639638287	NA	aag00981	NA	XP_001654245.2phosphoglycolate phosphatase 1B, chloroplastic
AAEL01395	prohibitin-2	0.0650984025337284	0.557050671335184	0.876835490527944	NA	NA	NA	XP_001662385.1prohibitin-2 isoform X4

AAEL0068(	uncharacterized aarF domain-containing protein kinase 1	0.065119099870486	0.549038944993627	0.873169431463929	NA	NA	KXJ79848.1 hypothet	XP_001652210.1uncharacterized aarF domain-containing protein kinase 1
AAEL0206:	NA	0.0652338128705794	0.618749898304556	0.897318336565571	04151 ; 05165 ; 04714 ;	aag04150 ; aag	NA	XP_021703833.1target of rapamycin
AAEL0198!	NA	0.0652775900374911	0.802031427008144	0.946312756934087	NA	NA	NA	XP_021703237.1zinc finger protein 184-like
AAEL0002:	gamma-glutamyl hydrolase	0.0653865628515989	0.619381708743251	0.897318336565571	790	aag00790	NA	XP_001654803.2gamma-glutamyl hydrolase isoform X2
AAEL0016!	tetracycline resistance protein, class D	0.0654118464260855	0.647811619638366	0.906554905014882	NA	NA	KXJ83555.1 hypothet	XP_001659954.2uncharacterized protein LOC5571822
AAEL0155:	polyserase-2	0.0654245947640328	0.559121266253898	0.877581080322399	NA	NA	NA	XP_019562634.1 serine protease 53
AAEL0041:	uncharacterized LOC5564242	0.0655821546525189	0.635617231265642	0.902538767814767	NA	NA	NA	XP_021698325.1uncharacterized protein LOC5564242
AAEL0104(	DNA-directed RNA polymerase I subunit RPA12	0.0656394942494602	0.672910371973037	0.912968243848131	NA	aag03020	NA	XP_001660857.1DNA-directed RNA polymerase I subunit RPA12
AAEL0122:	presequence protease, mitochondrial	0.0657405123213908	0.637088287495773	0.902893334596658	NA	NA	KXJ84515.1 hypothet	XP_001662373.2presequence protease, mitochondrial
AAEL0200!	NA	0.0657643525891548	0.601967625216178	0.892353896540881	NA	NA	NA	XP_021713168.1anoctamin-5 isoform X4
AAEL0134(	ecotropic viral integration site 5 ortholog	0.0658050518639848	0.683279481375336	0.914629465876247	NA	NA	NA	XP_001656716.1ecotropic viral integration site 5 ortholog isoform X2
AAEL0078:	pre-mRNA-splicing factor cwf16	0.0658355641208487	0.771977953059329	0.941147080452193	NA	NA	ETN57752.1 hypothe	XP_001652958.1pre-mRNA-splicing factor cwf16
AAEL0125(	serine/threonine-protein kinase 11-interacting protein	0.0658423555632882	0.687942888113328	0.916010156153545	NA	NA	KXJ80196.1 hypothet	XP_021700217.1serine/threonine-protein kinase 11-interacting protein isoform X2
AAEL0156(	multidrug resistance-associated protein 9	0.0658689455840097	0.714767250528967	0.920089841424905	NA	NA	NA	XP_021711796.1multidrug resistance-associated protein 9
AAEL0050(	serine/threonine-protein phosphatase 5	0.0661033569901157	0.749143481225393	0.932554085844433	04660 ; 04658	NA	XP_001850926.1 seri	XP_001650298.2serine/threonine-protein phosphatase 5
AAEL0141:	ADP-ribosylation factor-like protein 1	0.0661326294565413	0.645730633949178	0.906554905014882	NA	NA	NA	XP_001648164.1ADP-ribosylation factor-like protein 1
AAEL0081:	putative tRNA (cytidine(32)/guanosine(34)-2'-O)-methyltransferase	0.0663693400960163	0.706520973778546	0.917888747806039	NA	NA	KXJ83075.1 hypothet	XP_001658956.1putative tRNA (cytidine(32)/guanosine(34)-2'-O)-methyltransferase
AAEL0232!	NA	0.0663966247581251	0.793303506073097	0.945793949302124	NA	NA	NA	XP_001658699.2serine/arginine repetitive matrix protein 1
AAEL0281(	NA	0.0664384883753235	0.731591512091601	0.925260275963946	NA	NA	NA	XP_021710261.1protein couch potato isoform X4
AAEL0056(	insulin receptor	0.0664602520671683	0.78663023887878	0.944549897171207	NA	NA	KFB49143.1 AGAP00	XP_001651210.2insulin receptor
AAEL0195!	NA	0.06656339770905	0.710209068823478	0.918917864357936	NA	NA	NA	XP_021706445.1 rho GTPase-activating protein 26
AAEL0125(	ubiquitin-protein ligase E3A	0.066586185828152	0.607632538760669	0.893547770777888	NA	aag04120	KXJ73884.1 hypothet	XP_021710239.1ubiquitin-protein ligase E3A
AAEL0075:	protein D2	0.0666992119922528	0.601287272866049	0.892138904371716	NA	NA	KFB38652.1 phospha	XP_001658443.1protein D2
AAEL0125:	uncharacterized LOC5576523	0.0667838136612414	0.628832152451402	0.901045385683245	NA	NA	NA	XP_001662716.2uncharacterized protein LOC5576523
AAEL0036:	sodium-dependent nutrient amino acid transporter 1	0.0668821663521644	0.624453210927141	0.898970345267764	NA	NA	XP_019558042.1 PRE	XP_021705241.1sodium-dependent nutrient amino acid transporter 1 isoform X2
AAEL0245:	NA	0.0668833759271329	0.641673166845175	0.905347513233564	NA	aag03010	NA	XP_019538784.1 60S ribosomal protein L19
AAEL0042:	metallophosphoesterase 1	0.0669139649026786	0.842704231952414	0.963198678766584	NA	NA	XP_019555827.1 PRE	XP_001648851.1metallophosphoesterase 1 isoform X1
AAEL0039:	Y+L amino acid transporter 2	0.0669870460194941	0.663216846540864	0.911397525386277	NA	NA	KFB46283.1 cationic	XP_021693215.1Y+L amino acid transporter 2
AAEL0200:	NA	0.0669978780856076	0.572924168634319	0.880923047085115	NA	NA	NA	XP_021699194.1protein Smaug isoform X2
AAEL0120:	protein krasavietz	0.0670522489888532	0.569896075477401	0.880635379772131	NA	NA	KXJ73292.1 hypothet	XP_001655819.1protein krasavietz
AAEL0019:	protein msta	0.0670647524524591	0.651740384330157	0.907172139858737	NA	NA	KXJ78702.1 hypothet	XP_021706626.1protein msta
AAEL0045(	26S proteasome regulatory subunit 10B	0.0670967019001943	0.566428517591964	0.880302387766415	NA	aag03050	ETN64193.1 26S prot	XP_001649399.126S proteasome regulatory subunit 10B
AAEL0050(	ras-related protein Rab-1A	0.0671912055583499	0.539851139457285	0.869709828277711	NA	aag04140	ETN59196.1 Rab-pro	XP_001650303.1ras-related protein Rab-1A
AAEL0255:	NA	0.0671972324859838	0.787602059919567	0.944774185961398	NA	aag03008	NA	XP_021709805.1elongation factor-like GTPase 1
AAEL0155:	NA	0.0672129960117401	0.692890994876057	0.916122517126697	NA	NA	NA	XP_019528575.1 polyserase-2-like
AAEL0008:	phosphatidylinositol phosphatase PTPRQ	0.067269746737159	0.628823133664547	0.901045385683245	NA	NA	KXJ69868.1 hypothet	XP_021710456.1phosphatidylinositol phosphatase PTPRQ
AAEL0038(	28S ribosomal protein S5, mitochondrial	0.0675226272165175	0.556957835662205	0.876835490527944	NA	aag03010	KXJ74583.1 hypothet	XP_001664173.228S ribosomal protein S5, mitochondrial
AAEL0202(	NA	0.0675245415737633	0.563408663201861	0.879621058497374	NA	aag04144	NA	XP_021697863.1vacuolar protein sorting-associated protein 26B-like
AAEL0262!	NA	0.0675251912725939	0.577918605712566	0.882161363828145	NA	NA	NA	XP_021713390.1ectopic P granules protein 5 homolog isoform X1
AAEL0009!	poly(A) RNA polymerase, mitochondrial	0.0675732327734315	0.706891960550621	0.91797784850989	NA	NA	XP_019548151.1 PRE	XP_001657730.2poly(A) RNA polymerase, mitochondrial
AAEL0008:	GDP-fucose protein O-fucosyltransferase 1	0.0675860939107506	0.785664108859085	0.944372632355572	514	aag00514	KXJ69367.1 hypothet	XP_001651293.1GDP-fucose protein O-fucosyltransferase 1
AAEL0261(	NA	0.0675943885609028	0.708430948341041	0.918037119649945	230	NA	NA	XP_021698103.1adenosine deaminase 2
AAEL0098:	signal recognition particle receptor subunit beta	0.0677082136115633	0.623931636772448	0.898781922601764	NA	aag03060	NA	XP_001660441.2signal recognition particle receptor subunit beta
AAEL0107:	DNA-(apurinic or apyrimidinic site) lyase	0.0677189447403037	0.73467290331735	0.926618579210021	NA	aag03410	KXJ77335.1 hypothet	XP_001654881.1recombination repair protein 1 isoform X1
AAEL0280:	NA	0.0677302036107522	0.868526105460178	0.96863134437578	NA	NA	NA	NA
AAEL0076:	toll 5A	0.0678477864360781	0.550798109702675	0.87417112892101	NA	NA	XP_019530873.1 PRE	XP_021703075.1protein toll
AAEL0112!	NA	0.067865971352745	0.678604747617724	0.914481546552978	NA	NA	KXJ78440.1 hypothet	XP_001661576.2uncharacterized protein LOC5574656
AAEL0028:	dipeptidyl peptidase 3	0.0678785622200068	0.609239347909825	0.893547770777888	NA	NA	XP_001846798.1 dip	XP_001656105.2dipeptidyl peptidase 3
AAEL0054!	CTP synthase	0.0679735824500451	0.534218595140247	0.866700945213732	240	aag01100 ; aag	KXJ80340.1 hypothet	XP_001650904.1CTP synthase
AAEL0084:	27 kDa hemolymph protein	0.068044327008883	0.558298296593741	0.877581080322399	NA	NA	KXJ70685.1 hypothet	XP_001659257.127 kDa hemolymph protein
AAEL0088(	uncharacterized LOC5571163	0.0680819443028906	0.843424103971769	0.963198678766584	NA	NA	XP_001859663.1 dec	XP_001859663.1deoxyribonuclease I
AAEL0199:	NA	0.068095257253311	0.678954741642296	0.914481546552978	NA	NA	NA	XP_021695174.1uncharacterized protein LOC5569552 isoform X3
AAEL0120:	transmembrane 9 superfamily member 3	0.0681147562213779	0.557266461273637	0.877025137102789	NA	NA	XP_019541670.1 PRE	XP_001662153.1transmembrane 9 superfamily member 3
AAEL0099!	trans-1,2-dihydrobenzene-1,2-diol dehydrogenase	0.0681512705698356	0.6663822325976	0.911964500946328	NA	aag01100 ; aag	NA	XP_001660494.2trans-1,2-dihydrobenzene-1,2-diol dehydrogenase
AAEL0035!	DNA-directed RNA polymerases I, II, and III subunit RPABC2	0.068276135313185	0.624764595744971	0.898996689809879	NA	aag03020	ETN61755.1 DNA-dir	XP_001663881.1DNA-directed RNA polymerases I, II, and III subunit RPABC2
AAEL0144!	uncharacterized LOC5564473	0.068400718708531	0.711471701360577	0.918935795219545	NA	NA	NA	XP_001648812.2uncharacterized protein LOC5564473
AAEL0021:	phosphatidylinositol transfer protein CSR1	0.0684020402171363	0.613749243676842	0.896036893592622	NA	NA	KXJ78906.1 hypothet	XP_001654815.1phosphatidylinositol transfer protein CSR1
AAEL0011:	S-adenosylmethionine decarboxylase proenzyme	0.0684755362412708	0.541411207661149	0.869735874782513	00270 ; 00330	aag01100 ; aag	XP_019538204.1 PRE	XP_001658186.1S-adenosylmethionine decarboxylase proenzyme isoform X2
AAEL0139:	uncharacterized LOC5578946	0.0684900426845873	0.826960322388992	0.956432333168643	NA	NA	NA	XP_021711082.1uncharacterized protein LOC5578946
AAEL0072!	fasciculation and elongation protein zeta-2	0.0685446506369953	0.615508511172118	0.897164966965308	NA	NA	ETN58498.1 hypothe	XP_021705484.1fasciculation and elongation protein zeta-2
AAEL0140(	ranBP-type and C3HC4-type zinc finger-containing protein 1	0.06872293122785	0.836688048537121	0.961679168453483	NA	NA	NA	XP_021702951.1ranBP-type and C3HC4-type zinc finger-containing protein 1 isoform X1
AAEL0200:	NA	0.0687377123586285	0.771989687016545	0.941147080452193	NA	NA	NA	XP_021712458.1carboxypeptidase D-like
AAEL0233:	NA	0.0688443843858055	0.648952917404417	0.906581557985861	NA	NA	NA	XP_021704646.1protein fem-1 homolog CG6966
AAEL0123(	uncharacterized LOC5564953	0.068854630633831	0.868282532992261	0.96863134437578	NA	NA	NA	XP_021697669.1uncharacterized protein LOC5564953 isoform X1
AAEL0055:	carbonic anhydrase 2	0.0688619738661886	0.585745312012725	0.885239904594145	NA	aag01100 ; aag	XP_001861856.1 carl	XP_001651049.1carbonic anhydrase 2
AAEL0076(	phosphatidylinositide phosphatase SAC1	0.0688805357668556	0.605706375080372	0.893547770777888	NA	aag01100 ; aag	CRL04071.1 CLUMA_	XP_001658556.1phosphatidylinositide phosphatase SAC1
AAEL0097:	NA	0.0691689584766171	0.618969770237238	0.897318336565571	NA	NA	NA	XP_001653995.1uncharacterized protein LOC5572320
AAEL0134(	probable ATP-dependent RNA helicase DDX10	0.0692253852619812	0.696092671890206	0.916122517126697	NA	NA	NA	XP_001663578.2probable ATP-dependent RNA helicase DDX10
AAEL0021:	putative mediator of RNA polymerase II transcription subunit 26	0.0692958421040118	0.804878172008077	0.94691992227708	NA	NA	KXJ71628.1 hypothet	XP_001654818.1putative mediator of RNA polymerase II transcription subunit 26
AAEL0013(	AT-rich interactive domain-containing protein 2	0.0693506788894864	0.646468813925314	0.906554905014882	NA	NA	NA	XP_021699477.1AT-rich interactive domain-containing protein 2 isoform X1
AAEL0013:	cytochrome P450 9b2	0.0694105706106142	0.673600448119652	0.912974567314009	NA	NA	KXJ82769.1 hypothet	XP_001653042.2cytochrome P450 9b2
AAEL0140:	small integral membrane protein 12-A	0.0694744829140852	0.697241633653356	0.916312126946326	NA	NA	NA	XP_001664246.1small integral membrane protein 12-A
AAEL0126(	uncharacterized LOC5568438	0.0695192904441803	0.715074581213198	0.920167290773856	NA	NA	KFB36617.1 AGAP00	KFB36617.1AGAP007671-like protein
AAEL0047:	UBX domain-containing protein 7	0.0695735929653245	0.742126067894912	0.929911685600432	NA	NA	KFB37650.1 hypothe	XP_021702533.1UBX domain-containing protein 7
AAEL0246!	NA	0.0696088415953609	0.635923137979732	0.902538767814767	NA	aag04013 ; aag	NA	XP_021701325.1protein son of sevenless isoform X1
AAEL0084!	ubiquitin-like protein 5	0.0697291926591605	0.679854794101709	0.914629465876247	NA	NA	CRK92457.1 CLUMA_	XP_001659249.1ubiquitin-like protein 5
AAEL0045:	prion-like-(Q/N-rich) domain-bearing protein 25	0.0697630893072358	0.763462360059302	0.937968042358571	NA	NA	KXJ71982.1 hypothet	XP_001649380.2prion-like-(Q/N-rich) domain-bearing protein 25 isoform X1
AAEL0109(	coiled-coil domain-containing protein 58	0.0699078656645466	0.706289686206997	0.917845823832587	NA	NA	ETN64893.1 hypothe	XP_001661137.1coiled-coil domain-containing protein 58
AAEL0130:	zinc finger protein 425	0.0699125770311184	0.8295249172877	0.958094785081062	NA	NA	NA	XP_001656265.2zinc finger protein 425
AAEL0082!	heterogeneous nuclear ribonucleoprotein 27C	0.0699665886325285	0.522348100877175	0.865018698932415	NA	aag03015	ETN64244.1 heterog	XP_001653144.1heterogeneous nuclear ribonucleoprotein 27C
AAEL0111!	required for meiotic nuclear division protein 1 homolog	0.0701143413582389	0.683640658205948	0.914629465876247	NA	NA	KXJ79091.1 hypothet	XP_001661451.2required for meiotic nuclear division protein 1 homolog
AAEL0081:	tRNA (adenine(58)-N(1))-methyltransferase catalytic subunit TRMT61A	0.0701314926631582	0.722595212239199	0.921934742632737	NA	NA	KXJ80807.1 hypothet	XP_001658963.1tRNA (adenine(58)-N(1))-methyltransferase catalytic subunit TRMT61A

AAEL01450	peptide-N(4)-(N-acetyl-beta-glucosaminyl)asparagine amidase	0.0701431715510086	0.710596056507613	0.918935795219545	NA	aag04141	NA	XP_001648880.2peptide-N(4)-(N-acetyl-beta-glucosaminyl)asparagine amidase
AAEL01287	exosome complex component RRP4	0.0701810947716553	0.753299486417479	0.933081420650645	NA	aag03018	NA	XP_001656299.2exosome complex component RRP4
AAEL00442	prefoldin subunit 3	0.0703059798793253	0.572954187872681	0.880923047085115	NA	NA	ABY58034.1 prefoldi	XP_001649116.1prefoldin subunit 3
AAEL00842	kanadaptin	0.0703525309641602	0.662599370263644	0.910715370450493	NA	NA	KXJ73281.1 hypothet	XP_019561066.1 kanadaptin-like
AAEL01017	zinc finger protein 436	0.0703595506791256	0.825428136746863	0.95542008985467	NA	NA	NA	XP_001654284.2zinc finger protein 436
AAEL01742	nucleolar protein 56	0.0704656410269238	0.514918872580331	0.861448202130317	NA	aag03008	NA	XP_011493522.1nucleolar protein 56
AAEL01427	vesicle-associated membrane protein 7	0.0704692535399438	0.58669972325143	0.885639613312618	NA	aag04130	NA	XP_021697066.1vesicle-associated membrane protein 7
AAEL01738	spidroin-2	0.0704817660501863	0.523549213212172	0.865215478807089	NA	NA	NA	XP_011493439.1spidroin-2 isoform X1
AAEL00530	dnaJ homolog subfamily B member 6	0.0705434340727072	0.552581290230913	0.874531710113044	NA	NA	XP_019525270.1 PRE	XP_021707244.1dnaJ homolog subfamily B member 6 isoform X2
AAEL00142	E3 SUMO-protein ligase NSE2	0.0705769247882165	0.833876190417449	0.959935536312959	NA	NA	KXJ77410.1 hypothet	XP_001659204.1E3 SUMO-protein ligase NSE2
AAEL00125	transmembrane protein 161B	0.0706623245802499	0.589213870783149	0.887098501136541	NA	NA	KXJ75260.1 hypothet	XP_001652869.2transmembrane protein 161B
AAEL00193	CTL-like protein 1	0.0706724154477924	0.638536368782665	0.90377215326912	NA	NA	KFB36013.1 AGAP00	XP_001654328.1CTL-like protein 1
AAEL01008	GPI ethanolamine phosphate transferase 2	0.070676795433621	0.764192633357935	0.93832620035364	NA	aag00563	NA	XP_021699397.1GPI ethanolamine phosphate transferase 2 isoform X3
AAEL01241	26S proteasome non-ATPase regulatory subunit 11	0.070766653878658	0.559813186426047	0.877581080322399	NA	aag03050	ETN60697.1 26S prot	XP_011493668.126S proteasome non-ATPase regulatory subunit 11
AAEL00543	protein mesh	0.0708690814895978	0.563958649338435	0.879838896781538	NA	NA	KXJ79338.1 hypothet	XP_001650885.2protein mesh isoform X2
AAEL00716	arrestin domain-containing protein 17	0.0708735094033587	0.820944386482112	0.954106679666959	NA	NA	ETN65977.1 up-regul	XP_001652563.1arrestin domain-containing protein 4 isoform X1
AAEL00807	plasminogen activator inhibitor 1 RNA-binding protein	0.0709518650615829	0.540142541687364	0.869709828277711	NA	NA	NA	XP_001658869.1plasminogen activator inhibitor 1 RNA-binding protein
AAEL01941	NA	0.071040817092734	0.752022113575721	0.932629041660887	NA	NA	NA	XP_021695492.1zinc finger MIZ domain-containing protein 2
AAEL01239	multidrug resistance-associated protein 4	0.0710417912838132	0.700291325572908	0.916629842461068	NA	NA	KXJ62420.1 hypothet	XP_001662529.1multidrug resistance-associated protein 4
AAEL00024	uncharacterized protein C20orf24 homolog	0.0710742988572136	0.516382121598656	0.861448202130317	NA	NA	ETN64878.1 hypothe	XP_307560.4Anopheles gambiae str. PEST AGAP012695
AAEL01024	longitudinals lacking protein-like	0.0710907856916671	0.504905840689647	0.858744381072989	NA	NA	NA	XP_001654392.1longitudinals lacking protein-like
AAEL01165	importin subunit alpha-3	0.0710932134705434	0.51462187212742	0.861448202130317	NA	NA	ETN59709.1 importir	XP_001661825.1importin subunit alpha-3
AAEL00700	protein-lysine N-methyltransferase EEF2KMT	0.0711193453050977	0.647648158934623	0.906554905014882	NA	NA	KXJ76315.1 hypothet	XP_001652497.1protein-lysine N-methyltransferase EEF2KMT
AAEL01742	zinc finger protein 189	0.0711323422288622	0.746633014864337	0.931365937538534	NA	NA	NA	XP_021705254.1zinc finger protein 189
AAEL01029	methylcrotonoyl-CoA carboxylase subunit alpha, mitochondrial	0.0711427294056246	0.627330349215828	0.900716784753324	NA	aag01100 ; aag	NA	XP_001654405.1methylcrotonoyl-CoA carboxylase subunit alpha, mitochondrial
AAEL00857	polycomb protein suz12	0.0711939899855742	0.646305489015265	0.906554905014882	NA	NA	XP_019563048.1 PRE	XP_021707583.1polycomb protein suz12 isoform X2
AAEL02681	NA	0.0712096286304866	0.683451869904734	0.914629465876247	NA	NA	NA	XP_021704732.1uncharacterized protein LOC110677749
AAEL02069	NA	0.0712734790883108	0.539967092213361	0.869709828277711	NA	NA	NA	XP_021697596.1zinc transporter 1
AAEL02822	NA	0.0713945308372282	0.857912121314068	0.964980728557884	NA	NA	NA	XP_021703167.1coiled-coil domain-containing protein 85C-A
AAEL00867	mitochondrial fission 1 protein	0.0714833451599369	0.570951911016041	0.880923047085115	NA	aag04137	ETN67096.1 mitocho	XP_001659404.1mitochondrial fission 1 protein
AAEL00113	cation-dependent mannose-6-phosphate receptor	0.0715435853162535	0.612915601633558	0.895501122588404	NA	NA	ETN63509.1 hypothe	XP_001652380.1cation-dependent mannose-6-phosphate receptor
AAEL00591	S-adenosylmethionine mitochondrial carrier protein homolog	0.0715612720371863	0.689271250981567	0.916122517126697	NA	NA	XP_001850176.1 mit	XP_001651589.1S-adenosylmethionine mitochondrial carrier protein homolog
AAEL00746	mitotic checkpoint protein BUB3	0.0716032819287794	0.749261981002039	0.932554085844433	NA	NA	ETN62426.1 mitotic c	XP_001658374.1mitotic checkpoint protein BUB3
AAEL00056	26S proteasome non-ATPase regulatory subunit 4	0.0716857739046384	0.509901022117967	0.86030605161891	NA	aag03050	XP_001846286.1 26S	XP_001648653.126S proteasome non-ATPase regulatory subunit 4
AAEL00732	lysM and putative peptidoglycan-binding domain-containing protein 3	0.0717065604691355	0.825287800764045	0.955377826472606	NA	NA	KXJ79903.1 hypothet	XP_001658286.1lysM and putative peptidoglycan-binding domain-containing protein 3
AAEL00274	aspartate--tRNA ligase, cytoplasmic	0.0718061707151239	0.528724405320019	0.865973876319045	NA	aag00970	XP_001865617.1 asp	XP_021697293.1aspartate--tRNA ligase, cytoplasmic
AAEL00614	chaoptin	0.071824372162512	0.528304713065318	0.865973876319045	NA	NA	XP_019545136.1 PRE	XP_019555761.1 toll-like receptor 6 isoform X1
AAEL01386	phosphatidylinositol-binding clathrin assembly protein LAP	0.071831317421309	0.525625155179918	0.865680248818465	NA	NA	NA	XP_021708314.1phosphatidylinositol-binding clathrin assembly protein LAP isoform X9
AAEL00038	phosphatidylinositol 4-phosphate 3-kinase C2 domain-containing subunit	0.0719398420361368	0.564217488579423	0.879838896781538	NA	aag01100 ; aag	KXJ84505.1 hypothet	XP_001656061.1phosphatidylinositol 4-phosphate 3-kinase C2 domain-containing subunit beta
AAEL01077	thioredoxin-2	0.0720212393317323	0.541911335838865	0.869900911343623	NA	NA	CRK90569.1 CLUMA_	XP_019549299.1 thioredoxin-2-like
AAEL00879	DNA-directed RNA polymerase III subunit RPC4	0.0720665167755878	0.721140606584716	0.921583569204186	NA	aag03020	KXJ83359.1 hypothet	XP_001659518.1DNA-directed RNA polymerase III subunit RPC4
AAEL01173	transcription elongation regulator 1	0.0721488803941652	0.712096013734196	0.919162227294442	NA	aag03040	KXJ68605.1 hypothet	XP_021710551.1transcription elongation regulator 1
AAEL00865	solute carrier family 35 member B1 homolog	0.0721812102571805	0.559731242400872	0.877581080322399	NA	NA	KXJ76289.1 hypothet	XP_001659396.1solute carrier family 35 member B1 homolog
AAEL00229	uncharacterized LOC5574195	0.0721882664597633	0.734666562805697	0.926618579210021	NA	NA	KXJ75866.1 hypothet	XP_001661252.1uncharacterized protein LOC5574195
AAEL02456	NA	0.0722328316696487	0.772288807269562	0.941147080452193	NA	NA	NA	XP_021696762.1protein yellow isoform X1
AAEL02520	NA	0.0722894875499633	0.532837260593628	0.866700945213732	NA	NA	NA	XP_021701664.1apoptosis inhibitor 5 homolog
AAEL02179	NA	0.072388687591778	0.612177678649052	0.895020495539971	NA	aag04144	NA	XP_021701499.1brefeldin A-inhibited guanine nucleotide-exchange protein 1
AAEL00310	ubiquitin-conjugating enzyme E2-17 kDa	0.072470739026963	0.534329065897329	0.866700945213732	NA	aag04141 ; aag	NP_731941.1 effete,	NP_731941.1effete, isoform A
AAEL00672	innexin inx4	0.0725615692352357	0.636999539930932	0.902893334596658	NA	NA	KFB36085.1 AGAP00	XP_001652174.1innexin inx4
AAEL00045	ras GTPase-activating protein 1	0.0726067321470603	0.564123468813764	0.879838896781538	NA	aag04013	KFB40576.1 hypothe	XP_001656445.1ras GTPase-activating protein 1
AAEL00849	membrane magnesium transporter 1	0.0727154407738745	0.526035845274121	0.865680248818465	NA	NA	ETN65038.1 hypothe	XP_001659284.1membrane magnesium transporter 1
AAEL01081	V-type proton ATPase subunit e 2	0.0727279780703528	0.508568594841748	0.860083070787677	NA	aag01100 ; aag	ETN64432.1 Vacuola	XP_011493566.1V-type proton ATPase subunit e 2
AAEL02409	NA	0.0727666940141973	0.553137402268447	0.874531710113044	NA	NA	NA	XP_019547992.1 constitutive coactivator of PPAR-gamma-like protein 1
AAEL00876	uncharacterized LOC5571040	0.0728557631512864	0.656915609999754	0.909239282344833	NA	NA	NA	ABF18046.1proline rich salivary secreted peptide
AAEL00535	KH domain-containing, RNA-binding, signal transduction-associated prote	0.0729092983587862	0.705916708426351	0.917749125552377	NA	NA	KXJ79135.1 hypothet	XP_001650787.1KH domain-containing, RNA-binding, signal transduction-associated protein 2 isoform X1
AAEL02723	NA	0.0733456009784448	0.85702701062028	0.964980728557884	NA	NA	NA	XP_021704724.1speract receptor isoform X2
AAEL00263	probable cytochrome P450 9f2	0.0733764282529569	0.741193691906066	0.929218576692046	NA	NA	KXJ83348.1 hypothet	XP_001661968.1probable cytochrome P450 9f2
AAEL00893	polymerase delta-interacting protein 3	0.0733772656997534	0.659037140592451	0.909580313002131	NA	NA	XP_001862738.1 con	XP_001653630.1polymerase delta-interacting protein 3
AAEL00713	erythroid differentiation-related factor 1	0.0734109570249117	0.653471892828701	0.907272485895905	NA	NA	KXJ73436.1 hypothet	XP_021711092.1erythroid differentiation-related factor 1
AAEL00755	ras-GEF domain-containing family member 1B	0.0734254974729953	0.855170062265213	0.964980728557884	NA	NA	KFB39261.1 AGAP00	XP_021700467.1ras-GEF domain-containing family member 1B
AAEL00406	protein Notchless	0.0734431001640146	0.600916482961534	0.892093596129407	NA	NA	KXJ77490.1 hypothet	XP_021703839.1protein Notchless
AAEL00949	DENN domain-containing protein 5A	0.0734928923635047	0.595838838803611	0.889282316160442	NA	NA	NA	XP_021707932.1DENN domain-containing protein 5A isoform X3
AAEL02591	NA	0.0734995959787376	0.580673228460477	0.883199181536083	NA	NA	NA	XP_021708073.1uncharacterized protein LOC5565730 isoform X1
AAEL01942	NA	0.0735357729870896	0.740354144899616	0.928618984286445	NA	aag04013 ; aag	NA	XP_021694078.1ETS-like protein pointed isoform X1
AAEL00918	arginine kinase	0.0735505484578813	0.584483517085205	0.885175928718656	NA	aag00330	XP_019547605.1 PRE	XP_021697332.1arginine kinase isoform X1
AAEL00652	NA	0.0735591526205254	0.508252059331809	0.859730781244726	NA	NA	XP_001844041.1 tub	XP_011493034.2tubulin-specific chaperone C
AAEL01179	vacuolar protein-sorting-associated protein 25	0.0735870454573716	0.758972917882942	0.935439571457873	NA	aag04144	KXJ75526.1 hypothet	XP_001655741.1vacuolar protein-sorting-associated protein 25
AAEL00541	28S ribosomal protein S11, mitochondrial	0.073705339114551	0.740224749930821	0.928618984286445	NA	aag03010	XP_001849182.1 mit	XP_001650870.128S ribosomal protein S11, mitochondrial
AAEL01209	aminopeptidase N	0.0737315247922339	0.491634939865793	0.851821637477881	480	NA	KXJ78008.1 hypothet	XP_021694450.1aminopeptidase N isoform X1
AAEL00139	DIS3-like exonuclease 2	0.0738130287357843	0.69021398877002	0.916122517126697	NA	NA	KXJ81335.1 hypothet	XP_001659195.2DIS3-like exonuclease 2
AAEL00788	lipoyltransferase 1, mitochondrial	0.0738206567713188	0.522564545464605	0.865018698932415	00785 ; 00785	aag01100 ; aag	KXJ79275.1 hypothet	XP_001652965.2lipoyltransferase 1, mitochondrial
AAEL00702	transcription initiation factor IIA subunit 1	0.0738752060945212	0.51326230213293	0.861304697755515	NA	aag03022	KXJ80930.1 hypothet	XP_021708806.1transcription initiation factor IIA subunit 1
AAEL00235	protein zer-1 homolog	0.0739018662139823	0.510555903184803	0.860452247709668	NA	NA	XP_019556441.1 PRE	XP_021698015.1protein zer-1 homolog isoform X2
AAEL00076	serine-arginine protein 55	0.0739195050442013	0.835007514733692	0.960827704211508	NA	NA	XP_019534390.1 PRE	XP_001651001.2serine-arginine protein 55
AAEL01327	importin subunit beta	0.0739199478708969	0.63177911642311	0.902506916959539	NA	aag03013	NA	XP_001663435.1importin subunit beta isoform X1
AAEL01056	forkhead box protein K2	0.0739503740611061	0.516333231279774	0.861448202130317	NA	NA	NA	XP_021699106.1forkhead box protein K2 isoform X1
AAEL01305	ubiquitin carboxyl-terminal hydrolase	0.0739605088800934	0.559667939094394	0.877581080322399	NA	NA	NA	XP_001663268.1ubiquitin carboxyl-terminal hydrolase
AAEL00849	zinc finger protein 624	0.0741397871533509	0.853670011091677	0.964653958545747	NA	NA	ETN65035.1 zinc fing	XP_001659281.2zinc finger protein 624
AAEL02582	NA	0.0741405286363553	0.673929644491554	0.913194019822012	NA	aag03040	NA	XP_021698009.1calcium homeostasis endoplasmic reticulum protein isoform X2
AAEL00673	protein Gemin2	0.0741587232718979	0.800121161664573	0.946215055875588	NA	aag03013	KXJ75889.1 hypothet	XP_001652190.1protein Gemin2
AAEL01109	lysine-specific demethylase lid	0.0741690350874367	0.53104781061225	0.866691320214768	NA	NA	XP_001850672.1 jum	XP_001661408.2lysine-specific demethylase lid
AAEL00412	solute carrier family 25 member 44	0.0741831162525357	0.607533456417892	0.893547770777888	NA	NA	ETN68126.1 Mitocho	XP_011493224.1solute carrier family 25 member 44

AAEL00408	protein dj-1beta	0.0742361271837634	0.640555592101838	0.904464102888003	NA	NA	XP_002020428.1 GL1	XP_001648396.2protein dj-1beta
AAEL00971	RING finger and transmembrane domain-containing protein 2	0.0743166981599975	0.519849030682027	0.864080221844267	NA	NA	NA	XP_001654025.2RING finger and transmembrane domain-containing protein 2
AAEL00664	dynein light chain Tctex-type	0.0744343863685477	0.511599712236782	0.860862214418965	NA	NA	KXJ71932.1 hypothet	XP_001652154.1dynein light chain Tctex-type isoform X2
AAEL00636	phosphoribosyl pyrophosphate synthase-associated protein 2	0.0744841577290052	0.512353646776891	0.861177338330732	00230 ; 00030	NA	XP_001862760.1 ribo	XP_021699375.1phosphoribosyl pyrophosphate synthase-associated protein 2 isoform X1
AAEL00500	roundabout homolog 2	0.0746615761486306	0.79084612724817	0.945793949302124	NA	NA	ETN66355.1 roundab	XP_021710986.1roundabout homolog 2
AAEL01469	uncharacterized LOC5565015	0.0746768999412531	0.54053012238784	0.869735874782513	NA	NA	NA	XP_021701984.1uncharacterized protein LOC5565015
AAEL01752	serine/arginine repetitive matrix protein 1	0.0747109180020093	0.598445501132117	0.890651059585596	NA	aag03013 ; aag	NA	XP_021698970.1serine/arginine repetitive matrix protein 1 isoform X1
AAEL00109	NADP-dependent malic enzyme	0.0748117557623891	0.597046975719512	0.889911899315825	NA	aag01100 ; aag	KXJ72035.1 hypothet	XP_021693932.1NADP-dependent malic enzyme
AAEL01814	NA	0.0748413607888405	0.640145596178077	0.904463241434479	760	aag01100 ; aag	NA	XP_021704068.1nicotinamide/nicotinic acid mononucleotide adenylyltransferase 1 isoform X1
AAEL00084	N-terminal Xaa-Pro-Lys N-methyltransferase 1	0.0748709869160688	0.705211263647356	0.917749125552377	NA	NA	KFB45941.1 AGAP01	XP_001651286.2N-terminal Xaao-Lys N-methyltransferase 1
AAEL00579	peptidyl-prolyl cis-trans isomerase G	0.0748802723791321	0.523285775398123	0.865215478807089	NA	NA	ETN63466.1 hypothe	XP_021712916.1peptidyl-prolyl cis-trans isomerase G
AAEL02568	NA	0.0749086054338341	0.818762948098763	0.952859067712763	NA	NA	NA	XP_019553426.1 inner centromere protein B-like
AAEL00715	ribosomal RNA processing protein 1 homolog	0.074928301583945	0.534210359717186	0.866700945213732	NA	NA	XP_019534960.1 PRE	XP_001652567.2ribosomal RNA processing protein 1 homolog isoform X2
AAEL00577	general odorant-binding protein 99a	0.0750208990652375	0.653020881193616	0.907272485895905	NA	NA	ETN65059.1 odorant	XP_001651445.1general odorant-binding protein 99a
AAEL00516	peroxisomal carnitine O-octanoyltransferase	0.0750365433645283	0.704257514569076	0.917749125552377	NA	aag04146	KXJ72757.1 hypothet	XP_001650455.1peroxisomal carnitine O-octanoyltransferase
AAEL00570	protein Star	0.0752476283625835	0.688914010332661	0.916010156153545	NA	NA	XP_019551048.1 PRE	XP_021701727.1protein Star isoform X1
AAEL01945	NA	0.0752539513681883	0.779783344725382	0.943566949556407	NA	NA	NA	XP_021698373.1regulator of G-protein signaling 17 isoform X1
AAEL00586	CDK5 and ABL1 enzyme substrate 2	0.0752563872931424	0.633533786874087	0.902506916959539	NA	NA	KFB53918.1 hypothe	XP_001651506.2CDK5 and ABL1 enzyme substrate 2
AAEL00780	NA	0.0753602373681023	0.691503501570824	0.916122517126697	NA	aag04711	NA	XP_001652918.2ataxin-2 homolog isoform X1
AAEL01745	thiamine transporter 1	0.0754536013030063	0.671608522761118	0.912812708172959	NA	NA	NA	XP_011493518.1thiamine transporter 1
AAEL00612	transmembrane protease serine 9	0.0754866149432621	0.732276708357343	0.925440879854609	NA	NA	XP_001865845.1 seri	XP_001657536.2transmembrane protease serine 9
AAEL01227	peptidyl-prolyl cis-trans isomerase FKBP8	0.0755006284134293	0.515539337804728	0.861448202130317	NA	NA	KXJ84517.1 hypothet	XP_001662371.2peptidyl-prolyl cis-trans isomerase FKBP8 isoform X2
AAEL01868	NA	0.0756004339893726	0.718954768823896	0.921583569204186	NA	aag01100 ; aag	NA	YP_009389271.1cytochrome b (mitochondrion)
AAEL00574	probable ATP-dependent RNA helicase pitchoune	0.0756562250933714	0.546434131054728	0.871000671048954	NA	NA	KXJ77783.1 hypothet	XP_001651396.1probable ATP-dependent RNA helicase pitchoune
AAEL01079	F-box/LRR-repeat protein 7	0.0757139481028953	0.515702573017094	0.861448202130317	NA	NA	KXJ69328.1 hypothet	XP_001654905.1F-box/LRR-repeat protein 7 isoform X5
AAEL02515	NA	0.0758483842577084	0.654242941864138	0.907635089858856	NA	aag04141 ; aag	NA	XP_021712817.1translocation protein SEC63 homolog
AAEL01830	NA	0.0760523647226085	0.783911396160567	0.944372632355572	NA	NA	NA	XP_021708266.1acetylcholine receptor subunit beta-like 2
AAEL00376	methionine aminopeptidase 1	0.0760626819064341	0.546019608121888	0.870931414970375	NA	NA	ETN63473.1 methion	XP_001657229.1methionine aminopeptidase 1
AAEL00486	protein farnesyltransferase subunit beta	0.0761757267687586	0.694567915096046	0.916122517126697	900	aag00900	KFB42753.1 AGAP00	XP_001649963.1protein farnesyltransferase subunit beta
AAEL00749	2,3-bisphosphoglycerate-dependent phosphoglycerate mutase	0.0763181254015447	0.58143056314008	0.883594430630159	00680 ; 00010 ; 00260	aag01100 ; aag	XP_001863396.1 pho	XP_001863396.1phosphoglycerate mutase
AAEL02493	NA	0.0764295289280115	0.837977752310193	0.962348963212321	NA	aag04330	NA	XP_021693929.1protein serrate
AAEL00375	sorting nexin-32	0.0765889693914659	0.531059559687188	0.866691320214768	NA	aag04144	KXJ81601.1 hypothet	XP_001657203.2sorting nexin-32 isoform X2
AAEL01520	transmembrane protein 147	0.0765990091701065	0.573916002481695	0.881197098233729	NA	NA	NA	XP_021700389.1transmembrane protein 147
AAEL02079	NA	0.0767782007631328	0.780674835723552	0.94381989277494	NA	NA	NA	NA
AAEL02724	NA	0.0768225456248239	0.507865487218366	0.859709016442863	NA	NA	NA	XP_021703192.1coatomer subunit gamma
AAEL01055	vacuolar protein sorting-associated protein 16 homolog	0.0768329888325373	0.718819961139151	0.921583569204186	NA	NA	KXJ77917.1 hypothet	XP_001660915.1vacuolar protein sorting-associated protein 16 homolog
AAEL00663	ribosomal RNA-processing protein 8	0.0768659675415977	0.703588480155397	0.917303584765266	NA	NA	KXJ71242.1 hypothet	XP_001657919.2ribosomal RNA-processing protein 8
AAEL01297	uncharacterized LOC5577078	0.0769044342658565	0.526298960842623	0.865680248818465	NA	NA	NA	XP_021697918.1uncharacterized protein LOC5577078 isoform X1
AAEL00140	leucine-rich repeat transmembrane neuronal protein 3	0.0769205607897437	0.583781648430736	0.885175928718656	NA	NA	KXJ74864.1 hypothet	XP_001659164.1leucine-rich repeat transmembrane neuronal protein 3
AAEL02254	NA	0.0769973596679711	0.671611642504608	0.912812708172959	NA	aag03013	NA	XP_021707045.1CCA tRNA nucleotidyltransferase 1, mitochondrial
AAEL02649	NA	0.0771236569148844	0.552900987534024	0.874531710113044	NA	aag04141	NA	XP_021698549.1NSFL1 cofactor p47
AAEL00328	ADP-ribosylation factor-like protein 2	0.0771428301858837	0.848162702804536	0.963435772275933	NA	NA	KXJ71587.1 hypothet	XP_021693821.1ADP-ribosylation factor-like protein 2
AAEL00814	long-chain fatty acid transport protein 4	0.0773154857564137	0.523714333250175	0.865215478807089	NA	NA	XP_001867718.1 AM	XP_021704718.1long-chain fatty acid transport protein 4
AAEL00778	alkyldihydroxyacetonephosphate synthase	0.0773774100474091	0.796472112740248	0.945890571556554	565	NA	KXJ71501.1 hypothet	XP_001658665.2alkyldihydroxyacetonephosphate synthase
AAEL00414	stress-induced-phosphoprotein 1	0.0774063784682298	0.480181447699101	0.845454613425911	NA	NA	KXJ84399.1 hypothet	XP_001648490.1stress-induced-phosphoprotein 1
AAEL00721	TBC1 domain family member 5	0.0774898429115298	0.800021429627021	0.946215055875588	NA	NA	KXJ72713.1 hypothet	XP_001658225.2TBC1 domain family member 5
AAEL00421	serine/threonine-protein kinase 32A	0.0774968447172038	0.837977752304401	0.962348963212321	NA	NA	XP_017143224.1 PRE	XP_021693444.1serine/threonine-protein kinase 32A
AAEL01105	26S proteasome non-ATPase regulatory subunit 5	0.0775276200964449	0.684633000310953	0.914629465876247	NA	NA	KXJ73008.1 hypothet	XP_001661342.226S proteasome non-ATPase regulatory subunit 5
AAEL00946	replication factor C subunit 5	0.0775760169713032	0.803826149960729	0.94649029363412	NA	aag03420 ; aag	NA	XP_001660080.1replication factor C subunit 5
AAEL00521	uncharacterized serine-rich protein C215.13	0.0776133602426162	0.580216876116613	0.882796907080865	NA	NA	KXJ75015.1 hypothet	XP_021708264.1uncharacterized serine-rich protein C215.13
AAEL00603	OTU domain-containing protein 7B	0.0776730287807248	0.625782641812364	0.899577186361051	NA	NA	NA	XP_021708188.1OTU domain-containing protein 7B isoform X3
AAEL00256	prion-like-(Q/N-rich) domain-bearing protein 25	0.0777049703426337	0.607531238071146	0.893547770777888	NA	NA	XP_019549640.1 PRE	XP_001655599.2prion-like-(Q/N-rich) domain-bearing protein 25 isoform X3
AAEL01324	UDP-N-acetylglucosamine transferase subunit ALG13 homolog	0.0777481672250794	0.746742739296496	0.931365937538534	00550 ; 00513 ; 00510	aag01100 ; aag	NA	XP_001663412.1UDP-N-acetylglucosamine transferase subunit ALG13 homolog
AAEL02327	NA	0.07776518349111	0.677034410782003	0.914438668430353	NA	NA	NA	XP_021701486.1G-protein-signaling modulator 2
AAEL00303	opsin-3	0.0777746179454707	0.490292025412189	0.850081180289834	NA	NA	KXJ71420.1 hypothet	XP_019555625.1 opsin-3-like
AAEL00581	NAD-dependent protein deacetylase Sirt2	0.0777901640632092	0.513575811098995	0.861309357197051	NA	aag01100 ; aag	KFB52704.1 AGAP00	XP_001651467.2NAD-dependent protein deacetylase Sirt2
AAEL00915	tRNA (guanine-N(7)-)-methyltransferase	0.0778315321516004	0.622842610628466	0.898271200863489	NA	NA	KXJ73224.1 hypothet	XP_001653710.2tRNA (guanine-N(7)-)-methyltransferase
AAEL00491	post-GPI attachment to proteins factor 3	0.0778440168590609	0.691800660929417	0.916122517126697	NA	NA	KXJ70175.1 hypothet	XP_001650006.1post-GPI attachment to proteins factor 3
AAEL00411	probable ATP-dependent RNA helicase YTHDC2	0.0778955642132339	0.517465821515279	0.861881144752444	NA	NA	KXJ83370.1 hypothet	XP_021711078.1probable ATP-dependent RNA helicase YTHDC2
AAEL00617	histone acetyltransferase type B catalytic subunit	0.0779557122955613	0.636305972554488	0.902624772241117	NA	NA	KXJ78107.1 hypothet	XP_001651817.1histone acetyltransferase type B catalytic subunit
AAEL01270	kelch-like protein 13	0.0780448388296886	0.819658439043222	0.953298401930704	NA	NA	KXJ72357.1 hypothet	XP_021698349.1kelch-like protein 13
AAEL00603	dehydrodolichyl diphosphate synthase complex subunit DHDDS	0.0780537504894825	0.685197348584513	0.914629465876247	NA	aag00900	KXJ76936.1 hypothet	XP_001657391.1dehydrodolichyl diphosphate synthase complex subunit DHDDS
AAEL01370	NA	0.0781698088718787	0.524188184484043	0.865216084954648	NA	NA	NA	XP_001844767.1meiotic recombination protein spo11
AAEL01831	NA	0.0781862190536717	0.646254915992475	0.906554905014882	NA	aag04141 ; aag	NA	XP_001648798.2E3 ubiquitin-protein ligase parkin
AAEL01304	sphingolipid delta(4)-desaturase DES1	0.078197347010927	0.563347217921017	0.879621058497374	600	aag01100 ; aag	NA	XP_001663266.1sphingolipid delta(4)-desaturase DES1
AAEL02490	NA	0.0782822382256027	0.858742897454646	0.965328928656042	NA	NA	NA	XP_021703449.1zinc finger protein 90-like
AAEL00841	uncharacterized LOC5570082	0.0783494516840936	0.682919771147641	0.914629465876247	NA	NA	KXJ81514.1 hypothet	XP_021698488.1uncharacterized protein LOC5570082 isoform X2
AAEL00473	dedicator of cytokinesis protein 9	0.0783902746986351	0.696446181415813	0.916122517126697	NA	NA	XP_019549602.1 PRE	XP_021702530.1dedicator of cytokinesis protein 9 isoform X3
AAEL00145	protein deltex	0.0784602262190504	0.786908552039826	0.944549897171207	NA	aag04330	KXJ79977.1 hypothet	XP_001659292.1protein deltex
AAEL01443	uncharacterized LOC5564430	0.0784654246075483	0.75461769290518	0.934085554225648	901	NA	NA	XP_001648768.2uncharacterized protein LOC5564430
AAEL00130	uncharacterized LOC5569813	0.0785295040575853	0.636805398464867	0.902893334596658	NA	NA	KXJ77968.1 hypothet	XP_001653030.2uncharacterized protein LOC5569813
AAEL01454	vacuolar protein sorting-associated protein VTA1 homolog	0.0786263260201457	0.602001478532421	0.892353896540881	NA	aag04144	NA	XP_001648971.2vacuolar protein sorting-associated protein VTA1 homolog
AAEL00780	cytochrome P450 4d1	0.0786777193485315	0.569460581835395	0.880635379772131	NA	NA	KXJ72909.1 hypothet	XP_001652928.1cytochrome P450 4d1
AAEL00807	diuretic hormone class 2	0.0788273367382852	0.652961064824024	0.907272485895905	NA	NA	CRK90576.1 CLUMA_	XP_001658868.1diuretic hormone class 2 isoform X2
AAEL00778	histone H3-like centromeric protein cid	0.0788320855526515	0.769240465022628	0.940921275972115	NA	NA	KXJ75024.1 hypothet	XP_021708849.1histone H3-like centromeric protein cid
AAEL00353	nuclear inhibitor of protein phosphatase 1	0.078945044230935	0.622826244750882	0.898271200863489	NA	NA	XP_001848337.1 nuc	XP_001656914.2nuclear inhibitor of protein phosphatase 1
AAEL01990	NA	0.0791004245609537	0.61985931678336	0.897318336565571	NA	NA	NA	XP_021704622.1fat-like cadherin-related tumor suppressor homolog isoform X6
AAEL01300	cyclin-dependent kinase 9	0.0791473951336666	0.512382734988128	0.861177338330732	NA	NA	NA	XP_021701929.1cyclin-dependent kinase 9
AAEL00268	probable 4-coumarate--CoA ligase 1	0.079160669849831	0.746555003633653	0.931365937538534	NA	NA	KXJ83978.1 hypothet	XP_001662060.1probable 4-coumarate--CoA ligase 1
AAEL00818	60S ribosomal protein L6	0.0791912086432034	0.600005671913133	0.891963197567794	NA	aag03010	ETN58373.1 60S ribo	XP_021704947.160S ribosomal protein L6
AAEL00157	OCIA domain-containing protein 1	0.0792437598545299	0.668883126873595	0.912812708172959	NA	NA	KXJ79231.1 hypothet	XP_001653565.1OCIA domain-containing protein 1
AAEL00443	DNA replication complex GINS protein PSF3	0.0792600598103251	0.759220441999218	0.935439571457873	NA	NA	KFB51246.1 AGAP01	XP_001649124.1DNA replication complex GINS protein PSF3

AAEL00831	protein disulfide-isomerase A5	0.0794981308424599	0.663669941758218	0.911573284643631	NA	NA	KXJ81065.1 hypothet	XP_001659136.1protein disulfide-isomerase A5
AAEL01438	serine protease easter	0.0795616352438266	0.559476706290028	0.877581080322399	NA	NA		XP_021698113.1serine protease easter
AAEL00018	gamma-glutamylcyclotransferase	0.0795827931063495	0.515445990560042	0.861448202130317	480	aag01100 ; aag	KFB35967.1 AGAP01	XP_019527493.1 gamma-glutamylcyclotransferase-like
AAEL0209	NA	0.0796178453553701	0.783607403454461	0.944186896240402	00740 ; 00730	aag01100 ; aag	NA	XP_021712595.1low molecular weight phosphotyrosine protein phosphatase 1-like
AAEL01353	hepatoma-derived growth factor	0.079735336654316	0.575455153325027	0.881674712104046	NA	NA	NA	XP_001663721.2hepatoma-derived growth factor
AAEL02775	NA	0.0797451273503648	0.597179864848395	0.889918885236426	NA	NA	NA	XP_021709399.1uncharacterized protein LOC5573737 isoform X2
AAEL0099	complex III assembly factor LYRM7	0.0797977680011441	0.714914406236478	0.920089841424905	NA	NA	NA	XP_001660516.1complex III assembly factor LYRM7
AAEL00303	PHD finger protein 14	0.0798070665436184	0.648647280901119	0.906554905014882	NA	NA	KXJ72448.1 hypothet	XP_001662997.2PHD finger protein 14
AAEL00517	uncharacterized LOC5566116	0.0798561070840686	0.509140825432892	0.860083070787677	NA	NA	NA	XP_021695414.1uncharacterized protein LOC5566116 isoform X5
AAEL0088	polyphosphoinositide phosphatase	0.0798574814076308	0.72336109995744	0.921934742632737	NA	aag01100 ; aag	KXJ83357.1 hypothet	XP_001659514.1polyphosphoinositide phosphatase
AAEL00399	probable enoyl-CoA hydratase, mitochondrial	0.0798688632453191	0.511197405592773	0.86059980356105	NA	aag01100 ; aag	ETN64671.1 enoyl-Co	XP_001648219.1probable enoyl-CoA hydratase, mitochondrial
AAEL01972	NA	0.0798845861613425	0.535628252516944	0.867370387366011	NA	NA	NA	XP_019552950.1 transmembrane protein 170A-like
AAEL0012	uncharacterized LOC5569606	0.0800263119581478	0.554434397726728	0.875297464116348	NA	NA	XP_001843976.1 con	XP_001652887.1uncharacterized protein LOC5569606
AAEL00574	elastase-1	0.0800289726130737	0.561942803750871	0.878972918598592	NA	NA	KXJ77691.1 hypothet	XP_019932152.1 elastase-1-like
AAEL00745	DDB1- and CUL4-associated factor 11	0.080038956151864	0.484241828986385	0.847655581875201	NA	NA	CRK89608.1 CLUMA_	XP_001652768.1DDB1- and CUL4-associated factor 11
AAEL00377	ER membrane protein complex subunit 8/9 homolog	0.0800603330351325	0.699588486569958	0.916629842461068	NA	NA	XP_019525790.1 PRE	XP_001664192.2ER membrane protein complex subunit 8/9 homolog
AAEL00123	NA	0.0800651376792464	0.584014790459784	0.885175928718656	NA	NA	KXJ80043.1 hypothet	XP_019538390.1 putative GPI-anchored protein pfl2
AAEL00414	N-acetylglucosaminyl-phosphatidylinositol de-N-acetylase	0.0800811712455049	0.670206657095426	0.912812708172959	563	aag01100 ; aag	XP_001861826.1 N-a	XP_021706244.1N-acetylglucosaminyl-phosphatidylinositol de-N-acetylase
AAEL0203	NA	0.0802119618229955	0.505656957267321	0.858744381072989	NA	NA	NA	XP_021700258.1protein SDA1 homolog
AAEL01965	NA	0.0803681096909856	0.73537682398791	0.926702094411052	NA	NA	NA	XP_021705207.1A disintegrin and metalloproteinase with thrombospondin motifs 4 isoform X1
AAEL01347	zinc finger protein 808	0.0803896537167454	0.817664464520584	0.952514760254484	NA	NA	NA	XP_001663657.2zinc finger protein 808
AAEL0040	tRNA modification GTPase GTPBP3, mitochondrial	0.0803914967662067	0.803613170332177	0.94649029363412	NA	NA	KXJ70807.1 hypothet	XP_021707487.1tRNA modification GTPase GTPBP3, mitochondrial
AAEL0028	phenylalanine--tRNA ligase alpha subunit	0.0804394343280785	0.502816640205852	0.858744381072989	970	aag00970	KXJ74423.1 hypothet	XP_001656084.1phenylalanine--tRNA ligase alpha subunit
AAEL01317	uncharacterized LOC5577431	0.0804468139295881	0.794775686150166	0.945793949302124	NA	NA	NA	XP_021702659.1uncharacterized protein LOC5577431
AAEL00785	serine/arginine repetitive matrix protein 2	0.080464074707559	0.71263386978094	0.919252089784076	NA	NA	KXJ75541.1 hypothet	XP_001658697.2serine/arginine repetitive matrix protein 2
AAEL00749	uncharacterized LOC5569224	0.0806537428649869	0.644144231931835	0.906332602788493	NA	NA	KXJ82202.1 hypothet	XP_001652779.2uncharacterized protein LOC5569224
AAEL0047	PIH1 domain-containing protein 1	0.0806930385960102	0.647913349611829	0.906554905014882	NA	NA	KFB46636.1 hypothe	XP_001649737.1PIH1 domain-containing protein 1
AAEL0154	lysozyme c-1	0.0810963007231389	0.470435820222615	0.841282642598396	NA	NA	NA	XP_021696193.1lysozyme c-1
AAEL00725	protein commissureless 2 homolog	0.0811373289413479	0.726747969651047	0.923437560995434	NA	NA	KXJ69499.1 hypothet	XP_001652624.2protein commissureless 2 homolog
AAEL00672	uncharacterized LOC5568305	0.0811493838165433	0.683320681824897	0.914629465876247	NA	NA	KXJ80155.1 hypothet	XP_021697087.1uncharacterized protein LOC5568305
AAEL00341	signal peptidase complex subunit 2	0.0811548707807077	0.497789260203312	0.856197527549697	NA	aag03060	ETN68146.1 signal pe	XP_001656797.1signal peptidase complex subunit 2
AAEL0196	NA	0.0811985427540483	0.711157951950199	0.918935795219545	NA	NA	NA	XP_021708571.1zwei Ig domain protein zig-8-like isoform X2
AAEL02681	NA	0.0812627730844793	0.595882934301813	0.889282316160442	NA	aag04150	NA	XP_021698655.1GATOR complex protein DEPDC5 isoform X1
AAEL00827	alpha-tocopherol transfer protein-like	0.0813025100173613	0.728762804224593	0.923566968708516	NA	NA	KFB47433.1 hypothe	XP_001659081.1alpha-tocopherol transfer protein-like
AAEL0175	mitochondrial dicarboxylate carrier	0.0813025883250556	0.467443292789154	0.839233979689189	NA	NA	NA	XP_011493675.1mitochondrial dicarboxylate carrier
AAEL02182	NA	0.0813335288199082	0.606310500695351	0.893547770777888	NA	NA	NA	XP_021703987.1ral GTPase-activating protein subunit beta isoform X5
AAEL00818	E3 ubiquitin-protein ligase NRDP1	0.081336083921836	0.705908991039229	0.917749125552377	NA	aag04144	ETN58397.1 hypothe	XP_001658984.1E3 ubiquitin-protein ligase NRDP1
AAEL0199	NA	0.0816608115024017	0.552315196617209	0.874531710113044	NA	NA	NA	XP_021704796.1E3 ubiquitin-protein ligase RBBP6 isoform X1
AAEL01977	NA	0.0817166280946159	0.659403231669287	0.909580313002131	NA	NA	NA	XP_021700357.1semaphorin-1A isoform X1
AAEL02624	NA	0.0817225991804292	0.817664464543922	0.952514760254484	NA	NA	NA	XP_021703648.1gastrula zinc finger protein XlCGF7.1-like
AAEL0022	bis(5'-nucleosyl)-tetraphosphatase [asymmetrical]	0.0817713783129069	0.544671870140177	0.870453377740519	00240 ; 00230	aag01100 ; aag	XP_001654994.2 bis(	XP_001654994.2bis(5'-nucleosyl)-tetraphosphatase [asymmetrical]
AAEL02102	NA	0.0818662680770528	0.781849414534443	0.943962424451135	NA	NA	NA	XP_021694828.1DNA polymerase subunit gamma-1, mitochondrial
AAEL01818	NA	0.081922909766575	0.659467188238895	0.909580313002131	NA	NA	NA	XP_019563453.1 uncharacterized ATP-dependent helicase C29A10.10c isoform X2
AAEL01083	ADP-ribosylation factor GTPase-activating protein 1	0.0820877205694848	0.469789828316268	0.84072462080247	NA	aag04144	KXJ68162.1 hypothet	XP_001661085.2ADP-ribosylation factor GTPase-activating protein 1 isoform X1
AAEL02136	NA	0.0821005692059651	0.663293765215377	0.911397525386277	NA	NA	NA	XP_021712338.1proline-rich protein 36
AAEL01216	transmembrane protein 18	0.0821704880364738	0.570384838215986	0.880923047085115	NA	NA	ETN59464.1 hypothe	XP_001655911.1transmembrane protein 18
AAEL02554	NA	0.0823187642641522	0.749149087497789	0.932554085844433	NA	aag03013	NA	XP_021694125.1nuclear pore complex protein Nup93
AAEL00981	uncharacterized LOC5572472	0.082333355843751	0.616335657637346	0.897164966965308	NA	NA	NA	XP_307448.4Anopheles gambiae str. PEST AGAP012518
AAEL0109	zinc transporter foi	0.0824124596503721	0.564192152740929	0.879838896781538	NA	NA	KXJ77503.1 hypothet	XP_021697583.1zinc transporter foi
AAEL00264	cytohesin-1	0.0824881653772123	0.541562049301472	0.869735874782513	NA	aag04144	XP_019558984.1 PRE	XP_021700365.1cytohesin-1 isoform X1
AAEL02157	NA	0.0825212186800681	0.689428887043039	0.916122517126697	NA	NA	NA	NA
AAEL01331	calcyclin-binding protein	0.0825442658225607	0.584250481665622	0.885175928718656	NA	aag04310	NA	XP_001663461.1calcyclin-binding protein
AAEL01048	serine--pyruvate aminotransferase, mitochondrial	0.0825586451650717	0.691044031061008	0.916122517126697	440	aag01100 ; aag	NA	XP_001660875.1serine--pyruvate aminotransferase, mitochondrial
AAEL01031	probable ATP-dependent RNA helicase DDX27	0.0826122733315341	0.535299152029093	0.867069670299544	NA	NA	NA	XP_001654429.1probable ATP-dependent RNA helicase DDX27
AAEL0002	uncharacterized LOC5573746	0.0827833099968755	0.824481256971655	0.954996283354524	NA	NA	XP_001866336.1 con	XP_001654774.2uncharacterized protein LOC5573746
AAEL01533	ganglioside-induced differentiation-associated protein 1	0.0828097051803611	0.565406326753219	0.880302387766415	NA	NA	NA	XP_001653860.2ganglioside-induced differentiation-associated protein 1
AAEL0063	Golgi resident protein GCP60	0.0828958389816844	0.616703413459516	0.897164966965308	NA	NA	KXJ82561.1 hypothet	XP_021705078.1Golgi resident protein GCP60
AAEL0140	calcium load-activated calcium channel	0.0829058271800867	0.482485193178877	0.846972290658532	NA	NA	NA	XP_001664309.1calcium load-activated calcium channel
AAEL00155	zinc finger and SCAN domain-containing protein 12	0.0829264610004157	0.771418084852047	0.941147080452193	NA	NA	NA	XP_001653543.1zinc finger and SCAN domain-containing protein 12
AAEL01004	MOB kinase activator-like 1	0.0831345476821125	0.710723473183064	0.918935795219545	NA	aag04391 ; aag	NA	XP_001654177.1MOB kinase activator-like 1
AAEL00718	cullin-3	0.0832132034808494	0.449584905449258	0.829406635915011	NA	aag04120 ; aag	KXJ68248.1 hypothet	XP_001652595.1cullin-3 isoform X2
AAEL0115	ras-like GTP-binding protein RhoL	0.0832329032073016	0.670016830321442	0.912812708172959	NA	aag04144 ; aag	XP_001851173.1 Cdc	XP_001655406.1ras-like GTP-binding protein RhoL isoform X1
AAEL02264	NA	0.0832536385174334	0.726437864598117	0.923177868750456	NA	NA	NA	XP_001650076.2zinc finger protein 454
AAEL02557	NA	0.0833173219699704	0.811336831441101	0.95010454605765	NA	NA	NA	XP_021702541.1uncharacterized protein LOC5572937 isoform X2
AAEL02705	NA	0.0833388492889384	0.453085023638335	0.832617462606238	NA	aag03040	NA	XP_019529771.1 serine-arginine protein 55
AAEL01403	serine/threonine-protein phosphatase 2A 56 kDa regulatory subunit gam	0.083387601933702	0.490353282436257	0.850081180289834	NA	aag03015	NA	XP_001664252.1serine/threonine-protein phosphatase 2A 56 kDa regulatory subunit gamma isoform
AAEL00653	serine/threonine-protein kinase 3	0.0833922245177414	0.568227448053973	0.880422457819946	04151 ; 05165 ; 04714 ;	aag04391 ; aag	XP_001841861.1 seri	XP_021696615.1serine/threonine-protein kinase 3
AAEL01321	zinc finger E-box-binding homeobox 1	0.083486220331068	0.690987992590559	0.916122517126697	NA	NA	NA	XP_001663393.1zinc finger E-box-binding homeobox 1
AAEL00562	60S ribosomal protein L35	0.0835561926069282	0.541576457563986	0.869735874782513	NA	aag03010	KXJ72836.1 hypothet	XP_001651180.160S ribosomal protein L35
AAEL0020	putative neutral sphingomyelinase	0.083664407535992	0.60467408412193	0.893547770777888	NA	aag01100 ; aag	KXJ74809.1 hypothet	XP_001660755.1putative neutral sphingomyelinase
AAEL0269	NA	0.083696379689727	0.552788646226578	0.874531710113044	NA	NA	NA	XP_021694088.1uncharacterized membrane protein DDB_G0293934-like
AAEL00272	histidine triad nucleotide-binding protein 1	0.0837550351337785	0.627082542090786	0.900535116157945	NA	NA	KXJ68748.1 hypothet	XP_001662177.1histidine triad nucleotide-binding protein 1
AAEL02745	NA	0.0838400203091129	0.617334224913441	0.897318336565571	NA	NA	NA	NA
AAEL00563	nuclear pore complex protein Nup50	0.0839591242736311	0.593475082604886	0.888529762455765	NA	aag03013	KXJ72838.1 hypothet	XP_001651179.1nuclear pore complex protein Nup50
AAEL02228	NA	0.0840470766418801	0.458974455340354	0.833631645771888	NA	NA	NA	XP_021693700.1RNA-binding protein cabeza-like isoform X4
AAEL02219	NA	0.0840720740541157	0.576816979463893	0.881961491587822	NA	NA	NA	XP_017891599.1 U8-agatoxin-Ao1a-like
AAEL00522	uncharacterized LOC5566177	0.0841119108914912	0.661163490811828	0.909914495882133	NA	NA	KFB35497.1 AGAP00	DAA64998.1TPA_exp: septate junction protein megatrachea
AAEL0129	vacuolar protein sorting-associated protein 51 homolog	0.0841566165688611	0.75051548059119	0.932554085844433	NA	NA	NA	XP_001663147.2vacuolar protein sorting-associated protein 51 homolog
AAEL02268	NA	0.0841783300063964	0.712756726856221	0.919252089784076	NA	NA	NA	XP_021705270.1 N-acetylneuraminate 9-O-acetyltransferase
AAEL00697	uncharacterized LOC5568623	0.0842038260106196	0.731420027052231	0.925260275963946	NA	NA	KXJ70490.1 hypothet	XP_001652457.1uncharacterized protein LOC5568623
AAEL01954	NA	0.0842046137339248	0.605880873409297	0.893547770777888	NA	NA	NA	XP_021696470.1uncharacterized protein LOC5570478
AAEL00385	protein flightless-1	0.0842407858297351	0.528316731457604	0.865973876319045	NA	NA	KXJ70670.1 hypothet	XP_001657298.2protein flightless-1

AAEL0084	neuroguidin	0.0842476463291958	0.685283292918283	0.914629465876247	NA	NA	KXJ79149.1 hypothet	XP_019543706.1 neuroguidin-like
AAEL0049	inositol monophosphatase 2	0.0842626236540356	0.657980550920435	0.909516556517693	04070 ; 00562 ; 00521	aag01100 ; aag	XP_019537636.1 PRE	XP_001650092.2inositol monophosphatase 2
AAEL0210	NA	0.0843526996890193	0.60075778745005	0.892093596129407	NA	NA	NA	XP_021709612.1LIM domain-binding protein 2 isoform X1
AAEL0046	signal peptidase complex subunit 1	0.0844732339071557	0.541469157972139	0.869735874782513	NA	aag03060	KXJ82450.1 hypothet	XP_001649527.2signal peptidase complex subunit 1
AAEL0267	NA	0.0845422631628718	0.545680101052265	0.870854715553754	NA	NA	NA	XP_021701854.1MOB kinase activator-like 3
AAEL0083	transcription termination factor 3, mitochondrial	0.0845812247690815	0.681045317287785	0.914629465876247	NA	NA	KXJ71060.1 hypothet	XP_001653246.2transcription termination factor 3, mitochondrial
AAEL0209	NA	0.0851191650954692	0.448402464340649	0.829288931094171	NA	aag04120	NA	XP_021705936.1E3 ubiquitin-protein ligase hyd isoform X6
AAEL0076	serine/threonine-protein phosphatase 4 regulatory subunit 1	0.0851764757285297	0.494773731683316	0.853723585027166	NA	NA	XP_019531998.1 PRE	XP_021697580.1serine/threonine-protein phosphatase 4 regulatory subunit 1 isoform X2
AAEL0031	carboxypeptidase N subunit 2	0.0853472678966743	0.70742267468276	0.91797784805989	NA	NA	KXJ73689.1 hypothet	XP_001663226.2carboxypeptidase N subunit 2
AAEL0058	BEACH domain-containing protein lvsF	0.0854502518987032	0.545351608571391	0.870571167410854	NA	NA	KXJ80361.1 hypothet	XP_001651497.2protein FAN isoform X1
AAEL0027	DNA repair protein XRCC1	0.0854737870476298	0.679949898408618	0.914629465876247	NA	aag03410	KXJ74751.1 hypothet	XP_021704208.1DNA repair protein XRCC1
AAEL0073	cleft lip and palate transmembrane protein 1-like protein	0.0858854112994698	0.634078079542551	0.902506916959539	NA	NA	KXJ70172.1 hypothet	XP_001652638.1cleft lip and palate transmembrane protein 1-like protein
AAEL0062	solute carrier family 2, facilitated glucose transporter member 3	0.0859073181374914	0.436936514880385	0.820960220424486	NA	NA	XP_001868999.1 glu	XP_001651862.1solute carrier family 2, facilitated glucose transporter member 3 isoform X1
AAEL0030	zinc finger and BTB domain-containing protein 14	0.0859815201738446	0.614912726200735	0.896845306410123	NA	NA	XP_019539384.1 PRE	XP_001663069.1zinc finger and BTB domain-containing protein 14 isoform X3
AAEL0111	NADH dehydrogenase [ubiquinone] 1 alpha subcomplex subunit 7	0.0860387579506228	0.534694769178261	0.866700945213732	NA	aag01100 ; aag	ETN64396.1 NADH de	XP_001661446.1NADH dehydrogenase [ubiquinone] 1 alpha subcomplex subunit 7
AAEL0207	NA	0.0860596325116724	0.48736997216702	0.848846834445739	NA	NA	NA	NA
AAEL0053	probable pyruvate dehydrogenase E1 component subunit alpha, mitocho	0.0861646240252598	0.456515115359774	0.832617462606238	00010 ; 00620 ; 00020	aag01100 ; aag	KXJ72252.1 hypothet	XP_001650695.2probable pyruvate dehydrogenase E1 component subunit alpha, mitochondrial isoform X2
AAEL0059	LYR motif-containing protein 4	0.0862922072943574	0.523777955325816	0.865215478807089	NA	NA	KXJ71126.1 hypothet	XP_011493308.1LYR motif-containing protein 4
AAEL0001	2-methoxy-6-polyprenyl-1,4-benzoquinol methylase, mitochondrial	0.0863137103839553	0.65348859340557	0.907272485895905	130	aag01100 ; aag	ETN66021.1 ubiquin	XP_001658935.12-methoxy-6-polyprenyl-1,4-benzoquinol methylase, mitochondrial
AAEL0030	nudC domain-containing protein 1	0.0863155573529806	0.718771747035117	0.921583569204186	NA	NA	KXJ82098.1 hypothet	XP_001662978.1nudC domain-containing protein 1
AAEL0104	peroxisomal membrane protein PEX16-like	0.0863593404028877	0.752865157696096	0.932920169824868	NA	NA	NA	XP_021711958.1peroxisomal membrane protein PEX16-like
AAEL0059	D-2-hydroxyglutarate dehydrogenase, mitochondrial	0.0863993987040547	0.620588163584674	0.897385728224103	NA	NA	KXJ71128.1 hypothet	XP_001651707.1D-2-hydroxyglutarate dehydrogenase, mitochondrial isoform X1
AAEL0034	copper-transporting ATPase 1	0.0865832213077287	0.444724957383958	0.826574967752089	NA	NA	NA	XP_021709336.1copper-transporting ATPase 1 isoform X2
AAEL0121	MKI67 FHA domain-interacting nucleolar phosphoprotein	0.0865990602936559	0.493734879578522	0.852730548401194	NA	NA	KXJ69664.1 hypothet	XP_001655849.1MKI67 FHA domain-interacting nucleolar phosphoprotein
AAEL0128	V-type proton ATPase subunit G	0.086695159648471	0.424678626842795	0.814623464645531	NA	aag01100 ; aag	ETN62197.1 vacuolar	XP_001662942.1V-type proton ATPase subunit G
AAEL0079	moesin/ezrin/radixin homolog 1	0.0867162538185447	0.45570894445841	0.832617462606238	NA	NA	XP_019539429.1 PRE	XP_021693568.1moesin/ezrin/radixin homolog 1 isoform X1
AAEL0194	NA	0.086716948158405	0.510439849024325	0.860452247709668	NA	NA	NA	XP_021695142.1uncharacterized protein LOC5578376 isoform X1
AAEL0105	pleiotropic regulator 1	0.08684406427363	0.717372837234616	0.921283483156443	NA	aag03040	KXJ76615.1 hypothet	XP_021697677.1pleiotropic regulator 1
AAEL0053	breast cancer anti-estrogen resistance protein 3	0.0871293920499496	0.6014251341355	0.892140251748536	NA	NA	NA	XP_021702385.1breast cancer anti-estrogen resistance protein 3 isoform X5
AAEL0234	NA	0.0873136897890563	0.497415617746985	0.856052403704179	NA	NA	NA	XP_021697178.1calcium release-activated calcium channel protein 1 isoform X2
AAEL0196	NA	0.0874297463821671	0.487372524979904	0.848846834445739	230	aag01100 ; aag	NA	XP_021695155.1AMP deaminase 2 isoform X3
AAEL0118	28S ribosomal protein S30, mitochondrial	0.0875186949350685	0.458015925797128	0.833283524364043	NA	NA	KXJ78947.1 hypothet	XP_001661930.228S ribosomal protein S30, mitochondrial
AAEL0020	manganese-transporting ATPase 13A1	0.0875796920018302	0.4687203392199	0.840117756428033	NA	NA	KXJ78797.1 hypothet	XP_001654560.1manganese-transporting ATPase 13A1
AAEL0272	NA	0.0878022603741438	0.81491888795177	0.952015623780574	NA	NA	NA	XP_021702364.1uncharacterized protein LOC5567052
AAEL0041	aldose reductase	0.08782589041578	0.5200961559474	0.864134369681863	NA	aag01100 ; aag	KXJ83375.1 hypothet	XP_001648458.1aldose reductase
AAEL0105	gamma-soluble NSF attachment protein	0.0878979384939051	0.579165409804003	0.882283691803025	NA	NA	KXJ76616.1 hypothet	XP_001654683.1gamma-soluble NSF attachment protein
AAEL0041	uncharacterized LOC5564170	0.0879701351225618	0.756366657358857	0.934127170333935	NA	NA	KXJ73142.1 hypothet	XP_001648495.1uncharacterized protein LOC5564170
AAEL0067	UPF0489 protein C5orf22 homolog	0.0880347948228803	0.594870896868204	0.888948786307326	NA	NA	ETN64291.1 Misexpr	XP_001652189.1UPF0489 protein C5orf22 homolog
AAEL0237	NA	0.0880437899145669	0.775729051571477	0.941847042163971	NA	NA	NA	XP_021712138.1E3 ubiquitin-protein ligase Mdm2-like
AAEL0114	clavesin-2	0.0880964131837667	0.648398783583733	0.906554905014882	NA	NA	KXJ75162.1 hypothet	XP_019527191.1 clavesin-2-like
AAEL0195	NA	0.0882231872065822	0.448817093509899	0.829306073695304	NA	NA	NA	XP_021696550.1protein rogdi isoform X1
AAEL0065	asparagine--tRNA ligase, cytoplasmic	0.0882250282123932	0.410715787747868	0.805855841282375	970	aag00970	KXJ76111.1 hypothet	XP_001652096.1asparagine--tRNA ligase, cytoplasmic
AAEL0079	ficolin-1	0.0883061332896317	0.50767552732999	0.859703754598927	NA	NA	KXJ71085.1 hypothet	XP_019558100.1 ficolin-2-like
AAEL0155	prominin-like protein	0.0883599852782225	0.436377014175768	0.820960220424486	NA	NA	NA	XP_021695516.1prominin-like protein isoform X2
AAEL0273	NA	0.0884473441276588	0.638908509267279	0.90408059392242	NA	aag04130	NA	XP_021693079.1vesicle transport v-SNARE 12
AAEL0090	ras guanine nucleotide exchange factor P	0.0885552249571404	0.504201559125245	0.858744381072989	NA	aag04350	XP_019562564.1 PRE	XP_001653659.2ras guanine nucleotide exchange factor P isoform X1
AAEL0078	endoplasmic reticulum lectin 1	0.0886966897701784	0.440404621219855	0.822798752521693	NA	aag04141	XP_019540889.1 PRE	XP_001652998.2endoplasmic reticulum lectin 1 isoform X1
AAEL0142	facilitated trehalose transporter Tret1	0.0887194334360611	0.715491012959697	0.920421919040713	NA	NA	NA	XP_021701653.1facilitated trehalose transporter Tret1 isoform X2
AAEL0046	elongin-C	0.0888091111461505	0.516712374511919	0.861530579862055	NA	aag04120	BAM18297.1 conserv	XP_019550229.1 transcription elongation factor B polypeptide 1
AAEL0154	brain protein I3	0.0888306295854739	0.60412242387789	0.893547770777888	NA	NA	NA	XP_001647695.2brain protein I3
AAEL0258	NA	0.0889221438521659	0.81811991872919	0.952713172886105	NA	NA	NA	NA
AAEL0113	uncharacterized LOC5574692	0.0889783346561197	0.617979224406045	0.897318336565571	NA	NA	KXJ70099.1 hypothet	XP_021713383.1uncharacterized protein LOC5574692
AAEL0053	phosphoribosylformylglycinamidine synthase	0.0893474324654652	0.532094474501599	0.866700945213732	230	aag01100 ; aag	KXJ72591.1 hypothet	XP_001650832.1phosphoribosylformylglycinamidine synthase
AAEL0098	glycogen debranching enzyme	0.0893527705475356	0.477878701598244	0.845011493487133	500	aag01100 ; aag	NA	XP_001660447.1glycogen debranching enzyme isoform X1
AAEL0221	NA	0.0893965498737848	0.795485516000765	0.945890571556554	NA	NA	NA	XP_021711740.139S ribosomal protein L44, mitochondrial-like
AAEL0107	protein rolling stone	0.089412200401649	0.515581014086403	0.861448202130317	NA	NA	KXJ77339.1 hypothet	XP_001654876.1protein rolling stone
AAEL0119	G patch domain-containing protein 4	0.0894969705505404	0.446625507278828	0.82821796189507	NA	NA	KXJ68819.1 hypothet	XP_001655788.1G patch domain-containing protein 4
AAEL0042	transmembrane protein 181	0.0895255518371872	0.76645831035383	0.939178856256477	NA	NA	KXJ79321.1 hypothet	XP_001648740.2transmembrane protein 181
AAEL0011	protein phosphatase 1 regulatory subunit 7	0.0895418183574001	0.547853151406727	0.872355891892144	NA	NA	XP_019538232.1 PRE	XP_001658167.2protein phosphatase 1 regulatory subunit 7 isoform X3
AAEL0149	heterogeneous nuclear ribonucleoprotein K	0.089578796650589	0.410117212022826	0.805356078257463	NA	aag03040	NA	XP_021699822.1heterogeneous nuclear ribonucleoprotein K isoform X1
AAEL0061	BRCA1-associated protein	0.0896539125744581	0.607178136851685	0.893547770777888	NA	NA	KXJ79188.1 hypothet	XP_001657524.1BRCA1-associated protein isoform X1
AAEL0019	chitinase-like protein Idgf4	0.089780810393819	0.423745783558562	0.814221904428041	NA	NA	XP_019551534.1 PRE	XP_001660745.2chitinase-like protein Idgf4
AAEL0085	nucleoporin NUP188 homolog	0.0898209327245907	0.599119524922194	0.890933721450232	NA	aag03013	KXJ71256.1 hypothet	XP_021698606.1nucleoporin NUP188 homolog
AAEL0004	transmembrane emp24 domain-containing protein eca	0.0898880346455343	0.48438879627615	0.847655581875201	NA	NA	ETN67877.1 glycopro	XP_001656404.1transmembrane emp24 domain-containing protein eca
AAEL0052	ornithine aminotransferase, mitochondrial	0.0899692750285146	0.498629862760588	0.856678287334277	330	aag01100 ; aag	KXJ74780.1 hypothet	XP_001650687.1ornithine aminotransferase, mitochondrial isoform X1
AAEL0073	eukaryotic translation elongation factor 1 epsilon-1	0.0901376752341465	0.523702500994644	0.865215478807089	NA	NA	KXJ79756.1 hypothet	XP_001652723.2eukaryotic translation elongation factor 1 epsilon-1
AAEL0112	max-like protein X	0.0902163312363446	0.435125718518808	0.820960220424486	NA	NA	ETN63756.1 bhlhzip	XP_001655177.1max-like protein X
AAEL0064	transformer-2 protein homolog alpha	0.0902666791638325	0.700467965304126	0.916629842461068	NA	NA	NA	XP_021697706.1transformer-2 protein homolog alpha isoform X2
AAEL0101	conserved oligomeric Golgi complex subunit 4	0.0903232567718444	0.678641871620043	0.914481546552978	NA	NA	NA	XP_001654256.1conserved oligomeric Golgi complex subunit 4
AAEL0109	NA	0.0903773025531311	0.758932368594111	0.935439571457873	NA	NA	KXJ78351.1 hypothet	XP_001661200.1kinesin-like protein KIF3A isoform X1
AAEL0273	NA	0.0903936814797195	0.704923336934626	0.917749125552377	NA	NA	NA	NA
AAEL0115	proteasomal ubiquitin receptor ADRM1 homolog	0.0904994877019923	0.464172497039176	0.837274753622609	NA	aag03050	KXJ81714.1 hypothet	XP_021704606.1proteasomal ubiquitin receptor ADRM1 homolog isoform X2
AAEL0194	NA	0.0905131020346839	0.517484603864079	0.861881144752444	NA	NA	NA	XP_021705518.1uncharacterized protein LOC5570798 isoform X1
AAEL0140	polycomb group protein Pc	0.0905437509645273	0.665883204989161	0.911964500946328	NA	NA	NA	XP_001648030.2polycomb group protein Pc
AAEL0045	glyoxylate reductase/hydroxypyruvate reductase	0.0905817894618607	0.550792893276028	0.87417112892101	NA	aag01100 ; aag	KFB39544.1 AGAP00	XP_001649337.1glyoxylate reductase/hydroxypyruvate reductase
AAEL0068	NEDD4-binding protein 2	0.0908136296281859	0.624074387778927	0.898836174387357	NA	NA	KXJ70254.1 hypothet	XP_021710375.1NEDD4-binding protein 2
AAEL0115	nuclear pore membrane glycoprotein 210	0.0909041246460826	0.561817331084169	0.878972918598592	NA	aag03013	KXJ69238.1 hypothet	XP_021697029.1nuclear pore membrane glycoprotein 210
AAEL0072	thioredoxin domain-containing protein 15	0.0910349992590145	0.611403631813213	0.894646314231935	NA	NA	KFB44083.1 hypothe	XP_001652621.1thioredoxin domain-containing protein 15
AAEL0223	NA	0.0910457592765294	0.575986994484683	0.881764069566298	NA	aag01100 ; aag	NA	XP_021712515.1 4-hydroxybenzoate polyprenyltransferase, mitochondrial-like
AAEL0043	probable tRNA pseudouridine synthase 2	0.0910626652121587	0.630552425847954	0.90233929580663	NA	NA	XP_001850528.1 con	XP_001649000.2probable tRNA pseudouridine synthase 2
AAEL0043	casein kinase I	0.0911774337898568	0.448010593834297	0.828995271357974	04151 ; 05165 ; 04714 ;	aag04341	XP_019540443.1 PRE	XP_021709013.1casein kinase I isoform X4
AAEL0037	uncharacterized LOC5578978	0.0912185617534003	0.503013518344268	0.858744381072989	NA	NA	KXJ78149.1 hypothet	XP_021712988.1uncharacterized protein LOC5578978

AAEL02183	NA	0.0912350173096564	0.58855972619924	0.886693942450132	NA	NA	NA	XP_021709370.1telomere-associated protein RIF1 isoform X2
AAEL00715	5'-AMP-activated protein kinase catalytic subunit alpha-2	0.0914451158250233	0.455608344714584	0.832617462606238	04151 ; 05165 ; 04714 ;	aag04150 ; aag	KXJ72273.1 hypothet	XP_001652572.15'-AMP-activated protein kinase catalytic subunit alpha-2
AAEL01128	25S rRNA (cytosine-C(5))-methyltransferase nop2	0.0914459109150485	0.528737396798019	0.865973876319045	NA	NA	KXJ69146.1 hypothet	XP_001661560.225S rRNA (cytosine-C(5))-methyltransferase nop2
AAEL00007	probable dimethyladenosine transferase	0.0915045363324198	0.611771341166464	0.894913329966487	NA	NA	XP_001864755.1 dim	XP_001647892.2probable dimethyladenosine transferase
AAEL01333	transmembrane protein 245	0.0915500221713834	0.664742823511154	0.911808086169482	NA	NA	NA	XP_021711695.1transmembrane protein 245 isoform X6
AAEL00748	RNA pseudouridylate synthase domain-containing protein 1	0.0916206780627482	0.737684309572126	0.927599209713406	NA	NA	XP_019547530.1 PRE	XP_001652796.1RNA pseudouridylate synthase domain-containing protein 1 isoform X2
AAEL01457	E3 ubiquitin-protein ligase MARCH6	0.0916396308534208	0.660990572596174	0.909914495882133	NA	aag04141	NA	XP_021696396.1E3 ubiquitin-protein ligase MARCH6
AAEL01418	immunoglobulin-binding protein 1	0.0917509498845162	0.56891956247377	0.880635379772131	NA	aag04140 ; aag	NA	XP_001648171.2immunoglobulin-binding protein 1
AAEL00205	nitric oxide synthase-interacting protein homolog	0.091810476390979	0.651298439918786	0.907105833832704	NA	NA	XP_001865022.1 nitr	XP_001654595.2nitric oxide synthase-interacting protein homolog
AAEL00509	glycylpeptide N-tetradecanoyltransferase	0.0918194095300628	0.519931774435091	0.864080221844267	NA	NA	KXJ70872.1 hypothet	XP_001650345.1glycylpeptide N-tetradecanoyltransferase
AAEL01971	NA	0.0918456137009244	0.556895140875168	0.876835490527944	NA	NA	NA	XP_021701868.1PQ-loop repeat-containing protein 1 isoform X1
AAEL00390	nuclear pore complex protein Nup107	0.0918580301257318	0.708870423100455	0.918089462824826	NA	aag03013	KXJ68046.1 hypothet	XP_001648004.2nuclear pore complex protein Nup107
AAEL01043	N-sulphoglucosamine sulphohydrolase	0.0918955974963824	0.566867916472408	0.880302387766415	NA	aag01100 ; aag	NA	XP_021709525.1N-sulphoglucosamine sulphohydrolase
AAEL00664	TNF receptor-associated factor 4	0.0919152763660679	0.689995234576779	0.916122517126697	NA	aag04214	ETN66724.1 TNF rece	XP_001652160.1TNF receptor-associated factor 4
AAEL01449	NA	0.0921372592094117	0.628174566588388	0.901045385683245	NA	NA	NA	XP_001648870.2coiled-coil domain-containing protein 130 homolog
AAEL02130	NA	0.0921887838028447	0.391853346625206	0.793285976044306	NA	NA	NA	XP_021702882.1ATP-dependent zinc metalloprotease YME1 homolog
AAEL01184	transmembrane protein 120 homolog	0.0923593358597779	0.413511429612094	0.808061732662297	NA	NA	CRK96466.1 CLUMA_	XP_001661986.1transmembrane protein 120 homolog
AAEL00480	uncharacterized LOC5565498	0.0923820623795561	0.685668479379128	0.914803661828742	NA	NA	KXJ81192.1 hypothet	XP_021705339.1uncharacterized protein LOC5565498
AAEL00737	pyruvate dehydrogenase (acetyl-transferring) kinase, mitochondrial	0.0924355566645047	0.386481582846985	0.791367050591446	NA	NA	XP_019543835.1 PRE	XP_001652717.1pyruvate dehydrogenase (acetyl-transferring) kinase, mitochondrial isoform X1
AAEL01980	NA	0.0925022694090121	0.423005763270334	0.814192545906728	04151 ; 05165 ; 04714 ;	NA	NA	XP_019555392.1 AF4/FMR2 family member 4
AAEL00226	trafficking protein particle complex subunit 11	0.0926022342836506	0.506884509458037	0.859256178927183	NA	NA	XP_019933136.1 PRE	XP_021696132.1trafficking protein particle complex subunit 11 isoform X1
AAEL00298	serine/arginine repetitive matrix protein 1	0.0926782839479073	0.467497650498455	0.839233979689189	NA	NA	KXJ82807.1 hypothet	XP_021711017.1serine/arginine repetitive matrix protein 1
AAEL01101	GRAM domain-containing protein 1B	0.0927099215843883	0.586136553215266	0.885362534912488	NA	NA	KXJ71254.1 hypothet	XP_001661305.2GRAM domain-containing protein 1B
AAEL00277	centrosome-associated zinc finger protein CP190	0.0927326759970828	0.483983933116094	0.847655581875201	NA	NA	XP_019563757.1 PRE	XP_021709796.1centrosome-associated zinc finger protein CP190 isoform X1
AAEL00411	uncharacterized LOC5564115	0.0927715839276927	0.597912779457517	0.890434276835745	NA	NA	XP_001844849.1 con	XP_001648450.1uncharacterized protein LOC5564115
AAEL00127	ADP-ribosylation factor GTPase-activating protein 2	0.0927833395239203	0.425911513794533	0.814623464645531	NA	aag04144	KXJ73131.1 hypothet	XP_021698486.1ADP-ribosylation factor GTPase-activating protein 2 isoform X2
AAEL00399	enoyl-[acyl-carrier-protein] reductase, mitochondrial	0.0928521162864717	0.552616985507306	0.874531710113044	NA	aag01100 ; aag	KFB50132.1 AGAP01	XP_001648220.1enoyl-[acyl-carrier-protein] reductase, mitochondrial
AAEL01989	NA	0.0928548099305964	0.812172206396204	0.950268140513817	NA	NA	NA	XP_021701589.1uncharacterized protein LOC5568235
AAEL02082	NA	0.092902401848102	0.431082469014692	0.819760462879354	NA	NA	NA	XP_021706105.1leukocyte elastase inhibitor
AAEL02139	NA	0.0931602708785935	0.466585952322305	0.838900928502398	NA	aag04140	NA	XP_001659907.2AT-rich interactive domain-containing protein 4B isoform X3
AAEL00265	uncharacterized LOC5575437	0.0931966495745423	0.505949203043095	0.858744381072989	NA	NA	KXJ82244.1 hypothet	XP_021700362.1uncharacterized protein LOC5575437
AAEL01115	protein sex-lethal	0.093249181231787	0.453108290667933	0.832617462606238	NA	NA	KXJ79619.1 hypothet	XP_001661445.1protein sex-lethal isoform X1
AAEL01348	rho guanine nucleotide exchange factor 18	0.0932634717556387	0.591711520892592	0.888387896109256	NA	NA	NA	XP_021695954.1rho guanine nucleotide exchange factor 18 isoform X2
AAEL00544	uncharacterized LOC5566519	0.0932891542907769	0.754851186240815	0.934127170333935	NA	NA	KXJ82351.1 hypothet	XP_001650912.1uncharacterized protein LOC5566519
AAEL02673	NA	0.0934201061509433	0.467063138458569	0.839144816051655	NA	aag03013 ; aag	NA	XP_021710512.1putative mediator of RNA polymerase II transcription subunit 12
AAEL01990	NA	0.093459966095972	0.704838858803376	0.917749125552377	NA	NA	NA	XP_021697265.1nuclear mitotic apparatus protein 1 isoform X1
AAEL01151	solute carrier family 45 member 4	0.0935145201138922	0.565286657867325	0.880302387766415	NA	NA	KXJ69491.1 hypothet	XP_001661721.2solute carrier family 45 member 4
AAEL00882	uncharacterized LOC5571130	0.0937516211601038	0.607878107681773	0.893547770777888	NA	NA	ETN66129.1 hypothe	XP_001659573.1uncharacterized protein LOC5571130
AAEL00690	uncharacterized LOC5571212	0.0938042879738721	0.435945433130247	0.820960220424486	NA	NA	NA	XP_021701780.1uncharacterized protein LOC5571212
AAEL01974	NA	0.0938435406942561	0.44188812572192	0.824296479402616	NA	NA	NA	XP_021700800.1Krueppel homolog 2
AAEL02361	NA	0.0938520125670064	0.71836123195928	0.921583569204186	NA	aag03040	NA	XP_021694994.1pre-mRNA-splicing factor Slu7
AAEL01983	NA	0.0939177515778711	0.42307896953175	0.814192545906728	NA	NA	NA	XP_021702355.1zinc finger protein 84 isoform X39
AAEL01142	AP-3 complex subunit mu-1	0.0939572811206855	0.559380721162769	0.877581080322399	NA	aag04142	KXJ84264.1 hypothet	XP_001655331.2AP-3 complex subunit mu-1
AAEL01326	acidic endochitinase SP2	0.0939602702832808	0.590047915786583	0.887668474886052	520	NA	NA	XP_001663424.2acidic endochitinase SP2
AAEL00491	D-glucuronyl C5-epimerase	0.093986645217459	0.621552477791483	0.89816390813084	534	aag01100 ; aag	XP_019525223.1 PRE	XP_021696906.1D-glucuronyl C5-epimerase isoform X2
AAEL00821	solute carrier family 25 member 35	0.0939945307188255	0.70777496034926	0.91797784850989	NA	NA	KXJ80482.1 hypothet	XP_001659038.2solute carrier family 25 member 35
AAEL01224	uncharacterized LOC5576001	0.094029351875326	0.397605682345584	0.797539006884967	NA	NA	XP_001845020.1 con	XP_001662345.2uncharacterized protein LOC5576001
AAEL00397	ell-associated factor Eaf	0.0941601672639418	0.47509799129966	0.84437941358363	NA	NA	XP_019552619.1 PRE	XP_001648111.1ell-associated factor Eaf isoform X2
AAEL00478	enoyl-CoA delta isomerase 1, mitochondrial	0.0942501589930996	0.750971900215943	0.932554085844433	NA	NA	XP_019528351.1 PRE	XP_001649721.2enoyl-CoA delta isomerase 1, mitochondrial
AAEL00354	stress-activated map kinase-interacting protein 1	0.0943409166477675	0.743105802671817	0.930265645309704	NA	aag04150	KXJ84552.1 hypothet	XP_001656925.1stress-activated map kinase-interacting protein 1
AAEL02478	NA	0.0944064190398707	0.722922586768022	0.921934742632737	NA	NA	NA	XP_021698912.1transcription factor grauzone isoform X1
AAEL01313	ras-like GTP-binding protein Rho1	0.0944146960351296	0.4199672029924	0.813636239562413	NA	aag04144 ; aag	NA	XP_001656384.1ras-like GTP-binding protein Rho1 isoform X1
AAEL00641	sodium- and chloride-dependent GABA transporter ine	0.0946013539833321	0.374922239823286	0.782198815065306	NA	NA	XP_019535863.1 PRE	XP_001657800.1sodium- and chloride-dependent GABA transporter ine isoform X2
AAEL00776	serine protease inhibitor 42Dd	0.0946330346498832	0.386134310792247	0.791224994795546	NA	NA	XP_019537028.1 PRE	XP_001658642.1serine protease inhibitor 42Dd isoform X5
AAEL01464	UDP-sugar transporter UST74c	0.0947919965530242	0.523781613543535	0.865215478807089	NA	NA	NA	XP_001649245.2UDP-sugar transporter UST74c
AAEL01369	protein aubergine	0.0948603645426749	0.544320361746136	0.870383849238518	NA	aag04320	NA	XP_001663870.2protein aubergine
AAEL01226	RNA polymerase I-specific transcription initiation factor RRN3	0.094980522981161	0.471262385443865	0.841643532909954	NA	NA	KFB38508.1 hypothe	XP_001655975.1RNA polymerase I-specific transcription initiation factor RRN3
AAEL00350	stromal membrane-associated protein 1	0.0950085952286661	0.546296036427842	0.870931414970375	NA	aag04144	KXJ68016.1 hypothet	XP_021704458.1stromal membrane-associated protein 1
AAEL01273	uncharacterized LOC5576750	0.0950774926768616	0.635441681078128	0.902538767814767	NA	NA	KXJ72427.1 hypothet	XP_001656247.1uncharacterized protein LOC5576750
AAEL00117	tRNA-dihydrouridine(47) synthase [NAD(P)(+)]-like	0.0951250517153074	0.428219638746406	0.816509970108666	NA	NA	KXJ71336.1 hypothet	XP_001658172.2tRNA-dihydrouridine(47) synthase [NAD(P)(+)]-like
AAEL00720	GATA-binding factor C	0.0951508098775868	0.850094426832997	0.96443951617769	NA	NA	XP_001866045.1 GAT	XP_021693422.1GATA-binding factor C isoform X2
AAEL00879	UPF0183 protein CG7083	0.0952319369678445	0.418060823207053	0.812788254564712	NA	NA	ETN62316.1 T01G9.2	XP_001659495.1UPF0183 protein CG7083
AAEL00601	serine protease inhibitor dipetalogastin	0.095286402068277	0.468067360652132	0.839644234660078	NA	NA	XP_019540925.1 PRE	XP_001651771.2serine protease inhibitor dipetalogastin
AAEL01383	proline-rich receptor-like protein kinase PERK9	0.0952887492789047	0.750888457940643	0.932554085844433	NA	NA	NA	XP_021694738.1proline-rich receptor-like protein kinase PERK9 isoform X1
AAEL00806	remodeling and spacing factor 1	0.0953846223675268	0.59296325080166	0.888529762455765	NA	NA	KXJ68291.1 hypothet	XP_021700020.1remodeling and spacing factor 1
AAEL00525	dynactin subunit 4	0.0953918749454309	0.634898234800665	0.902538767814767	NA	NA	KFB35593.1 AGAP00	XP_001650614.1dynactin subunit 4
AAEL01751	beta-glucuronidase	0.0954697300353095	0.413816851856304	0.80830878174097	00531 ; 00600 ; 00604 ;	aag01100 ; aag	NA	XP_021710751.1beta-glucuronidase isoform X2
AAEL01000	pre-mRNA-splicing factor SPF27	0.0954744210676473	0.695145239716568	0.916122517126697	NA	aag03040	NA	XP_001660542.1pre-mRNA-splicing factor SPF27
AAEL00440	HIG1 domain family member 2A, mitochondrial	0.0954998897108153	0.49218256802875	0.852141102645529	NA	NA	KFB48981.1 AGAP00	XP_001649065.1HIG1 domain family member 2A, mitochondrial
AAEL01357	synaptobrevin	0.0955782080419116	0.390785438715008	0.793285976044306	NA	aag04130	NA	XP_001663759.1synaptobrevin isoform X3
AAEL00894	tRNA methyltransferase 10 homolog A	0.0957540678100948	0.648464126751625	0.906554905014882	NA	NA	KXJ73194.1 hypothet	XP_001653611.1tRNA methyltransferase 10 homolog A
AAEL00105	10 kDa heat shock protein, mitochondrial	0.095778436601121	0.418141664541333	0.812788254564712	NA	NA	ETN60307.1 heat sho	XP_001657881.110 kDa heat shock protein, mitochondrial
AAEL00703	pyrroline-5-carboxylate reductase	0.0958190506980396	0.448439656718604	0.829288931094171	330	aag01100 ; aag	XP_019544355.1 PRE	XP_001658084.2pyrroline-5-carboxylate reductase
AAEL00167	cell division control protein 1	0.0958227781444209	0.543390356604924	0.869900911343623	NA	NA	KXJ70800.1 hypothet	XP_001653751.2cell division control protein 1
AAEL01942	NA	0.0958560696304687	0.385716657754838	0.791032913897932	NA	NA	NA	XP_021710726.1uncharacterized protein LOC5576935 isoform X4
AAEL01122	protein lifeguard 1	0.095901426285395	0.39336471227753	0.793953421864587	NA	NA	XP_019544410.1 PRE	XP_001647853.1protein lifeguard 1
AAEL01272	dual specificity mitogen-activated protein kinase kinase dSOR1	0.0960618970227726	0.539173068519124	0.869670565558805	NA	aag04013 ; aag	ETN62470.1 mitogen	XP_001662837.1dual specificity mitogen-activated protein kinase kinase dSOR1
AAEL01189	uncharacterized LOC5575504	0.0960871737469315	0.439119936724819	0.822299889650343	NA	NA	XP_001842572.1 con	XP_021702726.1uncharacterized protein LOC5575504 isoform X2
AAEL00576	glucose dehydrogenase [FAD, quinone]	0.0962138072101013	0.432171993587217	0.820005280371151	NA	aag01100 ; aag	XP_001651431.1 glu	XP_001651431.1glucose dehydrogenase [FAD, quinone]
AAEL00842	broad-complex core protein isoforms 1/2/3/4/5	0.0963012305018666	0.72644326750946	0.923177868750456	NA	aag04214 ; aag	XP_019546501.1 PRE	XP_001659219.2broad-complex core protein isoforms 1/2/3/4/5 isoform X7
AAEL01314	eukaryotic translation initiation factor 3 subunit I	0.0963228388293224	0.472344283048247	0.84283732647081	NA	aag03013	NA	XP_001656392.1eukaryotic translation initiation factor 3 subunit I
AAEL00756	leucine-rich PPR motif-containing protein, mitochondrial	0.0963333786750337	0.390838593796792	0.793285976044306	NA	NA	KXJ80628.1 hypothet	XP_021700466.1leucine-rich PPR motif-containing protein, mitochondrial

AAEL01545	NA	0.096369257068492	0.485172542250445	0.847655581875201	NA	NA	NA	XP_001664073.1rutC family protein UK114
AAEL00681	exosome complex component RRP46	0.096374092155966	0.692834811081602	0.916122517126697	NA	aag03018	KXJ68907.1 hypothet	XP_001658056.1exosome complex component RRP46
AAEL00988	pre-mRNA 3' end processing protein WDR33	0.0964240710557606	0.532371453583252	0.866700945213732	NA	aag03015	NA	XP_001654074.2pre-mRNA 3' end processing protein WDR33
AAEL00121	alanine--tRNA ligase, cytoplasmic	0.0964383402301149	0.423439187682002	0.814192545906728	970	aag00970	KFB53790.1 AGAP00	XP_001658296.1alanine--tRNA ligase, cytoplasmic
AAEL00337	beta-TrCP	0.0965010960166682	0.432782981308427	0.820005280371151	NA	aag04120 ; aag	KXJ73522.1 hypothet	XP_019530133.1 beta-TrCP-like
AAEL00624	poly(A) RNA polymerase gld-2 homolog B	0.0965431935230935	0.456402297595057	0.832617462606238	NA	NA	KXJ76885.1 hypothet	XP_021708653.1poly(A) RNA polymerase gld-2 homolog B
AAEL00352	SET and MYND domain-containing protein 4	0.0965967946055689	0.675728329166838	0.914438668430353	NA	NA	XP_001865734.1 con	XP_001656930.2SET and MYND domain-containing protein 4
AAEL01395	NA	0.0966557969299198	0.391405349184743	0.793285976044306	NA	NA	NA	XP_001664143.2probable ATP-dependent RNA helicase DDX47
AAEL01008	uncharacterized LOC5572865	0.0966693363490038	0.653268361533099	0.907272485895905	NA	aag04624	NA	XP_001660624.1uncharacterized protein LOC5572865
AAEL00368	nuclear distribution protein nudE homolog	0.096678651894879	0.508181731446439	0.859730781244726	NA	NA	KXJ69053.1 hypothet	XP_001657137.1nuclear distribution protein nudE homolog
AAEL00837	E3 ubiquitin-protein ligase Nedd-4	0.0966817994000992	0.389401320568568	0.793285976044306	NA	NA	XP_012277396.1 E3	XP_021706978.1E3 ubiquitin-protein ligase Nedd-4 isoform X3
AAEL00116	protein grainyhead	0.0967381929734188	0.642961467185174	0.905972350539329	NA	NA	XP_019532205.1 PRE	XP_021698368.1protein grainyhead isoform X1
AAEL00238	uncharacterized protein KIAA2013 homolog	0.0967627659524777	0.46651838025395	0.838900928502398	NA	NA	KFB40985.1 AGAP01	KFB40985.1AGAP010005-like protein
AAEL02130	NA	0.09689230321187	0.43770658286108	0.820960220424486	NA	aag04013 ; aag	NA	XP_021709031.1protein enhancer of sevenless 2B isoform X1
AAEL00045	histone acetyltransferase KAT2A	0.0968937569456818	0.558115581620879	0.877488406385106	NA	aag04330	ETN67891.1 histone	XP_001656424.1histone acetyltransferase KAT2A
AAEL00583	programmed cell death protein 4	0.0969186271971129	0.400617185794131	0.798648539435638	NA	NA	XP_001865859.1 pro	XP_021694820.1programmed cell death protein 4
AAEL00991	zinc finger and BTB domain-containing protein 17	0.0969578828247218	0.727288700264097	0.923566968708516	NA	NA	NA	XP_021713039.1zinc finger and BTB domain-containing protein 17
AAEL00342	zinc carboxypeptidase	0.0970047077399227	0.410502947738002	0.805855841282375	NA	NA	KXJ81228.1 hypothet	XP_001656792.2zinc carboxypeptidase
AAEL02322	NA	0.0972627304543856	0.708775906025014	0.918089462824826	NA	NA	NA	XP_021707606.1zinc finger protein 675-like
AAEL00883	ubiquitin-conjugating enzyme E2 R2	0.0973213842123558	0.439028091265703	0.822295236276614	NA	aag04120	XP_001843054.1 ubi	XP_001653472.1ubiquitin-conjugating enzyme E2 R2
AAEL00807	coiled-coil domain-containing protein 186	0.0973272068026185	0.658168957819096	0.909516556517693	NA	NA	XP_019534157.1 PRE	XP_021707172.1coiled-coil domain-containing protein 186 isoform X1
AAEL00540	uncharacterized LOC5566463	0.0973754356755165	0.780751714941712	0.94381989277494	NA	NA	KXJ68484.1 hypothet	XP_001650845.1uncharacterized protein LOC5566463
AAEL00456	uncharacterized LOC5565071	0.0973952500660679	0.682081901447659	0.914629465876247	NA	NA	KXJ73892.1 hypothet	XP_001649381.2uncharacterized protein LOC5565071
AAEL00734	trans-Golgi network integral membrane protein TGN38	0.0974607458135777	0.487818319390417	0.848846834445739	NA	NA	KXJ77432.1 hypothet	XP_001652700.1trans-Golgi network integral membrane protein TGN38
AAEL00851	elongation factor Tu, mitochondrial	0.0974673961203109	0.421070901238532	0.813840460658889	NA	NA	KXJ76856.1 hypothet	XP_001659326.1elongation factor Tu, mitochondrial
AAEL00887	NA	0.097536409805283	0.550115715045194	0.873690854305467	NA	NA	XP_001859578.1 hyp	XP_019548531.1PREDICTED: uncharacterized protein LOC109418759 isoform X3
AAEL02128	NA	0.097567605356244	0.381860477323931	0.787865496039196	NA	NA	NA	XP_021713176.1GIGYF family protein CG11148
AAEL00655	WD repeat-containing protein 26 homolog	0.0975901136358106	0.356170706894795	0.772867683747772	NA	NA	XP_001841859.1 WD	XP_021696622.1WD repeat-containing protein 26 homolog
AAEL00686	zinc finger protein 675	0.0976351347671534	0.770108946714402	0.941147080452193	NA	NA	KXJ84567.1 hypothet	XP_001652279.2zinc finger protein 675 isoform X2
AAEL01054	heat shock factor-binding protein 1	0.0976729700476638	0.393885560445385	0.794331564150434	NA	NA	ETN60862.1 heat shc	XP_001654647.1heat shock factor-binding protein 1
AAEL00600	coiled-coil domain-containing protein 28A	0.0977450324749382	0.647803052742859	0.906554905014882	NA	NA	XP_019550786.1 PRE	XP_001651762.1coiled-coil domain-containing protein 28A isoform X2
AAEL00431	epidermal growth factor receptor	0.0978034830072388	0.555881981200431	0.876514925250035	NA	aag04144 ; aag	KXJ75973.1 hypothet	XP_021702635.1epidermal growth factor receptor isoform X1
AAEL00093	uncharacterized LOC5567486	0.0978603796928116	0.691975659659354	0.916122517126697	NA	NA	KXJ69527.1 hypothet	ABI64119.1insulin-like peptide 4 precursor
AAEL01081	protein ARV1	0.0980010599456412	0.782774052612148	0.943962424451135	NA	NA	XP_019529595.1 PRE	XP_001655002.2protein ARV1
AAEL02025	NA	0.0981404391230289	0.676891325129845	0.914438668430353	NA	NA	NA	XP_021698324.1uncharacterized protein LOC5564245
AAEL00272	peroxisomal membrane protein PMP34	0.0981638613770754	0.5136025566799	0.861309357197051	NA	aag04146	KXJ68746.1 hypothet	XP_021695616.1peroxisomal membrane protein PMP34
AAEL01817	NA	0.0981874168042325	0.74586740445603	0.931365937538534	NA	NA	NA	XP_021708580.1putative helicase mov-10-B.1
AAEL02797	NA	0.098297640745915	0.678795708430052	0.914481546552978	NA	NA	NA	XP_021705561.1Down syndrome cell adhesion molecule-like protein Dscam2 isoform X17
AAEL00100	1-acyl-sn-glycerol-3-phosphate acyltransferase alpha	0.0983335535621938	0.625028975270181	0.899236496315855	00561 ; 00564	aag01100 ; aag	XP_001843537.1 1-a	XP_021704643.11-acyl-sn-glycerol-3-phosphate acyltransferase alpha
AAEL01955	NA	0.0983823815663615	0.681970158457811	0.914629465876247	NA	NA	NA	XP_021698931.1uncharacterized protein LOC5566023
AAEL01352	peroxiredoxin-2	0.0983981330040416	0.390666523545883	0.793285976044306	480	NA	NA	XP_019541392.1 peroxiredoxin-2-like
AAEL01972	NA	0.0984536053242017	0.365202564230501	0.779358533406556	NA	NA	NA	XP_021693813.1uncharacterized protein LOC5569692 isoform X1
AAEL00574	transcription initiation factor TFIID subunit 10	0.0984803127133627	0.540991284183386	0.869735874782513	NA	aag03022	ETN66463.1 transcrip	XP_001651390.1transcription initiation factor TFIID subunit 10
AAEL02316	NA	0.0985039409654957	0.371987679661796	0.781817769662763	NA	NA	NA	XP_021693516.1attractin isoform X1
AAEL00190	probable actin-related protein 2/3 complex subunit 2	0.0985255955326601	0.424231718664541	0.814488795690304	NA	aag04144	ETN62083.1 arp2/3 [	XP_001654200.1probable actin-related protein 2/3 complex subunit 2
AAEL00723	protein asteroid	0.0985444526900702	0.700411011069401	0.916629842461068	NA	NA	KXJ83147.1 hypothet	XP_021703178.1protein asteroid
AAEL01317	peroxidase	0.0986628793434951	0.367204251292746	0.780578423384551	NA	NA	NA	XP_019558882.1 peroxidase
AAEL01183	probable methylthioribulose-1-phosphate dehydratase	0.0987543645182283	0.370612842239038	0.781817769662763	270	aag01100 ; aag	NA	XP_001655752.2probable methylthioribulose-1-phosphate dehydratase isoform X3
AAEL01021	facilitated trehalose transporter Tret1	0.0988332054282753	0.37171145362648	0.781817769662763	NA	NA	NA	XP_001654366.1facilitated trehalose transporter Tret1
AAEL00714	potassium voltage-gated channel protein Shaw	0.0988956209560169	0.771332508106362	0.941147080452193	NA	NA	KFB36686.1 voltage-	XP_019551611.1 potassium voltage-gated channel protein Shaw-like
AAEL00764	RWD domain-containing protein 1	0.098988032478921	0.66766437447243	0.912418872165226	NA	NA	KXJ80047.1 hypothet	XP_001658541.1RWD domain-containing protein 1
AAEL00273	leukocyte elastase inhibitor	0.0990263771052399	0.664893536702346	0.911808086169482	NA	NA	KXJ73356.1 hypothet	XP_001662189.2leukocyte elastase inhibitor
AAEL01052	nodal modulator 1	0.0990979977361714	0.351545836563074	0.769127557920186	NA	NA	NA	XP_021696015.1nodal modulator 1 isoform X1
AAEL01350	membralin	0.0991486963345938	0.425974229151813	0.814623464645531	NA	NA	NA	XP_019550873.1 membralin
AAEL02389	NA	0.0992282921950758	0.691992190102704	0.916122517126697	NA	NA	NA	XP_019539093.1 nascent polypeptide-associated complex subunit alpha, muscle-specific form-like
AAEL00111	DNA-binding protein Ewg	0.0992588677088624	0.523819025579771	0.865215478807089	NA	NA	KXJ80611.1 hypothet	XP_021710094.1DNA-binding protein Ewg isoform X1
AAEL00770	T-complex protein 1 subunit delta	0.0992926628753844	0.384483816425118	0.790080064171371	NA	NA	KXJ76960.1 hypothet	XP_001652824.1T-complex protein 1 subunit delta
AAEL00093	heparan-alpha-glucosaminide N-acetyltransferase	0.0994056420764506	0.659172661713116	0.909580313002131	NA	aag01100 ; aag	ETN58096.1 hypothe	XP_001657450.1heparan-alpha-glucosaminide N-acetyltransferase
AAEL01398	glutaredoxin-C4	0.0995102323400384	0.444081889368657	0.825840341231698	NA	NA	NA	XP_001657239.1glutaredoxin-C4 isoform X2
AAEL00154	zinc finger protein 91	0.0995778518075547	0.747435944721582	0.931839258004064	NA	NA	XP_019553892.1 PRE	XP_021695926.1zinc finger protein 91 isoform X1
AAEL00813	conserved oligomeric Golgi complex subunit 3	0.0996444248747554	0.634559851415803	0.902506916959539	NA	NA	KXJ76586.1 hypothet	XP_001658958.2conserved oligomeric Golgi complex subunit 3
AAEL01985	NA	0.0997579997202332	0.513300908768881	0.861304697755515	NA	aag04624	NA	XP_021693939.1serine protease persephone
AAEL01163	cyclic nucleotide-gated cation channel beta-3	0.0998093343896179	0.679703167304539	0.914629465876247	NA	NA	KXJ69066.1 hypothet	XP_021705480.1cyclic nucleotide-gated cation channel beta-3
AAEL00148	phosphoglucomutase-2	0.100049212924499	0.460665323137151	0.834457146359854	520	aag01100 ; aag	KXJ84032.1 hypothet	AAX47079.1phosphoglucomutase 2
AAEL01105	mitotic-spindle organizing protein 1	0.100141341151453	0.673465381356348	0.912968243848131	NA	NA	XP_001848899.1 con	XP_001661328.2mitotic-spindle organizing protein 1
AAEL02140	NA	0.100243272913187	0.56646425763329	0.880302387766415	NA	aag03460	NA	XP_021696042.1WD repeat-containing protein 48 homolog
AAEL01207	GTPase HRas	0.100287967285411	0.352549143546896	0.76935039265884	NA	aag04013 ; aag	ETN59979.1 ras [Ano	XP_001662234.1GTPase HRas
AAEL00550	protein YIF1B-A	0.100290987318745	0.377660345176871	0.78394552138903	NA	NA	XP_001869256.1 con	XP_001651014.1protein YIF1B-A isoform X2
AAEL00630	etoposide-induced protein 2.4 homolog	0.100339216777563	0.626170687827061	0.899753696031326	NA	NA	XP_019525334.1 PRE	XP_021702146.1etoposide-induced protein 2.4 homolog
AAEL00245	zinc finger protein 1	0.100374125797997	0.616170518559217	0.897164966965308	NA	NA	KXJ83658.1 hypothet	XP_021699347.1zinc finger protein 1
AAEL00175	H(+)/Cl(-) exchange transporter 5	0.100386035633475	0.361193904252217	0.775660748408146	NA	NA	XP_019562569.1 PRE	XP_001653972.2 H(+)/Cl(-) exchange transporter 5
AAEL00181	solute carrier organic anion transporter family member 4A1	0.100386927193961	0.420228839226862	0.813636239562413	NA	NA	KFB39168.1 AGAP00	XP_021702469.1solute carrier organic anion transporter family member 4A1
AAEL02427	NA	0.100517013958829	0.766524802604195	0.939178856256477	NA	NA	NA	XP_021703328.1uncharacterized protein LOC110677056 isoform X2
AAEL01313	6-phosphofructo-2-kinase/fructose-2,6-bisphosphatase	0.100521974804385	0.397989471812189	0.797539006884967	51	NA	NA	XP_021706733.16-phosphofructo-2-kinase/fructose-2,6-bisphosphatase isoform X4
AAEL01277	aminopeptidase N	0.100553162773383	0.561932360382413	0.878972918598592	NA	NA	XP_019531218.1 PRE	XP_001662886.2aminopeptidase N
AAEL00173	uncharacterized LOC5572062	0.100558930677896	0.788686981875868	0.945232821987723	NA	NA	KXJ82905.1 hypothet	XP_019530900.1 UPF0764 protein C16orf89 homolog
AAEL01022	phosphatidylserine decarboxylase proenzyme, mitochondrial	0.100590685445822	0.400368607508944	0.798648539435638	564	aag01100 ; aag	NA	XP_021704735.1phosphatidylserine decarboxylase proenzyme, mitochondrial isoform X4
AAEL00523	glycogen synthase kinase-3 beta	0.10060881423634	0.368365173625797	0.78164928156096	NA	aag04150 ; aag	XP_019529378.1 PRE	XP_001650547.1glycogen synthase kinase-3 beta isoform X2
AAEL00226	GTP cyclohydrolase 1	0.100668370940925	0.430713159810778	0.819396836175062	790	aag01100 ; aag	KXJ72579.1 hypothet	XP_001661181.2GTP cyclohydrolase 1 isoform X2
AAEL02063	NA	0.100953982527642	0.700481613028791	0.916629842461068	NA	NA	NA	XP_021697365.1 DNA replication factor Cdt1
AAEL01999	NA	0.101099160167533	0.366378691924869	0.780420537752928	NA	aag04140 ; aag	NA	XP_021694839.1high mobility group protein DSP1
AAEL01000	zinc finger protein 37	0.101119096156915	0.673668475555544	0.912974567314009	NA	NA	NA	XP_001660561.1zinc finger protein 37

AAEL0274(	NA	0.101134366820519	0.691183765334242	0.916122517126697	NA	NA	NA	XP_021705810.1uncharacterized protein LOC5565978
AAEL00803	uncharacterized LOC5569950	0.101135742678369	0.449741214393962	0.829440335346263	NA	NA	XP_001862159.1 con	XP_001658850.1uncharacterized protein LOC5569950
AAEL02302	NA	0.101146970940731	0.569498407716945	0.880635379772131	NA	NA	NA	XP_021695482.1density-regulated protein homolog
AAEL00589	tRNA-dihydrouridine(20a/20b) synthase [NAD(P)+]-like	0.10117233757746	0.585622700661433	0.885239904594145	NA	NA	KXJ80677.1 hypothet	XP_021707169.1tRNA-dihydrouridine(20a/20b) synthase [NAD(P)+]-like
AAEL02389	NA	0.101313665482838	0.719489756318736	0.921583569204186	NA	NA	NA	XP_021699122.1alcohol dehydrogenase 1 isoform X1
AAEL00502	zinc finger protein 729	0.101392935398835	0.809122803611409	0.948933370455872	NA	NA	KFB39028.1 AGAP00	XP_021703639.1zinc finger protein 729
AAEL01178	probable DNA mismatch repair protein Msh6	0.101503464417129	0.702039572117994	0.916881840462067	NA	aag03430	KXJ83487.1 hypothet	XP_001661901.2probable DNA mismatch repair protein Msh6
AAEL01971	NA	0.10151012824294	0.401927193649233	0.799283473604722	NA	NA	NA	XP_021705622.1microtubule-associated protein Jupiter isoform X4
AAEL00648	uncharacterized LOC5568042	0.101614912717332	0.778086901005519	0.942719639638287	NA	NA	KXJ77182.1 hypothet	XP_001651975.1uncharacterized protein LOC5568042 isoform X1
AAEL01108	HD domain-containing protein 2	0.101622415001461	0.392038033045834	0.793285976044306	240	NA	KXJ81631.1 hypothet	XP_001661397.1HD domain-containing protein 2 isoform X1
AAEL00132	replication factor C subunit 1	0.10175602481927	0.618111091241657	0.897318336565571	NA	aag03420 ; aag	KXJ73659.1 hypothet	XP_021704728.1replication factor C subunit 1
AAEL00512	rutC family protein UK114	0.101883788054773	0.524874801532939	0.865680248818465	NA	NA	KXJ80163.1 hypothet	XP_001650394.1rutC family protein UK114
AAEL0100(	exosome component 10	0.101984474288183	0.517044293684098	0.861841953516531	NA	aag03018	NA	XP_001660547.2exosome component 10 isoform X1
AAEL00739	N-acetylglucosamine-1-phosphotransferase subunits alpha/beta	0.102024767538321	0.58932759510161	0.887124575515297	NA	NA	XP_001847152.1 N-a	XP_001652738.2N-acetylglucosamine-1-phosphotransferase subunits alpha/beta
AAEL00602	vanin-like protein 1	0.102027637054123	0.485296945685827	0.847655581875201	NA	NA	XP_019537474.1 PRE	XP_001657370.2vanin-like protein 1
AAEL02463	NA	0.102070228333478	0.838528061658683	0.962348963212321	NA	NA	NA	XP_021693135.1head peptide
AAEL00127	nucleobindin-2	0.102162932771764	0.353567564886244	0.770323402046695	NA	NA	KXJ83296.1 hypothet	XP_019535949.1 nucleobindin-2-like
AAEL02551	NA	0.102180306606998	0.624668397367224	0.898996686480879	NA	NA	NA	XP_021695187.1zinc finger CCCH domain-containing protein 18
AAEL00281	splicing factor U2AF 50 kDa subunit	0.102203595483558	0.369552670595708	0.781817769662763	NA	aag03040	KXJ70060.1 hypothet	XP_001662443.1splicing factor U2AF 50 kDa subunit
AAEL01415	transmembrane protein 198	0.102206376959067	0.702834479555772	0.917106722424789	NA	NA	NA	XP_001648098.1transmembrane protein 198
AAEL02048	NA	0.102221492021907	0.72836265496081	0.923566968708516	NA	NA	NA	XP_001230905.1Anopheles gambiae str. PEST AGAP012443
AAEL01022	probable dolichol-phosphate mannosyltransferase	0.102234968265259	0.570391150384015	0.880923047085115	510	aag01100 ; aag	NA	XP_001660687.1probable dolichol-phosphate mannosyltransferase
AAEL00135	NA	0.10229212821878	0.422481529071928	0.814192545906728	NA	NA	NA	ABF18516.1possible mucin
AAEL01529	protein ABHD1	0.102325559629967	0.409961782693113	0.805356078752463	NA	NA	NA	XP_021698925.1protein ABHD1
AAEL01385	raf homolog serine/threonine-protein kinase phl	0.102348216530733	0.505891791514549	0.858744381072989	NA	aag04013 ; aag	NA	XP_021710700.1raf homolog serine/threonine-protein kinase phl
AAEL00671	DNA polymerase iota	0.102365104917113	0.629066512273525	0.901240860336444	NA	aag03460	XP_001868410.1 DN/	XP_001657975.2 DNA polymerase iota
AAEL0046(	adenylyltransferase and sulfurtransferase MOCS3	0.102367966874102	0.464222537815969	0.837274753622609	NA	aag04122	KXJ75321.1 hypothet	XP_001649510.2adenylyltransferase and sulfurtransferase MOCS3
AAEL00181	gastrula zinc finger protein XlCGF57.1	0.102547260657797	0.594871368912545	0.888948786307326	NA	NA	KXJ72304.1 hypothet	XP_001660404.2gastrula zinc finger protein XlCGF57.1
AAEL01103	integrin alpha-PS3	0.102707549935387	0.526770409497516	0.865973876319045	NA	NA	KFB36449.1 AGAP00	XP_021698952.1integrin alpha3
AAEL00539	importin-13	0.102783201038233	0.605461494531011	0.893547770777888	NA	NA	KXJ70045.1 hypothet	XP_019537091.1 importin-13-like
AAEL0027(	pyridoxine-5'-phosphate oxidase	0.102907135929412	0.450882484871399	0.830259097859495	750	aag01100 ; aag	KXJ80141.1 hypothet	XP_001662205.1pyridoxine-5'-phosphate oxidase
AAEL02648	NA	0.103062730684848	0.774896125574577	0.941456845463065	NA	aag03460 ; aag	NA	XP_021697634.1mismatch repair endonuclease PMS2 isoform X1
AAEL0232(	NA	0.103063440566498	0.682408509019607	0.914629465876247	NA	NA	NA	XP_021693778.1zinc finger protein 91
AAEL01127	zinc finger protein ubi-d4	0.103118484230786	0.668182788136987	0.912528645953778	NA	NA	KXJ67920.1 hypothet	XP_001661565.1zinc finger protein ubi-d4
AAEL0072(	pre-mRNA-splicing factor ISY1 homolog	0.103189098838628	0.779374148256471	0.943566949556407	NA	aag03040	KXJ74036.1 hypothet	XP_001658236.2pre-mRNA-splicing factor ISY1 homolog
AAEL0139(	E3 ubiquitin-protein ligase RNF126	0.103505255892016	0.699406153238997	0.916629842461068	NA	NA	NA	XP_021705203.1E3 ubiquitin-protein ligase RNF126
AAEL01954	NA	0.103645781923719	0.415764007280563	0.809705903702803	NA	NA	NA	XP_021696822.1neuroglian isoform X2
AAEL0255(	NA	0.10369427521047	0.350869385805914	0.768189409513686	NA	NA	NA	XP_001657095.2phosphofurin acidic cluster sorting protein 2 isoform X1
AAEL00007	zinc transporter 7	0.103920819351947	0.395199223127336	0.795934176235007	NA	NA	ETN60040.1 cation e	XP_001647887.1zinc transporter 7
AAEL00567	adenosine deaminase 2	0.104100820260016	0.79993922642465	0.946215055875588	230	aag01100 ; aag	KXJ73474.1 hypothet	XP_001651236.2adenosine deaminase 2
AAEL00343	probable cGMP 3',5'-cyclic phosphodiesterase subunit delta	0.104117114359756	0.691359853602455	0.916122517126697	NA	aag01100 ; aag	XP_001851502.1 reti	XP_001656796.2probable cGMP 3',5'-cyclic phosphodiesterase subunit delta
AAEL00535	dynein light chain roadblock-type 2	0.104213655706078	0.467627594704595	0.839303515988236	NA	NA	NP_523771.1 roadbl	XP_001650782.1dynein light chain roadblock-type 2
AAEL00685	protein NPC2 homolog	0.104338012533139	0.450949247022578	0.830259097859495	NA	aag04142	XP_001846187.1 con	XP_001652290.1protein NPC2 homolog
AAEL00927	carnosine N-methyltransferase	0.104342614984865	0.691933555491905	0.916122517126697	340	aag01100 ; aag	NA	XP_001653776.1carnosine N-methyltransferase
AAEL01734	hyphally-regulated protein	0.104485589913582	0.412040384116514	0.806884477085645	NA	NA	NA	XP_011493149.1hyphally-regulated protein
AAEL00284	inositol polyphosphate multikinase	0.104575010212317	0.650442278977662	0.906932039402093	NA	aag01100 ; aag	XP_019552511.1 PRE	XP_021709290.1inositol polyphosphate multikinase isoform X1
AAEL02222	NA	0.104576593915833	0.422819902611006	0.814192545906728	NA	NA	NA	XP_021710880.1nuclear export mediator factor NEMF homolog
AAEL0041(	splicing factor 1	0.104754055242614	0.510859647948436	0.860452247709668	NA	NA	KXJ74390.1 hypothet	XP_001648609.1splicing factor 1
AAEL00913	probable cytochrome P450 6d4	0.104823248805003	0.604336370570426	0.893547770777888	NA	NA	AEB77682.1 cytochro	XP_001653689.3 probable cytochrome P450 6d4
AAEL0181(	NA	0.104827575815706	0.386772712783593	0.791623050051228	NA	NA	NA	XP_021711138.1ankyrin repeat domain-containing protein 27 isoform X1
AAEL00455	tRNA (guanine-N(7)-)-methyltransferase non-catalytic subunit wuho	0.104898829352045	0.655630925148116	0.908328180248866	NA	NA	KXJ69319.1 hypothet	XP_001649398.2tRNA (guanine-N(7)-)-methyltransferase non-catalytic subunit wuho
AAEL00989	A-kinase anchor protein 17A	0.104937615986161	0.507296917330226	0.859406428557307	NA	NA	NA	XP_021710774.1A-kinase anchor protein 17A isoform X2
AAEL01387	metallophosphoesterase 1 homolog	0.104950486655678	0.670921565681749	0.912812708172959	NA	NA	NA	XP_001657197.2metallophosphoesterase 1 homolog
AAEL01959	NA	0.105006342469311	0.574936596891201	0.881197098233729	NA	aag03008	NA	XP_021704985.1periodic tryptophan protein 2 homolog
AAEL00442	protein canopy homolog 4	0.105067698477519	0.459447107776635	0.833904614487257	NA	NA	KFB47184.1 AGAP01	XP_001649130.1protein canopy homolog 4
AAEL00802	histone-lysine N-methyltransferase Suv4-20-like	0.105110723491687	0.563626011689978	0.879811121216485	310	aag01100 ; aag	KXJ74254.1 hypothet	XP_021705001.1histone-lysine N-methyltransferase Suv4-20-like
AAEL0073(	uncharacterized LOC5569111	0.105123602564563	0.702929114677465	0.917106722424789	NA	NA	KXJ68910.1 hypothet	XP_001652710.2uncharacterized protein LOC5569111
AAEL02228	NA	0.105165454202382	0.465985889873002	0.838483018891546	NA	aag03010	NA	XP_001847201.140S ribosomal protein S27
AAEL00907	death-associated inhibitor of apoptosis 1	0.105182729764364	0.355410573494412	0.772799644918615	NA	aag04120 ; aag	XP_019538758.1 PRE	XP_021695480.1death-associated inhibitor of apoptosis 1
AAEL02615	NA	0.105206998618579	0.48939518376254	0.849220154814801	NA	NA	NA	NA
AAEL01173	enoyl-CoA hydratase domain-containing protein 3, mitochondrial	0.10522632747465	0.640443914313542	0.904464102888003	NA	NA	KXJ71236.1 hypothet	XP_001661868.1enoyl-CoA hydratase domain-containing protein 3, mitochondrial
AAEL00857	tRNA-splicing endonuclease subunit Sen34	0.105227904218912	0.782453664095413	0.943962424451135	NA	NA	KXJ68944.1 hypothet	XP_001653315.2tRNA-splicing endonuclease subunit Sen34
AAEL00988	probable histone-binding protein Caf1	0.105300577530636	0.419565223834218	0.813550586736479	NA	NA	NA	XP_001654079.1probable histone-binding protein Caf1
AAEL01428	myb protein	0.105330580353384	0.636797470396348	0.902893334596658	NA	NA	NA	XP_001648344.3myb protein isoform X1
AAEL0143(	fatty-acid amide hydrolase 2	0.105548484381324	0.423642024538298	0.814192545906728	970	NA	NA	XP_001648552.1fatty-acid amide hydrolase 2
AAEL02052	NA	0.105553752680541	0.469582535928065	0.84065046403825	NA	NA	NA	XP_021711753.1 facilitated trehalose transporter Tret1
AAEL01708	pyrimidodiazepine synthase	0.105607037386454	0.413695855283661	0.808244004314278	00980 ; 00982 ; 00053 ;	aag01100 ; aag	NA	XP_011493093.2pyrimidodiazepine synthase
AAEL00565	protein PRRC1	0.105748270623962	0.454820885649281	0.832617462606238	NA	NA	KXJ68999.1 hypothet	XP_001651220.2protein PRRC1
AAEL0092(	zinc finger protein 853	0.105785217403143	0.466673967942162	0.838900928502398	NA	NA	NA	XP_021713295.1zinc finger protein 853
AAEL01307	46 kDa FK506-binding nuclear protein	0.10584899177886	0.356449733734574	0.772867683747772	NA	NA	NA	XP_021695012.146 kDa FK506-binding nuclear protein
AAEL01564	transcription factor IIIA	0.105913188535602	0.766389423753087	0.939178856256477	NA	NA	NA	XP_001647577.1transcription factor IIIA
AAEL00177	bax inhibitor 1	0.105949289505152	0.348460251296802	0.766929259132545	NA	NA	XP_001846573.1 bax	XP_001653970.1bax inhibitor 1
AAEL02557	NA	0.105959796502856	0.379514094641775	0.785666748907449	NA	NA	NA	XP_001652005.2mitochondrial import receptor subunit TOM70
AAEL0052(	U3 small nucleolar ribonucleoprotein protein IMP3	0.106027871531457	0.53987098047056	0.869709828277711	NA	aag03008	XP_001867852.1 U3	XP_001650598.2U3 small nucleolar ribonucleoprotein protein IMP3
AAEL01171	non-structural maintenance of chromosomes element 1 homolog	0.106046625205867	0.735276067821762	0.926702094411052	NA	NA	KXJ74565.1 hypothet	XP_001655653.2non-structural maintenance of chromosomes element 1 homolog
AAEL0102(	ecto-NOX disulfide-thiol exchanger 2	0.106057164196994	0.688948738398032	0.916010156153545	NA	NA	NA	XP_001660681.1ecto-NOX disulfide-thiol exchanger 2
AAEL00021	39S ribosomal protein L2, mitochondrial	0.1061294233177	0.380247818282863	0.786294135283846	NA	aag03010	ETN67827.1 mitocho	XP_001658945.139S ribosomal protein L2, mitochondrial
AAEL00615	histone H3.3	0.106155334808338	0.356119646734772	0.772867683747772	NA	NA	3AV2_A Chain A, The	XP_015819260.1 histone H3.3 isoform X1
AAEL01445	uncharacterized LOC5564468	0.106399048952781	0.653747189625459	0.907357713262968	NA	NA	NA	XP_001648815.2uncharacterized protein LOC5564468
AAEL00787	NA	0.106417099026045	0.571347669751597	0.880923047085115	NA	NA	NA	AAL76018.1putative 34 kDa secreted protein
AAEL0017(	chaoptin	0.106439936453088	0.583053432469507	0.884917975521096	NA	NA	KXJ72853.1 hypothet	XP_021698716.1chaoptin isoform X1
AAEL01309	protein tantalus	0.106452976803235	0.728628618227766	0.923566968708516	NA	NA	NA	XP_001663302.1protein tantalus

AAEL00262	tryptase	0.106530877312991	0.426874946599871	0.8154667341939	NA	NA	KXJ73246.1 hypothet	XP_019531763.1 tryptase-like
AAEL00466	tRNA pseudouridine(38/39) synthase	0.106545752492876	0.551590006188677	0.874531710113044	NA	NA	KXJ71421.1 hypothet	XP_001649570.2tRNA pseudouridine(38/39) synthase
AAEL00984	G kinase-anchoring protein 1-A	0.106547633994367	0.691920092478287	0.916122517126697	NA	NA	NA	XP_001660455.1G kinase-anchoring protein 1-A
AAEL01075	trafficking protein particle complex subunit 10	0.106664436403156	0.618980627245348	0.897318336565571	NA	NA	KXJ68771.1 hypothet	XP_001654913.2 trafficking protein particle complex subunit 10
AAEL00729	uncharacterized LOC5568990	0.106719490570997	0.699104558363984	0.916629842461068	NA	NA	XP_001866650.1 con	XP_019564685.1 F-box/LRR-repeat protein 13-like isoform X2
AAEL00717	tumor protein D54	0.106876769511845	0.324586795073003	0.748164514778316	NA	NA	XP_019532251.1 PRE	XP_021698407.1tumor protein D52 isoform X3
AAEL00931	ras-related protein Rab-11B	0.106898658335828	0.319602209024874	0.745684464362801	NA	aag04144	NA	XP_001659918.1ras-related protein Rab-11B
AAEL00634	epoxide hydrolase 1	0.106926940740776	0.400016861099315	0.798648539435638	NA	NA	KXJ68369.1 hypothet	XP_001651935.1epoxide hydrolase 1
AAEL02700	NA	0.106956121141178	0.549795870371051	0.873333609382774	NA	NA	NA	XP_021695083.1guanine nucleotide-releasing factor 2 isoform X1
AAEL00346	uncharacterized protein C16orf52 homolog A	0.106956262526516	0.692977129435095	0.916122517126697	NA	NA	ETN63891.1 hypothe	XP_001656833.1uncharacterized protein C16orf52 homolog A
AAEL00185	mitochondrial Rho GTPase	0.1069668901274	0.396814508946194	0.797267928236437	NA	aag04214 ; aag	KXJ78964.1 hypothet	XP_021700837.1 mitochondrial Rho GTPase
AAEL00131	zinc finger protein 25	0.106973208184108	0.682201381467212	0.914629465876247	NA	NA	XP_001864295.1 zinc	XP_001653044.1zinc finger protein 25
AAEL00066	uncharacterized LOC5565425	0.10722007299292	0.713087345885843	0.919252089784076	00561 ; 00073	NA	KXJ71301.1 hypothet	XP_021710466.1uncharacterized protein LOC5565425
AAEL00623	glycerol kinase	0.107229383453207	0.443742033921654	0.82574604573247	561	aag01100 ; aag	XP_019548025.1 PRE	XP_001651832.2glycerol kinase
AAEL00321	guanine deaminase	0.107332842806602	0.620699521115078	0.897385728224103	230	aag01100 ; aag	KXJ70867.1 hypothet	XP_021693845.1guanine deaminase
AAEL01353	arrestin homolog	0.107343587813655	0.365501606050829	0.779635090143656	NA	NA	NA	XP_001663732.1arrestin homolog
AAEL02470	NA	0.107457449958462	0.504531974616948	0.858744381072989	NA	NA	NA	XP_021700136.1dehydrogenase/reductase SDR family protein 7-like
AAEL01528	synaptic vesicle glycoprotein 2B	0.107706130841323	0.59784510070174	0.890434276835745	NA	NA	NA	XP_001647788.2synaptic vesicle glycoprotein 2B
AAEL00405	glucose-induced degradation protein 8 homolog	0.10773260418299	0.399326571721811	0.798648539435638	NA	NA	KFB40237.1 hypothe	XP_001648371.1glucose-induced degradation protein 8 homolog
AAEL01817	NA	0.10777376748356	0.638099532807961	0.903769724626575	04660 ; 04658	NA	NA	XP_021711633.1dual specificity protein phosphatase Mpk3 isoform X2
AAEL01493	uncharacterized LOC5565671	0.10778726257962	0.683442975308083	0.914629465876247	NA	NA	NA	XP_001650026.1uncharacterized protein LOC5565671
AAEL00127	uncharacterized LOC5569608	0.107825687097089	0.418471959122376	0.813258493736875	NA	NA	NA	XP_001652888.1uncharacterized protein LOC5569608 isoform X1
AAEL02691	NA	0.107826771174213	0.650773019578879	0.906932039402093	NA	NA	NA	XP_021694990.1myrosinase 1-like
AAEL01243	delta(3,5)-Delta(2,4)-dienoyl-CoA isomerase, mitochondrial	0.107857103157192	0.548962542506005	0.873169431463929	NA	aag04146	KXJ76177.1 hypothet	XP_001662577.2delta(3,5)-Delta(2,4)-dienoyl-CoA isomerase, mitochondrial
AAEL00017	ubiquitin-conjugating enzyme E2 L3	0.107917662066867	0.397805969690597	0.797539006884967	NA	aag04120	ETN61834.1 ubiquitin	XP_019528124.1 ubiquitin-conjugating enzyme E2 L3-like
AAEL01975	NA	0.107945158285367	0.340799385187517	0.761728429073483	NA	NA	NA	XP_021711664.1low-density lipoprotein receptor-related protein 8 isoform X11
AAEL00522	transmembrane protein 216	0.108070676168319	0.4659123607539	0.838483018891546	NA	NA	ETN64373.1 hypothe	XP_001650541.1transmembrane protein 216
AAEL00123	disintegrin and metalloproteinase domain-containing protein 12	0.108128545884098	0.821937799631355	0.954414522300763	NA	NA	NA	XP_021701463.1disintegrin and metalloproteinase domain-containing protein 12 isoform X8
AAEL00311	arrestin homolog	0.108236765787619	0.425422645280022	0.814623464645531	NA	aag04745	ETN62161.1 arrestin,	XP_001663234.1arrestin homolog
AAEL02717	NA	0.108319429327983	0.666314620700486	0.911964500946328	NA	NA	NA	XP_021707022.1FGFR1 oncogene partner-like
AAEL00864	ran-binding protein 9	0.108389231082876	0.348318839853856	0.766929259132545	NA	NA	XP_019536942.1 PRE	XP_001659381.2ran-binding protein 9
AAEL01123	probable small nuclear ribonucleoprotein E	0.108430851208515	0.464103356920685	0.837274753622609	NA	aag03040	ETN61245.1 small nu	XP_001661504.1probable small nuclear ribonucleoprotein E
AAEL01269	sodium-coupled monocarboxylate transporter 1	0.108717343470489	0.488598531754236	0.848846834445739	NA	NA	ETN58506.1 sodium/	XP_001656201.1sodium-coupled monocarboxylate transporter 1
AAEL00222	pyroglutamyl-peptidase 1	0.108774791676223	0.543475368323679	0.869900911343623	NA	NA	XP_001845154.1 pyr	XP_001654990.2pyroglutamyl-peptidase 1
AAEL01110	lysosomal-associated transmembrane protein 4B	0.108911460936448	0.350570950766541	0.768176246660341	NA	aag04142	XP_019535637.1 PRE	XP_001661421.2lysosomal-associated transmembrane protein 4B isoform X1
AAEL01321	triokinase/FMN cyclase	0.108978110430993	0.374952122831571	0.782198815065306	NA	aag01100 ; aag	NA	XP_001663385.2triokinase/FMN cyclase
AAEL01138	protein VAC14 homolog	0.109091465474325	0.613521857640329	0.895847053952127	NA	NA	KFB41860.1 AGAP00	XP_001655315.1protein VAC14 homolog
AAEL00625	opsin-1	0.109188259802483	0.479590033397951	0.845454613425911	NA	NA	XP_001862198.1 ops	XP_019527761.1 opsin-1-like
AAEL00473	26S proteasome non-ATPase regulatory subunit 14	0.109350839266887	0.447294233644271	0.828502725039167	NA	aag03050	CRL02753.1 CLUMA_	XP_001649644.126S proteasome non-ATPase regulatory subunit 14
AAEL00646	uncharacterized LOC5568012	0.10937680778192	0.577390056227206	0.881961491587822	NA	NA	KXJ78275.1 hypothet	XP_001657829.2uncharacterized protein LOC5568012
AAEL02468	NA	0.109385499507677	0.69222631868375	0.916122517126697	NA	NA	NA	XP_021711205.1uncharacterized protein LOC110674002
AAEL00413	signal peptide peptidase-like 3	0.109411551611629	0.597413255913885	0.890122535770655	NA	NA	KXJ83249.1 hypothet	XP_001648511.1signal peptide peptidase-like 3
AAEL00235	uncharacterized LOC5574355	0.109484518608236	0.746765277708738	0.931365937538534	NA	NA	KXJ76379.1 hypothet	XP_021698902.1uncharacterized protein LOC5574355
AAEL00270	stromal cell-derived factor 2	0.10962095106423	0.4539182092154	0.832617462606238	NA	NA	KXJ78631.1 hypothet	XP_001662201.1stromal cell-derived factor 2
AAEL01953	NA	0.109628376377297	0.557549722099632	0.877259057086403	NA	aag04013	NA	XP_021700382.1striatin-3 isoform X1
AAEL02338	NA	0.109696749867846	0.45397105504611	0.832617462606238	NA	NA	NA	KFB52316.1AGAP011636-like protein
AAEL00661	ER membrane protein complex subunit 3	0.109712544316906	0.428909583064622	0.816951613943997	NA	NA	KXJ82694.1 hypothet	XP_001652133.2ER membrane protein complex subunit 3
AAEL00793	mitochondrial tRNA-specific 2-thiouridylase 1	0.109754092170245	0.719027212557687	0.921583569204186	NA	NA	KXJ74144.1 hypothet	XP_021708082.1mitochondrial tRNA-specific 2-thiouridylase 1
AAEL02305	NA	0.109772082202685	0.63353224205204	0.902506916959539	NA	NA	NA	XP_021703457.1zinc finger protein 62-like
AAEL01037	CTD nuclear envelope phosphatase 1 homolog	0.10979117273067	0.350619918561095	0.768176246660341	NA	NA	NA	XP_019537572.1 CTD nuclear envelope phosphatase 1 homolog isoform X2
AAEL02345	NA	0.10981326811971	0.335366425577358	0.757775574591595	NA	NA	NA	XP_001647679.1protein-L-isoaspartate(D-aspartate) O-methyltransferase
AAEL01165	carboxypeptidase Q	0.109881053152194	0.323914061522623	0.74778153390145	NA	NA	KXJ78049.1 hypothet	XP_001661833.2carboxypeptidase Q
AAEL00986	uncharacterized LOC5572533	0.109997546190483	0.375064825554854	0.782198815065306	NA	NA	NA	XP_001654062.1uncharacterized protein LOC5572533 isoform X2
AAEL00964	leucine zipper putative tumor suppressor 3	0.110025061986418	0.740085930340303	0.928618984286445	NA	NA	NA	XP_021713335.1leucine zipper putative tumor suppressor 3 isoform X1
AAEL01074	elongation factor G, mitochondrial	0.110104616854599	0.324641381179386	0.748164514778316	NA	NA	KXJ76412.1 hypothet	XP_001661064.2elongation factor G, mitochondrial
AAEL01304	exosome complex component RRP41	0.110185650061785	0.66590094302753	0.911964500946328	NA	aag03018	NA	XP_001663263.1exosome complex component RRP41
AAEL00164	ATP-sensitive inward rectifier potassium channel 11	0.110219927519323	0.616133391525893	0.897164966965308	NA	NA	KXJ73981.1 hypothet	XP_021701200.1ATP-sensitive inward rectifier potassium channel 11 isoform X2
AAEL01339	cyclin-C	0.110328654720437	0.633134091454943	0.902506916959539	NA	NA	NA	XP_019560623.1 cyclin-C
AAEL02657	NA	0.110376408465931	0.493380751145824	0.852598999444858	NA	NA	NA	XP_021712979.1 protein SPT2 homolog
AAEL00477	peptidyl-prolyl cis-trans isomerase-like 3	0.110515663787464	0.578960506828085	0.882283691803025	NA	NA	ETN59390.1 cyclophi	XP_001649729.1peptidyl-prolyl cis-trans isomerase-like 3
AAEL02522	NA	0.110525391021881	0.483440564630384	0.847379421050056	NA	aag03018	NA	XP_021701681.1enhancer of mRNA-decapping protein 4 homolog isoform X2
AAEL01236	putative protein TPRXL	0.110676397091043	0.667902505824187	0.912418872165226	NA	NA	KXJ75412.1 hypothet	XP_021697657.1 TPRXL
AAEL01819	NA	0.110792151521697	0.527157239979007	0.865973876319045	NA	NA	NA	XP_021698491.1uncharacterized protein LOC5569599
AAEL00691	serine/threonine-protein phosphatase Pgam5, mitochondrial	0.110813581509768	0.586704964437483	0.885639613312618	NA	aag04137	XP_019532781.1 PRE	XP_001652326.1serine/threonine-protein phosphatase Pgam5, mitochondrial isoform X2
AAEL00276	homeobox protein prospero	0.110978194502291	0.634042865708501	0.902506916959539	NA	aag04013	XP_019526960.1 PRE	XP_021692953.1homeobox protein prospero isoform X1
AAEL00483	cyclin-T	0.11108841338872	0.458018753165901	0.833283524364043	NA	NA	XP_019544260.1 PRE	XP_021697729.1cyclin-T isoform X3
AAEL00687	mitochondrial folate transporter/carrier	0.111175757723547	0.312560576668901	0.744018629406202	NA	NA	XP_001862889.1 fola	XP_021695981.1mitochondrial folate transporter/carrier
AAEL01242	fatty acid hydroxylase domain-containing protein 2	0.111216423669287	0.50529352399919	0.858744381072989	NA	NA	KXJ76657.1 hypothet	XP_001662559.2fatty acid hydroxylase domain-containing protein 2
AAEL01190	beta-1,4-glucuronyltransferase 1	0.111225455842767	0.580083631501224	0.886401261871374	NA	NA	ETN58802.1 N-acetyl	XP_021703709.1beta-1,4-glucuronyltransferase 1
AAEL02406	NA	0.111233165519221	0.484090268502748	0.847655581875201	NA	NA	NA	XP_001654311.1immediate early response 3-interacting protein 1
AAEL00386	uncharacterized LOC5579115	0.11134008923927	0.497242529193135	0.856052403704179	NA	NA	KXJ80222.1 hypothet	XP_001657314.2uncharacterized protein LOC5579115
AAEL01224	uncharacterized LOC5576002	0.111473750679896	0.762131255829792	0.937006693824152	NA	NA	XP_001845024.1 con	XP_019541344.1 THAP domain-containing protein 1 B
AAEL00661	zinc finger protein 37 homolog	0.111533989862571	0.640740102203618	0.904492244035828	NA	NA	KXJ82689.1 hypothet	XP_021703921.1zinc finger protein 37 homolog
AAEL02671	NA	0.111560310146154	0.488004449972825	0.848846834445739	NA	aag03018	NA	XP_021711453.1PAB-dependent poly(A)-specific ribonuclease subunit PAN2
AAEL01163	protein TEX261	0.111639439407125	0.524606129233818	0.865595409935377	NA	NA	ETN66050.1 hypothe	XP_021708673.1protein TEX261
AAEL00230	tryptase	0.111725491025974	0.325990894556491	0.748896673349414	NA	NA	XP_019531493.1 PRE	XP_001661225.1tryptase isoform X1
AAEL00660	epsilon-sarcoglycan	0.111822402944203	0.642188084413939	0.905763856274616	NA	NA	XP_019526240.1 PRE	XP_021703910.1epsilon-sarcoglycan isoform X2
AAEL02084	NA	0.111856114029849	0.692499588202569	0.916122517126697	NA	NA	NA	XP_021694183.1zinc finger protein 271
AAEL01828	NA	0.11190449451805	0.632826685316364	0.902506916959539	NA	NA	NA	XP_021697299.1zinc finger protein 93 isoform X2
AAEL00063	ceramide glucosyltransferase	0.111970617315976	0.4927529971678	0.852158101574964	600	aag01100 ; aag	KFB50159.1 hypothe	XP_021710513.1ceramide glucosyltransferase
AAEL02371	NA	0.111994676814946	0.298489191550024	0.730438157844279	NA	NA	NA	XP_001663402.2probable ATP-dependent RNA helicase spindle-E
AAEL02553	NA	0.11202824422404	0.477861699986947	0.845011493487133	NA	NA	NA	XP_021713150.1tubulin--tyrosine ligase-like protein 12

AAEL01805	NA	0.112029550672545	0.569334503427507	0.880635379772131	NA	NA	NA	XP_021704473.1zinc finger protein 236 isoform X1
AAEL00224	zinc finger and BTB domain-containing protein 17	0.112061689009222	0.591431630224577	0.888365874849728	NA	NA	KXJ70502.1 hypothet	XP_021696096.1zinc finger and BTB domain-containing protein 17
AAEL01464	SOSS complex subunit B homolog	0.112098282470424	0.655604791816558	0.908328180248866	NA	NA	NA	XP_001649250.2SOSS complex subunit B homolog
AAEL00029	zinc finger protein 691	0.112142335642567	0.78864285671274	0.945232821987723	NA	NA	KXJ74458.1 hypothet	XP_021709400.1zinc finger protein 691
AAEL00868	E3 ubiquitin-protein ligase Topors	0.11220831178724	0.482214459720224	0.846941547268585	NA	NA	KXJ83825.1 hypothet	XP_001653408.1E3 ubiquitin-protein ligase Topors
AAEL01720	transmembrane 9 superfamily member 2	0.112332225182227	0.297095860076566	0.729422653261622	NA	NA	NA	XP_011493097.1transmembrane 9 superfamily member 2
AAEL00839	endoplasmic reticulum-Golgi intermediate compartment protein 3	0.112359155302516	0.635593225264186	0.902538767814767	NA	NA	KXJ71061.1 hypothet	XP_001653244.2endoplasmic reticulum-Golgi intermediate compartment protein 3
AAEL02739	NA	0.112416002010358	0.556084996045393	0.876514925250035	NA	NA	NA	XP_001656158.2uncharacterized protein LOC5576584
AAEL01509	glutamate receptor-interacting protein 2	0.112465758991969	0.675528642482894	0.914438668430353	NA	NA	NA	XP_021694871.1glutamate receptor-interacting protein 2
AAEL00451	uncharacterized LOC5564977	0.112550530993939	0.631641537951896	0.902506916959539	NA	NA	KXJ78986.1 hypothet	XP_021701378.1uncharacterized protein LOC5564977
AAEL00017	uncharacterized protein CG3556	0.112644307054355	0.649877155961778	0.90663577761678	NA	NA	KXJ76978.1 hypothet	XP_021706045.1uncharacterized protein CG3556
AAEL01964	NA	0.112691078173187	0.345645067328904	0.764941296190615	NA	NA	NA	XP_021700762.1ATP-binding cassette sub-family G member 1 isoform X1
AAEL02561	NA	0.112810328155805	0.543375178080435	0.869900911343623	NA	NA	NA	XP_021699028.1uncharacterized protein LOC110676240
AAEL02324	NA	0.112897203282114	0.425991419378885	0.814623464645531	NA	NA	NA	XP_001656303.1Golgi to ER traffic protein 4 homolog
AAEL00131	ATP-dependent RNA helicase DDX42	0.112965668291025	0.488355190754145	0.848846834445739	NA	aag03040	KXJ79862.1 hypothet	XP_001653010.1ATP-dependent RNA helicase DDX42
AAEL01433	protein phosphatase 1H	0.112971909495122	0.379384564499148	0.785666748907449	04660 ; 04658	NA	NA	XP_001648484.2protein phosphatase 1H
AAEL01731	DNA-directed RNA polymerases I, II, and III subunit RPABC4	0.112972010320368	0.422913781569183	0.814192545906728	NA	aag03020	NA	XP_001844548.1RNA polymerase small Zn-binding subunit
AAEL01013	leucine-rich repeat-containing protein 15	0.113151677710862	0.543252671230551	0.869900911343623	NA	NA	NA	XP_001660653.2leucine-rich repeat-containing protein 15
AAEL00209	DNA repair endonuclease XPF	0.113288469164505	0.567473469331277	0.880302387766415	NA	aag03420 ; aag	XP_001845955.1 me	XP_021706205.1DNA repair endonuclease XPF
AAEL00633	sulfotransferase family cytosolic 1B member 1	0.113462346791763	0.643002110559917	0.905972350539329	NA	NA	ETN60369.1 sulfotra	XP_001651919.1sulfotransferase family cytosolic 1B member 1
AAEL01402	transmembrane emp24 domain-containing protein 5	0.113472132048491	0.482644723030921	0.847090738380801	NA	NA	NA	XP_001664248.2transmembrane emp24 domain-containing protein 5
AAEL00800	Golgi reassembly-stacking protein 2	0.113493119497232	0.502544518616766	0.858744381072989	NA	NA	KXJ69951.1 hypothet	XP_001658795.2Golgi reassembly-stacking protein 2
AAEL02277	NA	0.113522277736083	0.593288485630111	0.888529762455765	NA	NA	NA	NA
AAEL01972	NA	0.113629138813908	0.35757182286253	0.773736338358957	NA	NA	NA	XP_021701902.1acyl-CoA synthetase family member 2, mitochondrial isoform X1
AAEL01318	putative ATP-dependent RNA helicase DHX57	0.113672496280806	0.403210095889373	0.800159435491484	NA	NA	NA	XP_001663363.1putative ATP-dependent RNA helicase DHX57
AAEL01463	rho-related BTB domain-containing protein 1	0.113793356394763	0.38225715518722	0.788330421791305	NA	aag04120	NA	XP_021697937.1rho-related BTB domain-containing protein 1 isoform X4
AAEL00869	mothers against decapentaplegic homolog 3	0.113881739562404	0.396242370090952	0.796693358396638	NA	aag04144 ; aag	ETN61574.1 smad [A	XP_001659425.2mothers against decapentaplegic homolog 3
AAEL01036	cytosolic Fe-S cluster assembly factor NUBP1 homolog	0.113888473259327	0.567671633232548	0.880302387766415	NA	NA	NA	XP_021707203.1cytosolic Fe-S cluster assembly factor NUBP1 homolog
AAEL00366	leukotriene A-4 hydrolase	0.113926433851073	0.504487400131946	0.858744381072989	NA	aag01100 ; aag	XP_019526546.1 PRE	XP_021701450.1leukotriene A-4 hydrolase isoform X1
AAEL01137	5-demethoxyubiquinone hydroxylase, mitochondrial	0.113971667732631	0.470555450914702	0.841282642598396	NA	aag01100 ; aag	KXJ79674.1 hypothet	XP_011493599.15-demethoxyubiquinone hydroxylase, mitochondrial
AAEL01372	uncharacterized LOC5578517	0.114062287775512	0.453619529021617	0.832617462606238	NA	NA	NA	XP_021713311.1uncharacterized protein LOC5578517
AAEL01130	rRNA methyltransferase 2, mitochondrial	0.114065983558122	0.690687525837974	0.916122517126697	NA	NA	XP_001843114.1 ribo	XP_001655238.1rRNA methyltransferase 2, mitochondrial
AAEL01178	peroxisomal biogenesis factor 3	0.114091324631595	0.505392627045948	0.858744381072989	NA	aag04146	KFB35048.1 AGAP00	XP_001661904.1peroxisomal biogenesis factor 3
AAEL00716	E3 ubiquitin-protein ligase RNF4	0.114095294240846	0.755895899459868	0.934127170333935	NA	NA	KXJ72271.1 hypothet	XP_001652570.1E3 ubiquitin-protein ligase RNF4
AAEL02780	NA	0.114220337580446	0.528647023790785	0.865973876319045	NA	NA	NA	XP_021696022.1zinc finger Y-chromosomal protein isoform X2
AAEL00070	KRR1 small subunit processome component homolog	0.114358902499553	0.492577731533047	0.852158101574964	NA	NA	KXJ79854.1 hypothet	XP_001650245.1KRR1 small subunit processome component homolog
AAEL00725	ATP-dependent DNA helicase Q1	0.114415115507125	0.396436567628493	0.796856552057098	NA	NA	KXJ76945.1 hypothet	XP_001652627.2ATP-dependent DNA helicase Q1
AAEL00507	dnaJ homolog subfamily C member 8	0.114536504419085	0.569537490588425	0.880635379772131	NA	NA	KFB42715.1 hypothe	XP_001650318.1dnaJ homolog subfamily C member 8
AAEL00430	protein NDRG3	0.114598907033492	0.306973194776207	0.739080936245593	NA	NA	XP_019558684.1 PRE	XP_001648890.1protein NDRG3 isoform X5
AAEL00174	JNK-interacting protein 1	0.114800810705162	0.633075382549104	0.902506916959539	NA	NA	KXJ81888.1 hypothet	XP_021696287.1JNK-interacting protein 1
AAEL00710	polyserase-2	0.114836447237389	0.364681994488429	0.778761496713185	NA	NA	XP_001864286.1 coa	XP_019563561.1 polyserase-2-like
AAEL00029	staphylococcal nuclease domain-containing protein 1	0.114944842047312	0.312981095622171	0.744189298893767	NA	NA	KXJ70956.1 hypothet	XP_001654799.1staphylococcal nuclease domain-containing protein 1
AAEL02450	NA	0.115100018369897	0.379426901483053	0.785666748907449	NA	NA	NA	XP_021698114.1coatomer subunit epsilon
AAEL02235	NA	0.115117770747418	0.773174392253364	0.941230906670349	NA	NA	NA	XP_021696872.1uncharacterized protein LOC110675637
AAEL00392	protein MON2 homolog	0.115247516792696	0.449424155889769	0.829306073695304	NA	NA	KXJ75821.1 hypothet	XP_021702026.1protein MON2 homolog
AAEL00635	general transcription factor IIH subunit 4	0.115515394638363	0.551866003775506	0.874531710113044	NA	aag03420 ; aag	KXJ69446.1 hypothet	XP_001651923.1general transcription factor IIH subunit 4
AAEL00413	mediator of RNA polymerase II transcription subunit 31	0.115516923128589	0.52575600249359	0.865680248818465	NA	NA	XP_019531128.1 PRE	XP_011493225.2mediator of RNA polymerase II transcription subunit 31 isoform X2
AAEL00088	BAG domain-containing protein Samui	0.115517305680798	0.416765892540042	0.81148534556781	NA	NA	KXJ68901.1 hypothet	XP_001651673.1BAG domain-containing protein Samui
AAEL00229	copper transporter 5.1	0.115632740660754	0.384210499812105	0.790063144962496	NA	NA	KXJ81682.1 hypothet	XP_001661239.1copper transporter 5.1 isoform X1
AAEL00022	mediator of RNA polymerase II transcription subunit 9	0.115645786852962	0.283974338835005	0.716319042203869	NA	NA	NA	XP_001660146.2uncharacterized protein NCBP2-AS2 homolog isoform X2
AAEL00000	bystin	0.115680159312508	0.449873583320278	0.829440335346263	NA	NA	KXJ70378.1 hypothet	XP_019528897.1 bystin
AAEL01485	mediator of RNA polymerase II transcription subunit 14	0.115744042298935	0.517394428608503	0.861881144752444	NA	NA	NA	XP_021697618.1mediator of RNA polymerase II transcription subunit 14 isoform X1
AAEL02474	NA	0.115904429762423	0.72828349749414	0.923566968708516	NA	NA	NA	XP_021712453.1probable electron transfer flavoprotein subunit alpha, mitochondrial
AAEL00034	class E basic helix-loop-helix protein 23	0.115964520756902	0.774415514286197	0.941230906670349	NA	NA	XP_001842466.1 bas	XP_021702298.1class E basic helix-loop-helix protein 23
AAEL00349	ribonuclease kappa	0.116022544307905	0.33753274996454	0.759630496362757	NA	NA	ETN66636.1 salivary	XP_001663793.1ribonuclease kappa
AAEL00085	uncharacterized LOC5566893	0.116046621411351	0.653652320773969	0.907357713262968	NA	NA	XP_001864697.1 con	XP_001651306.2uncharacterized protein LOC5566893
AAEL00657	serine/threonine-protein kinase pelle	0.116047136025128	0.580194860840888	0.882796907080865	04151 ; 05165 ; 04714 ;	aag04624	KXJ82174.1 hypothet	XP_021704321.1serine/threonine-protein kinase pelle
AAEL00410	nucleoside diphosphate kinase 6	0.116121491244034	0.526214513087544	0.865680248818465	00240 ; 00983 ; 00230	aag01100 ; aag	KXJ83369.1 hypothet	XP_001648448.1nucleoside diphosphate kinase 6
AAEL00809	mitochondrial ornithine transporter 1	0.116241351209588	0.53468442843066	0.866700945213732	NA	NA	KXJ71598.1 hypothet	XP_001653099.1mitochondrial ornithine transporter 1
AAEL01313	DNA-directed RNA polymerase I subunit RPA1	0.116256828820421	0.484703225340234	0.847655581875201	NA	aag03020	NA	XP_021706774.1DNA-directed RNA polymerase I subunit RPA1
AAEL02046	NA	0.116284641221775	0.638526436171988	0.90377215326912	NA	aag04013	NA	XP_021707149.1docking protein 2
AAEL01196	RRP12-like protein	0.116294561368087	0.30443974377246	0.737814869687351	NA	NA	ETN63296.1 RRP12 p	XP_001655773.1RRP12-like protein
AAEL01218	chromatin modification-related protein MEAF6	0.116314198705968	0.62037234874969	0.897385728224103	NA	NA	KXJ80879.1 hypothet	XP_001662295.2chromatin modification-related protein MEAF6
AAEL02621	NA	0.116325607613927	0.603040529491659	0.892951506950708	NA	NA	NA	XP_011493320.1heat shock 70 kDa protein cognate 3
AAEL00773	syntaxin-7	0.116729943852846	0.289045864936835	0.721217831038796	NA	aag04145 ; aag	XP_001865762.1 Pep	XP_019546969.1 syntaxin-7
AAEL00422	myosin heavy chain 95F	0.116848514851867	0.285848449082657	0.717970179170389	NA	NA	XP_019530392.1 PRE	XP_021700076.1myosin heavy chain 95F isoform X1
AAEL00606	protein neuralized	0.116905168300645	0.61142861687736	0.894646314231935	NA	NA	KXJ74740.1 hypothet	XP_021704555.1protein neuralized isoform X2
AAEL01829	NA	0.116954024181671	0.681700886482552	0.914629465876247	NA	NA	NA	XP_021705914.1neuroligin-4, Y-linked
AAEL00389	DNA repair protein complementing XP-C cells homolog	0.117030924759385	0.693876504479186	0.916122517126697	NA	NA	KXJ68158.1 hypothet	XP_001664270.2DNA repair protein complementing XP-C cells homolog
AAEL00370	NA	0.117044155360453	0.604029060716137	0.893547770777888	NA	NA	XP_001867395.1 acti	XP_001657135.1 actin-binding protein IPP
AAEL01175	cell division cycle and apoptosis regulator protein 1-like	0.117069818357348	0.652912449390587	0.907272485895905	NA	NA	KXJ73619.1 hypothet	XP_021712234.1cell division cycle and apoptosis regulator protein 1-like
AAEL02095	NA	0.117170343923524	0.544344604691118	0.870383849238518	NA	NA	NA	XP_021708968.1zinc finger CCCH domain-containing protein 13 isoform X1
AAEL01030	zinc finger and SCAN domain-containing protein 31	0.117218347182484	0.565377407667438	0.880302387766415	NA	NA	NA	XP_001654448.1zinc finger and SCAN domain-containing protein 31
AAEL00103	pre-rRNA processing protein FTSJ3	0.117297520319148	0.283750096425675	0.716146019558164	NA	NA	ETN58486.1 ribosom	XP_001657887.1pre-rRNA processing protein FTSJ3
AAEL02581	NA	0.117322929222202	0.600884166319941	0.892093596129407	NA	NA	NA	XP_001653102.2glycosaminoglycan xylosylkinase homolog
AAEL01286	uncharacterized LOC5576915	0.11758434356844	0.536023055652466	0.867783982778675	NA	NA	NA	XP_001663012.2uncharacterized protein LOC5576915
AAEL00304	DNA translocase FtsK	0.117760047515249	0.753824247984583	0.933480114379509	NA	NA	KXJ68908.1 hypothet	XP_019555472.1 pupal cuticle protein 27-like
AAEL01341	transmembrane protein 70 homolog, mitochondrial	0.117841129032444	0.415670460283622	0.80969508372775	NA	NA	NA	XP_001663601.1transmembrane protein 70 homolog, mitochondrial
AAEL01095	uncharacterized protein C9orf85 homolog	0.117846447403111	0.344388730949288	0.764706052029763	NA	NA	ETN60610.1 hypothe	KFB43515.1AGAP003732-like protein
AAEL00382	SH3 domain-binding protein 5 homolog	0.118076422962119	0.413514746697105	0.808061732662297	NA	NA	XP_019527086.1 PRE	XP_001657297.2SH3 domain-binding protein 5 homolog isoform X2
AAEL02117	NA	0.118108337128355	0.38421349420856	0.790063144962496	NA	aag04150	NA	XP_021699205.1ragulator complex protein LAMTOR4 homolog
AAEL00779	protein BUD31 homolog	0.118156880929559	0.636053456778827	0.902538767814767	NA	aag03040	ETN62571.1 cell cycle	XP_001658667.1protein BUD31 homolog

AAEL0018З	DNA-directed RNA polymerase II subunit RPB9	0.118167622697384	0.473276910299823	0.843301826204891	NA	aag03020	ETN66377.1 DNA-dir	XP_001660391.1DNA-directed RNA polymerase II subunit RPB9
AAEL0006ǀ	DNA replication complex GINS protein PSF1	0.118197880647349	0.6733809463045	0.912968243848131	NA	NA	KFB45715.1 AGAP01	XP_001650268.1DNA replication complex GINS protein PSF1
AAEL0099ŀ	pre-mRNA-processing-splicing factor 8	0.118224550305038	0.293601996079277	0.724246906570544	NA	aag03040	NA	XP_001660510.1pre-mRNA-processing-splicing factor 8
AAEL0036ŀ	phospholipase D3	0.118237230106816	0.475667925040345	0.844836179544731	NA	aag01100 ; aag	XP_001867389.1 pho	XP_001657144.2phospholipase D3
AAEL0024ŀ	GPI ethanolamine phosphate transferase 3	0.118305768341404	0.417139652238017	0.811697838844202	NA	aag01100 ; aag	KXJ79255.1 hypothet	XP_021697011.1GPI ethanolamine phosphate transferase 3
AAEL0072ŀ	transcription factor E2F5	0.118355112335856	0.659613614456362	0.909580313002131	NA	aag04350	KXJ68782.1 hypothet	XP_001658273.1transcription factor E2F5
AAEL0098ŀ	protein I'm not dead yet	0.118383196826281	0.30535821481158	0.738299809812121	NA	NA	NA	XP_001654053.1protein I'm not dead yet
AAEL0138ŀ	uncharacterized LOC5578828	0.118556992203859	0.593425764256069	0.888529762455765	NA	NA	NA	XP_001664045.2uncharacterized protein LOC5578828
AAEL0007ŀ	uncharacterized LOC5566279	0.11859622642263	0.681311158191695	0.914629465876247	NA	NA	KXJ74349.1 hypothet	XP_021696554.1uncharacterized protein LOC5566279
AAEL0104ŀ	thioredoxin domain-containing protein 9	0.118641875598094	0.340635163011345	0.761546348403887	NA	NA	NA	XP_021693607.1thioredoxin domain-containing protein 9
AAEL0106ŀ	sodium- and chloride-dependent GABA transporter 1	0.118645889088427	0.453468378066725	0.832617462606238	NA	NA	KXJ82007.1 hypothet	XP_001661017.2sodium- and chloride-dependent GABA transporter 1
AAEL0043ŀ	E3 ubiquitin-protein ligase arkadia-B	0.118836864574849	0.463055772545347	0.837264939413983	NA	NA	XP_001866274.1 con	XP_001649008.2E3 ubiquitin-protein ligase arkadia-B
AAEL0082ŀ	diuretic hormone receptor	0.118862055418503	0.64702408720937	0.906554905014882	NA	NA	NA	XP_001659109.2diuretic hormone receptor isoform X2
AAEL0055ŀ	lysophospholipid acyltransferase 5	0.118977890881571	0.371413411086033	0.781817769662763	NA	aag00564	ETN65406.1 c3f [Anc	XP_001651111.1lysophospholipid acyltransferase 5
AAEL0140ŀ	GATOR complex protein MIOS	0.119066096079788	0.619489528538408	0.897318336565571	NA	aag04150	NA	XP_001664223.1GATOR complex protein MIOS
AAEL0101ŀ	protein cornichon homolog 4	0.119153840956903	0.596788025803716	0.889767241323038	NA	NA	NA	XP_001660639.1protein cornichon homolog 4
AAEL0134ŀ	NA	0.119163388517992	0.548936332105314	0.873169431463929	NA	NA	NA	XP_001663654.1neuralized-like protein 2
AAEL0091ŀ	uncharacterized LOC5571570	0.119239006969446	0.609147668830617	0.893547770777888	NA	NA	KXJ68251.1 hypothet	XP_021701217.1uncharacterized protein LOC5571570
AAEL0125ŀ	E3 ubiquitin-protein ligase TRIM33	0.11934186264788	0.415134998207705	0.809508847956623	NA	NA	KXJ75495.1 hypothet	XP_001656144.1E3 ubiquitin-protein ligase TRIM33
AAEL0002ŀ	protein mahjong	0.119364780612307	0.351995762187789	0.769212685559608	NA	NA	NA	XP_021708370.1protein mahjong isoform X2
AAEL0029ŀ	uncharacterized LOC5576708	0.119370847901723	0.553035360861173	0.874531710113044	NA	NA	KXJ69656.1 hypothet	XP_001656214.2uncharacterized protein LOC5576708
AAEL0067ŀ	mitochondrial inner membrane protein OXA1L	0.119732240141525	0.407268482790877	0.803535299237735	NA	aag03060	KFB48215.1 AGAP00	XP_001652200.1mitochondrial inner membrane protein OXA1L
AAEL0030ŀ	autophagy-related protein 101	0.11975034061693	0.338827514953635	0.7608323066382	NA	NA	XP_019542151.1 PRE	XP_001662977.2serine/threonine-protein kinase S6KL isoform X1
AAEL0225ŀ	NA	0.119769349787901	0.748757822835577	0.932554085844433	NA	NA	NA	XP_021694331.1lipase 3-like
AAEL0084ŀ	uncharacterized LOC5570621	0.119930782247733	0.6243984784419	0.898970345267764	NA	NA	XP_019562336.1 PRE	XP_019562336.1 zinc finger protein ZIC 2-like isoform X2
AAEL0005ŀ	mitochondrial fission process protein 1	0.119944132095587	0.523034877914325	0.865215478807089	NA	NA	ETN57986.1 mitocho	XP_001648677.1mitochondrial fission process protein 1
AAEL0116ŀ	NA	0.11995991867782	0.314839333483558	0.745407435136825	NA	NA	KXJ72209.1 hypothet	XP_001655624.2WD repeat-containing protein 74
AAEL0262ŀ	NA	0.120003555824505	0.583150182878298	0.884918862606546	NA	NA	NA	XP_021706177.1zinc finger protein 768-like isoform X2
AAEL0048ŀ	zinc finger protein 82 homolog	0.120009458152079	0.563909430653687	0.879838896781538	NA	NA	KXJ70197.1 hypothet	XP_021693798.1zinc finger protein 82 homolog
AAEL0129ŀ	MRG/MORF4L-binding protein	0.120016844158392	0.545129676591981	0.870466764449305	NA	NA	NA	XP_001663179.2MRG/MORF4L-binding protein
AAEL0047ŀ	para-nitrobenzyl esterase	0.120149510447965	0.74314092139444	0.930265645309704	NA	NA	KXJ76131.1 hypothet	XP_019549561.1 esterase B1-like isoform X1
AAEL0134ŀ	biogenesis of lysosome-related organelles complex 1 subunit 4	0.120191048383859	0.600704738865997	0.892093596129407	NA	NA	NA	XP_001663658.2biogenesis of lysosome-related organelles complex 1 subunit 4
AAEL0036ŀ	PIN2/TERF1-interacting telomerase inhibitor 1	0.120214887785054	0.299302778508054	0.730496185008314	NA	NA	XP_019526573.1 PRE	XP_001657164.2PIN2/TERF1-interacting telomerase inhibitor 1 isoform X1
AAEL0033ŀ	probable RNA polymerase II nuclear localization protein SLC7A6OS	0.120220202549492	0.615008670745077	0.896845306410123	NA	NA	XP_001847835.1 con	XP_001656686.2probable RNA polymerase II nuclear localization protein SLC7A6OS
AAEL0063ŀ	vesicle transport protein SEC20	0.120351256681845	0.62879794437174	0.901045385683245	NA	aag04130	XP_001846938.1 con	XP_001651963.2vesicle transport protein SEC20
AAEL0051ŀ	venom carboxylesterase-6	0.120486390303524	0.346701151103395	0.765398366276275	NA	NA	KXJ81529.1 hypothet	XP_001650475.2venom carboxylesterase-6
AAEL0013ŀ	anaphase-promoting complex subunit 15	0.120573630795659	0.447601566512857	0.828571638513641	NA	NA	ETN63356.1 hypothe	XP_001653076.1anaphase-promoting complex subunit 15
AAEL0053ŀ	U6 snRNA-associated Sm-like protein LSm4	0.120595143339045	0.455331009384359	0.832617462606238	NA	aag03040 ; aag	ETN59535.1 small nu	XP_001650762.1U6 snRNA-associated Sm-like protein LSm4
AAEL0045ŀ	NA	0.12067134215649	0.713830593224396	0.919466561989206	NA	NA	XP_001864486.1 con	XP_021696153.1uncharacterized protein LOC5565109
AAEL0011ŀ	transmembrane protein 62	0.120752825432397	0.500894392567911	0.858744381072989	NA	NA	KXJ76277.1 hypothet	XP_001658187.1transmembrane protein 62 isoform X2
AAEL0124ŀ	TIP41-like protein	0.120903071661124	0.468098926490769	0.839644234660078	NA	NA	KXJ67859.1 hypothet	XP_001662575.2TIP41-like protein
AAEL0035ŀ	zinc finger and BTB domain-containing protein 17	0.120906479141314	0.657122385799116	0.909239282344833	NA	NA	KXJ80499.1 hypothet	XP_021700522.1zinc finger and BTB domain-containing protein 17
AAEL0039ŀ	hydroxylysine kinase	0.120979021594482	0.364753537365119	0.778761496713185	NA	aag01100 ; aag	KXJ75081.1 hypothet	XP_001647991.1hydroxylysine kinase
AAEL0054ŀ	tyrosine-protein phosphatase non-receptor type 9	0.121093552442104	0.300173662185569	0.73190197123254	NA	NA	XP_019540403.1 PRE	XP_021706716.1tyrosine-protein phosphatase non-receptor type 9 isoform X2
AAEL0226ŀ	NA	0.121106310232002	0.539672073754068	0.869709828277711	NA	aag04013	NA	XP_021707815.1uncharacterized protein LOC110678777
AAEL0090ŀ	mediator of RNA polymerase II transcription subunit 24	0.121130651805422	0.56670471593767	0.880302387766415	NA	NA	XP_001866892.1 thy	XP_001653641.1mediator of RNA polymerase II transcription subunit 24
AAEL0052ŀ	huntingtin	0.121226737446403	0.430896099169152	0.819575424670223	NA	NA	KFB36381.1 AGAP00	XP_019564080.1 huntingtin-like
AAEL0015ŀ	dnaJ homolog subfamily C member 5 homolog	0.121229005421592	0.28706548046413	0.718902142868179	NA	aag04141	XP_019553897.1 PRE	XP_001653560.1dnaJ homolog subfamily C member 5 homolog isoform X6
AAEL0037ŀ	39S ribosomal protein L13, mitochondrial	0.121340714072096	0.46546286133414	0.838483018891546	NA	aag03010	ETN67322.1 39S ribo	XP_001657133.139S ribosomal protein L13, mitochondrial
AAEL0077ŀ	eukaryotic translation initiation factor 3 subunit B	0.121513940940287	0.325560198945454	0.748896673349414	NA	aag03013	XP_001843846.1 euk	XP_001652847.2eukaryotic translation initiation factor 3 subunit B
AAEL0116ŀ	homeobox protein homothorax	0.121722536116713	0.372937687313724	0.781903075566391	NA	NA	NP_001027170.1 hor	XP_019533867.1 homeobox protein homothorax-like
AAEL0056ŀ	uncharacterized LOC5566746	0.121758117661729	0.47199276341608	0.842604161062625	NA	NA	KXJ71187.1 hypothet	XP_021695515.1uncharacterized protein LOC5566746
AAEL0085ŀ	uncharacterized LOC5570779	0.121926813571384	0.403081644837318	0.800077069843183	NA	NA	ETN62088.1 hypothe	KFB41504.1AGAP002439-like protein
AAEL0069ŀ	UPF0528 protein CG10038	0.121962316927809	0.34667675088728	0.765398366276275	NA	NA	NA	XP_001652331.2UPF0528 protein CG10038 isoform X2
AAEL0029ŀ	splicing factor, arginine/serine-rich 15	0.122039641046764	0.321300997399914	0.746588710355934	NA	NA	NA	XP_021708193.1protein SCAF8 isoform X2
AAEL0061ŀ	tRNA (cytosine(38)-C(5))-methyltransferase	0.122168564636951	0.555956685227889	0.876514925250035	270	NA	KXJ78955.1 hypothet	XP_001657555.2tRNA (cytosine(38)-C(5))-methyltransferase
AAEL0070ŀ	general transcription factor IIH subunit 2	0.122418773692117	0.64702408723493	0.906554905014882	NA	aag03420 ; aag	KFB38620.1 hypothe	XP_001652513.1general transcription factor IIH subunit 2
AAEL0225ŀ	NA	0.122420868481751	0.645970445272273	0.906554905014882	NA	NA	NA	XP_021707901.1probable cytochrome P450 313a4
AAEL0214ŀ	NA	0.122488313219463	0.735041752125036	0.926618579210021	NA	NA	NA	XP_021699137.1uncharacterized protein LOC110676261
AAEL0127ŀ	aminopeptidase N	0.122531126116269	0.400625285116701	0.798648539435638	480	aag01100 ; aag	NA	XP_001662888.1aminopeptidase N
AAEL0111ŀ	14-3-3 protein epsilon	0.122554095895942	0.3118890778865	0.743490640759055	NA	aag04391	ETN59683.1 14-3-3 p	XP_001655111.114-3-3 protein epsilon
AAEL0196ŀ	NA	0.122573451932878	0.318416531708601	0.745684464362801	NA	NA	NA	XP_021699476.1Y-box factor homolog
AAEL0044ŀ	protein SMG9	0.122585688340012	0.67306951471153	0.912968243848131	NA	NA	XP_019555239.1 PRE	XP_001649122.1protein SMG9 isoform X4
AAEL0078ŀ	TBC1 domain family member 15	0.122993065006314	0.335522939268093	0.757775574591595	NA	aag04137	KXJ74651.1 hypothet	XP_001658686.2TBC1 domain family member 15
AAEL0264ŀ	NA	0.123021809557541	0.559665751701334	0.877581080322399	NA	NA	NA	XP_021701153.1protein arginine N-methyltransferase 9-like
AAEL0194ŀ	NA	0.123185372003994	0.320287162574677	0.74633640668832	NA	NA	NA	CRL02246.1CLUMA_CG015133, isoform A
AAEL0049ŀ	uncharacterized LOC5565743	0.123201162191613	0.479955715607554	0.845454613425911	NA	NA	KXJ73306.1 hypothet	XP_019560326.1 neuronal acetylcholine receptor subunit alpha-9-like isoform X1
AAEL0257ŀ	NA	0.123359982435149	0.423463888049533	0.814192545906728	00561 ; 00073	NA	NA	XP_021700504.1uncharacterized protein LOC110676540
AAEL0090ŀ	membrane-bound alkaline phosphatase	0.123441092090191	0.692683816185289	0.916122517126697	00790 ; 00730	NA	KXJ74098.1 hypothet	XP_021695477.1membrane-bound alkaline phosphatase
AAEL0105ŀ	transitional endoplasmic reticulum ATPase TER94	0.123454984936415	0.323691547262744	0.74762391301976	NA	aag04141	KXJ76614.1 hypothet	XP_001654680.1transitional endoplasmic reticulum ATPase TER94
AAEL0067ŀ	uncharacterized LOC5568315	0.123618187511152	0.64261138527317	0.905972350539329	NA	NA	KXJ82072.1 hypothet	XP_001652192.2uncharacterized protein LOC5568315
AAEL0075ŀ	GATA zinc finger domain-containing protein 10	0.123671598926573	0.426079410491083	0.814623464645531	NA	NA	KXJ78548.1 hypothet	XP_001658429.1GATA zinc finger domain-containing protein 10
AAEL0142ŀ	motile sperm domain-containing protein 2	0.123866877533653	0.283628327494369	0.716035080823918	NA	NA	NA	XP_001648403.1motile sperm domain-containing protein 2
AAEL0106ŀ	farnesol dehydrogenase-like	0.123962026036512	0.664023598618623	0.911592424421865	NA	NA	KXJ71182.1 hypothet	XP_001661002.2farnesol dehydrogenase-like
AAEL0053ŀ	hepatocyte growth factor-regulated tyrosine kinase substrate	0.124065568008448	0.411148860896539	0.806005926282478	NA	aag04144 ; aag	KXJ80561.1 hypothet	XP_001650711.1hepatocyte growth factor-regulated tyrosine kinase substrate isoform X1
AAEL0076ŀ	protein phosphatase 1 regulatory subunit 11	0.124128616257501	0.425121418724457	0.814623464645531	NA	NA	KXJ75986.1 hypothet	XP_001658586.1protein phosphatase 1 regulatory subunit 11
AAEL0056ŀ	NA	0.124201935727946	0.659134525828494	0.909580313002131	NA	NA	NA	XP_021698107.1uncharacterized protein LOC5566834
AAEL0078ŀ	serine/threonine-protein kinase fused	0.124273059777024	0.648329812920484	0.906554905014882	NA	aag04341	KXJ76696.1 hypothet	XP_001658684.2serine/threonine-protein kinase fused
AAEL0027ŀ	transport and Golgi organization protein 11	0.124323419147285	0.315311443768909	0.745627727379793	NA	NA	XP_019534588.1 PRE	XP_001662199.1transport and Golgi organization protein 11 isoform X1
AAEL0057ŀ	zinc finger MYND domain-containing protein 11	0.124433285096271	0.460189764780276	0.834252603527995	NA	NA	KXJ68557.1 hypothet	XP_001651370.2zinc finger MYND domain-containing protein 11
AAEL0093ŀ	coiled-coil domain-containing protein 22 homolog	0.124469409195588	0.532751744537917	0.866700945213732	NA	NA	NA	XP_001660026.1coiled-coil domain-containing protein 22 homolog
AAEL0131ŀ	WASH complex subunit 4	0.124479344550907	0.446230836035429	0.828189271329869	NA	aag04144	NA	XP_021704895.1WASH complex subunit 4

AAEL0128	stathmin-1-A	0.124596991033684	0.490300883152457	0.850081180289834	NA	NA	NA	XP_001662959.1trichoplein keratin filament-binding protein isoform X1
AAEL0099	uncharacterized LOC5572693	0.124705729936139	0.413360463611153	0.808061732662297	NA	NA	NA	XP_001660512.1uncharacterized protein LOC5572693
AAEL0132	uncharacterized LOC5570083	0.124832714942797	0.350716700221509	0.768176246660341	NA	NA	NA	XP_001653069.1uncharacterized protein LOC5570083
AAEL0198	NA	0.124854821675151	0.509038400994844	0.860083070787677	NA	NA	NA	XP_021709477.1uncharacterized protein LOC5575615
AAEL0124	transcriptional adapter 1	0.124879190077347	0.707971207540531	0.91797784850989	NA	NA	ETN58037.1 SPT3-as	XP_001662578.1transcriptional adapter 1
AAEL0029	NA	0.124919155609477	0.560983493762758	0.878368233725523	NA	aag04144	KXJ74841.1 hypothet	XP_021697350.1vacuolar protein sorting-associated protein 4B-like
AAEL0090	cholinesterase	0.124973921580771	0.527435360760383	0.865973876319045	NA	NA	XP_001842152.1 glio	XP_021700741.1 cholinesterase
AAEL0195	NA	0.125065428017387	0.30990056639155	0.742912550320373	310	aag01100 ; aag	NA	XP_021708257.1histone-lysine N-methyltransferase 2D isoform X5
AAEL0116	transmembrane protein 11 homolog, mitochondrial	0.125094573048677	0.273408088588519	0.708614567599462	NA	NA	XP_019557137.1 PRE	XP_021698849.1transmembrane protein 11 homolog, mitochondrial isoform X1
AAEL0149	26S proteasome non-ATPase regulatory subunit 13	0.125167838615797	0.259136500685508	0.691547675112889	NA	aag03050	NA	XP_001649990.226S proteasome non-ATPase regulatory subunit 13
AAEL0128	Hermansky-Pudlak syndrome 1 protein homolog	0.125241019360456	0.549005745115705	0.873169431463929	NA	NA	NA	XP_001656301.2Hermanskydlak syndrome 1 protein homolog
AAEL0044	death domain-associated protein 6	0.125264759267709	0.527828480715035	0.865973876319045	NA	NA	KXJ78140.1 hypothet	XP_001649274.2death domain-associated protein 6
AAEL0065	kelch domain-containing protein 3	0.125300469518763	0.338440891278212	0.760706663786543	NA	NA	KXJ76044.1 hypothet	XP_021703993.1kelch domain-containing protein 3
AAEL0182	NA	0.125325271675251	0.659752687612519	0.909580313002131	NA	NA	NA	XP_021699019.1zinc finger protein 33A isoform X1
AAEL0107	sperm-associated antigen 7	0.125427905619069	0.542478397711543	0.869900911343623	NA	NA	XP_001846071.1 con	XP_001654859.1sperm-associated antigen 7
AAEL0145	pentatricopeptide repeat-containing protein 2, mitochondrial	0.125439257506444	0.44008703398898	0.822798752521693	NA	NA	NA	XP_001649051.1pentatricopeptide repeat-containing protein 2, mitochondrial
AAEL0073	UPF0160 protein	0.125469990241031	0.391625067182076	0.793285976044306	NA	NA	KXJ71925.1 hypothet	XP_001658325.1UPF0160 protein
AAEL0132	uncharacterized LOC5570086	0.125537890608212	0.324073008512238	0.747806049390669	NA	NA	KXJ73795.1 hypothet	XP_001653073.1uncharacterized protein LOC5570086
AAEL0140	nucleoside diphosphate-linked moiety X motif 19	0.125544998816476	0.460413777645008	0.834457146359854	NA	aag04146	NA	XP_001664294.2nucleoside diphosphate-linked moiety X motif 19
AAEL0132	protein SDE2 homolog	0.125546756001496	0.584139106017497	0.885175928718656	NA	NA	NA	XP_001663425.2protein SDE2 homolog
AAEL0209	NA	0.125548176078241	0.475318991780396	0.84437941358363	NA	NA	NA	XP_021713105.1dentin sialophosphoprotein isoform X2
AAEL0107	ephrin type-B receptor 5	0.125719742017435	0.707839583744784	0.91797784850989	NA	NA	NA	XP_021694767.1ephrin type-B receptor 5 isoform X1
AAEL0069	uncharacterized LOC5568622	0.125760939553547	0.298847498220635	0.730450295042154	NA	NA	KXJ70489.1 hypothet	XP_001652456.1uncharacterized protein LOC5568622
AAEL0227	NA	0.125823355903523	0.258667321669696	0.691547675112889	NA	NA	NA	XP_019541710.1 akirin
AAEL0097	influenza virus NS1A-binding protein	0.125844040256995	0.258814000938026	0.691547675112889	NA	NA	NA	XP_001654000.2influenza virus NS1A-binding protein isoform X2
AAEL0043	uncharacterized LOC5564548	0.125857245046106	0.629954580460287	0.902091822190407	NA	NA	KXJ74716.1 hypothet	XP_001648905.1uncharacterized protein LOC5564548
AAEL0015	serine/threonine-protein kinase N	0.125889869195603	0.32376453959832	0.74762391301976	4070	NA	NA	XP_021695825.1serine/threonine-protein kinase N isoform X2
AAEL0205	NA	0.125899756177047	0.575781756625354	0.88173468538301	NA	NA	NA	XP_001659237.1septin-interacting protein 1
AAEL0040	zinc finger protein 883	0.125942720942788	0.448631380967641	0.829306073695304	NA	NA	KFB38161.1 AGAP00	XP_021705782.1zinc finger protein 883
AAEL0226	NA	0.126055240843982	0.62313653380393	0.898271200863489	NA	NA	NA	XP_021701586.1AF4/FMR2 family member 4 isoform X5
AAEL0031	NA	0.126060629153143	0.754593647794958	0.934085554225648	NA	NA	KXJ76620.1 hypothet	XP_001656526.1solute carrier family 22 member 5
AAEL0061	39S ribosomal protein L12, mitochondrial	0.126118330055487	0.330985580068344	0.754128446928769	NA	aag03010	KFB35064.1 hypothe	XP_001651798.139S ribosomal protein L12, mitochondrial
AAEL0077	vacuole membrane protein 1	0.12612510831331	0.291607518132523	0.722646576583698	NA	aag04140	XP_019537025.1 PRE	XP_001658647.1vacuole membrane protein 1 isoform X1
AAEL0182	NA	0.126138230826491	0.319406831873897	0.745684464362801	NA	NA	NA	XP_021700856.1alpha-protein kinase 1
AAEL0004	cathepsin O	0.126168838637702	0.530001705988781	0.866573507197719	NA	aag04142	KXJ81289.1 hypothet	XP_001656461.1cathepsin O
AAEL0018	carboxypeptidase B	0.126242851604833	0.361989346394877	0.776825085243856	NA	NA	NA	XP_001654129.2carboxypeptidase B
AAEL0123	proteasome-associated protein ECM29 homolog	0.126295371239743	0.395373935888109	0.796111807011461	NA	NA	XP_001870891.1 con	XP_001662429.2proteasome-associated protein ECM29 homolog
AAEL0128	NA	0.126451155819763	0.559688636483074	0.877581080322399	NA	NA	NA	XP_021706079.1latrophilin-like protein LAT-2
AAEL0132	F-box only protein 28	0.126507526664077	0.53856534524157	0.869670565558805	NA	NA	NA	XP_001663390.2F-box only protein 28
AAEL0016	probable aminopeptidase NPEPL1	0.126512167760446	0.448183290909497	0.829148098703094	480	NA	KXJ80721.1 hypothet	XP_001653737.2probable aminopeptidase NPEPL1
AAEL0029	uncharacterized LOC5576488	0.126553665343748	0.329561470684806	0.753059688565404	NA	NA	XP_019531897.1 PRE	ABF18404.1hypothetical conserved protein
AAEL0197	NA	0.126599557784602	0.718724643155609	0.921583569204186	NA	aag04150 ; aag	NA	XP_019933462.1 frizzled-like
AAEL0111	DDB1- and CUL4-associated factor 7	0.12669787645851	0.442447556996121	0.824760338905837	NA	NA	KFB41910.1 hypothe	XP_019533960.1 DDB1- and CUL4-associated factor 7 isoform X1
AAEL0212	NA	0.12677360872312	0.351548631668366	0.769127557920186	00550 ; 00510	aag01100 ; aag	NA	XP_021701732.1UDP-N-acetylglucosamine--dolichyl-phosphate N-acetylglucosaminephosphotransferase
AAEL0044	syntaxin-18	0.126805139005229	0.464212047080646	0.837274753622609	NA	aag04145 ; aag	KXJ69717.1 hypothet	XP_001649142.1syntaxin-18 isoform X1
AAEL0110	U2 small nuclear ribonucleoprotein auxiliary factor 35 kDa subunit-relate	0.12683566331578	0.455590360318341	0.832617462606238	NA	NA	KXJ83982.1 hypothet	XP_021700855.1U2 small nuclear ribonucleoprotein auxiliary factor 35 kDa subunit-related protein 2 isoform X1
AAEL0198	NA	0.126841230888173	0.349270466109654	0.767244408961813	NA	aag03008	NA	XP_019536538.1 midasin-like
AAEL0034	zinc finger protein 394	0.126998356109408	0.657027992280405	0.909239282344833	NA	NA	KXJ76570.1 hypothet	XP_001656841.2zinc finger protein 394
AAEL0005	RING finger protein 44	0.127079184170898	0.391253736829737	0.793285976044306	NA	NA	XP_019526927.1 PRE	XP_021709948.1RING finger protein 44 isoform X1
AAEL0013	uncharacterized LOC5570085	0.12715296502971	0.528597550893091	0.865973876319045	NA	NA	XP_001844466.1 con	XP_001653072.1uncharacterized protein LOC5570085
AAEL0195	NA	0.127228074282102	0.63056202132641	0.90233929580663	NA	NA	NA	XP_021703364.1sodium- and chloride-dependent glycine transporter 1
AAEL0138	mothers against decapentaplegic homolog 4	0.127265618352417	0.359051928755086	0.77388983997572	NA	aag04310 ; aag	NA	XP_021710320.1mothers against decapentaplegic homolog 4 isoform X3
AAEL0142	zinc finger protein 62 homolog	0.127514350495456	0.787642543263902	0.944774185961398	NA	NA	NA	XP_001648352.1zinc finger protein 62 homolog
AAEL0098	ATPase family AAA domain-containing protein 3A homolog	0.127583076028509	0.375458831356603	0.782198815065306	NA	NA	NA	XP_001654071.1ATPase family AAA domain-containing protein 3A homolog
AAEL0239	NA	0.127901242645896	0.368980920430274	0.781817769662763	NA	NA	NA	NA
AAEL0214	NA	0.127946834955909	0.350236992409165	0.768176246660341	NA	NA	NA	NA
AAEL0062	TP53-regulating kinase	0.128037390975377	0.488305254701832	0.848846834445739	04151 ; 05165 ; 04714 ;	NA	KXJ78775.1 hypothet	XP_001657589.2TP53-regulating kinase
AAEL0061	eukaryotic translation initiation factor 3 subunit K	0.128161251583213	0.283210989536279	0.715753918648043	NA	NA	B0XEA7.2 RecName:	XP_001651796.1eukaryotic translation initiation factor 3 subunit K
AAEL0075	dynactin subunit 5	0.128245516654305	0.531731054568657	0.866700945213732	NA	NA	KXJ68586.1 hypothet	XP_001658449.1dynactin subunit 5
AAEL0003	protein ultraspiracle	0.128271305849894	0.372419962187239	0.781817769662763	NA	aag04214	XP_019540060.1 PRE	XP_001656078.2protein ultraspiracle isoform X1
AAEL0015	uncharacterized LOC5571114	0.128287558717572	0.632522514271716	0.902506916959539	NA	NA	KXJ81649.1 hypothet	XP_021706642.1uncharacterized protein LOC5571114
AAEL0078	protein pelota	0.128546177826491	0.353150620551814	0.770161486404145	NA	aag03015	KFB45245.1 pelota [/	XP_021703871.1protein pelota
AAEL0146	nuclear transcription factor Y subunit beta	0.128561626962156	0.427986623822607	0.816404003402908	NA	NA	NA	XP_021702955.1rho GTPase-activating protein gacF isoform X1
AAEL0015	TBC1 domain family member 20	0.128619690944604	0.424862572472059	0.814623464645531	NA	NA	KXJ70365.1 hypothet	XP_001653390.1TBC1 domain family member 20
AAEL0276	NA	0.128747700068218	0.336426919024933	0.758445425575872	NA	NA	NA	XP_021708619.1protein pecanex isoform X1
AAEL0170	2-oxoisovalerate dehydrogenase subunit beta, mitochondrial	0.128755755191761	0.504279318365477	0.858744381072989	NA	aag01100 ; aag	NA	XP_021696447.12-oxoisovalerate dehydrogenase subunit beta, mitochondrial
AAEL0199	NA	0.128797043680304	0.691852609434049	0.916122517126697	NA	NA	NA	XP_021709824.1proton-coupled amino acid transporter-like protein CG1139 isoform X1
AAEL0016	endoribonuclease Dcr-1	0.128933520474583	0.603534304560156	0.893456028082779	NA	NA	XP_019558822.1 PRE	XP_001659747.2endoribonuclease Dcr-1
AAEL0011	putative inorganic phosphate cotransporter	0.129013076338433	0.287185859289657	0.718902142868179	NA	NA	KXJ80169.1 hypothet	XP_001652412.2putative inorganic phosphate cotransporter isoform X2
AAEL0023	pre-mRNA-splicing factor CWC25 homolog	0.129079445664355	0.509365130389205	0.860083070787677	NA	NA	KXJ71712.1 hypothet	XP_001655160.1pre-mRNA-splicing factor CWC25 homolog
AAEL0131	dnaJ homolog subfamily B member 12	0.129121555073539	0.247029702947375	0.680385311739523	NA	aag04141	NA	XP_001663382.1dnaJ homolog subfamily B member 12
AAEL0143	tetraspanin-18	0.12931286688081	0.352270518375918	0.769243785024963	NA	NA	NA	XP_019545962.1 tetraspanin-18-like
AAEL0046	50S ribosomal protein L1	0.129384903860949	0.432369929440785	0.820005280371151	NA	aag03010	KXJ81406.1 hypothet	XP_001649513.2uncharacterized protein LOC5565138
AAEL0147	E3 ubiquitin-protein ligase TRAIP	0.129386854527786	0.647627407284952	0.906554905014882	NA	NA	NA	XP_001649476.1E3 ubiquitin-protein ligase TRAIP
AAEL0221	NA	0.129484493891876	0.565053473921448	0.880302387766415	NA	NA	NA	NA
AAEL0022	autophagy protein 5	0.129489617487746	0.536528758988586	0.868144476914536	NA	aag04140 ; aag	KFB52402.1 AGAP01	XP_001661241.1autophagy protein 5
AAEL0247	NA	0.129490783020018	0.314906903457555	0.745407435136825	NA	NA	NA	XP_021695666.1E3 ubiquitin-protein ligase ZNF598
AAEL0107	titin homolog	0.129545757117897	0.559659040577142	0.877581080322399	NA	NA	XP_001846738.1 con	XP_021708339.1titin homolog
AAEL0064	protein YIPF6	0.129578662635163	0.500800602882654	0.858744381072989	NA	NA	ETN66004.1 integral	XP_001657814.1protein YIPF6
AAEL0103	tyrosine-protein kinase transmembrane receptor Ror	0.129605809643329	0.606369252388072	0.893547770777888	NA	aag04310	NA	XP_021699066.1tyrosine-protein kinase transmembrane receptor Ror isoform X1
AAEL0028	uncharacterized LOC5576359	0.129617964094923	0.694408208814944	0.916122517126697	NA	NA	KXJ78309.1 hypothet	XP_001656097.1uncharacterized protein LOC5576359
AAEL0087	dynein assembly factor 1, axonemal homolog	0.129812695582957	0.420699981290802	0.813636239562413	NA	NA	XP_001866425.1 con	XP_001659459.2dynein assembly factor 1, axonemal homolog

AAEL0012(	phosphatidylinositol-glycan biosynthesis class X protein	0.129853786101208	0.659984465325282	0.909580313002131	NA	aag01100 ; aag	ETN67380.1 hypothe	XP_001658312.1phosphatidylinositol-glycan biosynthesis class X protein
AAEL0196	NA	0.129874858232821	0.299962667107577	0.731581357732289	NA	NA	NA	XP_021710136.1TATA-binding protein-associated factor 172
AAEL0237	NA	0.129891982634191	0.258665117266317	0.691547675112889	NA	NA	NA	NA
AAEL0085	NA	0.13011652828083	0.678897792513195	0.914481546552978	NA	NA	XP_019539417.1 PRE	XP_001659359.1transcription factor grauzone
AAEL0039	dehydrogenase/reductase SDR family member 7	0.130175381820868	0.333373500331218	0.755632763673653	NA	NA	ETN67780.1 short-ch	XP_001648134.2dehydrogenase/reductase SDR family member 7
AAEL0219	NA	0.13035121896859	0.657567582564003	0.909505019503075	NA	aag04130	NA	XP_021706990.1syntaxin-16 isoform X1
AAEL0112	innexin inx3	0.130367453525814	0.366963014619483	0.780578423384551	NA	NA	XP_019541028.1 PRE	XP_001661531.2innexin inx3
AAEL0044	ribosome production factor 2 homolog	0.130462430034393	0.363490561090395	0.778250660803901	NA	NA	KXJ74674.1 hypothe	XP_001649208.2ribosome production factor 2 homolog
AAEL0015	protein dopey-1 homolog	0.130503292097683	0.338430913002187	0.760706663786543	NA	NA	NA	XP_001653582.2protein dopey-1 homolog isoform X3
AAEL0217	NA	0.130510182309665	0.251018084161446	0.683858606596939	NA	NA	NA	XP_021705740.1uncharacterized protein LOC5575778 isoform X3
AAEL0142	mitochondrial amidoxime-reducing component 1	0.130559938937052	0.284812708459313	0.71725411692463	790	NA	NA	XP_001648334.2mitochondrial amidoxime-reducing component 1
AAEL0073	polycomb protein Scm	0.13075672960078	0.483453810097022	0.847379421050056	NA	NA	KXJ79567.1 hypothe	XP_021710690.1polycomb protein Scm
AAEL0109	alpha-tocopherol transfer protein-like	0.130757723151234	0.302653305054015	0.735250294168217	NA	NA	ETN60608.1 hypothe	XP_001661205.1alpha-tocopherol transfer protein-like
AAEL0186	NA	0.130855440081435	0.799647877416325	0.946215055875588	NA	aag01100 ; aag	NA	YP_009389269.1NADH dehydrogenase subunit 4L (mitochondrion)
AAEL0260	NA	0.130873785606164	0.593522562233064	0.888529762455765	NA	NA	NA	NA
AAEL0239	NA	0.130905465674923	0.7071168124068	0.91797784850989	NA	NA	NA	XP_001655670.2protein YIPF5 homolog
AAEL0181	NA	0.130960957335581	0.294100366769427	0.724764746928686	NA	aag04711	NA	XP_021711112.1nuclear pore complex protein DDB_G0274915 isoform X5
AAEL0095	ribonucleases P/MRP protein subunit POP1	0.131009947656821	0.572598831423943	0.880923047085115	NA	aag03013 ; aag	NA	XP_021710581.1ribonucleases P/MRP protein subunit POP1
AAEL0009	tenascin	0.131010906566331	0.394128721750953	0.794388440103915	NA	NA	KFB35181.1 AGAP01	XP_019525043.1 tenascin
AAEL0072	cAMP-dependent protein kinase catalytic subunit	0.131041947758486	0.246784056068664	0.680385311739523	4150	aag04140 ; aag	ETN65541.1 camp-de	XP_001652671.1cAMP-dependent protein kinase catalytic subunit
AAEL0022	YTH domain-containing protein 1	0.131082640173742	0.315786743938246	0.745684464362801	NA	NA	ETN64938.1 splicing	XP_001661159.1YTH domain-containing protein 1
AAEL0005	esterase B1	0.131140624944223	0.360141833853963	0.774570517699107	NA	NA	XP_001869625.1 cho	XP_021709378.1esterase B1
AAEL0072	derlin-1	0.131201868585476	0.281248302027992	0.715107379448722	NA	aag04141	XP_001863080.1 der	ABF18448.1derlin 1
AAEL0113	uncharacterized LOC5574673	0.131335624363278	0.363220344434755	0.778250660803901	NA	NA	XP_001847686.1 con	XP_019555384.1 zinc finger protein 180-like
AAEL0011(	drebrin-like protein	0.131353687595929	0.49734475089941	0.856052403704179	NA	NA	XP_019536179.1 PRE	XP_001652425.2drebrin-like protein isoform X2
AAEL0140	probable tRNA(His) guanylyltransferase	0.131395097148037	0.561178364921934	0.878523871046553	NA	NA	NA	XP_021705065.1probable tRNA(His) guanylyltransferase
AAEL0043	soma ferritin	0.131490056785666	0.631180584994292	0.902506916959539	NA	NA	KXJ70598.1 hypothe	XP_001648938.1soma ferritin
AAEL0014(	acidic leucine-rich nuclear phosphoprotein 32 family member A	0.131590314905941	0.348437374680489	0.766929259132545	NA	NA	KXJ74865.1 hypothe	XP_001659163.2acidic leucine-rich nuclear phosphoprotein 32 family member A
AAEL0018	U4/U6.U5 tri-snRNP-associated protein 2	0.131819943023942	0.502323079943112	0.858744381072989	NA	aag03040	KXJ72305.1 hypothe	XP_001660405.1U4/U6.U5 tri-snRNP-associated protein 2
AAEL0248	NA	0.131919798519264	0.318146351431223	0.745684464362801	NA	NA	NA	XP_021698400.1death-inducer obliterator 1
AAEL0194	NA	0.131933324570485	0.523727347557517	0.865215478807089	908	aag01100	NA	XP_021694471.1tRNA dimethylallyltransferase, mitochondrial
AAEL0244	NA	0.132006379379378	0.478592452614861	0.845416382162313	NA	NA	NA	XP_021695710.1PAS domain-containing serine/threonine-protein kinase isoform X1
AAEL0124	prostaglandin reductase 1	0.132051742322513	0.454971654763055	0.832617462606238	NA	NA	KXJ84395.1 hypothe	XP_001656050.2prostaglandin reductase 1
AAEL0259	NA	0.132138610419751	0.495438240850339	0.854260523077286	NA	NA	NA	XP_021695176.1brefeldin A-inhibited guanine nucleotide-exchange protein 3
AAEL0194	NA	0.132159808587081	0.452436072170249	0.832078850936909	NA	NA	NA	XP_021705923.1prostaglandin E2 receptor EP4 subtype isoform X1
AAEL0145(	mediator of RNA polymerase II transcription subunit 10	0.132230002938066	0.480436616644687	0.84563461101079	NA	NA	NA	XP_001648875.1mediator of RNA polymerase II transcription subunit 10
AAEL0093	probable elongation factor 1-delta	0.132331955178677	0.346364374860084	0.765398366276275	NA	NA	NA	XP_001659921.1probable elongation factor 1-delta isoform X3
AAEL0078	beta-1,4-galactosyltransferase 7	0.132356684618971	0.306477415431927	0.738783696372872	NA	aag01100 ; aag	KXJ82587.1 hypothe	XP_001653000.2beta-1,4-galactosyltransferase 7
AAEL0282	NA	0.132360028526037	0.682548095638324	0.914629465876247	NA	NA	NA	XP_021712733.1ubiquitin thioesterase OTU1-like
AAEL0088	uncharacterized protein C05D11.1	0.132681802096418	0.272895297239827	0.708173300959077	NA	NA	KXJ72958.1 hypothe	XP_001653480.2uncharacterized protein C05D11.1
AAEL0173	E3 ubiquitin-protein ligase TRIP12	0.132691248015319	0.236957196361912	0.67133008649086	NA	aag04120	NA	XP_021710575.1E3 ubiquitin-protein ligase TRIP12
AAEL0101	probable cytochrome P450 6a14	0.132909361403588	0.389682328986538	0.793285976044306	NA	NA	NA	XP_021702544.1probable cytochrome P450 6a14
AAEL0003	splicing factor 3A subunit 2	0.132977718679735	0.581650712797878	0.883632292340932	NA	aag03040	ETN62686.1 splicing	XP_001656059.1splicing factor 3A subunit 2
AAEL0059	protein CLEC16A	0.13307254710843	0.304050926665144	0.737447714067647	NA	NA	KXJ83871.1 hypothe	XP_001651585.1protein CLEC16A isoform X2
AAEL0103	peptidyl-tRNA hydrolase ICT1, mitochondrial	0.133135431795481	0.470655491915682	0.841292120553244	NA	NA	NA	XP_001654462.1peptidyl-tRNA hydrolase ICT1, mitochondrial
AAEL0032	low molecular weight phosphotyrosine protein phosphatase 1	0.13321699901558	0.509538177307548	0.860083070787677	00740 ; 00730	aag01100 ; aag	ETN61007.1 low mol	XP_001656635.1low molecular weight phosphotyrosine protein phosphatase 1
AAEL0070	probable cytochrome P450 28a5	0.133237601412299	0.41560628204265	0.80969508372775	NA	NA	NA	XP_001652490.2probable cytochrome P450 28a5
AAEL0015(	uncharacterized LOC5571117	0.133315329606135	0.405263013350348	0.801349782813648	NA	NA	NA	XP_001659559.1uncharacterized protein LOC5571117
AAEL0122	fatty acid hydroxylase domain-containing protein 2	0.133331190841582	0.432896569179618	0.820005280371151	NA	NA	KXJ69286.1 hypothe	XP_001662400.2fatty acid hydroxylase domain-containing protein 2
AAEL0282	NA	0.133344231778284	0.480575016627757	0.84571644731471	NA	NA	NA	XP_021701456.1uncharacterized protein LOC110676736
AAEL0047	E3 ubiquitin-protein ligase TRIM37	0.133384820065423	0.482173838321777	0.846941547268585	NA	NA	XP_001843697.1 con	XP_001649653.1E3 ubiquitin-protein ligase TRIM37
AAEL0050	lactosylceramide 4-alpha-galactosyltransferase	0.133474390360586	0.731227151492867	0.925260275963946	NA	aag01100 ; aag	KXJ68372.1 hypothe	XP_001650194.1lactosylceramide 4-alpha-galactosyltransferase
AAEL0120	synaptic vesicle glycoprotein 2C	0.133577893823611	0.340145562813016	0.761379832492257	NA	NA	XP_019530358.1 PRE	XP_021694442.1synaptic vesicle glycoprotein 2C isoform X2
AAEL0207	NA	0.133606565654369	0.265951902866428	0.701229057357269	NA	NA	NA	XP_021699881.1protein lifeguard 1 isoform X1
AAEL0072	protein ABHD11	0.133743958297837	0.315744215361878	0.745684464362801	NA	NA	KXJ77107.1 hypothe	XP_001658246.1protein ABHD11
AAEL0048	probable elongator complex protein 2	0.13375233725149	0.628048147796278	0.901045385683245	NA	NA	KXJ73085.1 hypothe	XP_021696899.1probable elongator complex protein 2
AAEL0198	NA	0.133772281715553	0.357538452204427	0.773736338358957	NA	NA	NA	XP_021700775.1E3 ubiquitin-protein ligase MIB2 isoform X2
AAEL0060	RNA polymerase II subunit A C-terminal domain phosphatase SSU72	0.133793140212249	0.359069277875227	0.77388983997572	04660 ; 04658	aag03015	ETN66657.1 phospha	XP_019552112.1 RNA polymerase II subunit A C-terminal domain phosphatase SSU72-like
AAEL0041	ankyrin repeat and MYND domain-containing protein 2	0.134008348223273	0.407737634116845	0.803607536382376	NA	NA	KXJ78877.1 hypothe	XP_001648596.1ankyrin repeat and MYND domain-containing protein 2
AAEL0004	U3 small nucleolar RNA-associated protein 15 homolog	0.134017909566765	0.342028329688834	0.762438298723025	NA	aag03008	KXJ70589.1 hypothe	XP_001656443.1U3 small nucleolar RNA-associated protein 15 homolog
AAEL0034	UBX domain-containing protein 6	0.13411283222129	0.311905409575834	0.743490640759055	NA	aag04141	KXJ81030.1 hypothe	XP_001663795.2UBX domain-containing protein 6
AAEL0060	myotubularin-related protein 13	0.134241843327456	0.368594393964061	0.781702146406381	NA	NA	KXJ76934.1 hypothe	XP_021707699.1myotubularin-related protein 13
AAEL0141	phosphatidylinositol glycan anchor biosynthesis class U protein	0.134254669391871	0.356026369890602	0.772867683747772	NA	aag01100 ; aag	NA	XP_001648170.1phosphatidylinositol glycan anchor biosynthesis class U protein
AAEL0226	NA	0.134408945013494	0.404538249908609	0.800780923576657	NA	NA	NA	XP_021696108.1uncharacterized protein LOC5573286
AAEL0112	von Willebrand factor A domain-containing protein 8	0.134411237091254	0.387566080154464	0.791825725928371	NA	NA	KXJ69322.1 hypothe	XP_001661571.1von Willebrand factor A domain-containing protein 8
AAEL0135	pinin	0.134617471796865	0.503426678509638	0.858744381072989	NA	aag03013 ; aag	NA	XP_019534546.1 pinin
AAEL0123	putative DNA helicase Ino80	0.134721225222773	0.503256745100426	0.858744381072989	NA	NA	XP_001869591.1 heli	XP_021709328.1putative DNA helicase Ino80
AAEL0064	proteasome subunit beta type-6	0.134735827620797	0.317427844888937	0.745684464362801	NA	aag03050	AEW48021.1 proteas	XP_001657823.1proteasome subunit beta type-6
AAEL0181	NA	0.134791869507204	0.287849824624823	0.719976647512265	NA	NA	NA	XP_021704442.1transmembrane protease serine 9
AAEL0024	mitochondrial import inner membrane translocase subunit Tim10B	0.134818350888805	0.417066152154418	0.811697838844202	NA	NA	ETN59643.1 mitocho	XP_001655352.1mitochondrial import inner membrane translocase subunit Tim10B isoform X2
AAEL0009	glutactin	0.134845000520901	0.464702565172031	0.83781210907387	NA	NA	KXJ82747.1 hypothe	XP_019564208.1 glutactin-like
AAEL0045	beta-galactosidase	0.135022837339678	0.823277185947015	0.954491201346155	00531 ; 00600 ; 00604 ;	NA	XP_019541283.1 PRE	XP_019558431.1 beta-galactosidase-like isoform X1
AAEL0079	EGF domain-specific O-linked N-acetylglucosamine transferase	0.135057188369557	0.666093357004724	0.911964500946328	NA	aag00514	KXJ78286.1 hypothe	XP_001658768.2EGF domain-specific O-linked N-acetylglucosamine transferase
AAEL0068	dihydropteridine reductase	0.135154473953348	0.298138122437034	0.7302583490254	NA	aag01100 ; aag	XP_001869487.1 dih	XP_001652256.1dihydropteridine reductase isoform X1
AAEL0105(	transcriptional regulator ATRX	0.135166650028612	0.360767863692327	0.77528862253545	NA	NA	NA	XP_001654617.2transcriptional regulator ATRX
AAEL0277	NA	0.135190018853269	0.651469343414779	0.907172139858737	NA	NA	NA	XP_021712780.1DDRGK domain-containing protein 1-like
AAEL0244	NA	0.135196463053863	0.604595225501204	0.893547770777888	NA	NA	NA	ABF18419.1polyadenylate-binding protein-like protein
AAEL0040(	NAD-dependent protein deacylase Sirt4	0.135361974374231	0.454001420579679	0.832617462606238	NA	aag01100 ; aag	KXJ80886.1 hypothe	XP_001648309.2NAD-dependent protein deacylase Sirt4
AAEL0173	mitochondrial 2-oxoglutarate/malate carrier protein	0.135728541422808	0.228980196463788	0.662455890682856	NA	NA	NA	XP_011493174.1mitochondrial 2-oxoglutarate/malate carrier protein
AAEL0057	uncharacterized LOC5566986	0.135743742740296	0.357608841853813	0.773736338358957	NA	NA	ETN66464.1 leucine-	ETN66464.1leucine-rich transmembrane protein
AAEL0034	farnesyl pyrophosphate synthase	0.135775755906903	0.26291634061747	0.696819172339274	900	aag01100 ; aag	KXJ81031.1 hypothe	XP_001663796.1farnesyl pyrophosphate synthase
AAEL0067	putative uncharacterized protein DDB_G0290989	0.136108122200927	0.213785929688892	0.647124383222757	NA	NA	XP_001862122.1 con	XP_019539605.1 GATA zinc finger domain-containing protein 14-like

AAEL00121	uncharacterized LOC5569075	0.136271013449452	0.244464868207036	0.67962710490669	NA	NA	KFB42910.1 hypothe	XP_001658305.2uncharacterized protein LOC5569075
AAEL01436	MFS-type transporter SLC18B1	0.136426780698998	0.386886331972004	0.791623050051228	NA	NA	NA	XP_021706800.1MFS-type transporter SLC18B1 isoform X2
AAEL00937	39S ribosomal protein L33, mitochondrial	0.13662364758687	0.270589945696979	0.706237745304114	NA	aag03010	NA	XP_001653807.139S ribosomal protein L33, mitochondrial
AAEL00749	calcineurin B homologous protein 1	0.136733520201531	0.229516463005097	0.662487607456997	NA	NA	AEZ67827.1 AGAP00	XP_001652782.1calcineurin B homologous protein 1
AAEL00103	metaxin-1 homolog	0.136929194540219	0.516300119500492	0.861448202130317	NA	NA	KXJ67984.1 hypothet	XP_021710248.1metaxin-1 homolog
AAEL00874	exportin-2	0.136946418526355	0.41902514783005	0.813348977896628	NA	NA	KXJ71133.1 hypothet	XP_019548290.1 exportin-2
AAEL00213	uncharacterized LOC5573573	0.13696961936941	0.513073269310917	0.861297436611866	NA	NA	KFB51831.1 AGAP00	KFB51831.1AGAP003856-like protein
AAEL00963	cathepsin B	0.137064802106117	0.26782163880332	0.704026692311667	NA	aag04140 ; aag	NA	XP_001653890.2cathepsin B
AAEL00498	inhibitor of growth protein 4	0.137160382033432	0.387713965366116	0.791952033140732	NA	NA	KFB41009.1 AGAP00	XP_001650124.1inhibitor of growth protein 4 isoform X2
AAEL01022	NA	0.137383250159266	0.449809018100348	0.829440335346263	NA	NA	NA	XP_001654375.1box A-binding factor isoform X4
AAEL02326	NA	0.137619996557492	0.572850005249515	0.880923047085115	NA	NA	NA	XP_021706210.1cell division cycle and apoptosis regulator protein 1
AAEL00382	transmembrane protein 60	0.137635563645414	0.631678310398266	0.902506916959539	NA	NA	XP_001842970.1 con	XP_021704988.1transmembrane protein 60
AAEL00251	zinc finger protein 91	0.13775039876458	0.554891627141141	0.875683888347243	NA	NA	KXJ75736.1 hypothet	XP_021697768.1zinc finger protein 91
AAEL01748	keratin, type I cytoskeletal 9	0.137795798177009	0.253456637076445	0.685145258966747	NA	NA	NA	XP_011493298.1keratin, type I cytoskeletal 9
AAEL02653	NA	0.137807093602361	0.370397888208131	0.781817769662763	NA	NA	NA	XP_021710701.1uncharacterized protein LOC5577812 isoform X1
AAEL01377	uncharacterized LOC5578590	0.137831121509305	0.52591391576303	0.865680248818465	NA	NA	NA	XP_001657009.1uncharacterized protein LOC5578590
AAEL02131	NA	0.13788851866033	0.649648849899419	0.90663577761678	NA	NA	NA	XP_001656408.1solute carrier family 35 member F6
AAEL02592	NA	0.137969252933083	0.401768562691696	0.799283473604722	NA	NA	NA	XP_021700125.1ubiquitin carboxyl-terminal hydrolase 35 isoform X1
AAEL02555	NA	0.137970753904998	0.649244917215693	0.906609198022654	NA	NA	NA	NA
AAEL00812	mitochondrial import inner membrane translocase subunit Tim13	0.138127030624561	0.286346592611212	0.718756504421269	NA	NA	ETN64060.1 Mitocho	XP_001658948.1mitochondrial import inner membrane translocase subunit Tim13
AAEL02718	NA	0.138313258694521	0.244645811102547	0.679727114240672	NA	NA	NA	XP_021711668.1uncharacterized protein LOC5569487 isoform X1
AAEL00104	tyrosine--tRNA ligase, cytoplasmic	0.138347770170283	0.248351423062201	0.680588444480365	970	aag00970	KXJ72637.1 hypothet	XP_001657882.2tyrosine--tRNA ligase, cytoplasmic
AAEL00984	uncharacterized LOC5572525	0.138417545035048	0.671814068286643	0.912813925547137	NA	NA	NA	XP_001660464.1uncharacterized protein LOC5572525
AAEL00305	LETM1 domain-containing protein 1	0.138433558760608	0.244407517736393	0.67962710490669	NA	NA	KXJ83266.1 hypothet	XP_001663087.2LETM1 domain-containing protein 1
AAEL00619	hornerin	0.138505979411438	0.680242600443992	0.914629465876247	NA	NA	XP_019556040.1 PRE	XP_021713385.1hornerin isoform X1
AAEL00919	NA	0.138507317376533	0.492712786758539	0.852158101574964	NA	aag00514	NA	XP_001653726.2 xyloside xylosyltransferase 1
AAEL00939	syntaxin-7	0.138532802522998	0.322564643438753	0.746650319748046	NA	NA	NA	XP_019525017.1 syntaxin-7-like
AAEL02109	NA	0.138619301152176	0.403813706448274	0.800429773223221	NA	NA	NA	XP_011493699.2alcohol dehydrogenase 2
AAEL00927	uncharacterized LOC5571752	0.138683961690688	0.394627698410075	0.794957110501207	NA	NA	NA	XP_001653779.1uncharacterized protein LOC5571752
AAEL00750	homeobox protein caupolican	0.138867133758607	0.505982753796469	0.858744381072989	NA	NA	KXJ81971.1 hypothet	XP_001658394.2homeobox protein caupolican
AAEL00613	uncharacterized LOC5567546	0.138897571076792	0.337549347927333	0.759630496362757	NA	NA	KXJ73430.1 hypothet	XP_001657541.2uncharacterized protein LOC5567546
AAEL00666	protein smoothened	0.13893733803589	0.334479785550692	0.757087156181969	NA	aag04341	KXJ72879.1 hypothet	XP_001657927.2protein smoothened
AAEL01095	protein OPI10 homolog	0.138967269494018	0.460946328138861	0.834801832618342	NA	NA	ETN67492.1 protein	XP_001661201.1protein OPI10 homolog
AAEL02488	NA	0.138998120433618	0.501800879024332	0.858744381072989	NA	NA	NA	NA
AAEL02407	NA	0.139094914372377	0.354346283326869	0.771579389298118	NA	aag03040	NA	XP_019546938.1 small nuclear ribonucleoprotein F
AAEL00885	choline/ethanolamine kinase	0.139262276989656	0.252409425103538	0.684556086619159	NA	aag01100 ; aag	NA	XP_001653456.2choline/ethanolamine kinase isoform X2
AAEL01414	caspase-8	0.139276725281441	0.69677555322402	0.91622301239889	NA	aag04214 ; aag	NA	ABI74776.1initiator caspase
AAEL00652	COX assembly mitochondrial protein homolog	0.139287783602998	0.421795515995571	0.814192545906728	NA	NA	KFB39732.1 AGAP00	XP_001652025.1COX assembly mitochondrial protein homolog
AAEL00975	sialin	0.139321175767031	0.692659395010796	0.916122517126697	NA	NA	NA	XP_019540662.1 sialin-like
AAEL01156	NA	0.139332442614352	0.254535365715536	0.686571057876479	NA	NA	KXJ81715.1 hypothet	XP_019541231.1 protein Exd1 homolog
AAEL01707	nucleolar and coiled-body phosphoprotein 1	0.139333295933403	0.270665119452713	0.706237745304114	NA	NA	NA	XP_011493704.2nucleolar and coiled-body phosphoprotein 1 isoform X4
AAEL00573	BUB3-interacting and GLEBS motif-containing protein ZNF207	0.139391922985884	0.336572443369083	0.758445425575872	NA	NA	XP_019555866.1 PRE	XP_001651384.1BUB3-interacting and GLEBS motif-containing protein ZNF207 isoform X5
AAEL01194	chorion peroxidase	0.139398329906827	0.483292049968306	0.847379421050056	NA	NA	KXJ70077.1 hypothet	XP_001662097.2chorion peroxidase
AAEL01162	ribose-5-phosphate isomerase	0.139403101501759	0.342984991863376	0.76358426442382	00030 ; 00051 ; 00710	aag01100 ; aag	KXJ68390.1 hypothet	XP_001661804.1ribose-5-phosphate isomerase
AAEL01016	tetratricopeptide repeat protein 39C	0.139595330461947	0.639614953110637	0.904449291387452	NA	NA	NA	XP_021705414.1tetratricopeptide repeat protein 39C
AAEL00998	ATP synthase mitochondrial F1 complex assembly factor 1	0.139596238674532	0.30508035461928	0.738299809812121	NA	NA	NA	XP_001660532.2ATP synthase mitochondrial F1 complex assembly factor 1
AAEL00424	kinesin-like protein KIN-14Q	0.139614919262097	0.693006860014561	0.916122517126697	NA	NA	KXJ76992.1 hypothet	XP_001648736.2kinesin-like protein KIN-14Q
AAEL01312	elongation of very long chain fatty acids protein 6	0.139649317682186	0.208612433465276	0.639832374753494	62	aag01100 ; aag	NA	XP_021698977.1elongation of very long chain fatty acids protein 6
AAEL02105	NA	0.139708097056917	0.34194757797294	0.762438298723025	00562 ; 04070	aag01100 ; aag	NA	XP_021695356.1type I inositol 1,4,5-trisphosphate 5-phosphatase isoform X2
AAEL01821	NA	0.139797397750608	0.252319743118207	0.684556086619159	NA	NA	NA	XP_021705465.1FERM, RhoGEF and pleckstrin domain-containing protein 2
AAEL01426	translation initiation factor eIF-2B subunit alpha	0.139982800917417	0.497556092850888	0.856052403704179	270	aag03013	NA	XP_001654378.1translation initiation factor eIF-2B subunit alpha
AAEL01736	DNA-directed RNA polymerase II subunit RPB1	0.140056781714778	0.208783296809232	0.639832374753494	NA	aag03020	NA	XP_011493480.1DNA-directed RNA polymerase II subunit RPB1
AAEL02116	NA	0.140127994452905	0.516643692928238	0.861530579862055	NA	NA	NA	NA
AAEL00522	COMM domain-containing protein 10	0.140163138365871	0.587745521219727	0.886401261871374	NA	NA	KXJ83639.1 hypothet	XP_001650542.2COMM domain-containing protein 10
AAEL02485	NA	0.140293854248318	0.234805580580614	0.668737559309875	NA	NA	NA	XP_021696955.1mitochondrial import receptor subunit TOM22 homolog
AAEL02323	NA	0.140426234607691	0.478658916655689	0.845416382162313	NA	NA	NA	NA
AAEL00573	BLOC-1-related complex subunit 8 homolog	0.140477764355278	0.719344982021933	0.921583569204186	NA	NA	KXJ77786.1 hypothet	XP_001651399.1BLOC-1-related complex subunit 8 homolog
AAEL01246	zinc finger protein 234	0.140645140784235	0.550415169042101	0.873864800815288	NA	NA	XP_019551936.1 PRE	XP_001656049.2zinc finger protein 93 isoform X1
AAEL00147	protein numb	0.140665322158269	0.389561975724423	0.793285976044306	NA	aag04330	XP_019529434.1 PRE	XP_001653377.1protein numb isoform X1
AAEL00527	zinc finger protein on ecdysone puffs	0.140672426199359	0.22049597338672	0.652412844728166	NA	NA	XP_019539024.1 PRE	XP_021696852.1ABC transporter F family member 4 isoform X1
AAEL02220	NA	0.14068439531106	0.512113066062844	0.861177338330732	NA	NA	NA	NA
AAEL02240	NA	0.14070819482832	0.447569524051039	0.828571638513641	NA	NA	NA	XP_021712430.1protein 4.1 homolog
AAEL01805	NA	0.140770834668006	0.461520288250952	0.835512432123797	NA	NA	NA	XP_001662612.2bromodomain-containing protein 3
AAEL00906	dynamin-1-like protein	0.140782444049969	0.261906649997097	0.695344775901121	NA	aag04214	KFB43094.1 hypothe	XP_001659672.1dynamin-1-like protein
AAEL00072	mediator of RNA polymerase II transcription subunit 11	0.140928620320571	0.567818560877166	0.880381869787984	NA	NA	ETN59305.1 Mediatc	XP_001650252.1mediator of RNA polymerase II transcription subunit 11
AAEL00950	methylglutaconyl-CoA hydratase, mitochondrial	0.14121824922767	0.381774320189202	0.787864385373635	NA	aag01100 ; aag	NA	XP_001660163.1methylglutaconyl-CoA hydratase, mitochondrial
AAEL00655	programmed cell death protein 5	0.141271174451091	0.309766207257149	0.742889924206485	NA	NA	ETN62962.1 hypothe	XP_001652052.1programmed cell death protein 5
AAEL01269	exonuclease 3'-5' domain-containing protein 2	0.141402381523624	0.474989668521395	0.84437941358363	NA	NA	KFB41194.1 AGAP00	XP_001656205.1exonuclease 3'-5' domain-containing protein 2
AAEL00500	constitutive coactivator of PPAR-gamma-like protein 1	0.141565257666168	0.47926097170765	0.845454613425911	NA	NA	NA	XP_001650162.2constitutive coactivator of PPAR-gamma-like protein 1
AAEL01030	uncharacterized LOC5573208	0.141632366226023	0.441337541880029	0.82360333814237	NA	NA	NA	XP_021704307.1uncharacterized protein LOC5573208 isoform X1
AAEL01309	solute carrier family 12 member 9	0.141652021461651	0.239028457840118	0.673672241667617	NA	NA	NA	XP_021701758.1solute carrier family 12 member 9 isoform X2
AAEL01250	partner of Y14 and mago	0.141661107609972	0.316634293105897	0.745684464362801	NA	aag03013 ; aag	KXJ74493.1 hypothet	XP_001656113.1partner of Y14 and mago
AAEL01708	probable splicing factor, arginine/serine-rich 6	0.141854719255581	0.192899772706791	0.623923974849874	NA	aag03040	NA	XP_011493455.1probable splicing factor, arginine/serine-rich 6
AAEL00768	DNA topoisomerase 3-alpha	0.141944921435001	0.691076721113752	0.916122517126697	NA	aag03460 ; aag	ETN60096.1 DNA top	XP_001658595.1DNA topoisomerase 3-alpha
AAEL01327	peptidyl-prolyl cis-trans isomerase 5	0.142032206129107	0.205692815754498	0.638023927878659	NA	NA	NA	XP_001663442.1peptidyl-prolyl cis-trans isomerase 5
AAEL00210	zinc finger protein 652-A	0.142036359124126	0.653345677294807	0.907272485895905	NA	NA	KXJ82739.1 hypothet	XP_001654709.2zinc finger protein 652-A
AAEL00855	serine/threonine-protein kinase PRP4 homolog	0.142169472817158	0.290626730395187	0.722005075789413	NA	NA	KXJ81314.1 hypothet	XP_001653292.1serine/threonine-protein kinase PRP4 homolog isoform X1
AAEL02335	NA	0.142178452727586	0.737496927025676	0.927599209713406	NA	NA	NA	XP_019555614.1 teneurin-a-like isoform X1
AAEL00853	sorting nexin-29	0.142333334167783	0.583371733883403	0.885109100609245	NA	NA	KXJ69160.1 hypothet	XP_021693712.1sorting nexin-29
AAEL00646	alcohol dehydrogenase class-3	0.142397477703592	0.492696168390035	0.852158101574964	00626 ; 00260 ; 00592 ;	NA	XP_019527276.1 PRE	XP_021706998.1alcohol dehydrogenase class-3
AAEL01135	protein MAK16 homolog A	0.142420749327197	0.267469267703052	0.703818187418782	NA	NA	KFB50748.1 hypothe	XP_001655305.2protein MAK16 homolog A
AAEL00740	ras-interacting protein RIP3	0.142430846610031	0.298274452709757	0.730367619434589	NA	NA	NA	XP_021704198.1ras-interacting protein RIP3 isoform X7

AAEL00796	glutathione S-transferase 1	0.142557515361714	0.248926428943691	0.681164287957569	NA	aag01100 ; aag	AEJ87232.1 glutathic	XP_001658750.1glutathione S-transferase 1
AAEL01209	26S proteasome regulatory subunit 4	0.142670944414875	0.214472153777142	0.648028428468258	NA	aag03050	CRK88438.1 CLUMA_	XP_001655853.126S proteasome regulatory subunit 4
AAEL00279	L-asparaginase	0.142720701283905	0.357504145153675	0.773736338358957	00250 ; 00460	NA	XP_019532439.1 PRE	XP_019532440.1 L-asparaginase-like isoform X2
AAEL00449	transmembrane protein 115	0.142754490821499	0.336725347824724	0.758445425575872	NA	NA	KXJ72928.1 hypothet	XP_001649289.2transmembrane protein 115
AAEL01405	zinc finger matrin-type protein CG9776	0.142783644587398	0.332536256790954	0.755231182999982	NA	NA	NA	XP_021708060.1zinc finger matrin-type protein CG9776
AAEL00744	protein Gawky	0.14297430674409	0.245755774183136	0.679727114240672	NA	NA	NA	XP_021701059.1protein Gawky isoform X2
AAEL02170	NA	0.143012767529117	0.512526592880535	0.861217671252058	NA	NA	NA	XP_021693062.1uncharacterized protein LOC5563548
AAEL00096	uncharacterized LOC5567452	0.143021324904991	0.251110829995452	0.683858606596939	NA	NA	KXJ80288.1 hypothet	XP_021708553.1uncharacterized protein LOC5567452
AAEL00058	5-oxoprolinase	0.143162757215858	0.192072677393397	0.622342527244381	NA	aag01100 ; aag	KXJ77230.1 hypothet	XP_019551825.1 5-oxoprolinase
AAEL00936	cyclin-dependent kinase 12	0.143167128024716	0.292722911292769	0.723512283028756	NA	NA	NA	XP_021708726.1cyclin-dependent kinase 12 isoform X2
AAEL00540	uncharacterized LOC5566479	0.143338125477234	0.635657152156862	0.902538767814767	NA	NA	KXJ73566.1 hypothet	XP_021693094.1uncharacterized protein LOC5566479
AAEL00531	leucine-rich melanocyte differentiation-associated protein	0.143477757694662	0.24537163706568	0.679727114240672	NA	NA	NA	XP_011493022.2leucine-rich melanocyte differentiation-associated protein isoform X4
AAEL01384	endoplasmic reticulum resident protein 44	0.143478480041109	0.190288554017917	0.618521820583848	NA	NA	NA	XP_021699849.1endoplasmic reticulum resident protein 44 isoform X2
AAEL00273	zinc finger protein 836	0.143500394598874	0.612071474439947	0.895007422182805	NA	NA	XP_019557893.1 PRE	XP_001655898.2zinc finger protein 836 isoform X1
AAEL01541	NA	0.143634942747832	0.365849887285852	0.779835687469172	00760 ; 00760	aag01100 ; aag	NA	XP_021694759.1probable glutamine-dependent NAD(+) synthetase isoform X2
AAEL02512	NA	0.143677644187236	0.202792525347085	0.635808115244934	NA	NA	NA	XP_021708967.1cytochrome c oxidase assembly factor 7 homolog
AAEL02711	NA	0.143720021772659	0.390859194365528	0.793285976044306	NA	NA	NA	XP_021702663.1serine/threonine-protein kinase GL21140 isoform X1
AAEL00269	transmembrane protein 87A	0.143742636632547	0.319493535062073	0.745684464362801	NA	NA	KXJ82185.1 hypothet	XP_001662124.2transmembrane protein 87A isoform X1
AAEL01222	zinc finger protein 431	0.143840378747303	0.634528272962644	0.902506916959539	NA	NA	KXJ78783.1 hypothet	XP_001662329.1zinc finger protein 431
AAEL00183	prefoldin subunit 1	0.143853281896938	0.247948756284672	0.68058844480365	NA	NA	ETN63812.1 prefoldi	XP_001654136.1prefoldin subunit 1
AAEL00481	nucleolar complex protein 2 homolog	0.14391174462749	0.194971558806476	0.627098316720443	NA	NA	KXJ81191.1 hypothet	XP_021705136.1nucleolar complex protein 2 homolog
AAEL00191	tyrosine-protein phosphatase non-receptor type 61F	0.144136163032652	0.228145248765056	0.66175420142127	NA	NA	XP_019560031.1 PRE	XP_021699286.1tyrosine-protein phosphatase non-receptor type 61F isoform X2
AAEL01009	maternal protein exuperantia-2	0.144227501674052	0.219495916741388	0.652412844728166	NA	NA	NA	XP_001654240.1maternal protein exuperantia-2
AAEL00451	glycine receptor subunit alpha-2	0.144253486397384	0.293649772703184	0.724246906570544	NA	NA	KXJ70313.1 hypothet	XP_001649333.2glycine receptor subunit alpha-2
AAEL00994	homeotic protein Sex combs reduced	0.144298125539867	0.474540790912999	0.844332076309675	NA	NA	NA	NP_001345957.1homeotic protein Sex combs reduced
AAEL01967	NA	0.144322384432124	0.479856289351101	0.845454613425911	NA	NA	NA	XP_021698883.1esterase B1 isoform X1
AAEL00924	E3 ubiquitin-protein ligase RFWD3	0.14432333304955	0.560895859433151	0.878368233725523	NA	NA	NA	XP_001659858.1E3 ubiquitin-protein ligase RFWD3
AAEL00540	annexin B10	0.144358607787487	0.243170174317723	0.677870931254677	NA	NA	XP_001850033.1 anr	XP_001650852.2annexin B10
AAEL01108	dnaJ-like protein 60	0.144414003688493	0.249284947756697	0.681571330200593	NA	NA	KXJ82436.1 hypothet	XP_001661398.1dnaJ-like protein 60
AAEL01953	NA	0.144485968158855	0.530646515134656	0.866691320214768	310	NA	NA	XP_021701074.1titin homolog isoform X1
AAEL00604	PAX3- and PAX7-binding protein 1	0.144552119835009	0.486599202962289	0.848528684036191	NA	NA	XP_019548183.1 PRE	XP_021709599.1PAX3- and PAX7-binding protein 1 isoform X1
AAEL01407	apoptotic chromatin condensation inducer in the nucleus	0.144666796457214	0.289089016874928	0.721217831038796	NA	aag03013 ; aag	NA	XP_021702950.1apoptotic chromatin condensation inducer in the nucleus
AAEL01079	ubiquitin-conjugating enzyme E2 G1	0.144697878344234	0.200898233882394	0.634514122727206	NA	aag04141 ; aag	NP_001103772.1 ubi	XP_001654923.1ubiquitin-conjugating enzyme E2 G1 isoform X1
AAEL01144	calcium/calmodulin-dependent protein kinase type 1	0.144786634292216	0.316880476382727	0.745684464362801	NA	NA	ETN62818.1 calcium/	XP_001661659.2calcium/calmodulin-dependent protein kinase type 1
AAEL00080	lachesin	0.144965926849914	0.524050407964277	0.865215478807089	NA	NA	XP_019537947.1 PRE	XP_019537947.1 hemicentin-2-like
AAEL00046	probable 39S ribosomal protein L49, mitochondrial	0.144993189224175	0.397400043729672	0.797539006884967	NA	NA	KXJ81052.1 hypothet	XP_001656467.1probable 39S ribosomal protein L49, mitochondrial
AAEL00252	mitochondrial import inner membrane translocase subunit Tim9	0.145012171149604	0.294894232865987	0.726148442823872	NA	NA	ETN59010.1 mitocho	XP_001655468.1mitochondrial import inner membrane translocase subunit Tim9
AAEL00833	ubiquinone biosynthesis O-methyltransferase, mitochondrial	0.145081719871686	0.246925569237142	0.680385311739523	130	NA	KFB47348.1 AGAP01	XP_001659135.1ubiquinone biosynthesis O-methyltransferase, mitochondrial
AAEL00111	ubiquitin carboxyl-terminal hydrolase 5	0.14513277127035	0.264259337047521	0.698659663885272	NA	NA	KXJ84469.1 hypothet	XP_021711605.1ubiquitin carboxyl-terminal hydrolase 5
AAEL01461	NA	0.145148805204101	0.27642483322726	0.711749963236253	NA	NA	NA	XP_001649107.2probable cytochrome P450 9f2
AAEL02203	NA	0.145150175578146	0.370333133176874	0.781817769662763	NA	NA	NA	NA
AAEL01435	serine protease easter	0.145165593719493	0.436279063557305	0.820960220424486	NA	NA	NA	XP_021700235.1serine protease easter
AAEL00922	ribosome-recycling factor, mitochondrial	0.14542607932172	0.323268720985657	0.747553575589157	NA	NA	NA	XP_001659843.2ribosome-recycling factor, mitochondrial
AAEL02703	NA	0.145438181264482	0.584641156714354	0.885175928718656	NA	aag03040	NA	XP_021700110.1pre-mRNA-processing factor 17
AAEL00502	pre-mRNA-processing factor 40 homolog A	0.14554373940275	0.339852963310603	0.761379832492257	NA	aag03040	KXJ74426.1 hypothet	XP_001650202.1pre-mRNA-processing factor 40 homolog A
AAEL00873	m7GpppX diphosphatase	0.145989024255472	0.274408516412588	0.710214476791569	NA	aag03018	XP_019542345.1 PRE	XP_001653428.2m7GpppX diphosphatase isoform X2
AAEL01028	beta-ureidopropionase	0.145999411750801	0.341498479986569	0.762180211699347	NA	aag01100 ; aag	NA	XP_019546157.1 beta-ureidopropionase-like
AAEL01988	NA	0.146078592324438	0.437549765401066	0.820960220424486	NA	NA	NA	XP_021709343.1dystrobrevin beta isoform X1
AAEL00926	serine-rich adhesin for platelets	0.146104149278191	0.26728204850917	0.703526719216641	520	NA	NA	XP_021700736.1uncharacterized protein LOC5571731 isoform X2
AAEL01461	probable cytochrome P450 9f2	0.146176903824032	0.323757026410977	0.74762391301976	NA	NA	NA	XP_001649101.2probable cytochrome P450 9f2
AAEL00593	protein Mpv17	0.146328141638067	0.405833384492993	0.802044651724185	NA	aag04146	XP_001861838.1 mp	XP_011493309.1protein Mpv17
AAEL01429	protein CWC15 homolog	0.146333142359573	0.596569273556411	0.889585230151692	NA	aag03040	NA	XP_021694973.1protein CWC15 homolog
AAEL00561	uncharacterized LOC5566760	0.146605889121466	0.238158452184662	0.67280858706937	NA	aag01100 ; aag	XP_321951.2 AGAP0	AAF34699.2chitin synthase
AAEL00746	RINT1-like protein	0.146640373974654	0.463530352705592	0.837274753622609	NA	NA	KXJ69082.1 hypothet	XP_001658388.1RINT1-like protein
AAEL01371	NA	0.146964577725144	0.836113547565516	0.961379090928137	NA	aag04080	NA	XP_001663895.2trypsin 5G1
AAEL01084	eukaryotic translation initiation factor eIF1	0.146998485603091	0.228975668016046	0.662455890682856	NA	aag03013	XP_002432016.1 tra	XP_001661080.1eukaryotic translation initiation factor eIF1
AAEL02384	NA	0.147011756915157	0.274661003244564	0.710351476069837	NA	NA	NA	XP_001650372.3esterase B1
AAEL01047	facilitated trehalose transporter Tret1	0.147067655694612	0.303886583614813	0.737301362252096	NA	NA	NA	XP_021693618.1facilitated trehalose transporter Tret1
AAEL00746	zinc finger protein 37 homolog	0.14710747586764	0.6528270852741	0.907272485895905	NA	NA	KXJ69974.1 hypothet	XP_001658375.2zinc finger protein 37 homolog
AAEL01190	1-acyl-sn-glycerol-3-phosphate acyltransferase alpha	0.147170823307728	0.551464053354911	0.874531710113044	00561 ; 00564	NA	KXJ73650.1 hypothet	XP_001662024.21-acyl-sn-glycerol-3-phosphate acyltransferase alpha
AAEL00873	SHC-transforming protein 4	0.147268713496543	0.392642452216514	0.793563770106823	NA	aag04013	KXJ73540.1 hypothet	XP_001653446.1SHC-transforming protein 4
AAEL02117	NA	0.147342627000684	0.290222349972998	0.721952579380462	NA	NA	NA	XP_021713151.1transmembrane protein 179
AAEL00964	eukaryotic translation initiation factor 4B	0.147478363251203	0.184278811312621	0.611957279573706	NA	aag03013 ; aag	NA	XP_021692916.1eukaryotic translation initiation factor 4B isoform X1
AAEL00821	cytoplasmic aconitate hydratase	0.147542919302811	0.178635474309784	0.604117469532757	00290 ; 00630 ; 00720 ;	aag01100 ; aag	KXJ79410.1 hypothet	XP_001659032.1cytoplasmic aconitate hydratase
AAEL01697	apolipoprotein D	0.147613035688064	0.191721419590887	0.621678529880687	NA	NA	NA	XP_021709994.1apolipoprotein D
AAEL02599	NA	0.147864685037571	0.209126456280555	0.639832374753494	NA	aag01100 ; aag	NA	XP_021694038.1dihydropyrimidine dehydrogenase [NADP(+)]
AAEL01126	yemanuclein	0.14792162200332	0.487014838449375	0.848846834445739	NA	NA	XP_019537115.1 PRE	XP_001661544.2yemanuclein isoform X2
AAEL01075	cell cycle checkpoint control protein RAD9A	0.147933981246901	0.681347188218572	0.914629465876247	NA	NA	KXJ68302.1 hypothet	XP_001654863.1cell cycle checkpoint control protein RAD9A
AAEL00307	tetratricopeptide repeat protein 27	0.147986785383465	0.43330555065486	0.820258728065422	NA	NA	KXJ83058.1 hypothet	XP_001663057.1tetratricopeptide repeat protein 27
AAEL00552	lysosomal Pro-X carboxypeptidase	0.147996501600268	0.202746576430974	0.635808115244934	NA	NA	KXJ79295.1 hypothet	XP_001651053.1lysosomal Pro-X carboxypeptidase
AAEL00393	cleavage stimulation factor subunit 1	0.148165436688866	0.454194833008463	0.832617462606238	NA	aag03015	ETN63599.1 mRNA cl	XP_001648071.1cleavage stimulation factor subunit 1
AAEL01307	tudor and KH domain-containing protein homolog	0.14835140509358	0.185375367037125	0.612504175036132	NA	NA	NA	XP_001663280.1tudor and KH domain-containing protein homolog
AAEL02315	NA	0.148427755652916	0.322273003344924	0.746650319748046	NA	NA	NA	XP_021694598.1stromal interaction molecule homolog isoform X1
AAEL01980	NA	0.148453251942503	0.579650473668967	0.882372813681032	NA	aag04080	NA	XP_021705609.1muscarinic acetylcholine receptor M3
AAEL00871	solute carrier family 25 member 46	0.148722694508378	0.312935563950386	0.744189298893767	NA	NA	KXJ72322.1 hypothet	XP_001659451.2solute carrier family 25 member 46
AAEL00698	probable cytochrome P450 28a5	0.148853619913744	0.577305983580793	0.881961491587822	NA	NA	KXJ73463.1 hypothet	XP_001652487.1probable cytochrome P450 28a5
AAEL00018	39S ribosomal protein L18, mitochondrial	0.148903361490882	0.269339637679	0.705504817546424	NA	aag03010	KXJ71317.1 hypothet	XP_001658909.239S ribosomal protein L18, mitochondrial
AAEL01388	NA	0.149086695153152	0.372363853591385	0.781817769662763	NA	NA	NA	XP_001664091.2uncharacterized protein LOC5578882
AAEL02653	NA	0.149097412745081	0.633748278737143	0.902506916959539	NA	NA	NA	XP_021696189.1uncharacterized protein LOC5572885
AAEL00685	uncharacterized LOC5568443	0.149119552400988	0.633873164133304	0.902506916959539	NA	NA	KXJ84566.1 hypothet	XP_021706283.1uncharacterized protein LOC5568443
AAEL02496	NA	0.149166775179672	0.720810434803504	0.921583569204186	NA	NA	NA	XP_019554814.1 prisilkin-39-like
AAEL01813	NA	0.149268989084702	0.34781812825511	0.766249082165077	NA	NA	NA	XP_021709655.1lysine-specific demethylase 3B isoform X1

AAEL0137€	glia maturation factor gamma	0.149303842337819	0.344678531484511	0.764706052029763	NA	NA	NA	XP_001663974.1glia maturation factor gamma
AAEL0053€	high mobility group protein 20A	0.149375463461719	0.585551220112677	0.885239904594145	NA	NA	KXJ74782.1 hypothet	XP_001650689.2high mobility group protein 20A
AAEL0131!	methionine aminopeptidase 1D, mitochondrial	0.149379441139121	0.418126806870854	0.812788254564712	NA	NA	NA	XP_001663333.1methionine aminopeptidase 1D, mitochondrial
AAEL0098!	uncharacterized LOC5572513	0.149437629300945	0.533590542393881	0.866700945213732	NA	NA	NA	XP_019547928.1 galectin-12-like
AAEL0088€	zinc finger protein 2 homolog	0.149470160411898	0.499155165121175	0.857105025087713	NA	NA	KXJ74922.1 hypothet	XP_001653492.2zinc finger protein 2 homolog isoform X1
AAEL0197;	NA	0.149772987567435	0.713567531105384	0.919256393844567	NA	NA	NA	XP_021699678.1Down syndrome cell adhesion molecule-like protein Dscam2 isoform X4
AAEL0232:	NA	0.149994854024254	0.217345411216921	0.650018359996468	NA	NA	NA	XP_021711612.1uncharacterized protein LOC5578603
AAEL0122;	protein brambleberry	0.150054611257649	0.455999586902143	0.832617462606238	NA	NA	KXJ78781.1 hypothet	XP_001662331.2protein brambleberry
AAEL0013:	charged multivesicular body protein 2a	0.150166436652014	0.327406754624136	0.75045132093389	NA	aag04144	KFB42764.1 AGAP00	XP_001653066.1charged multivesicular body protein 2a
AAEL0055!	trypsin epsilon	0.150292563325992	0.279205685508128	0.713604060506006	NA	NA	XP_001847488.1 tryp	XP_001651142.1trypsin epsilon
AAEL0035;	transportin-3	0.15035749797571	0.184216431927258	0.611957279573706	NA	NA	KXJ70442.1 hypothet	XP_001663941.1transportin-3 isoform X2
AAEL0027€	antichymotrypsin-2	0.150367074179618	0.436868850561923	0.820960220424486	NA	NA	AAV90672.1 salivary	ABF18509.1salivary serpin
AAEL0120;	probable 39S ribosomal protein L45, mitochondrial	0.150429149882125	0.341393843396464	0.762180211699347	NA	NA	KXJ73294.1 hypothet	XP_001655824.1probable 39S ribosomal protein L45, mitochondrial
AAEL0143€	activating signal cointegrator 1	0.150621237985229	0.556908061061494	0.876835490527944	NA	NA	NA	XP_001648636.2activating signal cointegrator 1
AAEL0064!	zinc finger protein Xfin	0.150792797481331	0.540723553101703	0.869735874782513	NA	NA	KXJ76171.1 hypothet	XP_001657842.2zinc finger protein Xfin
AAEL0257€	NA	0.150887921445126	0.431483039426046	0.820005280371151	NA	NA	NA	XP_021708963.1ribosomal RNA processing protein 36 homolog
AAEL0063⁄	BAG family molecular chaperone regulator 2	0.15100168491261	0.283394355529585	0.715753918648043	NA	aag04141	XP_001847574.1 con	XP_001651926.1BAG family molecular chaperone regulator 2 isoform X2
AAEL0117⁄	U4/U6 small nuclear ribonucleoprotein Prp3	0.151057058016529	0.505961834953643	0.858744381072989	NA	aag03040	ETN68110.1 Trisn sm	XP_001661880.1U4/U6 small nuclear ribonucleoprotein Prp3
AAEL0107!	uncharacterized LOC5573878	0.151204583092298	0.247441898221244	0.68058844480365	NA	NA	NA	XP_001654910.1uncharacterized protein LOC5573878 isoform X1
AAEL0107!	prenylated Rab acceptor protein 1	0.151268767205291	0.17830354100355	0.603965932107854	NA	NA	ETN61824.1 prenylat	XP_001654904.1prenylated Rab acceptor protein 1
AAEL0279€	NA	0.15135941448472	0.510007606447819	0.860328138319493	NA	NA	NA	XP_021704907.1peptidoglycan-recognition protein LE
AAEL0049:	protein bicaudal D	0.151460919193604	0.269994022122266	0.706020173790591	NA	NA	XP_019529418.1 PRE	XP_001650055.2protein bicaudal D
AAEL0052€	hydroxymethylglutaryl-CoA synthase 1	0.151496018887829	0.321317749889908	0.746588710355934	00900 ; 00072 ; 00280 ;	aag01100 ; aag	KXJ80783.1 hypothet	XP_001650491.1hydroxymethylglutaryl-CoA synthase 1
AAEL0115€	probable splicing factor, arginine/serine-rich 7	0.151566403727818	0.312581615005909	0.744018629406202	NA	NA	ETN64825.1 Srp54 [A	XP_001661752.1probable splicing factor, arginine/serine-rich 7
AAEL0110!	complement component 1 Q subcomponent-binding protein, mitochond	0.151992547495318	0.17461778745493	0.598225197379102	NA	NA	XP_001850674.1 con	XP_001661410.2complement component 1 Q subcomponent-binding protein, mitochondrial
AAEL0216€	NA	0.152050327414777	0.697235494058848	0.916312126946326	NA	NA	NA	XP_021703715.1beta-1,4-glucuronyltransferase 1 isoform X2
AAEL0025:	glutamate receptor ionotropic, kainate 2	0.15228615280663	0.522375732306543	0.865018698932415	NA	NA	KFB52145.1 AGAP00	XP_021697667.1glutamate receptor ionotropic, kainate 2 isoform X2
AAEL0243!	NA	0.1523652094165	0.33924760651685	0.761115461750945	NA	NA	NA	XP_021712014.1transmembrane emp24 domain-containing protein 7-like
AAEL0022:	ATR-interacting protein mus304	0.152378954914727	0.45576469885482	0.832617462606238	NA	NA	KXJ74954.1 hypothet	XP_001654988.2ATR-interacting protein mus304
AAEL0019⁄	uncharacterized LOC5572996	0.152488982995964	0.454282699242514	0.832617462606238	NA	NA	KXJ77027.1 hypothet	XP_001654324.2uncharacterized protein LOC5572996
AAEL0126:	vasorin	0.152531876191593	0.628792343394994	0.901045385683245	NA	NA	KXJ82890.1 hypothet	XP_019548652.1 chondroadherin-like
AAEL0064⁄	ras-interacting protein RIP3	0.152573477267065	0.380415289098422	0.786294135283846	NA	NA	XP_019930974.1 PRE	XP_021703527.1ras-interacting protein RIP3
AAEL0020€	protein nessun dorma	0.152604817978799	0.552670237184901	0.874531710113044	NA	NA	KXJ69138.1 hypothet	XP_001660798.1protein nessun dorma isoform X1
AAEL0098!	leucine-rich repeat transmembrane neuronal protein 3	0.152818623655154	0.289709995221581	0.721952579380462	NA	NA	NA	XP_021706029.1leucine-rich repeat transmembrane neuronal protein 3 isoform X2
AAEL0052:	uncharacterized LOC5566165	0.152928196587359	0.335300516385129	0.757775574591595	NA	NA	KXJ83638.1 hypothet	XP_021704954.1uncharacterized protein LOC5566165
AAEL0173:	flocculation protein FLO11	0.153034687102598	0.293987359489147	0.724764746928686	NA	NA	NA	XP_011493129.2flocculation protein FLO11
AAEL0270!	NA	0.153086842247648	0.501119534863482	0.858744381072989	NA	NA	NA	XP_021693950.1collagen alpha chain CG42342 isoform X7
AAEL0123!	uncharacterized LOC5576203	0.153281582334771	0.327426570676007	0.75045132093389	NA	NA	NA	XP_021693718.1uncharacterized protein LOC5576203
AAEL0227;	NA	0.153334409977674	0.465880044715942	0.838483018891546	NA	NA	NA	XP_021700284.1tudor domain-containing protein 5 isoform X2
AAEL0217€	NA	0.153390413540762	0.273178012238745	0.70830771164298	NA	NA	NA	NA
AAEL0110;	NA	0.153487892810614	0.372678523307421	0.781817769662763	NA	NA	CRK89802.1 CLUMA_	XP_001661299.1V-type proton ATPase subunit d 1
AAEL0017€	serine protease SP24D	0.153501169784808	0.349246629976814	0.767244408961813	NA	NA	NA	XP_001659969.2serine protease SP24D
AAEL0066!	apyrase	0.153508578541224	0.353192944210551	0.770161486404145	00230 ; 00240	aag01100 ; aag	KXJ69928.1 hypothet	XP_019553166.1 apyrase
AAEL0132!	uncharacterized LOC5577588	0.153666041316021	0.538533131612927	0.869670565558805	NA	NA	NA	XP_001656581.2uncharacterized protein LOC5577588
AAEL0116;	plasma kallikrein	0.153668767694219	0.273046772269971	0.70830771164298	NA	NA	XP_019557176.1 PRE	XP_001661778.2plasma kallikrein
AAEL0249€	NA	0.153744416044397	0.2173556285274	0.650018359996468	NA	NA	NA	XP_019532292.1 egl nine homolog 2
AAEL0018!	uncharacterized LOC5572641	0.153864257265416	0.409998717255575	0.805356078752463	NA	NA	KXJ70371.1 hypothet	XP_001654139.1uncharacterized protein LOC5572641
AAEL0142€	deoxycytidylate deaminase	0.153886358347058	0.535673322256145	0.867370387366011	240	aag01100 ; aag	NA	XP_001648345.1deoxycytidylate deaminase
AAEL0011€	ribosome biogenesis protein BOP1 homolog	0.153894607821314	0.244178048218388	0.679240145013785	NA	NA	KXJ81945.1 hypothet	XP_001658204.2ribosome biogenesis protein BOP1 homolog
AAEL0150;	membrane-bound alkaline phosphatase	0.153907204315443	0.476495459737925	0.844867278451347	00790 ; 00730	NA	NA	XP_001650469.2membrane-bound alkaline phosphatase
AAEL0060!	nuclear RNA export factor 2	0.153991909176089	0.390528953753301	0.793285976044306	NA	aag03013 ; aag	KXJ75486.1 hypothet	XP_001651793.1nuclear RNA export factor 2
AAEL0170⁄	uncharacterized LOC23687462	0.154128616140168	0.164115374284287	0.582410209858854	NA	NA	NA	XP_021696794.1uncharacterized protein LOC23687462
AAEL0121:	allatostatins MIP	0.154187975654536	0.484997267838327	0.847655581875201	NA	NA	XP_001842234.1 B-ty	XP_001655873.1allatostatins MIP
AAEL0076:	protein FAM160B1	0.154288758037007	0.362155979805611	0.777001474975806	NA	NA	KXJ75560.1 hypothet	XP_021704456.1protein FAM160B1
AAEL0114€	clavesin-1	0.154474448590089	0.382704634981711	0.788722967771939	NA	NA	KXJ71483.1 hypothet	XP_019527185.1 clavesin-2-like
AAEL0143:	uncharacterized LOC5564096	0.154592133289878	0.487793456551915	0.848846834445739	NA	NA	NA	XP_021696524.1uncharacterized protein LOC5564096 isoform X1
AAEL0252:	NA	0.154602781081134	0.531801942020834	0.866700945213732	NA	NA	NA	XP_021707983.1mitochondrial genome maintenance exonuclease 1
AAEL0036:	uncharacterized LOC5578668	0.15483203433024	0.519503170457588	0.864080221844267	NA	NA	KXJ80826.1 hypothet	XP_001657065.1uncharacterized protein LOC5578668 isoform X1
AAEL0051{	leucine-rich repeat-containing protein 59	0.155042631407067	0.531927536697695	0.866700945213732	NA	NA	XP_019538680.1 PRE	XP_001650501.2leucine-rich repeat-containing protein 59
AAEL0103€	protein anon-73B1	0.15511831112266	0.496003346966758	0.854563339971561	NA	NA	NA	XP_001654432.1protein anon-73B1
AAEL0066!	RWD domain-containing protein 4	0.155140426028931	0.473281999452666	0.843301826204891	NA	NA	KFB37093.1 AGAP01	XP_001652156.1RWD domain-containing protein 4
AAEL0127;	NA	0.155176845147037	0.702658270905089	0.917106722424789	NA	NA	XP_001842718.1 pro	XP_001662884.2aminopeptidase N
AAEL0002:	glyoxalase domain-containing protein 4	0.155316055021687	0.245595270019512	0.679727114240672	NA	NA	KFB51801.1 AGAP00	XP_001660129.1glyoxalase domain-containing protein 4
AAEL0042⁄	sialin	0.155338079845953	0.320379634128275	0.746362884366679	NA	NA	KXJ81973.1 hypothet	XP_019539846.1 sialin-like
AAEL0228€	NA	0.155645097641033	0.315198416479543	0.745618464895825	NA	NA	NA	XP_021699699.1leucine-rich repeat flightless-interacting protein 2 isoform X1
AAEL0043{	uncharacterized LOC5564638	0.155791182908057	0.415432077745145	0.809573481450831	NA	NA	XP_001850514.1 con	XP_001648988.2uncharacterized protein LOC5564638
AAEL0067!	tetratricopeptide repeat protein 37	0.155987423656171	0.517827748051539	0.862140571299125	NA	aag03018	KXJ71399.1 hypothet	XP_021704391.1tetratricopeptide repeat protein 37
AAEL0122€	U3 small nucleolar RNA-interacting protein 2	0.156113425203615	0.428177138108593	0.816509970108666	NA	NA	KXJ70058.1 hypothet	XP_021695966.1U3 small nucleolar RNA-interacting protein 2
AAEL0231!	NA	0.15612256357213	0.201688778507288	0.634701781910801	NA	NA	NA	XP_021702613.1CCAAT/enhancer-binding protein zeta
AAEL0079⁄	selT-like protein	0.156227113868579	0.176480977225611	0.601473315714841	NA	NA	KFB52536.1 selenopr	XP_001658760.1selT-like protein
AAEL0093{	transaldolase	0.156527970658213	0.148725375287643	0.563197902632466	30	aag01100 ; aag	NA	XP_019539685.1 probable transaldolase
AAEL0198⁄	NA	0.156543779928073	0.235907044785425	0.670211987068687	NA	aag01100 ; aag	NA	XP_021706254.1inositol-trisphosphate 3-kinase A isoform X2
AAEL0107;	zinc carboxypeptidase A 1	0.156600409720002	0.503765312246249	0.858744381072989	NA	NA	KXJ79110.1 hypothet	XP_001654886.2zinc carboxypeptidase A 1
AAEL0117€	DNA-directed RNA polymerase III subunit RPC5	0.156622766841493	0.474741812956559	0.844332076309675	NA	aag03020	KXJ82267.1 hypothet	XP_001655645.1DNA-directed RNA polymerase III subunit RPC5 isoform X2
AAEL0011;	cryptochrome-2	0.156697545691408	0.509066871982461	0.860083070787677	NA	NA	KXJ68201.1 hypothet	XP_019549386.1 cryptochrome-2
AAEL0093€	WD repeat-containing protein 36	0.156699565459689	0.400241695116998	0.798648539435638	NA	aag03008	NA	XP_001659920.2WD repeat-containing protein 36
AAEL0085!	zinc carboxypeptidase	0.15687026958985l	0.244870445531325	0.679727114240672	NA	NA	KXJ71765.1 hypothet	XP_001653329.2zinc carboxypeptidase
AAEL0084;	folliculin	0.15698755332332	0.450298094469397	0.829854816242881	NA	aag04150	KXJ71555.1 hypothet	XP_019537850.1 folliculin
AAEL0105!	uncharacterized LOC5573532	0.157060751634873	0.206792853823409	0.639632887691767	NA	NA	ETN60730.1 hypothe	XP_001660923.1uncharacterized protein LOC5573532
AAEL0075⁄	ovarian-specific serine/threonine-protein kinase Lok	0.157141738304208	0.426791784718676	0.8154667341939	NA	NA	XP_001850809.1 seri	XP_001658430.1ovarian-specific serine/threonine-protein kinase Lok isoform X1
AAEL0257:	NA	0.157222227641346	0.367216256773715	0.780578423384551	NA	NA	NA	XP_021693631.1uncharacterized protein LOC5576567 isoform X2
AAEL0062{	juvenile hormone acid O-methyltransferase	0.157342926889673	0.566852075024018	0.880302387766415	NA	aag00981	KXJ72554.1 hypothet	XP_001651876.1juvenile hormone acid O-methyltransferase

AAEL00404	serine/threonine-protein phosphatase 4 catalytic subunit	0.157548962220527	0.23657610906287	0.670870063357945	04660 ; 04658	NA	KRT82643.1 Calcineu	XP_001648308.1serine/threonine-protein phosphatase 4 catalytic subunit
AAEL00821	NA	0.157637704172606	0.448966498422885	0.829306073695304	62	NA	KXJ75141.1 hypothet	XP_001659028.2elongation of very long chain fatty acids protein 4
AAEL02126	NA	0.157654677626433	0.216863196384159	0.649748124914163	04070 ; 00562	aag01100 ; aag	NA	XP_019552722.1 synaptojanin-1-like
AAEL02120	NA	0.15771414506320	0.567556019741934	0.880302387766415	NA	NA	NA	XP_021697492.1non-homologous end-joining factor 1
AAEL02645	NA	0.157727712668944	0.304469233999108	0.737814869687351	NA	NA	NA	XP_021701816.12',5'-phosphodiesterase 12
AAEL02026	NA	0.157743023192528	0.323327888594311	0.747553575589157	NA	NA	NA	XP_021697664.1DDB1- and CUL4-associated factor 8
AAEL01439	dual specificity protein phosphatase 18	0.157760621880535	0.531788793755231	0.866700945213732	NA	NA	NA	XP_021697774.1dual specificity protein phosphatase 18
AAEL01835	NA	0.1578689486585	0.459454074706628	0.833904614487257	NA	NA	NA	XP_021701107.1flocculation protein FLO11 isoform X1
AAEL01220	protein split ends	0.157894231166863	0.289672238678901	0.721952579380462	NA	NA	NA	XP_021698277.1protein split ends isoform X4
AAEL00110	probable prefoldin subunit 6	0.157916687615525	0.196250126605608	0.629450562922554	NA	NA	ETN64552.1 prefoldi	XP_001652434.1probable prefoldin subunit 6
AAEL00395	uncharacterized LOC5579807	0.15791864389374	0.290062505515551	0.721952579380462	901	NA	KXJ68116.1 hypothet	XP_001648120.2uncharacterized protein LOC5579807
AAEL00355	aquaporin AQPcic	0.157926798164199	0.212816442689359	0.646249117936982	NA	NA	XP_019536730.1 PRE	XP_001656932.1aquaporin AQPcic isoform X2
AAEL00130	nuclear hormone receptor FTZ-F1 beta	0.15795175934205	0.44346252067482	0.82545872799161	NA	NA	NA	XP_001653014.2nuclear hormone receptor FTZ-F1 beta isoform X2
AAEL00100	CD2-associated protein	0.157957433579501	0.389173992298841	0.793285976044306	NA	NA	XP_001848823.1 dab	XP_021713139.1CD2-associated protein
AAEL01483	DNA-directed RNA polymerases I, II, and III subunit RPABC5	0.15808021413824	0.252486780915608	0.684556086619159	NA	aag03020	NA	KOX77626.1DNA-directed RNA polymerases I, II, and III subunit RPABC5
AAEL00455	glycolipid transfer protein	0.15832253569956	0.331414902012465	0.754128446928769	NA	NA	KXJ74558.1 hypothet	XP_001649389.2glycolipid transfer protein
AAEL01036	putative tRNA pseudouridine synthase Pus10	0.158408728245805	0.467046928762995	0.839144816051655	NA	NA	NA	XP_001654471.1putative tRNA pseudouridine synthase Pus10
AAEL01325	uncharacterized LOC5577538	0.158508047573759	0.392198878315561	0.793341218606811	NA	NA	NA	XP_001846506.1TBC1 domain family
AAEL00152	protein FAM117B	0.158540805574393	0.246811931759051	0.680385311739523	NA	NA	KXJ69918.1 hypothet	XP_001659539.2protein FAM117B
AAEL01139	NA	0.158573553632053	0.248691403213587	0.680886132809113	NA	NA	XP_001846685.1 con	XP_001661638.1protein MEMO1
AAEL00041	lipid droplet-associated hydrolase	0.158706343920776	0.335769628307933	0.757849428425215	NA	NA	KXJ69269.1 hypothet	XP_001656423.1lipid droplet-associated hydrolase isoform X2
AAEL01950	NA	0.158726624652145	0.504965266750354	0.858744381072989	NA	NA	NA	XP_021705962.1probable cytochrome P450 9f2
AAEL00373	putative ankyrin repeat protein RF_0381	0.158885660423579	0.434859567504171	0.820960220424486	NA	NA	KXJ73550.1 hypothet	XP_019560093.1 alpha-latroinsectotoxin-Lt1a-like
AAEL00835	protein spinster	0.158951123097699	0.264508411206949	0.698659663885272	NA	NA	XP_019547727.1 PRE	XP_021696331.1protein spinster isoform X1
AAEL00021	serine/threonine-protein kinase Tao	0.158952748376787	0.326929844925281	0.750039499626635	NA	NA	XP_019538537.1 PRE	XP_001658893.2serine/threonine-protein kinase Tao isoform X2
AAEL01212	F-box/LRR-repeat protein 15	0.158989076002093	0.542612126263128	0.869900911343623	NA	NA	KXJ80693.1 hypothet	XP_021702948.1F-box/LRR-repeat protein 15
AAEL00951	zinc finger protein OZF	0.159033396374142	0.598727256191922	0.890782249228467	NA	NA	NA	XP_021710308.1zinc finger protein OZF
AAEL01373	acyl-CoA synthetase family member 3, mitochondrial	0.159050197023918	0.163594683069253	0.581632574875608	NA	aag01100 ; aag	NA	XP_021711359.1acyl-CoA synthetase family member 3, mitochondrial
AAEL00216	UPF0184 protein AAEL002161	0.15923556069641	0.310829700218529	0.742918550320373	NA	NA	KXJ69690.1 hypothet	XP_001654810.1UPF0184 protein AAEL002161
AAEL02309	NA	0.159325695595364	0.692965027697146	0.916122517126697	NA	aag03460	NA	XP_021709481.1Fanconi anemia group D2 protein homolog
AAEL00057	probable trafficking protein particle complex subunit 13 homolog	0.15943038003302	0.523527578559706	0.865215478807089	NA	NA	KXJ68972.1 hypothet	XP_001648651.1probable trafficking protein particle complex subunit 13 homolog
AAEL01068	trehalose-phosphate phosphatase B	0.159534639627466	0.205820542077421	0.638023927878659	NA	NA	KXJ74000.1 hypothet	XP_001661021.2trehalose-phosphate phosphatase B
AAEL01005	retinol-binding protein pinta	0.159548658699483	0.444073305006621	0.825840341231698	NA	NA	NA	XP_001654175.2retinol-binding protein pinta
AAEL01348	protein MTO1 homolog, mitochondrial	0.159573148951257	0.405903275709577	0.802044651724185	NA	NA	NA	XP_021695962.1protein MTO1 homolog, mitochondrial
AAEL00186	GMP synthase [glutamine-hydrolyzing]	0.159624155605462	0.176573237198924	0.601511466248332	00983 ; 00230	aag01100 ; aag	KXJ77721.1 hypothet	XP_021700844.1GMP synthase [glutamine-hydrolyzing] isoform X2
AAEL00737	synaptic vesicle glycoprotein 2B	0.159668997618498	0.438328509775297	0.82148654724079	NA	aag04512	KXJ83668.1 hypothet	XP_021700163.1synaptic vesicle glycoprotein 2B
AAEL01711	hrp65 protein	0.159838701592722	0.178406685796935	0.604009684585502	NA	NA	NA	XP_011493272.1hrp65 protein isoform X2
AAEL00974	nitric oxide synthase	0.159911477537447	0.548695035643846	0.873169431463929	00460 ; 00330 ; 00220	aag01100 ; aag	NA	XP_021703595.1nitric oxide synthase isoform X1
AAEL01046	heterogeneous nuclear ribonucleoprotein 87F	0.15998022337136	0.141329862150228	0.552330595273689	NA	aag03040	NA	XP_021712996.1heterogeneous nuclear ribonucleoprotein 87F
AAEL02206	NA	0.160153445466831	0.606964278567833	0.893547770777888	NA	NA	NA	XP_001652914.1regulator of chromosome condensation
AAEL00590	DALR anticodon-binding domain-containing protein 3	0.160466646645206	0.670054637755316	0.912812708172959	NA	NA	KXJ83872.1 hypothet	XP_001651586.1DALR anticodon-binding domain-containing protein 3
AAEL01117	ras-related protein Rab5	0.160497428851945	0.36171172713831	0.776410383281253	NA	NA	ETN65323.1 low-Mr	XP_001661468.1ras-related protein Rab5
AAEL00277	ribokinase	0.160675929975663	0.371794190235554	0.781817769662763	30	aag01100 ; aag	ETN66586.1 ribokina	XP_019537351.1 ribokinase-like
AAEL01059	regulator of nonsense transcripts 2	0.160831370894745	0.390610159833837	0.793285976044306	NA	aag03013 ; aag	XP_001851054.1 con	XP_001660932.2regulator of nonsense transcripts 2
AAEL01829	NA	0.160849978962098	0.471309402856443	0.841643532909954	NA	NA	NA	XP_021711398.1zinc finger protein 37
AAEL02736	NA	0.160894310379789	0.455569264665342	0.832617462606238	NA	NA	NA	XP_021710666.1leucine-rich repeat-containing protein 57
AAEL02519	NA	0.160897280450514	0.456885781415022	0.83269220847317	NA	NA	NA	XP_021693425.1integrator complex subunit 5
AAEL00621	tonsoku-like protein	0.160905669060851	0.516315477286512	0.861448202130317	NA	NA	KXJ78777.1 hypothet	XP_021696489.1tonsoku-like protein
AAEL00142	poly(A)-specific ribonuclease PARN	0.160925903877707	0.473883104566911	0.843946647614615	NA	aag03018	KXJ67919.1 hypothet	XP_001659202.1poly(A)-specific ribonuclease PARN isoform X1
AAEL00468	beta-1,4-mannosyltransferase egh	0.161109681324398	0.168319681160269	0.588255870124114	NA	aag04320	KFB37249.1 AGAP00	XP_021701766.1beta-1,4-mannosyltransferase egh
AAEL01033	alpha-tocopherol transfer protein	0.161167811687507	0.38144516086203	0.787361680182234	NA	NA	NA	XP_001654468.1alpha-tocopherol transfer protein
AAEL01306	WD repeat domain phosphoinositide-interacting protein 2	0.161598584384798	0.269413598006752	0.705504817546424	NA	aag04140 ; aag	NA	XP_021701709.1WD repeat domain phosphoinositide-interacting protein 2 isoform X2
AAEL00825	uncharacterized LOC5570316	0.161711478312972	0.425120295673906	0.814623464645531	NA	NA	KFB42819.1 AGAP01	KFB42819.1AGAP013013-like protein
AAEL02467	NA	0.162106146905871	0.319541305408795	0.745684464362801	310	aag01100 ; aag	NA	XP_021710256.1histone-lysine N-methyltransferase SETD1
AAEL00240	DNA damage-binding protein 1	0.162198312636425	0.1598972269341	0.577560236664366	NA	aag04120 ; aag	XP_001864517.1 con	XP_001655231.2DNA damage-binding protein 1
AAEL01324	eukaryotic translation initiation factor 4 gamma 3	0.16221122046857	0.165681365660079	0.584439853413553	NA	aag03013	NA	XP_001656561.1eukaryotic translation initiation factor 4 gamma 3
AAEL00697	U3 small nucleolar RNA-associated protein 6 homolog	0.1622120338143	0.273104073768691	0.70830771164298	NA	aag03008	KFB39092.1 AGAP00	XP_001652439.1U3 small nucleolar RNA-associated protein 6 homolog
AAEL02062	NA	0.162262280661934	0.617499486958641	0.897318336565571	NA	NA	NA	NA
AAEL00246	endophilin-A	0.162592289789349	0.256396079291618	0.688461255220739	NA	aag04144	XP_019538898.1 PRE	XP_019538898.1 endophilin-A-like isoform X5
AAEL00170	AP-1 complex subunit gamma-1	0.162606351650151	0.272439289283239	0.707756103918734	NA	aag04142	NA	XP_021703589.1AP-1 complex subunit gamma-1 isoform X4
AAEL00827	alpha-tocopherol transfer protein-like	0.162791631640732	0.340558281244792	0.761546348403887	NA	NA	KXJ62416.1 hypothet	XP_001659077.2alpha-tocopherol transfer protein-like
AAEL02805	NA	0.162820263963675	0.191092499082081	0.62047747937661	NA	NA	NA	XP_021693375.1protein BCCIP homolog
AAEL01239	N(G),N(G)-dimethylarginine dimethylaminohydrolase 1	0.162917493312759	0.510371389476029	0.860452247709668	NA	NA	ETN58299.1 ng,ng-di	XP_001662541.1N(G),N(G)-dimethylarginine dimethylaminohydrolase 1
AAEL00342	uncharacterized LOC5578086	0.162942421411369	0.67341156388052	0.912968243848131	NA	NA	KXJ81431.1 hypothet	XP_021706803.1uncharacterized protein LOC5578086
AAEL01251	transmembrane protein 258 homolog	0.162966123867602	0.315364389802451	0.745627727379793	NA	NA	KXJ74489.1 hypothet	XP_001656116.1transmembrane protein 258 homolog
AAEL01405	nucleolar protein 14 homolog	0.163099865360205	0.63697831196268	0.902893334596658	NA	NA	NA	XP_001657340.2nucleolar protein 14 homolog
AAEL01976	NA	0.163118428708875	0.493351686785753	0.852598999444858	NA	NA	NA	XP_021707018.1selenocysteine insertion sequence-binding protein 2
AAEL01331	transmembrane and TPR repeat-containing protein CG5038	0.16333176099774	0.567484760597782	0.880302387766415	NA	NA	NA	XP_021704553.1transmembrane and TPR repeat-containing protein CG5038
AAEL01501	armadillo repeat-containing protein 8	0.163448974274727	0.249161557338703	0.681564997214846	NA	NA	NA	XP_021697851.1armadillo repeat-containing protein 8 isoform X2
AAEL01954	NA	0.163569253155788	0.568367972078960	0.880491932503467	NA	aag01100 ; aag	NA	XP_021707075.1phosphopantothenoylcysteine decarboxylase
AAEL00678	replication factor C subunit 4	0.163682970055582	0.503825715901914	0.858744381072989	NA	aag03420 ; aag	KXJ78997.1 hypothet	XP_001652243.1replication factor C subunit 4
AAEL00180	hsp70-binding protein 1	0.163700381233228	0.306648920352071	0.738847307609842	NA	aag04141	XP_019550442.1 PRE	XP_001660393.2hsp70-binding protein 1 isoform X1
AAEL01087	cell death-inducing p53-target protein 1	0.163838862609345	0.174746944653409	0.598287730234279	NA	NA	KFB48064.1 AGAP00	XP_001661132.1cell death-inducing p53-target protein 1
AAEL01414	melanization protease 1	0.163982319740386	0.333647598105402	0.755945471909813	NA	NA	NA	XP_001648091.1melanization protease 1 isoform X2
AAEL00756	DNA-directed RNA polymerases I and III subunit RPAC1	0.164025217172591	0.27174877967186	0.70679261462421	NA	aag03020	ETN57785.1 DNA-dir	XP_001658447.1DNA-directed RNA polymerases I and III subunit RPAC1
AAEL00771	viral IAP-associated factor homolog	0.164484869260963	0.245697661111349	0.679727114240672	NA	NA	XP_001849547.1 vira	XP_001652851.2viral IAP-associated factor homolog
AAEL00094	transmembrane protein 214	0.164526240496736	0.138020423963681	0.547441354014568	NA	NA	XP_019538323.1 PRE	XP_021701227.1transmembrane protein 214
AAEL00654	U3 small nucleolar RNA-associated protein 4 homolog	0.164663889621148	0.342709124450618	0.763457766092916	NA	aag03008	KXJ80661.1 hypothet	XP_001652058.1U3 small nucleolar RNA-associated protein 4 homolog
AAEL00238	BRCA2-interacting transcriptional repressor EMSY	0.164706563451161	0.302673628422937	0.735250294168217	NA	NA	KXJ71713.1 hypothet	XP_001655158.2BRCA2-interacting transcriptional repressor EMSY
AAEL00858	gametocyte-specific factor 1 homolog	0.16477455323094	0.447595574115325	0.828571638513641	NA	NA	KXJ76371.1 hypothet	XP_001653321.1gametocyte-specific factor 1 homolog
AAEL02568	NA	0.164785432535593	0.260887796857744	0.694041487911236	270	NA	NA	Q17Q32.1RecName: Full=Enolase-phosphatase E1; AltName: Full=2,3-diketo-5-methylthio-1-phosphopentane phosphatase
AAEL00118	reticulon-4-interacting protein 1 homolog, mitochondrial	0.164916607847728	0.299492775239786	0.730628981377654	NA	NA	KXJ76271.1 hypothet	XP_001658181.2reticulon-4-interacting protein 1 homolog, mitochondrial

AAEL01215	activin receptor type-1	0.164989307315806	0.272580703356661	0.707756103918734	NA	aag04350	XP_019553764.1 PRE	XP_021696047.1activin receptor type-1 isoform X1
AAEL00706	sorting nexin-14	0.16507930293918	0.27592317048521	0.711135357225659	NA	NA	KFB49972.1 AGAP01	XP_001652537.1sorting nexin-14
AAEL01042	histone RNA hairpin-binding protein	0.165191778870246	0.434770564725687	0.820960220424486	NA	NA	NA	XP_001654544.1histone RNA hairpin-binding protein
AAEL02580	NA	0.165198993625342	0.608866298360425	0.893547770777888	NA	NA	NA	XP_021694410.1C-type lectin 37Db-like
AAEL01076	serine protease inhibitor 28Dc	0.165335304156019	0.316801736361425	0.745684464362801	NA	NA	KXJ75606.1 hypothet	XP_021701288.1serine protease inhibitor 28Dc
AAEL02560	NA	0.165348194621671	0.539121883830797	0.869670565558805	NA	aag03040	NA	XP_021709179.1pre-mRNA-splicing factor SYF1
AAEL00961	spindle and kinetochore-associated protein 1	0.165529029980564	0.618446333090999	0.897318336565571	NA	NA	NA	XP_001653867.1spindle and kinetochore-associated protein 1
AAEL01985	NA	0.165701762984789	0.644947983606307	0.906554905014882	NA	NA	NA	XP_021705809.1somatomedin-B and thrombospondin type-1 domain-containing protein
AAEL01823	NA	0.165807617962457	0.411863810197655	0.806720047134699	NA	NA	NA	XP_021701220.1carbohydrate sulfotransferase 5
AAEL01049	zinc finger protein 569	0.165827083103478	0.617815908940437	0.897318336565571	NA	NA	NA	XP_021703660.1zinc finger protein 569
AAEL00923	cold shock domain-containing protein CG9705	0.165854087866029	0.475309741483222	0.84437941358363	NA	NA	NA	XP_001659860.1cold shock domain-containing protein CG9705
AAEL00163	uncharacterized LOC5571653	0.165861251033219	0.612397232076025	0.895199258072055	NA	NA	KXJ80719.1 hypothet	XP_019536876.1 protein NEDD1
AAEL00297	putative aminopeptidase W07G4.4	0.165894494966069	0.156088358099575	0.573938116672425	480	aag01100 ; aag	KXJ76319.1 hypothet	XP_021693377.1putative aminopeptidase W07G4.4
AAEL02355	NA	0.166045975851038	0.690027031640526	0.916122517126697	NA	NA	NA	XP_021696708.1mitochondrial GTPase 1
AAEL00024	PCI domain-containing protein 2 homolog	0.166138086692012	0.391452896073356	0.793285976044306	NA	NA	KXJ75976.1 hypothet	XP_001660135.1PCI domain-containing protein 2 homolog
AAEL00999	thioredoxin domain-containing protein	0.166282526107659	0.228255578712357	0.66175420142127	NA	NA	NA	XP_001660548.1thioredoxin domain-containing protein
AAEL00637	BTB/POZ domain-containing protein KCTD9	0.166292208239653	0.400810991936458	0.798648539435638	NA	NA	KXJ70463.1 hypothet	XP_001651968.2BTB/POZ domain-containing protein KCTD9
AAEL00057	thioredoxin-related transmembrane protein 1	0.166312231329751	0.157102618748182	0.574474189691589	NA	NA	XP_019544279.1 PRE	XP_001648666.1thioredoxin-related transmembrane protein 1 isoform X2
AAEL01236	uncharacterized LOC5571963	0.166378396175453	0.666313222877694	0.911964500946328	NA	NA	NA	XP_021697663.1uncharacterized protein LOC5571963
AAEL02416	NA	0.166485922808122	0.264597797070221	0.698659663885272	NA	NA	NA	NA
AAEL00300	E3 ubiquitin-protein ligase RNF13	0.16665508586055	0.13779940132812	0.547441354014568	NA	NA	XP_019549005.1 PRE	XP_021709469.1E3 ubiquitin-protein ligase RNF13 isoform X2
AAEL01355	gastrula zinc finger protein XlCGF26.1	0.166660247891562	0.446780478466325	0.82821796189507	NA	NA	NA	XP_001663745.2gastrula zinc finger protein XlCGF26.1
AAEL00075	glutamate--cysteine ligase regulatory subunit	0.166814274374628	0.187178862277677	0.614196367514984	NA	aag01100 ; aag	ETN62001.1 gamma-	XP_001650980.2glutamate--cysteine ligase regulatory subunit
AAEL00780	lamin-B receptor	0.166922848428921	0.517086440487369	0.861841953516531	NA	aag01100 ; aag	KXJ69057.1 hypothet	XP_001652910.1lamin-B receptor
AAEL02090	NA	0.166960699575995	0.291606240152535	0.722646576583698	NA	aag03018	NA	XP_021698799.1uncharacterized protein LOC5572197
AAEL00193	uncharacterized LOC5572990	0.166966812950467	0.308278500513217	0.741056393558619	NA	NA	KXJ78705.1 hypothet	XP_021705665.1uncharacterized protein LOC5572990
AAEL00370	enoyl-CoA delta isomerase 2, mitochondrial	0.167086774423143	0.295984654185393	0.72758553286062	NA	aag04146 ; aag	ETN60624.1 peroxisc	XP_001664062.1enoyl-CoA delta isomerase 2, mitochondrial
AAEL00503	ubiquitin-conjugating enzyme E2 E1	0.167305858336033	0.136291586807179	0.545589928345625	NA	aag04120	AAV90728.1 ubiquiti	XP_001650207.1ubiquitin-conjugating enzyme E2 E1 isoform X1
AAEL01263	zinc finger protein rotund	0.167641976440562	0.147430221669632	0.561952643558699	NA	NA	KXJ82887.1 hypothet	XP_001662761.1zinc finger protein rotund
AAEL00212	zinc finger protein OZF-like	0.167663920369945	0.604729393293648	0.893547770777888	NA	NA	XP_019560491.1 PRE	XP_021705715.1zinc finger protein OZF-like isoform X1
AAEL00914	uncharacterized LOC5571558	0.167734678094045	0.385979173514997	0.791224994795546	NA	NA	XP_001843672.1 con	XP_307249.3Anopheles gambiae str. PEST AGAP012635
AAEL01995	NA	0.167814970073191	0.534281728703494	0.866700945213732	NA	NA	NA	XP_021701507.1developmental protein eyes absent isoform X1
AAEL02447	NA	0.167832492958599	0.202118114901704	0.634991769657042	NA	NA	NA	XP_001662723.2titin homolog
AAEL00497	Golgi phosphoprotein 3 homolog sauron	0.168323740381122	0.132459736532825	0.539973327406722	NA	NA	KXJ83218.1 hypothet	XP_001650135.1Golgi phosphoprotein 3 homolog sauron
AAEL00658	uncharacterized LOC5568146	0.16834391774071	0.570004091259356	0.880654406945701	901	NA	KXJ82177.1 hypothet	XP_001866594.1Juvenile hormone-inducible protein
AAEL01173	insulin-degrading enzyme	0.168387246746002	0.231232523966006	0.66425254967488	NA	NA	KXJ82317.1 hypothet	XP_001661876.2insulin-degrading enzyme
AAEL01137	zinc finger protein 600	0.168456029420776	0.518804907778243	0.863455012004955	NA	NA	KXJ69360.1 hypothet	XP_001661620.1zinc finger protein 600
AAEL00521	uncharacterized LOC5563800	0.168469046299902	0.329851858228434	0.753175384470979	NA	NA	KXJ74814.1 hypothet	XP_021697714.1uncharacterized protein LOC5563800
AAEL00710	toll-like receptor Tollo	0.168481650461466	0.155801183749055	0.573702478134775	NA	NA	KXJ81829.1 hypothet	XP_001658133.2toll-like receptor Tollo
AAEL00457	SAGA-associated factor 11 homolog	0.168511312112757	0.393445075276492	0.793953421864587	NA	NA	KXJ71814.1 hypothet	XP_001649382.2SAGA-associated factor 11 homolog
AAEL00199	uncharacterized LOC5573200	0.168677461210601	0.153148584621791	0.568713993417967	NA	NA	KXJ74701.1 hypothet	XP_001660741.1uncharacterized protein LOC5573200
AAEL00025	uncharacterized LOC5573764	0.168713204428042	0.353535987171491	0.770323402046695	NA	NA	KXJ69379.1 hypothet	XP_001654793.1uncharacterized protein LOC5573764
AAEL01309	NA	0.16878958783243	0.144613505318248	0.557492030137627	NA	NA	NA	XP_001663309.2coatomer subunit alpha
AAEL00734	lipopolysaccharide-induced tumor necrosis factor-alpha factor homolog	0.168790771576832	0.413338601681836	0.808061732662297	NA	aag04142	XP_001865935.1 con	XP_021701847.1lipopolysaccharide-induced tumor necrosis factor-alpha factor homolog isoform X2
AAEL00229	small integral membrane protein 20	0.168791350116246	0.208177021368017	0.639832374753494	NA	NA	XP_001689283.1 AG	XP_001661253.1small integral membrane protein 20
AAEL00185	adenosine kinase	0.168842772183132	0.24300221675948	0.677870931254677	230	aag01100 ; aag	KXJ77213.1 hypothet	XP_001654167.1adenosine kinase
AAEL00282	uncharacterized LOC5576241	0.168964530771419	0.420697319568114	0.813636239562413	NA	NA	KXJ80660.1 hypothet	XP_021711026.1uncharacterized protein LOC5576241 isoform X1
AAEL01211	mitochondrial carrier protein Rim2	0.168972607123713	0.166107023887665	0.584439853413553	NA	NA	KXJ80695.1 hypothet	XP_021702947.1mitochondrial carrier protein Rim2
AAEL00096	NHL repeat-containing protein 2	0.169096738117785	0.195620692504539	0.628307718124525	NA	NA	XP_019531472.1 PRE	XP_001657496.1NHL repeat-containing protein 2 isoform X1
AAEL01104	ubiquinol-cytochrome-c reductase complex assembly factor 1	0.169155905866925	0.20051564846461	0.633852626991187	NA	NA	ETN63370.1 basic FG	XP_001661327.1ubiquinol-cytochrome-c reductase complex assembly factor 1
AAEL00115	ribonuclease Oy	0.169181427439307	0.163596496379242	0.581632574875608	NA	NA	KFB46641.1 AGAP00	XP_001658164.1ribonuclease Oy
AAEL01560	ceramide kinase	0.169361834366245	0.262643461913273	0.696497157500271	NA	aag00600	NA	XP_021705291.1ceramide kinase
AAEL00071	carbohydrate sulfotransferase 11	0.169479690423211	0.142681310307514	0.555160007378328	NA	aag00532	XP_001846392.1 cho	XP_001650261.2carbohydrate sulfotransferase 11
AAEL00074	isocitrate dehydrogenase [NADP] cytoplasmic	0.169481211609494	0.122844737004604	0.525286835462997	00480 ; 00020 ; 00720	aag01100 ; aag	KFB52983.1 AGAP00	XP_001650675.1isocitrate dehydrogenase [NADP] cytoplasmic
AAEL00129	patched domain-containing protein 3	0.169526799483655	0.487800379750887	0.848846834445739	NA	NA	KFB45939.1 hypothe	XP_021704287.1patched domain-containing protein 3
AAEL01831	NA	0.169617590717667	0.174766074643528	0.598287730234279	NA	NA	NA	XP_021711280.1rab-like protein 6
AAEL00433	WD repeat-containing protein 19	0.169732300050096	0.482152747588106	0.846941547268585	NA	NA	KXJ73906.1 hypothet	XP_001648929.2WD repeat-containing protein 19
AAEL00219	uncharacterized LOC5573951	0.169749287854608	0.197148268389544	0.630372589300064	NA	NA	KXJ74953.1 hypothet	XP_001654989.2uncharacterized protein LOC5573951
AAEL00007	general odorant-binding protein 72	0.170071450057573	0.593727001741227	0.888659054980931	NA	NA	KXJ69460.1 hypothet	XP_001647923.1general odorant-binding protein 72
AAEL00582	translation initiation factor eIF-2B subunit beta	0.170116676769983	0.358959370288428	0.77388983997572	270	aag03013	KXJ69967.1 hypothet	XP_001651487.2translation initiation factor eIF-2B subunit beta
AAEL00042	protein prenyltransferase alpha subunit repeat-containing protein 1-B	0.170283801255074	0.384936415671498	0.790080064171371	NA	NA	KXJ70548.1 hypothet	XP_001656447.1protein prenyltransferase alpha subunit repeat-containing protein 1-B
AAEL00469	protein unc-13 homolog 4B	0.170342752209785	0.281487207655375	0.715107379448722	NA	NA	KXJ81993.1 hypothet	XP_021705726.1protein unc-13 homolog 4B isoform X4
AAEL00616	melanization protease 1	0.170427184079647	0.360181182920504	0.774570517699107	NA	NA	XP_019551988.1 PRE	XP_001657552.2melanization protease 1
AAEL01184	NA	0.170513015838509	0.454026347246122	0.832617462606238	NA	NA	KFB50127.1 AGAP01	XP_001661978.1mitochondrial uncoupling protein Bmcp
AAEL00367	diphosphoinositol polyphosphate phosphohydrolase 1	0.170574072407954	0.134514869280055	0.542662316284181	NA	NA	KXJ67995.1 hypothet	XP_001657121.1diphosphoinositol polyphosphate phosphohydrolase 1
AAEL02174	NA	0.170603524385057	0.18938227410621	0.61732046982832	NA	NA	NA	XP_021700152.1mucin-17 isoform X1
AAEL00935	NA	0.170622369511382	0.277872405027963	0.713122992430066	NA	NA	NA	XP_001659976.1thioredoxin, mitochondrial
AAEL00909	ceramide synthase 6	0.170769696669026	0.12564342810171	0.530481545423179	NA	aag01100 ; aag	KXJ79489.1 hypothet	XP_001659711.1ceramide synthase 6
AAEL02643	NA	0.170833587765585	0.389452102103265	0.793285976044306	NA	NA	NA	XP_021697644.1uncharacterized protein LOC5572767
AAEL00537	sensory neuron membrane protein 1	0.170968180041034	0.598424490058815	0.890651059585596	NA	NA	XP_019548116.1 PRE	XP_021707212.1sensory neuron membrane protein 1 isoform X1
AAEL02656	NA	0.171052418809183	0.581726192892998	0.883632292340932	NA	NA	NA	XP_021700527.132 kDa beta-galactoside-binding lectin-like
AAEL01196	transport and Golgi organization protein 2	0.171126004405105	0.357360214430556	0.773736338358957	NA	NA	KXJ71467.1 hypothet	XP_001655770.1transport and Golgi organization protein 2
AAEL02248	NA	0.171199796782138	0.561699238357585	0.878972918598592	NA	NA	NA	XP_021711257.1nephrin isoform X6
AAEL00602	zinc finger FYVE domain-containing protein 1	0.171230095296445	0.463026457001863	0.837264939413983	NA	aag04140	XP_019548178.1 PRE	XP_001657385.1zinc finger FYVE domain-containing protein 1 isoform X1
AAEL00811	immunoglobulin domain-containing protein oig-4	0.171314136028287	0.21898568204635	0.652412844728166	NA	NA	XP_001862208.1 con	XP_001653113.1immunoglobulin domain-containing protein oig-4
AAEL00225	DNA repair protein REV1	0.171600859498876	0.622918042854756	0.898271200863489	NA	aag03460	KFB47136.1 AGAP00	XP_021696127.1DNA repair protein REV1
AAEL02272	NA	0.171616514973461	0.611812623214428	0.894913329966487	NA	NA	NA	NA
AAEL00824	girdin	0.17163355072503	0.319141075047613	0.745684464362801	NA	NA	KXJ82814.1 hypothet	XP_019543480.1 girdin
AAEL00278	zinc finger protein ZPR1	0.171871828162407	0.157101181748425	0.574474189691589	NA	NA	KXJ74313.1 hypothet	XP_001662456.2zinc finger protein ZPR1
AAEL00918	zinc finger protein 184	0.171922588276816	0.570724367795383	0.880923047085115	NA	NA	KXJ80012.1 hypothet	XP_001653713.2zinc finger protein 184
AAEL00079	uncharacterized LOC5566608	0.172065023177841	0.198219982424097	0.631696238860921	NA	NA	KFB50114.1 hypothe	XP_001651005.2uncharacterized protein LOC5566608
AAEL00629	uncharacterized LOC5567723	0.172113459324448	0.436217790816571	0.820960220424486	NA	NA	NA	XP_001657630.2uncharacterized protein LOC5567723 isoform X1

AAEL00154	probable protein BRICK1-B	0.172153688917794	0.281721592901735	0.715107379448722	NA	NA	XP_002063473.1 pro	XP_001841820.1syntaxin Interacting protein 1
AAEL00630	PX domain-containing protein kinase-like protein	0.172341163904647	0.236450482686304	0.670870063357945	NA	NA	KXJ80914.1 hypothet	XP_001651882.1PX domain-containing protein kinase-like protein
AAEL00917	galactosylgalactosylxylosylprotein 3-beta-glucuronosyltransferase I	0.172369409451227	0.514873659984248	0.861448202130317	NA	aag01100 ; aag	KXJ79571.1 hypothet	XP_001659794.1galactosylgalactosylxylosylprotein 3-beta-glucuronosyltransferase I
AAEL01969	NA	0.172388717106575	0.374902498881005	0.782198815065306	NA	NA	NA	XP_021701785.1TBC1 domain family member 24 isoform X1
AAEL01715	DNA-directed RNA polymerase III subunit RPC2	0.1724190015538	0.202896009156827	0.635896319876174	NA	aag03020	NA	XP_011493724.1DNA-directed RNA polymerase III subunit RPC2
AAEL00028	EEF1A lysine methyltransferase 2	0.172425290365513	0.316979297673333	0.745684464362801	NA	NA	ETN64596.1 hypothe	XP_001654798.1EEF1A lysine methyltransferase 2
AAEL01963	NA	0.17246865392805	0.50497860454706	0.858744381072989	NA	NA	NA	XP_021698853.1chymotrypsinogen B
AAEL02791	NA	0.172520552032843	0.484505892678634	0.847655581875201	NA	NA	NA	XP_021704767.1integral membrane protein GPR180-like
AAEL00536	transcription initiation factor IIB	0.172703913836328	0.246132111963079	0.679744205967664	NA	aag03022	KXJ72164.1 hypothet	XP_019550553.1 transcription initiation factor IIB isoform X1
AAEL02174	NA	0.172715944918929	0.613087282097088	0.895501122588404	510	NA	NA	XP_021712232.1alpha-glucosidase 2-like
AAEL02390	NA	0.17280780490974	0.463125249034685	0.837264939413983	NA	NA	NA	XP_021713293.1uncharacterized protein LOC5574044
AAEL00474	transcriptional activator hap3	0.17281587320711	0.440445404881555	0.822798752521693	NA	NA	XP_001862047.1 cca	XP_001649728.1transcriptional activator hap3
AAEL02170	NA	0.172898526042872	0.634177142598538	0.902506916959539	NA	NA	NA	NA
AAEL00058	transcription factor SOX-3	0.172998774389494	0.525326174880499	0.865680248818465	NA	NA	KXJ82519.1 hypothet	XP_001648654.2transcription factor SOX-3
AAEL01825	NA	0.173144164475422	0.331744398301202	0.754128446928769	04151 ; 05165 ; 04714 ;	NA	NA	XP_021701713.1serine/threonine-protein kinase grp
AAEL00726	uncharacterized LOC5568974	0.173156807935938	0.502377271302288	0.858744381072989	NA	NA	ETN67004.1 suppres	XP_001867567.1suppressor of ty3
AAEL00864	CWF19-like protein 1 homolog	0.173289366266468	0.397608931001111	0.797539006884967	NA	NA	KXJ78614.1 hypothet	XP_001659386.1CWF19-like protein 1 homolog
AAEL01812	NA	0.17330313117225	0.336732532418486	0.758445425575872	NA	NA	NA	XP_021705918.1uncharacterized protein LOC5579942 isoform X1
AAEL01333	serine/threonine-protein phosphatase 4 regulatory subunit 3	0.173311710424995	0.163306692191355	0.581366301644056	NA	NA	NA	XP_001663481.2serine/threonine-protein phosphatase 4 regulatory subunit 3 isoform X2
AAEL01960	NA	0.173530011499083	0.375384023081468	0.782198815065306	NA	NA	NA	XP_021701062.1protein Gawky isoform X1
AAEL00713	facilitated trehalose transporter Tret1-2 homolog	0.173545811464213	0.425894236922116	0.814623464645531	NA	NA	XP_019533136.1 PRE	XP_021700715.1facilitated trehalose transporter Tret1-2 homolog isoform X1
AAEL02200	NA	0.173668003599224	0.457364428830695	0.833069571278712	NA	NA	NA	KFB42741.1AGAP013115-like protein
AAEL00648	uncharacterized LOC5568054	0.173698572718787	0.128729018843104	0.535335481333802	NA	NA	AAR18426.1 putative	AAR18426.1putative 14.5 kDa salivary peptide
AAEL00923	uncharacterized LOC5571717	0.173772534178261	0.522041432643941	0.865018698932415	NA	NA	NA	XP_001659861.2uncharacterized protein LOC5571717
AAEL00468	ruvB-like helicase 1	0.173822904191076	0.247625033322163	0.680588444880365	NA	aag04310	ETN67049.1 pontin [.	XP_001649604.1ruvB-like helicase 1
AAEL00503	putative RNA polymerase II subunit B1 CTD phosphatase RPAP2 homolog	0.173889406315695	0.551341996890255	0.874531710113044	04660 ; 04658	NA	ETN62861.1 hypothe	XP_001650225.1putative RNA polymerase II subunit B1 CTD phosphatase RPAP2 homolog
AAEL00150	anamorsin homolog	0.174016605933468	0.186711581255368	0.614196367514984	NA	NA	KXJ80974.1 hypothet	XP_001653395.1anamorsin homolog
AAEL00878	apolipophorin-3	0.174056332635741	0.177595162299467	0.602370322299997	NA	NA	ADN52300.1 apolipo	XP_019537313.1 apolipophorin-3-like
AAEL00579	eukaryotic translation initiation factor 4E type 2	0.174060601222128	0.205016434008872	0.637782699712521	NA	aag03013 ; aag	XP_019561967.1 PRE	XP_001651468.2eukaryotic translation initiation factor 4E type 2 isoform X2
AAEL00668	guanine nucleotide-binding protein subunit gamma-e	0.174269127698457	0.150457476106941	0.566025396924239	NA	aag04745	AJC01918.1 G-protei	XP_001842064.1G-protein, gamma-subunit
AAEL01171	nucleoporin Nup43	0.174327116898918	0.480242476024477	0.845454613425911	NA	aag03013	XP_019544657.1 PRE	XP_001661858.2nucleoporin Nup43
AAEL00503	zinc finger protein 624	0.174384651563914	0.626989944535482	0.900535116157945	NA	NA	KXJ72832.1 hypothet	XP_001650212.2zinc finger protein 624
AAEL00810	pre-mRNA 3'-end-processing factor FIP1	0.17446112957457 3	0.33277036783186	0.755341125996244	NA	aag03015	KXJ83845.1 hypothet	XP_001653120.1pre-mRNA 3'-end-processing factor FIP1
AAEL02076	NA	0.174467502961651	0.389942502031755	0.793285976044306	NA	aag03040	NA	XP_021701917.1U4/U6.U5 tri-snRNP-associated protein 1
AAEL01061	kinesin-like protein costa	0.174652088141188	0.431941988479489	0.820005280371151	NA	aag04341	KXJ75957.1 hypothet	XP_001660954.2kinesin-like protein costa
AAEL01208	probable serine/threonine-protein kinase kinX	0.17468574327875	0.400176889704966	0.798648539435638	NA	NA	KXJ71354.1 hypothet	XP_021705391.1probable serine/threonine-protein kinase kinX isoform X2
AAEL00480	coiled-coil and C2 domain-containing protein 1-like	0.17510898989126	0.241879024291395	0.676742771771529	NA	NA	XP_019544155.1 PRE	XP_001649841.1coiled-coil and C2 domain-containing protein 1-like isoform X1
AAEL01142	zinc finger protein 879	0.175177413646044	0.652463299563066	0.907272485895905	NA	NA	ERL94190.1 hypothe	XP_021709596.1zinc finger protein 879
AAEL00120	aspartate--tRNA ligase, mitochondrial	0.175272983295475	0.403688159363512	0.800429773223221	NA	aag00970	XP_001847765.1 asp	XP_021703031.1aspartate--tRNA ligase, mitochondrial isoform X1
AAEL02257	NA	0.175473782554975	0.213536072847761	0.646580764180685	NA	NA	NA	NA
AAEL01066	integrator complex subunit 9	0.175500856040922	0.507406555786987	0.859406428557307	NA	NA	ETN59597.1 cleavage	XP_001660997.1integrator complex subunit 9
AAEL00937	ras-like protein 3	0.175556902302243	0.126630029015182	0.530481545423179	NA	NA	NA	XP_019557365.1 ras-like protein 3
AAEL00552	uncharacterized LOC5579825	0.175729463465163	0.515923460152134	0.861448202130317	NA	NA	NA	XP_021694561.1uncharacterized protein LOC5579825 isoform X4
AAEL02577	NA	0.175736122298574	0.570652822292273	0.880923047085115	NA	NA	NA	XP_021701853.1SET and MYND domain-containing protein 4
AAEL00981	sideroflexin-2	0.175759064810691	0.132021812095561	0.538981683630591	NA	NA	NA	ABF18481.1mitochondrial sideroflexin tricarboxylate transporter
AAEL00050	uncharacterized LOC5578455	0.175790764002217	0.591105053763701	0.888297094263466	NA	NA	KXJ82409.1 hypothet	XP_001656976.1uncharacterized protein LOC5578455
AAEL00428	ATPase inhibitor, mitochondrial	0.175830641870386	0.13591873062408	0.545589928345625	NA	NA	ETN58173.1 mitocho	XP_001648854.1ATPase inhibitor, mitochondrial
AAEL02257	NA	0.176011726256039	0.598983796332312	0.890875851600119	NA	NA	NA	XP_021712107.1zinc finger protein 91
AAEL00860	zinc carboxypeptidase	0.17602601905704	0.229501187722637	0.662487607456997	NA	NA	KXJ71766.1 hypothet	XP_001653330.2zinc carboxypeptidase
AAEL01455	2-oxo-4-hydroxy-4-carboxy-5-ureidoimidazoline decarboxylase	0.176091864966721	0.157546824642084	0.574839762234915	230	aag01100 ; aag	NA	XP_001649017.12-oxo-4-hydroxy-4-carboxy-5-ureidoimidazoline decarboxylase
AAEL01959	NA	0.176173148856408	0.555004448891592	0.875711752177714	NA	NA	NA	XP_019559006.1 kelch repeat and BTB domain-containing protein 4-like isoform X1
AAEL02613	NA	0.176364025150256	0.493656704575356	0.852730548401194	NA	NA	NA	NA
AAEL02405	NA	0.176486518323775	0.141105945347825	0.552330595273689	NA	aag04350	NA	XP_021692944.1activin receptor type-2A
AAEL00012	retinoblastoma-like protein 2	0.176762407376989	0.132322152087092	0.539968267629898	NA	aag04350	XP_001867835.1 reti	XP_021697604.1retinoblastoma-like protein 2
AAEL01304	glucose dehydrogenase [FAD, quinone]	0.176773700086162	0.393565765257997	0.793953421864587	NA	NA	NA	XP_001663267.1glucose dehydrogenase [FAD, quinone]
AAEL00238	esterase B1	0.176858319183408	0.225400336857064	0.658262697942846	NA	NA	XP_001841717.1 carl	XP_001655126.3esterase B1
AAEL01252	5-methylcytosine rRNA methyltransferase NSUN4	0.17705262161689	0.40289213852184	0.799923085895579	NA	NA	KXJ77388.1 hypothet	XP_001662635.15-methylcytosine rRNA methyltransferase NSUN4
AAEL00665	iron-sulfur cluster assembly 2 homolog, mitochondrial	0.177072330930523	0.21107747013654	0.643371914491948	NA	NA	KXJ69085.1 hypothet	XP_001652138.1iron-sulfur cluster assembly 2 homolog, mitochondrial
AAEL01217	S-methyl-5'-thioadenosine phosphorylase	0.177176624056085	0.204958917436872	0.637782699712521	NA	aag01100 ; aag	E3XFR6.2 RecName: I	XP_001655913.1S-methyl-5'-thioadenosine phosphorylase
AAEL00806	GPI ethanolamine phosphate transferase 1	0.177198626089833	0.361020707867851	0.77557561132884	NA	aag01100 ; aag	XP_019532069.1 PRE	XP_001658859.2GPI ethanolamine phosphate transferase 1
AAEL00123	HEAT repeat-containing protein 1 homolog	0.177400475691803	0.314244853581911	0.74451110779113	NA	aag03008	XP_001844163.1 bap	XP_021702400.1HEAT repeat-containing protein 1 homolog
AAEL01312	coatomer subunit zeta-1	0.177512184618739	0.164097662934074	0.582410209858854	NA	NA	NA	XP_001656371.1coatomer subunit zeta-1 isoform X1
AAEL00728	mesencephalic astrocyte-derived neurotrophic factor homolog	0.177551572231418	0.132709168445275	0.540110467949322	NA	NA	ETN62746.1 Mesenc	XP_001652674.1mesencephalic astrocyte-derived neurotrophic factor homolog
AAEL00970	persulfide dioxygenase ETHE1, mitochondrial	0.177649746585174	0.212699306293031	0.646173329979686	NA	aag01100 ; aag	NA	XP_001654008.2persulfide dioxygenase ETHE1, mitochondrial isoform X1
AAEL00485	glycerol kinase	0.177706927294439	0.377140144975457	0.783395849675881	561	aag01100 ; aag	KFB51593.1 AGAP00	XP_001649900.1glycerol kinase
AAEL01963	NA	0.177806628497655	0.367914269623109	0.781479833870814	NA	NA	NA	XP_001651120.2uncharacterized protein LOC5566712 isoform X1
AAEL02300	NA	0.177925357193624	0.61008599347146	0.894093217379261	NA	NA	NA	NA
AAEL01047	uncharacterized LOC5573375	0.177926808155359	0.214656283968247	0.648028428468258	NA	NA	NA	XP_001660864.2uncharacterized protein LOC5573375 isoform X1
AAEL00022	scavenger receptor class B member 1	0.178076886334836	0.127134364380924	0.531526770119611	NA	NA	XP_001688563.1 AG.	XP_001660152.2scavenger receptor class B member 1
AAEL00245	nuclear pore complex protein Nup153	0.178283394925714	0.20903141908439	0.639832374753494	NA	aag03013	XP_019525543.1 PRE	XP_021699158.1nuclear pore complex protein Nup153 isoform X1
AAEL01824	NA	0.17836696825129	0.207113134625981	0.639832374753494	NA	NA	NA	XP_001656142.2DNA topoisomerase 2
AAEL02816	NA	0.178399407036811	0.264557780883238	0.698659663885272	NA	aag04013 ; aag	NA	XP_021713000.1mitogen-activated protein kinase kinase kinase 4 isoform X2
AAEL00106	glutathione S-transferase 1	0.178427056030433	0.179266315884566	0.604695248815902	NA	aag01100 ; aag	AAC79994.1 glutathi	XP_001658009.2glutathione S-transferase 1 isoform X1
AAEL00086	uncharacterized LOC5567193	0.17847216616532 7	0.420665328509141	0.813636239562413	NA	NA	NA	XP_021707703.1uncharacterized protein LOC5567193 isoform X3
AAEL02011	NA	0.178650546892334	0.231282891671193	0.66425254967488	NA	NA	NA	XP_021707412.1uncharacterized protein LOC110678608
AAEL00002	protein FAM114A2	0.178911973538849	0.176447702432193	0.601473315714841	NA	NA	XP_019528878.1 PRE	XP_001647891.2protein FAM114A2
AAEL02209	NA	0.178985372786853	0.138649036315146	0.548805616375239	NA	NA	NA	XP_021703944.1transport and Golgi organization protein 1 isoform X1
AAEL01102	uncharacterized LOC5574257	0.178996083093078	0.345982892658236	0.765136884941381	NA	NA	KXJ70674.1 hypothet	XP_021695746.1uncharacterized protein LOC5574257
AAEL01230	nedd8-activating enzyme E1 catalytic subunit	0.179285534441847	0.313356730328714	0.744189298893767	NA	aag04120	ETN66743.1 ubiquiti	XP_001662412.1nedd8-activating enzyme E1 catalytic subunit
AAEL00623	GRIP and coiled-coil domain-containing protein 2	0.179301167755435	0.31889303613382	0.745684464362801	NA	NA	KXJ76211.1 hypothet	XP_021710264.1GRIP and coiled-coil domain-containing protein 2
AAEL00090	glutactin	0.17957934269603	0.339101630418121	0.761115461750945	NA	NA	KXJ82746.1 hypothet	XP_019564202.1 glutactin-like
AAEL01510	calnexin	0.179797163484588	0.128442024712534	0.535049122410476	NA	aag04141 ; aag	NA	XP_001647823.2calnexin isoform X4

AAEL0058	heat shock protein 67B2	0.179801875599039	0.38054859422199	0.786393030323546	NA	NA	XP_019539090.1 PRE	XP_001651545.2heat shock protein 67B2 isoform X2
AAEL0253	NA	0.179835745029732	0.384955542807487	0.790080064171371	NA	NA	NA	XP_021711825.1protein EFR3 homolog cmp44E isoform X1
AAEL0001	omega-amidase NIT2	0.180008554091866	0.213991664836885	0.647322709661982	NA	NA	KXJ70610.1 hypothet	XP_001657673.2omega-amidase NIT2
AAEL0037	uncharacterized protein F54F2.9	0.180077618508069	0.393062651833997	0.793953421864587	NA	aag04141	KXJ81926.1 hypothet	XP_001664189.2uncharacterized protein F54F2.9
AAEL0033	tRNA-splicing ligase RtcB homolog	0.1801157032311	0.322176541988691	0.746650319748046	NA	NA	KXJ80670.1 hypothet	XP_001656701.1tRNA-splicing ligase RtcB homolog
AAEL0040	DNA polymerase V	0.180284454759351	0.126243109187348	0.530481545423179	NA	NA	KXJ72124.1 hypothet	XP_001648288.1DNA polymerase V
AAEL0066	gamma-tubulin complex component 5	0.180301596091753	0.558819472272775	0.877581080322399	NA	NA	XP_001849107.1 con	XP_021708416.1gamma-tubulin complex component 5
AAEL0256	NA	0.180339608298254	0.409797520956443	0.805356078752463	NA	aag04330	NA	XP_021710439.1neurogenic locus protein delta isoform X1
AAEL0110	guanylate kinase	0.180373864629646	0.186998565156103	0.614196367514984	230	aag01100 ; aag	XP_019556832.1 PRE	XP_001661287.1guanylate kinase isoform X4
AAEL0087	proteasome subunit beta type-1	0.180395193866002	0.228043741917698	0.661754201421 27	NA	aag03050	KFB48418.1 AGAP00	XP_001659482.1proteasome subunit beta type-1
AAEL0129	importin subunit alpha	0.180448220483643	0.327062114635623	0.750155926938435	NA	NA	NA	XP_001663145.1importin subunit alpha
AAEL0056	RNA-binding protein NOB1	0.180462957095722	0.116809281823036	0.514582987743323	NA	aag03008	KXJ70465.1 hypothet	XP_001651182.2RNA-binding protein NOB1
AAEL0197	NA	0.180543318868834	0.318084774736897	0.745684464362801	310	aag01100 ; aag	NA	XP_021707846.1histone-lysine N-methyltransferase, H3 lysine-79 specific isoform X4
AAEL0066	exportin-4	0.180719554756533	0.410972698857532	0.805855841282375	NA	NA	ETN67739.1 exportin	XP_019551191.1 exportin-4-like
AAEL0060	probable peptidyl-tRNA hydrolase 2	0.180725295912754	0.180428224530802	0.605728027045765	NA	NA	AAF87580.1 unknow	XP_001657407.2probable peptidyl-tRNA hydrolase 2
AAEL0069	protein phosphatase PTC7 homolog	0.180802134421851	0.126538747126387	0.530481545423179	04660 ; 04658	NA	KXJ73804.1 hypothet	XP_001652369.2protein phosphatase PTC7 homolog
AAEL0236	NA	0.180881015355151	0.173507014999164	0.596418211439039	NA	NA	NA	XP_021700931.1UPF0430 protein CG31712
AAEL0072	ADP-ribosylation factor-like protein 8	0.180915747620693	0.169190698481613	0.589285695468509	NA	NA	ETN65197.1 ADP-rib	XP_001658238.1ADP-ribosylation factor-like protein 8
AAEL0047	ubiquitin-protein ligase E3B	0.181001180216998	0.313332922420961	0.744189298893767	NA	aag04120	XP_019932577.1 PRE	XP_001649826.2ubiquitin-protein ligase E3B
AAEL0016	exosome complex component CSL4	0.181188798150472	0.311675813945015	0.743434862302544	NA	aag03018	XP_001844743.1 exo	XP_001659760.2exosome complex component CSL4
AAEL0017	decaprenyl-diphosphate synthase subunit 2	0.181223629416282	0.399898453509938	0.798648539435638	NA	aag00900	KXJ81757.1 hypothet	XP_001660253.2decaprenyl-diphosphate synthase subunit 2
AAEL0136	ubiquitin-conjugating enzyme E2 H	0.181273592275605	0.243682322670413	0.678681819979763	NA	aag04120	NA	XP_019544083.1 ubiquitin-conjugating enzyme E2 H
AAEL0073	protein UBASH3A homolog	0.181352408747865	0.29873130824702	0.730450295042154	NA	NA	KXJ75426.1 hypothet	XP_021693349.1protein UBASH3A homolog isoform X2
AAEL0110	GATA zinc finger domain-containing protein 1	0.181390428752442	0.590769073521883	0.88813217032321	NA	NA	KXJ71252.1 hypothet	XP_001661303.2GATA zinc finger domain-containing protein 1
AAEL0066	baculoviral IAP repeat-containing protein 7	0.18149914127307	0.217953352007222	0.65121672666453	NA	aag04120 ; aag	XP_001869659.1 bac	XP_001657917.3baculoviral IAP repeat-containing protein 7
AAEL0021	ras-related protein Rab-10	0.18152244868902	0.122730742494177	0.525286835462997	NA	aag04144	ETN64009.1 Rab-pro	XP_011493555.1ras-related protein Rab-10
AAEL0085	transcription factor grauzone	0.181595973530454	0.64008633003504	0.904463241434479	NA	NA	KXJ73859.1 hypothet	XP_001659342.2transcription factor grauzone
AAEL0257	NA	0.181624862500288	0.183407970069718	0.610828860145329	NA	NA	NA	XP_021710222.1vanin-like protein 2
AAEL0146	nuclear receptor coactivator 5	0.181902262390133	0.371909038863676	0.781817769662763	NA	NA	NA	XP_001649256.2nuclear receptor coactivator 5
AAEL0028	extracellular serine/threonine protein CG31145	0.181937795349233	0.436829284894897	0.820960220424486	NA	NA	KXJ79682.1 hypothet	XP_001662441.1extracellular serine/threonine protein CG31145
AAEL0027	glutamyl-tRNA(Gln) amidotransferase subunit A, mitochondrial	0.182178381958748	0.305775196571109	0.738515317282768	970	aag01100 ; aag	KXJ84113.1 hypothet	XP_001655938.2glutamyl-tRNA(Gln) amidotransferase subunit A, mitochondrial
AAEL0080	carbonic anhydrase-related protein 10	0.18218783801256	0.495456249909093	0.854260523077286	NA	NA	ACM89452.1 CA-rela	XP_021704115.1carbonic anhydrase-related protein 10
AAEL0006	protein Wnt-6	0.182189943875125	0.602488488666697	0.892663752727165	NA	aag04150 ; aag	ETN65414.1 wingless	XP_021703000.1protein Wnt-6
AAEL0021	dolichyl-diphosphooligosaccharide--protein glycosyltransferase 48 kDa su	0.182206665755511	0.112337201521289	0.503547757511443	NA	aag01100 ; aag	KXJ72237.1 hypothet	XP_001654826.2dolichyl-diphosphooligosaccharide--protein glycosyltransferase 48 kDa subunit
AAEL0180	NA	0.182263116537728	0.539443449085933	0.869709828277711	NA	NA	NA	XP_021696914.1epidermal growth factor-like protein 7
AAEL0244	NA	0.182494272175752	0.421982195405828	0.814192545906728	NA	NA	NA	XP_021694740.1uncharacterized protein DDB_G0271670-like
AAEL0115	ubiquitin-protein ligase E3C	0.182752342197541	0.302200822183533	0.73489745394632	NA	aag04120	KXJ81712.1 hypothet	XP_021710999.1ubiquitin-protein ligase E3C
AAEL0130	peptide chain release factor 1-like, mitochondrial	0.182766199341761	0.283156523593564	0.715753918648043	NA	NA	NA	XP_001663262.1peptide chain release factor 1-like, mitochondrial
AAEL0135	tryptophan--tRNA ligase, cytoplasmic	0.182767320145591	0.268468245124588	0.704184172798867	970	aag00970	NA	XP_001663714.2tryptophan--tRNA ligase, cytoplasmic
AAEL0143	coiled-coil domain-containing protein 47	0.182842371395611	0.0952309752794235	0.466773927728342	NA	NA	NA	XP_001648535.1coiled-coil domain-containing protein 47
AAEL0123	putative homeodomain transcription factor	0.182953961548157	0.282986198506717	0.715753918648043	NA	NA	KXJ82541.1 hypothet	XP_021697972.1putative homeodomain transcription factor
AAEL0067	splicing factor 3B subunit 2	0.183128252845809	0.204657745445082	0.637782699712521	NA	aag03040	KXJ79495.1 hypothet	XP_021697097.1splicing factor 3B subunit 2 isoform X2
AAEL0121	NA	0.183242358899556	0.290588156343521	0.722005075789413	NA	aag01100 ; aag	XP_019554406.1 PRE	XP_001655925.2aldehyde dehydrogenase, dimeric NADP-preferring
AAEL0120	ubiquitin-like protein 7	0.18333350029922	0.328707481439856	0.75186831822261	NA	NA	KXJ81898.1 hypothet	XP_001662231.1ubiquitin-like protein 7
AAEL0114	multiple inositol polyphosphate phosphatase 1	0.183339232529355	0.177506913278276	0.602292999995093	NA	aag01100 ; aag	KXJ84255.1 hypothet	XP_001655344.2multiple inositol polyphosphate phosphatase 1
AAEL0137	39S ribosomal protein L54, mitochondrial	0.18349387964798	0.371992189647914	0.781817769662763	NA	NA	NA	XP_001657016.139S ribosomal protein L54, mitochondrial
AAEL0096	high affinity cGMP-specific 3',5'-cyclic phosphodiesterase 9A	0.183631139976471	0.320824618502226	0.746566934826029	NA	aag01100 ; aag	NA	XP_021708719.1high affinity cGMP-specific 3',5'-cyclic phosphodiesterase 9A isoform X2
AAEL0201	NA	0.183703457813292	0.634667558714413	0.902520611233199	NA	NA	NA	XP_021712394.1double-strand-break repair protein rad21 homolog
AAEL0070	mitochondrial uncoupling protein 2	0.183851601104334	0.619665226036912	0.897318336565571	NA	NA	XP_001845818.1 mit	XP_001652516.1mitochondrial uncoupling protein 2
AAEL0126	uncharacterized LOC5576661	0.183879158929686	0.242601689472119	0.677312119697343	901	NA	KXJ77777.1 hypothet	XP_001847554.1Juvenile hormone-inducible protein
AAEL0011	mRNA-decapping enzyme 1A	0.184108157314579	0.278599995412528	0.713122992430066	NA	aag03018	KXJ76786.1 hypothet	XP_001658183.2mRNA-decapping enzyme 1A
AAEL0018	putative ankyrin repeat protein RF_0381	0.184254947964528	0.20765753562357	0.639832374753494	NA	NA	NA	XP_001660394.2uncharacterized protein LOC5572449 isoform X1
AAEL0133	DCN1-like protein	0.184269035007656	0.241221747382703	0.676332272826214	NA	NA	NA	XP_001663582.2DCN1-like protein isoform X3
AAEL0243	NA	0.184301000659807	0.455780029953711	0.832617462606238	NA	NA	NA	XP_021712253.1putative defense protein Hdd11-like
AAEL0093	protein CLP1 homolog	0.184498660512256	0.48599181601292	0.847790841886424	NA	aag03015	NA	XP_001659890.2protein CLP1 homolog
AAEL0266	NA	0.184689985015545	0.479557121177891	0.845454613425911	NA	NA	NA	XP_021711845.1ras GTPase-activating protein-binding protein 1-like isoform X1
AAEL0022	transcription factor grauzone	0.184692766584204	0.415402567965687	0.809573481450831	NA	NA	KXJ81801.1 hypothet	XP_001661157.1transcription factor grauzone isoform X1
AAEL0195	NA	0.184769026523122	0.174191634770445	0.597209919209252	NA	NA	NA	XP_021695857.1septin-7 isoform X5
AAEL0199	NA	0.184784057305626	0.293512411486331	0.724246906570544	NA	NA	NA	XP_019541558.1 S phase cyclin A-associated protein in the endoplasmic reticulum-like
AAEL0119	protein-S-isoprenylcysteine O-methyltransferase	0.184880096230285	0.426339015694604	0.814868383824891	900	aag00900	KXJ73635.1 hypothet	XP_021706343.1protein-S-isoprenylcysteine O-methyltransferase
AAEL0122	general transcription factor IIE subunit 1	0.184959620493403	0.369948253526582	0.781817769662763	NA	aag03022	KXJ72607.1 hypothet	XP_001662313.1general transcription factor IIE subunit 1
AAEL0260	NA	0.185035667670032	0.505294128376109	0.858744381072989	NA	NA	NA	XP_021695189.1anillin-like
AAEL0072	BET1 homolog	0.185134484097384	0.431947005052031	0.820005280371151	NA	aag04130	ETN64339.1 hypothe	XP_001652611.1BET1 homolog
AAEL0100	proteasome subunit alpha type-4	0.185147533046207	0.136784642606828	0.545589928345625	NA	aag03050	NA	XP_001660626.1proteasome subunit alpha type-4
AAEL0133	CUE domain-containing protein 1	0.185167896171917	0.224356461103895	0.656544198985955	NA	NA	NA	XP_001663565.2CUE domain-containing protein 1 isoform X1
AAEL0105	cyclin-Y-like protein 1	0.185381391510761	0.329171043638067	0.752367596512046	NA	NA	ETN65852.1 cyclin fo	XP_001660900.1cyclin-Y-like protein 1
AAEL0053	myotubularin-related protein 2	0.185467843003571	0.240718026840543	0.675511149976115	NA	aag01100 ; aag	ETN58165.1 myotub	XP_021702361.1myotubularin-related protein 2
AAEL0073	COMM domain-containing protein 2	0.185522397468015	0.538310100121465	0.869670565558805	NA	NA	KXJ71820.1 hypothet	XP_001658322.1COMM domain-containing protein 2 isoform X1
AAEL0019	KDEL motif-containing protein 1	0.18554049667226	0.40625042274676	0.802558263227927	NA	NA	KXJ69136.1 hypothet	XP_001660800.1KDEL motif-containing protein 1
AAEL0024	endocuticle structural glycoprotein SgAbd-5	0.185897118669333	0.509805436337009	0.860302516994894	NA	NA	ETN58752.1 cuticula	XP_001655289.1endocuticle structural glycoprotein SgAbd-5
AAEL0224	NA	0.18591007519843	0.581330393905022	0.883594430630159	NA	NA	NA	XP_021701633.1augmin complex subunit dgt3
AAEL0214	NA	0.185917505739126	0.270719723302435	0.706237745304114	NA	aag03450	NA	XP_021699626.1DNA ligase 4
AAEL0010	mucin-19	0.186020110921947	0.258304477150054	0.691547675112889	NA	aag03013	XP_019526680.1 PRE	XP_019539479.1 nuclear pore complex protein Nup214
AAEL0064	GPI-anchored wall transfer protein 1	0.186037167515125	0.579247391040123	0.882283691803025	NA	aag01100 ; aag	KXJ74693.1 hypothet	XP_001657815.1GPI-anchored wall transfer protein 1
AAEL0116	uncharacterized LOC5575108	0.186038388453065	0.107750590116735	0.493785323831772	NA	NA	XP_001842846.1 tra	ABF18468.1possible transcription factor
AAEL0107	mediator of RNA polymerase II transcription subunit 21	0.186038917009681	0.324572643148274	0.748164514778316	NA	NA	ETN58654.1 Srb7p: R	XP_001654915.1mediator of RNA polymerase II transcription subunit 21
AAEL0127	uncharacterized LOC5576762	0.186134771506572	0.405131121132832	0.801349782813648	NA	NA	ETN62183.1 hypothe	XP_021704245.1uncharacterized protein LOC5576762
AAEL0233	NA	0.186462733617313	0.248102926559636	0.680588444480365	NA	NA	NA	XP_001650896.22-(3-amino-3-carboxypropyl)histidine synthase subunit 2
AAEL0073	putative phosphatidate phosphatase	0.186516373175886	0.159621180192826	0.577560236664366	NA	aag01100 ; aag	KFB48359.1 phospha	XP_001658284.1putative phosphatidate phosphatase
AAEL0097	BTB/POZ domain-containing protein 6	0.186553279707318	0.241606983303268	0.676742771771529	NA	NA	NA	XP_021710151.1kelch repeat and BTB domain-containing protein 12 isoform X1
AAEL0135	NA	0.186596526346733	0.130567734089638	0.537336444138125	NA	NA	NA	XP_001663723.2transcription elongation factor SPT5
AAEL0045	putative inositol monophosphatase 3	0.186791115557636	0.316920918290429	0.745684464362801	NA	aag01100 ; aag	KXJ74557.1 hypothet	XP_001649386.2putative inositol monophosphatase 3

AAEL00291	arf-GAP with coiled-coil, ANK repeat and PH domain-containing protein 1	0.18683922045667	0.457110106595003	0.832771174200597	NA		aag04144	KXJ82961.1 hypothet	XP_021700451.1arf-GAP with coiled-coil, ANK repeat and PH domain-containing protein 1
AAEL01298	pre-mRNA-splicing regulator female-lethal(2)D	0.186850624586657	0.187222431088843	0.614196367514984	NA		NA	NA	XP_001663177.1pre-mRNA-splicing regulator female-lethal(2)D
AAEL00121	ATP-dependent RNA helicase WM6	0.186992551611219	0.121896928361493	0.523911973275319	NA		aag03013 ; aag	KFB42913.1 Dere\GG	XP_001658306.1ATP-dependent RNA helicase WM6
AAEL00218	dolichyl-diphosphooligosaccharide--protein glycosyltransferase subunit D	0.187055261903575	0.153423968500645	0.568818435996349	NA		aag01100 ; aag	ETN66435.1 defende	XP_001654985.1dolichyl-diphosphooligosaccharide--protein glycosyltransferase subunit DAD1
AAEL01505	steroid receptor RNA activator 1	0.187064288722954	0.456858811392501	0.83269220847317	NA		NA	NA	XP_001650403.1steroid receptor RNA activator 1
AAEL02762	NA	0.187142050489046	0.596445666780499	0.889585230151692	NA		NA	NA	XP_021699405.1zinc finger protein Xfin-like
AAEL02665	NA	0.187206672406735	0.483045929651123	0.847379421050056	NA		NA	NA	XP_021697696.1lariat debranching enzyme
AAEL00716	gamma-aminobutyric acid receptor-associated protein	0.187234869529297	0.141019883908482	0.552330595273689	NA		aag04140 ; aag	PNF39469.1 Gamma-	XP_001652571.1gamma-aminobutyric acid receptor-associated protein
AAEL01820	NA	0.187574262507982	0.161639624649692	0.578890994715483	NA		NA	NA	XP_021709995.1tyrosine-protein phosphatase non-receptor type 23 isoform X1
AAEL01017	COMM domain-containing protein 4	0.18767239039201	0.60950371868532	0.893659351676678	NA		NA	NA	XP_001654285.2COMM domain-containing protein 4
AAEL00000	zinc finger protein 782	0.187715997402223	0.393611770326695	0.793953421864587	NA		NA	XP_019528894.1 PRE	XP_021705406.1zinc finger protein 782 isoform X2
AAEL00987	amyloid protein-binding protein 2	0.187770364202717	0.236779229594439	0.671032482515562	NA		NA	NA	XP_021698157.1amyloid protein-binding protein 2
AAEL00662	uncharacterized LOC5568172	0.187897507267221	0.224034940045429	0.656259917301973		901	NA	KXJ69934.1 hypothet	XP_001652108.2uncharacterized protein LOC5568172
AAEL00190	transmembrane inner ear expressed protein	0.187981917896828	0.522842255125488	0.865167133908424	NA		NA	KFB47802.1 AGAP00	KFB47802.1AGAP009155-like protein
AAEL01284	prolactin regulatory element-binding protein	0.188144047058278	0.331453027142962	0.754128446928769	NA		aag04141	NA	XP_001662958.1prolactin regulatory element-binding protein
AAEL00951	glycosylphosphatidylinositol anchor attachment 1 protein	0.188242097685174	0.185216937403962	0.612420502332469	NA		aag01100 ; aag	NA	XP_001660170.1glycosylphosphatidylinositol anchor attachment 1 protein
AAEL00135	uncharacterized LOC5570060	0.188274730994981	0.306357234769323	0.738783696372872	NA		NA	KXJ69832.1 hypothet	XP_001653049.1uncharacterized protein LOC5570060
AAEL02254	NA	0.188286616567277	0.553837250227485	0.874920236325033	NA		NA	NA	XP_021702833.1ras guanine nucleotide exchange factor V
AAEL02517	NA	0.18834777476173	0.218542131413261	0.65208323387316	NA		aag04013	NA	XP_021702670.1uncharacterized protein LOC5579501
AAEL00286	cyclin-L2	0.1885758010321	0.124185653813618	0.527832049142223	NA		NA	XP_001845603.1 cyc	XP_019546515.1 cyclin-L2
AAEL00895	ras-related protein Rab-39B	0.188610641142465	0.215243900738521	0.648208527708858	NA		aag04140	KFB38786.1 AGAP00	XP_001653610.1ras-related protein Rab-39B
AAEL01982	NA	0.188668844508956	0.129784257735758	0.536511563200559	NA		aag04068	NA	XP_021702256.1ubiquitin carboxyl-terminal hydrolase 7
AAEL00075	BSD domain-containing protein 1-A	0.188715932035515	0.0941542213605044	0.464823706598816	NA		NA	KXJ70002.1 hypothet	XP_001650970.2BSD domain-containing protein 1-A
AAEL01136	NKAP family protein CG6066	0.188720195707209	0.372727076234573	0.781817769662763	NA		NA	KXJ73350.1 hypothet	XP_001655294.1NKAP family protein CG6066
AAEL02026	NA	0.188827426352622	0.375455985067626	0.782198815065306	NA		NA	NA	XP_021701120.1protein sidekick isoform X2
AAEL00696	nucleolar protein 58	0.188997669393529	0.546077280097014	0.870931414970375	NA		NA	KOB65529.1 Fibroin-	XP_001652441.1nucleolar protein 58
AAEL00449	excitatory amino acid transporter 3	0.189056504265228	0.0908123291842696	0.458582874647784	NA		NA	XP_019537426.1 PRE	XP_021703753.1excitatory amino acid transporter 3 isoform X1
AAEL00723	mediator of RNA polymerase II transcription subunit 30	0.189218112889892	0.379727957825438	0.785755940613825	NA		NA	KFB38707.1 AGAP00	XP_001658256.1mediator of RNA polymerase II transcription subunit 30
AAEL00987	protein virilizer	0.189491901306331	0.347603198219946	0.766142426352082	NA		NA	NA	XP_001660468.2protein virilizer
AAEL01534	zinc finger protein 615	0.189496067014959	0.584950302009751	0.885175928718656	NA		NA	NA	XP_001647773.1zinc finger protein 615
AAEL00531	uncharacterized protein C1orf50 homolog	0.189536378946409	0.3455204952868	0.764941296190615	NA		NA	KXJ80580.1 hypothet	XP_001650716.1uncharacterized protein C1orf50 homolog
AAEL01701	DNA-directed RNA polymerase I subunit RPA2	0.189544318110508	0.149659009692988	0.565105542550216	NA		aag03020	NA	XP_011493248.1DNA-directed RNA polymerase I subunit RPA2
AAEL00483	KAT8 regulatory NSL complex subunit 2	0.189838692003332	0.198254590070674	0.631696238860921	NA		NA	KXJ82637.1 hypothet	XP_021697731.1KAT8 regulatory NSL complex subunit 2 isoform X2
AAEL02059	NA	0.189854007481295	0.130381844580377	0.537292312507224	NA		aag03013	NA	XP_021705721.1E3 SUMO-protein ligase RanBP2 isoform X2
AAEL00809	procollagen-lysine,2-oxoglutarate 5-dioxygenase 3	0.189856444562235	0.117606730026292	0.515237493575542	NA		aag01100 ; aag	XP_001862209.1 pro	XP_021706328.1procollagen-lysine,2-oxoglutarate 5-dioxygenase 3
AAEL00551	RNA-binding protein squid	0.189936074598396	0.124763254478454	0.529309113743998	NA		aag04320	XP_019555705.1 PRE	XP_021694535.1RNA-binding protein squid isoform X1
AAEL01482	uncharacterized LOC5565341	0.189999556690044	0.270296463465741	0.706188123755524	NA		NA	NA	XP_019537659.1 mucin-5AC
AAEL00648	uncharacterized LOC5568046	0.190046506619773	0.18903670950658	0.616603527036593	NA		NA	KXJ80819.1 hypothet	XP_021710738.1uncharacterized protein LOC5568046
AAEL02797	NA	0.190112666234045	0.398847931655475	0.798563678653977	NA		NA	NA	XP_021712732.1general transcription factor IIF subunit 2-like
AAEL02806	NA	0.19024012564839	0.34550532725807	0.764941296190615	NA		NA	NA	XP_021695197.1flocculation protein FLO11
AAEL02582	NA	0.190454279682743	0.578964313887329	0.882283691803025	NA		NA	NA	NA
AAEL00193	selenium-binding protein 1-B	0.190577140131023	0.131782200109875	0.538981683630591	NA		aag01100 ; aag	KXJ77029.1 hypothet	XP_001654322.2selenium-binding protein 1-B
AAEL00595	caspase-3	0.19059320797128	0.413132794691304	0.808061732662297	NA		aag04214	XP_019529649.1 PRE	XP_001651737.1caspase-3 isoform X1
AAEL00038	beta-1,3-galactosyltransferase brn	0.190652008076896	0.449397485386002	0.829306073695304	NA		aag04320	ETN59830.1 beta-1,3	XP_021705783.1beta-1,3-galactosyltransferase brn
AAEL01168	uncharacterized protein C18orf19 homolog A	0.190791254116782	0.273480238966375	0.708614567599462	NA		NA	KXJ83811.1 hypothet	XP_001655636.2uncharacterized protein C18orf19 homolog A
AAEL02465	NA	0.190809142507977	0.139722015360983	0.549453839893918	NA		NA	NA	XP_021703893.1maestro heat-like repeat-containing protein family member 1
AAEL01124	zinc finger protein 236	0.190904486630893	0.0936422910401838	0.464823706598816	NA		NA	NA	XP_001661515.2myb-like protein AA isoform X6
AAEL01076	protein DPCD	0.190932915197722	0.605119730669997	0.893547770777888	NA		NA	KXJ74639.1 hypothet	XP_001654855.1protein DPCD
AAEL00140	AP-3 complex subunit sigma-2	0.190956915736799	0.235655743856294	0.669872619304915	NA		aag04142	ETN62596.1 clathrin	XP_001659193.1AP-3 complex subunit sigma-2
AAEL00189	uncharacterized LOC5572802	0.191085676968986	0.427479275814098	0.815943641576713	NA		NA	KXJ75781.1 hypothet	XP_021702685.1uncharacterized protein LOC5572802
AAEL01393	uncharacterized LOC5578951	0.191265268774396	0.3336938173112	0.755945471909813	NA		NA	NA	KFB37302.1AGAP009671-like protein
AAEL00277	protein FAM177A1	0.191298154444647	0.276046022170186	0.711135357225659	NA		NA	KXJ74748.1 hypothet	XP_001662449.2protein FAM177A1
AAEL00172	gastrulation defective protein 1 homolog	0.191374700644919	0.434178825940773	0.820898614404559	NA		NA	KXJ80146.1 hypothet	XP_001653821.1gastrulation defective protein 1 homolog
AAEL00346	27 kDa hemolymph protein	0.191376466202311	0.13157297422291	0.538981683630591	NA		NA	KXJ73107.1 hypothet	XP_001656852.227 kDa hemolymph protein
AAEL00441	probable peroxisomal membrane protein PEX13	0.191467951940205	0.118114855248342	0.515958562099766	NA		aag04146	KXJ74891.1 hypothet	XP_001649113.1probable peroxisomal membrane protein PEX13
AAEL02209	NA	0.191689520271925	0.170829161857328	0.592079076237713	NA		NA	NA	XP_021695374.1pyridine nucleotide-disulfide oxidoreductase domain-containing protein 1
AAEL01022	uncharacterized LOC5580033	0.191929532263256	0.229910760168698	0.662910167685841	NA		NA	NA	XP_001660684.1uncharacterized protein LOC5580033
AAEL00149	cGMP-dependent 3',5'-cyclic phosphodiesterase	0.192059665578944	0.464573820025689	0.837744129311462	NA		aag01100 ; aag	KFB53135.1 AGAP00	XP_001653383.2cGMP-dependent 3',5'-cyclic phosphodiesterase
AAEL00770	putative E3 ubiquitin-protein ligase UBR7	0.19207992846356	0.265600469228664	0.70050315787967	NA		NA	KXJ77855.1 hypothet	XP_021704020.1putative E3 ubiquitin-protein ligase UBR7
AAEL00227	coagulation factor XI	0.192187206635962	0.208031794222596	0.639832374753494	NA		NA	KXJ81805.1 hypothet	XP_001661183.2coagulation factor XI
AAEL00825	UPF0687 protein C20orf27 homolog	0.19224586623115	0.360321181947305	0.774690541186705	NA		NA	KXJ74595.1 hypothet	XP_001653140.2UPF0687 protein C20orf27 homolog
AAEL00832	transmembrane protein 138	0.19234542536542	0.369472792518481	0.781817769662763	NA		NA	KFB48125.1 AGAP01	XP_001659138.1transmembrane protein 138
AAEL00857	protein RCC2 homolog	0.192428902988406	0.302140504163561	0.73489745394632	NA		NA	CRK93336.1 CLUMA_	XP_001653306.1protein RCC2 homolog
AAEL00368	tRNA pseudouridine synthase-like 1	0.192609132962372	0.425459512634837	0.814623464645531	NA		NA	KXJ73784.1 hypothet	XP_001657123.1tRNA pseudouridine synthase-like 1
AAEL00927	mitochondrial pyruvate carrier 2	0.192741473947287	0.159130717693238	0.577560236664366	NA		NA	NA	XP_001653781.1mitochondrial pyruvate carrier 2
AAEL00142	mitochondrial inner membrane protease subunit 2	0.192917915970866	0.297447179605018	0.729668411360336	NA		aag03060	XP_001866123.1 mit	XP_001659200.1mitochondrial inner membrane protease subunit 2
AAEL02398	NA	0.192926303461172	0.458582186123267	0.833308308986236	NA		NA	NA	XP_021698939.1transient receptor potential cation channel trpm isoform X7
AAEL00824	DNA polymerase alpha catalytic subunit	0.192943251929787	0.356275583199476	0.772867683747772	NA		aag03030	KXJ74598.1 hypothet	XP_001653143.2DNA polymerase alpha catalytic subunit
AAEL01051	zinc transporter 10	0.193028840673416	0.218039419132082	0.65121672666453	NA		NA	NA	XP_021705046.1zinc transporter 10
AAEL01239	uncharacterized LOC5576202	0.193040611658048	0.363336860249872	0.778250660803901	NA		NA	NA	XP_021693708.1uncharacterized protein LOC5576202 isoform X1
AAEL00598	autophagy-related protein 13 homolog	0.193062165852468	0.105402235979239	0.489854230040886	NA		aag04140 ; aag	XP_019533226.1 PRE	XP_001664333.1autophagy-related protein 13 homolog isoform X2
AAEL00881	5'-3' exoribonuclease 1	0.193340774836297	0.206207009699282	0.638433378885942	NA		aag03008 ; aag	KXJ79181.1 hypothet	XP_021709659.15'-3' exoribonuclease 1
AAEL01153	uncharacterized LOC5574939	0.1933948366458	0.399493861372551	0.798648539435638	NA		NA	KXJ73536.1 hypothet	XP_001661739.1uncharacterized protein LOC5574939
AAEL02010	NA	0.193540208911889	0.335304583300681	0.757775574591595	NA		aag04141	NA	XP_021705974.1eukaryotic translation initiation factor 2-alpha kinase 1
AAEL00032	ecdysone-induced protein 78C	0.193575428984055	0.549794608866399	0.873333609382774	NA		NA	XP_019527424.1 PRE	XP_021697912.1ecdysone-induced protein 78C isoform X1
AAEL02268	NA	0.193623418525428	0.181203603736463	0.607444649028391	NA		NA	NA	XP_021699337.1nuclear receptor coactivator 6 isoform X1
AAEL00183	geranylgeranyl transferase type-1 subunit beta	0.193784350091364	0.138709643586527	0.548805616375239	NA		NA	KFB49541.1 AGAP00	XP_001660375.1geranylgeranyl transferase type-1 subunit beta
AAEL00846	probable ATP-dependent RNA helicase CG8611	0.193784475664424	0.327435563306843	0.75045132093389	NA		NA	KXJ75088.1 hypothet	XP_001659252.2probable ATP-dependent RNA helicase CG8611
AAEL01335	profilin	0.193914518306581	0.0891200449283844	0.456108261085091	NA		aag04013	NA	XP_019559091.1 profilin isoform X1
AAEL01242	uncharacterized LOC5576268	0.193923516124997	0.183016113839459	0.610281543227372	NA		NA	KXJ76659.1 hypothet	XP_001662561.2uncharacterized protein LOC5576268
AAEL01721	acidic leucine-rich nuclear phosphoprotein 32 family member B	0.194196237359936	0.122237303065408	0.524358469989517	NA		NA	NA	XP_011493143.1acidic leucine-rich nuclear phosphoprotein 32 family member B
AAEL01159	pyridoxal phosphate phosphatase PHOSPHO2	0.194218600310883	0.340586492565507	0.761546348403887	NA		aag01100 ; aag	KFB46093.1 AGAP00	XP_001655487.1pyridoxal phosphate phosphatase PHOSPHO2

AAEL00912	probable cytochrome P450 6a20	0.194250660423268	0.407268923673636	0.803535299237735	NA	NA	KXJ79534.1 hypothet	XP_001653675.1probable cytochrome P450 6a20
AAEL0064€	RNA cytidine acetyltransferase	0.19443700979649	0.163297653868392	0.581366301644056	NA	aag03008	KXJ76163.1 hypothet	XP_001657833.1RNA cytidine acetyltransferase
AAEL01127	apolipoprotein D	0.194464137116165	0.139534498503578	0.549341242757296	NA	NA	XP_019546903.1 PRE	XP_021699909.1apolipoprotein D
AAEL02709	NA	0.194656482420151	0.443497929927246	0.82545872799161	NA	NA	NA	XP_021703877.1zinc finger protein OZF
AAEL01045	protein mago nashi	0.194844907545519	0.171918902964962	0.594065995149672	NA	aag03013 ; aag	NA	XP_001655409.1protein mago nashi
AAEL01195	thioredoxin-related transmembrane protein 2 homolog	0.195279424095602	0.238307618994952	0.67280858706937	NA	NA	ETN59388.1 thioredo	XP_001655808.1thioredoxin-related transmembrane protein 2 homolog
AAEL00495	protein N-terminal asparagine amidohydrolase	0.195283599827699	0.240226092876074	0.674980307372712	NA	NA	KXJ71088.1 hypothet	XP_021711015.1protein N-terminal asparagine amidohydrolase
AAEL01203	alpha-1,3/1,6-mannosyltransferase ALG2	0.195311625008455	0.247908747164023	0.68058844480365	00510 ; 00513	aag01100 ; aag	KXJ73291.1 hypothet	XP_001655818.2alpha-1,3/1,6-mannosyltransferase ALG2
AAEL00355	ribosome biogenesis protein BMS1 homolog	0.19540784648431	0.303869767999615	0.737301362252096	NA	aag03008	ETN61756.1 ribosom	XP_001663880.2ribosome biogenesis protein BMS1 homolog
AAEL00519	ras-related GTP-binding protein C	0.195436573322019	0.209037310805734	0.639832374753494	NA	aag04150 ; aag	ETN61182.1 GTP-bin	XP_001650499.1ras-related GTP-binding protein C
AAEL01989	NA	0.195519292962045	0.545153223595017	0.870466764449305	NA	NA	NA	XP_021703240.1zinc finger protein 14-like
AAEL01813	NA	0.19558588405782	0.12211720343752	0.524358469989517	NA	NA	NA	XP_019556438.1 insulin-like growth factor-binding protein complex acid labile subunit
AAEL01438	histone deacetylase complex subunit SAP18	0.195794090629112	0.39607321344298	0.796693358396638	NA	aag03013 ; aag	NA	XP_021705703.1histone deacetylase complex subunit SAP18
AAEL01190	zinc finger protein 620,zinc finger and SCAN domain-containing protein 3	0.195952275655122	0.627521752640798	0.900851040218507	NA	NA	KXJ75043.1 hypothet	XP_001662038.2zinc finger and SCAN domain-containing protein 31-like
AAEL02366	NA	0.195956797698644	0.446494998377253	0.828189271329869	NA	NA	NA	XP_001844519.1phosphatasepp1 regulatory subunit
AAEL01403	WD repeat-containing protein 18	0.196012064650985	0.126070411354591	0.530481545423179	NA	NA	NA	XP_001657325.2WD repeat-containing protein 18
AAEL00839	phosphatidylserine synthase 1	0.196161232276321	0.162988213849372	0.581366301644056	NA	aag01100 ; aag	KXJ71062.1 hypothet	XP_001653243.2phosphatidylserine synthase 1
AAEL00332	serine/threonine-protein phosphatase rdgC	0.196175474913959	0.531973611473498	0.866700945213732	04660 ; 04658	aag04745	XP_019560220.1 PRE	XP_001663541.2serine/threonine-protein phosphatase rdgC
AAEL00043	SET and MYND domain-containing protein 5	0.196198492539775	0.105362370818539	0.489854230040886	NA	NA	KXJ84211.1 hypothet	XP_001656403.1SET and MYND domain-containing protein 5
AAEL00941	synembryn	0.19620807872708	0.435181101228916	0.820960220424486	NA	NA	NA	XP_019538873.1 synembryn-like
AAEL00592	zinc finger protein 768	0.196275597869198	0.401970577936172	0.799283473604722	NA	NA	XP_019545855.1 PRE	XP_021705951.1zinc finger protein 768 isoform X1
AAEL00267	arginase, hepatic	0.196542421544951	0.14887944024046	0.563549407277957	00220 ; 00330	aag01100 ; aag	ETN58471.1 arginase	XP_001662057.1arginase, hepatic
AAEL02493	NA	0.196564044332951	0.283191371131609	0.715753918648043	NA	NA	NA	XP_021703425.1uncharacterized protein LOC110677082
AAEL00080	protein ABHD17B	0.196771113452411	0.221840273785339	0.654118491874251	NA	NA	ETN67913.1 abhydro	XP_001651277.1protein ABHD17B isoform X2
AAEL00593	zinc finger protein with KRAB and SCAN domains 5	0.196802412511771	0.527810637951693	0.865973876319045	NA	NA	KXJ83337.1 hypothet	XP_001651684.2zinc finger protein with KRAB and SCAN domains 5
AAEL01981	NA	0.196807906386944	0.28237639395334	0.715753918648043	NA	NA	NA	XP_021709755.1uncharacterized protein LOC110679459
AAEL00206	hydroxymethylglutaryl-CoA lyase, mitochondrial	0.197047931198081	0.274453670883593	0.710214476791569	00072 ; 00650 ; 00281 ;	aag01100 ; aag	XP_001843753.1 hyd	XP_001654565.1hydroxymethylglutaryl-CoA lyase, mitochondrial
AAEL01461	cytochrome P450 9e2	0.197085091641453	0.444151689714809	0.825840341231698	NA	NA	NA	XP_001649100.3cytochrome P450 9e2
AAEL01001	GTP-binding protein SAR1	0.19719428957972	0.106308768832981	0.490844601505817	NA	aag04141	NA	XP_001660557.1GTP-binding protein SAR1
AAEL02241	NA	0.197210851117398	0.277994894293452	0.713122992430066	NA	NA	NA	XP_021708900.1 nucleolysin TIAR
AAEL00945	syntaxin-4	0.197252946504718	0.368518599428493	0.781702146406381	NA	NA	NA	XP_001660092.1syntaxin-4 isoform X1
AAEL00391	uncharacterized LOC5563720	0.197270813329303	0.117934586952936	0.515795660238079	NA	NA	KXJ69032.1 hypothet	XP_019546799.1 putative mediator of RNA polymerase II transcription subunit 26
AAEL00189	dnaJ homolog subfamily C member 7	0.197514064741039	0.0901235720893881	0.458542183179048	NA	NA	KXJ70278.1 hypothet	XP_001654193.2dnaJ homolog subfamily C member 7 isoform X1
AAEL00243	N-acetylglucosamine-6-phosphate deacetylase	0.197623805423719	0.311188637022574	0.743202138043531	00052 ; 00520	aag01100 ; aag	KXJ82845.1 hypothet	XP_001655220.2N-acetylglucosamine-6-phosphate deacetylase
AAEL01866	NA	0.197624027279196	0.269737493235484	0.705750472775923	NA	aag01100 ; aag	NA	YP_003934127.1cytochrome c oxidase subunit III (mitochondrion)
AAEL01213	transcriptional activator protein Pur-beta	0.19779330460255	0.216376670152917	0.64939292703965	NA	NA	ETN62404.1 pur-alph	XP_021710824.1transcriptional activator protein Pur-beta isoform X1
AAEL00698	transcription initiation factor TFIID subunit 6	0.197968253381921	0.412357840967794	0.806884477085645	NA	aag03022	KXJ76316.1 hypothet	XP_021705791.1transcription initiation factor TFIID subunit 6 isoform X1
AAEL02007	NA	0.19850754284801	0.386882217511286	0.791623050051228	NA	NA	NA	XP_021698881.1dentin sialophosphoprotein
AAEL01980	NA	0.198573558042778	0.103653211509368	0.486291766544946	NA	NA	NA	XP_021707990.1CTL-like protein 2 isoform X1
AAEL02354	NA	0.198607983285001	0.628504256271917	0.901045385683245	NA	NA	NA	NA
AAEL02563	NA	0.198677054682497	0.218679318105253	0.652281064896124	NA	NA	NA	XP_021704687.1PAX-interacting protein 1-like
AAEL00094	tetraspanin-2A	0.198703596587445	0.152255762720664	0.568113296551786	NA	NA	CRK86592.1 CLUMA_	XP_019531731.1 tetraspanin-2A-like
AAEL00928	ATP-dependent RNA helicase DDX54	0.19891542114373	0.130207100041509	0.537053220341536	NA	NA	NA	XP_001659898.2ATP-dependent RNA helicase DDX54
AAEL00399	myeloid leukemia factor	0.19919308363853	0.116934190976935	0.514582987743323	NA	NA	KXJ78261.1 hypothet	XP_001648224.1myeloid leukemia factor
AAEL00583	zinc finger and SCAN domain-containing protein 21	0.199194940022526	0.63167271193117	0.902506916959539	NA	NA	KXJ81995.1 hypothet	XP_021701655.1zinc finger and SCAN domain-containing protein 21
AAEL02031	NA	0.199320503688465	0.103565048262076	0.486291766544946	NA	aag03015	NA	XP_021697649.1 polyadenylate-binding protein 2
AAEL02596	NA	0.199612530678243	0.503304642225738	0.858744381072989	NA	NA	NA	XP_019525475.1 rho GTPase-activating protein gacF-like isoform X2
AAEL00670	angiopoietin-2	0.199694350479319	0.24076607306398	0.675511149976115	NA	NA	KXJ84156.1 hypothet	ABF18152.1salivary secreted angiopoietin
AAEL01470	methionine--tRNA ligase, cytoplasmic	0.20018592459346	0.083295112714696	0.438013925780757	00970 ; 00450	aag01100 ; aag	NA	XP_001649418.2methionine--tRNA ligase, cytoplasmic
AAEL01311	charged multivesicular body protein 1b-2	0.200202577633817	0.159350515997809	0.577560236664366	NA	aag04144	NA	XP_011493702.1charged multivesicular body protein 1b-2
AAEL00866	protein ABHD4	0.200338898779743	0.136842159815443	0.545589929345625	NA	NA	KXJ70456.1 hypothet	XP_021700237.1protein ABHD4
AAEL02101	NA	0.200375863943311	0.472454101582295	0.842869841558797	NA	NA	NA	KFB52180.1AGAP000294-like protein
AAEL02450	NA	0.200380229150562	0.112562240513045	0.503547757511443	NA	NA	NA	XP_021693033.1transcription elongation factor B polypeptide 3
AAEL01204	zinc finger protein 62	0.20059319003442	0.617869564811818	0.897318336565571	NA	NA	KXJ81850.1 hypothet	XP_001655846.2zinc finger protein 62
AAEL02703	NA	0.200757920921931	0.162371713541924	0.580707394694867	NA	NA	NA	XP_021699471.1congested-like trachea protein
AAEL02087	NA	0.200862375472193	0.0803457157102552	0.433079891352508	NA	NA	NA	XP_021698017.1large proline-rich protein bag6-A isoform X1
AAEL01138	vacuolar-sorting protein SNF8	0.201009240107443	0.467225103180728	0.839170326093322	NA	aag04144	KXJ71323.1 hypothet	XP_001655319.2vacuolar-sorting protein SNF8
AAEL00728	3-phosphoinositide-dependent protein kinase 1	0.201087269098136	0.256341510408579	0.688461255220739	04151 ; 05165 ; 04714 ;	aag04150 ; aag	XP_001863432.1 3-p	XP_001652653.13-phosphoinositide-dependent protein kinase 1 isoform X1
AAEL00139	WD repeat and HMG-box DNA-binding protein 1	0.201154629454903	0.56052779803228	0.87800595572912	NA	NA	XP_019542352.1 PRE	XP_021696464.1WD repeat and HMG-box DNA-binding protein 1
AAEL02559	NA	0.201156800754506	0.0875721206821877	0.452464151890787	NA	aag03050	NA	XP_021709572.126S proteasome regulatory subunit 6B
AAEL02635	NA	0.201240749923562	0.0754370674203723	0.420455417566485	00270 ; 00430	aag01100 ; aag	NA	XP_021698171.1cysteine dioxygenase type 1
AAEL00613	proclotting enzyme	0.201337948521272	0.113993452054617	0.507483186166707	NA	NA	XP_001865845.1 seri	XP_001657539.2proclotting enzyme
AAEL00594	serine/threonine-protein kinase stk11	0.201354745709418	0.445636378076218	0.82793174864877	04151 ; 05165 ; 04714 ;	aag04150 ; aag	KXJ72635.1 hypothet	XP_021693314.1serine/threonine-protein kinase stk11
AAEL00910	protein UXT	0.20144417416061	0.46330587818451	0.837274753622609	NA	NA	KXJ70034.1 hypothet	XP_001659764.2protein UXT
AAEL02647	NA	0.201658402407292	0.330797859788115	0.754128446928769	NA	aag03420	NA	XP_021703063.1DNA repair protein complementing XP-G cells homolog isoform X2
AAEL01322	formin-J	0.201724168449454	0.433281219501318	0.820258728065422	NA	NA	NA	XP_021711096.1formin-J isoform X2
AAEL01827	NA	0.201881277491474	0.110137841724219	0.500079481483548	NA	NA	NA	XP_021702450.1thioredoxin domain-containing protein 11
AAEL02159	NA	0.201942298057886	0.193423999726849	0.624628157151667	NA	NA	NA	XP_021710635.1NADPH-dependent diflavin oxidoreductase 1 isoform X2
AAEL00116	CD109 antigen	0.202265224669659	0.28645357171939	0.718828951993952	NA	NA	XP_019525400.1 PRE	XP_021701342.1CD109 antigen
AAEL00385	essential MCU regulator, mitochondrial	0.202433326362211	0.077207203844289	0.424916680487529	NA	NA	KFB35868.1 AGAP01	XP_021707664.1essential MCU regulator, mitochondrial
AAEL01036	peroxisomal membrane protein PEX14	0.20248769658851	0.144240686376574	0.55675452853911	NA	aag04146	NA	XP_001654503.1peroxisomal membrane protein PEX14
AAEL01824	NA	0.202548167720286	0.342013782659432	0.762438298723025	NA	NA	NA	XP_021700633.1uncharacterized protein LOC5570910 isoform X1
AAEL01354	chorion transcription factor Cf2	0.202697756451082	0.550228581437217	0.873719310851643	NA	NA	NA	XP_021701461.1zinc finger protein 853 isoform X1
AAEL01568	histone H2B	0.202758068256307	0.648313125938219	0.906554905014882	NA	NA	NA	XP_001657158.1histone H2B
AAEL00502	palmitoyltransferase ZDHHC23	0.203210511223923	0.146432207174996	0.560978006005125	NA	NA	KXJ74427.1 hypothet	XP_001650198.2palmitoyltransferase ZDHHC23 isoform X1
AAEL00546	phosphatidylinositol N-acetylglucosaminyltransferase subunit A	0.203232141644861	0.378876022995193	0.785666748907449	563	aag01100 ; aag	KXJ70160.1 hypothet	XP_001650931.2phosphatidylinositol N-acetylglucosaminyltransferase subunit A
AAEL01995	NA	0.203304151277977	0.279129487024239	0.713604060506006	NA	NA	NA	XP_021694335.1uncharacterized protein LOC5572394
AAEL02434	NA	0.203468165125637	0.0672766586515285	0.4007529665455	NA	NA	NA	XP_021708772.1TAR DNA-binding protein 43-like
AAEL00664	probable ATP-dependent RNA helicase DDX55 homolog	0.203567337788724	0.304172453893041	0.737548068701915	NA	NA	KXJ72571.1 hypothet	XP_001652148.1probable ATP-dependent RNA helicase DDX55 homolog
AAEL01032	homeobox protein six1b	0.2035756488635	0.291472931856199	0.722646576583698	NA	NA	NA	XP_001654439.2homeobox protein six1b isoform X1
AAEL00268	xanthine dehydrogenase	0.203874981180994	0.0877238805971703	0.452647141876812	230	aag01100 ; aag	KXJ76128.1 hypothet	XP_001662131.2xanthine dehydrogenase
AAEL00412	ubiquitin-conjugating enzyme E2 Q2	0.20394363415831	0.0930149907102381	0.463413072287824	NA	aag04120	XP_013105449.1 PRE	XP_021710068.1 ubiquitin-conjugating enzyme E2 Q2

AAEL0075	DNA polymerase delta small subunit	0.203961715137953	0.259082083345936	0.691547675112889	NA	aag03420 ; aag	XP_019535810.1 PRE	XP_001658441.1DNA polymerase delta small subunit isoform X1
AAEL0036	multifunctional protein ADE2	0.203973299198931	0.131827040646348	0.538981683630591	00230 ; 00230	aag01100 ; aag	KXJ72751.1 hypothet	XP_001657043.1multifunctional protein ADE2
AAEL0132	NA	0.20403770858843	0.20785016317526	0.639832374753494	NA	aag03420 ; aag	NA	XP_001663396.2DNA excision repair protein haywire
AAEL0268	NA	0.204198184872303	0.129168504597427	0.53537966541735	NA	NA	NA	NA
AAEL0101	zinc finger protein 676	0.204321742326293	0.44673377555481	0.82821796189507	NA	NA	NA	XP_021707264.1zinc finger protein 676
AAEL0094	MAP kinase-activated protein kinase 2	0.204423160680814	0.25192786001693	0.684556086619159	04151 ; 05165 ; 04714 ;	NA	NA	XP_021708638.1MAP kinase-activated protein kinase 2 isoform X1
AAEL0225	NA	0.204427850925432	0.375387173514301	0.782198815065306	NA	NA	NA	XP_001649479.2COP9 signalosome complex subunit 5
AAEL0038	NF-kappa-B inhibitor-interacting Ras-like protein	0.204632171112598	0.459206508257511	0.833784192775378	NA	NA	KXJ80224.1 hypothet	XP_021711273.1NF-kappa-B inhibitor-interacting Ras-like protein
AAEL0099	protein penguin	0.204915127530598	0.0927208364121501	0.462398967492132	NA	NA	NA	XP_001654109.2protein penguin
AAEL0149	RNA-binding protein Nova-1	0.204915599694404	0.0981014013450038	0.473943787004209	NA	NA	NA	XP_021693772.1RNA-binding protein Nova-1 isoform X3
AAEL0122	probable 39S ribosomal protein L24, mitochondrial	0.204992435023977	0.121518899231784	0.523754993316568	NA	aag03010	KXJ76249.1 hypothet	XP_001655972.2probable 39S ribosomal protein L24, mitochondrial
AAEL0268	NA	0.205009229740372	0.151778715498588	0.567477492258654	NA	NA	NA	XP_001652019.2protein brunelleschi
AAEL0101	uncharacterized protein KIAA0513	0.205193639593092	0.409521350058704	0.805356078752463	NA	NA	NA	XP_001654263.2uncharacterized protein KIAA0513
AAEL0236	NA	0.205261685394119	0.0798228209412844	0.431435389689367	NA	NA	NA	XP_001850140.1histone h2a
AAEL0137	TATA box-binding protein-associated factor RNA polymerase I subunit B	0.205308642895158	0.50250961462486	0.858744381072989	NA	NA	NA	XP_001663907.2TATA box-binding protein-associated factor RNA polymerase I subunit B
AAEL0090	rRNA methyltransferase 3, mitochondrial	0.205357644684742	0.204907094283215	0.637782699712521	NA	NA	KXJ73491.1 hypothet	XP_001653652.2rRNA methyltransferase 3, mitochondrial
AAEL0235	NA	0.205429926040275	0.288261053999543	0.720809298615161	NA	NA	NA	XP_001659916.2angiotensin-converting enzyme
AAEL0070	transcription initiation factor TFIID subunit 5	0.20549380099556	0.352570159296058	0.76935039265884	NA	aag03022	XP_001845816.1 tra	XP_001652518.2transcription initiation factor TFIID subunit 5
AAEL0066	gametocyte-specific factor 1	0.205679073592687	0.368124138499048	0.781605519720406	NA	NA	KFB49114.1 AGAP00	XP_001657928.1gametocyte-specific factor 1
AAEL0144	NA	0.205726906326824	0.367980017405155	0.781479833870814	NA	NA	NA	XP_021693752.1PR domain zinc finger protein 5
AAEL0134	uncharacterized LOC5577982	0.205841527998404	0.552815122461967	0.874531710113044	NA	NA	NA	XP_001656722.2uncharacterized protein LOC5577982
AAEL0018	transmembrane protein 65	0.205870841124644	0.27643929239365	0.711749963236253	NA	NA	XP_319767.4 AGAP0	XP_021702420.1transmembrane protein 65 isoform X2
AAEL0009	AP-1 complex subunit sigma-2	0.205917853099348	0.227874632362586	0.66175420142127	NA	aag04142	XP_003436771.1 AG	XP_001657742.1AP-1 complex subunit sigma-2 isoform X1
AAEL0081	latrophilin Cirl	0.206411688879268	0.605837523893297	0.893547770777888	NA	NA	KXJ83076.1 hypothet	XP_021705980.1latrophilin Cirl
AAEL0036	uncharacterized LOC5578764	0.206603372766243	0.556070605069775	0.876514925250035	NA	NA	KXJ69052.1 hypothet	XP_001657134.1uncharacterized protein LOC5578764
AAEL0065	mitoferrin	0.206698495209789	0.0606489169977615	0.384941803020695	NA	NA	ETN62633.1 mitocho	XP_019526011.1 mitoferrin-like
AAEL0121	ribosome biogenesis regulatory protein homolog	0.206755632042259	0.1508320613515	0.566025396924239	NA	NA	KXJ80947.1 hypothet	XP_001662293.1ribosome biogenesis regulatory protein homolog
AAEL0116	cationic amino acid transporter 3	0.206982120050604	0.542295216585915	0.869900911343623	NA	NA	KFB43755.1 AGAP00	XP_001661832.1cationic amino acid transporter 3 isoform X1
AAEL0264	NA	0.207013574235865	0.403916951530506	0.800429773223221	NA	aag03020	NA	XP_021694575.1uncharacterized protein LOC110674739
AAEL0031	protein arginine N-methyltransferase 1	0.207210186607555	0.071701808311989	0.411860199804571	NA	aag04068	KXJ77137.1 hypothet	XP_001656487.1protein arginine N-methyltransferase 1
AAEL0014	uncharacterized LOC5570502	0.207271517830957	0.421489718276195	0.814192545906728	NA	NA	KXJ70395.1 hypothet	XP_001659187.2uncharacterized protein LOC5570502 isoform X1
AAEL0095	transmembrane channel-like protein 5	0.207361170501896	0.106944685055053	0.492398491132955	NA	NA	NA	XP_021695152.1transmembrane channel-like protein 5
AAEL0043	nucleolar protein 6	0.207399673557942	0.172850459834309	0.595942274782807	NA	aag03008	XP_001850517.1 nuc	XP_001648991.2nucleolar protein 6
AAEL0079	uncharacterized LOC5569870	0.207487940916049	0.427306094144714	0.815782298406567	NA	NA	KXJ67937.1 hypothet	XP_001658763.2uncharacterized protein LOC5569870
AAEL0062	ubiquitin-fold modifier 1	0.207598033137724	0.124124728066929	0.527832049142223	NA	NA	XP_018573392.1 ubi	XP_001657613.1ubiquitin-fold modifier 1
AAEL0109	dnaJ homolog subfamily C member 30	0.207657033571228	0.102362139693054	0.484636597025644	NA	NA	KXJ80775.1 hypothet	XP_001661210.1dnaJ homolog subfamily C member 30
AAEL0013	U6 snRNA-associated Sm-like protein LSm2	0.207926796299626	0.331674951763549	0.754128446928769	NA	aag03040 ; aag	ETN59459.1 u6 snRN	XP_001653182.1U6 snRNA-associated Sm-like protein LSm2
AAEL0023	probable prefoldin subunit 4	0.207958192807342	0.0941568560204514	0.464823706598816	NA	NA	ETN66629.1 prefoldi	XP_001661366.1probable prefoldin subunit 4
AAEL0267	NA	0.208041484782544	0.481228042450155	0.846541855596699	NA	NA	NA	XP_021703441.1zinc finger protein 121-like
AAEL0073	tRNA (adenine(37)-N6)-methyltransferase	0.208092804001372	0.359789577826281	0.774255626424296	NA	NA	KXJ79900.1 hypothet	XP_021699810.1uncharacterized protein LOC5569027
AAEL0099	DEAD-box helicase Dbp80	0.208255492889132	0.17510541010916	0.598647199141587	NA	NA	NA	XP_001654105.1DEAD-box helicase Dbp80
AAEL0152	ras-related C3 botulinum toxin substrate 1	0.208373896170119	0.211298818357286	0.643406565246605	NA	aag04013 ; aag	NA	XP_021708351.1ras-related C3 botulinum toxin substrate 1 isoform X1
AAEL0131	39S ribosomal protein L3, mitochondrial	0.208397445245004	0.0838799948420731	0.439557922856923	NA	aag03010	NA	XP_001656360.139S ribosomal protein L3, mitochondrial
AAEL0038	methyltransferase-like protein 17, mitochondrial	0.20853669599701	0.160956766317779	0.578121300046961	NA	NA	KXJ69558.1 hypothet	XP_001657302.2methyltransferase-like protein 17, mitochondrial
AAEL0202	NA	0.208837638289978	0.100521644634319	0.480769321166839	NA	NA	NA	NA
AAEL0207	NA	0.208882000624448	0.139551408559783	0.549341242757296	NA	NA	NA	XP_001655704.2serine protease 55
AAEL0000	proteasome assembly chaperone 2	0.208980291920775	0.32073158629244	0.746566934846029	NA	NA	KXJ68095.1 hypothet	XP_001647946.2proteasome assembly chaperone 2
AAEL0083	uncharacterized LOC5570563	0.208987904449355	0.526123940349227	0.865680248818465	NA	NA	XP_001851816.1 con	XP_001653231.2uncharacterized protein LOC5570563
AAEL0054	NA	0.209012788864645	0.540310838730664	0.869735874782513	860	aag01100 ; aag	KXJ74971.1 hypothet	XP_001650846.1ferrochelatase, mitochondrial
AAEL0123	transcription factor 25	0.209018878626403	0.1800798053745	0.605441859355553	NA	NA	KXJ74381.1 hypothet	XP_001662408.2transcription factor 25
AAEL0049	signal recognition particle 19 kDa protein	0.209103505072445	0.277545019890905	0.713084757541644	NA	aag03060	KXJ75286.1 hypothet	XP_001650015.1signal recognition particle 19 kDa protein
AAEL0228	NA	0.209133982444391	0.215271165037847	0.648208527708858	NA	NA	NA	XP_021702401.1zinc transporter 2
AAEL0064	partitioning defective 6 homolog beta	0.209186703006264	0.291639962816707	0.722646576583698	NA	aag04144 ; aag	ETN66015.1 par-6 ga	XP_001657836.1partitioning defective 6 homolog beta
AAEL0070	low-density lipoprotein receptor-related protein 1	0.209239154343053	0.174182144104029	0.597209919209252	NA	NA	KXJ81450.1 hypothet	XP_021706493.1low-density lipoprotein receptor-related protein 1
AAEL0181	NA	0.209445231093277	0.317753295974875	0.745684464362801	NA	NA	NA	XP_001657719.2sodium-coupled monocarboxylate transporter 1 isoform X2
AAEL0101	ATP synthase mitochondrial F1 complex assembly factor 2	0.209458028846885	0.185324819464055	0.612504175036132	NA	NA	NA	XP_001654255.1ATP synthase mitochondrial F1 complex assembly factor 2
AAEL0105	chondroitin sulfate synthase 2	0.209530070061556	0.310660797396899	0.742918550320373	NA	aag01100 ; aag	KXJ71000.1 hypothet	XP_001660919.2chondroitin sulfate synthase 2
AAEL0147	dnaJ homolog subfamily C member 28	0.209531764650032	0.134404626227112	0.542662316284181	NA	NA	NA	XP_001649466.1dnaJ homolog subfamily C member 28
AAEL0041	rhophilin-2	0.209627923153185	0.267920562864979	0.704026692311667	NA	NA	XP_019541446.1 PRE	XP_021694700.1rhophilin-2 isoform X1
AAEL0217	NA	0.209663596512851	0.290077549749014	0.721952579380462	NA	NA	NA	KFB48251.1AGAP005815-like protein
AAEL0139	probable methylcrotonoyl-CoA carboxylase beta chain, mitochondrial	0.209855084713594	0.140318564494086	0.550646976570049	NA	aag01100 ; aag	NA	XP_001664206.2probable methylcrotonoyl-CoA carboxylase beta chain, mitochondrial
AAEL0077	cAMP-regulated phosphoprotein 19	0.209880645092762	0.079276025228354	0.430779070308269	NA	NA	XP_019531827.1 PRE	XP_021706661.1cAMP-regulated phosphoprotein 19 isoform X2
AAEL0241	NA	0.210097839143766	0.0688040121394577	0.403270394718019	NA	NA	NA	XP_021695035.1mitochondrial coenzyme A transporter SLC25A42
AAEL0106	general transcription factor 3C polypeptide 3	0.210119112840859	0.50280960452975	0.858744381072989	NA	NA	XP_001847609.1 gen	XP_001660944.1general transcription factor 3C polypeptide 3
AAEL0173	DNA topoisomerase 1	0.210168715505722	0.463571066769747	0.837274753622609	NA	NA	NA	XP_011493613.1DNA topoisomerase 1
AAEL0181	NA	0.210190901196185	0.504770830974503	0.858744381072989	NA	NA	NA	XP_021703669.1zinc finger protein 14
AAEL0218	NA	0.210249424004935	0.253405746372521	0.685145258966747	NA	NA	NA	XP_021699195.1gastrula zinc finger protein XlCGF8.2DB isoform X1
AAEL0035	protein arginine N-methyltransferase 6	0.210301459859836	0.251892948612216	0.684556086619159	NA	NA	KXJ84406.1 hypothet	XP_001656935.1protein arginine N-methyltransferase 6 isoform X1
AAEL0068	microsomal glutathione S-transferase 1	0.210342418990637	0.0832965944501652	0.438013925780757	NA	aag01100 ; aag	KXJ71801.1 hypothet	XP_001658060.1microsomal glutathione S-transferase 1
AAEL0014	leucine-rich repeat transmembrane protein FLRT3	0.210342826673257	0.108649275808659	0.495725018486141	NA	NA	KXJ74861.1 hypothet	XP_001659167.2leucine-rich repeat transmembrane neuronal protein 4 isoform X1
AAEL0007	coiled-coil-helix-coiled-coil-helix domain-containing protein 7	0.210356152215375	0.275203137341127	0.710952708392882	NA	NA	ETN67042.1 hypothe	XP_001650676.1coiled-coil-helix-coiled-coil-helix domain-containing protein 7
AAEL0005	microspherule protein 1	0.210387417661022	0.211704685843621	0.644215118760914	NA	NA	KXJ68971.1 hypothet	XP_001648652.1microspherule protein 1
AAEL0013	protein ST7 homolog	0.210537253007799	0.362553258300746	0.77767251349265	NA	NA	XP_001865018.1 sup	XP_021696429.1protein ST7 homolog
AAEL0017	nuclear hormone receptor HR78	0.210660922869923	0.21491161802459	0.648028428468258	NA	NA	XP_019536112.1 PRE	XP_021702465.1nuclear hormone receptor HR78 isoform X2
AAEL0105	palmitoyltransferase ZDHHC15	0.210672341548352	0.363314379492332	0.778250660803901	NA	NA	XP_019533697.1 PRE	XP_001654678.1palmitoyltransferase ZDHHC15 isoform X3
AAEL0032	protein msta	0.210833970280716	0.363606732810947	0.778250660803901	NA	NA	XP_019560227.1 PRE	XP_001663543.2protein msta isoform X2
AAEL0145	uncharacterized LOC5564602	0.210928411291322	0.384898759507095	0.790080064171371	NA	NA	NA	XP_019535682.1 cell wall protein RBR3 isoform X1
AAEL0125	proliferating cell nuclear antigen	0.210988042508028	0.185160617641039	0.612420502332469	NA	aag03420 ; aag	AAY82460.1 prolifera	XP_001662644.1proliferating cell nuclear antigen
AAEL0092	serine/threonine-protein phosphatase alpha-2 isoform	0.211050408827323	0.0652383501859995	0.39730198438886	04660 ; 04658	aag03015	NA	XP_001653770.1serine/threonine-protein phosphatase alpha-2 isoform isoform X1
AAEL0067	RNA exonuclease 1	0.211090105217096	0.0796147437206943	0.431226849261068	NA	aag03008	KXJ79496.1 hypothet	XP_001652178.2RNA exonuclease 1
AAEL0004	UDP-glucose:glycoprotein glucosyltransferase	0.211094059730243	0.071887017071789	0.411912175099304	NA	aag04141	KXJ84210.1 hypothet	XP_001656400.1UDP-glucose:glycoprotein glucosyltransferase
AAEL0025	protein snakeskin	0.211312092705818	0.111233543997588	0.502609810643532	NA	NA	ABV44719.1 ryanodi	XP_019565150.1 protein snakeskin-like

AAEL0245	NA	0.211446833242199	0.335431782831098	0.757775574591595	NA		aag01100 ; aag	NA	XP_001653482.2 GPI mannosyltransferase 1
AAEL0096	venom allergen 5.02	0.211600407235226	0.149567820482731	0.565105542550216	NA		NA	NA	XP_021706275.1venom allergen 5.02
AAEL0130	zinc finger protein 14	0.211747158566859	0.136619626108262	0.545589928345625	NA		NA	NA	XP_001663295.2zinc finger protein 14
AAEL0026	ER degradation-enhancing alpha-mannosidase-like protein 3	0.211800956253464	0.134692537534565	0.542662316284181	00510 ; 00513		aag04141	KFB39227.1 AGAP00	XP_001661964.1ER degradation-enhancing alpha-mannosidase-like protein 3
AAEL0072	titin	0.211956258574292	0.110886845509841	0.502155882077536	NA		NA	KXJ82622.1 hypothe	XP_021709793.1titin isoform X2
AAEL0145	decapping nuclease DXO homolog	0.212339617635311	0.552846314191039	0.874531710113044	NA		NA	NA	XP_001649052.1decapping nuclease DXO homolog
AAEL0039	probable phenylalanine--tRNA ligase, mitochondrial	0.212556717876976	0.152634278846003	0.568113296551786		970	aag00970	KXJ72414.1 hypothe	XP_001648213.2probable phenylalanine--tRNA ligase, mitochondrial
AAEL0128	box C/D snoRNA protein 1	0.21260509859107	0.275653607757719	0.711001645592863	NA		NA	NA	XP_001656295.1box C/D snoRNA protein 1
AAEL0109	solute carrier organic anion transporter family member 5A1	0.21262891741704	0.150277390797298	0.566025396924239	NA		NA	KXJ82215.1 hypothe	XP_001661188.2solute carrier organic anion transporter family member 5A1
AAEL0085	transcription factor grauzone	0.21272077455811	0.433799428479973	0.820605381401592	NA		NA	XP_019533154.1 PRE	XP_001659339.1transcription factor grauzone
AAEL0087	endothelial differentiation-related factor 1 homolog	0.212855939023418	0.0679413917501841	0.401232883533236	NA		NA	XP_001845668.1 mu	XP_001659486.1endothelial differentiation-related factor 1 homolog isoform X2
AAEL0089	potassium channel subfamily T member 2	0.21296115238059	0.432380160190456	0.820005280371151	NA		NA	NA	XP_021695804.1potassium channel subfamily T member 2 isoform X5
AAEL0186	NA	0.213039865315012	0.264542115962971	0.698659663885272		190	aag01100 ; aag	NA	YP_009389261.1cytochrome c oxidase subunit I (mitochondrion)
AAEL0174	angiotensin-converting enzyme	0.213320696289979	0.0737230255973667	0.417913179290884	NA		NA	NA	XP_011493087.1angiotensin-converting enzyme
AAEL0035	H/ACA ribonucleoprotein complex subunit 4	0.213842557269881	0.080666179706339	0.433079891352508	NA		aag03008	KXJ82572.1 hypothe	XP_001656912.2H/ACA ribonucleoprotein complex subunit 4
AAEL0107	transmembrane protein 53-B	0.213848117311321	0.397494530493598	0.797539006884967	NA		NA	ETN60377.1 transme	XP_001654882.1transmembrane protein 53-B
AAEL0232	NA	0.213893797778777	0.402133486699113	0.799283473604722	NA		NA	NA	XP_021697019.1zinc finger protein 778-like
AAEL0145	polycomb protein eed-B	0.213979655015743	0.444101834340477	0.825840341231698	NA		NA	NA	XP_001648965.1polycomb protein eed-B
AAEL0101	ras-related protein Rab-8A	0.213997690235928	0.07750431145019	0.426296876249043	NA		aag04144 ; aag	NA	XP_001654277.1ras-related protein Rab-8A
AAEL0114	small nuclear ribonucleoprotein Sm D3	0.214169911132527	0.0925297967051513	0.4622471168734	NA		aag03040	CRK99257.1 CLUMA_	XP_001661657.1small nuclear ribonucleoprotein Sm D3
AAEL0071	uncharacterized LOC5568858	0.214357992959877	0.366977516219166	0.780578423384551	04660 ; 04658		NA	KXJ79434.1 hypothe	XP_021704207.1uncharacterized protein LOC5568858
AAEL0215	NA	0.214365467724781	0.47288641217909	0.843297185756203	NA		NA	NA	XP_021692948.1epithelial chloride channel protein-like
AAEL0012	E3 ubiquitin-protein ligase Su(dx)	0.214433734698144	0.0905418568881146	0.458582874647784	NA		aag04144 ; aag	XP_019535884.1 PRE	XP_021703044.1E3 ubiquitin-protein ligase Su(dx)
AAEL0068	UDP-xylose and UDP-N-acetylglucosamine transporter	0.214530911161212	0.0825856127090947	0.437257081788889	NA		NA	KXJ75916.1 hypothe	XP_001652285.1UDP-xylose and UDP-N-acetylglucosamine transporter
AAEL0111	sorbitol dehydrogenase	0.214545871746332	0.425797424210824	0.814623464645531	NA		aag01100 ; aag	KXJ69733.1 hypothe	XP_001655106.1sorbitol dehydrogenase
AAEL0181	NA	0.214570905443039	0.235714794752153	0.669872619304915	NA		NA	NA	XP_021713041.1forkhead box protein N2 isoform X1
AAEL0140	serine protease inhibitor 27A	0.214596385556941	0.0796659034713993	0.431226849261068	NA		NA	NA	XP_001648011.1serine protease inhibitor 27A
AAEL0084	histidine triad nucleotide-binding protein 3	0.214643320044482	0.20174964355873	0.634701781910801	NA		NA	KXJ71557.1 hypothe	XP_001659264.1histidine triad nucleotide-binding protein 3
AAEL0054	twinfilin	0.214680643812517	0.151828905231559	0.567477492258654	NA		NA	XP_019528940.1 PRE	Q17A58.1RecName: Full=Twinfilin
AAEL0106	uncharacterized LOC5573666	0.214726186291683	0.580772009659235	0.883203442882875	NA		NA	NA	XP_021698216.1uncharacterized protein LOC5573666
AAEL0104	putative GPI-anchor transamidase	0.214826617560141	0.294604129199958	0.72562826469433	NA		aag01100 ; aag	NA	XP_001660868.1putative GPI-anchor transamidase
AAEL0102	rab3 GTPase-activating protein catalytic subunit	0.214876470858446	0.616617424550124	0.897164966965308	NA		NA	NA	XP_021701819.1rab3 GTPase-activating protein catalytic subunit isoform X1
AAEL0264	NA	0.215323173065085	0.197948318768452	0.631376231995595	NA		NA	NA	XP_021713350.1protein toll
AAEL0138	cap-specific mRNA (nucleoside-2'-O-)-methyltransferase 1	0.215468423034302	0.405290359619074	0.801349782813648	NA		NA	NA	XP_001664002.2cap-specific mRNA (nucleoside-2'-O-)-methyltransferase 1
AAEL0049	uncharacterized LOC5565798	0.215590871702035	0.295339049798588	0.726854756952823	NA		NA	XP_001850875.1 con	XP_001650165.1uncharacterized protein LOC5565798
AAEL0121	proton-associated sugar transporter A	0.215692373160685	0.233912871048466	0.667431392058289	NA		NA	XP_019530347.1 PRE	XP_021694446.1proton-associated sugar transporter A
AAEL0122	zinc finger protein 888	0.215801025624595	0.541152228176307	0.869735874782513	NA		NA	KXJ76253.1 hypothe	XP_021702617.1zinc finger protein 888
AAEL0119	something about silencing protein 10	0.215968789652706	0.209408057692826	0.639832374753494	NA		NA	KXJ75192.1 hypothe	XP_021706178.1something about silencing protein 10
AAEL0130	signal recognition particle 54 kDa protein	0.215977203083429	0.0927611925657983	0.462398967492132	NA		aag03060	NA	XP_021695011.1signal recognition particle 54 kDa protein
AAEL0017	serine/threonine-protein phosphatase 6 regulatory subunit 3	0.21615028589534	0.0640077950238497	0.395036706780325	NA		NA	XP_019527150.1 PRE	XP_021696271.1serine/threonine-protein phosphatase 6 regulatory subunit 3
AAEL0195	NA	0.216205280803158	0.260499541022916	0.693409539049138	NA		aag04013	NA	XP_021706656.1protein tramtrack, beta isoform isoform X1
AAEL0215	NA	0.216491445425199	0.13752854416376	0.547141229310386		510	aag01100 ; aag	NA	XP_021699725.1mannosyl-oligosaccharide glucosidase GCS1
AAEL0105	glutathione S-transferase 1	0.216612710108685	0.189095322027523	0.616603527036593	NA		NA	KXJ72088.1 hypothe	XP_001654675.1glutathione S-transferase 1
AAEL0051	nicotinamide riboside kinase 1	0.216806878831826	0.0746474048171577	0.419283716747526	NA		NA	KFB51041.1 AGAP00	XP_001650392.1nicotinamide riboside kinase 1
AAEL0070	NA	0.216842041303592	0.503794697163077	0.858744381072989	NA		NA	KXJ70459.1 hypothe	XP_001658106.1cysteine and histidine-rich domain-containing protein
AAEL0147	lymphokine-activated killer T-cell-originated protein kinase	0.216905595652654	0.340228466733502	0.761379832492257	NA		NA	NA	XP_021694229.1 lymphokine-activated killer T-cell-originated protein kinase
AAEL0140	rho GTPase-activating protein gacF	0.217018763767088	0.197008733631774	0.630345746481079	NA		NA	NA	XP_021700251.1rho GTPase-activating protein gacF isoform X4
AAEL0069	SWI/SNF-related matrix-associated actin-dependent regulator of chromat	0.217187098294199	0.432604368165257	0.820005280371151	NA		NA	KXJ62488.1 hypothe	XP_001652438.1SWI/SNF-related matrix-associated actin-dependent regulator of chromatin subfamily A-like protein 1
AAEL0139	WD repeat domain phosphoinositide-interacting protein 2	0.217364747749695	0.111048859587495	0.502395086491705	NA		aag04140 ; aag	NA	XP_001657258.1WD repeat domain phosphoinositide-interacting protein 2 isoform X1
AAEL0053	sugar transporter SWEET1	0.217469123548663	0.434996426545446	0.820960220424486	NA		NA	KXJ79136.1 hypothe	XP_001650784.1sugar transporter SWEET1
AAEL0258	NA	0.217717407906904	0.427739742889992	0.816177865920377	NA		NA	NA	XP_001858111.1polynucleotide kinase- 3'-phosphatase
AAEL0271	NA	0.217842139331815	0.379082028475724	0.785666748907449	00562 ; 04070		aag01100 ; aag	NA	XP_021709977.11-phosphatidylinositol 4,5-bisphosphate phosphodiesterase epsilon-1
AAEL0248	NA	0.218011597081958	0.0744300076356613	0.419158464053461	NA		NA	NA	XP_021707794.1uncharacterized protein LOC5573843
AAEL0219	NA	0.218039003864828	0.348985846200931	0.767244408961813	NA		aag03018	NA	XP_021701567.1protein PAT1 homolog 1
AAEL0173	ragulator complex protein LAMTOR5 homolog	0.218069683607865	0.214828669168464	0.648028428468258	NA		aag04150	NA	XP_011493184.1ragulator complex protein LAMTOR5 homolog
AAEL0227	NA	0.218129172593595	0.179678393466753	0.604991500927545	NA		aag03010	NA	XP_021694846.139S ribosomal protein L32, mitochondrial
AAEL0108	protein RRP5 homolog	0.218201886306147	0.162264638779732	0.580707394694867	NA		NA	KXJ74020.1 hypothe	XP_001655011.1protein RRP5 homolog
AAEL0129	puromycin-sensitive aminopeptidase	0.218347517963524	0.0689936518991304	0.403354246998601	NA		NA	NA	XP_021698653.1puromycin-sensitive aminopeptidase isoform X2
AAEL0139	uncharacterized LOC5579059	0.218480110987614	0.342734626356209	0.763457766092916	NA		NA	NA	XP_001657255.2uncharacterized protein LOC5579059
AAEL0028	diphthine methyl ester synthase	0.218552625401515	0.204240193557119	0.637782699712521	NA		NA	KXJ74317.1 hypothe	XP_001662462.1diphthine methyl ester synthase
AAEL0110	uncharacterized LOC5574366	0.218577432704539	0.242267078468653	0.676994125742042	NA		NA	KXJ70009.1 hypothe	XP_021700188.1uncharacterized protein LOC5574366
AAEL0069	uncharacterized LOC5568492	0.218656240125494	0.44249530462705	0.824760338905837	NA		NA	KXJ79769.1 hypothe	XP_001658081.1uncharacterized protein LOC5568492
AAEL0014	vigilin	0.218916791510851	0.0829580851480795	0.437969190781771	NA		NA	KXJ82396.1 hypothe	XP_019535078.1 vigilin
AAEL0252	NA	0.218972160325353	0.449440272714462	0.829306073695304	NA		NA	NA	XP_021712701.1digestive organ expansion factor homolog
AAEL0014	NA	0.219509770292161	0.111541632824118	0.502879021478986	NA		NA	XP_019547811.1 PRE	XP_021696434.1coronin-1C isoform X4
AAEL0067	histone-arginine methyltransferase CARMER	0.219694507291983	0.183838797161908	0.611378609137648	NA		NA	KFB35345.1 AGAP00	XP_021710079.1histone-arginine methyltransferase CARMER
AAEL0033	protein phosphatase 1B	0.219716302011189	0.08892111256937	0.455343393357453	04660 ; 04658		aag04013	KXJ75624.1 hypothe	XP_021702814.1protein phosphatase 1B isoform X1
AAEL0060	heat shock protein STI1	0.219832145665049	0.245759569890572	0.679727114240672	NA		NA	KXJ70410.1 hypothe	XP_001657382.2heat shock protein STI1
AAEL0210	NA	0.219833830467312	0.108790568851166	0.495854325366716		970	NA	NA	XP_021705204.1leucine-rich repeat-containing protein 47
AAEL0124	tether containing UBX domain for GLUT4	0.2199909274302	0.236505419629604	0.670870063357945	NA		NA	KXJ84386.1 hypothe	XP_001656042.2tether containing UBX domain for GLUT4
AAEL0233	NA	0.220005909802825	0.131941875761965	0.538981683630591	NA		NA	NA	XP_021709001.1perlucin-like protein
AAEL0132	cyclic AMP-dependent transcription factor ATF-2	0.220101736015018	0.468521724523224	0.840088840311577	NA		aag04013 ; aag	NA	XP_001656558.1cyclic AMP-dependent transcription factor ATF-2
AAEL0042	vacuolar protein sorting-associated protein 18 homolog	0.220190716910412	0.219884254665629	0.652412844728166	NA		NA	KXJ74279.1 hypothe	XP_001648749.2vacuolar protein sorting-associated protein 18 homolog
AAEL0197	NA	0.220349462814767	0.157282304003673	0.574474189691589	NA		NA	NA	XP_021697739.1PHD finger protein rhinoceros
AAEL0047	integrator complex subunit 11	0.220379634442933	0.124948363356854	0.529850156502197	NA		NA	ETN63983.1 cleavage	XP_001649735.1integrator complex subunit 11
AAEL0238	NA	0.220490518043481	0.513678086535255	0.861309357197051	NA		NA	NA	XP_019562160.1 pikachurin
AAEL0019	protein catecholamines up	0.220714041896723	0.0845934907579148	0.441787345036511	NA		NA	XP_019551461.1 PRE	XP_001660807.2protein catecholamines up isoform X1
AAEL0239	NA	0.2207309007923	0.300768237557123	0.732963273834917	NA		NA	NA	XP_021701264.1nuclear receptor coactivator 2 isoform X6
AAEL0000	glutathione S-transferase D4	0.220831543562413	0.329770078152105	0.753175384470979	NA		NA	KXJ80496.1 hypothe	XP_001647878.2glutathione S-transferase D4
AAEL0036	tRNA (adenine(58)-N(1))-methyltransferase non-catalytic subunit TRM6	0.221105340498184	0.222138696708002	0.654118491874251	NA		NA	KXJ73785.1 hypothe	XP_021701460.1tRNA (adenine(58)-N(1))-methyltransferase non-catalytic subunit TRM6
AAEL0151	venom dipeptidyl peptidase 4	0.221253107876127	0.0564837193903162	0.374195955783275	NA		NA	NA	XP_021701697.1venom dipeptidyl peptidase 4 isoform X1
AAEL0010	uncharacterized LOC5568339	0.22132891573652	0.107180414881472	0.492398491132955	NA		aag01100 ; aag	XP_312251.5 AGAP0	XP_021710736.1uncharacterized protein LOC5568339 isoform X2

AAEL0153€	NA	0.221374099550346	0.375159990768223	0.782198815065306	NA	NA	NA	XP_001647772.2nuclear envelope integral membrane protein 1
AAEL0054(	protein CREG1	0.221584078374837	0.0994882416651205	0.478312852561358	NA	NA	XP_019526093.1 PRE	XP_001650872.2protein CREG1 isoform X1
AAEL0123€	protein bric-a-brac 1	0.221911731363043	0.410115471780975	0.805356078752463	NA	NA	XP_019530668.1 PRE	XP_001662540.2protein bric-a-brac 1 isoform X1
AAEL0152€	phosphatidylinositol 4-phosphate 5-kinase type-1 alpha	0.22193787396815	0.178967039925694	0.604350349136232	NA	aag01100 ; aag	NA	XP_021702857.1phosphatidylinositol 4-phosphate 5-kinase type-1 gamma isoform X2
AAEL0048€	uncharacterized LOC5565646	0.22204393120493	0.118565753734115	0.516591887244947	NA	NA	NA	ABF18027.1basic tail-containing putative salivary secreted peptide
AAEL0011⁄	vam6/Vps39-like protein	0.222068343605246	0.277851605365302	0.713122992430066	NA	NA	KXJ72966.1 hypothet	XP_001652387.1vam6/Vps39-like protein isoform X1
AAEL0107€	sodium-driven chloride bicarbonate exchanger	0.222196256900984	0.0640702666220496	0.395157234219907	NA	NA	XP_019554473.1 PRE	XP_021702997.1sodium bicarbonate cotransporter 3 isoform X17
AAEL0061⁄	Hermansky-Pudlak syndrome 5 protein homolog	0.222247806903086	0.231154743123719	0.66425254967488	NA	NA	KXJ79184.1 hypothet	XP_001657527.2Hermanskydlak syndrome 5 protein homolog
AAEL0093€	nucleoporin Nup37	0.222355420195207	0.47457287500548	0.844332076309675	NA	aag03013	NA	XP_001660021.2nucleoporin Nup37
AAEL0131€	zinc finger protein 600	0.222536958476115	0.190131886998037	0.618521820583848	NA	NA	NA	XP_021702661.1zinc finger protein 600
AAEL0198€	NA	0.222544399490586	0.0912645842484554	0.459220287049195	NA	NA	NA	XP_021704543.1basement membrane proteoglycan isoform X1
AAEL0092€	jmjC domain-containing protein 7	0.222585107104616	0.275762320486951	0.711001645592863	NA	NA	NA	XP_021700731.1jmjC domain-containing protein 7
AAEL0199€	NA	0.222643624107733	0.580939833218615	0.883312680977808	NA	NA	NA	XP_001651146.1trypsin alpha-3
AAEL0021€	seipin	0.222658990985309	0.130337866107844	0.537292312507224	NA	NA	KXJ80401.1 hypothet	XP_019560517.1 seipin
AAEL0282€	NA	0.222741013595257	0.145333774341498	0.558950568057442	NA	NA	NA	XP_021705302.1ubiquitin domain-containing protein 1
AAEL0003€	trans-1,2-dihydrobenzene-1,2-diol dehydrogenase	0.222988889058647	0.167260011302649	0.587554508928878	NA	NA	KXJ70788.1 hypothet	XP_001655538.2trans-1,2-dihydrobenzene-1,2-diol dehydrogenase isoform X1
AAEL0081⁄	zinc transporter ZIP14	0.223367819565853	0.101764979378471	0.483848092317719	NA	NA	XP_019558136.2 PRE	XP_021705481.1zinc transporter ZIP14
AAEL0290€	NA	0.22349491909343	0.0947177858788491	0.465843434343757	NA	NA	NA	XP_001661683.2perlucin-like protein
AAEL0087€	ATP synthase subunit s, mitochondrial	0.223643993913199	0.197385597848407	0.630455491635209	NA	NA	KFB40571.1 AGAP00	XP_001659515.1ATP synthase subunit s, mitochondrial
AAEL0052€	CLIP-associating protein	0.223937425811075	0.0577066182959579	0.3766073060705	NA	NA	NA	XP_021695279.1CLIP-associating protein isoform X4
AAEL0024€	protein RUFY3	0.22413499227838	0.221649019378891	0.654067488761644	NA	NA	XP_019554034.1 PRE	XP_001655214.2protein RUFY3
AAEL0077€	leucine-rich repeat-containing G-protein coupled receptor 4	0.224385531789888	0.0786773348177155	0.429137779293678	NA	NA	KXJ76602.1 hypothet	XP_001658654.1leucine-rich repeat-containing G-protein coupled receptor 4
AAEL0120€	sodium-independent sulfate anion transporter	0.224407286829773	0.337944772655189	0.760148569536312	NA	NA	KXJ78345.1 hypothet	XP_021713110.1sodium-independent sulfate anion transporter
AAEL0110€	guanine nucleotide exchange factor subunit Rich	0.224413965844218	0.0945099923811805	0.465640264332134	NA	NA	KXJ77594.1 hypothet	XP_021700193.1guanine nucleotide exchange factor subunit Rich
AAEL0138€	methylthioribose-1-phosphate isomerase	0.224568131393144	0.147119766654722	0.561952643558699	270	aag01100 ; aag	NA	XP_001664017.2methylthioribose-1-phosphate isomerase
AAEL0087€	ATP-dependent RNA helicase DBP2	0.224622350826859	0.0785046656384458	0.428723995967346	NA	aag03040	KXJ77345.1 hypothet	XP_001653435.1ATP-dependent RNA helicase DBP2
AAEL0037€	non-structural maintenance of chromosomes element 1 homolog	0.224647306232203	0.44055036689078	0.822798752521693	NA	NA	ETN66854.1 hypothe	XP_001657224.1non-structural maintenance of chromosomes element 1 homolog
AAEL0275€	NA	0.224647792787795	0.173492244384546	0.596418211439039	NA	NA	NA	XP_021702018.1RING finger protein 17 isoform X2
AAEL0087€	chymotrypsin-1	0.224686383165178	0.149990505896285	0.565429182817541	NA	NA	KXJ69221.1 hypothet	XP_019562485.1 chymotrypsin-1-like
AAEL0018€	UPF0047 protein YjbQ	0.224702621950388	0.215654685898721	0.648302652610268	NA	NA	CRK86555.1 CLUMA_	CRK86555.1CLUMA_CG000051, isoform A
AAEL0050€	ubiA prenyltransferase domain-containing protein 1 homolog	0.22471229039587	0.35297246615429	0.770045669405352	NA	NA	ETN59324.1 1,4-dihy	XP_001650206.1ubiA prenyltransferase domain-containing protein 1 homolog
AAEL0182€	NA	0.224884653928899	0.0722455173132179	0.413177905727925	NA	NA	NA	XP_021703930.1blastoderm-specific protein 25D isoform X1
AAEL0055(	putative transferase CAF17, mitochondrial	0.224985853016938	0.216223090483523	0.649374960388178	00670 ; 00260	NA	XP_015591189.1 PRE	XP_001651016.2putative transferase CAF17, mitochondrial
AAEL0081(	keratin, type I cytoskeletal 9	0.22507428849904	0.0611903785479909	0.386196065431147	NA	NA	KXJ80232.1 hypothet	XP_001653116.2keratin, type I cytoskeletal 9
AAEL0079⁄	glutathione S-transferase 1	0.225544360552029	0.205335902731376	0.638023927878659	NA	NA	KFB39337.1 glutathic	XP_021701982.1glutathione S-transferase 1
AAEL0053€	adenosine monophosphate-protein transferase FICD homolog	0.225650557226678	0.260352783185442	0.693219418655219	NA	NA	KFB36134.1 AGAP00	XP_001650824.1adenosine monophosphate-protein transferase FICD homolog
AAEL0019€	ribosome biogenesis protein BRX1 homolog	0.22569583985027	0.0704341757278528	0.408402826117014	NA	NA	XP_019552814.1 PRE	XP_001654329.2ribosome biogenesis protein BRX1 homolog
AAEL0063€	acyl-CoA:lysophosphatidylglycerol acyltransferase 1	0.225859386923817	0.100914476557251	0.481896737560883	00561 ; 00564	aag00564	KXJ83573.1 hypothet	XP_001651895.1acyl-CoA:lysophosphatidylglycerol acyltransferase 1
AAEL0194€	NA	0.22588799978509	0.0423474502730106	0.323939778330243	NA	NA	NA	XP_019559974.1 mucin-5AC-like
AAEL0018€	uncharacterized LOC5572814	0.225907873374035	0.333040415795817	0.755603950945478	NA	NA	KXJ78471.1 hypothet	XP_001654217.1uncharacterized protein LOC5572814
AAEL0023€	alpha-taxilin	0.225910789997731	0.176860006413035	0.601873438984005	NA	NA	KXJ80171.1 hypothet	XP_019549275.1 alpha-taxilin
AAEL0092€	inosine-5'-monophosphate dehydrogenase	0.225955703768151	0.105642199469491	0.490349840292762	00230 ; 00983	aag01100 ; aag	NA	XP_001653775.1inosine-5'-monophosphate dehydrogenase
AAEL0025€	THO complex subunit 1	0.225962151066474	0.302445462559466	0.735250294168217	NA	aag03013 ; aag	KXJ80892.1 hypothet	XP_001655616.2THO complex subunit 1
AAEL0033€	actin-related protein 1	0.225994365675152	0.0930960000357585	0.463565688489745	NA	NA	KFB38052.1 AGAP00	XP_001656729.1actin-related protein 1
AAEL0042€	hemK methyltransferase family member 1	0.226122916209165	0.200406282443313	0.633770569380376	NA	NA	KFB45626.1 hypothe	XP_021701078.1 hemK methyltransferase family member 1
AAEL0078€	protein spaetzle 4	0.226437963292104	0.433665965632909	0.820603375643436	NA	NA	XP_019540913.1 PRE	XP_021704087.1protein spaetzle 4
AAEL0082€	ribosomal L1 domain-containing protein CG13096	0.226551423315856	0.0666818304536926	0.399540230684495	NA	NA	KXJ80483.1 hypothet	XP_001659039.2ribosomal L1 domain-containing protein CG13096
AAEL0105€	ragulator complex protein LAMTOR2 homolog	0.226730852920822	0.238853014080156	0.673590387853384	NA	aag04150	KFB48713.1 AGAP00	XP_001660910.1ragulator complex protein LAMTOR2 homolog
AAEL0042€	NA	0.227192617083759	0.14409390393747	0.556638423329024	NA	NA	XP_001851449.1 trai	XP_019547656.1 translin-like
AAEL0009€	transmembrane channel-like protein 7	0.227206877953235	0.149507643207835	0.565105542550216	NA	NA	KFB49906.1 tmc7 prc	XP_021708478.1transmembrane channel-like protein 7
AAEL0055€	uncharacterized LOC5579825	0.227490258164667	0.258892925103917	0.691547675112889	NA	aag03015	NA	XP_021694561.1uncharacterized protein LOC5579825 isoform X4
AAEL0093€	cationic amino acid transporter 2	0.227566765994266	0.142427513206577	0.554732123210059	NA	NA	NA	XP_021709767.1cationic amino acid transporter 2 isoform X2
AAEL0109€	calcium-activated chloride channel regulator 2	0.227636955234422	0.477694165381537	0.845011493487133	NA	NA	KXJ75225.1 hypothet	XP_021692951.1calcium-activated chloride channel regulator 2
AAEL0035€	uncharacterized LOC5578357	0.227667694634894	0.439315936063199	0.822472169172494	NA	NA	KXJ84411.1 hypothet	XP_011493001.1uncharacterized protein LOC5578357
AAEL0101€	protein artichoke	0.227698207283216	0.118231282732635	0.516112079556788	NA	NA	NA	XP_001660651.2protein artichoke
AAEL0027€	37 kDa salivary gland allergen Aed a 2-like	0.227771216955714	0.386229056823481	0.791224994795546	NA	NA	KXJ84269.1 hypothet	XP_001662172.237 kDa salivary gland allergen Aed a 2-like isoform X1
AAEL0170€	cell wall protein DAN4	0.227907437064093	0.076046514157853	0.421910635808236	NA	NA	NA	XP_011493515.1cell wall protein DAN4 isoform X2
AAEL0103€	ribonuclease Z, mitochondrial	0.228189273481123	0.291822938337864	0.722646575683698	NA	aag03013	NA	XP_001654456.2ribonuclease Z, mitochondrial
AAEL0031€	probable RNA-binding protein EIF1AD	0.228336558358414	0.214426049043604	0.648028428468258	NA	NA	KXJ82248.1 hypothet	XP_001656497.1probable RNA-binding protein EIF1AD
AAEL0235€	NA	0.228420575615396	0.395988099474487	0.796693358396638	NA	NA	NA	XP_021708613.1regulator of nonsense transcripts 1 isoform X3
AAEL0016€	28S ribosomal protein S18c, mitochondrial	0.228446493502281	0.0664402716039952	0.399540230684495	NA	aag03010	KXJ77613.1 hypothet	XP_001659742.228S ribosomal protein S18c, mitochondrial
AAEL0019€	probable tRNA N6-adenosine threonylcarbamoyltransferase	0.228559393886713	0.241719542885498	0.676742771771529	NA	NA	XP_019552816.1 PRE	XP_001654333.2probable tRNA N6-adenosine threonylcarbamoyltransferase
AAEL0023€	vacuolar protein sorting-associated protein 28 homolog	0.22872349345791	0.160107087676178	0.577674739374419	NA	aag04144	ETN64264.1 vacuola	XP_001661380.1vacuolar protein sorting-associated protein 28 homolog
AAEL0084(	DNA damage-regulated autophagy modulator protein 1	0.228851981631893	0.180624549854646	0.605944990070163	NA	NA	KXJ75021.1 hypothet	XP_001653258.1DNA damage-regulated autophagy modulator protein 1
AAEL0027€	THUMP domain-containing protein 1 homolog	0.228924853916022	0.215432114398287	0.64825542223124	NA	NA	KFB36606.1 hypothe	XP_001662178.1THUMP domain-containing protein 1 homolog
AAEL0013€	COP9 signalosome complex subunit 9	0.229011563101635	0.141384477605782	0.552330595273689	NA	NA	ETN66899.1 Myelom	XP_019544319.1 COP9 signalosome complex subunit 9-like
AAEL0049€	protein SMG5	0.229275673647213	0.0546369871383279	0.367496244171354	NA	aag03015	XP_001848300.1 smg	XP_021700878.1protein SMG5
AAEL0129€	uncharacterized LOC5577100	0.229326069875197	0.318588292845853	0.745684464362801	NA	NA	NA	XP_001663189.2uncharacterized protein LOC5577100 isoform X2
AAEL0120€	39S ribosomal protein L47, mitochondrial	0.229370363000701	0.292659869446938	0.723512283028756	NA	NA	KXJ76220.1 hypothet	XP_021707300.139S ribosomal protein L47, mitochondrial
AAEL0024€	sphingomyelin phosphodiesterase	0.22946618583056	0.0718899101509978	0.411912175099304	NA	NA	KXJ72004.1 hypothet	XP_001655198.2sphingomyelin phosphodiesterase
AAEL0281€	NA	0.229543784859997	0.386240238711323	0.791224994795546	NA	NA	NA	XP_021712123.1zinc finger protein 275
AAEL0259(	NA	0.229599139001645	0.088489562858447	0.454905562806385	620	aag01100 ; aag	NA	XP_021712009.1hydroxyacylglutathione hydrolase, mitochondrial isoform X2
AAEL0004€	embryonic stem cell-specific 5-hydroxymethylcytosine-binding protein	0.229646919183286	0.607738203712257	0.893547770777888	NA	NA	KXJ68232.1 hypothet	XP_001656417.2embryonic stem cell-specific 5-hydroxymethylcytosine-binding protein
AAEL0066€	androgen-induced gene 1 protein	0.229849067223666	0.278015634649623	0.713122992430066	NA	NA	KXJ72878.1 hypothet	XP_001657926.1androgen-induced gene 1 protein isoform X1
AAEL0052€	protein sidekick-1	0.229873921913202	0.464205496974256	0.837274753622609	NA	NA	ETN61108.1 protein	XP_021710369.1protein sidekick-1
AAEL0068€	polycomb group RING finger protein 3	0.229896268108026	0.272541928559425	0.707756103918734	NA	NA	ETN58998.1 ring fing	XP_001652267.1polycomb group RING finger protein 3
AAEL0051€	venom carboxylesterase-6	0.229988815622331	0.390718818925948	0.793285976044306	NA	NA	KXJ83278.1 hypothet	XP_001650374.3venom carboxylesterase-6
AAEL0030€	NCK-interacting protein with SH3 domain	0.230013543192057	0.17939977008557	0.604797111430702	NA	NA	KXJ82100.1 hypothet	XP_001662980.2NCK-interacting protein with SH3 domain
AAEL0092€	serine protease SP24D	0.230277341081462	0.126653766061152	0.530481545423179	NA	NA	NA	XP_001659851.2serine protease SP24D
AAEL0271€	NA	0.230322505179249	0.443322317901372	0.82545872799161	NA	NA	NA	XP_019535588.1 ras-interacting protein RIP3
AAEL0138€	galectin-3-binding protein A	0.230449415780061	0.199782070031387	0.633375309392567	NA	NA	NA	XP_021697499.1galectin-3-binding protein A
AAEL0277€	NA	0.230453249983786	0.512104514326193	0.861177338330732	NA	NA	NA	NA

AAEL0001(	luciferin 4-monooxygenase	0.23070916466238	0.1159599534696	0.512764772622422	NA	NA	KXJ68600.1 hypothe	XP_001657680.2luciferin 4-monooxygenase
AAEL0054!	zinc transporter ZIP2	0.230757304772624	0.528024076476234	0.865973876319045	NA	NA	KXJ72646.1 hypothe	XP_001650950.2zinc transporter ZIP2
AAEL0026I	tryptase	0.231018952931012	0.104027779975314	0.486414446815465	NA	NA	XP_019531743.1 PRE	XP_019531780.1 tryptase-like
AAEL0067⁄	RNA-binding protein 8A	0.231323670286446	0.196216627386217	0.629450562922554	NA	aag03013 ; aag	ETN62618.1 RNA-bin	XP_001652167.1RNA-binding protein 8A
AAEL0128:	replication protein A 70 kDa DNA-binding subunit	0.231450906347788	0.187820419977442	0.614842940104027	NA	aag03420 ; aag	XP_019524885.1 PRE	XP_001662954.2replication protein A 70 kDa DNA-binding subunit
AAEL0147:	nuclear pore complex protein Nup214	0.231797503054234	0.126632566342416	0.530481545423179	NA	aag03013	NA	XP_001649467.1nuclear pore complex protein Nup214
AAEL0013:	uncharacterized LOC5570322	0.23193403070086	0.0527824526442165	0.362633125106993	NA	NA	KXJ71646.1 hypothe	XP_001653149.2uncharacterized protein LOC5570322 isoform X1
AAEL0004⁄	maternal effect protein oskar	0.23201450559302	0.150919649396022	0.560025396924239	NA	NA	KXJ73715.1 hypothe	XP_001656415.1maternal effect protein oskar
AAEL0127:	adenylate kinase	0.232205595054723	0.0520838744983567	0.361717594818022	00230 ; 00730	aag01100 ; aag	KFB36586.1 AGAP00	XP_001662844.1adenylate kinase
AAEL0114(	major facilitator superfamily domain-containing protein 6	0.23221504055065I	0.056395709017669	0.373885673184863	NA	NA	KXJ72609.1 hypothe	XP_021698279.1major facilitator superfamily domain-containing protein 6 isoform X2
AAEL0117(	NA	0.232221293231862	0.0547057825487988	0.367496244171354	NA	aag04141	KXJ68448.1 hypothe	XP_001655641.2heat shock protein 83
AAEL0262(	NA	0.232246445449753	0.455779756419738	0.832617462606238	NA	NA	NA	XP_021693645.1uncharacterized protein LOC5576566 isoform X1
AAEL0023(	delta-aminolevulinic acid dehydratase	0.232350364948926	0.130173760874273	0.537053220341536	860	aag01100 ; aag	KXJ72354.1 hypothe	XP_001661237.1delta-aminolevulinic acid dehydratase
AAEL0058!	uncharacterized LOC5567159	0.23235576972331	0.170249552458427	0.590883473644663	NA	NA	KFB34830.1 AGAP00	KFB34830.1AGAP003535-like protein
AAEL0060!	ras-related protein Rab6	0.232416150247635	0.111692313224623	0.50308011076504	NA	NA	XP_008200264.1 PRE	XP_019525274.1 ras-related protein Rab6 isoform X1
AAEL0125:	CD151 antigen	0.232417902809388	0.240476129793231	0.675476601452171	NA	NA	KXJ76303.1 hypothe	XP_001656130.2CD151 antigen
AAEL0032:	ubiquitin-like domain-containing CTD phosphatase 1	0.232439499707207	0.188228737270759	0.615250207631631	04660 ; 04658	NA	KXJ71025.1 hypothe	XP_001656652.2ubiquitin-like domain-containing CTD phosphatase 1
AAEL0275!	NA	0.232463718008942	0.0393136714383386	0.313353264954028	NA	NA	NA	XP_021701416.1zinc finger CCCH domain-containing protein 15 homolog
AAEL0090	nurim homolog	0.232472320491813	0.295966441612155	0.72758553286062	NA	NA	KXJ74099.1 hypothe	XP_001659696.2nurim homolog
AAEL0072⁄	anaphase-promoting complex subunit 11	0.232491923123377	0.420389542444772	0.813636239562413	NA	aag04120	ETN61936.1 RING-bc	XP_001658265.1anaphase-promoting complex subunit 11
AAEL0007:	basement membrane-specific heparan sulfate proteoglycan core protein	0.232566169314738	0.0621740271178565	0.389280355822487	NA	NA	XP_019529814.1 PRE	XP_001650977.2basement membrane-specific heparan sulfate proteoglycan core protein
AAEL0002!	DNA/RNA-binding protein KIN17	0.23257734216287	0.457598773582635	0.833283524364043	NA	NA	KXJ74463.1 hypothe	XP_001654770.1DNA/RNA-binding protein KIN17
AAEL0001(	mitochondrial import inner membrane translocase subunit Tim22	0.232798613619971	0.215209456578065	0.648208527708858	NA	NA	ETN62370.1 mitocho	XP_001658891.1mitochondrial import inner membrane translocase subunit Tim22
AAEL0218(	NA	0.232826047272754	0.546218581579164	0.870931414970375	NA	NA	NA	XP_021701485.1probable methyltransferase BMT2 homolog
AAEL0145:	GATOR complex protein WDR59	0.232848931258554	0.203097074996313	0.635896319876174	NA	aag04150	NA	XP_001648959.1GATOR complex protein WDR59 isoform X2
AAEL0224!	NA	0.233104193557761	0.0662199665537164	0.399054441537196	564	aag01100 ; aag	NA	XP_021713201.1CDP-diacylglycerol--glycerol-3-phosphate 3-phosphatidyltransferase, mitochondrial isoform X3
AAEL0005(	uncharacterized LOC5578434	0.233618147647336	0.331125201341393	0.754128446928769	901	NA	KXJ70817.1 hypothe	XP_001656949.2uncharacterized protein LOC5578434
AAEL0069!	cap-specific mRNA (nucleoside-2'-O-)-methyltransferase 1	0.233716525497112	0.285822099448824	0.717970179170389	NA	NA	KFB44010.1 AGAP00	XP_001652367.1cap-specific mRNA (nucleoside-2'-O-)-methyltransferase 1
AAEL0139!	zinc finger protein 1 homolog	0.233883959069763	0.485695943523958	0.847790841886424	NA	NA	NA	XP_001657252.1zinc finger protein 1 homolog
AAEL0145!	homeotic protein caudal	0.233891768685336	0.278923033674118	0.713552892930006	NA	NA	NA	XP_001649020.1homeotic protein caudal isoform X2
AAEL0226!	NA	0.233919134812495	0.349709406507035	0.767801448844281	NA	NA	NA	XP_021700269.1uncharacterized protein LOC5576459
AAEL0118!	serine/threonine-protein kinase RIO1	0.234077442713184	0.10677964734673	0.492031204248678	04151 ; 05165 ; 04714 ;	aag03008	KXJ80837.1 hypothe	XP_001661999.1serine/threonine-protein kinase RIO1
AAEL0067(	venom serine protease 34	0.234141512783429	0.468029153265263	0.839644234660078	NA	NA	ETN67733.1 achelase	XP_001657965.2venom serine protease 34
AAEL0004	monocarboxylate transporter 5	0.234355648100351	0.0705414496085817	0.408510018438117	NA	NA	KXJ81625.1 hypothe	XP_001656435.2monocarboxylate transporter 5
AAEL0069⁄	suppressor of cytokine signaling 7	0.234593771734805	0.306252947395537	0.738783696372872	NA	NA	XP_003437124.1 AG,	ABV01934.1suppressor of cytokine signaling 7
AAEL0251!	NA	0.234643152239264	0.321634283105594	0.746595392577842	NA	NA	NA	XP_021694756.1uncharacterized protein LOC110674775
AAEL0062	sugar transporter SWEET1	0.23467020204885	0.329054132340262	0.752367596512046	NA	NA	XP_019537842.1 PRE	XP_001651861.1sugar transporter SWEET1
AAEL0117!	CD2 antigen cytoplasmic tail-binding protein 2 homolog	0.234715543565657	0.208984878008484	0.639832374753494	NA	NA	KXJ68124.1 hypothe	XP_001656211.1CD2 antigen cytoplasmic tail-binding protein 2 homolog isoform X1
AAEL0004:	tryptophan 2,3-dioxygenase	0.234722956555081	0.0907514911425007	0.458582874647784	380	aag01100 ; aag	KXJ81288.1 hypothe	XP_001656460.1tryptophan 2,3-dioxygenase
AAEL0226:	NA	0.234739500847413	0.272481707096508	0.707756103918734	NA	NA	NA	XP_021711436.1uncharacterized protein LOC5577501
AAEL0033⁄	argininosuccinate lyase	0.234759988638403	0.131769257887358	0.538981683630591	00250 ; 00220	aag01100 ; aag	KXJ75669.1 hypothe	XP_001656695.1argininosuccinate lyase
AAEL0182(	NA	0.234805326656341	0.202411824714887	0.635477395133979	NA	NA	NA	XP_021704441.1zinc finger protein 420 isoform X2
AAEL0028(	UDP-N-acetylglucosamine transferase subunit ALG14 homolog	0.234976901948352	0.117596015225379	0.515237493575542	NA	aag01100 ; aag	KXJ84314.1 hypothe	XP_001662485.2UDP-N-acetylglucosamine transferase subunit ALG14 homolog
AAEL0100:	uncharacterized LOC5572757	0.235152749906142	0.410717333141469	0.805855841282375	NA	NA	NA	XP_021697630.1uncharacterized protein LOC5572757
AAEL0031(	nuclear protein 1	0.235552088017971	0.0659811771902428	0.398594933512758	NA	NA	KDR15750.1 Nuclear	XP_021711870.1nuclear protein 1
AAEL0027!	uncharacterized LOC5576123	0.235722104913596	0.368399579888055	0.78164928156096	NA	NA	KXJ72709.1 hypothe	XP_001662469.1uncharacterized protein LOC5576123
AAEL0053(	tubulin-specific chaperone D	0.235759503188434	0.344588778316466	0.764706052029763	NA	NA	KFB46964.1 AGAP00	XP_001650699.1tubulin-specific chaperone D
AAEL0279(	NA	0.235875017117795	0.0952253577693305	0.466773927728342	NA	NA	NA	XP_001654033.1SUMO-conjugating enzyme UBC9-B
AAEL0236	NA	0.235929271285212	0.143016261069521	0.555375365647863	NA	NA	NA	XP_019562518.1 roquin-1
AAEL0197(	NA	0.235978447756828	0.532621890560962	0.866700945213732	NA	NA	NA	XP_019549915.1 contactin-2-like
AAEL0002(	transmembrane emp24 domain-containing protein 2	0.236194078041433	0.0441533028688937	0.332214793948945	NA	NA	KFB43477.1 AGAP00	XP_001658907.1transmembrane emp24 domain-containing protein 2
AAEL0209:	NA	0.236230428678464	0.212006751125682	0.644921032680504	NA	aag04013	NA	XP_021695449.1protein daughter of sevenless
AAEL0055:	synaptic vesicle glycoprotein 2B	0.236281224546457	0.423265596001744	0.814192545906728	NA	NA	KXJ70135.1 hypothe	XP_001651077.2synaptic vesicle glycoprotein 2B
AAEL0005	uncharacterized LOC5564304	0.236286569240369	0.512593912784995	0.861217671252058	NA	NA	KXJ84135.1 hypothe	XP_021700920.1uncharacterized protein LOC5564304 isoform X1
AAEL0119!	src substrate cortactin-like	0.236307594633469	0.132793177428649	0.540213447700453	NA	NA	XP_019530652.1 PRE	XP_021693740.1src substrate cortactin-like isoform X2
AAEL0078!	spermatogenesis-associated protein 5	0.236517774701655	0.350677539818838	0.768176246660341	NA	aag03008	KXJ82590.1 hypothe	XP_001652994.2spermatogenesis-associated protein 5
AAEL0145:	puff-specific protein Bx42	0.236786726395701	0.0839331112178474	0.439585935928646	NA	aag03040 ; aag	NA	XP_001648953.1puff-specific protein Bx42
AAEL0267!	NA	0.237015323669835	0.131792130240485	0.538981683630591	NA	NA	NA	NA
AAEL0047	leucine carboxyl methyltransferase 1	0.237075646438398	0.144150115495877	0.556638423329024	NA	NA	ETN57780.1 leucine	XP_001649746.1leucine carboxyl methyltransferase 1
AAEL0196(	NA	0.237093540033457	0.10616576095047	0.490844601505817	00513 ; 00603 ; 00604 ;	aag01100 ; aag	NA	XP_021697274.1probable beta-hexosaminidase fdl isoform X1
AAEL0035(	uncharacterized LOC5578605	0.237115424187006	0.244081882045512	0.679177949374903	NA	NA	NA	XP_001657033.2uncharacterized protein LOC5578605
AAEL0281(	NA	0.237178110918345	0.454383150590943	0.832617462606238	NA	NA	NA	XP_021695339.1 general transcription factor 3C polypeptide 5
AAEL0078:	3-oxoacyl-[acyl-carrier-protein] reductase FabG	0.237228653229956	0.0743240379791017	0.418818002133309	NA	NA	XP_001863343.1 3-h	XP_001863343.13-hydroxybutyrate dehydrogenase type 2
AAEL0079!	zinc transporter ZIP9	0.237256786997737	0.0519792901569521	0.361717594818022	NA	NA	KXJ76161.1 hypothe	XP_001658744.1zinc transporter ZIP9
AAEL0095(	lazarillo protein	0.237407502366225	0.235033233673219	0.668761847947112	NA	NA	NA	XP_001660226.2lazarillo protein
AAEL0211:	NA	0.237440868257035	0.630471397525509	0.90233929580663	NA	NA	NA	NA
AAEL0040(	retinitis pigmentosa 1-like 1 protein	0.237497020867063	0.156903861763509	0.574474189691589	NA	NA	KXJ74542.1 hypothe	XP_001648378.2retinitis pigmentosa 1-like 1 protein
AAEL0093(	RCC1-like G exchanging factor-like protein	0.237510558493066	0.343983837228501	0.764585185963472	NA	NA	NA	XP_001659905.2RCC1-like G exchanging factor-like protein
AAEL0006	tRNA (uracil-5-)-methyltransferase homolog A	0.237636495462122	0.137593460092239	0.547162929891436	NA	NA	KXJ76670.1 hypothe	XP_001649776.1tRNA (uracil-5-)-methyltransferase homolog A
AAEL0198:	NA	0.237661579134907	0.261328517891172	0.694591903258144	NA	NA	NA	XP_001648230.2uncharacterized protein LOC5563908
AAEL0186(	NA	0.237765423044296	0.544540853966764	0.870429396451444	NA	aag01100 ; aag	NA	YP_003934134.1NADH dehydrogenase subunit 1 (mitochondrion)
AAEL0096(	tRNA (guanine(37)-N1)-methyltransferase	0.238098103907773	0.197291138576851	0.630372589300064	NA	NA	NA	XP_021698796.1tRNA (guanine(37)-N1)-methyltransferase
AAEL0013:	transcription factor Dp-1	0.238144777071453	0.0737618812037167	0.417913179290884	NA	NA	XP_019544297.1 PRE	XP_021710033.1transcription factor Dp-1 isoform X1
AAEL0100(	signal recognition particle subunit SRP68	0.238255588853241	0.0771865549206578	0.424916680487529	NA	aag03060	NA	XP_001660604.1signal recognition particle subunit SRP68
AAEL0014!	EP300-interacting inhibitor of differentiation 3	0.23829350234407	0.404121189568684	0.800429773223221	NA	NA	KXJ72412.1 hypothe	XP_001659296.2EP300-interacting inhibitor of differentiation 3
AAEL0055(	nucleosome assembly protein 1-like 4	0.238507536473406	0.0578900429899024	0.377002247411947	NA	NA	ETN64246.1 nucleos	XP_001651086.1nucleosome assembly protein 1-like 4
AAEL0195:	NA	0.238669062325398	0.132431253688534	0.539973327406722	NA	aag04144 ; aag	NA	XP_021710117.1cation-independent mannose-6-phosphate receptor
AAEL0128	protein SYS1 homolog	0.238851866585647	0.273527379277689	0.708614567599462	NA	NA	KFB37453.1 AGAP00	XP_001662943.2protein SYS1 homolog
AAEL0017(	protein odr-4 homolog	0.238864022895239	0.094146536760932	0.464823706598816	NA	NA	KXJ76417.1 hypothe	XP_001659942.1protein odr-4 homolog
AAEL0051⁄	DNA-binding protein D-ETS-4	0.238895151528847	0.0889103105325796	0.455343393357453	NA	NA	XP_019540239.1 PRE	XP_001650415.2DNA-binding protein D-ETS-4 isoform X1
AAEL0104:	facilitated trehalose transporter Tret1	0.239082671768633	0.243260371822212	0.677917002273772	NA	NA	NA	XP_001660879.1facilitated trehalose transporter Tret1
AAEL0001	anaphase-promoting complex subunit 4	0.239553256216746	0.516340125149996	0.861448202130317	NA	aag04120	KXJ68308.1 hypothe	XP_001657691.1anaphase-promoting complex subunit 4

AAEL00110	glutaredoxin-related protein 5, mitochondrial	0.239557414389482	0.14749725661827	0.561952643558699	NA	NA	ETN60332.1 glutared	XP_001652385.1glutaredoxin-related protein 5, mitochondrial
AAEL00953	acyl-protein thioesterase 2	0.239601521875703	0.0526094957335558	0.362633125106993	NA	aag00564	NA	XP_001660206.1acyl-protein thioesterase 2
AAEL00799	arf-GAP with dual PH domain-containing protein 1	0.239870215243247	0.237274305475157	0.671758630247645	NA	NA	KFB35906.1 AGAP00	XP_021696245.1arf-GAP with dual PH domain-containing protein 1
AAEL00666	CMP-sialic acid transporter 1	0.239898622038331	0.171513354194841	0.593333039586814	NA	NA	KXJ80369.1 hypothet	XP_001657933.2CMP-sialic acid transporter 1
AAEL00596	ubiquitin-fold modifier-conjugating enzyme 1	0.239932672363897	0.109053577002371	0.496541818691649	NA	NA	ETN62096.1 Ufm1-cc	XP_001651749.1ubiquitin-fold modifier-conjugating enzyme 1
AAEL01831	NA	0.24015622000694	0.112353744309725	0.503547757511443	NA	NA	NA	XP_021706270.1uncharacterized protein LOC5577382 isoform X1
AAEL01521	nucleosomal histone kinase 1	0.240560859369279	0.139004352292288	0.549029662748535	NA	NA	NA	XP_021693350.1nucleosomal histone kinase 1
AAEL01832	NA	0.240670938130345	0.0353461482809462	0.297809790794065	NA	NA	NA	XP_021707339.1ribosome-binding protein 1 isoform X1
AAEL00399	uncharacterized LOC5563909	0.24070122632388	0.473337745310613	0.843301826204891	NA	NA	KXJ78260.1 hypothet	XP_001648228.1uncharacterized protein LOC5563909
AAEL00443	RNA/RNP complex-1-interacting phosphatase	0.240798590923974	0.192404684515399	0.622979559081881	NA	NA	XP_019541177.1 PRE	XP_021706032.1RNA/RNP complex-1-interacting phosphatase
AAEL01216	uncharacterized LOC5575915	0.24087071604189	0.310273726091772	0.742918550320373	NA	NA	KXJ79692.1 hypothet	XP_021699455.1uncharacterized protein LOC5575915
AAEL02576	NA	0.240874362870519	0.0319830727528057	0.284442054201394	NA	NA	NA	XP_021705306.1ADP-ribosylation factor 2
AAEL00188	AF4/FMR2 family member 4	0.240944915297374	0.134683663512057	0.542662316284181	NA	NA	NA	XP_001654220.2uncharacterized protein LOC5572816 isoform X3
AAEL01283	histone chaperone asf1	0.241084510506711	0.205856666145592	0.638023927878659	00730 ; 00230	NA	KFB44452.1 AGAP00	XP_001656285.1histone chaperone asf1 isoform X1
AAEL02353	NA	0.241236781941653	0.458827747765485	0.833589918052911	NA	NA	NA	XP_021704702.1methyltransferase-like protein 25
AAEL00362	acyl-CoA Delta(11) desaturase	0.241248121064988	0.322664981703575	0.746650319748046	NA	aag01100 ; aag	XP_019541612.1 PRE	XP_001657078.2acyl-CoA Delta(11) desaturase isoform X1
AAEL00709	phosphoglycolate phosphatase 1B, chloroplastic	0.241342658116899	0.436321057664702	0.820960220424486	NA	aag00981	KXJ81037.1 hypothet	XP_001658126.1phosphoglycolate phosphatase 1B, chloroplastic
AAEL00004	uncharacterized LOC5563618	0.241367693791189	0.135641625930347	0.544816342999152	NA	NA	KXJ84464.1 hypothet	XP_019931946.1 three-prime repair exonuclease 1-like
AAEL00391	trafficking protein particle complex subunit 6B	0.241374828618346	0.153350726836425	0.568818435996349	NA	NA	XP_001847380.1 traf	XP_001647996.1trafficking protein particle complex subunit 6B
AAEL00825	probable trafficking protein particle complex subunit 2	0.241652305635559	0.216948640213237	0.649748124914163	NA	NA	KFB42813.1 AGAP00	XP_021708394.1probable trafficking protein particle complex subunit 2
AAEL02600	NA	0.241711899919584	0.53339178567011	0.866700945213732	NA	NA	NA	XP_021702274.1hormone receptor 4-like
AAEL00211	zinc finger E-box-binding homeobox 1	0.241901592476256	0.232516043246761	0.666707644289445	NA	NA	KXJ82740.1 hypothet	XP_021705166.1zinc finger E-box-binding homeobox 1 isoform X2
AAEL01174	ATP-dependent RNA helicase abstrakt	0.241981028312545	0.27832389527094	0.713122992430066	NA	NA	KFB44006.1 DEAD bc	XP_001661885.1ATP-dependent RNA helicase abstrakt
AAEL00085	surface antigen CRP170	0.242092808436892	0.390379265297909	0.793285976044306	NA	NA	KXJ74044.1 hypothet	XP_001651623.2surface antigen CRP170
AAEL00794	glutathione S-transferase 1	0.242379825963735	0.112882188975106	0.503928507434265	NA	NA	KXJ72421.1 hypothet	XP_001658755.1glutathione S-transferase 1
AAEL02118	NA	0.242619817467767	0.427259503312549	0.815782298406567	NA	NA	NA	NA
AAEL00824	small integral membrane protein 8	0.242818622323256	0.0388569065427493	0.310922829570765	NA	NA	KFB42815.1 AGAP00	XP_001653136.2small integral membrane protein 8
AAEL00356	bromodomain-containing protein 8	0.242845650307195	0.366615721953274	0.780578423384551	NA	NA	KXJ70441.1 hypothet	XP_001663940.1bromodomain-containing protein 8
AAEL02156	NA	0.242918327720156	0.563245146811749	0.879621058497374	NA	NA	NA	NA
AAEL00341	sorting nexin lst-4	0.243253012997349	0.0880245041286436	0.453528268192485	NA	NA	KXJ75862.1 hypothet	XP_001656772.1sorting nexin lst-4
AAEL01162	DNA cross-link repair 1A protein	0.243370518451957	0.281357196451957	0.715107379448722	NA	NA	XP_019542278.1 PRE	XP_021705427.1DNA cross-link repair 1A protein isoform X2
AAEL00180	uncharacterized LOC5572461	0.243442079195687	0.048002260209218	0.348632042971763	NA	NA	KXJ77996.1 hypothet	XP_021702480.1uncharacterized protein LOC5572461
AAEL01482	flavin reductase (NADPH)	0.244005141970174	0.0350050622245845	0.296063035469325	NA	aag01100 ; aag	NA	XP_001649677.1flavin reductase (NADPH)
AAEL00547	peroxisome biogenesis factor 2	0.244142250060236	0.474710774312107	0.844332076309675	NA	aag04146	KXJ76804.1 hypothet	XP_001650927.1peroxisome biogenesis factor 2
AAEL02181	NA	0.244336002712571	0.085315142218134	0.443793068790994	901	NA	NA	EAT42254.1AAEL006190
AAEL00894	chaoptin	0.244584689678673	0.218000810779508	0.65121672666453	NA	NA	XP_019539219.1 PRE	XP_021696265.1 chaoptin
AAEL01732	B-box type zinc finger protein ncl-1	0.244940835374453	0.245213036148257	0.679727114240672	NA	NA	NA	XP_021711806.1B-box type zinc finger protein ncl-1
AAEL01009	G2/mitotic-specific cyclin-B	0.244968376259722	0.141998091206005	0.553914007314906	NA	aag04068	NA	XP_001660628.1G2/mitotic-specific cyclin-B
AAEL00204	protein cycle	0.24509332051756	0.0512694562398898	0.359690131564899	NA	aag04711	NA	XP_021697826.1protein cycle isoform X3
AAEL00455	biorientation of chromosomes in cell division protein 1-like 1	0.245181269038911	0.0683484958948182	0.401556773593974	NA	NA	XP_001845074.1 con	XP_001649413.2biorientation of chromosomes in cell division protein 1-like 1
AAEL00612	mitochondrial import inner membrane translocase subunit TIM50-C	0.24540284570751	0.118140044592937	0.515958562099766	NA	NA	KXJ74443.1 hypothet	XP_001657532.1mitochondrial import inner membrane translocase subunit TIM50-C
AAEL00104	guanine nucleotide-binding protein subunit beta-1	0.245868025859691	0.0279065052026256	0.271454186970995	NA	NA	KFB44464.1 AGAP00	XP_021697411.1guanine nucleotide-binding protein subunit beta-1
AAEL00219	putative oligosaccharyltransferase complex subunit CG9662	0.246005940637533	0.108102881761794	0.494284290519081	NA	NA	KFB38682.1 hypothe	XP_001654974.1putative oligosaccharyltransferase complex subunit CG9662
AAEL02452	NA	0.246092309816095	0.0752837327382342	0.420011717015769	NA	NA	NA	XP_021699084.1proton-coupled amino acid transporter 1-like
AAEL02011	NA	0.246353951319003	0.102380904805032	0.484636597025644	NA	NA	NA	XP_021706163.1dynactin subunit 2
AAEL01070	AP-1 complex subunit mu-1	0.246381514584596	0.0506868150019136	0.35814179653755	NA	aag04142	ETN64452.1 clathrin	XP_019532664.1 AP-1 complex subunit mu-1
AAEL00394	nucleolar protein 16	0.24688356523756	0.121769745668418	0.523911973275319	NA	NA	KXJ76210.1 hypothet	XP_001648060.1nucleolar protein 16
AAEL02045	NA	0.247484666724587	0.169155962111058	0.589285695468509	NA	NA	NA	XP_021704042.1protein KBP homolog
AAEL01469	uncharacterized LOC5565018	0.247517983793668	0.2263726797122	0.660446829146891	NA	NA	NA	XP_001649358.1uncharacterized protein LOC5565018
AAEL02436	NA	0.247884358407469	0.318786726684725	0.745684464362801	NA	NA	NA	NA
AAEL00842	sodium-dependent nutrient amino acid transporter 1	0.247921155624066	0.34677696969068	0.765398366276275	NA	NA	NA	XP_021706426.1sodium-dependent nutrient amino acid transporter 1 isoform X1
AAEL00196	transmembrane protein 184C	0.248066936764966	0.230849585947334	0.664044354450569	NA	NA	KXJ74361.1 hypothet	XP_021693214.1transmembrane protein 184C-like
AAEL00231	insulin-like receptor	0.248402211084863	0.266852466060179	0.702817294887306	NA	NA	Q93105.2 RecName:	XP_021707182.1insulin-like receptor isoform X1
AAEL00913	cytochrome P450 6a2	0.24867784507519	0.146927405566177	0.561705852106341	NA	NA	KXJ81552.1 hypothet	XP_021703140.1cytochrome P450 6a2
AAEL02318	NA	0.248777594285177	0.0625772637973965	0.391192922189974	NA	aag01100 ; aag	NA	XP_021696014.1dolichyl-diphosphooligosaccharide--protein glycosyltransferase subunit 2
AAEL00565	sorting nexin-2	0.2489805160741	0.130010658635908	0.536965027274516	NA	aag04144	KFB49515.1 AGAP00	XP_001651217.2sorting nexin-2
AAEL00077	E3 ubiquitin-protein ligase TRIM37	0.248990868188217	0.23264141030883	0.666707644289445	NA	NA	KXJ80965.1 hypothet	XP_001650995.2E3 ubiquitin-protein ligase TRIM37
AAEL00002	serine protease easter	0.249523644651942	0.0715898099608445	0.411730894537307	NA	NA	XP_001866674.1 ani	XP_001647865.1serine protease easter
AAEL00728	uncharacterized LOC5568991	0.249764698133252	0.262247888317197	0.695849212311086	NA	NA	CRK88797.1 CLUMA_	CRK88797.1CLUMA_CG002625, isoform A
AAEL01940	NA	0.250030001672647	0.142136065951616	0.553975467550517	NA	aag03450	NA	XP_021706756.1X-ray repair cross-complementing protein 6
AAEL01427	multiple coagulation factor deficiency protein 2	0.250102943608472	0.0526507684850824	0.362633125106993	NA	NA	NA	XP_021693749.1nuclear transcription factor Y subunit beta isoform X3
AAEL02592	NA	0.25020005404841	0.147150628424342	0.561952643558699	NA	NA	NA	XP_021710290.1uncharacterized protein LOC5574819
AAEL01045	WASH complex subunit 1	0.250260044491654	0.294103983676241	0.724764746928686	NA	aag04144	NA	XP_001660831.2WASH complex subunit 1
AAEL00566	caltractin	0.250308548386198	0.135351384639743	0.543887965700835	NA	NA	ETN61930.1 centrin [	XP_019530267.1 caltractin-like
AAEL02259	NA	0.250467961631219	0.392062116128905	0.793285976044306	NA	NA	NA	XP_021698402.1kinesin-like protein subito isoform X2
AAEL01228	NA	0.250870649593854	0.232825706066159	0.666707644289445	NA	aag03020	KFB49101.1 AGAP00	XP_001649871.1probable DNA-directed RNA polymerases I and III subunit RPAC2
AAEL01038	pre-mRNA-processing factor 6	0.250899388858174	0.258345503501176	0.691547675112889	NA	aag03040	NA	XP_001654491.2pre-mRNA-processing factor 6
AAEL01867	NA	0.250910804559266	0.384740693123454	0.790080064171371	190	aag01100 ; aag	NA	YP_009389267.1NADH dehydrogenase subunit 5 (mitochondrion)
AAEL00448	sodium/hydrogen exchanger 8	0.250966864839912	0.11234846390323	0.503547757511443	NA	NA	KXJ72926.1 hypothet	XP_001649290.2sodium/hydrogen exchanger 8
AAEL00025	suppressor of cytokine signaling 6	0.251079874074094	0.446466567380597	0.828189271329869	NA	NA	ETN64882.1 hypothe	XP_001660156.2suppressor of cytokine signaling 6
AAEL00602	leucine-rich PPR motif-containing protein, mitochondrial	0.251214099801079	0.121781062707971	0.523911973275319	NA	NA	KXJ67811.1 hypothet	XP_001657384.1leucine-rich PPR motif-containing protein, mitochondrial
AAEL01455	zinc finger protein 679	0.251480572307901	0.48919281938055	0.849220154814801	NA	NA	NA	XP_001649016.2zinc finger protein 679
AAEL00126	mediator of RNA polymerase II transcription subunit 20	0.251483820368786	0.415095214097865	0.809508847956623	NA	NA	ETN64165.1 trf-proxi	XP_001652880.1mediator of RNA polymerase II transcription subunit 20
AAEL00904	WASH complex subunit 5	0.251510253114731	0.198443381681326	0.631728026149226	NA	aag04144	KXJ78902.1 hypothet	XP_001653658.2WASH complex subunit 5 isoform X2
AAEL02667	NA	0.251620080098434	0.432236540100893	0.820005280371151	NA	NA	NA	XP_021699607.1UDP-glucuronosyltransferase 2B15-like
AAEL00805	liprin-alpha-1	0.251627598990466	0.12122685941327	0.523231501088606	NA	NA	NA	XP_001658870.1liprin-alpha-1 isoform X3
AAEL02738	NA	0.251689715804891	0.0546131158837698	0.367496244171354	NA	NA	NA	XP_021695783.1LOW QUALITY PROTEIN: uncharacterized protein LOC5570673
AAEL01363	uncharacterized LOC5578321	0.251819535678824	0.221477432891408	0.654054986350045	NA	NA	NA	XP_001663823.1uncharacterized protein LOC5578321
AAEL02592	NA	0.251951781323682	0.0692430867290276	0.403786365070033	NA	NA	NA	XP_021702718.1mitochondrial basic amino acids transporter-like
AAEL00515	uncharacterized LOC5566048	0.252022081398938	0.256076338315972	0.688406212440425	NA	NA	NA	XP_021705655.1uncharacterized protein LOC5566048 isoform X2
AAEL01382	helicase SKI2W	0.252083473944126	0.116765306059524	0.514582987743323	NA	aag03018	NA	XP_021707890.1helicase SKI2W
AAEL01495	N-acetylgalactosamine kinase	0.252089398731785	0.0831996090535203	0.438013925780757	00052 ; 00520	NA	NA	XP_001650075.2N-acetylgalactosamine kinase

AAEL00396	zinc transporter 9	0.252300671539894	0.268923802796148	0.7050247388405	NA	NA	KXJ70959.1 hypothet	XP_001648127.2zinc transporter 9
AAEL00780	cytochrome P450 4d1	0.252318614289944	0.0937732971096018	0.464823706598816	NA	NA	KXJ72911.1 hypothet	XP_001652927.1cytochrome P450 4d1 isoform X2
AAEL00730	conserved oligomeric Golgi complex subunit 7	0.252373667340312367	0.139661640348971	0.549451224664913	NA	NA	XP_019546236.1 PRE	XP_001652664.2conserved oligomeric Golgi complex subunit 7 isoform X2
AAEL01244	ileal sodium/bile acid cotransporter	0.252416415350905	0.067516173488667	0.400828276414654	NA	NA	XP_019546936.1 PRE	XP_001662576.2ileal sodium/bile acid cotransporter isoform X1
AAEL00330	ras-related protein Rap-1b	0.252431211643726	0.491836536511162	0.852010506207776	NA	NA	KFB40428.1 AGAP01	XP_021704696.1ras-related protein Rap-1b
AAEL01811	NA	0.252626810343146	0.196647073310346	0.630345746481079	NA	NA	NA	XP_001651828.2RNA polymerase II-associated protein 1
AAEL00651	cleft lip and palate transmembrane protein 1 homolog	0.252718196515022	0.0384423572618775	0.309719436954163	NA	NA	KXJ77620.1 hypothet	XP_001652027.2cleft lip and palate transmembrane protein 1 homolog
AAEL00656	serine protease easter	0.252796135303564	0.031002918531214	0.282437393567471	NA	NA	KXJ73949.1 hypothet	XP_001652079.1serine protease easter
AAEL00318	probable prefoldin subunit 5	0.252868091577752	0.0976012544746093	0.473196387605561	NA	NA	KFB43327.1 AGAP00	XP_001656531.1probable prefoldin subunit 5
AAEL01186	transcription factor grauzone	0.252958425787451	0.328016383034814	0.750955871353141	NA	NA	XP_001850003.1 zinc	XP_001662002.2transcription factor grauzone
AAEL00846	gamma-tubulin complex component 3 homolog	0.253058309248279	0.297200087669871	0.729483917508176	NA	NA	KXJ73570.1 hypothet	XP_001659247.1gamma-tubulin complex component 3 homolog
AAEL01089	carbonic anhydrase 2	0.253079106097861	0.247505406383518	0.68058844480365	910	NA	KXJ71698.1 hypothet	XP_001655031.2carbonic anhydrase 2
AAEL02474	NA	0.25342935318384	0.407728477969358	0.803607536382376	NA	NA	NA	XP_021702869.1uncharacterized protein LOC110676980
AAEL01041	uncharacterized LOC5573312	0.253704678530943	0.0565820719268026	0.374365048731287	NA	NA	NA	XP_019561593.1 clarin-3-like
AAEL01388	pre-mRNA-processing factor 39	0.253798478722914	0.105108457176893	0.489439579864721	NA	NA	NA	XP_021695292.1pre-mRNA-processing factor 39 isoform X1
AAEL00044	MMS19 nucleotide excision repair protein homolog	0.253955093168504	0.437025270062606	0.820960220424486	NA	NA	XP_019558282.1 PRE	XP_021710798.1MMS19 nucleotide excision repair protein homolog isoform X1
AAEL00514	CD151 antigen	0.254172261902861	0.296031323085217	0.72758553286062	NA	NA	KFB38243.1 platelet	XP_021708421.1CD151 antigen
AAEL02285	NA	0.254261127249437	0.321531303970937	0.746588710355934	NA	NA	NA	XP_021694870.1inhibitor of growth protein 3 isoform X2
AAEL00156	peptidyl-alpha-hydroxyglycine alpha-amidating lyase 1	0.254372845916495	0.229734045042418	0.662907708523151	NA	NA	KXJ82556.1 hypothet	XP_001653580.1peptidyl-alpha-hydroxyglycine alpha-amidating lyase 1
AAEL01066	RNA-binding protein 45	0.25437405463356	0.060744663401152	0.384941803020695	NA	NA	KFB44585.1 AGAP00	XP_001661004.2RNA-binding protein 45
AAEL00577	E3 ubiquitin-protein ligase HECW2	0.254620821741469	0.304681442839548	0.737849598013971	NA	NA	XP_019551909.1 PRE	XP_021702564.1E3 ubiquitin-protein ligase HECW2 isoform X1
AAEL00548	macrophage mannose receptor 1	0.254643430015912	0.148443662420464	0.562679383080301	NA	NA	KXJ73227.1 hypothet	XP_001650951.1macrophage mannose receptor 1
AAEL02810	NA	0.254645582929069	0.209615312448332	0.640185896166462	NA	aag04142	NA	ABF18066.1possible mucin
AAEL01371	trypsin 5G1-like	0.254908969155307	0.576728193466951	0.881961491587822	NA	NA	NA	XP_001663898.2trypsin 5G1-like
AAEL00786	uncharacterized LOC5569698	0.254963231059561	0.208365718763583	0.639832374753494	NA	NA	KXJ75539.1 hypothet	XP_021703852.1uncharacterized protein LOC5569698
AAEL00160	acidic leucine-rich nuclear phosphoprotein 32 family member A	0.255050369191768	0.023622290118149	0.249853234100238	NA	NA	KXJ83730.1 hypothet	XP_001659738.1acidic leucine-rich nuclear phosphoprotein 32 family member A isoform X2
AAEL00564	rap guanine nucleotide exchange factor 2	0.255110336971585	0.219150962555581	0.652412844728166	NA	NA	XP_019530262.1 PRE	XP_021702556.1rap guanine nucleotide exchange factor 2 isoform X1
AAEL01306	U6 snRNA-associated Sm-like protein LSm7	0.255165147838789	0.291306973763399	0.722646576558698	NA	aag03040 ; aag	NA	XP_021701716.1U6 snRNA-associated Sm-like protein LSm7
AAEL00160	PXMP2/4 family protein 4	0.255179610517941	0.144567771455436	0.557492030137627	NA	aag04146	XP_001844751.1 con	XP_001659752.1PXMP2/4 family protein 4
AAEL00043	THO complex subunit 7 homolog	0.255368153913149	0.170532383043431	0.591273168336719	NA	aag03013	XP_001844369.1 THO	XP_001656429.1THO complex subunit 7 homolog
AAEL00008	nuclear valosin-containing protein-like	0.255453713179234	0.0423130546604966	0.323939778330243	NA	aag03008	XP_019537518.1 PRE	XP_021699088.1nuclear valosin-containing protein-like
AAEL00121	WD repeat-containing protein 82	0.255611095712788	0.139050209096645	0.549029662748535	NA	aag03015	ETN61282.1 WD repe	XP_019547825.1 WD repeat-containing protein 82
AAEL00324	secretion-regulating guanine nucleotide exchange factor	0.255652224430638	0.380082234808732	0.792352501599724	NA	NA	KXJ72406.1 hypothet	XP_001656618.2secretion-regulating guanine nucleotide exchange factor
AAEL00019	N-lysine methyltransferase KMT5A-B	0.255758317703219	0.070181150838946	0.407517366963152	310	aag01100 ; aag	XP_019528147.1 PRE	XP_001658906.1N-lysine methyltransferase KMT5A-B
AAEL01394	L-aminoadipate-semialdehyde dehydrogenase-phosphopantetheinyl trar	0.255764337495315	0.278511896622737	0.713122992430066	770	aag00770	NA	XP_001664136.2L-aminoadipate-semialdehyde dehydrogenase-phosphopantetheinyl transferase
AAEL00306	zinc finger CCCH domain-containing protein 3	0.255958299677907	0.331699836075788	0.754128446928769	NA	NA	KXJ83177.1 hypothet	XP_001663088.2zinc finger CCCH domain-containing protein 3
AAEL01324	cyclin-H	0.256001514777201	0.245978188483095	0.679727114240672	NA	aag03420 ; aag	NA	XP_019564538.1 cyclin-H
AAEL01094	vacuolar protein sorting-associated protein 37B	0.256092976821941	0.196923751346682	0.630345746481079	NA	aag04144	KXJ80772.1 hypothet	XP_001661208.1vacuolar protein sorting-associated protein 37B
AAEL02210	NA	0.256094037227038	0.0983051727100002	0.474608708959823	NA	NA	NA	XP_021693762.1ubiquitin carboxyl-terminal hydrolase 34
AAEL01996	NA	0.256121893046897	0.484712993488574	0.847655581875201	NA	NA	NA	XP_021699310.1transcription factor grauzone-like
AAEL01756	synaptobrevin homolog YKT6	0.256291158386423	0.157321773312628	0.574474189691589	NA	aag04130	NA	XP_011493172.2synaptobrevin homolog YKT6
AAEL00095	uncharacterized LOC5567497	0.256342068808155	0.18289654670865	0.610281543227372	NA	NA	NA	XP_021699634.1uncharacterized protein LOC5567497 isoform X1
AAEL00191	uncharacterized LOC5572795	0.256404054637748	0.232581075474666	0.666707644289445	NA	NA	ACF34410.1 venus ki	DAA06509.1TPA_inf: venus kinase receptor
AAEL01833	NA	0.256435417990188	0.0900313917241252	0.458542183179048	NA	aag04120	NA	XP_019534771.1 RING-box protein 2
AAEL00465	transmembrane protein 50A	0.256436680409326	0.0440168967037782	0.332002855301776	NA	NA	ETN60439.1 hypothe	XP_001649547.1transmembrane protein 50A
AAEL00410	uncharacterized LOC5564122	0.25649729461491	0.470558765643767	0.841282642598396	NA	NA	NA	XP_001648459.2uncharacterized protein LOC5564122
AAEL00040	dynein assembly factor 5, axonemal	0.256592346435533	0.504217307204444	0.858744381072989	NA	NA	ETN62685.1 hypothe	XP_001656072.1dynein assembly factor 5, axonemal
AAEL02617	NA	0.256637513641987	0.239540320100399	0.67449511186165	NA	aag04013 ; aag	NA	XP_021707903.1mitogen-activated protein kinase kinase kinase 7-interacting protein 3 homolog isoform X1
AAEL00375	zinc finger protein 593 homolog	0.256666603987798	0.0889153690640805	0.455343393357453	NA	NA	ETN63474.1 zinc fing	XP_001657231.1zinc finger protein 593 homolog
AAEL01076	lysosome-associated membrane glycoprotein 1	0.256965219657102	0.0296086236762882	0.278018933744085	NA	aag04140 ; aag	KXJ72577.1 hypothet	XP_001654894.1lysosome-associated membrane glycoprotein 1
AAEL00062	tetratricopeptide repeat protein 14 homolog	0.257028592190807	0.0319511350246296	0.284442054201394	NA	NA	KXJ68883.1 hypothet	XP_021700940.1tetratricopeptide repeat protein 14 homolog
AAEL00388	programmed cell death protein 2-like	0.257161996922384	0.178481258579589	0.604039919620956	NA	NA	ETN63405.1 toys are	XP_001664263.1programmed cell death protein 2-like
AAEL00928	39S ribosomal protein L48, mitochondrial	0.25724750132293	0.131257984275032	0.538981683630591	NA	NA	NA	XP_001659904.139S ribosomal protein L48, mitochondrial
AAEL00150	protein HID1	0.257281635704142	0.087839705880362	0.452829677037026	NA	NA	XP_001859432.1 con	XP_001659543.2protein HID1
AAEL00758	nuclear pore complex protein Nup98-Nup96	0.257871821712129	0.051323148748007	0.359690131564899	NA	aag03013	KXJ76050.1 hypothet	XP_001658483.2nuclear pore complex protein Nup98-Nup96
AAEL01216	elongation factor Tu	0.257888200936341	0.184251866243201	0.611957279573706	NA	NA	XP_001843948.1 elo	XP_001843948.1elongation factor Tu, mitochondrial
AAEL00691	ubiquitin conjugation factor E4 B	0.258660477151805	0.0753118162438621	0.420011717015769	NA	aag04141 ; aag	KXJ68611.1 hypothet	XP_021710070.1ubiquitin conjugation factor E4 B
AAEL02107	NA	0.25870541353735	0.503976670701035	0.858744381072989	NA	NA	NA	NA
AAEL00358	amidophosphoribosyltransferase	0.258763076883483	0.0654642891524226	0.397837025777134	00230 ; 00250	aag01100 ; aag	KXJ72752.1 hypothet	XP_019552328.1 amidophosphoribosyltransferase-like
AAEL00963	UPF0415 protein C7orf25 homolog	0.258824554656401	0.317894669068317	0.745684464362801	NA	NA	NA	XP_001653894.1UPF0415 protein C7orf25 homolog
AAEL01314	polynucleotide 5'-hydroxyl-kinase NOL9	0.258886038426278	0.120396946201505	0.521361270092351	NA	NA	NA	XP_021699171.1polynucleotide 5'-hydroxyl-kinase NOL9
AAEL02508	NA	0.258910934817419	0.290287387927375	0.721952579380462	NA	NA	NA	XP_001655662.1peptidyl-prolyl cis-trans isomerase
AAEL02723	NA	0.259213534117393	0.39262609650873	0.793563770106823	NA	NA	NA	XP_021704051.1zinc finger protein 239
AAEL01299	splicing factor, arginine/serine-rich 15	0.259271838020316	0.232726440380729	0.666707644289445	NA	NA	NA	XP_001663186.1splicing factor, arginine/serine-rich 15
AAEL02722	NA	0.259393302618797	0.333391547545645	0.755632763673653	NA	NA	NA	NA
AAEL02631	NA	0.260164811273713	0.494233972953664	0.853272236232574	NA	NA	NA	XP_021702005.1ankyrin repeat domain-containing protein 39
AAEL00317	translation initiation factor IF-2, mitochondrial	0.260309001814858	0.341429183313578	0.762180211699347	NA	NA	KXJ80094.1 hypothet	XP_001656471.2translation initiation factor IF-2, mitochondrial
AAEL00939	exportin-5	0.260323204794215	0.0648802975490525	0.39730198438886	NA	aag03013	NA	XP_019539785.1 exportin-5-like
AAEL00232	uncharacterized LOC5574346	0.260523479501143	0.119325034991511	0.518470808496326	NA	NA	KXJ76374.1 hypothet	XP_019527516.1 nucleolar protein 11-like
AAEL00501	transient receptor potential-gamma protein	0.26056859366137	0.215098918317388	0.648208527708858	NA	aag04745	XP_019561236.1 PRE	XP_021703629.1transient receptor potential-gamma protein isoform X1
AAEL00174	protein rhomboid	0.260656836708811	0.370680584210595	0.781817769662763	NA	NA	XP_001845651.1 ste	XP_021698730.1protein rhomboid isoform X1
AAEL00208	ralA-binding protein 1	0.260722458881138	0.0514401448749627	0.359690131564899	NA	NA	KXJ69329.1 hypothet	XP_001654712.2ralA-binding protein 1
AAEL00175	anaphase-promoting complex subunit 2	0.260884577136861	0.285646410597769	0.717970179170389	NA	aag04120	KXJ71845.1 hypothet	XP_001653953.1anaphase-promoting complex subunit 2
AAEL00552	exonuclease mut-7 homolog	0.261125342484012	0.0945752721187522	0.465640264332134	NA	NA	KXJ74525.1 hypothet	XP_001651037.2exonuclease mut-7 homolog
AAEL00917	molybdenum cofactor synthesis protein cinnamon	0.261151932834061	0.172456415771993	0.595029598025453	790	aag01100 ; aag	XP_019526842.1 PRE	XP_021704341.1molybdenum cofactor synthesis protein cinnamon isoform X1
AAEL00051	uncharacterized LOC5578484	0.261327611579845	0.0659192325274034	0.398547160129544	901	NA	KXJ70818.1 hypothet	XP_001656950.2uncharacterized protein LOC5578484
AAEL01713	3-oxoacyl-[acyl-carrier-protein] reductase FabG	0.261385976627786	0.134593078081192	0.542662316284181	NA	NA	NA	XP_001863196.13-oxoacyl-[acyl-carrier-protein] reductase
AAEL00316	protein cappuccino	0.261821933591388	0.26433415297532	0.698659663885272	NA	aag04320	XP_019549916.1 PRE	XP_021700510.1protein cappuccino isoform X3
AAEL00415	zinc finger protein 784	0.261901049333799	0.271690355676498	0.70679261462421	NA	NA	KXJ69848.1 hypothet	XP_001648560.2zinc finger protein 784
AAEL00776	myeloid differentiation primary response protein MyD88	0.26202147201618	0.159789596088078	0.577560236664366	NA	aag04624	KXJ84016.1 hypothet	XP_001658635.1myeloid differentiation primary response protein MyD88
AAEL02661	NA	0.262307469655318	0.0586745409830698	0.379692775053592	910	NA	NA	XP_021707077.1beta carbonic anhydrase 1
AAEL00582	uncharacterized LOC5567112	0.262409616657406	0.36987132255305	0.781817769662763	NA	NA	NA	XP_001651488.2uncharacterized protein LOC5567112

AAEL00553	beta-1,4-mannosyl-glycoprotein 4-beta-N-acetylglucosaminyltransferase	0.262467528117958	0.134487411169713	0.542662316284181	510	aag01100 ; aag	KXJ69009.1 hypothe	XP_021701730.1beta-1,4-mannosyl-glycoprotein 4-beta-N-acetylglucosaminyltransferase
AAEL00011	NA	0.262504902751365	0.123558522521692	0.526870029770442	NA	aag03008	XP_019528074.1 PRE	XP_001657684.2ribosomal RNA-processing protein 7 homolog A
AAEL00493	beta-hexosaminidase subunit beta	0.262551462188022	0.0635747499343393	0.393419535235905	00513 ; 00603 ; 00604 ;	aag01100 ; aag	XP_001867057.1 bet	XP_001650049.1beta-hexosaminidase subunit beta isoform X1
AAEL01434	serine protease easter	0.262615835059967	0.0633015659562168	0.392785576486249	NA	NA	NA	XP_001648541.3serine protease easter
AAEL02020	NA	0.263312670894573	0.310667686867595	0.742918550320373	NA	NA	NA	XP_021708616.1putative helicase MOV-10 isoform X1
AAEL02491	NA	0.263507412695032	0.178884768488669	0.604350349136232	NA	NA	NA	XP_021699168.1organic cation transporter protein
AAEL00472	uncharacterized LOC5565316	0.263599321352631	0.0676024522368269	0.40094231299198	NA	NA	ETN60516.1 hypothe	XP_001649661.1uncharacterized protein LOC5565316
AAEL00581	LMBR1 domain-containing protein 2 homolog	0.263604653019725	0.31169003462896	0.743434862302544	NA	NA	XP_019539148.1 PRE	XP_021712781.1LMBR1 domain-containing protein 2 homolog
AAEL00597	carbohydrate sulfotransferase 11	0.264078000816848	0.0947050275810875	0.465843434343757	NA	NA	XP_019533236.1 PRE	XP_021695556.1carbohydrate sulfotransferase 11 isoform X2
AAEL00458	histone deacetylase Rpd3	0.264229875300322	0.0258114822298674	0.262052657185525	NA	aag04213 ; aag	KXJ82549.1 hypothe	XP_021696170.1histone deacetylase Rpd3
AAEL00962	uncharacterized LOC5572220	0.26423676096308	0.0561223330242516	0.372610179285111	NA	NA	NA	XP_001660288.1uncharacterized protein LOC5572220
AAEL02311	NA	0.264579867563192	0.552899266560433	0.874531710113044	NA	NA	NA	XP_021703371.1uncharacterized protein LOC110677076
AAEL00861	traB domain-containing protein	0.265127795361017	0.136729480782188	0.545589928345625	NA	NA	XP_001847327.1 Tra	XP_001653346.2traB domain-containing protein
AAEL00873	chorion transcription factor Cf2	0.265201292493898	0.154822371192929	0.5722398898892	NA	NA	XP_001867652.1 con	XP_001653424.1chorion transcription factor Cf2
AAEL01081	uncharacterized LOC5573969	0.265269682704389	0.456294327757653	0.832617462606238	NA	NA	XP_001863469.1 con	XP_019539549.1 neurofilament heavy polypeptide-like
AAEL00373	28S ribosomal protein S28, mitochondrial	0.26546767280709	0.158963986348203	0.577491749852415	NA	NA	KXJ78921.1 hypothe	XP_001664061.128S ribosomal protein S28, mitochondrial
AAEL00290	uncharacterized LOC5576479	0.265802376910885	0.465402139653062	0.838483018891546	NA	NA	XP_001848261.1 con	XP_001662671.2uncharacterized protein LOC5576479
AAEL00988	aurora kinase C	0.265810834325024	0.450849212701116	0.830259097859495	04151 ; 05165 ; 04714 ;	NA	NA	XP_001654066.1aurora kinase C
AAEL02263	NA	0.266014313895874	0.152863347344297	0.568113296551786	NA	NA	NA	ABF18174.1putative 14.5 kDa salivary protein
AAEL00399	reticulon-4-interacting protein 1 homolog, mitochondrial	0.266182613847383	0.0578271951476034	0.376859666960514	NA	NA	NA	XP_021704774.1reticulon-4-interacting protein 1 homolog, mitochondrial isoform X4
AAEL01993	NA	0.266223301300982	0.0702269473275303	0.407517366963152	NA	NA	NA	XP_021709493.1protein groucho isoform X2
AAEL01339	dephospho-CoA kinase domain-containing protein	0.266517210649471	0.0571614109334601	0.375057077651547	770	NA	NA	XP_001663575.1dephospho-CoA kinase domain-containing protein
AAEL00143	uncharacterized LOC5570539	0.266646832096045	0.455402084726549	0.832617462606238	NA	NA	XP_320790.4 AGAP0	XP_001659178.2uncharacterized protein LOC5570539
AAEL00117	signal recognition particle 14 kDa protein	0.266734459965684	0.0847316289361314	0.441820669235316	NA	aag03060	ETN63273.1 Srp14 [A	XP_001658168.2signal recognition particle 14 kDa protein
AAEL02352	NA	0.266816221260867	0.117158792343117	0.514858748441671	NA	NA	NA	XP_021706702.1angio-associated migratory cell protein
AAEL01218	lethal(3)malignant brain tumor-like protein 3	0.2668394298155	0.230220687177313	0.663064401691905	NA	NA	NA	XP_021699159.1lethal(3)malignant brain tumor-like protein 3 isoform X2
AAEL02452	NA	0.266934976715034	0.246648650962848	0.680353982661909	NA	NA	NA	XP_021708467.1uncharacterized protein LOC110678987
AAEL00695	adhesion G protein-coupled receptor A3	0.267040981925307	0.310739288973757	0.742918550320373	NA	NA	KXJ69468.1 hypothe	XP_021707573.1adhesion G protein-coupled receptor A3
AAEL00575	cGMP-dependent protein kinase, isozyme 1	0.267120264868914	0.298863873161673	0.730450295042154	NA	NA	KXJ74903.1 hypothe	XP_021703513.1cGMP-dependent protein kinase, isozyme 1
AAEL00288	probable 28S rRNA (cytosine-C(5))-methyltransferase	0.267324785114192	0.0816293900772847	0.435199100516323	NA	NA	ETN64028.1 williams	XP_021700376.1probable 28S rRNA (cytosine-C(5))-methyltransferase
AAEL02075	NA	0.267345585871757	0.125980136094629	0.530481545423179	NA	NA	NA	XP_021698314.1uncharacterized Golgi apparatus membrane protein-like protein CG5021
AAEL01096	ubiquitin carboxyl-terminal hydrolase isozyme L5	0.267356984677988	0.06693013810193	0.399699227060601	NA	NA	KFB50740.1 hypothe	XP_001655068.1ubiquitin carboxyl-terminal hydrolase isozyme L5
AAEL00293	nuclear nucleic acid-binding protein C1D	0.267578968637444	0.0552441845882014	0.369714172058639	NA	aag03018	KFB49035.1 hypothe	XP_001656164.1nuclear nucleic acid-binding protein C1D
AAEL00997	uncharacterized LOC5572703	0.267753057037026	0.530518578900242	0.866691320214768	NA	NA	NA	XP_001660526.1uncharacterized protein LOC5572703
AAEL00334	uncharacterized LOC5577898	0.267785071582878	0.457782284526645	0.833283524364043	NA	NA	KXJ75668.1 hypothe	XP_001656694.1uncharacterized protein LOC5577898
AAEL02736	NA	0.267839281105686	0.036151164776095	0.300966157937731	NA	NA	NA	XP_021703784.1ATP-binding cassette sub-family G member 1
AAEL02044	NA	0.267952404787902	0.380952584154045	0.787051117958132	NA	NA	NA	XP_021703423.1uncharacterized protein LOC110677081 isoform X1
AAEL00158	otefin	0.268287459301906	0.322936338975465	0.74702367804229	NA	NA	KXJ82553.1 hypothe	XP_019932391.1 otefin-like
AAEL00143	protein disulfide-isomerase A3	0.268408838794564	0.0348772034496427	0.295524885951761	NA	aag04141	KXJ81333.1 hypothe	XP_001659196.2protein disulfide-isomerase A3
AAEL01956	NA	0.268759238636765	0.0471715304696337	0.346180765881814	NA	NA	NA	XP_021704294.1cysteine-rich motor neuron 1 protein isoform X2
AAEL01977	NA	0.268846784324889	0.182309366383574	0.608933135920744	NA	aag04140 ; aag	NA	XP_021709272.1autophagy-related protein 16-1 isoform X1
AAEL02602	NA	0.268953554425437	0.137174361123299	0.546204444420855	NA	NA	NA	XP_021696640.1N-terminal kinase-like protein isoform X1
AAEL00596	uncharacterized LOC5567304	0.268998354616713	0.28628034576069	0.718756504421269	NA	NA	KXJ72195.1 hypothe	XP_001651739.1uncharacterized protein LOC5567304
AAEL02519	NA	0.269081503000223	0.0360303911672775	0.300639062767631	00670 ; 00720 ; 00670 ;	aag01100 ; aag	NA	XP_021710488.1C-1-tetrahydrofolate synthase, cytoplasmic isoform X2
AAEL02791	NA	0.269128597600033	0.165785279047697	0.584439853413553	NA	NA	NA	XP_021697182.1NECAP-like protein CG9132
AAEL01097	titin	0.269230564083761	0.107063729561905	0.492398491132955	NA	NA	XP_001845762.1 con	XP_019546031.1 titin-like
AAEL01199	hypertrehalosaemic prohormone	0.269406574125983	0.392289210278431	0.793341218606811	NA	NA	KFB42998.1 AGAP00	XP_001655817.1hypertrehalosaemic prohormone
AAEL00815	arrestin domain-containing protein 2	0.269625620936339	0.311690034640645	0.743434862302544	NA	NA	KXJ71066.1 hypothe	XP_001658999.1arrestin domain-containing protein 2
AAEL00553	venom allergen 5	0.269762662860042	0.15275645024519	0.568113296551786	NA	NA	KXJ68868.1 hypothe	XP_001651036.1venom allergen 5
AAEL01756	DNA ligase 1	0.269799046025762	0.306273541267746	0.738783696372872	NA	aag03420 ; aag	NA	XP_011493575.2DNA ligase 1 isoform X2
AAEL00168	uncharacterized LOC5571808	0.269811452666803	0.476014363092443	0.844867278451347	NA	NA	KXJ83100.1 hypothe	XP_021697115.1uncharacterized protein LOC5571808
AAEL00457	putative zinc metalloproteinase C607.06c	0.269822470495996	0.482296387583358	0.846941547268585	NA	NA	KXJ69045.1 hypothe	XP_001649385.2putative zinc metalloproteinase C607.06c
AAEL01012	leucine-rich repeat transmembrane neuronal protein 4	0.270012546362766	0.0300590601458968	0.279963027796095	NA	NA	NA	XP_001660654.2leucine-rich repeat transmembrane neuronal protein 4
AAEL00995	protein Lilipod	0.270168131692961	0.251342064106013	0.684238204149262	NA	NA	NA	XP_021697243.1protein Lilipod
AAEL00162	mpv17-like protein	0.270300190968121	0.0861144909157471	0.446940521943996	NA	aag04146	XP_001844750.1 con	XP_001659753.1mpv17-like protein
AAEL01506	small ubiquitin-related modifier 3	0.270390773081461	0.027497421010514	0.269756149401652	NA	aag03013	NA	XP_001650445.1small ubiquitin-related modifier 3
AAEL00640	thioredoxin-like protein 4A	0.270970853318382	0.229330538241024	0.662487607456997	NA	aag03040	XP_001848597.1 mit	XP_001657795.1thioredoxin-like protein 4A
AAEL01431	DNA primase small subunit	0.270985572773349	0.424659767166362	0.814623464645531	NA	aag03030	NA	XP_001648424.1DNA primase small subunit
AAEL00304	uncharacterized LOC5576891	0.271027131777296	0.42858824237821	0.816740033999805	NA	NA	NA	XP_021703357.1uncharacterized protein LOC5576891 isoform X1
AAEL01719	protein pygopus	0.271196116667274	0.159423794464304	0.577560236664366	NA	NA	NA	XP_021709751.1protein pygopus
AAEL02762	NA	0.271274413581163	0.469605584836369	0.84065046403825	NA	NA	NA	XP_021701820.1rab3 GTPase-activating protein catalytic subunit
AAEL01416	U6 snRNA-associated Sm-like protein LSm5	0.271461670889988	0.173312344291005	0.596392068481585	NA	aag03040 ; aag	NA	XP_001648155.1U6 snRNA-associated Sm-like protein LSm5 isoform X1
AAEL01334	protein lethal(2)essential for life	0.271986719142085	0.0307967917638729	0.281422122950505	NA	aag04141 ; aag	NA	XP_001663497.1protein lethal(2)essential for life
AAEL00033	multifunctional methyltransferase subunit TRM112-like protein	0.272003740127101	0.0539522531095335	0.366668118991083	NA	NA	XP_019527411.1 PRE	XP_001655512.2multifunctional methyltransferase subunit TRM112-like protein
AAEL00090	enhancer of yellow 2 transcription factor	0.272049369606902	0.157174665310396	0.574474189691589	NA	NA	XP_001847707.1 enh	XP_001651605.1enhancer of yellow 2 transcription factor
AAEL01121	NF-X1-type zinc finger protein NFXL1	0.2723211359383	0.30469772576104	0.737849598013971	NA	NA	XP_019552802.1 PRE	XP_001655190.2NF-X1-type zinc finger protein NFXL1
AAEL02628	NA	0.272327232285339	0.482011787273562	0.846941547268585	NA	NA	NA	XP_021713155.1endothelin-converting enzyme homolog
AAEL00145	head-specific guanylate cyclase	0.272494973473393	0.219826198889638	0.652412844728166	230	aag01100 ; aag	KXJ79976.1 hypothe	XP_001659294.2head-specific guanylate cyclase
AAEL00545	ATPase family AAA domain-containing protein 1-B	0.27260304714762	0.0222336164398489	0.242316778828459	NA	NA	ETN61589.1 aaa atpa	XP_001650903.1ATPase family AAA domain-containing protein 1-B
AAEL02724	NA	0.272608224058902	0.1335601760717	0.54189626993465	NA	NA	NA	XP_021694577.1zinc finger protein 236 isoform X1
AAEL02351	NA	0.27264494962453	0.205434357853407	0.638023927878659	NA	NA	NA	XP_021700390.1protein SCO1 homolog, mitochondrial
AAEL01302	putative helicase MOV-10	0.273253280906896	0.356224946628478	0.772867683747772	NA	NA	NA	XP_001663217.2putative helicase MOV-10
AAEL02475	NA	0.273581845161033	0.161263950360428	0.5783128882372	NA	NA	NA	XP_019539093.1 nascent polypeptide-associated complex subunit alpha, muscle-specific form-like
AAEL00502	60S acidic ribosomal protein P1	0.273691562437271	0.192819702935698	0.623884284955799	NA	aag03010	XP_001857537.1 acid	XP_001650193.160S acidic ribosomal protein P1
AAEL00402	glucose dehydrogenase [FAD, quinone]	0.273743540033423	0.0591809196963372	0.381093648037575	NA	NA	XP_019531589.1 PRE	XP_021710641.1glucose dehydrogenase [FAD, quinone] isoform X1
AAEL00705	transmembrane protein 14 homolog	0.273750229819483	0.161207461462514	0.5783128882372	NA	NA	XP_001844526.1 con	XP_001652542.1transmembrane protein 14 homolog
AAEL00585	signal recognition particle receptor subunit alpha homolog	0.27378149964188	0.0182686742824823	0.219462585832118	NA	aag03060	KXJ75055.1 hypothe	XP_001651530.1signal recognition particle receptor subunit alpha homolog
AAEL00000	L-dopachrome tautomerase yellow-f2	0.274090939976981	0.0219879156499021	0.242070925206103	NA	aag01100 ; aag	NA	XP_001647841.1L-dopachrome tautomerase yellow-f2
AAEL00679	cytochrome P450 9e2	0.274094424895739	0.26031061095773	0.693219418655219	NA	NA	KXJ79844.1 hypothe	XP_021705958.1cytochrome P450 9e2
AAEL00518	conserved oligomeric Golgi complex subunit 5	0.274174814297772	0.117437186825192	0.515089129249485	NA	NA	KXJ71324.1 hypothe	XP_001650494.1conserved oligomeric Golgi complex subunit 5
AAEL01974	NA	0.274179627279802	0.0513674378962283	0.359690131564899	NA	NA	NA	XP_021698846.1peptidoglycan-recognition protein 3 isoform X2
AAEL00329	nucleolar GTP-binding protein 2	0.274236951082549	0.0267479888991554	0.266963842267995	NA	aag03008	KXJ79211.1 hypothe	XP_001663521.2nucleolar GTP-binding protein 2

AAEL00662	ribosome maturation protein SBDS	0.274473044262716	0.058796384973321	0.380164744858701	NA	aag03008	KFB39753.1 AGAP00	XP_001652129.1ribosome maturation protein SBDS
AAEL01363	guanine nucleotide-binding protein-like 1	0.274499958967964	0.0922051870272387	0.460875682251304	NA	NA	NA	XP_001656892.1guanine nucleotide-binding protein-like 1
AAEL01952	NA	0.2747539652478	0.0420609474449491	0.323800309636003	NA	NA	NA	ABF18491.1putative secreted protein
AAEL01144	translation initiation factor eIF-2B subunit gamma	0.274960453389644	0.131860262292282	0.538981683630591	NA	aag03013	KXJ76516.1 hypothet	XP_001661660.2translation initiation factor eIF-2B subunit gamma
AAEL01024	protein FAM98B	0.275566749430119	0.147353747173412	0.561952643558699	NA	NA	NA	XP_001654395.2protein FAM98B
AAEL01127	apoptosis-stimulating of p53 protein 2	0.275873281328647	0.0609957236855592	0.385761271034031	NA	NA	KXJ82130.1 hypothet	XP_021699879.1apoptosis-stimulating of p53 protein 2 isoform X2
AAEL00683	uncharacterized LOC5568420	0.275896016356541	0.355768759684999	0.772867683747772	NA	NA	KXJ73898.1 hypothet	XP_021708111.1uncharacterized protein LOC5568420
AAEL00475	general vesicular transport factor p115	0.276053677917118	0.147964487536921	0.562679383080301	NA	NA	KXJ81342.1 hypothet	XP_001649736.1general vesicular transport factor p115
AAEL00539	glycosyltransferase-like domain-containing protein 1-like	0.276365486649705	0.19846754153561	0.631728026149226	NA	NA	KXJ70040.1 hypothet	XP_001650816.1glycosyltransferase-like domain-containing protein 1-like
AAEL01131	uncharacterized LOC5574677	0.27640266746423	0.0218541096856645	0.241418388148241	NA	NA	NA	XP_021701648.1uncharacterized protein LOC5574677 isoform X2
AAEL00439	chorion peroxidase-like	0.276653367901094	0.0636853838570718	0.39383931603009	940	NA	XP_019526999.1 PRE	XP_001649029.3chorion peroxidase-like
AAEL00237	small nuclear ribonucleoprotein-associated protein B	0.276746253160838	0.0680560607268797	0.401232883533236	NA	aag03040	ETN66295.1 small nu	XP_001655156.1small nuclear ribonucleoprotein-associated protein B
AAEL00498	ubiquitin carboxyl-terminal hydrolase 3	0.276749927857233	0.0522889526433411	0.362627777546831	NA	NA	KXJ68147.1 hypothet	XP_001650143.1ubiquitin carboxyl-terminal hydrolase 3
AAEL01381	uncharacterized LOC5578708	0.276939240523297	0.220041519337205	0.652412844728166	NA	NA	NA	XP_001664007.2uncharacterized protein LOC5578708
AAEL00277	tubulin polyglutamylase TTLL6	0.277043951510004	0.332639859237006	0.755231182999982	NA	NA	KXJ74316.1 hypothet	XP_021694090.1tubulin polyglutamylase TTLL6
AAEL00787	monoacylglycerol lipase ABHD12	0.277062682801232	0.0267486049305685	0.266963842267995	561	NA	XP_019539708.1 PRE	XP_001652963.1monoacylglycerol lipase ABHD12 isoform X2
AAEL02329	NA	0.277608713133138	0.0320734688212683	0.284442054201394	NA	NA	NA	XP_021712775.1uncharacterized protein LOC110681328
AAEL01470	DDB1- and CUL4-associated factor 13	0.277721358050293	0.0653351480561994	0.397628328315573	NA	NA	NA	XP_001649367.2DDB1- and CUL4-associated factor 13
AAEL00073	E3 ubiquitin-protein ligase Bre1	0.277759221889917	0.08236284610995	0.437083569725352	NA	NA	XP_019536462.1 PRE	XP_021696559.1E3 ubiquitin-protein ligase Bre1 isoform X1
AAEL00323	probable palmitoyltransferase ZDHHC16	0.278116305921669	0.293170310090351	0.723646242878596	NA	NA	KXJ71586.1 hypothet	XP_001656662.2probable palmitoyltransferase ZDHHC16
AAEL01388	synaptic vesicle glycoprotein 2C	0.278300028530346	0.0528462313375873	0.362633125106993	NA	NA	NA	XP_001664089.2synaptic vesicle glycoprotein 2C isoform X2
AAEL01310	exosome complex component RRP40	0.27863084783165	0.201000724597892	0.634514122727206	NA	aag03018	NA	XP_001663303.2exosome complex component RRP40
AAEL01093	probable isoaspartyl peptidase/L-asparaginase GA20639	0.278743451688555	0.258019872811737	0.691310673420561	00250 ; 00460	aag01100 ; aag	KXJ82767.1 hypothet	XP_001655052.2probable isoaspartyl peptidase/L-asparaginase GA20639
AAEL00742	serpin B5	0.278843195197471	0.117400481935498	0.515089129249485	NA	NA	AAV90669.1 FXa-dire	XP_001658365.3serpin B5
AAEL02658	NA	0.278935963773624	0.0574918391202885	0.375471897505248	NA	NA	NA	XP_021693512.1probable cytochrome P450 6a14
AAEL00392	pollen-specific leucine-rich repeat extensin-like protein 1	0.279143793217899	0.0221268085902435	0.242070925206103	NA	NA	KXJ70079.1 hypothet	XP_001648072.2brain acid soluble protein 1 isoform X1
AAEL02038	NA	0.279284539119809	0.374437231902637	0.782198815065306	NA	NA	NA	NA
AAEL02339	NA	0.279284539119809	0.374437231902637	0.782198815065306	NA	NA	NA	NA
AAEL02734	NA	0.279311502408277	0.542113710808572	0.869900911343623	NA	NA	NA	XP_021701214.1TSC22 domain family protein 1
AAEL02451	NA	0.279651860437683	0.400886503331147	0.798648539435638	NA	NA	NA	XP_021695116.1uncharacterized protein LOC5577442
AAEL00307	pseudouridylate synthase 7 homolog	0.27969146353966	0.0157318027244642	0.202259474938964	NA	NA	KXJ73088.1 hypothet	XP_001663070.2pseudouridylate synthase 7 homolog
AAEL00502	mediator of RNA polymerase II transcription subunit 15	0.279746738983524	0.147814784156199	0.562527561540671	NA	NA	KXJ72831.1 hypothet	XP_021703618.1mediator of RNA polymerase II transcription subunit 15
AAEL01198	minor histocompatibility antigen H13	0.279889273232697	0.0204361317338689	0.232451525605763	NA	NA	XP_001842495.1 sigr	XP_001655809.1minor histocompatibility antigen H13 isoform X1
AAEL02501	NA	0.279980555051384	0.363922873092092	0.778432886609352	NA	aag03022	NA	XP_021710567.1transcription initiation factor TFIID subunit 1 isoform X2
AAEL00335	uncharacterized LOC5577900	0.28003652045839	0.355646290440389	0.772867683747772	NA	NA	KXJ82190.1 hypothet	XP_023951993.1transcription factor Adf-1-like
AAEL02047	NA	0.280145329144856	0.0347164869949257	0.295097377978161	NA	NA	NA	XP_021694161.1transmembrane protein 192 isoform X2
AAEL02003	NA	0.280211201294198	0.216440785397806	0.64939292703965	NA	aag04013	NA	XP_021695007.1zinc finger protein 236 isoform X3
AAEL01307	dolichyl-diphosphooligosaccharide--protein glycosyltransferase subunit 1	0.280226272415005	0.015249592647324	0.198907702459398	NA	aag01100 ; aag	NA	XP_001663283.1dolichyl-diphosphooligosaccharide--protein glycosyltransferase subunit 1
AAEL00485	DAZ-associated protein 2	0.280308947905245	0.321432214761127	0.746588710355934	NA	NA	KXJ82636.1 hypothet	XP_001649910.1DAZ-associated protein 2
AAEL00782	COP9 signalosome complex subunit 8	0.280412527091059	0.110635557207611	0.501758697597061	NA	NA	ETN61942.1 COP9 sig	XP_001652939.1COP9 signalosome complex subunit 8
AAEL01198	NA	0.280909906770663	0.103684464894717	0.486291766544946	NA	NA	XP_001851524.1 con	XP_001655793.2uncharacterized protein LOC5575647
AAEL02715	NA	0.281017885690532	0.374966533061817	0.782198815065306	NA	NA	NA	EAT43735.1AAEL004831
AAEL01156	caspase Dronc	0.281160595895161	0.138862095730624	0.549029662748535	NA	aag04214 ; aag	KXJ81718.1 hypothet	XP_001655433.2caspase Dronc
AAEL01384	paraplegin	0.281420643247831	0.107352722180444	0.492585616254939	NA	NA	NA	XP_019565659.1 paraplegin-like
AAEL00482	sodium/potassium/calcium exchanger 5	0.281627104231599	0.132918683024905	0.540278981726584	NA	NA	XP_019544176.1 PRE	XP_001649854.2sodium/potassium/calcium exchanger 5 isoform X2
AAEL00334	heparin sulfate O-sulfotransferase	0.281644611804823	0.259198862363003	0.691547675112889	NA	aag00534	KXJ72861.1 hypothet	XP_001656685.2heparin sulfate O-sulfotransferase
AAEL01351	endocuticle structural glycoprotein SgAbd-5	0.281656985646115	0.0599175666228357	0.383636203860763	NA	NA	NA	XP_001663700.1endocuticle structural glycoprotein SgAbd-5
AAEL01191	patatin-like phospholipase domain-containing protein 3	0.281725930438527	0.0230526894420173	0.246091471282416	NA	NA	XP_019527707.1 PRE	XP_021708557.1patatin-like phospholipase domain-containing protein 3 isoform X3
AAEL00181	protein grindelwald	0.281728370889434	0.387168075771612	0.791825725928371	NA	NA	ETN60506.1 hypothe	XP_001660384.1protein grindelwald
AAEL00631	ketohexokinase	0.281742240573174	0.0863464509801825	0.447639460236416	51	NA	KXJ83575.1 hypothet	XP_021705342.1ketohexokinase isoform X1
AAEL02346	NA	0.281802099068493	0.549283847890012	0.873274009724238	NA	NA	NA	XP_001654172.1protein FAM50 homolog
AAEL02154	NA	0.281902308921616	0.0932049447752875	0.463658666957633	NA	aag03013	NA	XP_021713334.1nuclear pore complex protein Nup205
AAEL00403	Bloom syndrome protein homolog	0.282041638681202	0.160583784320774	0.577674739374419	NA	aag03460 ; aag	KXJ72127.1 hypothet	XP_001648286.1Bloom syndrome protein homolog
AAEL01811	NA	0.282570268728193	0.255309102877006	0.687548833676972	NA	NA	NA	XP_021710638.1transcription termination factor 2 isoform X1
AAEL00597	scavenger receptor class B member 1	0.283091771090144	0.10244574922234	0.484636597025644	NA	NA	XP_019533241.1 PRE	XP_001664322.3scavenger receptor class B member 1
AAEL00170	NTF2-related export protein	0.283141011129139	0.126584624768949	0.530481545423179	NA	aag03013 ; aag	KFB45189.1 p15-2a p	XP_001653822.1NTF2-related export protein
AAEL00875	DNA-directed RNA polymerase II subunit RPB3	0.283154166266917	0.105159857401293	0.489439579864721	NA	aag03020	ETN64477.1 DNA-dir	XP_001659470.1DNA-directed RNA polymerase II subunit RPB3
AAEL00934	biotin--protein ligase 2	0.283163608366929	0.155781388724436	0.573702478134775	00785 ; 00780	aag01100 ; aag	NA	XP_021702937.1biotin--protein ligase 2 isoform X1
AAEL02065	NA	0.283197375068398	0.0444491895742758	0.333895055071417	790	aag01100 ; aag	NA	XP_021704955.1folylpolyglutamate synthase, mitochondrial
AAEL00802	sorting nexin-25	0.283379277431057	0.111479957127767	0.502879021478986	NA	NA	KXJ81886.1 hypothet	XP_001658823.2sorting nexin-25
AAEL00873	anillin	0.283977730301706	0.255826806401325	0.68833867617105	NA	NA	KXJ80001.1 hypothet	XP_001653425.2anillin isoform X1
AAEL00286	mitochondrial pyruvate carrier 1	0.284021495375993	0.0194419696779083	0.22702918113002	NA	NA	ETN67943.1 hypothe	XP_001656098.1mitochondrial pyruvate carrier 1
AAEL00286	saccharopine dehydrogenase-like oxidoreductase	0.284028538160034	0.0209930206482113	0.23627229311125	NA	NA	KXJ72502.1 hypothet	XP_001656086.1saccharopine dehydrogenase-like oxidoreductase
AAEL00051	uncharacterized LOC5578431	0.284049808152731	0.0241930022844468	0.254427436595976	901	NA	KXJ70815.1 hypothet	XP_021704726.1uncharacterized protein LOC5578431 isoform X1
AAEL01375	zinc transporter ZIP1	0.284117846640712	0.060975178518868	0.385761271034031	NA	NA	NA	XP_001663959.1zinc transporter ZIP1 isoform X2
AAEL01349	zinc transporter ZIP11	0.284140850368213	0.0325503377476767	0.28463170766102	NA	NA	NA	XP_001663675.1zinc transporter ZIP11
AAEL00176	RNA exonuclease 4	0.28424608396787	0.198645566553054	0.63193663887035	NA	NA	KXJ71847.1 hypothet	XP_001653951.1RNA exonuclease 4
AAEL00744	cAMP-responsive element-binding protein-like 2	0.284265967775896	0.0213856086923467	0.238631723223164	NA	NA	ETN63654.1 cAMP-re	XP_001652760.1cAMP-responsive element-binding protein-like 2 isoform X1
AAEL00861	protein hairless	0.284308739333453	0.183013196805935	0.610281543227372	NA	aag04330	XP_019525055.1 PRE	XP_021693038.1 protein hairless
AAEL00859	probable GDP-L-fucose synthase	0.284424054758357	0.121360039449495	0.523315409097589	00520 ; 00051	aag01100 ; aag	KXJ68536.1 hypothet	XP_001653344.1probable GDP-L-fucose synthase
AAEL00228	uncharacterized LOC5574198	0.284426186872496	0.447283682667353	0.828502725039167	NA	NA	KXJ81504.1 hypothet	XP_001661255.1uncharacterized protein LOC5574198
AAEL01317	iron-sulfur protein NUBPL	0.284482356109873	0.211078005852118	0.643371914491948	NA	NA	NA	XP_001663356.2iron-sulfur protein NUBPL isoform X1
AAEL00837	mitogen-activated protein kinase 14B	0.284688632359336	0.01762833384282	0.214955509175633	4150	aag04013 ; aag	KFB51279.1 P38 map	XP_001653240.1mitogen-activated protein kinase 14B isoform X1
AAEL00075	terminal uridylyltransferase cid1	0.284714996997122	0.125834369729901	0.530481545423179	NA	NA	KXJ81447.1 hypothet	XP_001650674.2terminal uridylyltransferase cid1
AAEL00725	zinc finger protein 626	0.284910117429598	0.244915881567514	0.679727114240672	NA	NA	KXJ76947.1 hypothet	XP_001652626.2zinc finger protein 626
AAEL00532	SAC3 domain-containing protein 1	0.284965381094035	0.421776830025714	0.814192545906728	NA	NA	KXJ74575.1 hypothet	XP_001650739.2SAC3 domain-containing protein 1
AAEL00666	ethanolaminephosphotransferase 1	0.284984860848421	0.0195684057726146	0.227335564593981	NA	aag01100 ; aag	XP_019535787.1 PRE	XP_001657923.1ethanolaminephosphotransferase 1 isoform X2
AAEL01301	general odorant-binding protein 83a	0.285078650849155	0.305504334999565	0.738400482188558	NA	NA	NA	XP_001663216.2general odorant-binding protein 83a
AAEL01735	NA	0.28526071231904	0.169556313320174	0.589443594700505	NA	NA	NA	XP_021697163.1uncharacterized protein LOC5571052 isoform X1
AAEL00938	hexokinase type 2	0.28561541055871	0.0269333964121721	0.267809217385466	00524 ; 00520 ; 00051 ;	aag01100 ; aag	NA	XP_001660031.1hexokinase type 2 isoform X5
AAEL02048	NA	0.285864335300952	0.371951663062208	0.781817769662763	NA	NA	NA	XP_019560237.1 menin-like
AAEL00534	uncharacterized LOC5566384	0.285902789386304	0.314947197110626	0.745407435136825	NA	NA	KXJ78091.1 hypothet	XP_021702372.1uncharacterized protein LOC5566384

AAEL0086?	ras-related protein Rab-9B	0.285978948133704	0.148421312505639	0.562679383080301	NA	NA	ETN60938.1 ras-relat	XP_001659411.1ras-related protein Rab-9B
AAEL0065?	transcription elongation factor SPT4	0.286042200415903	0.11844550064832	0.51638627117789	NA	NA	XP_001870822.1 trai	XP_001652051.1transcription elongation factor SPT4
AAEL0064?	zinc finger protein Noc	0.286241511057588	0.0487931308742345	0.351987741182022	NA	NA	ETN58091.1 zinc fing	XP_021700198.1zinc finger protein Noc
AAEL0128?	polypeptide N-acetylgalactosaminyltransferase 1	0.286293262635831	0.144392336942253	0.557106198969649	NA	aag01100 ; aag	XP_001861920.1 pol	XP_001662949.1polypeptide N-acetylgalactosaminyltransferase 1
AAEL0243?	NA	0.286478671378633	0.0599926643507539	0.383636203860763	NA	NA	NA	AAL76036.1putative 18.2 kDa secreted protein
AAEL0091?	uncharacterized LOC5571590	0.28658532023897	0.282193630126942	0.715554087745416	NA	NA	KXJ79575.1 hypothet	XP_001659798.1uncharacterized protein LOC5571590
AAEL0137?	ADP-ribosylation factor-like protein 5B	0.286921187555406	0.0190620005695675	0.225171411092632	NA	NA	NA	XP_001663965.1ADP-ribosylation factor-like protein 5B
AAEL0007?	matrix metalloproteinase-2	0.287094126285208	0.23747346492019	0.671758630247645	NA	NA	KXJ80966.1 hypothet	XP_021704764.1matrix metalloproteinase-2
AAEL0034?	caspase	0.287164576460282	0.24814396132269	0.68058844480365	NA	aag04214 ; aag	KXJ71200.1 hypothet	XP_019528553.1 caspase-like
AAEL0079?	NA	0.287342161190897	0.188431509329471	0.615311124503122	NA	NA	KXJ82594.1 hypothet	XP_001652990.2 sulfotransferase family cytosolic 1B member 1
AAEL0236?	NA	0.287365300848019	0.0490169821694811	0.352661665303804	NA	NA	NA	XP_021708722.1neurofilament light polypeptide-like
AAEL0221?	NA	0.287668967612425	0.121857598642785	0.523911973275319	NA	aag01100 ; aag	NA	XP_001657412.2endoplasmic reticulum mannosyl-oligosaccharide 1,2-alpha-mannosidase
AAEL0124?	protein argonaute-2	0.287783905460479	0.0447800625442903	0.335285708325923	NA	NA	NA	XP_021704835.1protein argonaute-2 isoform X15
AAEL0116?	NA	0.287843967243547	0.10956272741258	0.498613361844988	NA	NA	KXJ70303.1 hypothet	XP_001661846.2acyl-CoA synthetase family member 2, mitochondrial isoform X1
AAEL0111?	dual specificity protein kinase TTK	0.287855983966556	0.29806508223623	0.7302583490254	NA	NA	KXJ70495.1 hypothet	XP_021706968.1dual specificity protein kinase TTK isoform X1
AAEL0045?	tyrosine-protein kinase Src64B	0.28790371767514	0.278422360121029	0.713122992430066	NA	aag04144 ; aag	ETN60976.1 tyrosine	XP_021696171.1tyrosine-protein kinase Src64B
AAEL0119?	zinc finger protein 883	0.288200972230145	0.380131241731761	0.786236836685921	NA	NA	KXJ73653.1 hypothet	XP_021703716.1zinc finger protein 883
AAEL0209?	NA	0.28850622737221	0.284401806143025	0.716726857594605	NA	NA	NA	XP_021692939.1uncharacterized protein LOC5565478
AAEL0119?	mannose-1-phosphate guanyltransferase alpha-A	0.288671270992631	0.0734478471315489	0.417762644616207	NA	aag01100 ; aag	KFB42733.1 AGAP01	XP_001662032.1mannose-1-phosphate guanyltransferase alpha-A
AAEL0098?	uncharacterized LOC5572487	0.288708869071956	0.484744408572527	0.847655581875201	NA	NA	NA	XP_021706934.1uncharacterized protein LOC5572487 isoform X2
AAEL0109?	NA	0.289192886166091	0.0332013175705019	0.286066033973556	NA	NA	XP_019552321.1 PRE	XP_001661213.2probable Xaao aminopeptidase 3
AAEL0064?	transcription initiation factor TFIID subunit 7	0.289312864833938	0.18899008497727	0.616603527036593	NA	aag03022	KXJ76165.1 hypothet	XP_021708299.1transcription initiation factor TFIID subunit 7
AAEL0202?	NA	0.28969876946565	0.26822608935315	0.704184172798867	NA	NA	NA	NA
AAEL0071?	N-alpha-acetyltransferase 35, NatC auxiliary subunit	0.289803439588728	0.0656567180406831	0.39800600751671	NA	NA	KXJ83695.1 hypothet	XP_001658127.2N-alpha-acetyltransferase 35, NatC auxiliary subunit isoform X2
AAEL0005?	GDP-Man:Man(3)GlcNAc(2)-PP-Dol alpha-1,2-mannosyltransferase	0.289927723978979	0.0271160910271242	0.268836665026084	00510 ; 00513	aag01100 ; aag	KXJ73015.1 hypothet	XP_001647965.1GDP-Man:Man(3)GlcNAc(2)-PP-Dol alpha-1,2-mannosyltransferase
AAEL0209?	NA	0.289969561311581	0.298065082248915	0.7302583490254	NA	NA	NA	XP_021697595.1ATP-dependent DNA helicase Q4
AAEL0076?	uncharacterized LOC5569508	0.290026942474603	0.0348185093626959	0.295299468346109	NA	NA	KXJ80023.1 hypothet	XP_001658593.2uncharacterized protein LOC5569508
AAEL0115?	uncharacterized LOC5574953	0.290047420238365	0.0316605055263593	0.28423411888152	NA	NA	XP_001844554.1 con	XP_001655423.2uncharacterized protein LOC5574953
AAEL0036?	uncharacterized LOC5578747	0.290049526634272	0.201514654698273	0.634701781910801	NA	NA	KXJ72952.1 hypothet	XP_001657114.1uncharacterized protein LOC5578747
AAEL0094?	uncharacterized LOC5572008	0.290109777352188	0.047860853104398	0.348612005978262	NA	NA	NA	XP_001660114.1uncharacterized protein LOC5572008
AAEL0054?	probable salivary secreted peptide	0.290115743666833	0.0347519889234433	0.295097377978161	NA	NA	ACF72876.1 unknow	XP_001650838.1probable salivary secreted peptide
AAEL0219?	NA	0.290152745242688	0.469008121905914	0.840469861300529	NA	NA	NA	NA
AAEL0063?	nucleoporin SEH1	0.290576857863907	0.166047209423908	0.584439853413553	NA	aag03013 ; aag	ETN60738.1 nucleop	XP_001651881.1nucleoporin SEH1
AAEL0125?	probable phospholipid-transporting ATPase IF	0.290725242356094	0.0303542937516128	0.281006248593904	NA	NA	NA	XP_021694978.1probable phospholipid-transporting ATPase IF isoform X3
AAEL0096?	NA	0.2910013636885	0.0349296060069993	0.295696627853181	NA	aag04120 ; aag	NA	XP_001653865.1 E3 ubiquitin-protein ligase sina
AAEL0203?	NA	0.291471916159995	0.0248754439319239	0.258356472981449	NA	aag04120	NA	XP_021695631.1cell division cycle protein 27 homolog
AAEL0125?	probable phosphoserine aminotransferase	0.291486007582663	0.0300304923162512	0.279963027796095	00680 ; 00750 ; 00260	aag01100 ; aag	KXJ81423.1 hypothet	XP_001662710.2probable phosphoserine aminotransferase
AAEL0150?	uncharacterized LOC5566035	0.291550465577622	0.245386841042825	0.679727114240672	NA	NA	NA	XP_001650400.2uncharacterized protein LOC5566035 isoform X1
AAEL0052?	galectin-4	0.291627657536226	0.0718116958028221	0.411912175099304	NA	NA	KFB46933.1 AGAP00	XP_019525286.1 galectin-4
AAEL0053?	beta-1,3-glucosyltransferase	0.291729841382457	0.358426311118842	0.773854174836904	NA	aag00514	KFB44855.1 hypothe	XP_021702362.1beta-1,3-glucosyltransferase isoform X1
AAEL0103?	soma ferritin	0.29187196560753	0.473831419852425	0.843946647614615	NA	NA	NA	XP_001654517.1soma ferritin
AAEL0065?	protein TRC8 homolog	0.29239749080003	0.0190217458551999	0.225171411092632	NA	NA	NA	XP_001657898.2protein TRC8 homolog isoform X4
AAEL0030?	cuticle protein 2	0.2930478562095	0.391024148519694	0.793285976044306	NA	NA	KXJ68961.1 hypothet	XP_001662974.1cuticle protein 2
AAEL0063?	probable Ufm1-specific protease 2	0.293108089209454	0.0516214850534827	0.360410398681448	NA	NA	KXJ82331.1 hypothet	XP_001651941.1probable Ufm1-specific protease 2 isoform X1
AAEL0119?	uncharacterized LOC5575679	0.293345759018662	0.443116203374377	0.82545872799161	NA	NA	ETN58632.1 hypothe	KFB49190.1AGAP009812-like protein
AAEL0085?	UHRF1-binding protein 1-like	0.293511995886243	0.0140925417192058	0.190425211307095	NA	NA	KXJ68451.1 hypothet	XP_021702177.1 UHRF1-binding protein 1-like
AAEL0040?	probable RNA-binding protein 19	0.293800108697173	0.0565900655058922	0.374365048731287	NA	NA	KXJ77485.1 hypothet	XP_001648356.2probable RNA-binding protein 19
AAEL0119?	kelch domain-containing protein 10 homolog	0.294119960515342	0.0655923908122178	0.397878167603183	NA	NA	XP_001841785.1 kel	XP_001662113.2kelch domain-containing protein 10 homolog
AAEL0125?	bifunctional heparan sulfate N-deacetylase/N-sulfotransferase	0.294286376562684	0.122445178350766	0.524553319917944	NA	aag01100 ; aag	XP_001849969.1 hep	XP_021697038.1bifunctional heparan sulfate N-deacetylase/N-sulfotransferase
AAEL0064?	solute carrier family 17 member 9	0.294788200645093	0.156641155816618	0.574474189691589	NA	NA	ETN59876.1 sialin [A	XP_001657797.1solute carrier family 17 member 9
AAEL0034?	alsin homolog	0.29480746837034	0.141448064583437	0.552330595273689	NA	NA	KXJ77257.1 hypothet	XP_001656816.2alsin homolog
AAEL0080?	NA	0.294903500656507	0.142511056212626	0.554732123210059	NA	aag01100 ; aag	KXJ80605.1 hypothet	XP_001658839.2polypeptide N-acetylgalactosaminyltransferase 35A
AAEL0100?	zinc finger protein 547	0.295361105490181	0.317855600353882	0.745684464362801	NA	NA	NA	XP_001660559.2zinc finger protein 547
AAEL0087?	probable small nuclear ribonucleoprotein G	0.295424551436223	0.0288385083881957	0.274601260220884	NA	aag03040	ETN65074.1 small rib	XP_001659450.1probable small nuclear ribonucleoprotein G
AAEL0092?	venom serine carboxypeptidase	0.295759530290368	0.0135529021744867	0.187825791015408	NA	NA	NA	XP_001659906.2venom serine carboxypeptidase
AAEL0010?	uncharacterized LOC5568351	0.295824873114082	0.213273913839925	0.646373717545434	NA	NA	KFB53537.1 AGAP00	KFB53537.1AGAP004167-like protein
AAEL0148?	28S ribosomal protein S15, mitochondrial	0.295885783417208	0.0908939219887835	0.458582874647784	NA	aag03010	NA	XP_001649689.128S ribosomal protein S15, mitochondrial
AAEL0094?	uncharacterized LOC5572010	0.296008622485627	0.219758076425397	0.652412844728166	NA	NA	NA	XP_001660116.2uncharacterized protein LOC5572010
AAEL0078?	WW domain-containing oxidoreductase	0.296109678696959	0.165438579833181	0.584439853413553	NA	NA	KXJ80241.1 hypothet	XP_001652932.1WW domain-containing oxidoreductase
AAEL0182?	NA	0.296230679035691	0.155386228074278	0.573091811919643	NA	NA	NA	XP_021707393.1protein ovarian tumor locus isoform X2
AAEL0278?	NA	0.296289304962966	0.38386399998279	0.79005066603481	NA	NA	NA	XP_021694992.1D-beta-hydroxybutyrate dehydrogenase, mitochondrial
AAEL0089?	uncharacterized LOC5571308	0.296374811711583	0.159906398605117	0.577560236664366	NA	NA	NA	XP_021696508.1uncharacterized protein LOC5571308 isoform X2
AAEL0039?	mitochondrial inner membrane protease subunit 1	0.296475259663217	0.115253470024554	0.511637141340239	NA	aag03060	KXJ76957.1 hypothet	XP_001648009.2mitochondrial inner membrane protease subunit 1
AAEL0281?	NA	0.296769868064268	0.279408327537833	0.713604060506006	NA	NA	NA	XP_021695491.1DNA-directed RNA polymerase III subunit RPC9 isoform X2
AAEL0115?	ras-related and estrogen-regulated growth inhibitor-like protein	0.296774106934989	0.423598887231653	0.814192545906728	NA	NA	KFB43135.1 MRAS2,	XP_001661722.1ras-related and estrogen-regulated growth inhibitor-like protein
AAEL0006?	UDP-glucuronosyltransferase 1-3	0.296822167123384	0.102893421954154	0.485796149609891	NA	NA	XP_019561072.1 PRE	XP_001650274.2UDP-glucuronosyltransferase 1-3
AAEL0268?	NA	0.296825531453109	0.0264834692320497	0.266145614979556	NA	NA	NA	XP_021695141.1BTB/POZ domain-containing protein 9
AAEL0142?	6-phosphogluconolactonase	0.297010276140184	0.0272531874385873	0.268836665026084	00030 ; 00520	aag01100 ; aag	NA	XP_019933445.1 6-phosphogluconolactonase-like
AAEL0039?	general transcription factor IIF subunit 1	0.297033917004798	0.0199624961485207	0.228870494380427	NA	aag03022	KXJ69666.1 hypothet	XP_001648068.1general transcription factor IIF subunit 1
AAEL0094?	methyltransferase-like protein 16 homolog	0.297304753499853	0.408849316727523	0.805099810084884	NA	NA	NA	XP_001660022.2methyltransferase-like protein 16 homolog
AAEL0182?	NA	0.297634142672469	0.181695793006413	0.607917241925556	NA	NA	NA	XP_021704941.1uncharacterized protein LOC5575802 isoform X2
AAEL0020?	probable malonyl-CoA-acyl carrier protein transacylase, mitochondrial	0.297785734282143	0.190270883061588	0.618521820583848	NA	aag01100 ; aag	KXJ81258.1 hypothet	XP_001654591.1probable malonyl-CoA-acyl carrier protein transacylase, mitochondrial
AAEL0000?	integrator complex subunit 10	0.29779272136785	0.248243644562367	0.68058844480365	NA	NA	XP_001864745.1 con	XP_001647882.2integrator complex subunit 10
AAEL0019?	uncharacterized LOC5573121	0.297997191312816	0.0894945208017473	0.457261843652237	NA	NA	KFB39439.1 AGAP00	KFB39439.1AGAP008073-like protein
AAEL0030?	UDP-glucuronosyltransferase 2B31	0.298047531240008	0.0263054851240474	0.264839249574928	NA	NA	ETN60648.1 glucosyl	XP_001663067.1UDP-glucuronosyltransferase 2B31
AAEL0038?	zinc finger protein 711	0.298142795129703	0.183327467105089	0.610828860145329	NA	NA	KXJ71510.1 hypothet	XP_021704986.1zinc finger protein 711
AAEL0252?	NA	0.298406302265094	0.0807782117917575	0.433079891352508	NA	NA	NA	XP_021706038.1dnaJ homolog subfamily C member 11
AAEL0074?	transmembrane protein 223	0.298740752673172	0.212933847316495	0.646249117936982	NA	NA	XP_001861585.1 con	XP_001658357.1transmembrane protein 223
AAEL0100?	male-specific lethal 3 homolog	0.299116287023877	0.183044670648506	0.610281543227372	NA	NA	NA	XP_021704347.1male-specific lethal 3 homolog isoform X1
AAEL0135?	NA	0.299286832388122	0.23988247176532	0.674660318489854	NA	NA	NA	XP_021712963.1uncharacterized protein LOC110681487
AAEL0056?	60S ribosomal protein L7	0.299410266673572	0.0269788246594574	0.267809217385466	NA	aag03010	KXJ77011.1 hypothet	XP_001651184.260S ribosomal protein L7
AAEL0065?	THO complex subunit 6	0.299506716374013	0.341293804108588	0.762180211699347	NA	aag03013	KXJ72283.1 hypothet	XP_001652050.1THO complex subunit 6

AAEL0096; U6 snRNA-associated Sm-like protein LSm3	0.300604465749345	0.141471503309834	0.552330595273689	NA	aag03040 ; aag	NA	XP_001653925.1U6 snRNA-associated Sm-like protein LSm3
AAEL0120{ NA	0.300871395358902	0.0257353616187658	0.261965484088366	NA	NA	KFB41964.1 AGAP00	KFB41964.1AGAP000743-like protein
AAEL0140; mediator of RNA polymerase II transcription subunit 23	0.301451091024707	0.219674131418795	0.652412844728166	NA	NA	NA	XP_001664240.1mediator of RNA polymerase II transcription subunit 23
AAEL0016{ laccase-2	0.301451942027348	0.0952300982786771	0.466773927728342	NA	NA	KXJ78644.1 hypothe	AAY32604.1laccase-like multicopper oxidase 2
AAEL0128: protein archease-like	0.301483913566636	0.145520312138736	0.558950568057442	NA	NA	KXJ74990.1 hypothe	XP_001662944.2protein archease-like
AAEL0081(guanine nucleotide-binding protein subunit beta-2	0.302106672705384	0.015486721502174	0.201125113930603	NA	aag04745	ETN60310.1 guanine	XP_001653114.1guanine nucleotide-binding protein subunit beta-2
AAEL0105: translocation protein SEC62	0.302146137404781	0.00993067134175744	0.161167614967993	NA	aag04141 ; aag	XP_019547986.1 PRE	XP_001660898.2translocation protein SEC62
AAEL0004; U6 snRNA-associated Sm-like protein LSm1	0.302238149992851	0.12890425462045	0.535335481333802	NA	aag03018	ETN58022.1 CaSm [A	XP_001656966.1U6 snRNA-associated Sm-like protein LSm1
AAEL0011: serine/threonine-protein phosphatase 4 regulatory subunit 2	0.3022528436876	0.0697135068568972	0.405758184754692	NA	NA	KFB35038.1 AGAP00	XP_001652384.1serine/threonine-protein phosphatase 4 regulatory subunit 2
AAEL0273: NA	0.302263845633584	0.283439174044063	0.715753918648043	NA	aag01100 ; aag	NA	XP_021706268.1putative aldehyde dehydrogenase family 7 member A1 homolog isoform X1
AAEL0028: electron transfer flavoprotein regulatory factor 1	0.302448516849551	0.0605934510231881	0.384941803020695	NA	NA	KXJ77322.1 hypothe	XP_001662481.2electron transfer flavoprotein regulatory factor 1
AAEL0035: parafibromin	0.30259601186484	0.0534055287605082	0.365578024386907	NA	NA	ETN60952.1 cdc73 d	XP_019536663.1 parafibromin-like
AAEL0055: uncharacterized LOC5579813	0.302719315534313	0.159977236618603	0.577560236664366	NA	NA	KFB36823.1 AGAP00	KFB36823.1AGAP000359-like protein
AAEL0072: zinc finger protein 2 homolog	0.302891800357659	0.359726908740113	0.774255626424296	NA	NA	KXJ83148.1 hypothe	XP_001658253.2zinc finger protein 2 homolog
AAEL0066; exosome complex component MTR3	0.303091991883457	0.167694245502444	0.587554508928878	NA	aag03018	KXJ73413.1 hypothe	XP_001657962.2exosome complex component MTR3
AAEL0002: vacuolar protein sorting-associated protein 33A	0.303266163855993	0.186849680255836	0.614196367514984	NA	NA	XP_001864004.1 vac	XP_001658892.1vacuolar protein sorting-associated protein 33A
AAEL0267! NA	0.303288443177211	0.227770660460314	0.661754201421 27	NA	aag03013	NA	XP_021696643.1probable nucleoporin Nup54 isoform X1
AAEL0197: NA	0.303460175038975	0.0121115552439778	0.178320850168134	NA	NA	NA	XP_021704328.1heat shock factor protein isoform X1
AAEL0091: probable cytochrome P450 6d4	0.303508145034413	0.00917894752644156	0.156997537431813	NA	NA	XP_019538184.1 PRE	XP_021703132.1probable cytochrome P450 6d4
AAEL0073! ecdysone-inducible protein E75	0.303535003426154	0.0644014230467676	0.395929840165835	NA	NA	XP_019536151.1 PRE	XP_001652743.3ecdysone-inducible protein E75 isoform X3
AAEL0110: condensin complex subunit 3	0.303573242810289	0.133522225015391	0.54189626993465	NA	NA	XP_001848901.1 cap	XP_001661330.2condensin complex subunit 3
AAEL0111: GPN-loop GTPase 3	0.303750322211944	0.198943389736256	0.63257673543643	NA	NA	ETN59682.1 transcrip	XP_001655116.1GPN-loop GTPase 3
AAEL0277{ NA	0.304228030957062	0.218829176043143	0.652412844728166	NA	NA	NA	NA
AAEL0096; Golgi-specific brefeldin A-resistance guanine nucleotide exchange factor :	0.30438360991211	0.0197763311052356	0.227477248537973	NA	aag04144	NA	XP_001653922.2Golgi-specific brefeldin A-resistance guanine nucleotide exchange factor 1
AAEL0115: phosphoacetylglucosamine mutase	0.30458525342965	0.0686385728056338	0.402813869233063	00520 ; 00520	aag01100 ; aag	KXJ82014.1 hypothe	XP_001661733.1phosphoacetylglucosamine mutase
AAEL0169! protein white	0.304652468387459	0.130537457814293	0.537336444138125	NA	NA	NA	XP_011492980.2protein white isoform X2
AAEL0172{ protein SREK1IP1	0.304799811049926	0.43742874775079	0.820960220424486	NA	NA	NA	XP_011493475.2protein SREK1IP1
AAEL0074: H/ACA ribonucleoprotein complex subunit 3	0.30486883937006	0.00945925295760156	0.158773875613038	NA	aag03008	KFB44403.1 AGAP00	XP_001658356.1H/ACA ribonucleoprotein complex subunit 3
AAEL0181{ NA	0.305496027913792	0.397892715104265	0.797539006884967	NA	NA	NA	XP_021703021.1tripartite motif-containing protein 45 isoform X1
AAEL0213; NA	0.305682114592396	0.171674990234778	0.593446003058012	NA	NA	NA	XP_019544042.1 suppressor of lurcher protein 1-like
AAEL0052! natterin-4	0.305965039531257	0.111260180860778	0.502609810643532	NA	NA	XP_019550105.1 PRE	XP_001650603.2natterin-4 isoform X2
AAEL0116! pachytene checkpoint protein 2 homolog	0.306171397830772	0.386950714967444	0.791623050051228	NA	NA	KXJ75937.1 hypothe	XP_001661836.1pachytene checkpoint protein 2 homolog
AAEL0103{ HMG box-containing protein 4	0.306388315280541	0.160804026958748	0.578015100029063	NA	NA	NA	XP_001654490.2HMG box-containing protein 4
AAEL0175: holotricin-3	0.306570773881201	0.0234404747033435	0.248788060230873	NA	NA	NA	XP_021699492.1holotricin-3 isoform X2
AAEL0128(AH receptor-interacting protein	0.306639146611605	0.25245680910579	0.684556086619159	NA	NA	KXJ84107.1 hypothe	XP_001656273.2AH receptor-interacting protein
AAEL0070: MICAL-like protein 1	0.307690574686978	0.0084454114163927	0.148894695587464	NA	NA	XP_019526798.1 PRE	XP_021707713.1MICAL-like protein 1
AAEL0023: acetyl-coenzyme A transporter 1	0.307693911747391	0.0231514220693964	0.24632223885158	NA	aag01100 ; aag	KXJ76381.1 hypothe	XP_001661369.2acetyl-coenzyme A transporter 1
AAEL0231! NA	0.307739391055919	0.503841561110126	0.858744381072989	NA	NA	NA	NA
AAEL0019: fas-associated death domain protein	0.307852996978337	0.387458383925249	0.791825725928371	NA	aag04624 ; aag	KXJ81517.1 hypothe	XP_001654342.2fas-associated death domain protein
AAEL0015! zinc finger protein 880	0.308830541536519	0.206096509885086	0.638337288442465	NA	NA	NA	XP_001653557.2zinc finger protein 880
AAEL0248: NA	0.309028990486013	0.00983717100442653	0.161070547300237	NA	NA	NA	XP_021694529.1serine/threonine-protein kinase tousled-like 2 isoform X3
AAEL0016: proteasome activator complex subunit 3	0.309209501968931	0.00799000523304946	0.144528409372715	NA	aag03050	CRK96527.1 CLUMA_	XP_001659750.2proteasome activator complex subunit 3
AAEL0113: borealin	0.309337702118897	0.495147687653463	0.854048551412777	NA	NA	XP_019539181.1 PRE	XP_001655255.1borealin isoform X1
AAEL0008: flavin-containing monooxygenase FMO GS-OX-like 6	0.309856541234852	0.23481174686289	0.668737559309875	NA	NA	KXJ76796.1 hypothe	XP_001651280.2flavin-containing monooxygenase FMO GS-OX-like 6
AAEL0114: E3 ubiquitin-protein ligase RNF25	0.309945669378273	0.163972442745181	0.582352149031707	NA	NA	KXJ84258.1 hypothe	XP_001655338.2E3 ubiquitin-protein ligase RNF25
AAEL0218! NA	0.310247187465705	0.267908744220901	0.704026692311667	NA	NA	NA	NA
AAEL0261: NA	0.310330810735644	0.0136556437672428	0.187831676988403	NA	NA	NA	XP_021699115.1uncharacterized protein LOC5576069
AAEL0268: NA	0.310634438749052	0.0307824757100015	0.281422122950505	NA	NA	NA	XP_021693614.1facilitated trehalose transporter Tret1
AAEL0023! esterase B1	0.310672156082075	0.39661476753307	0.797040640060998	NA	NA	XP_019539369.1 PRE	XP_001655128.2esterase B1 isoform X1
AAEL0048: dolichol-phosphate mannosyltransferase subunit 3	0.310763485194744	0.101654036725607	0.483848092317719	NA	aag01100 ; aag	ETN62497.1 hypothe	XP_001649950.2dolichol-phosphate mannosyltransferase subunit 3
AAEL0065(probable RNA methyltransferase CG11342	0.310841292506574	0.129051476828599	0.535335481333802	NA	NA	KXJ83505.1 hypothe	XP_001652031.2probable RNA methyltransferase CG11342
AAEL0099(glutamyl-tRNA(Gln) amidotransferase subunit B, mitochondrial	0.310896355158737	0.132666038990147	0.540110467949322	NA	aag01100 ; aag	NA	XP_001654104.2glutamyl-tRNA(Gln) amidotransferase subunit B, mitochondrial
AAEL0034: uncharacterized LOC5578080	0.311267573671753	0.25098146537054	0.683858606596939	NA	NA	KXJ78717.1 hypothe	XP_001656773.1uncharacterized protein LOC5578080
AAEL0094: protein peste	0.311327225146618	0.090833657196815	0.458582874647784	NA	NA	NA	XP_021700410.1protein peste
AAEL0121: cationic amino acid transporter 2	0.31157569993017	0.0358432552465263	0.299945082305895	NA	NA	XP_001845825.1 cati	XP_021707900.1cationic amino acid transporter 2
AAEL0110! putative methyltransferase C9orf114	0.31172185690501	0.143235806017126	0.555375365647863	NA	NA	ETN59855.1 hypothe	XP_001661329.1putative methyltransferase C9orf114
AAEL0094{ bis(5'-adenosyl)-triphosphatase ENPP4	0.31172895666949	0.00864915814965281	0.150424209534052	NA	NA	NA	XP_001660105.2bis(5'-adenosyl)-triphosphatase ENPP4
AAEL0107: glutaminyl-peptide cyclotransferase	0.311758616021313	0.0314924016519187	0.28423411888152	NA	NA	XP_019538241.1 PRE	XP_001661052.1glutaminyl-peptide cyclotransferase
AAEL0009! protein LTV1 homolog	0.312554880974789	0.0201596740858207	0.230446361413319	NA	NA	XP_001869839.1 con	XP_001657473.1protein LTV1 homolog
AAEL0052: probable Golgi SNAP receptor complex member 2	0.312861014499456	0.17974861590262	0.604991500927545	NA	aag04130	XP_001847979.1 me	XP_001650534.2probable Golgi SNAP receptor complex member 2
AAEL0110{ zinc finger protein Elbow	0.313035191912512	0.160220163716794	0.577674739374419	NA	NA	XP_001842450.1 elbo	XP_001661395.3zinc finger protein Elbow
AAEL0244; NA	0.313198748663844	0.077979973001469	0.42745012137719	901	NA	NA	XP_021705735.1uncharacterized protein LOC5578432
AAEL0056(ectonucleoside triphosphate diphosphohydrolase 5	0.313401460881974	0.00943592100889265	0.158773875613038	NA	aag01100 ; aag	XP_019530277.1 PRE	XP_021702551.1ectonucleoside triphosphate diphosphohydrolase 5 isoform X2
AAEL0035: SET and MYND domain-containing protein 4	0.313446897851697	0.229251118475018	0.662487607456997	NA	NA	KXJ84411.1 hypothe	XP_001656929.2SET and MYND domain-containing protein 4
AAEL0119: NA	0.313459696794814	0.219945507083508	0.652412844728166	NA	NA	ETN66088.1 hypothe	XP_021699284.1biogenesis of lysosome-related organelles complex 1 subunit 2
AAEL0020! cytoplasmic tRNA 2-thiolation protein 2	0.313479513136524	0.220170299612858	0.652412844728166	NA	aag04122	KXJ81259.1 hypothe	XP_001654590.2cytoplasmic tRNA 2-thiolation protein 2
AAEL0096(uncharacterized LOC5572221	0.313891420158076	0.067679226189092	0.40094231299198	NA	NA	NA	XP_021710698.1uncharacterized protein LOC5572221
AAEL0249: NA	0.314113220834107	0.17384952784703	0.597149442048662	NA	aag00981	NA	XP_021713213.1phosphoglycolate phosphatase 1B, chloroplastic
AAEL0017: RING finger protein 113A	0.314158944650485	0.14835626370712	0.562679383080301	NA	NA	ETN58117.1 RING fin	XP_001653974.1RING finger protein 113A
AAEL0244! NA	0.314173104477892	0.016480315505746	0.207174676617315	NA	NA	NA	NA
AAEL0261: NA	0.314323949006187	0.377286286710516	0.783522548027572	NA	NA	NA	XP_021697437.1zinc finger protein 836
AAEL0079(uncharacterized LOC5569765	0.314352473501916	0.0828699711573078	0.437924968110882	NA	NA	NA	XP_001652986.2uncharacterized protein LOC5569765 isoform X1
AAEL0146: NA	0.314499390107051	0.29927345516235	0.730496185008314	NA	NA	NA	XP_001649106.2probable cytochrome P450 9f2
AAEL0084: derlin-2	0.314532755191195	0.0237943150355454	0.251095512565469	NA	aag04141	XP_012256136.1 der	XP_019546776.1 derlin-2-like
AAEL0267: NA	0.315048176063124	0.336448440907948	0.758445425575872	NA	NA	NA	XP_021705964.1uncharacterized protein LOC5576006
AAEL0040! ragulator complex protein LAMTOR3 homolog	0.315220624859034	0.0614557060904336	0.38668618011863	NA	aag04150	XP_001851099.1 mit	XP_001648368.1ragulator complex protein LAMTOR3 homolog
AAEL0174: nucleolar protein 58	0.315278123664031	0.0159393729445388	0.203220814696528	NA	aag03008	NA	XP_021706707.1nucleolar protein 58 isoform X2
AAEL0030! UDP-glucuronosyltransferase 2B15	0.315511348760655	0.177347198564617	0.602182613972581	NA	NA	XP_001841707.1 UDI	XP_001663065.1UDP-glucuronosyltransferase 2B15
AAEL0118: poly [ADP-ribose] polymerase	0.31567905572014	0.0352656102217958	0.297446512613167	NA	aag04214 ; aag	KXJ68804.1 hypothe	XP_001661932.1poly [ADP-ribose] polymerase
AAEL0077: anoctamin-4	0.315681862495048	0.406913563761267	0.803523307667635	NA	NA	XP_019531842.1 PRE	XP_001652855.2anoctamin-4 isoform X2
AAEL0066: acetyl-CoA acetyltransferase, mitochondrial	0.315709678122148	0.0277853671325825	0.271255206147924	NA	aag01100 ; aag	KXJ71243.1 hypothe	XP_001657918.2acetyl-CoA acetyltransferase, mitochondrial
AAEL0058: serine--tRNA ligase, mitochondrial	0.316726341848548	0.113054593672703	0.504035063457468	970	aag00970	KXJ74080.1 hypothe	XP_021701584.1 serine--tRNA ligase, mitochondrial

AAEL0090	ubiquitin fusion degradation protein 1 homolog	0.317597980552522	0.0491622041203028	0.352961636078127	NA	aag04141	KXJ77245.1 hypothet	XP_001653664.1ubiquitin fusion degradation protein 1 homolog isoform X1
AAEL0039	armadillo repeat-containing protein 7	0.317605270979274	0.115666683420394	0.512764772622422	NA	NA	KXJ76209.1 hypothet	XP_021707588.1 armadillo repeat-containing protein 7
AAEL0196	NA	0.317771110783504	0.131240606354606	0.538981683630591	NA	NA	NA	XP_021698888.1esterase B1
AAEL0290	NA	0.317794115686058	0.0364432024884294	0.301845498918566	NA	NA	NA	XP_001661894.2protein HGH1 homolog isoform X1
AAEL0088	gametocyte-specific factor 1 homolog	0.317932806906382	0.128628455460409	0.535335481333802	NA	NA	XP_001849746.1 con	XP_001653467.1gametocyte-specific factor 1 homolog
AAEL0015	splicing factor 3A subunit 2	0.317996028519831	0.0172421555375448	0.213830613553218	NA	NA	KXJ83030.1 hypothet	XP_001653574.1splicing factor 3A subunit 2
AAEL0140	breast cancer metastasis-suppressor 1-like protein	0.318137576213131	0.120238115955713	0.521361270092351	NA	NA	NA	XP_001664237.1breast cancer metastasis-suppressor 1-like protein
AAEL0082	retinol-binding protein pinta	0.31830098757207	0.201787166427921	0.634701781910801	NA	NA	ETN68020.1 SEC14 [	XP_001659080.1retinol-binding protein pinta
AAEL0128	vesicle-trafficking protein SEC22b-B	0.318659930176875	0.0183709559516418	0.220116584201833	NA	aag04145 ; aag	NA	XP_001656296.1vesicle-trafficking protein SEC22b-B
AAEL0095	probable nuclear hormone receptor HR3	0.31899528495833	0.35494697339294	0.772704530201522	NA	NA	NA	XP_021702098.1probable nuclear hormone receptor HR3 isoform X5
AAEL0025	Y+L amino acid transporter 2	0.319130594060107	0.0184589646336602	0.220883475369234	NA	NA	XP_001866233.1 am	XP_001655456.2Y+L amino acid transporter 2
AAEL0050	zinc finger protein 658B	0.319202523919749	0.219494773900671	0.652412844728166	NA	NA	NA	XP_021693926.1zinc finger protein 25 isoform X3
AAEL0120	uncharacterized LOC5575820	0.319305808761706	0.051750021631466	0.361033888591926	NA	NA	KXJ76207.1 hypothet	XP_019562172.1 toll-like receptor 6
AAEL0279	NA	0.319509863705796	0.256026055006505	0.688406212440425	NA	NA	NA	XP_021697493.1zinc finger protein 84
AAEL0015	U3 small nucleolar ribonucleoprotein protein MPP10	0.319804946465589	0.0825623586435964	0.437257081788889	NA	aag03008	KXJ80975.1 hypothet	XP_021700627.1U3 small nucleolar ribonucleoprotein protein MPP10
AAEL0052	pseudouridine-5'-phosphate glycosidase	0.320026714134019	0.0691779096845606	0.403786365070033	240	NA	XP_001865719.1 con	XP_001650532.2uncharacterized protein LOC5566172
AAEL0036	splicing factor 3B subunit 1	0.320075989848347	0.0164190153585321	0.206858269058754	NA	aag03040	XP_019552331.1 PRE	XP_001657046.1splicing factor 3B subunit 1 isoform X1
AAEL0072	carbohydrate-responsive element-binding protein	0.320416489674831	0.00760380421031267	0.142450721693651	NA	NA	XP_019932809.1 PRE	XP_021701391.1carbohydrate-responsive element-binding protein isoform X1
AAEL0127	nuclear pore complex protein Nup160 homolog	0.320861038200576	0.0590606108252478	0.381093648037575	NA	NA	KXJ72815.1 hypothet	XP_021698910.1nuclear pore complex protein Nup160 homolog
AAEL0008	FAST kinase domain-containing protein 2, mitochondrial	0.320937600407664	0.167311304853891	0.587554508928878	NA	NA	XP_001863504.1 con	XP_001651269.2FAST kinase domain-containing protein 2, mitochondrial
AAEL0123	cytochrome c oxidase assembly factor 5	0.321408805061367	0.0468893136876553	0.345456737032669	NA	NA	XP_001870888.1 PET	XP_001662427.1cytochrome c oxidase assembly factor 5
AAEL0084	vacuolar protein sorting-associated protein 8 homolog	0.321708656552875	0.0740777955785969	0.418712453878531	NA	NA	KXJ71558.1 hypothet	XP_001659262.2vacuolar protein sorting-associated protein 8 homolog
AAEL0170	cytochrome P450 6a8	0.322011842590029	0.0260394213481312	0.262907960607041	NA	NA	NA	XP_021693513.1cytochrome P450 6a8
AAEL0136	adenylate kinase isoenzyme 6 homolog	0.322149744779306	0.0828931326835357	0.437924968110882	00230 ; 00730	aag01100 ; aag	NA	XP_021694849.1adenylate kinase isoenzyme 6 homolog
AAEL0255	NA	0.322294108203103	0.0400792183467499	0.31441514682591	NA	NA	NA	XP_021701466.1proton-coupled amino acid transporter-like protein CG1139
AAEL0139	uncharacterized LOC5579042	0.322335707323703	0.0130368653809205	0.183852669789472	NA	NA	NA	XP_001657234.2uncharacterized protein LOC5579042
AAEL0027	zinc finger protein 62	0.32238759543806	0.330987136987661	0.754124484469288769	NA	NA	KXJ70239.1 hypothet	XP_001655897.2zinc finger protein 62
AAEL0219	NA	0.322456705205223	0.325051807542677	0.748530213465395	NA	aag03460	NA	XP_001660079.2uncharacterized protein LOC5571977
AAEL0149	abhydrolase domain-containing protein 2	0.322800802220463	0.0331236730555548	0.285726819198035	NA	NA	NA	XP_021701922.1abhydrolase domain-containing protein 2
AAEL0093	bifunctional lysine-specific demethylase and histidyl-hydroxylase NO66	0.322960063904497	0.0914591300739778	0.459288434349932	NA	NA	NA	XP_001653808.2bifunctional lysine-specific demethylase and histidyl-hydroxylase NO66
AAEL0068	mRNA turnover protein 4 homolog	0.323012956426651	0.0527903353822827	0.362633125106993	NA	NA	KXJ75567.1 hypothet	XP_001652303.1mRNA turnover protein 4 homolog
AAEL0140	NA	0.323365179333577	0.0678263067628659	0.40094231299198	NA	NA	NA	XP_001648026.2solute carrier family 35 member C2
AAEL0011	uncharacterized LOC5568567	0.323502819233653	0.436932697126338	0.820960220424486	NA	NA	XP_001853340.1 def	XP_019540255.1 peroxidasin
AAEL0017	uncharacterized LOC5572296	0.323738967290574	0.0273046370291959	0.268836665026084	NA	NA	ETN66575.1 hypothe	XP_001653984.2uncharacterized protein LOC5572296 isoform X2
AAEL0236	NA	0.323801929657327	0.288897293828004	0.721217831038796	NA	NA	NA	XP_021712454.1bis(5'-adenosyl)-triphosphatase enpp4-like
AAEL0041	40S ribosomal protein S17	0.323905884921978	0.0689527823371701	0.403354246998601	NA	NA	ETN57963.1 40S ribo	XP_001648567.140S ribosomal protein S17
AAEL0015	zinc finger protein 664	0.323914515309567	0.0235772006813761	0.249663291910268	NA	NA	KXJ69280.1 hypothet	XP_001653546.1zinc finger protein 664
AAEL0060	protein FAM8A1	0.323927443785205	0.0451943997586144	0.337290240534282	NA	NA	KXJ81603.1 hypothet	XP_001657447.2protein FAM8A1
AAEL0277	NA	0.32393429422762	0.127119432202239	0.531526770119611	NA	aag04140	NA	XP_001652417.2beclin 1-associated autophagy-related key regulator
AAEL0050	aquaporin	0.324031831623032	0.0123481302424725	0.179504888805347	NA	NA	KXJ77077.1 hypothet	ABF18403.1aquaporin 3
AAEL0028	H/ACA ribonucleoprotein complex non-core subunit NAF1	0.324078194076349	0.0358552043617131	0.299945082305895	NA	NA	KXJ73049.1 hypothet	XP_001655996.2H/ACA ribonucleoprotein complex non-core subunit NAF1
AAEL0068	uncharacterized LOC5568470	0.324123336813032	0.155351969485631	0.573091811919643	NA	NA	KXJ78898.1 hypothet	XP_001652299.1uncharacterized protein LOC5568470
AAEL0118	protein LSM14 homolog B	0.324156303945343	0.00636485109205366	0.130056109164417	NA	NA	XP_019541686.1 PRE	XP_001662014.1protein LSM14 homolog B isoform X2
AAEL0124	transmembrane protein 68	0.324275389836462	0.0999883629638345	0.479706814084761	NA	NA	KXJ77564.1 hypothet	XP_001656028.1transmembrane protein 68 isoform X1
AAEL0254	NA	0.324384233144503	0.0170521448753939	0.211759564296052	04151 ; 05165 ; 04714 ;	NA	NA	XP_021711031.1 transcription-associated protein 1
AAEL0064	GATOR complex protein WDR24	0.324501893879368	0.281729514503194	0.715107379448722	NA	aag04150	KFB43727.1 AGAP00	XP_001652000.1GATOR complex protein WDR24
AAEL0016	probable galactose-1-phosphate uridylyltransferase	0.324591668735691	0.0110826093098814	0.169687472328666	00052 ; 00520	aag01100 ; aag	KXJ77611.1 hypothet	XP_001659745.2probable galactose-1-phosphate uridylyltransferase
AAEL0254	NA	0.32474203049755	0.229253845178015	0.662487607456997	NA	NA	NA	XP_021704235.1uncharacterized protein LOC5571494
AAEL0171	NA	0.324904471290819	0.0230898556613163	0.246202609264696	NA	NA	NA	XP_021712670.1double-strand-break repair protein rad21 homolog isoform X1
AAEL0112	uncharacterized LOC5574533	0.325891581003776	0.0364891797922086	0.301874170044602	NA	NA	NA	XP_021694480.1uncharacterized protein LOC5574533
AAEL0038	dolichyldiphosphatase 1	0.325918551643956	0.0836760811207859	0.438989337784191	510	aag00510	ETN66078.1 dolichyl	XP_001664281.1dolichyldiphosphatase 1
AAEL0132	alpha-methylacyl-CoA racemase	0.326431443931722	0.253386126786549	0.685145257466747	NA	aag01100 ; aag	NA	XP_001656563.1alpha-methylacyl-CoA racemase
AAEL0072	histone deacetylase complex subunit SAP130	0.326437013491857	0.0347163807770774	0.295097377978161	NA	NA	KXJ82623.1 hypothet	XP_001652616.2histone deacetylase complex subunit SAP130
AAEL0044	iron-sulfur cluster co-chaperone protein HscB, mitochondrial	0.326519584815706	0.0735513960133999	0.417790090194633	NA	NA	KXJ76974.1 hypothet	XP_001649231.1iron-sulfur cluster co-chaperone protein HscB, mitochondrial
AAEL0107	protein transport protein Sec61 subunit alpha	0.326774078038093	0.0116350690983722	0.173701685309403	NA	aag04141 ; aag	KXJ70627.1 hypothet	XP_001661042.1protein transport protein Sec61 subunit alpha
AAEL0230	NA	0.3272508677534	0.0361900923407011	0.300966157937731	00230 ; 00030	aag01100 ; aag	NA	XP_021698341.1ribose-phosphate pyrophosphokinase 1 isoform X1
AAEL0085	putative ATP-dependent RNA helicase me31b	0.327260078404012	0.0105098377216918	0.165036735008546	NA	aag03018	ETN65039.1 DEAD bo	XP_001659287.1putative ATP-dependent RNA helicase me31b
AAEL0066	RUS1 family protein C16orf58 homolog	0.327331219947015	0.105151062585218	0.489439579842721	NA	NA	XP_001846695.1 con	XP_001652155.2RUS1 family protein C16orf58 homolog
AAEL0078	ubiquitin-conjugating enzyme E2 G2	0.32735924679585	0.0227603436587951	0.245124275357034	NA	aag04141 ; aag	ETN58426.1 ubiquiti	XP_001652937.1ubiquitin-conjugating enzyme E2 G2
AAEL0011	protein phosphatase methylesterase 1	0.327437013223286	0.104634079776741	0.488256999039335	NA	NA	KXJ71337.1 hypothet	XP_001658170.1protein phosphatase methylesterase 1
AAEL0096	uncharacterized LOC5572240	0.327458157792693	0.0354393974814579	0.298092628541477	NA	NA	NA	XP_001653921.1uncharacterized protein LOC5572240
AAEL0075	transportin-1	0.327553184381866	0.065914254248855	0.398547160129544	NA	NA	ETN63269.1 importir	XP_019536976.1 transportin-1
AAEL0220	NA	0.327995911590152	0.28672745918327	0.718895294966742	NA	NA	NA	KFB47085.1AGAP002328-like protein
AAEL0171	uncharacterized LOC23687575	0.328066771054909	0.12116057385529	0.523190802729414	NA	NA	NA	XP_019540891.1 intraflagellar transport protein 43 homolog
AAEL0015	conserved oligomeric Golgi complex subunit 6	0.328377835282975	0.0493453621083759	0.353091774588861	NA	NA	KXJ75063.1 hypothet	XP_001653603.2conserved oligomeric Golgi complex subunit 6 isoform X1
AAEL0213	NA	0.328453696248194	0.0237530066635883	0.250947379240344	NA	NA	NA	XP_021702339.1transmembrane emp24 domain-containing protein bai isoform X2
AAEL0074	proteoglycan 4	0.328476213273625	0.050354711049254	0.356983090196637	NA	NA	KXJ80218.1 hypothet	XP_001652748.1verprolin isoform X1
AAEL0061	adipocyte plasma membrane-associated protein	0.328495977537479	0.00662301706860141	0.133651322511557	NA	NA	KXJ82283.1 hypothet	XP_001657568.1adipocyte plasma membrane-associated protein isoform X1
AAEL0097	zinc finger protein 2 homolog	0.328842511936836	0.318662703726554	0.745684464362801	NA	NA	NA	XP_021699448.1zinc finger protein 2 homolog
AAEL0113	uncharacterized LOC5580159	0.328911106197329	0.0825625748995472	0.437257081788889	NA	NA	KXJ70103.1 hypothet	XP_001661632.1uncharacterized protein LOC5580159
AAEL0183	NA	0.329002622735174	0.285554858823445	0.717970179170389	NA	NA	NA	XP_021705190.1uncharacterized protein LOC5568849
AAEL0051	uncharacterized LOC5566061	0.329081677839138	0.119616790428285	0.519433807634405	NA	NA	KXJ79214.1 hypothet	XP_001650421.1uncharacterized protein LOC5566061
AAEL0059	ER lumen protein-retaining receptor 3	0.329245872642652	0.0194906599753005	0.22702918113002	NA	NA	KXJ72572.1 hypothet	XP_001651695.1ER lumen protein-retaining receptor 3
AAEL0074	chromatin accessibility complex protein 1	0.329513692907781	0.0667461325576973	0.399540230684495	NA	NA	ETN65956.1 histone-	XP_001658389.1chromatin accessibility complex protein 1
AAEL0022	uncharacterized LOC5574113	0.329874261031003	0.0673728362579753	0.4007529665455	NA	NA	KXJ69667.1 hypothet	XP_001661185.1uncharacterized protein LOC5574113
AAEL0123	copper chaperone for superoxide dismutase	0.330327394072354	0.247886071329852	0.680588844480365	NA	NA	KXJ73503.1 hypothet	XP_001662518.1copper chaperone for superoxide dismutase
AAEL0019	dentin sialophosphoprotein	0.330705130133221	0.0227523504076048	0.245124275357034	NA	NA	KXJ70275.1 hypothet	XP_001654194.2dentin sialophosphoprotein
AAEL0045	transmembrane protein 186	0.330732645078705	0.102110570673136	0.484636597025644	NA	NA	KXJ78991.1 hypothet	XP_001649335.1transmembrane protein 186
AAEL0004	chitobiosyldiphosphodolichol beta-mannosyltransferase	0.330778046572318	0.0394658641530738	0.313353264954028	00510 ; 00513	aag01100 ; aag	KXJ84208.1 hypothet	XP_001656402.1chitobiosyldiphosphodolichol beta-mannosyltransferase
AAEL0139	probable ATP-dependent RNA helicase DDX46	0.331457842800284	0.0384709102845695	0.309719436954163	NA	aag03040	NA	XP_001657248.1probable ATP-dependent RNA helicase DDX46
AAEL0022	uncharacterized LOC5574109	0.331695481593392	0.0163863314509427	0.206858269058754	NA	NA	KXJ69486.1 hypothet	XP_019933167.1 probable salivary secreted peptide
AAEL0204	NA	0.332129592384714	0.0105053832676545	0.165036735008546	NA	aag03060	NA	XP_021693781.1signal peptidase complex catalytic subunit SEC11A

AAEL01292	NA	0.332290415956379	0.391893834747892	0.793285976044306	NA	NA	NA	XP_021713014.1unconventional myosin-Ib isoform X1
AAEL00135	uncharacterized LOC5570088	0.332315695506042	0.0287222456604676	0.274601260220884	NA	NA	KXJ73796.1 hypothet	XP_001653075.1uncharacterized protein LOC5570088 isoform X2
AAEL01183	RNA-binding protein pno1	0.332424170356377	0.00934847920016108	0.158773875613038	NA	NA	ETN61285.1 RNA-bin	XP_001655746.1RNA-binding protein pno1
AAEL01109	homer protein homolog 2	0.332661406238129	0.139136836940837	0.549029662748535	NA	aag04068	XP_019543884.1 PRE	XP_021700111.1homer protein homolog 2 isoform X1
AAEL01945	NA	0.332867818665522	0.00853112031964383	0.149464458238032	NA	aag04141	NA	XP_021705785.1membrane-bound transcription factor site-1 protease isoform X1
AAEL00275	zinc finger protein 37 homolog	0.333006849361553	0.247177791555824	0.680407406434919	NA	NA	KXJ67900.1 hypothet	XP_001655892.1zinc finger protein 37 homolog
AAEL02291	NA	0.333619104966307	0.0210031227738538	0.23627229311125	NA	NA	NA	XP_021705148.1extracellular serine/threonine protein CG31145-like
AAEL00441	mimitin, mitochondrial	0.333670408128383	0.0572245313000187	0.375057077651547	NA	NA	KXJ72316.1 hypothet	XP_001649076.2mimitin, mitochondrial
AAEL01998	NA	0.333778826864137	0.112504380101148	0.503547757511443	NA	NA	NA	XP_019558833.1 protein trapped in endoderm-1 isoform X1
AAEL00793	transmembrane protein 165	0.333876089297698	0.0133033469255901	0.18599306889727	NA	NA	XP_019529274.1 PRE	XP_021710722.1transmembrane protein 165
AAEL02213	NA	0.33418266446795	0.0603235349926467	0.384941803020695	NA	NA	NA	XP_021699834.1nuclear factor related to kappa-B-binding protein isoform X3
AAEL02160	NA	0.334761829648619	0.0102073635272452	0.163069720794636	NA	NA	NA	XP_021699636.1dolichyl-diphosphooligosaccharide--protein glycosyltransferase subunit 4
AAEL01197	calmodulin	0.334818298779781	0.040506992112376	0.316225178415075	NA	NA	ETN65227.1 calmodu	XP_019530667.1 calmodulin-like
AAEL00231	gamma-tubulin complex component 6	0.335020294035742	0.080537484646423	0.433079891352508	NA	NA	ETN60468.1 hypothe	XP_001661248.1gamma-tubulin complex component 6
AAEL00325	putative protein TPRXL	0.335130847537596	0.262492969101622	0.696298732105253	NA	NA	XP_019549716.1 PRE	XP_001656590.2 TPRXL isoform X1
AAEL02110	NA	0.335326190435424	0.091275147252008	0.459220287049195	NA	NA	NA	XP_021694898.1mediator of RNA polymerase II transcription subunit 25 isoform X1
AAEL02150	NA	0.33557919382791	0.0378882156540111	0.307682856236235	NA	NA	NA	XP_021705426.1transmembrane protein 185B
AAEL02484	NA	0.335832371214053	0.00763146838521319	0.142450721693651	NA	NA	NA	XP_001657383.2coatomer subunit beta' isoform X2
AAEL00493	carbonic anhydrase	0.335990031322479	0.0230435521394947	0.246091471282416	910	aag01100 ; aag	ABF66618.1 putative	XP_001650043.1carbonic anhydrase
AAEL01986	NA	0.336201464254093	0.286591869536395	0.718895294966742	NA	NA	NA	XP_021697610.1voltage-dependent calcium channel subunit alpha-2/delta-3 isoform X3
AAEL00569	peptidyl-prolyl cis-trans isomerase sig-7	0.336838057204378	0.0405505010356215	0.316225178415075	NA	NA	KXJ73541.1 hypothet	XP_001651320.2peptidyl-prolyl cis-trans isomerase sig-7
AAEL01034	GILT-like protein 1	0.337783704655058	0.0394747333989298	0.313353264954028	NA	NA	NA	XP_001654451.1GILT-like protein 1
AAEL00336	phospholipid phosphatase 5	0.338091949457213	0.0123753305109173	0.179504888805347	00564 ; 00600 ; 00565 ;	aag00564 ; aag	KXJ67924.1 hypothet	XP_001656750.1phospholipid phosphatase 5
AAEL00142	proteasome inhibitor PI31 subunit	0.338128819555779	0.0559271568574173	0.371974308318541	NA	aag03050	XP_001841849.1 pro	XP_001659180.1proteasome inhibitor PI31 subunit
AAEL00886	protein regulator of cytokinesis 1	0.338377531199889	0.13850468606547	0.548805616375239	NA	NA	KXJ62513.1 hypothet	XP_001653481.2protein regulator of cytokinesis 1
AAEL00336	uncharacterized LOC5577998	0.338383296080446	0.277090165742391	0.712429087779124	NA	NA	KXJ76393.1 hypothet	XP_001656736.2uncharacterized protein LOC5577998
AAEL00793	cell division control protein 45 homolog	0.33849211066624	0.224346215781176	0.656544198985955	NA	NA	KXJ74145.1 hypothet	XP_001658728.1cell division control protein 45 homolog
AAEL00765	p21-activated protein kinase-interacting protein 1-like	0.338591500148176	0.122976126944161	0.525360408607322	NA	NA	KXJ69078.1 hypothet	XP_001658557.1p21-activated protein kinase-interacting protein 1-like
AAEL00309	malate synthase	0.338901373171801	0.0284180981815432	0.272967995267809	00620 ; 00630	NA	KXJ82678.1 hypothet	XP_001663102.2malate synthase
AAEL00841	uncharacterized LOC5570601	0.340091118060252	0.422142808405264	0.814192545906728	NA	NA	KXJ74981.1 hypothet	XP_021709314.1uncharacterized protein LOC5570601 isoform X2
AAEL00430	uncharacterized LOC5564558	0.340170614297199	0.254410311496829	0.686571057876479	NA	NA	KXJ69519.1 hypothet	XP_001648909.1uncharacterized protein LOC5564558
AAEL01825	NA	0.340209537725761	0.137526376663631	0.547141229310386	NA	NA	NA	XP_001663454.2DNA repair protein complementing XP-C cells homolog
AAEL00295	endochitinase	0.340614842295541	0.0567584167385668	0.374465659921115	NA	NA	XP_019538015.1 PRE	XP_019933030.1 endochitinase-like
AAEL00140	cyclin-dependent kinase 4	0.341399765384871	0.251952517035667	0.684556086619159	NA	aag04933	ETN63964.1 cell divis	XP_001659194.1cyclin-dependent kinase 4
AAEL02275	NA	0.341871424078939	0.00569044218673179	0.120826935771257	NA	aag04141	NA	XP_021697592.1protein ERGIC-53
AAEL00291	sphingomyelin phosphodiesterase 4	0.342735238109459	0.123763436179325	0.527255157278773	600	aag01100 ; aag	XP_001847782.1 con	XP_021700393.1sphingomyelin phosphodiesterase 4
AAEL01428	endothelial zinc finger protein induced by tumor necrosis factor alpha	0.343144705864045	0.333977049381856	0.756373801451385	NA	NA	NA	XP_001648351.2endothelial zinc finger protein induced by tumor necrosis factor alpha
AAEL02053	NA	0.343315991494059	0.322659732059468	0.746650319748046	NA	NA	NA	XP_021711616.1zinc finger protein 260
AAEL00364	stearoyl-CoA desaturase 5	0.343982905809002	0.0122744856150236	0.179285423221345	NA	aag01100 ; aag	KXJ72474.1 hypothet	XP_001657083.2stearoyl-CoA desaturase 5
AAEL00250	dnaJ homolog subfamily C member 16	0.344189473942302	0.0779967463260822	0.42745012137719	NA	NA	KXJ69854.1 hypothet	XP_001655444.2dnaJ homolog subfamily C member 16
AAEL02423	NA	0.344252135888674	0.297279646800785	0.729484615962886	NA	NA	NA	XP_021694121.1DNA-binding protein RFX2 isoform X8
AAEL01089	TM2 domain-containing protein CG11103	0.344320920867025	0.22823695589595	0.66175420142127	NA	NA	KFB46123.1 AGAP00	XP_001655039.1TM2 domain-containing protein CG11103
AAEL00720	nuclear hormone receptor HR96	0.344868904916002	0.102421788786399	0.484636597025644	NA	NA	KXJ74037.1 hypothet	XP_001658235.3nuclear hormone receptor HR96
AAEL02352	NA	0.345249018562419	0.252019305477938	0.684556086619159	NA	NA	NA	XP_021701403.1ATP-binding cassette sub-family C member Sur
AAEL00471	protein BTG1	0.34538611143641	0.0193971686895332	0.22702918113002	NA	aag03018	ETN60513.1 BTG1 pr	XP_021702494.1protein BTG1
AAEL00880	KAT8 regulatory NSL complex subunit 3	0.345806533839023	0.0310279937860376	0.282437393567471	NA	NA	KXJ83358.1 hypothet	XP_001659517.1KAT8 regulatory NSL complex subunit 3
AAEL00008	conserved oligomeric Golgi complex subunit 2	0.345930564618654	0.091720923185573	0.459954188094628	NA	NA	KXJ68153.1 hypothet	XP_001647944.1conserved oligomeric Golgi complex subunit 2
AAEL01396	DNA topoisomerase 2-binding protein 1-B	0.345935062071462	0.143095963343781	0.555375365647863	NA	aag03440	NA	XP_021704809.1DNA topoisomerase 2-binding protein 1-B
AAEL02472	NA	0.345997363050156	0.347314956185274	0.765755091527201	NA	NA	NA	NA
AAEL00264	uncharacterized LOC5575435	0.346329078475994	0.0567680499445725	0.374465659921115	NA	NA	KXJ71471.1 hypothet	XP_019527809.1 coiled-coil domain-containing protein 51-like
AAEL02089	NA	0.346332392996327	0.111835628805382	0.503232985949696	NA	NA	NA	---NA---
AAEL01811	NA	0.346380747288608	0.0932154459760511	0.463658666957633	NA	aag01100 ; aag	NA	XP_021712201.1fatty acid hydroxylase domain-containing protein 2 isoform X2
AAEL00912	probable cytochrome P450 6a14	0.34649277009551	0.163130378113085	0.581366301644056	NA	NA	ADY68483.1 cytochr	XP_001653671.1probable cytochrome P450 6a14
AAEL00021	ribonuclease P protein subunit p29	0.346699198453392	0.139869928622884	0.5498005481366	NA	aag03013 ; aag	XP_001842292.1 ribo	XP_001658911.2ribonuclease P protein subunit p29
AAEL00217	uncharacterized protein C1683.06c	0.347208640103101	0.167641664595129	0.587554508928878	NA	NA	KXJ78910.1 hypothet	XP_021694881.1uncharacterized protein C1683.06c
AAEL02268	NA	0.347366020350021	0.076064958140172	0.421910635808236	NA	aag01100 ; aag	NA	XP_021693979.1N-acetylgalactosaminyltransferase 7
AAEL00804	hexamerin-1.1	0.347437494024512	0.204514766753575	0.637782699712521	NA	NA	KXJ73032.1 hypothet	XP_019549144.1 hexamerin-1.1
AAEL00370	zinc finger protein 287	0.347655547143837	0.257904738855807	0.691301895412506	NA	NA	KXJ74992.1 hypothet	XP_001657161.2zinc finger protein 287
AAEL01979	NA	0.347796463290011	0.0108368228656407	0.167879535369741	NA	NA	NA	XP_021707090.1RNA-binding protein fusilli isoform X1
AAEL00999	myosin-IIIb	0.348140366949072	0.0392619775140611	0.313353264954028	04151 ; 05165 ; 04714 ;	aag04745	NA	XP_001660541.1myosin-IIIb isoform X4
AAEL00178	peroxisomal leader peptide-processing protease	0.348164379661231	0.278565329296217	0.713122992430066	NA	NA	ETN58120.1 hypothe	XP_001653957.2peroxisomal leader peptide-processing protease
AAEL02289	NA	0.348253212412302	0.0116300379869566	0.173701685309403	NA	NA	NA	XP_021698307.1solute carrier family 12 member 8
AAEL00421	monocarboxylate transporter 7	0.348459044392874	0.0142501575771941	0.19121517032758	NA	NA	KXJ83164.1 hypothet	XP_001648705.1monocarboxylate transporter 7
AAEL01003	translocon-associated protein subunit beta	0.34866035666823	0.0160152584822308	0.20383368851522	NA	aag04141	NA	XP_001660576.1translocon-associated protein subunit beta
AAEL02632	NA	0.349204106006419	0.296008985626134	0.72758553286062	NA	NA	NA	CRK89669.1CLUMA_CG003390, isoform A
AAEL00108	probable RNA 3'-terminal phosphate cyclase-like protein	0.349395881112716	0.147339420296466	0.561952643558699	NA	aag03008	KXJ69402.1 hypothet	XP_001657986.2probable RNA 3'-terminal phosphate cyclase-like protein
AAEL02599	NA	0.349529535984523	0.0136810400680443	0.187831676988403	NA	NA	NA	NA
AAEL00293	uncharacterized LOC5576593	0.349935444617799	0.0954782955323597	0.467087334124813	NA	NA	NA	XP_001656176.2uncharacterized protein LOC5576593
AAEL00436	uncharacterized family 31 glucosidase KIAA1161	0.350008404705494	0.598940773747585	0.890875851600119	NA	NA	NA	XP_001648987.2uncharacterized family 31 glucosidase KIAA1161
AAEL01408	kinesin-like protein Klp61F	0.350410702898062	0.202479865521933	0.635477395133979	NA	NA	NA	XP_001648022.2 kinesin-like protein Klp61F
AAEL01868	NA	0.350471080485303	0.231080668105778	0.66425254967488	190	aag01100 ; aag	NA	YP_009389268.1NADH dehydrogenase subunit 4 (mitochondrion)
AAEL00280	dolichyl-diphosphooligosaccharide--protein glycosyltransferase subunit S	0.350482127403078	0.00990706840748006	0.161167614967993	NA	aag01100 ; aag	KFB44137.1 hypothe	XP_001662467.1dolichyl-diphosphooligosaccharide--protein glycosyltransferase subunit STT3B
AAEL00344	SET and MYND domain-containing protein 4	0.350712081300239	0.100649724291949	0.48111031297656	NA	NA	NA	XP_021706822.1SET and MYND domain-containing protein 4
AAEL01489	estradiol 17-beta-dehydrogenase 8	0.350782659195841	0.0528283732744568	0.362633125106993	NA	aag01100 ; aag	NA	XP_001649973.1estradiol 17-beta-dehydrogenase 8
AAEL00545	tumor suppressor candidate 3	0.350989712702505	0.0117437889131709	0.174019880159419	NA	aag01100 ; aag	KXJ82352.1 hypothet	XP_001650913.1tumor suppressor candidate 3
AAEL00542	probable 2-oxoglutarate dehydrogenase E1 component DHKTD1 homolog	0.351497281942304	0.0265220092301948	0.266145614979556	00020 ; 00310 ; 00380	aag01100 ; aag	KXJ79339.1 hypothet	XP_001650884.2probable 2-oxoglutarate dehydrogenase E1 component DHKTD1 homolog, mitochondrial
AAEL01138	protein shifted	0.351541585532578	0.0952620556698354	0.466773927728342	NA	aag04310	KXJ79675.1 hypothet	XP_001655312.1protein shifted isoform X2
AAEL02753	NA	0.351661521929351	0.0260565159827887	0.262907960607041	NA	NA	NA	XP_021693515.1uncharacterized protein LOC110674122
AAEL00643	probable dolichyl pyrophosphate Man9GlcNAc2 alpha-1,3-glucosyltransf	0.352166628110797	0.0560788282035141	0.372590163991868	510	aag01100 ; aag	KXJ69167.1 hypothet	XP_001657824.1probable dolichyl pyrophosphate Man9GlcNAc2 alpha-1,3-glucosyltransferase
AAEL00806	transmembrane and coiled-coil domains protein 2	0.352313273296424	0.0599754100865979	0.383636203860763	NA	NA	NA	XP_021700012.1transmembrane and coiled-coil domains protein 2 isoform X1
AAEL02186	NA	0.352434304906708	0.00627636291253989	0.129262654794788	NA	NA	NA	XP_021710177.1probable cytochrome P450 28a5
AAEL01272	chromatin assembly factor 1 subunit A-A	0.352614418609175	0.125962442097107	0.530481545423179	NA	NA	KXJ69338.1 hypothet	XP_021710918.1chromatin assembly factor 1 subunit A-A

AAEL0172!	integumentary mucin C.1-like	0.352941846913069	0.176706871671113	0.601574781027594	NA	NA	NA	XP_011493076.1integumentary mucin C.1 isoform X1
AAEL0083؛	uncharacterized LOC5570430	0.35302461737099	0.213226421061513	0.646373717545434	NA	NA	NA	XP_021700795.1uncharacterized protein LOC5570430 isoform X1
AAEL0262	NA	0.35306257219216	0.0674654179149415	0.400828276414654	NA	NA	NA	XP_021712546.1uncharacterized protein LOC5576059
AAEL0031	uncharacterized LOC5577234	0.353266357346386	0.166931986873894	0.586748717805031	NA	NA	KXJ78211.1 hypothe	XP_021707247.1uncharacterized protein LOC5577234
AAEL0081	uncharacterized LOC5570144	0.353553555661679	0.233173244768923	0.666962220912214	NA	NA	KXJ70169.1 hypothe	XP_001653127.1uncharacterized protein LOC5570144
AAEL0096	rhomboid-related protein 2	0.354031020060188	0.0727579097288087	0.414305869631496	NA	NA	NA	XP_001653937.1rhomboid-related protein 2 isoform X2
AAEL0067	NA	0.354057276412163	0.115958080779367	0.512764772622422	04151 ; 05165 ; 04714 ;	NA	ETN65350.1 serine/t	XP_001657974.1 aurora kinase B
AAEL0099	zinc finger protein 62 homolog	0.35416383566922	0.384497146730162	0.790080064171371	NA	NA	NA	XP_001654106.2zinc finger protein 62 homolog
AAEL0018	carboxypeptidase B	0.354495692640943	0.0471904305895132	0.346180765881814	NA	NA	AAT36736.1 carboxy	XP_001654133.2carboxypeptidase B
AAEL0211	NA	0.354741181524451	0.0675094700864258	0.400828276414654	NA	NA	NA	XP_019540390.1 mantle protein-like
AAEL0131	dnaJ homolog subfamily C member 25 homolog	0.35476026479265	0.0520636189356094	0.361717594818022	NA	NA	NA	XP_001656359.2dnaJ homolog subfamily C member 25 homolog isoform X2
AAEL0069	mediator of RNA polymerase II transcription subunit 19	0.354856823034867	0.0383459693382474	0.309719436954163	NA	NA	ETN60723.1 mediato	XP_001652347.1mediator of RNA polymerase II transcription subunit 19 isoform X2
AAEL0096	RNA 3'-terminal phosphate cyclase	0.354898837457612	0.318011152157394	0.745684464362801	NA	NA	NA	XP_001653847.2RNA 3'-terminal phosphate cyclase
AAEL0010	3-ketodihydrosphingosine reductase	0.354980535429651	0.0474377453985041	0.347549469074072	NA	aag01100 ; aag	KXJ67985.1 hypothe	XP_001657877.23-ketodihydrosphingosine reductase
AAEL0001	uncharacterized protein C7orf26 homolog	0.355165185846787	0.306154104374021	0.738783696372872	NA	NA	NA	ABF18189.1conserved protein
AAEL0223	NA	0.35592645898091	0.319517823627017	0.745684464362801	NA	NA	NA	XP_021707049.1uncharacterized protein LOC5571112 isoform X1
AAEL0021	45 kDa calcium-binding protein	0.356026255259397	0.204601852292267	0.637782699712521	NA	NA	KXJ75503.1 hypothe	XP_001654735.145 kDa calcium-binding protein
AAEL0076	rRNA-processing protein FCF1 homolog	0.356044513488652	0.0523828190708132	0.362633125106993	NA	aag03008	XP_001844058.1 rRN	XP_001652823.2rRNA-processing protein FCF1 homolog
AAEL0059	probable multidrug resistance-associated protein lethal(2)03659	0.356213693404525	0.0109136684082779	0.168274304687137	NA	NA	XP_019545884.1 PRE	XP_021706784.1probable multidrug resistance-associated protein lethal(2)03659 isoform X2
AAEL0203	NA	0.356229731353368	0.349774361394 83	0.767801448844281	NA	NA	NA	XP_021698399.1uncharacterized protein LOC110676114 isoform X3
AAEL0256	NA	0.356382714159598	0.0342466938978285	0.292607314064826	NA	NA	NA	XP_021701725.1otopetrin-2 isoform X3
AAEL0257	NA	0.357067302124102	0.0779022527620417	0.42745012137719	NA	NA	NA	NA
AAEL0144	uncharacterized LOC5564522	0.357419042839241	0.252694803407234	0.684918285995101	NA	NA	NA	XP_001648864.1uncharacterized protein LOC5564522
AAEL0085	transcription factor grauzone	0.357504621355703	0.256279136019349	0.688461255220739	NA	NA	NA	XP_021693802.1transcription factor grauzone
AAEL0024	zinc finger and BTB domain-containing protein 49	0.357531150495116	0.227822402425386	0.66175420142127	NA	NA	KXJ69472.1 hypothe	XP_001655440.2zinc finger and BTB domain-containing protein 49
AAEL0025	methionine-R-sulfoxide reductase B1,structural maintenance of chromos	0.358206094918019	0.101765893320696	0.483848092317719	NA	NA	XP_019533564.1 PRE	XP_021709547.1structural maintenance of chromosomes protein 6
AAEL0041	DDB1- and CUL4-associated factor 12	0.358736533053211	0.288768467403372	0.721217831038796	NA	NA	KFB42361.1 AGAP00	XP_001648513.1DDB1- and CUL4-associated factor 12
AAEL0269	NA	0.358959777050437	0.0249686212718444	0.258627500477641	NA	NA	NA	XP_019565646.1 C-type lectin 37Da-like
AAEL0079	serine/threonine-protein kinase NIM1	0.358982353119236	0.128186812298849	0.53471216988849	NA	NA	KXJ82596.1 hypothe	XP_021695401.1serine/threonine-protein kinase NIM1
AAEL0262	NA	0.359495370419384	0.0244136743548197	0.255291093250631	NA	aag01100 ; aag	NA	XP_021706597.1 alanine--glyoxylate aminotransferase 2, mitochondrial
AAEL0109	uncharacterized LOC5574072	0.360116180103955	0.0806909820156655	0.433079891352508	NA	NA	ETN59097.1 hypothe	XP_001661145.2uncharacterized protein LOC5574072
AAEL0063	epoxide hydrolase 4	0.360913838832358	0.334691431357124	0.757087156181969	NA	NA	KXJ73838.1 hypothe	XP_001651933.1epoxide hydrolase 4
AAEL0052	methionyl-tRNA formyltransferase, mitochondrial	0.360926898109251	0.214406274324679	0.648028428468258	00970 ; 00670	aag00970 ; aag	KXJ83424.1 hypothe	XP_001650620.1methionyl-tRNA formyltransferase, mitochondrial
AAEL0051	uncharacterized LOC5566043	0.361178951357886	0.199808662187664	0.633375309392567	NA	NA	KXJ78830.1 hypothe	XP_001650418.1uncharacterized protein LOC5566043
AAEL0079	protein FAM151B	0.361307158866516	0.0265165061752151	0.266145614979556	NA	NA	XP_019550350.1 PRE	XP_001652988.2protein FAM151B isoform X2
AAEL0066	WD repeat-containing protein 75	0.361350852738654	0.0208684279460986	0.235621195042944	NA	aag03008	KXJ71008.1 hypothe	XP_001652102.2WD repeat-containing protein 75
AAEL0259	NA	0.361778108567146	0.409406726510337	0.805356078752463	NA	NA	NA	XP_021697462.1 centrosomal protein of 290 kDa
AAEL0077	cytochrome P450 4d1	0.361822811544998	0.0708336086580704	0.409428936477113	NA	NA	XP_001237479.3 AG	XP_001652930.1cytochrome P450 4d1
AAEL0058	multivesicular body subunit 12A	0.362340971517928	0.0607824863930744	0.384941803020695	NA	aag04144	KFB43991.1 AGAP00	XP_001651512.1 multivesicular body subunit 12A
AAEL0075	cellular tumor antigen p53	0.362583154017757	0.0362389157786357	0.300966157937731	NA	NA	XP_019542011.1 PRE	XP_021694051.1cellular tumor antigen p53 isoform X1
AAEL0224	NA	0.362812968393719	0.340007250580087	0.761379832492257	NA	NA	NA	XP_021699288.1zinc finger protein 25 isoform X1
AAEL0142	INO80 complex subunit B	0.363164521836936	0.0882608064387468	0.454083910037829	NA	NA	NA	XP_001648253.1INO80 complex subunit B
AAEL0126	deoxyuridine 5'-triphosphate nucleotidohydrolase	0.363179383020877	0.141287152367174	0.552330595273689	00240 ; 00983	aag01100 ; aag	XP_001847068.1 deo	XP_001662757.1deoxyuridine 5'-triphosphate nucleotidohydrolase
AAEL0203	NA	0.363843066584813	0.135336714521635	0.543887965700835	NA	NA	NA	XP_021709713.1protein AF-9
AAEL0199	NA	0.364354861101516	0.129980285957413	0.536965027274516	NA	NA	NA	XP_021706345.1oocyte zinc finger protein XlCOF28
AAEL0027	irregular chiasm C-roughest protein	0.364794074763746	0.35033974214125	0.768176246660341	NA	NA	KXJ82106.1 hypothe	XP_021703710.1irregular chiasm C-roughest protein
AAEL0108	protein arginine N-methyltransferase 1	0.365021746098346	0.0170230274659005	0.21168364694759	NA	NA	KXJ74021.1 hypothe	XP_021704596.1protein arginine N-methyltransferase 1
AAEL0025	uncharacterized LOC5575336	0.365285918307954	0.166164753287582	0.584439853413553	NA	NA	XP_001863143.1 con	ABF18392.1possible HHH motif antimicrobial peptide
AAEL0114	mitochondrial import inner membrane translocase subunit Tim29	0.365389158123705	0.0227984176389395	0.245124275357034	NA	NA	KFB40802.1 AGAP00	XP_001655397.1mitochondrial import inner membrane translocase subunit Tim29
AAEL0085	NA	0.365451088277662	0.00445259050274601	0.103991720320479	NA	aag04120	XP_001688635.1 AG	XP_001659332.1ubiquitin-conjugating enzyme E2 W isoform X1
AAEL0181	NA	0.365465657321655	0.0678403804964696	0.40094231299198	510	aag01100 ; aag	NA	XP_001652565.2dolichol kinase isoform X1
AAEL0134	selenoprotein F	0.36583496272279	0.0216147490716016	0.240216088112171	NA	NA	NA	XP_001663680.2selenoprotein F
AAEL0103	probable tRNA (guanine(26)-N(2))-dimethyltransferase	0.365917342955085	0.0680911673728758	0.401232883533236	NA	NA	NA	XP_001654461.1probable tRNA (guanine(26)-N(2))-dimethyltransferase
AAEL0141	serine protease inhibitor 28Dc	0.366573665476535	0.0606923340239614	0.384941803020695	NA	NA	NA	XP_001648092.1serine protease inhibitor 28Dc
AAEL0024	lethal(2)neighbour of tid protein 2	0.36658391683562	0.0196289581304261	0.227335564593981	00510 ; 00513	aag01100 ; aag	KXJ82472.1 hypothe	XP_001655350.2lethal(2)neighbour of tid protein 2
AAEL0029	probable dolichyl pyrophosphate Glc1Man9GlcNAc2 alpha-1,3-glucosyltr	0.366612521929368	0.143638161325291	0.555843555723525	510	aag01100 ; aag	KXJ73443.1 hypothe	XP_001662923.2probable dolichyl pyrophosphate Glc1Man9GlcNAc2 alpha-1,3-glucosyltransferase
AAEL0125	tyrosine-protein kinase hopscotch	0.366637225902797	0.00815008749303247	0.145627738554632	NA	aag04933	AAQ18517.1 jak kina	XP_001662694.2tyrosine-protein kinase hopscotch
AAEL0236	NA	0.366840781508967	0.00944377332223647	0.158773875613038	NA	NA	NA	NA
AAEL0200	NA	0.367115237016855	0.152648772851455	0.568113296551786	NA	NA	NA	XP_021703668.1zinc finger protein 2-like
AAEL0025	band 4.1-like protein 4	0.367221735824828	0.280157035233762	0.71432669388226	NA	NA	NA	XP_021692922.1band 4.1-like protein 4 isoform X5
AAEL0065	DNA replication complex GINS protein SLD5	0.367222541722558	0.0626601691258839	0.391445265645882	NA	NA	KXJ76508.1 hypothe	XP_001651996.1DNA replication complex GINS protein SLD5
AAEL0144	D-aminoacyl-tRNA deacylase	0.367498212178452	0.0893556029532527	0.457059621109412	NA	NA	NA	XP_019527818.1 D-tyrosyl-tRNA(Tyr) deacylase 1-like
AAEL0071	proton-coupled amino acid transporter-like protein CG1139	0.367965539482428	0.00797861210495883	0.144528409372715	NA	NA	KXJ81293.1 hypothe	XP_021701465.1proton-coupled amino acid transporter-like protein CG1139
AAEL0115	NA	0.36837052944265	0.0978901058174535	0.473599304703091	NA	NA	XP_001861544.1 tRN	XP_001661731.2mitochondrial ribonuclease P protein 1 homolog
AAEL0271	NA	0.368668258977622	0.00966212919133349	0.159911713702609	00250 ; 00230	aag01100 ; aag	NA	XP_021694181.1adenylosuccinate lyase
AAEL0078	augmin complex subunit dgt6	0.368738707424743	0.246433420722044	0.679999107601274	NA	NA	XP_001862015.1 con	XP_001652911.1augmin complex subunit dgt6
AAEL0110	activating signal cointegrator 1 complex subunit 1	0.368827483466667	0.0585371759765332	0.37907043866014	NA	NA	XP_001848897.1 con	XP_001661326.1activating signal cointegrator 1 complex subunit 1
AAEL0058	probable low-specificity L-threonine aldolase 2	0.368943782550389	0.0222777651476719	0.242316778828459	NA	aag01100 ; aag	KXJ77888.1 hypothe	XP_001651472.2probable low-specificity L-threonine aldolase 2
AAEL0016	6-pyruvoyl tetrahydrobiopterin synthase	0.369352762257804	0.0181125833613647	0.217871885086637	790	aag01100 ; aag	KFB39192.1 AGAP00	XP_001653732.16-pyruvoyl tetrahydrobiopterin synthase
AAEL0010	SAYSvFN domain-containing protein 1	0.369395040232398	0.0904495060712086	0.458582874647784	NA	NA	ETN58662.1 hypothe	XP_001657996.1SAYSvFN domain-containing protein 1
AAEL0028	uncharacterized LOC5576229	0.369469523854805	0.129250105061009	0.53537966541735	NA	NA	KXJ78078.1 hypothe	XP_001656007.2uncharacterized protein LOC5576229
AAEL0113	hepatocyte nuclear factor 4-gamma	0.369537355634295	0.00384893832201423	0.0960041238111189	NA	NA	XP_019532072.1 PRE	XP_021700064.1transcription factor HNF-4 homolog isoform X2
AAEL0224	NA	0.369538964489866	0.136540226542341	0.545589928345625	NA	NA	NA	NA
AAEL0129	glycine cleavage system H protein, mitochondrial	0.369601977011662	0.0400391333823447	0.31441514682591	NA	aag01100 ; aag	NA	XP_021697917.1glycine cleavage system H protein, mitochondrial
AAEL0232	NA	0.369610050866331	0.236508091947819	0.670870063357945	230	NA	NA	XP_001654573.22-oxo-4-hydroxy-4-carboxy-5-ureidoimidazoline decarboxylase
AAEL0086	protein AF-10	0.369644539849501	0.0222570498589247	0.242316778828459	NA	NA	NA	XP_021709087.1protein AF-10 isoform X1
AAEL0043	elongator complex protein 4	0.36971105089267	0.200832931949246	0.634514122727206	NA	NA	ETN57760.1 pax neig	XP_001648941.1elongator complex protein 4
AAEL0114	alpha-tocopherol transfer protein-like	0.369737383931924	0.0547130900363786	0.367496244171354	NA	NA	KXJ75163.1 hypothe	XP_001661668.2alpha-tocopherol transfer protein-like
AAEL0065	E3 ubiquitin-protein ligase RNF185	0.370047902858927	0.00641001743479753	0.130497744325236	NA	aag04141	XP_001841858.1 RIN	XP_001652037.1E3 ubiquitin-protein ligase RNF185
AAEL0032	transmembrane protein 242	0.37018793326575	0.149952935295732	0.565429182817541	NA	NA	KXJ68722.1 hypothe	XP_001656613.1transmembrane protein 242
AAEL0087	survival motor neuron protein	0.370191258323404	0.0545628837266605	0.367496244171354	NA	aag03013	KXJ72701.1 hypothe	XP_001659432.1survival motor neuron protein
AAEL0120	centrosomal protein of 152 kDa	0.370200239858195	0.165046924652089	0.583914571568059	NA	NA	KXJ68546.1 hypothe	XP_001655828.1centrosomal protein of 152 kDa

AAEL02561	NA	0.370421483295997	0.00348872793404567	0.0906120951064792	NA	NA	NA	NA
AAEL00957	probable U3 small nucleolar RNA-associated protein 11	0.370429165459133	0.107112345658115	0.492398491132955	NA	NA	NA	XP_001660244.1probable U3 small nucleolar RNA-associated protein 11
AAEL01503	palmitoyl-protein thioesterase 1	0.370790976277271	0.0191128895701138	0.225483089518189	62	aag01100 ; aag	NA	XP_001650360.2palmitoyl-protein thioesterase 1
AAEL00416	syntaxin-6	0.370991770943441	0.0224461227118247	0.243572194804494	NA	aag04130	XP_019561835.1 PRE	XP_001648589.2syntaxin-6 isoform X1
AAEL00432	origin recognition complex subunit 3	0.371019195718482	0.299358956060243	0.730496185008314	NA	NA	KXJ73230.1 hypothet	XP_021707551.1origin recognition complex subunit 3
AAEL00429	glucose-6-phosphatase 3	0.371259089590124	0.240837114184021	0.675511149976115	00052 ; 00010 ; 00500	aag01100 ; aag	KXJ82121.1 hypothet	XP_001648840.1glucose-6-phosphatase 3 isoform X1
AAEL00849	CKLF-like MARVEL transmembrane domain-containing protein 4	0.371688681184822	0.0488462852834531	0.351987741182022	NA	NA	ETN65036.1 hypothe	XP_001659283.1CKLF-like MARVEL transmembrane domain-containing protein 4
AAEL01332	translocon-associated protein subunit delta	0.371959591901989	0.011251973285281	0.171994448789296	NA	aag04141	NA	XP_001663469.1translocon-associated protein subunit delta
AAEL00872	ATP-dependent RNA helicase DDX24	0.372243027911455	0.0691004854015004	0.40372232804102	NA	NA	KXJ77346.1 hypothet	XP_001653437.1ATP-dependent RNA helicase DDX24
AAEL01116	anaphase-promoting complex subunit 7	0.37243983636128	0.0618686670050055	0.387817080231649	NA	aag04120	ETN64398.1 anaphas	XP_001661450.1anaphase-promoting complex subunit 7
AAEL01464	peptidoglycan-recognition protein LC	0.37256787257629	0.00354521247475977	0.091238621981258	NA	NA	NA	XP_021698615.1peptidoglycan-recognition protein LC isoform X3
AAEL00825	cyclin-J	0.372622776989981	0.484318574491033	0.847655581875201	NA	NA	KXJ75396.1 hypothet	XP_019543485.1 cyclin-J
AAEL00285	N-alpha-acetyltransferase 40	0.37278885075696	0.315751917847675	0.745684464362801	NA	NA	KXJ70207.1 hypothet	XP_021700664.1N-alpha-acetyltransferase 40
AAEL00054	cholinesterase 2	0.372791677907528	0.011443420284659	0.172833431546296	NA	NA	XP_001866816.1 cho	XP_001647975.3cholinesterase 2
AAEL00798	trafficking protein particle complex subunit 3	0.372889669152072	0.0439334992793758	0.331645660679914	NA	NA	XP_019527210.1 PRE	XP_001658790.2trafficking protein particle complex subunit 3 isoform X2
AAEL01158	E3 ubiquitin-protein ligase RNF181	0.373031402150597	0.367528787367619	0.781062332876865	NA	NA	XP_001865645.1 HSF	XP_001661757.2E3 ubiquitin-protein ligase RNF181
AAEL00849	calmodulin-lysine N-methyltransferase	0.37318606735775	0.136307811385169	0.545589928345625	310	aag01100 ; aag	KXJ73094.1 hypothet	XP_021702713.1calmodulin-lysine N-methyltransferase
AAEL01486	zinc finger protein 708	0.374369727024918	0.133664097451532	0.542078900286029	NA	NA	NA	XP_001649861.2zinc finger protein 708
AAEL01282	bifunctional purine biosynthesis protein PURH	0.374543271202526	0.0106685731329512	0.166147088221529	00230 ; 00670	aag01100 ; aag	XP_001844127.1 bifu	XP_001662952.2bifunctional purine biosynthesis protein PURH
AAEL01235	perlucin-like protein	0.374570955902691	0.247296499235634	0.680509086712412	NA	NA	KXJ73227.1 hypothet	XP_001662503.1perlucin-like protein
AAEL00309	calmodulin-binding transcription activator 1	0.374816694566395	0.0438486981554217	0.331489380583096	NA	NA	NA	XP_021696363.1calmodulin-binding transcription activator 1 isoform X6
AAEL01754	U4/U6 small nuclear ribonucleoprotein Prp31	0.375044572601463	0.0535890558587174	0.365820839771452	NA	aag03040	NA	XP_011493441.1U4/U6 small nuclear ribonucleoprotein Prp31
AAEL01008	COMM domain-containing protein 3	0.375129961853367	0.14343848555652	0.555756186985725	NA	NA	NA	XP_001660625.1COMM domain-containing protein 3
AAEL00443	C-Myc-binding protein	0.375245975936667	0.00710302307213827	0.137861850831157	NA	NA	KXJ74887.1 hypothet	XP_001649117.1C-Myc-binding protein
AAEL00631	DNA-binding protein SMUBP-2	0.375335605731545	0.238930391535388	0.673602163881324	NA	NA	KXJ82562.1 hypothet	XP_001651893.2DNA-binding protein SMUBP-2
AAEL01013	uncharacterized LOC5572919	0.376034438718129	0.0541528578950533	0.367217832240442	NA	NA	NA	KFB43068.1AGAP008874-like protein
AAEL02344	NA	0.376443800677133	0.00469770445287751	0.108001438767828	NA	NA	NA	XP_001650384.2zinc finger protein GLI2
AAEL01381	splicing factor 3A subunit 1	0.377049455448199	0.0458784187733844	0.340510283013877	NA	aag03040	NA	XP_001664008.2splicing factor 3A subunit 1
AAEL00995	apolipophorins	0.377326157247716	0.0151937429748679	0.198907702459398	NA	NA	NA	XP_019527837.1 apolipophorins-like
AAEL01316	NA	0.377416258018643	0.143922953179059	0.556228061803319	NA	NA	NA	XP_001663348.2uncharacterized protein YER152C
AAEL00934	ras-related protein Rab-30	0.37741744012906	0.0666327057868338	0.399540230684495	NA	NA	NA	XP_001653797.2 ras-related protein Rab-30
AAEL00520	UPF0553 protein C9orf64	0.377764714630809	0.228468520211778	0.66216293637442	NA	NA	KXJ80788.1 hypothet	XP_001650487.2UPF0553 protein C9orf64
AAEL01354	uncharacterized LOC5578152	0.377839446776492	0.275734697227725	0.711001645592863	NA	NA	NA	XP_001663724.2uncharacterized protein LOC5578152
AAEL00024	alpha-(1,3)-fucosyltransferase C	0.378134306532243	0.0581134261017752	0.377750712592071	NA	aag01100 ; aag	KXJ77548.1 hypothet	XP_001660128.1alpha-(1,3)-fucosyltransferase C
AAEL00810	uncharacterized LOC5570125	0.378455891505225	0.160553687782423	0.577674739374419	NA	NA	KXJ71624.1 hypothet	XP_021694879.1uncharacterized protein LOC5570125 isoform X2
AAEL00691	Golgi apparatus protein 1	0.379044325525076	0.00227320963206381	0.0747074108366112	NA	NA	XP_019546014.1 PRE	XP_001652319.2Golgi apparatus protein 1 isoform X2
AAEL01386	arginine--tRNA ligase, cytoplasmic	0.380495675830358	0.0918106197681078	0.46005268034546	970	aag00970	NA	XP_001664040.1arginine--tRNA ligase, cytoplasmic isoform X2
AAEL00724	uncharacterized LOC5568929	0.380876252589725	0.0193452752464284	0.226893947646744	NA	NA	NA	XP_021703188.1uncharacterized protein LOC5568929 isoform X6
AAEL00617	UPF0545 protein C22orf39 homolog	0.381016792873339	0.0591594194627475	0.381093648037575	NA	NA	XP_001843122.1 con	XP_001651814.1UPF0545 protein C22orf39 homolog isoform X1
AAEL00475	NA	0.381021009130312	0.0255621047555677	0.260925069284669	NA	NA	NA	XP_001649820.2probable chitinase 10
AAEL00327	homeobox protein GBX-2	0.38139465638439	0.225189393237825	0.658047884618818	NA	NA	KXJ70854.1 hypothet	XP_021693837.1homeobox protein GBX-2
AAEL02611	NA	0.381940762326039	0.29854139444168	0.730438157844279	NA	NA	NA	XP_021698339.1tubulin alpha-8 chain-like
AAEL02696	NA	0.381942450363368	0.0141177567379149	0.190486213346471	NA	NA	NA	XP_021710704.1protein RFT1 homolog
AAEL00308	augmin complex subunit dgt5	0.382087233769337	0.156401057886499	0.574474189691589	NA	NA	KXJ83175.1 hypothet	XP_001663091.2augmin complex subunit dgt5
AAEL02645	NA	0.382156640104746	0.301087982622911	0.733161661578971	NA	NA	NA	XP_021693816.1aminopeptidase N
AAEL00528	E3 ubiquitin-protein ligase KCMF1	0.382204233600959	0.233767681940854	0.667224010303889	NA	NA	XP_019525280.1 PRE	XP_001650701.1E3 ubiquitin-protein ligase KCMF1
AAEL00735	uncharacterized LOC5569090	0.383028676870116	0.0478832988393118	0.348612005978262	NA	NA	KXJ71821.1 hypothet	XP_001658321.2uncharacterized protein LOC5569090
AAEL02803	NA	0.383384011363934	0.14559927316167	0.558950568057442	NA	NA	NA	XP_021705778.1glutenin, high molecular weight subunit DX5 isoform X2
AAEL00708	methyltransferase-like protein	0.384212117481741	0.00303516695178952	0.0867379077340594	NA	NA	KXJ81035.1 hypothet	XP_001658128.2methyltransferase-like protein isoform X2
AAEL02015	NA	0.38450942152904	0.03862430078875	0.31014032797389	NA	NA	NA	XP_021710730.1protein cramped
AAEL01026	uncharacterized LOC5573076	0.384639847643589	0.355267854724002	0.772799644918615	NA	NA	NA	XP_001660707.1uncharacterized protein LOC5573076
AAEL00210	ribosomal RNA small subunit methyltransferase NEP1	0.385427715329466	0.0304347781757042	0.281224109837052	NA	aag03008	KXJ75505.1 hypothet	XP_001654738.1ribosomal RNA small subunit methyltransferase NEP1
AAEL02119	NA	0.38561242085275	0.373903519378169	0.782198815065306	NA	NA	NA	XP_019542927.1 nicalin-1
AAEL02651	NA	0.385663218788726	0.276924266919485	0.712300862136256	NA	NA	NA	XP_021707430.1zinc finger protein 189-like isoform X2
AAEL01494	CAAX prenyl protease 2	0.385724465554989	0.116448016522327	0.514181692916724	NA	aag00900	NA	XP_001650063.2CAAX prenyl protease 2
AAEL01175	NA	0.385830179158456	0.157717698499964	0.575235141338353	NA	NA	NA	XP_001655660.2uncharacterized protein LOC5575294
AAEL01815	NA	0.385843398591	0.0838168453766324	0.43947727131383	NA	NA	NA	XP_001653236.2peptide transporter family 1
AAEL01088	netrin receptor unc-5	0.386359416371149	0.121789409614167	0.523911973275319	NA	NA	ETN61279.1 netrin re	XP_021701483.1netrin receptor unc-5
AAEL01851	NA	0.386954165173393	0.125024009942119	0.529926733987739	NA	NA	NA	NA
AAEL00662	chromatin complexes subunit BAP18	0.387583570037387	0.132595834308975	0.540110467949322	NA	NA	NA	XP_001652100.2chromatin complexes subunit BAP18 isoform X3
AAEL02497	NA	0.387875130758151	0.00241646053137171	0.0760003211995279	NA	NA	NA	XP_021695723.1sulfhydryl oxidase 1
AAEL00514	WW domain-binding protein 4	0.388125741964804	0.178328128400026	0.603965932107854	NA	NA	KXJ82908.1 hypothet	XP_001650420.1WW domain-binding protein 4
AAEL01133	uncharacterized LOC5574708	0.388174886853598	0.12649994346123	0.530481545423179	NA	NA	NA	XP_001661608.2uncharacterized protein LOC5574708 isoform X1
AAEL00371	leucine-rich repeat-containing G-protein coupled receptor 5	0.388246396384755	0.0527296542227655	0.362633125106993	NA	NA	ETN60620.1 leucine-r	XP_001664063.1leucine-rich repeat-containing G-protein coupled receptor 5
AAEL00386	zinc finger protein chinmo	0.38878473855549	0.178911626476679	0.604350349136232	NA	NA	XP_019526561.1 PRE	XP_021701436.1zinc finger protein chinmo isoform X4
AAEL00905	peroxiredoxin-6	0.389215105797575	0.0293844270839977	0.277044567650561	480	aag01100	KXJ78903.1 hypothet	XP_019562615.1 peroxiredoxin-6
AAEL02065	NA	0.389286784203146	0.366349231186551	0.780420537752928	NA	NA	NA	XP_021705597.1uncharacterized protein LOC5570969
AAEL00164	uncharacterized LOC5571667	0.389363828802045	0.00346912870327327	0.0904332077266873	NA	NA	NA	XP_001653754.2uncharacterized protein LOC5571667 isoform X2
AAEL01212	NA	0.389594311967757	0.00266487598798006	0.0792437883464128	NA	NA	KXJ80698.1 hypothet	XP_001662263.2FAD-dependent oxidoreductase domain-containing protein 1
AAEL01431	bladder cancer-associated protein	0.390151918176491	0.0268564462546752	0.267459976661819	NA	NA	NA	XP_021696522.1bladder cancer-associated protein
AAEL02504	NA	0.390297494709518	0.157210553334338	0.574474189691589	NA	NA	NA	XP_021711855.1Werner syndrome ATP-dependent helicase
AAEL01118	putative U5 small nuclear ribonucleoprotein 200 kDa helicase	0.390411834040768	0.0379590020871275	0.307682856236235	NA	aag03040	XP_001863460.1 pre	XP_021708213.1putative U5 small nuclear ribonucleoprotein 200 kDa helicase
AAEL00712	GATA zinc finger domain-containing protein 7	0.390963735651565	0.224067862709535	0.656259917301973	NA	NA	KXJ76255.1 hypothet	XP_021705790.1GATA zinc finger domain-containing protein 7
AAEL01211	nucleolin	0.391433128557233	0.00207699740156373	0.0715825096973388	NA	NA	XP_001866439.1 hyp	XP_019533302.1 X-linked retinitis pigmentosa GTPase regulator-interacting protein 1-like
AAEL00804	bleomycin hydrolase	0.391502923745197	0.00244469626983919	0.0760003211995279	NA	NA	XP_019537325.1 PRE	XP_001658844.1bleomycin hydrolase isoform X2
AAEL00369	PHD finger-like domain-containing protein 5A	0.391606171427886	0.0502433454373245	0.356468207181388	NA	aag03040	XP_001599584.1 PRE	XP_001657126.1PHD finger-like domain-containing protein 5A
AAEL00841	NF-kappa-B-repressing factor	0.391756551925577	0.0476316625332942	0.348415388419216	NA	NA	KXJ74982.1 hypothet	XP_001653266.2NF-kappa-B-repressing factor
AAEL02237	NA	0.392109658449514	0.184849852570845	0.612086485554846	NA	NA	NA	NA
AAEL01815	NA	0.392289410501433	0.254572967775978	0.686571057876479	NA	NA	NA	XP_021700177.1 trichohyalin
AAEL00795	glutathione S-transferase E14	0.39232654673361	0.183398107852037	0.610828860145329	NA	aag01100 ; aag	KXJ68754.1 hypothet	XP_001658748.2glutathione S-transferase E14
AAEL00106	uncharacterized LOC5568348	0.39281717904317	0.142451362892465	0.554732123210059	NA	NA	XP_001844256.1 con	XP_021693952.1uncharacterized protein LOC5568348
AAEL00431	terminal uridylyltransferase Tailor	0.394013040058787	0.00266959078324147	0.0792437883464128	NA	NA	KXJ74717.1 hypothet	XP_001648904.1terminal uridylyltransferase Tailor

AAEL00748	GDP-fucose transporter 1	0.394304193627988	0.163643418425663	0.581632574875608	NA	NA	KXJ77423.1 hypothet	XP_001652791.2GDP-fucose transporter 1
AAEL00620	uncharacterized LOC5567615	0.394767989730515	0.148466153240488	0.562679383080301	NA	NA	ETN62462.1 hypothe	XP_021713393.1uncharacterized protein LOC5567615
AAEL01987	NA	0.394803109231812	0.154485602424084	0.571373196746952	NA	NA	NA	XP_021710277.1activating signal cointegrator 1 complex subunit 2-like
AAEL01245	uncharacterized LOC5576303	0.394996721866737	0.188345993794643	0.615250207631631	NA	NA	KXJ84387.1 hypothet	XP_001656043.2uncharacterized protein LOC5576303
AAEL01957	NA	0.395311086850029	0.296323949421823	0.728110275722194	NA	NA	NA	XP_021700367.1 discoidin domain-containing receptor 2
AAEL01811	NA	0.395443895638287	0.220007662109811	0.652412844728166	NA	NA	NA	XP_001657699.2zinc finger protein 665
AAEL01999	NA	0.396028886236797	0.301561658869887	0.733730932025567	NA	NA	NA	XP_021693297.1putative thiamine transporter SLC35F3 isoform X1
AAEL00060	geranylgeranyl transferase type-2 subunit alpha	0.396124849398727	0.140977004737508	0.552330595273689	NA	NA	KXJ74894.1 hypothet	XP_021703016.1geranylgeranyl transferase type-2 subunit alpha
AAEL01286	uncharacterized LOC5576916	0.396443896203189	0.173051774926416	0.596189604220472	NA	NA	NA	XP_021713330.1uncharacterized protein LOC5576916
AAEL01033	ribosome biogenesis protein TSR3 homolog	0.39645063233593	0.102254735641327	0.484636597025644	NA	NA	NA	XP_001654463.1ribosome biogenesis protein TSR3 homolog
AAEL00866	L-galactose dehydrogenase	0.396538889292201	0.243486851535179	0.678342721110118	NA	NA	KXJ82380.1 hypothet	XP_001659401.2L-galactose dehydrogenase
AAEL02739	NA	0.396843424483101	0.105668738676434	0.490349840292762	NA	NA	NA	XP_021712811.1nucleolar protein 14 homolog
AAEL00383	nucleolar MIF4G domain-containing protein 1 homolog	0.397144750849259	0.0950745215091021	0.466773927728342	NA	NA	KXJ69606.1 hypothet	XP_001657307.2nucleolar MIF4G domain-containing protein 1 homolog
AAEL01206	PBAN-type neuropeptides	0.397901493773895	0.228027070525867	0.66175420142127	NA	NA	KXJ81110.1 hypothet	XP_001662212.2PBAN-type neuropeptides
AAEL00482	sodium/potassium/calcium exchanger 4	0.398065772803528	0.260120395763554	0.693202398440841	NA	NA	XP_019544180.1 PRE	XP_021706751.1sodium/potassium/calcium exchanger 4 isoform X2
AAEL02540	NA	0.399432105160606	0.17328802210984	0.596392068481585	NA	NA	NA	XP_021706341.1uncharacterized protein LOC5576422 isoform X2
AAEL00391	peripheral plasma membrane protein CASK	0.399638669898092	0.160484300672895	0.577674739374419	NA	NA	KFB42083.1 AGAP00	XP_021693229.1peripheral plasma membrane protein CASK isoform X3
AAEL02355	NA	0.400308900601604	0.0581281253021488	0.377750712592071	NA	NA	NA	XP_021711966.1 microsomal triglyceride transfer protein large subunit
AAEL02158	NA	0.400659245849302	0.240136547795233	0.674980307372712	NA	aag04214 ; aag	NA	XP_021711034.1bcl-2-related ovarian killer protein
AAEL00710	potassium voltage-gated channel protein Shal	0.401249368204075	0.291395887993417	0.722646576583698	NA	NA	XP_019562588.1 PRE	XP_001658131.1potassium voltage-gated channel protein Shal isoform X2
AAEL01156	F-box/WD repeat-containing protein 4	0.401461944111988	0.126629555109878	0.530481545423179	NA	NA	KXJ81717.1 hypothet	XP_001655432.1F-box/WD repeat-containing protein 4
AAEL00648	uncharacterized LOC5568047	0.401865843500631	0.0491848599987305	0.352961636078127	NA	NA	KXJ76983.1 hypothet	XP_001651983.1uncharacterized protein LOC5568047
AAEL01089	vesicle transport protein GOT1B	0.402256205815819	0.029314194607893	0.276665865417263	NA	NA	XP_019528566.1 PRE	XP_001655035.1vesicle transport protein GOT1B isoform X2
AAEL01238	zinc finger protein 830	0.402608460468834	0.193484941959938	0.624628157151667	NA	NA	KXJ70980.1 hypothet	XP_001662533.2zinc finger protein 830
AAEL01285	uncharacterized LOC5576905	0.403489237256482	0.00521071651042874	0.115262051271551	NA	NA	NA	XP_001663007.2uncharacterized protein LOC5576905
AAEL02346	NA	0.40367734017307	0.312349692149821	0.744018629406202	NA	NA	NA	NA
AAEL01259	NA	0.403936819519492	0.0208152972900498	0.235310031527075	NA	NA	NA	XP_021694789.1uncharacterized protein LOC5576540 isoform X2
AAEL00816	aminopeptidase N	0.404227194009643	0.029518046755156	0.277735241555159	NA	NA	KXJ84070.1 hypothet	XP_001658979.2aminopeptidase N isoform X1
AAEL00927	uncharacterized LOC5571749	0.404270760449367	0.130180518855442	0.537053220341536	NA	NA	NA	XP_021705050.1uncharacterized protein LOC5571749 isoform X1
AAEL00502	multidrug resistance-associated protein 1	0.404329700620099	0.305658632333088	0.73842760166161	NA	NA	KXJ83954.1 hypothet	XP_001650218.2multidrug resistance-associated protein 1
AAEL02744	NA	0.404448346465353	0.00242321886618999	0.0760003211995279	NA	NA	NA	AAV90649.1putative salivary protein
AAEL01263	uncharacterized LOC5576614	0.404455348380497	0.033409316443498	0.28758889608332	NA	NA	KXJ80512.1 hypothet	XP_001662768.2uncharacterized protein LOC5576614
AAEL00467	transcriptional adapter 3	0.404660086474937	0.00151266517231183	0.0563544328567346	NA	NA	ETN67882.1 hypothe	XP_001649558.1transcriptional adapter 3
AAEL02448	NA	0.404823089627289	0.281300230830506	0.715107379448722	NA	aag04080	NA	XP_021704022.1gamma-aminobutyric acid type B receptor subunit 2
AAEL00680	probable cytochrome P450 9f2	0.405167833469536	0.19979256906034	0.633375309392567	NA	NA	NA	XP_001652218.2probable cytochrome P450 9f2
AAEL00367	zinc finger protein draculin	0.405291575199091	0.140307000679813	0.550646976570049	NA	NA	KXJ74999.1 hypothet	XP_001657145.2zinc finger protein draculin
AAEL00130	histone PARylation factor 1-like	0.405533166764141	0.012148770997672	0.178583052269293	NA	NA	KXJ79863.1 hypothet	XP_001653009.1histone PARylation factor 1-like
AAEL02556	NA	0.405655851457476	0.00324949368123373	0.0889935739723594	480	aag04214	NA	XP_021696772.1peroxiredoxin-2
AAEL01823	NA	0.406188590781746	0.15067771324556	0.566025396924239	NA	NA	NA	XP_021702943.1uncharacterized protein LOC5575842 isoform X1
AAEL00683	protein yellow	0.407323518126371	0.0686903124950911	0.402860583543549	NA	NA	XP_019530244.1 PRE	XP_001658066.2protein yellow
AAEL00453	probable G-protein coupled receptor CG31760	0.407374927679386	0.17738222815097	0.602182613972581	NA	NA	KFB46242.1 AGAP00	XP_021701369.1probable G-protein coupled receptor CG31760 isoform X1
AAEL02314	NA	0.407824521549347	0.234882865754792	0.668737559309875	NA	NA	NA	XP_021697304.1zinc finger protein 354A
AAEL00455	protein lin-52 homolog	0.409339773871411	0.159285606810715	0.577560236664366	NA	NA	KXJ69767.1 hypothet	XP_001649405.1protein lin-52 homolog
AAEL00516	60S ribosome subunit biogenesis protein NIP7 homolog	0.409789137327929	0.0150090102004271	0.197869501238295	NA	NA	ETN67955.1 60S ribo	XP_001650454.160S ribosome subunit biogenesis protein NIP7 homolog
AAEL01268	stress-associated endoplasmic reticulum protein 2	0.410296016815408	0.00514664183690206	0.114938319578267	NA	NA	KFB44289.1 AGAP00	XP_001656189.1stress-associated endoplasmic reticulum protein 2
AAEL01129	aminopeptidase N	0.410386839449317	0.381115916915648	0.787211822100515	NA	NA	KXJ75860.1 hypothet	XP_001661567.1aminopeptidase N
AAEL02673	NA	0.411264887088461	0.0599857594200807	0.383636203860763	00480 ; 00460 ; 00430	NA	NA	XP_021699898.1gamma-glutamyltranspeptidase 1
AAEL02350	NA	0.411590410847015	0.0252163338667543	0.259075173517489	NA	NA	NA	XP_021705502.1ecdysone 20-monooxygenase isoform X1
AAEL00142	uncharacterized LOC5570480	0.411828296459227	0.00724297865966741	0.139143819679039	NA	NA	KXJ74862.1 hypothet	ABF18326.1membrane glycoprotein LIG-1
AAEL01249	histone H2A-beta, sperm	0.412119460858427	0.207238634408806	0.639832374753494	NA	NA	KXJ73882.1 hypothet	XP_001662624.1histone H2A-beta, sperm
AAEL00483	sialin	0.412331434647242	0.20328879541064	0.636063752250496	NA	NA	KXJ82288.1 hypothet	XP_019542723.1 sialin-like
AAEL01976	NA	0.412384856511526	0.149262086048673	0.564765508149627	NA	NA	NA	XP_021711058.1serine protease 7
AAEL01451	NA	0.412544406449636	0.0188080727537187	0.224186380155077	NA	NA	NA	XP_001648915.2seminal metalloprotease 1
AAEL02018	NA	0.412727580544655	0.292446822503408	0.723512283028756	NA	NA	NA	XP_021702896.1centrosomal protein of 104 kDa isoform X2
AAEL02459	NA	0.412816717078731	0.365434960134986	0.779635090143656	NA	NA	NA	XP_021702915.1protein nessun dorma-like
AAEL00603	NA	0.413019496442938	0.0507101456183574	0.35814179653755	NA	NA	XP_019537486.1 PRE	XP_021707763.1RNA polymerase II elongation factor Ell isoform X1
AAEL01315	cilia- and flagella-associated protein 20	0.413056444782029	0.0846513763018324	0.441820669235316	NA	NA	NA	XP_001663334.1cilia- and flagella-associated protein 20
AAEL01316	uncharacterized LOC5577316	0.413086619133636	0.00696245450837774	0.137411930736898	NA	NA	NA	XP_021699018.1uncharacterized protein LOC5577316
AAEL01390	programmed cell death protein 2	0.414704823105879	0.0430506927066912	0.327632237384908	NA	NA	NA	XP_021697305.1programmed cell death protein 2 isoform X2
AAEL01975	NA	0.415019192982869	0.249660758816008	0.682244883827679	NA	NA	NA	XP_021707441.1uncharacterized protein LOC5569631
AAEL00183	pancreatic lipase-related protein 2	0.415077503866955	0.0541017327013722	0.367217832240442	NA	NA	KXJ78099.1 hypothet	XP_021700824.1pancreatic lipase-related protein 2
AAEL00474	GTP-binding protein 10 homolog	0.41513854320181	0.174833342911718	0.598295433794581	NA	NA	KXJ81345.1 hypothet	XP_001649734.1GTP-binding protein 10 homolog
AAEL00368	E3 ubiquitin-protein ligase rnf146	0.416410692913476	0.0339665097131811	0.290483106301759	NA	NA	KXJ73782.1 hypothet	XP_001657125.1E3 ubiquitin-protein ligase rnf146
AAEL00941	putative helicase mov-10-B.1	0.416547817287241	0.164502067981076	0.583247700156998	NA	NA	NA	XP_021708618.1putative helicase MOV-10 isoform X1
AAEL01805	NA	0.416690263200778	0.187078073471842	0.614196367514984	NA	NA	NA	XP_001651835.2zinc finger protein 239
AAEL00547	protein transport protein Sec61 subunit gamma	0.41678155484219	0.0079166929110978	0.144528409372715	NA	aag04141 ; aag	CRL07548.1 CLUMA_	XP_001650928.1protein transport protein Sec61 subunit gamma
AAEL00748	wolframin	0.417527666163169	0.0488894729278704	0.352019506950128	NA	aag04141	KXJ77421.1 hypothet	XP_019528556.1 wolframin-like
AAEL00527	kxDL motif-containing protein CG10681	0.417784596602562	0.116968701272945	0.514582987743323	NA	NA	KXJ76459.1 hypothet	XP_001650600.1kxDL motif-containing protein CG10681
AAEL00818	uncharacterized LOC5570231	0.418364933482256	0.200171193061708	0.633770569380376	NA	NA	KXJ74734.1 hypothet	XP_001659010.1uncharacterized protein LOC5570231
AAEL02610	NA	0.419635607090624	0.00653404950249106	0.132685213264791	NA	aag03013	NA	XP_021700838.1nuclear pore complex protein Nup155
AAEL01239	uncharacterized LOC5576198	0.420469904020261	0.012663906111626	0.182308324322341	NA	NA	KXJ72120.1 hypothet	XP_001662539.2uncharacterized protein LOC5576198
AAEL00793	serine/threonine-protein kinase PAK mbt	0.42072111492699	0.0919328021155471	0.46005268034546	04151 ; 05165 ; 04714 ;	NA	XP_019529323.1 PRE	XP_021704460.1serine/threonine-protein kinase PAK mbt
AAEL00328	translocating chain-associated membrane protein 1	0.421446337704531	0.00321770145429268	0.088916783130334	NA	aag04141	ETN65729.1 transloc	XP_001663552.1translocating chain-associated membrane protein 1
AAEL00311	peroxisomal targeting signal 2 receptor	0.422942009946496	0.0135735873750539	0.187825791015408	NA	aag04146	ETN62162.1 peroxisc	XP_021699221.1peroxisomal targeting signal 2 receptor
AAEL01231	actin-related protein 3	0.423081142056349	0.0249906556387795	0.258627500477641	NA	NA	KFB44442.1 AGAP00	XP_001662409.1actin-related protein 3
AAEL01964	NA	0.423575243711538	0.0708272742842072	0.409428936477113	NA	NA	NA	XP_021697014.1zinc finger protein 14-like
AAEL01398	protein transport protein Sec61 subunit beta	0.424122693116994	0.0021427739968381	0.0727761081152129	NA	aag04141 ; aag	NA	XP_001657235.1protein transport protein Sec61 subunit beta
AAEL02884	NA	0.424136048547881	0.217558960995687	0.650415061430251	NA	NA	NA	NA
AAEL00297	uncharacterized LOC5576709	0.424190482418938	0.122562278458119	0.524810649777391	NA	NA	KXJ69657.1 hypothet	XP_021704626.1uncharacterized protein LOC5576709
AAEL01308	fibroblast growth factor receptor-like 1	0.425112397070803	0.027427738495127	0.269359711453744	NA	NA	NA	XP_001663291.2fibroblast growth factor receptor-like 1
AAEL02123	NA	0.425795762558146	0.207553865831845	0.639832374753494	NA	NA	NA	XP_021707047.1zinc finger protein 239-like
AAEL01342	U7 snRNA-associated Sm-like protein LSm10	0.426266465909602	0.131207455783393	0.538981683630591	NA	NA	NA	XP_021708463.1U7 snRNA-associated Sm-like protein LSm10

AAEL0011	GATOR complex protein NPRL3	0.427255709074852	0.0396229572634173	0.313353264954028	NA	aag04150	XP_019536167.1 PRE	XP_001652433.2GATOR complex protein NPRL3
AAEL0260	NA	0.427411492559925	0.0327196403180807	0.28463170766102	940	NA	NA	XP_021692994.1chorion peroxidase
AAEL0263	NA	0.427476549643991	0.248632907978468	0.680886132809113	NA	NA	NA	NA
AAEL0127	general transcription factor IIH subunit 1	0.427478074822328	0.00197126405897514	0.0693618362591811	NA	aag03420 ; aag	KXJ78206.1 hypothet	XP_001662850.2general transcription factor IIH subunit 1
AAEL0045	DNA polymerase eta	0.427769266351185	0.20096922164803	0.634514122727206	NA	aag03460	XP_001845071.1 DN	XP_001649408.2DNA polymerase eta
AAEL0224	NA	0.428525507310616	0.158009623278651	0.575615420985807	NA	NA	NA	XP_021703902.1dystrobrevin alpha
AAEL0099	rRNA-processing protein UTP23 homolog	0.429536948337748	0.0594307938446496	0.382167830159655	NA	NA	NA	XP_001660489.1rRNA-processing protein UTP23 homolog
AAEL0273	NA	0.429672787325812	0.0993368869511915	0.478084745672	NA	aag01100 ; aag	NA	XP_021699030.1ADP-dependent glucokinase
AAEL0038	mitochondrial ribonuclease P protein 3	0.431702948266506	0.24609569979073	0.679744205967664	NA	NA	KXJ68708.1 hypothet	XP_001664268.2mitochondrial ribonuclease P protein 3
AAEL0148	uncharacterized LOC5576391	0.43189381188031	0.0307129790516354	0.281224109837052	NA	NA	NA	XP_021694388.1uncharacterized protein LOC5576391
AAEL0006	ATP-dependent (S)-NAD(P)H-hydrate dehydratase	0.433035464376639	0.0100650551644679	0.161638111035661	NA	NA	E3XDZ8.1 RecName:	XP_001649762.2ATP-dependent (S)-NAD(P)H-hydrate dehydratase
AAEL0150	putative deoxyribonuclease TATDN1	0.433490029549928	0.0235594387617577	0.249663291910268	NA	NA	NA	XP_001650441.1putative deoxyribonuclease TATDN1
AAEL0087	serine protease SP24D	0.433663768334426	0.05592533156857	0.371974308318541	NA	NA	KXJ80635.1 hypothet	XP_001659492.2serine protease SP24D
AAEL0080	uncharacterized LOC5569953	0.434579732682858	0.115891601806824	0.512764772622422	NA	NA	KXJ76753.1 hypothet	XP_001658856.2uncharacterized protein LOC5569953
AAEL0128	uncharacterized LOC5576909	0.434789685290607	0.0108144522032421	0.167815496077966	NA	NA	NA	XP_001663008.2uncharacterized protein LOC5576909
AAEL0024	NA	0.43496191804434	0.204467976754667	0.637782699712521	NA	NA	XP_001862536.1 con	XP_021699204.1negative elongation factor D-like
AAEL0039	methionine--tRNA ligase, mitochondrial	0.435103879605779	0.0359014498486838	0.300059165765293	00970 ; 00450	aag01100 ; aag	KXJ69033.1 hypothet	XP_001647993.2methionine--tRNA ligase, mitochondrial
AAEL0228	NA	0.435706983226774	0.14311398988426	0.555375365647863	970	aag00970	NA	XP_021706185.1probable cysteine--tRNA ligase, mitochondrial
AAEL0031	NA	0.437122304091013	0.112412092430102	0.503547757511443	NA	aag01100 ; aag	KXJ76631.1 hypothet	XP_001656538.2sphingosine-1-phosphate lyase-like
AAEL0140	deoxynucleoside kinase	0.437147663473772	0.00349568504268638	0.0906120951064792	NA	NA	NA	XP_001657342.1deoxynucleoside kinase isoform X1
AAEL0046	DNA primase large subunit	0.437249852287018	0.275536643328067	0.711001645592863	NA	aag03030	KXJ76318.1 hypothet	XP_001649593.2DNA primase large subunit
AAEL0043	FK506-binding protein 2	0.437451884097737	0.0363434808373814	0.301562408174557	NA	NA	XP_018566457.1 FK5	XP_001648889.1FK506-binding protein 2
AAEL0251	NA	0.438750113470527	0.116354715942599	0.514016368748824	NA	NA	NA	NA
AAEL0187	NA	0.439273063096255	0.15534498892847	0.573091811919643	NA	NA	NA	XP_019544235.1 mediator of DNA damage checkpoint protein 1-like isoform X1
AAEL0137	RISC-loading complex subunit tarbp2	0.439341730093376	0.243067122781334	0.677870931254677	NA	NA	NA	XP_001663906.2RISC-loading complex subunit tarbp2
AAEL0050	zinc finger protein 883	0.439444837534694	0.187528217461545	0.614323480626963	NA	NA	KXJ83924.1 hypothet	XP_001650310.2zinc finger protein 883
AAEL0273	NA	0.439788927043306	0.293085536664488	0.723631099647605	NA	NA	NA	XP_021698976.1uncharacterized protein LOC5572002
AAEL0028	uncharacterized LOC5576228	0.440249531252256	0.1982269591699	0.631696238860921	NA	NA	KXJ78079.1 hypothet	XP_001656006.2uncharacterized protein LOC5576228
AAEL0067	MIP18 family protein CG7949	0.440365218492129	0.0106213497718003	0.166147088221529	NA	NA	KFB48216.1 AGAP00	XP_001652201.1MIP18 family protein CG7949
AAEL0107	actin-related protein 8	0.440892044622753	0.212466327763545	0.645843785766909	NA	NA	XP_001846070.1 con	XP_001654858.1actin-related protein 8
AAEL0103	zinc finger protein 626	0.441401828814052	0.160486047241113	0.577674739374419	NA	NA	NA	XP_001660817.2zinc finger protein 626
AAEL0045	retinol dehydrogenase 12	0.441552635207189	0.0389471845747044	0.311374450439991	NA	NA	XP_001845073.1 reti	XP_001649410.2retinol dehydrogenase 12 isoform X1
AAEL0200	NA	0.441892112256155	0.0415384467786871	0.321207384249982	NA	NA	NA	XP_021694510.1F-box/LRR-repeat protein 4
AAEL0063	digestive cysteine proteinase 1	0.44208786651882	0.0651114415618774	0.39730198438886	NA	NA	XP_019531167.1 PRE	XP_021710783.1digestive cysteine proteinase 1 isoform X2
AAEL0279	NA	0.443055989090547	0.00328589855364824	0.0891942138367879	NA	NA	NA	XP_021698366.1palmitoyltransferase ZDHHC6
AAEL0068	uncharacterized LOC5568446	0.443056300695574	0.271309109234572	0.706703671719641	NA	NA	KXJ84565.1 hypothet	XP_001652277.1uncharacterized protein LOC5568446
AAEL0056	adenosine 3'-phospho 5'-phosphosulfate transporter 1	0.443058752343282	0.0168327426728017	0.210455024558588	NA	NA	KXJ73986.1 hypothet	XP_001651353.1adenosine 3'-phospho 5'-phosphosulfate transporter 1
AAEL0266	NA	0.443356029578767	0.0535747505816866	0.365820839771452	NA	NA	NA	XP_021704795.1zinc finger protein 665-like
AAEL0108	calcium/calmodulin-dependent protein kinase kinase 1	0.443860283830712	0.0249505463920907	0.258627500477641	NA	aag04140	XP_019531088.1 PRE	XP_021708747.1calcium/calmodulin-dependent protein kinase kinase 1 isoform X1
AAEL0090	spermine oxidase	0.444987177264266	0.0649691886968375	0.39730198438886	NA	NA	XP_001863219.1 spe	XP_001653663.2spermine oxidase
AAEL0067	probable cytochrome P450 9f2	0.445023791166286	0.289208193494149	0.721217831038796	NA	NA	KXJ73267.1 hypothet	XP_001652217.1probable cytochrome P450 9f2
AAEL0235	NA	0.445576098868921	0.158348935587585	0.576186113377291	NA	NA	NA	NA
AAEL0134	phenoloxidase 2	0.446484068292501	0.00537450430335396	0.117414467687443	NA	NA	NA	XP_001663688.2phenoloxidase 2
AAEL0103	rac GTPase-activating protein 1	0.446594428673905	0.186319764977554	0.614196367514984	NA	NA	NA	XP_001654457.1rac GTPase-activating protein 1
AAEL0081	arrestin domain-containing protein 3	0.446767650905158	0.0368322130018559	0.303201029751424	NA	NA	KXJ68732.1 hypothet	XP_001659002.1arrestin domain-containing protein 3
AAEL0072	uncharacterized LOC5569011	0.447199118185434	0.0192007927416027	0.226230082981086	NA	aag03420 ; aag	KXJ82250.1 hypothet	XP_001844516.1replication factor A, 14kD-subunit
AAEL0197	NA	0.447396888918006	0.025604796518589	0.260925069284669	NA	NA	NA	XP_021703594.1uncharacterized protein LOC5572076
AAEL0141	multiple coagulation factor deficiency protein 2	0.447631004454372	0.30143852464371	0.733625311761814	NA	NA	NA	XP_001648086.1multiple coagulation factor deficiency protein 2
AAEL0130	zinc finger matrin-type protein 5	0.447664787417125	0.0847835937210013	0.441820669235316	NA	NA	NA	XP_001663304.1zinc finger matrin-type protein 5
AAEL0021	kinesin-associated protein 3	0.448416477233815	0.00387790438332669	0.0964445300956005	NA	NA	XP_001849818.1 kin	XP_001654812.1kinesin-associated protein 3
AAEL0155	uncharacterized LOC5579329	0.448444739684707	0.110138954702354	0.500079481483548	NA	NA	NA	XP_001647638.2uncharacterized protein LOC5579329 isoform X2
AAEL0112	cytoplasmic tRNA 2-thiolation protein 1	0.448531179655676	0.0175361523955276	0.214955509175633	NA	aag04122	XP_001850697.1 ATF	XP_001661559.1cytoplasmic tRNA 2-thiolation protein 1
AAEL0081	heme oxygenase 1	0.448976423971514	0.0178905023326304	0.21690171602749	860	aag01100 ; aag	KXJ73601.1 hypothet	XP_001658955.1uncharacterized protein LOC5570153
AAEL0068	probable cytochrome P450 9f2	0.449982306823302	0.00785908927200116	0.144528409372715	NA	NA	KXJ73267.1 hypothet	XP_001652216.1probable cytochrome P450 9f2
AAEL0018	uncharacterized LOC5572801	0.45007286597634	0.131500351619354	0.538981683630591	NA	NA	ETN64306.1 hypothe	XP_001654202.2uncharacterized protein LOC5572801
AAEL0198	NA	0.450509020814989	0.0151751234195222	0.198907702459398	NA	NA	NA	XP_021697881.1putative uncharacterized protein DDB_G0282133 isoform X1
AAEL0015	uncharacterized LOC5571095	0.450640460493546	0.0160373386747467	0.20383368851522	NA	NA	KXJ69372.1 hypothet	XP_021709984.1uncharacterized protein LOC5571095 isoform X1
AAEL0034	structural maintenance of chromosomes protein 2	0.451094104959508	0.053823669782977	0.366428852058877	NA	NA	KXJ74822.1 hypothet	XP_001656789.2structural maintenance of chromosomes protein 2
AAEL0083	transcription initiation factor TFIID subunit 12	0.451108530761742	0.0634655184729649	0.393227665373111	NA	aag03022	XP_019549050.1 PRE	XP_001653212.1transcription initiation factor TFIID subunit 12 isoform X2
AAEL0086	barrier-to-autointegration factor	0.451382950329898	0.00844366391850052	0.148894695587464	NA	NA	KOX72877.1 Barrier-t	XP_001659424.1barrier-to-autointegration factor
AAEL0090	AP-3 complex subunit beta-1	0.453107887844502	0.0194666401608962	0.22702918113002	NA	aag04142	KXJ79624.1 hypothet	XP_021704979.1AP-3 complex subunit beta-1
AAEL0281	NA	0.453197827263906	0.27527977613604	0.710952708392882	NA	NA	NA	ABF18025.1angiopoietin-like protein variant
AAEL0139	probable 3-hydroxyisobutyrate dehydrogenase, mitochondrial	0.45349450637724	0.000731578422637516	0.0369889266214859	280	aag01100 ; aag	NA	XP_001664110.1probable 3-hydroxyisobutyrate dehydrogenase, mitochondrial
AAEL0154	uncharacterized protein KIAA1143 homolog	0.453555527089494	0.081627525573549	0.435199100516323	NA	NA	NA	XP_001647746.1uncharacterized protein KIAA1143 homolog
AAEL0064	general odorant-binding protein lush	0.453837005315224	0.23286224394113	0.666707644289445	NA	NA	AFC60565.1 odorant	XP_001657830.1general odorant-binding protein lush
AAEL0215	NA	0.453850610261523	0.265242991214605	0.699760895973853	NA	NA	NA	XP_021712593.1zinc finger protein 426
AAEL0089	NADH dehydrogenase (ubiquinone) complex I, assembly factor 6 homolog	0.453883759022918	0.163066858252898	0.581366301644056	NA	NA	KXJ73195.1 hypothet	XP_001653613.1NADH dehydrogenase (ubiquinone) complex I, assembly factor 6 homolog
AAEL0213	NA	0.454134592303998	0.298678298844582	0.730450295042154	NA	NA	NA	XP_021698097.1uncharacterized protein LOC5566825
AAEL0133	oxygen-dependent coproporphyrinogen-III oxidase	0.454159857119252	0.017452189747831	0.214412616901924	860	aag01100 ; aag	NA	XP_001663577.1oxygen-dependent coproporphyrinogen-III oxidase
AAEL0022	ribonuclease P protein subunit p30	0.455634337672786	0.0296893849837009	0.278209491466411	NA	aag03013 ; aag	KXJ72345.1 hypothet	XP_001654977.1ribonuclease P protein subunit p30
AAEL0251	NA	0.457563543501234	0.136378441537563	0.545589928345625	NA	NA	NA	XP_021697636.1uncharacterized protein LOC110675918
AAEL0132	uncharacterized LOC5577590	0.457735613444575	0.0318920782340361	0.284442054201394	NA	NA	NA	XP_001656584.2uncharacterized protein LOC5577590
AAEL0118	guanine nucleotide-binding protein subunit beta-like protein 1	0.457751592995383	0.00254647738340809	0.0778494514356189	NA	NA	KXJ84034.1 hypothet	XP_001662018.2guanine nucleotide-binding protein subunit beta-like protein 1
AAEL0194	NA	0.459125511671814	0.19981401034195	0.633375309392567	NA	NA	NA	XP_021703131.1uncharacterized protein LOC5571525 isoform X4
AAEL0034	DNA-directed RNA polymerase II subunit RPB7	0.459245623953793	0.0607003741897564	0.384941803020695	NA	aag03020	XP_001842888.1 DN	XP_001656728.1DNA-directed RNA polymerase II subunit RPB7
AAEL0000	myotubularin-related protein 9	0.459414350251693	0.272696803567415	0.707857824097983	NA	NA	KXJ75459.1 hypothet	XP_001647861.1myotubularin-related protein 9
AAEL0290	NA	0.459460419115705	0.0820146164354189	0.435989890490309	NA	NA	NA	XP_019932676.1 lectin subunit alpha-like
AAEL0001	zinc finger protein 83	0.460569961473825	0.147541576487483	0.561952643558699	NA	NA	KXJ83439.1 hypothet	XP_001657702.2zinc finger protein 83
AAEL0253	NA	0.461052507666214	0.013748546206936	0.187831676988403	NA	NA	NA	NA
AAEL0210	NA	0.461383927237146	0.0724085470437431	0.413814782657084	NA	NA	NA	XP_021698617.1peptidoglycan-recognition protein LA-like isoform X1
AAEL0127	glycine N-methyltransferase	0.461424087682973	0.0066702647370218	0.134016978406276	NA	aag01100 ; aag	ETN61353.1 hypothe	XP_001662871.1glycine N-methyltransferase
AAEL0002	7-methylguanosine phosphate-specific 5'-nucleotidase	0.46213455779422	0.000549447226411778	0.0306425053178254	00240 ; 00230 ; 00760	aag01100 ; aag	XP_019552535.1 PRE	XP_001654766.17-methylguanosine phosphate-specific 5'-nucleotidase

AAEL0005	TCF3 fusion partner homolog	0.462135818955947	0.0580789821171192	0.377750712592071	NA	NA	ETN65560.1 hypothe	XP_001647963.1TCF3 fusion partner homolog
AAEL0120	zinc finger protein 691	0.462933599179318	0.118739429918802	0.517103754904313	NA	NA	KXJ78341.1 hypothet	XP_001662164.1zinc finger protein 691
AAEL02151	NA	0.463209521076643	0.0677149017753525	0.40094231299198	NA	NA	NA	XP_021710923.1microprocessor complex subunit DGCR8
AAEL0078	inactive ubiquitin carboxyl-terminal hydrolase MINDY-4B	0.463561771542093	0.483386840334059	0.847379421050056	NA	NA	KXJ78685.1 hypothet	XP_021706356.1inactive ubiquitin carboxyl-terminal hydrolase MINDY-4B isoform X1
AAEL0091	rho GTPase-activating protein 190	0.463567527376902	0.00303221362231575	0.0867379077340594	NA	NA	XP_019533919.1 PRE	XP_021701329.1rho GTPase-activating protein 190 isoform X1
AAEL0105	zinc finger protein ubi-d4	0.464035157022798	0.00219703249933792	0.0737239957572492	NA	NA	XP_001868889.1 zinc	XP_001660913.1zinc finger protein ubi-d4
AAEL0231	NA	0.464155649242855	0.0716422967864339	0.411775399768123	NA	NA	NA	XP_021704284.1uncharacterized protein LOC110677657
AAEL0199	NA	0.46498023967517	0.012870668129978	0.182489812221969	NA	NA	NA	XP_001654215.1uncharacterized protein LOC110673976
AAEL0006	uncharacterized LOC5564821	0.465122394456688	0.0280442073811749	0.271500221524543	NA	NA	KXJ75479.1 hypothet	XP_001649184.2uncharacterized protein LOC5564821
AAEL0126	probable U2 small nuclear ribonucleoprotein A'	0.465147808846135	0.0591104083731588	0.381093648037575	NA	aag03040	XP_019538081.1 PRE	XP_001662788.1probable U2 small nuclear ribonucleoprotein A' isoform X2
AAEL00171	protein Shroom	0.465164517636911	0.134461826872462	0.542662316284181	NA	NA	KXJ77745.1 hypothet	XP_021703576.1protein Shroom
AAEL01511	zinc finger protein 468	0.465192548297758	0.259727817079385	0.692557337804839	NA	NA	NA	XP_021699382.1zinc finger protein 468
AAEL0090	actin-related protein 2/3 complex subunit 4	0.465296973701501	0.0336137401663006	0.288269931976046	NA	aag04144	ETN59007.1 ARP2/3	XP_001653667.1actin-related protein 2/3 complex subunit 4
AAEL0198	NA	0.465900413559689	0.00033585923714882	0.0223954833350974	514	aag00514	NA	XP_021697652.1UDP-N-acetylglucosamine--peptide N-acetylglucosaminyltransferase 110 kDa subunit isoform X1
AAEL0002	DNA replication ATP-dependent helicase/nuclease DNA2	0.46683798442723	0.17972116083152	0.604991500927545	NA	aag03030	KXJ71310.1 hypothet	XP_021710496.1DNA replication ATP-dependent helicase/nuclease DNA2 isoform X1
AAEL0146	NA	0.466963511105053	0.30835741479417	0.741056393558619	NA	NA	NA	XP_001649103.2LOW QUALITY PROTEIN: uncharacterized protein LOC5564757
AAEL0118	oocyte zinc finger protein XlCOF22	0.467797842286257	0.104924006421793	0.489113833380617	NA	NA	KFB43619.1 AGAP00	XP_001661929.2oocyte zinc finger protein XlCOF22
AAEL0096	uncharacterized LOC5580029	0.469235118127192	0.0498728994749374	0.355484446915859	NA	NA	NA	XP_001653911.1uncharacterized protein LOC5580029
AAEL0088	myb-like protein AA	0.469533541112355	0.22752703602179	0.661726860136697	NA	NA	NA	XP_021700301.1CREB-regulated transcription coactivator 1 isoform X1
AAEL00901	glutathione S-transferase theta-3	0.469917994724993	0.171314323491667	0.592867395551078	00980 ; 00982 ; 00983 ;	aag01100 ; aag	KXJ75703.1 hypothet	XP_021694971.1glutathione S-transferase theta-3
AAEL0066	zinc finger protein 26	0.470074633782649	0.131749427494829	0.538981683630591	NA	NA	KXJ69935.1 hypothet	XP_001652107.2zinc finger protein 26
AAEL00731	protein inturned	0.470279828413307	0.18678249663191	0.614196367514984	NA	NA	KXJ79902.1 hypothet	XP_021699818.1protein inturned
AAEL0007	uncharacterized LOC5566292	0.470581647488683	0.00954405601057805	0.158773875613038	NA	NA	KXJ67848.1 hypothet	XP_001650647.1uncharacterized protein LOC5566292
AAEL0075	short/branched chain specific acyl-CoA dehydrogenase, mitochondrial	0.471256791858177	0.00215117381083872	0.0727761081152129	NA	aag01100 ; aag	KFB49225.1 AGAP00	XP_001658431.1short/branched chain specific acyl-CoA dehydrogenase, mitochondrial
AAEL00001	esterase AAEL000016	0.471285538993211	0.0470026686537293	0.345967136895395	NA	NA	KXJ78177.1 hypothet	XP_001647845.1esterase AAEL000016
AAEL0018	zinc finger protein 391	0.471504050495246	0.16975104644143	0.589897707459986	NA	NA	KXJ71195.1 hypothet	XP_001654188.2zinc finger protein 391
AAEL0032	CD63 antigen	0.471841347181474	0.000641985429063586	0.0341621442326704	NA	aag04142	KFB42265.1 AGAP00	XP_021702248.1CD63 antigen
AAEL0034	peptidyl-prolyl cis-trans isomerase H	0.471985412914937	0.00451765660737655	0.105244243293871	NA	aag03040	ETN67262.1 peptidyl	XP_001656850.1peptidyl-prolyl cis-trans isomerase H
AAEL0013	solute carrier family 25 member 40	0.472959857420209	0.00709602524028045	0.137861850831157	NA	NA	XP_001863317.1 solu	XP_001653053.1solute carrier family 25 member 40
AAEL0065	thiamin pyrophosphokinase 1	0.47295999139146	0.0189178016192001	0.225171411092632	730	aag01100 ; aag	KXJ82173.1 hypothet	XP_001652095.1thiamin pyrophosphokinase 1
AAEL0074	translocon-associated protein subunit gamma	0.473148372396509	0.00120323323307441	0.0487760009284173	NA	aag04141	KFB39242.1 transloc	XP_011493383.1translocon-associated protein subunit gamma
AAEL0062	deoxynucleoside triphosphate triphosphohydrolase SAMHD1	0.473520998678956	0.00359619442483388	0.0919227252703372	NA	NA	XP_001841882.1 san	XP_021696505.1deoxynucleoside triphosphate triphosphohydrolase SAMHD1
AAEL0097	sialin	0.473568682153059	0.0882803863353267	0.454083910037829	NA	NA	NA	XP_019562734.1 sialin-like
AAEL0016	meiosis regulator and mRNA stability factor 1	0.473623093150912	0.042446048528532	0.324139866024524	NA	NA	XP_001842624.1 con	XP_001653744.2meiosis regulator and mRNA stability factor 1
AAEL00571	zinc finger protein 423	0.474205356212824	0.193524763751293	0.624628157151667	NA	NA	KXJ69504.1 hypothet	XP_001651358.2zinc finger protein 423
AAEL0198	NA	0.474791650643447	0.224389426843168	0.656544198985955	NA	NA	NA	XP_021709909.1whirlin isoform X2
AAEL0203	NA	0.476011652512916	0.136835655721384	0.545589928345625	NA	NA	NA	NA
AAEL0036	X-ray repair cross-complementing protein 5	0.47610730824895	0.0130467065173359	0.183852669789472	NA	aag03450	XP_001861497.1 ku	XP_001657128.2X-ray repair cross-complementing protein 5
AAEL0263	NA	0.476816558751507	0.19041013668388	0.6186984737871	NA	NA	NA	XP_021695316.1uncharacterized protein LOC110675210
AAEL0148	heat shock protein 83	0.477219132131791	0.000341320875221325	0.0225959330488247	NA	aag04141	NA	XP_001649752.1heat shock protein 83
AAEL0044	NA	0.477591248507375	0.00841104726518484	0.148894695587464	NA	aag04120	XP_005184297.1 PRE	XP_001649222.1fizzy-related protein homolog
AAEL0033	peptide transporter family 1	0.478738892178062	0.00714141687092861	0.137861850831157	NA	NA	XP_019540480.1 PRE	XP_001663546.2peptide transporter family 1
AAEL0087	protein Dr1	0.479138065641239	0.0351102060572371	0.296679629144808	NA	NA	ETN64478.1 tata-bin	XP_001659471.1protein Dr1
AAEL0040	1,5-anhydro-D-fructose reductase	0.481302123250025	0.0710648220451799	0.409506895880945	NA	aag01100 ; aag	KXJ83373.1 hypothet	XP_001648456.11,5-anhydro-D-fructose reductase
AAEL0244	NA	0.482350123399707	0.00384873612979434	0.0960041238111189	NA	NA	NA	XP_021705076.1tetratricopeptide repeat protein 39B
AAEL0021	U11/U12 small nuclear ribonucleoprotein 48 kDa protein	0.483321029826122	0.248270428757127	0.68058844480365	NA	NA	ETN61850.1 hypothe	XP_001654736.1U11/U12 small nuclear ribonucleoprotein 48 kDa protein
AAEL0022	purine nucleoside phosphorylase	0.483458918670909	0.0361877100995364	0.300966157937731	00230 ; 00240 ; 00760	aag01100 ; aag	KXJ73157.1 hypothet	XP_001661167.1purine nucleoside phosphorylase isoform X2
AAEL0013	geranylgeranyl transferase type-2 subunit beta	0.48360361575857	0.0231546116720948	0.24632223885158	NA	NA	XP_001863319.1 ger	XP_001653055.1geranylgeranyl transferase type-2 subunit beta
AAEL00211	3-oxoacyl-[acyl-carrier-protein] synthase, mitochondrial	0.483698887753455	0.0479096090108002	0.348612005978262	NA	aag01100 ; aag	KFB43555.1 AGAP00	XP_001654737.13-oxoacyl-[acyl-carrier-protein] synthase, mitochondrial
AAEL0055	Down syndrome cell adhesion molecule	0.483903207842535	0.150553579561206	0.566025396924239	NA	NA	ETN59156.1 defectiv	XP_021706887.1Down syndrome cell adhesion molecule
AAEL0226	NA	0.484135409684523	0.142847437986193	0.555375365647863	NA	NA	NA	NA
AAEL0281	NA	0.484869218979516	0.00295755141038338	0.0855829813784525	NA	NA	NA	NA
AAEL00831	mitotic spindle assembly checkpoint protein MAD2A	0.486708825552005	0.196973405785874	0.630345746481079	NA	NA	KXJ69574.1 hypothet	XP_021698783.1mitotic spindle assembly checkpoint protein MAD2A
AAEL0000	nucleolar protein 12	0.489476416220294	0.00540745358700998	0.117414467687443	NA	NA	XP_019528881.1 PRE	XP_001647890.2nucleolar protein 12
AAEL0101	uncharacterized LOC5572877	0.490458729618605	0.0240693678128081	0.253416845095492	NA	NA	NA	XP_001654237.2uncharacterized protein LOC5572877
AAEL0050	uncharacterized LOC5565838	0.490618896716262	0.125794905264581	0.530481545423179	NA	NA	XP_001843820.1 con	XP_001650216.2uncharacterized protein LOC5565838
AAEL0044	organic cation transporter protein	0.491281365950901	0.0520279223234407	0.361717594818022	NA	NA	KXJ75870.1 hypothet	XP_021711011.1organic cation transporter protein isoform X2
AAEL0119	crossover junction endonuclease MUS81	0.49263779653346	0.0315716424786544	0.28423411888152	NA	aag03460 ; aag	XP_019538331.1 PRE	XP_001655814.2crossover junction endonuclease MUS81
AAEL0086	zinc finger protein 26	0.49347593112377	0.141473941866995	0.552330595273689	NA	NA	XP_019549767.1 PRE	XP_021702190.1zinc finger protein 26
AAEL0107	uncharacterized LOC5573847	0.493554583785891	0.0470337848572201	0.345967136895395	NA	NA	KXJ77337.1 hypothet	XP_001654879.1uncharacterized protein LOC5573847
AAEL0033	signal recognition particle subunit SRP72	0.494115068364792	0.0019590959682364	0.0693618362591811	NA	aag03060	KXJ68334.1 hypothet	XP_021704856.1signal recognition particle subunit SRP72
AAEL0065	protein disulfide-isomerase TMX3	0.494214265775456	0.169452462432595	0.589443594700505	NA	NA	XP_001870826.1 disu	XP_021696650.1protein disulfide-isomerase TMX3
AAEL0006	protein disulfide-isomerase	0.494428793371697	0.00232491460121249	0.0747232890974067	NA	aag04141	KXJ76669.1 hypothet	XP_011493116.1protein disulfide-isomerase
AAEL0251	NA	0.494543254736342	0.00702591120243967	0.137558372095425	00630 ; 00670 ; 00260 ;	NA	NA	XP_021712489.1serine hydroxymethyltransferase, mitochondrial-like
AAEL0197	NA	0.496190641311519	0.00409900927310065	0.0997859347382861	NA	NA	NA	XP_021707280.1beta-1,4-N-acetylgalactosaminyltransferase bre-4
AAEL0147	protein spindle-F	0.49666337273486	0.00823654847019393	0.146885114385125	NA	NA	NA	XP_001649427.2protein spindle-F
AAEL0125	cytochrome b561 domain-containing protein 1	0.497071323312326	0.000285906744041952	0.0207158571549137	NA	NA	KXJ73881.1 hypothet	XP_001662625.2cytochrome b561 domain-containing protein 1
AAEL0118	DNA-directed RNA polymerase III subunit RPC8	0.498136712730493	0.161417079190954	0.578565701322753	NA	aag03020	KFB51302.1 AGAP01	XP_001661998.1DNA-directed RNA polymerase III subunit RPC8
AAEL0029	uncharacterized LOC5576728	0.498296589611724	0.140663299543413	0.551740700084605	NA	NA	KXJ80792.1 hypothet	XP_001656236.2uncharacterized protein LOC5576728
AAEL0091	NA	0.50002993296661	0.0612958351796896	0.386518366788521	NA	NA	XP_019531928.1 PRE	XP_019553426.1 inner centromere protein B-like
AAEL0112	trafficking protein particle complex subunit 2-like protein	0.500084986799168	0.0154669151235585	0.201125113930603	NA	NA	KFB51459.1 AGAP00	XP_001661521.1retinol dehydrogenase 12 isoform X2
AAEL0090	uncharacterized LOC5571441	0.501662864813967	0.0344243239613811	0.293579823070091	NA	NA	NA	XP_001659693.2uncharacterized protein LOC5571441
AAEL0050	zinc finger protein 568	0.501799712460391	0.0755146827301389	0.420516274980827	NA	NA	XP_019535586.1 PRE	XP_001650213.1zinc finger protein 568
AAEL0011	probable methylmalonate-semialdehyde dehydrogenase [acylating], mitc	0.501951076362487	0.000299210986189051	0.0211740264428904	00640 ; 00280	aag01100 ; aag	KFB35036.1 AGAP00	XP_001652386.1probable methylmalonate-semialdehyde dehydrogenase [acylating], mitochondrial
AAEL0123	NA	0.502928397124571	0.15285466022307	0.568113296551786	NA	NA	KXJ76705.1 hypothet	XP_001662493.2lipase 1
AAEL0016	chymotrypsin-2	0.503262255696529	0.0294362285888359	0.277248900178575	NA	NA	KXJ83563.1 hypothet	XP_019557747.1 chymotrypsin-2-like
AAEL0133	phosphatidate cytidylyltransferase, mitochondrial	0.503931267871054	0.031370775009069	0.284127826410879	00564 ; 04070	NA	NA	XP_001663588.1phosphatidate cytidylyltransferase, mitochondrial
AAEL0100	uncharacterized LOC5572870	0.505373918414186	0.00654630371899754	0.132685213264791	NA	NA	NA	XP_001660630.2uncharacterized protein LOC5572870
AAEL0280	NA	0.50538933516299	0.0881241218143546	0.453787447641685	NA	NA	NA	NA
AAEL0010	myelin transcription factor 1-like protein	0.505896326629587	0.207693857299907	0.639832374753494	NA	NA	KXJ75487.1 hypothet	XP_021694946.1myelin transcription factor 1-like protein
AAEL0045	uncharacterized LOC5564993	0.50594706402686	0.00455951806565125	0.105525821267499	NA	NA	ACA05582.1 gambici	AAL76025.1putative secreted protein
AAEL0002	J domain-containing protein CG6693	0.506292113469456	0.00525286628505517	0.115915768717213	NA	NA	XP_001846201.1 con	XP_001660148.2J domain-containing protein CG6693

AAEL0077(	malignant T-cell-amplified sequence 1 homolog	0.506550179377832	0.0243533374268781	0.255291093250631	NA	NA	ETN66615.1 mct-1 pr	XP_001658619.1malignant T-cell-amplified sequence 1 homolog
AAEL0076(	trypsin 5G1-like	0.50722180637287	0.524760049993324	0.865680248818465	NA	NA	NA	XP_001658491.2trypsin 5G1-like
AAEL0023	protein trachealess	0.507642317610964	0.175999541111631	0.600722469328349	NA	NA	KXJ71775.1 hypothet	XP_021694596.1protein trachealess
AAEL0268	NA	0.507937833176146	0.0747668456971486	0.419458473238027	NA	NA	NA	XP_021698909.1uncharacterized protein LOC110676221
AAEL0075	vesicle transport protein USE1	0.508104999815546	0.0887074478573108	0.455313532016544	NA	aag04130	XP_001841767.1 Use	XP_001652813.2vesicle transport protein USE1 isoform X1
AAEL0271(	NA	0.510611855804203	0.00439129926041364	0.103594442242845	NA	NA	NA	XP_021712623.1angiotensin-converting enzyme
AAEL0090	U6 snRNA phosphodiesterase	0.512461105740334	0.0632550007481625	0.392761482378267	NA	NA	KXJ78759.1 hypothet	XP_001659707.2U6 snRNA phosphodiesterase
AAEL0213(	NA	0.512666925186966	0.12109995422173	0.523190802729414	NA	NA	NA	XP_021696077.1transcription factor grauzone-like
AAEL0035	ubiquitin carboxyl-terminal hydrolase nonstop	0.512819901025705	0.0649253813266841	0.39730198438886	NA	NA	KXJ82566.1 hypothet	XP_021706754.1ubiquitin carboxyl-terminal hydrolase nonstop
AAEL0051	protein NDUFAF4 homolog	0.51297279784762	0.0101283142626972	0.16237064084554	NA	NA	ETN61404.1 hypothe	XP_001650507.1protein NDUFAF4 homolog
AAEL0258	NA	0.513757538297335	0.280265698543152	0.714405805538527	NA	NA	NA	NA
AAEL0052	growth hormone-regulated TBC protein 1-A	0.514192312020284	0.00885501386484439	0.152533826983712	NA	NA	KXJ73249.1 hypothet	XP_001650538.2growth hormone-regulated TBC protein 1-A
AAEL0037	coiled-coil domain-containing protein 102A	0.514193795500174	0.044908666576672	0.335702315059737	NA	NA	XP_019554929.1 PRE	XP_001664051.2coiled-coil domain-containing protein 102A isoform X3
AAEL0118	nucleolar complex protein 3 homolog	0.514237652556093	0.00111430086184105	0.04722490950323	NA	NA	CRK98008.1 CLUMA_	XP_001661948.1nucleolar complex protein 3 homolog
AAEL0195	NA	0.514631023171482	0.000770149137397105	0.0378693050264971	NA	NA	NA	XP_021700663.1dual specificity tyrosine-phosphorylation-regulated kinase 2 isoform X4
AAEL0008	glutactin	0.515100179593422	0.0662166857615106	0.399054441537196	NA	NA	KXJ82747.1 hypothet	XP_019524858.1 glutactin-like
AAEL0125(	uncharacterized LOC5576404	0.515269964212282	0.0313635753492175	0.284127826410879	NA	NA	KXJ73879.1 hypothet	XP_019548615.1 probable serine/threonine-protein kinase mps1 isoform X2
AAEL0276	NA	0.516749227487992	0.0604728070448095	0.384941803020695	NA	NA	NA	XP_021693770.1zinc finger protein 652-A
AAEL0063	glutamyl aminopeptidase	0.516832556611594	0.000682264907989924	0.0352707959737263	NA	NA	KXJ83574.1 hypothet	XP_021706992.1glutamyl aminopeptidase
AAEL0202	NA	0.519561902384786	0.0300992155103891	0.280053570401012	NA	NA	NA	NA
AAEL0224	NA	0.521697694706648	0.0014988841207004	0.0562125780306163	NA	NA	NA	NA
AAEL0276(	NA	0.524079816366958	0.00562390244801463	0.120339965887477	NA	NA	NA	ABF18515.1putative conserved protein
AAEL0072	transmembrane protein 19	0.524648000418325	0.000530143091999822	0.0297461995889168	NA	NA	ETN60539.1 transme	XP_021709806.1transmembrane protein 19 isoform X1
AAEL0134	synaptic vesicle 2-related protein	0.525117560862011	0.167735893822665	0.587554508928878	NA	NA	NA	XP_021695967.1synaptic vesicle 2-related protein isoform X3
AAEL0116	zinc finger and BTB domain-containing protein 24	0.525525282155852	0.134373883359148	0.542662316284181	NA	NA	XP_019547949.1 PRE	XP_021705081.1transcription factor btd isoform X1
AAEL0118	aryl hydrocarbon receptor	0.527449618139536	0.0736586210865129	0.417913179290884	NA	NA	XP_019541156.1 PRE	XP_021710004.1aryl hydrocarbon receptor isoform X2
AAEL0014	uncharacterized LOC5570918	0.531130035842155	0.0286077662136919	0.274217359060826	NA	NA	KXJ80977.1 hypothet	XP_021700626.1uncharacterized protein LOC5570918 isoform X2
AAEL0033	NHP2-like protein 1 homolog	0.532716170746406	0.000128807535362094	0.0133178307910335	NA	aag03040 ; aag	ETN64755.1 ribosom	XP_001656680.1NHP2-like protein 1 homolog
AAEL0017	cyclin-related protein FAM58A	0.532954132481701	0.000236566403035787	0.01884379090581	NA	NA	KFB48029.1 AGAP00	XP_001653962.1cyclin-related protein FAM58A
AAEL0113	borealin	0.533280117485912	0.0738001430501725	0.417913179290884	NA	NA	XP_019548366.1 PRE	XP_001655256.2borealin isoform X2
AAEL0103	uncharacterized LOC5573304	0.535370903904862	0.0539471067322867	0.366668118991083	NA	NA	NA	XP_001654524.1uncharacterized protein LOC5573304
AAEL0077(	uncharacterized LOC5569528	0.535451211928698	0.350079128661171	0.768103991878897	NA	NA	XP_001844079.1 con	XP_001652839.2uncharacterized protein LOC5569528
AAEL0084	protein PBDC1	0.535571426306238	0.0122380226560676	0.179285423221345	NA	NA	KFB36548.1 AGAP00	XP_001659260.2protein PBDC1
AAEL0097	2-hydroxyacyl-CoA lyase 1	0.53570931827862	0.00116178214992058	0.047691139601999	NA	aag04146	NA	XP_001660326.12-hydroxyacyl-CoA lyase 1
AAEL0120	lanC-like protein 3 homolog	0.536229942512651	0.0667387938118311	0.399540230684495	NA	NA	KXJ76218.1 hypothet	XP_001662225.1lanC-like protein 3 homolog isoform X1
AAEL0104	uncharacterized LOC5573325	0.536243196759399	0.0804881550499672	0.433079891352508	NA	NA	NA	XP_001654545.2uncharacterized protein LOC5573325
AAEL0232(	NA	0.536337067936747	0.000375311015937174	0.0239834164489853	NA	NA	NA	XP_021699960.139S ribosomal protein L52, mitochondrial
AAEL0247	NA	0.536501857679982	0.0809715212850544	0.433079891352508	NA	NA	NA	NA
AAEL0205	NA	0.536650674971272	0.149657979341178	0.565105542550216	NA	NA	NA	XP_021707432.1transmembrane and TPR repeat-containing protein 1
AAEL0073	serine-rich adhesin for platelets	0.537044561529682	0.0999925329832112	0.479706814084761	NA	NA	KXJ83707.1 hypothet	XP_001658279.2uncharacterized protein LOC5569018
AAEL0200	NA	0.537112618180007	0.121304472591214	0.523315409097589	NA	NA	NA	XP_021703239.1zinc finger protein 883
AAEL0001	coiled-coil domain-containing protein 86	0.538833923416024	0.0051837469610318	0.115172122080007	NA	NA	KXJ71307.1 hypothet	XP_001658904.1coiled-coil domain-containing protein 86
AAEL0019	uncharacterized LOC5573190	0.53884561601066	0.000136192044636793	0.0137718592829425	NA	NA	XP_001237928.2 AG	XP_001660805.1uncharacterized protein LOC5573190
AAEL0011	uncharacterized LOC5568562	0.538976601180611	0.00501709092315595	0.112922895714546	NA	NA	KFB44349.1 AGAP00	KFB44349.1AGAP005178-like protein
AAEL0201(	NA	0.54115071524	0.000728934704161464	0.0369889266214859	NA	NA	NA	NA
AAEL0103	UDP-glucuronosyltransferase 1-6	0.54207131212971	0.239391832873544	0.674489787538993	NA	NA	NA	XP_021699048.1UDP-glucuronosyltransferase 2C1 isoform X1
AAEL0033	acylpyruvase FAHD1, mitochondrial	0.542605815823758	0.000248318232289299	0.0191849586638347	NA	aag01100 ; aag	XP_001867599.1 fun	XP_001656748.2acylpyruvase FAHD1, mitochondrial
AAEL0051(	uncharacterized LOC5566027	0.542728597041787	0.0870017802757065	0.45027580545391	NA	NA	KXJ84539.1 hypothet	XP_001650380.2uncharacterized protein LOC5566027
AAEL0182	NA	0.54354457269473	0.0958446364464455	0.468381489421238	NA	NA	NA	XP_021710847.1uncharacterized protein LOC5576761 isoform X2
AAEL0116	probable tyrosyl-DNA phosphodiesterase	0.544245899927363	0.0632402767814646	0.392761482378267	NA	NA	KXJ68209.1 hypothet	XP_021706145.1probable tyrosyl-DNA phosphodiesterase isoform X1
AAEL0085	ribonuclease 3	0.544896033985263	0.0137302812147905	0.187831676988403	NA	aag03008	KFB39415.1 AGAP00	XP_001653338.1ribonuclease 3
AAEL0280	NA	0.545133827754784	0.164612002123965	0.583247700156998	NA	NA	NA	XP_001656609.2uncharacterized protein LOC5577615
AAEL0271	NA	0.545714646337132	0.31338117074694	0.744189298893767	NA	NA	NA	NA
AAEL0080	neuronal acetylcholine receptor subunit alpha-7	0.545991226251444	0.19977302392242	0.633375309392567	NA	NA	XP_308042.3 AGAP0	XP_021700026.1neuronal acetylcholine receptor subunit alpha-7 isoform X1
AAEL0269	NA	0.546179562634366	0.179205825310316	0.604695248815902	NA	NA	NA	XP_001657339.1protein windbeutel
AAEL0267(	NA	0.54772758050478	0.100090967511708	0.479706814084761	NA	NA	NA	XP_021697822.1probable cytochrome P450 305a1
AAEL0084	protein PRY1	0.547888047990139	0.13420902224334	0.542662316284181	NA	NA	XP_019539610.1 PRE	XP_001659274.2protein PRY1
AAEL0055	Fanconi anemia group I protein	0.548942050945321	0.090193583085642	0.458542183179048	NA	aag03460	KXJ71417.1 hypothet	XP_021701733.1Fanconi anemia group I protein
AAEL0102	ribonuclease H2 subunit C	0.549316161451693	0.0584225410910894	0.378861327075549	NA	aag03030	NA	XP_001654399.1ribonuclease H2 subunit C
AAEL0028	protein C3orf33 homolog	0.549501120685216	0.00164673441486843	0.0606130003424772	NA	NA	XP_001845304.1 con	XP_001656095.2protein C3orf33 homolog
AAEL0030	UDP-glucuronosyltransferase 2B13	0.549660723821631	0.115871790938604	0.512764772622422	NA	NA	KXJ82204.1 hypothet	XP_001663165.2UDP-glucuronosyltransferase 2B13
AAEL0095	mucin-12	0.549716745854628	0.230159877707518	0.663064401691905	NA	NA	NA	XP_021704675.1mucin-12 isoform X1
AAEL0138	location of vulva defective 1	0.550637069615943	0.128387300336973	0.535049122410476	NA	NA	NA	XP_001664001.2location of vulva defective 1
AAEL0076	zinc finger protein 160	0.550685521055977	0.124505685781055	0.528664388933982	NA	NA	KXJ62477.1 hypothet	XP_001658563.1zinc finger protein 160
AAEL0005	ATPase WRNIP1	0.551026295201834	0.00946927490222306	0.158773875613038	NA	NA	ETN58327.1 werner I	XP_001647950.1ATPase WRNIP1
AAEL0064(	uncharacterized LOC5567974	0.551331721175666	0.093434666424914	0.464498001319318	NA	NA	KXJ83467.1 hypothet	XP_001657796.2uncharacterized protein LOC5567974
AAEL0033	zinc finger protein 267	0.551345017074399	0.0431309983899711	0.327738602134198	NA	NA	KXJ72860.1 hypothet	XP_001656681.2zinc finger protein 267
AAEL0236	NA	0.551620610505136	0.165639930997873	0.584439853413553	NA	NA	NA	XP_021699444.1uncharacterized protein LOC5569196
AAEL0045	protein transport protein Sec61 subunit alpha	0.552187605146222	0.000649074123679897	0.0341621442326704	NA	aag04141 ; aag	KXJ74290.1 hypothet	XP_001649329.1protein transport protein Sec61 subunit alpha
AAEL0142	ribonuclease H2 subunit B	0.552652983603411	0.0787889554610009	0.429137779293678	NA	aag03030	NA	XP_001648343.1ribonuclease H2 subunit B
AAEL0091	major facilitator superfamily domain-containing protein 8	0.553331548133864	8,09E+09	0.0099271231753841	NA	aag04142	NA	XP_001659802.1major facilitator superfamily domain-containing protein 8 isoform X2
AAEL0275	NA	0.553650003338181	0.0745120472958755	0.419169079991884	NA	NA	NA	NA
AAEL0030	dolichyl-phosphate beta-glucosyltransferase	0.553990778309141	0.00507517245267052	0.113906675388961	510	aag01100 ; aag	ETN58201.1 dolichyl-	XP_001663059.1dolichyl-phosphate beta-glucosyltransferase
AAEL0011	BLOC-1-related complex subunit 6	0.554745320348224	0.108014687167008	0.494284290519081	NA	NA	KXJ76274.1 hypothet	XP_001658185.1BLOC-1-related complex subunit 6 isoform X1
AAEL0009	leucine-zipper-like transcriptional regulator 1	0.555380875302349	0.0137755394703712	0.187831676988403	NA	NA	XP_019525020.1 PRE	XP_001651641.2leucine-zipper-like transcriptional regulator 1
AAEL0034	sialin	0.557760621179836	0.00690502295648544	0.137411930736898	NA	NA	KXJ75969.1 hypothet	XP_019526976.1 sialin
AAEL0125(	muscle M-line assembly protein unc-89	0.557783177376568	0.0246504349017596	0.257472533446075	NA	NA	NA	XP_001656117.2muscle M-line assembly protein unc-89 isoform X2
AAEL0253	NA	0.558976465380676	0.125782108684398	0.530481545423179	NA	NA	NA	XP_021706789.1coiled-coil domain-containing protein 174-like
AAEL0063	sulfotransferase family cytosolic 1B member 1	0.559165825601502	0.00560441618017463	0.120214073869387	NA	NA	KXJ69441.1 hypothet	XP_001651916.1sulfotransferase family cytosolic 1B member 1
AAEL0234	NA	0.560327224657587	0.232052733429477	0.666047801939502	NA	NA	NA	NA
AAEL0118(	zinc finger protein 260	0.561170870375146	0.177090130974403	0.602182613972581	NA	NA	XP_019534363.1 PRE	XP_021706403.1zinc finger protein 260
AAEL0233	NA	0.563456604911593	0.22774430791068	0.66175420142127	NA	NA	NA	XP_021705781.1mucin-5AC isoform X3

AAEL0200(	NA	0.563614108930104	0.0290202686881503	0.275227088745645	NA	NA	NA	XP_021708272.1prominin-1 isoform X3
AAEL0079ε	mucin-5AC	0.565298440089661	0.119335054596307	0.518470808496326	NA	NA	XP_019543259.1 PRE	XP_021696254.1mucin-5AC isoform X2
AAEL0172ε	uncharacterized LOC23687703	0.565773503936756	0.0741913777514099	0.418818002133309	NA	NA	NA	XP_021698490.1uncharacterized protein LOC23687703
AAEL0027€	DTW domain-containing protein 2	0.56662330392591	0.026662517000388	0.266963842267995	NA	NA	KXJ80735.1 hypothet	XP_001655946.2DTW domain-containing protein 2
AAEL0140(	uncharacterized LOC5579063	0.567247163735989	0.0941563978594546	0.464823706598816	NA	NA	NA	XP_001664221.1uncharacterized protein LOC5579063
AAEL00567	NA	0.567329362559539	0.221983170098983	0.654118491874251	NA	NA	KXJ73542.1 hypothet	XP_001651321.2putative mediator of RNA polymerase II transcription subunit 12
AAEL00167	serine protease SP24D	0.568181856718415	0.0832321050545158	0.438013925780757	NA	NA	KXJ77072.1 hypothet	XP_001659962.1serine protease SP24D
AAEL01362	zinc finger protein 436	0.568321985097482	0.078801190541757	0.429137779293678	NA	NA	NA	XP_021701688.1zinc finger protein 436
AAEL00862	cyclin-dependent kinase 1	0.568952315565229	0.0305125255214682	0.281224109837052	NA	NA	KFB39955.1 AGAP00	XP_001653367.1cyclin-dependent kinase 1
AAEL0029ε	uncharacterized LOC5580286	0.569418502366063	0.085237792067156	0.443641494684372	NA	NA	XP_001842359.1 con	XP_001662913.1uncharacterized protein LOC5580286
AAEL0052ε	uncharacterized LOC5566213	0.569491087601279	0.0252504668291265	0.259075173517489	NA	NA	KXJ83423.1 hypothet	XP_001650619.1uncharacterized protein LOC5566213 isoform X1
AAEL01064	nose resistant to fluoxetine protein 6	0.570661302618287	0.00852040226170875	0.149464458238032	NA	NA	KXJ74719.1 hypothet	XP_001660984.1nose resistant to fluoxetine protein 6
AAEL0058ε	DNA mismatch repair protein Mlh1	0.571066960622764	0.136416622315198	0.545589928345625	NA	aag03460 ; aag	KXJ82304.1 hypothet	XP_001651509.2DNA mismatch repair protein Mlh1
AAEL0067ε	uncharacterized LOC5568298	0.572068862965589	0.10334562079808	0.486189367374199	NA	NA	KFB36087.1 AGAP00	XP_019557659.1 cylicin-1-like
AAEL0085ε	kinase suppressor of Ras 2	0.572940178137542	0.0123685380669775	0.179504888805347	NA	aag04013	XP_019535352.2 PRE	XP_021705516.1kinase suppressor of Ras 2
AAEL0048ε	adipocyte plasma membrane-associated protein	0.573811558496129	0.0726253365657671	0.414063412068271	NA	NA	KXJ80335.1 hypothet	XP_001649961.1adipocyte plasma membrane-associated protein
AAEL00421	PDZ domain-containing protein 8	0.574299069153771	0.071873407426102	0.411912175099304	NA	NA	KFB53493.1 AGAP00	XP_001648694.1PDZ domain-containing protein 8
AAEL0135(	phenoloxidase 2	0.575484738753814	0.00273841480766662	0.0807656828850905	NA	NA	NA	XP_001663685.2phenoloxidase 2
AAEL0274ε	NA	0.577160929759915	0.174905754653864	0.598320726514817	NA	NA	NA	NA
AAEL0102ε	uncharacterized LOC5580045	0.578833597402464	0.119165164388522	0.518222042865395	NA	NA	NA	XP_001660696.2uncharacterized protein LOC5580045
AAEL01434	caspase	0.578873594733999	0.00960615608275979	0.159271798691091	NA	NA	NA	XP_001648537.2caspase-1 isoform X1
AAEL0279(	NA	0.57926160963188	0.0757072488022159	0.420687260554342	NA	NA	NA	NA
AAEL0019ε	zinc finger protein 808	0.579820782388343	0.112265003657612	0.503547757511443	NA	NA	XP_001862329.1 zinc	XP_001654305.2zinc finger protein 808
AAEL0078ε	zinc finger protein 665	0.582678707452856	0.160275078436525	0.577674739374419	NA	NA	KXJ82589.1 hypothet	XP_021704071.1zinc finger protein 665
AAEL0089€	regulator of telomere elongation helicase 1 homolog	0.583008373545982	0.0220433295213925	0.242070925206103	NA	NA	XP_001845338.1 fan	XP_001653621.2regulator of telomere elongation helicase 1 homolog
AAEL0197ε	NA	0.585682623474887	0.0599366979051319	0.383636203860763	NA	NA	NA	XP_021704428.1rho GTPase-activating protein 15 isoform X2
AAEL0055€	PR domain zinc finger protein 5	0.586197228046153	0.156347052871481	0.574474189691589	NA	NA	XP_019536364.1 PRE	XP_021711342.1PR domain zinc finger protein 5
AAEL0244ε	NA	0.586776525829395	0.0746800708269067	0.419283716747526	NA	NA	NA	ABF18067.1salivary secreted proline-rich mucin
AAEL00811	separase	0.587419381067042	0.1329267131742	0.540278981726584	NA	NA	KXJ71020.1 hypothet	XP_019551877.1 separase-like isoform X1
AAEL01477	uncharacterized LOC5565252	0.58811410008346	0.050444204364653	0.357342239078935	NA	aag03460 ; aag	NA	XP_001649592.2uncharacterized protein LOC5565252
AAEL01137	wee1-like protein kinase	0.588409810482164	0.00831370571255458	0.147688648584802	4660	NA	KXJ77197.1 hypothet	XP_021700707.1wee1-like protein kinase
AAEL0036ε	gastrula zinc finger protein XlCGF26.1	0.588419496743612	0.0683807285613733	0.401556773593974	NA	NA	KXJ74994.1 hypothet	XP_001657159.2gastrula zinc finger protein XlCGF26.1
AAEL0006ε	nuclear fragile X mental retardation-interacting protein 1	0.589487150861234	0.0213844772733116	0.238631723223164	NA	NA	ETN59304.1 hypothe	XP_001650253.2nuclear fragile X mental retardation-interacting protein 1
AAEL01311	protein G12	0.59025748697779	0.336293250375164	0.758445425575872	NA	NA	NA	XP_001656375.1protein G12
AAEL0236(	NA	0.590292149856126	0.0095588705813544	0.158773875613038	NA	aag03440 ; aag	NA	XP_021704981.1double-strand break repair protein MRE11
AAEL01141	high mobility group protein D	0.59077865117553	0.000106633461146141	0.0116814417793666	NA	NA	ETN63177.1 High mo	XP_001655340.1high mobility group protein D
AAEL0002€	zinc finger protein 684	0.591226276045671	0.0692058102984012	0.403786365070033	NA	NA	KXJ74459.1 hypothet	XP_001654765.2zinc finger protein 684
AAEL0258(	NA	0.591498107048862	3,32E+09	0.00499914174438099	NA	NA	NA	XP_021708461.15'-nucleotidase domain-containing protein 1
AAEL0118ε	zinc finger protein 43	0.591701621805468	0.249312007982525	0.681571330200593	NA	NA	NA	XP_021696062.1zinc finger protein 43 isoform X1
AAEL0111ε	sorbitol dehydrogenase	0.593990461099594	0.233386041223357	0.666962220912214	NA	aag01100 ; aag	KXJ69735.1 hypothet	XP_001655104.1sorbitol dehydrogenase
AAEL0275ε	NA	0.594997778439626	0.164723278230859	0.583247700156998	NA	NA	NA	XP_021694213.1muscle M-line assembly protein unc-89
AAEL0027ε	putative peptidyl-tRNA hydrolase PTRHD1	0.595048618736512	0.00798482690937653	0.144528409372715	NA	NA	KXJ68923.1 hypothet	XP_001655961.1putative peptidyl-tRNA hydrolase PTRHD1
AAEL01454	protein-glucosylgalactosylhydroxylysine glucosidase	0.59612923841817	0.104400065441897	0.487659595023521	NA	NA	NA	XP_001648961.1protein-glucosylgalactosylhydroxylysine glucosidase
AAEL00744	zinc finger protein 436	0.596935801997932	0.0544580509528152	0.367469712613638	NA	NA	KXJ72984.1 hypothet	XP_021701065.1zinc finger protein 436
AAEL02702	NA	0.597513565667246	0.103455464876564	0.486208982530205	NA	NA	NA	NA
AAEL0196€	NA	0.598568264433585	0.095943332794718	0.46861494075212	NA	NA	NA	XP_021693913.1zinc finger protein 431-like
AAEL0060ε	DNA repair protein RAD51 homolog 1	0.600719221572699	0.0347626122286815	0.295097377978161	NA	aag03460 ; aag	KFB52705.1 AGAP01	XP_001657421.1DNA repair protein RAD51 homolog 1
AAEL0050ε	tubulin beta chain	0.600835729843309	9,09E+09	0.0110005467443923	NA	aag04145	KXJ73036.1 hypothet	XP_001650331.1tubulin beta chain
AAEL0074€	zinc finger protein 223	0.600925906129689	0.148447406612447	0.562679383080301	NA	NA	KXJ69977.1 hypothet	XP_001658377.2zinc finger protein 223
AAEL00172	G-protein coupled receptor 143	0.604703560870549	0.00951834217436298	0.158773875613038	NA	NA	KXJ80144.1 hypothet	XP_001653825.3G-protein coupled receptor 143
AAEL01817	NA	0.60595516891603	0.00344402273076746	0.0901744430841277	NA	NA	NA	XP_021704949.1spermine oxidase
AAEL00744	STE20-related kinase adapter protein alpha	0.606930243530837	0.0142549018522843	0.19121517032758	NA	aag04150	XP_019528387.1 PRE	XP_021701050.1STE20-related kinase adapter protein alpha
AAEL0146(	NA	0.608289901305301	5,62E+08	0.00728611912788509	NA	NA	NA	XP_001649108.2probable cytochrome P450 9f2
AAEL0080€	transmembrane protein 208	0.609863796987917	0.00131337847272591	0.0509945515022101	NA	NA	XP_013098100.1 PRE	XP_001658877.1transmembrane protein 208
AAEL01741	15-hydroxyprostaglandin dehydrogenase [NAD(+)]	0.61015967885565	0.0967696225283275	0.470633908275356	NA	NA	NA	XP_011493201.115-hydroxyprostaglandin dehydrogenase [NAD(+)]
AAEL0199ε	NA	0.61087252757474	0.00569864052648616	0.120826935771257	NA	NA	NA	ABF18177.1probable truncated 34 kDa family member
AAEL0002ε	zinc finger protein 519	0.61138921886812	0.0854693855228344	0.444093328956026	NA	NA	KXJ74461.1 hypothet	XP_001654768.2zinc finger protein 519
AAEL0290€	NA	0.614428999946661	0.00213521078492271	0.0727761081152129	NA	NA	NA	EAT41683.1AAEL006701
AAEL0233(	NA	0.615182872249407	0.042349440701074	0.323939778330243	NA	NA	NA	NA
AAEL00872	sister chromatid cohesion protein DCC1	0.615483760592505	0.146140309426432	0.560092931004593	NA	NA	KXJ73534.1 hypothet	XP_001653438.1sister chromatid cohesion protein DCC1
AAEL01362	trypsin eta	0.615556892207871	0.0272323165238797	0.268836665026084	NA	NA	NA	XP_001656898.2trypsin eta
AAEL02214	NA	0.615718585825347	0.0849605707459046	0.442448880590727	NA	NA	NA	NA
AAEL01064	RNA-binding protein 42	0.615898070545302	0.0660137664283247	0.398594933512758	NA	NA	KXJ70274.1 hypothet	XP_001660974.1RNA-binding protein 42 isoform X2
AAEL0170ε	RNA-binding protein Rsf1	0.618588684836365	2,28E+09	0.00389254965361311	NA	NA	NA	XP_011493524.1RNA-binding protein Rsf1
AAEL00511	uncharacterized LOC5566019	0.62245741051159	0.052548641198626	0.362633125106993	NA	NA	KXJ84539.1 hypothet	XP_001650381.1uncharacterized protein LOC5566019
AAEL0024ε	retinol dehydrogenase 13	0.624114993747593	0.00791944361642093	0.144528409372715	NA	NA	KXJ81070.1 hypothet	XP_001655347.2retinol dehydrogenase 13
AAEL0201ε	NA	0.624509933546344	0.256679192484314	0.689020399428431	NA	NA	NA	NA
AAEL02721	NA	0.629451233215809	0.0969704292642985	0.470633908275356	NA	NA	NA	NA
AAEL00772	uncharacterized LOC5579981	0.630848903242502	0.00165412937371899	0.0606426234938731	NA	NA	KXJ83226.1 hypothet	XP_001658609.2uncharacterized protein LOC5579981
AAEL0030ε	UDP-glucuronosyltransferase 2B15	0.631303337972333	0.0278378536962325	0.271255206147924	NA	NA	XP_001841708.1 UDI	XP_001663066.1UDP-glucuronosyltransferase 2B15 isoform X1
AAEL0013ε	uncharacterized LOC5570349	0.63221847657065	0.0253511914847209	0.259779135904679	NA	NA	KFB35091.1 AGAP00	KFB35091.1AGAP005141-like protein
AAEL02197	NA	0.633564273283388	0.101796338483711	0.483848092317719	NA	NA	NA	XP_021697488.1GDNF-inducible zinc finger protein 1
AAEL0227ε	NA	0.635189122257223	0.101968521683945	0.484416281123213	NA	NA	NA	XP_021696198.1uncharacterized protein LOC5566491
AAEL00921	mitochondrial thiamine pyrophosphate carrier	0.636590815682401	0.0309372554246221	0.282425222636282	NA	NA	NA	XP_001659842.2mitochondrial thiamine pyrophosphate carrier
AAEL0061ε	probable chitinase 2	0.637512567941754	0.0295674293921367	0.277915715287479	520	NA	XP_019545134.1 PRE	XP_021707618.1probable chitinase 2
AAEL0049ε	DNA fragmentation factor subunit beta	0.638063489804073	0.0605556144822952	0.384941803020695	NA	NA	KXJ76541.1 hypothet	XP_001650118.2DNA fragmentation factor subunit beta
AAEL01142	histone H3.3	0.638842922467493	0.0268492516691308	0.267459976661819	NA	NA	PIS88409.1 core histo	XP_001870860.1histone H3.2
AAEL0007ε	nuclear pore glycoprotein p62-like	0.638925904142651	0.0642285627333442	0.395485211242887	NA	NA	KXJ83650.1 hypothet	XP_019529810.1 nuclear pore glycoprotein p62-like
AAEL0044(	lysine-specific demethylase 8	0.641960771447311	0.100697507367187	0.48111031297656	NA	NA	XP_019542117.1 PRE	XP_001649080.2lysine-specific demethylase 8 isoform X2
AAEL0106ε	putative alpha-L-fucosidase	0.643475000350272	0.000332336298281029	0.0223223256699418	511	aag00511	KXJ79327.1 hypothet	XP_001661015.2putative alpha-L-fucosidase
AAEL01022	erythroid transcription factor	0.644502231081962	0.0329842065667038	0.285413533048672	NA	NA	NA	XP_021708599.1GATA-binding factor 2 isoform X1
AAEL0075ε	protein trunk	0.645995951632592	0.0187363514537984	0.223621149257916	NA	aag04013	KXJ82931.1 hypothet	XP_001658474.2protein trunk

AAEL02641	NA	0.646633396353232	0.037983886620774	0.307682856236235	NA	NA	NA	XP_021697487.1zinc finger protein 157
AAEL00048	exostosin-3	0.646955201710663	0.0019625231795256	0.0693618362591811	NA	aag01100 ; aag	KXJ81979.1 hypothet	XP_019532115.1 exostosin-3-like
AAEL01494	aprataxin-like protein	0.647930719373874	0.0214462546300323	0.238631723223164	NA	NA	NA	XP_001650061.2aprataxin-like protein isoform X2
AAEL02043	NA	0.649706861038911	0.0169228168964165	0.21100780634258	00670 ; 00260	NA	NA	XP_021697632.1sarcosine dehydrogenase, mitochondrial
AAEL01417	phosphopantothenate--cysteine ligase	0.654254817977716	0.00253237356053339	0.0776763383467608	770	aag01100 ; aag	NA	XP_021693543.1phosphopantothenate--cysteine ligase
AAEL01157	acidic fibroblast growth factor intracellular-binding protein	0.654978680793886	0.0322400633049395	0.284442054201394	NA	NA	XP_001868818.1 acic	XP_001661749.2acidic fibroblast growth factor intracellular-binding protein
AAEL01957	NA	0.655881196191264	0.0787022818589849	0.429137779293678	NA	NA	NA	XP_021703652.1transcriptional regulator ATRX homolog isoform X2
AAEL00161	RING finger protein 37	0.656686759877841	0.0226588398663357	0.245124275357034	NA	aag04120	XP_019558812.1 PRE	XP_001659743.2RING finger protein 37
AAEL00949	probable phosphomannomutase	0.659192855230886	0.00462086393614549	0.106569398346894	00051 ; 00520	aag01100 ; aag	NA	XP_001660162.2probable phosphomannomutase
AAEL02117	NA	0.660393825128286	0.049214590766119	0.352961636078127	NA	aag03020	NA	XP_021696983.1probable DNA-directed RNA polymerase III subunit RPC6
AAEL02051	NA	0.660651562649634	0.0827240529283851	0.437738203017251	NA	aag04146	NA	XP_021707230.1protein Mpv17-like
AAEL02535	NA	0.661364568629713	0.0545135469637172	0.367496244171354	NA	NA	NA	NA
AAEL01006	uncharacterized LOC5572842	0.663588788770415	0.0243892542876901	0.255291093250631	901	NA	NA	XP_021705818.1uncharacterized protein LOC5572842
AAEL00601	vesicle transport protein SFT2C	0.665075282836247	0.00312295321181266	0.0881515811506138	NA	NA	ETN64448.1 hypothe	XP_001651760.1vesicle transport protein SFT2C
AAEL00397	uncharacterized LOC5579808	0.665803931124972	0.121136886898616	0.523190802729414	901	NA	KXJ69619.1 hypothet	XP_001648121.2uncharacterized protein LOC5579808
AAEL00094	signal peptidase complex subunit 3	0.66613444283804	0.0288642773786626	0.274601260220884	NA	NA	ETN61422.1 microso	XP_001657476.1signal peptidase complex subunit 3
AAEL00684	uncharacterized LOC5568425	0.66620735477316	0.00305646828497022	0.0868074727107901	NA	aag04140	KXJ70255.1 hypothet	XP_019543497.1 nuclear receptor-binding factor 2
AAEL00727	peptidyl-prolyl cis-trans isomerase E	0.667197038236859	0.01332079029821	0.18599306889727	NA	aag03040	XP_001850759.1 pep	XP_001658271.2peptidyl-prolyl cis-trans isomerase E
AAEL00224	protein fuzzy homolog	0.667313037451399	0.187031980501255	0.614196367514984	NA	NA	XP_001845150.1 fuz	XP_001654982.2protein fuzzy homolog isoform X1
AAEL00988	dynein heavy chain 5, axonemal	0.667350407319591	0.462461690807196	0.83694823576051	NA	NA	NA	XP_001654085.2dynein heavy chain 5, axonemal
AAEL00742	craniofacial development protein 1	0.671366225395174	0.00551438445698703	0.119115882096701	NA	NA	KXJ84185.1 hypothet	XP_001658353.2craniofacial development protein 1
AAEL00975	leucine-rich repeat transmembrane neuronal protein 1	0.672384556613468	0.0430302529392245	0.327632237384908	NA	NA	NA	XP_001660345.2leucine-rich repeat transmembrane neuronal protein 1
AAEL00262	general odorant-binding protein 56d	0.673152155729331	0.0784777956899503	0.428723995967346	NA	NA	KXJ80438.1 hypothet	XP_001655718.2general odorant-binding protein 56d
AAEL00412	short-chain specific acyl-CoA dehydrogenase, mitochondrial	0.67519674639757	0.00476236577651273	0.108742654777841	NA	aag01100 ; aag	KXJ73656.1 hypothet	XP_001648504.1short-chain specific acyl-CoA dehydrogenase, mitochondrial
AAEL00622	uncharacterized LOC5567643	0.68348240446622	0.0638433098165572	0.394550797412337	NA	NA	KXJ68038.1 hypothet	XP_021706882.1uncharacterized protein LOC5567643
AAEL00650	tRNA-splicing endonuclease subunit Sen2	0.684759092253655	0.0611531170507305	0.386196065431147	NA	NA	KXJ79508.1 hypothet	XP_001652026.2tRNA-splicing endonuclease subunit Sen2
AAEL00816	arrestin domain-containing protein 2	0.686450344214678	0.0400500438096688	0.31441514682591	NA	NA	KXJ71065.1 hypothet	XP_001659000.1arrestin domain-containing protein 2
AAEL02289	NA	0.687028780188186	0.0431690805487912	0.327757326080839	NA	NA	NA	XP_021712795.1uncharacterized protein LOC5572758
AAEL01330	UNC93-like protein MFSD11	0.687249903889582	0.000157819375255719	0.0151039840659274	NA	NA	NA	XP_001663450.2UNC93-like protein MFSD11
AAEL00688	growth arrest and DNA damage-inducible protein GADD45 alpha	0.693404602531358	0.00541009935877806	0.117414467687443	NA	aag04068	KXJ79566.1 hypothet	XP_021695984.1growth arrest and DNA damage-inducible protein GADD45 alpha
AAEL00712	LIM/homeobox protein Lhx3	0.699343683006541	0.00796385187127093	0.144528409372715	NA	NA	KXJ73420.1 hypothet	XP_001652548.1LIM/homeobox protein Lhx3 isoform X4
AAEL02234	NA	0.701264343113443	0.0767170340976549	0.423485391581656	NA	NA	NA	NA
AAEL02441	NA	0.7015953516523	0.0113260384960808	0.172681499108771	970	NA	NA	XP_021709815.1alanyl-tRNA editing protein Aarsd1-A
AAEL01286	uncharacterized LOC5576923	0.704945976106699	0.00700349875196751	0.137411930736898	NA	NA	NA	XP_001663020.2uncharacterized protein LOC5576923
AAEL02012	NA	0.704993114803084	0.0419553499881207	0.323344330477962	NA	NA	NA	CRK98979.1CLUMA_CG012201, isoform A
AAEL01971	NA	0.705776712925156	0.0543542662576293	0.367469712613638	NA	NA	NA	XP_021709415.1small conductance calcium-activated potassium channel protein isoform X1
AAEL01368	SAGA-associated factor 29	0.705893350829864	0.00888482191515642	0.152533826983712	NA	NA	NA	XP_001660055.1SAGA-associated factor 29
AAEL02515	NA	0.711188096576923	0.0114329718487414	0.172833431546296	NA	NA	NA	NA
AAEL01300	uncharacterized LOC5577108	0.711501353896041	0.00375504478524	0.0953776289296528	NA	NA	NA	XP_021701930.1uncharacterized protein LOC5577108
AAEL02683	NA	0.712303186091647	0.133855075587142	0.542614275573958	NA	NA	NA	XP_021701426.1myelin transcription factor 1 isoform X2
AAEL01073	CCR4-NOT transcription complex subunit 10	0.714370295160389	0.00318011366822486	0.0886769878030459	NA	aag03018	KXJ84054.1 hypothet	XP_001661053.1CCR4-NOT transcription complex subunit 10
AAEL01131	juvenile hormone epoxide hydrolase	0.715846826811194	0.0802868592142101	0.433079891352508	NA	aag00981	KXJ79035.1 hypothet	XP_001661590.1juvenile hormone epoxide hydrolase
AAEL01108	uncharacterized LOC5574367	0.718685878211041	0.00803055507803606	0.14461285289254	NA	NA	KXJ70008.1 hypothet	XP_001661403.1uncharacterized protein LOC5574367
AAEL01064	NA	0.726766913269241	0.0100609770645551	0.161638111035661	00983 ; 00230 ; 00480 ;	NA	KXJ70271.1 hypothet	XP_001660977.2ribonucleoside-diphosphate reductase large subunit
AAEL01136	zinc finger protein 567	0.728884498332761	0.0228995131034727	0.245803773576383	NA	NA	KXJ77489.1 hypothet	XP_001655302.1zinc finger protein 567
AAEL00839	uncharacterized LOC110680891	0.737390769504765	0.000649682160477865	0.0341621442326704	NA	NA	XP_001843842.1 con	XP_021712374.1uncharacterized protein LOC110680891
AAEL01125	aprataxin and PNK-like factor	0.739371430080279	1,81E+09	0.00333351334102285	NA	NA	NA	XP_001661528.2aprataxin and PNK-like factor isoform X2
AAEL01426	zinc finger protein 501	0.740667204758205	0.143536819754823	0.555843555723525	NA	NA	NA	XP_001648322.2zinc finger protein 501
AAEL02384	NA	0.741690744313846	0.0631139371639545	0.39272109689124	NA	NA	NA	XP_021712591.1zinc finger protein weckle-like
AAEL00314	retinoblastoma-binding protein 5 homolog	0.741706945915319	0.00130389706368162	0.0508409355084671	NA	NA	XP_001856893.1 reti	XP_001656334.1retinoblastoma-binding protein 5 homolog
AAEL00339	uncharacterized LOC5577987	0.744122435558794	0.0508351740876579	0.358731036775022	NA	NA	KXJ68705.1 hypothet	XP_001656724.2uncharacterized protein LOC5577987
AAEL00828	cuticle protein CP14.6	0.745472728192489	0.0475599108545095	0.348167302850594	NA	NA	ETN58133.1 cuticula	XP_001659105.1cuticle protein CP14.6
AAEL00343	caspase	0.746463313028063	0.0324261830019086	0.28463170766102	NA	aag04214 ; aag	KXJ71200.1 hypothet	XP_019528553.1 caspase-like
AAEL00619	mediator of RNA polymerase II transcription subunit 4	0.750585960516743	0.0227579642803645	0.245124275357034	NA	NA	KFB36470.1 AGAP00	XP_001657572.1mediator of RNA polymerase II transcription subunit 4
AAEL00976	kelch-like protein 40a	0.751604556993805	0.00330856759163292	0.0893406097730125	NA	NA	NA	XP_001660336.1kelch-like protein 40a
AAEL00004	ornithine decarboxylase 1	0.753932629340302	0.000246814858948405	0.0191849588638347	NA	NA	KXJ79077.1 hypothet	XP_001647940.2ornithine decarboxylase 1
AAEL02180	NA	0.756371130556047	0.016735799036493	0.209527649977971	NA	NA	NA	NA
AAEL00195	NEDD8 ultimate buster 1	0.757658365203967	0.000293274266177041	0.0210836702918838	NA	NA	KXJ78706.1 hypothet	XP_021706132.1NEDD8 ultimate buster 1
AAEL02545	NA	0.759517943928525	0.0381469290817046	0.308460493330269	NA	NA	NA	XP_001653712.2zinc finger protein 157
AAEL01513	protein NPC2 homolog	0.75980719579223	0.000166301339081916	0.0152111375514763	NA	aag04142	NA	XP_001647805.2protein NPC2 homolog
AAEL00653	uncharacterized LOC5568109	0.760904382341739	0.0172898241019953	0.214133191637362	NA	NA	KXJ73810.1 hypothet	XP_021696647.1uncharacterized protein LOC5568109
AAEL02153	NA	0.761202168014115	1,25E+09	0.00243862310008055	NA	NA	NA	XP_021699401.1rhythmically expressed gene 2 protein
AAEL00232	uncharacterized LOC5574200	0.7631457671219	0.0487352323627945	0.351987741182022	NA	NA	KXJ81505.1 hypothet	XP_001661257.2uncharacterized protein LOC5574200
AAEL01825	NA	0.763868096218486	0.0116656858858087	0.173701685309403	NA	NA	NA	XP_001663293.2serine/arginine repetitive matrix protein 2
AAEL00090	serendipity locus protein alpha	0.768620735173134	0.0281602935477283	0.271910830247844	NA	NA	KXJ69250.1 hypothet	XP_021707680.1serendipity locus protein alpha
AAEL01956	NA	0.7706228913501	0.066647699408294	0.399540230684495	NA	NA	NA	XP_021709807.1uncharacterized protein LOC5575087
AAEL01258	dimethyladenosine transferase 2, mitochondrial	0.771836143460674	0.0255840566598537	0.260925069284669	NA	NA	XP_019530681.1 PRE	XP_001662715.2dimethyladenosine transferase 2, mitochondrial
AAEL00259	general odorant-binding protein 56a	0.772580455530589	0.037369243421374	0.305927960955648	NA	NA	KXJ71994.1 hypothet	XP_001655722.2general odorant-binding protein 56a
AAEL00654	vitellogenic carboxypeptidase-like	0.773899363404931	0.0102355356610829	0.163088391445208	NA	NA	AND61388.1 putative	XP_001652055.1vitellogenic carboxypeptidase-like
AAEL02218	NA	0.774716779577738	0.000862441675145332	0.0409667993696665	NA	aag04933	NA	XP_021707159.1NADPH oxidase 4 isoform X2
AAEL01178	uncharacterized LOC5575383	0.775559246113973	0.0317336259958758	0.284442054201394	NA	NA	NA	XP_021705709.1uncharacterized protein LOC5575383 isoform X1
AAEL00439	octopamine receptor	0.777060183336251	0.040037094018125	0.31441514682591	NA	NA	KFB41294.1 AGAP01	XP_021692997.1octopamine receptor
AAEL00925	N-acetylneuraminate lyase B	0.780432179572858	0.00240413748081759	0.0760003211995279	NA	aag01100 ; aag	NA	XP_001659894.2N-acetylneuraminate lyase B
AAEL02449	NA	0.781452420757293	6,46E+08	0.00156538082998626	NA	aag01100 ; aag	NA	XP_021694819.1cytosolic purine 5'-nucleotidase isoform X3
AAEL00189	uncharacterized LOC5572815	0.782524296132749	0.00228278803624059	0.0747232890974067	NA	NA	NA	XP_001654216.2uncharacterized protein LOC5572815
AAEL02175	NA	0.786243034118207	0.0178370706514204	0.216539213897586	NA	NA	NA	---NA---
AAEL00772	protein MMS22-like	0.78902490501032	0.031819391690633	0.284442054201394	NA	NA	XP_001687810.1 Anc	XP_001652844.2protein MMS22-like
AAEL00452	NA	0.791715423683328	0.0273160488806116	0.268836665026084	NA	NA	KXJ73476.1 hypothet	XP_001649325.2uncharacterized protein LOC5564982
AAEL01305	zinc finger and SCAN domain-containing protein 21	0.792987516270552	0.0377792064096065	0.307682856236235	NA	NA	NA	XP_021704233.1zinc finger protein 771 isoform X1
AAEL01180	uncharacterized LOC5575396	0.795410144704435	0.0694658591024346	0.404817003493294	NA	NA	KXJ75511.1 hypothet	XP_001661913.1uncharacterized protein LOC5575396
AAEL02905	NA	0.801454786620324	0.00376245156503629	0.0953776289296528	NA	NA	NA	XP_019539622.1 probable endochitinase
AAEL01955	NA	0.805094037291407	0.0668650202354773	0.399699227060601	NA	NA	NA	XP_021693825.1zinc finger protein OZF

AAEL01264	uncharacterized LOC5576616	0.809355741997519	0.0367182461552222	0.303032557058614	NA	NA	KXJ80513.1 hypothet	XP_001662770.1uncharacterized protein LOC5576616
AAEL02001	NA	0.811564377789156	0.011549379467673	0.173372577261871	NA	NA	NA	XP_021703667.1zinc finger protein 84-like
AAEL00579	uncharacterized LOC5567077	0.811724737474457	0.00230243276247914	0.0747232890974067	NA	NA	KXJ77887.1 hypothet	XP_001651473.3uncharacterized protein LOC5567077
AAEL02725	NA	0.815673349661668	0.0537628331337459	0.366428852058877	NA	NA	NA	NA
AAEL02814	NA	0.816748074860441	1,04E+09	0.00215576414324494	NA	NA	NA	XP_001660472.2alanine aminotransferase 1
AAEL00435	uncharacterized LOC5564661	0.81802575302958	0.0514837142208328	0.359721441351635	NA	NA	KFB43740.1 AGAP00	KFB43740.1AGAP001147-like protein
AAEL01991	NA	0.818534612150424	0.0195270623933512	0.227165648727709	NA	NA	NA	XP_019553650.1 hemicentin-1-like
AAEL00779	E3 ubiquitin-protein ligase RNF220	0.820403080476176	0.0322064019805158	0.284442054201394	NA	NA	KXJ72570.1 hypothet	XP_001652913.1E3 ubiquitin-protein ligase RNF220
AAEL02806	NA	0.820718188759172	0.00571631490231733	0.120923056853159	NA	NA	NA	NA
AAEL00662	uncharacterized LOC5568169	0.82309091458439	0.00853971434444357	0.149464458238032	901	NA	NA	XP_021703894.1uncharacterized protein LOC5568169 isoform X1
AAEL02363	NA	0.82394273396195	0.000773682823840628	0.0378693050264971	NA	NA	NA	XP_021701795.1microsomal triglyceride transfer protein large subunit isoform X1
AAEL00198	ejaculatory bulb-specific protein 3	0.82480042325083	0.151245133434819	0.566025396924239	NA	NA	KXJ74787.1 hypothet	XP_001660775.1ejaculatory bulb-specific protein 3
AAEL00372	rutC family protein UK114	0.827333790668106	0.00185742511238367	0.0670275524868807	NA	NA	KXJ73551.1 hypothet	XP_021699358.1rutC family protein UK114
AAEL00947	proton-coupled amino acid transporter-like protein CG1139	0.829957665270163	0.013245919279559	0.185523514780064	NA	NA	NA	XP_021699097.1proton-coupled amino acid transporter-like protein CG1139
AAEL01437	uncharacterized LOC5564277	0.831764444869961	0.00699544322129595	0.137411930736898	NA	NA	NA	XP_021709625.1uncharacterized protein LOC5564277
AAEL00528	uncharacterized LOC5566255	0.833507824104594	0.0266081683065443	0.266719351586951	NA	NA	KXJ82494.1 hypothet	XP_001650634.2uncharacterized protein LOC5566255 isoform X2
AAEL00787	ornithine decarboxylase	0.843028234244049	0.00393770772159376	0.0974053399303919	NA	aag01100 ; aag	XP_001850295.1 orn	XP_021708713.1ornithine decarboxylase
AAEL01120	uncharacterized LOC5574528	0.845469065655518	0.0248656951962337	0.258356472981449	230	NA	KXJ75119.1 hypothet	XP_001655180.1uncharacterized protein LOC5574528
AAEL02721	NA	0.851697079084068	0.00556167760004677	0.119576068401005	NA	NA	NA	XP_021709470.1C-type lectin 37Db-like
AAEL00348	zinc finger protein 28	0.862119361342186	0.0306171600565468	0.281224109837052	NA	NA	KXJ76568.1 hypothet	XP_001656839.2zinc finger protein 28
AAEL00372	uncharacterized LOC5578859	0.868166993024917	0.0307140002593173	0.281224109837052	NA	NA	NA	XP_021699354.1uncharacterized protein LOC5578859 isoform X2
AAEL00698	probable cytochrome P450 28a5	0.869249696818145	0.000948174084539538	0.0425356957217985	NA	NA	KXJ78559.1 hypothet	XP_021705929.1probable cytochrome P450 28a5
AAEL02527	NA	0.872298265500311	0.0157764075474962	0.202259474938964	NA	NA	NA	XP_021697811.1uncharacterized protein LOC110675968
AAEL02718	NA	0.885356915086966	0.0742470441885545	0.418818002133309	NA	NA	NA	XP_021705271.1uncharacterized protein LOC110677856
AAEL00523	titin	0.892196959449452	0.014395051919619	0.192533819424904	NA	NA	KXJ75014.1 hypothet	XP_021705269.1titin
AAEL02614	NA	0.893920283528633	0.0379446948245576	0.307682856236235	NA	NA	NA	NA
AAEL00848	uncharacterized LOC5570668	0.89439034845183	0.00714628372597934	0.137861850831157	NA	NA	XP_001841966.1 con	XP_001659263.2uncharacterized protein LOC5570668
AAEL02045	NA	0.894639829477081	0.0119238046570389	0.176403296549955	NA	NA	NA	NA
AAEL00269	glutamyl aminopeptidase	0.896409333911808	3,03E+07	0.000278745599142822	NA	NA	XP_019544614.1 PRE	XP_001662133.2glutamyl aminopeptidase
AAEL01081	probable proline--tRNA ligase, mitochondrial	0.903616384047223	0.0278565713768516	0.271255206147924	970	aag00970	KFB37342.1 AGAP00	XP_001655003.1probable proline--tRNA ligase, mitochondrial
AAEL01362	trypsin alpha-3	0.907663183398077	0.000138714674186162	0.0138744829550115	NA	NA	NA	XP_021693230.1trypsin alpha-3
AAEL01980	NA	0.909214560058821	0.00266391542172951	0.0792437883464128	NA	NA	NA	XP_021706339.1uncharacterized protein LOC5568064
AAEL02581	NA	0.913106016872968	0.0163969019992976	0.206858269058754	NA	NA	NA	NA
AAEL02709	NA	0.913495981780415	0.000945547992443875	0.0425356957217985	NA	NA	NA	XP_021710913.1cyclic AMP response element-binding protein A isoform X1
AAEL00678	probable RNA methyltransferase bin3	0.91537704297791	0.00105665536892131	0.0454789543350121	NA	NA	KXJ71400.1 hypothet	XP_001652233.2probable RNA methyltransferase bin3
AAEL01091	structural maintenance of chromosomes protein 5	0.917573925115344	0.00153005923526651	0.0567726011408162	NA	NA	KXJ69342.1 hypothet	XP_001661151.2structural maintenance of chromosomes protein 5
AAEL00274	synaptic vesicle glycoprotein 2B	0.920099065140947	0.004021731742457	0.0986879346509048	NA	NA	KXJ72850.1 hypothet	XP_021709863.1synaptic vesicle glycoprotein 2B
AAEL00614	membrane-associated tyrosine- and threonine-specific cdc2-inhibitory ki	0.922469811045299	0.0114758994430327	0.172833431546296	04151 ; 05165 ; 04714 ;	NA	KXJ79183.1 hypothet	XP_001657528.2membrane-associated tyrosine- and threonine-specific cdc2-inhibitory kinase
AAEL01253	leucine-rich repeat-containing G-protein coupled receptor 4	0.926291423090095	1,08E+08	0.00215981691782796	NA	NA	KXJ78758.1 hypothet	XP_001662642.1leucine-rich repeat-containing G-protein coupled receptor 4
AAEL01963	NA	0.927565136855474	2,14E+08	0.000677903158822576	NA	NA	NA	XP_021693132.1uncharacterized protein LOC5566459
AAEL01425	replication protein A 32 kDa subunit	0.928117356125214	0.000973503275219486	0.0432547756744923	NA	aag03420 ; aag	NA	XP_001648269.1replication protein A 32 kDa subunit
AAEL02002	NA	0.930681305310234	0.00696450444666193	0.137411930736898	NA	NA	NA	XP_001650335.1islet cell autoantigen 1
AAEL02863	NA	0.931225907401579	5,71E+09	0.00729936398983891	NA	NA	NA	XP_001652222.1probable cytochrome P450 9f2
AAEL02255	NA	0.933929831880804	0.00168741400561161	0.0616173955541191	NA	NA	NA	NA
AAEL00521	uncharacterized LOC5566176	0.940247823736422	2,89E+06	0.000278745599142822	NA	NA	NA	DAA64999.1TPA_exp: septate junction protein sinuous
AAEL01151	multiple inositol polyphosphate phosphatase 1	0.941132678589303	0.00324707680649558	0.0889935739723594	NA	aag01100 ; aag	XP_019547215.1 PRE	XP_001661709.1multiple inositol polyphosphate phosphatase 1
AAEL02079	NA	0.946732534181153	0.00991520444301588	0.161167614967993	NA	NA	NA	XP_021703640.1mediator of RNA polymerase II transcription subunit 15-like
AAEL00259	uncharacterized LOC5575337	0.948365374025356	0.00844631939759359	0.148894695587464	NA	NA	KFB49738.1 hypothe	XP_001655715.1uncharacterized protein LOC5575337
AAEL02144	NA	0.967852640542229	0.00359379679349439	0.0919227252703372	NA	NA	NA	XP_021711798.1galactosylgalactosylxylosylprotein 3-beta-glucuronosyltransferase S-like
AAEL00949	transcription factor grauzone	0.968716279289587	0.0085571581807247	0.149464458238032	NA	NA	NA	XP_001660164.1transcription factor grauzone
AAEL00713	facilitated trehalose transporter Tret1	0.974181362020323	3,20E+08	0.000950861383579639	NA	NA	KXJ82002.1 hypothet	XP_001658145.2facilitated trehalose transporter Tret1
AAEL00113	U11/U12 small nuclear ribonucleoprotein 35 kDa protein	0.978399342915857	0.0217209377349654	0.240814541008616	NA	NA	KXJ70531.1 hypothet	XP_001652414.2U11/U12 small nuclear ribonucleoprotein 35 kDa protein
AAEL02793	NA	10.130.788.025.538	0.0152218467672975	0.198907702459398	NA	NA	NA	NA
AAEL00245	zinc finger protein 62	101.343.736.556.587	3,62E+09	0.00520672089581033	NA	NA	XP_019542227.1 PRE	XP_021699285.1zinc finger protein 62
AAEL02042	NA	102.258.117.794.985	0.129702466700771	0.536414426328313	NA	NA	NA	XP_021695750.1uncharacterized protein LOC110675359 isoform X2
AAEL02646	NA	102.947.929.799.857	0.0611078072618695	0.386196065431147	NA	NA	NA	XP_019542027.1 cystatin-like protein
AAEL01978	NA	103.450.042.151.961	0.0277999539492184	0.271255206147924	NA	NA	NA	XP_001663344.140S ribosomal protein S3a
AAEL02096	NA	104.271.988.246.878	0.0122559673656396	0.179285423221345	NA	NA	NA	NA
AAEL02617	NA	10.486.665.672.024	1,05E+09	0.00215576414324494	NA	NA	NA	XP_021711797.1LOW QUALITY PROTEIN: uncharacterized protein LOC110680285
AAEL01427	protein lin-37 homolog	10.534.403.559.463	0.0127462088854148	0.182308324322341	NA	NA	NA	XP_001648337.2protein lin-37 homolog
AAEL01146	NA	105.988.300.231.221	0.000903533352282452	0.0413193578047436	NA	aag00981	XP_019527184.1 PRE	XP_001661673.2cytochrome P450 302a1, mitochondrial
AAEL00101	zinc finger protein 32-like	106.297.415.930.447	0.000310551761359427	0.0214864459250334	NA	NA	KXJ68601.1 hypothet	XP_021711677.1zinc finger protein 771
AAEL01112	sorbitol dehydrogenase	106.655.951.928.056	7,24E+08	0.00162440588081637	NA	aag01100 ; aag	KXJ69734.1 hypothet	XP_001655105.1sorbitol dehydrogenase
AAEL00018	elongation of very long chain fatty acids protein 7	106.961.315.944.204	0.0109171658224539	0.168274304687137	62	NA	KXJ71832.1 hypothet	XP_001658888.1elongation of very long chain fatty acids protein 7
AAEL00039	uncharacterized LOC5576314	107.036.919.428.771	0.000192405418883998	0.0165468660240239	NA	NA	KXJ84507.1 hypothet	XP_001656058.2uncharacterized protein LOC5576314
AAEL00787	uncharacterized LOC5569746	108.487.919.159.491	2,07E+09	0.00366754463981726	NA	NA	KXJ79271.1 hypothet	XP_021708825.1uncharacterized protein LOC5569746
AAEL02233	NA	108.837.911.190.695	2,10E+07	0.000278745599142822	NA	NA	NA	NA
AAEL00912	cytochrome P450 6d3	109.166.028.863.535	0.00723509605525323	0.139143819679039	NA	NA	XP_019538136.1 PRE	XP_001653688.1cytochrome P450 6d3
AAEL00183	uncharacterized LOC5572468	109.681.524.179.032	0.000262912435891253	0.0195653835802185	901	NA	KFB39153.1 AGAP00	XP_001843741.1Juvenile hormone-inducible protein
AAEL02745	NA	110.258.345.055.223	0.00257342718343537	0.0784128375561996	NA	NA	NA	XP_021712702.139S ribosomal protein L40, mitochondrial-like
AAEL02536	NA	117.611.649.466.084	4,55E+07	0.000323434375000257	NA	NA	NA	NA
AAEL00136	uncharacterized LOC5570344	117.803.224.418.271	0.00733714336141673	0.140075504588707	NA	NA	XP_001688786.1 AG	XP_001653173.1uncharacterized protein LOC5570344
AAEL02617	NA	118.784.203.451.343	0.000171907816630614	0.0155087816532834	NA	NA	NA	XP_021712803.1acyl-CoA-binding domain-containing protein 6-like
AAEL02110	NA	120.446.841.339.963	0.0103870585186316	0.164229746543725	NA	NA	NA	NA
AAEL00879	zinc finger protein 888	122.136.458.841.022	0.0258293588423463	0.262052657185525	NA	NA	KXJ78519.1 hypothet	XP_021709660.1zinc finger protein 888
AAEL02426	NA	123.194.612.983.642	0.0161290117968084	0.204716091798939	NA	NA	NA	XP_019933249.1 mucin-22-like
AAEL01328	collagenase	126.038.489.178.042	0.131079944792334	0.538981683630591	NA	NA	NA	AAF82286.1late trypsin precursor
AAEL02376	NA	126.787.958.947.023	0.000486027537416994	0.0288543574149108	NA	NA	NA	XP_021704430.140S ribosomal protein S21-like
AAEL02701	NA	128.187.668.866.307	0.00592944081298327	0.124857469933803	NA	NA	NA	XP_021711976.1translocon-associated protein subunit alpha-like
AAEL02724	NA	128.720.324.227.817	0.00400166452775343	0.098458066803174	NA	NA	NA	NA
AAEL02078	NA	130.545.635.544.852	0.012590313235545	0.181877648969364	NA	NA	NA	NA
AAEL00372	uncharacterized LOC5578868	133.832.060.440.665	0.00216682625055587	0.0730371251194695	NA	NA	NA	XP_001664079.2uncharacterized protein LOC5578868

AAEL01008	protein AAR2 homolog	136.099.598.341.241	4,81E+09	0.00660452365101539	NA	NA	NA		XP_001660621.2protein AAR2 homolog
AAEL01385	serine protease Hayan	138.218.626.691.302	1,63E+08	0.000598861082494629	NA	NA	NA		XP_001647837.2serine protease Hayan isoform X2
AAEL00578	serine protease easter	143.711.967.575.771	9,40E+08	0.0110128080760421	NA	NA	KXJ80704.1 hypothet		XP_001651440.2serine protease easter
AAEL00637	trypsin	145.720.625.665.077	0.00076114482557134	0.0376562079833735	NA	NA	KXJ72009.1 hypothet		XP_019543394.1 trypsin-like
AAEL02134	NA	148.219.488.481.741	0.0131579481453997	0.184874636813685	NA	NA	NA		NA
AAEL02233	NA	15.740.248.405.351	0.000162390658398146	0.0151039840659274	NA	NA	NA		NA
AAEL02729	NA	157.899.489.429.446	0.000270066868644904	0.0198812426021633	NA	NA	NA		NA
AAEL01751	extensin	159.365.123.781.781	3,37E+02	3,10E+06	NA	NA	NA		XP_011493274.2extensin
AAEL00338	attacin-B	165.873.379.289.328	0.00490439866479967	0.111432781514782	NA	NA	XP_011296538.1 PRE		XP_019530162.1 attacin-B-like
AAEL00546	parkin coregulated gene protein homolog	166.504.948.265.204	0.00123821853661635	0.0493695062346524	NA	NA	ETN67242.1 hypothe		XP_001650935.1parkin coregulated gene protein homolog
AAEL00384	uncharacterized LOC5579101	171.870.079.511.183	0.00637419096209	0.130056109164417	NA	NA	KXJ69513.1 hypothet		EAT44835.1AAEL003848
AAEL00190	ras-related and estrogen-regulated growth inhibitor-like protein	183.755.416.164.514	0.00061394288297474	0.0332323671125503	NA	NA	ETN60858.1 MRAS2 |		XP_021702671.1ras-related and estrogen-regulated growth inhibitor-like protein
AAEL00964	cathepsin B-like cysteine proteinase 3	189.914.829.530.222	1,85E+08	0.00065460040988707	NA	NA	NA		XP_001653891.1cathepsin B-like cysteine proteinase 3
AAEL02151	NA	1.900.651.148.587	8,50E+07	0.000399983581196831	NA	NA	NA		XP_021697954.1mucin-2 isoform X2
AAEL02433	NA	192.570.353.149.024	3,49E+09	0.0050921831145908	NA	NA	NA		NA
AAEL00656	vitellogenic carboxypeptidase	214.037.576.845.652	0.00123933448600355	0.0493695062346524	NA	NA	KXJ80663.1 hypothet		XP_001652056.2vitellogenic carboxypeptidase
AAEL01947	NA	244.740.819.969.169	0.117329467634863	0.515089129249485	NA	NA	NA		XP_001660827.1protein G12
AAEL01043	vitellogenin-A1	349.560.344.070.568	0.015627415132632	0.201687901894081	NA	NA	NA		Q16927.2RecName: Full=Vitellogenin-A1; Short=VG; AltName: Full=PVG1;
AAEL00612	vitellogenin-A1-like	431.006.060.655.684	0.00626921801855768	0.129262654794788	NA	NA	KXJ79966.1 hypothet		XP_019932284.1 vitellogenin-A1-like
AAEL00613	vitellogenin-A1-like	500.465.433.098.021	0.0113344517997933	0.172681499108771	NA	NA	KXJ71699.1 hypothet		XP_019932282.1 vitellogenin-A1-like

Table S5: Status of *Aedes aegypti* genes previously investigated in other transcription studies, detected among the transcriptome dataset of 9202 genes by EdgeR from the present study.

		EdgeR		
**ID**	**Description**	**Log2 fold change**	***p*-value**	**Reference[a]**
**Receptors of Bti Cry toxins**				
AAEL012778	Protease m1 zinc metalloprotease	0.10055	0.56193	[1]
AAEL008155	APN2- aminopeptidade N	ND[b]	ND	[1]
AAEL012776	Protease m1 zinc metalloprotease	0.15517	0.70265	[2]
AAEL012217	Protease m1 zinc metalloprotease	-0.0636	0.68272	[2]
AAEL013330	Alkaline phosphatase	ND	ND	[1]
AAEL009077	Alkaline phosphatase	0.12344	0.69268	[1]
AAEL015070	Alkaline phosphatase	0.15390	0.47649	[1]
AAEL007478	Cadherin	ND	ND	[3]
AAEL013421[c]	Alpha-amylase	-1.48277	0.00005	[4]
AAEL012395	ATP-binding cassette transporter	0.07104	0.70029	[5]
AAEL005929	ATP-binding cassette transporter	0.35621	0.01091	[5]
**Peptidases that processing of Bti protoxins into toxins**				
AAEL007765	Serine Protease Inhibitor (serpin)	0.09463	0.38613	[6]
AAEL002730	Serine Protease Inhibitor (serpin)	0.09902	0.66489	[7]
AAEL002629	Serine Protease	0.10653	0.42687	[7]
AAEL006700	Trypsin, putative	0.23414	0.46802	[7]
AAEL006376	Trypsin	1.45721	0.00076	[6]
**Detoxifying enzimes**				
AAEL009131	Cytochrome P450	-0.12482	0.30646	[6]
AAEL002385	Carboxy/choline esterase Alpha Esterase	0.17685	0.22540	[7]
AAEL010592	Esterase, putative	0.00202	0.99309	[7]
AAEL010582	Glutathione transferase	-0.16042	0.44472	[7]
AAEL013441	Toll-like receptor	-0.16102	0.37269	[7]

AAEL014138	Serine Protease Inhibitor (serpin)	-0.16047	0.48813	[7]
AAEL003628	Clip-Domain Serine Protease family B	-1.11749	0.00339	[7]
AAEL010656[c]	leucine-rich immune protein	-1.26361	3.70 E-08	[7]
AAEL011593	Clip-Domain Serine Protease family C	-0.92700	0.04655	[7]
AAEL006168	Clip-Domain Serine Protease family B	-0.79489	0.00205	[7]
AAEL026297	Protein toll	-0.68685	0.00323	[6]
AAEL015099	Sumo ligase/E3 SUMO-protein ligase ...	-0.13891	0.37227	[7]
AAEL012471	Domeless	-0.03187	0.81127	[8]
AAEL012553	Hop	0.366637	0.00815	[9]
AAEL007696	Relish 1	-0.13348	0.23248	[10]
AAEL000709	Cactus	-0.24075	0.04839	[8]
AAEL003841	Defensin A	ND	ND	[11]
AAEL015515	Cecropin G	ND	ND	[10]
AAEL004522	Gambicin	0.50594	0.00455	[12]
AAEL013517	Pupal cuticle protein 78E, putative	0.28165	0.05991	[6]
AAEL014978	Adult cuticle protein, putative	-1.7588	0.00244	[6]
AAEL015424 [c]	Adult cuticle protein, putative	-1.52009	0.00016	[4]
AAEL002231	Cuticle protein, putative	-0.43406	0.19605	[4]
AAEL011496	Chitinase	-0.31410	0.15089	[7]
AAEL009782	Brain chitinase and chia/chitinase-like	0.03769	0.80105	[4]

[a] References of the differential expression of these genes: [1] Lee et al. 2014; [2] Tetreau et al. 2012; [3]Jiménez et al. 2012; [4] Stalinski et al. 2016; [5] Batool et al. 2018; [6]Després et al. 2014; [7] Paris et al. 2012; [8] Angleró et al. 2017; [9]Jupatanakul et al. 2017; [10]Chowdury et al. 2020; [11]Zhao et al. 2018; [12]Etebari et al. 2017. [b] Not detected.[c] Genes that were found among the DEGs presented in Table 2

References

1. Lee, S.B.; Aimanova, K.G.; Gill, S.S. Alkaline phosphatases and aminopeptidases are altered in a Cry11Aa resistant strain of *Aedes aegypti*. *Insect Biochem. Mol. Biol.* **2014**, *54*, 112-121, doi:10.1016/j.ibmb.2014.09.004.
2. Tetreau, G.; Bayyareddy, K.; Jones, C.M.; Stalinski, R.; Riaz, M.A.; Paris, M.; David, J.P.; Adang, M.J.; Despres, L. Larval midgut modifications associated with Bti resistance in the yellow fever mosquito using proteomic and transcriptomic approaches. *BMC Genomics* **2012**, *13*, 248, doi:10.1186/1471-2164-13-248.
3. Jimenez, A.I.; Reyes, E.Z.; Cancino-Rodezno, A.; Bedoya-Perez, L.P.; Caballero-Flores, G.G.; Muriel-Millan, L.F.; Likitvivatanavong, S.; Gill, S.S.; Bravo, A.; Soberón, M. *Aedes aegypti* alkaline phosphatase ALP1 is a functional receptor of *Bacillus thuringiensis* Cry4Ba and Cry11Aa toxins. *Insect Biochem. Mol. Biol.* **2012**, *42*, 683-689, doi:10.1016/j.ibmb.2012.06.001.
4. Stalinski, R.; Laporte, F.; Tetreau, G.; Despres, L. Receptors are affected by selection with each *Bacillus thuringiensis israelensis* Cry toxin but not with the full Bti mixture in *Aedes aegypti*. *Infect. Genet. Evol.* **2016**, *44*, 218-227, doi:10.1016/j.meegid.2016.07.009.
5. Batool, K.; Alam, I.; Wu, S.; Liu, W.; Zhao, G.; Chen, M.; Wang, J.; Xu, J.; Huang, T.; Pan, X.; et al. Transcriptomic Analysis of *Aedes aegypti* in Response to Mosquitocidal *Bacillus thuringiensis* LLP29 Toxin. *Sci Rep* **2018**, *8*, 12650, doi:10.1038/s41598-018-30741-x.
6. Despres, L.; Stalinski, R.; Tetreau, G.; Paris, M.; Bonin, A.; Navratil, V.; Reynaud, S.; David, J.P. Gene expression patterns and sequence polymorphisms associated with mosquito resistance to *Bacillus thuringiensis israelensis* toxins. *BMC Genomics* **2014**, *15*, 926, doi:10.1186/1471-2164-15-926.
7. Paris, M.; Melodelima, C.; Coissac, E.; Tetreau, G.; Reynaud, S.; David, J.P.; Despres, L. Transcription profiling of resistance to Bti toxins in the mosquito *Aedes aegypti* using next-generation sequencing. *J Invertebr Pathol* **2012**, *109*, 201-208, doi:10.1016/j.jip.2011.11.004.
8. Anglero-Rodriguez, Y.I.; MacLeod, H.J.; Kang, S.; Carlson, J.S.; Jupatanakul, N.; Dimopoulos, G. *Aedes aegypti* Molecular Responses to Zika Virus: Modulation of Infection by the Toll and Jak/Stat Immune Pathways and Virus Host Factors. *Front Microbiol* **2017**, *8*, 2050, doi:10.3389/fmicb.2017.02050.
9. Jupatanakul, N.; Sim, S.; Anglero-Rodriguez, Y.I.; Souza-Neto, J.; Das, S.; Poti, K.E.; Rossi, S.L.; Bergren, N.; Vasilakis, N.; Dimopoulos, G. Engineered *Aedes aegypti* JAK/STAT Pathway-Mediated Immunity to Dengue Virus. *PLoS Negl Trop Dis* **2017**, *11*, e0005187, doi:10.1371/journal.pntd.0005187.
10. Chowdhury, A.; Modahl, C.M.; Tan, S.T.; Wong Wei Xiang, B.; Misse, D.; Vial, T.; Kini, R.M.; Pompon, J.F. JNK pathway restricts DENV2, ZIKV and CHIKV infection by activating complement and apoptosis in mosquito salivary glands. *PLoS Pathog* **2020**, *16*, e1008754, doi:10.1371/journal.ppat.1008754.
11. Zhao, L.; Alto, B.W.; Smartt, C.T.; Shin, D. Transcription Profiling for Defensins of *Aedes aegypti* (Diptera: Culicidae) During Development and in Response to Infection With Chikungunya and Zika Viruses. *J Med Entomol* **2018**, *55*, 78-89, doi:10.1093/jme/tjx174.
12. Etebari, K.; Hegde, S.; Saldana, M.A.; Widen, S.G.; Wood, T.G.; Asgari, S.; Hughes, G.L. Global Transcriptome Analysis of *Aedes aegypti* Mosquitoes in Response to Zika Virus Infection. *mSphere* **2017**, *2*, doi:10.1128/mSphere.00456-17.

Table S6: KEGG pathways with enriched downregulated or upregulated terms in *Aedes aegypti* females from the RecBti strain.

ID	Downregulated terms	NES	*P*-value	p.adjust
aag00190	Oxidative phosphorylation	-211.532.483.662.193	1,50E+08	0.000163988864417927
aag04137	Mitophagy - animal	-18.323.206.408.079	0.00345046422874071	0.0537286572761053
aag00500	Starch and sucrose metabolism	-176.520.997.269.741	0.00722547508254559	0.0984470979996836
aag00020	Citrate cycle (TCA cycle)	-166.170.413.938.232	0.0147363180228477	0.144090402082107
aag04512	ECM-receptor interaction	-164.594.644.994.281	0.0158631635319751	0.144090402082107
aag00620	Pyruvate metabolism	-165.940.459.898.363	0.0208111839754096	0.174493773332281
aag04624	Toll and Imd signaling pathway	-157.576.775.942.046	0.029074032641383	0.211271303860716
**ID**	**Upregulated terms**	**NES**	***P*-value**	**p.adjust**
aag03008	Ribosome biogenesis in eukaryotes	208.273.558.127.666	2,78E+09	0.0015175757361982
aag00510	N-Glycan biosynthesis	211.998.495.196.002	0.000211866345951407	0.0076978105695678
aag03060	Protein export	199.226.188.011.787	0.000524574302897475	0.0142946497539562
aag04141	Protein processing in endoplasmic reticulum	171.306.129.616.463	0.00214777987219948	0.0468216012139488
aag03460	Fanconi anemia pathway	182.081.826.809.638	0.00286257060056326	0.0520033659102326
aag00513	Various types of N-glycan biosynthesis	168.547.365.899.462	0.0100489050030466	0.121703405036898
aag03440	Homologous recombination	171.754.062.990.104	0.0116687868245694	0.127189776387806
aag00051	Fructose and mannose metabolism	160.365.631.361.689	0.027160830488883	0.211271303860716
